# Validation of Genotyping-By-Sequencing Analysis in Populations of Tetraploid Alfalfa by 454 Sequencing

**DOI:** 10.1371/journal.pone.0131918

**Published:** 2015-06-26

**Authors:** Solen Rocher, Martine Jean, Yves Castonguay, François Belzile

**Affiliations:** 1 Centre de Recherche et de Développement sur les Sols et les Grandes Cultures, Agriculture et agroalimentaire Canada, Quebec City (QC), Canada; 2 Département de Phytologie and Institut de Biologie Intégrative et des Systèmes, Université Laval, Quebec City (QC), Canada; USDA/ARS, UNITED STATES

## Abstract

Genotyping-by-sequencing (GBS) is a relatively low-cost high throughput genotyping technology based on next generation sequencing and is applicable to orphan species with no reference genome. A combination of genome complexity reduction and multiplexing with DNA barcoding provides a simple and affordable way to resolve allelic variation between plant samples or populations. GBS was performed on *Ape*KI libraries using DNA from 48 genotypes each of two heterogeneous populations of tetraploid alfalfa (*Medicago sativa* spp. *sativa*): the synthetic cultivar Apica (ATF0) and a derived population (ATF5) obtained after five cycles of recurrent selection for superior tolerance to freezing (TF). Nearly 400 million reads were obtained from two lanes of an Illumina HiSeq 2000 sequencer and analyzed with the Universal Network-Enabled Analysis Kit (UNEAK) pipeline designed for species with no reference genome. Following the application of whole dataset-level filters, 11,694 single nucleotide polymorphism (SNP) loci were obtained. About 60% had a significant match on the *Medicago truncatula* syntenic genome. The accuracy of allelic ratios and genotype calls based on GBS data was directly assessed using 454 sequencing on a subset of SNP loci scored in eight plant samples. Sequencing depth in this study was not sufficient for accurate tetraploid allelic dosage, but reliable genotype calls based on diploid allelic dosage were obtained when using additional quality filtering. Principal Component Analysis of SNP loci in plant samples revealed that a small proportion (<5%) of the genetic variability assessed by GBS is able to differentiate ATF0 and ATF5. Our results confirm that analysis of GBS data using UNEAK is a reliable approach for genome-wide discovery of SNP loci in outcrossed polyploids.

## Introduction

Alfalfa (*Medicago sativa* spp.) is a perennial forage legume grown over 32 million ha worldwide [1]. It is an open-pollinated autotetraploid (2n = 4x = 32) with a relatively large genome (800–900 Mbp) showing high genetic variability at the genotype and population levels [2]. Alfalfa possesses several attributes for sustainable intensification of agricultural production including biological fixation of atmospheric nitrogen, carbon sequestration by belowground biomass and capture of mineral nutrients deep in the soil profile [3]. However, lack of winter hardiness due to insufficient capacity to withstand exposure to low subfreezing temperatures remains a major constraint to the reliable use of alfalfa in cold climates [4].

Alfalfa germplasm offers a large reservoir of genetic diversity to improve tolerance to environmental stresses. Recurrent selection is a cyclical breeding approach that progressively modifies the frequency of alleles affecting traits under selection and that promotes the optimal assortment of sequence variants conferring superior performance [5]. Populations of alfalfa with superior tolerance to freezing (TF) were developed by exposing broad-based synthetic varieties to recurrent cycles of selection for survival after exposure to freezing tests performed indoors under highly controlled conditions [6]. Evidence for changes in the frequency of alleles between TF populations and initial genetic backgrounds were obtained using sequence-related amplified polymorphism (SRAP) markers [7] and sequence analysis of candidate genes putatively associated with cold adaptation [8]. Even though PCR- or candidate-based searches can identify DNA variations linked to quantitative traits, these approaches are resource-intensive and provide only limited genome coverage. Although few genomic resources are currently available for cultivated alfalfa, several studies have shown that genome-wide synteny between *Medicago sativa* and the model legume *Medicago truncatula* can be effectively exploited for comparative genomics between these two species [9].

Next-generation sequencing (NGS) technologies allow the characterization of genetic variation such as SNPs on a genome-wide scale. However, genome-wide sequencing of large and complex genomes of allogamous forage species is difficult to achieve even with NGS and requires complexity reduction [10]. Genotyping-by-sequencing (GBS) is an affordable and simple NGS strategy to extensively characterize variation between plant genomes even in the absence of a reference genome [11]. GBS has been used in outbreeding polyploids to develop high-density linkage maps [12], to provide insights on genome diversity and complexity [13] and to characterize changes in allele frequencies within and between populations [14]. Robust sampling of alleles, however, is essential for the analysis of the impact of selection on the genetic composition of populations [15,16]. Thus, determination of read depths providing reliable GBS estimates of genotype calls and allelic ratios in tetraploid alfalfa is warranted.

In the present study, our objectives were to: 1- Use GBS for genome-wide SNP discovery in broad-based populations of alfalfa; 2- Assess the reliability of GBS genotype calls by comparing them with genotypes inferred after 454 resequencing of a subset of SNP loci and; 3- Compare the distribution of genotypes from a recurrently-selected TF population and its initial background in a multivariate space defined by genome-wide sampling of SNP loci.

## Material and Methods

### Plant material

Forty-eight (48) genotypes each of the alfalfa (*M*. *sativa ssp*. *sativa*) cultivar Apica (ATF0) adapted to harsh winter conditions of eastern Canada [17] and the ATF5 population obtained after five cycles of recurrent selection for superior freezing tolerance within ATF0 (described in [6]) were used in this work. Plants were grown for six weeks in a growth chamber set to 22°C/17°C (day/night) temperatures with a 16h photoperiod. DNA extraction and quantification were carried out as described in [18]. DNA concentrations were normalized to 10 ng/μl prior to library preparation.

### Genotyping-by-sequencing

#### Library preparation and sequencing

A 96-plex *Ape*KI GBS library was prepared by the Plateforme d’analyses génomiques (Institut de Biologie Intégrative et des Systèmes (IBIS), Laval University, Quebec City, QC, Canada) using 100 ng each of the 96 DNA samples from the ATF0 and ATF5 populations essentially as previously described [11]. Additional complexity reduction was achieved through the use of three selective bases (ACC) added to the 3’-primer during library amplification [19]. The resulting GBS library was used for single-end sequencing on two lanes of an Illumina HiSeq 2000 (McGill University-Genome Québec Innovation Center, Montreal, QC, Canada). All sequences were submitted to the National Center for Biotechnology Information Short Read Archive under the accession number #SRX964320.

#### SNP calling

Illumina reads were processed using the Universal Network-Enabled Analysis Kit (UNEAK) for species without a reference genome implemented on TASSEL software (*v3*.*0*) [13] using default parameters on either the complete dataset obtained from sequencing of 96 samples or a partial dataset of 72 samples obtained after exclusion of samples with read counts (RC) < 1,000,000. SNP loci (i.e. two 64-bp sequences differing by one SNP, termed reciprocal tag pairs [TP] in UNEAK terminology) with a Minor Allele Frequency (MAF) ≥ 0.05 and a minimum call rate (mnC) of 0.5 in the partial dataset of 72 samples were retained (identified as whole dataset-filter). As UNEAK records a maximum of 127 reads for each allele in the final output, read counts above 127 for SNP loci retained after additional quality filtering were recovered from the TagCount files generated by UNEAK for each sample. A filtered dataset was obtained after the application of genotype-level filters [12] to remove genotypes with low read counts (RC) or unbalanced RC between alleles. Using that approach, genotypes with total RC<11 (homozygotes), RC<2 for one or both alleles (heterozygotes) or with Minor Allelic read Frequency (MAF_g_) <0.1 (heterozygotes) were considered as missing (N).

### Sequence similarity with available genomic resources

#### Alignment with *Medicago truncatula*

The localization of each SNP locus on the *M*. *truncatula* reference genome was evaluated using the Basic Local Alignment Search Tool (BLAST+ v2.2.28) using 1 x 10^-8^ as cut-off E-value. For SNP loci with multiple hits on the *M*. *truncatula* genome, the single highest score was retained. SNPs having multiple hits with identical score and SNPs having the two alleles mapping at different positions were discarded. BLAST searches were performed on the *M*. *truncatula* genome (*v4*.*0*) [20].

#### SNPs shared between different *Medicago sativa* germplasm analyzed by GBS

The proportion of shared SNP loci captured by GBS in two different alfalfa germplasm was evaluated by performing a BLAST search of the 11,694 SNP loci identified in the current study (with cv. Apica) against the 301,258 SNP loci identified using a similar approach in an alfalfa mapping population [12].

### Validation of GBS allelic ratios

#### Selection of the genomic regions used for validation

To assess the accuracy of the estimated frequency of alleles in individual samples based on GBS data, eleven amplicons of ~550 bp encompassing a total of 14 SNP loci that each mapped to a unique position on the *M*. *truncatula* genome (*v4*.*0*) were sequenced on a 454 GS-FLX machine using the Titanium chemistry (Plateforme d’analyses génomiques, IBIS, Laval University, Quebec City, QC, Canada). To minimize ambiguities due to 454 sequencing errors, amplified regions were selected to contain a target SNP and no mononucleotide repeats greater than 5 bp in length in the first 100 bp. Specific primers were designed using the Primer3Plus software [21] and the *M*. *truncatula* (v4.0) genomic sequence as reference. Information on the location of the targeted regions on the *M*. *truncatula* genome (*v4*.*0*) [20], the targeted GBS SNP loci, the expected size of the amplicons and the primer characteristics are summarized in Table 1 and S1 Fig.

**Table 1:** PCR primers with respective Tm (°C) for the amplification of genome regions of *M*. *sativa* covering SNP loci identified with GBS.

Localisation Mt4.0C	GBS SNP loci	Predicted size (bp)	Observed size (bp)	F Primer	R Primer	Tm (°C)
chr2:10386991 10387501	TP67636	511	500	CCACAGGCAGCATTTACC	CACCAGAGTCAAAGCAAAGG	60
chr7:22184910 22185498	TP7278	587	587	CGATTTCCTCCATCTTCTCC	GAACTGCTAAGAGGTTAGGG	59
chr2:44865228 44865752	TP80194 TP79240	525	520	TAAGGGAGAAACAGCAGTGC	GATCCTTGGCAGCTAAGC	60
chr4:40624202 40624738	TP91313	537	520	GCAGAGGGAGATTGAATTCC	TCCGAGAGTCTCTTCATGG	60
chr1:15783990 15783448	TP32628	543	520	GCGAAAAGTTGGGTCTTGG	CATAAGCCTCTTTCCTAGCC	63
chr3:38223672 38224218	TP47889	547	546	CTCCAGTATGCCAGATATGC	TATCCTTCCAGGGTTTGTGG	65
chr2:11380116 11380658	TP61949 TP14949	543	550	TAAGCGGTTTACATTGGC	CCAAAACTTCCTTTCACAGC	59
chr1:38893124 38893685	TP31029	562	560	CTGCTGTTGCGATTAAGAGG	CCAAATGTGCCCATAACTCG	65
chr7:30091020 30091543	TP46847	550	550	CAGCGAGAACTCTTGATCC	CCTTGGGTTCTTACTGTAGC	59
chr7:46607532 46608181	TP17289	563	550	CTCTGGATAAGTGTTCCC	CAGAGCCTGTAATAGACG	53
chr5:8617244 8617764	TP1933 TP26408	520	550	AGCAAAGTCAGCATCTATGG	GGAGTGAGAAATTTGAAGGG	59

Predicted and observed size of amplified fragments and their location on the *Medicago truncatula* genome (*v4*.*0*) are indicated.

#### Resequencing of the selected genomic regions to obtain *M*. *sativa* consensus sequences

PCR amplifications were initially performed on bulked DNA of the same 48 samples from each population (ATF0 and ATF5) to verify the specificity of the 11 amplicons. PCR amplifications were conducted in a total volume of 25 μL in 0.2 mL PCR strips containing 2.5 μL of 10X PCR buffer, 1 μL each of 5 μM primers, 0.5 μL of 10 mM dNTP (Roche Diagnostics, Laval, QC, Canada), 0.5 μL of 5 PRIME GmbH Taq Polymerase 5 U/μL (Inter Medico, Markham, ON, Canada) and 5 μL of 10 ng/μL DNA (50 ng). PCR amplifications were performed on an Eppendorf Mastercycler ep System (Eppendorf Canada, Mississauga, ON, Canada) as follows: 3 min of initial denaturation at 94°C; followed by 40 cycles at 94°C for 1 min, annealing at Tm for 1 min, extension at 72°C for 1 min; and final extension at 72°C for 7 min. The specificity of amplification was confirmed by running 20 μL of each reaction for 2–3 h at 70 V on a 2% agarose gel stained with ethidium bromide and products were visualized using a UVP BioDoc-It system (UVP, Upland, CA, USA). DNA fragments were recovered from agarose gels using the QIAquick gel extraction kit (QIAGEN Inc., Mississauga, ON, Canada). A consensus sequence of each amplicon was obtained by bidirectional Sanger sequencing on an ABI *3130xl* DNA Sequencer (Plateforme d’analyses génomiques, IBIS, Laval University, Quebec City, QC, Canada).

#### 454 sequencing of the amplicons from the selected genomic regions

Amplification of the 11 genomic regions was subsequently performed on a subset of eight plant samples previously analyzed with GBS under the conditions previously described, using PCR primers including universal M13 adaptors. PCR products were purified using the AxyPrep Mag Fragment Select-I Kit (Axygen, Union City, CA, USA), following the manufacturer’s recommendations, and DNA concentration was determined on a Promega GloMax Multi Detection System using the Quantifluor dsDNA kit (Promega, Mississauga, ON, Canada). For each plant sample, the eleven PCR products were pooled in equimolar amount. Pooled PCR products were barcoded for sample multiplexing prior to sequencing on a 454 GS-FLX Titanium system (Plateforme d’analyses génomiques, IBIS, Laval University, Quebec City, QC, Canada). All sequences were submitted to the National Center for Biotechnology Information Short Read Archive under the accession number #SRX1024927.

Reads were separated by barcode and aligned to the Sanger consensus sequence of each amplicon using the Geneious software (v7.1.5, http://www.geneious.com/). The number of 454 reads displaying 64-bp identity with one of the two alleles of the GBS SNP loci was determined. Read counts supporting each allele of GBS SNP loci were also used to calculate allelic dosage (RC of A1/RC of A1+A2) in each sample. Genotype calls based on 454 allelic ratios were compared with those obtained from GBS analysis using the following criteria: heterozygous for samples with 0.1 > allelic ratio < 0.9; homozygous for samples with allelic ratio ≤ 0.1 or ≥ 0.9. GBS alleles with only one read were considered as being absent. In each plant sample, all SNPs present within the first 400 bp of the 454 sequences (0.05 minimum frequency in at least one plant sample) were used to define haplotypes (417 bp were used for TP91313 to include the ApeKI restriction site which generates the GBS fragment). The number of 454 reads supporting each haplotype with one allele of the GBS SNP loci (64bp identity with the GBS sequences) and a 0.05 minimum frequency in at least one plant sample were used to determine haplotype counts for each amplicon. Sequences containing additional SNPs or indels in the 64 bp of the GBS sequence were analyzed separately using the same criteria.

### Comparison of populations

The plant sample distribution in the multivariate space defined by genotypes at SNP loci was inferred by Principal Component Analysis (PCA). PCA was performed with the covariance method (TASSEL v4.3.5) using the initial dataset obtained after the application of whole dataset-level filters and the subset of SNP loci retained after additional genotype-level filtration. Individual genotypes were converted into numerical data using TASSEL with the “Collapse Non Major Alleles” method. Missing data were imputed using unweighted Manhattan distance for samples with maximum missing data frequency set to 0.5 for both datasets. A graphical representation of the sample distribution along the first three axes of PCA was achieved using the PCA 3D Visualiser developed by Prism Training & Consultancy (http://www.prismtc.co.uk).

## Results

### SNP discovery

Sequencing of 96 alfalfa genotypes on two lanes of an Illumina HiSeq yielded ~396 million reads (Table 2). After trimming to 64 bp, analysis of GBS data with the UNEAK pipeline retained ~370 million sequences devoid of ambiguities. These good reads were grouped into ~15.4 million distinct tags, of which ~1.9 million good tags were supported by more than five reads. Nearly 645,000 groups of tags that differ by a single nucleotide were identified, including 97,508 SNP loci putatively located at a single locus. A subset of 73,437 SNP loci with MAF ≥ 0.05 was retained, of which 7,438 having a call rate ≥ 0.5. The number of good reads per plant sample averaged about 3.8 million, but this number was highly variable between samples (380 reads to 4.9 million reads; data not shown). A subsequent UNEAK analysis excluding 24 plant samples (13 from ATF0 and 11 from ATF5) with insufficient read coverage (< 1 million reads) did not noticeably affect the number of good tags (~1.9 million) and SNP loci (95,775) while increasing by 57% the number of SNP loci (11,694) that met the quality filter requirements (MAF ≥ 0.05 and mnC ≥ 0.5) (Table 2, 72 samples). Sequences of both alleles of the 11,694 SNP loci are listed in S1 Table.

**Table 2:** Summary of GBS analysis of alfalfa samples with UNEAK in the complete 96 samples dataset and the subset of 72 samples with > 1million reads.

		96 samples	72 samples
**Illumina sequencing**	**Total Reads**	396,675,286	
**UNEAK Analysis**	**Good Reads**	371,308,770	363,877,063
	**Total Tag**	15,467,219	15,199,465
	**Good Tags**	1,899,657	1,867,892
	**Tag Pairs**	645,553	636,705
	**Reciprocal Tag Pairs**	97,508	95,775
	**SNP loci (MAF 0.05)**	73,437	72,438
	**SNP loci (MAF 0.05 + mnC 0.5)**	7,438	11,694

**Fig 1:**
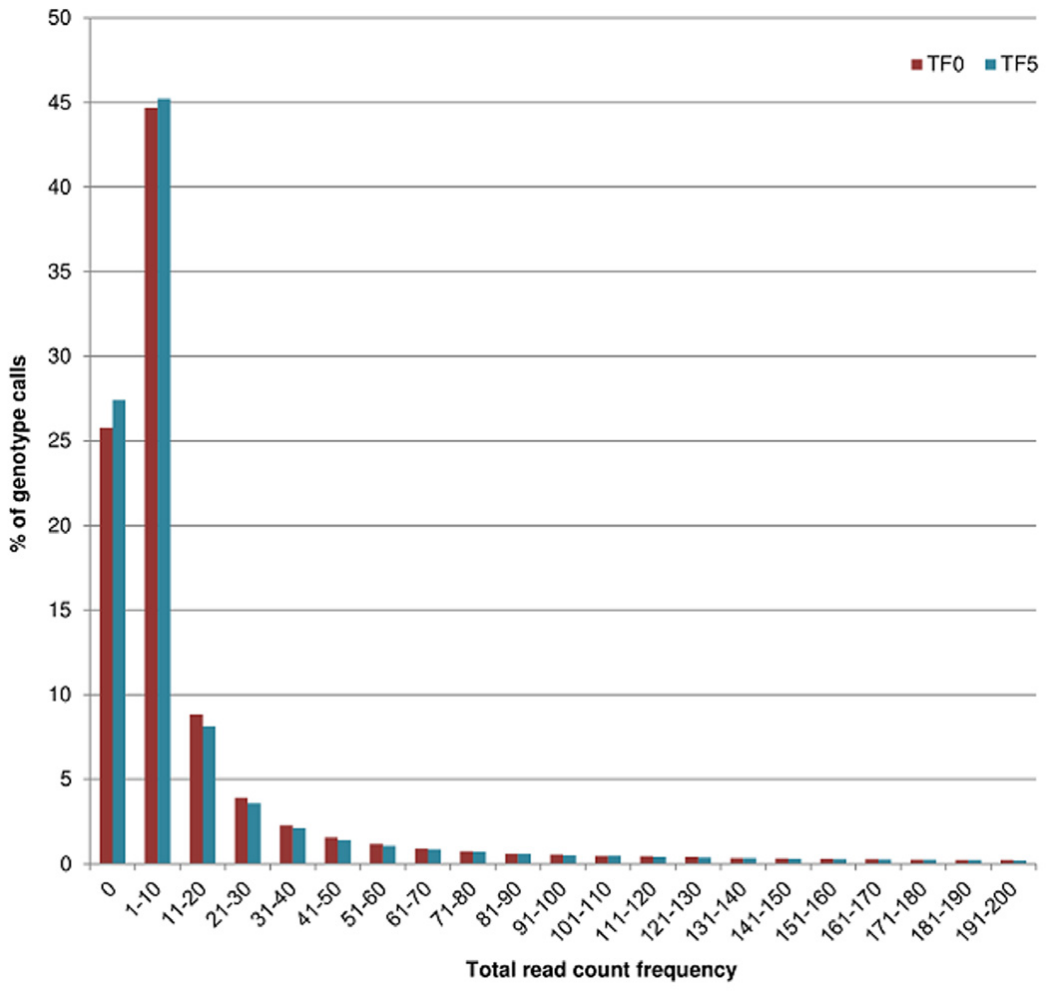
Observed frequency distribution of total read counts (A1+A2) supporting each genotype call in ATF0 and ATF5 populations. Genotype calls (5%) supported by >200 reads are not shown.

A comparative analysis of UNEAK results for ATF0 and ATF5 after the application of whole dataset-level quality filters (MAF ≥ 0.05 and mnC > 0.5) showed that SNP loci were supported by a similar number of total reads per sample (~500,000 to 570,000) or per locus (~1,600 to 1,700) in each population (Table 3 –Whole dataset-level filters). Depth of sequencing was highly variable between samples (~213,000 to ~1,175,000) and SNP loci (17 to 450,000) but the range was comparable between the two populations (S2 Table). The frequency of missing data per locus was also similar between the two populations (0.26 in ATF0 and 0.27 in ATF5), even if some SNP loci had missing genotype calls in over 78% of the samples in one of the two populations. A large proportion of genotype calls were either missing or supported by 1 to 10 reads (70% and 73% of genotype calls in ATF0 and ATF5, respectively), while 22% (ATF5) and 24% (ATF0) of genotypes calls were supported by 11 to 200 reads (Fig 1). A low proportion (~5% in both populations) of genotype calls were supported by high read counts (200 < RC < 25000) (data not shown). Finally, a large and similar proportion of homozygous genotype calls (about 80%) was observed in both populations (Table 3 –Whole dataset-level filters).

**Table 3:** Summary statistics of GBS analysis of two alfalfa populations with UNEAK using whole dataset-level filters^1^ and genotype-level filters^2^.

		Whole dataset-level filters ^(1)^		Genotype level-filters^(2)^	
		ATF0	ATF5	ATF0	ATF5
**SNP loci**		11,694		2,732	
**Plant samples**		35	37	35	37
**Total reads**		19,967,228	18,548,856	12,469,118	11,701,875
**Reads / sample**		570,492	501,320	358,053	317,715
**Reads / locus**		1,707	1,586	4,587	4,303
**Overall dataset**	**Homozygote frequency**	0.78	0.79	0.67	0.67
	**Heterozygote frequency**	0.22	0.21	0.33	0.33
	**N frequency**	0.26	0.27	0.27	0.28

The total number of reads in ATF0 and ATF5 populations, mean counts of reads per sample and SNP loci are reported. Homozygous, heterozygous and missing genotypes frequencies were calculated for each population. (Additional descriptive statistics are presented in S2 Table).

^(1)^ 72 samples with > 1million reads, MAF>0.05, mnC>0.5;

^(2)^ 72 samples with > 1million reads, MAF>0.05, mnC>0.5, RC≥ 11 for homozygous genotypes, RC≥2 reads of each allele (A1 and A2) for heterozygous genotypes, 0.1 ≤RC_A1_/RC_A1+A2_ ≤ 0.9.

A total of 2,732 SNP loci were retained after the application of a genotype level-filter to the initial 11,694 SNP loci dataset (Table 3 –Genotype-level filters). This additional quality filter slightly increased the frequency of missing data (0.27 in ATF0, 0.28 in ATF5) and decreased the number of total reads and reads per sample in each population (~320,000 to ~360,000). However, an increased number of reads per SNP locus (~4,300 to ~4,600) was observed in both populations. Variability of sequencing depth remains large but was markedly reduced both between samples (~135,000 to ~690,000) and SNP loci (158 to ~101,000) (S2 Table). The proportion of homozygosity decreased slightly to about 67% in both populations.

### SNP location on *M*. *truncatula* reference genome

About 60% of the 11,694 SNP loci had significant matches (E-value < 1 x 10^-8^) on the *M*. *truncatula* genome v4.0, including 5,952 SNP loci with a single hit and 1,110 SNP loci with multiple hits (Table 4). With the exception of chromosome Mt6, with only 241 single-hit SNP loci, *M*. *truncatula* chromosomes (Mt1 to Mt8) were covered with many hundred SNP loci (682 to 949). On average, a single-hit SNP loci was observed every 55 to 145 Kb. Finally, 88 single-hit SNP loci aligned to unmapped genome fragments of the *M*. *truncatula* genome.

**Table 4:** GBS SNP loci with significant homology (E value < 1 x 10^8^) with *Medicago truncatula* reference genome (*v4*.*0*).

Nb Hit	Chromosome	Size (bp)	Nb SNP loci
1	Mt1	52,787,282	949
	Mt2	45,459,969	748
	Mt3	55,424,720	853
	Mt4	56,509,316	967
	Mt5	43,527,414	682
	Mt6	34,898,058	241
	Mt7	48,921,887	704
	Mt8	45,078,774	720
	Scaffolds		88
>1			1110
Total	Located		7,062
	Unlocated		4,632

Counts of single hits on individual chromosomes and number of individual SNP with multiple hits are shown. Number of located and unlocated SNP loci among 11,694 SNP loci are also indicated.

### SNP resampling between *M*. *sativa* germplasms

About 50% (5,760) of the 11,694 SNP loci identified in the present study with the Apica germplasm were also found in the GBS analysis of a mapping population described [12] (S1 Table), and 935 of them are among those they used to build their genetic map of *M*. *sativa*. Among the shared loci, 4,743 (82%) had a perfect sequence identity for both alleles, and 1,017 (18%) had an identical sequence for one allele and a variant sequence for the second one.

### SNP validation

#### Resequencing of selected genomic regions

Eleven genomic regions covering 14 GBS-derived SNP loci were first amplified using bulked DNA of populations ATF0 and ATF5 and then analyzed by Sanger sequencing. Each primer pair yielded a single amplification product of the expected size (Table 1). Alignment of the *M*. *sativa* consensus sequences of these products with their respective *M*. *truncatula* reference sequences showed high similarity (E-value ≤ 2 x 10^-170^), confirming that the amplification products matched the targeted regions (S2 Fig).

**Fig 2:**
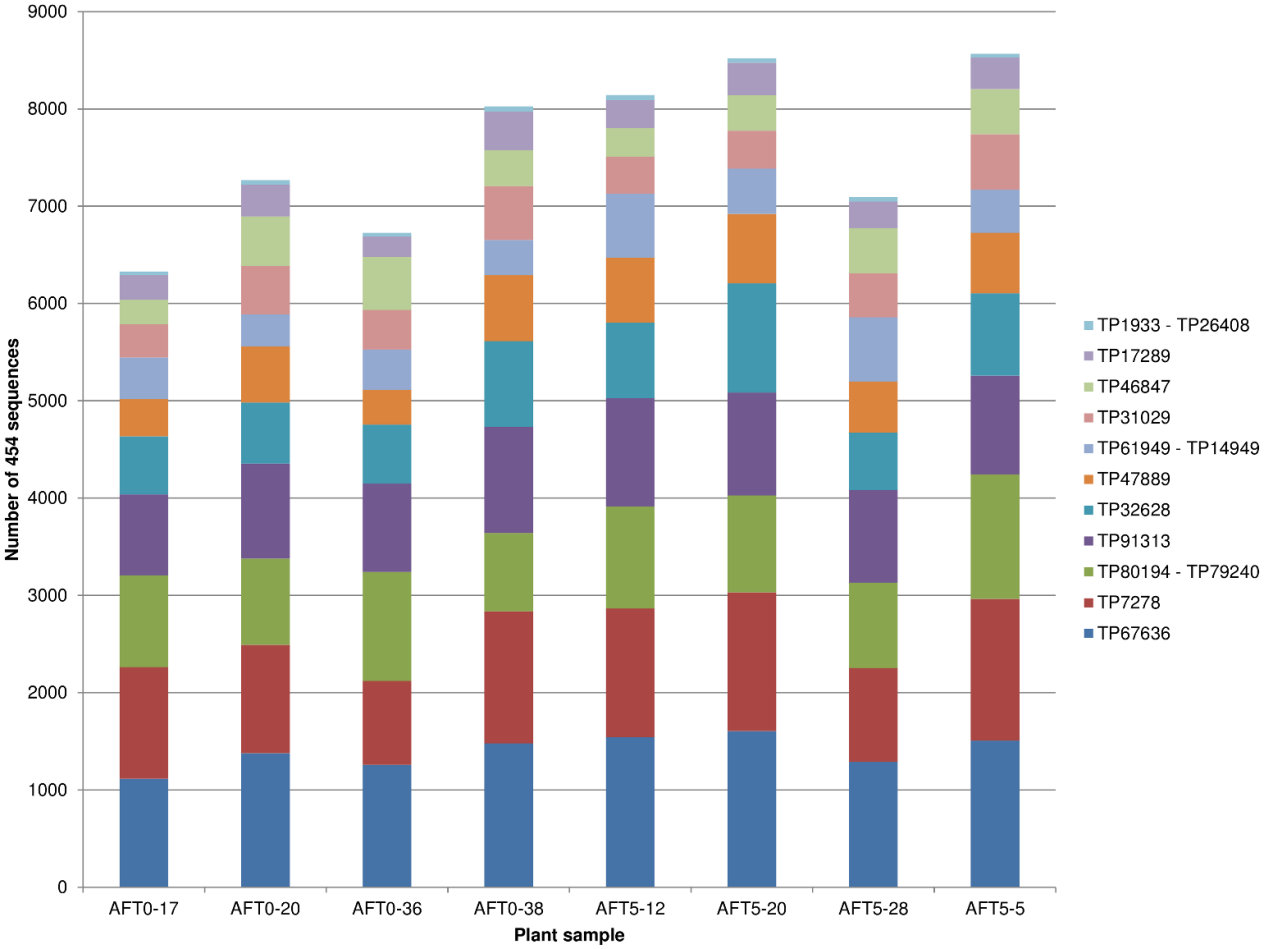
Number of 454 sequences retained for each of 11 amplicons covering 14 SNP loci identified with UNEAK in eight plant samples.

Approximately 80,000 reads were obtained by 454 sequencing of the amplification products of these 11 genomic regions from eight samples. After alignment with the Sanger consensus sequences, 60,684 (~75%) sequences were retained for further analysis. Rejected sequences were mostly short reads (about 90% ≤ 200 bp) (data not shown). Similar sequencing depths were observed between samples (6,329 to 8,567 reads/sample; Fig 2), whereas a large variability in the number of reads was observed between amplicons (362 to 11,172 reads/amplicon; Table 5).

**Table 5:** Results of 454 sequencing of regions covering 14 SNP loci identified with UNEAK.

Tag Pair	TP67636	TP7278	TP80194 TP79240		TP91313	TP32628	TP47889	TP61949 TP14949		TP31029	TP46847	TP17289	TP1933 TP26408	
Nb of 454 sequences	11,172	9,647	7,960		7,939	6,055	4,521	3,759		3,591	3, 266	2,412	362	
% of 454 sequences supporting GBS SNP loci	48%	40%	53% ^(1)^		68%	8%	78%	59% ^(1)^		75%	70%	83%	37% ^(1)^	
			15% ^(2)^	21% ^(2)^				5% ^(2)^	24% ^(2)^				19% ^(2)^	20% ^(2)^
SNP / 400 bp	31	43	8		12	16	6	6		8	9	5	4	
Total 454 haplotypes	8	9	9	10	7	11	6	10	7	11	5	7	8	8
GBS-like haplotypes^(3)^	7	5	8	10	5	2	6	10	6	9	5	7	7	8
Maximum GBS-like haplotype / plant sample	4	2	5		3	1	3	4		5	4	4	4	

Number of 454 sequences retained for each amplicon and proportion of those sequences with a perfect 64 bp alignment with the GBS read are presented. The number of SNP in the first 400 bp of 454 sequences are also indicated. Total number of haplotypes and maximum number of haplotypes in individual plant samples were determined using SNPs present in 400 bp.

^(1)^ Contains both SNP loci

^(2)^ Contains one single SNP locus

^(3)^ Haplotypes with 64bp identity with GBS alleles

**Fig 3:**
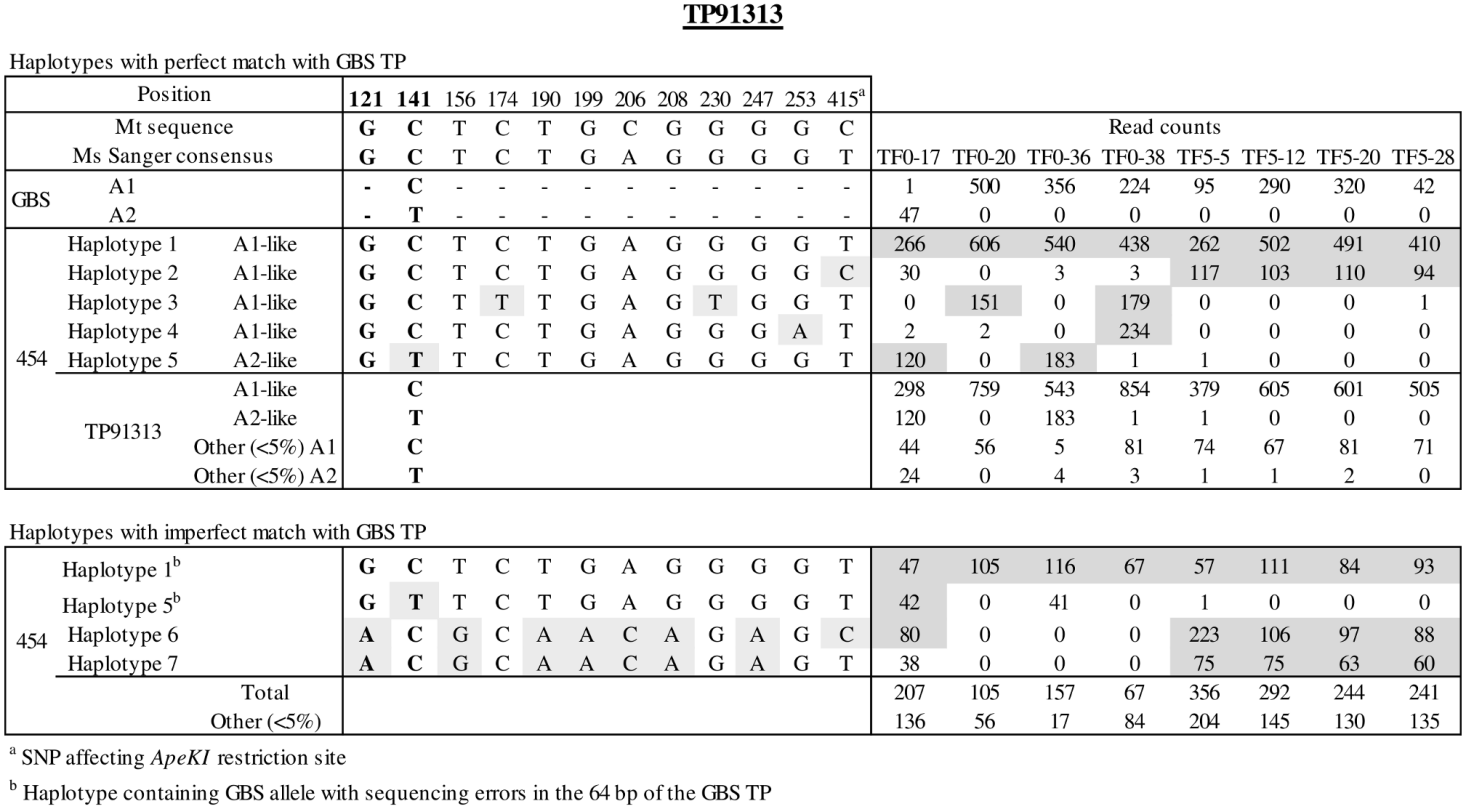
Haplotypes identified with 454 sequences covering TP91313 in eight plant samples. Haplotypes defined with 454 sequences with perfect and imperfect match with GBS 64bp sequence are listed separately. Position of SNPs is based on location on *M*. *truncatula* reference sequence. SNPs included in UNEAK TP are highlighted in bold. RC of GBS alleles (A1 and A2) and 454 sequences covering each haplotype in the eight genotyped plant samples are indicated. Cumulative number of A1 like and A2 like reads are also presented. SNPs with RC ≥ 5% in individual plant samples were used to define haplotypes. Haplotypes with frequency < 5% in all individual plant samples are not indicated but total read counts supporting those other haplotypes are reported. Haplotypes corresponding to each of the 14 GBS loci are presented in S3 Table.

A variable number of SNPs (4 to 43) were detected in the first 400 bp of the retained sequences and were used to define haplotypes as illustrated in Fig 3, S2 Fig and S3 Table. Among those SNPs, ten are affecting *Ape*KI restriction sites, including five affecting sites that generates GBS reads (TP7278, TP80194, TP91313 and TP31029; S2 Fig). Up to eleven different haplotypes per amplicon were detected among the eight plant samples. However, in most cases no more than four haplotypes containing GBS alleles were predicted in any given plant sample, except for TF5-5 in TP80194 and TP79240, TF0-36 in TP79240 and TF5-20 in TP31029.

About 60% (36,233 reads) of the 454 sequences had a perfect 64-bp identity with one or the other allele of the 14 SNP loci used for validation. This proportion was highly variable depending on the considered SNP loci (8% to 83%, Table 5). Haplotype analysis revealed that among the 40% sequences having an imperfect match with the targeted SNP loci, ~30% corresponded to sequences of the targeted GBS alleles with sequencing errors (SNP causing imperfect match with a frequency <5% in all plant samples) while ~10% were additional alleles or putative paralogs. Alleles identified with GBS were shared by several haplotypes (e.g. 4 haplotypes with GBS Allele 1 for TP91313 in Fig 3). On the other hand, some haplotypes defined alternative alleles either not sampled by GBS or not retained by UNEAK (haplotypes 6 and 7 in Fig 3). A comprehensive description of haplotypes identified for the 14 GBS loci and their alignment with *M*. *truncatula* reference sequence is provided in S3 Table and S2 Fig.

#### Comparative analysis of GBS and 454 genotype calls based on tetraploid or diploid allelic ratios

**Fig 4:**
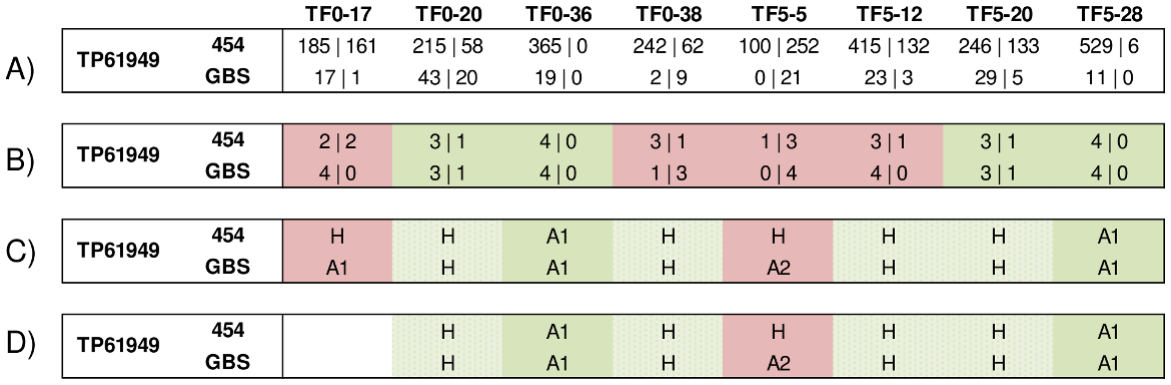
Example of comparison of GBS and 454 sequencing of TP61949 in eight plant samples. A) GBS and 454 read counts of each allele (A1|A2); B) predicted tetraploid allelic ratios (convergent ratios in green and discordant ratios in red); C) bi-allelic predicted genotype (A1, A2 and H) before genotype-level filtration and D) after genotype-level filtration of GBS data for minimum read counts (11 reads for homozygous genotypes, 2 reads of each allele for heterozygous genotypes, 0.1 as minimum minor allele frequency). Genotype calls showing concordance (green), discordance (red for GBS homozygotes and orange for GBS heterozygotes) with both sequencing methods or that are missing (white) before and after genotype-level filtration for minimum read counts. A complete representation of validation results for 14 SNP loci in eight plant samples is provided in S3 Fig.

Genotypes were called based either on tetraploid or diploid allelic ratios of read counts of SNP loci determined with the two sequencing methods, as illustrated for TP61949 in Fig 4. Genotypes calls obtained with both approaches were either concordant or discordant. Half of the genotypes called based on a tetraploid allelic ratio obtained with GBS and 454 sequencing concurred (Table 6). Of these concordant calls, 80% were homozygous (4|0 and 0|4) and 20% were heterozygous (3|1, 2|2 or 1|3). Discordant tetraploid allelic ratios were on average supported by lower GBS RC ($\overset{-}{RC}$ = 58) than concordant allelic ratios ($\overset{-}{RC}$ = 72) (S3 Fig).

**Table 6:** Observed consistency of genotype calls obtained with GBS and 454 sequencing of 14 SNP loci in eight plant samples.

Status	GBS	454	Tetraploid genotype calls	Diploid genotype calls before correction	Diploid genotype calls after correction
Concordant	Homozygous		45	44	36
	Heterozygous		12	41	37
	Total		57 (51%)	85 (75%)	73 (65%)
Discordant	Homozygous	Heterozygous	22	17	4
	Heterozygous	Homozygous	7	8	8
	Heterozygous (different ratio)		24	-	-
	Total		53 (47%)	25 (23%)	12 (11%)
Missing			2 (2%)	2 (2%)	27 (24%)

The number of homozygous and heterozygous genotype calls showing concordance, discordance with both sequencing methods or that are missing are indicated for tetraploid allelic dosage and diploid genotype call before and after genotype level filtration. Percentages of consistent and discordant observations are indicated in brackets.

On the other hand, genotype calls based on a diploid allelic ratio were in agreement (GBS *vs*. 454) in more than 75% of cases (Table 6). More than half of concordant calls (52%) were homozygous. By contrast, 70% of the discordant calls came from heterozygous 454 genotypes being called as homozygous genotypes in the GBS analysis. Observed discrepancies often arose from genotypes with low GBS RC for one or both alleles or from unbalanced RC of both alleles (S3 Fig). The application of the genotype-level filters of Li et *al*. (2104) increased the proportion of concordant *vs*. discordant genotype calls between the two sequencing methods (Table 6). However, as a result of this filtration, 13 of the 25 discordant calls and 12 of the 85 concordant calls were assigned to the missing category.

### Population diversity analysis

**Fig 5:**
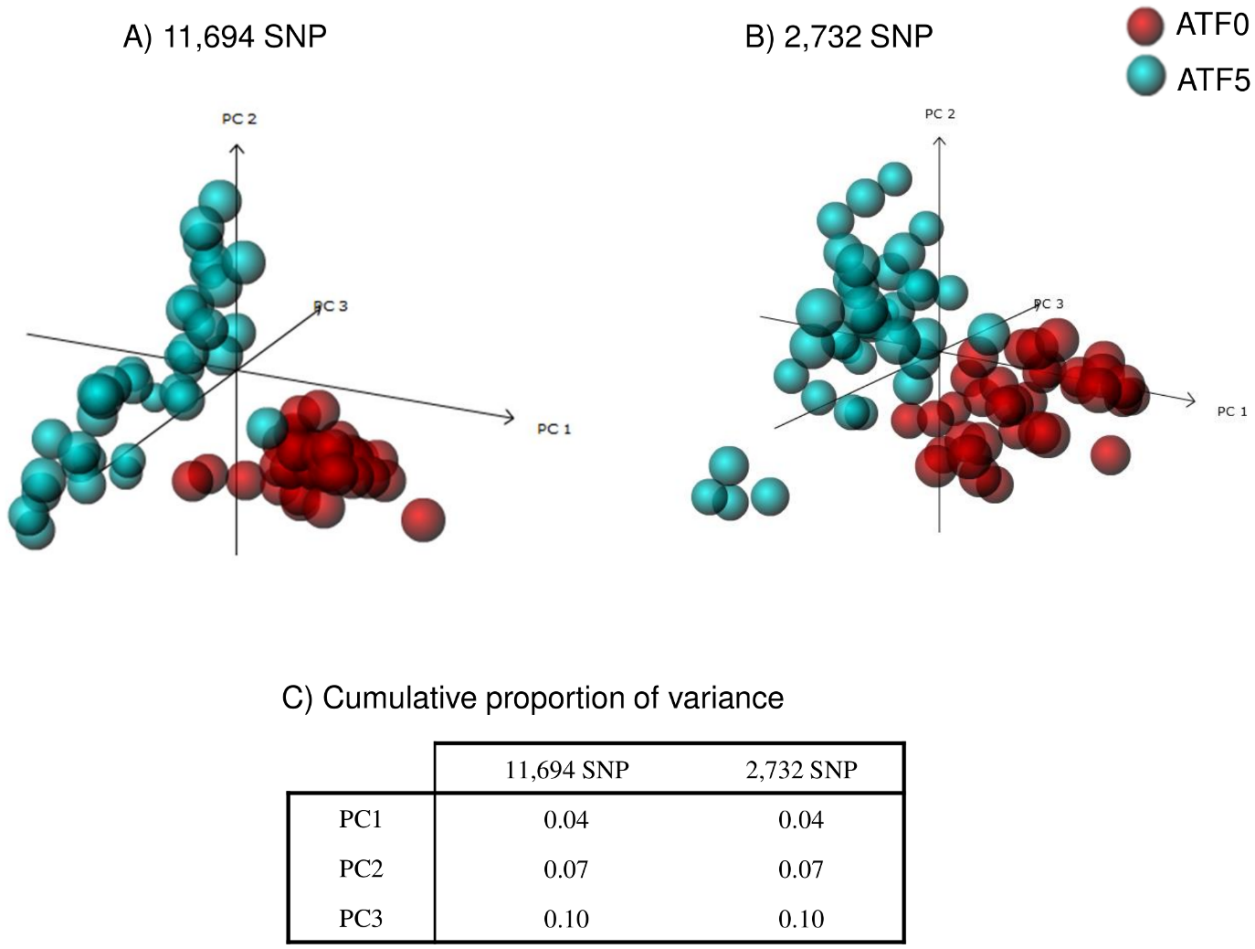
3D representation of PCA of 72 plant samples (35 ATF0 and 37 ATF5) genotyped with two SNP loci datasets A) 72 plant samples genotyped with 11,694 SNP loci and B) 72 plant samples genotyped with 2,732 SNP loci retained after genotype-level filtration for minimum read counts C) Cumulative proportion of variance explained by the first three components in the two SNP loci datasets.

Principal component analysis of plant sample distribution in the multivariate space using 11,694 SNP loci showed a clear separation between ATF0 and ATF5 along the first axis, and a slightly higher dispersion of members of the ATF5 population when compared to ATF0 (Fig 5A). PCA performed with the 2,732 SNP loci retained after genotype-level filtration showed a similar discrimination between the two populations (Fig 5B). It is noteworthy that in both datasets, only 4% of the variability within the SNP loci is explained by the first axis, and 10% of the variance is explained by the first three components. A large proportion of the genetic variability remains unstructured (Fig 5C).

## Discussion

Genetic analysis of open-pollinated polyploids is challenging in numerous respects including large genomes, complex inheritance and lack of genomic resources. This paradigm is rapidly evolving thanks to recent technological developments in high throughput sequencing, which make genome-wide marker coverage accessible at a reasonable cost even in these orphan species [11,22,23]. However, analysis of high throughput sequencing data in polyploid species without a reference genome remains a daunting task. The recent development of the UNEAK pipeline [13] offers new opportunities for the analysis of GBS data and genome-wide sampling of SNP loci in species without a reference genome, even in polyploid species like alfalfa. The application of network filters eliminates most SNPs that belong to repeats and paralogs and significantly increases the accuracy of SNP calling in the absence of a reference genome. Despite this, in the absence of marker validation based on their Mendelian behaviour (e.g. obtained by testing for proper segregation in the progeny of a biparental cross), other approaches are needed to validate the reliability of GBS data.

### SNP discovery in heterogeneous populations of alfalfa using GBS

Two lanes of Illumina HiSeq 2000 sequencing yielded nearly 370 million high quality reads from an *Ape*KI library. This number is comparable to a recent GBS analysis of an alfalfa mapping population using a similar approach [12]. Even though we used selective primers to achieve further genome reduction and increase the depth of coverage [19], we still noted a high proportion of missing genotype calls as observed in other GBS analyses [12,24,25]. Numerous biological (presence-absence variation, polymorphic restriction sites or methylation patterns among others) or technical issues (library complexity and sequence coverage) can explain the high rate of missing genotypes in GBS analyses [23,26].

Elshire et *al*. [11] observed an important variation of read coverage between samples in a GBS analysis of maize (*Zea mays*) RILs and doubled haploid barley (*Hordeum vulgare*) lines. Similarly, we observed considerable variation in the number of reads per sample even if no consistent explanation of this uneven coverage was identified in our study. However, samples with low read counts had a major impact on the number of SNP loci that were retained after filtering out loci with high number of missing genotypes. The exclusion of samples with less than 1 million reads resulted in a 30% increase in the number of SNP loci that were retained after filtration. This highlights the importance of read depth and homogenous sequencing among individuals for an optimal analysis of GBS data without a reference genome.

The distribution of read counts per SNP locus was not uniform either, and a large proportion of genotype calls in both populations were supported by low read counts (10 or less). The distribution frequency of read counts supporting genotype calls in our populations was similar to the observation of highly variable genome coverage at distinct loci in RILs of maize [15] and genotypes of hops (*Humulus lupulus* L.) [27]. The uneven distribution of reads among sites is not completely resolved, but was attributed to low-representation of fragments with GC content outside a 10–70% window, and over-representation of fragments from organellar DNA and repeats [15].

A large proportion of the ~640,000 tags that were found to differ by a single nucleotide from another tag were also able to form networks with additional tag variants, which was expected in a population of highly heterozygous tetraploid alfalfa. The main limitation of genetic analysis in complex polyploid genomes is the capacity to distinguish true alleles at a single locus from those arising from paralogs and duplicates [22]. Using UNEAK, a data analysis algorithm designed for identification of single loci in GBS data, 84% of the 640,000 tags were considered as originating from paralogs, repeats or sequencing errors and were discarded. The effectiveness of duplicate elimination is supported by the observation that 90% of the SNP loci (tag pairs) that aligned with the *M*. *truncatula* genome (v4.0) had a single match. This is in agreement with the observation that more than 85% of the SNP loci retained by UNEAK aligned with single locations in the maize genome [13].

### Haplotype diversity behind GBS alleles

We used haplotype analysis based on 454 sequences to assess how the complexity behind GBS SNP loci such as repeats, paralogs and sequencing errors is handled by UNEAK in complex populations of tetraploid alfalfa. Although a large proportion of the 454 sequences (60%) showed a perfect match with one of the targeted SNP alleles, nearly 30% of the sequences imperfectly matched GBS alleles due to sequencing errors while 10% were either additional alleles or paralogs. This high proportion of sequence with an imperfect match is due to the combination of our requirement for a 64 bp identity with GBS alleles and 454 tendency to generate artefactual SNPs or indels particularly in regions with mono-nucleotide repeats. Notwithstanding, the elimination of sequences with an imperfect match had no major impact on the representation of haplotypes containing GBS alleles, except for TP32628 where the presence of a “CCYCC” motif generated more frequent 454 sequencing errors in allele 2 than in allele 1 (S2 Fig and S3 Table).

Analysis of 454 sequences revealed a large haplotypic diversity underlying GBS SNP loci, underscoring the fact that bi-allelic sampling is a simplified representation of genetic diversity in heterogeneous populations of tetraploid alfalfa. Up to eleven different haplotypes at a given targeted SNP loci were identified in eight plant samples. This either reflects allelic diversity at a single locus or the presence of paralogs. The fact that four haplotypes or less were present in all individual plant samples for most GBS loci (maximum of one haplotype per homologous chromosome) supports the efficiency of UNEAK to reduce false SNP calls from paralogs. We nevertheless observed cases where GBS alleles shared by paralogs could not be eliminated by UNEAK (e.g. TP32628 in S3 Table).

We identified haplotypes defined with 454 that were not sampled by GBS in at least three loci (TP7278, TP80194 and TP91313). This occurred when a SNP within an ApeKI restriction site prevented sampling of those haplotypes. For instance, mutations at positions 131 and 242 in the sequence covering TP7278 excluded haplotypes 1, 2, 3 and 5 from read counts for that TP. Although mutations of ApeKI restriction sites impacted allelic dosage at a tetraploid level, they seldom altered biallelic genotype calls.

Conversely, there were instances of unambiguous heterozygous genotypes calls based on GBS that were not confirmed by 454 re-sequencing (e.g. TP46847). This shows the limitations of 454 re-sequencing approach as a validation for GBS analysis. It should be kept in mind that the use of a diploid genome (*M*. *truncatula*) to design locus-specific PCR primers for 454 re-sequencing of a tetraploid relative (*M*. *sativa*) could provide a partial representation of the existing diversity. For instance alternative alleles displaying polymorphisms in the annealing regions of the primers may not have been sampled or may have been less efficiently amplified.

### Validation of GBS genotype calls

We used 454 sequences with a perfect match with GBS alleles to directly validate allelic ratios and genotype calls based on GBS using a subset of SNP loci. Only 50% of GBS estimates of tetraploid allelic ratios (4|0; 3|1; 2|2; 1|3 or 0|4) were validated using 454 sequences having a perfect match with the targeted SNP loci. Aside from the previously reported 454 limitations, the low concordance of allelic ratio estimates is mainly attributable to insufficient GBS read depth in an outcrossed polyploid, and to some extent to allele missampling. In heterogeneous cultivars of tetraploid potato, it has been estimated that a sequencing depth of 60-80x is required for reliable dosage [28]. As most of our SNP loci did not meet this criterion, we used genotype calls based on diploid allelic dosage to distinguish between homozygous and heterozygous samples. Using that approach, consistency between the two sequencing methods increased to 75%. Our results showed that the remaining discrepancies in genotype calls often occur in homozygous genotypes supported by low GBS read counts. In a non-inbred tetraploid species the probability of miscalling a heterozygote as a homozygote using GBS is high compared to diploids when using only whole dataset-level filters [12]. The application of the genotype-level filter proposed by [12] markedly reduced genotype miscalling in our validation dataset, but had a huge impact on the proportion of missing genotype calls and thus on the overall size of our final dataset. Indeed, after application of this genotype-level filter to the entire GBS dataset, only 23% of 11,694 SNP loci were deemed as having reliable genotype calls in at least 50% of plant samples. Direct 454 sequencing of our validation SNP subset supports previous reports that stringent filtering of GBS data is required for reliable genotype calls in non-inbred polyploid species, particularly in cases of limited coverage [12,16].

### Transferability of SNP loci

Sixty percent of the 11,694 SNP loci retained by UNEAK were found to align on the *M*. *truncatula* genome at an E-value < 1 x 10^-8^. This proportion rose to 72% when considering the filtered set of 2,732 SNP loci. Using a less stringent homology requirement (E-value < 1 x 10^-5^), as much as 83% of 3,769 SNP loci from GBS analysis of an alfalfa mapping population could be aligned with the *M*. *truncatula* reference genome [12]. In *Populus*, digestion with *Ape*KI captured loci in coding regions more frequently than expected from chance alone [16], and confirms its utility for preferential representation of differences in coding regions. In our study, it is also more likely that a large proportion of the hits are located in highly conserved exons than in poorly conserved non-coding regions [29]. These results are a further confirmation of the high co-linearity between the *M*. *sativa* and *M*. *truncatula* genomes, which can be exploited for functional and comparative genomic studies [9,12,30,31]. In that perspective, *M*. *truncatula* could be used as a pseudo-reference genome to further explore haplotype diversity in populations of alfalfa by exploiting GBS reads with multiple SNPs that were not retained by UNEAK.

It should be noted that with a comparable sequencing depth, there was a 3-fold higher number of SNP loci (~300,000) retained by UNEAK analysis of the narrow-based mapping population of [12] than that observed in the broad-based Apica cultivar (~95,000). This reflects the impact of haplotype diversity on the number of SNPs filtered out by UNEAK. About 50% of the 11,694 SNP loci identified in the Apica background were also found in the mapping population [12]. Among these, 82% were identical for both alleles while 18% differed for one allele. This level of SNPs resampling between studies conducted with unrelated genetic material and a limited coverage of highly heterogeneous alfalfa is noteworthy as it shows the transferability of markers between alfalfa populations. This is confirmed by the localization of almost 1,000 of our SNP loci on the tetraploid map of alfalfa constructed with 3,591 single-dose SNP loci [12]. In that perspective, GBS is a promising tool to develop affordable and accurate genomic resources currently lacking in alfalfa.

### Population diversity

Alfalfa is a highly heterozygous species with larger intra- than inter-varietal genetic diversity [2,32]. In our study, we observed a high level of homozygosity within an heterogeneous genetic background using bi-allelic genotype calls based on GBS SNP loci. This is partly due to the simplification of genetic complexity inherent to the selection of short reads with a single point mutation by UNEAK. Indeed, higher intra-population variability was revealed by 454 sequencing of regions covering a subset of SNP loci in 8 plant samples, which shows the presence of several haplotypes (up to ten) covering each GBS locus.

In spite of the simplification of genetic diversity, our study supports previous reports that UNEAK analysis of GBS is an efficient tool to track allele variability in single genotypes or complex populations [12,13]. We analyzed the impact of recurrent selection on diversity within populations using multivariate analysis of genome wide distributed SNP loci. The relatively low proportion of variance explained by the first three component of PCA (10%) indicates no significant structure in our dataset, as expected from random sampling of SNP loci by GBS analysis of broad-based alfalfa populations. It also suggests that recurrent selection did not create a genetic bottleneck in our population. However, we observed that genotypes of both populations (ATF0 and ATF5) were not randomly distributed along the first PCA axis even though it explained only a small proportion of the overall variability. This differentiation reflects selection history, and indicates that recurrent selection had a slight but detectable effect on genome composition in ATF populations, as previously observed with SRAP markers [7]. We observed comparable distribution of ATF0 and AFT5 populations in the multivariate space before and after the application of stringent genotype-level filters. This suggests that erroneous genotype calls that remained after the application of whole dataset-level filters were buffered by a sufficient number of reliable observations in each population. A differentiation between five groups of *Solanum tuberosum* cultivars uncovered by PCA of sequence variants was also associated with a limited proportion of the total genetic variance [28]. Populations of perennial ryegrass (*Lolium perenne* L.) were also differentiated by GBS analysis of allele frequencies at a large number of SNP loci across the genome [14]. These authors concluded that GBS analysis of bulked DNA can be used to directly evaluate populations of outbreeding species on a genome-wide scale. This should also be a suitable approach to identify genomic regions under selection pressure in populations of alfalfa under recurrent selection.

## Conclusions

Our results confirm that GBS analysis with the UNEAK pipeline, developed for SNP discovery in diploid species without a reference genome, is suitable for the analysis of complex populations of autotetraploid alfalfa. Using direct validation of GBS by 454 sequencing, we have shown that although allele missampling and limited read depth does not allow accurate determination of tetraploid allelic dosage, accurate genotype calling simplified to a diploid state can be achieved. The reliability of genotype calls is strengthened by the combination of whole dataset- and genotype-level filters. We observed a clear differentiation between populations that was associated with a small portion of the overall genome-wide SNP variability. This suggests that a bulked DNA analysis of allelic imbalance could be an effective approach to identify genomic regions under selection pressure. This will be an important step towards the identification of polymorphisms associated to phenotypic variability in populations of open-pollinated alfalfa.

## Supporting Information

S1 Fig *M*. *truncatula* reference sequences used for 454 validation of 14 GBS SNP loci. Location of PCR primers used for amplification, *Ape*KI restriction sites, GBS fragment and 64 bp sequence obtained after UNEAK analysis are indicated.
(PDF)

S2 Fig Alignments of haplotypes defined within the first 400bp of 454 sequences against the 11 targeted genomic regions of *M*. *truncatula* and Sanger consensus sequences obtained from amplified DNA fragments of *M*. *sativa*. Sequences covering GBS SNP loci (64 bp sequence) are also aligned. SNPs used to define haplotypes, *Ape*KI restriction sites and PCR primer annealing regions are indicated.
(PDF)

S3 Fig Comparison of GBS and 454 sequencing of 14 SNP loci in eight plant samples. A) GBS and 454 read counts of each allele (A1|A2); B) predicted tetraploid allelic ratios, with ratios identified as convergent in green and discordant in red; C) bi-allelic predicted genotype (A1, A2 and H) before genotype-level filtration and D) after genotype-level filtration of GBS data for minimum read counts (11 reads for homozygous genotypes, 2 reads of each allele for heterozygous genotypes, 0.1 as minimum minor allele frequency). Genotype calls showing concordance (green), discordance (red) with both sequencing methods or that are missing (white) before and after genotype-level filtration for minimum read counts.
(PDF)

S1 Table Sequences (64bp) of the 11694 SNP loci obtained after the application of whole dataset-level (D) and genotype-level (D+G) filters. Number of hits on *M*. *truncatula* (v4.0) genome and chromosome assignation on *M*. *truncatula* according to [20] and *M*. *sativa* according to [12] are provided when available.
(PDF)

S2 Table Summary statistics of GBS analysis of two alfalfa populations with UNEAK using whole dataset-level filters and genotype-level filters. The total number of good reads in ATF0 and ATF5 populations, and the maximum, mean and minimum counts of good reads per sample and SNP loci are reported. Homozygote, heterozygotes and missing genotypes frequencies were calculated for each population.
(TIF)

S3 Table Haplotypes identified with 454 sequences covering 14 GBS SNP loci using SNPs with RC≥5% in individual plant samples. Haplotypes defined with 454 sequences with perfect and imperfect match with GBS 64bp sequence are listed separately. Position of SNPs is based on location on *M*. *truncatula* reference sequence. SNPs included in UNEAK TP are highlighted in bold. RC of GBS alleles (A1 and A2) and 454 sequences covering each haplotype in the eight genotyped plant samples are indicated. Cumulative number of A1-like, A2-like and total reads with perfect or imperfect match is also presented. Haplotypes with frequency <5% in all individual plant samples are not indicated but total read counts supporting those other haplotypes are reported.
(PDF)
