# Supplementary material for: Validation of Genotyping-By-Sequencing Analysis in Populations of Tetraploid Alfalfa by 454 Sequencing
Source: PLoS One. 2015 Jun 26;10(6):e0131918. doi: 10.1371/journal.pone.0131918 (PMC4482585; doi:10.1371/journal.pone.0131918)
Supplement: S1 Fig — Location of PCR primers used for amplification, ApeKI restriction sites, GBS fragment and 64 bp sequence obtained after UNEAK analysis are indicated. (PDF) [file pone.0131918.s001.pdf]

S1 Fig.: *M. truncatula* reference sequences used for 454 validation of 14 GBS SNP loci. Location of PCR primers used for amplification, *Ape*KI restriction sites, GBS fragment and 64 bp sequence obtained after UNEAK analysis are indicated.

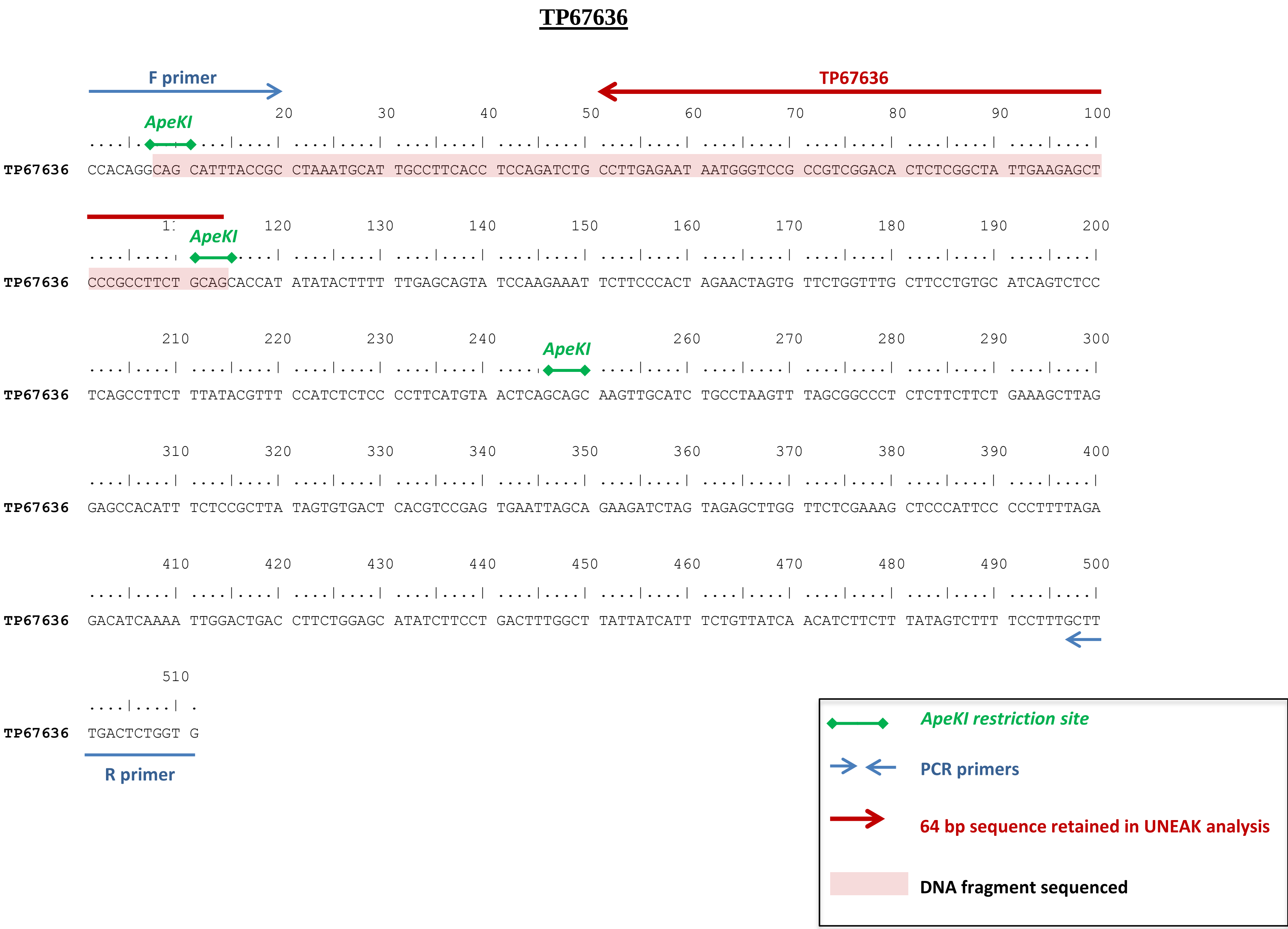

TP7278

F primer

```
                10         20         30         40         50   ApeKI   60         70         80         90    ApeKI
TP7278   CGATTTCCTC CATCTTCTCC CTCCTTCGAC TCGCTTTCTC TGACTCCAAC TGCAGCTCCA CTCGGCTTAA CCTCCATGCC GCCTCTTTCT TGTGCGCTGC

                                                        TP7278
               110        120          1  ApeKI  140        150        160        170        180        190        200
TP7278   CAATGCATGA TGCACTTCTT CCAACTCCTT GCAGCAATCA ACCAATTCAG ACATTACCAT GCTTTCGCCG TAACTTAGAA CAGCGCCATG TGGAGCTACG

               210        220        230        240        250          2  ApeKI  270        280        290        300
TP7278   TTCCCAAGAA CAAGAGGAGC GTTGTCCGGT CCTCGTACTG GTTGCATCCA CGGAGGAGGC GCTGCTACTG TCGAGGGAGA TAGGCTTAGT GTCACTGATG

               310         3:  ApeKI 330        340        350        360        370        380        390        400
TP7278   GTGAATTTGG CCTAACAGGT GCTGCATTAT TTGAATTCGA TAGCCATGAA GGAAGCAATC CCGGAGTTGG TGCTTGTGCA TCGGTATGAA GATAAGAGCT

                ApeKI     420        430        440        450        460        470        480        490        500
TP7278   ATTGGAAGCA GCCATGACAA ATGAAGGAGA TGGTCTCTCT TTCACTAGTT TCTCAGCAAA ACTTTCGAGT ATGTGTTCGT ACTTGCTATC GTTAATCGGG

               510        520        530        540        550        560        570        580
TP7278   TCGACAGTTC TGTTAATACT ACCTTTTGTT TCTCTTTGTT GTTTCTCTTT GAATACTTCC CACCACTTCC CTAACCTCTT AGCAGTTC
```

R primer

TP80194-TP79240

F primer

TP80194

ApeKI

```
                   10         20         30         40         50         60         70                    90        100
               ....|....| ....|....| ....|....| ....|....| ....|....| ....|....| ....|....| ....|....| ....|....| ....|....|
TP80194-TP79240 TAAGGGAGAA ACAGCAGTGC GATGATCAAA TTAATGAGCA AGTTTTGATG AAAACCCATG AAGTATTCTC ACAGAAGTCG CAGCACATCT CTCAGAATAT

                  110        120        130        140        150        160        170        180        190        200
               ....|....| ....|....| ....|....| ....|....| ....|....| ....|....| ....|....| ....|....| ....|....| ....|....|
TP80194-TP79240 GAAGCAAACC GGCAAAAAGA AGAAACTTTT GTCAGATTTA ATGTCTGAGA AGAACTCCTG GAATCTAAAG GGCGAATGTA TGCCAGAAAA GAAGGCTGAT
```

TP79240

ApeKI

```
                  210        220        230                   250        260        270        280        290        300
               ....|....| ....|....| ....|....| ....|....| ....|....| ....|....| ....|....| ....|....| ....|....| ....|....|
TP80194-TP79240 GATAATTCAA TTTCACGGTG TGCGAGGAAA CAGAAGGCAG CTCACGATAC TAATGAATAT TTCCATCATT CCCAAATTGC TGATGATAAA TCAACTTCAC
```

ApeKI

```
                  310          3             330        340        350        360        370        380        390        400
               ....|....| ....|....| ....|....| ....|....| ....|....| ....|....| ....|....| ....|....| ....|....| ....|....|
TP80194-TP79240 TGTGTGGCAC GAAACGGAAG GCAGCTTATG ATACTTCTGA CGATGATTTC CATAGTTACC AAATTTCTGG TAATAACTCA ACTTCACGGC GAGGAAGGAA

                  410        420        430        440        450        460        470        480        490        500
               ....|....| ....|....| ....|....| ....|....| ....|....| ....|....| ....|....| ....|....| ....|....| ....|....|
TP80194-TP79240 ACGAAAGCTA GCTTACAATG CTTTTGATGA TTGTTCCAAT AATTACCAAA CTGGAAACCA CATCCAGTTA CAGAATGTAT CGGTAGATGA GATGCAGTCT
```

ApeKI

```
                  510        520
               ....|....| ....|....| ....|
TP80194-TP79240 CAACTATGCT TAGCTGCCAA GGATC
```

R primer

TP91313

F primer

```
                    10         20         30         40         50         60         70         80   ApeKI  90        100
           ....|....| ....|....| ....|....| ....|....| ....|....| ....|....| ....|....| ....|....| ....|....| ....|....|
TP91313    GCAGAGGGAG ATTGAATTCC CGAAGAGGTT TGATCTTTCT CCGCCAATGA AGTGCTTGTT GAAGAGAAAT GTTGCCATGT TTGCTGCTGG TTTTAGAGCA
```

TP91313

```
                   110        120        130        140        150        160        170        180        190        200
           ....|....| ....|....| ....|....| ....|....| ....|....| ....|....| ....|....| ....|....| ....|....| ....|....|
TP91313    AACACCAAGC AATTGCAGGC GAAGGGGAAG GCCAATGTTC CTGGTCGTAG TGCTGTGTCA AATTTGGATT CTCCGGTTGT ATCTGGGGCT GAAACCACGG

                   210        220        230        240        250        260        270        280        290        300
           ....|....| ....|....| ....|....| ....|....| ....|....| ....|....| ....|....| ....|....| ....|....| ....|....|
TP91313    TTGATCCGTG TGGACACTCT CGGGTTGTTG ATAACCAAGA GGCTGTGAAC TCGGTGCTGA GTAAATTTCG TGACTTGAAT CTTGATGAAA ATTTGGAGTT

                   310        320        330        340        350        360        370        380        390        400
           ....|....| ....|....| ....|....| ....|....| ....|....| ....|....| ....|....| ....|....| ....|....| ....|....|
TP91313    TGTGGCTGAG GATCAGAAGG ATGAAGTGAT AGTGAGTATA TTTCATCAGA TAAAGGATTT GGAAAAACAG GCCAAGGAGC GGAAAGAATG GGCTTATCAG

                   410   ApeKI420        430        440        450        460        470        480        490        500
           ....|....| ....|....| ....|....| ....|....| ....|....| ....|....| ....|....| ....|....| ....|....| ....|....|
TP91313    AAAGCATTGC AGGCCGCGAA GAAGCTAAGC AGTGATTTGA CCGAGCTCAA AACATTACGG ATGGATAGAG AGGAGACCCA GAAATTGAAG AAAGGGAAAC

                   510        520        530
           ....|....| ....|....| ....|....| ....|..
TP91313    AGGCACTTGA GGATACAACC ATGAAGAGAC TCTCGGA
```

R primer

TP32628

F primer TP32628 ApeKI

```
TP32628 GCGAAAAGTT GGGTCTTGGT GTTAGATTAT TTAGCACTCA GGGTGCCTCA ACTGCAAGTA CTCCGCAGCC TCCACCACCA CCTCCACCTC CAGAGAAAAC
TP32628 CCATTTTGGT GGACTTAAGG ATGAGGACAG GATTTTTACC AACTTGTATG GGCTGCATGA TCCTTTCCTG AAAGGTGCCA TGAAACGTGG TGATTGGCAT
TP32628 CGCACTAAAG ATTTGGTAAT TAAGGGTACT GATTGGATCG TTAATGAAAT GAAGAAGTCT GGTCTCCGCG GACGTGGTGG TGCCGGTTTT CCTTCTGGCC
TP32628 TTAAATGGTC ATTTATGCCA AAAGTATCTG ATGGTCGTCC TTCTTACCTT GTTGTTAATG CTGATGAAAG TGAACCTGGC ACCTGCAAAG ACAGGGAAAT
TP32628 CATGCGCCAC GACCCACATA AGTTGTTAGA AGGTTGCTTA ATTGCTGGAG TGGGAATGAG GGCTACTGCT GCTTACATCT ACATCAGGGG TGAATATGTG
TP32628 AATGAACGTT TAAATCTTGA AAAGGCTAGG AAAGAGGCTT ATG
```

ApeKI (at positions 66–69, 152–155 and 468–472)

R primer

TP47889

```
F primer  ------------->                                                                   <------------- TP47889
                    10         20         30         40         50         60         70         80         90        100
          ....|....| ....|....| ....|....| ....|....| ....|....| ....|....| ....|....| ....|....| ....|....| ....|....|
TP47889   CTCCAGTATG CCAGATATGC ATGAATTGTT AAACACAGAT CTCATCCACT GGGGGATTTT ATGCAGTTTC AGCAACGCAC TAGCTACAAG GACTGCAGGC

                   110        120        130      ApeKI       150        160        170        180        190        200
          ....|....| ....|....| ....|....| ....|....| ....|....| ....|....| ....|....| ....|....| ....|....| ....|....|
TP47889   GTTTGATTGT TCATGAATGA TTGTGATGCT AAAGCAGGAG CTGCCAAAGA TACTGTCATC TCCTCCATAA TCAATCTACA CCAACTGCTC TTTCCTAACT

                   210        220        230        240        250        260        270        280        290        300
          ....|....| ....|....| ....|....| ....|....| ....|....| ....|....| ....|....| ....|....| ....|....| ....|....|
TP47889   GTTCCGTTTC CTTTGCTAGA TTTTCCCATC GCAGAAGAAT TGACAGACAG AGTGCACAAT TGGTAGCCAC TCTCAGATCA TTATAAAATA CCATCCCTTC

                   310        320        330        340        350        360        370        380        390        400
          ....|....| ....|....| ....|....| ....|....| ....|....| ....|....| ....|....| ....|....| ....|....| ....|....|
TP47889   CAATATACTC CTTATGGACA TTAAATACCC AACAGTACAT TTCAACTCTT CCTGTTTAGT GTTTATTTGA TCTGATATTC TGTTGAACAA TTCTAACAGG

                   410        420        430        440        450        460        470        480        490        500
          ....|....| ....|....| ....|....| ....|....| ....|....| ....|....| ....|....| ....|....| ....|....| ....|....|
TP47889   CTGTAAGAAG CAATAATCTT GACAACGGGA GAACCAAAAT GCAGAAGTCT GCACAAATCA TGACAACGGA TGCCTATGAA GGCTAGTGGT TCTGATGAAT

                   510        520        530        540
          ....|....| ....|....| ....|....| ....|....| ....|..
TP47889   TCATTGAAAC AAGGAATTTT TGCCAATCCA CAAACCCTGG AAGGATA
                                   <-------------------
                                         R primer
```

TP61949-TP14949

```
                                                                      TP14949
      F primer                                  TP61949
               10        20   ApeKI  40        50        60        70        80        90       100
               ....|....| ....|....| ....|....| ....|....| ....|....| ....|....| ....|....| ....|....| ....|....| ....|....|
TP61949-TP14949  TAAGCGGTTT ACATTGGCAG AAATCAACGC TGCAACAAAT AATTTCGACG ACAGTTTGGT CATCGGAGTT GGAGGCTTTG GTAAGGTGTA TAAGGGAGAG

              110    ApeKI     130       140       150       160       170       180       190       200
               ....|....| ....|....| ....|....| ....|....| ....|....| ....|....| ....|....| ....|....| ....|....| ....|....|
TP61949-TP14949  GTTGATGATG GTGTCCCCGC TGCAATTAAG CGCGCCAATC CACAATCTGA ACAAGGTCTG GCCGAATTTG AGACAGAAAT TGAGATGCTC TCAAAACTAA

              210       220       230       240       250       260       270       280       290       300
               ....|....| ....|....| ....|....| ....|....| ....|....| ....|....| ....|....| ....|....| ....|....| ....|....|
TP61949-TP14949  GACACAGACA TCTTGTGTCT TTGATTGGTT TCTGTGAAGA GAAAAGTGAA ATGATTTTGG TTTATGAGTA CATGGCAAAT GGAACTCTAA GAAGTCATCT

              310       320       330    ApeKI :0        350    ApeKI      370       380       390       400
               ....|....| ....|....| ....|....| ....|....| ....|....| ....|....| ....|....| ....|....| ....|....| ....|....|
TP61949-TP14949  ATTTGGGAGC GATCTCCCTC CACTAACTTG GAAGCAGCGT CTTGAAGCGT GCATAGGTGC TGCAAGGGGA CTTCACTACC TTCACACAGG AGCCGACCGT

              410       420       430       440       450       460       470       480       490       500
               ....|....| ....|....| ....|....| ....|....| ....|....| ....|....| ....|....| ....|....| ....|....| ....|....|
TP61949-TP14949  GGAATAATTC ACCGGGATGT CAAGACAACC AATATATTGT TAGACGAGAA TTTTGTAGCA AAAATGGCTG ATTTCGGTTT GTCAAAAGAC GGTCCTGCTT

              510       520       530       540
               ....|....| ....|....| ....|....| ....|....| ...
TP61949-TP14949  TTGAACATAC TCATGTTAGC ACTGCTGTGA AAGGAAGTTT TGG
                                         R primer
```

TP31029

F primer → TP31029 ← ApeKI ApeKI

```
                 10         20         30         40         50         60         70         80         90        100
         ....|....| ....|....| ....|....| ....|....| ....|....| ....|....| ....|....| ....|....| ....|....| ....|....|
TP31029  CTGCTGTTGC GATTAAGAGG CTTACTAATG GAGGGCAGCA GGGTGACAAA GAGCTTTTGG TTGAGGTTGA GATGCTTAGC CGGCTGCATC ACCGTAACCT

                110        120        130        140        150        160        170        180        190        200
         ....|....| ....|....| ....|....| ....|....| ....|....| ....|....| ....|....| ....|....| ....|....| ....|....|
TP31029  TGTAAAACTA GTTGGATACT ATAGCAATCG TGACTCATCA CAAAACCTAC TTTGCTATGA GCTTGTCCCA AATGGAAGTT TGGAGGCCTG GCTTCACGGT

                210        220        230        240        250        260        270        280        290        300
         ....|....| ....|....| ....|....| ....|....| ....|....| ....|....| ....|....| ....|....| ....|....| ....|....|
TP31029  ATTGTAATTT CTCGAATAAT GGGTTCAGTT TTTTAATCTT GAATGACCTG TTACATTGAT AGTTATTTTC AGAAATAATT TCATTATGCT TAATTGAAAT

                310        320        330        340        350        360        370                   390        400
                                                                                              ApeKI
         ....|....| ....|....| ....|....| ....|....| ....|....| ....|....| ....|....| ....|....| ....|....| ....|....|
TP31029  TTTCCCTGAT TTCCCTAGGT CCCTTGGGAA TAAATTGCTC TCTGGATTGG GACACCAGGA TGAAGATTGC ACTTGATGCT GCAAGAGGAC TTGCTTACCT

                410        420        430        440        450        460        470        480        490        500
         ....|....| ....|....| ....|....| ....|....| ....|....| ....|....| ....|....| ....|....| ....|....| ....|....|
TP31029  GCATGAAGAT TCACAGCCAT GTGTCATACA CAGAGATTTC AAGGCATCTA ATATATTGCT TGAGAACAAC TTTCATGCTA AAGTTGCTGA TTTTGGTCTA

                510        520        530        540        550        560
         ....|....| ....|....| ....|....| ....|....| ....|....| ....|....| ..
TP31029  GCTAAACTAG CACCTGAAGG CAGAGTTAAT TATCTGTCTA CTCGAGTTAT GGGCACATTT GG
```

← R primer

TP46847

F primer → TP46847 ← ApeKI

```
                 10         20         30         40         50         60         70         80         90        100
         ....|....| ....|....| ....|....| ....|....| ....|....| ....|....| ....|....| ....|....| ....|....| ....|....|
TP46847  CAGCGAGAAC TCTTGATCCA GGTGCCATTA TGTCAGGCTT CAAAATCCAA GGAAAGCCAT GTGATGGACC TCTTGATGAG TAATGAGCTG CAATTGGTGC

                110        120        130        140        150        160        170        180        190        200
         ....|....| ....|....| ....|....| ....|....| ....|....| ....|....| ....|....| ....|....| ....|....| ....|....|
TP46847  CGGTTTTATT CCAAGAAATG TTTGTTGAAA CTTAATGCTT GCTGTTGGAT TATTCTTATT GCGTTTTGCG TACTTGATCA CAGATTCTGC ATCTTTTGTC

                210        220        230        240        250        260        270        280        290        300
         ....|....| ....|....| ....|....| ....|....| ....|....| ....|....| ....|....| ....|....| ....|....| ....|....|
TP46847  TTGATAACAA TACCTGGAGC GTAGATATGT CTTAAATCAA TTAATTCAGG ACTATCAGAG AGAAACACTG CCCCTAACAT ATTGGTTTGT GTAACAACAT

                310        320        330        340        350        360        370        380        390        400
         ....|....| ....|....| ....|....| ....|....| ....|....| ....|....| ....|....| ....|....| ....|....| ....|....|
TP46847  CGATTTGATC AAACACTGAG CTTGAGTTTG ATATTGAGTC ACAAATAATT ATGACTTGCT TGTTAACTTG AGATAAACGC TTCACTGAAT TGCATGAAGA

                410        420        430        440        450        460        470        480        490        500
         ....|....| ....|....| ....|....| ....|....| ....|....| ....|....| ....|....| ....|....| ....|....| ....|....|
TP46847  TAAAGTGTTA TCGTAAACAA GAGGAAGATT TTCGACTATA GTTGAATTTG ATGCAAACAA AGTCCAACCA ATAATGTTTT GACCATTCCC CAAAACAAGG

         ApeKI  510        520  ApeKI     540        550
         ....|....| ....|....| ....|....| ....|....| ....|....|
TP46847  CTGCCAAAAG TTCTATCTAT GGTTCCTGCA GCTACAGTAA GAACCCAAGG
```

← R primer

TP17289

TP17289

F primer

ApeKI

```
                 10         20         30         40         50                    70         80         90        100
         ....|....| ....|....| ....|....| ....|....| ....|....| ....|....| ....|....| ....|....| ....|....| ....|....|
TP17289  TAGTTTTTGT CTCTGGATAA GTGTTCCCAT TCTCAATTAC TGGTTCAACT TTGGAATTAT GCTGCCAAAC CAGAAAGGTA GAATCAGACA TTGGAAACAA

                110        120        130        140        150        160        170        180        190        200
         ....|....| ....|....| ....|....| ....|....| ....|....| ....|....| ....|....| ....|....| ....|....| ....|....|
TP17289  AGAAATGGGA AACATTGCAA TGTTAATAGT GACAACAAAA ATGGAGTATA GGAAAATGTT CTCATTGCAA GTCTTTTGAT GCAAATTTTA GTTGATATGC

                210        220        230        240        250        260        270        280        290        300
         ....|....| ....|....| ....|....| ....|....| ....|....| ....|....| ....|....| ....|....| ....|....| ....|....|
TP17289  CTCTTACATG AATCTATTTC TAAAGATCAA ACCCTTAGTG CCATATCATG AAATCTGTAA ATGATATTAC AACAAAATCA CCCAACTATC TTAGTTGCAT

                310        320        330        340        350        360        370        380        390        400
         ....|....| ....|....| ....|....| ....|....| ....|....| ....|....| ....|....| ....|....| ....|....| ....|....|
TP17289  GAATTGTTGG CAGGTGAAAG GTATTATGAC ACAAGCAACT ATACAATACC CCGAATTTCA TTGGCCAAAA TATTGATTTC TTACTTGTTT GACCAACCAG

                410        420        430        440        450        460        470        480        490        500
         ....|....| ....|....| ....|....| ....|....| ....|....| ....|....| ....|....| ....|....| ....|....| ....|....|
TP17289  ATCGTGCTCA GTCAGTACCT ACTCTTAACG TTAGTTTGAT ATAATATATG TGTATAACAC GTTAAGCTAA ATCCAAGCCA AAAGCATAAG TACATCTCAG

                510        520        530        540        550        560        570
         ....|....| ....|....| ....|....| ....|....| ....|....| ....|....| ....|....| ...
TP17289  AAAGAACAAG ACTCGGTATT ATATATAGCA TTACGAATAC TCACATTCAT TTGAACGTCT ATTACAGGCT CTG
```

R primer

TP1933-TP26408

TP26408

TP1933

F primer

```
                       10        20        30        40        50        60        70        80        90       100
              ....|....| ....|....| ....|....| ....|....| ....|....| ....|....| ....|....| ....|....| ....|....| ....|....|
TP1933-TP26408 AGCAAAGTCA GCATCTATGG CATCATCTAG CAATGTTTTT CCGTGATGAG CTACTGTCTT GGTCTTGGAG AAGACCACTT GGGATGCCCA TGGCCCCTAT
```

ApeKI

```
                      110       120       130       140       150       160       170       180       190       200
              ....|....| ....|....| ....|....| ....|....| ....|....| ....|....| ....|....| ....|....| ....|....| ....|....|
TP1933-TP26408 GGCTGCAGGT GGTACTATGA GCCCTGTTGA CTTTAAACAA AAGGTTATTA CAAATGTTGA ACATGTAGTT GGGAGAGTTA AGGGAATAGC ACCGCAGAAT

                      210       220       230       240       250       260       270       280       290       300
              ....|....| ....|....| ....|....| ....|....| ....|....| ....|....| ....|....| ....|....| ....|....| ....|....|
TP1933-TP26408 TTTTCTGATG AGGTGAGTAG CTAAATATTG GCTCTATTTT TTAATTTTAA TTTTATGTTT TGTTCTAATA TGATTGTTAA GTCTAAGTTC ATGCTTCTTT

                      310       320       330       340       350       360       370       380       390       400
              ....|....| ....|....| ....|....| ....|....| ....|....| ....|....| ....|....| ....|....| ....|....| ....|....|
TP1933-TP26408 TTTAATTGAT GGAATGGGGA GATAAATGAT TATATAAAAA AATTCCAATG CGTTGTTCTA CCAAAAATTC AAGAATCCAA TTGATCCTCC ACACAAGTCT

                      410       420       430       440       450       460       470       480       490       500
              ....|....| ....|....| ....|....| ....|....| ....|....| ....|....| ....|....| ....|....| ....|....| ....|....|
TP1933-TP26408 CTATTGATTT CGTAATAACT ACTTAAAAAT CTAATGACGA AAACTGATGC CTTGCCTTTC CACTTTCAAT TCATTGATTG ATATTTGTTC CTGTTTTTTT

                      510       520
              ....|....| ....|....|
TP1933-TP26408 CCCTTCAAAT TTCTCACTCC
```

R primer
