# Supplementary material for: Validation of Genotyping-By-Sequencing Analysis in Populations of Tetraploid Alfalfa by 454 Sequencing
Source: PLoS One. 2015 Jun 26;10(6):e0131918. doi: 10.1371/journal.pone.0131918 (PMC4482585; doi:10.1371/journal.pone.0131918)
Supplement: S2 Fig — Sequences covering GBS SNP loci (64 bp sequence) are also aligned. SNPs used to define haplotypes, ApeKI restriction sites and PCR primer annealing regions are indicated. (PDF) [file pone.0131918.s002.pdf]

S2 Fig.: Alignments of haplotypes defined within the first 400bp of 454 sequences against the 11 targeted genomic regions of *M. truncatula* and Sanger consensus sequences obtained from amplified DNA fragments of *M. sativa*. Sequences covering GBS SNP loci (64 bp sequence) are also aligned. SNPs used to define haplotypes, *Ape*KI restriction sites and PCR primer annealing regions are indicated.

TP67636

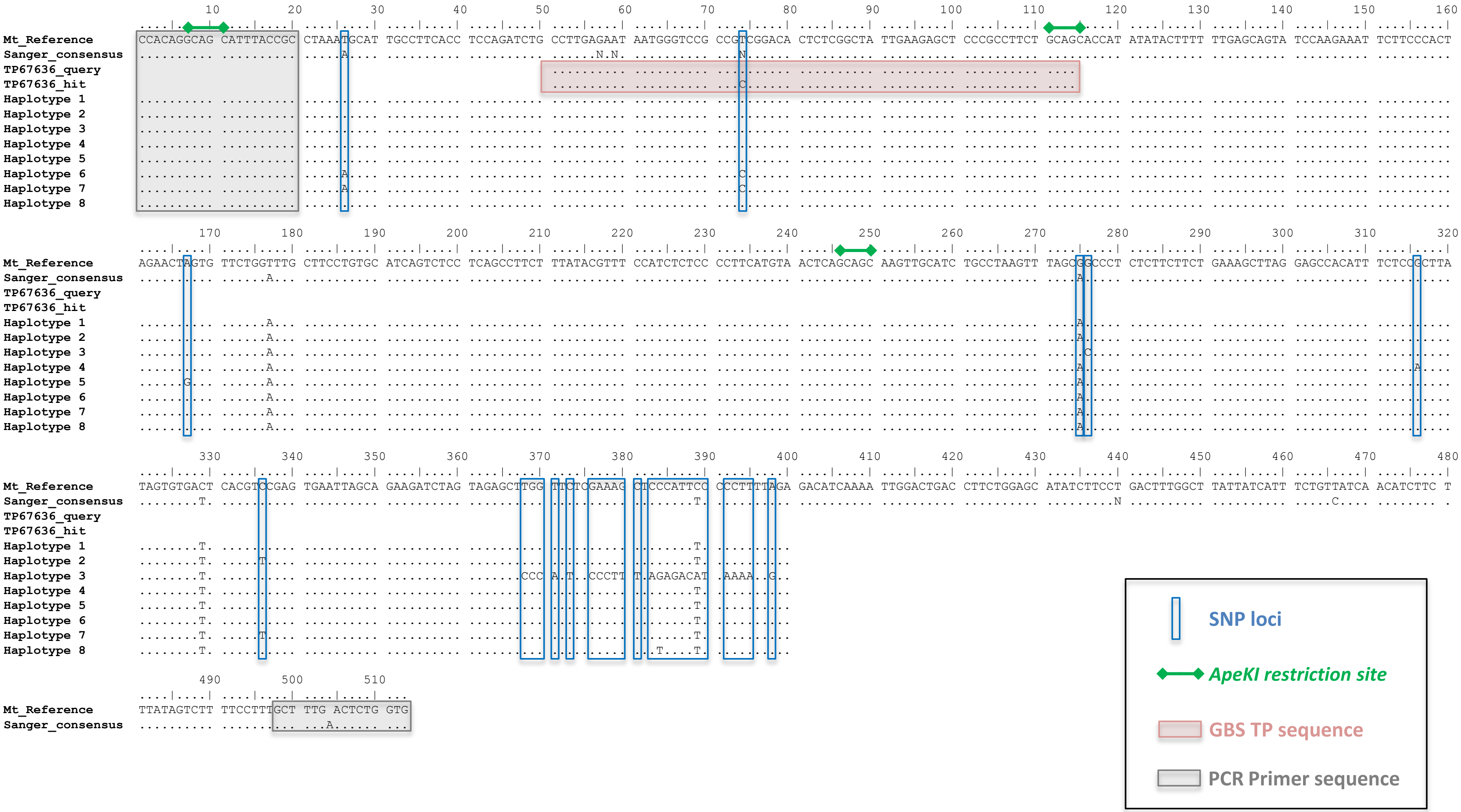

TP7278

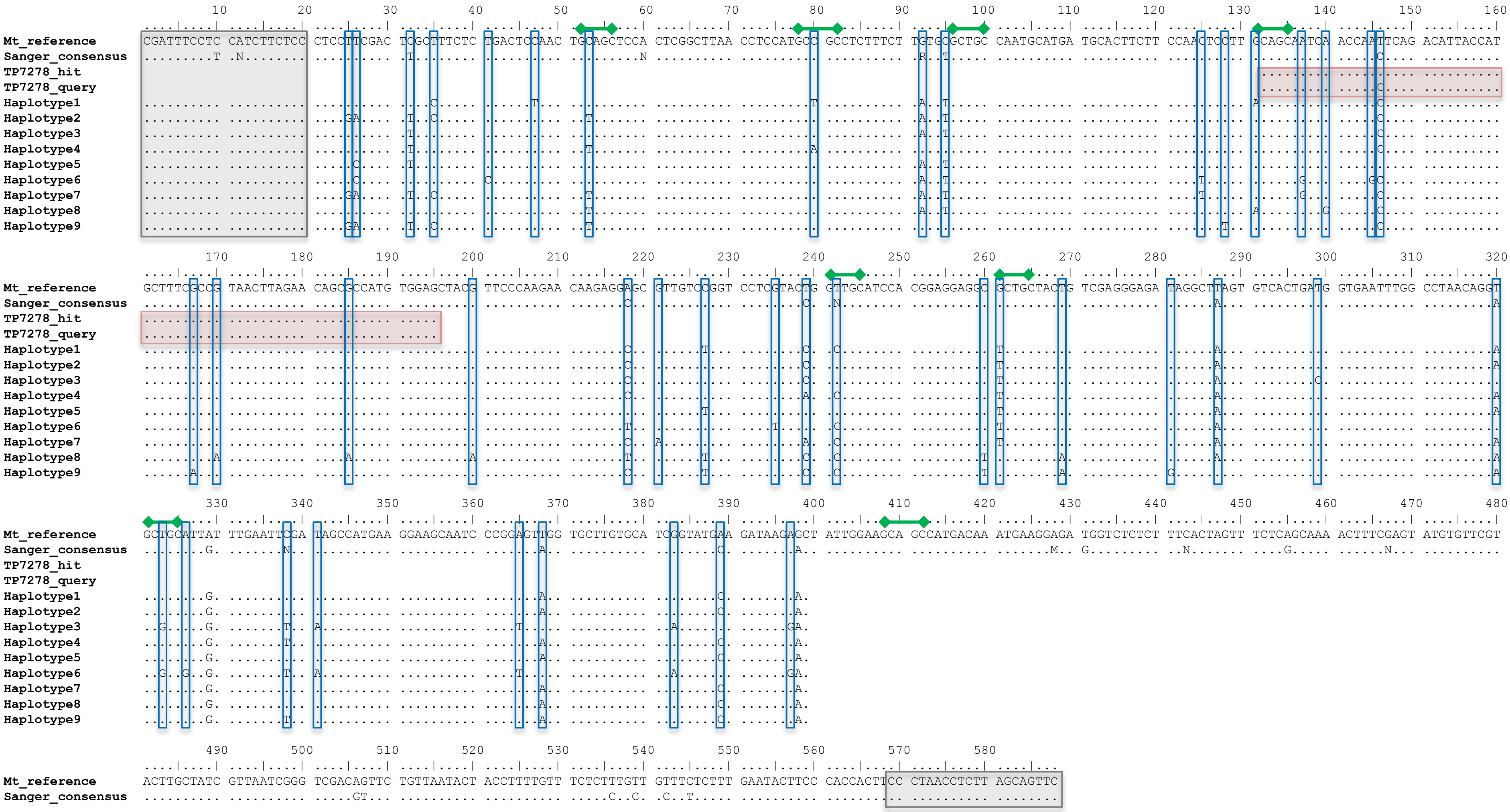
10 20 30 40 50 60 70 80 90 100 110 120 130 140 150 160
Mt_reference CGATTTCCTC CATCTTCTCC CTCCTTCGAC TCGCTTTCTC TGACTCCAAC TGCAGCTCCA CTCGGCTTAA CCTCCATGCC GCCTCTTTCT TGTGCGCTGC CAATGCATGA TGCACTTCTT CCAACTCCTT GCAGCAATCA ACCAATTCAG ACATTACCAT
Sanger_consensus
TP7278_hit
TP7278_query
Haplotype1
Haplotype2
Haplotype3
Haplotype4
Haplotype5
Haplotype6
Haplotype7
Haplotype8
Haplotype9
170 180 190 200 210 220 230 240 250 260 270 280 290 300 310 320
Mt_reference GCTTTCGCCG TAACTTAGAA CAGCGCCATG TGGAGCTACG TTCCCAAGAA CAAGAGGAGC GTTGTCCGGT CCTCGTACTG GTTGCATCCA CGGAGGAGGC GCTGCTACTG TCGAGGGAGA TAGGCTTAGT GTCACTGATG GTGAATTTGG CCTAACAGGT
330 340 350 360 370 380 390 400 410 420 430 440 450 460 470 480
Mt_reference GCTGCATTAT TTGAATTCGA TAGCCATGAA GGAAGCAATC CCGGAGTTGG TGCTTGTGCA TCGGTATGAA GATAAGAGCT ATTGGAAGCA GCCATGACAA ATGAAGGAGA TGGTCTCTCT TTCACTAGTT TCTCAGCAAA ACTTTCGAGT ATGTGTTCGT
490 500 510 520 530 540 550 560 570 580
Mt_reference ACTTGCTATC GTTAATCGGG TCGACAGTTC TGTTAATACT ACCTTTTGTT TCTCTTTGTT GTTTCTCTTT GAATACTTCC CACCACTTCC CTAACCTCTT AGCAGTTC
Sanger_consensus

TP80194-TP79240

```
                           10         20         30         40         50         60         70         80         90        100        110        120        130        140        150        160
Mt_reference      TAAGGGA-GA AACAGCAGTG CGATGATCAA ATTAATGAGC AAGTTTTGAT GAAAACCCAT GAAGTATTCT CACAGAAGTC GCAGCACATC TCTCAGAATA TGAAGCAAAC CGGCAAAAAG AAGAAACTTT TGTCAGATTT AATGTCTGAG AAGAACTCCT
Sanger_consensus  .......G.. .......... .......... .......... .......... ..N....... .......... .......... .......... .......... .......... T......... .......... .......... .......... ..........
TP80194_query                           .......... .......... .......... .......... .......... .......... .....
TP80194_hit                             .......... .......... ...A...... .......... .......... .......... .....
TP79240_query
TP79240_hit
Haplotype_1       .......G.. .......... .......... .......... .......... .......... .......... .......... .......... .......... .......... T......... .......... .......... .......... ..........
Haplotype_2       .......G.. .......... .......... .......... .......... .......... .......... .......... .......... .......... .......... T......... .......... .......... .......... ..........
Haplotype_3       .......G.. .......... .......... .......... .......... .......... .......... .......... .......... .......... .......... T......... .......... .......... .......... ..........
Haplotype_4       .......G.. .......... .......... .......... .......... .......... .......... .......... .......G.. .......... .......... T......... .......... .......... .......... ..........
Haplotype_5       .......G.. .......... .......... .......... .......... .......... .......... .......... .......... .......... .......... T......... .......... .......... .......... ..........
Haplotype_6       .......G.. .......... .......... .......... .......... .......... .......... .......... .......... .......... .......... T......... .......... .......... .......... ..........
Haplotype_7       .......G.. .......... .......... .......... .......... .......... .......... .......... .......... .......... .......... T......... .......... .......... .......... ..........
Haplotype_8       .......G.. .......... .......... .......... ...A...... .......... .......... .......... .......G.. .......... .......... T......... .......... .......... .......... ..........
Haplotype_9       .......G.. .......... .......... .......... .......... .......... .......... .......... T......... .......... .......... T......... .......... .......... .......... ..........
Haplotype_10      .......G.. .......... .......... .......... .......... .......... .......... .......... .......G.. .......... .......... T......... .......... .......... .......... ..........

                          170        180        190        200        210        220        230        240        250        260        270        280        290        300        310        320
Mt_reference      GGAATCTAAA GGGCGAATGT ATGCCAGAAA AGAAGGCTGA TGATAATTCA ATTTCACGGT GTGCGAGGAA ACAGAAGGCA GCTCACGATA CTAATGAATA TTTCCATCAT TCCCAAATTG CTGATGATAA ATCAACTTCA CTGTGTGGCA CGAAACGGAA
Sanger_consensus  .......... .......... .......R.. .......... .......... .......... .......... .......... .......G.. .......... .......... .......... .......... .......... .......... ..........
TP80194_query
TP80194_hit
TP79240_query                                                                                                               .......... .......... .......... .......... .......... ..........
TP79240_hit                                                                                                                 .......... .......... .G........ .......... .......... ..........
Haplotype_1       .......... ...T...... .......G.. .......... .......... .......... .......... .......... .......G.. .......... .......... .......... .......... .......... .......... ..........
Haplotype_2       .......... .......... .......G.. .......... .......... .......... .......... .......... .......G.. .......... .......... .......... .......... .......... .......... ..........
Haplotype_3       .......... .......... .......... .......... .......... .......... .......... .......... .......G.. .......... .......... .......... .......... .......... .......... ..........
Haplotype_4       .......... .......... .......... .......... .......... .......... .......... .......... .......G.. .......... .......... .......... .......... .......... .......... ..........
Haplotype_5       .......... .......... .......G.. .......... .......... .......... .......... .......... .......G.. .......... .......... .......... .......... .......... .......... ..........
Haplotype_6       .......... .......... .......G.. .......... .......... .......... ....A..... .......... .......G.. .......... .......... .......... .......... .......... .......... ..........
Haplotype_7       .......... ...T...... .......G.. .......... .......... .......... .......... .......... .......G.. .......... .......... .......... .......... .......... .......... ..........
Haplotype_8       .......... .......... .......... .......... .......... .......... .......... .......... .......G.. .......... .......... .......... .......... .......... .......... ..........
Haplotype_9       .......... .......... .......G.. .......... .......... .......... .......... .......... .......G.. .......... .......... .......... .......... .......... .......... ..........
Haplotype_10      .......... .......... .......... .......... .......... .......... .......... .......... .......G.. .......... .......... .......... .......... .......... .......... ..........

                          330        340        350        360        370        380        390        400        410        420        430        440        450        460        470        480
Mt_reference      GGCAGCTTAT GATACTTCTG ACGATGATTT CCATAGTTAC CAAATTTCTG GTAATAACTC AACTTCACGG CGAGGAAGGA AACGAAAGCT AGCTTACAAT GCTTTTGATG ATTGTTCCAA TAATTACCAA ACTGGAAACC ACATCCAGTT ACAGAATGTA
Sanger_consensus  .......... .......... .......... ..G....... .......... .......... ..A...T... .......... ........G. .......... .......... .......... .....C.... .......... .......... ..........
TP80194_query
TP80194_hit
TP79240_query     .....
TP79240_hit       .....
Haplotype_1       .......... .......... ....GA.... ..G....... .......... .......... ..A...T... ......G.
Haplotype_2       .......... .......... ....GA.A.. ..G....... .......... .......... ..A...T... ......G.
Haplotype_3       .......... .......... ....GA.... ..G....... .......... .......... ..A...T... ......G.
Haplotype_4       .......... .......... ....GA.... ..G....... .......... .......... ..A...T... ......G.
Haplotype_5       .......... .......... ....GA.... ..G....... .......... .......... ..A...T... ......G.
Haplotype_6       .......... .......... ....GA.... ..G....... .......... .......... ..A...T... ......G.
Haplotype_7       .......... .......... ....GA.A.. ..G....... .......... .......... ..A...T... ......G.
Haplotype_8       .......... .......... ....GA.... ..G....... .......... .......... ..A...T... ......G.
Haplotype_9       .......... .......... ....GA.A.. ..G....... .......... .......... ..A...T... ......G.
Haplotype_10      .......... .......... ....GA.A.. ..G....... .......... .......... ..A...T... ......G.

                          490        500        510        520
Mt_reference      TCGGTAGATG AGATGCAGTC TCAACTATGC TTAGCTGCCA AGGATC
Sanger_consensus  .......... .......... ........   ...
```

TP91313

```
                       10         20         30         40         50         60         70         80         90        100        110        120        130        140        150        160
Mt_Reference    GCAGAGGGAG ATTGAATTCC CGAAGAGGTT TGATCTTTCT CCGCCAATGA AGTGCTTGTT GAAGAGAAAT GTTGCCATGT TTGCTGCTGG TTTTAGAGCA AACACCAAGC AATTGCAGGC GAAGGGGAAG GCCAATGTTC CTGGTCGTAG TGCTGTGTCA
Ms_Consensus    NNN....NN. .......... .......... .......... ....S..WKR .......... .......... .......... .......... .......... .......... .......... .......... .......... .......... .........G
TP91313_hit                                                                                      ....... .......... .......... .......... .......... .......... T......
TP91313_query                                                                                    ....... .......... .......... .......... .......... .......... .......
Haplotype_1     .......... .......... .......... .......... .......... .......... .......... .......... .......... .......... .......... .......... .......... .......... .......... .........G
Haplotype_2     .......... .......... .......... .......... .......... .......... .......... .......... .......... .......... .......... .......... .......... .......... .......... .........G
Haplotype_3     .......... .......... .......... .......... .......... .......... .......... .......... .......... .......... .......... .......... .......... .......... .......... .........G
Haplotype_4     .......... .......... .......... .......... .......... .......... .......... .......... .......... .......... .......... .......... .......... .......... .......... .........G
Haplotype_5     .......... .......... .......... .......... .......... .......... .......... .......... .......... .......... .......... .......... .......... .......... T......... .........G
Haplotype_6     .......... .......... .......... .......... .......... .......... .......... .......... .......... .......... .......... .......... A......... .......... .......... .....G...G
Haplotype_7     .......... .......... .......... .......... .......... .......... .......... .......... .......... .......... .......... .......... A......... .......... .......... .....G...G

                      170        180        190        200        210        220        230        240        250        260        270        280        290        300        310        320
Mt_Reference    AATTTGGATT CTCCGGTTGT ATCTGGGGCT GAAACCACGG TTGATCCGTG TGGACACTCT CGGGTTGTTG ATAACCAAGA GGCTGTGAAC TCGGTGCTGA GTAAATTTCG TGACTTGAAT CTTGATGAAA ATTTGGAGTT TGTGGCTGAG GATCAGAAGG
Ms_Consensus    .......... .......... .......... .......... .....A.... .......... .......... .......... .......... .......... .......... .......... .......... .......... .......... ..........
TP91313_hit
TP91313_query
Haplotype_1     .......... .......... .......... .......... .....A.... .......... .......... .......... .......... .......... .......... .......... .......... .......... .......... ..........
Haplotype_2     .......... .......... .......... .......... .....A.... .......... .......... .......... .......... .......... .......... .......... .......... .......... .......... ..........
Haplotype_3     .......... ...T...... .......... .......... .....A.... .......... .........T .......... .......... .......... .......... .......... .......... .......... .......... ..........
Haplotype_4     .......... .......... .......... .......... .....A.... .......... .......... .......... .......... ..A....... .......... .......... .......... .......... .......... ..........
Haplotype_5     .......... .......... .......... .......... .....A.... .......... .......... .......... .......... .......... .......... .......... .......... .......... .......... ..........
Haplotype_6     .......... .......... .........A ........A. .......A.. .......... .......... .......... ......A... .......... .......... .......... .......... .......... .......... ..........
Haplotype_7     .......... .......... .........A ........A. .......A.. .......... .......... .......... ......A... .......... .......... .......... .......... .......... .......... ..........

                      330        340        350        360        370        380        390        400        410        420        430        440        450        460        470        480
Mt_Reference    ATGAAGTGAT AGTGAGTATA TTTCATCAGA TAAAGGATTT GGAAAAACAG GCCAAGGAGC GGAAAGAATG GGCTTATCAG AAAGCATTGC AGGCCGCGAA GAAGCTAAGC AGTGATTTGA CCGAGCTCAA AACATTACGG ATGGATAGAG AGGAGACCCA
Ms_Consensus    .......... .......... .......... .......... .......... .......... .......... .......... .......... ....T..... .......... .......... .T........ .......... .......... ..........
TP91313_hit
TP91313_query
Haplotype_1     .......... .......... .......... .......... .......... .......... .......... .......... .......... ....T..... .......... .......... .T........ .......... .......... ..........
Haplotype_2     .......... .......... .......... .......... .......... .......... .......... .......... .......... .......... .......... .......... .T........ .......... .......... ..........
Haplotype_3     .......... .......... .......... .......... .......... .......... .......... .......... .......... ....T..... .......... .......... .T........ .......... .......... ..........
Haplotype_4     .......... .......... .......... .......... .......... .......... .......... .......... .......... ....T..... .......... .......... .T........ .......... .......... ..........
Haplotype_5     .......... .......... .......... .......... .......... .......... .......... .......... .......... ....T..... .......... .......... .T........ .......... .......... ..........
Haplotype_6     .......... .......... .......... .......... .......... .......... .......... .......... .......... .......... .......... .......... .T........ .......... .......... ..........
Haplotype_7     .......... .......... .......... .......... .......... .......... .......... .......... .......... ....T..... .......... .......... .T........ .......... .......... ..........

                      490        500        510        520        530
Mt_Reference    GAAATTGAAG AAAGGGAAAC AGGCACTTGA GGATACAACC ATGAAGAGAC TCTCGGA
Ms_Consensus    ......R.-. ...A...... .......... ...C...... .......... .......
TP91313_hit
TP91313_query
Haplotype_1     .......... .......... .......... ...C...... .......... .......
Haplotype_2     .......... .......... .......... ...C...... .......... .......
Haplotype_3     .......... .......... .......... ...C...... .......... .......
Haplotype_4     .......... .......... .......... ...C...... .......... .......
Haplotype_5     .......... .......... .......... ...C...... .......... .......
Haplotype_6     .......... .......... .......... ...C...... .......... .......
Haplotype_7     .......... .......... .......... ...C...... .......... .......
```

TP32628

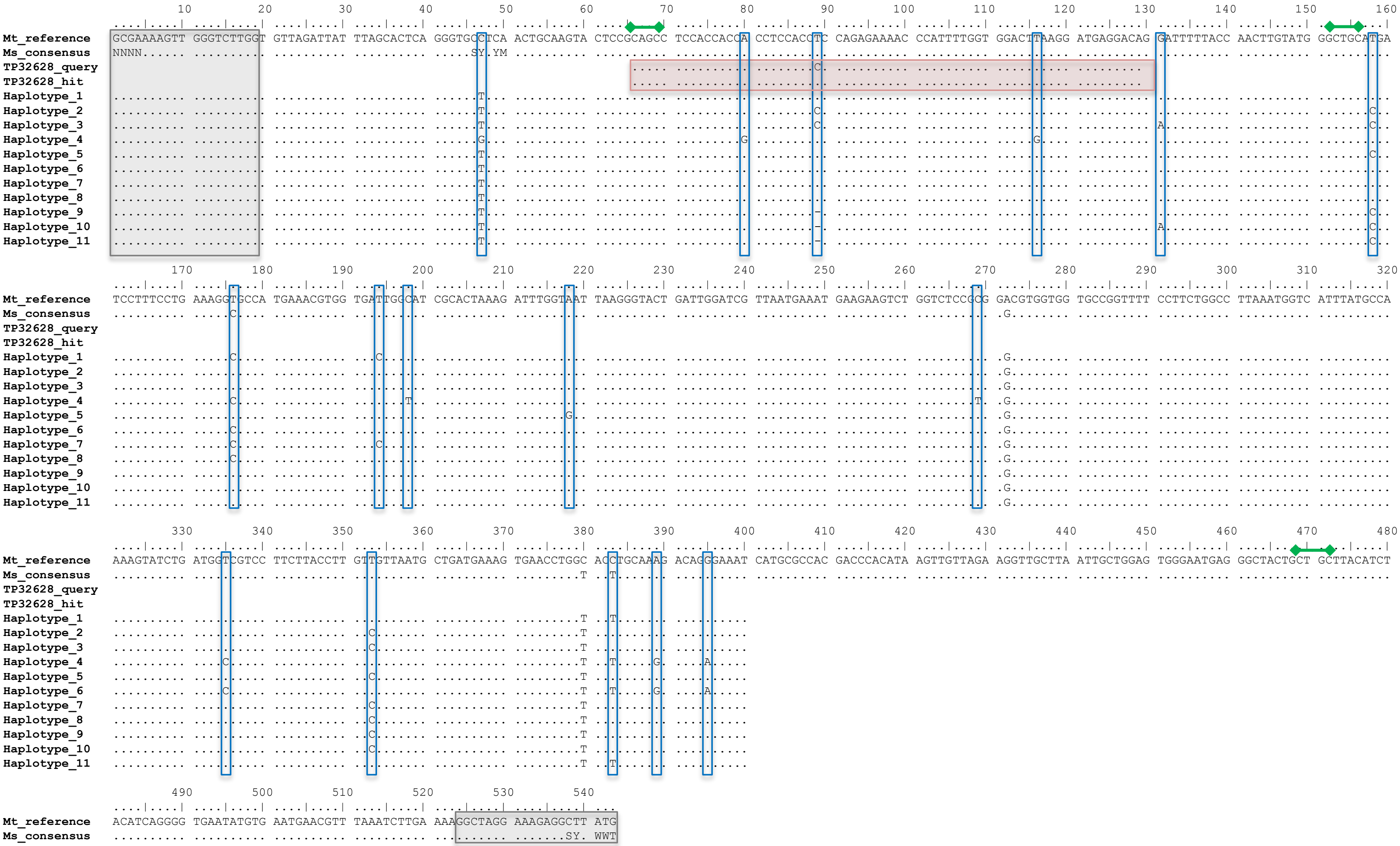

Mt_reference
Ms_consensus
TP32628_query
TP32628_hit
Haplotype_1
Haplotype_2
Haplotype_3
Haplotype_4
Haplotype_5
Haplotype_6
Haplotype_7
Haplotype_8
Haplotype_9
Haplotype_10
Haplotype_11
GCGAAAAGTT GGGTCTTGGT GTTAGATTAT TTAGCACTCA GGGTGCCTCA ACTGCAAGTA CTCCGCAGCC TCCACCACCA CCTCCACCTC CAGAGAAAAC CCATTTTGGT GGACTTAAGG ATGAGGACAG GATTTTTACC AACTTGTATG GGCTGCATGA
NNNN...... ....SY.YM
TCCTTTCCTG AAAGGTGCCA TGAAACGTGG TGATTGGCAT CGCACTAAAG ATTTGGTAAT TAAGGGTACT GATTGGATCG TTAATGAAAT GAAGAAGTCT GGTCTCCGCG GACGTGGTGG TGCCGGTTTT CCTTCTGGCC TTAAATGGTC ATTTATGCCA
AAAGTATCTG ATGGTCGTCC TTCTTACCTT GTTGTTAATG CTGATGAAAG TGAACCTGGC ACCTGCAAAG ACAGGGAAAT CATGCGCCAC GACCCACATA AGTTGTTAGA AGGTTGCTTA ATTGCTGGAG TGGGAATGAG GGCTACTGCT GCTTACATCT
ACATCAGGGG TGAATATGTG AATGAACGTT TAAATCTTGA AAAGGCTAGG AAAGAGGCTT ATG
....SY. WWT

TP47889

```
                           10         20         30         40         50         60         70         80         90        100        110        120        130        140        150        160
                  ....|....| ....|....| ....|....| ....|....| ....|....| ....|....| ....|....| ....|....| ....|....| ....|....| ....|....| ....|....| ....|....| ....|....| ....|....| ....|....|
MT_reference      CTCCAGTATG CCAGATATGC ATGAATTGTT AAACACAGAT CTCATCCACT GGGGGATTTT ATGCAGTTTC AGCAACGCAC TAGCTACAAG GACTGCAGGC GTTTGATTGT TCATGAATGA TTGTGATGCT AAAGCAGGAG CTGCCAAAGA TACTGTCATC
Sanger_Consensus               ........ .......... .......... .......... ......N... .......... .......... .......... .......... .......... .......... .......... .......... .......... ..........
TP47889_query                                                                                 . .......... .......... .......... .......... .......... .......... ...
TP47889_hit                                                                                   . .......... .......... .......... .......... .........C .......... ...
Haplotype1        .......... .......... .......... .......... .......... .......... .......... .......... .......... .......... .......... .......... .......... .......... .......... ..........
Haplotype2        .......... .......... .......... .......... .C........ .......... .......... .......... .......... .......... .......... .......... .......... .......... .......... ..........
Haplotype3        .......... .......... .......... .......... .......... .......... .......... .......... .......... .......... .......... .......... .......... .......... .......... ..........
Haplotype4        .......... .......... .......... .......... .......... .......... .......... .......... .......... .......... .......... .......... .......... .......... .......... ..........
Haplotype5        .......... .......... .......T.. .......... .......... .......... .......... .......... .......... .......... .......... .......... .......... .......... .......... ..........
Haplotype6        .......... .......... .......... .......... .......... .......... .......... .......... .......... .......... .......... .......... .........C .......... .......... ..........

                         170        180        190        200        210        220        230        240        250        260        270        280        290        300        310        320
                  ....|....| ....|....| ....|....| ....|....| ....|....| ....|....| ....|....| ....|....| ....|....| ....|....| ....|....| ....|....| ....|....| ....|....| ....|....| ....|....|
MT_reference      TCCTCCATAA TCAATCTACA CCAACTGCTC TTTCCTAACT GTTCCGTTTC CTTTGCTAGA TTTTCCCATC GCAGAAGAAT TGACAGACAG AGTGCACAAT TGGTAGCCAC TCTCAGATCA TTATAAAATA CCATCCCTTC CAATATACTC CTTATGGACA
Sanger_Consensus  ....T..... .......... .......... .......... .......... .......... ........C. .......... .......... .......... .......... ...A...... .......... ...G...... ...A...... ..........
TP47889_query
TP47889_hit
Haplotype1        ....T..... .......... .......... .......... .......... .......... ........C. .......... .......... .......... .......... ...A...... .......... ...G...... ...A...... ..........
Haplotype2        ....A..... .......... .......... .......... .......... .......... ........C. .......... .......... .......... .......... ...A...... .......... ...G...... ...A...... ..........
Haplotype3        ....T..... .......... .......... .......... ......A... .......... ........C. .......... .......... .......... .......... ...A...... .......... ...G...... ...A...... ..........
Haplotype4        ....T..... .......... .......... .......... .......... .......... ........C. .......... .......... .......... .......... ...A...... .......... ...G...... ...A...... ..........
Haplotype5        ....T..... .......... .......... .......... .......... .......... ........C. .......... .......... .......... .......... ...A...... .......... ...G...... ...A...... ..........
Haplotype6        ....T..... .......... .......... .......... .......... .......... ........C. .......... .......... .......... .......... ...A...... .......... ...G...... ...A...... ..........

                         330        340        350        360        370        380        390        400        410        420        430        440        450        460        470        480
                  ....|....| ....|....| ....|....| ....|....| ....|....| ....|....| ....|....| ....|....| ....|....| ....|....| ....|....| ....|....| ....|....| ....|....| ....|....| ....|....|
MT_reference      TTAAATACCC AACAGTACAT TTCAACTCTT CCTGTTTAGT GTTTATTTGA TCTGATATTC TGTTGAACAA TTCTAACAGG CTGTAAGAAG CAATAATCTT GACAACGGGA GAACCAAAAT GCAGAAGTCT GCACAAATCA TGACAACGGA TGCCTATGAA
Sanger_Consensus  .......... .......... .......... .A........ .......... .......... .......... .......... .......... .......... .......... .......... .......... .......... ......T... ......C...
TP47889_query
TP47889_hit
Haplotype1        .......... .......... .......... .A........ .......... .......... .......... ..........
Haplotype2        .......... .......... .......... .A........ .......... .......... .......... ..........
Haplotype3        .......... .......... .......... .A........ .......... .......... .......... ..........
Haplotype4        .......... .......... .......... .A........ .......... .......... .......... ..........
Haplotype5        .......... .......... .......... .A........ .......... .......... .......... ..........
Haplotype6        .......... .......... .......... .A........ .......... .......... .......... ..........

                         490        500        510        520        530        540
                  ....|....| ....|....| ....|....| ....|....| ....|....| ....|....| ....|...
MT_reference      GGCTAGTGGT TCTGATGAAT TCA TTGAAA CAAGGAATTT TTGCCAATCC ACAAACCCTG GAAGGATA
Sanger_Consensus  A......... .......... ... ...... .......... .......... ....
TP47889_query
TP47889_hit
Haplotype1
Haplotype2
Haplotype3
Haplotype4
Haplotype5
Haplotype6
```

TP61949-TP14949

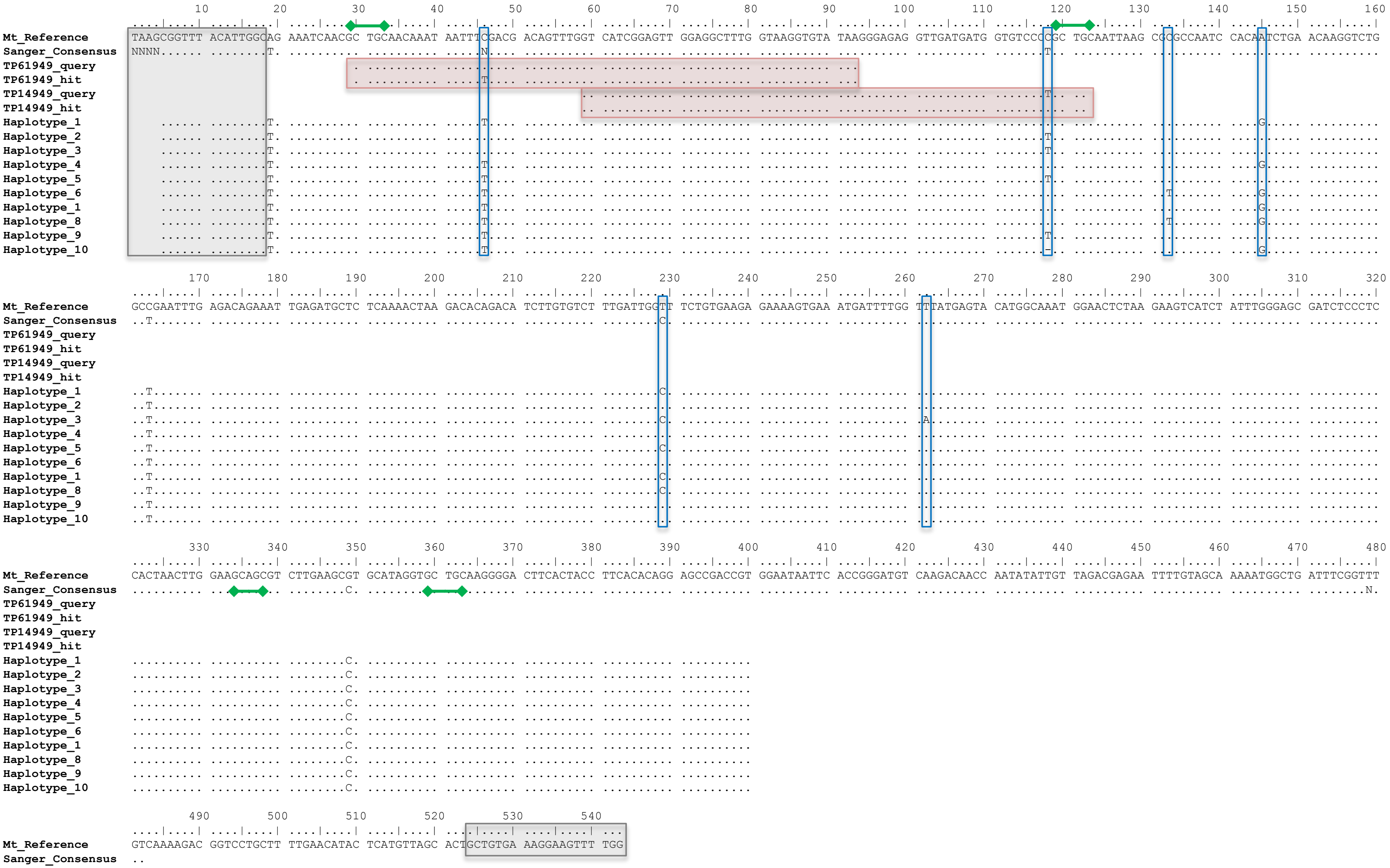

TP31029

```
                            10         20         30         40         50         60         70         80         90        100        110        120        130        140        150        160
Mt_reference      CTGCTGTTGC GATTAAGAGG CTTACTAATG GAGGGCAGCA GGGTGACAAA GAGCTTTTGG TTGAGGTTGA GATGCTTAGC CGGCTGCATC ACCGTAACCT TGTAAAACTA GTTGGATACT ATAGCAATCG TGACTCATCA CAAAACCTAC TTTGCTATGA
Sanger_Consensus             ...A...... ....A..... .......... .....R.... .......... .......... .......... .......... .......... .......... .....R.... .......... .......... .......... ..........
TP31029_Query                                .....  .......... .......... .......... .......... ......
TP31029_Hit                                  .....  .......... .......C.. .......... .......... ......
Haplotype1        .......... .......... ....A..... .......... .......... .......... .......... .......... .......... .......... .......... .....G.... .......... .......... .......... ..........
Haplotype2        .......... .......... ....A..... .......... .......... .......... .......... .......... .......... .......... .......... .......... .......... .......... .......... ..........
Haplotype3        .......... .......... ....A..... .......... .......... .......... .......... .......... .......... .......... .......... .......... .......... .......... .......... ..........
Haplotype4        .......... .......... ....A..... .......... .......... .......... .......... .......... .......... .......... .......... .....G.... .......... .......... .......... ..........
Haplotype5        .......... .......... ....A..... .......... .......... .......... .......... .......... .......... .......... .......... .....G.... .......... .......... .......... A.........
Haplotype6        .......... .......... ....A..... .......... .......... .......... .......... .......... .......... .......... .......... .....G.... .......... .......... .......... ..........
Haplotype7        .......... .......... ....A..... .......... .......... .......... .......... .......... .......... .......... .......... .......... .......... .......... .......... ..........
Haplotype8        .......... .......... ....A..... .......... .......... .......C.. .......... .......... .......... .......... .......... .......... .......... .......... .......... ..........
Haplotype9        .......... .......... ....A..... .......... .......... .......C.. .......... .......... .......... .......... .......... .......... .......... .......... .......... ..........
Haplotype11       .......... .......... ....A..... ......T... .......... .......... .......... .......... .......... .......... .......... .....G.... .......... .......... .......... ..........
Haplotype10       .......... .......... ....A..... .......... .......... A......... .......... .......... .......... .......... .......... .......... .......... .......... .......... ..........

                           170        180        190        200        210        220        230        240        250        260        270        280        290        300        310        320
Mt_reference      GCTTGTCCCA AATGGAAGTT TGGAGGCCTG GCTTCACGGT ATTGTAATTT CTCGAATAAT GGGTTCAGTT TTTTAATCTT GAATGACCTG ---------- ------TTAC ATTGATAGTT ATTTTCAGAA ATAATTTCAT TATGCTTAAT TGAAATTTTC
Sanger_Consensus  .......... .......... .......... ......T... .......... .......... .......... .......... .......... CTTTGTGTCT CTATTG.... .......... .......... .......... ......C... .......GG.
TP31029_Query
TP31029_Hit
Haplotype1        .......... .......... .......... ......T... .......... .......... .......... .......... .......... CTTTGTGTCT CTATTG.... .......... .......... .......... ......C... .......GG.
Haplotype2        .......... .......... .......... ......T... .......... .......... .......... .......... .......... CTTTGTGTCT CTATTG.... .......... .......... .......... ......C... .......GG.
Haplotype3        .......... .......... .......... ......T... .......... .......... .......... .......... .......... CTTTGTGTCT CTATTG.... .......... .......... .......... ......C... .......GG.
Haplotype4        .......... .......... .......... ......T... .......... .......... .......... .......... .......... CTTTGTGTCT CTATTG.... .......... .......... .......... ......C... .......GG.
Haplotype5        .......... .......... .......... ......T... .......... .......... .......... .......... .......... CTTTGTGTCT CTATTG.... .......... .......... .......... ......C... .......GG.
Haplotype6        .......... .......... .......... ......T... .......... .......... .......... .......... .......... CTTTGTGTCT CTATTG.... .......... .......... .......... ...C..C... .......GG.
Haplotype7        .......... .......... .......... ......T... .......... .......... .......... .......... .......... CTTTGTGTCT CTATTT.... .......... .......... .......... ......C... .......GG.
Haplotype8        .......... .......... .......... ......T... .......... .......... .......... .......... .......... CTTTGTGTCT CTATTG.... .......... .......... .......... ......C... .......GG.
Haplotype9        .......... .......... .......... ......T... .......... .......... .......... .......... .......... CTTTGTGTCT CTATTG.... .......... .......... .......... ...C..C... .......GG.
Haplotype11       .......... .......... .......... ......T... .......... .......... .......... .......... .......... CTTTGTGTCT CTATTG.... .......... .......... .......... ......C... .......GG.
Haplotype10       .......... .......... .......... ......T... .......... .......... .......... .......... .......... CTTTGTGTCT CTATTT.... .......... .......... .......... ......C... .......GG.

                           330        340        350        360        370        380        390        400        410        420        430        440        450        460        470        480
Mt_reference      CCTGATTTCC CTAGGTCCCT TGGGAATAAA TTGCTCTCTG GATTGGGACA CCAGGATGAA GATTGCACTT GATGCTGCAA GAGGACTTGC TTACCTGCAT GAAGATTCAC AGCCATGTGT CATACACAGA GATTTCAAGG CATCTAATAT ATTGCTTGAG
Sanger_Consensus  .......... .......... .......... .......... .......... .......... .......... .....C.... .......... .......... .......... .......... .......... .......... .......... ..........
TP31029_Query
TP31029_Hit
Haplotype1        .......... .......... .......... .......... .......... .......... .......... .....C....
Haplotype2        .......... .......... .......... .......... .......... .......... .......... .....C....
Haplotype3        .......... .......... .......... .......... .......... .......... .......... .....C....
Haplotype4        .......... .......... .......... .......... .......... .......... .......... .....C....
Haplotype5        .......... .......... .......... .......... .......... .......... .......... .....C....
Haplotype6        .......... .......... .......... .......... .......... .......... .......... .....C....
Haplotype7        .......... .......... .......... .......... .......... .......... .......... .....C....
Haplotype8        .......... .......... .......... .......... .......... .......... .......... .....C....
Haplotype9        .......... .......... .......... .......... .......... .......... .......... .....C....
Haplotype11       .......... .......... .......... .......... .......... .......... .......... .....C....
Haplotype10       .......... .......... .......... .......... .......... .......... .......... .....C....

                           490        500        510        520        530        540        550        560        570        580
Mt_reference      AACAACTTTC ATGCTAAAGT TGCTGATTTT GGTCTAGCTA AACTAGCACC TGAAGGCAGA GTTAATTATC TGTCTACTCG AGTTATGGGC A  CATTTGG
Sanger_Consensus  .......... .......... .......... .......... .......... ...G...... .......... .......... ........N. NNN.......
```

TP46847

```
                          10         20         30         40         50         60         70         80         90        100        110        120        130        140        150        160
                  ....|....| ....|....| ....|....| ....|....| ....|....| ....|....| ....|....| ....|....| ....|....| ....|....| ....|....| ....|....| ....|....| ....|....| ....|....| ....|....|
Mt_Reference      CAGCGAGAAC TCTTGATCCA GGTGCCATTA TGTCAGGCTT CAAAATCCAA GGAAAGCCAT GTGATGGACC TCTTGATGAG TAATGAGCTG CAATTGGTGC CGGTTTTATT CCAAGAAATG TTTGTTGAAA CTTAATGCTT GCTGTTGGAT TATTCTTATT
Sanger_Consensus      ...... .......... .......... .......... .........T .......... .......... .......... .......... .......... T..C...... .......... .......... ......A... .......... ....Y.....
TP46847_query                             .... .......... .........T .......... .......... .......... ..........
TP46847_hit                               .... .......... .......... .......... .......... .......... ..........
Haplotype1        .......... .......... .......... .......... .........T .......... .......... .......... .......... .......... T..C...... .......... .......... ......A... .......... ....T.....
Haplotype2        .......... .......... .......... .......... .........T .......... .......... .......... .......... .......... T..C...... .......... .......... ......A... .......... ..........
Haplotype3        .......... .......... .......... .......... .........T .......... .......... .......... .......... .......... T..C...... .......... .......... ......A... .......... ..........
Haplotype4        .......... .......... .......... .......... .......... .......... .......... .......... .......... .......... T..C...... .......... .......... ......A... ..A....... ..........
Haplotype5        .......... .......... .......... .......... .......... .......... .......... .......... .......... .......... T..C...... .......... .......... ......A... ..A....... ..........

                         170        180        190        200        210        220        230        240        250        260        270        280        290        300        310        320
                  ....|....| ....|....| ....|....| ....|....| ....|....| ....|....| ....|....| ....|....| ....|....| ....|....| ....|....| ....|....| ....|....| ....|....| ....|....| ....|....|
Mt_Reference      GCGTTTTGCG TACTTGATCA CAGATTCTGC ATCTTTTGTC TTGATAACAA TACCTGGAGC GTAGATATGT CTTAAATCAA TTAATTCAGG ACTATCAGAG AGAAACACTG CCCCTAACAT ATTGGTTTGT GTAACAACAT CGATTTGATC AAACACTGAG
Sanger_Consensus  .......... .......... .......... .......... .....T.... .......G.. A......... .......... .......... .......... .......... .........C ..C..C...C .......... .......... ..........
TP46847_query
TP46847_hit
Haplotype1        .......... .......... .......... .......... .....T.... .......G.. A......... .......... .......... .......... .......... .........C ..C..C...C .......... .......... ..........
Haplotype2        .......... .......... ......T... .......... .....T.... .......G.. A......... .......... .......... .......... .......... .........C ..C..C...C .......... .......... ..........
Haplotype3        .........A .......... .......... .......... .....T.... .......G.. A......... .......... .......... .......... .......... .........C ..C..C...C .......... .......... ..........
Haplotype4        .......... .......... .......... .......... .....T.... .......G.. A......... .......... .......... .......... .......... .........C ..C..C...C .......... .......... G.........
Haplotype5        .......... .......... .......... .......... .....T.... .......G.. A......... .......... .......... .......... .......... .........C ..C..C...C .......... .......... ..........

                         330        340        350        360        370        380        390        400        410        420        430        440        450        460        470        480
                  ....|....| ....|....| ....|....| ....|....| ....|....| ....|....| ....|....| ....|....| ....|....| ....|....| ....|....| ....|....| ....|....| ....|....| ....|....| ....|....|
Mt_Reference      CTTGAGTTTG ATATTGAGTC ACAAATAATT ATGACTTGCT TGTTAACTTG AGATAAACGC TTCACTGAAT TGCATGAAGA TAAAGTGTTA TCGTAAACAA GAGGAAGATT TTCGACTATA GTTGAATTTG ATGCAAACAA AGTCCAACCA ATAATGTTTT
Sanger_Consensus  .......... .N........ .........G .........C .......... .......... .......... .......... .......... .......... .......... ...A..A... .......... .......... .......... ..........
TP46847_query
TP46847_hit
Haplotype1        .......... .A........ .........G .........C .......... .......... .......... ..........
Haplotype2        .......... .A........ .........G .........C .......... .......... .......... ..........
Haplotype3        .......... .A........ .........G .........C .......... .......... .......... ..........
Haplotype4        .......... .C........ ...G.....G .......... .......... .......... .......... ..........
Haplotype5        .......... .A........ .........G .........C .......... .......... .......... ..........

                         490        500        510        520        530        540        550
                  ....|....| ....|....| ....|....| ....|....| ....|....| ....|....| ....|....| .
Mt_Reference      GACCATTCCC CAAAACAAGG CTGCCAAAAG TTCTATCTAT GGTTCCTGCA GCTACAGTAA GAA-CCCAAG G
Sanger_Consensus  .....N.N.. M...N..... .......... .......... .......... .......... ...A...... .
TP46847_query
TP46847_hit
Haplotype1
Haplotype2
Haplotype3
Haplotype4
Haplotype5
```

TP17289

```
                            10         20         30         40         50         60         70         80         90        100        110        120        130        140        150        160
                    ....|....| ....|....| ....|....| ....|....| ....|....| ....|....| ....|....| ....|....| ....|....| ....|....| ....|....| ....|....| ....|....| ....|....| ....|....| ....|....|
Mt_Reference        CTCTGGATAA GTGTTCCCAT TCTCAATTAC TGGTTCAACT TTGGAATTAT GCTGCCAAAC CAGAAAGGTA GAATCAGACA TTGGAAACAA AGAAATGGGA AACATTGCAA TGTTAATAGT GACAACAAAA ATGGAGTATA GGAAAATGTT CTCATTGCAA
Sanger_Consensus    .......... .......... .......... .......... .......... .......... .......... .......... .......... .....C.... .......... .......... .......... ......A... .......... ..........
TP17289_query       .......... .......... .......... .......... .........C ....
TP17289_hit         .......... .......... .......... .......... .......... ....
Haplotype1          .......... .......... .......... .......... .......... .......... .......... .......... .......... .....C.... .......... .......... .......... ......A... .......... ..........
Haplotype2          .......... .......... .......... .......... .......... .......... .......... .......... .......... .....C.... .......... .......... .......... ......A... .......... ..........
Haplotype3          .......... .......... .......... .......... .......... .......... .......... .......... .......... .....C.... .........G .......... .......... ......A... .......... ..........
Haplotype4          .......... .......... .......... .......... .......... .......... .......... .......... .......... .....C.... .......... .......... .......... ......A... .......... ..........
Haplotype5          .......... .......... .......... .......... .......... .......... .......... .......... .......... .....C.... .......... .......... .......... ......A... .......... ..........
Haplotype6          .......... .......... .......... .......... .........C .......... .......... .......... .......... .....C.... .......... .......... .......... ......A... .......... ..........
Haplotype7          .......... .......... .......... .......... .........C .......... .......... .......... .......... .....C.... .......... .......... .......... ......A... .......... ..........

                           170        180        190        200        210        220        230        240        250        260        270        280        290        300        310        320
                    ....|....| ....|....| ....|....| ....|....| ....|....| ....|....| ....|....| ....|....| ....|....| ....|....| ....|....| ....|....| ....|....| ....|....| ....|....| ....|....|
Mt_Reference        GTCTTTTGAT GCAAATTTTA GTTGATATGC CTCTTACATG AATCTATTTC TAAAGATCAA ACCCTTAGTG CCATATCATG AAATCTGTAA ATGATATTAC AACAAAATCA CCCAACTATC TTAGTTGCAT GAATTGTTGG CAGGTGAAAG GTATTATGAC
Sanger_Consensus    T......... .......... ....C..... ......A... .......... .......... ......G... .......... .......A.. .......... ..T....... ....G..... .......... ........T. .G.......T ..........
TP17289_query
TP17289_hit
Haplotype1          T......... .......... ....C..... ......A... .......... .......... ......G... .......... .......A.. .......... ..T....... ....G..... .......... ........T. .G.......T ..........
Haplotype2          T......... .......... ....C..... ......A... .......... .......... ......G... .......... .......A.. .......... .......... ....G..... .......... ........T. .G.......T ..........
Haplotype3          T......... .......... ....C..... ......A... .......... .......... ......G... .......... .......A.. .......... .......... ....G..... .......... ........T. .G.......T ..........
Haplotype4          T....A.... .......... ....C..... ......A... .......... .......... ......G... .......... .......A.. .......... .......... ....G..... .......... ........T. .G.......T ..........
Haplotype5          T......... .......... ....C..... ......A... .......... .......... ......G... .......... .......A.. .......... .......... ....G..... .......... ........T. .G.......T ..........
Haplotype6          T......... .......... ....C..... ......A... .......... .......... ......G... .......... .......A.. .......... ..........  ...G..... .......... ........T. .G.......T ..........
Haplotype7          T......... .......... ....C..... ......A... .......... .......... ......G... .......... .......A.. .......... ..T....... .. .G..... .......... ........T. .G.......T ..........

                           330        340        350        360        370        380        390        400        410        420        430        440        450        460        470        480
                    ....|....| ....|....| ....|....| ....|....| ....|....| ....|....| ....|....| ....|....| ....|....| ....|....| ....|....| ....|....| ....|....| ....|....| ....|....| ....|....|
Mt_Reference        ACAAGCAACT ATACAATACC CCGAATTTCA TTGGCCAAAA TATTGATTTC TTACTTGTTT GACC------ ---AACCAGA TCGTGCTCAG TCAGTACCTA CTCTTAACGT TAGTTTGATA TAATATATGT GTATAACACG TTAAGCTAAA TCCAAGCC-A
Sanger_Consensus    .......... .......... .......CT. .......... .......... .......... ....AATAAT GAA.....T. .......... .A.....T.. ....-..... .......... ...C...... .......... .A........ ........A.
TP17289_query
TP17289_hit
Haplotype1          .......... .......... .......CT. .......... .......... .......... ....AATAAT GAA.....
Haplotype2          .......... .......... .......CT. .......... .......... .......... ....AATAAT GAA.....
Haplotype3          .......... .......... .......CT. .......... .......... .......... ....AATAAT GAA.....
Haplotype4          .......... .......... .......CT. .......... .......... .......... ....AATAAT GAA.....
Haplotype5          .......... .......... .......CT. .......... .......... .......... ....AATGAT GAA.....
Haplotype6          .......... .......... .......CT. .......... .......... .......... ....AATAAT GAA.....
Haplotype7          .......... .......... .......CT. .......... .......... .......... ....AATAAT GAA.....

                           490        500        510        520        530        540        550        560        570
                    ....|....| ....|....| ....|....| ....|....| ....|....| ....|....| ....|....| ....|....| ....|....| ....
Mt_Reference        AAAGCATAAG TACATCTCAG AAAGAACAAG ACTCGGTATT ATATATAGCA TTACGAATAC TCACATTCAT TTGAACGTCT ATTACAGG-C TCTG
Sanger_Consensus    .......... ....A...C. .......... ....A..... .......... ....A..... .......... .......... ...NNNN.G. ...N
TP17289_query
TP17289_hit
Haplotype1
Haplotype2
Haplotype3
Haplotype4
Haplotype5
Haplotype6
Haplotype7
```

TP1933-TP26408

```
                          10         20         30         40         50         60         70         80         90        100        110        120        130        140        150        160
                  ....|....| ....|....| ....|....| ....|....| ....|....| ....|....| ....|....| ....|....| ....|....| ....|....| ....|....| ....|....| ....|....| ....|....| ....|....| ....|....|
Mt_reference       AGCAAAGTC AGCATCTATG GCATCATCTA GCAATGTTTT TCCGTGATGA GCTACTGTCT TGGTCTTGGA GAAGACCACT TGGGATGCCC ATGGCCCCTA TGGCTGCAGG TGGTACTATG AGCCCTGTTG ACTTTAAACA AAAGGTTATT ACAAATGTTG
Sanger_Consensus   T........ .......... .......... .......... .......... ...M...... .......... .......... .......... .......... .......... .......... .......... .......... .......... ........G.
TP1933_query      C......... .......... .......... .......... .......... .......... ....
TP1933_hit        C......... .......... .......... .......... .......... ...C...... ....
TP26408_query                                                  ......... ...C...... .......... .......... .......... .......... .......
TP26408_hit                                                    ......... .......... .......... .......... .......... .......... .......
Haplotype_1        ......... .......... .......... .......... .......... .......... .......... .......... .......... .......... .......... .......... .......... .......... .......... ........G.
Haplotype_2        ......... .......... .......... .......... .......... .......... .......... .......... .......... .......... .......... .......... .......... .......... .......... ........G.
Haplotype_3        ......... .......... .......... .......... .......... .......... .......... .......... .......... .......... .......... .......... .......... .......... .......... ........G.
Haplotype_4        ......... .......... .......... .......... .......... .......... .......... .......... .......... .......... .......... .......... .......... .......... .......... ........G.
Haplotype_5        ......... .......... .......... .......... .......... ...C...... .......... .......... .......... .......... .......... .......... .......... .......... .......... ........G.
Haplotype_6        ......... .......... .......... .......... .......... ...C...... .......... .......... .......... .......... .......... .......... .......... .......... .......... ........G.
Haplotype_7        ......... .......... .......... .......... .......... ...C...... .......... .......... .......... .......... .......... .......... .......... .......... .......... ........G.
Haplotype_8        ......... .......... .......... .......... .......... ...C...... .......... .......... .......... .......... .......... .......... .......... .......... .......... ........G.
Haplotype_9        ......... .......... .......... .......... .......... .......... .......... .......... .......... .......... .......... .......... .......... .......... .......... ........G.

                         170        180        190        200        210        220        230        240        250        260        270        280        290        300        310        320
                  ....|....| ....|....| ....|....| ....|....| ....|....| ....|....| ....|....| ....|....| ....|....| ....|....| ....|....| ....|....| ....|....| ....|....| ....|....| ....|....|
Mt_reference      AACATGTAGT TGGGAGAGTT AAGGGAATAG CACCGCAGAA TTTTTCTGAT GAGGTGAGTA GCTAAA~TAT TGGCTCTATT TTTTAATTTT ------AATT TTATGTTTTG TTCTAATATG ATTGTTAAGT CTAAGTTCAT GCTTCTTTTT TAATTGATGG
Sanger_Consensus  .......... .......... ....C..... .M........ .......... .......... ......A... ..C....... .......... TATTTC.... .......... .......... .......... .......... .......... ..........
TP1933_query
TP1933_hit
TP26408_query
TP26408_hit
Haplotype_1       .......... .......... ....C..... .......... .......... .......... ......A... ..C....... .......... AATTTC.... .......... .......... .......... .......... .......... ..........
Haplotype_2       .......... .......... ....C..... .......... .......... .......... ......A... ..C....... .......... TATTTC.... .......... .......... .......... .......... .......... ..........
Haplotype_3       .......... .......... ....C..... .......... .......... .......... ......A... ..C....... .........C AATTTC.... .......... .......... .......... .......... .......... ..........
Haplotype_4       .......... .......... ....C..... .......... .......... .......... ......AA.. ..C....... .......... AATTTC.... .......... .......... .......... .......... .......... ..........
Haplotype_5       .......... .......... ....C..... .......... .......... .......... ......AA.. ..C....... .......... AATTTC.... .......... .......... .......... .......... .......... ..........
Haplotype_6       .......... .......... ....C..... .......... .......... .......... ......AA.. ..C....... .........C AATTTC.... .......... .......... .......... .......... .......... ..........
Haplotype_7       .......... .......... ....C..... .......... .......... .......... ......AA.. ..C....... .........C AATTTC.... .......... .......... .......... .......... .......... ..........
Haplotype_8       .......... .......... ....C..... .......... .......... .......... ......AA.. ..C....... .......... TATTTC.... .......... .......... .......... .......... .......... ..........
Haplotype_9       .......... .......... ....C..... .......... .......... .......... ......AA.. ..C....... .........C AATTTC.... .......... .......... .......... .......... .......... ..........

                         330        340        350        360        370        380        390        400        410        420        430        440        450        460        470        480
                  ....|....| ....|....| ....|....| ....|....| ....|....| ....|....| ....|....| ....|....| ....|....| ....|....| ....|....| ....|....| ....|....| ....|....| ....|....| ....|....|
Mt_reference      AATGGGGAGA TAAATGATTA TATAAAAAAA TTCCAATGCG TTGTTCTACC AAAAA~TTCA AGAATCCAAT TGATCCTCCA CACAAGTCTC TATTGATTTC GTAATAACTA CTTAAAAATC TAATGACGAA AACT~GATGC CTTGCCTTTC CACTTTCAAT
Sanger_Consensus  .......... .......... .........T .......C.. .......... .....A.... .......... .......... .......... .......... ....G...C. .......... ......G... ....G..... ........YY MM...YM..Y
TP1933_query
TP1933_hit
TP26408_query
TP26408_hit
Haplotype_1       .......... .......... .........T .......C.. .......... .....~.... .......... .........M .N.GT
Haplotype_2       .......... .......... ........TT .......C.. .......... .....~.... .......... .........M .N.GT
Haplotype_3       .......... .......... .........T .......C.. .......... .....~.... .......... .........M .N.GT
Haplotype_4       .......... .......... .........T .......C.. .......... .....~.... .......... .........M .N.GT
Haplotype_5       .......... .......... .........T .......C.. .......... .....~.... .......... .........M .N.GT
Haplotype_6       .......... .......... .........T .......C.. .......... .....~.... .......... .........M .N.GT
Haplotype_7       .......... .......... .........T .......C.. .......... .....~.... .......... .........M .N.GT
Haplotype_8       .......... .......... ........TT .......C.. .......... .....~.... .......... .........M .N.GT
Haplotype_9       .......... .......... .........T .......C.. .......... .....~.... .......... .........M .N.GT

                         490        500        510        520        530
                  ....|....| ....|....| ....|....| ....|....| ....|....|
Mt_reference      TCATTGATTG ATATTTGTTC CTGTTTTTTT CCCTTCAAAT TTCTCACTCC
Sanger_Consensus  Y......... .....G.K.. .........N .......... .......NNN
```
