# Supplementary material for: Validation of Genotyping-By-Sequencing Analysis in Populations of Tetraploid Alfalfa by 454 Sequencing
Source: PLoS One. 2015 Jun 26;10(6):e0131918. doi: 10.1371/journal.pone.0131918 (PMC4482585; doi:10.1371/journal.pone.0131918)
Supplement: S3 Fig — A) GBS and 454 read counts of each allele (A1|A2); B) predicted tetraploid allelic ratios, with ratios identified as convergent in green and discordant in red; C) bi-allelic predicted genotype (A1, A2 and H) before genotype-level filtration and D) after genotype-level filtration of GBS data for minimum read counts (11 reads for homozygous genotypes, 2 reads of each allele for heterozygous genotypes, 0.1 as minimum minor allele frequency). Genotype calls showing concordance (green), discordance (red) with both sequencing methods or that are missing (white) before and after genotype-level filtration for minimum read counts. (PDF) [file pone.0131918.s003.pdf]

S3 Fig.: Comparison of GBS and 454 sequencing of 14 SNP loci in eight plant samples. A) GBS and 454 read counts of each allele (A1|A2); B) predicted tetraploid allelic ratios, with ratios identified as convergent in green and discordant in red; C) bi-allelic predicted genotype (A1, A2 and H) before genotype-level filtration and D) after genotype-level filtration of GBS data for minimum read counts (11 reads for homozygous genotypes, 2 reads of each allele for heterozygous genotypes, 0.1 as minimum minor allele frequency). Genotype calls showing concordance (green), discordance (red) with both sequencing methods or that are missing (white) before and after genotype-level filtration for minimum read counts.

A)

		TF0-17	TF0-20	TF0-36	TF0-38	TF5-5	TF5-12	TF5-20	TF5-28
TP67636	454	376 | 198	345 | 104	503 | 101	353 | 105	513 | 288	747 | 5	561 | 325	216 | 581
	GBS	4 | 0	5 | 110	25 | 49	1 | 37	40 | 175	9 | 0	6 | 224	3 | 94
TP7278	454	505 | 2	192 | 0	587 | 98	642 | 3	192 | 1	995 | 0	242 | 0	400 | 0
	GBS	11 | 0	251 | 0	500 | 342	24 | 0	55 | 1	133 | 0	43 | 0	186 | 0
TP80194	454	686 | 2	526 | 148	837 | 2	339 | 154	966 | 2	458 | 193	630 | 0	459 | 0
	GBS	16 | 0	33 | 21	16 | 0	6 | 0	7 | 0	6 | 0	20 | 0	2 | 0
TP79240	454	710 | 0	676 | 1	831 | 0	587 | 0	943 | 0	770 | 0	613 | 136	650 | 1
	GBS	18 | 0	29 | 0	6 | 0	31 | 0	2 | 0	49 | 0	20 | 1	17 | 0
TP91313	454	342 | 144	815 | 0	548 | 187	935 | 4	453 | 2	672 | 1	682 | 2	576 | 0
	GBS	1 | 47	500 | 0	356 | 0	224 | 0	95 | 0	290 | 0	320 | 0	42 | 0
TP32628	454	59 | 32	1 | 64	12 | 48	34 | 1	12 | 19	34 | 42	27 | 9	66 | 33
	GBS	11 | 23	0 | 35	0 | 25	38 | 0	9 | 9	14 | 28	13 | 15	15 | 27
TP47889	454	297 | 0	206 | 246	203 | 75	130 | 407	251 | 219	158 | 365	278 | 269	396 | 7
	GBS	14 | 0	10 | 3	4 | 0	2 | 5	0 | 0	4 | 4	16 | 8	13 | 0
TP61949	454	185 | 161	215 | 58	365 | 0	242 | 62	100 | 252	415 | 132	246 | 133	529 | 6
	GBS	17 | 1	43 | 20	19 | 0	2 | 9	0 | 21	23 | 3	29 | 5	11 | 0
TP14949	454	264 | 90	202 | 54	356 | 0	57 | 3	199 | 151	399 | 151	259 | 127	104 | 0
	GBS	0 | 0	124 | 30	39 | 0	11 | 0	10 | 0	24 | 153	113 | 91	61 | 1
TP31029	454	228 | 0	408 | 2	325 | 2	451 | 2	336 | 0	241 | 57	218 | 79	254 | 82
	GBS	31 | 0	39 | 0	10 | 0	14 | 0	9 | 0	6 | 0	11 | 5	0 | 8
TP46847	454	10 | 169	122 | 243	6 | 376	9 | 271	22 | 301	10 | 221	13 | 239	3 | 285
	GBS	29 | 45	117 | 32	64 | 50	32 | 31	54 | 6	37 | 12	93 | 20	52 | 8
TP17289	454	157 | 55	272 | 0	113 | 60	147 | 195	193 | 64	183 | 69	279 | 0	134 | 67
	GBS	8 | 12	50 | 0	34 | 32	10 | 9	14 | 15	37 | 7	45 | 0	0 | 2
TP1933	454	25 | 0	12 | 14	16 | 9	9 | 27	15 | 9	12 | 12	5 | 18	1 | 19
	GBS	34 | 0	19 | 50	13 | 5	20 | 17	16 | 9	29 | 20	7 | 19	9 | 24
TP26408	454	11 | 0	4 | 21	11 | 11	10 | 25	7 | 9	7 | 26	5 | 26	6 | 28
	GBS	19 | 0	13 | 12	11 | 19	4 | 43	22 | 0	10 | 8	24 | 64	0 | 6

B)

		TF0-17	TF0-20	TF0-36	TF0-38	TF5-5	TF5-12	TF5-20	TF5-28
TP67636	454	3 | 1	3 | 1	3 | 1	3 | 1	3 | 1	4 | 0	3 | 1	1 | 3
	GBS	4 | 0	0 | 4	1 | 3	0 | 4	1 | 3	4 | 0	0 | 4	0 | 4
TP7278	454	4 | 0	4 | 0	3 | 1	4 | 0	4 | 0	4 | 0	4 | 0	4 | 0
	GBS	4 | 0	4 | 0	2 | 2	4 | 0	4 | 0	4 | 0	4 | 0	4 | 0
TP80194	454	4 | 0	3 | 1	4 | 0	3 | 1	4 | 0	3 | 1	4 | 0	4 | 0
	GBS	4 | 0	2 | 2	4 | 0	4 | 0	4 | 0	4 | 0	4 | 0	4 | 0
TP79240	454	4 | 0	4 | 0	4 | 0	4 | 0	4 | 0	4 | 0	3 | 1	4 | 0
	GBS	4 | 0	4 | 0	4 | 0	4 | 0	4 | 0	4 | 0	4 | 0	4 | 0
TP91313	454	3 | 1	4 | 0	3 | 1	4 | 0	4 | 0	4 | 0	4 | 0	4 | 0
	GBS	0 | 4	4 | 0	4 | 0	4 | 0	4 | 0	4 | 0	4 | 0	4 | 0
TP32628	454	3 | 1	0 | 4	1 | 3	4 | 0	2 | 2	2 | 2	3 | 1	3 | 1
	GBS	1 | 3	0 | 4	0 | 4	4 | 0	2 | 2	1 | 3	2 | 2	1 | 3
TP47889	454	4 | 0	2 | 2	3 | 1	1 | 3	2 | 2	1 | 3	2 | 2	4 | 0
	GBS	4 | 0	3 | 1	4 | 0	1 | 3	0 | 0	2 | 2	3 | 1	4 | 0
TP61949	454	2 | 2	3 | 1	4 | 0	3 | 1	1 | 3	3 | 1	3 | 1	4 | 0
	GBS	4 | 0	3 | 1	4 | 0	1 | 3	0 | 4	4 | 0	3 | 1	4 | 0
TP14949	454	3 | 1	3 | 1	4 | 0	4 | 0	2 | 2	3 | 1	3 | 1	4 | 0
	GBS	0 | 0	3 | 1	4 | 0	4 | 0	4 | 0	1 | 3	2 | 2	4 | 0
TP31029	454	4 | 0	4 | 0	4 | 0	4 | 0	4 | 0	3 | 1	3 | 1	3 | 1
	GBS	4 | 0	4 | 0	4 | 0	4 | 0	4 | 0	4 | 0	3 | 1	0 | 4
TP46847	454	0 | 4	1 | 3	0 | 4	0 | 4	0 | 4	0 | 4	0 | 4	0 | 4
	GBS	2 | 2	3 | 1	2 | 2	2 | 2	0 | 4	3 | 1	3 | 1	3 | 1
TP17289	454	3 | 1	4 | 0	3 | 1	2 | 2	3 | 1	3 | 1	4 | 0	3 | 1
	GBS	2 | 2	4 | 0	2 | 2	2 | 2	2 | 2	3 | 1	4 | 0	0 | 4
TP1933	454	4 | 0	2 | 2	3 | 1	1 | 3	3 | 2	2 | 2	1 | 3	0 | 4
	GBS	4 | 0	1 | 3	3 | 1	2 | 2	3 | 1	2 | 2	1 | 3	1 | 3
TP26408	454	4 | 0	1 | 3	2 | 2	1 | 3	2 | 2	1 | 3	1 | 3	1 | 3
	GBS	4 | 0	2 | 2	1 | 3	0 | 4	4 | 0	2 | 2	1 | 3	0 | 4

C)

		TF0-17	TF0-20	TF0-36	TF0-38	TF5-5	TF5-12	TF5-20	TF5-28
TP67636	454	H	H	H	H	H	A1	H	H
	GBS	A1	H	H	A2	H	A1	H	H
TP7278	454	A1	A1	H	A1	A1	A1	A1	A1
	GBS	A1	A1	H	A1	A1	A1	A1	A1
TP80194	454	A1	H	A1	H	A1	H	A1	A1
	GBS	A1	H	A1	A1	A1	A1	A1	A1
TP79240	454	A1	A1	A1	A1	A1	A1	H	A1
	GBS	A1	A1	A1	A1	A1	A1	A1	A1
TP91313	454	H	A1	H	A1	A1	A1	A1	A1
	GBS	A2	A1	A1	A1	A1	A1	A1	A1
TP32628	454	H	A2	H	A1	H	H	H	H
	GBS	H	A2	A2	A1	H	H	H	H
TP47889	454	A1	H	H	H	H	H	H	A1
	GBS	A1	H	A1	H	N	H	H	A1
TP61949	454	H	H	A1	H	H	H	H	A1
	GBS	A1	H	A1	H	A2	H	H	A1
TP14949	454	H	H	A1	A1	H	H	H	A1
	GBS	N	H	A1	A1	A1	H	H	A1
TP31029	454	A1	A1	A1	A1	A1	H	H	H
	GBS	A1	A1	A1	A1	A1	A1	H	A2
TP46847	454	A2	H	A2	A2	A2	A2	A2	A2
	GBS	H	H	H	H	H	H	H	H
TP17289	454	H	A1	H	H	H	H	A1	H
	GBS	H	A1	H	H	H	H	A1	A2
TP1933	454	A1	H	H	H	H	H	H	A2
	GBS	A1	H	H	H	H	H	H	H
TP26408	454	A1	H	H	H	H	H	H	H
	GBS	A1	H	H	H	A1	H	H	A2

D)

		TF0-17	TF0-20	TF0-36	TF0-38	TF5-5	TF5-12	TF5-20	TF5-28
TP67636	454			H		H			
	GBS			H		H			
TP7278	454	A1	A1	H	A1		A1	A1	A1
	GBS	A1	A1	H	A1		A1	A1	A1
TP80194	454	A1	H	A1				A1	
	GBS	A1	H	A1				A1	
TP79240	454	A1	A1		A1		A1		A1
	GBS	A1	A1		A1		A1		A1
TP91313	454		A1	H	A1	A1	A1	A1	A1
	GBS		A1	A1	A1	A1	A1	A1	A1
TP32628	454	H	A2	H	A1	H	H	H	H
	GBS	H	A2	A2	A1	H	H	H	H
TP47889	454	A1	H		H		H	H	A1
	GBS	A1	H		H		H	H	A1
TP61949	454		H	A1	H	H	H	H	A1
	GBS		H	A1	H	A2	H	H	A1
TP14949	454		H	A1	A1		H	H	
	GBS		H	A1	A1		H	H	
TP31029	454	A1	A1	A1	A1			H	
	GBS	A1	A1	A1	A1			H	
TP46847	454	A2	H	A2	A2	A2	A2	A2	A2
	GBS	H	H	H	H	H	H	H	H
TP17289	454	H	A1	H	H	H	H	A1	
	GBS	H	A1	H	H	H	H	A1	
TP1933	454	A1	H	H	H	H	H	H	A2
	GBS	A1	H	H	H	H	H	H	H
TP26408	454	A1	H	H		H	H	H	
	GBS	A1	H	H		A1	H	H	

Genotype calls

**454**	A1 or A2	H	A1 or A2	H	
**GBS**	A1 or A2	H	H	A1 or A2	N
	Concordant		Discordant		

A1 or A2: Homozygous H : Heterozygous N: Missing
