# Supplementary material for: Validation of Genotyping-By-Sequencing Analysis in Populations of Tetraploid Alfalfa by 454 Sequencing
Source: PLoS One. 2015 Jun 26;10(6):e0131918. doi: 10.1371/journal.pone.0131918 (PMC4482585; doi:10.1371/journal.pone.0131918)
Supplement: S1 Table — Number of hits on M. truncatula (v4.0) genome and chromosome assignation on M. truncatula according to [20] and M. sativa according to [12] are provided when available. (PDF) [file pone.0131918.s004.pdf]

Name	Filter	Nb hit (Mt4.0)	Mt Chr (Mt4.0)	Ms Chr (Li et al., 2014)	Sequence
TP10038_Hit	D	1	chr1	.	CAGCACCGAATTTAGGCAGACTATCCTTTTTTAAACTTTTAAACGGCCCTGCTAAAGGTTCCAG
TP10038_Query	D	1	chr1	.	CAGCACCAAATTTAGGCAGACTATCCTTTTTTAAACTTTTAAACGGCCCTGCTAAAGGTTCCAG
TP10105_Hit	D	1	chr1	.	CAGCACCAAGCGATGTAAATGTTGCTTGGTAACTAAATGGTTGAAGGAGAAATTGAGTGGAAAA
TP10105_Query	D	1	chr1	.	CAGCACCAAGCAATGTAAATGTTGCTTGGTAACTAAATGGTTGAAGGAGAAATTGAGTGGAAAA
TP10202_Hit	D	1	chr1	.	CAGCACCACATTTCCACTTGTTTACAGGATTCTTCAAGTTAGTTACAGTTGCCAGGTATCCATT
TP10202_Query	D	1	chr1	.	CAGCACCACATTTCCACTTGTTTACAGGATTCTTCAAGTTAGTTACAGTGGCCAGGTATCCATT
TP10271_Hit	D	1	chr1	.	CAGCACCACTTACTAAGCTCCACATGTGATAGTGATGCTGAAAAAAAAAAAAAAAAAAAAAAAA
TP10271_Query	D	1	chr1	.	CAGCACCACTTACTAAGCTCCACATGTGATAATGATGCTGAAAAAAAAAAAAAAAAAAAAAAAA
TP10511_Hit	D	1	chr1	.	CAGCACCCAAGAATTATTGTCGTCTGCCTTGTGGAATGGATATAATTCAAATCTAACACCACAA
TP10511_Query	D	1	chr1	.	CAGCACCCAAGAATTATTGCCGTCTGCCTTGTGGAATGGATATAATTCAAATCTAACACCACAA
TP1054_Hit	D	1	chr1	.	CAGCAAAATTTCGATAACAAGCAATATAATGTGACATAAGTGATAGATGTTTGAAAACACTAGG
TP1054_Query	D	1	chr1	.	CAGCAAAATTTCGATAACAAACAATATAATGTGACATAAGTGATAGATGTTTGAAAACACTAGG
TP10558_Hit	D	1	chr1	.	CAGCACCCCATGAGGGCTTTGGAGTTGAAGTGCTTTTAGTGCCCCAAGCAGTATTCTGATTGTC
TP10558_Query	D	1	chr1	.	CAGCACCCCATGAGGGCTTTGGAGTTGAAGTACTTTTAGTGCCCCAAGCAGTATTCTGATTGTC
TP10590_Hit	D+G	1	chr1	.	CAGCACCCGATTGTGGAGATGCATAAACAGGAGCATTAGTGGGATAGGAATTATAGCCTTGGTT
TP10590_Query	D+G	1	chr1	.	CAGCACCCGATTGTGGAGATGCATAAACAGGAGCATTAGTGGGATAGGAATTAAAGCCTTGGTT
TP10608_Hit	D+G	1	chr1	.	CAGCATCCTCATGGCCCTCATTCCTGCTACATTGAGTATATTCGTAGCAACACTGGGTTAGTTA
TP10608_Query	D+G	1	chr1	.	CAGCACCCTCATGGCCCTCATTCCTGCTACATTGAGTATATTCGTAGCAACACTGGGTTAGTTA
TP10990_Hit	D	1	chr1	.	CAGCACCTGGATCAAATAGTTGTGACGTAAAATGGCCAGTTAAAGAGTGAAAAGTCAAAGAATT
TP10990_Query	D	1	chr1	.	CAGCACCTGGATCAAATAGTTGTGAAGTAAAATGGCCAGTTAAAGAGTGAAAAGTCAAAGAATT
TP11065_Hit	D	1	chr1	.	CAGCACCTTGCTGTTGAAGATATTAAGGAGAATCCACAGAGACCTTCTTTGGTTAGAAACTTTC
TP11065_Query	D	1	chr1	.	CAGCACCTTGCTGTTGAAGATATTAAGGAGAATCCACAGAGACCTTCTCTGGTTAGAAACTTTC
TP11140_Hit	D	1	chr1	.	CAGCACGAATATCAGTTGAAGACAATGAAGCAGGAATAGGGTAAGATAGTATTGAATTGGGAAA
TP11140_Query	D	1	chr1	.	CAGCACGAATATCAGTTGAAGACAATGAAGCAGGAATAGGGAAAGATAGTATTGAATTGGGAAA
TP11219_Hit	D	1	chr1	.	CAGCACGAGGTTCACTTTCATTTACTCTCTCTTCATTATCATTTCCACCATGCAGATAATCACG
TP11219_Query	D	1	chr1	.	CAGCACGAGGTTCACTTTCATTTACTCTCTCTTCATTACCATTTCCACCATGCAGATAATCACG
TP11450_Hit	D+G	1	chr1	.	CAGCACGGAGGTTACGGTTGTAGCATTCCTCGTGCATCTCCTTGAATAGAGCCCACTGGCTCCT
TP11450_Query	D+G	1	chr1	.	CAGCACGGAGGTTACGGTTGTAGCATTCCTCATGCATCTCCTTGAATAGAGCCCACTGGCTCCT
TP11589_Hit	D	1	chr1	.	CAGCACGTAGGAAAATCTGGTCAACTTCATCAAACTTGGGCAAGTCAGCATCTTTTTCCATGTT
TP11589_Query	D	1	chr1	.	CAGCACGTAGGAAAATCTGGTCAACCTCATCAAACTTGGGCAAGTCAGCATCTTTTTCCATGTT
TP11603_Hit	D	1	chr1	.	CAGCACGTCCTCCTGCACCACCTGTTGATCCTTACTCAAGATATGGCTCCAAAGCAGAAAAAAA
TP11603_Query	D	1	chr1	.	CAGCACGTCCTCCTGCACCACCTGTTGATCCTTACTCAAGATATGGCCCCAAAGCAGAAAAAAA
TP11669_Hit	D+G	1	chr1	.	CAGCTCGTTGGTATTTCAAATCAGAACCTGCGATGGATAAAGCCTGCAACAGGAAAGTTCAAAT
TP11669_Query	D+G	1	chr1	.	CAGCACGTTGGTATTTCAAATCAGAACCTGCGATGGATAAAGCCTGCAACAGGAAAGTTCAAAT
TP11808_Hit	D	1	chr1	.	CAGCGCTACTAAATATTTGGAAAAACTGATATCATAATTGAGGGCTGGGCCAATGGGCCCAAAA
TP11808_Query	D	1	chr1	.	CAGCACTACTAAATATTTGGAAAAACTGATATCATAATTGAGGGCTGGGCCAATGGGCCCAAAA
TP12046_Hit	D+G	1	chr1	.	CAGCACTCATTGTCAATTTACTATAATAAGCATACACGCTCTTTTTAGGTCTATCTGTTCATTC
TP12046_Query	D+G	1	chr1	.	CAGCACTCATTGTCAATTTACTATAATAAGCATACACACTCTTTTTAGGTCTATCTGTTCATTC
TP12048_Hit	D	1	chr1	.	CAGCACTCATTTGTTAATATATAATTGCAGGTTGTAACTTCGATAAATGATGCATCTGGAAGAG
TP12048_Query	D	1	chr1	.	CAGCACTCATTTATTAATATATAATTGCAGGTTGTAACTTCGATAAATGATGCATCTGGAAGAG
TP12085_Hit	D+G	1	chr1	.	CAGCACTCGGAGGGAAGTGTATCTGCAAGAATAGGGTGGTTGTTGTTGCTTACAGGTAAGGTGA
TP12085_Query	D+G	1	chr1	.	CAGCACTCGGAGGGAAGTGTATCTGCAAGAATAGGGTGGTTGTTGTTGCTGACAGGTAAGGTGA
TP12136_Hit	D	1	chr1	.	CAGCACTCTTGACCACTCCTCATGTGTAACATCTTTATGCGAAAGCAAATCGCTCATTTTCAAT
TP12136_Query	D	1	chr1	.	CAGCACTCTTGACCACTCCTCATGTGTAACATCTTTATGCGAAAGCAAATCACTCATTTTCAAT
TP12137_Hit	D	1	chr1	.	CAGCACTCTTGATTGAAGGAAGAGGTCTCTCAAGCTCAAAGTCTGTGTTGGGAAGATTTCTCCA
TP12137_Query	D	1	chr1	.	CAGCACTCTTGATAGAAGGAAGAGGTCTCTCAAGCTCAAAGTCTGTGTTGGGAAGATTTCTCCA
TP12279_Hit	D	1	chr1	.	CAGCACTGCTTTGATGAATGAAATGGTGTCTCTGATTTCTTGCAAATCTTGGTAGACGCCCAGC
TP12279_Query	D	1	chr1	.	CAGCACTGCTTTGATGAATGAAATGGTGTCTCTAATTTCTTGCAAATCTTGGTAGACGCCCAGC
TP12308_Hit	D	1	chr1	.	CAGCACTGGGGTGGACCCTGTTTGTACAACAAAATGAAATGCTCCTGTGGAGAAAATAACCGGG
TP12308_Query	D	1	chr1	.	CAGCACTGGGGTAGACCCTGTTTGTACAACAAAATGAAATGCTCCTGTGGAGAAAATAACCGGG
TP12351_Hit	D	1	chr1	.	CAGCACTGTCTGCGAAGATGCAGATTTGCTAGCTACTTGTTTATGGACCATTTGGAAGTGTAGA
TP12351_Query	D	1	chr1	.	CAGCACTGTCTGCGAAGATGCAGATTTGCTAGCTACTTGTTTACGGACCATTTGGAAGTGTAGA
TP12383_Hit	D+G	1	chr1	.	CAGCACTGTTCTGCTATGGTCCATGACATGTACAATCAATAGTACATGCAAATGTTGATGATGT
TP12383_Query	D+G	1	chr1	.	CAGCACTGTTCTGCTATGGTCCATGACATGCACAATCAATAGTACATGCAAATGTTGATGATGT

Name	Filter	Nb hit (Mt4.0)	Mt Chr (Mt4.0)	Ms Chr (Li et al., 2014)	Sequence
TP12503_Hit	D	1	chr1	.	CAGCACTTCCACTGGTTTCTCCTTGCTTACACATGTATAAACAAAAGGCACTTTGGTGCTGATT
TP12503_Query	D	1	chr1	.	CAGCACTTCCACTGCTTTCTCCTTGCTTACACATGTATAAACAAAAGGCACTTTGGTGCTGATT
TP12570_Hit	D	1	chr1	.	CAGCACTTGACTGTTGCTCATACACCAGAAACAAATAAGACCCTTCAAATTTCACATGCCAAAA
TP12570_Query	D	1	chr1	.	CAGCACTTGACCGTTGCTCATACACCAGAAACAAATAAGACCCTTCAAATTTCACATGCCAAAA
TP12622_Hit	D	1	chr1	.	CAGCACTTGGATTTTTTTGTGGAATGGTTGTGTATTTGCTTCTTAAGCTAAGGAATTGGACAAA
TP12622_Query	D	1	chr1	.	CAGCACTTGGATTTTTTTGTGGAATGATTGTGTATTTGCTTCTTAAGCTAAGGAATTGGACAAA
TP12685_Hit	D	1	chr1	.	CAGCACTTTCATATGATATAATTTCAGGAATAGAGACAACTAAGGAGAGATTTTATGCTGAGCT
TP12685_Query	D	1	chr1	.	CAGCACTTTCATATGATATAATTTCAGGAACAGAGACAACTAAGGAGAGATTTTATGCTGAGCT
TP12687_Hit	D+G	1	chr1	.	CAGCACTTTCCAAATAAGGAATAGCCTGCTTCACCTTACCTGTCAGCAACAACAACCACCCTAT
TP12687_Query	D+G	1	chr1	.	CAGCACTTTCCAAATAAGGAATAGCCTGCTTCACCTTACCTGTAAGCAACAACAACCACCCTAT
TP12743_Hit	D+G	1	chr1	.	CAGCACTTTTTTTTAAAAATTAGTACAATTATCAGGGTATAGACAATGTTATTAACAGAAAACG
TP12743_Query	D+G	1	chr1	.	CAGCACTTTTTTTAAAAAATTAGTACAATTATCAGGGTATAGACAATGTTATTAACAGAAAACG
TP12935_Hit	D	1	chr1	.	CAGCAGAAGTCGCTAAGACGATTCTCGTATCCCTCAAGGACCAGTTGAAAGAAGTAGACCTGTC
TP12935_Query	D	1	chr1	.	CAGCAGAAATCGCTAAGACGATTCTCGTATCCCTCAAGGACCAGTTGAAAGAAGTAGACCTGTC
TP1310_Hit	D	1	chr1	.	CAGCAAACCAGACTATGAGCGCGCCCCTTCTGAGCCAGAAAAACCTGTTCATGTTTATACACGT
TP1310_Query	D	1	chr1	.	CAGCAAACCAGACTATAAGCGCGCCCCTTCTGAGCCAGAAAAACCTGTTCATGTTTATACACGT
TP1323_Hit	D	1	chr1	.	CAGCAAACCATTTTGGAAAGAGCTAGGCATACCTCTCCAGCATAATGGGCAATGGTGAAATCTG
TP1323_Query	D	1	chr1	.	CAGCAAACCATTTTGGAAAGAGCTAGGCATACCTCTCCAGCATAATGAGCAATGGTGAAATCTG
TP13265_Hit	D	1	chr1	.	CAGCAGAAGTTTGTCCGGCTTATTCGCACAGAAGAGAAATGCATTGAATCCTTATTTTTGGCAA
TP13265_Query	D	1	chr1	.	CAGCAGAAGTTTGTCCGGCTTATTCGCACAAAAGAGAAATGCATTGAATCCTTATTTTTGGCAA
TP13283_Hit	D	1	chr1	.	CAGCAGAATATACATATATGTGCATAATAAAATTTTATTCATGTATACAGAGAATATTTGCTGA
TP13283_Query	D	1	chr1	.	CAGCAGAATATACATATATGTGCATAATAAAATTTGATTCATGTATACAGAGAATATTTGCTGA
TP13292_Hit	D+G	1	chr1	.	CAGCAGAATATTTACAAATGAATGAAAATTATGGTGAAAGGAATTTAATAGAAGTGACAAAAAA
TP13292_Query	D+G	1	chr1	.	CAGCAGAATATTTACAAATGAATGAAAATTATGGTGAAAGGAATTTAATAGAAGTAACAAAAAA
TP13346_Hit	D	1	chr1	.	CAGCAGAATGGAGAGAGGGTTTAGAGGCAGGAGCCAAGTTTTGGTTTGATTGGATCTTGTCACT
TP13346_Query	D	1	chr1	.	CAGCAGAATGGAGAGAGGGTTTAGAGGCAGGAGCCAAGTTTTGGTTTGAATGGATCTTGTCACT
TP13476_Hit	D	1	chr1	.	CAGCAGACATTACATTGGGAGTAGAAGAACATATGGGTGGGAATGGGAAAAATGGTGGAAGTTT
TP13476_Query	D	1	chr1	.	CAGCAGACATTACATTGGGAGTAGAAGAACATATAGGTGGGAATGGGAAAAATGGTGGAAGTTT
TP13531_Hit	D	1	chr1	.	CAGCAGACCTGAGAAGTTCCTGTTCAAAGTTCCGTATCCTAGCCTCAAGGTCACCCTTAATCTT
TP13531_Query	D	1	chr1	.	CAGCAGACCTGAGAAGTTCCTGTTCAAAGTTCCGTATCCTAGCCTCAAGATCACCCTTAATCTT
TP13548_Hit	D	1	chr1	.	CAGCAGACGCAGAAAACTTTAAGTTGATGAATATAATCTAGTTACCGGGTACAAGATGTCAAAA
TP13548_Query	D	1	chr1	.	CAGCAGACGCAGAAAACTTTAAGTTGATAAATATAATCTAGTTACCGGGTACAAGATGTCAAAA
TP13625_Hit	D	1	chr1	.	CAGCAGACTTTGCTTCTTTGAGCTCCGAAACACTCCTAATTAGCAAATTTTTCTCCTCAAGCAC
TP13625_Query	D	1	chr1	.	CAGCAGACTTTGCTTCTTTGAGCTCCAAAACACTCCTAATTAGCAAATTTTTCTCCTCAAGCAC
TP13630_Hit	D+G	1	chr1	.	CAGCAGAGAAAACATATCCTACCTTATTTCCAATAGCACGTCCAAAGCCACATGGAACTGCTGA
TP13630_Query	D+G	1	chr1	.	CAGCAGAGAAAACATACCCTACCTTATTTCCAATAGCACGTCCAAAGCCACATGGAACTGCTGA
TP13845_Hit	D+G	1	chr1	.	CAGCAGAGGCGTGCTTGTTAGCAGGCTCTGCTAGGAATGCCTACCACACAAAGTACCGAGGGAA
TP13845_Query	D+G	1	chr1	.	CAGCAGAGGCGTGCTTGTTAGCAGGCTCTGCCAGGAATGCCTACCACACAAAGTACCGAGGGAA
TP13908_Hit	D+G	1	chr1	.	CAGCAGAGTAGAGAAGTTTGGAGAATGAGGTTGCGGCGTTTGGAGGTGGTGGTGTTGTTGTTGT
TP13908_Query	D+G	1	chr1	.	CAGCAGAGTAGAGAAGTTTGGAGAATGAGGTTGCGGCGTTTGGAGGTGGTGGGGTTGTTGTTGT
TP13921_Hit	D	1	chr1	.	CAGCAGAGTCAAATACTGGTTTTCATTATGGTGATATGAATTCATCTTTGAATTGGCATGCAAT
TP13921_Query	D	1	chr1	.	CAGCAGAGTCAAATACTGATTTTCATTATGGTGATATGAATTCATCTTTGAATTGGCATGCAAT
TP14163_Hit	D	1	chr1	.	CAGCAGATCTTGCCTGGAATGCGGCCTGGTGTTGCTCCGAATTTTGTTATGCCATATCAGCTCC
TP14163_Query	D	1	chr1	.	CAGCAGATCTTGCCTGGAATGCGGCCTGGTGTTCCTCCGAATTTTGTTATGCCATATCAGCTCC
TP14403_Hit	D+G	1	chr1	.	CAGCAGGAAATACATTTAATGGGGGCAACTTTAAGCCAAATTTGGAAGGATGGAAAGTTGATTC
TP14403_Query	D+G	1	chr1	.	CAGCAGGAAATACATTTAATGGGGGCAACTTGAAGCCAAATTTGGAAGGATGGAAAGTTGATTC
TP14573_Hit	D	1	chr1	.	CAGCAGGAGACTCTTGGCTTTGCAATGGATTCAAATCACTCTTCTTTTCAGGCTCGGATATATC
TP14573_Query	D	1	chr1	.	CAGCAGGAGACGCTTGGCTTTGCAATGGATTCAAATCACTCTTCTTTTCAGGCTCGGATATATC
TP14586_Hit	D	1	chr1	.	CAGCAGGAGCAAGCTTATGCATAATCTTCTATGCGTCATCAAGAGACCCTTCTTTTCACAATTA
TP14586_Query	D	1	chr1	.	CAGCAGGAGCAAGCTTATGCATAATATTCTATGCGTCATCAAGAGACCCTTCTTTTCACAATTA
TP14656_Hit	D	1	chr1	.	CAGCAGGAGGGGCACCGACTACTTGGTCCTCACCAGTTTCCAGGATAGGTGCTTAGATCGGAAG
TP14656_Query	D	1	chr1	.	CAGCAGGAGGGGCACCGACTACTTGGTCCTCACCAGTTTCCAGGATAGGTGCTAAGATCGGAAG
TP14657_Hit	D	1	chr1	.	CAGCAGGAGGGGCACCGACTACTTGGTCCTCACCAGTTTCCAGGATAGGTGCTGATATCGGAAG
TP14657_Query	D	1	chr1	.	CAGCAGGAGGGGCACCGACTACTTGGTCCTCACCAGTTTCCAGGATAGGTGCTGAAATCGGAAG

Name	Filter	Nb hit (Mt4.0)	Mt Chr (Mt4.0)	Ms Chr (Li et al., 2014)	Sequence
TP14823_Hit	D+G	1	chr1	.	CAGCAGGTCCATTCATCATCATACCATCACCAGGCCCTAGCTGTGGCCCACGACCATTCTGCAT
TP14823_Query	D+G	1	chr1	.	CAGCAGGCCCATTCATCATCATACCATCACCAGGCCCTAGCTGTGGCCCACGACCATTCTGCAT
TP1485_Hit	D	1	chr1	.	CAGCAAACTGCACGGAGCTAAATGGCTGTGGAGGTTCAGCTACAAATTGCACTGAGTGCACAGG
TP1485_Query	D	1	chr1	.	CAGCAAACTGCACGGAGCTAAATGGCTGTGGAGGTTCAGCTACAAACTGCACTGAGTGCACAGG
TP14914_Hit	D+G	1	chr1	.	CAGCAGGCTTAGCTATCAGTTTGGCCACAGAACGTGGTTTTCGCCGAGAAGAAACCACCGGTGC
TP14914_Query	D+G	1	chr1	.	CAGCAGGCTTAGCTAACAGTTTGGCCACAGAACGTGGTTTTCGCCGAGAAGAAACCACCGGTGC
TP15003_Hit	D+G	1	chr1	.	CAGCAGGGATGAGGCCCCTAATACCTCCCCCATCAATACTAAGAATGGTTACCAAGTTCCCGTA
TP15003_Query	D+G	1	chr1	.	CAGCAGGGATGAGGCCCCTAATACCTCCCCCATCAATACTAAGAATGGTTACCAAGTTCCCATA
TP15061_Hit	D	1	chr1	.	CAGCAGGGGAGGAAAAAGTTATAACACTCCGCCATTTCAAAGGTCTTTGCAATGGACATATCTG
TP15061_Query	D	1	chr1	.	CAGCAGGGGAGGAAAAAGTTATAACACTCCGCCATTTCAAAGGTCTTAGCAATGGACATATCTG
TP15182_Hit	D	1	chr1	.	CAGCAGGTACAGGATACATGTACTTGGGTGTGGTCTCAATGAAATGAATCTACACATCCACTGT
TP15182_Query	D	1	chr1	.	CAGCAGGTACAAGATACATGTACTTGGGTGTGGTCTCAATGAAATGAATCTACACATCCACTGT
TP15267_Hit	D	1	chr1	.	CAGCAGGTCGGCCATATGCATCATCGCCGGTTCTCTCCCTTCTCAAAGCTGAAAAAAAAAAAAA
TP15267_Query	D	1	chr1	.	CAGCAGGTCGGCCATATGCATCATCGCCGGTTCTCTCCCTTCTCAAAGCAGAAAAAAAAAAAAA
TP15300_Hit	D+G	1	chr1	.	CAGCAGGTGCACTTAGCAATCTGGTTCGCAACTCTGACAGACTTTGTGAAGACATTGTGTCTAA
TP15300_Query	D+G	1	chr1	.	CAGCAGGTGCACTTAGCAATCTGGTTCGCAACTCTGACAGACTTTGTGAAGACATCGTGTCTAA
TP15505_Hit	D	1	chr1	.	CAGCAGTAAACAAAGCAGTTTCACCAAGTTCATTCTCTTCATTCACAACAAGAGCACGAACTTC
TP15505_Query	D	1	chr1	.	CAGCAGTAAACAAAGCAGTTTCACCAAGCTCATTCTCTTCATTCACAACAAGAGCACGAACTTC
TP15532_Hit	D	1	chr1	.	CAGCAGTAACAGCTTCAAATTGTGAATGTGAGCCCTCACTACCATACATCACATCATGCAAAAG
TP15532_Query	D	1	chr1	.	CAGCAGTAACAGCTTCAAATTGTGAATGTGAGCCCTCACTACCATACATCACATCATCCAAAAG
TP15724_Hit	D	1	chr1	.	CAGCAGTAGCCTGAGAGGTTTGCCTCGACATATGGAGGAGTTGTTGTTGCTGAAAAAAAAAAAA
TP15724_Query	D	1	chr1	.	CAGCAGTAGCCTGAGAGGTTTGCCTCGACATATGGAGGAGTTGTTGGTGCTGAAAAAAAAAAAA
TP15812_Hit	D	1	chr1	.	CAGCAGTATCAAGAAGGGACTTGCCATTTTGAGCCACGATTCTCAGTTGTTCAATCCATATTTT
TP15812_Query	D	1	chr1	.	CAGCAGTATCAAGAAGGGACTTGCCATTTTGAGCCACGATTCTCAGTTGTTCAACCCATATTTT
TP15846_Hit	D+G	1	chr1	.	CAGCAGTATTAAACAGGGATGCAGATGGCCTAAATGCCTGCGACGAAGAATTGTCCCCAAACAT
TP15846_Query	D+G	1	chr1	.	CAGCAGTATTAAACAGGGATGCAGATGGCCTAAATGCCTGCGACGAAGAATTGTCACCAAACAT
TP15922_Hit	D+G	1	chr1	.	CAGCAGTCATACAGTATTCTATGTGCATGGATTGGAGACCGAATATAACGCATACTCATAATAG
TP15922_Query	D+G	1	chr1	.	CAGCAGTCATACAGTATTCTACGTGCATGGATTGGAGACCGAATATAACGCATACTCATAATAG
TP16044_Hit	D	1	chr1	.	CAGCAGTCTGTCTTTCGGATCTGTCAATGTCCAATAACTGGCGTTACATGATGTTCAACTACGA
TP16044_Query	D	1	chr1	.	CAGCAGTCTGTCTTTCGGATCTGTCAATGTCCAATAACTGGAGTTACATGATGTTCAACTACGA
TP16086_Hit	D	1	chr1	.	CAGCAGTGAAATGGTGGTGGCGGCAGAACAATCGCGGCGGTGGCATGATGTTTTCTGGTTGGGA
TP16086_Query	D	1	chr1	.	CAGCAGTGAAATGGTGGTGGCGGCAGAACAATCGCGGCGGTGGCATGATGTTTTCTGGTTAGGA
TP16095_Hit	D+G	1	chr1	.	CAGCAGTGAAGACAAAGAGTCGTCTACAATCCACATAGAAGACATGTCACAAGAATCCTGCACG
TP16095_Query	D+G	1	chr1	.	CAGCAGTGAAGACAAAGAGTCGTCTACAATCCACATAGAAGACATGTCACAAGAATCCTGCACA
TP16165_Hit	D	1	chr1	.	CAGCAGTGATTGCCCATTTGCTGTCGTCAACCTGGTGAGTTAAGATCGCTAACAATTGAACTGA
TP16165_Query	D	1	chr1	.	CAGCAGTGATTGCCCATTTGCTGTCGTCAACCTGGTGAGTTAAGATCACTAACAATTGAACTGA
TP16241_Hit	D	1	chr1	.	CAGCAGTGGACGCGTTTCATCGCCACCTGCCCGATGAGCTACTGCTGAAAAAAAAAAAAAAAAA
TP16241_Query	D	1	chr1	.	CAGCAGTGGACGCGTTTCATCGCCACCTGCCCGATGAGCTACCGCTGAAAAAAAAAAAAAAAAA
TP16322_Hit	D	1	chr1	.	CAGCAGTGGTGGAAAAGAAAAGTTGCCGATGCTCCATCTGAAGCTGATTCTGGACCAACAGCTG
TP16322_Query	D	1	chr1	.	CAGCAGTGGTGGAAAAGAAAAGTTGCCGATGCTCCATCTGAAGCTGATTCTGGACCAACAGCAG
TP1636_Hit	D	1	chr1	.	CAGCAAAGAGAGATCTGACATCTTCAGTTGTATAAACTTCCTTTTTAATTTGACTCCGGGAGGC
TP1636_Query	D	1	chr1	.	CAGCAAAGAGAGATCTGACATCTTCAGGTGTATAAACTTCCTTTTTAATTTGACTCCGGGAGGC
TP16567_Hit	D+G	1	chr1	.	CAGCAGTTCCATGTGGCTTTGGACGTGCTATTGGAAATAAGGTAGGGTATGTTTTCTCTGCTGA
TP16567_Query	D+G	1	chr1	.	CAGCAGTTCCATGTGGCTTTGGACGTGCTATTGGAAATAAGGTAGGATATGTTTTCTCTGCTGA
TP16569_Hit	D+G	1	chr1	.	CAGCAGTTCCCACGAATGTGCAGGCTTTGTCATCTAAAATTTTTAAACAAATGAATAAGATGAT
TP16569_Query	D+G	1	chr1	.	CAGCAGTTCCCACGAATGTGCAGGCTTTGTCATCTAAAATTTTTAAACAAATAAATAAGATGAT
TP16625_Hit	D+G	1	chr1	.	CAGCAGTTGAAAGTCCAATGAAAATGAATGGACTTCCAATGAGTGTCTACGCACCTGAAACGGT
TP16625_Query	D+G	1	chr1	.	CAGCAGTTGAAAGTCCAATGAAAATGAATGGACTTCCAATGAGTGTCTACGCACCTGAAACAGT
TP16716_Hit	D	1	chr1	.	CAGCAGTTGGGAATAATATTTGCTCTGTATGATAAATTACAATTAGTCATCTAAGTGAGGGGTA
TP16716_Query	D	1	chr1	.	CAGCAGTTGGGAATAATATTTGCTCTGTATGATAAATTACAACTAGTCATCTAAGTGAGGGGTA
TP16746_Hit	D	1	chr1	.	CAGCAGTTGTCGGGGCCTCGGGCTCCTGTAAGTTTCAACCTGCGTTTCGGTGCGGAGATCGGAA
TP16746_Query	D	1	chr1	.	CAGCAGTTGTCGGGGCCTCGGGCTCCTGTAAGTTTCAACCTGCGTTTCGGTGCCGAGATCGGAA
TP16872_Hit	D	1	chr1	.	CAGCAGTTTTCGCAATGCAAGGCGTGGGAATCATCTTTGCCGGATTGGTTTCAATGTTATTTTC
TP16872_Query	D	1	chr1	.	CAGCAGTTTTCGCAATGCAAGGCGTGGGAATCATCTTTGCCGGATTGGTTTCAACGTTATTTTC

Name	Filter	Nb hit (Mt4.0)	Mt Chr (Mt4.0)	Ms Chr (Li et al., 2014)	Sequence
TP17021_Hit	D	1	chr1	.	CAGCATAAAGTTTTGCTAACAGAACATAATTTCCCGTGTTTTCTGGTTCAAGCTCAAAACTCCA
TP17021_Query	D	1	chr1	.	CAGCATAAAGTTTTGCCAACAGAACATAATTTCCCGTGTTTTCTGGTTCAAGCTCAAAACTCCA
TP17035_Hit	D	1	chr1	.	CAGCATAAATCTTGGTTAATTTAAACAAAGGTACAAAAAAATGAAAATAAAAAACATGAATAAA
TP17035_Query	D	1	chr1	.	CAGCATAAATCTTGGTTAATTTAAACAAAGGTACAAAAAAATGAAAATAAAAAACAAGAATAAA
TP17039_Hit	D+G	1	chr1	.	CAGCATAAATGGTTGGAGTATTTGGTATCGAGGAAACTTGATCACTTCCGTCAGAAAACCTACC
TP17039_Query	D+G	1	chr1	.	CAGCATAAATGGTTGGAGTATTTGGTATCGAGGAAACTTGATCACTTCCATCAGAAAACCTACC
TP17180_Hit	D	1	chr1	.	CAGCATAAGGTGTTTTACTATACTATGGAAAAGTGTATTCAACTTCTGATTGAGGGTTACTTGA
TP17180_Query	D	1	chr1	.	CAGCATAAGGTGTTTTACTATACTATGGAAAAGTGTATTCAACTTCCGATTGAGGGTTACTTGA
TP17244_Hit	D	1	chr1	.	CAGCATAATCTTGTCCACTGGTAGCTACCTGTGCATTAGGTGGAAGAAGGTTTCCAAGATCCAA
TP17244_Query	D	1	chr1	.	CAGCATAATCTTGTCCACTGGTAGCAACCTGTGCATTAGGTGGAAGAAGGTTTCCAAGATCCAA
TP17294_Hit	D	1	chr1	.	CAGCATAATTGAATTGAAAATCGTATTGTACTACATTAAATGATGTTACTCATATCTACATCCA
TP17294_Query	D	1	chr1	.	CAGCATAATTGAATTGAAAATCATATTGTACTACATTAAATGATGTTACTCATATCTACATCCA
TP17305_Hit	D+G	1	chr1	.	CAGCATAATTTCATATAACCTCCAAAAACTTCGGTAAATTGCAAATTAGTAATACCCTAAAATC
TP17305_Query	D+G	1	chr1	.	CAGCATAATTTCATATAACCTCCAAAAACTTCGGTAAATTGCAAAATAGTAATACCCTAAAATC
TP17447_Hit	D	1	chr1	.	CAGCATACCTGCTAGCACAGTAAACTCCAACGGCAGTTGCCGCCATTCCATAAACATTTGTAGT
TP17447_Query	D	1	chr1	.	CAGCATACCTGCTAGCACAATAAACTCCAACGGCAGTTGCCGCCATTCCATAAACATTTGTAGT
TP1750_Hit	D	1	chr1	.	CAGCAAAGCCCTACCCCAAACCCTAACCCTAATTTCACGCTTCTCTTCACTTTTCGTCCCTCTT
TP1750_Query	D	1	chr1	.	CAGCAAAGCCCTACCCCAAACCCTAACCCTAATTTCACGCTTCACTTCACTTTTCGTCCCTCTT
TP17515_Hit	D+G	1	chr1	.	CAGCATACTGCAAGGAGTTGAATTCCTTGTCCAAGTCAGGATTGCCTCCTCCATTTAATGTTCT
TP17515_Query	D+G	1	chr1	.	CAGCATACTGCAAGGAGTTGAATTCCTTGTCCAAGGCAGGATTGCCTCCTCCATTTAATGTTCT
TP17720_Hit	D	1	chr1	.	CAGCATAGTGTAGTGAAAGGGAGGCTTTACAGAAATATCAAATAGTTATAATAAAATGCAATGG
TP17720_Query	D	1	chr1	.	CAGCATAGTGTAGTAAAAGGGAGGCTTTACAGAAATATCAAATAGTTATAATAAAATGCAATGG
TP17746_Hit	D	1	chr1	.	CAGCATAGTTTAGCTGATGAACTTCTGAATGAAATGACAAAGAAGTTCAAGCATTCATGCAAAG
TP17746_Query	D	1	chr1	.	CAGCATAGTTTAGCAGATGAACTTCTGAATGAAATGACAAAGAAGTTCAAGCATTCATGCAAAG
TP1777_Hit	D+G	1	chr1	.	CAGCAAAGCTCCAAAGCTGAGCAAAATCCGGCCGCCACAACCCCGGCGGAGCAGTAGGTGCTGA
TP1777_Query	D+G	1	chr1	.	CAGCAAAGCTCCAAAGCTGAGCAAAATCCGGCCGCCAAAACCCCGGCGGAGCAGTAGGTGCTGA
TP17816_Hit	D+G	1	chr1	.	CAGCATATAGATTAATAGAGAAGACTGTTCTTTTACTCTATAATGGAGATATTCCTAGAAGCTA
TP17816_Query	D+G	1	chr1	.	CAGCATATAGATTAATAGAGAAGACTGTTCTCTTACTCTATAATGGAGATATTCCTAGAAGCTA
TP17902_Hit	D	1	chr1	.	CAGCATATCCATTGAATTTGTTAGATCCCCTGCTTGGTGATTCATCAGATGCTTTGCTATTGTT
TP17902_Query	D	1	chr1	.	CAGCATATCCATTGAATTTGTTAGATCCCCTGCTTGGCGATTCATCAGATGCTTTGCTATTGTT
TP1791_Hit	D	1	chr1	.	CAGCAAAGCTTGTTGAGTACTGGGAGACTGTTTTAAAGTACCTCCGCATATATAAAGCCAAGGT
TP1791_Query	D	1	chr1	.	CAGCAAAGCTTGTTGAGTACTGGGAGACTGTTTTAAAGTACCTCCACATATATAAAGCCAAGGT
TP17923_Hit	D	1	chr1	.	CAGCATATCTAAAACAAAAAGGTGTTCCAAGTTTAATACTAGAAAGATCAAATTGTATAGCTTC
TP17923_Query	D	1	chr1	.	CAGCATATCTAAAACAAAAAGGTGTTCCAAGTTTAATACTAGAAAGATCAAACTGTATAGCTTC
TP18011_Hit	D	1	chr1	.	CAGCATATGGAGGGTGTTAAAAAAAATTGCTAAATTAGAGGCTGAGTGTCAAAGGTTACGTGGTC
TP18011_Query	D	1	chr1	.	CAGCATATGGAGGGTGTTAAAAAAAATTGCTAAATTAGAGGCCGAGTGTCAAAGGTTACGTGGTC
TP18105_Hit	D	1	chr1	.	CAGCATATTGAAGATCTAACACAAGAAAAAATTTTCTTTGCAACGTGCTCTTGAGGCTTCTCGAG
TP18105_Query	D	1	chr1	.	CAGCATATTGAAGATCTAACACAAGAAAAAATTTTCTTTGCAACGCGCTCTTGAGGCTTCTCGAG
TP18138_Hit	D	1	chr1	.	CAGCATATTTCATTTCAAATTCCAACCCTAGAACACAGTCTGAGAAATGTATTCTTGTATTCCT
TP18138_Query	D	1	chr1	.	CAGCATATTTCATTTCAAATTCCAACCCTAGAACACAGTCTGAGAAATGTATTATTGTATTCCT
TP18187_Hit	D+G	1	chr1	.	CAGCATCAAAATTCTTTTGGTATATTTTCTTCAATTACTTCACTTTCTTGTACTACACCTTCTA
TP18187_Query	D+G	1	chr1	.	CAGCATCAAAATTCTTTTGGTATATTTTCTTCAATTACTTCACCTTCTTGTACTACACCTTCTA
TP18191_Hit	D+G	1	chr1	.	CAGCATCAAACAACCTGAAGAAACCTTGGTAGACGAGAACTTATCAGGAAATCAACATGAAGAG
TP18191_Query	D+G	1	chr1	.	CAGCATCAAACAACCTGAAGAAACCTTGGTAGACCAGAACTTATCAGGAAATCAACATGAAGAG
TP18221_Hit	D	1	chr1	.	CAGCATCAAAGTCCGGACCATGTTTCTCACGAAACTGATAAAGAGAGAATCAAGAAATCTCACG
TP18221_Query	D	1	chr1	.	CAGCATCAAAGTCCGGACCATGTTTCTCACGAAACTGATAAAGAGAGAACCAAGAAATCTCACG
TP18287_Hit	D	1	chr1	.	CAGCATCAACATCATTGTTGACACCATCAAATAGTATATTCAAATTTGGCCATAGTTCAAATCA
TP18287_Query	D	1	chr1	.	CAGCATCAACATCATTGTTGACACCACCAAATAGTATATTCAAATTTGGCCATAGTTCAAATCA
TP18432_Hit	D+G	1	chr1	.	CAGCATCAATTGATTTTCCTGTCCTTGCATGTGAGATTCGATTTGCATAAATGGTAACCAATTT
TP18432_Query	D+G	1	chr1	.	CAGCATCAATTGATTTTCCTGTCCTTGCATGTGAGATTCGATTTGCATAAATGGTAACCAACTT
TP18501_Hit	D	1	chr1	.	CAGCATCACCCTTTTCAGATATCAACTCCCCATTTTCCTCCTTCCCTCTTGGTCCTCTTTTCAA
TP18501_Query	D	1	chr1	.	CAGCATCACCCTTTTCAGATATCAACTCCCCATTCTCCTCCTTCCCTCTTGGTCCTCTTTTCAA
TP18540_Hit	D	1	chr1	.	CAGCATCACTCCTACCAATGAAAATGGTCTATTGTGCCTTCAAAGCACTTGGTGTTCGTGTCAT
TP18540_Query	D	1	chr1	.	CAGCATCACTCCTACCAATGAAAATGGTCTATTGTGCCATCAAAGCACTTGGTGTTCGTGTCAT

Name	Filter	Nb hit (Mt4.0)	Mt Chr (Mt4.0)	Ms Chr (Li et al., 2014)	Sequence
TP18645_Hit	D+G	1	chr1	.	CAGCATCAGGAGTACTCAGTTCAATTGTTAGTGATCTTAACTCACCAGGTTGACGACAGCAAAT
TP18645_Query	D+G	1	chr1	.	CAGCATCAGGAGTACTCAGTTCAATTGTTAGCGATCTTAACTCACCAGGTTGACGACAGCAAAT
TP18649_Hit	D	1	chr1	.	CAGCATCAGGATCACAACACAATTTTGATGTGTTTTATGTGAAACTAATCTGTGTAGGTGCGCT
TP18649_Query	D	1	chr1	.	CAGCATCAGGATCACAACACAATTTTGATGTGTTCTATGTGAAACTAATCTGTGTAGGTGCGCT
TP18746_Hit	D	1	chr1	.	CAGCATCATCCACTACCGACTTTGATGCTTTATCAACACCACCATCATCATCCTTGTTTTCAAC
TP18746_Query	D	1	chr1	.	CAGCATCATCCACTACCGACTTCGATGCTTTATCAACACCACCATCATCATCCTTGTTTTCAAC
TP18763_Hit	D	1	chr1	.	CAGCGTCATCTGCTGATTTTGTTCTCACGGCGAAACTAGTGTCGCGTCCTGCAACTTTAAGGAG
TP18763_Query	D	1	chr1	.	CAGCATCATCTGCTGATTTTGTTCTCACGGCGAAACTAGTGTCGCGTCCTGCAACTTTAAGGAG
TP18804_Hit	D	1	chr1	.	CAGCATCATTCACTTTACCAATATCAGATGTGGGATCAGATGACTTTCCATTGTTTTTAAGATC
TP18804_Query	D	1	chr1	.	CAGCATCATTCACTTTACCAATATCAGATGTGGGATCAGATGACTTTCCATTGTTTTCAAGATC
TP18818_Hit	D+G	1	chr1	.	CAGCATCATTGAAACTTTTGATAGAAGTGGCTGTTTGGGGATTGTGTCTACTCACTTGCATGAT
TP18818_Query	D+G	1	chr1	.	CAGCATCATTGAAACTCTTGATAGAAGTGGCTGTTTGGGGATTGTGTCTACTCACTTGCATGAT
TP18862_Hit	D	1	chr1	.	CAGCATCCAAATCATGAGTATTATGACACATGACAATGTAGTTAGATTTGAACTGTTTGATGAA
TP18862_Query	D	1	chr1	.	CAGCATCCAAATCATGAGTATTATAACACATGACAATGTAGTTAGATTTGAACTGTTTGATGAA
TP18921_Hit	D+G	1	chr1	.	CAGCATCCTGACTGCATTTTACATAACTGGTAGGTGCAGGTGTCCAATTTTCTGTCATGCAAAC
TP18921_Query	D+G	1	chr1	.	CAGCATCCAGACTGCATTTTACATAACTGGTAGGTGCAGGTGTCCAATTTTCTGTCATGCAAAC
TP18931_Hit	D+G	1	chr1	.	CAGCATCCATATCGGTTACATTCGAAGGTAGACCAATATTCAAGCAGGAGTTTTCAATTGCACG
TP18931_Query	D+G	1	chr1	.	CAGCATCCATATCGATTACATTCGAAGGTAGACCAATATTCAAGCAGGAGTTTTCAATTGCACG
TP18961_Hit	D	1	chr1	.	CAGCATCCCCAATTGATTCAAATGAGGACACTGAAAATTTCTCTTGACCTGCAGATCTCGGACT
TP18961_Query	D	1	chr1	.	CAGCATCCCCAATTGATTCAAATGAGGACACTGAAAATTTCTCTTCACCTGCAGATCTCGGACT
TP19023_Hit	D+G	1	chr1	.	CAGCATCCTATGCATAATAACAAGATGATGATGGCTATGATGCAGAATGGTCGTGGGCCACAGC
TP19023_Query	D+G	1	chr1	.	CAGCATCCTATGCATAATAACAAAATGATGATGGCTATGATGCAGAATGGTCGTGGGCCACAGC
TP19173_Hit	D	1	chr1	.	CAGCATCGCTGGACAACCGAAAGCCTTCTGAATCTGCTTTTGTCCCGACGTGAGATTTGTTCCC
TP19173_Query	D	1	chr1	.	CAGCATCGCTGGACAACCGAAAGCCTTCTGAATCTGCTTTTGGCCCGACGTGAGATTTGTTCCC
TP19265_Hit	D	1	chr1	.	CAGCATCTACAGTTTGTACAAATCTAGCGACCAATGGAAGAACCACCAATTCGCTATTGGAACC
TP19265_Query	D	1	chr1	.	CAGCATCTACAGTTTGTACAAATCTAGCCACCAATGGAAGAACCACCAATTCGCTATTGGAACC
TP19425_Hit	D+G	1	chr1	.	CAGCATCTGTAACTCATGCACTTGAACATTGCTCTGCATGGTCATCCCTTGCTCTAACTGTTGT
TP19425_Query	D+G	1	chr1	.	CAGCATCTGCAACTCATGCACTTGAACATTGCTCTGCATGGTCATCCCTTGCTCTAACTGTTGT
TP19540_Hit	D	1	chr1	.	CAGCATCTTCCCCTTCGCCGAATAGAGTTGCAACAACAGAAATTGATGGATTTTTCTTTTGTAA
TP19540_Query	D	1	chr1	.	CAGCATCTTCCCCTTCGCCGAATAGAGTTGCAACAACAGAAATTGATGGATTTTTCTTTTGCAA
TP19548_Hit	D	1	chr1	.	CAGCATCTTCGTTATTAGATGCCTACAGTAACAAGTATGTTCATTTTGAATTAGTTGTAATGCT
TP19548_Query	D	1	chr1	.	CAGCATCTTCGTTATTAGATGCCTACAGTAACAAGTATGTTCATTTTGAATTAGTTGCAATGCT
TP19572_Hit	D+G	1	chr1	.	CAGCATCTTGCTCTTCCCTTAAGCGTGCATTAGTTCGTCTTTCTTCTGCATCAAGCCTTGCTGA
TP19572_Query	D+G	1	chr1	.	CAGCATCTTGCTCTTCCCTTAAGCGTGCATTAGTTCGTCTTTCTTCTGCATCAAGCCGTGCTGA
TP19585_Hit	D+G	1	chr1	.	CAGCATCTTGTTCTGAAGCTGGAAGCAGTCGAATCCCAAGGCCCCTTTTCCTAGGTGCTGAAAA
TP19585_Query	D+G	1	chr1	.	CAGCATCTTGTTCTGAAGCTGGAAGCAGTCGAATCCCAAGGCCCCTTCTCCTAGGTGCTGAAAA
TP19587_Hit	D	1	chr1	.	CAGCATCTTGTTCTGAAGCTGGAAGCAGTCGAATCCCAAGGCCCCTTTTCCTAGGTGCGGAGAT
TP19587_Query	D	1	chr1	.	CAGCATCTTGTTCTGAAGCTGGAAGCAGTCGAATCCCAAGGCCCCTTTTCCTAGGTGCCGAGAT
TP19673_Hit	D	1	chr1	.	CAGCATGAAACTCTGTCAATAAGTCGCCGGGAAAAAAATTGAATTAGAATAATCTGTAACAATG
TP19673_Query	D	1	chr1	.	CAGCATGAAACTCTGTCAATAAGTCGCCAGGAAAAAAATTGAATTAGAATAATCTGTAACAATG
TP19759_Hit	D	1	chr1	.	CAGCATGAATTAAGAATTGCTGAAAATAAAGTGGCATCTCGAATCACCAAACCTTCAGATTTGG
TP19759_Query	D	1	chr1	.	CAGCATGAATTAAGAATTGCTGAAAATAAAGTGGCATCTCGAATCACCAAACCTTCAGAGTTGG
TP20021_Hit	D	1	chr1	.	CAGCATGCAAATACAAGCTACATAAGCAGAAATTAAGAAAAAATGATTGACATTAAACTAAAAA
TP20021_Query	D	1	chr1	.	CAGCATGCAAATAAAAGCTACATAAGCAGAAATTAAGAAAAAATGATTGACATTAAACTAAAAA
TP20051_Hit	D	1	chr1	.	CAGCATGCAATGGCGTGAGTCTGTGTATCATCACACTTTATGGCCAAATGGCTGTCAAACCAAT
TP20051_Query	D	1	chr1	.	CAGCATGCAATGGCGTGAGTCTGTGTATCATCACACTTTATGACCAAATGGCTGTCAAACCAAT
TP20117_Hit	D	1	chr1	.	CAGCATGCATCATACAAATATTCCGTGATGATCAAACCTTTCAACTATAGCAGTAGTAGTAGTC
TP20117_Query	D	1	chr1	.	CAGCATGCATCATACAAATATTCCGTGATGATTAAACCTTTCAACTATAGCAGTAGTAGTAGTC
TP20162_Hit	D	1	chr1	.	CAGCATGCCGCCATGTCTCAGAAGTCACTTCATATCGTCTTGTATTCCTCGACTCAAGTTCTGT
TP20162_Query	D	1	chr1	.	CAGCATGCCGCCATGTCTCAGAAGTCACTTCATACCGTCTTGTATTCCTCGACTCAAGTTCTGT
TP20210_Hit	D	1	chr1	.	CAGCATGCTTTTGAGAAGTTTCATTGTCTTCTTCATGATCAGGGCCTTCATCATCTGAAGTTTC
TP20210_Query	D	1	chr1	.	CAGCATGCGTTTGAGAAGTTTCATTGTCTTCTTCATGATCAGGGCCTTCATCATCTGAAGTTTC
TP2022_Hit	D	1	chr1	.	CAGCAAATAAATGGAAAGGAAACATAGATGCAATCCACTATATAGATAACAATGACGGGCCAGT
TP2022_Query	D	1	chr1	.	CAGCAAATAAATGGAAAGGAAACATAGATGCAATCCACTATATAGATAACAATGACAGGCCAGT

Name	Filter	Nb hit (Mt4.0)	Mt Chr (Mt4.0)	Ms Chr (Li et al., 2014)	Sequence
TP20221_Hit	D+G	1	chr1	.	CAGCATGCTAGTAATGATGTTGGTTTGAGTAGCATGCAGGTTGAACAATACGGTACTCGATTGT
TP20221_Query	D+G	1	chr1	.	CAGCATGCTAGTAACGATGTTGGTTTGAGTAGCATGCAGGTTGAACAATACGGTACTCGATTGT
TP20225_Hit	D+G	1	chr1	.	CAGCATGCTATTATGAGTATGCGTTATATTCGGTCTCCAATCCATGCACGTAGAATACTGTATG
TP20225_Query	D+G	1	chr1	.	CAGCATGCTATTATGAGTATGCGTTATATTCGGTCTCCAATCCATGCACATAGAATACTGTATG
TP20238_Hit	D	1	chr1	.	CAGCATGCTCCTCCATGTCCTCGGCGTCCTCTGTAATTGAAAACGAAACTAGAGATTCTAACTA
TP20238_Query	D	1	chr1	.	CAGCATGCTCCTCCATGTCCTCAGCGTCCTCTGTAATTGAAAACGAAACTAGAGATTCTAACTA
TP20243_Hit	D+G	1	chr1	.	CAGCATGCTCGCCGATCTGATTCTATTGCCGGCGACGAAGGACAAATGCGTGCTGAAAAAAAAA
TP20243_Query	D+G	1	chr1	.	CAGCATGCTCGCCGATCTGATTCTATTGCCGGCGACGAAGGACAAATGCATGCTGAAAAAAAAA
TP20258_Hit	D	1	chr1	.	CAGCATGCTGTTATTGACTGGGCACTGTGTCTGCACCCCTCAATATTTGGGTCGAAATTAAAGA
TP20258_Query	D	1	chr1	.	CAGCATGCTGTTATTGACTGGGCACTGTGTCTGCACCCCTCAATATTTGGGTCGAAATGAAAGA
TP20299_Hit	D	1	chr1	.	CAGCATGGAAAGCATCAAAATGTACAGGACAGTATGAATGCAGTCCTAAAAGAGCTTTGGGAGG
TP20299_Query	D	1	chr1	.	CAGCATGGAAAGCATCAAAATGTACAGGACAATATGAATGCAGTCCTAAAAGAGCTTTGGGAGG
TP2035_Hit	D	1	chr1	.	CAGCAAATAACCCTAAATCCAGCAATAATCAATCAACAGTTTGAAAAAGAATCTGCATCAAAAC
TP2035_Query	D	1	chr1	.	CAGCAAATAACCCCAAATCCAGCAATAATCAATCAACAGTTTGAAAAAGAATCTGCATCAAAAC
TP20362_Hit	D	1	chr1	.	CAGCATGGATGAGATCATTAGGGAATGCAATAGCATGTCAGGACATCTTGATGTTTTGAAATTG
TP20362_Query	D	1	chr1	.	CAGCATGGATGAAATCATTAGGGAATGCAATAGCATGTCAGGACATCTTGATGTTTTGAAATTG
TP20460_Hit	D	1	chr1	.	CAGCATGGTAAGATTATTTGAAAGTAATGTGTAGAAGTTCAATACTGAAACCCATGTTCGTTCG
TP20460_Query	D	1	chr1	.	CAGCATGGTAAGATTATTTGAAAGTAATGTGTAGAAGTTCAATACTGAAACCCATGTTCGTTCA
TP20537_Hit	D+G	1	chr1	.	CAGCATGGTTTTGCAAGCCATTGTGGATCCGGATTTGAGATTCCGTGACATAGTTACTGGATGG
TP20537_Query	D+G	1	chr1	.	CAGCATGGTTTTGCAAGCCATTGTGGATCCGGATATGAGATTCCGTGACATAGTTACTGGATGG
TP20773_Hit	D	1	chr1	.	CAGCATGTTAAAGGAAATTCATGAGGATAAAAGTGGTTCCTCTGATCAAAATGCAACGGCAGGT
TP20773_Query	D	1	chr1	.	CAGCATGTTAAAGGAAATTCATGAGGATAAAAGTGGTTCCTCTGATCAAAATGCAACGGCAGGG
TP20801_Hit	D	1	chr1	.	CAGCATGTTCAGTCAGTTTCGACAATATAATATCCAATCTCACAAGCAATTCCTCTCAACAGGC
TP20801_Query	D	1	chr1	.	CAGCATGTTCAGTCAGTTTCGACAATATAACATCCAATCTCACAAGCAATTCCTCTCAACAGGC
TP20810_Hit	D+G	1	chr1	.	CAGCATGTTCGATCCAAAAACCATGGTCCTCTTTCGGTTCCTTTTTGTCACATAAGCTGGATTC
TP20810_Query	D+G	1	chr1	.	CAGCATGTTCGATCCAAAAACCATGGTCCTCTTTCGGTTCCTTTTTGTCACATAAACTGGATTC
TP20815_Hit	D+G	1	chr1	.	CAGCATGTTCTAATGAAGATCAGATTGAGCAGGTTACAAGTGTGATTAGAACTATGCACAAGGA
TP20815_Query	D+G	1	chr1	.	CAGCATGTTCTAATGAAGATCAGATTGAACAGGTTACAAGTGTGATTAGAACTATGCACAAGGA
TP21023_Hit	D+G	1	chr1	.	CAGCATTACCTCTGTCGTTTATTTCCTCCTTTGGCTCACTTCTCTTCGCTACAGACCCAACTTT
TP21023_Query	D+G	1	chr1	.	CAGCATTACCTCTGTCATTTATTTCCTCCTTTGGCTCACTTCTCTTCGCTACAGACCCAACTTT
TP21072_Hit	D	1	chr1	.	CAGCGTTAGAGTGGGATCGTAGACAGGCCAAGAAAGGAAACTACAGCAATGGTCACTGGAAAAT
TP21072_Query	D	1	chr1	.	CAGCATTAGAGTGGGATCGTAGACAGGCCAAGAAAGGAAACTACAGCAATGGTCACTGGAAAAT
TP21086_Hit	D	1	chr1	.	CAGCATTAGCAATAAATTAAAATGATCAGCGCCAAATTAGATGCCACAGTTTTATGTGTTCCCT
TP21086_Query	D	1	chr1	.	CAGCATTAGCAATAAATTAAAATGATCAGCGCCAAAGTAGATGCCACAGTTTTATGTGTTCCCT
TP21102_Hit	D+G	1	chr1	.	CAGCATTAGCTATAGGAGGCACAGATGGCGAATGTGCACCTGAGTTACAGCTTGCTGAAAAAAA
TP21102_Query	D+G	1	chr1	.	CAGCATTAGCTATAGGAGGCACAGATGGCGAATGTGCACCTGAGTTACAGCGTGCTGAAAAAAA
TP21129_Hit	D	1	chr1	.	CAGCATTAGGCCATTTATTGTCGCTAATTTCCAAGGTTAATTCAAAACAGCCAGCATGTATGTA
TP21129_Query	D	1	chr1	.	CAGCATTAGGCCATTTATTGTCACTAATTTCCAAGGTTAATTCAAAACAGCCAGCATGTATGTA
TP21154_Hit	D	1	chr1	.	CAGCATTAGTTGGACAAAGTGGAAGTGGAAAGTCGACAGTTATCAGTTTGGTAGAGAGATTTTA
TP21154_Query	D	1	chr1	.	CAGCATTAGTTGGACAAAGTGGAAGTGGAAAGTCCACAGTTATCAGTTTGGTAGAGAGATTTTA
TP21169_Hit	D	1	chr1	.	CAGCATTATATAGTTGGTGATCACCAGATAAACAGAACTTTAATCCCTTCATTTGTTTACAATC
TP21169_Query	D	1	chr1	.	CAGCATTATAGAGTTGGTGATCACCAGATAAACAGAACTTTAATCCCTTCATTTGTTTACAATC
TP21239_Hit	D	1	chr1	.	CAGCATTATTGTGCTTCTAAGCTTCAAAGTCTACGGAGGATAACACCCGGTACACGTTTTATGC
TP21239_Query	D	1	chr1	.	CAGCATTATTGTGCTTCTAAGCTTCAAAGTCTACAGAGGATAACACCCGGTACACGTTTTATGC
TP21242_Hit	D	1	chr1	.	CAGCATTATTTATACAAGTCATAGCCTATAATCATTTTATAATTCTGCGTCGAATAAACATACT
TP21242_Query	D	1	chr1	.	CAGCATTATTTATACAAGTCATAGACTATAATCATTTTATAATTCTGCGTCGAATAAACATACT
TP21281_Hit	D+G	1	chr1	.	CAGCATTCAAGGGAGGAGGAGTTGAAAGTAGAAAAGAAAGACAATGTAGTGCAAAGCAATGAAC
TP21281_Query	D+G	1	chr1	.	CAGCATTCAAGGGAGGAGGAATTGAAAGTAGAAAAGAAAGACAATGTAGTGCAAAGCAATGAAC
TP21283_Hit	D	1	chr1	.	CAGCATTCAATATCTTTCTTGTTCTTTGTTGTATCTCACTTTTCTGTTTTGCTGGGTTTCGTGT
TP21283_Query	D	1	chr1	.	CAGCATTCAATATCTTTCTTGTTCTTTGTTGTATCTCACTTTTCTGTTTTGCTGGGTATCGTGT
TP21334_Hit	D	1	chr1	.	CAGCATTCAGTTTGAATCAGTGTTATGAATAGTGGATTGTGGAAATAGTGGAACACCGAATTCC
TP21334_Query	D	1	chr1	.	CAGCATTCAGTTTGAATCAGTGTTATGAATAGTGGATTGTGGAAATAGCGGAACACCGAATTCC
TP21498_Hit	D	1	chr1	.	CAGCATTCTCATATTTTGATAAAGATGGAAGTGGATACATCACAAAAGATGAGCTTCAAAAAGC
TP21498_Query	D	1	chr1	.	CAGCATTCTCATATTTCGATAAAGATGGAAGTGGATACATCACAAAAGATGAGCTTCAAAAAGC

Name	Filter	Nb hit (Mt4.0)	Mt Chr (Mt4.0)	Ms Chr (Li et al., 2014)	Sequence
TP2152_Hit	D	1	chr1	.	CAGCAAATATTTCTCAGGACCATATGGTGTGGCTCTCTAGTCTATTGATTTCTTGATCTTTTAA
TP2152_Query	D	1	chr1	.	CAGCAAATATTTCTCAGGACCATATGGTGTGGCTCTCTAGTCTATTAATTTCTTGATCTTTTAA
TP21520_Hit	D	1	chr1	.	CAGCATTCTCTTTACCTTGATGGCTCCCTTCTTCCTTCATCAATTTCAAAAGGGGCCCCACCCC
TP21520_Query	D	1	chr1	.	CAGCATTCTCTCTACCTTGATGGCTCCCTTCTTCCTTCATCAATTTCAAAAGGGGCCCCACCCC
TP21589_Hit	D	1	chr1	.	CAGCATTGAACTTCTACAACAGCACCAGTTAGTACCATCACCGCCACCATCAATTATCAATAAT
TP21589_Query	D	1	chr1	.	CAGCATTGAAATTCTACAACAGCACCAGTTAGTACCATCACCGCCACCATCAATTATCAATAAT
TP2160_Hit	D	1	chr1	.	CAGCAAATCAAGTTCCAAATGCCACAAAATACCCTAAAGAATGTTCTTCAAACGTCCCTTGGAA
TP2160_Query	D	1	chr1	.	CAGCAAATCAAGTTCCAAATGCCACAAAATACCCGAAAGAATGTTCTTCAAACGTCCCTTGGAA
TP2162_Hit	D	1	chr1	.	CAGCAAATCAAGTTCCTAATGCATCAAAACATCAGAAAAAATGTTCTTCAAACTCCCCTTCTTT
TP2162_Query	D	1	chr1	.	CAGCAAATCAAGTTCCTAATGCAACAAAACATCAGAAAAAATGTTCTTCAAACTCCCCTTCTTT
TP21643_Hit	D+G	1	chr1	.	CAGCATTGAGCCAAGCTGGTAGAAGAAGCATGTTGAAGCTGGCACAGCGTATGACCAACAACTT
TP21643_Query	D+G	1	chr1	.	CAGCATTGAGCCAAGCTGGTAGAAGAAGCATGTTGAAGCTGGCACAACGTATGACCAACAACTT
TP21689_Hit	D	1	chr1	.	CAGCATTGCAAGAACAACGGTACTGGCATGCTCATCGCGTGCGAGTATAATCCACCGGGTAATT
TP21689_Query	D	1	chr1	.	CAGCATTGCAAGAACAACGGTACTGGCATGCTCATCGCGTGCGAGTATAATCCACCGAGTAATT
TP21707_Hit	D	1	chr1	.	CAGCATTGCAGTCTGTTAGCTCAGAAATTATACTGGAAGATATTCCAGGATGTAATTTCTTCGC
TP21707_Query	D	1	chr1	.	CAGCATTGCAGTCTGTTAGCTCAGAAATTATACTAGAAGATATTCCAGGATGTAATTTCTTCGC
TP21779_Hit	D	1	chr1	.	CAGCATTGGACAGACTTATGAGCTTATTATCCAAAGCCATGTCCAAGCCATGATCTTGCTTTTG
TP21779_Query	D	1	chr1	.	CAGCATTGGACAGACTTATGAGCTTATTATCCAAAGCCATGTCCAAACCATGATCTTGCTTTTG
TP21857_Hit	D	1	chr1	.	CAGCATTGTAACCATATAGGATAAGTATGATTTTGTTTTCAATCATCAACTTTCTTGCGTAAAG
TP21857_Query	D	1	chr1	.	CAGCATTGTAACCATATAGGATAAGTATGATTTTGTTTTCAATCATCAACTTTCTTGAGTAAAG
TP21886_Hit	D	1	chr1	.	CAGCATTGTCCTAATCTTGGTTTTCTTGATTCATCATACTGCCCTAGCATATCCTTGGAGGTGT
TP21886_Query	D	1	chr1	.	CAGCATTGTCCTAATCTTGATTTTCTTGATTCATCATACTGCCCTAGCATATCCTTGGAGGTGT
TP21888_Hit	D	1	chr1	.	CAGCATTGTCCTCCCTGATATGTTCAGAATCAGGGCCACGCTGGATTCGTTGCTCTGAATATCC
TP21888_Query	D	1	chr1	.	CAGCATTGTCCTCCCTAATATGTTCAGAATCAGGGCCACGCTGGATTCGTTGCTCTGAATATCC
TP21926_Hit	D	1	chr1	.	CAGCATTGTTCACTAACATGTTAACCTTTTCTTGCAATGTCAACCTTCCAATGAGGTCATTCAC
TP21926_Query	D	1	chr1	.	CAGCATTGTTCACTAACATGCTAACCTTTTCTTGCAATGTCAACCTTCCAATGAGGTCATTCAC
TP22033_Hit	D	1	chr1	.	CAGCATTTATAGGCATATATGAAATGCTAACATAAAAATTGTTCAAGATTCACTTCATTAAAGA
TP22033_Query	D	1	chr1	.	CAGCATTTATAGGCATATATGAAAGGCTAACATAAAAATTGTTCAAGATTCACTTCATTAAAGA
TP22138_Hit	D	1	chr1	.	CAGCATTTCCTGCAAACCCTTCACCTGTCTCCATATCCATCCTTTGCCTACCCTTCTCCTCTGT
TP22138_Query	D	1	chr1	.	CAGCATTTCCTGCAAACCCTTCACCTGTCTCCATATCCATCCTTTGCATACCCTTCTCCTCTGT
TP22153_Hit	D	1	chr1	.	CAGCATTTCTAACGGGTGTGGTGGTTGCTAGCTTAACACTCTACACATTTTGGGCTGTTAAAAG
TP22153_Query	D	1	chr1	.	CAGCATTTCTAACAGGTGTGGTGGTTGCTAGCTTAACACTCTACACATTTTGGGCTGTTAAAAG
TP22158_Hit	D	1	chr1	.	CAGCATTTCTCAAGAAGAATAGTGTTGCTACAAAAGGACGTGTGTCCTCTGATGAGTCTGGCAT
TP22158_Query	D	1	chr1	.	CAGCATTTCTCAAGAAGAATAGTGTTGCTACAAAAGGACGTGTATCCTCTGATGAGTCTGGCAT
TP22206_Hit	D	1	chr1	.	CAGCATTTGAAGCATCATCTGCTTAGAGTCCGCGTCGAGAACTGTTGGATCAAGAACATCAGCT
TP22206_Query	D	1	chr1	.	CAGCATTTGAAGCATCATCTGCTTAGAGTCCGCGTCGAGAACTGTTGGATCAAGAACATCAGCA
TP22260_Hit	D	1	chr1	.	CAGCATTTGCAAGACAGTTCGAGGCTAATAACCCCGTGCTGGATATGATTGACGAAAAAATTCT
TP22260_Query	D	1	chr1	.	CAGCATTTGCAAGACAGTTCGAGGCTAATAACCCCGTGCTGGATATGATTGACAAAAAAATTCT
TP22290_Hit	D	1	chr1	.	CAGCATTTGCGGCCTTAAACTTAAATTCCAAGAACAGTTTAACAGACACTTGGAGTGGCATGCC
TP22290_Query	D	1	chr1	.	CAGCATTTGCGGCCTTAAACTTAAATTACAAGAACAGTTTAACAGACACTTGGAGTGGCATGCC
TP22297_Hit	D+G	1	chr1	.	CAGCATTTGCTCAAAATGGTTTAGGTATGAAAGTAATTGATACTTTTGAGGATATGTTGAAGAC
TP22297_Query	D+G	1	chr1	.	CAGCATTTGCTCAAAATGGTTTAGGTATGAAAGTAATTAATACTTTTGAGGATATGTTGAAGAC
TP22298_Hit	D+G	1	chr1	.	CAGCATTTGCTCATAGTCCTGTACACAAAGCCATAATATTGAAGGATTATGCTAATCTTAAGGA
TP22298_Query	D+G	1	chr1	.	CAGCATTTGCTCATAGCCCTGTACACAAAGCCATAATATTGAAGGATTATGCTAATCTTAAGGA
TP22409_Hit	D	1	chr1	.	CAGCATTTTACTTTGAGTTTTTGGTAGTATGTGAAAAGTGCTAATTTGTGATATTCATATCATC
TP22409_Query	D	1	chr1	.	CAGCATTTTACTTTGAGTTTTTGGCAGTATGTGAAAAGTGCTAATTTGTGATATTCATATCATC
TP22439_Hit	D+G	1	chr1	.	CAGCATTTTCAATCAATGCGAGGAGGGATAGGTGGTATTGGACCTGTGAAGATGGAACCACAGG
TP22439_Query	D+G	1	chr1	.	CAGCATTTTCAATCAATGCGAGGAGGGATAGGTGGTATCGGACCTGTGAAGATGGAACCACAGG
TP22442_Hit	D	1	chr1	.	CAGCATTTTCGCTCATATCATTATGCCCTACAATTCCATCTCTCTCATTTTTCATTGCCGCCGA
TP22442_Query	D	1	chr1	.	CAGCATTTTCACTCATATCATTATGCCCTACAATTCCATCTCTCTCATTTTTCATTGCCGCCGA
TP22544_Hit	D	1	chr1	.	CAGCATTTTTTCAGTCCTTTTATTCCTTAGTACCATACACTACACCAAGGATTGCATTTCATTA
TP22544_Query	D	1	chr1	.	CAGCATTTTTCCAGTCCTTTTATTCCTTAGTACCATACACTACACCAAGGATTGCATTTCATTA
TP22585_Hit	D	1	chr1	.	CAGCATTTTTTGCAAATCTTTAATACTCCTCCGTTTGAACCTGTCATGTCCAATGAAAAAAGGA
TP22585_Query	D	1	chr1	.	CAGCATTTTTTGCAAATCTTTAATACTCCTCCGTTTGAACCTATCATGTCCAATGAAAAAAGGA

Name	Filter	Nb hit (Mt4.0)	Mt Chr (Mt4.0)	Ms Chr (Li et al., 2014)	Sequence
TP22645_Hit	D	1	chr1	.	CAGCCAAAAAGAAGGTTTGTTGCTTTTTGTTGATGATCTCTCAAAACAGTATCCTATTTTAGTT
TP22645_Query	D	1	chr1	.	CAGCCAAAAAGAAGGTTTGTTGCTTTTTGTTGATGATCTCTCAAAACAGTATCCTATTTTAGTG
TP23012_Hit	D	1	chr1	.	CAGCCAAAGCTCGAGCTGATGGAGGAAAGAAGCATCGGACTCGTGACCGGGCTGTAAAAGCTGA
TP23012_Query	D	1	chr1	.	CAGCCAAAGCTCGAGCTGATGGAGGAAAGAAGCATCGGACACGTGACCGGGCTGTAAAAGCTGA
TP2324_Hit	D	1	chr1	.	CAGCAAATGCAACGGCAGGGCTCTGGTGGCACATCCTCAAATGGTGCGGAGATCGGAAGAGCGG
TP2324_Query	D	1	chr1	.	CAGCAAATGCAACGGCAGGGCTCTGGTGGCACATCCTCAAATGGTGCCGAGATCGGAAGAGCGG
TP23400_Hit	D	1	chr1	.	CAGCCGACCCAAGAGTCTGTACTATTCTGTTGAAGACTACATGGCTTGGTTTTTGCATCTTAT
TP23400_Query	D	1	chr1	.	CAGCCAACCCAAGAGTCTGTACTATTCTGTTGAAGACTACATGGCTTGGTTTTTGCATCTTAT
TP23417_Hit	D+G	1	chr1	.	CAGCCAACCTCAAACACAGTCTGGTTATGGACCCCCTCAAACTCAAAAGCCAAGTGGTGCTCCG
TP23417_Query	D+G	1	chr1	.	CAGCCAACCTCAAACACAGTCTGGTTATGGACCCCCTCAAACTCAAAAGCCAAGTGGTACTCCG
TP23466_Hit	D	1	chr1	.	CAGCCAACTAGAGATAGCATAGTCTATAAAAGCTGACAAACACACTCAAACTATATCAGATAAA
TP23466_Query	D	1	chr1	.	CAGCCAACTAGAGATAGCATAGTCTATAAAAGATGACAAACACACTCAAACTATATCAGATAAA
TP23597_Hit	D	1	chr1	.	CAGCCAAGAGAAGTAGGCATTCTAATGATACAAATTCAAATTTAAGGTCAGAAGGAATTATGAA
TP23597_Query	D	1	chr1	.	CAGCCAAGAGAAGTAGGCATTCTAATGATACAAATTCAAATTTAAGGTCAGAAGGAAGTATGAA
TP23650_Hit	D	1	chr1	.	CAGCCAAGCATTTGTTTGAAATGGAACCGGATAATGCTGGTAACCACGTCTTGCTAGCAAACAT
TP23650_Query	D	1	chr1	.	CAGCCAAGCATTTGTTTGAAATGGAACCGGATAATGCCGGTAACCACGTCTTGCTAGCAAACAT
TP23653_Hit	D	1	chr1	.	CAGCCAAGCCACCAGTGTCTGAGTTTGAGTCACCAAAGTTGAAGATTGCAGGGAAATTGCACTC
TP23653_Query	D	1	chr1	.	CAGCCAAGCCACCAGTGTCTGAGTTTGAGTCACCAAAGTTGAAGATTGCAGGGAAATAGCACTC
TP2370_Hit	D	1	chr1	.	CAGCAAATGGATTCAGAGAGAACATTAATATTTCTTAGAAGATGTTGTTATCTTACAGAATCTC
TP2370_Query	D	1	chr1	.	CAGCAAATGGATTAAGAGAGAACATTAATATTTCTTAGAAGATGTTGTTATCTTACAGAATCTC
TP23813_Hit	D	1	chr1	.	CAGCCAAGTTTTGCAGATTTGGAGGCTTTTCAGGCAAATTGATATTGATTCAAACCTCATTGGA
TP23813_Query	D	1	chr1	.	CAGCCAAGTTTTGCAGATTTGGAGGCTTTTCAGGCAAATTGATATTGATTCAAACCACATTGGA
TP2392_Hit	D	1	chr1	.	CAGCAAATGTAAGAATCCGTTGCCTAGACCGTCCCTCCTCATCAACACCTACAACGCTGTTTAT
TP2392_Query	D	1	chr1	.	CAGCAAATGTAAGAATCCGTTGCCTAGACCGCCCCTCCTCATCAACACCTACAACGCTGTTTAT
TP24004_Hit	D	1	chr1	.	CAGCCAATGATGCCAACTTTTCTGTCTAAAATGTGCATTTAAGTCTCCTCAATCGAACGGTTAC
TP24004_Query	D	1	chr1	.	CAGCCAATGATGCCAACTTTTCCGTCTAAAATGTGCATTTAAGTCTCCTCAATCGAACGGTTAC
TP24025_Hit	D	1	chr1	.	CAGCCAGTGCCCGAGCATTTGCTAACAGTCCACACAATCCTCCACATGCCCTAATAAATGGAGA
TP24025_Query	D	1	chr1	.	CAGCCAATGCCCGAGCATTTGCTAACAGTCCACACAATCCTCCACATGCCCTAATAAATGGAGA
TP24045_Hit	D	1	chr1	.	CAGCCAATGGGGAGAAGCATGAGATAAACTGTGCAATTGATCATATCATAGATGCGGAGATGAA
TP24045_Query	D	1	chr1	.	CAGCCAATGGGGAGAAGCATGAGATAAACTGTGCAATTGATCATATCATAGATGCCGAGATGAA
TP24047_Hit	D	1	chr1	.	CAGCCAATGGGGAGAAGCATGAGATAAACTGTGCAATTGATCATATCATAGATGCTGTGATGAA
TP24047_Query	D	1	chr1	.	CAGCCAATGGGGAGAAGCATGAGATAAACTGTGCAATTGATCATATCATAGATGCTGGGATGAA
TP24065_Hit	D	1	chr1	.	CAGCCAATGTTATCTTTTGATCTAATTCCTATTGCTTGGATAGTTCCTTCCATGTTTTTGCTTG
TP24065_Query	D	1	chr1	.	CAGCCAATGTGATCTTTTGATCTAATTCCTATTGCTTGGATAGTTCCTTCCATGTTTTTGCTTG
TP24114_Hit	D	1	chr1	.	CAGCCAATTGCCCTAAGAACGATAACCATGAGATTGGAAGGCTGTTTATTATACCATGCAAGAC
TP24114_Query	D	1	chr1	.	CAGCCAATTGCCCTAAGAACGATAACCAGGAGATTGGAAGGCTGTTTATTATACCATGCAAGAC
TP24186_Hit	D	1	chr1	.	CAGCCACAAAAGAACAATTGAATAAAATTGTTTTTATTAACAGCACTGCTATTCATCGCGACAC
TP24186_Query	D	1	chr1	.	CAGCCACAAAAGAACAATTGAATAAAATTGTTTTTATTAACAGCACTGCTATTCATCGCAACAC
TP2421_Hit	D	1	chr1	.	CAGCAAATGTTTCATTCTCACCTGTGGTCAATTCATGTTCAGGATTGAGTTTTGGTTCTGAAAA
TP2421_Query	D	1	chr1	.	CAGCAAATGTTTCATTCTCACCAGTGGTCAATTCATGTTCAGGATTGAGTTTTGGTTCTGAAAA
TP24460_Hit	D	1	chr1	.	CAGCCACAGAGCGACGCAACGCGGTGGCAAAGTCGGCTCCATTGTAACTGGACTGGAATTGGTT
TP24460_Query	D	1	chr1	.	CAGCCACAGAGCGACGCAACGCGGTGGCAAAATCGGCTCCATTGTAACTGGACTGGAATTGGTT
TP24668_Hit	D	1	chr1	.	CAGCCACCGAAACTTAATATTAACGGATGCGAAGAAAATGGTGTGTCGTTGAAATTCAGTAGTA
TP24668_Query	D	1	chr1	.	CAGCCACCAAAACTTAATATTAACGGATGCGAAGAAAATGGTGTGTCGTTGAAATTCAGTAGTA
TP24777_Hit	D	1	chr1	.	CAGCCACCATACCTGAGATTTGAAAACTTAATCAATAATTAATGCCATAAAGACAGTTGATCTT
TP24777_Query	D	1	chr1	.	CAGCCACCATACCTGAGATTTGAAAACTTAATCAATAATTAATGCAATAAAGACAGTTGATCTT
TP24790_Hit	D	1	chr1	.	CAGCCACCATCAGAGGTTTTGGACAAGAAAAAAGGTTCTTGAAGAGGAATCTCTATCTTCTGGA
TP24790_Query	D	1	chr1	.	CAGCCACCATCAGAGGTTTTGGACAAGAAAAAAGGTTCTTGAAGAGGAACCTCTATCTTCTGGA
TP24974_Hit	D+G	1	chr1	.	CAGCCACCTTAAAAAGGGGGTTTGACCTGATTTATTCCCTGTCATTATGTAAGGAAGCTAGAGT
TP24974_Query	D+G	1	chr1	.	CAGCCACCTTAAAAAGGGGGTTTGACCTAATTTATTCCCTGTCATTATGTAAGGAAGCTAGAGT
TP25207_Hit	D	1	chr1	.	CAGCCACTAGGCACTACAAGATCTGCTGTTGCATCGCAGTTTCAGGTAGCGGCTGTAGCTGACA
TP25207_Query	D	1	chr1	.	CAGCCACTAGGCACTACAAGATCTGCTGTTGCATCGCAGTTTCAGGTAGCGGCTGTAGCTGAAA
TP25230_Hit	D	1	chr1	.	CAGCCACTATTCCAAAATGTTGCATGCTTTCTTCTCTTGACTCTTGTTTTTACCTTTGAAGACG
TP25230_Query	D	1	chr1	.	CAGCCACTATTCCAAAATGTTGCACGCTTTCTTCTCTTGACTCTTGTTTTTACCTTTGAAGACG

Name	Filter	Nb hit (Mt4.0)	Mt Chr (Mt4.0)	Ms Chr (Li et al., 2014)	Sequence
TP25243_Hit	D	1	chr1	.	CAGCCACTCAGGATTAGTAGAAGAGGGTCTGCAGATTTTTGGGATAATGATGAAGGTATATGCA
TP25243_Query	D	1	chr1	.	CAGCCACTCAGGATTAGTAGAAGAGGGTCTGCAGATTTTTGGGATAATGATGAAGGAATATGCA
TP25386_Hit	D	1	chr1	.	CAGCCACTTAGACTCACCACAGCTCAGGAAATTGTCAAATATGACTTGAGCACCATTTGCAAAA
TP25386_Query	D	1	chr1	.	CAGCCACTTAGACTCACCACAGCTCAGGAAATTGTCAAATATGACTTGAGCACCATTCGCAAAA
TP2540_Hit	D	1	chr1	.	CAGCAAATTGGTGACCGGAGGTTACTATCACTCGGCCTAGAAGGCACAGCTGAAAAAAAAAAAA
TP2540_Query	D	1	chr1	.	CAGCAAATTGGTGACCGGAGGTTACTATCACTCGGCCTAGAAGGCACAGCAGAAAAAAAAAAAA
TP25423_Hit	D+G	1	chr1	.	CAGCCACTTGAGGATTCACATGCCTCGGAATTTCTAGCCTTTTGCCCATAAAACCAACAGCTGA
TP25423_Query	D+G	1	chr1	.	CAGCCACTTGAGGATTCACATGCCTCGGAATTTCTAGCCTTTTACCCATAAAACCAACAGCTGA
TP25598_Hit	D+G	1	chr1	.	CAGCCAGAGAAGAGGCCAACTATGGTAGATGTTGTTAAAATGATTGAGGATATTAGAGTGGAGC
TP25598_Query	D+G	1	chr1	.	CAGCCAGAGAAGAGGCCAACTATGGTAGATGTTGTTAAAATGATTGAGGATATTAGAGTCGAGC
TP25629_Hit	D	1	chr1	.	CAGCCAGAGCTTGAAATGTAGATTCTCTCCAGCATCTCTCCCGAAGTACAACAGGATTTGTGCT
TP25629_Query	D	1	chr1	.	CAGCCAGAGCTTGAAATGTAGATTCTCTCCAGCAACTCTCCCGAAGTACAACAGGATTTGTGCT
TP25779_Hit	D	1	chr1	.	CAGCCAGCATACTATAACCTTGGTGTGGTCTATTCTGAAATGATGCAATATGACATGGCCCTGA
TP25779_Query	D	1	chr1	.	CAGCCAGCATACTATAACCTTGGCGTGGTCTATTCTGAAATGATGCAATATGACATGGCCCTGA
TP2581_Hit	D	1	chr1	.	CAGCAAATTTCTTCAAGAATAATTCTTTCTCTTCTGATGTCCAAGGATTGATCGTACCCCTTTC
TP2581_Query	D	1	chr1	.	CAGCAAATTTCTTCAAGAATAATTCTTTCTCTTCTGATGTCCAAGGATTGATCATACCCCTTTC
TP2595_Hit	D	1	chr1	.	CAGCAAATTTGTAAAGATAAAATAACCAAAAGATGCACTTCATTGTAGTAGAGTATGCATGTGT
TP2595_Query	D	1	chr1	.	CAGCAAATTTGTAAAGATAAAATAACCAAAAGATGCACTCCATTGTAGTAGAGTATGCATGTGT
TP25988_Hit	D+G	1	chr1	.	CAGCCAGGGTCTACCTCGGATCCCCTCAGTTCTGAGGGTAATGCAGAAGATAATGCGTGGGGCA
TP25988_Query	D+G	1	chr1	.	CAGCCAGGGTCTACCTCGGATCCCCTCAGTTCTGAGGGTAATGCAGAAGATAATGCATGGGGCA
TP25997_Hit	D	1	chr1	.	CAGCCAGGTACAGTCTTTAAGAAAGACATCCTAATCTTGATCTTAACTTGTAGGCCGGTGATTG
TP25997_Query	D	1	chr1	.	CAGCCAGGTACAGTCTTAAAGAAAGACATCCTAATCTTGATCTTAACTTGTAGGCCGGTGATTG
TP26210_Hit	D	1	chr1	.	CAGCCATAAAAGAGGGACGCAACCAACTAGTGCTATATAGCCTTACCAATGGAGGAAATAGCTT
TP26210_Query	D	1	chr1	.	CAGCCATAAAAGAGGGACGCAACCAACTAGTGCTATATAGCCTTACCAATGGAGGAAACAGCTT
TP26230_Hit	D	1	chr1	.	CAGCCATAAACTCATCACTGCTACTTGAGCCACTCCCTGGCTGTGGACCAGTGTGTGAAGAAAC
TP26230_Query	D	1	chr1	.	CAGCCATAAACTCATCACTGCTACTTGAGCCACTCCCTGGCTGTGGACCAGTATGTGAAGAAAC
TP26292_Hit	D	1	chr1	.	CAGCCATAATGGTGTAAATGACCTTCAAAATGAACCTCCTTGGAGAATCCTCTGAACTAACTGC
TP26292_Query	D	1	chr1	.	CAGCCATAATGGTGTAAATGACCTTCAAAATGAACCTCCTTGGAGAATCATCTGAACTAACTGC
TP26377_Hit	D+G	1	chr1	.	CAGCCATAGAGTTTCTGAAGGGTGTCTTTAGATTGGTAGATACGGATAAGGTATGTAATAATTT
TP26377_Query	D+G	1	chr1	.	CAGCCATAGAGTTTCTGAAGGGTGTCTTTAGATTGGTAGATACAGATAAGGTATGTAATAATTT
TP26415_Hit	D	1	chr1	.	CAGCCATAGTATGAGTAGTTGATGGCATTGGCCTCAAAATAGTTTGTGATGACGTGGAACCTCC
TP26415_Query	D	1	chr1	.	CAGCCATAGTATGAGTAGTTGATGGCATTGGCCACAAAATAGTTTGTGATGACGTGGAACCTCC
TP26512_Hit	D	1	chr1	.	CAGCCATCAAAGCAGTCAGAAGTTGAAGTACCACCAAATTTTAATGAGTTGGGGGAAGCAAACA
TP26512_Query	D	1	chr1	.	CAGCCATCAAAGCAGTCAGAAGTTGAAGTACCACCAAATTTTAATGAGTTGGGGGAAACAAACA
TP2673_Hit	D	1	chr1	.	CAGCAACAAAACTCCAATCCTTCCCCAGAGGAACTAAGTTCATCTGAAATAAGTGAAATATGTG
TP2673_Query	D	1	chr1	.	CAGCAACAAAACTCCAATCCTGCCCCAGAGGAACTAAGTTCATCTGAAATAAGTGAAATATGTG
TP26747_Hit	D	1	chr1	.	CAGCCATCTGTCCCAATAGGTTGTTCCTAAAGCATGTTGAAAGCCAAAAAGTAAGCCAATGGAC
TP26747_Query	D	1	chr1	.	CAGCCATCTGTCCCAATAGGTTGTTCCTAAAGCATGTTGAAAGCCAAAAAGAAAGCCAATGGAC
TP26799_Hit	D	1	chr1	.	CTGCCATGAACATGCTTGCAAGATTGCTGGGGTTAAATGGCATTGAGCCAAATGAAGCCGGTGC
TP26799_Query	D	1	chr1	.	CAGCCATGAACATGCTTGCAAGATTGCTGGGGTTAAATGGCATTGAGCCAAATGAAGCCGGTGC
TP26827_Hit	D	1	chr1	.	CAGCCGTGACAATTGCTCCCAGAATAAAGATAAAATCTGCAATGATGGTTGCCTTCTTTCTTCC
TP26827_Query	D	1	chr1	.	CAGCCATGACAATTGCTCCCAGAATAAAGATAAAATCTGCAATGATGGTTGCCTTCTTTCTTCC
TP26830_Hit	D	1	chr1	.	CAGCCATGACGTACTCTGCATAGATGAAGTCCCAACTGAAAAAACAAAGATATGCAATGAGGGT
TP26830_Query	D	1	chr1	.	CAGCCATGACATACTCTGCATAGATGAAGTCCCAACTGAAAAAACAAAGATATGCAATGAGGGT
TP27148_Hit	D	1	chr1	.	CAGCCATGTCTCGAACACTCTCAAAAAAATCATCCTCGTCTGTTGATGAAGCTTTTCAACAATG
TP27148_Query	D	1	chr1	.	CAGCCATGTCTCGAACACTCTCAAAAAAATCATCCTCGTCTGTTGATGAAGATTTTCAACAATG
TP27170_Hit	D	1	chr1	.	CAGCCATGTGTGTATAGAAGATAACAAACTATGTAGACCTTGGTTTGTCCTAGTCCATTGCCTT
TP27170_Query	D	1	chr1	.	CAGCCATGTGTGTATAGAAGATAACAAACCATGTAGACCTTGGTTTGTCCTAGTCCATTGCCTT
TP27202_Hit	D	1	chr1	.	CAGCCATTAAACCTCTTTGTATGAAATAGTTTTAGTCGAGATTTTAGTTAAATACAAATCAAAT
TP27202_Query	D	1	chr1	.	CAGCCATTAAACCTCTTTGTATGAAATAGTTTTAGTAGAGATTTTAGTTAAATACAAATCAAAT
TP27282_Hit	D	1	chr1	.	CAGCCATTATCATTAGTGTGGACTATATGTATGGTCCACACTAATTTACTCTGTGCAGTTTCGA
TP27282_Query	D	1	chr1	.	CAGCCATTATCATTAGTGTGGACTATATGTATGGTCCACACTAATTTACCCTGTGCAGTTTCGA
TP27629_Hit	D	1	chr1	.	CAGCCCAAAAAGAAGGTTCGTTCAGGGATTCCTGCTTCGCAATGGATTTCAGTGTACAATGCTA
TP27629_Query	D	1	chr1	.	CAGCCCAAAAAGAAGGTTCGTTCAGGGATTCCTGCTTCACAATGGATTTCAGTGTACAATGCTA

Name	Filter	Nb hit (Mt4.0)	Mt Chr (Mt4.0)	Ms Chr (Li et al., 2014)	Sequence
TP27762_Hit	D	1	chr1	.	CAGCCCAACTATGTGGCATCCCTTGCCGATCAATTGAAAGGCCTCAGCCAGATTTAAGTGCAAA
TP27762_Query	D	1	chr1	.	CAGCCCAACTATGCGGCATCCCTTGCCGATCAATTGAAAGGCCTCAGCCAGATTTAAGTGCAAA
TP27824_Hit	D	1	chr1	.	CAGCCCAAGTGATTAGGAAAGATGCAAACCAGAATTTCTTATCCTTCATCACTTTCTTCAATTC
TP27824_Query	D	1	chr1	.	CAGCCCAAGTGATAAGGAAAGATGCAAACCAGAATTTCTTATCCTTCATCACTTTCTTCAATTC
TP27831_Hit	D+G	1	chr1	.	CAGCCCAAGTTTCCCAGTTTCAGTCAACGTCTCCCTCATCACATGAGGCCCTCCAAATGTAGAT
TP27831_Query	D+G	1	chr1	.	CAGCCCAAGTTTCCCAGTTTCAGTCAAAGTCTCCCTCATCACATGAGGCCCTCCAAATGTAGAT
TP27988_Hit	D	1	chr1	.	CAGCTCACGCTGTTCTTTATTTCTTCCCTTCCGCTCTCACCTACCTCGCTCTCATTCCCGCCAG
TP27988_Query	D	1	chr1	.	CAGCCCACGCTGTTCTTTATTTCTTCCCTTCCGCTCTCACCTACCTCGCTCTCATTCCCGCCAG
TP28003_Hit	D+G	1	chr1	.	CAGCCCACTCGTCAACCAACCCAACCACGTCAATGGAGACCTAAAAAATTAGGTAAAAAGCAGT
TP28003_Query	D+G	1	chr1	.	CAGCCCACTCGTCAACCAACCCAACCACGTCAATGGAGACCTAAAAAATTAGGTAAAAAACAGT
TP28456_Hit	D+G	1	chr1	.	CAGCCCCACCATCCTTCATTCTTTTGTAATTAAGATCTTTGTGATTTGCCAATTTATCCATCTA
TP28456_Query	D+G	1	chr1	.	CAGCCCCACCATCCTTCACTCTTTTGTAATTAAGATCTTTGTGATTTGCCAATTTATCCATCTA
TP28725_Hit	D	1	chr1	.	CAGCTCCGTCGTGAAAACCAACCAAACACCGTTACATTCCACCGTTGTTCAGAATCAAAACCCC
TP28725_Query	D	1	chr1	.	CAGCCCCGTCGTGAAAACCAACCAAACACCGTTACATTCCACCGTTGTTCAGAATCAAAACCCC
TP28805_Hit	D	1	chr1	.	CAGCCCCTGATTACGCAACCAATCTTGCATAGTTGCATGCTCTTTTTTCCTCGGTTACTCACGA
TP28805_Query	D	1	chr1	.	CAGCCCCTGATTACGCAACCAATCTTGCATAGTTGCATGCTCTTTTTTCCTCAGTTACTCACGA
TP28834_Hit	D	1	chr1	.	CAGCCCCTTCCTCTTTATCCCTAACGGAATCTTCCCGGACGAATGAACCACCGCGGCTGAAAAA
TP28834_Query	D	1	chr1	.	CAGCCCCTTCCTCTTTATCCCTAACGGAATCTTCCCGGACGAATGAACCACCGCGGCAGAAAAA
TP2905_Hit	D+G	1	chr1	.	CAGCAACAACAGAAACCCTAGAAGCAGAAGAAGAAAAGATGCCTGCAGGTCATGGTTTACGATC
TP2905_Query	D+G	1	chr1	.	CAGCAACAACAGAAACCCTAGAAGCAGAAGAAGAAAACATGCCTGCAGGTCATGGTTTACGATC
TP29154_Hit	D	1	chr1	.	CAGCCCGGTGCCCGCATATGCAGGGTCCGGGAAGGGATCCCACCAATTGATGTATTGTACGCTG
TP29154_Query	D	1	chr1	.	CAGCCCGGTGCCCGCATATGCAGGGTCCGGGAAGGGATCCCACCAATTGATGTATTGTACGCAG
TP2919_Hit	D+G	1	chr1	.	CAGCAACAACAGCATATGGTGGCTCCCAACTCAAATGGTGATCAAAACTCTGTGCAAGCTGAAA
TP2919_Query	D+G	1	chr1	.	CAGCAACAACAGCATATGGTGGCCCCCAACTCAAATGGTGATCAAAACTCTGTGCAAGCTGAAA
TP29320_Hit	D+G	1	chr1	.	CAGCTCTAACAAGCTCAGCAAAATCTTCATTACCCTCTAATGATGCTGATGATCTTCTTGATGA
TP29320_Query	D+G	1	chr1	.	CAGCCCTAACAAGCTCAGCAAAATCTTCATTACCCTCTAATGATGCTGATGATCTTCTTGATGA
TP29373_Hit	D	1	chr1	.	CAGCCCTACGGATTTGATGTAGGTGAATCTGCGAGAGGAAATGTCTTAAAAGGTTTGGATGTAG
TP29373_Query	D	1	chr1	.	CAGCCCTACGGATTTGATGTAGGCGAATCTGCGAGAGGAAATGTCTTAAAAGGTTTGGATGTAG
TP29761_Hit	D+G	1	chr1	.	CAGCCCTTGCACGTGCCATTGATCTTGCAGAATCTTCATTTGTTGACAAATGAATTTGGGTTTA
TP29761_Query	D+G	1	chr1	.	CAGCCCTTGCACGTGCCATTGATCTTGCAGAATCTTCATTTGTAGACAAATGAATTTGGGTTTA
TP29853_Hit	D+G	1	chr1	.	CAGCCGAAAAATTATTTGAGCTTGATGATGAAGACTTTGGCAACCATAGTGCTTTCTGATAGGC
TP29853_Query	D+G	1	chr1	.	CAGCCGAAAAATTATTTGAGCTTGATGATGAAGACTTTGGCAACCATAGTGCTTTCTAATAGGC
TP29992_Hit	D	1	chr1	.	CAGCCGAGTGTGTTGGTTGTGCAGAAGAAAGATTTTACTTGGTTGGGGATACCGTTTACGGAAG
TP29992_Query	D	1	chr1	.	CAGCCGAATGTGTTGGTTGTGCAGAAGAAAGATTTTACTTGGTTGGGGATACCGTTTACGGAAG
TP3001_Hit	D+G	1	chr1	.	CAGCAACAAGGTTGTTTCAGTGTTACGTACCCCATACAAAAACGACGGTTCAGTGTTGTTGAGT
TP3001_Query	D+G	1	chr1	.	CAGCAACAACGTTGTTTCAGTGTTACGTACCCCATACAAAAACGACGGTTCAGTGTTGTTGAGT
TP30104_Hit	D	1	chr1	.	CAGCGGAGAGTTTACGTCCAGATATTCAACATCTGCTCTCACACTTGAATACTGATGTGAATTC
TP30104_Query	D	1	chr1	.	CAGCCGAGAGTTTACGTCCAGATATTCAACATCTGCTCTCACACTTGAATACTGATGTGAATTC
TP30106_Hit	D	1	chr1	.	CAGCGGAGATACGTGATCCATCAAGGAAAGGAAGTAGGGTTTGGTTAGGTACATTTGACTGTGA
TP30106_Query	D	1	chr1	.	CAGCCGAGATACGTGATCCATCAAGGAAAGGAAGTAGGGTTTGGTTAGGTACATTTGACTGTGA
TP30138_Hit	D	1	chr1	.	CAGCCGAGCTATTGCAGATGAAGATTGACAGTCATTCATTCCTTCTAGTCAATTCAGAGAACTG
TP30138_Query	D	1	chr1	.	CAGCCGAGCTATTGCAGATGAAGATTGACAGCCATTCATTCCTTCTAGTCAATTCAGAGAACTG
TP30181_Hit	D	1	chr1	.	CAGCTGAGTTTCTACAAATGAGTGAAGATTATGGAGAAGGAAACCTAATCATACAAACTGAAAA
TP30181_Query	D	1	chr1	.	CAGCCGAGTTTCTACAAATGAGTGAAGATTATGGAGAAGGAAACCTAATCATACAAACTGAAAA
TP30240_Hit	D	1	chr1	.	CAGCTGATGGGTATAAACAGATAGATCCTTTACGTTGTTACAGTAAGCTAGTTTCTTGAAGGCC
TP30240_Query	D	1	chr1	.	CAGCCGATGGGTATAAACAGATAGATCCTTTACGTTGTTACAGTAAGCTAGTTTCTTGAAGGCC
TP30447_Hit	D	1	chr1	.	CAGCCGCATAAAGCGGCCAATAAGTTTCGGTTAACCCGGCATGATTCCCGGTAAGACCAGGAGT
TP30447_Query	D	1	chr1	.	CAGCCGCATAAAGCGGCCAATAAGTTTCGGTTAACCCGGCATGATTCCCAGTAAGACCAGGAGT
TP30474_Hit	D	1	chr1	.	CAGCCGCATCTCCCTCCTCCGGGCGGCATGCTCAAAAGACCCCGTACTGAATATGGTTAGCTGA
TP30474_Query	D	1	chr1	.	CAGCCGCATCTCCCTCCTCCGGGCGGCATGCTCAAAAGACCCCGTACTGAATATGGTTAGCAGA
TP30636_Hit	D	1	chr1	.	CAGCCGCGAATTCGCTACGAATCCAATTCATAAACCCGTCTCTCTCATCAACAAACCATTGTTG
TP30636_Query	D	1	chr1	.	CAGCCGCGAATTCGCTACGAATCCAATTCATAAACCCATCTCTCTCATCAACAAACCATTGTTG
TP30644_Hit	D+G	1	chr1	.	CAGCCGCGACTCTCTTTAGTTTCTCGGTTCTAGACATGAACAAAGTTGCAATAGGAGCACCAGG
TP30644_Query	D+G	1	chr1	.	CAGCCGCGACTCTCTTTAGTTTCTCGGTTCTAGACAAGAACAAAGTTGCAATAGGAGCACCAGG

Name	Filter	Nb hit (Mt4.0)	Mt Chr (Mt4.0)	Ms Chr (Li et al., 2014)	Sequence
TP3076_Hit	D	1	chr1	.	CAGCAACAAGGACTTGGGAGTCACTAAGAAAAGTCATGCTTGAAATGTGCATTTGTTGCACCCA
TP3076_Query	D	1	chr1	.	CAGCAACAAGGACTTGGGAGTCACTAAGAAAAGTCATGCTTGAAATGTGCATTTGTTACACCCA
TP30826_Hit	D	1	chr1	.	CAGCCGCTGTGGAAAACTTGAAGCTCGCCCCATTTGCTATATCATCCTCGTTGGTGGAATTAGA
TP30826_Query	D	1	chr1	.	CAGCCGCTGTGGAAAACTTGAAGCTCGCCCCATTTGCTATATCATCCTCGTTAGTGGAATTAGA
TP30875_Hit	D	1	chr1	.	CAGCCGGAACGAAATCAACAGGATCAGGGAAGAGTACTATGGGCAGTGGTGCAAACTTAGCCAT
TP30875_Query	D	1	chr1	.	CAGCCGGAACGAAATCAACAGGATCAGGGAAGAGCACTATGGGCAGTGGTGCAAACTTAGCCAT
TP30929_Hit	D+G	1	chr1	.	CAGCCGGAGGTCAGATGGCGTCAACTATGTCTACTCAATGGCATCTTAGGCCTGAAATCAGTCC
TP30929_Query	D+G	1	chr1	.	CAGCCGGAGGTCAGATGGCGTCAACTATGTCTACTCAATGGCATCGTAGGCCTGAAATCAGTCC
TP31029_Hit	D	1	chr1	.	CAGCCGGCTAAGCATCTCAACCTCAACCGAAAGCTCTTTGTCACCCTGCTGAAAAAAAAAAAAA
TP31029_Query	D	1	chr1	.	CAGCCGGCTAAGCATCTCAACCTCAACCAAAAGCTCTTTGTCACCCTGCTGAAAAAAAAAAAAA
TP31613_Hit	D	1	chr1	.	CAGCCGTTTTCAGGTGGGATTCGGCTTTCCTATCTTTAGGAATTGATGAGTCGGTTTCCTTTCC
TP31613_Query	D	1	chr1	.	CAGCCGTTTTCAGGTGGAATTCGGCTTTCCTATCTTTAGGAATTGATGAGTCGGTTTCCTTTCC
TP31888_Hit	D	1	chr1	.	CAGCCTACCGATTATTTGGATAACATGGAGAAAAGAAAAAGTATGTTGACTTTTTCTTTCTTTC
TP31888_Query	D	1	chr1	.	CAGCCTACCGATTATTTGGATAACATGGAGAAAAGAAAAAGTATGTTGAATTTTTCTTTCTTTC
TP31890_Hit	D+G	1	chr1	.	CAGCCTACCTATTTGCAACGTTGAAAGTTTTGAATTGAAGTCCTCCGTAACAGCTGGTGCTGAA
TP31890_Query	D+G	1	chr1	.	CAGCCTACCTATTTGCAACGATGAAAGTTTTGAATTGAAGTCCTCCGTAACAGCTGGTGCTGAA
TP31946_Hit	D	1	chr1	.	CAGCCTAGCAACGGCATCAAGGCCAACTATAGACCTGACAGAAGTGAAGAAAGGCTGAAAAAAA
TP31946_Query	D	1	chr1	.	CAGCCTAGCAACGGCATCAAGGCCAACTATAGACCTGACAGAAGTGAAGAAAGGCAGAAAAAAA
TP31975_Hit	D+G	1	chr1	.	CAGCCTAGGATCTGGCACTGCTAGTACAGAATCAGCACAGCAGAATGGCACTGCAAAACCTCGA
TP31975_Query	D+G	1	chr1	.	CAGCCTAGGATCTGGCACTGCTAGTACAGAATCAGCACAGCAGAATGGCACCGCAAAACCTCGA
TP32022_Hit	D	1	chr1	.	CAGCCTATAAATAAAGAGAATTTATTAGAGTAAATTGAGTGATTTAGGGCCTTTTGGCATTGGT
TP32022_Query	D	1	chr1	.	CAGCCTATAAATAAAGAGAAATTATTAGAGTAAATTGAGTGATTTAGGGCCTTTTGGCATTGGT
TP32040_Hit	D	1	chr1	.	CAGCCTATACTATACTATTGAACTGTTTCAAGCTTAGCCAGCTATCACAATGCAATCCTGCTAC
TP32040_Query	D	1	chr1	.	CAGCCTATACTATACTATTGAACTGTCTCAAGCTTAGCCAGCTATCACAATGCAATCCTGCTAC
TP32179_Hit	D	1	chr1	.	CAGCCTATTACAATTATGGTAAGAACACATCTCTTCATAGAACAATTGTTTTGAGCTTAATATT
TP32179_Query	D	1	chr1	.	CAGCCTATTAAAATTATGGTAAGAACACATCTCTTCATAGAACAATTGTTTTGAGCTTAATATT
TP32244_Hit	D	1	chr1	.	CAGCTTCAAAGCTTCATCCATCTTTCCTCGTCTGCAGTAACCATCCATCATTGTGTTAAATATC
TP32244_Query	D	1	chr1	.	CAGCCTCAAAGCTTCATCCATCTTTCCTCGTCTGCAGTAACCATCCATCATTGTGTTAAATATC
TP32260_Hit	D+G	1	chr1	.	CAGCCTCAACAACAACAACCCCACCACCTCCAAACGCCGCAACCTCATTCTCCAAACTTCTCTA
TP32260_Query	D+G	1	chr1	.	CAGCCTCAACAACAACAACACCACCACCTCCAAACGCCGCAACCTCATTCTCCAAACTTCTCTA
TP32291_Hit	D	1	chr1	.	CAGCCTCAACTATACCCGAATGTCCATAGTGGGGGAATGATGAAGCCACATTTTTGTGTATATC
TP32291_Query	D	1	chr1	.	CAGCCTCAACTATACCCGAATGCCCATAGTGGGGGAATGATGAAGCCACATTTTTGTGTATATC
TP32422_Hit	D	1	chr1	.	CAGCCTCAGATTTTCTTCACAATTATTGTTCAAACCTCGCAAATGACGTTAACTCCATTGTCGT
TP32422_Query	D	1	chr1	.	CAGCCTCAGATTTCCTTCACAATTATTGTTCAAACCTCGCAAATGACGTTAACTCCATTGTCGT
TP32430_Hit	D	1	chr1	.	CAGCCTCAGCAGAAGGCCACAAAGGAAGCCTGACATTTGGATCATAGGACAACAAAGCACCACC
TP32430_Query	D	1	chr1	.	CAGCCTCAGCAGAAGGCCACAAAGGAAGCCTAACATTTGGATCATAGGACAACAAAGCACCACC
TP32470_Hit	D	1	chr1	.	CAGCCTCAGTCCCACCGGCTATCATCAAATCAGCCTCACCTCTACGAATGTGATTGGCTGAAAA
TP32470_Query	D	1	chr1	.	CAGCCTCAGTCCCACCGGCTATCATCAAATCAGCCTCACCTCTACGAATGTGATTGGCAGAAAA
TP32628_Hit	D+G	1	chr1	.	CAGCCTCCACCACCACCTCCACCTCCAGAGAAAACCCATTTTGGTGGACTTAAGGATGAGGACA
TP32628_Query	D+G	1	chr1	.	CAGCCTCCACCACCACCTCCACCCCCAGAGAAAACCCATTTTGGTGGACTTAAGGATGAGGACA
TP32708_Hit	D	1	chr1	.	CAGCCTCCCTAGCCGCTTCATCCCTTTCTCTTTCCCATCTCGCACCACCTTCCTCCCTTGCAAG
TP32708_Query	D	1	chr1	.	CAGCCTCCCTAGCCGCTTCATCCCTTTCTCTTTCCCACCTCGCACCACCTTCCTCCCTTGCAAG
TP32728_Hit	D+G	1	chr1	.	CAGCCTCCGAGATTATGATGACGGCCTTATAGATATCACAAGACAAAGTACCGATATCACTATA
TP32728_Query	D+G	1	chr1	.	CAGCCTCCGAGATTATGATGACGGCCTTATAGATATCACAAGACAAAGCACCGATATCACTATA
TP3279_Hit	D	1	chr1	.	CAGCAACACCAAAATTCTTATGCAACACCTCCTCACACAATTCTGAATACCCTCGCCCTCTGCC
TP3279_Query	D	1	chr1	.	CAGCAACACCAAAATTCTTATGCAACACCTCCTCACACAATTCCGAATACCCTCGCCCTCTGCC
TP32934_Hit	D+G	1	chr1	.	CAGCCTCGTCGGTTAAGAAGAGGGTATACAAAAGACATTTTTATGAAGATTACCTATGATGTCA
TP32934_Query	D+G	1	chr1	.	CAGCCTCGTCGGTTAAGAAGAGGGTATACAAAAGACATTTTTATGAAAATTACCTATGATGTCA
TP33107_Hit	D	1	chr1	.	CAGCCTCTGCTGATGCAGTGGTGTTGGTTGTTGGATTGGACCAATCTATAGAAGCAGAAGGGCT
TP33107_Query	D	1	chr1	.	CAGCCTCTGCTGATACAGTGGTGTTGGTTGTTGGATTGGACCAATCTATAGAAGCAGAAGGGCT
TP33133_Hit	D	1	chr1	.	CAGCCTCTGTCTTTTTTTTTCATAATTAAGCTTTTAAATTTTAATTTAGTTGTTCCAACCTATG
TP33133_Query	D	1	chr1	.	CAGCCTCTGTCTTTTGTTTTCATAATTAAGCTTTTAAATTTTAATTTAGTTGTTCCAACCTATG
TP33142_Hit	D+G	1	chr1	.	CAGCCTCTGTTTTGCTGTCGTCTATTTCTTTCTTTCACCTTTCATATGCTTGTTATATATGACC
TP33142_Query	D+G	1	chr1	.	CAGCCTCTGTTTTGCTCTCGTCTATTTCTTTCTTTCACCTTTCATATGCTTGTTATATATGACC

Name	Filter	Nb hit (Mt4.0)	Mt Chr (Mt4.0)	Ms Chr (Li et al., 2014)	Sequence
TP33164_Hit	D	1	chr1	.	CAGCCTCTTCCAGTGAGATTCGTGATGCTTTGAATGATAAGGCCCTTCAAGAACTAATTTGCCA
TP33164_Query	D	1	chr1	.	CAGCCTCTTCCAGCGAGATTCGTGATGCTTTGAATGATAAGGCCCTTCAAGAACTAATTTGCCA
TP33396_Hit	D	1	chr1	.	CAGCCTGATAAGACCTTTTCCTGGCCCAAAATTGCGGTCCAATATGCGCACGGAGCGCATGATG
TP33396_Query	D	1	chr1	.	CAGCCTGATAAGACCTTTTCCTGGCCCAAAATTGCGGTCCAATATGCACACGGAGCGCATGATG
TP33568_Hit	D	1	chr1	.	CAGCCTGCCCTTTTTAAATTCAAGAACCTGCAGGAGTAAGATTTCAAATGGTTCTTAGTAATGA
TP33568_Query	D	1	chr1	.	CAGCCTGCCCTTCTTAAATTCAAGAACCTGCAGGAGTAAGATTTCAAATGGTTCTTAGTAATGA
TP33598_Hit	D	1	chr1	.	CAGCCTGCGGGTCTTCTTGATACACTCAAAATGGAGAGGGTCAGAACTATTCTGACCCATACTT
TP33598_Query	D	1	chr1	.	CAGCCTGCGGGTCTCCTTGATACACTCAAAATGGAGAGGGTCAGAACTATTCTGACCCATACTT
TP33661_Hit	D	1	chr1	.	CAGCCTGGAAAGCAAGGGTTGCATTCTGCTCTATGTATTCTGGATGGTCAAGTAAATTCACTTT
TP33661_Query	D	1	chr1	.	CAGCCTGGAAAGCAAGGGTTGCATTCTGCTCTATATATTCTGGATGGTCAAGTAAATTCACTTT
TP33699_Hit	D	1	chr1	.	CTGCCTGGCACCACATATTGGGGTGGAAAATCCCTGTTTAGATTAAAACCTAATAATGAGCATT
TP33699_Query	D	1	chr1	.	CAGCCTGGCACCACATATTGGGGTGGAAAATCCCTGTTTAGATTAAAACCTAATAATGAGCATT
TP33737_Hit	D	1	chr1	.	CAGCCTGGGGAATATCTGTGGCCGGATAGTGCAAACCCAGATATGGCTCTAGGCCTGTCTTTGT
TP33737_Query	D	1	chr1	.	CAGCCTGGGGAATATCTGTGGCCGGATAGTGCAAACCCAGATATGGCTCTAGACCTGTCTTTGT
TP33759_Hit	D+G	1	chr1	.	CAGCCTGGTCCAAGGGGCTCTCTTATTCAGTGCTATATTAAACGAAATCGCGGCGACCAAATTT
TP33759_Query	D+G	1	chr1	.	CAGCCTGGTCCAAGGGGCTCTCTCATTCAGTGCTATATTAAACGAAATCGCGGCGACCAAATTT
TP33827_Hit	D	1	chr1	.	CAGCTTGTCCAAAAGAGCAGTGGCGAGAAGGAACTTCATGAGAAATCTTGGATTCTTTCTTAAG
TP33827_Query	D	1	chr1	.	CAGCCTGTCCAAAAGAGCAGTGGCGAGAAGGAACTTCATGAGAAATCTTGGATTCTTTCTTAAG
TP3397_Hit	D+G	1	chr1	.	CAGCAACAGACCTCCTAGTCAATGATGAAAGGATCCTCTCATTAAGAGGTGGTGGTGAGGTGTA
TP3397_Query	D+G	1	chr1	.	CAGCAACAGACCTCCTAGTCAATGATGAAAGGATCCTCTCATTAAGAGGTGGAGGTGAGGTGTA
TP34056_Hit	D	1	chr1	.	CAGCCTTAATGGAAGGTGGATTACTAGGTTGGCTGGTGGCGGATGAAGTGTCAACGGGCTTGAT
TP34056_Query	D	1	chr1	.	CAGCCTTAATGGAAGGTGGATTACTAGGTTGGCTGGTGGCGGATGAAGGGTCAACGGGCTTGAT
TP3448_Hit	D	1	chr1	.	CAGCAACAGCAATGAACTAAACCAAGTATCAGAAAGAAGGTCATTTAAGTACAACAGTCGCAGA
TP3448_Query	D	1	chr1	.	CAGCAACAGCAATGAACTAAACCAAGTATCAGAAAGAAGGTCATTTAAGTACAACAGTCACAGA
TP34613_Hit	D	1	chr1	.	CAGCCTTGCAATTGGATCCCAACAATGAGTCTGTTAGAGAAAATATAAGGGTTTGTTCTGTTCT
TP34613_Query	D	1	chr1	.	CAGCCTTGCAATTGGATCCCAACAATGAGTCTGTTAAAGAAAATATAAGGGTTTGTTCTGTTCT
TP3467_Hit	D	1	chr1	.	CAGCAACAGCATCGATAGCCCTTCCAATAATGTTTTTAATAGCTTGATGTAGATGACGAGATAA
TP3467_Query	D	1	chr1	.	CAGCAACAGCATCGATAGCCCTTCCAATAATGTTTTTAATAGCTTGATGCAGATGACGAGATAA
TP34676_Hit	D	1	chr1	.	CAGCCTTGGATTTGGATCTCAGTCTGAACCATTTATTTCCTGCCATGGAATCCTCGAAAATGTT
TP34676_Query	D	1	chr1	.	CAGCCTTGGATTTGGATCTCAATCTGAACCATTTATTTCCTGCCATGGAATCCTCGAAAATGTT
TP34796_Hit	D	1	chr1	.	CAGCGTTGTTTGGAGCAAGAGAAGATGCAAGCCATGAATTGGAAGAATTGAATAAGACTATAGG
TP34796_Query	D	1	chr1	.	CAGCCTTGTTTGGAGCAAGAGAAGATGCAAGCCATGAATTGGAAGAATTGAATAAGACTATAGG
TP3483_Hit	D+G	1	chr1	.	CAGCAACGGCCGCGCCTGTAGCAACAGCTGTGGCTAGAATCAATGAAAAGGCTTGTTTGTCCTG
TP3483_Query	D+G	1	chr1	.	CAGCAACAGCCGCGCCTGTAGCAACAGCTGTGGCTAGAATCAATGAAAAGGCTTGTTTGTCCTG
TP34874_Hit	D+G	1	chr1	.	CAGCCTTTCATGCTCAAACCGTCCTGTACCCCCACCATGGGATTCTGTAATGGGTATCTTATCC
TP34874_Query	D+G	1	chr1	.	CAGCCTTTCATGCTCAAACCGTCCTGTACCCCCACCATGGGATTCTGTAATGGGTATCTCATCC
TP34875_Hit	D+G	1	chr1	.	CAGCCTTTCATGCTCAAATCTCCCTGTTCCTCCACCGTGGGATTCTGTAATGGGTATCTTATCC
TP34875_Query	D+G	1	chr1	.	CAGCCTTTCATGCTCAAATCTCCCTGTTCCTCCACCGTCGGATTCTGTAATGGGTATCTTATCC
TP34964_Hit	D+G	1	chr1	.	CAGCCTTTGCAGAGTTCTTCTCTATGCTCATTTTTGTTTTTGCTGGCCAAGGCTCCGGCATGGC
TP34964_Query	D+G	1	chr1	.	CAGCCTTTGCAGAGTTCTTCTCTATGCTCATTTTTGTTTTTGCTGGCCAAGGCTCCAGCATGGC
TP35080_Hit	D	1	chr1	.	CAGCCTTTTCTCTTCTAGTTTTTGTCCATCTCATCAACCTTGCTTCTTCAAATCCTACTAAGAA
TP35080_Query	D	1	chr1	.	CAGCCTTTTCTATTCTAGTTTTTGTCCATCTCATCAACCTTGCTTCTTCAAATCCTACTAAGAA
TP35140_Hit	D	1	chr1	.	CAGCCTTTTTTGTGCTGTTTGTCTTTTGGCTTAAATTTCCAACAAAGCTTTGCCATTTTCTACT
TP35140_Query	D	1	chr1	.	CAGCCTTTTTTGTGCTGTTTGTCTTTTGGCTTAAATTACCAACAAAGCTTTGCCATTTTCTACT
TP35204_Hit	D	1	chr1	.	CAGCGAAAATTGAGCTTGCTGGAAGATTATGGAGGTGACTGTGGTGGTTGTCGGGGACGGGTCT
TP35204_Query	D	1	chr1	.	CAGCGAAAATTGAGCTTGCTGGAAGATTATGGAGGTGACTATGGTGGTTGTCGGGGACGGGTCT
TP3524_Hit	D+G	1	chr1	.	CAGCAATAGGCACAGACGGCTCTGGAGCCGATGTTGATGATACCTGAACCACTTCAACATCCTT
TP3524_Query	D+G	1	chr1	.	CAGCAACAGGCACAGACGGCTCTGGAGCCGATGTTGATGATACCTGAACCACTTCAACATCCTT
TP35333_Hit	D	1	chr1	.	CAGCGAACAATTAAGTGTCATTGTTTTTTAAGTTGAATTGCATAGAACACATGATTTAATTCTG
TP35333_Query	D	1	chr1	.	CAGCGAACAATTAAGTGTCATTGTTTATTAAGTTGAATTGCATAGAACACATGATTTAATTCTG
TP35456_Hit	D	1	chr1	.	CAGCGAAGCATCACCATCCCCATGTTCCTCCTCTAATTGCCATCCGTAGTGCGGTCAAGTTGGT
TP35456_Query	D	1	chr1	.	CAGCGAAGCATCACCATCCCCATGTTCCACCTCTAATTGCCATCCGTAGTGCGGTCAAGTTGGT
TP3546_Hit	D	1	chr1	.	CAGCAACAGGTAGAGGATGGACAACAGGTTCTGGGATGGAAGGTCCCCCTGTTCCTGCTGTTGG
TP3546_Query	D	1	chr1	.	CAGCAACAGGTAGAGGATGGACAACAGGTTCTGGGATGGAAGGTCCCCCTGTTCCTGCGGTTGG

Name	Filter	Nb hit (Mt4.0)	Mt Chr (Mt4.0)	Ms Chr (Li et al., 2014)	Sequence
TP3552_Hit	D+G	1	chr1	.	CAGCAACAGGTGGTTGGATTAATGATGCTTACGGACGAAAGAAGGCAACTCTGATCGCAGATGT
TP3552_Query	D+G	1	chr1	.	CAGCAACAGGTGGTTGGATTAACGATGCTTACGGACGAAAGAAGGCAACTCTGATCGCAGATGT
TP35620_Hit	D	1	chr1	.	CTGCGAATTCGCTACGAATCCAATTCATAAACCCATCTCTCTCATCAACAAACCATTGTTGTTG
TP35620_Query	D	1	chr1	.	CAGCGAATTCGCTACGAATCCAATTCATAAACCCATCTCTCTCATCAACAAACCATTGTTGTTG
TP35666_Hit	D	1	chr1	.	CAGCGACAAATTTCTCTGCTTCATCTTGGTTTCAGTGGATAAAGGCTCCATACCGCCCTAGTTC
TP35666_Query	D	1	chr1	.	CAGCGACAAATTTCTCAGCTTCATCTTGGTTTCAGTGGATAAAGGCTCCATACCGCCCTAGTTC
TP35667_Hit	D	1	chr1	.	CAGCGACAACACAATAGAGATAAAGATGAGACAAAGTAAAGTATTGGACCACTCACTCACTATC
TP35667_Query	D	1	chr1	.	CAGCGACAACACAATAGAGATAAAGATGAGACAAAGTAAAGTATTGGACCACTCACTCACTACC
TP35768_Hit	D	1	chr1	.	CAGCGACATATAGTGTTCATGGAGTGAGTGGTGGTGTGATTTTTCCATTAGGAAGTACTATTTT
TP35768_Query	D	1	chr1	.	CAGCGACATATACTGTTCATGGAGTGAGTGGTGGTGTGATTTTTCCATTAGGAAGTACTATTTT
TP3584_Hit	D+G	1	chr1	.	CAGCAACGGTCCTTACATTGGCTATAGCCTCTCCAGCTAGTTGTGTAGCCTTGGCATGAGCGGC
TP3584_Query	D+G	1	chr1	.	CAGCAACAGTCCTTACATTGGCTATAGCCTCTCCAGCTAGTTGTGTAGCCTTGGCATGAGCGGC
TP35973_Hit	D+G	1	chr1	.	CAGCGACTCCAATCTCAGCTGAATACTCTTCATGTTGATTTCCTGATAAGTTCTGGTCTACCAA
TP35973_Query	D+G	1	chr1	.	CAGCGACTCCAATCTCAGCTGAATACTCTTCATGTTGATTTCCTGATAAGTTCTCGTCTACCAA
TP36104_Hit	D	1	chr1	.	CAGCGAGACATTCTTTCTGGAACGGAACTGGAGGTGCTGATCATTTTCTAGTTGGTTGTCATGA
TP36104_Query	D	1	chr1	.	CAGCGAGACATTCTTTCTGGAACAGAACTGGAGGTGCTGATCATTTTCTAGTTGGTTGTCATGA
TP3613_Hit	D	1	chr1	.	CAGCAACAGTTTTAGCTTTTGTAACAACCTTAGTCTTGACAACAGCTTTAGCCTTGGGCTTAGG
TP3613_Query	D	1	chr1	.	CAGCAACAGTTTTAGCATTTGTAACAACCTTAGTCTTGACAACAGCTTTAGCCTTGGGCTTAGG
TP36174_Hit	D	1	chr1	.	CAGCGAGATGGAGGAATTCAACGATCGTGCCACCGGACTTGGGTATGACTATAAGGTGCTGAAA
TP36174_Query	D	1	chr1	.	CAGCGAGATGGAGGAATTCAACAATCGTGCCACCGGACTTGGGTATGACTATAAGGTGCTGAAA
TP36592_Hit	D	1	chr1	.	CAGCGATCTCAGCCGTTAATTTGAGATTGAACGTCTTAAATTACAAATTAACAAAATCAGTTTT
TP36592_Query	D	1	chr1	.	CAGCGATCTCACCCGTTAATTTGAGATTGAACGTCTTAAATTACAAATTAACAAAATCAGTTTT
TP36606_Hit	D+G	1	chr1	.	CAGCGATCTTTGATTTATTATTTGTTTATATATTTATTACTAACGTCATGCCAATACTATTTCT
TP36606_Query	D+G	1	chr1	.	CAGCGATCTTTGATTTATTAGTTGTTTATATATTTATTACTAACGTCATGCCAATACTATTTCT
TP36727_Hit	D	1	chr1	.	CAGCGATGTATTCAGAGAATCAATTCTCCCTCCTCCAGACCCAGGACCAAACTATGCTAAATTC
TP36727_Query	D	1	chr1	.	CAGCGATGGATTCAGAGAATCAATTCTCCCTCCTCCAGACCCAGGACCAAACTATGCTAAATTC
TP3678_Hit	D+G	1	chr1	.	CAGCAACATCAACAACAATGGTTTGTTGATGAGAGAGATGGGTTTATGAATTGGATTCGTAGCG
TP3678_Query	D+G	1	chr1	.	CAGCAACATCAACAACAATGGTTTGTTGATGAGAGAGACGGGTTTATGAATTGGATTCGTAGCG
TP36847_Hit	D	1	chr1	.	CAGCGATTGCCCATTTGCTGTCGTCAACCTGGTGAGTTAAGATCGCTAACAATTGAACTGAGTA
TP36847_Query	D	1	chr1	.	CAGCGATTGCCCATTTGCTGTCGTCAACCTGGTGAGTTAAGATCACTAACAATTGAACTGAGTA
TP36973_Hit	D	1	chr1	.	CAGCGCAAGAGGAAATCACGTAAAGGTGGATCTTATTCTACACTTTCATTATTGAGGAAGGTGC
TP36973_Query	D	1	chr1	.	CAGCGCAAGAGGAAATCACGTAAAGGTGGATCTTATTCTACACTTTCATTATTGAGGAAGGAGC
TP3756_Hit	D	1	chr1	.	CTGCAACATGAAATGCATTCATATCAGATTTAGACCTGATTTTGAGTATCTCCAAATCACATAA
TP3756_Query	D	1	chr1	.	CAGCAACATGAAATGCATTCATATCAGATTTAGACCTGATTTTGAGTATCTCCAAATCACATAA
TP37685_Hit	D	1	chr1	.	CAGCGCCTTTATTCTTAACTCCTATAACATCTGATCTTGCTGAAGCTGAGTTCAAACCAGACTT
TP37685_Query	D	1	chr1	.	CAGCGCCTTTATTCTTAACTCCTATAACATCTGATCTTGCTGAAGCTGAATTCAAACCAGACTT
TP37790_Hit	D	1	chr1	.	CAGCGCGCCGATGCTTTGATGCTTTCTGGTGAGTCAGCTATGGGCCAGTTCCCTGATAAAGCCT
TP37790_Query	D	1	chr1	.	CAGCGCGCCGATGCTTTGATGCTTTCTGGTGAGTCAGCAATGGGCCAGTTCCCTGATAAAGCCT
TP37970_Hit	D	1	chr1	.	CAGCGCGTGAATTGCAGTTGATGCACCTTCCCACACCTGATTTCACCACCTGGCGAACCTGGTT
TP37970_Query	D	1	chr1	.	CAGCGCGTGAATTGCAGTTGATGCACCTTCCCACACCGGATTTCACCACCTGGCGAACCTGGTT
TP3814_Hit	D+G	1	chr1	.	CAGCAACATTAATGTTCTCTACTATGTAAGTCACATTATCTTCACTTATTTCACCTTTCATCTC
TP3814_Query	D+G	1	chr1	.	CAGCAACATTAATGTTCTCTACTATGTAAGTCACATGATCTTCACTTATTTCACCTTTCATCTC
TP38299_Hit	D	1	chr1	.	CAGCGTTTGCAAGTTCAGATGCAGATCTTATCTCTTCAATCGTTGCTCCATCTTTACCATATGA
TP38299_Query	D	1	chr1	.	CAGCGCTTGCAAGTTCAGATGCAGATCTTATCTCTTCAATCGTTGCTCCATCTTTACCATATGA
TP38311_Hit	D+G	1	chr1	.	CAGCGCTTGTAGGGACACTGGCTTTGTCCATAACCTGGATGGTACACTTGTCCTTTGTGATTGA
TP38311_Query	D+G	1	chr1	.	CAGCGCTTGTAGGGACACAGGCTTTGTCCATAACCTGGATGGTACACTTGTCCTTTGTGATTGA
TP38319_Hit	D	1	chr1	.	CAGCGCTTTACCTTTGTTTCATTGGTTGTCTAGCATTGTACACTGAAATCCATTGTGAAGCAGG
TP38319_Query	D	1	chr1	.	CAGCGCTTTACCTTTGTTTCATTGGTTGTCTAGCATTGTACACTGAAATCCATTGCGAAGCAGG
TP38361_Hit	D	1	chr1	.	CAGCGGAAAACTTGCAGAAGGTGCAGGGTTTAGTGGACATGGCCAAAGGCCACCTATAGACACT
TP38361_Query	D	1	chr1	.	CAGCGGAAAACTTGCAGAAGGTGCAGGGTTTAGTGGACATGGCCAAAGGCCACCCATAGACACT
TP3843_Hit	D	1	chr1	.	CAGCAACATTCTTCGTATCTCAGTTCTTTCTTGGTCTTGAGTAAAGTCTAGGGAGTCTTCAAAA
TP3843_Query	D	1	chr1	.	CAGCAACATTCTTCGTATCTCAGTTCTTTCTTGGTCTTGAGTAAAGCCTAGGGAGTCTTCAAAA
TP38488_Hit	D+G	1	chr1	.	CAGCGGACAAGCACGTCGTCTCCTTCATTTGCGAACAAATTTCCATCCTTCGTACTGACTCGTT
TP38488_Query	D+G	1	chr1	.	CAGCGGACAAGCACGTCGTCTCCTTCATTTGCGAACAAATTTCCATCCTGCGTACTGACTCGTT

Name	Filter	Nb hit (Mt4.0)	Mt Chr (Mt4.0)	Ms Chr (Li et al., 2014)	Sequence
TP38527_Hit	D+G	1	chr1	.	CAGCGGCCGTCATTTCAGTTTTGGGTAGGGGAGCACTTCTAATGGGGTTGTAAGCTGAAAAAAA
TP38527_Query	D+G	1	chr1	.	CAGCGGACGTCATTTCAGTTTTGGGTAGGGGAGCACTTCTAATGGGGTTGTAAGCTGAAAAAAA
TP38645_Hit	D+G	1	chr1	.	CAGCGGAGTCGGGATCGTTTGCTTGATCGTGGACTTCGTATTGATGCTTATGCTGATGCTCTTG
TP38645_Query	D+G	1	chr1	.	CAGCGGAGTCGGGATCGTTTGCTTGATCGTGGACTTCGTATTGATGCTTATGCTGATGCTCGTG
TP38648_Hit	D	1	chr1	.	CAGCTGAGTCTTCAGATGAGTGGTGTTTGACAACTCTTCGGAAAATAAGAGAAGCCTCCCCATT
TP38648_Query	D	1	chr1	.	CAGCGGAGTCTTCAGATGAGTGGTGTTTGACAACTCTTCGGAAAATAAGAGAAGCCTCCCCATT
TP38813_Hit	D	1	chr1	.	CAGCGGCAAGTTCACGGAAAGTAAAAGTTTGAGCGGCAATTTGGCCAGGTGGTCCGTCTTTGGG
TP38813_Query	D	1	chr1	.	CAGCGGCAAGTTCACGGAAAGTAAAAGTTTGAGCGGCAATTTGGCCAGGTGGTCCGTCTTTGAG
TP38979_Hit	D	1	chr1	.	CAGCGGCCCTCCAGGCAATGACAGAGTTTGGGATTCCTGAAGCAAAAGTGAAACGGGCGCTAAA
TP38979_Query	D	1	chr1	.	CAGCGGCCCTCCAGGCAATGACAGAGTTTGGGATTCCTGAAGCAAAAGTGAAACGGGCACTAAA
TP39011_Hit	D+G	1	chr1	.	CAGCGGCCGTTATTGATCAAGAAGGAAATCTAAAAGGAACTTTAAGTGTCTATAACAGACTTCT
TP39011_Query	D+G	1	chr1	.	CAGCGGCCGTCATTGATCAAGAAGGAAATCTAAAAGGAACTTTAAGTGTCTATAACAGACTTCT
TP3924_Hit	D	1	chr1	.	CAGCAACCAATGGAACATTGTCTATTATTGCAGATATGACTCCAATAGCACTTGCAATCAGTTC
TP3924_Query	D	1	chr1	.	CAGCAACCAATGGAACATTGTCTATAATTGCAGATATGACTCCAATAGCACTTGCAATCAGTTC
TP39346_Hit	D	1	chr1	.	CAGCGGCTTGTTTTGCGTTCCCTTCTACGATTGCAGTGTAACGACCGGTTTCAGGCATTTTCAT
TP39346_Query	D	1	chr1	.	CAGCGGCTTGTTTCGCGTTCCCTTCTACGATTGCAGTGTAACGACCGGTTTCAGGCATTTTCAT
TP3943_Hit	D	1	chr1	.	CAGCAACCACAGATTCAAGATCTTTCATTTTGACACGTTTTCTGAGGGGTTCATGCTTATCTCC
TP3943_Query	D	1	chr1	.	CAGCAACCACAGATTCAAGATCTTTCATTTTGACACGTTTTCTAAGGGGTTCATGCTTATCTCC
TP39620_Hit	D+G	1	chr1	.	CAGCGGGTTCCGTTCAATTCCACCAGACTCAATGCTCCATGTTAATATTGAATTGGTGTCCTTC
TP39620_Query	D+G	1	chr1	.	CAGCGGGTTCCGTTCAATTCCACCAGACTCAATGCTCCATGTTAATATTGAATTAGTGTCCTTC
TP39865_Hit	D+G	1	chr1	.	CAGCGGTGGAGAAGTTTAGGGTGACGCATATGTTTTTTGCTCCGCCGGTGATGGTGGAGCTGAT
TP39865_Query	D+G	1	chr1	.	CAGCGGTGGAGAAGTTTAGGGTGACACATATGTTTTTTGCTCCGCCGGTGATGGTGGAGCTGAT
TP39882_Hit	D	1	chr1	.	CAGCTGTGGCAAGGTTCTTTGCCTTGAAGCAGGTTACCATTGCATTCTGCAATGCTAACCTCAA
TP39882_Query	D	1	chr1	.	CAGCGGTGGCAAGGTTCTTTGCCTTGAAGCAGGTTACCATTGCATTCTGCAATGCTAACCTCAA
TP399_Hit	D	1	chr1	.	CAGCAAAACATTTTAAGTAACAATCTTTTTGCTCCTAAATCATTTTGCAGTCCAACTCCATTTG
TP399_Query	D	1	chr1	.	CAGCAAAACATTTTAAGTAACAATCTTTTTGCTCCTAAATCATTTGGCAGTCCAACTCCATTTG
TP39979_Hit	D+G	1	chr1	.	CAGCGGTTAAGCTTGAAGCAGAGGATCATGAGGTAAAGGAATGTACAGAAGTCAATGTGTTTGT
TP39979_Query	D+G	1	chr1	.	CAGCGGTTAAGCTTGAAGCAGAGGATCATGAGGTAAAGGAATGTACAGAAGTCAAGGTGTTTGT
TP39987_Hit	D	1	chr1	.	CAGCGGTTAGCCCAGCAGGACCAGCTCCAATGATAATCACTCTCTTTTTAGCCTCTAAATTGGG
TP39987_Query	D	1	chr1	.	CAGCGGTTAGCCCAGCAGGACCAGCTCCAATGATAATCACTCTCTTTTTAGCCTCTAAATTGGA
TP40101_Hit	D+G	1	chr1	.	CAGCGGTTTTTACAGGTAAATGGTAGTTATTTAAAAATGAAATTTGTTTTTCTAATATATGGGT
TP40101_Query	D+G	1	chr1	.	CAGCGGTTTTTACAGGTAAATGGTAATTATTTAAAAATGAAATTTGTTTTTCTAATATATGGGT
TP40434_Hit	D	1	chr1	.	CAGCGTCACCCAATCTGTATCGGTTGCCTGCACACTTTAGAACACACCAGCATTGTTCAACAAC
TP40434_Query	D	1	chr1	.	CAGCGTCACCCAATCTGTATCGGTTCCCTGCACACTTTAGAACACACCAGCATTGTTCAACAAC
TP4047_Hit	D	1	chr1	.	CAGCAACCCAAACAGAAACAGCATCTTCTTGCGAAACTGATGTGGAGTTCTCAGACCTCTCTTG
TP4047_Query	D	1	chr1	.	CAGCAACCCAAACAGAAACAGCATCTTCTGGCGAAACTGATGTGGAGTTCTCAGACCTCTCTTG
TP40969_Hit	D	1	chr1	.	CAGCGTGCTTCCATGTTCCTTTACCACTCACTCCTCCTTCTTCTGCATCTTTTTCTTCTATCCC
TP40969_Query	D	1	chr1	.	CAGCGTGCTTCCATGTTCCTTTACCACTCACTCCTCCTTCTTCTACATCTTTTTCTTCTATCCC
TP40988_Hit	D+G	1	chr1	.	CAGCGTGGAAGCTTCAATACTATCAATGGGCAATGCAGTTATTTGAACGTTATAACATTAGTGA
TP40988_Query	D+G	1	chr1	.	CAGCGTGGAAGCTTCAATACTATCAATGGGCAATGCAGTTATTTGAACGTTATAACATCAGTGA
TP41222_Hit	D	1	chr1	.	CAGCGTTAAGAAGTCTGTCGAAAACCGTACCCAATTTTCTAGACATTCAATTATAATCCTCTTT
TP41222_Query	D	1	chr1	.	CAGCGTTAAGAAGTCTGTCGAAAACCATACCCAATTTTCTAGACATTCAATTATAATCCTCTTT
TP41414_Hit	D	1	chr1	.	CAGCGTTGATTTAACAGGACACTCCGCCAAGAAAAAAGATAATTTCAAACCTTTATCTTCAGAT
TP41414_Query	D	1	chr1	.	CAGCGTTGATTTAACAGGACACTCCACCAAGAAAAAAGATAATTTCAAACCTTTATCTTCAGAT
TP41481_Hit	D	1	chr1	.	CAGCGTTGTAGGGCAAAAGTAGAAATCGAAAAAGTTCCTTTTTGAAGAATAATAAAAATTCTGT
TP41481_Query	D	1	chr1	.	CAGCGTTGTAGGGCAAAAGTAGAAATCAAAAAAGTTCCTTTTTGAAGAATAATAAAAATTCTGT
TP4151_Hit	D	1	chr1	.	CAGCAACCTACAATCCATTACTGAAGGCCCTCTGTAGAGACAAAAACATTGATGAAGCTATTGC
TP4151_Query	D	1	chr1	.	CAGCAACCTACAATCCATTACTGAAGGCCCTCTGTAGAGACAAAAACATTGAGGAAGCTATTGC
TP41711_Hit	D	1	chr1	.	CAGCTAAAAACTGAAATGGTACACATACCTTTTGTCCCAATAAGCCTAACACACTCATTCTCAC
TP41711_Query	D	1	chr1	.	CAGCTAAAAACTGAAATGATACACATACCTTTTGTCCCAATAAGCCTAACACACTCATTCTCAC
TP41757_Hit	D	1	chr1	.	CAGCTAAAACCTAATCTATTTAATTCCTCCATATGATTTATTTAACTTATTTCTTTATTGTGTG
TP41757_Query	D	1	chr1	.	CAGCTAAAACCTAATCTATTTAATTCCTCCAGATGATTTATTTAACTTATTTCTTTATTGTGTG
TP41793_Hit	D	1	chr1	.	CAGCTAAAAGGGGACTTGAAAGTATTGTTAAGTTACTTCTTTTGCATGGAGGTATGGTCAAGTG
TP41793_Query	D	1	chr1	.	CAGCTAAAAGGGGACTTGAAAGTATTGTTAAGTTACTTCTTTTACATGGAGGTATGGTCAAGTG

Name	Filter	Nb hit (Mt4.0)	Mt Chr (Mt4.0)	Ms Chr (Li et al., 2014)	Sequence
TP41826_Hit	D	1	chr1	.	CAGCTAAAATTGTTTGGTTGGGGCCTATTGTATATCAGCAAGAGGCAGAATTTGGATCTATCTT
TP41826_Query	D	1	chr1	.	CAGCTAAAATTGTTTGGTTGGGGCCTATTGTATATCAGCAAGAGACAGAATTTGGATCTATCTT
TP41834_Hit	D+G	1	chr1	.	CAGCTAAACAAAAGACGGTATTTCCATCAGTTCTACGAATTTCCATATCTTTTTTGTTTGTACG
TP41834_Query	D+G	1	chr1	.	CAGCTAAACAAAAGACGGTATTTCCATCAGTTCTACGAATTTCCATATCCTTTTTGTTTGTACG
TP41937_Hit	D	1	chr1	.	CAGCTAAAGCGGCCGCTATCTGATTCTGCACCGCTACGATGATTACCGTAGCGGTTCGTTGCCG
TP41937_Query	D	1	chr1	.	CAGCTAAAGCGGCCGCTATCTGATTCTGCACCGCTACGATGATTACCGTAGCGGTTCGTCGCCG
TP42053_Hit	D+G	1	chr1	.	CAGCTAAATGATAAGGTTCCATAGTGCATTAAACATTAACCTCGCGGTTTTAGCACTAGACTCC
TP42053_Query	D+G	1	chr1	.	CAGCTAAATGATAAGGTTCCATAGTGCACTAAACATTAACCTCGCGGTTTTAGCACTAGACTCC
TP42070_Hit	D	1	chr1	.	CAGCTAAATGTTTCCGGGAACATGAAAGAATTTAAGAAGACTCACCCTTCCAGGTTCTGAGCCT
TP42070_Query	D	1	chr1	.	CAGCTAAATGTTTCCGGGAACATGAAAGAATTTAAGAAAACTCACCCTTCCAGGTTCTGAGCCT
TP42198_Hit	D	1	chr1	.	CAGCTAACATTGAAAGAGTTTGGTTAGCGATACCACTAGCATTAGACACGGTAGTATTTGCTCC
TP42198_Query	D	1	chr1	.	CAGCTAACATTGAAAGAGTTTGGTTAGCGATACCACTAGCATTAGACACGGCAGTATTTGCTCC
TP42348_Hit	D	1	chr1	.	CAGCTAAGAACGAAATTTCACTCACTATTTTCATGTCAATTTTAGAAATATATATGTGAAATTC
TP42348_Query	D	1	chr1	.	CAGCTAAGAACGAAATTTCACTCACTATTTTCAAGTCAATTTTAGAAATATATATGTGAAATTC
TP42349_Hit	D	1	chr1	.	CAGCTAAGAGCTCCAGCTCTAGGAAAACCAATGGGAAGAGATATATTATCGGTGATCAAGATCA
TP42349_Query	D	1	chr1	.	CAGCTAAGAACTCCAGCTCTAGGAAAACCAATGGGAAGAGATATATTATCGGTGATCAAGATCA
TP42410_Hit	D+G	1	chr1	.	CAGCTAAGCCAAGGTATGGGTTTGCAGAACCGTCAAATGATTTCACTTCAAAATTACTCACTAA
TP42410_Query	D+G	1	chr1	.	CAGCTAAGCCAAGGTATGGGTTTGCAGAACCATCAAATGATTTCACTTCAAAATTACTCACTAA
TP42469_Hit	D	1	chr1	.	CAGCTAAGGCTAAGTATGGGTTTGCAGTACCATCTAATGATTTCAACTCAAAATTACTCACTAA
TP42469_Query	D	1	chr1	.	CAGCTAAGGCTAAGTATGGATTTGCAGTACCATCTAATGATTTCAACTCAAAATTACTCACTAA
TP42484_Hit	D	1	chr1	.	CAGCTAAGGTCAGAATCAAATTTGAGCCAATGGCATCAATGAATAACCTCAAGAGAATGGTTAG
TP42484_Query	D	1	chr1	.	CAGCTAAGGTCAGAATCAAATTTGAGCCAATGGCATCAATGAATAACCTCAAGAGAAGGGTTAG
TP42524_Hit	D	1	chr1	.	CAGCTATGTTATGTGGGATTTTATTTTGTTAAGTTTGGAGGTGAAGGAAACACTGATAAAAAGT
TP42524_Query	D	1	chr1	.	CAGCTAAGTTATGTGGGATTTTATTTTGTTAAGTTTGGAGGTGAAGGAAACACTGATAAAAAGT
TP42545_Hit	D	1	chr1	.	CAGCTAATAAGTACGACATCGGTGATCTCAAAGACGCATGCGAGGAAAGCCTACTTGAAGATCT
TP42545_Query	D	1	chr1	.	CAGCTAATAAATACGACATCGGTGATCTCAAAGACGCATGCGAGGAAAGCCTACTTGAAGATCT
TP42601_Hit	D	1	chr1	.	CAGCTAATAGTCCATACCACCCATACCAGGCATGGCAGGAGCATCTTTATCTTCTTTTGGTAGT
TP42601_Query	D	1	chr1	.	CAGCTAATAGTCCATACCACCCATACCAGGCATGGAAGGAGCATCTTTATCTTCTTTTGGTAGT
TP42690_Hit	D	1	chr1	.	CAGCTAATGAGAACTATTTCGGAACAAAGAAGCCAAACAAAGACTCAACCTAGCAGTTCTCAGA
TP42690_Query	D	1	chr1	.	CAGCTAATGAGAACTATTTAGGAACAAAGAAGCCAAACAAAGACTCAACCTAGCAGTTCTCAGA
TP43074_Hit	D+G	1	chr1	.	CAGCTAGAGAATATTTCAATTCAGAACTAGATGTGATTCAAGAACTAGAATAGAATAGAATATA
TP43074_Query	D+G	1	chr1	.	CAGCTACAGAATATTTCAATTCAGAACTAGATGTGATTCAAGAACTAGAATAGAATAGAATATA
TP43101_Hit	D	1	chr1	.	CAGCTACAGCATTCAAAGAGTGAAAATATTTACAATGAGCTTCAGCGAGTGCATATCTTTTCTC
TP43101_Query	D	1	chr1	.	CAGCTACAGCATTCAAAGAGTGAAAATATTTACAATGAGCTTCAGCAAGTGCATATCTTTTCTC
TP43274_Hit	D+G	1	chr1	.	CAGCTACCATAATCAATATGGATTTGCACTTCTGCGTTGTCATTCCAATCATAATGACATGAAT
TP43274_Query	D+G	1	chr1	.	CAGCTACCATAATCAATATGGATTTGCACTTCTGCCTTGTCATTCCAATCATAATGACATGAAT
TP4330_Hit	D	1	chr1	.	CAGCAACGATGAAAATTGCTCTAAAAATCTGACCAGGAACATCTGCAAGATAATAACAGTATAT
TP4330_Query	D	1	chr1	.	CAGCAACGATGAAAATTGCTCTAAAAATCCGACCAGGAACATCTGCAAGATAATAACAGTATAT
TP43343_Hit	D	1	chr1	.	CTGCTACCTCTGGTTCAACATCCGCCTGAAAAATCAATCAAATTCATCATTCAACAATGCGAAA
TP43343_Query	D	1	chr1	.	CAGCTACCTCTGGTTCAACATCCGCCTGAAAAATCAATCAAATTCATCATTCAACAATGCGAAA
TP43345_Hit	D	1	chr1	.	CAGCTACCTGAGTTTTCCACTTTGTCTCTCAAAGCTAATCTCAATTTTGTTGAGAAGCTTTTCG
TP43345_Query	D	1	chr1	.	CAGCTACCTGAGTTTTCCACTTTGTCTCTCAAAGCTAATCTCAATTTTGTCGAGAAGCTTTTCG
TP43388_Hit	D+G	1	chr1	.	CAGCTATGAAGATAAGGAAGGAAGAAGAGGAAGAAACTAAGCGATTCCTTCAGTTACAAAGAAA
TP43388_Query	D+G	1	chr1	.	CAGCTACGAAGATAAGGAAGGAAGAAGAGGAAGAAACTAAGCGATTCCTTCAGTTACAAAGAAA
TP43439_Hit	D+G	1	chr1	.	CAGCTATGGTAACGGCTATGGTAATAGTAACCACAACGATAAGCCGTTTGTAAGTAGTAGCAAT
TP43439_Query	D+G	1	chr1	.	CAGCTACGGTAACGGCTATGGTAATAGTAACCACAACGATAAGCCGTTTGTAAGTAGTAGCAAT
TP43520_Hit	D	1	chr1	.	CAGCTACTATTATTTAGGAAGTACCTAGTTTGGTTAAATCAGGAATTAAGAATTTTCACTTGAG
TP43520_Query	D	1	chr1	.	CAGCTACTATTATTTAGGAAGTACCTAGTTTGGTTAAAACAGGAATTAAGAATTTTCACTTGAG
TP43637_Hit	D	1	chr1	.	CAGCTTCTGTTGGTGGTGTTTCTCTCATTTTGATTTTAGTGATTCTATATTTAATGAGACCACC
TP43637_Query	D	1	chr1	.	CAGCTACTGTTGGTGGTGTTTCTCTCATTTTGATTTTAGTGATTCTATATTTAATGAGACCACC
TP43735_Hit	D	1	chr1	.	CAGCTAGAACAATTCTGAGAAACTTGTAATTTGATAGCAATTTCACCGCTTAGCACGTTAAAAA
TP43735_Query	D	1	chr1	.	CAGCTAGAACAATTCTGAGAAACTTGTAATTTGATAGCAATTTCACCGCTTAACACGTTAAAAA
TP43811_Hit	D	1	chr1	.	CAGCTAGACTGGGAAATTAAAATGAGTCATGTTTATCGTGAAGCGGATTTTGTGTGGATGGTCT
TP43811_Query	D	1	chr1	.	CAGCTAGACTGGGAAATTAAAATGAGTCATGTTTATCGTGAAGCGGATTTTGTGCGGATGGTCT

Name	Filter	Nb hit (Mt4.0)	Mt Chr (Mt4.0)	Ms Chr (Li et al., 2014)	Sequence
TP43840_Hit	D	1	chr1	.	CAGCTAGAGCAGAGAGCCACCATAACTGATCAAAGTCAGGAATTTGGGAGAAAAAGATTTCCAC
TP43840_Query	D	1	chr1	.	CAGCTAGAGCAGAGAGCCACCATAACTGATCAAAGTCAGGAATTTGAGAGAAAAAGATTTCCAC
TP43893_Hit	D	1	chr1	.	CAGCTAGATAAATAAGATTCTCCTTGTCAACAGTGTTAATCATATGCTCTAACCAGTCTTTAAA
TP43893_Query	D	1	chr1	.	CAGCTAGATAAATAAGATTCTCCTTGTCAACAGTGTTAATCATAGGCTCTAACCAGTCTTTAAA
TP44147_Hit	D+G	1	chr1	.	CAGCTAGGATATAGCATGGATGTCGTGAATAATGGAGTGGAAGCCATACGCGCAGTTCAGAGCC
TP44147_Query	D+G	1	chr1	.	CAGCTAGGATATAGCATGGATGTCGTCAATAATGGAGTGGAAGCCATACGCGCAGTTCAGAGCC
TP44289_Hit	D	1	chr1	.	CAGCTAGTCGAAGAGTCTCCATGGGAGGAGCAATGCTTCAAACTCCAAAACCTGATTCAAAAGC
TP44289_Query	D	1	chr1	.	CAGCTAGTAGAAGAGTCTCCATGGGAGGAGCAATGCTTCAAACTCCAAAACCTGATTCAAAAGC
TP44326_Hit	D	1	chr1	.	CAGCTAGTCTGCTGATTTGGTTCTGGATTATCAACTCCATGTGTTTTTTAGTCTATGTTGTTTG
TP44326_Query	D	1	chr1	.	CAGCTAGTCTGCTGATTTGGTTCTGGATTATCAACTCCATGTGTTTTTTAGTCTATGTTGCTTG
TP44343_Hit	D	1	chr1	.	CAGCTAGTGAGAAAAAATACTGGTTAATGGATGCAAACCTTAGATTCCCATTACCAATGATGGA
TP44343_Query	D	1	chr1	.	CAGCTAGTGAGAAAAAATACTGGTTAATGGATGCAAACCTTAGATTCCCATTACCAACGATGGA
TP44484_Hit	D	1	chr1	.	CAGCTATAAAGTCTGGAAAGCTGACCCCCGTCAATGGATGCGTGACCAACAATCTCTGCATTGT
TP44484_Query	D	1	chr1	.	CAGCTATAAAGTCTGGAAAGCTGACCCCCGCCAATGGATGCGTGACCAACAATCTCTGCATTGT
TP44504_Hit	D	1	chr1	.	CAGCTATAACTATTATTTAATACTAATTATCGTATACTAGAACAATTGTGGTTTGTTCCAATTC
TP44504_Query	D	1	chr1	.	CAGCTATAACTATTATTTAATACTAATTATCGTATACTAGAACAATAGTGGTTTGTTCCAATTC
TP44570_Hit	D+G	1	chr1	.	CAGCTATACAAGTGAGAGGGCCCATATGGTGAAGATATGGTGTCCAAACCACTTTTTGGTTGCT
TP44570_Query	D+G	1	chr1	.	CAGCTATACAAGTGAGAGAGCCCATATGGTGAAGATATGGTGTCCAAACCACTTTTTGGTTGCT
TP4458_Hit	D	1	chr1	.	CAGCAACTAAGAGCCTCGTTGAGAAGTTTGATGAAGAAGGAGTACCTACAGGGAAAGTTGCTAC
TP4458_Query	D	1	chr1	.	CAGCAACTAAAAGCCTCGTTGAGAAGTTTGATGAAGAAGGAGTACCTACAGGGAAAGTTGCTAC
TP44621_Hit	D	1	chr1	.	CAGCTATAGACATAAAATCGGTAAAGTAATGCAGATAAGTTGCCAACAGAAAATTATGAATGTT
TP44621_Query	D	1	chr1	.	CAGCTATAGACATAAAATCGATAAAGTAATGCAGATAAGTTGCCAACAGAAAATTATGAATGTT
TP44635_Hit	D	1	chr1	.	CAGCTATAGCAAAATTATTAAAATTCAATACAGAAGAAAATGCAAGGTTAGATTTCCTACAAAA
TP44635_Query	D	1	chr1	.	CAGCTATAGCAAAATTATTAAAATTCAATACAGAAGAAAATGCAAGATTAGATTTCCTACAAAA
TP44687_Hit	D	1	chr1	.	CAGCTATAGGTAACTGCATAAACACCTCTTTATTTAAATCGTGATTCAATAAGGCATTATTGAT
TP44687_Query	D	1	chr1	.	CAGCTATAGGTAACTGCATAAACACCTCTTCATTTAAATCGTGATTCAATAAGGCATTATTGAT
TP44729_Hit	D	1	chr1	.	CAGCTATATAAAGACAGTAAAATATATAGGTCAATTTATGTATGTTGTATAAGAGCGCATCTAA
TP44729_Query	D	1	chr1	.	CAGCTATATAAAGACAGTAAAATATATAGGTCAATTTATGTATGTTGTATAAGAGCGCATCCAA
TP44760_Hit	D+G	1	chr1	.	CAGCTATATCATCTAGCATGCATGTATCTGGTGGACATGTCAACCCTGCAGTTACTTTTGGTGC
TP44760_Query	D+G	1	chr1	.	CAGCTATATCATCTAGCATGCATGTATCTGGTGGACATATCAACCCTGCAGTTACTTTTGGTGC
TP44894_Hit	D	1	chr1	.	CAGCTATTATGAATGGACTTACTCTTGTTGGTGTTGCTGTTGGATTTGTTCTTCTTCGAATTGA
TP44894_Query	D	1	chr1	.	CAGCTATCATGAATGGACTTACTCTTGTTGGTGTTGCTGTTGGATTTGTTCTTCTTCGAATTGA
TP44974_Hit	D	1	chr1	.	CAGCTATCTCTCCCAGTTCAACATTTTCATGTATCACACAGGCGCCAAGCAGTGCACCCCATAC
TP44974_Query	D	1	chr1	.	CAGCTATCTCTCCCAGTTCAACATTTTCATATATCACACAGGCGCCAAGCAGTGCACCCCATAC
TP44983_Hit	D	1	chr1	.	CAGCTATCTGTGCCAACTTCTCACCAGAGAAAATGTTGCCCTTTGCTTCTCATGTGGTAAGCTG
TP44983_Query	D	1	chr1	.	CAGCTATCTGTGCCAACTTCTCACCAGAGAAAATGTTGCCCTTTGCTTCTCATGTGGTAAGCAG
TP45124_Hit	D	1	chr1	.	CAGCTATGCCACATGCACAGTTTTAAATTGCAGTCTACAACTTTATTTGAGTTTGCAACATCAA
TP45124_Query	D	1	chr1	.	CAGCTATGCCACATGCACAGTTTTAAATTGCAGTCAACAACTTTATTTGAGTTTGCAACATCAA
TP45192_Hit	D	1	chr1	.	CAGCTATGGCTACTAATGTCATTTTTTTGAAATTGTTTACAGATGTCTTCTAATCAGATCAATG
TP45192_Query	D	1	chr1	.	CAGCTATGGCTACTAACGTCATTTTTTTGAAATTGTTTACAGATGTCTTCTAATCAGATCAATG
TP45314_Hit	D	1	chr1	.	CAGCTATTAACATCATCTCCTTATCCATCACTCTCTTCCTCCTCCGAAACTCCCACCAATTCAC
TP45314_Query	D	1	chr1	.	CAGCTATTAACATCATCTCCTGATCCATCACTCTCTTCCTCCTCCGAAACTCCCACCAATTCAC
TP45338_Hit	D	1	chr1	.	CAGCTATTACAGCTGGCCTATGTTTATTCCTCAGCTCCTGCAATCGAACTTCATTTATATTTTG
TP45338_Query	D	1	chr1	.	CAGCTATTACAGCTGGCCTATGTTTATTCCTCAGCTCCTGCAATCGAACTTCAGTTATATTTTG
TP4537_Hit	D	1	chr1	.	CAGCAACTAGAACAAAAAGAAAGACAAACACTCAGAATATCAACAGATCACAATAACTTTCAGA
TP4537_Query	D	1	chr1	.	CAGCAACTAGAACAAAAAGAAAGACAAACACTCAGAATATCAACAGATCACAATAACTTCCAGA
TP45398_Hit	D	1	chr1	.	CAGCTATTCATTGTTCTCTATTGAAGGTCTTTTTCCATACCAGGAAAGGGGGAATGACATGTAA
TP45398_Query	D	1	chr1	.	CAGCTATTCACTGTTCTCTATTGAAGGTCTTTTTCCATACCAGGAAAGGGGGAATGACATGTAA
TP45413_Hit	D	1	chr1	.	CAGCTATTCGAATGTCTTGCCTGAGCCTGCTGAGTAAGAATTCATATTTTGCTTTCTTACTTAC
TP45413_Query	D	1	chr1	.	CAGCTATTCGAATGTCATGCCTGAGCCTGCTGAGTAAGAATTCATATTTTGCTTTCTTACTTAC
TP45416_Hit	D	1	chr1	.	CAGCTATTCGGTCGGCAGTAATGACAACATCAGACATATTTGATAATGCTAAGGAACACATCAA
TP45416_Query	D	1	chr1	.	CAGCTATTCGGTCGGCAATAATGACAACATCAGACATATTTGATAATGCTAAGGAACACATCAA
TP45620_Hit	D+G	1	chr1	.	CAGCTATTTGTGGATTCAAATTGCGTGGGATCTCAAGCCTTTTATGCTTGAAGCCAACGGCTGA
TP45620_Query	D+G	1	chr1	.	CAGCTATTTGTGGATTCAAATTGCGTGGGATCTCAAGCCTTTTATGCTTGAAGCCAACGGCAGA

Name	Filter	Nb hit (Mt4.0)	Mt Chr (Mt4.0)	Ms Chr (Li et al., 2014)	Sequence
TP45742_Hit	D	1	chr1	.	CAGCTCAAACTTGTTGGAAAACCGTTTGTGTCTGAAATGATCAAGAATCATGCTGAGTCAAACA
TP45742_Query	D	1	chr1	.	CAGCTCAAACTTGTTGGAAAACCGTTTGTGTCTGAAATGATCAAGAATCATGCAGAGTCAAACA
TP45939_Hit	D+G	1	chr1	.	CAGCTCAAGAGACTGCTTTCTGATGGTATGATTACTTTTGTTTTTAAGTTTGTCATTCATTTGG
TP45939_Query	D+G	1	chr1	.	CAGCTCAAGAGACTGCTTTCTGATGGTATGATTACCTTTGTTTTTAAGTTTGTCATTCATTTGG
TP45951_Hit	D	1	chr1	.	CAGCTCAAGATGGCAACAATAAAAACGACTAGGGTTTCCAATACACCCTCTCACACCAAAGATT
TP45951_Query	D	1	chr1	.	CAGCTCAAGATGGCAACAATAAAAACGACTAAGGTTTCCAATACACCCTCTCACACCAAAGATT
TP46003_Hit	D+G	1	chr1	.	CAGCTCAAGTAATCCTGAGTGTCAGGATGGTCCTTCTAACTACACCCTGTACATTTTGAAACGT
TP46003_Query	D+G	1	chr1	.	CAGCTCAAGTAATCCCGAGTGTCAGGATGGTCCTTCTAACTACACCCTGTACATTTTGAAACGT
TP46085_Hit	D	1	chr1	.	CAGCTCAATGTTGTCGTCAAGCACTTGTTATCAACCCTCTAATGGTAATTTTACTATTTGTTTG
TP46085_Query	D	1	chr1	.	CAGCTCAATGTTGTCGTCAAGCACTTGCTATCAACCCTCTAATGGTAATTTTACTATTTGTTTG
TP46204_Hit	D	1	chr1	.	CAGCTTACCATAGAAAAAGCCTTCAATTTTGCACCACAGTTAAAAAGGACGCCGAAAAACCCAA
TP46204_Query	D	1	chr1	.	CAGCTCACCATAGAAAAAGCCTTCAATTTTGCACCACAGTTAAAAAGGACGCCGAAAAACCCAA
TP46297_Hit	D	1	chr1	.	CAGCTCACTCCTTGACATGTATGCCAAAGATGGTAAAATTCATGAAGCTCGAACAGTGTTTGAA
TP46297_Query	D	1	chr1	.	CAGCTCACTCCTTGACATGTATGCCAAAGATGGTAAAATTCATGAAGCTCAAACAGTGTTTGAA
TP46320_Hit	D+G	1	chr1	.	CAGCTCACTTGCCTGGTAAATTACTTTAATTGTTCTTTGTACAATTTTCGCAAAGATCAACTGT
TP46320_Query	D+G	1	chr1	.	CAGCTCACTTGCCTGGTAAATTACTTTAATTGTTCTTTGTACAATTTCCGCAAAGATCAACTGT
TP4641_Hit	D	1	chr1	.	CAGCAACTCATGAAGAATACTATGCCAAGAATCTGTGTAGGATTGTTGATAAGAGTAGCAAACT
TP4641_Query	D	1	chr1	.	CAGCAACTCATGAAGAATACTATGCCAAGAATCTGTGTAGGATTGTTGATAAGAGTACCAAACT
TP46498_Hit	D	1	chr1	.	CAGCTCAGGAAATTGTCAAATATGACTTGAGCACCATTTGCAAAATCACCAAATTGTGCCTCCC
TP46498_Query	D	1	chr1	.	CAGCTCAGGAAATTGTCAAATATGACTTGAGCACCATTCGCAAAATCACCAAATTGTGCCTCCC
TP4654_Hit	D	1	chr1	.	CAGCAACTCCCTCTGTGCATTCTCTTTAATCCTTCTCTCAAGCTCGGGCCTGAAATGCCTGATA
TP4654_Query	D	1	chr1	.	CAGCAACTCCCTCTGCGCATTCTCTTTAATCCTTCTCTCAAGCTCGGGCCTGAAATGCCTGATA
TP46619_Hit	D	1	chr1	.	CAGCTCATAATCCTTCACTCCTCCATCACAGTTTAATTTGACAGTTCCACTACTAGGAGGGATC
TP46619_Query	D	1	chr1	.	CAGCTCATAATCCTTCACTCCTCCATCACAGTTTAATTTGACAGTTCCACTACTAGGAGGGACC
TP46637_Hit	D	1	chr1	.	CAGCTGATACTTAACCTTCTGAGGTAGATGGGTTTAGGCAATTAAGTTTAGAATTTCATTTTCT
TP46637_Query	D	1	chr1	.	CAGCTCATACTTAACCTTCTGAGGTAGATGGGTTTAGGCAATTAAGTTTAGAATTTCATTTTCT
TP46849_Hit	D+G	1	chr1	.	CAGCTCATTAGATCATGAATCTACATTTGGAGGGCCTCATGTGATGAGGGAGACTTTGACTGAA
TP46849_Query	D+G	1	chr1	.	CAGCTCATTAGATCATGAATCTACATTTGGAGGGCCTCATGTGATGAGGGAGACGTTGACTGAA
TP47016_Hit	D	1	chr1	.	CAGCTCCAAGAGTTCCTCCTGCAAGCAAAGAAGTTGTCTCAAAACTTCCTGTCATTACTTTCAC
TP47016_Query	D	1	chr1	.	CAGCTCCAAGAGTTCCTCCTGCAAGCAAAGAAGTTGTCTCAAAACTTCCTGTCATTACTCTCAC
TP4705_Hit	D	1	chr1	.	CAGCAACTGAAGAATGAAAATAACTATTCCAACAAGGTCAATCCAGCTGTGCTTTTAAGGATGT
TP4705_Query	D	1	chr1	.	CAGCAACTGAAGAATGAAAATAACTATTCCAACAAGATCAATCCAGCTGTGCTTTTAAGGATGT
TP4745_Hit	D	1	chr1	.	CAGCAACTGCGCTGTGGAGTAAGTCTTGCACTTTTCCTGGTTTTGGACCATATAGTTAATGTGA
TP4745_Query	D	1	chr1	.	CAGCAACTGCGCTGTGGAGTAAGTCGTGCACTTTTCCTGGTTTTGGACCATATAGTTAATGTGA
TP47684_Hit	D+G	1	chr1	.	CAGCTCCTAAGTGCTCCTTTTTCATGCACTCTTTGCTCACACTTTGCCCTTCTTTTCCAGCTTG
TP47684_Query	D+G	1	chr1	.	CAGCTCCTAAGTGCTCCTTTTTCATGCACTCTTTGCTCACACTTTGCCCTTCGTTTCCAGCTTG
TP47731_Hit	D	1	chr1	.	CAGCTCCTATTGCTTCAGCAACAGGTTCAGTGAGGTTAACCCCCTCTCCCTCGACACCGTCAAG
TP47731_Query	D	1	chr1	.	CAGCTCCTATTGCTTCAGCAACAGGTTCAGTGAGGTTAACCCCCTCTCCCTCAACACCGTCAAG
TP47875_Hit	D+G	1	chr1	.	CAGCTCCTGCACCCACTACAATGGCCAAACCCTTATTAGCTGGCAGATGTCGGGGAGCCAAACC
TP47875_Query	D+G	1	chr1	.	CAGCTCCTGCACCCACTACAAGGGCCAAACCCTTATTAGCTGGCAGATGTCGGGGAGCCAAACC
TP47925_Hit	D	1	chr1	.	CAGCTCCTTATGGTCAGAATCCTTTGTTGCTTTTATTAATCGGTCCATTAATTCCGGTTCCACA
TP47925_Query	D	1	chr1	.	CAGCTCCTTATGGTCAGAATCCTTTGTTGCTTTTATTAATCCGTCCATTAATTCCGGTTCCACA
TP47981_Hit	D	1	chr1	.	CAGCTCCTTGTATTTACAAACATCCCGCCTGTACTGTCTCTCTCTCATTTCATGGGCTAAAAAG
TP47981_Query	D	1	chr1	.	CAGCTCCTTGTATTTACAAACATCCCGCCTGTACTGTCTCTCTCTCATTGCATGGGCTAAAAAG
TP48169_Hit	D+G	1	chr1	.	CAGCTCGCAACGGAGCCTCCTTATTTTCATTTCCCCAAAACAAGTATGCACCACTCCATGTGTT
TP48169_Query	D+G	1	chr1	.	CAGCTCGCAACGGAGCCTCCTTATTTTCATTTCCCCAAAACAAGTATGCACCACTCCATGTGTG
TP48282_Hit	D	1	chr1	.	CAGCTCGCGCAGTTCATCGGCCCCAACGCCACCAACGCCGAAGCCGCGGCAAATTTCATCTGCA
TP48282_Query	D	1	chr1	.	CAGCTCGCGCAGTTCATCGGCCCCAACGCCACCAACGCCGAAGCCGCCGCAAATTTCATCTGCA
TP48331_Hit	D	1	chr1	.	CAGCTCGGCAGGCAACAGTAAGCTTCTCTTTCGCTAATGATAACTCATCCAATGCTGTCACTAC
TP48331_Query	D	1	chr1	.	CAGCTCGGCAGGCAACAGTAAGCTTCTCCTTCGCTAATGATAACTCATCCAATGCTGTCACTAC
TP48409_Hit	D	1	chr1	.	CAGCTCGGTTGCGACCACCACGTTGGAGAATTTTTTTGTCAATCATATCCAGCACGGGGTTATT
TP48409_Query	D	1	chr1	.	CAGCTCGGTTGCGACCACCACGTTGGAGAATTTTTTCGTCAATCATATCCAGCACGGGGTTATT
TP48530_Hit	D	1	chr1	.	CAGCTCGTTCCGTTCATTTTTATATGCTTCTGAGCTCTGATATAATTTGGCTTCATCAATTTCA
TP48530_Query	D	1	chr1	.	CAGCTCGTTCCGCTCATTTTTATATGCTTCTGAGCTCTGATATAATTTGGCTTCATCAATTTCA

Name	Filter	Nb hit (Mt4.0)	Mt Chr (Mt4.0)	Ms Chr (Li et al., 2014)	Sequence
TP48561_Hit	D	1	chr1	.	CAGCTCTAAAATAATCTTTTTCATTTTATCTAGGGTATACCACACCCTTTGAACTATATTTTTT
TP48561_Query	D	1	chr1	.	CAGCTCTAAAATAATCTTTTTCATTTTATCCAGGGTATACCACACCCTTTGAACTATATTTTTT
TP48595_Hit	D	1	chr1	.	CAGCTTTAAGACCTACTGTCTTCTGTAGTGTTCCTCGCCTATATAATAGAATATATGCCGGGTA
TP48595_Query	D	1	chr1	.	CAGCTCTAAGACCTACTGTCTTCTGTAGTGTTCCTCGCCTATATAATAGAATATATGCCGGGTA
TP48599_Hit	D	1	chr1	.	CAGCTCTAAGGACAGAGATTCTACCACCAAGAAGAGCACCAAAAGTAACTGCAGGGTTGATATG
TP48599_Query	D	1	chr1	.	CAGCTCTAAGGACAGAGATTCTACCACCAAGAAGAGCACCAAAAGTAACTGCAGGGTTGACATG
TP48656_Hit	D	1	chr1	.	CAGCTCTAGAAAACGAATGGCGTATCGGTCAATAGGGCGTAGCTCATTCTCAAAGGCTGATAAA
TP48656_Query	D	1	chr1	.	CAGCTCTAGAAAACGAATGGCGTATCGGTCAATAGGGCGTAGCTCATTCTCAAAGGCTGAAAAA
TP48707_Hit	D	1	chr1	.	CAGCTCTATCCTCAAAGTCCGTTGTTTGCGCTGAAATGGCCCTGTATAACAGGAACTGCATGCT
TP48707_Query	D	1	chr1	.	CAGCTCTATACTCAAAGTCCGTTGTTTGCGCTGAAATGGCCCTGTATAACAGGAACTGCATGCT
TP48770_Hit	D	1	chr1	.	CAGCTCTATTCAGCCTCTCTGTAATGGAGAACGGTAAAGCAAAAATCGGCCGTTCTGGTGCAAT
TP48770_Query	D	1	chr1	.	CAGCTCTATTCAGCCTCTCTGTAATGGAGAACGGTAAAGCAAAAATCGGCCGTTCCGGTGCAAT
TP48864_Hit	D	1	chr1	.	CAGCTCTCGTCTGAACAAGTCTCATCATCAGCATTCTGTGAAATATCAGGTGTCAAGGGTTGTT
TP48864_Query	D	1	chr1	.	CAGCTCTCATCTGAACAAGTCTCATCATCAGCATTCTGTGAAATATCAGGTGTCAAGGGTTGTT
TP48955_Hit	D+G	1	chr1	.	CAGCTCTCTACAAGTGGCATTTCTGATGATCAGAGGAAGACCACCATGAATATGGTAAATCCTA
TP48955_Query	D+G	1	chr1	.	CAGCTCTCTACAAATGGCATTTCTGATGATCAGAGGAAGACCACCATGAATATGGTAAATCCTA
TP48969_Hit	D	1	chr1	.	CAGCTCTCTCAGAAGTCCCATAAAATTTTGTATATCTCCTACAAGAAAAGAATTGAATGAATTT
TP48969_Query	D	1	chr1	.	CAGCTCTCTCAGAAGTCCCATAAAATTTTGTATATCTCCTACAAGAAAAGAATTGAACGAATTT
TP48986_Hit	D	1	chr1	.	CAGCTCTCTCTAACCCTTCTCTTGAGGTTATTCATTGATGCCATTGGCTCAAATTTGATTCTGA
TP48986_Query	D	1	chr1	.	CAGCTCTCTCTAACCATTCTCTTGAGGTTATTCATTGATGCCATTGGCTCAAATTTGATTCTGA
TP49031_Hit	D	1	chr1	.	CAGCTCTGAAAATAGTGAGCAAAGACAGGAAAAGGTTGATGATGTCCAAATATAAGGAGACAGA
TP49031_Query	D	1	chr1	.	CAGCTCTGAAAATAGTGAGCAAAGACAGGAAAAGGTTGATGATGTCCAAATATAAGGAAACAGA
TP4919_Hit	D	1	chr1	.	CAGCAACTTGGGCTTTTCCCATTTTCAGAAGAATGATGATGATAGTGTCTAGCAAAATTTTAAC
TP4919_Query	D	1	chr1	.	CAGCAACTTGGGCTTTTCCCATTTTCAGAAGAATGATGATGATAGTGTCTACCAAAATTTTAAC
TP49216_Hit	D	1	chr1	.	CAGCTCTGTGAGATTCCAGCAGTTCCATAGTTCACTTGCTACTTTTCTTTCTCTCACCACTGCT
TP49216_Query	D	1	chr1	.	CAGCTCTGTGAGATTCCAGCAGTTCCATAGTTCACTTGCTACTCTTCTTTCTCTCACCACTGCT
TP49229_Hit	D+G	1	chr1	.	CAGCTCTGTGTCAGATCCAATTCAATCTGATGGATGCTCTATACAATCTTCAATTCCAGATGAA
TP49229_Query	D+G	1	chr1	.	CAGCTCTGTGTCAGATCCAATTCAATCTGATGGATGCTCCATACAATCTTCAATTCCAGATGAA
TP49233_Hit	D	1	chr1	.	CAGCTTTGTGTTCTTTTTCATGCAAGAATCTTCGGTGAGTAATGAAACCTCGAATATGCGATTG
TP49233_Query	D	1	chr1	.	CAGCTCTGTGTTCTTTTTCATGCAAGAATCTTCGGTGAGTAATGAAACCTCGAATATGCGATTG
TP4927_Hit	D	1	chr1	.	CAGCAACTTGTCTAGGATTAAATGGTAACATGGTCTCTGGATATCCATAACCTATTCCAGGAGG
TP4927_Query	D	1	chr1	.	CAGCAACTTGTCTAGGATTAAATGGTAACATGGTCTCTGGATATCCATAACCTATTCCAGCAGG
TP49307_Hit	D	1	chr1	.	CAGCTCTTATACCCACCATAACTGAAGAGGATAAAGTTATACTGAACAATGTCCAGAAAGTGCT
TP49307_Query	D	1	chr1	.	CAGCTCTTATACCCACCATAACCGAAGAGGATAAAGTTATACTGAACAATGTCCAGAAAGTGCT
TP49348_Hit	D+G	1	chr1	.	CAGCTCTTCCGAAACTGGTCGGAAAGCTTCCAGTGAAGTAGTTTTGGCTCACATCAAAGCTTTT
TP49348_Query	D+G	1	chr1	.	CAGCTCTTCCGAAACCGGTCGGAAAGCTTCCAGTGAAGTAGTTTTGGCTCACATCAAAGCTTTT
TP49429_Hit	D	1	chr1	.	CAGCTCTTGAGTTTATAGTTGGCACGCTGGTCTCTAAGGGAGATATATCTTCCAGCACAGTATG
TP49429_Query	D	1	chr1	.	CAGCTCTTGAGTTTATAGTTGGCACACTGGTCTCTAAGGGAGATATATCTTCCAGCACAGTATG
TP49470_Hit	D+G	1	chr1	.	CAGCTCTTGGATGCTATGATATCTTCAGGAGATTTGGTTCTTCAACATATTACTGATATACTTG
TP49470_Query	D+G	1	chr1	.	CAGCTCTTGGATGCCATGATATCTTCAGGAGATTTGGTTCTTCAACATATTACTGATATACTTG
TP4949_Hit	D	1	chr1	.	CAGCAACTTTCTTAGCATCCTTCAAGTATGATTTGAGAAGGGGAACAGGTGGGAACTTTTCCAC
TP4949_Query	D	1	chr1	.	CAGCAACTTTCTTAGCATCCTTCAAGTATGATTTGAGAAGGGGAACAGGTGGGAACTTATCCAC
TP49803_Hit	D+G	1	chr1	.	CAGCTGAAAGATTCCCGGATAGTGATTTCGTCAACAAAGACATTGAAATTTCGATCCCTTGCGT
TP49803_Query	D+G	1	chr1	.	CAGCTGAAAGATTCCCGGATAGTGATTTCGTCAACAAAGACATTGAAATTTCGATCCCTTGCAT
TP50053_Hit	D	1	chr1	.	CAGCTGAAGCTGACCTGTATGTAGCAAAAATAGACAACAAGATTATTGTAAAGATAGGACCAAA
TP50053_Query	D	1	chr1	.	CAGCTGAAGCTGACATGTATGTAGCAAAAATAGACAACAAGATTATTGTAAAGATAGGACCAAA
TP50080_Hit	D+G	1	chr1	.	CAGCTGAAGGAGTGGGAAGTAGCCGTGACCAAGAATAACCTTTCTTCTAATGGAAGCCTGGATA
TP50080_Query	D+G	1	chr1	.	CAGCTGAAGGAGTGGGAAGTAGCCGTGACCAAGAATAACCTTTCTTCTAATGGAAGCCTGGAGA
TP50164_Hit	D	1	chr1	.	CAGCTGAATACCTTGTATCAGCCGGTTATACCCGACCCAAAAAGTTATGCATTGAAGGTGGAAG
TP50164_Query	D	1	chr1	.	CAGCTGAATACCTTCTATCAGCCGGTTATACCCGACCCAAAAAGTTATGCATTGAAGGTGGAAG
TP50168_Hit	D+G	1	chr1	.	CAGCTGAATAGATGAAAGTCAGATGGACTTGGCTTGAATGTTGCACAAACTCTTTGCTGTGTTG
TP50168_Query	D+G	1	chr1	.	CAGCTGAATAGATGAAAGTCAGATGGACTTGGCTTGAATGTTGCACAAACTCTTTGCTATGTTG
TP50247_Hit	D	1	chr1	.	CAGCTGAATTGCATATTTCTTCAGATATTTTTTCTGAAAAATAACTCCCAACGGATGGGAGTTG
TP50247_Query	D	1	chr1	.	CAGCTGAATTGCATATTTCTTCAGATATTTTTTCTGAAAAATAACTCCCAACAGATGGGAGTTG

Name	Filter	Nb hit (Mt4.0)	Mt Chr (Mt4.0)	Ms Chr (Li et al., 2014)	Sequence
TP5032_Hit	D	1	chr1	.	CAGCAAGAAATCCATTGCTACCAAAATTTTATCCTCAGTCTAGCAGTGGTGCGAGTTATTCCTA
TP5032_Query	D	1	chr1	.	CAGCAAGAAATCCACTGCTACCAAAATTTTATCCTCAGTCTAGCAGTGGTGCGAGTTATTCCTA
TP50346_Hit	D	1	chr1	.	CAGCTGACCCCAGAATAATCAGCATTGGTCTCTTTGGTACATTTTGTTGTTTCATAGAAACGAT
TP50346_Query	D	1	chr1	.	CAGCTGACCCCAGAATAATCAGCATTGGTCTCTTTGGTACATTCTGTTGTTTCATAGAAACGAT
TP50388_Hit	D	1	chr1	.	CAGCTGACGTGAGCAACTTGCAGAGAGAGGTTGAACACCTAACTGACCAGATCTCTGACATCTA
TP50388_Query	D	1	chr1	.	CAGCTGACGTGAGCAACTTGCAGAGAGAGGTTGAACACCTAACAGACCAGATCTCTGACATCTA
TP50452_Hit	D	1	chr1	.	CAGCTGAGACAGCTAGTAAGCAACATTTAGAAAGCATCAAGAAAGTAGCCAAGCTTGAGTCTGA
TP50452_Query	D	1	chr1	.	CAGCTGAGACAGCTAGTAAGCAACATTTAGAAAGCATCAAGAAAGTAGCCAAGCTTGAGTCCGA
TP50490_Hit	D+G	1	chr1	.	CAGCTGAGCCAATAACCAACATGGATCCGCCAGTACCAGCACAAAATGCAACCAATTGCCAGAA
TP50490_Query	D+G	1	chr1	.	CAGCTGAGCCAATAACCAACATGGATCCACCAGTACCAGCACAAAATGCAACCAATTGCCAGAA
TP50773_Hit	D	1	chr1	.	CAGCTGATCCAATTTTCATCAGACCAGGAAGAATGTTAAGTTTTGTTAGTTATACATAAATCGG
TP50773_Query	D	1	chr1	.	CAGCTGATCCAATTTTCATCAGACCAGGAAGAATGTTAAGTTTTGTTAGCTATACATAAATCGG
TP50958_Hit	D	1	chr1	.	CAGCTGATGTTTATGAAACAACCCTAAGACGTTTGGTTAAAAGCAATGTGATATCATCAACCAA
TP50958_Query	D	1	chr1	.	CAGCTGATGTTTATGAAACAACCCTAAAACGTTTGGTTAAAAGCAATGTGATATCATCAACCAA
TP50994_Hit	D	1	chr1	.	CAGCTGATTCCCAGAGTGCATGTGGTGGCTGAGGATCATCTTGAATAGTAACTTTTGCAAGCTC
TP50994_Query	D	1	chr1	.	CAGCTGATTCCCAGAGTGCATGTGGTGGCTGAGGATCATCTTGAATAATAACTTTTGCAAGCTC
TP51031_Hit	D+G	1	chr1	.	CAGCTGATTGGTGAAAGAGAGACACTGGAGTGTATAGCTGGGTTTTGTGTGGATGATATACGAA
TP51031_Query	D+G	1	chr1	.	CAGCTGATTGGTGAAAGAGAGACACTGAAGTGTATAGCTGGGTTTTGTGTGGATGATATACGAA
TP51160_Hit	D	1	chr1	.	CAGCTGGAAGAAATTCGACTTGGTGGGTCACAAAAATAACTGTTTTATCTGCTAGTTCCGACAA
TP51160_Query	D	1	chr1	.	CAGCTGGAAGAAATTCAACTTGGTGGGTCACAAAAATAACTGTTTTATCTGCTAGTTCCGACAA
TP51192_Hit	D	1	chr1	.	CAGCTGGAATATTCTTTTCAGGCCAGCTTTTATCAAAATTGAACTCAGTAACTTTTCCAACCAT
TP51192_Query	D	1	chr1	.	CAGCTGGAATATTCTTTTCAGGCCAGCTTTTATCAAAATTGAACTCAGTAACATTTCCAACCAT
TP51203_Hit	D	1	chr1	.	CAGCTGGAATGATTGCCGCATGGGTCTCTAAGCGCCTTCCTGATTATCAATCAGAAAAATATGC
TP51203_Query	D	1	chr1	.	CAGCTGGAATGATTGCCGCATGGGTCTCTAAGCGCCTTCCTGATTATCAAACAGAAAAATATGC
TP51220_Hit	D	1	chr1	.	CAGCTGGACAGGCTTTATCAGCCTTTGAGAATGAGCTACGCCCTATTGACCGATACGCCATTCG
TP51220_Query	D	1	chr1	.	CAGCTGGACAAGCTTTATCAGCCTTTGAGAATGAGCTACGCCCTATTGACCGATACGCCATTCG
TP5124_Hit	D	1	chr1	.	CAGCAAGAAGGGCATTGCCTCGTGTGATCGAAATATTGGCGAATGAGCACAAATGGGACAAATC
TP5124_Query	D	1	chr1	.	CAGCAAGAAGGGCATTGCCTCGTGTGATCGAAATATTGGCAAATGAGCACAAATGGGACAAATC
TP51266_Hit	D	1	chr1	.	CAGCTGGACTGTTTTCTATGCATGACAAAACAACCTGTGATTTTCAGATTTTTATTTTTCGAAA
TP51266_Query	D	1	chr1	.	CAGCTGGACTGTTTTCTATGCATGACAAAACAACCTGTGATTTTCAGATTTTTATTTTTCAAAA
TP51323_Hit	D	1	chr1	.	CAGCTGGAGGTTGAGCATTTTCATCAAGAAATGGCTCCACCCTATTGGCATCAAACCATATTGG
TP51323_Query	D	1	chr1	.	CAGCTGGAGGTTGAGCATTTTCATCAAGAAATGGCTCCACCCTATTGGCATCAAACCATACTGG
TP51476_Hit	D+G	1	chr1	.	CAGCTGGCCGGTTAGGTCCTGATCCCTCACCCGCCACTCATAGTCATGTTCCTCAATCCTACAG
TP51476_Query	D+G	1	chr1	.	CAGCTGGCCGGTTAGGTCCTGATCCCTCACCCGACACTCATAGTCATGTTCCTCAATCCTACAG
TP51564_Hit	D	1	chr1	.	CAGCTGGGAAGATATTGGTGGTCTTGAAAATGTTAAGCGAGAACTCCAGGAGGTGACTCAAGCT
TP51564_Query	D	1	chr1	.	CAGCTGGGAAGATATTGGTGGTCTTGAAAATGTTAAGCGAGAACTCCAGGAGGTAACTCAAGCT
TP51631_Hit	D+G	1	chr1	.	CAGCTGGGCTTACCGAAAAAACTGAAGCTGTATTGAAGGAGATTGAAGGTGAAAACTTGAAAGA
TP51631_Query	D+G	1	chr1	.	CAGCTGGGCTTACCGAAAAAACTGAAGCTATATTGAAGGAGATTGAAGGTGAAAACTTGAAAGA
TP51696_Hit	D	1	chr1	.	CAGCTGGGTTCTTAAAACACATGTTAGGAGTATTGTAAAACAGGTTTTAATTACACATTTTTGT
TP51696_Query	D	1	chr1	.	CAGCTGGGTTCTTAAAACACATGTTAGGAGTATTGTAAAACAGGTTTTAATTACACATGTTTGT
TP5172_Hit	D+G	1	chr1	.	CAGCAAGAATGTTCACACCAGGTGCTACTATATCAGGTTTTAGGATTTCAAGTGTGAGTAAACT
TP5172_Query	D+G	1	chr1	.	CAGCAAGAATGTTCACACCAGGTGCTACTATATCAGGTTTTAGAATTTCAAGTGTGAGTAAACT
TP51726_Hit	D+G	1	chr1	.	CAGCTGGTACCAATGATGAAATTCCTACTATATCCACTTGTTTGATTAATTTTGTTGCGCTGTC
TP51726_Query	D+G	1	chr1	.	CAGCTGGTACCAATGATGAAATTCCAACTATATCCACTTGTTTGATTAATTTTGTTGCGCTGTC
TP51828_Hit	D	1	chr1	.	CAGCTGGTCTTCTTTCATCTTTTACAGCTCCCAGATCAGATTCATGATGACCCAATGATTGAAA
TP51828_Query	D	1	chr1	.	CAGCTGGTCTTCGTTCATCTTTTACAGCTCCCAGATCAGATTCATGATGACCCAATGATTGAAA
TP52203_Hit	D	1	chr1	.	CAGCTGTAGGTTCTCAGATATAATGGGAGCTGGCAATGAAGACGAGGAGGGGGGGAAGGAGAGA
TP52203_Query	D	1	chr1	.	CAGCTGTAGGTTCTCAGATATAATGGGAGCTGGCAATGAAGACGAGGAGGAGGGGAAGGAGAGA
TP52251_Hit	D	1	chr1	.	CAGCTGTTTGGGGTGGAGCCAGTTGAAAGTCCTGTACTGTCCGGAGGAAAGCCTGGTTAGTTAT
TP52251_Query	D	1	chr1	.	CAGCTGTATGGGGTGGAGCCAGTTGAAAGTCCTGTACTGTCCGGAGGAAAGCCTGGTTAGTTAT
TP5230_Hit	D+G	1	chr1	.	CAGCAAGACGATGAGGAACATGCAGATACCGGTCTCGATAGTTACCGTTATTTGAAATCCTCTT
TP5230_Query	D+G	1	chr1	.	CAGCAAGACGATGAGGAACATACAGATACCGGTCTCGATAGTTACCGTTATTTGAAATCCTCTT
TP52350_Hit	D	1	chr1	.	CTGCTGTCCATCGCCAGCTGGAGGGGACTTCTGAAAGCAAATAATATCACCATCCTCTAGCTGA
TP52350_Query	D	1	chr1	.	CAGCTGTCCATCGCCAGCTGGAGGGGACTTCTGAAAGCAAATAATATCACCATCCTCTAGCTGA

Name	Filter	Nb hit (Mt4.0)	Mt Chr (Mt4.0)	Ms Chr (Li et al., 2014)	Sequence
TP52431_Hit	D	1	chr1	.	CAGCTGTCTTCTTTGGGTTCCATGCCCCCGGTTGGCATGTACCATAGGATTCTAAGTAGGTGCT
TP52431_Query	D	1	chr1	.	CAGCTGTCTTCTTTGGGTTCCATGCCCCCGGTTGGCATGTACCATAGGATTCTAAGTAAGTGCT
TP52545_Hit	D	1	chr1	.	CAGCTGTGCCTACTATTGAGCTTTTTTTGCAAAATGAAAATGTGGTTTGGAGGATATTTGGTGC
TP52545_Query	D	1	chr1	.	CAGCTGTGCCTACTATTGAGCTCTTTTTGCAAAATGAAAATGTGGTTTGGAGGATATTTGGTGC
TP52653_Hit	D	1	chr1	.	CAGCTGTGTAATACGTGGATGTGTGCCAAAGAAATTACTCGTCTATGCCTCAAAATTTTCTCAC
TP52653_Query	D	1	chr1	.	CAGCTGTGTAATACGTGGATGTGTGCCAAAGAAATTACTCGTCTATGCCTCAAAATCTTCTCAC
TP52686_Hit	D	1	chr1	.	CAGCTGTGTGTCCTTTAGCGCCATTGTCACCAAGAACTTGAATGGAAGGTTGATCATTCTTAAT
TP52686_Query	D	1	chr1	.	CAGCTGTGTGTCCTTTAGCGCCACTGTCACCAAGAACTTGAATGGAAGGTTGATCATTCTTAAT
TP5270_Hit	D	1	chr1	.	CAGCAAGAGACAAAATCAGTAAATGGCAAACAAGAGTTATGAGGTTGTATTGCATCAACTCAAA
TP5270_Query	D	1	chr1	.	CAGCAAGAGACAAAATCAGTAAATGGCAAACAAGAGTTATGAAGTTGTATTGCATCAACTCAAA
TP52770_Hit	D+G	1	chr1	.	CAGCTGTTACCAATCCTGATCGGTACGATATAACCTGCCATGGGCATAGTTAGCATTGCATACA
TP52770_Query	D+G	1	chr1	.	CAGCTGTTACCAATCCTGATCGGTACGATATAACCTGCCATGGGCAAAGTTAGCATTGCATACA
TP5281_Hit	D+G	1	chr1	.	CAGCAATAGAGATCTGTTAGGGAGAGAGAAAGAGCATGAATCATGTGTGGTTAGGAGTAAAGAG
TP5281_Query	D+G	1	chr1	.	CAGCAAGAGAGATCTGTTAGGGAGAGAGAAAGAGCATGAATCATGTGTGGTTAGGAGTAAAGAG
TP52836_Hit	D	1	chr1	.	CAGCTTTTCAAAATTAGTGAAGAGGTGGTAGTTGATGCAACTGATAAAGGAAATATTGCGCGGC
TP52836_Query	D	1	chr1	.	CAGCTGTTCAAAATTAGTGAAGAGGTGGTAGTTGATGCAACTGATAAAGGAAATATTGCGCGGC
TP52849_Hit	D+G	1	chr1	.	CAGCTGTTCACTGTTATAAGTTGTGACTTGTAACTAGAGTGGAATATTTATTCCTTGAGTGTCT
TP52849_Query	D+G	1	chr1	.	CAGCTGTTCACTGTTATAACTTGTGACTTGTAACTAGAGTGGAATATTTATTCCTTGAGTGTCT
TP52868_Hit	D+G	1	chr1	.	CAGCTGTTCCAGTCCCCGCACCGGCACCGGAGACAGGTCTAAAACACAAACTAGAGAGAAGATG
TP52868_Query	D+G	1	chr1	.	CAGCTGTTCCAGTCCCCGCACCGGCACCGGAGACAGGGCTAAAACACAAACTAGAGAGAAGATG
TP5294_Hit	D+G	1	chr1	.	CAGCAAGAGCAAACTGGCAAGCACCTTCACTGATGTTATAACGTTCAAATAACTGCATTGCCCA
TP5294_Query	D+G	1	chr1	.	CAGCAAGAGCAAACTGGCAAGCACCTTCACTAATGTTATAACGTTCAAATAACTGCATTGCCCA
TP52991_Hit	D+G	1	chr1	.	CAGCTGTTGGATACGGTACAGAAAATGGGAAAGATTATTGGATTGTGAGAAATTCATGGGGTGG
TP52991_Query	D+G	1	chr1	.	CAGCTGTTGGATACGGTACAGAAAACGGGAAAGATTATTGGATTGTGAGAAATTCATGGGGTGG
TP5303_Hit	D	1	chr1	.	CAGCAAGAGCATCAGCATAAGCATCAATACGAAGTCCACGATCAAGCAAACGATCCCGACTCTG
TP5303_Query	D	1	chr1	.	CAGCAAGAGCATCAGCATAAGCATCAATACGAAGTCCACGATCAAGCAAACGATCCCGACTCCG
TP53305_Hit	D	1	chr1	.	CAGCTTAACACAGTCGTGGAAAATCATGTTGGAGTCTCATGAAACTCAGAAGATGATTCTTTCC
TP53305_Query	D	1	chr1	.	CAGCTTAACACAATCGTGGAAAATCATGTTGGAGTCTCATGAAACTCAGAAGATGATTCTTTCC
TP53327_Hit	D	1	chr1	.	CAGCTTAACGGTATTCAGGGAATAGCCGGCCTGACTTTTAACGAATACCACAAGCTTGCAGACT
TP53327_Query	D	1	chr1	.	CAGCTTAACCGTATTCAGGGAATAGCCGGCCTGACTTTTAACGAATACCACAAGCTTGCAGACT
TP53394_Hit	D	1	chr1	.	CAGCTTAAGGACCTAAGGAGGGTTAATCAAGAACTGCTGGTCCAGACCGAGGAGTTGTTGCAGA
TP53394_Query	D	1	chr1	.	CAGCTTAAGGACCTAAGGAGGGTTAATCAAGAACTACTGGTCCAGACCGAGGAGTTGTTGCAGA
TP53416_Hit	D	1	chr1	.	CAGCTTAAGTTTTGGAAATGGGAAGTGATGATCCTTGTTCTAATATGTGGTAGATTGGTTTCTG
TP53416_Query	D	1	chr1	.	CAGCTTAAGTTTTGGAAATGGGAAGTGATGATTCTTGTTCTAATATGTGGTAGATTGGTTTCTG
TP53436_Hit	D	1	chr1	.	CAGCTTAATCAAAACTGCAATTTTGCAACATGTGTAACTAAGACTCAAAGTGGCATGTTGATTC
TP53436_Query	D	1	chr1	.	CAGCTTAATCAAAACTGCAATTTTGCAACATGTGTAACTAAGACTCAAAGTGGCATATTGATTC
TP53758_Hit	D	1	chr1	.	CAGCTTAGATCCGGACGTCTGTCGAGAGGTAATCTCTTTAACCCTCCTTTTTCAGTTTTTCGTA
TP53758_Query	D	1	chr1	.	CAGCTTAGATCCGGACGTCTGTCGAGAGGTAATCTCTTTAACCCTCCTTTTTCAGTTTTTCATA
TP53852_Hit	D+G	1	chr1	.	CAGCTTAGTTCCATTGGATAATGACATATTGATAAATTCAACTTTAGGCTATTATCAGTGAATA
TP53852_Query	D+G	1	chr1	.	CAGCTTAGTTCCATTGGACAATGACATATTGATAAATTCAACTTTAGGCTATTATCAGTGAATA
TP53857_Hit	D	1	chr1	.	CAGCTTAGTTTACTTCCTCGCTAAGGTGAAGAAGTTTCTTTGTAAGAAGGGAGCATTGTTTTTC
TP53857_Query	D	1	chr1	.	CAGCTTAGTTTACTTCCTCGCTAAGGTGAAGAAGTTTCTTTGTAAGAAGGGAGCATTGTTTTCC
TP54013_Hit	D+G	1	chr1	.	CAGCTTATATGAGGTTGGCTGAAGATAGGTTCTACAACAGGTTTCCGTGCATAATCGTGACGGC
TP54013_Query	D+G	1	chr1	.	CAGCTTATATGAGGTTGGCCGAAGATAGGTTCTACAACAGGTTTCCGTGCATAATCGTGACGGC
TP54105_Hit	D	1	chr1	.	CAGCTTATGAAATCTAAAGGATTGATCAACAAAGGAGATGTTGTTCTAGTGGTTTCAGATGTTG
TP54105_Query	D	1	chr1	.	CAGCTTATGAAATCTAAAGGATTGATCAACAAAGGAGATGTTGTTCTAGTGGTATCAGATGTTG
TP54126_Hit	D	1	chr1	.	CAGCTTATGACACTTTTCAGAAGTTTTTTTCAGCTTAATTTCATAAGTCTCCATGATAGCTTAC
TP54126_Query	D	1	chr1	.	CAGCTTATGACACTTTTCAGAAGTTTTTTTCAGCTTAATTTCATAAGTCTCCACGATAGCTTAC
TP54209_Hit	D	1	chr1	.	CAGCTTATGTTACATTGTTGAGTTTAATCAACGCGCAAATAGATGAGTATATGAAACCACTTTC
TP54209_Query	D	1	chr1	.	CAGCTTATGTTACATTGCTGAGTTTAATCAACGCGCAAATAGATGAGTATATGAAACCACTTTC
TP54487_Hit	D	1	chr1	.	CAGCTTCAACCATGGTTGGAAATGTTACTGAGTTCAATTTTGATAAAAGCTGGCCTGAAAAGAA
TP54487_Query	D	1	chr1	.	CAGCTTCAACCATGGTTGGAAAAGTTACTGAGTTCAATTTTGATAAAAGCTGGCCTGAAAAGAA
TP54502_Hit	D	1	chr1	.	CAGCTTCAACTCTGAAGAGAGATGAAATTCCTGTTTTATCCTTCATTTTCTCCATTGGCTACTA
TP54502_Query	D	1	chr1	.	CAGCTTCAACTCTGAAGAGAGATGAAATTCCTGTTTTATCCTCCATTTTCTCCATTGGCTACTA

Name	Filter	Nb hit (Mt4.0)	Mt Chr (Mt4.0)	Ms Chr (Li et al., 2014)	Sequence
TP54511_Hit	D+G	1	chr1	.	CAGCTTCAACTTCCGTAGTTATTAATGTTCTATTATGATTCTCTTGGCAATAGCATTTGCCTGT
TP54511_Query	D+G	1	chr1	.	CAGCTTCAACTTCCGTAGTTATTAATGTTCTATTATGATTATCTTGGCAATAGCATTTGCCTGT
TP5458_Hit	D+G	1	chr1	.	CAGCAAGATCCATAGAACCCATGTTGATGGAGGAATGAACTGAATTTCTTGTCATCATCTCATC
TP5458_Query	D+G	1	chr1	.	CAGCAAGATCCATAGAACCCATGTTGATGGAAGAATGAACTGAATTTCTTGTCATCATCTCATC
TP5459_Hit	D	1	chr1	.	CAGCAAGATCCCCTCCTGCTTTTATTGCAAATGGCTCATATACTGTGACCAGTTTTACTCTTGT
TP5459_Query	D	1	chr1	.	CAGCAAGATCCCCTCCTGCTTTTATTGCAAATGGCTCATATACTGTGACCAGTTTTACTCTCGT
TP54601_Hit	D+G	1	chr1	.	CAGCTTCAATCTCTGATGTTTTGCATGTGTATGCTTCCTCTCCTGTTCTTTTCACATGTTTCTC
TP54601_Query	D+G	1	chr1	.	CAGCTTCAATCTCTGATGTTTTGCATGTGTATGCTTCCTCCCCTGTTCTTTTCACATGTTTCTC
TP54809_Hit	D+G	1	chr1	.	CAGCTTCAGAGCAATACCATATCGAGACAAAGAACTCAAAGCTTTGATTTTGATGAGACATTAA
TP54809_Query	D+G	1	chr1	.	CAGCTTCAGAGCAATACAATATCGAGACAAAGAACTCAAAGCTTTGATTTTGATGAGACATTAA
TP54850_Hit	D	1	chr1	.	CAGCTTCAGCAGATGAATCAGTGGACTCTCCACTACCGTCATTTTTTGGTTTTGAGAAGAAAGG
TP54850_Query	D	1	chr1	.	CAGCTTCAGCAGATGAATCAGTGGACTCTCCACTACCATCATTTTTTGGTTTTGAGAAGAAAGG
TP55067_Hit	D	1	chr1	.	CAGCTTCATGAAGCCAAGATGCAACACCAGGAAAAGTACTTTTGAAGTTACTAATCCTTTCCGA
TP55067_Query	D	1	chr1	.	CAGCTTCATGAAGCCAAGATGCAACACCAGGAAAAGTACTTTTGAAGTTACTAATCCTTTCCAA
TP55073_Hit	D	1	chr1	.	CAGCTTCATGAGGCTAGATGCATTCCTAATGAAGATACCTATGCCCTTCTTATAAAGGCCTATT
TP55073_Query	D	1	chr1	.	CAGCTTCATGAGGCTAGATGCATTCCTAATGAAGATACCTATGCCCTTCTTATAAAGGCCTACT
TP55089_Hit	D	1	chr1	.	CAGCTTCATGGTGCCCAGAGAGACTCCATCGTCGTCGGTGGGGCTGTAGCTTCCCGCGGAAGCT
TP55089_Query	D	1	chr1	.	CAGCTTCATGGTGCCCAGAGAGACTCCATCGTCGTCGGTGGGGCTGTAGCTTCCCGCGGAAACT
TP55154_Hit	D	1	chr1	.	CAGCTTCATTTGTCCTCTTTTGAGGCAATGAATTATTTTGATAATCCTCTTGAATAACACCAGT
TP55154_Query	D	1	chr1	.	CAGCTTCATTTGTCCTCTTTTGAGGCAATGAATTATTTTGATAATCCTCTTGAATAACACAAGT
TP55159_Hit	D	1	chr1	.	CAGCTTCATTTTATCATTTGAGATGGTTGTTTTGATGTCAAACTTAGTAGTCTTACCTAAATTG
TP55159_Query	D	1	chr1	.	CAGCTTCATTTTATCATTTGAGATGGTTATTTTGATGTCAAACTTAGTAGTCTTACCTAAATTG
TP55223_Hit	D+G	1	chr1	.	CAGCTTCCACGCCCTCCACAACCACCTCAGGTACCTAGGCCACCTGTTCAAGCTTTACAACAGT
TP55223_Query	D+G	1	chr1	.	CAGCTTCCACGCCCTCCACAACCACCTCAGGTACCTAGGCCACCTGTTCAAGCCTTACAACAGT
TP55232_Hit	D	1	chr1	.	CAGCTTCCAGATAAACTCTATAGACTTGTTAGTCCAAATCTCAGTCATGAACGACCGACGATGA
TP55232_Query	D	1	chr1	.	CAGCTTCCAGATAAACTCTATAGACTTGTAAGTCCAAATCTCAGTCATGAACGACCGACGATGA
TP5531_Hit	D+G	1	chr1	.	CAGCAAGATTTTCCTCGATTAAACCTAGGGCTTGTTTGTTCTGTTCCACTAGCGCACCTGTGGT
TP5531_Query	D+G	1	chr1	.	CAGCAAGATTTTCCTCGATCAAACCTAGGGCTTGTTTGTTCTGTTCCACTAGCGCACCTGTGGT
TP55402_Hit	D+G	1	chr1	.	CAGCTTCCTACAATGGTTTCTATTCTTAGTGTCTGATGTGATCACTGGCACTGATTCGTCTGTG
TP55402_Query	D+G	1	chr1	.	CAGCTTCCTACAATGGTTTCTATTCTTAGTGTCTGATGTGATCACCGGCACTGATTCGTCTGTG
TP55652_Hit	D	1	chr1	.	CAGCTTCGTCAAATCAAAACTGGATCCCTCCTAGTAGTGGAACTGTCAAATTAAACTGTGATGG
TP55652_Query	D	1	chr1	.	CAGCTTCGTCAAATCAAAACTGGATCCCTCCTAGAAGTGGAACTGTCAAATTAAACTGTGATGG
TP55781_Hit	D	1	chr1	.	CAGCTTCTAGCACCATCTCTTATCATGATAATCGCTTATGTATAAGCTATTTCTACAACAAAAT
TP55781_Query	D	1	chr1	.	CAGCTTCTAGCACCATCTCTTATCATGATAATCGCTAATGTATAAGCTATTTCTACAACAAAAT
TP55782_Hit	D	1	chr1	.	CAGCTTCTAGCTATGAGCAGAGAACAACATCTTCAATTTCACCAAAAGCATATGCAATGGAAAT
TP55782_Query	D	1	chr1	.	CAGCTTCTAGCTATGAGCAAAGAACAACATCTTCAATTTCACCAAAAGCATATGCAATGGAAAT
TP55894_Hit	D	1	chr1	.	CAGCTTCTCCTAGCTCTGAAACTCTGACAAGAACTCTGGAAGAATCTGATCAGCTTGAGAAACC
TP55894_Query	D	1	chr1	.	CAGCTTCTCCTAGCTCTGAAACTCTGACAAGAACTCTGGAAGAATCTGATCAGATTGAGAAACC
TP5623_Hit	D	1	chr1	.	CAGCAAGCACGTTTGGATTCTTTCGAGTGATCAACCACGGTGTGGCACCGGATTTGCTACGAAG
TP5623_Query	D	1	chr1	.	CAGCAAGCACGTTTGGATTCTTTCGAGTGATCAACCACGGTGTGGCACCAGATTTGCTACGAAG
TP56331_Hit	D+G	1	chr1	.	CAGCTTTTGATGTAACACAAATATTATCATCCAAACTGTGAACTTCTTCGACTAAACAACCGTT
TP56331_Query	D+G	1	chr1	.	CAGCTTCTGATGTAACACAAATATTATCATCCAAACTGTGAACTTCTTCGACTAAACAACCGTT
TP56391_Hit	D	1	chr1	.	CAGCTTCTGCTTCTTCGTTGGTGGCATCGTGATCATTTTCAGAGAAGAATGATTGTTGTTGGCT
TP56391_Query	D	1	chr1	.	CAGCTTCTGCTTCTTCGGTGGTGGCATCGTGATCATTTTCAGAGAAGAATGATTGTTGTTGGCT
TP56481_Hit	D	1	chr1	.	CAGCTTCTTGAAGTGTGGTGGCTTGATATAGGTGGGATGGTAAATATCCTTCCCTTGTCCCCAA
TP56481_Query	D	1	chr1	.	CAGCTTCTTAAAGTGTGGTGGCTTGATATAGGTGGGATGGTAAATATCCTTCCCTTGTCCCCAA
TP56528_Hit	D	1	chr1	.	CAGCTTCTTCACTCTGTTCCTCCTTCACCTCCACAACTTCGCACTCGCAACATCGTCTTCACGA
TP56528_Query	D	1	chr1	.	CAGCTTCTTCACTCTGTTCCTCCTTCACCTCCACAACTTCGCACTCGCAACATCGTCTTCACCA
TP56680_Hit	D	1	chr1	.	CAGCTTCTTTGATCATTTTTCTTGAGTGTAGGAAGCAATTAATTGCACATCACAAATCAGAAGA
TP56680_Query	D	1	chr1	.	CAGCTTCTTTGATCATTTTTCTTGAATGTAGGAAGCAATTAATTGCACATCACAAATCAGAAGA
TP56843_Hit	D	1	chr1	.	CAGCTTGAAGTCGCACCCTTAGCACCTATTCCTTTCACATTTACACGACTCTCAGCTTCATTAT
TP56843_Query	D	1	chr1	.	CAGCTTGAACTCGCACCCTTAGCACCTATTCCTTTCACATTTACACGACTCTCAGCTTCATTAT
TP56961_Hit	D	1	chr1	.	CAGCTTGACACCACTAAAACTGACCCCTCCTAAGCTTGATTGTGAGAACATCGATAAATTCATG
TP56961_Query	D	1	chr1	.	CAGCTTGACACCACTAAAACTGACCCCTCCTAAGATTGATTGTGAGAACATCGATAAATTCATG

Name	Filter	Nb hit (Mt4.0)	Mt Chr (Mt4.0)	Ms Chr (Li et al., 2014)	Sequence
TP57170_Hit	D+G	1	chr1	.	CAGCTTGATATCCTTGTCGCGCAACATCAGGCAGGCTTTGAATTACCAGTGGATTCTGTTTCAT
TP57170_Query	D+G	1	chr1	.	CAGCTTGATATCCTTGTCGCACAACATCAGGCAGGCTTTGAATTACCAGTGGATTCTGTTTCAT
TP57342_Hit	D+G	1	chr1	.	CAGCTTGCACAGAGTTTTGATCACCATTTGAGTTGGGGGCCACCATATGCTGTTGGTGCTGAAA
TP57342_Query	D+G	1	chr1	.	CAGCTTGCACAGAGTTTTGATCACCATTTGAGTTGGGAGCCACCATATGCTGTTGGTGCTGAAA
TP57346_Hit	D+G	1	chr1	.	CAGCTTGCACTGATGTTTCTACCCCTTTAATGATTGTAGCTGGTCCGGGAAGTGGAAAGGTTTT
TP57346_Query	D+G	1	chr1	.	CAGCTTGCACCGATGTTTCTACCCCTTTAATGATTGTAGCTGGTCCGGGAAGTGGAAAGGTTTT
TP57486_Hit	D	1	chr1	.	CAGCTTGCGCATCGGCACACTTCTTGAAAACCACTCTTGCCCGCTTGCTTCCTCTATCAATTTC
TP57486_Query	D	1	chr1	.	CAGCTTGCGCATCAGCACACTTCTTGAAAACCACTCTTGCCCGCTTGCTTCCTCTATCAATTTC
TP5771_Hit	D	1	chr1	.	CAGCAAGCGCAGGAGTTGTCATGCTAATTCCAGTGGAAGCAGAAGTGGCATTAATGCCACCGCT
TP5771_Query	D	1	chr1	.	CAGCAAGCGCAGGAGTTGTCATGCTAATTCCAGTGGAAGCAGAAGTGGCATTAATGCCACCACT
TP58000_Hit	D	1	chr1	.	CAGCTTGTAGATACAACACCCCAAAACTTCCACCATTTTTCCCATTCCCACCTATATGTTCTTC
TP58000_Query	D	1	chr1	.	CAGCTTGTAGATACAACACCCCAAAACTTCCACCATTTTTCCCATTCCCACCCATATGTTCTTC
TP58020_Hit	D+G	1	chr1	.	CAGCTTGTATCAGAAAACGTGAAGGCAAACCAATTACATAGCACTTTACTGTCTGAAAAGCAAG
TP58020_Query	D+G	1	chr1	.	CAGCTTGTATCAGAAAACGTGAAGGCAAAACAATTACATAGCACTTTACTGTCTGAAAAGCAAG
TP5804_Hit	D	1	chr1	.	CAGCAAGCTAGTGAGCTAACCCGGGTTAGGCTTAGGGAAATGGAATTGCCGGATTCTATTCTTG
TP5804_Query	D	1	chr1	.	CAGCAAGCTAGTGAGCTAACCCGGGTTAAGCTTAGGGAAATGGAATTGCCGGATTCTATTCTTG
TP58160_Hit	D+G	1	chr1	.	CAGCTTGTGAGATTTTAAATTAGGAGATGGAGAAGAAAGTGTAAACCAAATGAGAAGAAGGAAT
TP58160_Query	D+G	1	chr1	.	CAGCTTGTGAGATTTTAAATTAGGAGATGGAGAAGAAAGGGTAAACCAAATGAGAAGAAGGAAT
TP58195_Hit	D+G	1	chr1	.	CAGCTTGTGCTGGACTAACAGTCATGTTCATTATTCTCTCAATTGGTCATATCTCAGGTGCACA
TP58195_Query	D+G	1	chr1	.	CAGCTTGTGCTGGACTAACAGTCATGTTCATTATTCTATCAATTGGTCATATCTCAGGTGCACA
TP58603_Hit	D+G	1	chr1	.	CAGCTTTACAGTCTTGGAGCATTGGATGACGAGGGTCTTCTAACCAAATTGGGTAGGAAAATGG
TP58603_Query	D+G	1	chr1	.	CAGCTTTACAGTCTTGGAGCACTGGATGACGAGGGTCTTCTAACCAAATTGGGTAGGAAAATGG
TP58900_Hit	D+G	1	chr1	.	CAGCTTTCAAATCAACCAAAGTTGAGAGCAATTAAGAAACAGTAAATTTACCAATTATCATACT
TP58900_Query	D+G	1	chr1	.	CAGCTTTCAAATCAACCAAAGTTGAGAGCAACTAAGAAACAGTAAATTTACCAATTATCATACT
TP58921_Hit	D	1	chr1	.	CAGCTTTCAACTAAGCTGAGAAATCGTTTCACAAAGCGGCAATGTACCAACCTTGCGCATTTGC
TP58921_Query	D	1	chr1	.	CAGCTTTCAACTAAGCTGAGAAATCGTTTCACAAAGCGGCAATGCACCAACCTTGCGCATTTGC
TP58963_Hit	D+G	1	chr1	.	CAGCTTTCACTGCATTCTTTGAAGAGTCAACGAGGCCTTTTGTGATAGTTCCAAACAAATTAGA
TP58963_Query	D+G	1	chr1	.	CAGCTTTCACCGCATTCTTTGAAGAGTCAACGAGGCCTTTTGTGATAGTTCCAAACAAATTAGA
TP58983_Hit	D	1	chr1	.	CAGCTTTCACTTTTAACTCCCTATGTTCAGTTACTTGGTATCCCCCATACTTGGGCCGCATACA
TP58983_Query	D	1	chr1	.	CAGCTTTCACTTTTAACTCCCTATGTTCAGTTACTTGGCATCCCCCATACTTGGGCCGCATACA
TP59073_Hit	D	1	chr1	.	CAGCTTTCCAGACATACAGTAGGCCTTTATAAGAAGGGCATAGGTATCTTCATTAGGAATGCAT
TP59073_Query	D	1	chr1	.	CAGCTTTCCAGACATACAATAGGCCTTTATAAGAAGGGCATAGGTATCTTCATTAGGAATGCAT
TP59189_Hit	D	1	chr1	.	CAGCTTTCTCCTATATCGCCATACTTGTCAAAGATAGATTGCAGGATGGGAGCAAAGCTTTCCT
TP59189_Query	D	1	chr1	.	CAGCTTTCTCCTATATCACCATACTTGTCAAAGATAGATTGCAGGATGGGAGCAAAGCTTTCCT
TP59243_Hit	D	1	chr1	.	CAGCTTTCTTAGAAAAGTTAAAAGCAGAAAGGGATATCCTCAAGATGCAAAGTCAAGAACAAAC
TP59243_Query	D	1	chr1	.	CAGCTTTCTTAGAAAAGTTAAAAGAAGAAAGGGATATCCTCAAGATGCAAAGTCAAGAACAAAC
TP59419_Hit	D	1	chr1	.	CAGCTTTGATGGCTGGAACGATTCTTCTGAACTCTAGTGAAGTCCTACAAGTTCAACAATGTAT
TP59419_Query	D	1	chr1	.	CAGCTTTGATGGCTGGAACGATTCTTCTGAACTCTAGTGAAGTCCTACAAGTTCAACAATATAT
TP5946_Hit	D	1	chr1	.	CAGCAAGGAAGATAGTATTCCTCTTCGATATGCTTCCGTTTTGATAACAAAGGGTTTCTTTTGT
TP5946_Query	D	1	chr1	.	CAGCAAGGAAGATAGCATTCCTCTTCGATATGCTTCCGTTTTGATAACAAAGGGTTTCTTTTGT
TP5957_Hit	D	1	chr1	.	CAGCAAGGAATCCCAGATGCAACAACTACCTCAGGTGATAAAAGAGTAGTTTTTTCGCCCAAAA
TP5957_Query	D	1	chr1	.	CAGCAAGGAATCCCAGATGCAACAACTACCTCAGGTGATAAAAGAGAAGTTTTTTCGCCCAAAA
TP59671_Hit	D	1	chr1	.	CAGCTTTGTAAACAAACCCATAACTTCCTCTCCCAAGCTCATTAAACTCCTTGAAACCGTTAGT
TP59671_Query	D	1	chr1	.	CAGCTTTGTAAACAAACCCATAACTTCCTCTCCCAAGCTCATTAAACTCCTTGAAACCATTAGT
TP59770_Hit	D	1	chr1	.	CAGCTTTGTTCTCGCATTCTCTTTGCTCATCGGCTGGATCACTGTCAGCCACAATTCCTGCTCC
TP59770_Query	D	1	chr1	.	CAGCTTTGTTCTCGCATTCTCTTTGCTCATCAGCTGGATCACTGTCAGCCACAATTCCTGCTCC
TP5986_Hit	D	1	chr1	.	CAGCAAGGAGAAAGCAGATAGTATATATTTAGTATCCAAAGCTTCACATTTTCCCTTTTGGTGT
TP5986_Query	D	1	chr1	.	CAGCAAGGAGAAAGCAGATAGTATATATTTAGTATCCAAAGCTTCACATTTTCCCTTTCGGTGT
TP59879_Hit	D+G	1	chr1	.	CAGCTTTTAGCTGAGTAGTGATCACAAAAGTCAGTTGCTTAAAAGCCTACATCAAACAGGAAGC
TP59879_Query	D+G	1	chr1	.	CAGCTTTTAGCTGAGTAATGATCACAAAAGTCAGTTGCTTAAAAGCCTACATCAAACAGGAAGC
TP59881_Hit	D	1	chr1	.	CAGCTTTTAGGAAAACTTAATGAAATGCTAACTTAACCAATAGTCCAAGTAATCTAGGTGACTT
TP59881_Query	D	1	chr1	.	CAGCTTTTAGGAAAACTTAAGGAAATGCTAACTTAACCAATAGTCCAAGTAATCTAGGTGACTT
TP59884_Hit	D	1	chr1	.	CAGCTTTTAGGGGTCATGAAGATCAACATTAGCCATTCATCAAATCCAAAGCCAAATCGGCGTA
TP59884_Query	D	1	chr1	.	CAGCTTTTAGGGGTCATGAAGATCAACATTAGCCATACATCAAATCCAAAGCCAAATCGGCGTA

Name	Filter	Nb hit (Mt4.0)	Mt Chr (Mt4.0)	Ms Chr (Li et al., 2014)	Sequence
TP59897_Hit	D	1	chr1	.	CAGCTTTTATATTGGGGACAGGGAAGATCTTACTCAAGGTAAGTCAACACTTTTCATCATTTAA
TP59897_Query	D	1	chr1	.	CAGCTTTTATATTGGGGACAGGGAAGATCTTACTCAAGGTAAGTCAACACATTTCATCATTTAA
TP59944_Hit	D	1	chr1	.	CAGCTTTTCACAATCAGAGGCAAGGTACACTTCAAAAACAAAAGTCAGCGGCTGGCTTCTGCTT
TP59944_Query	D	1	chr1	.	CAGCTTTTCACAATCAGAGGCAAAGTACACTTCAAAAACAAAAGTCAGCGGCTGGCTTCTGCTT
TP60283_Hit	D+G	1	chr1	.	CAGCTTTTTGAAATTATGCAAAATGAATATCAATTGGAGCCCTGGCCAGAGCATTATGCTTGCC
TP60283_Query	D+G	1	chr1	.	CAGCTTTTTAAAATTATGCAAAATGAATATCAATTGGAGCCCTGGCCAGAGCATTATGCTTGCC
TP60327_Hit	D+G	1	chr1	.	CAGCTTTTTCATAGAAAGTCAGGGCCATGTCATATTGCATCATTTCAGAATAGACCACGCCAAG
TP60327_Query	D+G	1	chr1	.	CAGCTTTTTCATAGAAAGTCAGGGCCATGTCATATTGCATCATTTCAGAATAGACCACACCAAG
TP6059_Hit	D	1	chr1	.	CAGCAAGGCAGTCCTCAACCCGTGGCTTTGACTCACAAACCTTCCAAATTCTCTCCACTTCATC
TP6059_Query	D	1	chr1	.	CAGCAAGGCAGTCCTCAACCCGTGCCTTTGACTCACAAACCTTCCAAATTCTCTCCACTTCATC
TP60605_Hit	D+G	1	chr1	.	CTGCTAAAATCTTGTTGGAAGAGATGCAAAGTAAGGGAGTTGACCTAAATCTAGTGATATTTAA
TP60605_Query	D+G	1	chr1	.	CTGCAAAAATCTTGTTGGAAGAGATGCAAAGTAAGGGAGTTGACCTAAATCTAGTGATATTTAA
TP60852_Hit	D	1	chr1	.	CTGCAAAATAGCACAAAGCTCAAGTTCCAAAATATTTCTTACTCAAGAATATCAAGTGCAAACG
TP60852_Query	D	1	chr1	.	CTGCAAAATAGCACAAAGCACAAGTTCCAAAATATTTCTTACTCAAGAATATCAAGTGCAAACG
TP60947_Hit	D+G	1	chr1	.	CTGCAAAATTCATGGTTTTGGCGAGCACAAAAGTTCCCTCGTCATCTCTGACACACATGCCGAT
TP60947_Query	D+G	1	chr1	.	CTGCAAAATTCATGGCTTTGGCGAGCACAAAAGTTCCCTCGTCATCTCTGACACACATGCCGAT
TP60949_Hit	D	1	chr1	.	CTGCAAAATTCCAGAAAAGTAACACAAAACTGATTAGCAAATTGAAATTGCAGGAAGAAAGGAT
TP60949_Query	D	1	chr1	.	CTGCAAAATTCCAGAAAAGTAACAAAAAACTGATTAGCAAATTGAAATTGCAGGAAGAAAGGAT
TP60973_Hit	D	1	chr1	.	CTGCAAAATTTTCAGAAGCTTGAGGCCTTGTAAAAAAAATGACTATTTAATATTACCACTCTTG
TP60973_Query	D	1	chr1	.	CTGCAAAATTTTCAGAAGCTTGAGGACTTGTAAAAAAAATGACTATTTAATATTACCACTCTTG
TP61041_Hit	D+G	1	chr1	.	CTGCAAACTGTTACTCTCAAGATCTTGAATCTCTTAGGACACAGGAACATAATTTAAAAGAACA
TP61041_Query	D+G	1	chr1	.	CTGCAAACAGTTACTCTCAAGATCTTGAATCTCTTAGGACACAGGAACATAATTTAAAAGAACA
TP61101_Hit	D	1	chr1	.	CTGCAAACCTGAAGAAAAAATCTCAAAATCATTTGCAACAAAGATAATAGGTTCAATTAGGTCC
TP61101_Query	D	1	chr1	.	CTGCAAACCTGAAGAAAAAATCTCAAAATCATTTGCAACAAAGATAACAGGTTCAATTAGGTCC
TP61198_Hit	D+G	1	chr1	.	CTGCAAACTTTAATATTGTGGCAACATATAGATGATTCAACGCAATTACGGTTACTATGTCATT
TP61198_Query	D+G	1	chr1	.	CTGCAAACTTTAATATTGTGGCAACATATAGATGATTCAACACAATTACGGTTACTATGTCATT
TP61319_Hit	D	1	chr1	.	CTGCATAGCCTTTCTTGACTCCTTATTCAGAAGAGAAGAAGTGTCTACAACCAAGTCCCAGCTC
TP61319_Query	D	1	chr1	.	CTGCAAAGCCTTTCTTGACTCCTTATTCAGAAGAGAAGAAGTGTCTACAACCAAGTCCCAGCTC
TP61444_Hit	D	1	chr1	.	CTGCAAAGTTTGGGCAAAGGCTTGGTACTTGCGAATGTCAGCGTCACTAACACTTCTTCGTGCA
TP61444_Query	D	1	chr1	.	CTGCAAAGTTTGGGCAAAGGCTTGATACTTGCGAATGTCAGCGTCACTAACACTTCTTCGTGCA
TP61522_Hit	D+G	1	chr1	.	CTGCAAATAGGATGTCTTATATTGTAGAGGGTGATGTTGAGCCACTAATCACACTAGCTAAAAC
TP61522_Query	D+G	1	chr1	.	CTGCAAATAGGATGTCTTATATTGTAGAGGGTGATGTTGAGCCACTAATCAAACTAGCTAAAAC
TP61532_Hit	D	1	chr1	.	CTGCAAATATATTGTTTATCTAACATTGCAATCATATATAATACCTTAGAAGCCATGACCCATC
TP61532_Query	D	1	chr1	.	CTGCAAATATATTGTTTATCCAACATTGCAATCATATATAATACCTTAGAAGCCATGACCCATC
TP61573_Hit	D	1	chr1	.	CTGCAAATCATAGCATTTTCAACTGTGTGAGTGACCGTGCAGTGACTCTAAATGGAATAGCTAA
TP61573_Query	D	1	chr1	.	CTGCAAATCATAGCATCTTCAACTGTGTGAGTGACCGTGCAGTGACTCTAAATGGAATAGCTAA
TP61700_Hit	D	1	chr1	.	CTGCGAATGCTATTATTGATTGTTTGTGTCAGCATTTAGGTGTTATTGGGGAGAGTGGTGAGTA
TP61700_Query	D	1	chr1	.	CTGCAAATGCTATTATTGATTGTTTGTGTCAGCATTTAGGTGTTATTGGGGAGAGTGGTGAGTA
TP61710_Hit	D	1	chr1	.	CTGCAAATGCTTCAACATCTTTAGCCACAACCCTCCCTGAAGGTCCAGTTCCAATAATTTGTCC
TP61710_Query	D	1	chr1	.	CTGCAAATGCTTCAACATCTTTAGCCACAACCCTCCCTGAAGGTCCAGTCCCAATAATTTGTCC
TP61754_Hit	D	1	chr1	.	CTGCAAATGTTGCTATAGCTACGGCATCTTCATACAAAGGCACGTCATTAATTCCAAATGATAA
TP61754_Query	D	1	chr1	.	CTGCAAATGTTGCTATAGCTACGGCATCTTCATACAAAGGCACATCATTAATTCCAAATGATAA
TP61826_Hit	D+G	1	chr1	.	CTGCAAATTCGCTTCGAAGCCAATTCATAAACCCGTCTCTCTCGTCTACAAACCATTGGTGCTG
TP61826_Query	D+G	1	chr1	.	CTGCAAATTCGCTTCGAAGCCAATTCATAAACCCATCTCTCTCGTCTACAAACCATTGGTGCTG
TP6212_Hit	D+G	1	chr1	.	CAGCAAGGTCTGCTTTTACAATTTCTGTACTAAGATGCAACTGTACCCCTGCAATCATTGGTTC
TP6212_Query	D+G	1	chr1	.	CAGCAAGGTCTGCTTTTACAATTTCGGTACTAAGATGCAACTGTACCCCTGCAATCATTGGTTC
TP62187_Hit	D	1	chr1	.	CTGCAACAGGGTGTAGGAAGTTGGAGGGGACAGTGAAGAATGTGTTTGCAACCGGAAAAGTGAC
TP62187_Query	D	1	chr1	.	CTGCAACAGGGTGTAGGAAGTTGGAAGGGACAGTGAAGAATGTGTTTGCAACCGGAAAAGTGAC
TP62192_Hit	D+G	1	chr1	.	CTGCAACAGGTATATGTGATTGTGATTTATGATACAAACAAAATTTCAAGGATCATTCATTTTC
TP62192_Query	D+G	1	chr1	.	CTGCAACAGGTATATGTGATTGTGATTTATGATAAAAACAAAATTTCAAGGATCATTCATTTTC
TP62283_Hit	D	1	chr1	.	CTGCAACATCTGGGCAGGCTTTCCCCCAGTGTGTCTTTGATCATTGGGATACCATGACCTCAGA
TP62283_Query	D	1	chr1	.	CTGCAACATCTGGGCAGGCTTTCCCCCAGTGTGTCTTTGATCATTGGGACACCATGACCTCAGA
TP62361_Hit	D	1	chr1	.	CTGCAACCACATTCATTCTGATATACACAAAAATGTGGCTTCATCATTCCCCCACTATGGGCAT
TP62361_Query	D	1	chr1	.	CTGCAACCACATTCATTCTGATATACACAAAAATGTGGCTTCATCATTCCCCCACTATGGACAT

Name	Filter	Nb hit (Mt4.0)	Mt Chr (Mt4.0)	Ms Chr (Li et al., 2014)	Sequence
TP62452_Hit	D	1	chr1	.	CTGCAACCTACCCCACAACCAAACACGTGCTTAAACTAATAAGTTTTTTGGGACACGTGTAAAA
TP62452_Query	D	1	chr1	.	CTGCAACCTACCCCACAACCAAACACGTGCTTAAACTAATAAGTTTTTTGAGACACGTGTAAAA
TP62670_Hit	D	1	chr1	.	CTGCAACTATGATCTCTGGAAGATCAACGGCTGAGGAACTTGCAGGAATCAGGCCATAAGCTGA
TP62670_Query	D	1	chr1	.	CTGCAACTATGATCTCTGGAAGATCAACGGCTGAGGAACTTGCAGGAATCAGGCCATAAGCAGA
TP62893_Hit	D+G	1	chr1	.	CTGCAACTTCTAAGCAAAACGCAGATCCCGTTTTGGTTCCACCGGCTTACAGGACCACCGCTTA
TP62893_Query	D+G	1	chr1	.	CTGCAACTTCGAAGCAAAACGCAGATCCCGTTTTGGTTCCACCGGCTTACAGGACCACCGCTTA
TP62971_Hit	D	1	chr1	.	CTGCAAGAAACTGAGGAGAAAGTATGTGAGCTCATTCGACAGTTTTCTGATCAAGGTAGAACAA
TP62971_Query	D	1	chr1	.	CTGCAAGAAACTGAGGAGAAAGTATGTGAGCTCATTCGACAGTTTTATGATCAAGGTAGAACAA
TP63073_Hit	D+G	1	chr1	.	CTGCAAGACATGTAGTAGTACTGTAGTAATTAAGTCATAAGAATAACACCTTTGCCCAAGCATT
TP63073_Query	D+G	1	chr1	.	CTGCAAGACATGTAGTAGTACTGTAGTAATTAAGTCATAAGAATAAAACCTTTGCCCAAGCATT
TP63089_Hit	D+G	1	chr1	.	CTGCAAGACTGCAGAAGAATGGTCGTGCAAAACAATCCAGAAGATAGAGGTTCCTCTTCAAGAA
TP63089_Query	D+G	1	chr1	.	CTGCAAGACTGCAGAAGAATGGTCGTGCAAAACAATCCAGAAGATAGAGATTCCTCTTCAAGAA
TP63170_Hit	D	1	chr1	.	CTGCAAGAGTTAGAGCTTAAAAAGTCAGCAAAAAAGACTCAAGAAGCTATGGTTCTGGCTACTG
TP63170_Query	D	1	chr1	.	CTGCAAGAGTTAGAGCTTAAAAAGTCAGCAAAAAAGACTCAAGAAGCTATGGTACTGGCTACTG
TP63204_Hit	D+G	1	chr1	.	CTGCAAGATTACCTGCGTTGTTGAGCGAGGGTGCTTTCAAGATTGGAAACTGATGATGATTATA
TP63204_Query	D+G	1	chr1	.	CTGCAAGATTACCTGCGTTGTTGAACGAGGGTGCTTTCAAGATTGGAAACTGATGATGATTATA
TP6329_Hit	D	1	chr1	.	CAGCAAGTACTGATTGTATGTCTCTGTACCCAGATCAAGTGAAACAATAACAAATTGTTCAACG
TP6329_Query	D	1	chr1	.	CAGCAAGTACTGATTGTATGTCTCTGTACCCAGATCAAGGGAAACAATAACAAATTGTTCAACG
TP63331_Hit	D+G	1	chr1	.	CTGCAAGCTCTGGCTTAAATTTGTGGATCCTTCTTCACGTGTGTTTCGAAAATCATTCAAATTA
TP63331_Query	D+G	1	chr1	.	CTGCAAGCTCTGGCTTAAATTTGTGGATCCTTCTTCACGTGCGTTTCGAAAATCATTCAAATTA
TP63838_Hit	D	1	chr1	.	CTGCAATACTTCTCCACGCTTAAGATATCGCATATTGGAGTCTCCATATGCTAGAATCTCCTTT
TP63838_Query	D	1	chr1	.	CTGCAATACTTCTCCACGCTTAAGATATCGCATATTGGAGTCTCCATATGCTAGAATCTCCTCT
TP64074_Hit	D+G	1	chr1	.	CTGCAATCATCTCAACAAGTTTGGGTGCACGTGAGAGGCCATACTTACGGCACGCTGAAAAAAA
TP64074_Query	D+G	1	chr1	.	CTGCAATCATCTCAACAAGTTTGGGTGCACGTGAGAGGCCATACTTACGGCACGCAGAAAAAAA
TP64107_Hit	D	1	chr1	.	CTGCAATCCTAGCACTCTCATTTTCCTCTCGATCAAACCATGCCATTTCATTTTTGAGTATCGC
TP64107_Query	D	1	chr1	.	CTGCAATCCTAGCACTCTCATTTTCCTCTCGATCAAACCATGCCATTTCATTTTTGAGTACCGC
TP6417_Hit	D	1	chr1	.	CAGCAAGTCTCCAAAATCTTGACTACATTTGTTACCCACTATTTTTGTTCTTTTTTTAGGTTAC
TP6417_Query	D	1	chr1	.	CAGCAAGTCTCCAAAATCTTGACTACATTTGTTACCCACTATTTTTGTTCTGTTTTTAGGTTAC
TP64229_Hit	D	1	chr1	.	CTGCAATGAAGGCAGGCCAAAGTTGCCACAATTAAGCTCTGTAGTCTATAGAGGCCGCGTTGTA
TP64229_Query	D	1	chr1	.	CTGCAATGAAGGCAGGCCAAAGTTGCCACAATTAAGCTCTGTAGTCTATAGAGGCCGCATTGTA
TP64342_Hit	D	1	chr1	.	CTGCAATGCTAAGTTGTGGTGGTAGGGAGGTAAGGAACAAAAGCCAAGCCACCCACTTAAAGCT
TP64342_Query	D	1	chr1	.	CTGCAATGCTAAGTTGTGGTGGTAGGGAGGTAAGGAACAAAAGCCAAGCCACCCACTTAAAGCA
TP64377_Hit	D	1	chr1	.	CTGCAATGGAGTACATGGAGCAATTCGATAGAGATGCTGATGGTCTTATTGAGAATGATGGTTT
TP64377_Query	D	1	chr1	.	CTGCAATGGAGTACATGGAGCAATTCGATAGAGATGCTGATGGTCTTATTGAGAATGACGGTTT
TP64510_Hit	D	1	chr1	.	CTGCAATGTGGACTTTTTTTTTCTTTAGTAACTCCAAGTGATTTGTTAAATGAGTTTAAAGGTG
TP64510_Query	D	1	chr1	.	CTGCAATGTGGACATTTTTTTTCTTTAGTAACTCCAAGTGATTTGTTAAATGAGTTTAAAGGTG
TP64550_Hit	D	1	chr1	.	CTGCAATGTTTTTGCCATCTGTTTGCAGTGATGCAATCCTGCGTTCTGCTATTGCTGGTCTGTG
TP64550_Query	D	1	chr1	.	CTGCAATGTTTTTGCCATCTGTTTGCAGGGATGCAATCCTGCGTTCTGCTATTGCTGGTCTGTG
TP64710_Hit	D	1	chr1	.	CTGCAATTGAAGAAATTTAATTTTCACACATAGCCATATATGGCAATAATGCAGTATAATCATC
TP64710_Query	D	1	chr1	.	CTGCAATTGAAGAAATTTAATTTTCACACATAGCCATATATGGAAATAATGCAGTATAATCATC
TP64970_Hit	D	1	chr1	.	CTGCAATTTTTTCAACATTCTTCACACCTTCTTCAGATTCAACTTGAACCATTACCAAAATCTC
TP64970_Query	D	1	chr1	.	CTGCAATTTTTTCAACATTCTTCACACCTTCTTCAGATTCAACTTGAACCATTACCAAAAGCTC
TP65080_Hit	D+G	1	chr1	.	CTGCACAACACTAAAGGTGGTAAAACAGTCCATAGAAGTGTGGCATATATTTATGCTGTGTTGG
TP65080_Query	D+G	1	chr1	.	CTGCACAACACTAAAGGTGGTAAAACAGTCCATAGAAGTGTGCCATATATTTATGCTGTGTTGG
TP6514_Hit	D+G	1	chr1	.	CAGCAAGTGTTCGGACAGATGCTCTAAAGCAGGGGTTCAAGATAGGTGTGTGAAATACTGTGAA
TP6514_Query	D+G	1	chr1	.	CAGCAAGTGTTCGGACAGATGCTCTAAAGCAGGCGTTCAAGATAGGTGTGTGAAATACTGTGAA
TP65156_Hit	D	1	chr1	.	CTGCACAAGCCGGATTTGCAAAATCTACAAAACAAAGGATTAAAGGATCTCCACCTCGCTGAAA
TP65156_Query	D	1	chr1	.	CTGCACAAGCCGGATTCGCAAAATCTACAAAACAAAGGATTAAAGGATCTCCACCTCGCTGAAA
TP65234_Hit	D	1	chr1	.	CTGCACAATGAGTTCTGATTCAGCATCCAGTGCACTGGTGGCCTCATCTAAAAGCAATATTTTT
TP65234_Query	D	1	chr1	.	CTGCACAATGAGTTCTGATTCAGCATCCAGTGCACTGGTGGCCTCATCTAAAAGCAATATTTTC
TP65410_Hit	D	1	chr1	.	CTGCACAGAGTAATAGAGTTTCAGTTTTCTTACATGGTGTTGTGTTTTTGTTTTTGTTCCACGT
TP65410_Query	D	1	chr1	.	CTGCACAGAGTAATAGAGTTTCAATTTTCTTACATGGTGTTGTGTTTTTGTTTTTGTTCCACGT
TP65457_Hit	D+G	1	chr1	.	CTGCACAGGCACAGGATCATATGGGAGGAACAGTTAACCAAGGCTTTAATTCCTATCCCACTAA
TP65457_Query	D+G	1	chr1	.	CTGCACAGGCACAGGATCATATGGGAGGAACAGTTAACCAAGGCTATAATTCCTATCCCACTAA

Name	Filter	Nb hit (Mt4.0)	Mt Chr (Mt4.0)	Ms Chr (Li et al., 2014)	Sequence
TP65650_Hit	D	1	chr1	.	CTGCACATGGGAGTAAAATTGTGGAAATACTTATTTTTTATGAGTCAGATACTTAATACTTTTA
TP65650_Query	D	1	chr1	.	CTGCACATGGGAGTAAAATTGTGGAAATACTTATTTTTTACGAGTCAGATACTTAATACTTTTA
TP65904_Hit	D	1	chr1	.	CTGCACCAGCTGTTACGGAGGACTTCAATTCAAAACTTTCATCGTTGCAAATAGGTAGGCTGAA
TP65904_Query	D	1	chr1	.	CTGCACCAGCTGTTACGGAGGACTTCAATTCAAAACTTTCAACGTTGCAAATAGGTAGGCTGAA
TP6597_Hit	D	1	chr1	.	CAGCAATTTGAAATTACATGTTAAGAACGGATTTATTATTTTGACGAATTTCAATTGTAGCATA
TP6597_Query	D	1	chr1	.	CAGCAAGTTGAAATTACATGTTAAGAACGGATTTATTATTTTGACGAATTTCAATTGTAGCATA
TP66145_Hit	D	1	chr1	.	CTGCACCGGTGCGAGATGGAAGAAACTCACAACAACGATGCTCCTCCAAATAACAAACCTTCCA
TP66145_Query	D	1	chr1	.	CTGCACCGGTGCAAGATGGAAGAAACTCACAACAACGATGCTCCTCCAAATAACAAACCTTCCA
TP6639_Hit	D+G	1	chr1	.	CAGCAAGTTTATGGACCTGGTTATTATCTTTTGAATATTAACTATCTATCTGTGTGTTTATTCC
TP6639_Query	D+G	1	chr1	.	CAGCAAGTTTATGGACCTGGTTATTATCTTTTGAATATTAACTATCTATCTGTGAGTTTATTCC
TP66407_Hit	D+G	1	chr1	.	CTGCACGAAATACAAATTGAGATTTTTGTGGTGTCTTGTGTGTGATTGATTGTTTTTGTTTGGT
TP66407_Query	D+G	1	chr1	.	CTGCACGAAATACAAATTGAGATTTTTGTGGTGTCTTATGTGTGATTGATTGTTTTTGTTTGGT
TP66529_Hit	D+G	1	chr1	.	CTGCACGTGTTGTCTTGGAACCACGACCATTGGTGGAACCAGTCTCCTGAAGGTCTGGCTCCGT
TP66529_Query	D+G	1	chr1	.	CTGCACGCGTTGTCTTGGAACCACGACCATTGGTGGAACCAGTCTCCTGAAGGTCTGGCTCCGT
TP66702_Hit	D+G	1	chr1	.	CTGCACTAAGTGAAGCTGGTAGAAAACAGATCGACGTTGCCAAATTGCACGGTGCTGAAAAAAA
TP66702_Query	D+G	1	chr1	.	CTGCACTAAGTGAAGCCGGTAGAAAACAGATCGACGTTGCCAAATTGCACGGTGCTGAAAAAAA
TP67067_Hit	D	1	chr1	.	CTGCACTGTCATTGATGTCCCTGGACACAAAAATTTCATTAAGAACATGATCACTGGGACGTCC
TP67067_Query	D	1	chr1	.	CTGCACTGTCATTGATGTCCCTGGACACAAAAAATTCATTAAGAACATGATCACTGGGACGTCC
TP671_Hit	D	1	chr1	.	CAGCAAAAGCTCTTGTTGAGATGCATAATTGGAAGGTATTGTTAGAGAATGACCAGGTATGATA
TP671_Query	D	1	chr1	.	CAGCAAAAGCTCTTGTTGAGATGCATAATTGGAAGGTATTGTTAGAAAATGACCAGGTATGATA
TP67477_Hit	D+G	1	chr1	.	CTGCAGAAAATGGTGTTAAGGATGTTTTCAGCTTCTTGTTAAGGTTATGTGATTTGGAGATACT
TP67477_Query	D+G	1	chr1	.	CTGCAGAAAATGGTGTTAACGATGTTTTCAGCTTCTTGTTAAGGTTATGTGATTTGGAGATACT
TP67542_Hit	D	1	chr1	.	CTGCAGAACACCCTCATTTGAGTATTCCAGAAACTGAGTTTGTGCGGAAAAGAAAGCAACACCA
TP67542_Query	D	1	chr1	.	CTGCAGAACACCCTCATTTGAGTATTCCAGAAACTGAGTTTGTGAGGAAAAGAAAGCAACACCA
TP67843_Hit	D	1	chr1	.	CTGCTGACTGAGCTATGGCACCCAACCCACCAGGCTCAGTATGATTCTTTCCGGTAGCTCTCAT
TP67843_Query	D	1	chr1	.	CTGCAGACTGAGCTATGGCACCCAACCCACCAGGCTCAGTATGATTCTTTCCGGTAGCTCTCAT
TP6827_Hit	D	1	chr1	.	CAGCAATAATAAGTTATCAACATTTGATTCAAAATAAACGCAAATCACCACGATGAAATAAAAA
TP6827_Query	D	1	chr1	.	CAGCAATAATAAGTTATCAACATTTGATTCAAAATAAACGCAAACCACCACGATGAAATAAAAA
TP68847_Hit	D	1	chr1	.	CTGCAGATTCTATGAAGGGTAGTCACCTAGATGAGGTGAAGCGTATGGTTGAGGAATACCGGAA
TP68847_Query	D	1	chr1	.	CTGCAGATTCTATGAAGGGTAGTCACCTAGATGAGGTGAAGCGTATGGTAGAGGAATACCGGAA
TP68993_Hit	D	1	chr1	.	CTGCAGGAATATTTCCGGACAACTGATTGTCTGAGAGTTTTAGAATCTCCAAGTGCTGAAGTGT
TP68993_Query	D	1	chr1	.	CTGCAGGAATATTTCCAGACAACTGATTGTCTGAGAGTTTTAGAATCTCCAAGTGCTGAAGTGT
TP69144_Hit	D	1	chr1	.	CTGCAGGCATAAATGCTCTTTGTGGCGTTGGAATCCTTTCGGTCCCTTATGCTCTCAAAGAAGG
TP69144_Query	D	1	chr1	.	CTGCAGGCATAAATGCTCTTTGTGGCGTTGGAATCCTTTCAGTCCCTTATGCTCTCAAAGAAGG
TP69147_Hit	D+G	1	chr1	.	CTGCAGGCATACGCCAACTTCTGAAACAGTTGGGAAAGTCGCTAAAGAAAAAGGAAAAGCAGAC
TP69147_Query	D+G	1	chr1	.	CTGCAGGCATACGCCAACTTCTGAAACAGTTGGGAAAGTCGCTAAAGAAAAAAGAAAAGCAGAC
TP6951_Hit	D	1	chr1	.	CAGCAATAGAAGAGGTTAACCCAACACGTTTGAGTGATGGGGATTTTGCACATGAGTTGAGAAC
TP6951_Query	D	1	chr1	.	CAGCAATAGAAGAGGTTAACACAACACGTTTGAGTGATGGGGATTTTGCACATGAGTTGAGAAC
TP69823_Hit	D	1	chr1	.	CTGCAGTGCAATGTGTTTGAAAGTTGTGGATGTTTGTGGGCATACTTGGCTGATAGGCGGGCAT
TP69823_Query	D	1	chr1	.	CTGCAGTGCAATGTGTTTGAAAGTTGTGGATGTTTGTGGGCATACTTGGCTGATAAGCGGGCAT
TP69845_Hit	D	1	chr1	.	CTGCAGTGCTCCCTAATCTCACCTTGAGAACCTGTCAACACATGTACGTTACCCATATGCACCA
TP69845_Query	D	1	chr1	.	CTGCAGTGCTCCCTAATCTCACCTTGAGAACCTGTCAACACATCTACGTTACCCATATGCACCA
TP69889_Hit	D+G	1	chr1	.	CTGCAGTGGTGCAAGGAGTGATTGCATTGATGGGACAAAGTACTGAGCAGTTGATGGAAGATTT
TP69889_Query	D+G	1	chr1	.	CTGCAGTGGTGCAAGGAGTGATTGCATTGATGGGACAAAGTACTGAACAGTTGATGGAAGATTT
TP69971_Hit	D	1	chr1	.	CTGCAGTTAGAGGGTTGGCCCCTGTTGATATCCCATTGCCAGTCAAATTTCCGCTTCCAGATCC
TP69971_Query	D	1	chr1	.	CTGCAGTTAGAGGGTTGGCCCCTGTTGATAGCCCATTGCCAGTCAAATTTCCGCTTCCAGATCC
TP70149_Hit	D	1	chr1	.	CTGCAGTTGTTGATGACATTACTGTCGTTTTTGTGGTTGCCTTCGACTTTGCTTGCCGTCGCTG
TP70149_Query	D	1	chr1	.	CTGCAGTTGTTGATGACATTACTGTCGTTTTTGTGGTTGCCTTCAACTTTGCTTGCCGTCGCTG
TP7018_Hit	D	1	chr1	.	CAGCAATAGCTGGACTGATTTCAGGCCTACGATGCCATTGAGTAGACATAGTTGACGCCATCTG
TP7018_Query	D	1	chr1	.	CAGCAATAGCTGGACTGATTTCAGGCCTAAGATGCCATTGAGTAGACATAGTTGACGCCATCTG
TP70225_Hit	D	1	chr1	.	CTGCCGTTTTCAAATGGAAATGAGATTTTTCATCTTTAGGTGTAGAGGAATCCATCCCTTCATC
TP70225_Query	D	1	chr1	.	CTGCAGTTTTCAAATGGAAATGAGATTTTTCATCTTTAGGTGTAGAGGAATCCATCCCTTCATC
TP70344_Hit	D+G	1	chr1	.	CTGCATAACTTGTTATTAAAACCAAAAGATTGAAGTACTTTAATGAGAAAACTCCATTCAATTG
TP70344_Query	D+G	1	chr1	.	CTGCATAACTTGTTATTAAAACCAAAAGATTGAAGTACTTTAATAAGAAAACTCCATTCAATTG

Name	Filter	Nb hit (Mt4.0)	Mt Chr (Mt4.0)	Ms Chr (Li et al., 2014)	Sequence
TP70393_Hit	D	1	chr1	.	CTGCATCATATCCTTGCAAGTAAAGAGATTTGGAATTGGGAAACAATCAGTCACGAAAAGTACA
TP70393_Query	D	1	chr1	.	CTGCATAATATCCTTGCAAGTAAAGAGATTTGGAATTGGGAAACAATCAGTCACGAAAAGTACA
TP70590_Hit	D	1	chr1	.	CTGCATAGCTGTCTCCACATGCCCCACACAAAGTTTCTCCGTGTTCTTCCTCATCTTCCTCGTC
TP70590_Query	D	1	chr1	.	CTGCATAGCTGTCTCCACATGCCCCACACAAAGTCTCTCCGTGTTCTTCCTCATCTTCCTCGTC
TP70787_Hit	D+G	1	chr1	.	CTGCATATGGGAAATATACATCATTAAAATATAAGTATTGGGCACATTGGAAAGACACAAAACA
TP70787_Query	D+G	1	chr1	.	CTGCATATGGGAAATATACATCATTAAAAGATAAGTATTGGGCACATTGGAAAGACACAAAACA
TP70996_Hit	D	1	chr1	.	CTGCATCACCGAGAAAATTTGCATGTGGATAAGATACCCATTACAGAATCCGACGGTGGAGGAA
TP70996_Query	D	1	chr1	.	CTGCATCACCGAGAAAATTTGCATGTGGATAAGATACCCATTACAGAATCCCACGGTGGAGGAA
TP7124_Hit	D	1	chr1	.	CAGCAATATCAACAACATAATTATTTAAGTTAATTCCAACATTTATAAGTTAAGTCATCAGTTG
TP7124_Query	D	1	chr1	.	CAGCAATATCAACAACATAATTATTTAAGTTAATACCAACATTTATAAGTTAAGTCATCAGTTG
TP71337_Hit	D	1	chr1	.	CTGCATCGCCTCCCGGAACTCCCGGTGGTTTAGTGAGTAATTTTGAAGTGAAATCATTTGATGG
TP71337_Query	D	1	chr1	.	CTGCATCGCCTCCCGGAACTCCCGGTGGTTTAGTGAGTAATTTTGAAGTGAAATCATTTGACGG
TP71410_Hit	D	1	chr1	.	CTGCGTCTAGGAAAGGGGCCTTGGGATTCGACTGCTTCCAGCTTCAGAACAAGGTGCTGAAAAA
TP71410_Query	D	1	chr1	.	CTGCATCTAGGAAAGGGGCCTTGGGATTCGACTGCTTCCAGCTTCAGAACAAGGTGCTGAAAAA
TP71882_Hit	D	1	chr1	.	CTGCATGCTAGAACTTTAGTATCCTATTTGCTTCCTCTAAACAATTGCGATTTCTTTTTTTAGT
TP71882_Query	D	1	chr1	.	CTGCATGCTAGAACTTTAGTATCCTATTTGCTTCCTCTAAACAACTGCGATTTCTTTTTTTAGT
TP71971_Hit	D+G	1	chr1	.	CTGCATGGGAATTATTTCACTTGATCTTGACAGAGCGGCACTGGGCATTCACACATTTAGCATT
TP71971_Query	D+G	1	chr1	.	CTGCATGGGAATCATTTCACTTGATCTTGACAGAGCGGCACTGGGCATTCACACATTTAGCATT
TP72136_Hit	D	1	chr1	.	CTGCGTGTGGACATCCAGCTCCTAATGCATTTGAAACTCTTGCGCTGAACAACACAACAAAAAG
TP72136_Query	D	1	chr1	.	CTGCATGTGGACATCCAGCTCCTAATGCATTTGAAACTCTTGCGCTGAACAACACAACAAAAAG
TP72265_Hit	D	1	chr1	.	CTGCATTGAGCTTCTTGGCTAATTTTGAAGATGTGTATAAACTATGGACTTTTGTTGCTAGGTT
TP72265_Query	D	1	chr1	.	CTGCATTAAGCTTCTTGGCTAATTTTGAAGATGTGTATAAACTATGGACTTTTGTTGCTAGGTT
TP72283_Hit	D	1	chr1	.	CTGCTTTAATTGGAGCCACTGCAAGGCCTGGAGGATGGAAAAAAGGCATAGCCATAATGGATTT
TP72283_Query	D	1	chr1	.	CTGCATTAATTGGAGCCACTGCAAGGCCTGGAGGATGGAAAAAAGGCATAGCCATAATGGATTT
TP72315_Hit	D	1	chr1	.	CTGCTTTAGAAATTGTTCGTCAGGGTGCAGATGTGATGCCAAAGAGCCAGCTTAATCAAGTTTT
TP72315_Query	D	1	chr1	.	CTGCATTAGAAATTGTTCGTCAGGGTGCAGATGTGATGCCAAAGAGCCAGCTTAATCAAGTTTT
TP72399_Hit	D	1	chr1	.	CTGCATTATGTCGATTTTCTAAATGACTACTACTACTGCTATAGTTGAAAGGTTTGATCATCAC
TP72399_Query	D	1	chr1	.	CTGCATTATGTCGATTTTCTAAATGACTACTACTACTGCTATAGTTGAAAGGTTTAATCATCAC
TP72606_Hit	D	1	chr1	.	CTGCATTGAGAATCTTGGTGCCACTCCTTATCTTATTTCATGCTTTGAAGTCCTACAATACAAG
TP72606_Query	D	1	chr1	.	CTGCATTGAGAATCTTGGTGCCACTCCTTATCTTATTTCATGCTTCGAAGTCCTACAATACAAG
TP72853_Hit	D	1	chr1	.	CTGCATTTACCACATTTGTTTGGCGCATTAAATTAGGATTCTGTAACAGATTCATCAAGGTGCT
TP72853_Query	D	1	chr1	.	CTGCATTTACCACATTTGTTTGGCGCATTAAATTAGGATTCCGTAACAGATTCATCAAGGTGCT
TP7296_Hit	D	1	chr1	.	CAGCAATCAATACTACCTATCTCTAATATTAGTTTGAGACAGGCATTTGAAAGAGGAAGGCTCA
TP7296_Query	D	1	chr1	.	CAGCAATCAATACTACCTATCTCTAATATTAGTTTGAGACAGCCATTTGAAAGAGGAAGGCTCA
TP72986_Hit	D	1	chr1	.	CTGCATTTCTTTTGACAGGACGGTGATGACATAAGAAGAGAAAGAAAAAGATTGTCAAACAGGA
TP72986_Query	D	1	chr1	.	CTGCATTTCTTTTGACAGGAAGGTGATGACATAAGAAGAGAAAGAAAAAGATTGTCAAACAGGA
TP73009_Hit	D	1	chr1	.	CTGCATTTGAGGTTGTAGATTTATGACGTATAATTGTGTGACAGCATTGTACAGTCTTGAACAA
TP73009_Query	D	1	chr1	.	CTGCATTTGAGGTTGTAGATTTATGACGTATAATTGTGTGACAGCATTGCACAGTCTTGAACAA
TP73041_Hit	D	1	chr1	.	CTGCATTTGGACAAAAGGTTGTTATTTTTTATCTGCTAAAATGCTTTAGATCTCTTTTAGGTTC
TP73041_Query	D	1	chr1	.	CTGCATTTGGACAAAAGGTTGTTATTTTTTATCTGCTAAAATGCTTTAGATATCTTTTAGGTTC
TP73059_Hit	D	1	chr1	.	CTGCATTTGGTTTTTCAAGTGAAAGTGAACCCTTATTCTATCGTTCATTGCTCATAATGCAAGT
TP73059_Query	D	1	chr1	.	CTGCATTTGGTTTTTCAAGTGAAAATGAACCCTTATTCTATCGTTCATTGCTCATAATGCAAGT
TP73138_Hit	D	1	chr1	.	CTGCATTTTGGCAATAGCTTGAACACATCGCAATGTTCCAACAGCATGCCTTCTAACCAAGTGC
TP73138_Query	D	1	chr1	.	CTGCATTTTAGCAATAGCTTGAACACATCGCAATGTTCCAACAGCATGCCTTCTAACCAAGTGC
TP73547_Hit	D+G	1	chr1	.	CTGCCAACAAGTGCTAACAAATGAACTCTTTACCACGAATTGCATGCACTTCTAGAACAACAGA
TP73547_Query	D+G	1	chr1	.	CTGCCAACAAGTGCTAACAAATGAACTCTTTACCAAGAATTGCATGCACTTCTAGAACAACAGA
TP7363_Hit	D	1	chr1	.	CAGCAATCATATCATACATAACTTGAGAAACTCCGGTTGAGTCACGGTTCCTAGCCCGGTTCAT
TP7363_Query	D	1	chr1	.	CAGCAATCATATCATACATAACTTGAGAAACCCCGGTTGAGTCACGGTTCCTAGCCCGGTTCAT
TP73672_Hit	D	1	chr1	.	CTGCTAACTTCGCTAGGAGGCTACTTGAGACAAATCCCACCATTGAAAACCAAGCGAAGACAGC
TP73672_Query	D	1	chr1	.	CTGCCAACTTCGCTAGGAGGCTACTTGAGACAAATCCCACCATTGAAAACCAAGCGAAGACAGC
TP73956_Hit	D	1	chr1	.	CTGCCAATTCAATTCTTTCCCATCTCTTTTTTCTATTCATATCTTCTTGATGTGCCACTGATTC
TP73956_Query	D	1	chr1	.	CTGCCAATTCAATTCTTTCCCATCTCTTTTGTCTATTCATATCTTCTTGATGTGCCACTGATTC
TP73971_Hit	D+G	1	chr1	.	CTGCCAATTGATACTTTAATGCCAGACTCATCAGAGGATACACGTCCTTTTGTAGCAACACTAT
TP73971_Query	D+G	1	chr1	.	CTGCCAATTGATACTTTAATGCCAGACTCATCAGAGGACACACGTCCTTTTGTAGCAACACTAT

Name	Filter	Nb hit (Mt4.0)	Mt Chr (Mt4.0)	Ms Chr (Li et al., 2014)	Sequence
TP74208_Hit	D	1	chr1	.	CTGCCACATGTTTGGAAGCCTTTTTGCAGTGATTTGAAGATGCCAAAGTTTTGGCCTGATGTGA
TP74208_Query	D	1	chr1	.	CTGCCACATGTTTGGAAGCCTTTTTGCAGTGATTTGAAGATGCCAAAGTTTTGCCCTGATGTGA
TP7426_Hit	D	1	chr1	.	CAGCAATCTTATAAGGTCAGTGAATCATTTCCATTCAAGTGGATTAATAAAAAATGGAAAGAAG
TP7426_Query	D	1	chr1	.	CAGCAATCCTATAAGGTCAGTGAATCATTTCCATTCAAGTGGATTAATAAAAAATGGAAAGAAG
TP7432_Hit	D+G	1	chr1	.	CAGCAATCCTGTATAGAAGTGGGAAGGAAGATGACCACGATATTCTTAGCGGAGCTTCACTATC
TP7432_Query	D+G	1	chr1	.	CAGCAATCCTGTATAGAAGTGGGAAGGAAGATGACCACGATATTCTCAGCGGAGCTTCACTATC
TP74525_Hit	D	1	chr1	.	CTGCCACTGCCACCGCTACCTTGCAGTTCACTACCACCCTGACCACTAGCATTACTATTTGGAG
TP74525_Query	D	1	chr1	.	CTGCCACTGCCACCACTACCTTGCAGTTCACTACCACCCTGACCACTAGCATTACTATTTGGAG
TP74727_Hit	D	1	chr1	.	CTGCCAGATTGAAGTTTTTGTCACACTTAATGCAGGGCACCAAAGAAATTTTACAAACTGGAGT
TP74727_Query	D	1	chr1	.	CTGCCAGATTGAAGTTTTTGTCACACTTAATGCAGGGCACCAAAGAAATTTTACAAACTGGAAT
TP74784_Hit	D+G	1	chr1	.	CTGCCAGCCTTCTCCCAACAATAAATATATCAACTGCTTCATAGCTCAAAATTGGTTCAGTGGC
TP74784_Query	D+G	1	chr1	.	CTGCCAGCCTTCTCCCAACAATAAATATATCAACTGCTTCATAGCTCAAAATTGGTCCAGTGGC
TP74823_Hit	D	1	chr1	.	CTGCTAGGAATGCCTACCACACAAAGTACCGAGGGAATTTTGTGACACACGACTTGTCTTGCGC
TP74823_Query	D	1	chr1	.	CTGCCAGGAATGCCTACCACACAAAGTACCGAGGGAATTTTGTGACACACGACTTGTCTTGCGC
TP75008_Hit	D	1	chr1	.	CTGCCATAACCTTGCCTTCCAACGACAAAGTCCTTGACATGACTGCAGAATCCTGGTTCAGCTC
TP75008_Query	D	1	chr1	.	CTGCCATAACCTTGCCTTCCAACGACAAAATCCTTGACATGACTGCAGAATCCTGGTTCAGCTC
TP75207_Hit	D	1	chr1	.	CTGCCATCAGGTATTACTATGGCTGTGATATAAACACCGCGTTCAAATTGTTCAATGACCGACG
TP75207_Query	D	1	chr1	.	CTGCCATCAGGTATTACTATGGCTGTGATATAAACACCGCGTTCAAATTGTTCAATGACAGACG
TP7551_Hit	D	1	chr1	.	CAGCAATCTTGAAACCCCTTCCACCAAGGTAGAAAAGGCAAGTGAGGTAATCAAATCTGCATGT
TP7551_Query	D	1	chr1	.	CAGCAATCTTGAAACCCCTTCCACCAAGGTAGAAAAGGCAAGTGAGGCAATCAAATCTGCATGT
TP75518_Hit	D+G	1	chr1	.	CTGCCATGTTCAAAATTGGTAATAGCAAGGAGCTCCCAACAATTCCAGATCATCTCTCAAATGA
TP75518_Query	D+G	1	chr1	.	CTGCCATGTTCAAAATTGGTAACAGCAAGGAGCTCCCAACAATTCCAGATCATCTCTCAAATGA
TP75577_Hit	D	1	chr1	.	CTGCCATTCAGAGATATGTACCATCCCTGGTATAAACATTATGACTTTCTGCTCTTTTCTTTTA
TP75577_Query	D	1	chr1	.	CTGCCATTCAGAGATATGTACCATCCCTGGTATAAACATTATGACTTTCTGCTCTTTTATTTTA
TP7563_Hit	D	1	chr1	.	CAGCAATCTTTTATTAGAAAACACACCGAAAGCTCTGAGAGACGATGATGTTCGAGTGTGGGAT
TP7563_Query	D	1	chr1	.	CAGCAATCTTTTATCAGAAAACACACCGAAAGCTCTGAGAGACGATGATGTTCGAGTGTGGGAT
TP75687_Hit	D+G	1	chr1	.	CTGCCATTGTTTGGTTGTTTAGAATTGGGAGGGAAATTTGAAGGAATGGATTGTAGTGAGGATT
TP75687_Query	D+G	1	chr1	.	CTGCCATTGTTTGGTTGTTTAGAATTGAGAGGGAAATTTGAAGGAATGGATTGTAGTGAGGATT
TP75806_Hit	D	1	chr1	.	CTGCCCAAACTGATCGTTGTTTCCCTGTGGTTCCATCTTCACAGGTCCGATACCACCTATCCCT
TP75806_Query	D	1	chr1	.	CTGCCCAAACTGATCGTTGTTTCCCTGTGGTTCCATCTTCACAGGTCCAATACCACCTATCCCT
TP75809_Hit	D+G	1	chr1	.	CTGCCCAAACTTTATAAGCACTACATAGGCCATATCTCTATGAAAATGTGGGACTAAATCAACC
TP75809_Query	D+G	1	chr1	.	CTGCCCAAACTTTATAAGCACTACATAGGCCATATCTCTATGAAAATGAGGGACTAAATCAACC
TP75878_Hit	D+G	1	chr1	.	CTGCCCAATACTGTGTTCAAAAGCAAGATCATGGTTTGGACATGGCTTTGGATAATAAGCTCAT
TP75878_Query	D+G	1	chr1	.	CTGCCCAATACTGTGTTCAAAAGCAAGATCATGGCTTGGACATGGCTTTGGATAATAAGCTCAT
TP75889_Hit	D	1	chr1	.	CTGCCCAATGCACTCTGTCATTTCCGTCCCCACCATCACTGCTTTCATACTTCGCCTCTACCAC
TP75889_Query	D	1	chr1	.	CTGCCCAATGCACTCTGTCATTTCCGTCCCCACCATCACTGCTTCCATACTTCGCCTCTACCAC
TP7643_Hit	D+G	1	chr1	.	CAGCAATGACATTTCCAACACGTATTTCATAAGTCGTGTGAGGCAAATAAAATATCCTGAATTT
TP7643_Query	D+G	1	chr1	.	CAGCAATGACATTTCCAACACATATTTCATAAGTCGTGTGAGGCAAATAAAATATCCTGAATTT
TP76666_Hit	D	1	chr1	.	CTGCCCTCCAAACCAGGTGCTTATCCTTCTCAGAAGCAAAATAATGTATATGCAGAACCCTTAG
TP76666_Query	D	1	chr1	.	CTGCCCTCCAAACCAAGTGCTTATCCTTCTCAGAAGCAAAATAATGTATATGCAGAACCCTTAG
TP76783_Hit	D	1	chr1	.	CTGCCCTGACCTTTTATGTGACAACAATGAGTACAAAATTACCATCTTAACATAGAAATACAAA
TP76783_Query	D	1	chr1	.	CTGCCCTGACCTTTTATGTAACAACAATGAGTACAAAATTACCATCTTAACATAGAAATACAAA
TP77122_Hit	D	1	chr1	.	CTGCCGAGGCGTGTGAGAGCTTCTCTCAGGACCAGGCTTAAGCCATATTCCATGGCTGAAAAAA
TP77122_Query	D	1	chr1	.	CTGCCGAGGCGTGTAAGAGCTTCTCTCAGGACCAGGCTTAAGCCATATTCCATGGCTGAAAAAA
TP77138_Hit	D	1	chr1	.	CTGCCGAGTCAGAAGACTGATTTGTCAAGTTGAAATCTGCAATCTTAGCCTCATAATCATTGAA
TP77138_Query	D	1	chr1	.	CTGCCGAGTCAGAAGACTGATTTGTCAAGTTGAAATCTGCAATCTTAGCCTCATAATCATTAAA
TP77434_Hit	D+G	1	chr1	.	CTGCCGCGGCAACACCTCCAGGGACGATGTTAGTGCGTCCTGTGGCTCTGACTTCTGCTGAAAA
TP77434_Query	D+G	1	chr1	.	CTGCCGCGGCAACACCTCCAGGGACGATGTTAGTGCGCCCTGTGGCTCTGACTTCTGCTGAAAA
TP7811_Hit	D	1	chr1	.	CAGCAATGCTCGTGCCCGTGATAGAGTCGAGGCGCTTCAAGCATACTATCACCAGCTGAAAAAA
TP7811_Query	D	1	chr1	.	CAGCAATGCTCGTGCCCGTGATAGAGTCGAGGCGCTTCAAGCATACTATCACCAGCAGAAAAAA
TP78171_Hit	D+G	1	chr1	.	CTGCCTACTCAAACTGACCTCAATACGCCGCCGCTTCTTCCTGTTGGTGCTGAAAAAAAAAAAA
TP78171_Query	D+G	1	chr1	.	CTGCCTACTCAAACTGACCTCAATACGCCACCGCTTCTTCCTGTTGGTGCTGAAAAAAAAAAAA
TP78508_Hit	D	1	chr1	.	CTGCCTCCCTTATATTTTTGTGTATTTTCCATTAGCTGAAAAGGATGAAAATGGTTGTGCAAAG
TP78508_Query	D	1	chr1	.	CTGCCTCCCTTATATTTTTGTGAATTTTCCATTAGCTGAAAAGGATGAAAATGGTTGTGCAAAG

Name	Filter	Nb hit (Mt4.0)	Mt Chr (Mt4.0)	Ms Chr (Li et al., 2014)	Sequence
TP78516_Hit	D+G	1	chr1	.	CTGCCTCTGCCACGCCACCTGCTGTAACGCATACACCACCTCGTTAACCGAATAGTACTGCTGA
TP78516_Query	D+G	1	chr1	.	CTGCCTCCGCCACGCCACCTGCTGTAACGCATACACCACCTCGTTAACCGAATAGTACTGCTGA
TP78627_Hit	D+G	1	chr1	.	CTGCCTCGTGGTTTTGAGGAAGTTCCACTTACTGAACCAACAGTTGGTGTGTTGGGGTGCTGAA
TP78627_Query	D+G	1	chr1	.	CTGCCTCGTGGTTTTGAGGAAGTTCCACCTACTGAACCAACAGTTGGTGTGTTGGGGTGCTGAA
TP78811_Hit	D	1	chr1	.	CTGCCTGACCTTGTGCTTGTTCCAGAATTTGAGTGACCAACTTGTAAGTACGGCCGTTAAGGGC
TP78811_Query	D	1	chr1	.	CTGCCTGACCTTGTGCTTGTTCCAGAAGTTGAGTGACCAACTTGTAAGTACGGCCGTTAAGGGC
TP7893_Hit	D	1	chr1	.	CAGCAATGGCTACTTCACTTTCCCTTTCTTGTTCTTCTCCCTTACTTACCCGAAACACTCTCTT
TP7893_Query	D	1	chr1	.	CAGCAATGGCTACTTCACTTTCCCTCTCTTGTTCTTCTCCCTTACTTACCCGAAACACTCTCTT
TP79184_Hit	D	1	chr1	.	CTGCCTTCAAAGCATTTTGGAATAGTCCAGTTGGTCCTAAAACAACTCACTTTTGGGGACCTGT
TP79184_Query	D	1	chr1	.	CTGCCTTCAAAGCATTTTGGAATAGCCCAGTTGGTCCTAAAACAACTCACTTTTGGGGACCTGT
TP79473_Hit	D	1	chr1	.	CTGCCTTTCACAAACTTCACTTTCGGCGATGACCATGCAAGACTAAATGCAACAGTACATTTTC
TP79473_Query	D	1	chr1	.	CTGCCTTTCACAAACTTCACTTTCGGAGATGACCATGCAAGACTAAATGCAACAGTACATTTTC
TP79624_Hit	D	1	chr1	.	CTGCGAAAAATGCAAGAAGTGCCCTATCTGCCGTGGGACAATTGAAGAACGGATGCCTATATAT
TP79624_Query	D	1	chr1	.	CTGCGAAAAATGCAAGAAGTGCCCTATCTGCCGTGGGACAATCGAAGAACGGATGCCTATATAT
TP79948_Hit	D	1	chr1	.	CTGCGACAAATCCCCAGTTAGCAACAGGTCCCCAAAAGTGAGTTGTTTTAGGACCAACTGGGCT
TP79948_Query	D	1	chr1	.	CTGCGACAAATCCCCAGTTAGCAACAGGTCCCCAAAAGTGAGTTGTTTTAGGACCAACTGGACT
TP80063_Hit	D	1	chr1	.	CTGCGACCGTGAGATTTCTTGGTTCTCTCTTTATCAGTTTCGTGAGAAACATGGTCCGGACTTT
TP80063_Query	D	1	chr1	.	CTGCGACCGTGAGATTTCTTGATTCTCTCTTTATCAGTTTCGTGAGAAACATGGTCCGGACTTT
TP80064_Hit	D	1	chr1	.	CTGCGACCTAATTCACTGGTGCGTTGATGGTCGCTATGGTGGTTGCGACCGCAAGTCGCGGTGG
TP80064_Query	D	1	chr1	.	CTGCGACCTAATTCAATGGTGCGTTGATGGTCGCTATGGTGGTTGCGACCGCAAGTCGCGGTGG
TP8012_Hit	D	1	chr1	.	CAGCAATGTCTTCAAGATCTTCTTTTGACAAATCTGGTGCGATTTTTACTAGCAATGGAGGGGG
TP8012_Query	D	1	chr1	.	CAGCAATGTCTTCAAGATCTTCTTTTGACAAATCTGGTGCGATTTTTACCAGCAATGGAGGGGG
TP80590_Hit	D+G	1	chr1	.	CTGCGATGCAGGAGAACGCATCCGGGATTGTGGGTTTGGTATTTGAGTCAACGTAATAAGGGGA
TP80590_Query	D+G	1	chr1	.	CTGCGATGCAGGAGAACGCATCCGGGATTGTGGGTTTGGTATTTGAGTCAACGTAACAAGGGGA
TP80750_Hit	D	1	chr1	.	CTGCGATTTCTGCTGTAGTAGATAGAAGAGATGTGTTAAATGGCGACACACCTCTTCACTTGTC
TP80750_Query	D	1	chr1	.	CTGCGATTTCTGCTGTAGTAGATAGAAGAGATGTGTTAAATGGCGACACACCGCTTCACTTGTC
TP81320_Hit	D	1	chr1	.	CTGCGCCTGTGATTGATGACTTCGCTTGGTATTATAAAATAGAATAGATATGATCAATCTGTTG
TP81320_Query	D	1	chr1	.	CTGCGCCTGTGATTGATGACTTCGCTTGGTATTATAAAATAGAATAAATATGATCAATCTGTTG
TP81490_Hit	D	1	chr1	.	CTGCGCGTAATGACGTTATTTGGAATGACACGCGTCAATCTTCGATGGCAATTGGGAGGTCTGC
TP81490_Query	D	1	chr1	.	CTGCGCGTAATGACGTTATTTGGAATGACACGCGTCAATCTTCGATGACAATTGGGAGGTCTGC
TP81786_Hit	D+G	1	chr1	.	CTGCGCTTGCAGAAATTTTTCTTCTTTTGGACTCTAGCTCCAGCCTTCTCTTACCAGACATGGA
TP81786_Query	D+G	1	chr1	.	CTGCGCTTGCAGAAATTTTTCTTCTTTTAGACTCTAGCTCCAGCCTTCTCTTACCAGACATGGA
TP81813_Hit	D	1	chr1	.	CTGCGGAAAAGCTGTTTCCTCCATTGGTAAGGCTATATAGCACTAGTTGGTTGCGTCCCTCTTT
TP81813_Query	D	1	chr1	.	CTGCGGAAAAGCTATTTCCTCCATTGGTAAGGCTATATAGCACTAGTTGGTTGCGTCCCTCTTT
TP81940_Hit	D	1	chr1	.	CTGCGGAGCCCCACGGAGAAGGTTTGCAAGCTTGTCAAGGGGTGCGGAGATCGGAAGAGCGGTT
TP81940_Query	D	1	chr1	.	CTGCGGAGCCCCACGGAGAAGGTTTGCAAGCTTGTCAAGGGGTGCCGAGATCGGAAGAGCGGTT
TP82060_Hit	D+G	1	chr1	.	CTGCGGATTTTGATTTTTCATCCCCAAAGATTATGCAAGACACTATCCCAGAATTTATTGAGCA
TP82060_Query	D+G	1	chr1	.	CTGCGGATTTTGATTTTTCATCCCCAAAGATTATGCAAGACACCATCCCAGAATTTATTGAGCA
TP82345_Hit	D	1	chr1	.	CTGCGGCTCCTCCTCAGCAGGATGATAAAGTTCAGCCTGTTCCTGTAGTAGGTTCTGCCCCTGG
TP82345_Query	D	1	chr1	.	CTGCGGCTCCTCCTCAGCAGGATGATAAAGTTCAGCCGGTTCCTGTAGTAGGTTCTGCCCCTGG
TP82417_Hit	D	1	chr1	.	CTGCGGCTTGGGAAGCATTTTGTTGAGAATTTAAAAAAGTAGCAGGTATTCCAAACTTGAGGTT
TP82417_Query	D	1	chr1	.	CTGCGGCTTGGGAAGCATTTTGTTGAGAATTTAAAAAAGTAGCAGGGATTCCAAACTTGAGGTT
TP82421_Hit	D+G	1	chr1	.	CTGCGGCTTTCGGAATCTGGTCTGAGAAGACGGAGATTGGGAAAACAGTGAGTGGAGCTATTGT
TP82421_Query	D+G	1	chr1	.	CTGCGGCTTTCGGAATCTGGTCGGAGAAGACGGAGATTGGGAAAACAGTGAGTGGAGCTATTGT
TP82456_Hit	D+G	1	chr1	.	CTGCGGGAGAGGCCGTGGTTGGTGTTCTGCGGATCGCTGGGGGCCATGAGGGGATCCAAGCTGT
TP82456_Query	D+G	1	chr1	.	CTGCGGGAGAGGCCGTGGTTGGTGTTCTGCGGATCGCTGGGGGCCATGAGGGGATCCAAGCTGA
TP82688_Hit	D	1	chr1	.	CTGCGGTATACTCTCTAAAGCACGCACGCCAATGATGAAGCTCGGCTTCAAGCTGGAGAGTTGC
TP82688_Query	D	1	chr1	.	CTGCGGTATACTCTCTAAAGCACGCACGCCAATGATGAAGCTCGGCTTCAAGCTGAAGAGTTGC
TP8279_Hit	D+G	1	chr1	.	CAGCAATTCATACACGCACTAGCCTGGCTTGATTCTTTGAAATATAAATGATACATATATTTTT
TP8279_Query	D+G	1	chr1	.	CAGCAATTCATACACACACTAGCCTGGCTTGATTCTTTGAAATATAAATGATACATATATTTTT
TP82857_Hit	D	1	chr1	.	CTGCGGTGGTTAACTCCGCCGTGAATGTTAGATCTGCTGGTGATATTATGACTTTAACTGCTGA
TP82857_Query	D	1	chr1	.	CTGCGGTGGTTAACTCCGCCGTGAATGTTAGATCTGCTGGTGATATCATGACTTTAACTGCTGA
TP829_Hit	D	1	chr1	.	CAGCAAAATACAGACCTCAAAATTGCTATTTCTTGAGTAAACTCATCCTCTAAGGCATCATTCA
TP829_Query	D	1	chr1	.	CAGCAAAATACAGACCTCAAAATTGCTATTTCTTGAGTAAACTCATCCTCTAAAGCATCATTCA

Name	Filter	Nb hit (Mt4.0)	Mt Chr (Mt4.0)	Ms Chr (Li et al., 2014)	Sequence
TP8294_Hit	D	1	chr1	.	CAGCAATTCCAATGTATTATTGTTAAATCCGTTGCTCTCAAACTGCTGTGCCTGATTGTAGGAG
TP8294_Query	D	1	chr1	.	CAGCAATTCCAATGTATTATTGTTAAATCCGTTGCTCTCAAACTGCTGTACCTGATTGTAGGAG
TP83111_Hit	D	1	chr1	.	CTGCGTAGCTTAGCAAGATGGTTATTGATTCTCTCTCTGCGTCTCCTTTCAGCTTCACTATGAC
TP83111_Query	D	1	chr1	.	CTGCGTAGCTTAGCAAGATGGTTATTGATTCTTTCTCTGCGTCTCCTTTCAGCTTCACTATGAC
TP83130_Hit	D	1	chr1	.	CTGCGTAGTGGCATGTAAATGTAATATTTATTCTGCACAATGTCTAACTTATACTGCACCATTT
TP83130_Query	D	1	chr1	.	CTGCGTAGTGGCATGTAAATGTAATATTTATTCTGCACAATGTCTAACTTATACAGCACCATTT
TP83936_Hit	D	1	chr1	.	CTGCGTTTAGTAATTATCCTACATCATCTTCCAATTTGGAGCAACAGCCACCATTGCAGGCCTT
TP83936_Query	D	1	chr1	.	CTGCGTTTAGTAATTATCCTACATCATCTTCCAATTTGGAGCAACAACCACCATTGCAGGCCTT
TP8399_Hit	D	1	chr1	.	CAGCAATTGATGCTCCTGGTGGTTTTCTTTTCGAGTGGTGGTCTGTTTTCTGGGACATTTTTAT
TP8399_Query	D	1	chr1	.	CAGCAATTGATGCTCCTGGAGGTTTTCTTTTCGAGTGGTGGTCTGTTTTCTGGGACATTTTTAT
TP84094_Hit	D	1	chr1	.	CTGCTAAACATCAAACTGTTTCGAAGCCTCTGGCTGAAGAAACTGAAGATAGAGAACAAGCCAA
TP84094_Query	D	1	chr1	.	CTGCTAAAAATCAAACTGTTTCGAAGCCTCTGGCTGAAGAAACTGAAGATAGAGAACAAGCCAA
TP84216_Hit	D+G	1	chr1	.	CTGCTAAATAAGCAGATGGGTCAGAGGAATTAGCGGGTTTCACGGAGCCAAGAGTGTCATGACA
TP84216_Query	D+G	1	chr1	.	CTGCTAAATAAGCAGATGGGTCAGAGGAATTAGCGGGTTTCACAGAGCCAAGAGTGTCATGACA
TP8428_Hit	D	1	chr1	.	CAGCAATTGCTGGGAGTTTCACACGTGAGGATTTCATGAATGAGGCATTCCTGCATATGAAAGC
TP8428_Query	D	1	chr1	.	CAGCAATTGCCGGGAGTTTCACACGTGAGGATTTCATGAATGAGGCATTCCTGCATATGAAAGC
TP84361_Hit	D	1	chr1	.	CTGCTAACTCAGACACAATCTCGTTAGATTATGCTTTGTTTAAGCAAAACCCTGGTGTTGTTGA
TP84361_Query	D	1	chr1	.	CTGCTAACTCAGACACAATCTCATTAGATTATGCTTTGTTTAAGCAAAACCCTGGTGTTGTTGA
TP84421_Hit	D+G	1	chr1	.	CTGCTAAGATTTTGGTTTTGGCTGGGAAAGCTCAACAGGAGGAAATTGGTGATGGCGCTAATTT
TP84421_Query	D+G	1	chr1	.	CTGCTAAGATTTTGGTTTTGGCTGGGAAAGCTCAACAAGAGGAAATTGGTGATGGCGCTAATTT
TP84560_Hit	D	1	chr1	.	CTGCTAGTCACACTGAAGCAAACATCGGATATAAAACTCTTGAAGCCATGAGAAGATAACCACC
TP84560_Query	D	1	chr1	.	CTGCTAATCACACTGAAGCAAACATCGGATATAAAACTCTTGAAGCCATGAGAAGATAACCACC
TP8457_Hit	D+G	1	chr1	.	CAGCAATTGGAGGTACCACAGGTGCGCTAGTGGAACAGAACAAACAAGCCCTAGGTTTGATCGA
TP8457_Query	D+G	1	chr1	.	CAGCAATTGGAGGTACCACAGGTGCGCTAGTGGAACAGAACAAACAAGCCCTAGGTTTAATCGA
TP84686_Hit	D	1	chr1	.	CTGCTAATTGATTTCCATTTTCTTTATTTGAATGAGACAATTGCATAATTATATGAAGTAGTAA
TP84686_Query	D	1	chr1	.	CTGCTAATTGATTTCCATTTTCTCTATTTGAATGAGACAATTGCATAATTATATGAAGTAGTAA
TP84788_Hit	D	1	chr1	.	CTGCTACAATGTAACAATTCCATATAGAGAACCCTGTGTTTTGGTTAGGATCTGTTCTTCCTTG
TP84788_Query	D	1	chr1	.	CTGCTACAATGTAACAATTCCATATAGAGAACCCTGTGTTGTGGTTAGGATCTGTTCTTCCTTG
TP84985_Hit	D+G	1	chr1	.	CTGCTACCACTTTCGGAACGCTCATCGTTGATGGCTGGAAGTAGCGGTGCTGAAAAAAAAAAAA
TP84985_Query	D+G	1	chr1	.	CTGCTACCACTTTCGGAACGCTCATCGTTGATGGCTGGAAGTAGAGGTGCTGAAAAAAAAAAAA
TP85185_Hit	D	1	chr1	.	CTGCTACTAGTGTCAGTGTCACTGCCACCACAATCCTTATAAACAGAACTGAGGGATTTCTTTA
TP85185_Query	D	1	chr1	.	CTGCTACTAGTATCAGTGTCACTGCCACCACAATCCTTATAAACAGAACTGAGGGATTTCTTTA
TP85286_Hit	D	1	chr1	.	CTGCTACTGGAAGATATGACATACAGGCTTTGGCTGGTTCTGGCCACGTTTCCCCTGAAACACT
TP85286_Query	D	1	chr1	.	CTGCTACTGGAAGATATGACATACAGGCTTTGGCTGGTTCTGGCCACGTTTCCCCTGAAACACG
TP8560_Hit	D	1	chr1	.	CAGCAATTTCCAGTTGGGATTTTAGGTGTGTTACTTTTGTTTGCTGGTATTGAACTTGCTATGT
TP8560_Query	D	1	chr1	.	CAGCAATTTCCAGTTGGGATATTAGGTGTGTTACTTTTGTTTGCTGGTATTGAACTTGCTATGT
TP85782_Hit	D+G	1	chr1	.	CTGCTAGGTGGAGTTTTGAGCTTGAACCAGAAAACACGGGAAATTATGTTCTGTTGGCAAAACT
TP85782_Query	D+G	1	chr1	.	CTGCTAGGTGGAGTTTTGAGCTTGAACCAGAAAACACGGGAAATTATGTTCTGTTAGCAAAACT
TP85820_Hit	D	1	chr1	.	CTGCTAGTAGACTCAAGTTCAGCCGCGTCACTCTTTTTGGTAGGCTTCCTTGATGATTGTCTCT
TP85820_Query	D	1	chr1	.	CTGCTAGTAGACTCAAGTTCAGCCGCGTCACTCTTTTTGGTAGGCTTCCATGATGATTGTCTCT
TP85878_Hit	D+G	1	chr1	.	CTGCTAGTGCCTATGAATTAGAATCTCCTCTGACAAGGCAACCTTTTTCTCCCCTCTCATCTAA
TP85878_Query	D+G	1	chr1	.	CTGCTAGTGCCTACGAATTAGAATCTCCTCTGACAAGGCAACCTTTTTCTCCCCTCTCATCTAA
TP85884_Hit	D+G	1	chr1	.	CTGCTAGTGGACAACCACATGTTCCTCCACCAATTACTATATAATCATATGTTAAGATTGGAGG
TP85884_Query	D+G	1	chr1	.	CTGCTAGTGGACAACCACATGTTCCTCCACCAATTACTATATAATCATACGTTAAGATTGGAGG
TP86064_Hit	D	1	chr1	.	CTGCTATGGCACCTAATCTTAGAACAAAATCCATTATGGCTATGCCTTTTTTCCATCCTCCAGG
TP86064_Query	D	1	chr1	.	CTGCTATAGCACCTAATCTTAGAACAAAATCCATTATGGCTATGCCTTTTTTCCATCCTCCAGG
TP86094_Hit	D	1	chr1	.	CTGCTATAGCGGCCGGCGTAGCCGCTTTTGACAACAGAGGTTTCTAATAAGGTACACATCAAGA
TP86094_Query	D	1	chr1	.	CTGCTATAGCGGCCGGCGTAGCCGCTTTTGACAACAGAGGGTTCTAATAAGGTACACATCAAGA
TP86227_Hit	D	1	chr1	.	CTGCTATCAGAATTTACAGAAGAAGCTTTGGCATCTTTTTTCCTCCGTTGCTCATCGCCCCTCG
TP86227_Query	D	1	chr1	.	CTGCTATCAGAATTTACAGAAGAAGCTTTGGCATCTTTTTTCCTCCGTTGCTCATCGCCCCTCA
TP86566_Hit	D	1	chr1	.	CTGCTATGTTAATGGAACCTCTTTGTATAATGCAGAAATAGGAGCGAAAGCATCAAATTATGAT
TP86566_Query	D	1	chr1	.	CTGCTATGTTAATAGAACCTCTTTGTATAATGCAGAAATAGGAGCGAAAGCATCAAATTATGAT
TP86594_Hit	D+G	1	chr1	.	CTGCTATTAAAGGTAACTTGCAGGCATGTATTCTATTAGTACATGCTGGGAAAAATAAAGACCT
TP86594_Query	D+G	1	chr1	.	CTGCTATTAAAGGTAACTTGCAGGCATGTATTCTATTAGTACATGCTGGAAAAAATAAAGACCT

Name	Filter	Nb hit (Mt4.0)	Mt Chr (Mt4.0)	Ms Chr (Li et al., 2014)	Sequence
TP86760_Hit	D	1	chr1	.	CTGCTATTGCGTAAAGCAACAGATCATCTGTTGGTGGTGCACGCTTTCGAACTTTGTTCCGTGC
TP86760_Query	D	1	chr1	.	CTGCTATTGCGTAAAGCAACAGATCATCTGTTGGTGGTGCACGCTTTCGAACTTTGTTCCATGC
TP87400_Hit	D	1	chr1	.	CTGCTCAGTTAGCTCTCCGTGCCATATTTCTTGCTAAAGCAGTTTCTCTAGGATTGCAGGCTGA
TP87400_Query	D	1	chr1	.	CTGCTCAGTTAGCTCTCCGTGCCATATTTCTTGCTAAAACAGTTTCTCTAGGATTGCAGGCTGA
TP87401_Hit	D	1	chr1	.	CTGCTCAGTTAGTTGCTTGAGTAAAGAATGTGTTGATATATTTCTACTTTGTTTCTTTTAATGT
TP87401_Query	D	1	chr1	.	CTGCTCAGTTAGTTGCTTGAGTAAAGAATGTGTTGATATATTTCTACTTTGTTTCTTCTAATGT
TP87483_Hit	D	1	chr1	.	CTGCTCATCTTCTTCAGTAGAAATGACTATGCCTTCTCTACTCCATTCTAGATATCTTATGGTA
TP87483_Query	D	1	chr1	.	CTGCTCATCTTCTTCAGTAGAAATAACTATGCCTTCTCTACTCCATTCTAGATATCTTATGGTA
TP87649_Hit	D	1	chr1	.	CTGCTCCAATTAGCCTGGGCAGGCTGTGATTTCTGAGGCGTAGGCACTGGGATCGCAGGCACAG
TP87649_Query	D	1	chr1	.	CTGCTCCAATTAGCCTGGGCAGGCTGTGATTTCTGAGGCATAGGCACTGGGATCGCAGGCACAG
TP87726_Hit	D	1	chr1	.	CTGCTCCAGCCGTGTTTAGAAATGTTATGTTAACTGCCACGAACCCTTGAGCAACCACAGCTGA
TP87726_Query	D	1	chr1	.	CTGCTCCAGCCGTGTTGAGAAATGTTATGTTAACTGCCACGAACCCTTGAGCAACCACAGCTGA
TP87964_Hit	D+G	1	chr1	.	CTGCTCCGGCTGAACCATCAACGGTGCCGCCATGCTCTCGTGCTGAAAAAAAAAAAAAAAAAAA
TP87964_Query	D+G	1	chr1	.	CTGCTCCGGCTGAACCATCAACGGTGCCGCCATGCTCTCGGGCTGAAAAAAAAAAAAAAAAAAA
TP88068_Hit	D	1	chr1	.	CTGCTCCTCCACTTCCGACTGCCCACAGAATGTTGCGGACACCTTGGAGACCTTCTTCTCTGCG
TP88068_Query	D	1	chr1	.	CTGCTCCTCCACCTCCGACTGCCCACAGAATGTTGCGGACACCTTGGAGACCTTCTTCTCTGCG
TP88259_Hit	D+G	1	chr1	.	CTGCTCCTTGTGTTTTGTTAATTTTTTTCTGCACTAAGTGAATGTACGTATAATGGCCACGTAA
TP88259_Query	D+G	1	chr1	.	CTGCTCCTTGTGTTTTGTTAATTTTTTTCTGCACTAAGTGAATGTAAGTATAATGGCCACGTAA
TP8839_Hit	D	1	chr1	.	CAGCACAAATCCCAACTAATAAGATGATCTCTTTTGGATCTCCCATCCCAACCTTGGCCTAGGA
TP8839_Query	D	1	chr1	.	CAGCACAAATCCCAACTAATAAGATGATCTCTTTTGGATCTCCCATCCCAACCTTGACCTAGGA
TP88438_Hit	D+G	1	chr1	.	CTGCTCGCCTAGTCTAATTACGCGTTCCTCATGCTCTCTTAGAGAACTATTTTTCTCATCCAAT
TP88438_Query	D+G	1	chr1	.	CTGCTCGCCTAGTCTAATTACGCGTTCCTCATGCTCTCTAAGAGAACTATTTTTCTCATCCAAT
TP8857_Hit	D+G	1	chr1	.	CAGCACAAATTCTGGAAAACAATCTACACCGCGCTTCAATCTGCTTTTGCTGATGAGTATGATA
TP8857_Query	D+G	1	chr1	.	CAGCACAAATTCTGGAAAACAATCTACACCGCGCTTCAATCTGCTTTTGCCGATGAGTATGATA
TP88667_Hit	D+G	1	chr1	.	CTGCTCGTTAGTTTTTTGGTGGACAGAAATCATTAAGGAGCTGTATGAGTGCACATCTGACATA
TP88667_Query	D+G	1	chr1	.	CTGCTCGTTAGTTTGTTGGTGGACAGAAATCATTAAGGAGCTGTATGAGTGCACATCTGACATA
TP88695_Hit	D+G	1	chr1	.	CTGCTCTAACTGGAAATAATGACGTGGAAGTGATGTTTAGCCTTGGACTTGAAAATGCAATTCA
TP88695_Query	D+G	1	chr1	.	CTGCTCTAACTGGAAATAATGACCTGGAAGTGATGTTTAGCCTTGGACTTGAAAATGCAATTCA
TP88802_Hit	D+G	1	chr1	.	CTGCTCTTTTCACTTCAACCATCATGAGCAAGCTTCTTGCAAAACCTAACGTAAAACTTTTCAA
TP88802_Query	D+G	1	chr1	.	CTGCTCTATTCACTTCAACCATCATGAGCAAGCTTCTTGCAAAACCTAACGTAAAACTTTTCAA
TP88817_Hit	D+G	1	chr1	.	CTGCTCTCAAAGACGGACCACCTGGCCAAATTGCCGCTCAAACTTTTACTTTCCGTGAACTTGG
TP88817_Query	D+G	1	chr1	.	CTGCTCTCAAAGACGGACCACCTGGCCAAATTGCCGCTCAAACTTTTACTTTCCGTGAACTTGC
TP89227_Hit	D+G	1	chr1	.	CTGCTCTTGAGACAATTTCGACCGATTACACAAACTGTTCCACCTGAACCGCCACCAGTGATCT
TP89227_Query	D+G	1	chr1	.	CTGCTCTTGAGACAATTTCGACCGATTACACAAACCGTTCCACCTGAACCGCCACCAGTGATCT
TP8929_Hit	D	1	chr1	.	CAGCACAACTACATTATCATCTCCAAACTTTGAGTTTGTTTCCAAGAGGGTGTTGAAGCTTTAC
TP8929_Query	D	1	chr1	.	CAGCACAACTACATTATCATCTCCAAACTTTGAGGTTGTTTCCAAGAGGGTGTTGAAGCTTTAC
TP8943_Hit	D+G	1	chr1	.	CAGCACAACTTGTTTTCCCTACCCCTCCTTTCCCACCTAGCATGTAATACTTCCTCTCAGTCCC
TP8943_Query	D+G	1	chr1	.	CAGCACAACTTGTCTTCCCTACCCCTCCTTTCCCACCTAGCATGTAATACTTCCTCTCAGTCCC
TP89501_Hit	D	1	chr1	.	CTGCTGAAATGGGCAAGACTTCAGTCAAGTATGCCTGGATCCTTGACAAGCTCAAGGCTGAACG
TP89501_Query	D	1	chr1	.	CTGCTGAAATGGGCAAGACTTCAGTCAAGTATGCCTGGATCCTTGACAAGCTCAAGACTGAACG
TP89516_Hit	D	1	chr1	.	CTGCTGAAATTTGTATAGTCAGGATTTATTTGTTTATATTCTGTGTAATTTTACATCAAATTCT
TP89516_Query	D	1	chr1	.	CTGCTGAAATTTGCATAGTCAGGATTTATTTGTTTATATTCTGTGTAATTTTACATCAAATTCT
TP8957_Hit	D	1	chr1	.	CAGCACAAGAAACTTTGGTGGCAAAATTCGGAATCAAGGAACAGAGGAACCCAAAACAGAGCGA
TP8957_Query	D	1	chr1	.	CAGCACAAGAAACTTTGGTAGCAAAATTCGGAATCAAGGAACAGAGGAACCCAAAACAGAGCGA
TP89667_Hit	D	1	chr1	.	CTGCTGAAGTTGGAAAAGTTCGCTTGCGGTTTGCACCTGAACCCAGTGGCTATCTTCACATTGG
TP89667_Query	D	1	chr1	.	CTGCTGAAGTTGGAAAAGTTCGCTTGCGATTTGCACCTGAACCCAGTGGCTATCTTCACATTGG
TP8967_Hit	D	1	chr1	.	CAGCACAAGAAGCCTGTGACGAGAAGGAGCATATCTTGACTCTATTTTTCAAGCAGGCTTCACA
TP8967_Query	D	1	chr1	.	CAGCACAAGAAGCCTGCGACGAGAAGGAGCATATCTTGACTCTATTTTTCAAGCAGGCTTCACA
TP89706_Hit	D	1	chr1	.	CTGCTGAATCTGTTTTGCTCTTCAAGTTTGGATATTATGCATTCATGCAAGAATTTGTCAAGAC
TP89706_Query	D	1	chr1	.	CTGCTGAATCTGTTTTGCTCTTCAAGTTTGGATAGTATGCATTCATGCAAGAATTTGTCAAGAC
TP89757_Hit	D+G	1	chr1	.	CTGCTGACAAGTTCACTTCTTTATTGGAGTATAGTTGACTTTTGCAAAGGATGTTAAGGATATA
TP89757_Query	D+G	1	chr1	.	CTGCTGACAAGTTCACTTCTTTATTGGAGTATAGTTGACTTGTGCAAAGGATGTTAAGGATATA
TP89779_Hit	D+G	1	chr1	.	CTGCTGACCACTCAATCACAAAGGACAAGTGTACCATCCAGGTTATGGACAAAGCCTGTGTCCC
TP89779_Query	D+G	1	chr1	.	CTGCTGACCACTCAATCACAAAGGACAAGTGTACCATCCAGGTTATGGACAAAGCCAGTGTCCC

Name	Filter	Nb hit (Mt4.0)	Mt Chr (Mt4.0)	Ms Chr (Li et al., 2014)	Sequence
TP89824_Hit	D+G	1	chr1	.	CTGCTGACTCGTTGGTTCAATAGCTCACAACATGTATAGTGTTAACGTGTGTGCTTCTGATCCA
TP89824_Query	D+G	1	chr1	.	CTGCTGACTCGTTGGTTCAATAGCTCACAACATGTATAGTGTCAACGTGTGTGCTTCTGATCCA
TP89910_Hit	D	1	chr1	.	CTGCTGAGCTTATGTTAGAGAATCCAAATTATTTCTTAGTCAACTCTCGATCTCTTCATATTTC
TP89910_Query	D	1	chr1	.	CTGCTGAGCTTATGTTAGAGAATCCAAATTATTTCCTAGTCAACTCTCGATCTCTTCATATTTC
TP90196_Hit	D	1	chr1	.	CTGCTGATGCAGATGATGGTGTATTCCCCGTTGCCTTTGCCATTGTTGATGCTGAATCCGATGA
TP90196_Query	D	1	chr1	.	CTGCTGATGCAGATGATGGTGTATTCCCCGTTGCCTTTGCCATTGTTGACGCTGAATCCGATGA
TP90199_Hit	D	1	chr1	.	CTGCTGATGCATAATCAACAACATTTTCGGATATTCGAGAAAGACATTCTTGTCCTCCCCCAGT
TP90199_Query	D	1	chr1	.	CTGCTGATGCATAATCAACAACATTTTCGGATATTCGAGAAAGACATTCTTGTCCTCCACCAGT
TP90350_Hit	D	1	chr1	.	CTGCTGGAAAGTACTGGATCAAGAAGTGGGCGACAATCAATGAGTGTTACTACCGCATATTTTT
TP90350_Query	D	1	chr1	.	CTGCTGGAAAGTACTGGATCAAGAAGTGGGCGACAATCAATGAGTGTTACTACAGCATATTTTT
TP904_Hit	D	1	chr1	.	CAGCAAAATCCATTCTTGAATAGGCAAATAGGTGTTGAGTACTTTACAAACCAAATTCTTGTTC
TP904_Query	D	1	chr1	.	CAGCAAAATCCATTCTCGAATAGGCAAATAGGTGTTGAGTACTTTACAAACCAAATTCTTGTTC
TP90555_Hit	D+G	1	chr1	.	CTGCTGGATGTCCTAGACGAGAATGCCATAACTCGGTAGATGAAGCGGCAAAGGCTTATGGTGG
TP90555_Query	D+G	1	chr1	.	CTGCTGGATGTCCTAGACGACAATGCCATAACTCGGTAGATGAAGCGGCAAAGGCTTATGGTGG
TP90615_Hit	D	1	chr1	.	CTGCTGGCCAGAATGGATTTATCCTAGCAATTTGCTGGTTATACAATATCTTTTGAGCAATGTG
TP90615_Query	D	1	chr1	.	CTGCTGGCCAGAATGGATTCATCCTAGCAATTTGCTGGTTATACAATATCTTTTGAGCAATGTG
TP90824_Hit	D	1	chr1	.	CTGCTGGGAGGATCCTTCACCATGATTGAGGGTTAGTCTTAGGGTTGATGCAACACTACCCTTC
TP90824_Query	D	1	chr1	.	CTGCTGGGAGGACCCTTCACCATGATTGAGGGTTAGTCTTAGGGTTGATGCAACACTACCCTTC
TP90897_Hit	D	1	chr1	.	CTGCTGGGGGCAGAAGAGGACCGCTGTACTTCAGTGGATCATAAAAGGGATTACAATGCACATG
TP90897_Query	D	1	chr1	.	CTGCTGGGGGCAGAAGAGGACCACTGTACTTCAGTGGATCATAAAAGGGATTACAATGCACATG
TP91226_Hit	D	1	chr1	.	CTGCTGGTGTTGCTTTCTTTTCCTCACAAACTCAGTTTCTGGAATACTCAAATGAGGGTGTTCT
TP91226_Query	D	1	chr1	.	CTGCTGGTGTTGCTTTCTTTTCCGCACAAACTCAGTTTCTGGAATACTCAAATGAGGGTGTTCT
TP91257_Hit	D+G	1	chr1	.	CTGCTGGTTCTGCTGATGTTGTTGGGTGTTCATCGGAGCCGAAAACTGCTGTGCTGACTGCTGA
TP91257_Query	D+G	1	chr1	.	CTGCTGGTTCTGCTGATGTTGTTGAGTGTTCATCGGAGCCGAAAACTGCTGTGCTGACTGCTGA
TP9143_Hit	D	1	chr1	.	CAGCACAATCTAATTTGTTTGGAACTATCACAAAAGGCCTCGTTGACTCTTCAAAGAATGCGGT
TP9143_Query	D	1	chr1	.	CAGCACAATCTAATTTGTTTGGAACTATCACAAAAGGCCTCGTTGACTCTTCAAAGAATGCAGT
TP9148_Hit	D	1	chr1	.	CAGCACAATCTCGCATAAGCAACTATCATTTTCTGTGATATTGCACTTTCTACAAAAATCTTCC
TP9148_Query	D	1	chr1	.	CAGCACAATCTCGCATAAGCAACTATCATTTTCTGTAATATTGCACTTTCTACAAAAATCTTCC
TP91675_Hit	D+G	1	chr1	.	CTGCTGTCCATGAATTTCGAATTCCTCCCAAAGCTCTTTTAGGACTGCATTCATATTGTCCTGT
TP91675_Query	D+G	1	chr1	.	CTGCTGTCCATGAATTTCGAATTCCTCCCAAAGCTCTTTTAGGACTGCATTCATACTGTCCTGT
TP91747_Hit	D	1	chr1	.	CTGCTGTCGGGGTCAAATCCATAATTTAGTCTAAGTCTTCACTTTATTGCTTGTTTTCAATTTA
TP91747_Query	D	1	chr1	.	CTGCTGTCGGGGTCAAATCCATAATTTAGTCTAAGTCTTCACTTTATTACTTGTTTTCAATTTA
TP91854_Hit	D+G	1	chr1	.	CTGCTGTGACCGTGAGATTTCTTGGTTCTCTCTTTATCAGTTTCGTGAGAAACATGGTCCGGAC
TP91854_Query	D+G	1	chr1	.	CTGCTGTGACCGTGAGATTTCTTGATTCTCTCTTTATCAGTTTCGTGAGAAACATGGTCCGGAC
TP91922_Hit	D+G	1	chr1	.	CTGCTGTGCCTGCGATCCCAGTGCCTATGCCTCAGAAATCACAGCCTGCCCAGGCTAATTGGTG
TP91922_Query	D+G	1	chr1	.	CTGCTGTGCCTGCGATCCCAGTGCCTACGCCTCAGAAATCACAGCCTGCCCAGGCTAATTGGTG
TP91978_Hit	D	1	chr1	.	CTGCTGTTGATTTAAATCGAAAGCCTGCTATTGATTTTTCTGCGACAATTGGTACTCCTGGTAT
TP91978_Query	D	1	chr1	.	CTGCTGTGGATTTAAATCGAAAGCCTGCTATTGATTTTTCTGCGACAATTGGTACTCCTGGTAT
TP92119_Hit	D+G	1	chr1	.	CTGCTGTTACCAGTGGCGTTTTACTCTCCGATCAAGGTCTTCCTACTGTTGTTGCTAGGAATAC
TP92119_Query	D+G	1	chr1	.	CTGCTGTTACCAGTGGCGTTTTACTCTCCGATCAAGGTCTTCCCACTGTTGTTGCTAGGAATAC
TP9221_Hit	D	1	chr1	.	CAGCACACAACTGGTCCACACATTGCACAAGGTTGTTGTTGGGTTTATAAAGTTGATCACGGTG
TP9221_Query	D	1	chr1	.	CAGCACACAACTGGTCCACACATTGCACAAGGTTCTTGTTGGGTTTATAAAGTTGATCACGGTG
TP92276_Hit	D+G	1	chr1	.	CTGCTGTTCTATCAACGCCGCTCCGCTTGGTGACTACAAATGTTTATGGAATGGCGGCAACTGC
TP92276_Query	D+G	1	chr1	.	CTGCTGTTCTATCAACGCCGCTCCGCTTAGTGACTACAAATGTTTATGGAATGGCGGCAACTGC
TP92405_Hit	D	1	chr1	.	CTGCTGTTGCTGATTCTCGATCGTTCCGCGTCTTTGGTGAGCCTGAGCACGTTTGAAAGCTGAA
TP92405_Query	D	1	chr1	.	CTGCTGTTGCTGATTCTCGATCGTTCCGCGTCGTTGGTGAGCCTGAGCACGTTTGAAAGCTGAA
TP9245_Hit	D	1	chr1	.	CAGCACACACCATCAAAGTTAGCCACACACCCATGAATCTTTGATGTAAAACACGTAAGCACGC
TP9245_Query	D	1	chr1	.	CAGCACACACCATCAAAGTTAGCCACACACCCATGAATCTTTGATGTAAAACACGTAAGCACAC
TP92489_Hit	D+G	1	chr1	.	CTGCTGTTGTTAATGTGTGACTTCCATGTCCATCATCATCTCTAGCTGATTTTGATTCTGTTGT
TP92489_Query	D+G	1	chr1	.	CTGCTGTTGTTAATGTGTGACTTCCATGTCCATCATCATCTCTAGCTGATCTTGATTCTGTTGT
TP92542_Hit	D	1	chr1	.	CTGCTGTTTCGGCTTCCGCATGGGTGGAACCCCATGGAAAATGTACTGTTGCATTTAGTCTTGC
TP92542_Query	D	1	chr1	.	CTGCTGTTTCAGCTTCCGCATGGGTGGAACCCCATGGAAAATGTACTGTTGCATTTAGTCTTGC
TP92628_Hit	D	1	chr1	.	CTGCTGTTTTCAATTCGGTACTTTTCTAATATTGGTATGTTGCTCTTTTTTACTATTTCCTTTG
TP92628_Query	D	1	chr1	.	CTGCTGTTTTCAATTCGGTACTTTTCTAATATTGGTATATTGCTCTTTTTTACTATTTCCTTTG

Name	Filter	Nb hit (Mt4.0)	Mt Chr (Mt4.0)	Ms Chr (Li et al., 2014)	Sequence
TP9264_Hit	D+G	1	chr1	.	CAGCACACAGTTGAAGTTGGTTACCATTTATGCAAATCGAATCTCACATGCAAGGACAGGAAAA
TP9264_Query	D+G	1	chr1	.	CAGCACACAGTTGAAATTGGTTACCATTTATGCAAATCGAATCTCACATGCAAGGACAGGAAAA
TP92672_Hit	D	1	chr1	.	CTGCTGTTTTTCCACTTCATCTTGAAACTGATGTGCTTGTTGCATAGGATCTACTTCCTCTTGT
TP92672_Query	D	1	chr1	.	CTGCTGTTTTTCCACTTCATCTTGAAACTGATGTGCTTGTTGCATAGGATCTACTTCCTCTCGT
TP92723_Hit	D	1	chr1	.	CTGCTTAAAGGGCATCATAGGATGTACACACCAAAGGGTTCGCAGAACAACGATACACACACCA
TP92723_Query	D	1	chr1	.	CTGCTTAAAGGGCATCATAGGATGTACACACCAAAGGGTTCGCAGAACAACAATACACACACCA
TP92823_Hit	D	1	chr1	.	CTGCTTACATTTACATCAGGGGTGAATATGTGAATGAACGTTTAAATCTTGAAAAGGCTAGGAA
TP92823_Query	D	1	chr1	.	CTGCTTACATCTACATCAGGGGTGAATATGTGAATGAACGTTTAAATCTTGAAAAGGCTAGGAA
TP92887_Hit	D+G	1	chr1	.	CTGCTTAGAAAAATAAGAATACTTTGTAGAAATGGTACTTTGCAAGAATGTTAAATTTATATAA
TP92887_Query	D+G	1	chr1	.	CTGCTTAGAAAAATAAGAATACTTTGTAGAAATGGTACTTTGCAAGAATGTTAAATTTATAGAA
TP93397_Hit	D	1	chr1	.	CTGCTTCATTGTTCCGGCTACTACCTTCTGATCTTCACTTCCAATCCGAGCAAACCCAACACAC
TP93397_Query	D	1	chr1	.	CTGCTTCATTGTTCCGGCTACTACATTCTGATCTTCACTTCCAATCCGAGCAAACCCAACACAC
TP93414_Hit	D	1	chr1	.	CTGCTTCCAAGAGTCATAGTGAAGCTGAAAGGAGACGCAGAGAGAGAATCAATAACCATCTTGC
TP93414_Query	D	1	chr1	.	CTGCTTCCAAGAGTCATAGTGAAGCTGAAAGGAGACGCAGAGAAAGAATCAATAACCATCTTGC
TP9345_Hit	D	1	chr1	.	CAGCACACCTTCCTTCACGGTCCACAACTACGCACCCGACTGTTTCAGGTGCGTAGACACTCAT
TP9345_Query	D	1	chr1	.	CAGCACACCTTCCTTCACGGTCCACAACTACGCACCCGACCGTTTCAGGTGCGTAGACACTCAT
TP93458_Hit	D	1	chr1	.	CTGCTTCGATGCAATCGGCGGCAATGAAAAATGAGAGAGATGGAATTGTAGGGCATAATGATAT
TP93458_Query	D	1	chr1	.	CTGCTTCCATGCAATCGGCGGCAATGAAAAATGAGAGAGATGGAATTGTAGGGCATAATGATAT
TP9346_Hit	D	1	chr1	.	CAGCACACCTTCCTTCACTGTCCACCACCACACACCCGACGGTCTCTGGAGAATAAGCGCTTAG
TP9346_Query	D	1	chr1	.	CAGCACACCTTCCTTCACTGTCCACCACAACACACCCGACGGTCTCTGGAGAATAAGCGCTTAG
TP9353_Hit	D	1	chr1	.	CAGCACACGCACACATGCGGCCATCAATATCAGAAGTAGTGAAGGAAATTCAAGACGCAATTGG
TP9353_Query	D	1	chr1	.	CAGCACACGCACACATGCGGCCATCAATATCAGAAGTAATGAAGGAAATTCAAGACGCAATTGG
TP93617_Hit	D	1	chr1	.	CTGCTTCGATATGGCACGCGATTACTCCTTGCGGCGGCGATGGATTCCGAAGCTGAAAAAAAAA
TP93617_Query	D	1	chr1	.	CTGCTTCGATATGGCACGCGATTACTCCTTGCGGCGGCGATGGATTCCGAAGCAGAAAAAAAAA
TP93641_Hit	D	1	chr1	.	CTGCTTCGGAATCCATCGCCGCCGCAAGGAGTAATCGCGTGCCATATCGAAGCTGAAAAAAAAA
TP93641_Query	D	1	chr1	.	CTGCTTCGGAATCCATCGCCGCCGCAAGGAGTAATCGCGTGCCATATCGAAGCAGAAAAAAAAA
TP93709_Hit	D	1	chr1	.	CTGCTTCTACAAGACACAGACACACCTTTTGGAACAAGTTGAATCTCTTCACCTCCCTCTTCTT
TP93709_Query	D	1	chr1	.	CTGCTTCTACAAGACACAGACACACCTTTTGGAACAAGTTGAATCTCTTCACCTACCTCTTCTT
TP94286_Hit	D+G	1	chr1	.	CTGCTTGTCTACTTCCTATGCTCCTATTTTCGTGTTTTTCAGTTACAAGAGATTCAATAATTGT
TP94286_Query	D+G	1	chr1	.	CTGCTTGCCTACTTCCTATGCTCCTATTTTCGTGTTTTTCAGTTACAAGAGATTCAATAATTGT
TP94571_Hit	D	1	chr1	.	CTGCTTGTCTTGAATATATATAGCCTATGGTGTGTAACATGTCAACTCCAAACTCTGCAAACAG
TP94571_Query	D	1	chr1	.	CTGCTTGTCTTGAATATATATAGCCTATGGTATGTAACATGTCAACTCCAAACTCTGCAAACAG
TP94580_Hit	D+G	1	chr1	.	CTGCTTGTGGTGGTAGAGCCAGAAGGTGAAGAGCTACCAACTATGGCGTTGCTGATGGTGTTGG
TP94580_Query	D+G	1	chr1	.	CTGCTTGTGATGGTAGAGCCAGAAGGTGAAGAGCTACCAACTATGGCGTTGCTGATGGTGTTGG
TP94852_Hit	D	1	chr1	.	CTGCTTTATCCTCTGAGTTAAAGAAATCCGGTAGAGCTTCAAATACTTCGAAGAAGGCAGGGCT
TP94852_Query	D	1	chr1	.	CTGCTTTATCCTCCGAGTTAAAGAAATCCGGTAGAGCTTCAAATACTTCGAAGAAGGCAGGGCT
TP94959_Hit	D	1	chr1	.	CTGCTTTCCATTAATTAGAATATGTTGTAAATTTGTATTGCTATATGACTTGGTATGATTTTTT
TP94959_Query	D	1	chr1	.	CTGCTTTCCATTAATTAGAATATGTTGTAAATTTGTATTGCTATATGACTTAGTATGATTTTTT
TP95166_Hit	D	1	chr1	.	CTGCTTTGATTATCGCAAGAGCAGGGATACAATCAATCTTACTGATTTACATTCTTCAAAGGGG
TP95166_Query	D	1	chr1	.	CTGCTTTGATTATCGCAAGAGCAGGGATACAATCAATCTCACTGATTTACATTCTTCAAAGGGG
TP95202_Hit	D	1	chr1	.	CTGCTTTGCCGCCGGCACCGATCATGGCTTCCGCATCTACAACTGTGACCCCTTCCGCGAAATT
TP95202_Query	D	1	chr1	.	CTGCTTTGCCGCCGGCACCGATCATGGCTTCCGCATCTACAACTGCGACCCCTTCCGCGAAATT
TP95316_Hit	D	1	chr1	.	CTGCTTTGTAGAACTTGTGTAATAGTAGTATATAATGTGTTAACTATTGCATGCAATTGCAAAA
TP95316_Query	D	1	chr1	.	CTGCTTTGTAGAACTTGTGTAATAGTACTATATAATGTGTTAACTATTGCATGCAATTGCAAAA
TP95561_Hit	D	1	chr1	.	CTGCTTTTGAGTGTCCAATGTGAAGATAGCCACTGGGTTCAGGTGCAAATCGCAAGCGAACTTT
TP95561_Query	D	1	chr1	.	CTGCTTTTGAGTGTCCAATGTGAAGATAGCCACTGGGTTCAGGTGCAAACCGCAAGCGAACTTT
TP9664_Hit	D	1	chr1	.	CAGCACATACTCCTAATCTATGTTTCTTATTGATTTAAGTGTATCGCAATCTTTAACACTGTGC
TP9664_Query	D	1	chr1	.	CAGCACATACTCCTAATCTATGTTTCATATTGATTTAAGTGTATCGCAATCTTTAACACTGTGC
TP9690_Hit	D	1	chr1	.	CAGCACATATAGTGAATTGCTATTTTATCTTTTACTGCTTAGTCAGAATGGAATAAAATGGACT
TP9690_Query	D	1	chr1	.	CAGCACATATAGTGAATTGCTATTTTATCTTTTACTGCTTAGTCAGAATGGAATAAAATAGACT
TP9797_Hit	D	1	chr1	.	CAGCACATGACTTGTAGTTGTACAGACCTGATTCTTGAAACTCTAAAGCATATCATCTATCAAA
TP9797_Query	D	1	chr1	.	CAGCACATGACTTGTAGTTGTACAGACCTGATTCTTGAAACTCCAAAGCATATCATCTATCAAA
TP9983_Hit	D	1	chr1	.	CAGCACCAAAAACATATGCTAGATAGTCCAAACAAGTTAGACATAAAATGGAAATACTAAGTAG
TP9983_Query	D	1	chr1	.	CAGCACCAAAAACATATGCTAGATAGTCCAAACAAGTTAGACATAAAATGGAAATACCAAGTAG

Name	Filter	Nb hit (Mt4.0)	Mt Chr (Mt4.0)	Ms Chr (Li et al., 2014)	Sequence
TP12009_Hit	D+G	1	chr1	1A	CAGCACTCACAGATCAAGATACTTGCCTTGATGGATTTCAAGGCAAAAACGGTAGGACAATTCA
TP12009_Query	D+G	1	chr1	1A	CAGCACTCACAGATCAAGATACTTGCCTTGATGGATTTCAAGGCAAAAACAGTAGGACAATTCA
TP13807_Hit	D	1	chr1	1A	CAGCAGAGGAAAATTATTGGAGTTTAACCTGCCGGGAGGAATTCCAGAATCTTGGCCGAAGTTG
TP13807_Query	D	1	chr1	1A	CAGCAGAGGAAAATTATTGGAGTTTAACCTGCCGGGAGGAATCCCAGAATCTTGGCCGAAGTTG
TP13952_Hit	D	1	chr1	1A	CAGCAGAGTGCTTATCCTCGTAATCATCAAGATTAAGAGCATGTAGCAACTTCGGCCAAGATTC
TP13952_Query	D	1	chr1	1A	CAGCAGAGTGCTTATCCTCATAATCATCAAGATTAAGAGCATGTAGCAACTTCGGCCAAGATTC
TP18407_Hit	D+G	1	chr1	1A	CAGCATCAATCTTGAACAACTCTCTCAAATTTCTGCAAAAGGTCACAGGGTCATATATAACAAG
TP18407_Query	D+G	1	chr1	1A	CAGCATCAATCTTGAACAACTCTCTCAAATTTCTGCAAAAGGTCACAAGGTCATATATAACAAG
TP24228_Hit	D+G	1	chr1	1A	CAGCCACTAATAAAGCCCACACAAACCACGGATCCTCACACGTCCTACTAAAAAACATCATCCA
TP24228_Query	D+G	1	chr1	1A	CAGCCACAAATAAAGCCCACACAAACCACGGATCCTCACACGTCCTACTAAAAAACATCATCCA
TP24994_Hit	D+G	1	chr1	1A	CAGCCACCTTTAAGTAATTTACCTCAGAAGTAGCTTTCTCTATGTTAAGCTTTACTTCTTCAAG
TP24994_Query	D+G	1	chr1	1A	CAGCCACCTTTAAGTAATTTACCTCAGAAGTAGCTTTCTCTATGCTAAGCTTTACTTCTTCAAG
TP26214_Hit	D	1	chr1	1A	CAGCCATAAAATGGGGAACAGACTACTTGATGAAAGCTCATCAACAACCAAATGTTTTGTATGG
TP26214_Query	D	1	chr1	1A	CAGCCATAAAATGGGGAACAGACTACTTGATGAAAGCACATCAACAACCAAATGTTTTGTATGG
TP28274_Hit	D+G	1	chr1	1A	CAGCCCATGTCCTCCACTGATGTGAATAGAATCTAAAGGTTTGTCTCAGTTGTTTGCTATGTAA
TP28274_Query	D+G	1	chr1	1A	CAGCCCATGTCCTCCACTGATGTGAATAGAATCTAAAGGTTTGTCGCAGTTGTTTGCTATGTAA
TP30936_Hit	D	1	chr1	1A	CTGCCGGAGTGTGGAAGAATCTGAAGGAAAGATTTTCCCAATGGAGATATTGTCAGAATCGCTG
TP30936_Query	D	1	chr1	1A	CAGCCGGAGTGTGGAAGAATCTGAAGGAAAGATTTTCCCAATGGAGATATTGTCAGAATCGCTG
TP339_Hit	D+G	1	chr1	1A	CAGCGAAACAAGGTTGGAAGCCCACCTTTGTTGACTTGGATATTGGCCAAGGGTCTATAACAAT
TP339_Query	D+G	1	chr1	1A	CAGCAAAACAAGGTTGGAAGCCCACCTTTGTTGACTTGGATATTGGCCAAGGGTCTATAACAAT
TP3472_Hit	D	1	chr1	1A	CAGCAACAGCATCTTCGGCTTTGTTAATAGCTTCGCCTTTTTCATCAACTAAAGAAGCGTATTC
TP3472_Query	D	1	chr1	1A	CAGCAACAGCATCTTCGGCTTTGTTAATAGCTTCACCTTTTTCATCAACTAAAGAAGCGTATTC
TP43745_Hit	D+G	1	chr1	1A	CAGCTAGAACTGGAGCTTTTCAAACTTCATTTACATAAAGTGGTTACATTGTTTTTACACTAAA
TP43745_Query	D+G	1	chr1	1A	CAGCTAGAACTGGAGCTTTTCAAACTTCATTTACATAAAGTGATTACATTGTTTTTACACTAAA
TP46990_Hit	D	1	chr1	1A	CAGCTCCAACAACGTATGAGTAAAATACGAAACATCGTGACCATTGACATGACCATAATAGCAA
TP46990_Query	D	1	chr1	1A	CAGCTCCAACAACATATGAGTAAAATACGAAACATCGTGACCATTGACATGACCATAATAGCAA
TP521_Hit	D	1	chr1	1A	CAGCAAAACTTCCCACTTCTTTGTAAATATCAACTCTTGTTACATTGTTTCCATTGATAAACAC
TP521_Query	D	1	chr1	1A	CAGCAAAACTTCCCACTTCTTTGTAAATATCAACTCTTGTTACATTATTTCCATTGATAAACAC
TP5228_Hit	D	1	chr1	1A	CAGCAAGACCTACTGGGTTGGAGAACTTTCTTCCCCAAACCTCTAAACCTAAAATTTGTGGGTC
TP5228_Query	D	1	chr1	1A	CAGCAAGACCTACTGGGTTGGAGAACTTTCTTCCCCAAACCTCTAAACCTAAAATTGGTGGGTC
TP5452_Hit	D	1	chr1	1A	CAGCAAGATCAGAGCCAGGATGTTGTTCATCAATCCTATAAGAATTTCTAGGTGTTGACATATC
TP5452_Query	D	1	chr1	1A	CAGCAAGATCAGAGCCAGGATGTTGTTCATCAATCCTATAAGAATTCCTAGGTGTTGACATATC
TP57578_Hit	D	1	chr1	1A	CAGCTTGCTTCAAGTTACCTGCAAACCTATTAAGTCTATCCAACAAATTCTTTGTTAAAGTGCT
TP57578_Query	D	1	chr1	1A	CAGCTTGCTTCAAGTTACCTGCAAACCTATTAAGTCTATCCAACAAATTCTTTGTCAAAGTGCT
TP59466_Hit	D+G	1	chr1	1A	CAGCTTTGCAGTCACAACCTCATATCATGCATTTAGTGTAAAAACAATGTAATCACTTTATGTA
TP59466_Query	D+G	1	chr1	1A	CAGCTTTGCAGTCACAACCTCATATCATGCATTTAGTGTAAAAACAATGTAACCACTTTATGTA
TP62553_Hit	D	1	chr1	1A	CTGCAACGCTCCCTAATCTCACCTTCAGAACCTGTCAGCACATCTAAGGAACCCATATGCACCA
TP62553_Query	D	1	chr1	1A	CTGCAACGCTCCCTAATCTCACCTTCAGAACCTGACAGCACATCTAAGGAACCCATATGCACCA
TP62662_Hit	D	1	chr1	1A	CTGCAACTATAGCTAGAATTTTTCCTAGTTTATTTGGAGGTGAGTGTGATGGTGACTTTGGACA
TP62662_Query	D	1	chr1	1A	CTGCAACTATAGCTAGAATTTTTCCTAGTTTATTAGGAGGTGAGTGTGATGGTGACTTTGGACA
TP64292_Hit	D	1	chr1	1A	CTGCAATGATGTGGAAAATCATCAAATAATCCACTAATTACAGATGGATGCAACTTTCTGATTA
TP64292_Query	D	1	chr1	1A	CTGCAATGATGAGGAAAATCATCAAATAATCCACTAATTACAGATGGATGCAACTTTCTGATTA
TP69273_Hit	D	1	chr1	1A	CTGCAGGGCTTCCTGGGTACCATCCAGGGGCACCAAGACTTGCCCCTCAGCAGTTGTATTATGG
TP69273_Query	D	1	chr1	1A	CTGCAGGGCTTCCTGGGTACCATCCAGGGGCACCAAGACTTGCCCCTCAGCAGTTGTATTACGG
TP71336_Hit	D+G	1	chr1	1A	CTGCGTCGCCGGGCCGTGGAGGAAGTAAGAACTCACACATAAGGAAAACAAGAAGTGCTCAATT
TP71336_Query	D+G	1	chr1	1A	CTGCATCGCCGGGCCGTGGAGGAAGTAAGAACTCACACATAAGGAAAACAAGAAGTGCTCAATT
TP75426_Hit	D+G	1	chr1	1A	CTGCCATGCTGATTGGTCGGTGCCGAAGCCGTGAGCGAAGACGATGTATTTGTCGCCGGAGCCT
TP75426_Query	D+G	1	chr1	1A	CTGCCATGCTGATTGGTCGGTGCCGAAGCCGTGAGCGAAGACGATGTATTTGTCGCCGGAACCT
TP82897_Hit	D	1	chr1	1A	CTGCGGTTCCAAGGGTTGGGATCAAAGGTTATGAGTGGTGGTCAGAGGCACTTCATGGAGTTTC
TP82897_Query	D	1	chr1	1A	CTGCGGTTCCAAGGGTTGGGATCAAAGGCTATGAGTGGTGGTCAGAGGCACTTCATGGAGTTTC
TP95474_Hit	D	1	chr1	1A	CTGCTTTTCGAGCTCTAAATTATGGGAAAGAAATGGAGCGTAATCATCACATGTCTGAAGCATC
TP95474_Query	D	1	chr1	1A	CTGCTTTTCGAGCTCTAAATTATGGGAAAGAAATGGAGCGTAATCATCACATGTCAGAAGCATC
TP95691_Hit	D	1	chr1	1A	CTGCTTTTTCATCAAAGATATCTTCAAATATTGACCGCCCTTTTGGAGATGAGGTATTTCAAAC
TP95691_Query	D	1	chr1	1A	CTGCTTTTTCATCAAAGATATCTTCAAATATTGACCGCCCTTTTGGAGATGAGGTAATTCAAAC

Name	Filter	Nb hit (Mt4.0)	Mt Chr (Mt4.0)	Ms Chr (Li et al., 2014)	Sequence
TP9732_Hit	D	1	chr1	1A	CAGCACATCATTAGGCTAATAAATCCTGACAAAACAGAACATGTCAAAGTAATCAGTTCTGCGG
TP9732_Query	D	1	chr1	1A	CAGCACATCATTAGGCTAATAAATCCTGACAAAACAGAACATGTCAAAGTAATCAGTTCTGCGA
TP16926_Hit	D	1	chr1	1B	CAGCATAAAAACAACGCGGCCATCCTTAGTTTTAGGAACAATTAAGCCCATACCCTCAGGAGAA
TP16926_Query	D	1	chr1	1B	CAGCATAAAAACAACGCGCCCATCCTTAGTTTTAGGAACAATTAAGCCCATACCCTCAGGAGAA
TP2119_Hit	D+G	1	chr1	1B	CAGCAAATATGGAACGATATTGGAGAGAGCGAGAAGGACAAAGACCGTATGTTGATGGAGCTGG
TP2119_Query	D+G	1	chr1	1B	CAGCAAATATGGAACGACATTGGAGAGAGCGAGAAGGACAAAGACCGTATGTTGATGGAGCTGG
TP24968_Hit	D+G	1	chr1	1B	CAGCCACCTGTTAGGCATAAGGACGGCACCTGCATGGCCACCGTTGCCTTCAAATTCAACCCTT
TP24968_Query	D+G	1	chr1	1B	CAGCCACCTGTTAGGCATAAGGACGGCACCTGCATGGCCACCATTGCCTTCAAATTCAACCCTT
TP25605_Hit	D+G	1	chr1	1B	CAGCCAGAGACTGTACTGGAAGCCCTCACTCATTGCTTGGAGCAGGTATGCTCTCCAGCTTACA
TP25605_Query	D+G	1	chr1	1B	CAGCCAGAGACTGTACTGGAAGCCCTCACTCATTGCTTAGAGCAGGTATGCTCTCCAGCTTACA
TP2712_Hit	D	1	chr1	1B	CAGCAACAAAATGAAACGAATATGCAGGTTGTTGAACGTGCCACACACCTACTTTGTGAATGGG
TP2712_Query	D	1	chr1	1B	CAGCAACAAAATGAAACGAATATGCAGGTTGTTGAACGTGCCACACACCTACTTTGTGAATGGA
TP29048_Hit	D+G	1	chr1	1B	CAGCCCGCCTGAAGATTGTGGCACAGTGTTAAAGATTACGATACACTTAAATCAGTAAGAAACA
TP29048_Query	D+G	1	chr1	1B	CAGCCCGCCTGAAGATTGTGACACAGTGTTAAAGATTACGATACACTTAAATCAGTAAGAAACA
TP42685_Hit	D	1	chr1	1B	CAGCTAATGAATCATCCCTCAAACATTGTCTTAGATCTTTCCTTAGATCAGATTTCTTCCTCTC
TP42685_Query	D	1	chr1	1B	CAGCTAATGAATCATCCCTCAAACATTGTCTCAGATCTTTCCTTAGATCAGATTTCTTCCTCTC
TP47872_Hit	D+G	1	chr1	1B	CAGCTCCTGCAAGTCTAAGGGTTGAATTTGAAGGCAATGGTGGCCATGCAGGTGCCGTCCTTAT
TP47872_Query	D+G	1	chr1	1B	CAGCTCCTGCAAGTCTAAGGGTTGAATTTGAAGGCAACGGTGGCCATGCAGGTGCCGTCCTTAT
TP5187_Hit	D	1	chr1	1B	CAGCAAGAATTTGTGCACCAAGATATTTGTCATCCCAACTAAACATTGTTCTTGCACCTCCATT
TP5187_Query	D	1	chr1	1B	CAGCAAGAATTTGTGCACCAAGATACTTGTCATCCCAACTAAACATTGTTCTTGCACCTCCATT
TP62416_Hit	D	1	chr1	1B	CTGCTACCCTTCTCTTTTTTGTCCTTAGGGTTTTCCATCAACCTGCTCCCCTAGGGAATAGTGA
TP62416_Query	D	1	chr1	1B	CTGCAACCCTTCTCTTTTTTGTCCTTAGGGTTTTCCATCAACCTGCTCCCCTAGGGAATAGTGA
TP7561_Hit	D	1	chr1	1B	CAGCAATCTTTGCAGAGACGGAATTCTTGCTGTTTTGTTTTTTCCTGAGCTTTTTGTTATGTTT
TP7561_Query	D	1	chr1	1B	CAGCAATCTTTGCAGAGACGGAATTCTTGCTGTTTTGTTTTTTCCTGAGCTTTTTGTTATGCTT
TP84956_Hit	D+G	1	chr1	1B	CTGCTACATTCTATTTTGATCTTCAAACTTCATCCATTAGTGAAGCATTGTTGGAAAATAAGGT
TP84956_Query	D+G	1	chr1	1B	CTGCTACATTCTATTTTGATCTTCAAACCTCATCCATTAGTGAAGCATTGTTGGAAAATAAGGT
TP86396_Hit	D	1	chr1	1B	CTGCTATGACCCTTGTTAAATCTGACATAGGTGGTAATATAACGGTATGTTTCTGTTAATTGTT
TP86396_Query	D	1	chr1	1B	CTGCTATGACCCTTGTTAAATCTGACATAGGTGGTAATATAACGGTATGTTTCTGTCAATTGTT
TP86797_Hit	D	1	chr1	1B	CTGCTATTGTAAGTGGGAACAAGCTATTTGTTGCTAATAGTGGTGATTGTCGGGCAATCTTATG
TP86797_Query	D	1	chr1	1B	CTGCTATTGTAAGTGGGAACAAGCTATTTGTTGCTAATAGTGGCGATTGTCGGGCAATCTTATG
TP87923_Hit	D	1	chr1	1B	CTGCTCCGAAACAATCTTTCTCAAATTTCGCTGTAGGTCTCATATCGTGAGCCCTCATCAGCAT
TP87923_Query	D	1	chr1	1B	CTGCTCCGAAACAATCTTTCTCAAATTTCGCTGTAGGTCTCATATCATGAGCCCTCATCAGCAT
TP9707_Hit	D	1	chr1	1B	CAGCACATATTCCTAATCTATGTTTCTTACTGATTTAAGTGTATCGTAATCTTTAACACTGTGT
TP9707_Query	D	1	chr1	1B	CAGCACATATTCCTAATCTATGTTTCTTACTGATTTAAGTGTATCGTAATCTTTAACACTGTGC
TP18861_Hit	D	1	chr1	1C	CAGCATCCAAAGTATCCTTCAGGTGATACAATGAAAGTGATTCTAGAGCTTCTTCTATGGCTTC
TP18861_Query	D	1	chr1	1C	CAGCATCCAAAGTATCCTTCAGGTGATACAATGAAAGTGATTCTAGAGCTTCTTCCATGGCTTC
TP40494_Hit	D	1	chr1	1C	CAGCGTCATTCGACAAAGTTGTATCTTTCCGCAGAATAATTTCTGAATTCCGGTTAGAGCAATC
TP40494_Query	D	1	chr1	1C	CAGCGTCATTCGACAAAGTTGTATCTTTCAGCAGAATAATTTCTGAATTCCGGTTAGAGCAATC
TP46089_Hit	D+G	1	chr1	1C	CAGCTCAATTATGCGGAAGTGGTGTCTCCGTTCGATCAATCGATAAGCCTCAAGCTTCATTAAC
TP46089_Query	D+G	1	chr1	1C	CAGCTCAATTATGCGGAAGTGGTGTCTCCATTCGATCAATCGATAAGCCTCAAGCTTCATTAAC
TP55951_Hit	D+G	1	chr1	1C	CAGCTTCTCTGTTGGCACGTCTAATGCTGGCTTTTAGTGATAGCATTCCCAAAGTAATAGACTC
TP55951_Query	D+G	1	chr1	1C	CAGCTTCTCTGTTGGCACGTCTAATGCTGGCTTTTAGTGATAGCATCCCCAAAGTAATAGACTC
TP59524_Hit	D	1	chr1	1C	CAGCTTTGCTCTTGTTTGGACGAGACAGAGCACATAAGGCTCGAGCAGACTGAGTGGCAACACC
TP59524_Query	D	1	chr1	1C	CAGCTTTGCTCTTGTTAGGACGAGACAGAGCACATAAGGCTCGAGCAGACTGAGTGGCAACACC
TP59808_Hit	D+G	1	chr1	1C	CAGCTTTTAAAGGTGAAAGAGAAATCAAAGAAGCCATGGAAGAAGCTCTAGAATCACTTTCATT
TP59808_Query	D+G	1	chr1	1C	CAGCTTTTAAAGGTGAAAGAGAAATCAAAGAAGCCATAGAAGAAGCTCTAGAATCACTTTCATT
TP6268_Hit	D	1	chr1	1C	CAGCAAGGTTTTGATTCACATCATTCAAAACGCATTCAATTTGTGCATACACATTTTTCTGTAC
TP6268_Query	D	1	chr1	1C	CAGCAAGGTTTTGATTCACATCATTCAAAACCCATTCAATTTGTGCATACACATTTTTCTGTAC
TP73154_Hit	D+G	1	chr1	1C	CTGCGTTTTATTTTTAAAATGAGGAAATTAAATACCTTCAAATTTTCCTCTGGACTAGGGAATA
TP73154_Query	D+G	1	chr1	1C	CTGCATTTTATTTTTAAAATGAGGAAATTAAATACCTTCAAATTTTCCTCTGGACTAGGGAATA
TP8124_Hit	D	1	chr1	1C	CAGCAATTAAATAAGAGAGACCATTCACTCGTGACATTAGAAGAAACAAAGTAGAAATATATCA
TP8124_Query	D	1	chr1	1C	CAGCAATTAAATAAGAGAGACCATTCACTCGTGACATTAAAAGAAACAAAGTAGAAATATATCA
TP83480_Hit	D	1	chr1	1C	CTGCGTGAGATGCTTCTGTTTATAACCTTGTGTTATTTGTTCTGGGCAGTTGATTGATGATAAT
TP83480_Query	D	1	chr1	1C	CTGCGTGAGATGCTTCTGTTTATAACCTTATGTTATTTGTTCTGGGCAGTTGATTGATGATAAT

Name	Filter	Nb hit (Mt4.0)	Mt Chr (Mt4.0)	Ms Chr (Li et al., 2014)	Sequence
TP8777_Hit	D	1	chr1	1C	CAGCACAAACATTTGTCCTCTTGAGTCGGTAAATGTTGTTTTGCTACATCCATACTGCAGTATA
TP8777_Query	D	1	chr1	1C	CAGCACAAACATTTGTCCTCTTGAGTCGGTAAATGTTGTTTTGCTACATCCATACTGCAATATA
TP95749_Hit	D+G	1	chr1	1C	CTGCTTTTTTGTATGATGAGACCATTGAATCATTTAATTGGCTGTTTCGAACTTTCATTGGCGC
TP95749_Query	D+G	1	chr1	1C	CTGCTTTTTTGTATGATGAGACCATCGAATCATTTAATTGGCTGTTTCGAACTTTCATTGGCGC
TP10706_Hit	D	1	chr1	1D	CAGCACCGATGCTGGCTCCGGCGTTGTTGGACACAACATAGTTGATTGCCTTAGCGCAATGAGC
TP10706_Query	D	1	chr1	1D	CAGCACCGATGCTGGCTCCGGCGTTGTTGGACACAACATAGTTGATTGCCTTAGCGCAATGAAC
TP16668_Hit	D	1	chr1	1D	CAGCAGTTGCAGGTGACCTAGATAAGGTAATCTTCAATTTTGACCATATGGTAAAACTTGGGCA
TP16668_Query	D	1	chr1	1D	CAGCAGTTGCAGGTGACCTAGACAAGGTAATCTTCAATTTTGACCATATGGTAAAACTTGGGCA
TP18748_Hit	D	1	chr1	1D	CAGCATCATCCATCTCTTTCCCTGGACATTCAATTTGGAGACACTCAGCAATTTCACTATCATA
TP18748_Query	D	1	chr1	1D	CAGCATCATCCATCTCCTTCCCTGGACATTCAATTTGGAGACACTCAGCAATTTCACTATCATA
TP21656_Hit	D	1	chr1	1D	CAGCATTGATACTGACAGATTTATCTAACTCTCCATAGTGACTAGATTGAGCGTATTTCCCATC
TP21656_Query	D	1	chr1	1D	CAGCATTGATACCGACAGATTTATCTAACTCTCCATAGTGACTAGATTGAGCGTATTTCCCATC
TP22869_Hit	D+G	1	chr1	1D	CAGCCAAACAGACGAAAAAGCAAATAGAAAAGAAGGAATATTTGGATAAACTAAAATCAGCAAT
TP22869_Query	D+G	1	chr1	1D	CAGCCAAACAGAAGAAAAAGCAAATAGAAAAGAAGGAATATTTGGATAAACTAAAATCAGCAAT
TP40811_Hit	D+G	1	chr1	1D	CAGCGTGAGTAATTCATTTGCCAGAGTAAAAAGCAGGGAAACAAGTTGTAATCAATGTACCAAA
TP40811_Query	D+G	1	chr1	1D	CAGCGTGAGTAATTCATTTGACAGAGTAAAAAGCAGGGAAACAAGTTGTAATCAATGTACCAAA
TP41437_Hit	D	1	chr1	1D	CAGCGTTGCGGAGTCCTAGTTGATCAATCAAAGCTTGAACACGTGCATTTTTCTTTGATTTTGA
TP41437_Query	D	1	chr1	1D	CAGCGTTGCGGAGTCCTAGTTGATCAATCAAAGCTTGAACACGAGCATTTTTCTTTGATTTTGA
TP50244_Hit	D+G	1	chr1	1D	CAGCTGAATTCCGTTTACCGCGATCACTCTCAAAATCAAAGAAAAATGCTCGTGTTCAAGCTTT
TP50244_Query	D+G	1	chr1	1D	CAGCTGAATTCCGTTTACCGCGATCACTCTCAAAATCAAAGAAAAATGCACGTGTTCAAGCTTT
TP5090_Hit	D	1	chr1	1D	CAGCAAGAAGAACAGTCACCACAAAAATGATTACAAAGTTATCAACAAAGAGCGCCGATAGAAT
TP5090_Query	D	1	chr1	1D	CAGCAAGAAGAACAGTCACCACAAAAATGATAACAAAGTTATCAACAAAGAGCGCCGATAGAAT
TP54293_Hit	D	1	chr1	1D	CAGCTTATTGCCTTGGAGGAAGAGAAAGTTGAGTTTCAGATCTCCTAAATCTAAAGGAGAGCCC
TP54293_Query	D	1	chr1	1D	CAGCTTATTGCCTTGGAGGAAGAGAAAGTTGAGTTTCAGATCTCCAAAATCTAAAGGAGAGCCC
TP62190_Hit	D+G	1	chr1	1D	CTGCAACAGGTAAAAGGAGCTAGGGGATTGATTATTTAGTGACTAACCATTGGAAACTACTGAA
TP62190_Query	D+G	1	chr1	1D	CTGCAACAGGTAAAAGGAGCTAGGGGATTGATTATTTAGTGACTAACCATTGGAAACTACGGAA
TP64716_Hit	D	1	chr1	1D	CTGCAATTGACGCCGCAATATCTGACGGTGTCGATGTTATTTCATTATCATTTGGAATTAATGA
TP64716_Query	D	1	chr1	1D	CTGCAATTGACGCCGCAATATCTGACGGAGTCGATGTTATTTCATTATCATTTGGAATTAATGA
TP69628_Hit	D	1	chr1	1D	CTGCAGTATCAAAGACACCATGGCCTCTATGGACCATGTGGTCATCCATAGGAATAACCATAGC
TP69628_Query	D	1	chr1	1D	CTGCAGTATCAAAGACACCATGGCCTCTATGAACCATGTGGTCATCCATAGGAATAACCATAGC
TP69849_Hit	D	1	chr1	1D	CTGCAGTGCTTTCAAAATACAAGTTTGATCTGGTGGCAGAAAGTAGATCATAGGAAGTGGGTGG
TP69849_Query	D	1	chr1	1D	CTGCAGTGCTTTCAAAATAAAAGTTTGATCTGGTGGCAGAAAGTAGATCATAGGAAGTGGGTGG
TP7053_Hit	D	1	chr1	1D	CAGCAATAGTGACTGTAAAAACCTTGATGTTGCAAACTCAAATAAAGTTGTTGACTGCAATTTA
TP7053_Query	D	1	chr1	1D	CAGCAATAGTGACTGTAAAAACCTTGATGTTGCAAACTCAAATAAAGTTGTAGACTGCAATTTA
TP73543_Hit	D	1	chr1	1D	CTGCCAACAACACAACTTCATCAAGGAGTACCATGTTGATGTTCCCTTTAAAGTCCAACTATAT
TP73543_Query	D	1	chr1	1D	CTGCCAACAACACAACTTCATCAAGGAGTACCATGTTGATGTTCCCTTTAAAGTCCAACAATAT
TP736_Hit	D	1	chr1	1D	CAGCAAAAGGTGTGTCCTTGAGAAACATGTGAAAAGAACAGGGGAGGAAGCATACACATGCAAA
TP736_Query	D	1	chr1	1D	CAGCAAAAGGTGTGTCCTTGAGAAACATGTGAAAAGAACAGGAGAGGAAGCATACACATGCAAA
TP76575_Hit	D+G	1	chr1	1D	CTGCCCGTTTCGACAAAGAAATAGGTGGAGCGTATGCGAAGCAAACACTCAGTTCAGCTGAAAA
TP76575_Query	D+G	1	chr1	1D	CTGCCCGTTTCGACAAAGAAATAGGTGGAGCGTATGCCAAGCAAACACTCAGTTCAGCTGAAAA
TP84849_Hit	D	1	chr1	1D	CTGCTACAGAACCACAAGTTGAAGAACCCCCGTCTCCTGCCTCTAGCACAACTTCAAGTCAGTT
TP84849_Query	D	1	chr1	1D	CTGCTACAGAACCACAAGTTGAAGAACCCCCATCTCCTGCCTCTAGCACAACTTCAAGTCAGTT
TP85179_Hit	D	1	chr1	1D	CTGCTACTAGGTTTTATATGTACATGTGTACTAACAAGTTGGTCAGTGTGGCGGTGAATGCTTT
TP85179_Query	D	1	chr1	1D	CTGCTACTAGGTTTTATATGTACATGTGTACTAACAAGTTGGTAAGTGTGGCGGTGAATGCTTT
TP88919_Hit	D+G	1	chr1	1D	CTGCTCTCTCAGCTTATCTGAATGAGGTAAACGTGATTGTCTCATGCTCCTTCATTGTACTTTA
TP88919_Query	D+G	1	chr1	1D	CTGCTCTCTCAGCTTATCTGAATGAGGTAAACGTGATCGTCTCATGCTCCTTCATTGTACTTTA
TP89750_Hit	D+G	1	chr1	1D	CTGCTGACAAATGAATCAGTATCATTTGTTTCATCTTCACAAAAGCCATCACATTGGACAGCAT
TP89750_Query	D+G	1	chr1	1D	CTGCTGACAAATGAATCAGTATCATTTGTTTCATCTTCACAAAAGCCATCACATTGAACAGCAT
TP90798_Hit	D+G	1	chr1	1D	CTGCTGGCTTTGGTCCTCTGCTTTGATTATCGCAAGAGCAGGGATACAATCAATCTTACTGATT
TP90798_Query	D+G	1	chr1	1D	CTGCTGGCTTTGGTCCTCTGCTTTGATTATCGCAAGAGCAGGGATACAATCAATCTCACTGATT
TP93577_Hit	D	1	chr1	1D	CTGCTTCCTCTTTTGCTTTTTGCGATCCTGTCTCTAACAACTGCACCAATGGTGGAATCCCTCC
TP93577_Query	D	1	chr1	1D	CTGCTTCCTCTTTTGCTTTTTGCGATCCTGTCTCTAACAACTGCACCAACGGTGGAATCCCTCC
TP12535_Hit	D	1	chr1	4D	CAGCACTTCTCATTCTAAGATCTTGGGTTTGGGAGGGTTGTTTGCCAGCTTTTCCAGTACAGAT
TP12535_Query	D	1	chr1	4D	CAGCACTTCTCATTCTAAGATCTTGGCTTTGGGAGGGTTGTTTGCCAGCTTTTCCAGTACAGAT

Name	Filter	Nb hit (Mt4.0)	Mt Chr (Mt4.0)	Ms Chr (Li et al., 2014)	Sequence
TP80667_Hit	D	1	chr1	5B	CTGCGATTACTAACAATGCTTTATCCATTTGAAAGTAATTGGTTATTTGATCCAGATAAAAAAC
TP80667_Query	D	1	chr1	5B	CTGCGATTACTAACAATGCTTGATCCATTTGAAAGTAATTGGTTATTTGATCCAGATAAAAAAC
TP25838_Hit	D	1	chr1	7B	CAGCCAGCCTTAAAAGTGTGAAGATATTGAAGCAATTTTGGGGTGATGTTGAGGATGATGACTC
TP25838_Query	D	1	chr1	7B	CAGCCAGCCTTAAAAGTGTGAAGATATTGAAGCAATTTTGGGGTGATGTTGAAGATGATGACTC
TP71888_Hit	D	1	chr1	7B	CTGCATGCTCCATATCAGTGTTAGTGTCTGAGGCAGAGTCATCATCTTCAACATCACCCCAAAA
TP71888_Query	D	1	chr1	7B	CTGCATGCTCCATATCAGTGTTAGTGTCTGAGGCAGAGTCATCATCCTCAACATCACCCCAAAA
TP42593_Hit	D+G	1	chr1	8B	CAGCTAATAGCAACATTTATTGGGGGTTTTGTGATAGCATTTACCAAAGGGTGGCTTCTTACTG
TP42593_Query	D+G	1	chr1	8B	CAGCTAATAGCAACATTTATTGGGGGTTTTGTGATAGCATTTACAAAAGGGTGGCTTCTTACTG
TP10148_Hit	D+G	1	chr2	.	CAGCACCAATGGCAATGATGGACATTATGGGGCTGAATCTGTCTAGCTAGATGCCTGAACCTAC
TP10148_Query	D+G	1	chr2	.	CAGCACCAATGGCAATGATGGACATTATGGGGCTGAATCTGTCTAACTAGATGCCTGAACCTAC
TP10282_Hit	D	1	chr2	.	CAGCACCAGAACCAATATACTTCAAACTCGAAAGATCATACTTATCAACCAAATCATGTTTCGC
TP10282_Query	D	1	chr2	.	CAGCACCAGAACCAATAGACTTCAAACTCGAAAGATCATACTTATCAACCAAATCATGTTTCGC
TP10289_Hit	D	1	chr2	.	CAGCACCAGACAAGCAAGAGAAACTGCTTGTTCTTCGCATTTCAGGGCGTATTTTGCTGAAAAA
TP10289_Query	D	1	chr2	.	CAGCACCAGACAAGCAAGAGAAACTGCTTGTTCTTCGCATTTCAGGGCGTATTTTGCAGAAAAA
TP10367_Hit	D+G	1	chr2	.	CAGCACCAGTTATAGTTCCAGTGCCTGTGGTATTGCCAGTTCCAGTAGTTTCACCGGTGCCAAT
TP10367_Query	D+G	1	chr2	.	CAGCACCAGTTATAGTTCCAGTGCCTGTGGTATCGCCAGTTCCAGTAGTTTCACCGGTGCCAAT
TP10549_Hit	D	1	chr2	.	CAGCACCCCAAACTTATGCCCACTGATTGGTTAAATTTTCACATTGATGTTCTCTCGCATTTGG
TP10549_Query	D	1	chr2	.	CAGCACCCCAAACTTATGCCCACTGATTGGTTAAACTTTCACATTGATGTTCTCTCGCATTTGG
TP10661_Hit	D+G	1	chr2	.	CAGCACCGAAATGACAAACCCTAGCATTGAATTCCGGTTCGGTAACAACAATCGCACTACTCTT
TP10661_Query	D+G	1	chr2	.	CAGCACCGAAATGACAAACCCTAGCATTGAAATCCGGTTCGGTAACAACAATCGCACTACTCTT
TP10838_Hit	D	1	chr2	.	CAGCACCTAAAGCCCCGGCAAAACCCGCCCCCGCCACACCAGCTCCTGCAACAGCACCAACCAA
TP10838_Query	D	1	chr2	.	CAGCACCTAAAGCCCCGGCAAAACCCGCCCCCGCCACACAAGCTCCTGCAACAGCACCAACCAA
TP11150_Hit	D	1	chr2	.	CAGCACGACACATATCTATTAAAAATGACATTTGTTGGCAGAGTATTGAGATGTAGAATGCACC
TP11150_Query	D	1	chr2	.	CAGCACGACACATATCTATTAAAAATGACATTTGTTGGCAGAGGATTGAGATGTAGAATGCACC
TP11445_Hit	D	1	chr2	.	CAGCACGGACTAAGGAGACACTCAAGATAATGCAGGAGCAAGTGCAGGAACTACTGGACGCCGA
TP11445_Query	D	1	chr2	.	CAGCACGGACTAAGGAGACACTCAAGATAATGCAGGAACAAGTGCAGGAACTACTGGACGCCGA
TP11836_Hit	D	1	chr2	.	CAGCGCTAGAAACCGTGTCTTTTGTTGCTTCATAACCTTGTTGTGTTTTCTCAAGAGCTGAAAA
TP11836_Query	D	1	chr2	.	CAGCACTAGAAACCGTGTCTTTTGTTGCTTCATAACCTTGTTGTGTTTTCTCAAGAGCTGAAAA
TP11923_Hit	D	1	chr2	.	CAGCACTATCAGAAGAGGAACGCGGTCATATCATGCCTGCTTTGTCAGAAAGATTAAAGCTTCC
TP11923_Query	D	1	chr2	.	CAGCACTATCAGAAGAGGAACGCGGTCATATCATGCCTGCTTTGTCAGAAAGATTAAAACTTCC
TP11940_Hit	D+G	1	chr2	.	CAGCACTATGAAAACCCGAGATTGTGTTTGTTTTAACTTTAGCATCTCTTCACTAACTAGCTCT
TP11940_Query	D+G	1	chr2	.	CAGCACTATGAAAACCCGAGATTGTGTTTGTTTTAACTTTAGCATCTCTTCACTAACCAGCTCT
TP1196_Hit	D+G	1	chr2	.	CAGCAAACACGTTACCGGAAATCTCCTTACGCTCCTTCCGGTGAAGGTATGTTTTAATTTGATG
TP1196_Query	D+G	1	chr2	.	CAGCAAACACGTTACCGGAAATCCCCTTACGCTCCTTCCGGTGAAGGTATGTTTTAATTTGATG
TP12034_Hit	D+G	1	chr2	.	CAGCACTCATGCTTTTATCAACTGTCTTGTTCTTTCTTGCTTCTCCTTTTTATGTGAAAATTAC
TP12034_Query	D+G	1	chr2	.	CAGCACTCATGCTTTTATCAACTGTCTTGTTCTTTCTTGCTTCTCCTCTTTATGTGAAAATTAC
TP12170_Hit	D+G	1	chr2	.	CAGCACTGAATGATAGGCTCCTAGTTTCAAAGGATCTGTGGCCATTTGGGGAACAGTTGAGAAG
TP12170_Query	D+G	1	chr2	.	CAGCACTGAATGATAGGCTCCTAGTTTCAAAGGATCTGTGGCCATTTGGGGAACAATTGAGAAG
TP12226_Hit	D	1	chr2	.	CAGCACTGCAACAAAGAAGTGAAGAGAAATTAGCATTGGAAACGTCATTACCGGAACATAATGG
TP12226_Query	D	1	chr2	.	CAGCACTGCAACAAAGAAGTGAAGAGAAATTAGCATTGGAAACGTCATTACCGGAACATAATAG
TP12292_Hit	D+G	1	chr2	.	CAGCACTGGATCCAATTACACTCCCTGGACCCCTTCTTCCTTTTCTTTTCTCAGCAACCCCTTG
TP12292_Query	D+G	1	chr2	.	CAGCACTGGATCCAATAACACTCCCTGGACCCCTTCTTCCTTTTCTTTTCTCAGCAACCCCTTG
TP12326_Hit	D	1	chr2	.	CAGCACTGGTTTTGTTTACAAGGGACAAATCAAGTACTCTTTCAATTTGATCAGGCTTCTCATC
TP12326_Query	D	1	chr2	.	CAGCACTGGTTTTGTTTACAAGCGACAAATCAAGTACTCTTTCAATTTGATCAGGCTTCTCATC
TP12395_Hit	D	1	chr2	.	CAGCACTGTTTCTTGCCTTCTTTCAACCTCCAACCTTGCCTGCAACCTATCCTCTTCCTTTGCT
TP12395_Query	D	1	chr2	.	CAGCACTGTTTCTTGCCTTCTTTCAACCTCCAACCTTGCCTGCAACCTATCCTCTTCCTTTGCA
TP12421_Hit	D+G	1	chr2	.	CAGCACTTAAGCCACCTATTGGATTTGAGTTGATTGAAGATGCTTTACCTGAAGGGTTTAGTGA
TP12421_Query	D+G	1	chr2	.	CAGCACTTAAGCCACCTATTGGATTTGAGTCGATTGAAGATGCTTTACCTGAAGGGTTTAGTGA
TP12468_Hit	D	1	chr2	.	CAGCACTTATGTGGGGAATGAACGAAGGAGCTGTTTTTCTTTGGACTCTACCTATGTTTCATTG
TP12468_Query	D	1	chr2	.	CAGCACTTATGTGGGGAATGAACGAAGGAGCTGTTTTTCTTTGGACTCTACCCATGTTTCATTG
TP12657_Hit	D	1	chr2	.	CAGCACTTGTTGAGTCACAAGGACAACAATTGAATAACATAGAGAGTCATGTTGCACATGCTAG
TP12657_Query	D	1	chr2	.	CAGCACTTGTTGAGTCACAAGGACAACAATTGAACAACATAGAGAGTCATGTTGCACATGCTAG
TP12677_Hit	D	1	chr2	.	CAGCACTTTATCTGAGGAATAGTTGGGGAAATATTCAAACTATTCTTGAAATTTCATCCTCTAT
TP12677_Query	D	1	chr2	.	CAGCACTTTATCTGAGGAATAGTTGGGGAAATGTTCAAACTATTCTTGAAATTTCATCCTCTAT

Name	Filter	Nb hit (Mt4.0)	Mt Chr (Mt4.0)	Ms Chr (Li et al., 2014)	Sequence
TP12695_Hit	D	1	chr2	.	CAGCACTTTTTCAACTGCTTCCTCAGAATCATATGTGATGAATCCAAAACCTCTAGGACGTTGA
TP12695_Query	D	1	chr2	.	CAGCACTTTCTCAACTGCTTCCTCAGAATCATATGTGATGAATCCAAAACCTCTAGGACGTTGA
TP12840_Hit	D+G	1	chr2	.	CAGCAGAAAGAGAACAAGCGCGACGACAGAGATGATGCCGCTCGAGAGATATAGACCCCAAAAG
TP12840_Query	D+G	1	chr2	.	CAGCAGAAACAGAACAAGCGCGACGACAGAGATGATGCCGCTCGAGAGATATAGACCCCAAAAG
TP12873_Hit	D	1	chr2	.	CAGCAGAAACTTCGATAAAAACGCATAAAAAGTACACGGGTGCTCTGTTATATATACATGCACA
TP12873_Query	D	1	chr2	.	CAGCAGAAACTTCGATAAAAACACATAAAAAGTACACGGGTGCTCTGTTATATATACATGCACA
TP12902_Hit	D	1	chr2	.	CAGCAGAAAGTAGCATCATATTACATCCTTAAATTATACATATGCTTTAACTGGCTTTCATTGG
TP12902_Query	D	1	chr2	.	CAGCAGAAAGTAGCATCATATTACATCCTTAAATTATACATATGCTTTAACTGGCTTTCATAGG
TP13086_Hit	D	1	chr2	.	CAGCAGAAGAAGCAGATGAAGCAGAAGCACCTAACCTTGAACGTTGTGGTGAAACTGAGTGAGA
TP13086_Query	D	1	chr2	.	CAGCAGAAGAAGCAGATGAAGCAGAAGCACCTAACCTTGAACGTTGTGGTGAAACTGAATGAGA
TP13125_Hit	D	1	chr2	.	CAGCAGAAGATGGAAGAATCCCAGGAATCGAACCAGCAACTCCATTTGTGTTATTACTAAATCC
TP13125_Query	D	1	chr2	.	CAGCAGAAGATGGAAGAATCCCAGGAATAGAACCAGCAACTCCATTTGTGTTATTACTAAATCC
TP1314_Hit	D	1	chr2	.	CAGCAAACCAGTAAAAGCTATAACAAGTGCTTCTGTCATTACCAACAATAGTCAACAGTTACAA
TP1314_Query	D	1	chr2	.	CAGCAAACCAGTAAAAGCTATAACAACTGCTTCTGTCATTACCAACAATAGTCAACAGTTACAA
TP13145_Hit	D	1	chr2	.	CAGCAGAAGCAATAGAACCAATGGCAGACTGCAAGAATGTTCAGGAGGAAAGTATAGATTTAAA
TP13145_Query	D	1	chr2	.	CAGCAGAAGCAATAGAACCAATGGCAGACTGCAAGAATGTTCAGGAGGAAAGTACAGATTTAAA
TP13149_Hit	D+G	1	chr2	.	CAGCTGAAGCAATGAAATTCTGGTGAGTTAGAATCACTCCTTTGCTTACACCGGTGGTACCGGA
TP13149_Query	D+G	1	chr2	.	CAGCAGAAGCAATGAAATTCTGGTGAGTTAGAATCACTCCTTTGCTTACACCGGTGGTACCGGA
TP13231_Hit	D	1	chr2	.	CAGCAGAAGTAGAAGTAGAAGTAGGAGTAGGAGCTGGAGCAGGGGTCGCAGTAGAAGTCGCTGA
TP13231_Query	D	1	chr2	.	CAGCAGAAGTAGAAGTAGAAGTAGGAGTAGGAGCTGGAGCAGGGGTCGCAGTAGAAGTCGCAGA
TP13286_Hit	D	1	chr2	.	CAGCAGAATATCGAATTAAGCAGAGAATCGTATCCTTGCTGGATTTCAAGAACCTACTTAATAA
TP13286_Query	D	1	chr2	.	CAGCAGAATATCGAATTAAGCAGAGAACCGTATCCTTGCTGGATTTCAAGAACCTACTTAATAA
TP13319_Hit	D	1	chr2	.	CAGCAGAATCGACAAGTTTTCGACGTTTGTGATTTTTGAGCCAATGAATGCTCACTCTAACAAC
TP13319_Query	D	1	chr2	.	CAGCAGAATCGACAAGTTTTCGACGTTTGTGATTTTTAAGCCAATGAATGCTCACTCTAACAAC
TP13342_Hit	D	1	chr2	.	CAGCAGAATGCGGCATTTATTTCTGGTGCGAGGGAGAGGGTTATTAGGATGGTTGAGATGCCTG
TP13342_Query	D	1	chr2	.	CAGCAGAATGCGGCATTTAATTCTGGTGCGAGGGAGAGGGTTATTAGGATGGTTGAGATGCCTG
TP13380_Hit	D	1	chr2	.	CAGCAGAATTGGCAACTCCCGAAGCTATGGCTTTTATAGTAAAGCATGGAACTGGCATAGTTTG
TP13380_Query	D	1	chr2	.	CAGCAGAATTGGCAACGCCCGAAGCTATGGCTTTTATAGTAAAGCATGGAACTGGCATAGTTTG
TP13586_Hit	D	1	chr2	.	CAGCAGACTGAACATCAGTCGGAACGTTAAAACCCTCTTTTCCTAACCTCTCAATCAACACTTC
TP13586_Query	D	1	chr2	.	CAGCAGACTGAACATCAGTCGGAACATTAAAACCCTCTTTTCCTAACCTCTCAATCAACACTTC
TP13893_Hit	D	1	chr2	.	CAGCGGAGGTTGAAGAGCAAGATGACATGTATTTCTTGGTGGCTCCTCAAAATGCTGTAGGGAA
TP13893_Query	D	1	chr2	.	CAGCAGAGGTTGAAGAGCAAGATGACATGTATTTCTTGGTGGCTCCTCAAAATGCTGTAGGGAA
TP13919_Hit	D	1	chr2	.	CAGCAGAGTATTGCATCAGTGTGATGCCACAAGGACTTGAGTAATGATTCGCTCCCCTCGTTAT
TP13919_Query	D	1	chr2	.	CAGCAGAGTATTGCATCACTGTGATGCCACAAGGACTTGAGTAATGATTCGCTCCCCTCGTTAT
TP14005_Hit	D+G	1	chr2	.	CAGCAGATAAGCTTGAATCAGATTGTAAATAAAGTCAAATTGTTTTTTCGTATTATGGTAATAA
TP14005_Query	D+G	1	chr2	.	CAGCAGATAAGCTTGAATCAGATCGTAAATAAAGTCAAATTGTTTTTTCGTATTATGGTAATAA
TP14023_Hit	D+G	1	chr2	.	CAGCAGATACACCATCACCTTTTCCATCTTGCATACATGAGTTTCCCCTCCAAGAAACATTGTT
TP14023_Query	D+G	1	chr2	.	CAGCAGATACACCATCACCCTTTCCATCTTGCATACATGAGTTTCCCCTCCAAGAAACATTGTT
TP14069_Hit	D	1	chr2	.	CAGCAGATATGAATACATTTGATCCTGGCTTTGGATCTGCTAGTACCTCTATTCCCACCCGTGC
TP14069_Query	D	1	chr2	.	CAGCAGATATGAATACATTTGATCCTGGCTTTGGATCTGCTAGTACCTCTATTCCCACCCATGC
TP14095_Hit	D	1	chr2	.	CAGCATATCACTATGGGACACAAGATGAAGTTGCGACTCACTAAGAAAAGGTGGGAATAAACCA
TP14095_Query	D	1	chr2	.	CAGCAGATCACTATGGGACACAAGATGAAGTTGCGACTCACTAAGAAAAGGTGGGAATAAACCA
TP14104_Hit	D	1	chr2	.	CAGCAGATCAGTAATTAGTGCTTGAGAATGGTATGTTAAAAGTAGGTGTTTAACTGGATGACAT
TP14104_Query	D	1	chr2	.	CAGCAGATCAGTAATTAGTGCTCGAGAATGGTATGTTAAAAGTAGGTGTTTAACTGGATGACAT
TP14141_Hit	D+G	1	chr2	.	CAGCAGATCGAAGAGACTCAAATTATTGTGACAACTCCTGAGAAGTGGGATATTATCACTCGAA
TP14141_Query	D+G	1	chr2	.	CAGCAGATCGAAGAGACTCAAATTATTGTGACAACTCCTGAGAAGTGGGATATTATCACCCGAA
TP14150_Hit	D+G	1	chr2	.	CAGCAGATCTACCACCTGATAAAAACAACAGTCATCCACCGTAAGAGATCCCAAATAATAACAC
TP14150_Query	D+G	1	chr2	.	CAGCAGATCTACCACCTGATAAAAACAACAGTCATCCACCGTAAGAGATCCCAAATAATAACAA
TP14265_Hit	D+G	1	chr2	.	CAGCAGATGTTGATATAATTGCAAACGAACTAGAGGTAATTTCTTCAACAGATTGTACCGAAAT
TP14265_Query	D+G	1	chr2	.	CAGCAGATGTTGATATAATTGCAAACGAACTAGAGGTAACTTCTTCAACAGATTGTACCGAAAT
TP14371_Hit	D	1	chr2	.	CAGCAGATTTTGGTGAATTCCTCATTTAATCTGATCTTTCAGGATTATTCATTAGTTATGAGGG
TP14371_Query	D	1	chr2	.	CAGCAGATTTTGGTGAATTCCTCATTTAATCTGAGCTTTCAGGATTATTCATTAGTTATGAGGG
TP14523_Hit	D	1	chr2	.	CAGCAGGGCATATGAAAAGATACCTTTGGTTCATCATGTGACCTAGTTGAGTAATTGTAAAATG
TP14523_Query	D	1	chr2	.	CAGCAGGACATATGAAAAGATACCTTTGGTTCATCATGTGACCTAGTTGAGTAATTGTAAAATG

Name	Filter	Nb hit (Mt4.0)	Mt Chr (Mt4.0)	Ms Chr (Li et al., 2014)	Sequence
TP14537_Hit	D+G	1	chr2	.	CAGCAGGATGAAGATATATATGACTTTTTTGAGCCAGAGCCTTCTTCTTTTGGTGCAAGTCTTC
TP14537_Query	D+G	1	chr2	.	CAGCAGGACGAAGATATATATGACTTTTTTGAGCCAGAGCCTTCTTCTTTTGGTGCAAGTCTTC
TP14553_Hit	D+G	1	chr2	.	CAGCAGGACTCAATTACCAATGATTTAATTGCAAACTTCATATGCATATGAAATAAGAGTAAAC
TP14553_Query	D+G	1	chr2	.	CAGCAGGACTCAATTACCAATAATTTAATTGCAAACTTCATATGCATATGAAATAAGAGTAAAC
TP14560_Hit	D	1	chr2	.	CAGCAGGACTTTACAGTCCTGAGAACTCAAAACTAAGATTTGGAGATGGGCAACTGCAACATAT
TP14560_Query	D	1	chr2	.	CAGCAGGACTTTACAGTCCTGAGAACTCAAAACTAAGATTTGGAGATGGGCAACTGCAACATAG
TP14675_Hit	D	1	chr2	.	CAGCAGGAGTACCCATTATAATTGCTTTAAACAAGGTAGGCCAACATATGTCTCCAACCCCAAT
TP14675_Query	D	1	chr2	.	CAGCAGGAGTACCCATTATAATTGCTATAAACAAGGTAGGCCAACATATGTCTCCAACCCCAAT
TP1499_Hit	D	1	chr2	.	CAGCAAACTGTCGGATCTAGGTTTGTGTTATTAAGTGAGGAATATTCGAATTTGAAATTTGTGT
TP1499_Query	D	1	chr2	.	CAGCAAACTGTCGGATCTAGGTTTGTGTTATTAAGTGAGGAAAATTCGAATTTGAAATTTGTGT
TP15088_Hit	D	1	chr2	.	CAGCAGGGGGATTGGTACAGGAAGCTACAACTTGAGTTTAAGGGTTTTGACACAACTCACAAGA
TP15088_Query	D	1	chr2	.	CAGCAGGGGGATTGGTACAGGAAGCTACAACTTGAGTTTAAGGGTTCTGACACAACTCACAAGA
TP15252_Hit	D+G	1	chr2	.	CAGCAGGTCCATTCTCTGGTGGGTCAATCAACCCTGCATGTGCTTTTGGATCTGCTTCCATTGC
TP15252_Query	D+G	1	chr2	.	CAGCAGGTCCATTCTCTGGTGGGTCAATCAACCCTGCATGTGCTTTCGGATCTGCTTCCATTGC
TP15263_Hit	D	1	chr2	.	CAGCGGGTCCTGATGTGTTTTTGCATCAAGAAACCTATCATTTTCAATGTCCTTAACATAGTTC
TP15263_Query	D	1	chr2	.	CAGCAGGTCCTGATGTGTTTTTGCATCAAGAAACCTATCATTTTCAATGTCCTTAACATAGTTC
TP15264_Hit	D+G	1	chr2	.	CAGCGGGTCCTGATGTGTTTTTTGCATCAAGAAACCTATCATTTTCAATGTCCTTAACATAGTT
TP15264_Query	D+G	1	chr2	.	CAGCAGGTCCTGATGTGTTTTTTGCATCAAGAAACCTATCATTTTCAATGTCCTTAACATAGTT
TP15277_Hit	D	1	chr2	.	CAGCAGGTCTTACACAATACAGAGCCTATTCAACTGATGAAGTAATATTCTGAACCATTCTAGT
TP15277_Query	D	1	chr2	.	CAGCAGGTCTTACACAATACAGAGCCTATTCAACTGATGAAGTAATATTCAGAACCATTCTAGT
TP15340_Hit	D	1	chr2	.	CAGCAGGTGGCCTAGGCAAGGAGCCAAGAAGTATGTCTGTTGCATTTAAAGACTGGCTGAAAAA
TP15340_Query	D	1	chr2	.	CAGCAGGTGGCCTAGGCAAGGAGCCAAGAAGTATATCTGTTGCATTTAAAGACTGGCTGAAAAA
TP15356_Hit	D	1	chr2	.	CAGCAGGTGTAATTCGGAGAAGCGTTGATTGTCCGAGTTTTCCAGTGAAGTCCTCTTCGGACGA
TP15356_Query	D	1	chr2	.	CAGCAGGTGTAATTCGGAGAAGCGTTGATTGTCCAAGTTTTCCAGTGAAGTCCTCTTCGGACGA
TP15416_Hit	D	1	chr2	.	CAGCAGGTTCTCTAACCGGAGCCTGATGATTTATTCACAATTAAACTAAGAATTCGAGTTTTTG
TP15416_Query	D	1	chr2	.	CAGCAGGTTCTCTAACCGGAGCCTGATGATTTATTCACAATTAAACTAAGAATTCGAGTTTTTA
TP15431_Hit	D	1	chr2	.	CAGCAGGTTGCTCCTCAGTTGCAGAGGCTTCTTTCTGGGGTTCCTCTGCAGGGCCGTTAGAGGC
TP15431_Query	D	1	chr2	.	CAGCAGGTTGCTCCTCAGTTGCAGAGGCTTCTTTCTGGGGTTCCTCTGCAGGGCCATTAGAGGC
TP15437_Hit	D	1	chr2	.	CAGCAGGTTGGCTCAAAGGCTAATATTTGGAAAAACAAACTATGGGGACCTAAGAAAATTTCTA
TP15437_Query	D	1	chr2	.	CAGCAGGTTGGCGCAAAGGCTAATATTTGGAAAAACAAACTATGGGGACCTAAGAAAATTTCTA
TP15556_Hit	D	1	chr2	.	CAGCAGTAACTGCTGAGGAAGAGAATGTGAAACCAGTTGAGTCAGTTGAAGCTCCAGCTGAAAA
TP15556_Query	D	1	chr2	.	CAGCAGTAACTGCTGAGGAAGAGAATGTGAAACCAGTTGAGTCAGTTGAAGCTCCAGCAGAAAA
TP15646_Hit	D	1	chr2	.	CAGCAGTACCGGCTTTAACACAAATCGTATTGATGCTTATGCTCCCAGAATCACCTCGTTGGTT
TP15646_Query	D	1	chr2	.	CAGCAGTACCGGCTTTAACACAAATCATATTGATGCTTATGCTCCCAGAATCACCTCGTTGGTT
TP15736_Hit	D	1	chr2	.	CAGCAGTAGGGTCATGAGATGCAATGACATTCGCATCGGGTGATAAAGTGGTGAACATAGGTAG
TP15736_Query	D	1	chr2	.	CAGCAGTAGCGTCATGAGATGCAATGACATTCGCATCGGGTGATAAAGTGGTGAACATAGGTAG
TP15781_Hit	D	1	chr2	.	CAGCAGTAGTGTTTAGTTGTGTGCAACATTCCTTGATATATGTTTAGTTGTGTGCAACATTCCT
TP15781_Query	D	1	chr2	.	CAGCAGTAGTGTTTAGTTGTGTGCAACATTCCTGGATATATGTTTAGTTGTGTGCAACATTCCT
TP15936_Hit	D	1	chr2	.	CAGCAGTCATGGATCGCCTGCTCCTACTGCTATACGCACAAAAAAGATATTTGTTGGAGGTCTA
TP15936_Query	D	1	chr2	.	CAGCAGTCATGGATCGCCTGCTCCTACTCCTATACGCACAAAAAAGATATTTGTTGGAGGTCTA
TP16171_Hit	D	1	chr2	.	CAGCAGTGCAAAGGAAGCAATGGAACTATAAATAGCACTGTAGGTCCTCTAACTGAGCGGATTC
TP16171_Query	D	1	chr2	.	CAGCAGTGCAAAGGAAGCAATGGAACTATAAATAGCACTATAGGTCCTCTAACTGAGCGGATTC
TP16196_Hit	D+G	1	chr2	.	CAGCAGTGCCGACTCCATCTAGAACGCCATTAATCAATAGATTTAGACCTTCAATGCAAGCTTC
TP16196_Query	D+G	1	chr2	.	CAGCAGTGCCGACTCCATCTAGAACGCCATTAATCAATAGATTCAGACCTTCAATGCAAGCTTC
TP16543_Hit	D	1	chr2	.	CAGCAGTTCAGGTATGTGAATTTCTATTTTTTCTCCTATTTTCCACACAAAAGAAACTATTTCC
TP16543_Query	D	1	chr2	.	CAGCAGTTCAGGTATGTGAATTTCCATTTTTTCTCCTATTTTCCACACAAAAGAAACTATTTCC
TP16550_Hit	D+G	1	chr2	.	CAGCAGTTCATGTAGGAAGCAAGTTCGGTCAAAATGAGATGACATATATGAGGACAAGAATCAT
TP16550_Query	D+G	1	chr2	.	CAGCAGTTCATGTAGGAAGCAAGTTCGGTCAAAATGAGATGACATATATGAGGACAAAAATCAT
TP16558_Hit	D	1	chr2	.	CAGCAGTTCCAAGGCCAACAGGACGAACAAGCGGGGTGCTAGTAGCAACAGGTTTTCCAGATGA
TP16558_Query	D	1	chr2	.	CAGCAGTTCCAAGGCCAACAGGACGAACAAGCGGGGTGCTAGTAGCAACAGATTTTCCAGATGA
TP16598_Hit	D+G	1	chr2	.	CAGCAGTTCTAAGCCATCACCACCAGAATCTCAGACTCAACAGCACAACAACAACAATGGTGCT
TP16598_Query	D+G	1	chr2	.	CAGCAGTTCTAAGCCATCACAACCAGAATCTCAGACTCAACAGCACAACAACAACAATGGTGCT
TP16737_Hit	D	1	chr2	.	CAGCAGTTGTACCTCCACATTTCGATTGTCGGCAGATGTCTTTGTGTCATCAAAGTGGATTTTT
TP16737_Query	D	1	chr2	.	CAGCAGTTGTACCTCCACATTTCGATTGTCGGCAGATGTCTTTGTGTCATCAAAGTGGATTCTT

Name	Filter	Nb hit (Mt4.0)	Mt Chr (Mt4.0)	Ms Chr (Li et al., 2014)	Sequence
TP16738_Hit	D	1	chr2	.	CTGCAGTTGTACTACCATTCATCTTCTCTTTGTTTTGCTCTATGTTTTGTCCTCTGCTCTTGTT
TP16738_Query	D	1	chr2	.	CAGCAGTTGTACTACCATTCATCTTCTCTTTGTTTTGCTCTATGTTTTGTCCTCTGCTCTTGTT
TP16979_Hit	D	1	chr2	.	CAGCATAAACAGAACACCACCAAGTAGATATGGTTACATTGCAAATTTATGTGTTGCCAAATTG
TP16979_Query	D	1	chr2	.	CAGCATAAACAGAACACCACCAAGTAGATATGGTTACATTGCAAATTTATGTGTTACCAAATTG
TP17098_Hit	D	1	chr2	.	CAGCATAACCTCCAGTAGCAAACGCCCGCAAGAGGTTGAGAGCAGAAAAAAAAAAAAAAAAAAA
TP17098_Query	D	1	chr2	.	CAGCATAACCTCCAGTAGCAAACGCCCGCAAAAGGTTGAGAGCAGAAAAAAAAAAAAAAAAAAA
TP17099_Hit	D+G	1	chr2	.	CAGCATAACCTCCAGTAGCAAACGCCCGCAAGAGGTTGAGAGTAGCCACAGACTGGCAGTAGGC
TP17099_Query	D+G	1	chr2	.	CAGCATAACCTCCAGTAGCAAACGCCCGCAAAAGGTTGAGAGTAGCCACAGACTGGCAGTAGGC
TP17408_Hit	D	1	chr2	.	CAGCATACCAATCCCTTACGAAATTCTTCCACAATCTCATTTTGAATTTTTCTAGTTTGGTTTT
TP17408_Query	D	1	chr2	.	CAGCATACCAATCCCTTACGAAATTCTTCCACGATCTCATTTTGAATTTTTCTAGTTTGGTTTT
TP17414_Hit	D	1	chr2	.	CAGCATACCAGGAAGAATTAGCGAGAGCGAAAATTGAAAAAGAGGAAAATCTTAAAGAACTCAG
TP17414_Query	D	1	chr2	.	CAGCATACCAGGAAGAATTAGCGAGAGCGAAAATTGAAAAAGAGGAAAATCTTAAAGAACTCAA
TP17433_Hit	D	1	chr2	.	CAGCATACCCTTTTATATAATTGATATTTCAACTGCTACGTGACTTCTGTTGTTTGCCCACAAT
TP17433_Query	D	1	chr2	.	CAGCATACCCTTTTATATAATTGATATTTCAACTGCTACGTGACTTCCGTTGTTTGCCCACAAT
TP1749_Hit	D	1	chr2	.	CAGCAAAGCCATTTTGGGTTCGGTTTAACGGTAGAGGTAGAGTAAGAGGATGGCGAAGACTCAC
TP1749_Query	D	1	chr2	.	CAGCAAAGCCATTTTGGGTTCGGTTTAACGGTAGAGGTAAAGTAAGAGGATGGCGAAGACTCAC
TP17616_Hit	D	1	chr2	.	CAGCGTAGCACACAGGACTGAGTAAATTCACCCTGAAAATTTTGTAAATCTTCCTTTATTAAAC
TP17616_Query	D	1	chr2	.	CAGCATAGCACACAGGACTGAGTAAATTCACCCTGAAAATTTTGTAAATCTTCCTTTATTAAAC
TP17783_Hit	D	1	chr2	.	CAGCATATACACCTCTGCCATTTATGAAAGTAGATTGATCAATGTTTTAAATTAGATTTTAGAG
TP17783_Query	D	1	chr2	.	CAGCATATACACCTCTGCCATTTATGAAAGTAGATTGAACAATGTTTTAAATTAGATTTTAGAG
TP17832_Hit	D+G	1	chr2	.	CAGCATATATAGTTGAAATAATCCTTGAGTCCTTCATATTCAAATTGATGTTATTTGTGGAAGC
TP17832_Query	D+G	1	chr2	.	CAGCATATATAGTTGAAATAATCCTCGAGTCCTTCATATTCAAATTGATGTTATTTGTGGAAGC
TP17983_Hit	D+G	1	chr2	.	CAGCATATGCATTTGCTCCAGCTATATTGGTCACTCCGCTTGGTGCTCTTAGCATTATTATCAG
TP17983_Query	D+G	1	chr2	.	CAGCATATGCATTTGCTCCAGCCATATTGGTCACTCCGCTTGGTGCTCTTAGCATTATTATCAG
TP18250_Hit	D	1	chr2	.	CAGCATCAAATTTCTCTAACTTCACGTCAAAAGCTGTTTTCTCTGCCTTTTTCTCCTCAACCTT
TP18250_Query	D	1	chr2	.	CAGCATCAAATTTCTCTAACTTCACGTCAAAAGCTGTTTTCTCCGCCTTTTTCTCCTCAACCTT
TP18319_Hit	D	1	chr2	.	CAGCATCAACGTTCTAGTGGCCGTATTTCTGAAATTCCCACTCACTGTTGTCTACACCAAGCTG
TP18319_Query	D	1	chr2	.	CAGCATCAACGTTCTAGTGGCCGTATTTCTGAAATTCCCACTCACTGTTGTCTACACCAAGCAG
TP18415_Hit	D	1	chr2	.	CAGCATCGATGGAAGTTTTATTTGCCTCGCTAAAGAAAGAAAGCCAAATTCCATCACCAAGAGC
TP18415_Query	D	1	chr2	.	CAGCATCAATGGAAGTTTTATTTGCCTCGCTAAAGAAAGAAAGCCAAATTCCATCACCAAGAGC
TP18458_Hit	D	1	chr2	.	CAGCATCACAGATCTCAATTACCCTTGACATTTTAAACATCAGAACCTTGTATAGTTATATTTA
TP18458_Query	D	1	chr2	.	CAGCATCACAGATCTCAATTACCCTTGAAATTTTAAACATCAGAACCTTGTATAGTTATATTTA
TP18551_Hit	D	1	chr2	.	CAGCATCACTTCAATCTTTTCCTCCTCAACAAGATCACCCTCAGGTATAATTTCATTTTCAGTT
TP18551_Query	D	1	chr2	.	CAGCATCACTTCAATCTTTTACTCCTCAACAAGATCACCCTCAGGTATAATTTCATTTTCAGTT
TP18628_Hit	D+G	1	chr2	.	CAGCATCAGCGCGTTCATCTTCTCCACCTGTTGCCCCCCTCTGGCTGAAAAAAAAAAAAAAAAA
TP18628_Query	D+G	1	chr2	.	CAGCATCAGCGCGTTCATCTTCTCCACCTGTTGCCCCCCTCTGGCAGAAAAAAAAAAAAAAAAA
TP18722_Hit	D	1	chr2	.	CAGCATCATCAAAATGTTGGAGATATGTATTGGATTGGGAAAAGATGAGACGAGCAATGCGAGG
TP18722_Query	D	1	chr2	.	CAGCATCATCAAAATGTTGGAGATATGTATTGGATTGAGAAAAGATGAGACGAGCAATGCGAGG
TP18771_Hit	D	1	chr2	.	CAGCATCATGATGATATCTTAAGCGAGCAGGATAATCTTTCTGAAGCAGTTGAAGAAGAGCAAC
TP18771_Query	D	1	chr2	.	CAGCATCATGATGATATCTTAAGCGAGCAGGATAATCTTTCCGAAGCAGTTGAAGAAGAGCAAC
TP1878_Hit	D	1	chr2	.	CAGCAAAGGGTGCAACAGGATAATCTTTACTCTCAGCAAAAGTATCTACACAAGAGGAATGGTA
TP1878_Query	D	1	chr2	.	CAGCAAAGGGTGCAACAGGAAAATCTTTACTCTCAGCAAAAGTATCTACACAAGAGGAATGGTA
TP18789_Hit	D	1	chr2	.	CAGCATCATGTTAATATTTATGTCTCCAAACCAGACAGTGGATGCACCGATTTTGAAACATGCA
TP18789_Query	D	1	chr2	.	CAGCATCATGTTAATATTTATGTCTCCAAACCAGACAGTGGATGCACCAATTTTGAAACATGCA
TP18844_Hit	D	1	chr2	.	CAGCATCATTTGTAGTTGTTTCTGCTGATGATATAGATATTGTATAGTGAAAGAATCTTTGGTA
TP18844_Query	D	1	chr2	.	CAGCATCATTTGTAGTTGTTTCTGCTGATGATATAGATATTGTATAGTGAAAGAATCTTTCGTA
TP18947_Hit	D+G	1	chr2	.	CAGCATCCATTTTCATTACCTTCCTTATAATCTGTACAAGAAAACACAAGAACACAGCCAAAAG
TP18947_Query	D+G	1	chr2	.	CAGCATCCATTTTCATTACCTTCCTTATAATCTGTACAAAAAAACACAAGAACACAGCCAAAAG
TP19194_Hit	D	1	chr2	.	CAGCATCGGCTCTACCTGATGGCCCTAATGGTACTCCTTCCAGTTATGACATGTTTGTTACACA
TP19194_Query	D	1	chr2	.	CAGCATCGGCTCCACCTGATGGCCCTAATGGTACTCCTTCCAGTTATGACATGTTTGTTACACA
TP19227_Hit	D+G	1	chr2	.	CAGCATCGTGAGTGGGAAACATTTGACCGATGTATGGAATGTGATGATGAGTTGTTGTTGTTGT
TP19227_Query	D+G	1	chr2	.	CAGCATCGTGAGTGGGAAACATTTGACCGATGTATGGAATGTGATGATGAGTTGTTGTTGTTGG
TP19560_Hit	D	1	chr2	.	CAGCATCTTGAAATTTTCGGTGTGGACCAACAGGTACCGTGAATTGGCGCATCTAGTGGACGAG
TP19560_Query	D	1	chr2	.	CAGCATCTTGAAATTTTCGGTGTGGACCAACAGGTACCGTGAATTGGCGCATCGAGTGGACGAG

Name	Filter	Nb hit (Mt4.0)	Mt Chr (Mt4.0)	Ms Chr (Li et al., 2014)	Sequence
TP19615_Hit	D	1	chr2	.	CAGCATCTTTGGGTTGGCAGAGGACAATGAGTCTGTGTGTATGTCCTTGCTGAGCATGAAATGG
TP19615_Query	D	1	chr2	.	CAGCATCTTTGGGTTGGCAGAGGACAATGAGTCTGTGTGTATGTCCTTGCCGAGCATGAAATGG
TP19749_Hit	D	1	chr2	.	CAGCATGAATCAACAATAGGTCAAGATATAACTTTTGTGCAAAAGGACTTGAAAGGAACGCCAC
TP19749_Query	D	1	chr2	.	CAGCATGAATCAACAATAGGTCAAGATATAACTTTTGTGCAAAAGGACTTGAAAGGAACACCAC
TP19781_Hit	D	1	chr2	.	CAGCATGACGCCTTATGGTTTCCATGGTGCTGGAGGTGGTGGTGGTGTTGGAAACATTGGTGGA
TP19781_Query	D	1	chr2	.	CAGCATGACACCTTATGGTTTCCATGGTGCTGGAGGTGGTGGTGGTGTTGGAAACATTGGTGGA
TP19898_Hit	D	1	chr2	.	CAGCATGATAAACAACACGACATGGTTGATCTAGAGTTATGTTCTTCCTTGTTTCACTGTCGAA
TP19898_Query	D	1	chr2	.	CAGCATGATAAACAACACGACATGGTTGATCTGGAGTTATGTTCTTCCTTGTTTCACTGTCGAA
TP20044_Hit	D	1	chr2	.	CAGCATGCAAGCTATTTCAGCGGAATCCTCACAAACAACCCCGAGTGTTTGCACCGTAGGTAGA
TP20044_Query	D	1	chr2	.	CAGCATGCAAGCTATTTCAGCGGAATCCTCACAAACAACCCCGAGTGTTTGCACCGTAGGAAGA
TP20092_Hit	D+G	1	chr2	.	CAGCATGCAGTATCACTCCTACATACATATTTTAAACAAAAACTTAAAAATCCACTAATAAGAG
TP20092_Query	D+G	1	chr2	.	CAGCATGCAGTATCACTCCTACATACATATTTTAAACAAAAACTTAAAAATCAACTAATAAGAG
TP20574_Hit	D+G	1	chr2	.	CAGCATGTGCCAACATATTCAATTGCGGAAGAGATGACAGGATTTGGAGCATCAATATTAGAGG
TP20574_Query	D+G	1	chr2	.	CAGCATGTACCAACATATTCAATTGCGGAAGAGATGACAGGATTTGGAGCATCAATATTAGAGG
TP20589_Hit	D+G	1	chr2	.	CAGCATGTAGTGTAGGTTCAGGCATCTAGTTAGACAGATTCAGCCCCATAATGTCCATCATTGC
TP20589_Query	D+G	1	chr2	.	CAGCATGTAGTGTAGGTTCAGGCATCTAGCTAGACAGATTCAGCCCCATAATGTCCATCATTGC
TP20764_Hit	D+G	1	chr2	.	CAGCATGTGTGATATGAATGATTCAATAGAATAACTTTGTAACATGACCATGAATTTGAAAATT
TP20764_Query	D+G	1	chr2	.	CAGCATGTGTGATATGAATGATTCAATAGAAGAACTTTGTAACATGACCATGAATTTGAAAATT
TP20838_Hit	D+G	1	chr2	.	CAGCATGTTGGCGAGAGACAGATTGATGATCAAGCACAAAATCACAAGTCTGGATTTGTCTCCC
TP20838_Query	D+G	1	chr2	.	CAGCATGTTGGCGAGAGACAGATTGATGATCAAGCACAAAATCACAAGTCTGGATTTGCCTCCC
TP20953_Hit	D	1	chr2	.	CAGCATTAAGGCATTAACATATCAAAACTGAAAAACGAAATTTTATTTATACAGAATAACATCT
TP20953_Query	D	1	chr2	.	CAGCATTAAGGCATTAACATATCAAAACTGAAAAAAGAAATTTTATTTATACAGAATAACATCT
TP210_Hit	D+G	1	chr2	.	CAGCAAAAAGGATTGCAAGGAAGGTTAGTGCTCGTGGTGGTGGGCTTCCTACTGTGCAAACGCT
TP210_Query	D+G	1	chr2	.	CAGCAAAAAGGATTGCAAGGAAGGTTAGTGCTCGTGGTGGTGGGCTTCCTACGGTGCAAACGCT
TP21118_Hit	D+G	1	chr2	.	CAGCATTAGGACCAGCACCAGAACCAGACAGTGACCTTTCACAAACCTTCCAAATTGAAATTAT
TP21118_Query	D+G	1	chr2	.	CAGCATTAGGACCAGCACCAGAACCAGACAGTGACCTTTCACAAACCTTCCAAAGTGAAATTAT
TP21173_Hit	D	1	chr2	.	CAGCATTATATCGTCTGTCCAAAGGGTAGCATTGGCACTTTCACCTTCTAATTTGAATTCACAA
TP21173_Query	D	1	chr2	.	CAGCATTATATCGTCTGTCCAAAGGGTAGCATTGGCACTTTCACCTTCGAATTTGAATTCACAA
TP2132_Hit	D+G	1	chr2	.	CAGCAAATATTCCTCACATTATAAAATAAAATCATGTGAGACCAAATATAATAATCATATCAAA
TP2132_Query	D+G	1	chr2	.	CAGCAAATATTCCTCACATTACAAAATAAAATCATGTGAGACCAAATATAATAATCATATCAAA
TP21327_Hit	D	1	chr2	.	CAGCATTCAGGTAAAAGTAATAACCTAATACTATTATTTATGTCGCTAAATATAAGTCCGCGGA
TP21327_Query	D	1	chr2	.	CAGCATTCAGGTAAAAGTAATAACCTAATACTATTATTTATGTCCCTAAATATAAGTCCGCGGA
TP21337_Hit	D	1	chr2	.	CAGCATTCATAAGGATACCACGTGGGGAAGTGATGGTCCATTCTGTGTTGCACGTGAGTTACAG
TP21337_Query	D	1	chr2	.	CAGCATTCATAAGGATACCACGTGGGGAAGTGGTGGTCCATTCTGTGTTGCACGTGAGTTACAG
TP21547_Hit	D	1	chr2	.	CAGCATTCTTAGGAGAATCTAACTACATAAGGTCAGTCCTTGTATAATATCCCAAACAAAAGAT
TP21547_Query	D	1	chr2	.	CAGCATTCTTAGGAGAATCTAACTACATAAAGTCAGTCCTTGTATAATATCCCAAACAAAAGAT
TP21603_Hit	D+G	1	chr2	.	CAGCATTGAAGATAAATAGGAAATAATCCCAAATCACCTTAAAAATTCATGGGCATCTTCCTGA
TP21603_Query	D+G	1	chr2	.	CAGCATTGAAGATAAATAGGAAATAATCCCAAATCACCTCAAAAATTCATGGGCATCTTCCTGA
TP21667_Hit	D+G	1	chr2	.	CAGCATTGATGCAAATAATTATGGAAATAATTCTATACTGGGACAGGACAGTTTGTTCTCTCAG
TP21667_Query	D+G	1	chr2	.	CAGCATTGATGCAAATAATCATGGAAATAATTCTATACTGGGACAGGACAGTTTGTTCTCTCAG
TP21678_Hit	D	1	chr2	.	CAGCATTGATTCCAATAGTTACAGAAACTAAGATAATGTATTTTATTATATAATATTTCAGGCG
TP21678_Query	D	1	chr2	.	CAGCATTGATTCCAATAGCTACAGAAACTAAGATAATGTATTTTATTATATAATATTTCAGGCG
TP21841_Hit	D	1	chr2	.	CAGCATTGGTGCTGAGTATATGAGGATTGCATGTCCTGAATTCGTTAAAGCTTGCGATGAGATG
TP21841_Query	D	1	chr2	.	CAGCATTGGTGCTGAGTATATGAGAATTGCATGTCCTGAATTCGTTAAAGCTTGCGATGAGATG
TP22025_Hit	D	1	chr2	.	CAGCATTTAGTATCACTCCCGAAAAACCTCCAACCTCATTGGGATGCACACATGGCCATAGGTG
TP22025_Query	D	1	chr2	.	CAGCATTTAGTATCACTCCCGAAAAACCTCCAACCACATTGGGATGCACACATGGCCATAGGTG
TP22088_Hit	D	1	chr2	.	CAGCATTTCACACATCGCCTCACTTCATTCTTCCCCCACTCTCCCTTTCTCTCTCTCAAATGGT
TP22088_Query	D	1	chr2	.	CAGCATTTCACACATCACCTCACTTCATTCTTCCCCCACTCTCCCTTTCTCTCTCTCAAATGGT
TP22196_Hit	D	1	chr2	.	CAGCTTTTGAAATTGGGGAACAAGTTCAAGCTCAGCTTATGAAAACGCCTTTTTTTGCAGACAT
TP22196_Query	D	1	chr2	.	CAGCATTTGAAATTGGGGAACAAGTTCAAGCTCAGCTTATGAAAACGCCTTTTTTTGCAGACAT
TP22323_Hit	D	1	chr2	.	CAGCATTTGGCATCTTTGATTATAATTTGTTCAAATGTTCTTCAAAATTTCAGGTAGCACACAA
TP22323_Query	D	1	chr2	.	CAGCATTTGGCATCTTTGATTATAATTAGTTCAAATGTTCTTCAAAATTTCAGGTAGCACACAA
TP22381_Hit	D	1	chr2	.	CAGCATTTGTTGAAAATGGTAAGAAATTGTTGTTTATGTTCAGTAGGATCCCCAAATTGTTTTA
TP22381_Query	D	1	chr2	.	CAGCATTTGTTGAAAATGGTAAGAAATTGTTGTTTATGTTCAGTAGGATCCCCAAATGGTTTTA

Name	Filter	Nb hit (Mt4.0)	Mt Chr (Mt4.0)	Ms Chr (Li et al., 2014)	Sequence
TP22859_Hit	D	1	chr2	.	CAGCCAAACACACTTCTTTAAAGGAAACTTGTGTCACAGCTATAAGGGAGTAAAATTAACAGCC
TP22859_Query	D	1	chr2	.	CAGCCAAACACACTTCTTTAAAGGAAACTTGTGCCACAGCTATAAGGGAGTAAAATTAACAGCC
TP22980_Hit	D	1	chr2	.	CAGCCAAAGATAGTCCAAGGTGACGGGGCAATAACATCAGAAGAAGAAAGTGTCCAGTCTATAG
TP22980_Query	D	1	chr2	.	CAGCCAAAGATAGTCCAAGGTGACGGGGCAATAACATCAGAAGAAGAAAGTGTCCAGTCCATAG
TP2304_Hit	D+G	1	chr2	.	CAGCAAATGATCAGGATAAAAAGTCTCCTTCTGAAGAGGACATTTAGAGTCCTATTCGCGATTA
TP2304_Query	D+G	1	chr2	.	CAGCAAATGATCAGGATAAAAAGTCTCCTTCTGAAGAGGACATTTAGAATCCTATTCGCGATTA
TP23065_Hit	D	1	chr2	.	CAGCCAAATAAAGATTCTATCTTTTCTTCACTGAACCTGGTTAAGTTGTGATAAAATTCAGCAA
TP23065_Query	D	1	chr2	.	CAGCCAAATAAAGACTCTATCTTTTCTTCACTGAACCTGGTTAAGTTGTGATAAAATTCAGCAA
TP23153_Hit	D+G	1	chr2	.	CAGCCAAATGCGGCCTATACGGTTGCAATGTTGTTAAGGAGACATCAAAATTTTTTATATTGGT
TP23153_Query	D+G	1	chr2	.	CAGCCAAATGCGGCCTATACGGTTGCAATGTTGTTAAGGAGACATCAAAATTCTTTATATTGGT
TP2318_Hit	D	1	chr2	.	CAGCAAATGATTTTGATGAAGAACGTAGTAGCACCAAAAAAGCAAAGATGCTTCATGTTTAAAC
TP2318_Query	D	1	chr2	.	CAGCAAATGATTTTGATGAAGAACGTAGGAGCACCAAAAAAGCAAAGATGCTTCATGTTTAAAC
TP23243_Hit	D	1	chr2	.	CAGCCAACAAGAAAAGGAGTCTGAGCTCAATCCATGTGGCGGAGCCGATACGGCTGAAGAATGG
TP23243_Query	D	1	chr2	.	CAGCCAACAAGAAAAGGAGTCTAAGCTCAATCCATGTGGCGGAGCCGATACGGCTGAAGAATGG
TP23246_Hit	D+G	1	chr2	.	CAGCCAGCAAGAGTCTGCTCAAGATTCCTAGATCGCTCTCGGTTAGTGTCTTCTTGATCTTCCA
TP23246_Query	D+G	1	chr2	.	CAGCCAACAAGAGTCTGCTCAAGATTCCTAGATCGCTCTCGGTTAGTGTCTTCTTGATCTTCCA
TP23276_Hit	D	1	chr2	.	CAGCCAACACAAGTTGTTCTTGAAGCTTCGAAAAGGCAAAAGGAGTGAGGACCGTAGCTGAAAA
TP23276_Query	D	1	chr2	.	CAGCCAACACAAGTTGTTCTTGAAGCTTCGAAAAGGCAAAAGGAGTGAGGACCGTAGCAGAAAA
TP23304_Hit	D	1	chr2	.	CAGCCAACACTTCATCAATTTGATATCTCTTCCGGTGGAATTGGGACTAATAATGCTTTCAAGG
TP23304_Query	D	1	chr2	.	CAGCCAACACTTCATCAATTTGATATCTCTTCCGGAGGAATTGGGACTAATAATGCTTTCAAGG
TP23606_Hit	D	1	chr2	.	CAGCCAAGAGGTTGAACAAATGGGAGGATAAGAAAAAGGAGCCCCTTCTAAACTAAAAAATTGG
TP23606_Query	D	1	chr2	.	CAGCCAAGAGGTTGAACAAATGGGAGGATAAGAAAAAGGAGCCCCTTCTAAACTAAAAAAATGG
TP23698_Hit	D+G	1	chr2	.	CAGCCAAGGATTGTTGAGAAACTGCGCATTGTCGTATAACTTTTTAGATTTCATTGTCTTGGTG
TP23698_Query	D+G	1	chr2	.	CAGCCAAGGATTGTTGAGAAACTGCGCATTGTCGTATAACTTTTTAGATTTCATTGTCTCGGTG
TP23871_Hit	D	1	chr2	.	CAGCCAATACGGGTATTGTGGTAATGGTGATGCATATTGTGGCACAGGATGCAAACAAGGTCCT
TP23871_Query	D	1	chr2	.	CAGCCAATACGGGTATTGCGGTAATGGTGATGCATATTGTGGCACAGGATGCAAACAAGGTCCT
TP23985_Hit	D+G	1	chr2	.	CAGCCAATGACTGAGCCTGCAGTAATGATCCAGTGACGAATCCACCCCCAATCTCTGTACCACC
TP23985_Query	D+G	1	chr2	.	CAGCCAATGACTGAGCCTGCAGTAATGATCCAGTGACAAATCCACCCCCAATCTCTGTACCACC
TP24132_Hit	D	1	chr2	.	CAGCCAATTTAGCATTCTTTTGCTGAGTCTTCAGAAAAGGATTATTCCTAGGCCGTTTGTCTTG
TP24132_Query	D	1	chr2	.	CAGCCAATTTAGCATTCTTTTGCTGAGTCTTCAGAAAAGGATTATTCCTAGACCGTTTGTCTTG
TP24404_Hit	D	1	chr2	.	CAGCCACACCACACGATAACTCCCTTGGATTATATCCTACCATGTAAAATCAAGTCTTTTGAAT
TP24404_Query	D	1	chr2	.	CAGCCACACCACACGATAACTCCCTTGGATTATATCATACCATGTAAAATCAAGTCTTTTGAAT
TP24453_Hit	D	1	chr2	.	CAGCCAGAGAATGGTGCAATCACTATGCCTGTAGCAAATATGAAAGGTGACGAAATCACTGGAG
TP24453_Query	D	1	chr2	.	CAGCCACAGAATGGTGCAATCACTATGCCTGTAGCAAATATGAAAGGTGACGAAATCACTGGAG
TP2453_Hit	D	1	chr2	.	CAGCAAATTAGAACTAAATGCAGATAACCTGATTAGATATTATCAAACATGTTAAGGCACACTT
TP2453_Query	D	1	chr2	.	CAGCAAATTAGAACAAAATGCAGATAACCTGATTAGATATTATCAAACATGTTAAGGCACACTT
TP24565_Hit	D+G	1	chr2	.	CAGCCACATATTTTGGCAATATGTAATTGCATAAAACAAAAAAGATAGATGTAGGGATAAATTC
TP24565_Query	D+G	1	chr2	.	CAGCCACATATTTTGGCAATACGTAATTGCATAAAACAAAAAAGATAGATGTAGGGATAAATTC
TP24812_Hit	D	1	chr2	.	CAGCCACCCAAGAAGCACCAAGTGCAACAACCTTTACTTCTTCGCTTTCAAAGCGCATAGACCG
TP24812_Query	D	1	chr2	.	CAGCCACCCAAGAAGCACCAAGTGCAACAACCTTTACTTCTTCGCTTTCAAAGCGCATAGACCA
TP25011_Hit	D	1	chr2	.	CAGCCACGAATTCGGATTTTGTGGTTAACACAATTTTCTATTTGCTCCAAAATTTACCTATGAA
TP25011_Query	D	1	chr2	.	CAGCCACGAATTCGGATTTTGCGGTTAACACAATTTTCTATTTGCTCCAAAATTTACCTATGAA
TP25026_Hit	D+G	1	chr2	.	CAGCCACGATTTTAACGAGATCCTCACAGTACTGACTTGGTTTATAGTCTTCCATGAAGGAAAT
TP25026_Query	D+G	1	chr2	.	CAGCCACGACTTTAACGAGATCCTCACAGTACTGACTTGGTTTATAGTCTTCCATGAAGGAAAT
TP2515_Hit	D	1	chr2	.	CAGCAAATTGAAAACCAAACTGAGGACAATCTCAAATCTGTCAAGGACAATCTCAAAGTACTCC
TP2515_Query	D	1	chr2	.	CAGCAAATTGAAAACCAAACTGAGGACAATCGCAAATCTGTCAAGGACAATCTCAAAGTACTCC
TP25206_Hit	D	1	chr2	.	CAGCCACTAGCTCTGCATGTCCATCTTCTGGACCTACTAAATCAACGAAACCAAAAGTGAATAA
TP25206_Query	D	1	chr2	.	CAGCCACTAGCTCTGCATGTCCATCTTCTGGACCTACTAAAACAACGAAACCAAAAGTGAATAA
TP25212_Hit	D	1	chr2	.	CAGCCACTATAAATGATTTTCTGAACAACATGGAAAGGGAAAAGCCAATATGATTCACTGGTCA
TP25212_Query	D	1	chr2	.	CAGCCACTATAAATGATTTTCTGAACAACATGGAAAGGGAAAAGCCAATAAGATTCACTGGTCA
TP25254_Hit	D	1	chr2	.	CAGCCACTCCATTCGGTCTCAAATGAACTCTAAATCTCTCAGAGTTGCTAAGGCCAGGTTCAAG
TP25254_Query	D	1	chr2	.	CAGCCACTCCATTCGGTCTCAAATGAACTCTAAATCTCTCAGAGTTGCCAAGGCCAGGTTCAAG
TP2530_Hit	D	1	chr2	.	CAGCAAATTGCTATTTTGATTGGTTGTGCTGTGTTGCCCCATCTGAGATCTCTTGTGGAAATTA
TP2530_Query	D	1	chr2	.	CAGCAAATTGCCATTTTGATTGGTTGTGCTGTGTTGCCCCATCTGAGATCTCTTGTGGAAATTA

Name	Filter	Nb hit (Mt4.0)	Mt Chr (Mt4.0)	Ms Chr (Li et al., 2014)	Sequence
TP25405_Hit	D+G	1	chr2	.	CTGCCACTTCATTCACCTCTTGGAAATCACATTTTCCTTCAAGCTTTGAATCAACAATCTCCTC
TP25405_Query	D+G	1	chr2	.	CAGCCACTTCATTCACCTCTTGGAAATCACATTTTCCTTCAAGCTTTGAATCAACAATCTCCTC
TP25416_Hit	D	1	chr2	.	CAGCCACTTCTTGATGGCATAGTTAGTTATTTGCCGTGTCCGATTGAAGTTAGTAATTATTCTC
TP25416_Query	D	1	chr2	.	CAGCCACTTCTTGATGGCATAGTTAGTTATTTGCCGTGTCCGATTGAAGTTAGTAATTATGCTC
TP25646_Hit	D	1	chr2	.	CAGCTAGAGTGTAGGTCCCAATAAGGAATCATTTTCAAGATTAGGTTTTAGCTACAAGACACCT
TP25646_Query	D	1	chr2	.	CAGCCAGAGTGTAGGTCCCAATAAGGAATCATTTTCAAGATTAGGTTTTAGCTACAAGACACCT
TP25672_Hit	D	1	chr2	.	CAGCCAGATCTGCTGGTGGGGAAAAGACTCAAAAAGAGTCCTAGCTGATCCATCTTTGAGAGTT
TP25672_Query	D	1	chr2	.	CAGCCAGATCTGCTGGTGGGGAAAAGACTCAAAAAGAGTCCTAGCCGATCCATCTTTGAGAGTT
TP25978_Hit	D	1	chr2	.	CAGCCAGGGCAGGTGCCTCTGGTGTGTCTTTAATTTCAGGAGGATAGATCGTGTATAAAATCAA
TP25978_Query	D	1	chr2	.	CAGCCAGGGCAGGTGCCTCTGGCGTGTCTTTAATTTCAGGAGGATAGATCGTGTATAAAATCAA
TP25984_Hit	D+G	1	chr2	.	CAGCCAGGGCTGAAAATGTACCTCCTGCAGGGCAGTTGGTTGCCAATGCTCCTCCAGCAGGTAT
TP25984_Query	D+G	1	chr2	.	CAGCCAGGGCTGAAAATGTACCTCCTGCAGGGCAGGTGGTTGCCAATGCTCCTCCAGCAGGTAT
TP26102_Hit	D	1	chr2	.	CAGCCAGTCTTTAAATGCAACAGATATACTTCTTGGCTCCTTGCCTAGGCCACCTGCTGAAAAA
TP26102_Query	D	1	chr2	.	CAGCCAGTCTTTAAATGCAACAGACATACTTCTTGGCTCCTTGCCTAGGCCACCTGCTGAAAAA
TP26110_Hit	D	1	chr2	.	CAGCCAGTGAATCATTATATATGTTCCAACCTTTTATACATTTTGATCTTCCCCATTACGATTC
TP26110_Query	D	1	chr2	.	CAGCCAGTGAATCATTATATATGTTCCAAACTTTTATACATTTTGATCTTCCCCATTACGATTC
TP26192_Hit	D	1	chr2	.	CAGCCAGTTTTCATGGTCACCTGAATCTGGTGATTCATTGACTGCTGAAAATCTTACAAGACTG
TP26192_Query	D	1	chr2	.	CAGCCAGTTTTCATGGTCACCTGAATCTGGTGATTCATTGACTGCTGAAAATCTTACAAGACTA
TP26277_Hit	D+G	1	chr2	.	CAGCCATAATAGAGGTTTTGCATAAAGCACTACGAGGGTTCTTCATTGCCTTCACCACCACCAA
TP26277_Query	D+G	1	chr2	.	CAGCCATAATAGAGGTTTTGCATAAAGCACTACGAGGATTCTTCATTGCCTTCACCACCACCAA
TP26436_Hit	D	1	chr2	.	CAGCCATATACGTGTACGGTTTGATCCCGCAGATCTTCGCTTACGCGGTGAACTTCCCCATACA
TP26436_Query	D	1	chr2	.	CAGCCATATACGTGTACGGTTTGATACCGCAGATCTTCGCTTACGCGGTGAACTTCCCCATACA
TP2662_Hit	D	1	chr2	.	CAGCAACAAAAATGGACACAAATATTGTTCCTTGAAGGCTAATCAAATTGTTTGCACCAGAAGT
TP2662_Query	D	1	chr2	.	CAGCAACAAAAATGGAAACAAATATTGTTCCTTGAAGGCTAATCAAATTGTTTGCACCAGAAGT
TP26640_Hit	D	1	chr2	.	CAGCCATCCAGCCCTCCACAACTCCAAGTTTGTGACCAATCTGGCATTGTTGGCAGTGCTGTGG
TP26640_Query	D	1	chr2	.	CAGCCATCCACCCCTCCACAACTCCAAGTTTGTGACCAATCTGGCATTGTTGGCAGTGCTGTGG
TP26802_Hit	D	1	chr2	.	CAGCCATGAACTTCCATTGCCTGCAGGAAGTATTGTAATTGTAAATTAAAGATTTAGATGATAT
TP26802_Query	D	1	chr2	.	CAGCCATGAACTTCCATTACCTGCAGGAAGTATTGTAATTGTAAATTAAAGATTTAGATGATAT
TP26995_Hit	D	1	chr2	.	CAGCCATGGAGAAGGAGAATCAAGGTATTTCCCCAAATGAAAGATCTTGAAATGTTATTTGGAC
TP26995_Query	D	1	chr2	.	CAGCCATGGAGAAGAAGAATCAAGGTATTTCCCCAAATGAAAGATCTTGAAATGTTATTTGGAC
TP27191_Hit	D	1	chr2	.	CAGCCATGTTTAATTGGCAAATCTTATTTTGAAAAAAAATATATAATGGAAACAGAAAAACATC
TP27191_Query	D	1	chr2	.	CAGCCATGTTTAATTGGCAAATCTTATTTTGAAAAAAAAAATATAATGGAAACAGAAAAACATC
TP27201_Hit	D+G	1	chr2	.	CAGCCATTAAAATTAAACAAATTAAGATGCAAATATCACATTCATCGCGATTTGTACATTTGTA
TP27201_Query	D+G	1	chr2	.	CAGCCATTAAAATTAAACAAATTAAGATGCAAATATCACATTCATCGCGATTTGTACATTTGGA
TP27768_Hit	D	1	chr2	.	CAGCCCATCTGATTCCAATTGGTGGGATACCATCTGTTTCAACAGTCTTGCCTGTGATAGGATC
TP27768_Query	D	1	chr2	.	CAGCCCAACTGATTCCAATTGGTGGGATACCATCTGTTTCAACAGTCTTGCCTGTGATAGGATC
TP27806_Hit	D	1	chr2	.	CAGCCCAAGGAAGTCCTGATTCCATGGCCGGACCAAAGGCTTTAAACATAGATTCTGCTGAAAA
TP27806_Query	D	1	chr2	.	CAGCCCAAGGAAGTCCTGATTCCATGGCCGGACCAAAGGCTTTAAACATAGATTCTGCAGAAAA
TP28045_Hit	D	1	chr2	.	CAGCCCAGAGCTACAACAACACTTCAAAGAACTACAAGTTAGCTCTCTACTTCAAGAACTCCAC
TP28045_Query	D	1	chr2	.	CAGCCCAGAACTACAACAACACTTCAAAGAACTACAAGTTAGCTCTCTACTTCAAGAACTCCAC
TP28158_Hit	D+G	1	chr2	.	CAGCCCATACAACCATCTTCTCTCTTGGTAGCAGAATCATGCAAATTACTTCCAACAGGATGTA
TP28158_Query	D+G	1	chr2	.	CAGCCCATACAACCATCTTCTCCCTTGGTAGCAGAATCATGCAAATTACTTCCAACAGGATGTA
TP28340_Hit	D	1	chr2	.	CAGCCCATTTGAACCTTCAATCACATCATCTTCTACCCTTTCACTTTTATAGTTTGGATTTCTC
TP28340_Query	D	1	chr2	.	CAGCCCATTTGAACCTTCAATCACATCATCTTCTACCCTTTCACTTTTATAGTTAGGATTTCTC
TP28341_Hit	D	1	chr2	.	CAGCCCATTTGAGAGCTTCTTCATCTTCATCAAGATTAGAAGTCCTTCTTGAATATCTTCCACT
TP28341_Query	D	1	chr2	.	CAGCCCATTTGAGAGCTTCTTCATCTTCATCAAGATTAGAAGTCCTTCTAGAATATCTTCCACT
TP28419_Hit	D	1	chr2	.	CAGCCCCAATATAAAGAATTTTGATGTCTCCTTAACAACATTGCAACCGTATAGGCCGCATTTG
TP28419_Query	D	1	chr2	.	CAGCCCCAATATAAAAAATTTTGATGTCTCCTTAACAACATTGCAACCGTATAGGCCGCATTTG
TP28490_Hit	D	1	chr2	.	CAGCTCCAGATGTACCAGCTTTTACTAATTCTTGAGGTCCCTCCAATAAGGATGCTATATTTCT
TP28490_Query	D	1	chr2	.	CAGCCCCAGATGTACCAGCTTTTACTAATTCTTGAGGTCCCTCCAATAAGGATGCTATATTTCT
TP29126_Hit	D	1	chr2	.	CAGCCCGGTGCACAAAGCTCCCGAATACGCAGGGTCCAGGAAAGGGTCCCACCATTTGTCAATC
TP29126_Query	D	1	chr2	.	CAGCCCGGTGCACAAAGCGCCCGAATACGCAGGGTCCAGGAAAGGGTCCCACCATTTGTCAATC
TP29445_Hit	D	1	chr2	.	CAGCCCTCAACCTCAGACAAGCCCTGGCATTCTGGTAAACATCAATCTTATATTCCTCCTTAAA
TP29445_Query	D	1	chr2	.	CAGCCCTCAACCTCAGACAAGCCCTGGCATTCTGGTAAACATCAACCTTATATTCCTCCTTAAA

Name	Filter	Nb hit (Mt4.0)	Mt Chr (Mt4.0)	Ms Chr (Li et al., 2014)	Sequence
TP29688_Hit	D+G	1	chr2	.	CAGCCCTTATCTTTGTTTTGCTATTGCATCATTTGAAAAGTTTTACCTGAACGCAGAGGGCAAT
TP29688_Query	D+G	1	chr2	.	CAGCCCTTATCTTTGTTTTGCTATTGCATCATTTGAAAAGTATTACCTGAACGCAGAGGGCAAT
TP29796_Hit	D	1	chr2	.	CAGCCCTTTACCTGCTGATGTAGTGTATGATTGTCTTAACATACTTGAGGAGGAATCTGTTGCT
TP29796_Query	D	1	chr2	.	CAGCCCTTTACCTGCTGATGTAGTGTATGACTGTCTTAACATACTTGAGGAGGAATCTGTTGCT
TP30011_Hit	D	1	chr2	.	CAGCTGACACATTACCAGCACAATCTGACAATGAAATTTTGCATGTTGAACCTAGAAGTAAACT
TP30011_Query	D	1	chr2	.	CAGCCGACACATTACCAGCACAATCTGACAATGAAATTTTGCATGTTGAACCTAGAAGTAAACT
TP30046_Hit	D	1	chr2	.	CAGCCGACCTAAATAGTTGAAGCTGTGGTGGATATTCATTAGGTACTATTGAAATCAAAGGTCT
TP30046_Query	D	1	chr2	.	CAGCCGACCTAAATAGTTGAAGCTGTGGTGGATATACATTAGGTACTATTGAAATCAAAGGTCT
TP30277_Hit	D	1	chr2	.	CAGCCGATTTCTCTGATCTGATTCCAAAAGTCAAAGCATCCCTGACAGAAGCACCGGTTGGTTT
TP30277_Query	D	1	chr2	.	CAGCCGATTTCTCTGATCTGATTCCAAAAGTCAAAGCATCCCTGACAGAAGCACCGGTTAGTTT
TP3047_Hit	D	1	chr2	.	CAGCAACAAGAGGATGTTAATGCTAGCTTAAGAAACATTAGGGTAAGTATCGAAAATTGGGTTA
TP3047_Query	D	1	chr2	.	CAGCAACAAGAGGATGTTAATGCTAGCTTAAGAAACATTAGGGTAAGTATCGAAAATTAGGTTA
TP30470_Hit	D	1	chr2	.	CAGCCGCATCATGTGAACACTGATCACCCTGTATGTGATGTCAAATTGAAGTTGCAAACATCCA
TP30470_Query	D	1	chr2	.	CAGCCGCATCATGTGAACACTGATCACCCTGTATGTGATGTCAAATTGAAGTTACAAACATCCA
TP30552_Hit	D	1	chr2	.	CTGCCGCCCCTCATTCCCATTCCTCCATAAGGGCTTGAAAAGCCAAAAACTTCTGCAAATATGC
TP30552_Query	D	1	chr2	.	CAGCCGCCCCTCATTCCCATTCCTCCATAAGGGCTTGAAAAGCCAAAAACTTCTGCAAATATGC
TP30564_Hit	D	1	chr2	.	CAGCCGCCGAGGAGAAAAAGAAAGGAAGTTGAAGATTATCTTGTAGTGACCTCAAGCTAGGAAA
TP30564_Query	D	1	chr2	.	CAGCCGCCGAGGAGAAAAAGAAAGGAAGCTGAAGATTATCTTGTAGTGACCTCAAGCTAGGAAA
TP30567_Hit	D+G	1	chr2	.	CAGCCGCCGATGGTTTCCCTTCCTTCTTCTCCGGGATCTACGGTCCTCGATGTCCACAACGGAA
TP30567_Query	D+G	1	chr2	.	CAGCCGCCGATGGTTTCCCTTACTTCTTCTCCGGGATCTACGGTCCTCGATGTCCACAACGGAA
TP30788_Hit	D	1	chr2	.	CAGCCGCTCCTATTGAGCGCGTGAAGCTTCTTATTCAAAATCAAGATGAGATGATTAAGGCTGG
TP30788_Query	D	1	chr2	.	CAGCCGCTCCTATTGAGCGCGTGAAGCTTCTGATTCAAAATCAAGATGAGATGATTAAGGCTGG
TP3089_Hit	D+G	1	chr2	.	CAGCAACAAGTCTTTGACCCCTCTCTCTACCCCTCTCCTTGTTTCATGTTTGATACTAAACTCC
TP3089_Query	D+G	1	chr2	.	CAGCAACAAGTCTTTGACCCCTCTCTCCACCCCTCTCCTTGTTTCATGTTTGATACTAAACTCC
TP31208_Hit	D+G	1	chr2	.	CAGCCGGTTTGTTTGGTGGGGAGTACGCTTCGTGCTCCTCATGGTTGTCATGCTCAGTATATGG
TP31208_Query	D+G	1	chr2	.	CAGCCGGTTTGTTTGGTGGGGAGTACGCTTCGTGCGCCTCATGGTTGTCATGCTCAGTATATGG
TP31254_Hit	D	1	chr2	.	CAGCCGTACGTTATGATGCCACCACAACAACCGCCAGCTATGTGGGCGGCCCAATCCGCTCAGC
TP31254_Query	D	1	chr2	.	CAGCCGTACGTCATGATGCCACCACAACAACCGCCAGCTATGTGGGCGGCCCAATCCGCTCAGC
TP31403_Hit	D	1	chr2	.	CAGCCGTGCAAGAGTGGTCTTTAAGAAGCGTGCAGATGCAGAGGTAGCGCTTGGTAGTGCTAAG
TP31403_Query	D	1	chr2	.	CAGCCGTGCAAGAGTGGTCTTTAAGAAGCGTGAAGATGCAGAGGTAGCGCTTGGTAGTGCTAAG
TP31464_Hit	D	1	chr2	.	CAGCTGTGTCTGCCTTTAGAGGAGCCCAAGAATTGAGACCTGATATACGCACATACCAAGGTTT
TP31464_Query	D	1	chr2	.	CAGCCGTGTCTGCCTTTAGAGGAGCCCAAGAATTGAGACCTGATATACGCACATACCAAGGTTT
TP32148_Hit	D	1	chr2	.	CAGCCTATGCTAAAAACAGCAATAACACCTATGGTGCTGAGACATTAATAATTGCCACAAAAAA
TP32148_Query	D	1	chr2	.	CAGCCTATGCTAAAAACAGCAAAAACACCTATGGTGCTGAGACATTAATAATTGCCACAAAAAA
TP32558_Hit	D+G	1	chr2	.	CAGCCTCATTTAAGATGGTGCCCGTCAGATTAGTCAATAGCGCGCTATAGAGGCGTAGCAACAA
TP32558_Query	D+G	1	chr2	.	CAGCCTCATTTAAGATGGTGCCCGTCAGATTAGTCAATAGCGCACTATAGAGGCGTAGCAACAA
TP32979_Hit	D	1	chr2	.	CAGCCTCTACTACTTTCTCCAACACATTATTTCTTTCTCCCATTTCTTCTCATTCTCCTTCCAA
TP32979_Query	D	1	chr2	.	CAGCCTCTACTACTTTCTCCAACACATTATTTCTTTCTCCAATTTCTTCTCATTCTCCTTCCAA
TP33102_Hit	D	1	chr2	.	CAGCCTCTGCCTCCTTTGTTGCCTCTTCAGCCTCCGCCATGAGGGCTTCCGCTTCAGCAACTGC
TP33102_Query	D	1	chr2	.	CAGCCTCTGCCTCCTTTGTTGCCTCCTCAGCCTCCGCCATGAGGGCTTCCGCTTCAGCAACTGC
TP33124_Hit	D	1	chr2	.	CAGCCTCTGGTTGACAAAGATGATAACGATGACAAAGAAAAAGGAATTTATGAAGATCATGCTG
TP33124_Query	D	1	chr2	.	CAGCCTCTGGTTGACAAAGATGATAAAGATGACAAAGAAAAAGGAATTTATGAAGATCATGCTG
TP33154_Hit	D	1	chr2	.	CAGCCTCTTCAACATTGCACTGCAATTGTGATGTGATCTTCAATCACGTGTGTGTACCACATTA
TP33154_Query	D	1	chr2	.	CAGCCTCTTCAACATTGCACTGCAATTGCGATGTGATCTTCAATCACGTGTGTGTACCACATTA
TP33203_Hit	D	1	chr2	.	CAGCCTCTTTCCTCTCATCACCGCTCTTAAACTCAGCCAGATAACGATAATAATCGCCTTTCCT
TP33203_Query	D	1	chr2	.	CAGCCTCTTTCCTCTCATCACCACTCTTAAACTCAGCCAGATAACGATAATAATCGCCTTTCCT
TP33251_Hit	D	1	chr2	.	CAGCCTGAAATTCCTTTTCGTTCGATTTTTTCCTCCATGAAGAGACTTATGTGTTACAGTATTG
TP33251_Query	D	1	chr2	.	CAGCCTGAAATTCCTTTTCGTTCGATCTTTTCCTCCATGAAGAGACTTATGTGTTACAGTATTG
TP33268_Hit	D	1	chr2	.	CAGCCTGAAGAAGGCTGAAATGCTAAGGATGAAACATTCATTTGACTGTTGAAGCTAGCCAACC
TP33268_Query	D	1	chr2	.	CAGCCTGAAGAAGGCTGAAAGGCTAAGGATGAAACATTCATTTGACTGTTGAAGCTAGCCAACC
TP33356_Hit	D	1	chr2	.	CAGCCTGAGCACAAACAAGCCTTGGAATCCTATCCACAAGACCTAACTCTCTACACATATGAAA
TP33356_Query	D	1	chr2	.	CAGCCTGAGCACAAACAAGCCTTGGAATCCTATCCACAAGACCTAACTCTCTACACAAATGAAA
TP33510_Hit	D	1	chr2	.	CAGCCTGCACTTCAGAGACAAGTTGTTGTAAGGAGATTTCAAATTGCATTTCAAATCGATTTGC
TP33510_Query	D	1	chr2	.	CAGCCTGCACCTCAGAGACAAGTTGTTGTAAGGAGATTTCAAATTGCATTTCAAATCGATTTGC

Name	Filter	Nb hit (Mt4.0)	Mt Chr (Mt4.0)	Ms Chr (Li et al., 2014)	Sequence
TP33636_Hit	D	1	chr2	.	CAGCCTGCTGTAGCAGGATCAATGGTTGGTGTAAGACTTGGCTTGTCAGAACATATTCAAGGCT
TP33636_Query	D	1	chr2	.	CAGCCTGCTGTAGCAGGATCAATGGTTGGTGTAAGACTTGGCTTGTCAGAACATATTCAAGACT
TP33790_Hit	D	1	chr2	.	CAGCCTGTAAACATCTTCCTTCAGTTTTTTATCTCCATTTGTTGCATGTTCATTTCACGCTTAG
TP33790_Query	D	1	chr2	.	CAGCCTGTAAACATCTTCCTTCAGTTTTTTATCTCCATTTGTTGCACGTTCATTTCACGCTTAG
TP33848_Hit	D+G	1	chr2	.	CAGCCTGTCTCAAGGTTTTTGTGACACCTATTTATTTCCTTGTCTTCTTCATTCATTAACTTAT
TP33848_Query	D+G	1	chr2	.	CAGCCTGTCTCAAGGTTTTTGTGACACCTATTTATTTCCATGTCTTCTTCATTCATTAACTTAT
TP34098_Hit	D	1	chr2	.	CAGCCTTACCCAAAATTTGAACAACCTTATCATTGTGTTGAGGCTCGCTACTATATTCAACATG
TP34098_Query	D	1	chr2	.	CAGCCTTACCCAAAATTTGAACAACCTTATCATTGTGTCGAGGCTCGCTACTATATTCAACATG
TP34135_Hit	D	1	chr2	.	CAGCCTTACGGCCTCTTGGTTTGCTTTCAAAGTTCTATTGAGTCTTAAAAAGGTAGCAATATGA
TP34135_Query	D	1	chr2	.	CAGCCTTACGGCCTCTTGGTTTGCTTTCAAAGTTCTACTGAGTCTTAAAAAGGTAGCAATATGA
TP34179_Hit	D	1	chr2	.	CAGCCTTAGTGCACAGAAAAATATGTAATCATTTAAACTTTATTTGAAGGACTACACATCACAT
TP34179_Query	D	1	chr2	.	CAGCCTTAGTGCACAGAAAAATATATAATCATTTAAACTTTATTTGAAGGACTACACATCACAT
TP34205_Hit	D	1	chr2	.	CAGCCTTATATGGAAAATCCTCTTTGGCCAAGAAATCTTCTAGCATTACAAGAGTCCTGATGTA
TP34205_Query	D	1	chr2	.	CAGCCTTATATGGAAAATCCTCTCTGGCCAAGAAATCTTCTAGCATTACAAGAGTCCTGATGTA
TP34368_Hit	D	1	chr2	.	CAGCCTTCCCGATCCAGTTGTCAGGCACATGACTGGAGACTTTCAGTTTGCGGCGAATAGATTC
TP34368_Query	D	1	chr2	.	CAGCCTTCCCGATCCAGTTGTCAGGCACATGACTGAAGACTTTCAGTTTGCGGCGAATAGATTC
TP34376_Hit	D	1	chr2	.	CAGCCTTCCGTGCCCCCGTTGGTGGTGTTCTGTTCGCACTCGAAGAAGCTGAAAAAAAAAAAAA
TP34376_Query	D	1	chr2	.	CAGCCTTCCGTGCCCCCGTTGGTGGTGTTCTGTTCGCACTCGAAGAAGCAGAAAAAAAAAAAAA
TP34390_Hit	D	1	chr2	.	CAGCCTTCCTTTCTTCACTTGGGATTGGAGGTACATCTCCATCTGACCATGTTGGCAATGTTCG
TP34390_Query	D	1	chr2	.	CAGCCTTCCTTTCTTCACTTGGGATTGGAGGCACATCTCCATCTGACCATGTTGGCAATGTTCG
TP34473_Hit	D	1	chr2	.	CAGCCTTTTGTTTTTCAACCTCTGCCTGCTCTTGAGCTTTTTTCATCTTTGACTCTGCATTCCT
TP34473_Query	D	1	chr2	.	CAGCCTTCTGTTTTTCAACCTCTGCCTGCTCTTGAGCTTTTTTCATCTTTGACTCTGCATTCCT
TP34534_Hit	D+G	1	chr2	.	CAGCCTTGAACCATAGACTAAACATTTAACACTGCCAAAAATATCTATAACTTTGTTCTTTGCT
TP34534_Query	D+G	1	chr2	.	CAGCCTTGAACCATAGACTAAACATTTAACACTGCCAAAAAAATCTATAACTTTGTTCTTTGCT
TP34541_Hit	D	1	chr2	.	CAGCCTTGAAGGATACTACTCATAAAATCAATCTCTTGTATAAAATGAAAAATAGGATTGTCTA
TP34541_Query	D	1	chr2	.	CAGCCTTGAAGGATACTACTCATAAAATCAATCTCTTGTATAAAATAAAAAATAGGATTGTCTA
TP34857_Hit	D	1	chr2	.	CAGCCTTTATTCTTTAAGTTAAAAATTAGAATTTCGTCTTTCTTTGTTCCTTTCCTTCCATGAA
TP34857_Query	D	1	chr2	.	CAGCCTTTATTCTTTAAGTTAAAAATTAGAAATTCGTCTTTCTTTGTTCCTTTCCTTCCATGAA
TP3526_Hit	D+G	1	chr2	.	CAGCAACAGGCGGAGATGGAATAATAGTAGTATTGAACGGATTCCCATTTTTACTGCAAGGGAC
TP3526_Query	D+G	1	chr2	.	CAGCAACAGGCGGAGATGGAATAATAGCAGTATTGAACGGATTCCCATTTTTACTGCAAGGGAC
TP35411_Hit	D	1	chr2	.	CAGCGAAGAAAGCCCAAGCCAAAGAATATGGATTTGAAGAAGACAAGTCAGATTCTGAAGATGA
TP35411_Query	D	1	chr2	.	CAGCGAAGAAAGCCCAAGCAAAAGAATATGGATTTGAAGAAGACAAGTCAGATTCTGAAGATGA
TP35481_Hit	D	1	chr2	.	CAGCGAAGGCTAAATTGTTGAAAGAATCCAAGTGCAGAGCAAAGAATCTTGTTCTTCCCATTTA
TP35481_Query	D	1	chr2	.	CAGCGAAGGCTAAATCGTTGAAAGAATCCAAGTGCAGAGCAAAGAATCTTGTTCTTCCCATTTA
TP35539_Hit	D	1	chr2	.	CAGCGAATAACTCCAAATTTACAAGAAGAGTGGCCTAAAATATTAAACACTTTTTTTATGATCT
TP35539_Query	D	1	chr2	.	CAGCGAATAACTCCAAATTTACAAAAAGAGTGGCCTAAAATATTAAACACTTTTTTTATGATCT
TP35783_Hit	D	1	chr2	.	CAGCGGCATTAGAAGGTCAAACAACAAACTGAATCATAATCTGACACTCAATCATTTGGAGAAG
TP35783_Query	D	1	chr2	.	CAGCGACATTAGAAGGTCAAACAACAAACTGAATCATAATCTGACACTCAATCATTTGGAGAAG
TP36404_Hit	D	1	chr2	.	CAGCGAGTGATGTTATAGTATGCGGGTGTTTGACAAACTATGTAACTATGTGTTTATTTAAAAA
TP36404_Query	D	1	chr2	.	CAGCGAGTGATGTTATAGTATGCGGGTGTTTGACAAACTATGTAACTATGTGCTTATTTAAAAA
TP3657_Hit	D	1	chr2	.	CAGCAATATATCAATAACAATTAGCTCCTAAGGAATTCCCATGGTCCTGATCCAGTTCCAATCC
TP3657_Query	D	1	chr2	.	CAGCAACATATCAATAACAATTAGCTCCTAAGGAATTCCCATGGTCCTGATCCAGTTCCAATCC
TP36707_Hit	D	1	chr2	.	CAGCGATGCTATCACCTCCGGTTCTAACTACGAGAAAGCCGCCGCTCTCATCGATCTGGTTCGT
TP36707_Query	D	1	chr2	.	CAGCGATGCTATCACCTACGGTTCTAACTACGAGAAAGCCGCCGCTCTCATCGATCTGGTTCGT
TP36832_Hit	D+G	1	chr2	.	CAGCGATTGAAAAAATTGAAACTAACCAAAGACGGCATAAAATACGGACAGGTTTCGGTGCTGA
TP36832_Query	D+G	1	chr2	.	CAGCGATTGAAAAAATTGAAACTAACCAAAAACGGCATAAAATACGGACAGGTTTCGGTGCTGA
TP37115_Hit	D	1	chr2	.	CTGCGCAGGAGATGCTTCTACAATCGATTGATCATTGCTAAGCATCTCCAGCACTGCCTTCATT
TP37115_Query	D	1	chr2	.	CAGCGCAGGAGATGCTTCTACAATCGATTGATCATTGCTAAGCATCTCCAGCACTGCCTTCATT
TP37293_Hit	D+G	1	chr2	.	CAGCGCATTTGCTAGCAGTCGCCGCTCTGCTTCAACTATGGACATTTTACCCCACGCCAGCTGA
TP37293_Query	D+G	1	chr2	.	CAGCGCATTTGCTAGCAGTCGCCGCTCTGCTTCAACTATGGACATTTTACCCCACACCAGCTGA
TP3737_Hit	D+G	1	chr2	.	CTGCAACATCGGTGAAGTTAGCTATTCCCCCTCCGATCACAAGGGCTCGCTTTTGTCCATCAGG
TP3737_Query	D+G	1	chr2	.	CAGCAACATCGGTGAAGTTAGCTATTCCCCCTCCGATCACAAGGGCTCGCTTTTGTCCATCAGG
TP3785_Hit	D+G	1	chr2	.	CAGCAACATGGACTGGAAAGACATGGTGTGACTGCTGACAGAATTGAAGATAGTGTCACTTCAT
TP3785_Query	D+G	1	chr2	.	CAGCAACATGGACTGGAAAGACATGGTGTGAATGCTGACAGAATTGAAGATAGTGTCACTTCAT

Name	Filter	Nb hit (Mt4.0)	Mt Chr (Mt4.0)	Ms Chr (Li et al., 2014)	Sequence
TP37905_Hit	D+G	1	chr2	.	CAGCGCGGCGGCTGGTGTGGCGTTTACGTTCTTTTCCGATGCCGGTGCTCTCAAACGGTGTCGC
TP37905_Query	D+G	1	chr2	.	CAGCGCGGCGGCTGGTGTGGCGTTTACGTTATTTTCCGATGCCGGTGCTCTCAAACGGTGTCGC
TP38091_Hit	D	1	chr2	.	CAGCGCTATATAGTATAGCTGACTTTGATGAATCCGCTATGAAACCAGCGCGGGAAATAGTGGC
TP38091_Query	D	1	chr2	.	CAGCGCTATATAGTATAGCTGACTTTGATGAATCCACTATGAAACCAGCGCGGGAAATAGTGGC
TP38106_Hit	D	1	chr2	.	CAGCGCTATTGCTTTGCAACGTAGCAAAATTTAAACAAACGTCTATTTTGTTTCAATGGACTAA
TP38106_Query	D	1	chr2	.	CAGCGCTATTGCTTTGCAACGTAGCAAAATTTGAACAAACGTCTATTTTGTTTCAATGGACTAA
TP38328_Hit	D	1	chr2	.	CAGCGCTTTGAATCAGGATATACAACATCTAAGCTCGTTTAAGGAAACAGAGTTCAAGAGAGAA
TP38328_Query	D	1	chr2	.	CAGCGCTTTGAATCAGGATAGACAACATCTAAGCTCGTTTAAGGAAACAGAGTTCAAGAGAGAA
TP38332_Hit	D	1	chr2	.	CAGCGCTTTGGAGTATGGGCTGACTGGAATAATCCCTATCTTACTCTAGATCCGGAATATGAAG
TP38332_Query	D	1	chr2	.	CAGCGCTTTGGAGTATGGGCTGACTGGAATAATCCCTATCTCACTCTAGATCCGGAATATGAAG
TP38420_Hit	D+G	1	chr2	.	CAGCGGAAGATGATGAAGGAAATGGCAGAATCGGTGAAAGATCTGCCAGGTGACTATGGAGAAA
TP38420_Query	D+G	1	chr2	.	CAGCGGAAGATGATGAAGGAAATGGCAGAATCGGTCAAAGATCTGCCAGGTGACTATGGAGAAA
TP3848_Hit	D+G	1	chr2	.	CAGCAACATTGATACTAATACTAATTGCAATTAGGCAAAATGCCATTGCATAGATGAGTGGAGT
TP3848_Query	D+G	1	chr2	.	CAGCAACATTGATACTAATACTAATTGCAATTAAGCAAAATGCCATTGCATAGATGAGTGGAGT
TP3856_Hit	D+G	1	chr2	.	CAGCAACATTGTTGTTGTTGTGCTGTTGAGTCTGAGATTCTGGTTGTGATGGCTTAGAACTGCT
TP3856_Query	D+G	1	chr2	.	CAGCAACATTGTTGTTGTTGTGCTGTTGAGTCTGAGATTCTGGTGGTGATGGCTTAGAACTGCT
TP38568_Hit	D+G	1	chr2	.	CAGCGGAGACGCTTTGCTTATTATAAAATTTAGGGTTTGGTTTTTCCATGAAGCATGAACAACA
TP38568_Query	D+G	1	chr2	.	CAGCGGAGACGCTTAGCTTATTATAAAATTTAGGGTTTGGTTTTTCCATGAAGCATGAACAACA
TP38665_Hit	D+G	1	chr2	.	CAGCTGATAACTTCTGTGTCTACAGAAATGAGATTCACCACTCTGTTGCTGAGCGGACTCAAGT
TP38665_Query	D+G	1	chr2	.	CAGCGGATAACTTCTGTGTCTACAGAAATGAGATTCACCACTCTGTTGCTGAGCGGACTCAAGT
TP38673_Hit	D	1	chr2	.	CAGCGGATAGATATTCCAATCCATTTCTCAGATACATAAGTGACATCCTCATGGATGAAGAAGA
TP38673_Query	D	1	chr2	.	CAGCGGATAGATATTCCAATCCATTCCTCAGATACATAAGTGACATCCTCATGGATGAAGAAGA
TP38715_Hit	D	1	chr2	.	CAGCGGATCTTCTGGGAAGGTTTACAAAGTGGTGTTGAGTAATGGAGAGGCAGTGGCTGAAAAA
TP38715_Query	D	1	chr2	.	CAGCGGATCTTCTGGGAAGGTTTACAAAGTGGTGTTGAGTAATGGAGAGGCAGTGGCAGAAAAA
TP38803_Hit	D	1	chr2	.	CAGCGGCGACTTGTAATGGCATCATCCAAATAGGATGAAACTGCTTCATCAAATCTGAGAGTTG
TP38803_Query	D	1	chr2	.	CAGCGGCAACTTGTAATGGCATCATCCAAATAGGATGAAACTGCTTCATCAAATCTGAGAGTTG
TP38855_Hit	D+G	1	chr2	.	CAGCGGCACTTCTGGTCAAAGCCGTGTCCATCTCTGTAGCAGTGACACTTCTGGTCAAAGCCGT
TP38855_Query	D+G	1	chr2	.	CAGCGGCACTTCTGGTCAAAGCCGTGTCCATCTCTGTAGCAGAGACACTTCTGGTCAAAGCCGT
TP38856_Hit	D	1	chr2	.	CAGCGGCACTTTCGCTTGCTGTGGAGGTGTCCAATTTAGATGATTTTAATGGGTCTACTGATGA
TP38856_Query	D	1	chr2	.	CAGCGGCACTTTCGCTGGCTGTGGAGGTGTCCAATTTAGATGATTTTAATGGGTCTACTGATGA
TP38970_Hit	D+G	1	chr2	.	CAGCGGCCCAGTACTTCATAAAGTTCTTTTTTACTCGCTTCATAAGATCCACGATATAGTCACC
TP38970_Query	D+G	1	chr2	.	CAGCGGCCCAGTACTTCATAAAGTTCTTCTTTACTCGCTTCATAAGATCCACGATATAGTCACC
TP3925_Hit	D+G	1	chr2	.	CAGCAACCAATGTATTCTCATCAATCCAACCCTTGAAAACTGATCCAAAACCACCTTCTCCTAA
TP3925_Query	D+G	1	chr2	.	CAGCAACCAATGTATTCTCATCAATCCAACCCTTGAAAACTGACCCAAAACCACCTTCTCCTAA
TP39391_Hit	D	1	chr2	.	CAGCGGGAGCAGAGGCCGTAACTGATTAACTATTCATAACAAACTGATTTCAATCTAGATTTCT
TP39391_Query	D	1	chr2	.	CAGCGGGAGCAAAGGCCGTAACTGATTAACTATTCATAACAAACTGATTTCAATCTAGATTTCT
TP39392_Hit	D	1	chr2	.	CAGCGGGAGCAAATCATGTTCACTTTGTCGAGGAATTGGTAAAACTAATGCACCGTGATCATTT
TP39392_Query	D	1	chr2	.	CAGCGGGAGCAAATCATGTTCACTTTGTCGAGGAATTGGTAAAACTAATGCACCATGATCATTT
TP39436_Hit	D	1	chr2	.	CAGCGGGCAAACTTCTCCTACCTTCAGCCTAGTTGCTACTAATCCATTTGGCTTCAGGGAAGAG
TP39436_Query	D	1	chr2	.	CAGCGGGCAAACTTCTCCTACCTTCAGCCTAGTCGCTACTAATCCATTTGGCTTCAGGGAAGAG
TP39728_Hit	D	1	chr2	.	CAGCGGTATGTTTTCATGATTTTTGACATGTTTCAATGAAGTAGAAGGCTATTGTTTGGTCCTA
TP39728_Query	D	1	chr2	.	CAGCGGTATGTTTTCATGATTTTTGACATGTTTCAATGAAGTAGAAAGCTATTGTTTGGTCCTA
TP39762_Hit	D	1	chr2	.	CAGCGGTCCTGTTCGGGAAGTTCGGTGGTTGCTGAAATTGAAGCAAGGAACCCGGAAGCTGTTT
TP39762_Query	D	1	chr2	.	CAGCGGTCCTGTTCGGGAAGTTCGGTGGTTGCTGAAATTGAAGCAAGGAACCCAGAAGCTGTTT
TP39825_Hit	D	1	chr2	.	CAGCGGTGATAGAGTAATCCGTGTGTAAGTTGTTATGTCATTTTTATCAGTGATTTTATGATTC
TP39825_Query	D	1	chr2	.	CAGCGGTGATAGAGTAATCCGTGTGTAAGTTGTTATGTCATTTTTATCAGTGATTTTATGAATC
TP3989_Hit	D+G	1	chr2	.	CAGCAACCCGCTACCGGCTACCCAGCCGCACCACCCTACAACAACCACAACGGCGCCGCCCCTC
TP3989_Query	D+G	1	chr2	.	CAGCAACCAGCTACCGGCTACCCAGCCGCACCACCCTACAACAACCACAACGGCGCCGCCCCTC
TP4024_Hit	D	1	chr2	.	CAGCAACCATGCTATTAAACCAAATCTTGCGCTTTCTTACCTCGTCATACTTTACTGCAGGAAT
TP4024_Query	D	1	chr2	.	CAGCAACCATGCTATTAAACCAAATCTTGCGCTTTCTTACCTCGTCATACTTCACTGCAGGAAT
TP40257_Hit	D	1	chr2	.	CAGCGTAGAAGGTCTCATTCAGTTTCACCACAACGTTCAAGGTTAGGTGCTTCTGCTTCATCTG
TP40257_Query	D	1	chr2	.	CAGCGTAGAAGGTCTCACTCAGTTTCACCACAACGTTCAAGGTTAGGTGCTTCTGCTTCATCTG
TP40295_Hit	D	1	chr2	.	CAGCGTAGGTCTTTAGCTTCTAAACAGGAAAAGTACACGCCTCGATATAAAATTTTGCTAAAGT
TP40295_Query	D	1	chr2	.	CAGCGTAGGTCTTTAGCTTCTAAACAGGAAAAGTACACACCTCGATATAAAATTTTGCTAAAGT

Name	Filter	Nb hit (Mt4.0)	Mt Chr (Mt4.0)	Ms Chr (Li et al., 2014)	Sequence
TP40313_Hit	D	1	chr2	.	CAGCGTATAATTTGATATTGTACATGTTGGCTAGCCATGTATGTATGTATGTATGTATGTACTT
TP40313_Query	D	1	chr2	.	CAGCGTATAATTTGATATTGTACATGTTAGCTAGCCATGTATGTATGTATGTATGTATGTACTT
TP40489_Hit	D	1	chr2	.	CAGCGTCATGCATCTGTGCAGGCCATCCATTTCTGGACCCGAGCATTGGATAACCATCATAACG
TP40489_Query	D	1	chr2	.	CAGCGTCATGCATCTGTGCAGGCCATCCATTTCTGGACCCGAGCATTGAATAACCATCATAACG
TP4052_Hit	D	1	chr2	.	CAGCAACCCAATTGATGCACAAGTGCACAACCAGAGCTTTTACATTTTGAGTCCATCTCATATT
TP4052_Query	D	1	chr2	.	CAGCAACCCAATTGATGCACAAGTGCACAACCAGAGCTTTTACATTTTGAGTCCATCTCATATG
TP40594_Hit	D+G	1	chr2	.	CAGCGTCGGAACAGTGTTTCATCATCGATGACAGACCTAACAAACCCTCTCGTATCACCAAAGA
TP40594_Query	D+G	1	chr2	.	CAGCGTCGGAACAGTGTCTCATCATCGATGACAGACCTAACAAACCCTCTCGTATCACCAAAGA
TP40716_Hit	D+G	1	chr2	.	CAGCGTCTTCTCCGGCGGTGGTTGTTTTTGAACCGTCATCTTCGTTGCCGGATCCGGTTGGACC
TP40716_Query	D+G	1	chr2	.	CAGCGTCTTCTCCGGCGGTGGTTGTTTCTGAACCGTCATCTTCGTTGCCGGATCCGGTTGGACC
TP4086_Hit	D+G	1	chr2	.	CAGCAACTCTTCCTGAACTTCGGACGCCCAACTTTCCGAACGACACACTAGATAATCCTTTGAA
TP4086_Query	D+G	1	chr2	.	CAGCAACCCTTCCTGAACTTCGGACGCCCAACTTTCCGAACGACACACTAGATAATCCTTTGAA
TP40926_Hit	D+G	1	chr2	.	CAGCGTGTGATTCCATGGGGGCTCCTGTTTTGAGGCTAACATTTTGGAGATTCTGCTGAAAAAA
TP40926_Query	D+G	1	chr2	.	CAGCGTGCGATTCCATGGGGGCTCCTGTTTTGAGGCTAACATTTTGGAGATTCTGCTGAAAAAA
TP41011_Hit	D	1	chr2	.	CAGCGTGGATAGGGGCGGCCCTGTCTTAACGCAATTATTTAATGATGTTGGTGGGTCCCTCTTT
TP41011_Query	D	1	chr2	.	CAGCGTGGATAGGGGCGGCCCTGTCTTAACGCAATTATTTAATGATGTTGGTGGGTCCCACTTT
TP41227_Hit	D	1	chr2	.	CAGCGTTAAGGAGGCGCATTTTAGAGGAGTAAGAAAGAGACCGTGGGGAAGATACGCCGCGGAG
TP41227_Query	D	1	chr2	.	CAGCGTTAAGGAGGCGCATTTTAGAGGAGTAAGAAAGAGACCATGGGGAAGATACGCCGCGGAG
TP41232_Hit	D	1	chr2	.	CAGCGTTAATGAAACTAGGCAAAGCTCGACTTCCAACAAAAGCTTCAGCGTCAATTTTCAGGTT
TP41232_Query	D	1	chr2	.	CAGCGTTAATGAAACTAGGCAAAGCTCGACTTCCAACAAAAGCTTCAGCATCAATTTTCAGGTT
TP41238_Hit	D	1	chr2	.	CAGCGTTACAAATTCAATGCAAAGTTGCTTTGAGAGCATTGGATCTCAATCTACACTCCATTAA
TP41238_Query	D	1	chr2	.	CAGCGTTACAAATTCAATGCAAAGTTGCTTTGAGAGCATTGGATCTCAATCTACACGCCATTAA
TP41415_Hit	D	1	chr2	.	CAGCGTTGATTTCAGCCAATGTAAACCGCTTACCAGCTCCTGTTGAGGTGACAGTACCATATAA
TP41415_Query	D	1	chr2	.	CAGCGTTGATTTCAGCCAATGTAAACCGCTTACCAGCACCTGTTGAGGTGACAGTACCATATAA
TP41499_Hit	D	1	chr2	.	CAGCGTTGTGCAACTGGATTTGGTGTATCAACTTTAGTGGAATGGGTATTGAGGAATGCATCTT
TP41499_Query	D	1	chr2	.	CAGCGTTGTGCAACAGGATTTGGTGTATCAACTTTAGTGGAATGGGTATTGAGGAATGCATCTT
TP41509_Hit	D	1	chr2	.	CAGCGTTGTTCACCTCCTTTGCAGATGGGTTCGAGTTGACTATTAGATACACCTATGGAGTCAG
TP41509_Query	D	1	chr2	.	CAGCGTTGTTCACCTCCTTTACAGATGGGTTCGAGTTGACTATTAGATACACCTATGGAGTCAG
TP41550_Hit	D	1	chr2	.	CAGCGTTTCATCTTCCTGGTTCCCCTCACACCATCTTGCGGGATGAAATTTCTATCCTGAAGGT
TP41550_Query	D	1	chr2	.	CAGCGTTTCATCTTCCTGGTTCCACTCACACCATCTTGCGGGATGAAATTTCTATCCTGAAGGT
TP41738_Hit	D	1	chr2	.	CAGCTAAAACAATGATTGCACGTGCCTTTGAAACTGAAACCTGCAAATAGATAATGAAGAACTA
TP41738_Query	D	1	chr2	.	CAGCTAAAACAATGATTGCACGTGCCTTTGAAACTGAAACCTGCAAATAAATAATGAAGAACTA
TP41939_Hit	D	1	chr2	.	CAGCTAAAGCGGCCTTACCTGACTCGTGGAAGCGACTTATCAACATGGGAGGTAATAACCATTC
TP41939_Query	D	1	chr2	.	CAGCTAAAGCGGCCTTACCTGACCCGTGGAAGCGACTTATCAACATGGGAGGTAATAACCATTC
TP41944_Hit	D	1	chr2	.	CAGCTAAAGCTGGAGAACATGTCCCACTTTGTCCAGGGGTCCACATGCGGGAATCACTGCCACA
TP41944_Query	D	1	chr2	.	CAGCTAAAGCTGGAGAACATGTCCCACTTTGTCCAGGGGTCCACATGCGGGAATCACTCCCACA
TP42136_Hit	D	1	chr2	.	CAGCTAACAACATTTTTGATGTGTATCTGCAGGTAAGTGAACAATTAATAAATATCTCGGGTAT
TP42136_Query	D	1	chr2	.	CAGCTAACAACATTTTTGATGTGTATCTGCAGGTAAATGAACAATTAATAAATATCTCGGGTAT
TP42395_Hit	D+G	1	chr2	.	CAGCTAAGATGTTGGCACCAACAATCAAACCAATAGCAATTGGTGCAATTGTCCCTAATGATCC
TP42395_Query	D+G	1	chr2	.	CAGCTAAGATGTTGGCACCAACAATCAAACCAATAGCAATTGGTGCAATTGTCCCCAATGATCC
TP42425_Hit	D	1	chr2	.	CAGCTAAGCGTCCTTTATAAACCTTTGCGAATCCACCTTGACCTATATTGTTTTTGTTGCTGAA
TP42425_Query	D	1	chr2	.	CAGCTAAGCGTCCTTTATAAACCTTTGCGAATCCACCTTGACCTATATTGTTTTTGGTGCTGAA
TP42910_Hit	D	1	chr2	.	CAGCTACAAATGTTAATGACAGCAATGAGTCTTTGGCAGATCCCATTCTCCTATGTTTTACTTT
TP42910_Query	D	1	chr2	.	CAGCTACAAATGTTAATGACAGCAAGGAGTCTTTGGCAGATCCCATTCTCCTATGTTTTACTTT
TP42948_Hit	D	1	chr2	.	CAGCTACAAGAAAAGCCCCTTCATCAGGTGATTCAGCTTCGTAAGTACAGCTGTTGCTCTCCTC
TP42948_Query	D	1	chr2	.	CAGCTACAAGAAAAGCCCCTTCATCAGGTGATTCAGCTTCATAAGTACAGCTGTTGCTCTCCTC
TP42964_Hit	D	1	chr2	.	CAGCTACAAGCTTTAGCTTTACCTTCAACGTGTGTGTATTGATTGAGAAAACAAACACGTACAA
TP42964_Query	D	1	chr2	.	CAGCTACAAGCTTTAGCTTTAACTTCAACGTGTGTGTATTGATTGAGAAAACAAACACGTACAA
TP42991_Hit	D	1	chr2	.	CAGCTACAATGCTGTTATTAGCGAATGCTTCATCATACTTGTATTCATAAACGTTTATGATATT
TP42991_Query	D	1	chr2	.	CAGCTACAATGCTGTTATTAGCGAATGCTTCATCATACTTGTATTCATAAACGCTTATGATATT
TP43095_Hit	D	1	chr2	.	CAGCTACAGCAATTGCATCAAAAGGTTCTCATATTTTGGACGTAGATGCATCACATGATCTCCC
TP43095_Query	D	1	chr2	.	CAGCTACAGCAATTGCATCAAAAGGTTCTCATATTTTGGACGTAGATGCATCACATGATCTACC
TP43723_Hit	D+G	1	chr2	.	CAGCTAGAAATGTTGTTATTAGAAAAAGATAGATACATGACATACAGTAGACAACACAGCAGTG
TP43723_Query	D+G	1	chr2	.	CAGCTAGAAATGTTGTTATTAGAAAAAGATAGGTACATGACATACAGTAGACAACACAGCAGTG

Name	Filter	Nb hit (Mt4.0)	Mt Chr (Mt4.0)	Ms Chr (Li et al., 2014)	Sequence
TP44014_Hit	D	1	chr2	.	CAGCTAGCATATGTTCATAAATAGACTCATCAACCTCATGTAACTGATTGAGAAATCATTCTAC
TP44014_Query	D	1	chr2	.	CAGCTAGCATATGTTCATAAATAGACTCATCAACCTCATGTAACTGATTGAGAAATCATTCTAA
TP44024_Hit	D	1	chr2	.	CAGCTAGCATGTAATTTTGAGTTCACTGTTGATCGGGAAAAGGCAAGTAAGCATTGGCAACACT
TP44024_Query	D	1	chr2	.	CAGCTAGCATGTAATTTTGAGTTCACTGATGATCGGGAAAAGGCAAGTAAGCATTGGCAACACT
TP44092_Hit	D	1	chr2	.	CAGCTAGTTGAGACTGATTAAACAACAAAATACAGAAGTCAAGTATATGAAACACATCAGTGAC
TP44092_Query	D	1	chr2	.	CAGCTAGCTGAGACTGATTAAACAACAAAATACAGAAGTCAAGTATATGAAACACATCAGTGAC
TP44162_Hit	D	1	chr2	.	CAGCTAGGCCCTCTAATGTTTGGCCAGCCAAATGAAGAAATCGCTCTGATGATCTGTCAACTTT
TP44162_Query	D	1	chr2	.	CAGCTAGGCCCTCTAATGTTTGGCCAGCCAAATGAAGAAATCGCTCCGATGATCTGTCAACTTT
TP44194_Hit	D	1	chr2	.	CAGCTAGGGGAAAAGTGTCTTCTTTGGCATCAGGAGATATTCATGCATATAGCTTTTCATGGCT
TP44194_Query	D	1	chr2	.	CAGCTAGGGGAAAAGTGTCTTCTTTGGCATCAGGAGATATTCATACATATAGCTTTTCATGGCT
TP44222_Hit	D+G	1	chr2	.	CAGCTAGGTAATTCAACATTTTAAATAATAGAGGTTTTCATGTGATGCTAAGATTCCATCTAGC
TP44222_Query	D+G	1	chr2	.	CAGCTAGGTAATTCAACATTTTAAATAATAGAGGTTTTCATGTGATGCTAAGATTCCATCCAGC
TP44272_Hit	D	1	chr2	.	CAGCTAGTAATCTTCGAGCAACTTCTCCCTCATATGCTCATGTCTCGCCTTGGCCAAGACCGCC
TP44272_Query	D	1	chr2	.	CAGCTAGTAATCTTCGAGCAACTTCTCCCTCATATGCTCATGTCTCGCCTTGGCAAAGACCGCC
TP44361_Hit	D+G	1	chr2	.	CAGCTAGTGCTAACCAAAAGGCATCCAATAAAGGGCGGGGGAAAGGTGGTCCTGGAATTGTTAA
TP44361_Query	D+G	1	chr2	.	CAGCTAGTGCTAACCAAAAGGCATCCAATAAAGGGCGGGGGAAAGGTGGTCCTGGAATCGTTAA
TP44380_Hit	D+G	1	chr2	.	CAGCTAGTTTACCTGCATTTGTTTGTCTTCTTGCTACACCGTTGATTTTATACACGATCTATCC
TP44380_Query	D+G	1	chr2	.	CAGCTAGTGTACCTGCATTTGTTTGTCTTCTTGCTACACCGTTGATTTTATACACGATCTATCC
TP44426_Hit	D	1	chr2	.	CAGCTAGTTGAGACCACAGCAGTTATTTTCTCTCAAAAATTTGTGGATATGGATGTTTTGTCTG
TP44426_Query	D	1	chr2	.	CAGCTAGTTGAGACCACAGCAGTTATTTTCTCTCAAAAACTTGTGGATATGGATGTTTTGTCTG
TP44448_Hit	D	1	chr2	.	CAGCTAGTTTGAAGAGGACCAGGATTGGATTTACTTCATCAAAGCTAGAATTTCCAGTTCCAAG
TP44448_Query	D	1	chr2	.	CAGCTAGTTTGAAGAGGACCAGGATTGGATTCACTTCATCAAAGCTAGAATTTCCAGTTCCAAG
TP44555_Hit	D	1	chr2	.	CAGCTTTAATTAATTATTTATGCCATATTGCTAATTTGATATGCAACATGATCACCCATATCAT
TP44555_Query	D	1	chr2	.	CAGCTATAATTAATTATTTATGCCATATTGCTAATTTGATATGCAACATGATCACCCATATCAT
TP44637_Hit	D	1	chr2	.	CAGCTATTGCACATGATGTAGCTGTTCTAGCACTTCATGGTACTTCAGCAATGTTCAATTTCCC
TP44637_Query	D	1	chr2	.	CAGCTATAGCACATGATGTAGCTGTTCTAGCACTTCATGGTACTTCAGCAATGTTCAATTTCCC
TP44745_Hit	D	1	chr2	.	CAGCTATATATTGTGTTACTCCATGGGATTTCCAAAGGGGGCAAACTTGCATATTTATTAATGA
TP44745_Query	D	1	chr2	.	CAGCTATATATTGTGTTACTCCATGGGATTTCCAAAGGGGGCAAACTTGCATATTTATTAACGA
TP44746_Hit	D	1	chr2	.	CAGCTATATATTTGTAGTAACGAGTTAGTTCAAGGGCCATCAAGCTTAAACTATCTCTAAACTT
TP44746_Query	D	1	chr2	.	CAGCTATATATTTGTAGTAACGAGTTAGTTCAAGGGCCATCAAGCTTAAACTATCTCCAAACTT
TP44821_Hit	D	1	chr2	.	CAGCTATATTTTGTATTACTTATGACTGAATCAGTGACATGTTTTATGTTTGTATATAATAGTG
TP44821_Query	D	1	chr2	.	CAGCTATATTTTGTATTACTTATGACTGAATCAGTGACATATTTTATGTTTGTATATAATAGTG
TP44841_Hit	D	1	chr2	.	CAGCTATCAAGCGCAACAGAGTGCTTGTAACCAACCTCCTTTAGAAAGGACAGGAGGGGAAGCA
TP44841_Query	D	1	chr2	.	CAGCTATCAAGCGCAACAGAGTGCTTGTAACCAACCTCCTTTAGAAAGAACAGGAGGGGAAGCA
TP44868_Hit	D	1	chr2	.	CAGCTATCACCTTCTGAACTGCAAATCATGTCTTCTCTTGTTCCATCCAACAACTTCGGCACCA
TP44868_Query	D	1	chr2	.	CAGCTATCACCTTCTCAACTGCAAATCATGTCTTCTCTTGTTCCATCCAACAACTTCGGCACCA
TP45024_Hit	D+G	1	chr2	.	CAGCTATGAAGGGGATCAAACCAAGCTCACTGCAGATAAACTAAGAGAAGAAGAACAATGTAAC
TP45024_Query	D+G	1	chr2	.	CAGCTATGAAGGGGATCAAACCAAGCTCACTGTAGATAAACTAAGAGAAGAAGAACAATGTAAC
TP45177_Hit	D	1	chr2	.	CAGCTATGGATGGTGTTGACATCATAAGTTTATCGATCACTCCAAATAGGCGTCCTCCCGGTGT
TP45177_Query	D	1	chr2	.	CAGCTATGGATGGTGTTGACATCATAAGTTTATCGATCACTCCAAATAGACGTCCTCCCGGTGT
TP45231_Hit	D	1	chr2	.	CAGCTATGTAGAAAAGCTGTGTAGCTGTAAGGATAATCAGAAAAGCCTCCATAGTTTTCTGCAA
TP45231_Query	D	1	chr2	.	CAGCTATGTAGAAAAGCTGTGTAGCTGTAAGGATAATCAGAAAAGCCTCCATAGCTTTCTGCAA
TP45444_Hit	D	1	chr2	.	CAGCTATTGAGTAATCTTATTATGTAATCAGATACTGAATATCTTTATAAGTAACTTTATGAAC
TP45444_Query	D	1	chr2	.	CAGCTATTGAGTAATCTTATTATGTAATCAGATACTGAATATCTTTATAAGCAACTTTATGAAC
TP455_Hit	D+G	1	chr2	.	CAGCAAAACCTTTATTAAACTTCATGGGAGTGACTTCTGTAAGTACTTTTGTTTAAACATTACA
TP455_Query	D+G	1	chr2	.	CAGCAAAACCTTTATTAAACTTCATGGGAGTGACTTCTGTAAGTACTTTTGTTTAAACATCACA
TP456_Hit	D	1	chr2	.	CAGCAAAACGAAAATTTATCAGCGTCGCTTGACGGGATCAAAATTGGATACAGCGATCACCGCA
TP456_Query	D	1	chr2	.	CAGCAAAACGAAAATTTATCAGCGTCGCTTGACGGGATCAAAATTGGATACAGCAATCACCGCA
TP45712_Hit	D	1	chr2	.	CAGCTCAAAATGTTACCCTACAATCTCACTATCTTCACCGTTGATTCTCTTTTAATCCCTTACG
TP45712_Query	D	1	chr2	.	CAGCTCAAAATGTTACCCTACAATCTCACTATCTTCACCGTTGATTCTCTCTTAATCCCTTACG
TP46220_Hit	D	1	chr2	.	CAGCTCACCGCCAACTGTATAGAATTTTGGGATTTGACAGTTTTTTGAGATCAAATACTGTTAC
TP46220_Query	D	1	chr2	.	CAGCTCACCGCCAACTGTAGAGAATTTTGGGATTTGACAGTTTTTTGAGATCAAATACTGTTAC
TP46316_Hit	D+G	1	chr2	.	CAGCTCACTTCTTCACAATTGTAAAGTAAAGTGTTGAGGCGTGTAACTGAATTAGAATCAACCA
TP46316_Query	D+G	1	chr2	.	CAGCTCACTTCTTCACAATTGTAAAGTAAAGTGTTGAGGCGTGCAACTGAATTAGAATCAACCA

Name	Filter	Nb hit (Mt4.0)	Mt Chr (Mt4.0)	Ms Chr (Li et al., 2014)	Sequence
TP46318_Hit	D+G	1	chr2	.	CAGCTCACTTCTTCATAATTGTGGAGTAAAGTGTTAAGGTGCGTAATTGAGTTAGAATCAACTG
TP46318_Query	D+G	1	chr2	.	CAGCTCACTTCTTCATAATTGTGGAGTAAAGTGTTAAGGTGCGTAACTGAGTTAGAATCAACTG
TP46322_Hit	D+G	1	chr2	.	CAGCTCACTTTAGTTCCTCCAGTTCAAACTGACCAGGTTTCTGCTTCAGGGTCTGGGTCTGCTG
TP46322_Query	D+G	1	chr2	.	CAGCTCACTTTAGTTCCTCCAGTTCAAACTGACCAGATTTCTGCTTCAGGGTCTGGGTCTGCTG
TP46346_Hit	D	1	chr2	.	CAGCTCAGAAGCACCATTTGTGATTGATGATGATGATGAGATGTATGAAACTGTATCTGATGAT
TP46346_Query	D	1	chr2	.	CAGCTCAGAAGCACCATTTGTGATCGATGATGATGATGAGATGTATGAAACTGTATCTGATGAT
TP46391_Hit	D	1	chr2	.	CTGCTCAGATGAGCATCGGAAAACAATGGAAGGAGTTTCTTGATTATAATGAAACCATTGGCTT
TP46391_Query	D	1	chr2	.	CAGCTCAGATGAGCATCGGAAAACAATGGAAGGAGTTTCTTGATTATAATGAAACCATTGGCTT
TP46409_Hit	D	1	chr2	.	CAGCTCAGCAACTTAATTTTCCTATTGTCTCTTCTGAACACATAAAGAGTTCTGACAGAGCTAT
TP46409_Query	D	1	chr2	.	CAGCTCAGCAACTTAATTTTCCTATTGTCTATTCTGAACACATAAAGAGTTCTGACAGAGCTAT
TP46514_Hit	D	1	chr2	.	CAGCTCAGGCTAGTGACTTCGCAAAAAGATTTACAAATCAAGGGCCACATGTTGGTACCTTTAA
TP46514_Query	D	1	chr2	.	CAGCTCAGGCTAGTGACTACGCAAAAAGATTTACAAATCAAGGGCCACATGTTGGTACCTTTAA
TP46647_Hit	D	1	chr2	.	CAGCTCATAGTATAAAAATTCTAGTTTTTGCTTAGACAAGATTTTGATGGTATATTCACAATCT
TP46647_Query	D	1	chr2	.	CAGCTCATAGTATAAAAATTCTAGTTTTTGCTTAGACAAGATTTTGATGGTATATTCACAATCG
TP46762_Hit	D+G	1	chr2	.	CAGCTCATGAATGACTTATCTTCCCAGTTATTGGTTTCAAAATTGAGACATTTTTGTTTGCGGT
TP46762_Query	D+G	1	chr2	.	CAGCTCATGAATGACTTATCTTCCCAGTTATTGGTTTCAAAATTGAAACATTTTTGTTTGCGGT
TP46828_Hit	D	1	chr2	.	CAGCTCATGTGTTTGCATCCTTTGTCAAAAATTTACTATATATCTTGCCAAACAAATCAAATAT
TP46828_Query	D	1	chr2	.	CAGCTCATGTGTTCGCATCCTTTGTCAAAAATTTACTATATATCTTGCCAAACAAATCAAATAT
TP46980_Hit	D	1	chr2	.	CAGCTCCAAATGTAAGAACAGTTATAGCTAAAGGAGAAGTAGTCAAAACTCCTATGCTAACCGC
TP46980_Query	D	1	chr2	.	CAGCTCCAAATGTAAGAACAGTTATAGCTAAAGGAGAAGTAGTCAAAACTCCTATGCTAACAGC
TP47105_Hit	D+G	1	chr2	.	CAGCTCCACCATTCTCAATTCAACCTTTCCCACAAGATGACTAATTCCATCACAAGAAACAACT
TP47105_Query	D+G	1	chr2	.	CAGCTCCACCATTATCAATTCAACCTTTCCCACAAGATGACTAATTCCATCACAAGAAACAACT
TP47131_Hit	D	1	chr2	.	CAGCTCCACTAAAGCCAGGTGTTCTCATGGCAACTACCTCAAGAGAAACATCACCATCAAACTT
TP47131_Query	D	1	chr2	.	CAGCTCCACTAAAGCCAGGTGTTCTCATGGCAACCACCTCAAGAGAAACATCACCATCAAACTT
TP47199_Hit	D	1	chr2	.	CAGCTCCAGCTCCAGTTGCCGTACCAGCTCCAGCTCCAGTAGCCGTACCAGTTGAAGCTCCAGC
TP47199_Query	D	1	chr2	.	CAGCTCCAGCTCCAGTTGCCGTACCAGCTCCAGCTCCAGTACCCGTACCAGTTGAAGCTCCAGC
TP47230_Hit	D+G	1	chr2	.	CAGCTCCAGTTGATTCAATAATCTCCAAATTCTTTTTCTGCGTAGCCAACCGACGTGCTGAAAA
TP47230_Query	D+G	1	chr2	.	CAGCTCCAGTTGATTCAATAATCTCCAAATTCTTTTTCTGCGTAGCCAACCGACGAGCTGAAAA
TP47279_Hit	D	1	chr2	.	CAGCTCCATCCCCACTTTCCTTTCTTTTCTTTCTCCACTTGGATTTCCTAAGGCAGTGTTCAAC
TP47279_Query	D	1	chr2	.	CAGCTCCATCCCCACTTTCCTTTCTTTTCTTTCTCCACTTGGATTTCCTAAGGAAGTGTTCAAC
TP4734_Hit	D	1	chr2	.	CAGCAACTGCAATAAGCGGGTTGGTTTGACAGGATTTAATTGTCGATGTGGTAACCTTTACTGT
TP4734_Query	D	1	chr2	.	CAGCAACTGCAATAAGCGGGTTGGTTTGACAGGATTTAATTGTCGATGTGGTAACCTTTACGGT
TP47419_Hit	D	1	chr2	.	CAGCTCCCCAAAGTTCGCGCCCAATGATCGGTTAAATTTTTTCATCGATGTTCTTTCACATTCA
TP47419_Query	D	1	chr2	.	CAGCTCCCCAAAGCTCGCGCCCAATGATCGGTTAAATTTTTTCATCGATGTTCTTTCACATTCA
TP47464_Hit	D	1	chr2	.	CAGCTCCCGGTGTTGTTTATGGCAGATGGTGGAATATGGCATAAGGCCAAAAATCCTCCATATA
TP47464_Query	D	1	chr2	.	CAGCTCCCGGTGTTGTTTATGGCAGATGGGGGAATATGGCATAAGGCCAAAAATCCTCCATATA
TP47737_Hit	D	1	chr2	.	CAGCTCCTCAATCAACACATTGTAGAACTTCTGGATGTCACTTAGCATTCTTTCGTCATCAGTT
TP47737_Query	D	1	chr2	.	CAGCTCCTCAATCAACACATTGTAGAACTTCTGGATGTCACTCAGCATTCTTTCGTCATCAGTT
TP48192_Hit	D	1	chr2	.	CAGCTCGCAGAAATGTAACCGGACAACATAATCAAAATCAGGATCTACTTCAAACTTCCATGAC
TP48192_Query	D	1	chr2	.	CAGCTCGCAGAAATGTAAACGGACAACATAATCAAAATCAGGATCTACTTCAAACTTCCATGAC
TP48279_Hit	D	1	chr2	.	CAGCTCGCGAGTTAAAGCAAACTGTAAGTGACATAAAGCACGAGGTACATTACCAATTAGAACA
TP48279_Query	D	1	chr2	.	CAGCTCGCGAGTTAAAGCAAACTGTAAGCGACATAAAGCACGAGGTACATTACCAATTAGAACA
TP48482_Hit	D	1	chr2	.	CAGCTCGTCGGTTGGCTACGCAGAAAAAGAATTTGGAGATTATTGAATCAACTGGTGCGGAGAT
TP48482_Query	D	1	chr2	.	CAGCTCGTCGGTTGGCTACGCAGAAAAAGAATTTGGAGATTATTGAATCAACTGGTGCCGAGAT
TP48521_Hit	D+G	1	chr2	.	CAGCTCGTGTTTGGTGGTAAGCCATGGTGTGGTGTAAAAACAGAAAAACAATATCTAATATTGG
TP48521_Query	D+G	1	chr2	.	CAGCTCGTGTTTGGTGGTAAGCCATGGTGTGGTGTAAAAACAGAAAAACAATATATAATATTGG
TP48598_Hit	D	1	chr2	.	CAGCTCTAAGCCATCACCACCAGAATCTCAGACTCAACAGCACAACAACAACAATGGTGCTGAA
TP48598_Query	D	1	chr2	.	CAGCTCTAAGCCATCACAACCAGAATCTCAGACTCAACAGCACAACAACAACAATGGTGCTGAA
TP48667_Hit	D	1	chr2	.	CAGCTCTAGATCCATTGCCTGGAGCAGACGCCGCTATCCTATCGATCGTGCTGAAAAAAAAAAA
TP48667_Query	D	1	chr2	.	CAGCTCTAGATCCATTGCCTGGAGCAGACGCCGCTATCCTATCGATCATGCTGAAAAAAAAAAA
TP48689_Hit	D	1	chr2	.	CAGCTCTAGTATTGAAGCATAATTATGAAGAACGCGTCCTTGCTCAACAAACTGGTCAAATTTT
TP48689_Query	D	1	chr2	.	CAGCTCTAGTATTGAAGCATAATTATGAAGAACGCGTCCTTGCTCAACAAACTGATCAAATTTT
TP48844_Hit	D	1	chr2	.	CAGCTCTCAGCATAATCCTCAGCTCTGCTCTGCTCAGGTAATTCTTCATGCACAAATCTCTTTA
TP48844_Query	D	1	chr2	.	CAGCTCTCAGCATAATCCTCAGCTCTGCTCCGCTCAGGTAATTCTTCATGCACAAATCTCTTTA

Name	Filter	Nb hit (Mt4.0)	Mt Chr (Mt4.0)	Ms Chr (Li et al., 2014)	Sequence
TP48876_Hit	D	1	chr2	.	CAGCTCTCCAAAACAGTTCTCGTATTTTAAAAGAAACGTGCATGGTAGGATTCTGTATCCTATA
TP48876_Query	D	1	chr2	.	CAGCTCTCCAAAACAGTTCTCGTATTTTAAAAGAAACATGCATGGTAGGATTCTGTATCCTATA
TP49010_Hit	D	1	chr2	.	CAGCTCTCTGGACAATGAATCTGATCCTGATTTGTCAAAAGAAAATCTCGAGCCAATCAAACCA
TP49010_Query	D	1	chr2	.	CAGCTCTCTGGACAATGAATCTGATCCTGATTTGCCAAAAGAAAATCTCGAGCCAATCAAACCA
TP49095_Hit	D+G	1	chr2	.	CAGCTCTGCAAGCTCTTTTACTTAAGTGTTTGCAGGATGAGACTAGCAACCGAGTTCGAGGTGC
TP49095_Query	D+G	1	chr2	.	CAGCTCTGCAAGCTCTTTTACTCAAGTGTTTGCAGGATGAGACTAGCAACCGAGTTCGAGGTGC
TP49176_Hit	D+G	1	chr2	.	CAGCTCTGGGAATTGTCAAATAATCCACTGGAGGCTAATTCACAAACAAGAGTGCCTGCCATTG
TP49176_Query	D+G	1	chr2	.	CAGCTCTGGGAATTGTCAAATAATCCACTGGAGGCTAATACACAAACAAGAGTGCCTGCCATTG
TP49358_Hit	D	1	chr2	.	CAGCTCTTCGAGGTCTTGAAGATTTTGATTTTGAAGATTTCCTCCTTGATGATTTTTTTCCACT
TP49358_Query	D	1	chr2	.	CAGCTCTTCGAGGTCTGGAAGATTTTGATTTTGAAGATTTCCTCCTTGATGATTTTTTTCCACT
TP49383_Hit	D	1	chr2	.	CAGCTCTTCTGATTCCCAATCTTAACTCTCCGTTTTCACCCCTATATAGAGATCCATGAGCAGT
TP49383_Query	D	1	chr2	.	CAGCTCTTCTGATTCCCAATCTTAACTCTCCGTTTTCACCCCTACATAGAGATCCATGAGCAGT
TP49385_Hit	D+G	1	chr2	.	CAGCTCTTCTGGACTTCTAGTCAGCTTATATACGTATTGATTATGAGAACAATCGGTTGAAGTT
TP49385_Query	D+G	1	chr2	.	CAGCTCTTCTGGACTTCTAGTCAGCTTATATATGTATTGATTATGAGAACAATCGGTTGAAGTT
TP49441_Hit	D	1	chr2	.	CAGCTCTTGCAAATATAGAAAGTAAGTCCGATCTTTTGAAACTAAATTTAAGGTGGAATGCTAC
TP49441_Query	D	1	chr2	.	CAGCTCTTGCAAAAATAGAAAGTAAGTCCGATCTTTTGAAACTAAATTTAAGGTGGAATGCTAC
TP49453_Hit	D	1	chr2	.	CAGCTCTTGCCAGTATCTTCAAATTGATGGTACCGCTCCAATGTTCGTTTAACAAATCCCAATG
TP49453_Query	D	1	chr2	.	CAGCTCTTGCCAGTATCTTCAAATTCATGGTACCGCTCCAATGTTCGTTTAACAAATCCCAATG
TP49516_Hit	D	1	chr2	.	CAGCTCTTGTTAGTGGTGGAACTGCCCTTCTGTTTTCACCAATGACATTCATCAAGAAACCACT
TP49516_Query	D	1	chr2	.	CAGCTCTTGTTAGCGGTGGAACTGCCCTTCTGTTTTCACCAATGACATTCATCAAGAAACCACT
TP49520_Hit	D	1	chr2	.	CAGCTCTTGTTGATGTGTATGCTAAATGTGGCTTTCTAGACGAGGCTATGGAGTTATTTGAGAG
TP49520_Query	D	1	chr2	.	CAGCTCTTGTTGATGTGTATGCTAAATGTGGCTTTCTAGACGAGGCTATGGAGATATTTGAGAG
TP49527_Hit	D	1	chr2	.	CAGCTCTTTAGTGCCATAGAACAGAAACTTTACCTGGAAAAACACAGCTTCACCATGATTGCAT
TP49527_Query	D	1	chr2	.	CAGCTCTTTAGTGCCATAGAAAAGAAACTTTACCTGGAAAAACACAGCTTCACCATGATTGCAT
TP49557_Hit	D	1	chr2	.	CAGCTCTTTCTGATTGGCAATCCTTGTTTTTGCCATTTGTTGCACTACCAAGCTCAGTGGATTT
TP49557_Query	D	1	chr2	.	CAGCTCTTTCTGATTGGCAATCCTTGTTTTTGCCATTAGTTGCACTACCAAGCTCAGTGGATTT
TP49867_Hit	D	1	chr2	.	CAGCTGAAATTAGAGATTCATCAAAGAAAGGTTCAAGAGTTTGGCTTGGTACATTTGATACAGC
TP49867_Query	D	1	chr2	.	CAGCTGAAATTAGAGATTCATCAAAGAAAGGTGCAAGAGTTTGGCTTGGTACATTTGATACAGC
TP50287_Hit	D	1	chr2	.	CAGCTGACAAATCCAAATTATGTTGTTCTTCCAGAAGCATCACAATCACTCTCCTTGCCGGTTA
TP50287_Query	D	1	chr2	.	CAGCTGACAAATCCAAATTATGCTGTTCTTCCAGAAGCATCACAATCACTCTCCTTGCCGGTTA
TP50407_Hit	D	1	chr2	.	CAGCTGACTTAGGAGCAAAGTATTTTATCACTTTTTTTATGAGTGACATAAGCATATACCTAAA
TP50407_Query	D	1	chr2	.	CAGCTGACTTAGGAGCAAAATATTTTATCACTTTTTTTATGAGTGACATAAGCATATACCTAAA
TP50431_Hit	D	1	chr2	.	CAGCTGAGAACATGTGGGAAATAAATAGAACAGAGTATGCGCCGAACAGAGAAATGGCTGTGCG
TP50431_Query	D	1	chr2	.	CAGCTGAGAACATGTGGAAAATAAATAGAACAGAGTATGCGCCGAACAGAGAAATGGCTGTGCG
TP50495_Hit	D	1	chr2	.	CAGCTGAGTCGGCTCCTCCTCCCGCGGAGAAACAGGAGGAAAAACCGGTTGAACCAGCTGAAAA
TP50495_Query	D	1	chr2	.	CAGCTGAGCCGGCTCCTCCTCCCGCGGAGAAACAGGAGGAAAAACCGGTTGAACCAGCTGAAAA
TP50636_Hit	D	1	chr2	.	CAGCTGAGTTACGGATACCTGTTAAAGGCATGCATGTGTATGGTTTTGCAGAGCATGGGAATGA
TP50636_Query	D	1	chr2	.	CAGCTGAGTTACGGATACCTGTTAAAGGCATGCATGTGTATGGTTTTGCAGAGCATGGAAATGA
TP50784_Hit	D+G	1	chr2	.	CAGCTGATCCTAAGAAAGGATCATTGGGGACAATTGCACCAATTGCTATTGGTTTGATTGTTGG
TP50784_Query	D+G	1	chr2	.	CAGCTGATCCTAAGAAAGGATCATTAGGGACAATTGCACCAATTGCTATTGGTTTGATTGTTGG
TP50926_Hit	D+G	1	chr2	.	CAGCTGATGTCGACAATCAAGTCTCAGACAGGCAGAGGGGGCCAACAATTGGGTCCCATGGCTC
TP50926_Query	D+G	1	chr2	.	CAGCTGATGTCGACAATCAAGTCTCAGACAGGCAGAGAGGGCCAACAATTGGGTCCCATGGCTC
TP50931_Hit	D	1	chr2	.	CAGCTGATGTGCTCTTGACAAGGAAGCCAAAACACTTTGCAGAAAAAAAAAAAAAAAAAAAAAA
TP50931_Query	D	1	chr2	.	CAGCTGATGTGCTCCTGACAAGGAAGCCAAAACACTTTGCAGAAAAAAAAAAAAAAAAAAAAAA
TP50982_Hit	D	1	chr2	.	CAGCTGATTCAGAAGGGAGTACTAAGGTTTACCTGTCTCAGATTTCTAATGAAGAAAATCAAGG
TP50982_Query	D	1	chr2	.	CAGCTGATTCAGAAGGGAGTACAAAGGTTTACCTGTCTCAGATTTCTAATGAAGAAAATCAAGG
TP51107_Hit	D	1	chr2	.	CAGCTGGAAAGAGTAGTGGTTTGGTTAACAAAGGTAAAGTTAAGGATAATACTCATAACTTTGG
TP51107_Query	D	1	chr2	.	CAGCTGGAAAGAGTAGTGCTTTGGTTAACAAAGGTAAAGTTAAGGATAATACTCATAACTTTGG
TP51185_Hit	D	1	chr2	.	CAGCTGGAATAATTGTGTCCAACCATGGAGCAAGACAACTTGATTATGTTCCAGCCACCATTAG
TP51185_Query	D	1	chr2	.	CAGCTGGAATAAATGTGTCCAACCATGGAGCAAGACAACTTGATTATGTTCCAGCCACCATTAG
TP51191_Hit	D	1	chr2	.	CAGCTGGAATAGCATTAAACCATGCTGACATTTCACTCCAAACCAATTCTTGAGACTGTGAAAC
TP51191_Query	D	1	chr2	.	CAGCTGGAATAGCATTAAACCATGCTGACATTTCACTCCAAACCAATTCTTGAGACTGCGAAAC
TP51293_Hit	D+G	1	chr2	.	CAGCTGGAGCATTTCACAACATATTTGAAGTACGTGAATCAGCCATGACTATGGAAGCACGAAA
TP51293_Query	D+G	1	chr2	.	CAGCTGGAGCATTTCACAACATATTTGAAGTACGTGAATAAGCCATGACTATGGAAGCACGAAA

Name	Filter	Nb hit (Mt4.0)	Mt Chr (Mt4.0)	Ms Chr (Li et al., 2014)	Sequence
TP51352_Hit	D	1	chr2	.	CAGCTGGATACTTGGTTATTGTTCCTGATCTTCTATATGGTGACTACTTTGATATTGGTAATCC
TP51352_Query	D	1	chr2	.	CAGCTGGATACTTGGTTATTGTTCCTGATCTTCTATATGGTGACTACTTTGATATTGATAATCC
TP51478_Hit	D	1	chr2	.	CAGCTGGCCTAGCAAATGACCACTTAATCACTTGCCACAGGCACTTGCAATGCACATTATTGGC
TP51478_Query	D	1	chr2	.	CAGCTGGCCTAACAAATGACCACTTAATCACTTGCCACAGGCACTTGCAATGCACATTATTGGC
TP5163_Hit	D	1	chr2	.	CAGCAAGAATGCCGATATGCATGCTGTCACTTGATATTACCGATGAAGATAGTGCTGGCTGAAA
TP5163_Query	D	1	chr2	.	CAGCAAGAATGCCAATATGCATGCTGTCACTTGATATTACCGATGAAGATAGTGCTGGCTGAAA
TP5164_Hit	D+G	1	chr2	.	CAGCAAGAATGCCGATATGCATGCTGTCACTTGATATTACCGATGAAGATAGTGCTGGTTGCTG
TP5164_Query	D+G	1	chr2	.	CAGCAAGAATGCCAATATGCATGCTGTCACTTGATATTACCGATGAAGATAGTGCTGGTTGCTG
TP51649_Hit	D+G	1	chr2	.	CAGCTGGGGGGAATAGTACTGATCTTTATGAGCTGGATTCAGCTGAGGTATTCGATCCGATTAG
TP51649_Query	D+G	1	chr2	.	CAGCTGGGGGGAATAGTACTGATCTTTATGAGCTGGATTCAGCTGAGGTATTAGATCCGATTAG
TP517_Hit	D+G	1	chr2	.	CAGCAAAACTGTACCTGTGGAGGAAATACTAACAATTGAAATCCAACCAGGTTGGAAAAAGGGA
TP517_Query	D+G	1	chr2	.	CAGCAAAACTGTACCTGTGGAGGAAATACTAACAATTGAAATCCAACCAGGCTGGAAAAAGGGA
TP51890_Hit	D	1	chr2	.	CAGCTGGTGCTGTGGCAAGGATGATTATGGTGCACCCGGAGTCTATTCCTTTAAATCAGGTGAT
TP51890_Query	D	1	chr2	.	CAGCTGGTGCTGTGGCAAGGATGATTATGGTACACCCGGAGTCTATTCCTTTAAATCAGGTGAT
TP51958_Hit	D	1	chr2	.	CAGCTGGTTCAACCGGTTTTTCCTCCTGTTTCTCCGCGGGAGGAGGAGCCGGCTCAGCTGAAAA
TP51958_Query	D	1	chr2	.	CAGCTGGTTCAACCGGTTTTTCCTCCTGTTTCTCCGCGGGAGGAGGAGCCGACTCAGCTGAAAA
TP52003_Hit	D	1	chr2	.	CAGCTGGTTTGACTTGGAGGCTACCTGAATCCTCCTGCGGTGAAGCTAAATTGAGAACCTCGTT
TP52003_Query	D	1	chr2	.	CAGCTGGTTTGACTTGGAGGCTAACTGAATCCTCCTGCGGTGAAGCTAAATTGAGAACCTCGTT
TP52084_Hit	D+G	1	chr2	.	CAGCTGTAATCTGGTTCAGAATTTAGGTGTTTAGTTTCTGAATAAATTAGAAAGAAAAGGTGTA
TP52084_Query	D+G	1	chr2	.	CAGCTGTAATCTGGTTCAGAATTTAGGTGTTTAGATTCTGAATAAATTAGAAAGAAAAGGTGTA
TP52124_Hit	D+G	1	chr2	.	CAGCTGTACCATCACTATTTGAGGAAGATGTTGAATTTGTAGGCGTAGAGTTAGAAGGTACTGA
TP52124_Query	D+G	1	chr2	.	CAGCTGTACCATCACTATTTGAGGAAGATGTTGAATTTGTAGGCGTAGAGCTAGAAGGTACTGA
TP52381_Hit	D	1	chr2	.	CAGCTGTCGATGATATCTGAATTGGGACCTAACAATGAAATCTCCGATTCCTTATAGCCTAGCT
TP52381_Query	D	1	chr2	.	CAGCTGTCGATGATATCTGAATTGGGACCTAACAATGAAATCTCCGATTCCTTATAGCCCAGCT
TP52421_Hit	D	1	chr2	.	CAGCTGTCTGTCCAACTCAAATGGTAGCCTTCACCAAGGTACATCTCAAGATAATGAGTTTCAA
TP52421_Query	D	1	chr2	.	CAGCTGTCTGTCCAACTCAAATGGTAGCCTTCACCAAGGTACATCTCAAGATAATGAATTTCAA
TP52426_Hit	D+G	1	chr2	.	CAGCTGTCTTATCTTCCACCTCTCTCTTGCCGATATGATTCCGGTCAGTCTCTGCAACTTTCTC
TP52426_Query	D+G	1	chr2	.	CAGCTGTCTTATCTTCCACCTCTCTCTCGCCGATATGATTCCGGTCAGTCTCTGCAACTTTCTC
TP52589_Hit	D+G	1	chr2	.	CAGCTGTGGAGAAAGTAGGAGTTGCTGATGTCATGAGCCACATATCCACCGGTGGTGGTGCAAG
TP52589_Query	D+G	1	chr2	.	CAGCTGTGGAGAAAGTAGGAGTTGCCGATGTCATGAGCCACATATCCACCGGTGGTGGTGCAAG
TP52616_Hit	D	1	chr2	.	CAGCTGTGGCTTCTTGATATACATCAGTGACAAATAAAATATCTGATGGCTTATCCACACCAAG
TP52616_Query	D	1	chr2	.	CAGCTGTGGCTTCTTGATATACATCAGTGACAAATAAAATATCTGATGGCTTATCAACACCAAG
TP52768_Hit	D	1	chr2	.	CAGCTGTTACATAGTTTTTTCTGCAATAGAACTTTAATTAATCATTTCAACTTTCTTGGTACTG
TP52768_Query	D	1	chr2	.	CAGCTGTTACATAGTTTTTTCTGCAATAGAACTTTAATTAATCATTTCAACTTTCTTGGTACTA
TP5302_Hit	D+G	1	chr2	.	CAGCAAGAGCAGGGAATTTAGATGTTGTGAGGGATATTTTTAATGATGCTCAAGAGGATGAATT
TP5302_Query	D+G	1	chr2	.	CAGCAAGAGCAGGGAATTTAGATGTTGTGAGGGATATTCTTAATGATGCTCAAGAGGATGAATT
TP53124_Hit	D	1	chr2	.	CAGCTGTTTGATCGCTCTGGCATGTTGTATGGGGAGCTAGCTAGATTTGTGTTGCGGTTGCTCG
TP53124_Query	D	1	chr2	.	CAGCTGTTTGATCGCTCCGGCATGTTGTATGGGGAGCTAGCTAGATTTGTGTTGCGGTTGCTCG
TP53158_Hit	D	1	chr2	.	CAGCTGTTTTCCCATGAAATTCTACCATAAGCAGATTATTTTTAAATATTATTTTTATAATCAC
TP53158_Query	D	1	chr2	.	CAGCTGTTTTCCCATGAAATTCTACCATAAGCAGATTATTTCTAAATATTATTTTTATAATCAC
TP53395_Hit	D+G	1	chr2	.	CAGCTTAAGGATTTGAGTATCAAAGATTTAATGGTGGAAAATGTGGAGGAAGACGATCCTAACA
TP53395_Query	D+G	1	chr2	.	CAGCTTAAGGATTTGAGTATCAAAGATTTAATGGTGGAAAATATGGAGGAAGACGATCCTAACA
TP53463_Hit	D	1	chr2	.	CAGCTTAATGGCAATGTTCTGGCGAGTTGCCTATTGCAGGGGCGGCCGTAAATCACGGTGCTAC
TP53463_Query	D	1	chr2	.	CAGCTTAATGGCAATGCTCTGGCGAGTTGCCTATTGCAGGGGCGGCCGTAAATCACGGTGCTAC
TP536_Hit	D	1	chr2	.	CAGCAAAAGAAACCATCTTTATCAGAAGAAGAATCTGAAATTCAGAAAGAAACTGATTCAAAAA
TP536_Query	D	1	chr2	.	CAGCAAAAGAAACCATCTTTATCAGAAGAAGAATCTGAAATTCAGAAAGAAACTGATCCAAAAA
TP53647_Hit	D+G	1	chr2	.	CAGCTTACGAAACCAGCTTGTATGATGACACGTTTACTTTATTTCATCTTTTATTATAGAAATA
TP53647_Query	D+G	1	chr2	.	CAGCTTACGAAAACAGCTTGTATGATGACACGTTTACTTTATTTCATCTTTTATTATAGAAATA
TP53783_Hit	D	1	chr2	.	CAGCTTAGCTAATTTAAAAGGCTGGATATCATCAAGTATTAACCAATTTTTACATGACAATTCT
TP53783_Query	D	1	chr2	.	CAGCTTAGCTAATTTAAAAGGCTGGATATCATCAAGTATTAAACAATTTTTACATGACAATTCT
TP54291_Hit	D	1	chr2	.	CAGCTTATTGCCCCAAGGCTTTTCAAAGGAGAGAATTCATTTCCCCTCCTAGGAGTTGCTAATA
TP54291_Query	D	1	chr2	.	CAGCTTATTGCCCCAAGGCTTTTCAAAGGAGAGAATTCATTTCCCCTCCTAGGAGTTGCTAACA
TP54346_Hit	D	1	chr2	.	CAGCTTATTTGTACGAGGCAAAACATCCTTTAGTCCTATGTACTGATGACACATGTGTATTCAA
TP54346_Query	D	1	chr2	.	CAGCTTATTTGTACAAGGCAAAACATCCTTTAGTCCTATGTACTGATGACACATGTGTATTCAA

Name	Filter	Nb hit (Mt4.0)	Mt Chr (Mt4.0)	Ms Chr (Li et al., 2014)	Sequence
TP54396_Hit	D+G	1	chr2	.	CAGCTTCAAACGATGGAAATTGATAATGGTGCAATGCACAAGCAGTTGAATGAACTGGTAAGCA
TP54396_Query	D+G	1	chr2	.	CAGCTTCAAACAATGGAAATTGATAATGGTGCAATGCACAAGCAGTTGAATGAACTGGTAAGCA
TP54470_Hit	D	1	chr2	.	CAGCTTCAACATTACTTGGTCCGTAAGTCCAACTACAAATCAAATAGTACATTATATTAAGAAT
TP54470_Query	D	1	chr2	.	CAGCTTCAACATCACTTGGTCCGTAAGTCCAACTACAAATCAAATAGTACATTATATTAAGAAT
TP54542_Hit	D	1	chr2	.	CAGCTTCAAGGAAGCATATATCCTTCACATACCATCAAGATGCCTTCATCTATATCCAGGTTGG
TP54542_Query	D	1	chr2	.	CAGCTTCAAGGAAGCATATATCCTTCACATACCATCAAGATGCCTTCATCTATATCCAGGTTAG
TP54630_Hit	D+G	1	chr2	.	CAGCTTCAATTCCTTAGTCTTGGATCAAATAGGCTCTTTGGAAATATTCCTTATAGCTTGAAAA
TP54630_Query	D+G	1	chr2	.	CAGCTTCAATTCCTTAGCCTTGGATCAAATAGGCTCTTTGGAAATATTCCTTATAGCTTGAAAA
TP54699_Hit	D	1	chr2	.	CAGCTTCACATTTTCATCAAGCTTTTTTCTCAGTGGCACAGAGGTATTAGTCATCTACCATCTT
TP54699_Query	D	1	chr2	.	CAGCTTCACATTTTCATCAAGCTTTTTTCTCAGTGGCACAGAGGTATTAGTCATCCACCATCTT
TP54831_Hit	D	1	chr2	.	CAGCTTCAGATTATTAAGAGATATAGTAAAGGAAAAATTTCGAATCTCAAGATCAGTTTTGATA
TP54831_Query	D	1	chr2	.	CAGCTTCAGATTATTAAGAGATATAGTAAAGGAAAAATTTCCAATCTCAAGATCAGTTTTGATA
TP54862_Hit	D	1	chr2	.	CAGCTTCAGCTATTGATCAACAAGCAGTAGTCCTCCGTCTTTTTCTCAGCAAACTCTGATATCA
TP54862_Query	D	1	chr2	.	CAGCTTCAGCTATTGATCAACAAGCAGTAGTCCTCCGTCTTTTTCTCAACAAACTCTGATATCA
TP55057_Hit	D+G	1	chr2	.	CAGCTTCGTCTGGATTTGTAGGAGATAATACTGGTGCGGGAGCTTCCACAGGTGATATTCCCGG
TP55057_Query	D+G	1	chr2	.	CAGCTTCATCTGGATTTGTAGGAGATAATACTGGTGCGGGAGCTTCCACAGGTGATATTCCCGG
TP55112_Hit	D	1	chr2	.	CAGCTTCATTCAAGAGGTTTGCAAGATCAGCTCCACTAAAGCCAGGTGTTCTCATGGCAACTAC
TP55112_Query	D	1	chr2	.	CAGCTTCATTCAAGAGGTTTGCAAGATCAGCTCCACTAAAGCCAGGTGTTCTCATGGCAACCAC
TP55177_Hit	D	1	chr2	.	CAGCTTCCAACACCTTTATTGCGCTGGGTGTAAAGGGTTGATTTAGTCCCACTTAAGATATGGC
TP55177_Query	D	1	chr2	.	CAGCTTCCAACACCTTTATTGCGCTGGGTGTAAAGGGTTGATTTAGTCCCACATAAGATATGGC
TP55189_Hit	D	1	chr2	.	CAGCTTCCAAGCATCCATTCTCCTCTACCTGTGAGCTTGCGTCGCTTAAGGCATCTTCCTCAGC
TP55189_Query	D	1	chr2	.	CAGCTTCCAAGCATCCATTCTCCTCTACCTGTGAGCTTGCGTCGCTTAAGGCATCTTCCTCAGA
TP55221_Hit	D	1	chr2	.	CAGCTTCCACCGGTTTACACAATTGGGCCTGTTATTGATCTTGTTGGACCAGCCGAATGGGATC
TP55221_Query	D	1	chr2	.	CAGCTTCCACCGGTTTACACAATTGGGCCAGTTATTGATCTTGTTGGACCAGCCGAATGGGATC
TP55314_Hit	D+G	1	chr2	.	CAGCTTCCCCAATTTATCAGCACACTGGTGTGAATACTTTGCTGGAGTCAATCGTTTTATCCTC
TP55314_Query	D+G	1	chr2	.	CAGCTTCCCCAATTTATCAGCACACTGGTGTGAATACTTTGCTGGAGTCAATCATTTTATCCTC
TP55480_Hit	D+G	1	chr2	.	CAGCTTCCTTGAGCTTCTTTGTGATGGTGGCAAGATATTTCCCTTGATGGAATGCTTGCTGAAA
TP55480_Query	D+G	1	chr2	.	CAGCTTCCTTGAGCTTCTTTGTGATGGTGGCAAGATATTTCCCTTGATGGAATGCGTGCTGAAA
TP55647_Hit	D+G	1	chr2	.	CAGCTTGGTATGGTAACAATGAAATTGTGAATCTTGTAATTGAACGTGTTCCAAAACTTTTGTT
TP55647_Query	D+G	1	chr2	.	CAGCTTCGTATGGTAACAATGAAATTGTGAATCTTGTAATTGAACGTGTTCCAAAACTTTTGTT
TP5588_Hit	D	1	chr2	.	CAGCAAGCAAGTGGCTTTCCTCTATCATCCATATTGATCTTGATACTTTGCAAAACCTTGAATC
TP5588_Query	D	1	chr2	.	CAGCAAGCAAGGGGCTTTCCTCTATCATCCATATTGATCTTGATACTTTGCAAAACCTTGAATC
TP55902_Hit	D	1	chr2	.	CAGCTTCTCCTTCACCATCTGATCATCCACAACAACCAGCTGATGACCAACAAAGTGTGTTACC
TP55902_Query	D	1	chr2	.	CAGCTTCTCCTTCACCATCTGATCATCCACAACAACCAGCTGATGACCAACAAAGTATGTTACC
TP55952_Hit	D	1	chr2	.	CAGCTTCTCTTATATCTTCAGGTGAAGTCGATTTTGAATCTGGAAGAAGAGAAACCGAATCAGG
TP55952_Query	D	1	chr2	.	CAGCTTCTCTTATATCTTCAGGTGAAGTCGATTTTGAAACTGGAAGAAGAGAAACCGAATCAGG
TP56015_Hit	D	1	chr2	.	CAGCTTCTGAGTTTGCTTCAAGAAATCATCTGCCACGTCGCGTACACAATCAAATGTTGGCACA
TP56015_Query	D	1	chr2	.	CAGCTTCTGAGTTTGCTTCAAGAAATCATCTGCCACGTCGCGTACACAATCAAATGGTGGCACA
TP5619_Hit	D	1	chr2	.	CAGCAAGCACCATGCATTTAACTCCAGGAGAACATACATAATATGATAGAACAAAATTCTTGAC
TP5619_Query	D	1	chr2	.	CAGCAAGCACCATGCATTTAACTCCAGGAGAACATAAATAATATGATAGAACAAAATTCTTGAC
TP56254_Hit	D+G	1	chr2	.	CTGCTTCTGATGATCTTGCCAAAGTACGAATATCATATCAGGTATGATCATGATGTGTGTGATG
TP56254_Query	D+G	1	chr2	.	CAGCTTCTGATGATCTTGCCAAAGTACGAATATCATATCAGGTATGATCATGATGTGTGTGATG
TP56350_Hit	D	1	chr2	.	CAGCTTCTGCAAGTTCTGATATGTATGGAGGGGCAGTTTTAGATGCATGTGAGCAAATAAAGGC
TP56350_Query	D	1	chr2	.	CAGCTTCTGCAAGTTCTGATATGTATGGAGCGGCAGTTTTAGATGCATGTGAGCAAATAAAGGC
TP56394_Hit	D+G	1	chr2	.	CAGCTTCTGCTTGTTTCGGATTGTGAAACTTGATATTCCAACTTCATTTCTCAATGCACCTATC
TP56394_Query	D+G	1	chr2	.	CAGCTTCTGCTTGTTTCGGATTGTGAAACCTGATATTCCAACTTCATTTCTCAATGCACCTATC
TP56452_Hit	D+G	1	chr2	.	CAGCTTTTGTATGATCTTTGTAGGTCTGGTAAAGCTAGAAAAGCTGTTAGGGTCATGGAGATTA
TP56452_Query	D+G	1	chr2	.	CAGCTTCTGTATGATCTTTGTAGGTCTGGTAAAGCTAGAAAAGCTGTTAGGGTCATGGAGATTA
TP56684_Hit	D	1	chr2	.	CAGCTTCTTTGCTTCGTAGTGTGGATGATGCTGGAGCTGACCAAGCAAGTAGGAAAAGATCAGA
TP56684_Query	D	1	chr2	.	CAGCTTCTTTGCTTCGTAGTGTGGATGATGCTGGAACTGACCAAGCAAGTAGGAAAAGATCAGA
TP5672_Hit	D	1	chr2	.	CAGCGAGCATCTTAAAGACCCGCTCTTGATCCTCGTTGCACATTTGAACGACGTCCAATGCTTT
TP5672_Query	D	1	chr2	.	CAGCAAGCATCTTAAAGACCCGCTCTTGATCCTCGTTGCACATTTGAACGACGTCCAATGCTTT
TP56976_Hit	D	1	chr2	.	CAGCTTGACCACTGCTATAGTGTGTAATTGCACCGAAAAGATTGGTATTATAACCTAAAGCAGG
TP56976_Query	D	1	chr2	.	CAGCTTGACCACTGCTATAGTGTGTAATTGCACCGAAAAGATTGGTATTATAACCTAAAGCAAG

Name	Filter	Nb hit (Mt4.0)	Mt Chr (Mt4.0)	Ms Chr (Li et al., 2014)	Sequence
TP57389_Hit	D+G	1	chr2	.	CAGCTTGCATACATTTCTAGTCAAGTTTAGGTGTCTTGTAGCTAAAACCTAATCTTGAAAATGA
TP57389_Query	D+G	1	chr2	.	CAGCTTGCATACATTTCTAGTCAAATTTAGGTGTCTTGTAGCTAAAACCTAATCTTGAAAATGA
TP57674_Hit	D	1	chr2	.	CAGCTTGGAGCAAATGAGCATTTGAAGTATGCTCTTGGGGATGGATTATCCCTTGTCACCTTTT
TP57674_Query	D	1	chr2	.	CAGCTTGGAGCAAATGAGCATTTGAAGTATGCTCTTGGAGATGGATTATCCCTTGTCACCTTTT
TP57692_Hit	D	1	chr2	.	CAGCTTGGAGTTCTCTTGGAACTGACTCAGTTGAATTTCAAGGTTTGTAACTACATAAGTTTTT
TP57692_Query	D	1	chr2	.	CAGCTTGGAGTTCTCTTGGAACCGACTCAGTTGAATTTCAAGGTTTGTAACTACATAAGTTTTT
TP58002_Hit	D	1	chr2	.	CAGCTTGTAGCAGTACTGTTATTCTCATGGTTTTTTATATCAAATATTTTTCCAAAATTATAAC
TP58002_Query	D	1	chr2	.	CAGCTTGTAGCAGTAATGTTATTCTCATGGTTTTTTATATCAAATATTTTTCCAAAATTATAAC
TP5808_Hit	D	1	chr2	.	CAGCAAGCTATCTTTGCTCTAATGCCTATGCCACCCTGAAATTCCTTCCCAGTTACCTCTCATC
TP5808_Query	D	1	chr2	.	CAGCAAGCTATCTTTGCTCTAAGGCCTATGCCACCCTGAAATTCCTTCCCAGTTACCTCTCATC
TP58294_Hit	D	1	chr2	.	CAGCTTGTTACTTAGTTTACTGTATACAAGCCTGCTGGGACAGAACCCTTTGCGCAAAGCCTCC
TP58294_Query	D	1	chr2	.	CAGCTTGTTACTCAGTTTACTGTATACAAGCCTGCTGGGACAGAACCCTTTGCGCAAAGCCTCC
TP58381_Hit	D	1	chr2	.	CAGCTTGTTGTCTCGAAAGTTGATGCAAATGCTGAAAAAAAAAAAAAAAAAAAAAAAAAAAAAA
TP58381_Query	D	1	chr2	.	CAGCTTGTTGTCTCAAAAGTTGATGCAAATGCTGAAAAAAAAAAAAAAAAAAAAAAAAAAAAAA
TP58686_Hit	D	1	chr2	.	CAGCTTTAGATTCGGTTCAAGGATAAAATGTGCTAGTTAAATTTATGAAATCCAAATACTGGCC
TP58686_Query	D	1	chr2	.	CAGCTTTAGATTCGGTTCAAGGATAAAATGTGCTAGTTAAATTTATGAAATCCAAATACTGGCA
TP58703_Hit	D+G	1	chr2	.	CAGCTTTAGCTGTTTAGTTTGGATACTCATCAACTTTTGTGGCCTAATTGGAGGTTGTTGTGAT
TP58703_Query	D+G	1	chr2	.	CAGCTTTAGCTGTTTAGTTTGGATACTCATCAACTTTTGTGGCCTAATTGGAGGTTGTCGTGAT
TP58704_Hit	D	1	chr2	.	CAGCTTTAGCTTCAGTTTGGAAAGACGAAAAGTATATTGTGTATCTAAAAAAGATCTAGAACAG
TP58704_Query	D	1	chr2	.	CAGCTTTAGCTTCAGTTTGGAAAGACGAAAAGTATATTGTGTATCTAAAAAAGATCTAGAACAA
TP58718_Hit	D+G	1	chr2	.	CAGCTTTAGGTCCCTTGATAGCACTGCAAATCATTCATACAACATCATATATTAGTCTCTAATT
TP58718_Query	D+G	1	chr2	.	CAGCTTTAGGTCCCTTGATAGCACTGCAAATCATTCATACAACATCATATATTAGTCTCTAATC
TP58752_Hit	D+G	1	chr2	.	CAGCTTTATAAAATAGTGAAAGGTTGACCAAAGGAATATTAGAGTTACCTCTGGTTTGTTCTTG
TP58752_Query	D+G	1	chr2	.	CAGCTTTATAAAATAGTGAAAGGTTGACCAAAGGAATATTAGAGTCACCTCTGGTTTGTTCTTG
TP58789_Hit	D	1	chr2	.	CAGCTTTATCATCAATATTGCCACAAGAGATATTATCACCAGTCTCCTGCACCAATCGAGGCGC
TP58789_Query	D	1	chr2	.	CAGCTTTATCATCAATATTGCCACAAGAGATATTATCACCAGTCTCCTGCACCAATCGAGACGC
TP58869_Hit	D	1	chr2	.	CAGCTTTATTTTAATATCTTGATCGCAAGCAGAACCTTTGTATTCTTGGTCTTGCAACTTGGCT
TP58869_Query	D	1	chr2	.	CAGCTTTATTTTAATATCTTGATCGCAAGCAAAACCTTTGTATTCTTGGTCTTGCAACTTGGCT
TP59164_Hit	D	1	chr2	.	CAGCTTTCTAGACAGTTAGATGAAATTAAACAAGGTAATTATACATTCTAAGATTTTGTACCTT
TP59164_Query	D	1	chr2	.	CAGCTTTCTAGACAGTTAGATGAAATTAAACAAGGTAATTATACATTCTAAAATTTTGTACCTT
TP59681_Hit	D	1	chr2	.	CAGCTTTGTACAGTCCCGAGGCCGTGCCAGAATGCGAAATTCAGATTACATATTGATGGTAAAA
TP59681_Query	D	1	chr2	.	CAGCTTTGTACAGTCCCGAGGCCGTGCCAGAATGCGAAATTCAGATTACATACTGATGGTAAAA
TP59702_Hit	D	1	chr2	.	CAGCTTTTTCAGAATTAGGAAATCTTCGATTCTAGTTCTATTGAACTTGATGCTCATTAATTTT
TP59702_Query	D	1	chr2	.	CAGCTTTGTCAGAATTAGGAAATCTTCGATTCTAGTTCTATTGAACTTGATGCTCATTAATTTT
TP59788_Hit	D	1	chr2	.	CAGCTTTGTTTATTGGTATGTTCTGCCATAATTAACTATAATTTACTACAGGTGGCTGATATTG
TP59788_Query	D	1	chr2	.	CAGCTTTGTTTATGGGTATGTTCTGCCATAATTAACTATAATTTACTACAGGTGGCTGATATTG
TP59892_Hit	D	1	chr2	.	CAGCTTTTATATATAGTTTATACATGATAGAAAAGGACATAGTTTTGAATATTAGAGTTTATAT
TP59892_Query	D	1	chr2	.	CAGCTTTTATACATAGTTTATACATGATAGAAAAGGACATAGTTTTGAATATTAGAGTTTATAT
TP5990_Hit	D	1	chr2	.	CAGCAAGGAGAATATTTGTCCATTTCCTTCCATTAAATGGATTTTTTCTGGACATCTTTGAGTC
TP5990_Query	D	1	chr2	.	CAGCAAGGAGAATATTTGTCCACTTCCTTCCATTAAATGGATTTTTTCTGGACATCTTTGAGTC
TP6005_Hit	D	1	chr2	.	CAGCAAGGAGGCTAATGGACTTGGGCTTAGTCTTCACACCCATTCAAGATGCTATCAGAGAAGC
TP6005_Query	D	1	chr2	.	CAGCAAGGAGGCTAATAGACTTGGGCTTAGTCTTCACACCCATTCAAGATGCTATCAGAGAAGC
TP60190_Hit	D+G	1	chr2	.	CAGCTTTTGGACTTATGCCTCCAAAGGAGCAGGTGATGAATTCAATACTTTTCACTCATTTCTG
TP60190_Query	D+G	1	chr2	.	CAGCTTTTGGACTTATGCCTCCAAAGGAGCAGGTGATGAATTCAATACTTCTCACTCATTTCTG
TP60204_Hit	D+G	1	chr2	.	CAGCTTTTGGGGTCTATATCTCTCGAGCGGCATCATCTCTGTCGTCGCGCTTGTTCTGTTTCTG
TP60204_Query	D+G	1	chr2	.	CAGCTTTTGGGGTCTATATCTCTCGAGCGGCATCATCTCTGTCGTCGCGCTTGTTCTCTTTCTG
TP60295_Hit	D	1	chr2	.	CAGCTTTTTACCAGATCATCTGGGTGATACACAACTGAAAATGAGAAACTTTGTTCTTGGTACT
TP60295_Query	D	1	chr2	.	CAGCTTTTTACCAGATCATCTGGGTGATACACAACTGAAAATGAGAAACTTTGTTCTTGGCACT
TP60312_Hit	D	1	chr2	.	CAGCTTTTTATTTCCTCTCGTTATTAATAGCCTTAAACATAATGCCTTGGCAGGTGGAGGTTGC
TP60312_Query	D	1	chr2	.	CAGCTTTTTATTTCCTCTCGTTATTAATAGCCTTAAACATAATGCCTTGGCAGGGGGAGGTTGC
TP6052_Hit	D	1	chr2	.	CAGCAAGGCACTAAAACATACAGAATCCATAAGAAACCCCTTCCTCAGCATCTTAGTTTGGAAC
TP6052_Query	D	1	chr2	.	CAGCAAGGCACTAAAACATACAGAATCCATAAGAAACCCCTTCCTCAGCATCTTAGTTTGCAAC
TP60743_Hit	D+G	1	chr2	.	CTGCAAAAGAGTTACATTCCCTTTCTGTGAGCCACTACAAGGTTGAATTTTCCTGGTTCTAGGG
TP60743_Query	D+G	1	chr2	.	CTGCAAAAGAGTTACATTCCCTTTCTGTGAGCCACTACAAGGTTGAATTTTCCTGGTTCAAGGG

Name	Filter	Nb hit (Mt4.0)	Mt Chr (Mt4.0)	Ms Chr (Li et al., 2014)	Sequence
TP60816_Hit	D	1	chr2	.	CTGCAAAAGTGATTGGAGAAGTTCAAGAACTTATTGTTTTCCCAATCATACCATATGGTATCCT
TP60816_Query	D	1	chr2	.	CTGCAAAAGTGATAGGAGAAGTTCAAGAACTTATTGTTTTCCCAATCATACCATATGGTATCCT
TP60845_Hit	D	1	chr2	.	CTGCAAAATACGCCCTGAAATGCGAAGAACAAGCAGTTTCTCTTGCTTGTCTGGTGCGGAGATC
TP60845_Query	D	1	chr2	.	CTGCAAAATACGCCCTGAAATGCGAAGAACAAGCAGTTTCTCTTGCTTGTCTGGTGCCGAGATC
TP60940_Hit	D	1	chr2	.	CTGCAAAATTAGTTATCAGTACTGTAGCACTCCTGAGAAAATGGAAAGAAAGTTCCATCAAAAT
TP60940_Query	D	1	chr2	.	CTGCAAAATTAGTTAGCAGTACTGTAGCACTCCTGAGAAAATGGAAAGAAAGTTCCATCAAAAT
TP6098_Hit	D	1	chr2	.	CAGCAAGGCTGTGAGAGAAACAACAAAATCAACAACCAGCTTAAAATCACCCCTCAAATTGATT
TP6098_Query	D	1	chr2	.	CAGCAAGGCTGTGAGAGAAACAACAAAATCAACAACCAGCTAAAAATCACCCCTCAAATTGATT
TP61012_Hit	D	1	chr2	.	CTGCAAACACAGCTTCTGCGTTTTCTCGCAATACATCAGCTCTGTTTAGGACCAGATTTTTTGC
TP61012_Query	D	1	chr2	.	CTGCAAACACAGCTTCTGCGTTTTCTCGCAATACATCAGCTCTGTTTAGGACCAGATTTTTTGA
TP61085_Hit	D	1	chr2	.	CTGCAAACCCGAAACACGGATACTTCTTTGCCATGTCCTTATATGCATACTTAAAGCCCTCATG
TP61085_Query	D	1	chr2	.	CTGCAAACCCAAAACACGGATACTTCTTTGCCATGTCCTTATATGCATACTTAAAGCCCTCATG
TP6121_Hit	D	1	chr2	.	CAGCAAGGGATCTTATCAGGGGTTTGCTTGTAAAGGAACCTCAGCACAGATTGGCTTATAAAAG
TP6121_Query	D	1	chr2	.	CAGCAAGGGATCTTATCAGGGGTTTGCTTGTAAAGGAACCTCAGCACAGATTGACTTATAAAAG
TP6132_Hit	D	1	chr2	.	CAGCAAGGGCTGATTGGATGATCCGGAAGTAGAAGCATCCAGACCAACTTGCATGTTCTGATAT
TP6132_Query	D	1	chr2	.	CAGCAAGGGCTGATTGGATGATCCGGAAGTAAAAGCATCCAGACCAACTTGCATGTTCTGATAT
TP61324_Hit	D	1	chr2	.	CTGCAAAGCTACTAAAACCTGAGTGATTATAAGTAGCTGGTACATCCCTTCAGCTCCTGAACTC
TP61324_Query	D	1	chr2	.	CTGCAAAGCTACTAAAACCTGAGTGATTATAAGTAGCTGGTACATCACTTCAGCTCCTGAACTC
TP61356_Hit	D	1	chr2	.	CTGCGAAGGCATTACAACAAGAGTGCAAACAAATGCTTGCAGAATTTGCTACCACCTCTAAGCA
TP61356_Query	D	1	chr2	.	CTGCAAAGGCATTACAACAAGAGTGCAAACAAATGCTTGCAGAATTTGCTACCACCTCTAAGCA
TP61742_Hit	D	1	chr2	.	CTGCAAATGTCTCAAAGAACTTGCTCTGCAATGCCAAGTTTATATCAACAAATGAGAGATCAAG
TP61742_Query	D	1	chr2	.	CTGCAAATGTCTCAAAGAACTTGCTCTGCAATGACAAGTTTATATCAACAAATGAGAGATCAAG
TP61842_Hit	D+G	1	chr2	.	CTGCAAATTGAATTGATTTTCTATGCAAGTTGAATTGATTTATTTGTTGTTGTTGGCTACTTTG
TP61842_Query	D+G	1	chr2	.	CTGCAAATTGAATTGATTTTCTATGCAAGTTGAATTGATTTATTTGTTGTTGTTGGCTACTTTA
TP61934_Hit	D+G	1	chr2	.	CTGCAACAAAAGTCAAAATGGCACAGAAAATGGAACAAGGGGTTGCTGAGAAAAGAAAAGGAAG
TP61934_Query	D+G	1	chr2	.	CTGCAACAAAAGTCAAAATGGCACAAAAAATGGAACAAGGGGTTGCTGAGAAAAGAAAAGGAAG
TP61949_Hit	D+G	1	chr2	.	CTGCAACAAATAATTTTGACGACAGTTTGGTCATCGGAGTTGGAGGCTTTGGTAAGGTGTATAA
TP61949_Query	D+G	1	chr2	.	CTGCAACAAATAATTTCGACGACAGTTTGGTCATCGGAGTTGGAGGCTTTGGTAAGGTGTATAA
TP62150_Hit	D	1	chr2	.	CTGCAACAGATGCTCGCTTGGGAAAATTTGCCTATACTAAGGAAGCAGGAAGTTTACCGCATGC
TP62150_Query	D	1	chr2	.	CTGCAACAGATGCTCGCTTGGGAAAATTTGCCTATACTAAGGAAGCAAGAAGTTTACCGCATGC
TP62158_Hit	D	1	chr2	.	CTGCAACAGCAGGATGCTGTAAGGCGTGTGAGCCGTCCGATCATAAATAAGTGGTTGAGATCAA
TP62158_Query	D	1	chr2	.	CTGCAACAGCAGGATGCTGTAAGGCGTGTGAGCCGTCCGATCATAAATAAGTAGTTGAGATCAA
TP62290_Hit	D+G	1	chr2	.	CTGCTACATGGAGCTTGAGGCTCCTCTTGTTGGATTTATAACTGCTAACTTTCCTGTTACGATG
TP62290_Query	D+G	1	chr2	.	CTGCAACATGGAGCTTGAGGCTCCTCTTGTTGGATTTATAACTGCTAACTTTCCTGTTACGATG
TP62308_Hit	D	1	chr2	.	CTGCAACATTATCCTGCAGACCGCTCTCAAAAGGAAACTGGAAGGCAACAGTCAACGCACATCG
TP62308_Query	D	1	chr2	.	CTGCAACATTATCCTGCAAACCGCTCTCAAAAGGAAACTGGAAGGCAACAGTCAACGCACATCG
TP62459_Hit	D	1	chr2	.	CTGCAACCTATGCTCCATAGGACCCTTTGTCCCATTTCTTCTATTGTTGCTCAAACTTCCTTCA
TP62459_Query	D	1	chr2	.	CTGCAACCTATGCTCAATAGGACCCTTTGTCCCATTTCTTCTATTGTTGCTCAAACTTCCTTCA
TP62579_Hit	D+G	1	chr2	.	CTGCAACGTCATCCGATCATCAATCAACCTCCTGTTATCCGCCACCAGCCTCCTCATTTCAGCG
TP62579_Query	D+G	1	chr2	.	CTGCAACGTCATCCGATCATCAATCAACCTCCTGTTATCCGCCACCAGCCTCCTCATTTCAGCA
TP6258_Hit	D	1	chr2	.	CAGCAAGGTTGATCCTCCTCACCAGTTGGAGAGATCTCCTAAGCTACGCACTCTACTCATTGAA
TP6258_Query	D	1	chr2	.	CAGCAAGGTTGATCCTCCTCACCAGTTGGAGAGATCTCCTAAGCTACGCACCCTACTCATTGAA
TP62618_Hit	D	1	chr2	.	CTGCAACTAAGCCTGACATTGTAGATAAGTCGCCGCCCACTGAAATCTTTGGAGCGTCATCCTG
TP62618_Query	D	1	chr2	.	CTGCAACTAAGCCTGACATTGTAGATAAGTCGCCGCCCACAGAAATCTTTGGAGCGTCATCCTG
TP6262_Hit	D	1	chr2	.	CAGCAAGGTTTAGACTTTGAAGATGTGTTCCTTGTCGAAATGGTAGGTAAAGTGCGCTTAAGCC
TP6262_Query	D	1	chr2	.	CAGCAAGGTTTAGACTTTGAAGATGTGTTCCTTGTCGAAATGGTAGGCAAAGTGCGCTTAAGCC
TP62673_Hit	D+G	1	chr2	.	CTGCAACTATGCGATTATTGAAAATGCTTTCCTTCTTGGACTTCCAGATCACCCAAATTACCGA
TP62673_Query	D+G	1	chr2	.	CTGCAACTATGCAATTATTGAAAATGCTTTCCTTCTTGGACTTCCAGATCACCCAAATTACCGA
TP62685_Hit	D	1	chr2	.	CTGCAACTCAACCCTTGAACCAGGAAAATTCAACCTTGTAGTGGCTCACAGAAAGGGAATGTAA
TP62685_Query	D	1	chr2	.	CTGCAACTCAACCCTAGAACCAGGAAAATTCAACCTTGTAGTGGCTCACAGAAAGGGAATGTAA
TP62687_Hit	D+G	1	chr2	.	CTGCAACTCAATCACCAAGGCCAAGGAGGTACAAGACCTGTGGAGCAGGCCCAAATCCCAGAAA
TP62687_Query	D+G	1	chr2	.	CTGCAACTCAATCACCAAGGCCAAGGAGGTACAAAACCTGTGGAGCAGGCCCAAATCCCAGAAA
TP62730_Hit	D	1	chr2	.	CTGCAACTCCTCCAAACTTTTTCCAAGTTCATCCACTGAGTCAGCAATCTGCTCCTTGCAGTCA
TP62730_Query	D	1	chr2	.	CTGCAACTCCTCCAAACTTCTTCCAAGTTCATCCACTGAGTCAGCAATCTGCTCCTTGCAGTCA

Name	Filter	Nb hit (Mt4.0)	Mt Chr (Mt4.0)	Ms Chr (Li et al., 2014)	Sequence
TP62762_Hit	D+G	1	chr2	.	CTGCAACTCTGGTGAAGAAGATGAAGGAACAAGAGTACCAACGGATGAACCCACAAAGTAGGGT
TP62762_Query	D+G	1	chr2	.	CTGCAACTCTGGTGAAGAAGATGAAGGAACAAGAGTACCAACGGAAGAACCCACAAAGTAGGGT
TP62941_Hit	D+G	1	chr2	.	CTGCAACTTTGGAAGTTTTAATCTTTCTGACAAAGCAGGCATGATATGACCGCGTTCCTCTTCT
TP62941_Query	D+G	1	chr2	.	CTGCAACTTTGGAAGCTTTAATCTTTCTGACAAAGCAGGCATGATATGACCGCGTTCCTCTTCT
TP63119_Hit	D	1	chr2	.	CTGCAAGAGATTGTTGGAAGGAAATTTTGTTGTCACCTGTAAGATTTTTTTGCCATTGATACAG
TP63119_Query	D	1	chr2	.	CTGCAAGAGATTGTTGGAAGGAAATTTTGTTGTCACCTGTAAGATTTTTTTGCCATTGATACAC
TP63266_Hit	D+G	1	chr2	.	CTGCAAGCATCCTACTTCGTCATTACAATTGGTAAATCATCATCTTTTTGGGTCACATGCTATG
TP63266_Query	D+G	1	chr2	.	CTGCAAGCATCCTACTTCGTCATTACAATTGGTAAATCATCATCTTTCTGGGTCACATGCTATG
TP63379_Hit	D	1	chr2	.	CTGCAAGGAATAATCCCTCTTTTAGAATTCCTCAAATACCAAAGAATGAGCAGAGACCTCAACA
TP63379_Query	D	1	chr2	.	CTGCAAGGAATAATCCCTCTTTTAGAATGCCTCAAATACCAAAGAATGAGCAGAGACCTCAACA
TP63400_Hit	D	1	chr2	.	CTGCAAGGAGAGACTTTTCCTGGGGTACTACTCATTCTTTCTCATTTACTGTTCTGTCTAGACT
TP63400_Query	D	1	chr2	.	CTGCAAGGAGAGACTTTTCCTGGGGTACTACTCATTCTTTCTCATCTACTGTTCTGTCTAGACT
TP63473_Hit	D	1	chr2	.	CTGCAAGGTCTTGTATACTTGTAAGTTACATGAATAAATTGATTTGACCTTTTTCTTTTTCTTT
TP63473_Query	D	1	chr2	.	CTGCAAGGTCTTGTATACTTGTAAGTTACATGAATAAATTGATTTGACCTTTCTCTTTTTCTTT
TP63875_Hit	D	1	chr2	.	CTGCAATAGCTGGAGAACATGCCCCTGACTGTCCAGGGGTCCACATGCGCGAACTGCTGAAAAA
TP63875_Query	D	1	chr2	.	CTGCAATAGCTGGAGAACATGCCCCTGACTGTCCAGGGGTCCACATGCGCGAACCGCTGAAAAA
TP64066_Hit	D	1	chr2	.	CTGCAATCAGAACTTTCCCTAGAAGTGCTCTGCACATGACTTCTTTGGGAAGTATGACATTGTG
TP64066_Query	D	1	chr2	.	CTGCAATCAGAACTTTCCCTAGAAGAGCTCTGCACATGACTTCTTTGGGAAGTATGACATTGTG
TP64112_Hit	D+G	1	chr2	.	CTGCAATCCTGAAAATCAATGGTGGAGGAAGGATCATCTGTGGCCGTCCGATTCCGAAGCTGGG
TP64112_Query	D+G	1	chr2	.	CTGCAATCCTGAAAATCAATGGTGGAGGAAGGATCATCTGTGGCCGTCCGATTCCGAAGCGGGG
TP64116_Hit	D	1	chr2	.	CTGCAATCGAATAGAAACTAGAAACAAAATGAACCTCGGCGTCCAGTAGTTCCTGCACTTGTTC
TP64116_Query	D	1	chr2	.	CTGCAATCGAATAGAAACTAGAAACAAAATGAACCTCGGCGTCCAGTAGTTCCTGCACTTGCTC
TP6414_Hit	D	1	chr2	.	CAGCAAGTCTACAAGGTATCCTCTAGTTCACCAACATATAACAATGAGAATGGCAAACTCAGTT
TP6414_Query	D	1	chr2	.	CAGCAAGTCTACAAGGTATCCTCTAGATCACCAACATATAACAATGAGAATGGCAAACTCAGTT
TP64341_Hit	D	1	chr2	.	CTGCAATGCTGAGCAAGAAGTTCTTGAGAGAAGGTGAAACGGCCGCTTCAGCCACATCAGGAAC
TP64341_Query	D	1	chr2	.	CTGCAATGCTAAGCAAGAAGTTCTTGAGAGAAGGTGAAACGGCCGCTTCAGCCACATCAGGAAC
TP64421_Hit	D	1	chr2	.	CTGCAATGGGAGGTGGAACCGGCATGAATCACTTCCTTCGCCGTTTCCCTACTAGATGTTTTGA
TP64421_Query	D	1	chr2	.	CTGCAATGGGAGGTGGAACCGGCATGAATCACTTCCTTCGCCATTTCCCTACTAGATGTTTTGA
TP64837_Hit	D	1	chr2	.	CTGCAATTGTGGGTGGTTCTAAGGTCTCATCTAAAATTGGAGTGATTGAGTCACTTCTAGAAAA
TP64837_Query	D	1	chr2	.	CTGCAATTGTGGGTGGTTCCAAGGTCTCATCTAAAATTGGAGTGATTGAGTCACTTCTAGAAAA
TP64889_Hit	D	1	chr2	.	CTGCAATTTCCTCGTCTGTGTAATCCCTTGAAGGAATAGGAGGAAGCCAATCAGAAAAAGTGTT
TP64889_Query	D	1	chr2	.	CTGCAATTTCCTCATCTGTGTAATCCCTTGAAGGAATAGGAGGAAGCCAATCAGAAAAAGTGTT
TP65309_Hit	D+G	1	chr2	.	CTGCACACATTGATAAAAGATGGGAACTCCACTCATCTATGCAATGGCATTTTGCTTAATTGCA
TP65309_Query	D+G	1	chr2	.	CTGCACACATTGATAAAAGATGGGAACTCCACTCATCTATGCAATGGCATTTTGCCTAATTGCA
TP65350_Hit	D	1	chr2	.	CTGCACACGTGTCCTCTGATCCTGAATCAGTTGCTCTACTTCCATGGTTTCCTCTAACCACTGC
TP65350_Query	D	1	chr2	.	CTGCACACGTGTCCTCTGATCCTGAATCAGTTGCTCTACTTCCATGGGTTCCTCTAACCACTGC
TP65367_Hit	D	1	chr2	.	CTGCACACTGTACAGCAAACATTGCTTGCTCCTCTAGGGTAAAGCAGGATGTTCCTACACCCAG
TP65367_Query	D	1	chr2	.	CTGCACACTGCACAGCAAACATTGCTTGCTCCTCTAGGGTAAAGCAGGATGTTCCTACACCCAG
TP65510_Hit	D	1	chr2	.	CTGCACAGTTGATCAGAAGCTGGAAAGAGCAAGATTCAGGTGGCCGTGCTGAAAAAAAAAAAAA
TP65510_Query	D	1	chr2	.	CTGCACAGTTGATCAGAAGCTGGAAAGAGCAAGATTCAGGTGGCCGGGCTGAAAAAAAAAAAAA
TP65520_Hit	D	1	chr2	.	CTGCACATAAATGCAAAGTTGGTGTGCCATCAATATGCAAGCAACCACACCAAACTACTACAAA
TP65520_Query	D	1	chr2	.	CTGCACATAAATGCAAAGTTGGTGTGCCATCAATATCCAAGCAACCACACCAAACTACTACAAA
TP6558_Hit	D	1	chr2	.	CAGCAAGTTCAACTGTCTGTCCAATCCTTCCGAGTGGATCAACCAATTGAAGCGAGGCTGAAAG
TP6558_Query	D	1	chr2	.	CAGCAAGTTCAACTGTCTGTCCAATCCTTCCGAGTGGATCAACCAATTGAAGCGAGGCTGAAAA
TP65584_Hit	D	1	chr2	.	CTGCACATCCATTAAGTCCCTTGTTATCCTTACATCACCCGGATATTACTGATGCAATCTTCCC
TP65584_Query	D	1	chr2	.	CTGCACATCCATTAAGTCCCTTGTTATCCTTACATCACCCAGATATTACTGATGCAATCTTCCC
TP6564_Hit	D	1	chr2	.	CAGCAAGTTCATCAAATGCTTACACTAAGACATTTACTGGGATTGATGAAGCTGGAAAGCGATT
TP6564_Query	D	1	chr2	.	CAGCAAGTTCATCAAATGCTTACACCAAGACATTTACTGGGATTGATGAAGCTGGAAAGCGATT
TP65828_Hit	D+G	1	chr2	.	CTGCACCACCACTTTCTTCCTCCACATTTTCTCCATAGTCACCTGGCAGATCTTTGACCGATTC
TP65828_Query	D+G	1	chr2	.	CTGCACCACCACTTTCTTCCTCCACATTTTCTCCATAGTCACCTGGCAGATCTTTCACCGATTC
TP66038_Hit	D	1	chr2	.	CTGCACCCCACCAGCCATCTTCTTCTGTTGGAAAACGTGGACCTGTTCCAGGAACTAAGGGCAA
TP66038_Query	D	1	chr2	.	CTGCACCCCACCAGCCATCTTCTTCGGTTGGAAAACGTGGACCTGTTCCAGGAACTAAGGGCAA
TP66079_Hit	D	1	chr2	.	CTGCGCCCTGAACAAAACATGGATTCAAGTATCAGCATCAATAGATTAAACACATTAAAATCAC
TP66079_Query	D	1	chr2	.	CTGCACCCTGAACAAAACATGGATTCAAGTATCAGCATCAATAGATTAAACACATTAAAATCAC

Name	Filter	Nb hit (Mt4.0)	Mt Chr (Mt4.0)	Ms Chr (Li et al., 2014)	Sequence
TP66429_Hit	D	1	chr2	.	CTGCACGAGAATAAAAATGACATTGTTAATTGTTTCTTCAAACGTTATCCAATGAAAATTGCAT
TP66429_Query	D	1	chr2	.	CTGCACGAGAATAAAAATGACATTGTTAATTGTTTCTACAAACGTTATCCAATGAAAATTGCAT
TP665_Hit	D	1	chr2	.	CAGCAAAAGCTGGAATCAAACTTGTCCTTGCCTGCCTCCAGTTGCAGAAGAAATTGGCTGAAAA
TP665_Query	D	1	chr2	.	CAGCAAAAGCTAGAATCAAACTTGTCCTTGCCTGCCTCCAGTTGCAGAAGAAATTGGCTGAAAA
TP66772_Hit	D	1	chr2	.	CTGCACTAGCTTTGGCTGGGCTCCATGTTATCGAGGGTCGAAATGGAGATGCCGTGTCTCTGCT
TP66772_Query	D	1	chr2	.	CTGCACTAGCTTTGGCTGAGCTCCATGTTATCGAGGGTCGAAATGGAGATGCCGTGTCTCTGCT
TP66805_Hit	D+G	1	chr2	.	CTGCACTATCTGTACTTGCTCTCGAACTAACTAGGTTAAGTTGGTCAGCCCAATCATCAGACCT
TP66805_Query	D+G	1	chr2	.	CTGCACTATCTGTACTTGCTCTCGAACTAACTAGGTTAAGTTGGACAGCCCAATCATCAGACCT
TP6682_Hit	D	1	chr2	.	CAGCAATAAAAAAACTTAAGTTTGATTATTAATATTATTAACACTGTTTCAATTTCAAGTAACT
TP6682_Query	D	1	chr2	.	CAGCAATAAAAAAACTTAAGTTTGATTATTAATATTATTAACACTGTTCCAATTTCAAGTAACT
TP67027_Hit	D	1	chr2	.	CTGCACTGCTTTTCCTTCTGGAGATTTCCTCACATTCCTAGCTCTCACACGTGCCTGAACCCTA
TP67027_Query	D	1	chr2	.	CTGCACTGCTTTGCCTTCTGGAGATTTCCTCACATTCCTAGCTCTCACACGTGCCTGAACCCTA
TP67147_Hit	D	1	chr2	.	CTGCACTTCCATCATCGTCACAAGCTGAAATGCAAACTGCTAGCCCACCTCTTGTGCCTTTGTA
TP67147_Query	D	1	chr2	.	CTGCACTTCCATCATCATCACAAGCTGAAATGCAAACTGCTAGCCCACCTCTTGTGCCTTTGTA
TP67262_Hit	D+G	1	chr2	.	CTGCACTTTAAGATTACTCTGCTCATTATTTGGGACACGAGAACCTTGTTCCAACTCGAGGGAA
TP67262_Query	D+G	1	chr2	.	CTGCACTTTAAGATTACTCTGCTCATTATTTGGGACACAAGAACCTTGTTCCAACTCGAGGGAA
TP67268_Hit	D	1	chr2	.	CTGCACTTTATGAGCCTCTTGGATTTGGTACTTCTGGTGGAAAGCATGGTGGTCAGAAAGAGGT
TP67268_Query	D	1	chr2	.	CTGCACTTTATGAGCCTCTTGGATTTGGTACTTCTGGTGGAAAGCATGGTGGTCAGAAAGAGGG
TP67636_Hit	D+G	1	chr2	.	CTGCAGAAGGCGGGAGCTCTTCAATAGCCGAGAGTGTCCGGCGGCGGACCCATTATTCTCAAGG
TP67636_Query	D+G	1	chr2	.	CTGCAGAAGGCGGGAGCTCTTCAATAGCCGAGAGTGTCCGACGGCGGACCCATTATTCTCAAGG
TP68974_Hit	D	1	chr2	.	CTGCAGGAGGAGGAGCACTAGCTGGTTGAGCCACCGGAGACATCGGTACTGAGATCGGAAGAGC
TP68974_Query	D	1	chr2	.	CTGCAGGAAGAGGAGCACTAGCTGGTTGAGCCACCGGAGACATCGGTACTGAGATCGGAAGAGC
TP68976_Hit	D+G	1	chr2	.	CTGCAGGAGGAGGAGCACTAGCTGGTTGAGCCACCGGAGACATCGGTGCTGAAAAAAAAAAAAA
TP68976_Query	D+G	1	chr2	.	CTGCAGGAAGAGGAGCACTAGCTGGTTGAGCCACCGGAGACATCGGTGCTGAAAAAAAAAAAAA
TP6966_Hit	D	1	chr2	.	CAGCAATAGAGTTGATGCATCTTTTGTTGATCATGTATATGGCGCTTCCACGTCAGTTGCCCGG
TP6966_Query	D	1	chr2	.	CAGCAATAGAGTTGATGCATCTTTTGTTGATCATGTATATGGCGCTTCCACGTCAGTTGCCCAG
TP7002_Hit	D+G	1	chr2	.	CAGCAATAGCATCATAAAATTCATCCTGCACTTCTGCATTTTCATATTCAGCTCCGGACAACGA
TP7002_Query	D+G	1	chr2	.	CAGCAATAGCATCATAAAATTCATCCTGCACTTCTGCATTTTCATATTCAGCTCCAGACAACGA
TP70475_Hit	D	1	chr2	.	CTGCATACAGAAATTTAGAAGAAATGGTATTCATGTTGTCCGTGTATCTGCTGGGGATGAGCGT
TP70475_Query	D	1	chr2	.	CTGCATACAGAAATTTAGAAGAAATGGTATTCATGTTGTCCGTGTATCTGCTGGGGATGAGCAT
TP70690_Hit	D	1	chr2	.	CTGCATATATGAGACCTTAAACCTGAAACCATAAATTACATTTAGTCAGAATCCAGATATTGAT
TP70690_Query	D	1	chr2	.	CTGCATATATGAGACCTTAAACCAGAAACCATAAATTACATTTAGTCAGAATCCAGATATTGAT
TP70735_Hit	D	1	chr2	.	CTGCATATCTCTCAGTCCACGGAAATGCCATTTTAGGGCGGTCCCTCTGGCAGAAAACCCATTC
TP70735_Query	D	1	chr2	.	CTGCATATCTCTCAGTCCACGGAAATGCCATTTTAGGGCGGTCCCTCTGGCACAAAACCCATTC
TP70847_Hit	D	1	chr2	.	CTGCATATTTCCTGTCTTCGGTTCTCACTTCTACGTTAGGTGTTTTGGATTGTGATGCATCACT
TP70847_Query	D	1	chr2	.	CTGCATATTTCCTCTCTTCGGTTCTCACTTCTACGTTAGGTGTTTTGGATTGTGATGCATCACT
TP71274_Hit	D	1	chr2	.	CTGCATCCTCAGATGGAACACTTCCACCTTCTATAAAAAGAATAATTTAGTTAGTTCATTGTGA
TP71274_Query	D	1	chr2	.	CTGCATCCTCAGATGGAACACTTCCACCTTCTATAAAAAGAAGAATTTAGTTAGTTCATTGTGA
TP71467_Hit	D	1	chr2	.	CTGCATCTGATAACTTGCTTCGCTGAAGACTTGCACCAAAAGAAGAAGGCTCTGGCTCAAAAAA
TP71467_Query	D	1	chr2	.	CTGCATCTGACAACTTGCTTCGCTGAAGACTTGCACCAAAAGAAGAAGGCTCTGGCTCAAAAAA
TP71644_Hit	D	1	chr2	.	CTGCATGAATAGTTCACTTGAATATATGTTTGCCAACATGAAATGCCTTGAAGAAAAAGGCATC
TP71644_Query	D	1	chr2	.	CTGCATGAATAGTTCACTTGAATATATGTTTGACAACATGAAATGCCTTGAAGAAAAAGGCATC
TP71912_Hit	D	1	chr2	.	CTGCATGGCATGGATGGTCCTAAACAGAAGCTGAAGTAAACGAGGAGGTTAGGAGAAATCACAG
TP71912_Query	D	1	chr2	.	CTGCATGGAATGGATGGTCCTAAACAGAAGCTGAAGTAAACGAGGAGGTTAGGAGAAATCACAG
TP71917_Hit	D	1	chr2	.	CTGCATGGATCATCTCTATCGTTTACTTTGTTTCATTTACCTAAACCAAACAACTATATATAAA
TP71917_Query	D	1	chr2	.	CTGCATGGACCATCTCTATCGTTTACTTTGTTTCATTTACCTAAACCAAACAACTATATATAAA
TP71981_Hit	D+G	1	chr2	.	CTGCATGGGCGAAAGCTGGGAAGAACCAGGATGACGCTCCAAAGATTTCTGTGGGCGGCGACTT
TP71981_Query	D+G	1	chr2	.	CTGCATGGGCGAAAGCTGGGAAGAACCAGGATGACGCTCCAAAGATTTCAGTGGGCGGCGACTT
TP72103_Hit	D	1	chr2	.	CTGCATGTGAAATTTGTAGTAGTTTGGTGTGGTTGCTTGGATATTGATGGCACACCAACTTTGC
TP72103_Query	D	1	chr2	.	CTGCATGTGAAATTTGTAGTAGTTTGGTGTGGTTGCTTGCATATTGATGGCACACCAACTTTGC
TP72112_Hit	D+G	1	chr2	.	CTGCATGTGACAGTCTTTGAAGGGATCAACAACGGTGTTTATTGAGAGTTGATGTTCAGGGATG
TP72112_Query	D+G	1	chr2	.	CTGCATGTGACAGTCTTTGAAGGGATCAACAACGGTGTTTATTGAGAGTTGATGTCCAGGGATG
TP72369_Hit	D	1	chr2	.	CTGCATTATACAGGTAATGTACCATGTAATTCATGGTAAATTTAAACATTTTAGAGACAACACG
TP72369_Query	D	1	chr2	.	CTGCATTATACAAGTAATGTACCATGTAATTCATGGTAAATTTAAACATTTTAGAGACAACACG

Name	Filter	Nb hit (Mt4.0)	Mt Chr (Mt4.0)	Ms Chr (Li et al., 2014)	Sequence
TP72694_Hit	D	1	chr2	.	CTGCATTGCTAAATTTCTAATCGTGGCTATTTTTATACATCTTCATTGGGTTGCAGTAGACCTG
TP72694_Query	D	1	chr2	.	CTGCATTGCTAAATTTCTAATCGTGGCTATTTTTATACATCTTCATTGGGTTGCAGTAGACCCG
TP72725_Hit	D	1	chr2	.	CTGCATTGGCATCTCTGAAGACATTCAGAAGGCCTGAATGCTCGTTGGGGGGGGAGGATGGTCA
TP72725_Query	D	1	chr2	.	CTGCATTGGCATCTCTGAAGACATTCAGAAGGCCTGAATGCTCGTTGGGGGGAGAGGATGGTCA
TP72815_Hit	D+G	1	chr2	.	CTGCATTGTTGCCGTTGCAAGGCAGAGAGGGATTGATGTTGTGCTCAACGATGAGTCCAAGCGT
TP72815_Query	D+G	1	chr2	.	CTGCATTGTTGCCGTTGCAAGGCAGAGAGGGATTGATGTTGTCCTCAACGATGAGTCCAAGCGT
TP72842_Hit	D	1	chr2	.	CTGCATTTAAGATGCATTCCTCAATACCCATTCCACTAAAGTTGATACACCAAATCCTGTTGCA
TP72842_Query	D	1	chr2	.	CTGCATTTAAGATGCATTCCTCAATACCCATTCCACTAAAGTTGATACACCAAATCCAGTTGCA
TP73388_Hit	D	1	chr2	.	CTGCCAAACCTCCGCGACCTCCACAAGGCCCGTCCTTGGACGCTGAAAAAAAAAAAAAAAAAAA
TP73388_Query	D	1	chr2	.	CTGCCAAACCTCCGCGACCTCCACAAGGCCCGTCCTTGGACGCAGAAAAAAAAAAAAAAAAAAA
TP73526_Hit	D	1	chr2	.	CTGCCAAATTTAAGGAGGAATATAAGGTTGATGTTTACCAGAATGCCAGGGCTTGTCTGAGGTT
TP73526_Query	D	1	chr2	.	CTGCCAAATTTAAGGAGGAATATAAGATTGATGTTTACCAGAATGCCAGGGCTTGTCTGAGGTT
TP73695_Hit	D	1	chr2	.	CTGCCAAGACAACTGAAAAAGAATAAAAGGTCAATTGCCTATGAAAGCCAGTTAAAGCATATGT
TP73695_Query	D	1	chr2	.	CTGCCAAGACAACTGAAAAAGAATAAAAGGTCAATTGCCAATGAAAGCCAGTTAAAGCATATGT
TP73747_Hit	D	1	chr2	.	CTGCCAAGGACTGTGCTTGGAGTTTCAAAAGTTGTTGTTTGCGTGCTAGCTGAAAAAAAAA
TP73747_Query	D	1	chr2	.	CTGCCAAGGACTGTGCTTGGAGTTTCAAAAGTTGCTGTTTGCGTGCTAGCTGAAAAAAAAA
TP73814_Hit	D+G	1	chr2	.	CTGCCAATAATGTGCATTGCAAGTGCCTGTGGCAAGTGATTAAGTGGTCATTTGTTAGGCCAGC
TP73814_Query	D+G	1	chr2	.	CTGCCAATAATGTGCATTGCAAGTGCCTGTGGCAAGTGATTAAGTGGTCATTTGCTAGGCCAGC
TP73950_Hit	D	1	chr2	.	CTGCCAATTACTATTTCGCTTGCTGGTTTAAAAGCTAATAGTAGGACTATATTAATATTTTTTG
TP73950_Query	D	1	chr2	.	CTGCCAATTACTATTTCACTTGCTGGTTTAAAAGCTAATAGTAGGACTATATTAATATTTTTTG
TP74011_Hit	D+G	1	chr2	.	CTGCCACAAACTCAGGTCTGATTTAATATTCTGCATAAATCAAAATGAAAATATCAATGCTATA
TP74011_Query	D+G	1	chr2	.	CTGCCACAAACTCAGGTCTGATTTAATATTCTGCATAAATCAAAATGAAAATATCAATACTATA
TP74270_Hit	D	1	chr2	.	CTGCCACCAGTTCCTGAAACCCATCCCAAACTGTAGAAGTGGCGACACAGTTCCGCCATCAGAG
TP74270_Query	D	1	chr2	.	CTGCCACCAGTTCCTGAAACCCATCCCAAACTGTAAAAGTGGCGACACAGTTCCGCCATCAGAG
TP74325_Hit	D	1	chr2	.	CTGCCACCGGCACCGGAACAATCCCAGCCAATTTCTCAACGCTCAACGTCTCACTCCGTAGCTG
TP74325_Query	D	1	chr2	.	CTGCCACCGGCACCGGAACAATCCCAGCCAATTTCTCAACGCTCAACGTCTCACTCCGTAGCAG
TP7462_Hit	D	1	chr2	.	CAGCAATTGCTCTTCAATTGTTTACTCATCCATGTTTGACGAGATAAGACATATGAAAATTTCG
TP7462_Query	D	1	chr2	.	CAGCAATCGCTCTTCAATTGTTTACTCATCCATGTTTGACGAGATAAGACATATGAAAATTTCG
TP74698_Hit	D	1	chr2	.	CTGCCAGAGGGGGGCAACAGGTGGAGAAGATGAACGCGCTGGTGCGGAGATCGGAAGAGCGGTT
TP74698_Query	D	1	chr2	.	CTGCCAGAGGGGGGCAACAGGTGGAGAAGATGAACGCGCTGGTGCCGAGATCGGAAGAGCGGTT
TP74782_Hit	D	1	chr2	.	CTGCCAGCCTGATCTCACAGGGTCCTCCAACTCGCCTTCCAGTCCGAACATTCGGGCGAGCTCA
TP74782_Query	D	1	chr2	.	CTGCCAGCCTGATCTCACAGGGTCCTCCAACTCACCTTCCAGTCCGAACATTCGGGCGAGCTCA
TP7522_Hit	D+G	1	chr2	.	CAGCAATCTGCTCCTGTCCATGAGGTGAAGATGCCGAGTTTGCCGAGTTGGGGTTCGTGGGGTG
TP7522_Query	D+G	1	chr2	.	CAGCAATCTGCTCCGGTCCATGAGGTGAAGATGCCGAGTTTGCCGAGTTGGGGTTCGTGGGGTG
TP7523_Hit	D	1	chr2	.	CAGCAATCTGCTCCTGTCCATGAGGTGAAGATGCCGAGTTTGCCGAGTTGGGGTTCGTGGGTGC
TP7523_Query	D	1	chr2	.	CAGCAATCTGCTCCGGTCCATGAGGTGAAGATGCCGAGTTTGCCGAGTTGGGGTTCGTGGGTGC
TP75690_Hit	D	1	chr2	.	CTGCCATTTAACTTAATTGTATCCATCCATTAGAGTAGAAGTGTTTAGAGATTGTTCTCATTTG
TP75690_Query	D	1	chr2	.	CTGCCATTTAACTTAATTGTATCCATCCATTAGAGTAGAAGTGTTTAGAGACTGTTCTCATTTG
TP76053_Hit	D	1	chr2	.	CTGCCCATGAATTCCATGACCTGAAATCACGGCCGTCCAAGATTTCACATCTTTATTTTCCATC
TP76053_Query	D	1	chr2	.	CTGCCCATGAATTCCATGACCTGAAATCACGACCGTCCAAGATTTCACATCTTTATTTTCCATC
TP7644_Hit	D+G	1	chr2	.	CAGCAATGACATTTGGTTGCTCATTTCCTTTCTCTGGGAAAGTGATTTTTGTTCCCTTTTTCCA
TP7644_Query	D+G	1	chr2	.	CAGCAATGACATTTGGTTGCTCATTTCCTTTCTCTGGAAAAGTGATTTTTGTTCCCTTTTTCCA
TP76533_Hit	D	1	chr2	.	CTGCCCGTGAGGATAAACAGTGGGAGGATAACTTAGAAACTTAGGATTTGGATTTGTTGTTGTC
TP76533_Query	D	1	chr2	.	CTGCCCGTGAGGATAAACAGTGGGAGGATAACTCAGAAACTTAGGATTTGGATTTGTTGTTGTC
TP76609_Hit	D	1	chr2	.	CTGCCCTGCCTGTGTTGCTGAAACATGTTGCTTGCCCTTCCTTCTCCTGATATTTGAAGATGGT
TP76609_Query	D	1	chr2	.	CTGCCCTACCTGTGTTGCTGAAACATGTTGCTTGCCCTTCCTTCTCCTGATATTTGAAGATGGT
TP76837_Hit	D	1	chr2	.	CTGCCTTGTTCCTTATTCTTTGAGTGTTCCTCTGTCTTGCTCTGCCATCGTTCTTCATCTCTAT
TP76837_Query	D	1	chr2	.	CTGCCCTGTTCCTTATTCTTTGAGTGTTCCTCTGTCTTGCTCTGCCATCGTTCTTCATCTCTAT
TP77439_Hit	D	1	chr2	.	CTGCCGCGGCCTTTATGGTTGCCCAGATCACAGCCCGTCTTGGTCACGACAGCTGAAAAAAAAA
TP77439_Query	D	1	chr2	.	CTGCCGCGGCCTTTATGGTTGCCCAGATCACAGCCCATCTTGGTCACGACAGCTGAAAAAAAAA
TP77467_Hit	D+G	1	chr2	.	CTGCCGCTACCATTAAAATAACCAAGGTCAGATCCTTGCAAGCATCCCACATAAACATCTGTTA
TP77467_Query	D+G	1	chr2	.	CTGCCGCTACCATTAAAATAACCAAGGTCAGATCCTTGCAAGCATCCCACAAAAACATCTGTTA
TP77609_Hit	D	1	chr2	.	CTGCCGGAGTTGGTCGCAGTAGGAAGGAAGGGTTTCAACTGATGTGTAGCTGAAAAAAAAAAAA
TP77609_Query	D	1	chr2	.	CTGCCGGAGTCGGTCGCAGTAGGAAGGAAGGGTTTCAACTGATGTGTAGCTGAAAAAAAAAAAA

Name	Filter	Nb hit (Mt4.0)	Mt Chr (Mt4.0)	Ms Chr (Li et al., 2014)	Sequence
TP77664_Hit	D	1	chr2	.	CTGCCGGCGGGGTGAAGCTTTTTGGTGTTAGATTAACCGATGGATCAATCATCAAGAAAAGTGC
TP77664_Query	D	1	chr2	.	CTGCCGGCGGGGGGAAGCTTTTTGGTGTTAGATTAACCGATGGATCAATCATCAAGAAAAGTGC
TP77887_Hit	D	1	chr2	.	CTGCCGTCATATCTGAAGTGCATCTTGTTAGTGATGTGAAAAAATGGGTGATAGACTCGGGGGC
TP77887_Query	D	1	chr2	.	CTGCCGTCATATCTGAAGTGCATCTTGTCAGTGATGTGAAAAAATGGGTGATAGACTCGGGGGC
TP78486_Hit	D	1	chr2	.	CTGCCTCCATGACCCATGATAATGGATTCCACATAAAACCCAAAAACTTGAGGAGTTTGCTCTC
TP78486_Query	D	1	chr2	.	CTGCCTCCATCACCCATGATAATGGATTCCACATAAAACCCAAAAACTTGAGGAGTTTGCTCTC
TP78487_Hit	D+G	1	chr2	.	CTGCTTCCATCATTTTCATCTCCCTCTTCACCCTCTCCAACTCAAGTTTGGAATCCGAGTCATG
TP78487_Query	D+G	1	chr2	.	CTGCCTCCATCATTTTCATCTCCCTCTTCACCCTCTCCAACTCAAGTTTGGAATCCGAGTCATG
TP79045_Hit	D+G	1	chr2	.	CTGCCTGTGAACTCAAGGAAGTTTTTACCTGTTTTGATGCCTATGATTCCTATTACACGATGTC
TP79045_Query	D+G	1	chr2	.	CTGCCTGTGAACTCAAGGAAGTTTTTACCTGGTTTGATGCCTATGATTCCTATTACACGATGTC
TP79240_Hit	D+G	1	chr2	.	CTGCCTTCCGTTTCGTGCCACACAGTGAAGTTGATTTATCATCCGCAATTTGGGAATGATGGAA
TP79240_Query	D+G	1	chr2	.	CTGCCTTCCGTTTCGTGCCACACAGTGAAGTTGATTTATCATCAGCAATTTGGGAATGATGGAA
TP7950_Hit	D	1	chr2	.	CAGCAATGGTTCATCCATCACATGCAAACCACCAACAAGAATCCAATACACAGTTTTATACATA
TP7950_Query	D	1	chr2	.	CAGCAATGGTTCATCCATCACATGCAAACCACCAACAAGAATCCAATACACAGTTCTATACATA
TP79599_Hit	D	1	chr2	.	CTGCCTTTTTACTTCCAGCTATCTTGGAAAGCTTACTTTGGTATTCCTTGTAATATTTTAACTG
TP79599_Query	D	1	chr2	.	CTGCCTTTTTACTTCCAGCTACCTTGGAAAGCTTACTTTGGTATTCCTTGTAATATTTTAACTG
TP79784_Hit	D	1	chr2	.	CTGCGAAGACGTAACCTCAGGACCCTGAAAAACATGCAAGAAAGTCTCAAGTAAATATGTCAAT
TP79784_Query	D	1	chr2	.	CTGCGAAGACGTAACCTCAGGACCCTGAAAAACATGCAAGAAAGTCTCAAGCAAATATGTCAAT
TP79898_Hit	D+G	1	chr2	.	CTGCGAATTATGTTTTGTTGGCTAAGATTTATGCTAGTGTTGGGAGACGAGAGGAGGCTGATAG
TP79898_Query	D+G	1	chr2	.	CTGCGAATTATGTTTTGTTGGCTAAGATGTATGCTAGTGTTGGGAGACGAGAGGAGGCTGATAG
TP79977_Hit	D+G	1	chr2	.	CTGCGACACGAATTCAGCTTGTGTCGCGTTTATGTGATATCGGGAAGCTTTAGTTCATTTGATC
TP79977_Query	D+G	1	chr2	.	CTGCGACACGAAATCAGCTTGTGTCGCGTTTATGTGATATCGGGAAGCTTTAGTTCATTTGATC
TP80047_Hit	D	1	chr2	.	CTGCGACCAGATGTCACAGACATTCAATGGCTGAAGGACAACAACAAGAAAGTTGGTTGTGATG
TP80047_Query	D	1	chr2	.	CTGCGACCAGATGTCACAGACATTCAATGGCTGAAGGACAACAACAAGAAAGTTGGTTGTGACG
TP80059_Hit	D	1	chr2	.	CTGCGACCGATGATAGTGGTATAGACAGTGATTTACAGGCAAGATTAGACAACTTAAGAAAAAT
TP80059_Query	D	1	chr2	.	CTGCGACCGATGATAGCGGTATAGACAGTGATTTACAGGCAAGATTAGACAACTTAAGAAAAAT
TP80093_Hit	D	1	chr2	.	CTGCGACGATCACACCTACCGTCCTGCTTATGACTGGAGTTGGTTTCTTTTCTCTCATATTATT
TP80093_Query	D	1	chr2	.	CTGCGACGATCACACCTACCGTCCTGCTCATGACTGGAGTTGGTTTCTTTTCTCTCATATTATT
TP80194_Hit	D+G	1	chr2	.	CTGCGACTTCTGTGAGAATACTTCATGGGTTTTCATCAAATCTTGCTCATTAATTTGATCATCG
TP80194_Query	D+G	1	chr2	.	CTGCGACTTCTGTGAGAATACTTCATGGGTTTTCATCAAAACTTGCTCATTAATTTGATCATCG
TP80205_Hit	D+G	1	chr2	.	CTGCGAGAAACAATGCTGTCTTGGTACAAGTTGTTGGTGATATATGAATGATTGATTCCGATGG
TP80205_Query	D+G	1	chr2	.	CTGCGAGAAACAATGCTGTCTTGGTACAAGTTGTTAGTGATATATGAATGATTGATTCCGATGG
TP80307_Hit	D+G	1	chr2	.	CTGCGAGCGCAACAAGTGATTGAGAAGATGACGAAGGATTTCCATTCCCCATCCGTGCTGAAAA
TP80307_Query	D+G	1	chr2	.	CTGCGAGCGCAACAAGTGATTGAGAAGATGACGAAGGATTTCCATTCCCCATCCATGCTGAAAA
TP804_Hit	D	1	chr2	.	CAGCAAAATAAACAGACAAAAGTTAGATGGAAGGCATATAATAAAACTATCAAGACGTTACCAA
TP804_Query	D	1	chr2	.	CAGCAAAATAAACAGACAAAAGTTAGATGGAAGGCATATAATAAAACTATCAAGACATTACCAA
TP80604_Hit	D	1	chr2	.	CTGCGATTGAAGGTGAAACGGAACTCTTCTTATTCTTGAAATTTTAATGGAACTTGAAACAAGA
TP80604_Query	D	1	chr2	.	CTGCGATGGAAGGTGAAACGGAACTCTTCTTATTCTTGAAATTTTAATGGAACTTGAAACAAGA
TP80723_Hit	D	1	chr2	.	CTGCGATTTACCAGCCTGTAGTTCCTGCATGAAAAGTAAATGTGAATTTTTATGTTAGTCACTA
TP80723_Query	D	1	chr2	.	CTGCGATTTACCAGCCTGTAGTTCCTGCAAGAAAAGTAAATGTGAATTTTTATGTTAGTCACTA
TP80739_Hit	D+G	1	chr2	.	CTGCGATTTCCTCCTTGCTAATTACCTTTGCCTTAGGAATTTTGTCTGAATTTGATCCTTGACT
TP80739_Query	D+G	1	chr2	.	CTGCGATTTCCTCCTTACTAATTACCTTTGCCTTAGGAATTTTGTCTGAATTTGATCCTTGACT
TP8075_Hit	D	1	chr2	.	CAGCAATGTTCATGACTAGAAACGCAATCTTCCGCTTCTCAAAGGCATTCCCTAACGTACCTTC
TP8075_Query	D	1	chr2	.	CAGCAATGTTCATCACTAGAAACGCAATCTTCCGCTTCTCAAAGGCATTCCCTAACGTACCTTC
TP81027_Hit	D	1	chr2	.	CTGCGCATTTCACGGATTATGAGCATGAGTACAGAAACATCTTAATTTATCATCGCCACAAAGA
TP81027_Query	D	1	chr2	.	CTGCGCATTTCACGGATTATGAGCATGAGTACAGAAACATCTTAATTTATCATCACCACAAAGA
TP81037_Hit	D+G	1	chr2	.	CTGCGCCAAAAATGGGATTGATGGTGCTTGACATGGCCATTCAAGTGCATGGTGCTGAAAAAAA
TP81037_Query	D+G	1	chr2	.	CTGCGCCAAAAATGGGATTGATGGTACTTGACATGGCCATTCAAGTGCATGGTGCTGAAAAAAA
TP8144_Hit	D	1	chr2	.	CAGCAATTAAGAACAGCTCTAATATTACATATGCTTCTGCGAATGATACTTCAGTGGCTCCTCT
TP8144_Query	D	1	chr2	.	CAGCAATTAAGAACAGCTCCAATATTACATATGCTTCTGCGAATGATACTTCAGTGGCTCCTCT
TP81506_Hit	D	1	chr2	.	CTGCGCGTCGGAAGCCATGGCCTTGATCCTTCACGCTCCCAACAACGGAAACAAAAATGTTTAC
TP81506_Query	D	1	chr2	.	CTGCGCGTCGGAAGCAATGGCCTTGATCCTTCACGCTCCCAACAACGGAAACAAAAATGTTTAC
TP81586_Hit	D+G	1	chr2	.	CTGCGCTATAGAGCTATATCACAGTGGAAATTGAATAAATCCCTATTTTTGTGTTATATGGTAG
TP81586_Query	D+G	1	chr2	.	CTGCGCTATAGAGCTATATCACAGTGGAAATTGAATAAATCCCTATTTTCGTGTTATATGGTAG

Name	Filter	Nb hit (Mt4.0)	Mt Chr (Mt4.0)	Ms Chr (Li et al., 2014)	Sequence
TP81697_Hit	D	1	chr2	.	CTGCGCTGAAAGGTCCTCCTGCACCAATGTGTAAGTTGCAATAGGTATTGCGAAAGATTAAAAA
TP81697_Query	D	1	chr2	.	CTGCGCTGAAAGGTCCTCCTGCACCAATGTGTAAGTTACAATAGGTATTGCGAAAGATTAAAAA
TP82158_Hit	D	1	chr2	.	CTGCGGCCCAGCTTCCTCAGAATCAGTATTTTCCTTCTAATCCACAGTACCAATATCATCCATC
TP82158_Query	D	1	chr2	.	CTGCGGCCCAGCTTCCTCAGAATCAGTATTTTCCTGCTAATCCACAGTACCAATATCATCCATC
TP82299_Hit	D	1	chr2	.	CTGCGGCGTATTTTCCGGCTACGGTGGGATCTTCACCTGGTGTCTCTTGGCCACGGCCCCACCG
TP82299_Query	D	1	chr2	.	CTGCGGCGTATTTTCCGGCTACGGTGGGATCTTCACCGGGTGTCTCTTGGCCACGGCCCCACCG
TP82334_Hit	D	1	chr2	.	CTGCGGCTATAACTGCGACCACGGCTACGACTGCGTGGGCTACGACTACGGCTATAGCTGAAAA
TP82334_Query	D	1	chr2	.	CTGCGGCTATAACTGCGACCACGGCTACGACTGCGTGGACTACGACTACGGCTATAGCTGAAAA
TP82416_Hit	D	1	chr2	.	CTGCGGCTTGGAATGCCGAGGTTCGTAGAGCCAAGGGGAGATTGATGGACGAAGTTCCCAAGCT
TP82416_Query	D	1	chr2	.	CTGCGGCTTGGAATGCCGAAGTTCGTAGAGCCAAGGGGAGATTGATGGACGAAGTTCCCAAGCT
TP82686_Hit	D+G	1	chr2	.	CTGCGGTATAACCGGCAAGGAGAGTGATTGTGATGCTTCTGGAAGAACAGCATAATTTGGATTT
TP82686_Query	D+G	1	chr2	.	CTGCGGTATAACCGGCAAGGAGAGTGATTGTGATGCTTCTGGAAGAACAACATAATTTGGATTT
TP82687_Hit	D	1	chr2	.	CTGCGGTATAACCGGCAAGGAGAGTGCTTGTGATGCTTCTGGAAGAACATCATAATTTGGATTT
TP82687_Query	D	1	chr2	.	CTGCGGTATAACCGGCAAGGAGAGTGCTTGAGATGCTTCTGGAAGAACATCATAATTTGGATTT
TP83114_Hit	D+G	1	chr2	.	CTGCGTAGGAGGTTTGAGACACTTCTCCTGCAAAAGGAAAAGATATATTTATTCTCTCTTTCTT
TP83114_Query	D+G	1	chr2	.	CTGCGTAGGAGGTTTGAGACACTTCTCCTGCAAAAGGAAAAGATATATTTATTCTCTATTTCTT
TP83115_Hit	D+G	1	chr2	.	CTGCGTGGGATCTGTGCTAAACTAGAATTCACCTGATAACAGGATTCAAATGCCAGAAGAGCTG
TP83115_Query	D+G	1	chr2	.	CTGCGTAGGATCTGTGCTAAACTAGAATTCACCTGATAACAGGATTCAAATGCCAGAAGAGCTG
TP83129_Hit	D+G	1	chr2	.	CTGCGTAGTGAGCTCGCCCGAATGTTCGGACTGGAAGGTGAGTTGGAGGACCCTGTGAGATCAG
TP83129_Query	D+G	1	chr2	.	CTGCGTAGTGAGCTCGCCCGAATGTTCGGACTGGAAGGCGAGTTGGAGGACCCTGTGAGATCAG
TP83247_Hit	D	1	chr2	.	CTGCGTCATAACTGGACTTTCCAAGGTTCCCACTTCAGCAATCTCTTTCTCTTCAGAATTGCTA
TP83247_Query	D	1	chr2	.	CTGCGTCATAACTGCACTTTCCAAGGTTCCCACTTCAGCAATCTCTTTCTCTTCAGAATTGCTA
TP83273_Hit	D	1	chr2	.	CTGCGTCCAAGGACGGGCCTTGTGGAGGTCGCGGAGGTTTGGCTGAAAAAAAAAAAAAAAAAAA
TP83273_Query	D	1	chr2	.	CTGCGTCCAAGGACGGGCCTTGTGGAGGTCGCGGAGGTTTGGCAGAAAAAAAAAAAAAAAAAAA
TP8349_Hit	D+G	1	chr2	.	CAGCAATTCTTGGTACTGCCATTGTCTTTTTCTTCACTGTATATCGATCGAAGAATAGTAATAG
TP8349_Query	D+G	1	chr2	.	CAGCAATTCTTGGTACTGCCATTGTCTTTTTCTTCACTGTATATCGATCAAAGAATAGTAATAG
TP8418_Hit	D	1	chr2	.	CAGCAATTGCATTAAGAGATACTTCTTCCCTTACAAATAAATGCAGTACAGATAAATTTGTCAT
TP8418_Query	D	1	chr2	.	CAGCAATTGCATTAAGAGATACTTCTTCCCTTACAAATAAATGCAGTACAAATAAATTTGTCAT
TP84227_Hit	D+G	1	chr2	.	CTGCTAAATCCATATTGTGACATTGAACCTGGATTGCTTAACACTTAACTGCCCAAAAGCTAAA
TP84227_Query	D+G	1	chr2	.	CTGCTAAATCCATATTATGACATTGAACCTGGATTGCTTAACACTTAACTGCCCAAAAGCTAAA
TP84274_Hit	D	1	chr2	.	CTGCTAGCAACAAGTGCAGGTCGGTTTGGCTTCAAACTTACGTCTGGACCAGCCTCAGTTGTAA
TP84274_Query	D	1	chr2	.	CTGCTAACAACAAGTGCAGGTCGGTTTGGCTTCAAACTTACGTCTGGACCAGCCTCAGTTGTAA
TP84318_Hit	D	1	chr2	.	CTGCTAACCATAAGAACATTATTTTTGTTGCTTTCACTACTCTTATCCTGTAATCCTCAGTCAA
TP84318_Query	D	1	chr2	.	CTGCTAACCATAAGAACATAATTTTTGTTGCTTTCACTACTCTTATCCTGTAATCCTCAGTCAA
TP84384_Hit	D+G	1	chr2	.	CTGCTAACTTTGTGTTAATGAAAAATAACCTGGAAGATGTGATCACAGCCATTCATCTTTCTCG
TP84384_Query	D+G	1	chr2	.	CTGCTAACTTTGTGTTAATGAAAAATAACCTGGAAGATGTGATCACAGCCATTCATCTTTCCCG
TP85033_Hit	D	1	chr2	.	CTGCTACCGGCACTGGGGACTTTACTGGTACTGGAACTGGAGCAGAAAAAAAAAAAAAAAAAAA
TP85033_Query	D	1	chr2	.	CTGCTACCGGCACTGGGGACTTTACTGGTACTGGAACAGGAGCAGAAAAAAAAAAAAAAAAAAA
TP85034_Hit	D+G	1	chr2	.	CTGCTACCGGCACTGGGGACTTTACTGGTACTGGAACTGGAGTAGCTATTGGCACCGGTGAAAC
TP85034_Query	D+G	1	chr2	.	CTGCTACCGGCACTGGGGACTTTACTGGTACTGGAACAGGAGTAGCTATTGGCACCGGTGAAAC
TP85265_Hit	D	1	chr2	.	CTGCTACTGCACTTCAGGACATGCGGACTGTAGTAGATCCTTCCAAACAGCTTCTGGGTTCCTT
TP85265_Query	D	1	chr2	.	CTGCTACTGCACTTCAGGACATGAGGACTGTAGTAGATCCTTCCAAACAGCTTCTGGGTTCCTT
TP85268_Hit	D	1	chr2	.	CTGCTACTGCCCTTTACTCTGCATCCTGCTTTACTTCCGGGAAGTATGGGAATGGCAATGCTTA
TP85268_Query	D	1	chr2	.	CTGCTACTGCCCTTTACTCTGCATCCTGCTTTACTTCCGGAAAGTATGGGAATGGCAATGCTTA
TP85422_Hit	D	1	chr2	.	CTGCTATTTTTGTACAAATCAGGGGAACTTTTTAATGAGAGTGAGCTTGCACTTCATAGTTTAT
TP85422_Query	D	1	chr2	.	CTGCTACTTTTGTACAAATCAGGGGAACTTTTTAATGAGAGTGAGCTTGCACTTCATAGTTTAT
TP85967_Hit	D	1	chr2	.	CTGCTATAAATATATTTCTCAACACTTACGATTTCTGTGATTTCTCCTAACCTCCTCGTTTACT
TP85967_Query	D	1	chr2	.	CTGCTATAAATATATTTCTCAACACTCACGATTTCTGTGATTTCTCCTAACCTCCTCGTTTACT
TP86045_Hit	D+G	1	chr2	.	CTGCTATACGCTTGGATGAGAAGCCTGATCAAATTGAAAGAGTACTTGATTTGTCGCTTGTAAA
TP86045_Query	D+G	1	chr2	.	CTGCTATACGCTTGGATGAGAAGCCTGATCAAATTGAAAGAGTACTTGATTTGTCCCTTGTAAA
TP86047_Hit	D+G	1	chr2	.	CTGCTATACTCACTACTTGACAACACATTGTACCAAATAGCACATCGCGGAACAATAACCATTT
TP86047_Query	D+G	1	chr2	.	CTGCTATACTCACTACTTGACAACACACTGTACCAAATAGCACATCGCGGAACAATAACCATTT
TP8611_Hit	D	1	chr2	.	CAGCAATTTGGATAAGAAACATCACTGTCTGCATTTGATGTCATGCCACAGATTTTTTATAACA
TP8611_Query	D	1	chr2	.	CAGCAATTTGGATAAGAAACATCACTGTCTGCATTTGATATCATGCCACAGATTTTTTATAACA

Name	Filter	Nb hit (Mt4.0)	Mt Chr (Mt4.0)	Ms Chr (Li et al., 2014)	Sequence
TP86162_Hit	D+G	1	chr2	.	CTGCTATATGGAAATCTTGCATTCCCCCTTCTCATTCATTTATTATTTGGCGCATAATGCATGG
TP86162_Query	D+G	1	chr2	.	CTGCTATATGGAAATCTTGCATTCCCCCTTCTTATTCATTTATTATTTGGCGCATAATGCATGG
TP86417_Hit	D	1	chr2	.	CTGCTATGATGCTTTACCAAATAAATAGAAGAGAATGGGAGAAAAGCATAAAAGGTAACTTTCG
TP86417_Query	D	1	chr2	.	CTGCTATGATGCTTTACCAAAAAAATAGAAGAGAATGGGAGAAAAGCATAAAAGGTAACTTTCG
TP86472_Hit	D+G	1	chr2	.	CTGCTATGGCAAAAAGATAATGATTGAATCATGGTTGTCAATTTGTCATTGTCCACTGAGGTCA
TP86472_Query	D+G	1	chr2	.	CTGCTATGGCAAAAAGATAACGATTGAATCATGGTTGTCAATTTGTCATTGTCCACTGAGGTCA
TP86560_Hit	D+G	1	chr2	.	CTGCTATGTGTGTTGAAGAGCAGTCTAGTATGCGGCCGGTTATAGCAGATGTGGGTGCTGAAAA
TP86560_Query	D+G	1	chr2	.	CTGCTATGTGTGTTAAAGAGCAGTCTAGTATGCGGCCGGTTATAGCAGATGTGGGTGCTGAAAA
TP86574_Hit	D	1	chr2	.	CTGCTATGTTGATAAACTATTTGTCAAAACCAAAACATATGAGAAATCTTTGCTAAATGCAGAG
TP86574_Query	D	1	chr2	.	CTGCTATGTTGATAAACTATTTGTCAAAACCAAAACATATGAGAAATCTTTGCTAAATGCAAAG
TP86783_Hit	D	1	chr2	.	CTGCTATTGGGAAACCTATGTCCATCATTTTCTTTAGCTCGTCCACCACCTGAAAAAACAAAAC
TP86783_Query	D	1	chr2	.	CTGCTATTGGGAAACCTATGTCCATCATTCTCTTTAGCTCGTCCACCACCTGAAAAAACAAAAC
TP86854_Hit	D	1	chr2	.	CTGCTATTTTATAAATTTGAGGCTAAATTGACTAATCCGCATAATTTCAAGGACTAAAATGCCT
TP86854_Query	D	1	chr2	.	CTGCTATTTCATAAATTTGAGGCTAAATTGACTAATCCGCATAATTTCAAGGACTAAAATGCCT
TP86910_Hit	D	1	chr2	.	CTGCTATTTGGAAATCTTGCATTCCCCCTTCTCATTCTTTTATTCTTTGGCGGATAATGCATGG
TP86910_Query	D	1	chr2	.	CTGCTATTTGGAAATCTTGCATTCCCCCTTCTCATTCTTTTATTCTTTAGCGGATAATGCATGG
TP86911_Hit	D	1	chr2	.	CTGCTATTTGGAAATCTTGCTTTCCCCCTTCTCATTCTTTTATTCTTTGGCGCATAATGCATAG
TP86911_Query	D	1	chr2	.	CTGCTATTTGGAAATCTTGCATTCCCCCTTCTCATTCTTTTATTCTTTGGCGCATAATGCATAG
TP870_Hit	D	1	chr2	.	CAGCAAAATCAATCTTCCGGATTACACACTGAGGATCCGAATCTTCATATTGCTACCTTTTTAA
TP870_Query	D	1	chr2	.	CAGCAAAATCAATCTTCCGGATTACACACTGAGGATCCGAATCTTCATATTGATACCTTTTTAA
TP87012_Hit	D	1	chr2	.	CTGCTCAAACGCGAAACAACTTGAAGACCGAGTTCGCGGTAGTGATCTTCTGATTCAAGATTAA
TP87012_Query	D	1	chr2	.	CTGCTCAAACGCGAAACAACTTGAAGACCGAGTTCGCGGTAATGATCTTCTGATTCAAGATTAA
TP8747_Hit	D	1	chr2	.	CAGCATAAAATAGCATCTGGATGATGCCACAAATGTTTCAGTAGCATATCACCAATAAGAGAAT
TP8747_Query	D	1	chr2	.	CAGCACAAAATAGCATCTGGATGATGCCACAAATGTTTCAGTAGCATATCACCAATAAGAGAAT
TP87933_Hit	D	1	chr2	.	CTGCTCCGAGTACGATTCAAGGGCTCGAGTTTGCTGGACTTGATTTGCCAGATTCAATAGGTGA
TP87933_Query	D	1	chr2	.	CTGCTCCGAGTACAATTCAAGGGCTCGAGTTTGCTGGACTTGATTTGCCAGATTCAATAGGTGA
TP8815_Hit	D+G	1	chr2	.	CAGCACAAAGCCGCTCTACAAAAGAAGCCATTCCTGTGACGTTTGATTCTCATCTCGGTGCTGA
TP8815_Query	D+G	1	chr2	.	CAGCACAAAGCCGCTCTACAAAAGAAGACATTCCTGTGACGTTTGATTCTCATCTCGGTGCTGA
TP88288_Hit	D	1	chr2	.	CTGCTCGAAATAGTAATTATGATTCACAAAAGATTCTTGAAGAGTTAGAAGCTCCTATAATCTT
TP88288_Query	D	1	chr2	.	CTGCTCGAAATAGTAATTATGATTCACAAAAGGTTCTTGAAGAGTTAGAAGCTCCTATAATCTT
TP88344_Hit	D+G	1	chr2	.	CTGCTCGAGCAGTTGCTGAAGCGGAAGCCCTCATGGCGGAGGCTGAGGAGGCAACAAAGGAGGC
TP88344_Query	D+G	1	chr2	.	CTGCTCGAGCAGTTGCTGAAGCGGAAGCCCTCATGGCGGAGGCTGAAGAGGCAACAAAGGAGGC
TP88357_Hit	D+G	1	chr2	.	CTGCTCGAGTAGCCCCATCGTTTGTAGCTATAGATCCTATCACAGGCAAGACTGTTGAAACAGA
TP88357_Query	D+G	1	chr2	.	CTGCTCGAGTAGCCCCATCGTTTGTAGCAATAGATCCTATCACAGGCAAGACTGTTGAAACAGA
TP88471_Hit	D	1	chr2	.	CTGCTCGCGACTTCTGGACTCTAAGATACACTACAAATTTGGAAAACGGCAGTGTTGTGGTGAG
TP88471_Query	D	1	chr2	.	CTGCTCGCGACTTCTGGACTCTAAGATACACTACAAATCTGGAAAACGGCAGTGTTGTGGTGAG
TP88955_Hit	D	1	chr2	.	CTGCTCTCTGCGATATTTTCTCCGTAACGCCCACCACCACCGGAGTGGATCGATTGACAATCAC
TP88955_Query	D	1	chr2	.	CTGCTCTCTGCGATATTTTCTCCGTAACGCCCACCACCACCGGAGTGGATCAATTGACAATCAC
TP89107_Hit	D	1	chr2	.	CTGCTCTGTTGTTTGCCGCTCCTGCAATTGCACTTGCTTATTGGCGCAAAAGAAAACCACAGGA
TP89107_Query	D	1	chr2	.	CTGCTCTGTTGTTTGCCGCTCCTGCAATTGCACTTGCTTATTGGCGAAAAAGAAAACCACAGGA
TP89364_Hit	D	1	chr2	.	CTGCTCTTTGAAGGTATGTCCATGATCTCATTTTAGTGTACAATTGAATCTGGAAGTGTATAAA
TP89364_Query	D	1	chr2	.	CTGCTCTTTGAAGGTATGTCCATGATCTCATTTTAGGGTACAATTGAATCTGGAAGTGTATAAA
TP89753_Hit	D	1	chr2	.	CTGCTGACAAGAATTCAGATGGGGCAATGCAAAATTCTTCGGAACAAGTTCAAGTGGCATCTAG
TP89753_Query	D	1	chr2	.	CTGCTGACAAGAATTCAGATGGGGCAACGCAAAATTCTTCGGAACAAGTTCAAGTGGCATCTAG
TP89897_Hit	D	1	chr2	.	CTGCTGAGCCGTTGTACGGGGCTCTGCAAGACTTATATTCCAGTTTAGGCTGTTAGTTCTACTT
TP89897_Query	D	1	chr2	.	CTGCTGAGCCGTTGTACGGGGCTCTGCAAGACTTATATTCCAGTTTAAGCTGTTAGTTCTACTT
TP90227_Hit	D+G	1	chr2	.	CTGCTGATGGACAAACCAGTGTAGAAATTAATGTCCTTCAGGGTGAGAGAGAATTTGTTAGGGA
TP90227_Query	D+G	1	chr2	.	CTGCTGATGGACAAACAAGTGTAGAAATTAATGTCCTTCAGGGTGAGAGAGAATTTGTTAGGGA
TP9035_Hit	D	1	chr2	.	CAGCACAAGCTTCATACCAGGTGAAAAGATTGATCCGTGGCAATGCTGTTTACTTGAAGACATG
TP9035_Query	D	1	chr2	.	CAGCACAAGCTTCATACCAGGTGAAAAAATTGATCCGTGGCAATGCTGTTTACTTGAAGACATG
TP90422_Hit	D+G	1	chr2	.	CTGCTGGACCAAACTATATCTTATCTGTAACAGAAGGTGGATCAGTATACTCTTTTGGATCAGG
TP90422_Query	D+G	1	chr2	.	CTGCTGGACCAAACTATATCTTATCTGTAACAGAAGGTGGATCAGTATACTCCTTTGGATCAGG
TP90423_Hit	D+G	1	chr2	.	CTGCTGGACCAAAGGTTGAGGAGAAAAAGGCGGAGAAAACAGCTTTTGACGTGAAGTTAGAGAA
TP90423_Query	D+G	1	chr2	.	CTGCTGGACCAAAGGTTGAGGAGAAAAAGGCAGAGAAAACAGCTTTTGACGTGAAGTTAGAGAA

Name	Filter	Nb hit (Mt4.0)	Mt Chr (Mt4.0)	Ms Chr (Li et al., 2014)	Sequence
TP90424_Hit	D	1	chr2	.	CTGCTGGACCAAAGGTTGAGGAGAAAAAGGCGGAGAAAGCAGAAAAAAAAAAAAAAAAAAAAAA
TP90424_Query	D	1	chr2	.	CTGCTGGACCAAAGGTTGAGGAGAAAAAGGCAGAGAAAGCAGAAAAAAAAAAAAAAAAAAAAAA
TP90621_Hit	D+G	1	chr2	.	CTGCTGGCCCACCCTCGCTCAATGCTTGGGTCCTTGATGAAGGAAGTTTGAGCAACAATAGAAG
TP90621_Query	D+G	1	chr2	.	CTGCTGGCCCACCCTCGCTCAATGCTTGGGTCCTCGATGAAGGAAGTTTGAGCAACAATAGAAG
TP91127_Hit	D	1	chr2	.	CTGCTGGTGCTGTTGTTGCCTTTGCTTTTGTTGGTTTGTGTGTTTGCTTGTTGGCGAGGAGAAA
TP91127_Query	D	1	chr2	.	CTGCTGGTGCTGTTGTTGCCTTTGCTTTTATTGGTTTGTGTGTTTGCTTGTTGGCGAGGAGAAA
TP91346_Hit	D	1	chr2	.	CTGCTGTAAACAGCACTGTCGGTGCCAAGGGATCAACAGGAAATCAAAAGTTATATGGTACTGT
TP91346_Query	D	1	chr2	.	CTGCTGTAAACAGCACTGTAGGTGCCAAGGGATCAACAGGAAATCAAAAGTTATATGGTACTGT
TP91535_Hit	D	1	chr2	.	CTGCTGTATCTGGAAACCTACAAATTGTTATTATATTGACCAAGAAAAATGGATGTCTACCACA
TP91535_Query	D	1	chr2	.	CTGCTGTATCTGGAAACCTACAAATAGTTATTATATTGACCAAGAAAAATGGATGTCTACCACA
TP9164_Hit	D	1	chr2	.	CAGCGCAATGCTTTCAAAGAGGCCATATTGCATTTCCAATTCTAGTGGTGAATACTTAACATGT
TP9164_Query	D	1	chr2	.	CAGCACAATGCTTTCAAAGAGGCCATATTGCATTTCCAATTCTAGTGGTGAATACTTAACATGT
TP91682_Hit	D	1	chr2	.	CTGCTGTCCCAGGTAGAACAGTGGAAACAATCTCAGTTGTTGTACATTTAGTTTTAATGCTTCT
TP91682_Query	D	1	chr2	.	CTGCTGTCCCAGGTAGAACAGTGGAAACAATCTCAGTTGTTGTACATCTAGTTTTAATGCTTCT
TP9180_Hit	D	1	chr2	.	CAGCACAATTCAAAATTGGACATCCTTGTAAGTAACAAACCTGCTACCTTACAAACCAGGAGCA
TP9180_Query	D	1	chr2	.	CAGCACAATTCAAAATTGGACATCCTTGTAAGTAACAAACCTGCTACCTTACAAACCAGAAGCA
TP92361_Hit	D	1	chr2	.	CTGCTGTTGCAACTTCTGTAACAACTGCAACTGTTGCTGTTGGTCTGAGATGTTTTGTGGCGAA
TP92361_Query	D	1	chr2	.	CTGCTGTTGCAACTTCTGTAACAACTGCAACTGTTGCTGTTGGTCTGAGATGTTTTGCGGCGAA
TP92419_Hit	D	1	chr2	.	CTGCTGTTGGACTCAGGAGCATCATGCTGGGGTGTAAGCGGGGTAGATGTTGGAAGAGATGGTA
TP92419_Query	D	1	chr2	.	CTGCTGTTGGACTCAGGAGCATCATGCTGGGGTGTAAGCGGAGTAGATGTTGGAAGAGATGGTA
TP92435_Hit	D+G	1	chr2	.	CTGCTGTTGGTACCACAATGTTCACCTGCATTATCTGGGCTGTCAACTGCCAGATTGCCTTGAC
TP92435_Query	D+G	1	chr2	.	CTGCTGTTGGTACCACAATGTTCACCTGCATCATCTGGGCTGTCAACTGCCAGATTGCCTTGAC
TP92604_Hit	D	1	chr2	.	CTGCTGTTTGGTTGATGTCCTGCAATCCAGGTGGAGGAGAATAAAGTAGAAAGCATCAAGAGGG
TP92604_Query	D	1	chr2	.	CTGCTGTTTGGTTGATGTCATGCAATCCAGGTGGAGGAGAATAAAGTAGAAAGCATCAAGAGGG
TP92877_Hit	D	1	chr2	.	CTGCTTACTTAAGTGCAAATTTGGAATGGGTTGAACAGCAGGAGATCCTTAGAGAACTTAATTC
TP92877_Query	D	1	chr2	.	CTGCTTACTTAAGTGCAAATTTGGAATGGGCTGAACAGCAGGAGATCCTTAGAGAACTTAATTC
TP92879_Hit	D+G	1	chr2	.	CTGCTTACTTCTCATGGCCTAGTCGTTTGCCTGATGCCGCCGAAGAGAGAGCGAACTACTTTTT
TP92879_Query	D+G	1	chr2	.	CTGCTTACTTCTCATGGCCTAGCCGTTTGCCTGATGCCGCCGAAGAGAGAGCGAACTACTTTTT
TP93424_Hit	D	1	chr2	.	CTGCTTCCGCGAATGGCTTGATATCAAGTTCGCCCATCCTTTTCACAGAAATTATGACTCGAGC
TP93424_Query	D	1	chr2	.	CTGCTTCCACGAATGGCTTGATATCAAGTTCGCCCATCCTTTTCACAGAAATTATGACTCGAGC
TP93892_Hit	D+G	1	chr2	.	CTGCTTCTGTCCTCCTGATAGCTGTGTGCCTCTTTCCCCGACAGAGGTGTCATAACCATTTGGA
TP93892_Query	D+G	1	chr2	.	CTGCTTCTGTCCTCCTGATAGCTGTGTGCCTCTTTCCCCGACAGAGGTGTCATAACCAATTGGA
TP94049_Hit	D	1	chr2	.	CTGCTTGAAGCTTGGCTAGACTTTGCGCCGAGAACTGGCCAGAAGCTGAAAGGGTTTGAAGATC
TP94049_Query	D	1	chr2	.	CTGCTTGAAGCTTGGCTAGACTTTGCACCGAGAACTGGCCAGAAGCTGAAAGGGTTTGAAGATC
TP94284_Hit	D	1	chr2	.	CTGCTTGCCTACCATCATCTACCACTATCAAATGTCCCTCTCTTTGAACTAAGATCTACAGGAA
TP94284_Query	D	1	chr2	.	CTGCTTGCCTACCATCATCTACAACTATCAAATGTCCCTCTCTTTGAACTAAGATCTACAGGAA
TP943_Hit	D	1	chr2	.	CAGCAAAATGAAACAAACGATGCGTGTATTCTTTTATTTGTTGCACTTGTAAATTTTGGCACGG
TP943_Query	D	1	chr2	.	CAGCAAAATGAAACAAACGATGCGTGCATTCTTTTATTTGTTGCACTTGTAAATTTTGGCACGG
TP9444_Hit	D+G	1	chr2	.	CAGCACAGAAGACTCTCCTTCTCATCAAACCACACTCCATTTGGTGGACCCAACAACAACAACT
TP9444_Query	D+G	1	chr2	.	CAGCACAGAAGACTCTCCTTCTCATCAAACCACACTCCATTTGGTGGACCCAACAACAACAACA
TP9445_Hit	D	1	chr2	.	CAGCACAGAAGACTCTCCTTCTCGTCAAACCACACTCCATTTGGTGGACCCAACAACAACTCAT
TP9445_Query	D	1	chr2	.	CAGCACAGAAGACTCTCCTTCTCATCAAACCACACTCCATTTGGTGGACCCAACAACAACTCAT
TP94533_Hit	D	1	chr2	.	CTGCTTGTATGACGTGGGAGTTAGCAGAATCGGGAAAGCACTTTATCACTACCATTATAAATGG
TP94533_Query	D	1	chr2	.	CTGCTTGTATGACATGGGAGTTAGCAGAATCGGGAAAGCACTTTATCACTACCATTATAAATGG
TP94824_Hit	D	1	chr2	.	CTGCTTTAGGGATTTCACTTCCTTTATTAGCAACTTCATATCTGTCTTTGATTTCTTCTCAAAC
TP94824_Query	D	1	chr2	.	CTGCTTTAGGGATTTCACTTCCTTTATTAGCAACTTCATATCTGTCTTTGATTTCATCTCAAAC
TP95238_Hit	D+G	1	chr2	.	CTGCTTTGGAAACACAACTTGGTGAGAAATTCCTTTGTTTGAAGAAACTGGAAAAGTGAACCAG
TP95238_Query	D+G	1	chr2	.	CTGCTTTGGAAACACAACTTGGTGAGAAATTCCTTTGTTTGAAGAAACTGGAAAAATGAACCAG
TP95422_Hit	D	1	chr2	.	CTGCTTTTATGTTCTTGTCAACTTTGTTGTTCTTCCTTGTTTCATCGTGTTATGTCAAGAACAA
TP95422_Query	D	1	chr2	.	CTGCTTTTATGTTCTTGTCAACTTTGTTGTTCTTCCTTGTTTCATCGCGTTATGTCAAGAACAA
TP9548_Hit	D	1	chr2	.	CAGCACAGGATGTTGCTACTAGATGCAAGGTCGGTAGATTTCTCATTGATATATAATGCTTTCT
TP9548_Query	D	1	chr2	.	CAGCACAGGATGTTGCTACTAGATGCAAGGTCGGTAGATTTCTCATTAATATATAATGCTTTCT
TP9567_Hit	D	1	chr2	.	CAGCACAGGTTTTTTTTTAGAGATTGTGATAAGCCAAAAGCACAAACTGATTTGGCAAAACGTC
TP9567_Query	D	1	chr2	.	CAGCACAGGGTTTTTTTTAGAGATTGTGATAAGCCAAAAGCACAAACTGATTTGGCAAAACGTC

Name	Filter	Nb hit (Mt4.0)	Mt Chr (Mt4.0)	Ms Chr (Li et al., 2014)	Sequence
TP95678_Hit	D	1	chr2	.	CTGCTTTTTATCCCGGTAAATTCTTTGAGATTGCGAATGCAACCGATCGTGATACTTGGATTGT
TP95678_Query	D	1	chr2	.	CTGCTTTTTATCCCGGTAAATTCTTTGAGATTGCGAATGCAACCGATCGTGATACTTGGATCGT
TP9675_Hit	D	1	chr2	.	CAGCACATAGATTGAGGAAAAAGTAAGGAAGAGAGTTAAGGCATTGTTCACATCAGACTTTATC
TP9675_Query	D	1	chr2	.	CAGCACATAGATTGAGGAAAAAGTAAGGAAGAGAGCTAAGGCATTGTTCACATCAGACTTTATC
TP9806_Hit	D	1	chr2	.	CAGCACATGATGATTTTGAGAGAAATTGGGGCTCCTCTTTCTCCTAGTAGGATACTTTGGCACA
TP9806_Query	D	1	chr2	.	CAGCACATGATGATTTTGAGAGAAATTGGCGCTCCTCTTTCTCCTAGTAGGATACTTTGGCACA
TP9837_Hit	D	1	chr2	.	CAGCACATGGAACAGCAGAGGCTACCGGTGAAAGGTTTACAAGAGATCAATGTTATAATTTTGG
TP9837_Query	D	1	chr2	.	CAGCACATGGAACAGCAGAGGCTACCAGTGAAAGGTTTACAAGAGATCAATGTTATAATTTTGG
TP16158_Hit	D	1	chr2	2A	CAGCAGTGATTACTACTCAATTAGCATTGGAGGGAGAAGCTCGTAGTGGTGCTGTTGGTATCAT
TP16158_Query	D	1	chr2	2A	CAGCAGTGATTACTACACAATTAGCATTGGAGGGAGAAGCTCGTAGTGGTGCTGTTGGTATCAT
TP17707_Hit	D	1	chr2	2A	CAGCATAGTGAATAGGAGTATTTCCATTTCCACCCCTTTTTACAGGTAATTGTGAGTTCCTATC
TP17707_Query	D	1	chr2	2A	CAGCATAGTGAATAGGAGTATTTCCATTTCCACCCCTTTTTACAGGCAATTGTGAGTTCCTATC
TP18320_Hit	D+G	1	chr2	2A	CAGCATCAACTACGAAGGGAATGGCTTCGAGAATATCCCAAGCAGGCAACTTTGGTCCAATAGA
TP18320_Query	D+G	1	chr2	2A	CAGCATCAACTACAAAGGGAATGGCTTCGAGAATATCCCAAGCAGGCAACTTTGGTCCAATAGA
TP18710_Hit	D	1	chr2	2A	CAGCATCATAGGATGCCATATTGGTTCCCATGTTGGCAATAGCGTGCCAATTCCCGCTAATCGG
TP18710_Query	D	1	chr2	2A	CAGCATCATAGGATGCCATATTGGTTCCCATGTTGGCAATAGCGCGCCAATTCCCGCTAATCGG
TP19940_Hit	D+G	1	chr2	2A	CAGCATGATCCCATATTTTCTCCTTGTAACCTGCCCTTGCAAATTTCATGAATACCTCTGCATC
TP19940_Query	D+G	1	chr2	2A	CAGCATGATCCCATATTTTCTCCTTGTAACCTGCCCTTGCAAATTTCATGAATACCTCGGCATC
TP21322_Hit	D	1	chr2	2A	CAGCATTCAGCATACACTACAAAAGGTAATTTAGTACACAAGGCCTTAAATTCAACTAACCAAC
TP21322_Query	D	1	chr2	2A	CAGCATTCAGCATACACTACAAAAGGTAATTTGGTACACAAGGCCTTAAATTCAACTAACCAAC
TP25839_Hit	D	1	chr2	2A	CAGCCAGCCTTAAAAGTGTGCAGATAATGAAGGAATTTTGGGGTGATGTTGATGATGACGACTC
TP25839_Query	D	1	chr2	2A	CAGCCAGCCTTAAAAGTGTGCAGATAATGAAGGAATTTTGGGGTGATGTTGATGACGACGACTC
TP31177_Hit	D+G	1	chr2	2A	CAGCCGGTTAAAGGTCCGGCTTATGCGGAAGGAGGAGTTAGTGTGGTGCGTTCGAGTGGATTAC
TP31177_Query	D+G	1	chr2	2A	CAGCCGGTTAAAGGTCCGGCGTATGCGGAAGGAGGAGTTAGTGTGGTGCGTTCGAGTGGATTAC
TP34186_Hit	D+G	1	chr2	2A	CAGCCTTAGTTGAAATTGAGAAGAACAGCCCGCACCGTTTTGAATTCTTTATGGGGCTTTCTGG
TP34186_Query	D+G	1	chr2	2A	CAGCCTTAGTTGAAATTGAGAAGAACAGCCCACACCGTTTTGAATTCTTTATGGGGCTTTCTGG
TP34514_Hit	D	1	chr2	2A	CAGCCTTCTTTTTCTCATTCACGGCAACCTTCTTCTCAGAATCAGCCCTTTTCTTTTCTTTCTC
TP34514_Query	D	1	chr2	2A	CAGCCTTCTTTTTCTCATTCACGGCAACCTTCTTCTCAGAATCAGCCCTTCTCTTTTCTTTCTC
TP34634_Hit	D+G	1	chr2	2A	CAGCCTTGCTAGTATGTAACCAAAGAAGAATCCAGAAGCATGAAGAAGGCAAGATGCTAAAATC
TP34634_Query	D+G	1	chr2	2A	CAGCCTTGCGAGTATGTAACCAAAGAAGAATCCAGAAGCATGAAGAAGGCAAGATGCTAAAATC
TP40795_Hit	D	1	chr2	2A	CAGCGTGAGATCAGATTTAGAAGAATTAGCTCACCAAGGTGAATTTTCCTTAGATAAAGTTTCT
TP40795_Query	D	1	chr2	2A	CAGCGTGAGATCAGATTTAGAAGAATTAGCTCACCAAGGTGAATTTTCCGTAGATAAAGTTTCT
TP55779_Hit	D	1	chr2	2A	CAGCTTCTTGACAAGCATAAAGTGAAAGGCGAGTTTAGAAGTCCAGTTGTACAAATAAATGCAT
TP55779_Query	D	1	chr2	2A	CAGCTTCTAGACAAGCATAAAGTGAAAGGCGAGTTTAGAAGTCCAGTTGTACAAATAAATGCAT
TP55815_Hit	D	1	chr2	2A	CAGCTTCTATCCGGATACAGAAACATGTCCGTGCACATAGAGCAAGAGTGTATTATGCATCATT
TP55815_Query	D	1	chr2	2A	CAGCTTCTATCCGAATACAGAAACATGTCCGTGCACATAGAGCAAGAGTGTATTATGCATCATT
TP58003_Hit	D+G	1	chr2	2A	CAGCTTGTAGCAGTACTGTTATTCTCATGGTTTTTTTATATCAAATATTTTTCCAAAATTATAA
TP58003_Query	D+G	1	chr2	2A	CAGCTTGTAGCAGTAATGTTATTCTCATGGTTTTTTTATATCAAATATTTTTCCAAAATTATAA
TP62111_Hit	D	1	chr2	2A	CTGCAACACCTTGCTTAGCGGTGGAGAGCCTTGATCTCTTCTTAGCCAAACTCTCACTGCGGCG
TP62111_Query	D	1	chr2	2A	CTGCAACACCTTGCTAAGCGGTGGAGAGCCTTGATCTCTTCTTAGCCAAACTCTCACTGCGGCG
TP62837_Hit	D	1	chr2	2A	CTGCAACTGTGAGTATGTCCAAAGTAGCAGTTTCATTCACTCACATCATCAAGAGTGGAGAGCC
TP62837_Query	D	1	chr2	2A	CTGCAACTGTGAGTATGTCCAAAGTAGCAGTTTCATTCACTCACATCATCAAGAGTGGAGAACC
TP63583_Hit	D	1	chr2	2A	CTGCAAGTCTCATAGCTCCTTCATATAGGCAATTAAGGGATTCTGGTGAACTTGAAGAATTTGA
TP63583_Query	D	1	chr2	2A	CTGCAAGTCTCATAGCTCCTTCATATAGGCAATTAAGGGATTCTGATGAACTTGAAGAATTTGA
TP63632_Hit	D	1	chr2	2A	CTGCAAGTGTCTTTGTACCTGCCACAGGTTTTGAATATTGGTTTCTTACAATTCAAGAAACAAA
TP63632_Query	D	1	chr2	2A	CTGCAAGTGTCTTTGTACCTGCCACAGGTTTTGAATATTAGTTTCTTACAATTCAAGAAACAAA
TP65474_Hit	D	1	chr2	2A	CTGCACAGGTCAAAGAGGCAGTTGAATGAGCAGAAGGATTTGGAGCTTGGTGAGAAGAACGTAA
TP65474_Query	D	1	chr2	2A	CTGCACAGGTCAAAGAGGCAGTTGAATGACCAGAAGGATTTGGAGCTTGGTGAGAAGAACGTAA
TP65867_Hit	D	1	chr2	2A	CTGCACCACTGATAATTCATGTGAGTCTGATTCGGTTAACCTTGTTGGCGAATTTGACTCTTCT
TP65867_Query	D	1	chr2	2A	CTGCACCACTGATAATTCATGTGAGTCTGATTCGGTTAACCTTGTTGGCGAATTTGACTCTTCC
TP66034_Hit	D	1	chr2	2A	CTGCACCCATGATACCAACAGCACCACTACGAGCTTCTCCCTCCAATGCTAATTGTGTAGTAAT
TP66034_Query	D	1	chr2	2A	CTGCACCCATGATACCAACAGCACCACTACGAGCTTCTCCCTCCAATGCTAATTGAGTAGTAAT
TP79112_Hit	D+G	1	chr2	2A	CTGCCTTAAGTTTGAGCCAGCCCACAACAGCCTTTCCTGAGGATCGGTTTCGCTCATCATCTAC
TP79112_Query	D+G	1	chr2	2A	CTGCCTTAAGTTTGAGCCAGCCCACAACAGCCTTTCCTGAGGAACGGTTTCGCTCATCATCTAC

Name	Filter	Nb hit (Mt4.0)	Mt Chr (Mt4.0)	Ms Chr (Li et al., 2014)	Sequence
TP79244_Hit	D	1	chr2	2A	CTGCCTTCCTTGCAGTCTCTGGGAAGCATTGAACATATTTTGAATGTTGGGATTCTGGCCAACA
TP79244_Query	D	1	chr2	2A	CTGCCTTCCTTGCAGTCTCTGGGAAGCATTGAACATATTTTGAATGTTGGAATTCTGGCCAACA
TP79724_Hit	D	1	chr2	2A	CTGCGAACAACTGCATATCAGAGGATTGGTTTGGATTTCTGGTGTATCCACGTAGATCAGTCTT
TP79724_Query	D	1	chr2	2A	CTGCGAACAACTGCATATCAGAGGATTGGTTTGGATTTCTGGTGTATCCACGTAGATCAATCTT
TP80383_Hit	D	1	chr2	2A	CTGCGAGTAGCTTCAGGCACCAAACCATTCCCTGAAACAAGTTGTCCACTAAGCCAACAAGGCA
TP80383_Query	D	1	chr2	2A	CTGCGAGTAGCTTCAGGCACCAAACCATTCCCTAAAACAAGTTGTCCACTAAGCCAACAAGGCA
TP8653_Hit	D+G	1	chr2	2A	CAGCAATTTTGATGAGTGGAAGAAATGCAAAAACTAACTTTCCAGTTAATGTGGAAAATCAAAC
TP8653_Query	D+G	1	chr2	2A	CAGCAATTTTGATGAGTGGAAGAAATGCAAAAACTAACTTTCCAATTAATGTGGAAAATCAAAC
TP86713_Hit	D	1	chr2	2A	CTGCTATTGAATATGAGAAGAAAGGATTCACGGAAAACTATGAACATGGTCAAGTGATGGAGAA
TP86713_Query	D	1	chr2	2A	CTGCTATTGAATATGAGAAGAAAGGATTCACAGAAAACTATGAACATGGTCAAGTGATGGAGAA
TP89394_Hit	D+G	1	chr2	2A	CTGCTTTTTTTAATCAGGAAAATGTCAGGACAAAGAATCCTCAGTTGCTGTATGAGTCCAAGTT
TP89394_Query	D+G	1	chr2	2A	CTGCTCTTTTTAATCAGGAAAATGTCAGGACAAAGAATCCTCAGTTGCTGTATGAGTCCAAGTT
TP90065_Hit	D	1	chr2	2A	CTGCTGATACGCCTGGATGGTATTGTGGACCTAACTTCTTTTCAACTGAGAAACTCCGCGAGTT
TP90065_Query	D	1	chr2	2A	CTGCTGATACGCCTGGATGGTATTGTGGACCTAACTTCTTTTCAACTGAGAAACTCCGCGAATT
TP93192_Hit	D	1	chr2	2A	CTGCTTCAAGTCCTACTATTCCTAAATTCTGTCTACCAAGTGTTTCTTGAGCTTTCTCAAGCAC
TP93192_Query	D	1	chr2	2A	CTGCTTCAAGCCCTACTATTCCTAAATTCTGTCTACCAAGTGTTTCTTGAGCTTTCTCAAGCAC
TP93579_Hit	D	1	chr2	2A	CTGCTTCCTGAGGTATTTTCTTAAACATGAATTTTATTTTCCCACTGGTTTTTCACTTCTTCGA
TP93579_Query	D	1	chr2	2A	CTGCTTCCTGAGGTATTTTCTTAAACATGAATTTTATTTTCCCACTGGTTTTTCACTTCTTAGA
TP9528_Hit	D	1	chr2	2A	CAGCACAGCGGGGGCAAAGCTTTTATTCACCCTACATCTAAAATTCACATCGGCCTCCAGATAC
TP9528_Query	D	1	chr2	2A	CAGCACAGCGGGGGCAAAGCTTTTATTCACCCTACAACTAAAATTCACATCGGCCTCCAGATAC
TP14127_Hit	D+G	1	chr2	2B	CAGCAGATCCGGAGACAGAAAATGGAAGCCCTCCGCTGGCTGGAACGCTGGAAAAGTCGTGGAC
TP14127_Query	D+G	1	chr2	2B	CAGCAGATCCGGAGACAGAAAATGGAAGCCCTCCGCTGGCTGGAACGCTGGAAAAGTCATGGAC
TP50193_Hit	D+G	1	chr2	2B	CAGCTGAATCTTGATTGGAGATTGATCCGTCATAATGAGCATGAATTTGACTTTTGAACCTTTT
TP50193_Query	D+G	1	chr2	2B	CAGCTGAATCTTGATTGGAGATTGATCCGTCATAATGAGCATGAATTTGAATTTTGAACCTTTT
TP5723_Hit	D	1	chr2	2B	CAGCAAGCCATGCAAAAAAACACATAACTGGAACAGGAACATGATCAATCAACCCTACAACAGA
TP5723_Query	D	1	chr2	2B	CAGCAAGCCATGCAAAAAAACACATAACCGGAACAGGAACATGATCAATCAACCCTACAACAGA
TP63821_Hit	D	1	chr2	2B	CTGCAATACCACCACAAATGGCATTCAGTACAAGTTCCATTAAGAAAAGAGCAAGGCAAAAGAC
TP63821_Query	D	1	chr2	2B	CTGCAATACCACCACAAATGGAATTCAGTACAAGTTCCATTAAGAAAAGAGCAAGGCAAAAGAC
TP7378_Hit	D	1	chr2	2B	CAGCAATCATTCCAACCTACATCATAGAATAATTAAAGGTGATTATCTTATCCCTACAACGACA
TP7378_Query	D	1	chr2	2B	CAGCAATCATTCCAACCTACATCATAGAATAATTAAAGGTGATTATCTTATCCCTACAACAACA
TP80608_Hit	D	1	chr2	2B	CTGCGATGGATCAGTTTTGTTGAACAACACTGATACAATTGTAAGTGAGCAGGATGCATTTCCA
TP80608_Query	D	1	chr2	2B	CTGCGATGGATCAGTTTTGTTGAACAACACTGATACAATTGTAAGTGAGCAAGATGCATTTCCA
TP92741_Hit	D	1	chr2	2B	CTGCTTAACCATTTACCTTATAGACAGGTCCATAACCTCCTTGTCCAAGCTTGTTTGAATCTGA
TP92741_Query	D	1	chr2	2B	CTGCTTAACCATTTACCTTATAGACAGGGCCATAACCTCCTTGTCCAAGCTTGTTTGAATCTGA
TP10802_Hit	D+G	1	chr2	2C	CAGCACTGGTTCAACCACTATGGGAATATCATTATTACGGACAATCTCATCAGTTACGTTGTCA
TP10802_Query	D+G	1	chr2	2C	CAGCACCGGTTCAACCACTATGGGAATATCATTATTACGGACAATCTCATCAGTTACGTTGTCA
TP1172_Hit	D	1	chr2	2C	CAGCAAACACAGACCATCATGAAAGGTCTGATGATTGGGCTGTCCAACTTAACCTAGTTAGTTC
TP1172_Query	D	1	chr2	2C	CAGCAAACACAGACCATCATGAAAGGTCTGATGATTGGGCTGACCAACTTAACCTAGTTAGTTC
TP15433_Hit	D+G	1	chr2	2C	CAGCAGGTTGCTTATACTCAAGAAGAAGAACACCAGTTGGCATATCGGTAATTCCAGACGTACC
TP15433_Query	D+G	1	chr2	2C	CAGCAGGTTGCTTATACTCAAGAAGAAGAACACCAGTTGGCATATCAGTAATTCCAGACGTACC
TP21721_Hit	D	1	chr2	2C	CAGCATTGCCCGTGCTGGCCACAGTTAACAAATTCACAGCCATGTTAGGATCGTCTTCCTCCAT
TP21721_Query	D	1	chr2	2C	CAGCATTGCCCGTGCTGGCCACAGTTAACAAATTCACAGCCATGTTAGGATCGTCTTCCTCCAC
TP24360_Hit	D+G	1	chr2	2C	CAGCCACAATTGCAACAGCAACATAAAAGATTTTGAGGTCTCTGCCATGACATCACTACCGCAT
TP24360_Query	D+G	1	chr2	2C	CAGCCACAATTGCAACAGCAACAGAAAAGATTTTGAGGTCTCTGCCATGACATCACTACCGCAT
TP29754_Hit	D	1	chr2	2C	CAGCCCTTGATCAATTCCATTTCCATGTGTAGATAGTAGAAACAAAATTAGATTTGCCTAGACA
TP29754_Query	D	1	chr2	2C	CAGCCCTTGATCAATTCCATTTCCATGTGTAGAAAGTAGAAACAAAATTAGATTTGCCTAGACA
TP30233_Hit	D	1	chr2	2C	CAGCCGATGCGGTAGTGATGTCATGGCAGAGACCTCAAAATCTTTTCTGTTGCTGTTGCAATTG
TP30233_Query	D	1	chr2	2C	CAGCCGATGCGGTAGTGATGTCATGGCAGAGACCTCAAAATCTTTTATGTTGCTGTTGCAATTG
TP31990_Hit	D+G	1	chr2	2C	CAGCCTAGTAGCGATAACGGTACGTCTGGAATTACTGATATGCCAACTGGTGTTCTTCTTCTTG
TP31990_Query	D+G	1	chr2	2C	CAGCCTAGTAGCGATAACGGTACGTCTGGAATTACCGATATGCCAACTGGTGTTCTTCTTCTTG
TP33770_Hit	D+G	1	chr2	2C	CAGCCTGGTGATTATTAGGAGTGTTGCATAATTTGTTCCTTAGATTAAGATCCATTCCATTGTG
TP33770_Query	D+G	1	chr2	2C	CAGCCTGGTGATTATTAGGAGTGTTACATAATTTGTTCCTTAGATTAAGATCCATTCCATTGTG
TP36357_Hit	D+G	1	chr2	2C	CAGCTAGGTTTAGGTTCCCCATACTGGCACTTTTCTTGATGATTGATCCATCGGTTAATCTAAC
TP36357_Query	D+G	1	chr2	2C	CAGCGAGGTTTAGGTTCCCCATACTGGCACTTTTCTTGATGATTGATCCATCGGTTAATCTAAC

Name	Filter	Nb hit (Mt4.0)	Mt Chr (Mt4.0)	Ms Chr (Li et al., 2014)	Sequence
TP37197_Hit	D	1	chr2	2C	CAGCGCAGTTTTTGAAAGGGTTTCCGGCCCGTAACTTCTGATTCATAATATTGTGAACCATCAT
TP37197_Query	D	1	chr2	2C	CAGCGCAGTTTTTGAAAGGGTTTCCAGCCCGTAACTTCTGATTCATAATATTGTGAACCATCAT
TP45486_Hit	D	1	chr2	2C	CAGCTCTTGGACATGTTTTAAGTTATTTTCATAAGCTCTTCAAACAAGTATCATAAGTGTTTAT
TP45486_Query	D	1	chr2	2C	CAGCTATTGGACATGTTTTAAGTTATTTTCATAAGCTCTTCAAACAAGTATCATAAGTGTTTAT
TP46491_Hit	D+G	1	chr2	2C	CAGCTCAGCTTCTTGGCTTTGAGAACTTCATTCCACCATTTGCAAACATTAATGGCTCTGACAT
TP46491_Query	D+G	1	chr2	2C	CAGCTCAGCTTCTTGGCTTTGAGAACTTCATTCCACCATTTGCAAACATTAATGGCTCAGACAT
TP47012_Hit	D	1	chr2	2C	CAGCTCCAAGAGCAAAATGTTGACTTCCAAGCCAGGGCTTTTGAACTTCAGAAACAGCGGTTTA
TP47012_Query	D	1	chr2	2C	CAGCTCCAAGAGCAAAATGTTGACTTCCAAGCCAGGGCTTTTGAACTTCAGAAACAGCGGTTCA
TP54745_Hit	D+G	1	chr2	2C	CAGCTTCACGTTTCATTTGCGCGGCTTTCCCACTGCCGCGGAAGTACATGTATACTTGCTGAAA
TP54745_Query	D+G	1	chr2	2C	CAGCTTCACGTTTCATTTGCGCGGCTTTCCCACTGCCGCGGAAGTACATGTATACGTGCTGAAA
TP79221_Hit	D	1	chr2	2C	CTGCCTTCATTCATCGCTACATCGTCATCACCACCCTCTTTCTGATGATCAGTAGTTGAATTGA
TP79221_Query	D	1	chr2	2C	CTGCCTTCATTCATCGCTACATCGTCATCACCACCCTCTTTCTGATGATCAGTAGTTGAACTGA
TP10130_Hit	D	1	chr2	2D	CAGCACCAATCATCTTCTTTGTCTGCACCGGACATTGAGAACTATGTTAAGGACATTGAAAATG
TP10130_Query	D	1	chr2	2D	CAGCACCAATCATCTTCTTTGTCTGCACCAGACATTGAGAACTATGTTAAGGACATTGAAAATG
TP14949_Hit	D+G	1	chr2	2D	CAGCGGGGACACCATCATCAACCTCTCCCTTATACACCTTACCAAAGCCTCCAACTCCGATGAC
TP14949_Query	D+G	1	chr2	2D	CAGCAGGGACACCATCATCAACCTCTCCCTTATACACCTTACCAAAGCCTCCAACTCCGATGAC
TP19246_Hit	D	1	chr2	2D	CAGCATCTAAGGTAAATTCTCTGCACCTAAAGTACTTCATCGTCTCTGGACGTATTATTGCATT
TP19246_Query	D	1	chr2	2D	CAGCATCTAAGGTAAATTCTCTGCACCTAAAGTACTTCATCCTCTCTGGACGTATTATTGCATT
TP23109_Hit	D	1	chr2	2D	CAGCCAAGTCACCACATGACTCTTCTTTCTCCTTCTCTAGAAGGTATTTCCATTGGAAAAAGAA
TP23109_Query	D	1	chr2	2D	CAGCCAAATCACCACATGACTCTTCTTTCTCCTTCTCTAGAAGGTATTTCCATTGGAAAAAGAA
TP24540_Hit	D	1	chr2	2D	CAGCCACATATGATAGAAATGAAAAATTCTGAAGGAATGACACCTCGCGAACTATTTACGAAAG
TP24540_Query	D	1	chr2	2D	CAGCCACATAAGATAGAAATGAAAAATTCTGAAGGAATGACACCTCGCGAACTATTTACGAAAG
TP27688_Hit	D+G	1	chr2	2D	CAGCCCAAAGCACGGTGGAGCTAGCCGTGTGGACTATTGCCAGTAGAAACTTGGTGGTGTCATT
TP27688_Query	D+G	1	chr2	2D	CAGCCCAAAGCACGGTGGAGCTAGCCGTGTGGACGATTGCCAGTAGAAACTTGGTGGTGTCATT
TP3007_Hit	D+G	1	chr2	2D	CAGCAATAACTACCTGTAGAAAATTGAACATTTATTTCTTGCGCTTAAATATTTTATAGATACT
TP3007_Query	D+G	1	chr2	2D	CAGCAACAACTACCTGTAGAAAATTGAACATTTATTTCTTGCGCTTAAATATTTTATAGATACT
TP30978_Hit	D	1	chr2	2D	CAGCCGGCAATGGTGGAGGAGCAACAGAAGGCATTTGGTAAGACTGGAAATAAACCCCGTCAGG
TP30978_Query	D	1	chr2	2D	CAGCCGGCAATGGTGGAGGAGCAACAGAAGGCATTTGGTAAGACTGGAAATAAACCCCATCAGG
TP32762_Hit	D	1	chr2	2D	CAGCCTCCTACCTCATCAACAATTTTGGGTTTTCCTCACAATCTGCTTCCAAATTTTGTTCCAC
TP32762_Query	D	1	chr2	2D	CAGCCTCCTACCTCATCAACAATTTTGGCTTTTCCTCACAATCTGCTTCCAAATTTTGTTCCAC
TP4003_Hit	D+G	1	chr2	2D	CAGCAACCATACCACAGTGTGCATATCCTAAAACCAAGTTGCTTATCCCACCGTCATTTAAAAC
TP4003_Query	D+G	1	chr2	2D	CAGCAACCATACCACAGTGTGCATATCCTAAAACCAAGTTGCTTATCCCACCATCATTTAAAAC
TP56547_Hit	D+G	1	chr2	2D	CAGCTTCTTCAGTTTCTCCCGTTTGTGCATATCCTGCCAGCATTGCTGACCATGCCATTAAGTC
TP56547_Query	D+G	1	chr2	2D	CAGCTTCTTCAGTTTCTCCCATTTGTGCATATCCTGCCAGCATTGCTGACCATGCCATTAAGTC
TP62827_Hit	D+G	1	chr2	2D	CTGCAACTGGTGCTGTCGTGCCCTTCCACCATTCGGTTTTAAATGATGGTGGGATAAGCAACTT
TP62827_Query	D+G	1	chr2	2D	CTGCAACTGGTGCTGTCGTGCCCTTCCACCATTCGGTTTTAAATGACGGTGGGATAAGCAACTT
TP70985_Hit	D	1	chr2	2D	CTGCATCACAGAGCTGTGGGTGATACATTCTAATTACTAAAAGTCATGTTGTGTATAAGTATCT
TP70985_Query	D	1	chr2	2D	CTGCATCACAGAGCTGTGGGTGATACATTCTAATTACTAAAAATCATGTTGTGTATAAGTATCT
TP77872_Hit	D+G	1	chr2	2D	CTGCCGTCAATGACACCACCAAGTTTCTACTGGCAATCGTCCACACGGCTAGCTCCACCGTGCT
TP77872_Query	D+G	1	chr2	2D	CTGCCGTCAATGACACCACCAAGTTTCTACTGGCAATAGTCCACACGGCTAGCTCCACCGTGCT
TP83307_Hit	D+G	1	chr2	2D	CTGCGTCCTCCTCGTCTTCGACAAACTCGACGAACTCTGCACCTCGCTGAAAAAAAAAAAAAAA
TP83307_Query	D+G	1	chr2	2D	CTGCGTCCTCCTAGTCTTCGACAAACTCGACGAACTCTGCACCTCGCTGAAAAAAAAAAAAAAA
TP69607_Hit	D	1	chr2	3C	CTGCAGTAGGAGTTTCCATAGCTCTATTCAATCAAGCATCAAGGATCTTTATATTCCCACTAGT
TP69607_Query	D	1	chr2	3C	CTGCAGTAGGAGTTTCAATAGCTCTATTCAATCAAGCATCAAGGATCTTTATATTCCCACTAGT
TP63538_Hit	D	1	chr2	5D	CTGCGAGTAGCTTCAGGCATCAAACCATTCCCTGAAACAAGTTGTCCATTAAGCCAACAAGGCA
TP63538_Query	D	1	chr2	5D	CTGCAAGTAGCTTCAGGCATCAAACCATTCCCTGAAACAAGTTGTCCATTAAGCCAACAAGGCA
TP10131_Hit	D	1	chr3	.	CAGCACCAATCATTGGGAAATTGGTGAATAATTTTAACGCTACATATGAGAATCAGTCGTCCAT
TP10131_Query	D	1	chr3	.	CAGCACCAATCATTGGGAAATTGGTGAATAATTTTAACGCTACATATGAGAAACAGTCGTCCAT
TP10171_Hit	D+G	1	chr3	.	CAGCACCACGAAACTCATTTGGCTCAAAAGGTGAGAATAGGTTTATACTACAGATTAGGATATT
TP10171_Query	D+G	1	chr3	.	CAGCACCACAAAACTCATTTGGCTCAAAAGGTGAGAATAGGTTTATACTACAGATTAGGATATT
TP10265_Hit	D	1	chr3	.	CAGCACCACTAGCACCATTGTCAAAATTTCCCATACCTAAAGGATGAGCCACATCAGCATGATC
TP10265_Query	D	1	chr3	.	CAGCACCACTAGCACCATTGTCAAAATTTCCCATACCTAAAGGATGAGCAACATCAGCATGATC
TP10317_Hit	D+G	1	chr3	.	CAGCACCAGTACTATAAGAGCTACTGGAATCTGTGGCGTAACTTGTATACGGATGAGAGGCATA
TP10317_Query	D+G	1	chr3	.	CAGCACCAGCACTATAAGAGCTACTGGAATCTGTGGCGTAACTTGTATACGGATGAGAGGCATA

Name	Filter	Nb hit (Mt4.0)	Mt Chr (Mt4.0)	Ms Chr (Li et al., 2014)	Sequence
TP1032_Hit	D+G	1	chr3	.	CAGCAAAATTCTAAACATCACGAGCCGTCCAAGGTTGCTTTTGTTTTGCCTGATGTCATGAAGC
TP1032_Query	D+G	1	chr3	.	CAGCAAAATTCTAAACATCACGAGCCGTCCAAGGTTGCTTTTGTGTTGCCTGATGTCATGAAGC
TP10409_Hit	D+G	1	chr3	.	CAGCACCATCACGAGAAAGCCATGCATGAGGAGATGTCTCAGGTGCCTGCTACGTGGGAGCCTG
TP10409_Query	D+G	1	chr3	.	CAGCACCATCACGAGAAAGCCATGCATGAGGAGATGTCTCAGGTGCCTGCTACATGGGAGCCTG
TP10421_Hit	D	1	chr3	.	CAGCACCATCCATCTCCATCTCCATCTCCACAGGGAATAAGGAGTTTTCATAATTATTATTATT
TP10421_Query	D	1	chr3	.	CAGCACCATCCATCTCCATCTCCATCTCCACAGGGAATAAGGAGTTTTCATAATTATTAGTATT
TP10497_Hit	D	1	chr3	.	CAGCACCCAAAACAACAATTACAAGACACTGTATTTTGTCTTCTTCATCAACGGCACTCCTTGA
TP10497_Query	D	1	chr3	.	CAGCACCCAAAACAACAATTACAAGACACTGTATTTTGTCTTCTTCAACAACGGCACTCCTTGA
TP1057_Hit	D	1	chr3	.	CAGCAAAATTTTTACACAATTTCTTATTCTGCAACTATCCGCATAAACTAATATAATGTAACAA
TP1057_Query	D	1	chr3	.	CAGCAAAATTTCTACACAATTTCTTATTCTGCAACTATCCGCATAAACTAATATAATGTAACAA
TP10610_Hit	D	1	chr3	.	CAGCACCCTCATGTTTTCCTCCGAAGGAGAAATGAAGAGGAACAACTGGGAAACTTTGAATCTC
TP10610_Query	D	1	chr3	.	CAGCACCCTCATGTTTTCCTCCAAAGGAGAAATGAAGAGGAACAACTGGGAAACTTTGAATCTC
TP10651_Hit	D+G	1	chr3	.	CAGCACCCTTTCAATAGAAGGATCTTTAGGAAGGATGAGAGGGGAATAGTGAGTGCAGTTCACC
TP10651_Query	D+G	1	chr3	.	CAGCACCCTTTCAATAGAAGGATCTTTAGGAAGGATGAGAGAGGAATAGTGAGTGCAGTTCACC
TP11120_Hit	D+G	1	chr3	.	CAGCACGAAATAGGAAGCCTTGCACTTTAGCTAGCCTGTTGCTCAAAGCAGTCCGCTGAAAAAA
TP11120_Query	D+G	1	chr3	.	CAGCACGAAATAGGAAGCCTTGCACTTTAGCTAGCCTGTTGCTCAAAGCAGTCCGCAGAAAAAA
TP11580_Hit	D+G	1	chr3	.	CTGCACGTAACACAGTCAATGCCTCTTTCCATCTCCATTGGCGTTCAGACAATGGAAGAAGGTG
TP11580_Query	D+G	1	chr3	.	CAGCACGTAACACAGTCAATGCCTCTTTCCATCTCCATTGGCGTTCAGACAATGGAAGAAGGTG
TP11612_Hit	D	1	chr3	.	CAGCACGTCTTTAATTGTGCATGTTAGCAATAGGGTTTGAACAATTTGACTATCCTTGGACATT
TP11612_Query	D	1	chr3	.	CAGCACGTCTTTAATTGTGCATGTTAGCAATAGGCTTTGAACAATTTGACTATCCTTGGACATT
TP11763_Hit	D+G	1	chr3	.	CAGCACTAATGTCAGATGCTAAGTTTGACGGTGCTGACATGACAGAAGTTGTAATGTCAAAGGC
TP11763_Query	D+G	1	chr3	.	CAGCACTAATGTCAGATGCCAAGTTTGACGGTGCTGACATGACAGAAGTTGTAATGTCAAAGGC
TP11800_Hit	D	1	chr3	.	CAGCACTGCCTTCATGTAGAAGGTCACTAAGAGAAACAACAGAAAGCATACTCCTCAAAGCATC
TP11800_Query	D	1	chr3	.	CAGCACTACCTTCATGTAGAAGGTCACTAAGAGAAACAACAGAAAGCATACTCCTCAAAGCATC
TP11835_Hit	D	1	chr3	.	CAGCACTAGAAACACTCATTGAAATAGCAAATTTGCGATCATCATTGTTGCACTCTTTTAACCA
TP11835_Query	D	1	chr3	.	CAGCACTAGAAACACTCATTGAAATAGCAAATTTGCGATCATCATTGTTACACTCTTTTAACCA
TP11868_Hit	D	1	chr3	.	CAGCACTAGGAGACATAAAATCCTCAGTTAATTGTTGGTGGATGCAAATTACCTTCAACCATAG
TP11868_Query	D	1	chr3	.	CAGCACTAGGAGACATAAAACCCTCAGTTAATTGTTGGTGGATGCAAATTACCTTCAACCATAG
TP11894_Hit	D	1	chr3	.	CAGCACTCTACATTATCCAACCTTTTTGCTTGGACGTCAACTACTCAAGTTGTTTTTTTACAGG
TP11894_Query	D	1	chr3	.	CAGCACTATACATTATCCAACCTTTTTGCTTGGACGTCAACTACTCAAGTTGTTTTTTTACAGG
TP11901_Hit	D+G	1	chr3	.	CAGCACTATACTTGTTTGCTGGATCTTTTTAGTCGGTCGGGGCACCTTGAGGAGGCAGAGAATC
TP11901_Query	D+G	1	chr3	.	CAGCACTATACTTGTTTGCTGGATCTTTTTAGTCGGTCAGGGCACCTTGAGGAGGCAGAGAATC
TP12371_Hit	D	1	chr3	.	CAGCACTGTGTTCAGAAACTTAAGCTTTTGGGACTTGAAGACACCTCATCTGTAATTAAGGAAG
TP12371_Query	D	1	chr3	.	CAGCACTGTGTTCAGAAACTTAAGCTTTTGGGACTTGAAGAAACCTCATCTGTAATTAAGGAAG
TP12497_Hit	D	1	chr3	.	CAGCACTTCATGGGAAAGTCCTTGTTGCCTGCAATTAACATAGTCAATTACACTGATAGATTGG
TP12497_Query	D	1	chr3	.	CAGCACTTCATGGGAAAGTCCTTGTTGCCTGCAATTAACATAGTCAATTACAATGATAGATTGG
TP12511_Hit	D	1	chr3	.	CAGCACTTCCTAAATTTCATATGGTTTATGGGTTTTAACCCTCTATAAAAACTATAACTGTTTC
TP12511_Query	D	1	chr3	.	CAGCACTTCCTAAATTCCATATGGTTTATGGGTTTTAACCCTCTATAAAAACTATAACTGTTTC
TP12633_Hit	D	1	chr3	.	CAGCACTTGGTGCTTTTTCCAAAGCTTTCTCCAGTGCAATCTTCCACTCGTATAAGTCCTCTGT
TP12633_Query	D	1	chr3	.	CAGCACTTGGTGCTTTTTCCAAAGCTTTCTCCAGTGCAATCTTCCACTCATATAAGTCCTCTGT
TP12643_Hit	D	1	chr3	.	CAGCACTTGTCACAGAAAAATTTGTCTTCTGCATTCAATGTCTCAGTTGAACTGAAATTTTTCA
TP12643_Query	D	1	chr3	.	CAGCACTTGTCACAGAAAAATTTGTCTTCTGCATTCAATGTCTCAGTCGAACTGAAATTTTTCA
TP12788_Hit	D+G	1	chr3	.	CAGCAGAAAAGCAGTTCAATGAAAGCATTAAGGCCAAGTCGAATGAAGTCATGCCTGATGATCA
TP12788_Query	D+G	1	chr3	.	CAGCAGAAAAGCAGTTCAATGAAAGCATTAAGGCCAAGTCGAATGAAGTAATGCCTGATGATCA
TP12804_Hit	D	1	chr3	.	CAGCAGAAAATAGTCCTGAGAGTAAATCACCGGAACTTGCACTTACGAAATCTGGACTGCTAGA
TP12804_Query	D	1	chr3	.	CAGCAGAAAATAGTACTGAGAGTAAATCACCGGAACTTGCACTTACGAAATCTGGACTGCTAGA
TP12939_Hit	D	1	chr3	.	CAGCAGATATGATGTACCTTTTCAGGGTCATAAGTGAGAAGCTTGCTCAACAAATCAAATCCAG
TP12939_Query	D	1	chr3	.	CAGCAGAAATGATGTACCTTTTCAGGGTCATAAGTGAGAAGCTTGCTCAACAAATCAAATCCAG
TP13022_Hit	D	1	chr3	.	CAGCAGAACCATTGCTCACACTAAAGCTTGGTAAAAGATTCTACTTTGAGGATGTTAGCACTGG
TP13022_Query	D	1	chr3	.	CAGCAGAACCATTGCTCACACTAAAGCTTGGTAAAAGATTCTACTTTGAGGATGTTACCACTGG
TP13213_Hit	D	1	chr3	.	CAGCAGAAGGGAAGAAGTGAGGAAATAGGAAGTGGCTTAGATTCTGCCATGGTAGCTCAACCAA
TP13213_Query	D	1	chr3	.	CAGCAGAAGGGAAGAAGTAAGGAAATAGGAAGTGGCTTAGATTCTGCCATGGTAGCTCAACCAA
TP13224_Hit	D	1	chr3	.	CAGCAGAAGGTGGCGGTGTTGGTGCATTCTCGGCCGCGGAATTTGCTGAAAAAAAAAAAAAAAA
TP13224_Query	D	1	chr3	.	CAGCAGAAGGTGGCGGTGTTGGTGCATTCTCGGCCGCGGAATTTGCAGAAAAAAAAAAAAAAAA

Name	Filter	Nb hit (Mt4.0)	Mt Chr (Mt4.0)	Ms Chr (Li et al., 2014)	Sequence
TP13243_Hit	D	1	chr3	.	CAGCAGAAGTGACTGTGGTGCCACCTTCCCGATTAATGGCTTTAATTGGTCAGGCTTTGAGGTG
TP13243_Query	D	1	chr3	.	CAGCAGAAGTGACTGTGGTGCCACCTTCCCGATTAATGGCTTTAATTGGTCAGGCTTTGAAGTG
TP1333_Hit	D	1	chr3	.	CAGCAAACCCTGGGCATTATACCAAGTACTTGGTACATACATATTGTAAGGTGGAGAGTAGAGC
TP1333_Query	D	1	chr3	.	CAGCAAACCCTGGGCATTATACCAAGTACTTAGTACATACATATTGTAAGGTGGAGAGTAGAGC
TP13339_Hit	D	1	chr3	.	CAGCAGAATGCATAGCAGAATGTAATACCTTTAAGTCTTTTATTCTTTTGGCAAGTCGGTGTTC
TP13339_Query	D	1	chr3	.	CAGCAGAATGCATAGCAGAATATAATACCTTTAAGTCTTTTATTCTTTTGGCAAGTCGGTGTTC
TP1337_Hit	D	1	chr3	.	CAGCAAACCCTTTTCATTTTCGATCCAAACTCTTTCTTTTTCGTCAACTTCACTTCAAACCCAC
TP1337_Query	D	1	chr3	.	CAGCAAACCCTTTTCATTTTCGATCCAAACTCTCTCTTTTTCGTCAACTTCACTTCAAACCCAC
TP13489_Hit	D+G	1	chr3	.	CAGCAGACCATTCCAAAAACAGAAGCTACTTTCTATGATCAGCACCAATCACAATCACAATCAC
TP13489_Query	D+G	1	chr3	.	CAGCAGACCATTCCAAAAACAGAAGCTACTTTCTATGATCAGCACCAATCACAATCACAATCAA
TP13646_Hit	D	1	chr3	.	CAGCAGAGAAATCGTTGTCCTTATTAGATCCCCTGCCATGTCTTCTTCTTTTACCACCGGCATC
TP13646_Query	D	1	chr3	.	CAGCAGAGAAATCGTTGTCCTTATTAGATCCCCTGCCATGTCTTCTTCTTTTACCACCAGCATC
TP13651_Hit	D	1	chr3	.	CAGCAGAGAACGTCACAGTCTCCGCCGGTGATACCGCATTTTCAATCACCGCGCCCGCAGTTAG
TP13651_Query	D	1	chr3	.	CAGCAGAGAACGTCACAGTCTCCGCCGGTGATGCCGCATTTTCAATCACCGCGCCCGCAGTTAG
TP13696_Hit	D	1	chr3	.	CAGCATAGACTCGATCCAACTTTACTCGGGAATGATCAGAATCAACAATACCAAGCCATGTTGG
TP13696_Query	D	1	chr3	.	CAGCAGAGACTCGATCCAACTTTACTCGGGAATGATCAGAATCAACAATACCAAGCCATGTTGG
TP1381_Hit	D	1	chr3	.	CAGCAAACGATTCCCAATTCAAAACCTCGAAAAAAGGAGGCACAAAATTGTCCGATATAATAAC
TP1381_Query	D	1	chr3	.	CAGCAAACGATTCCCAATTCAAAACCTCGAAAAAAGGAGGCACAAAATTGTCCGATATAACAAC
TP13832_Hit	D	1	chr3	.	CAGCAGAGGCAATTGGGAAGCTCTATGAGCACATCTTCATTGAATGATCCCAGGCAGAATAGAT
TP13832_Query	D	1	chr3	.	CAGCAGAGGCAATTGGGAAGCTCTATGAGCACATCTTCATTGAATGATCCCAGGCAAAATAGAT
TP13870_Hit	D	1	chr3	.	CAGCAGAGGGTCATCAATTCAATCTAATAGCTAGAGATTTCATGAAATTAGAGGTGTGGATCAA
TP13870_Query	D	1	chr3	.	CAGCAGAGGGTCAACAATTCAATCTAATAGCTAGAGATTTCATGAAATTAGAGGTGTGGATCAA
TP14050_Hit	D	1	chr3	.	CAGCAGATAGGTAAAATTACTATTTCATCAACTGCTTCTGTCTGGTTTTAATTATTTGCTTAAA
TP14050_Query	D	1	chr3	.	CAGCAGATAGGTAAAATTACTATTCCATCAACTGCTTCTGTCTGGTTTTAATTATTTGCTTAAA
TP14170_Hit	D+G	1	chr3	.	CAGCAGATGAACTCATATTAGAAGTACCTTCTCCACTTAAAGATTCATCGACGCTTTTTCCATG
TP14170_Query	D+G	1	chr3	.	CAGCAGATGAACTCATATGAGAAGTACCTTCTCCACTTAAAGATTCATCGACGCTTTTTCCATG
TP14171_Hit	D	1	chr3	.	CAGCAGATGAACTCATATTAGAAGTACCTTCTCCGCTTAAAGATTCATCGACGTTTTTTCCGTG
TP14171_Query	D	1	chr3	.	CAGCAGATGAACTCATATTAGAAGTACCTTCTCCGCTTAAAGATTCATCGACGTTTTTTCCATG
TP14183_Hit	D	1	chr3	.	CAGCAGATGAGCATTATTGTCAGCTGAGTCATCGATATCTTCCTCATCAGTCTGTTTAATGCGC
TP14183_Query	D	1	chr3	.	CAGCAGATGAGAATTATTGTCAGCTGAGTCATCGATATCTTCCTCATCAGTCTGTTTAATGCGC
TP14245_Hit	D+G	1	chr3	.	CAGCAGATGGTGTGAGCAGATTTAAAAGTAGTAGAATCAATCTTGTTGATCTGGCTGGGTCAGA
TP14245_Query	D+G	1	chr3	.	CAGCAGATGGTGTGAGCAGATTTAAAACTAGTAGAATCAATCTTGTTGATCTGGCTGGGTCAGA
TP14271_Hit	D	1	chr3	.	CAGCAGATTAACTTAAAGAATAACAATAGGATTTAATTGAACCTGACGCAGAAACCGCAAGCTT
TP14271_Query	D	1	chr3	.	CAGCAGATTAACTTAAAGAACAACAATAGGATTTAATTGAACCTGACGCAGAAACCGCAAGCTT
TP14392_Hit	D+G	1	chr3	.	CAGCAGGAAAGCCACATTGTTGAGAGCCATCCTTCCTGAGACAATGTTGGCTGAAAAAAAAAAA
TP14392_Query	D+G	1	chr3	.	CAGCAGGAAAGCCACATTGTTGAGAGCCATCCTTCCTGAGACAACGTTGGCTGAAAAAAAAAAA
TP14430_Hit	D	1	chr3	.	CAGCAGGAACGGCAAAAGGTGGATCCCCTGGATCAAGGTTGGCAATTTACATAGTGTGCAACTT
TP14430_Query	D	1	chr3	.	CAGCAGGAACGGCAAAAGGTGGATCCCCTGGATCAAGGTTGGCAATTTACAAAGTGTGCAACTT
TP14439_Hit	D	1	chr3	.	CAGCAGGAAGAGCGTGAGAAGGTGAAGAAGACAACTACTGGTCCCACTAATGCAGAGGAGGAAC
TP14439_Query	D	1	chr3	.	CAGCAGGAAGAGCGTGAGAAGGTGAAGAAGACAACTACTGGTCCCACTAATGCAAAGGAGGAAC
TP14555_Hit	D	1	chr3	.	CAGCAGGACTGCCAAAAAATATAGAACGACCGCTGTATGTTAACTCTGTTCCAGGAATCTGAGA
TP14555_Query	D	1	chr3	.	CAGCAGGACTGCCAAAAAATATAGAACGACCACTGTATGTTAACTCTGTTCCAGGAATCTGAGA
TP14631_Hit	D	1	chr3	.	CAGCAGGAGGAGCAACAACTGTTGATGCGCCAACAACAGCAACTACTTTCTCAGGTGCTGAAAA
TP14631_Query	D	1	chr3	.	CAGCAGGAGGAGCAACAACTGTTGATGCGCCAACAACAGCAACTACTTTCTCAAGTGCTGAAAA
TP14632_Hit	D	1	chr3	.	CAGCAGGAGGAGCAACAACTGTTGATGCGCCAACAACAGCAACTACTTTCTCAGGTGCGGAGAT
TP14632_Query	D	1	chr3	.	CAGCAGGAGGAGCAACAACTGTTGATGCGCCAACAACAGCAACTACTTTCTCAGGTGCCGAGAT
TP14642_Hit	D+G	1	chr3	.	CAGCGGGAGGAGGTTTTTTGGCATGGTATGTATTCAAAATAGATCTCGACTAACGTGATTGGGA
TP14642_Query	D+G	1	chr3	.	CAGCAGGAGGAGGTTTTTTGGCATGGTATGTATTCAAAATAGATCTCGACTAACGTGATTGGGA
TP14806_Hit	D	1	chr3	.	CAGCAGGCATGGGTTTGCTTGTCGTATCCTCCATTTGTTGAGAAATTTCTTCTATTTGATCGGA
TP14806_Query	D	1	chr3	.	CAGCAGGCATGGGTTTGCTTGTCGTATCCTCCATTTGTTGAGAAATTTCTTCTATTTGATCAGA
TP14820_Hit	D+G	1	chr3	.	CAGCAGGCCCACAGTACCTTGTTTCAGATGCAAAAAGACCCCGCTTGGTGTCAATTGTCCATAA
TP14820_Query	D+G	1	chr3	.	CAGCAGGCCCACAGTACCTTGTTTCAGATGCAAAAAGACCCCGCTTGGTATCAATTGTCCATAA
TP14920_Hit	D	1	chr3	.	CAGCAGGCTTCTGTGGCTCATAGTGGATTGTTGCCTCGAGCAGTTATATCACAGCTGAAAAAAA
TP14920_Query	D	1	chr3	.	CAGCAGGCTTCTGTGGCTCATAGTGGATTGTTGCCTCGAGCAGTTATATCACAGCAGAAAAAAA

Name	Filter	Nb hit (Mt4.0)	Mt Chr (Mt4.0)	Ms Chr (Li et al., 2014)	Sequence
TP15064_Hit	D	1	chr3	.	CAGCAGGGGAGTTTTCAATCTCATAAAGAAATTAAATGTTGTTCCTGATACCAAGATAAAAGGA
TP15064_Query	D	1	chr3	.	CAGCAGGGGAGTTTTCAATCTCATAAAGAAATTAAATGTCGTTCCTGATACCAAGATAAAAGGA
TP15282_Hit	D	1	chr3	.	CAGCAGGTGAATTTTTGTATATCAGATGTTCTAGAGTAATCATAGACAATAAAAAGGGCATGAT
TP15282_Query	D	1	chr3	.	CAGCAGGTGAAATTTTGTATATCAGATGTTCTAGAGTAATCATAGACAATAAAAAGGGCATGAT
TP15385_Hit	D	1	chr3	.	CAGCAGGTTACCATGATAGATCTCCTTCGTGTTTCCAGTGGCAAGGAATGGATCTTTCAGCAGT
TP15385_Query	D	1	chr3	.	CAGCAGGTTACCATGATAGATCTCCTTCGTATTTCCAGTGGCAAGGAATGGATCTTTCAGCAGT
TP1544_Hit	D+G	1	chr3	.	CAGCAGAGAAAAAATCCTAGGAGCAGGCGCATTAAACAGACTGATGAGGAAGATATCGATGACT
TP1544_Query	D+G	1	chr3	.	CAGCAAAGAAAAAATCCTAGGAGCAGGCGCATTAAACAGACTGATGAGGAAGATATCGATGACT
TP15441_Hit	D+G	1	chr3	.	CAGCAGGTTGTACATCACTCATCTTGGTGTACCCCCTTGATATTGCACACACCCGCCTTGCTGA
TP15441_Query	D+G	1	chr3	.	CAGCAGGTTGTACATCACTCATCTTGGTGTACCCCCTTGATATTGCACACACCCGCCGTGCTGA
TP15477_Hit	D	1	chr3	.	CAGCAGGTTTTACAAATATCAGGACTTGGCAAGGGAGGTAAACTCCAGTTATTAAAATACCTGA
TP15477_Query	D	1	chr3	.	CAGCAGGTTTTACAAATATCAGGACTTAGCAAGGGAGGTAAACTCCAGTTATTAAAATACCTGA
TP15570_Hit	D	1	chr3	.	CAGCAGTAAGCACAAGATAACTTGAAAAATAATTGTTTGAACTTGGTATTATAAATTTGAAGAA
TP15570_Query	D	1	chr3	.	CAGCAGTAAGCACAAGATAACTTGAAAAATAATTGTTGGAACTTGGTATTATAAATTTGAAGAA
TP15600_Hit	D	1	chr3	.	CAGCAGTAATTGGAGCAATGCAACCTATTCTGGGACTTTTAGTCTCCAAGATGATAAATACCTT
TP15600_Query	D	1	chr3	.	CAGCAGTAATTGGAGCAATGCAACCTATTCTGGGACTTTTAGTCTCCAAGATGATAAATACATT
TP15604_Hit	D+G	1	chr3	.	CAGCAGTAATTTGGTACGCCACTTCAAAGCATGCAACAACTCATAGTTAAAATAAAACGGTCAA
TP15604_Query	D+G	1	chr3	.	CAGCAGTAATTTGGTACACCACTTCAAAGCATGCAACAACTCATAGTTAAAATAAAACGGTCAA
TP1565_Hit	D	1	chr3	.	CAGCAAAGAAATGTATTAGACTTCATATTCAGCACACTTCAGTTCCTTAAGTGCAAATTTTTTG
TP1565_Query	D	1	chr3	.	CAGCAAAGAAATGTATTAGACTTCATATTCAGCACACTTCAGTTCCCTAAGTGCAAATTTTTTG
TP15892_Hit	D+G	1	chr3	.	CAGCAGTCAATGATCAAAGTATCCAACATGTTAAATGCGGAAAAGGGGTCGACACAACCATCGC
TP15892_Query	D+G	1	chr3	.	CAGCAGTCAATGATCAAAGTATCCAACATGTTAAATGCGGAAAAGGGGTCGACACAACCATCAC
TP16195_Hit	D	1	chr3	.	CAGCAGTGCCATGTTCACCAAATAAGAAAATCCCGCATGGCAGAGCTGTGCAACTTTCACCATA
TP16195_Query	D	1	chr3	.	CAGCAGTGCCATGTTCACCAAATAAGAAAATCCCACATGGCAGAGCTGTGCAACTTTCACCATA
TP16251_Hit	D+G	1	chr3	.	CAGCAGTGGCAAACTATGACTATATCATGGATTGGGAATTTCAAACTGATGGGCTAATCAGATC
TP16251_Query	D+G	1	chr3	.	CAGCAGTGGCAAACTATGACTATATCATGGATTGGGAATTTCAAACCGATGGGCTAATCAGATC
TP16264_Hit	D+G	1	chr3	.	CAGCAGTGTCCCCACCAGAGCCAGATGAATCATAGCCCATCCCAGTCGGAGGACTCCGTGCTGA
TP16264_Query	D+G	1	chr3	.	CAGCAGTGGCCCCACCAGAGCCAGATGAATCATAGCCCATCCCAGTCGGAGGACTCCGTGCTGA
TP16321_Hit	D	1	chr3	.	CAGCAGTGGTGGAAAAGAAAAGTTCTTGATGCTCCATCTGAAGCTGATGCTGGACTAACAGCTG
TP16321_Query	D	1	chr3	.	CAGCAGTGGTGGAAAAGAAAAGTTCTTGATGCTCCATCTGAAGCTGATGCTGGACTAACAGCAG
TP16382_Hit	D+G	1	chr3	.	CAGCAGTGTGCTCTGTAAGCCTTAAAGTTGGTTGTTGAGAGTGCTGATTCCATACCAATAGCTG
TP16382_Query	D+G	1	chr3	.	CAGCAGTGTGCTCTGTAAGCCTCAAAGTTGGTTGTTGAGAGTGCTGATTCCATACCAATAGCTG
TP16453_Hit	D+G	1	chr3	.	CAGCAGTTACACACAAGTTCTGCAATAGAAGAGCGAGCATGAAGGCATCAGTAACCTTATTCTT
TP16453_Query	D+G	1	chr3	.	CAGCAGTTACACACAAGTTCTGCAATAGAAGAGCGAGCATGAAGGCATCAATAACCTTATTCTT
TP16493_Hit	D	1	chr3	.	CAGCAGTTAGTAACCCGATTTTACAGTGTACCCTCTTACCAGATCCACCATCAAAATCCATGAA
TP16493_Query	D	1	chr3	.	CAGCAGTTAGTAACCCGATTTTACAGTGTACCCTCTTACCAGATCCACAATCAAAATCCATGAA
TP166_Hit	D	1	chr3	.	CAGCAAAAACTTAGTTTGCAAGTCATTTTATTATGCAGATGCAAGCACTTATAATGGGCTTACT
TP166_Query	D	1	chr3	.	CAGCAAAAACTTAGTTTGCAAGTCATTTTACTATGCAGATGCAAGCACTTATAATGGGCTTACT
TP1673_Hit	D	1	chr3	.	CAGCAAAGATATCCAAGTCAATATGTGAGTTTGACTACCATCTTCCAGAGCCTTCATTCAGTAG
TP1673_Query	D	1	chr3	.	CAGCAAAGATATCCAAGTCAATATGTGAGTTTGACTACCATCTTCCAAAGCCTTCATTCAGTAG
TP16784_Hit	D	1	chr3	.	CAGCAGTTTAGAATCTTTCTAAGTCAACCAGCGACACAGTCTTCGGTTGTGGGACCTTCGTGTG
TP16784_Query	D	1	chr3	.	CAGCAGTTTAGAAACTTTCTAAGTCAACCAGCGACACAGTCTTCGGTTGTGGGACCTTCGTGTG
TP16836_Hit	D	1	chr3	.	CAGCAGTTTGCAGGATACGCTTCAGATCAACATTACTTACTAAGTTTCGAATAACCCCTACATT
TP16836_Query	D	1	chr3	.	CAGCAGTTTGCAGGATACACTTCAGATCAACATTACTTACTAAGTTTCGAATAACCCCTACATT
TP16881_Hit	D	1	chr3	.	CAGCAGTTTTGGTGGCCACCCAAGAGAGGCAAGAAGAGCTCGATGATCTGCAAAAACTTGTGGC
TP16881_Query	D	1	chr3	.	CAGCAGTTTTGGTGGCCACCCAAGAGAAGCAAGAAGAGCTCGATGATCTGCAAAAACTTGTGGC
TP16977_Hit	D	1	chr3	.	CAGCATAAACAATCATATAATGAAAATCGATTTAAGATATAGAAACTTACAAGTATGTCAGAAG
TP16977_Query	D	1	chr3	.	CAGCATAAACAATCATATAATGAAAATCGATTTAAGATATAGAAACTTACAAGTATATCAGAAG
TP17069_Hit	D	1	chr3	.	CAGCATAACACATATCTCGTTGACTCGTTGCATCCAATCAGAGAAGCACACGCGTGTCTTTTTT
TP17069_Query	D	1	chr3	.	CAGCATAACACATATCTCGTTGACTCGTTGCATCCAATCAGAGAAGCACACGCGAGTCTTTTTT
TP17076_Hit	D+G	1	chr3	.	CAGCATAACAGTACTCTAATGTTTGCCAATATTCATCAACTGCATATCTATTAGACAGAATAAA
TP17076_Query	D+G	1	chr3	.	CAGCATAACAGTACTCTAACGTTTGCCAATATTCATCAACTGCATATCTATTAGACAGAATAAA
TP17226_Hit	D	1	chr3	.	CAGCATAATCAAAAGGGAACTTTTGAATGCTTTTCCAAACATTGGTGCCCAAAGTATGAACCTT
TP17226_Query	D	1	chr3	.	CAGCATAATCAAAAGGGAACTTCTGAATGCTTTTCCAAACATTGGTGCCCAAAGTATGAACCTT

Name	Filter	Nb hit (Mt4.0)	Mt Chr (Mt4.0)	Ms Chr (Li et al., 2014)	Sequence
TP17270_Hit	D	1	chr3	.	CAGCATAATGTACATAAAAATTATTTATGCTTAGCTAAATGGAATACAGTATAGAAACAAGTGA
TP17270_Query	D	1	chr3	.	CAGCATAATGTACATAAAAAATATTTATGCTTAGCTAAATGGAATACAGTATAGAAACAAGTGA
TP17298_Hit	D	1	chr3	.	CAGCATTATTGTTCCAAGAACACCAATGATTGCAGGAACACCTCTTTCCAAAGCAAATTGAACT
TP17298_Query	D	1	chr3	.	CAGCATAATTGTTCCAAGAACACCAATGATTGCAGGAACACCTCTTTCCAAAGCAAATTGAACT
TP17385_Hit	D	1	chr3	.	CAGCATACATACTCTGTATCCAAGTTTGCGGTTATAGGCATTGTTAAATCCATGGCTTCAGAGC
TP17385_Query	D	1	chr3	.	CAGCATACATACGCTGTATCCAAGTTTGCGGTTATAGGCATTGTTAAATCCATGGCTTCAGAGC
TP17392_Hit	D	1	chr3	.	CAGCATACATGGGAAACTTGTTATGAGAACACTCCATCAGGTTTTGTTCAGAAGAAGGCGGTCT
TP17392_Query	D	1	chr3	.	CAGCATACATGGGAAACTTGTTATGAGAACACTCCATCAGGTTTTGTTCAGAAGAAGGCGATCT
TP17429_Hit	D+G	1	chr3	.	CAGCATACCCGTCTTCCTCCGATTCAGCAGGGTGGACATGCCCAAATGCGGCAAGGTCCTAACC
TP17429_Query	D+G	1	chr3	.	CAGCATACCCGTCTTCCTCCGATTCAACAGGGTGGACATGCCCAAATGCGGCAAGGTCCTAACC
TP17512_Hit	D	1	chr3	.	CAGCATACTGAGTTAAATGAGGGGCAGTAATGAGAATTGGTAAGGAAGGCAAAATATTGAGGTA
TP17512_Query	D	1	chr3	.	CAGCATACTGAGTTAAATGAGGGGCAGTAATGAGAATTGGTAAGGAAGGCAAAATATGGAGGTA
TP17514_Hit	D	1	chr3	.	CAGCATACTGATGGATAAAAGATTTGGGGTTTAAAGAAAGTTTTGAAACCCAAGTTGAATCGAT
TP17514_Query	D	1	chr3	.	CAGCATACTGATGGATAAAAGATTTGGGGTTTAAAGAAAGTTTTGAAACCCAAGTTGAATCAAT
TP17635_Hit	D	1	chr3	.	CAGCATAGCGCCACAGATCTTCACCATGAACCTTCTCAAACGAAGGATTGCCATTAACCAAAAC
TP17635_Query	D	1	chr3	.	CAGCATAGCGCCACACATCTTCACCATGAACCTTCTCAAACGAAGGATTGCCATTAACCAAAAC
TP17664_Hit	D	1	chr3	.	CAGCATAGGCTTTGTTGGAAGATACTATGTTGACAATTTCATTCACATAACGTTCTTCAATTGG
TP17664_Query	D	1	chr3	.	CAGCATAGGCTTTGTTGGAAGATACTATGTTAACAATTTCATTCACATAACGTTCTTCAATTGG
TP17709_Hit	D	1	chr3	.	CAGCATAGTGACTTGGATGCACATGTTGTGATGCAGTTCCAGTGACAACAGCATAAAGGGTAGC
TP17709_Query	D	1	chr3	.	CAGCATAGTGACTTGGATGCACATGTTGTGATGCAGTTCCAGTGACAACAGCAAAAAGGGTAGC
TP17962_Hit	D	1	chr3	.	CAGCATATGACTCGCAAAATAGGGAGTTGCCATCGCCGGTGATAGTGTGGGAATGGGGTTTGAC
TP17962_Query	D	1	chr3	.	CAGCATATGACACGCAAAATAGGGAGTTGCCATCGCCGGTGATAGTGTGGGAATGGGGTTTGAC
TP17989_Hit	D+G	1	chr3	.	CAGCATATGCTTCATAAAACTCAACCTGACAATACCCTTTGGATTTCCCACTAGCTTTCTCATC
TP17989_Query	D+G	1	chr3	.	CAGCATATGCCTCATAAAACTCAACCTGACAATACCCTTTGGATTTCCCACTAGCTTTCTCATC
TP17995_Hit	D	1	chr3	.	CAGCATATGCTGTACCATATAAACAAGTAGTCTCCGGGTATACAGTCAGTTGTCCCCGTTTGCT
TP17995_Query	D	1	chr3	.	CAGCATATGCTGTACCATATAAACAAGTAGTCTCCGGGTATACAGTCAGTTGTCCCCGTTCGCT
TP1806_Hit	D	1	chr3	.	CAGCAAAGGAACTATTACCCTTCAATGGATCCCCAAAGAATGGGTGCTCTTCTTCCATCTCAAG
TP1806_Query	D	1	chr3	.	CAGCAAAGGAACTATTACCCTTCAATGGATCCCCAAAGAATGGGTGCTCTTCTTCCATCTCAAA
TP18188_Hit	D+G	1	chr3	.	CAGCATCAAAATTTGTAGTTCTAAGTGCTAGGTAGACCTGTCATTTGGAATGTGCACACATTTT
TP18188_Query	D+G	1	chr3	.	CAGCATCAAAATTTGTAGTTCTAAGTGCTAGGTAGACCTGTCATTTGGAATGCGCACACATTTT
TP18211_Hit	D	1	chr3	.	CAGCATCAAAGCCTCCTTGAACCCAAAACAGGTTTGTGTAGCCGGCATTATATAGTAGTTCACA
TP18211_Query	D	1	chr3	.	CAGCATCAAAGCCTCCTTGAACCCAAAACAGGTTTGTGTAACCGGCATTATATAGTAGTTCACA
TP18396_Hit	D+G	1	chr3	.	CAGCATCGATATCCCCATGATAACAATACAAAGCAATCATCGAATTATACATAGCCGTCGTCGG
TP18396_Query	D+G	1	chr3	.	CAGCATCAATATCCCCATGATAACAATACAAAGCAATCATCGAATTATACATAGCCGTCGTCGG
TP18412_Hit	D	1	chr3	.	CAGCATCAATGCCATCATTCTCCGGTAAAATTCTCCCCTCCCGGTTCCTGTGCTACTAAAAGGC
TP18412_Query	D	1	chr3	.	CAGCATCAATGCCATCATTCTCCGGTAAAATTCACCCCTCCCGGTTCCTGTGCTACTAAAAGGC
TP18413_Hit	D	1	chr3	.	CAGCATCAATGTTACTTTCAGGACCAATGACAAGTGCAGATGGTTTAATAAATCCAACAATATT
TP18413_Query	D	1	chr3	.	CAGCATCAATGCTACTTTCAGGACCAATGACAAGTGCAGATGGTTTAATAAATCCAACAATATT
TP18563_Hit	D+G	1	chr3	.	CTGCATCAGAAGCTGAGACAGAATCATGCTTATTCTCCAAACTGGAAATAGGAACTGACTCATC
TP18563_Query	D+G	1	chr3	.	CAGCATCAGAAGCTGAGACAGAATCATGCTTATTCTCCAAACTGGAAATAGGAACTGACTCATC
TP18606_Hit	D	1	chr3	.	CAGCATCTGCAATCAAACCAATCTCACTAGCAGATGCTTCTGACACCATACCTGAAGCTGAAAA
TP18606_Query	D	1	chr3	.	CAGCATCAGCAATCAAACCAATCTCACTAGCAGATGCTTCTGACACCATACCTGAAGCTGAAAA
TP18655_Hit	D	1	chr3	.	CAGCATCAGGGGACGAAAATGTTATCTTGCCCCACTTTTCTCTATCCTCAACATCCTTTGCCAT
TP18655_Query	D	1	chr3	.	CAGCATCAGGGGACGAAAATGTTATCTTGCCCCACTTTTCTCTATCCTCAACATCCTTCGCCAT
TP18732_Hit	D+G	1	chr3	.	CAGCATCATCACCCTTCAAGCTTGCTCGGTAAGTTGAAGAACCCTTTATCCTGTTAGTTTCACT
TP18732_Query	D+G	1	chr3	.	CAGCATCATCACCCTTCAAGCTTGCTCGGTAAGTTGAAGAACCCTTTATCCTGTTAGCTTCACT
TP18798_Hit	D	1	chr3	.	CAGCATCATTTCAAGAAAATAAATATGAAGAATCTAATGAAAAACATGATGATGAGGACAAGAA
TP18798_Query	D	1	chr3	.	CAGCATCATTACAAGAAAATAAATATGAAGAATCTAATGAAAAACATGATGATGAGGACAAGAA
TP1884_Hit	D	1	chr3	.	CAGCAAAGGTAATAACATGTCTAGTTGATATAGGAGTACGTTTTTCCCACCTCATGTGAATGAA
TP1884_Query	D	1	chr3	.	CAGCAAAGGTAATAACATGTCTAGTTGATATAGGAGCACGTTTTTCCCACCTCATGTGAATGAA
TP18908_Hit	D+G	1	chr3	.	CAGCATCCACCGCAGGATACTCGTCTTCCCCCGATTCAGCAGGGTGGACAACATGGCCAAATGC
TP18908_Query	D+G	1	chr3	.	CAGCATCCACCGCAGGATACTCATCTTCCCCCGATTCAGCAGGGTGGACAACATGGCCAAATGC
TP18920_Hit	D	1	chr3	.	CAGCATCCACTTTCATGTCAGCTAATTTATCGGATTCCTCGACTTTCCGTTTTAGAGACTCGAA
TP18920_Query	D	1	chr3	.	CAGCATCCACTTTCATGTCAGCTAATTTATCGGATTCCTCAACTTTCCGTTTTAGAGACTCGAA

Name	Filter	Nb hit (Mt4.0)	Mt Chr (Mt4.0)	Ms Chr (Li et al., 2014)	Sequence
TP19010_Hit	D	1	chr3	.	CAGCATCCGTTTTCATTCTGAAAAGATCCGAGTAATGATTCAAGACACTCTGAACAACTAGACG
TP19010_Query	D	1	chr3	.	CAGCATCCGTTTTCATTCTGAAAAGATCCGAGTAATGATTCAAGACACTCTGAACAACCAGACG
TP19043_Hit	D	1	chr3	.	CAGCATCCTCCGCGTGGAGGACAGTTGACTCCTTTTGCACACCAGCTAACTTCAAACAAGCTTA
TP19043_Query	D	1	chr3	.	CAGCATCCTCCGCGTGGAGGACAGTTGACTCCTTTTGCACACCAACTAACTTCAAACAAGCTTA
TP19275_Hit	D+G	1	chr3	.	CAGCATCTATGTCCTTCTTCTCATCCTCAATTCCGATTCTTCAACTCTTCTTCTTCATCAGCTT
TP19275_Query	D+G	1	chr3	.	CAGCATCTACGTCCTTCTTCTCATCCTCAATTCCGATTCTTCAACTCTTCTTCTTCATCAGCTT
TP19291_Hit	D	1	chr3	.	CAGCATCTAGGATCAGGAATTGAAGTTTTAGCTGAGTTGGTCCTTTTTTAGTAAATTTTACTTC
TP19291_Query	D	1	chr3	.	CAGCATCTAGGATCAGGAATTGAAGTCTTAGCTGAGTTGGTCCTTTTTTAGTAAATTTTACTTC
TP19299_Hit	D	1	chr3	.	CAGCATCTATAATCATCTACAGGAGTTTATTCAAAGATTATATCTTCAGTGCCAAAGATCACAG
TP19299_Query	D	1	chr3	.	CAGCATCTATAATCATCTACAGGAGTTTATCCAAAGATTATATCTTCAGTGCCAAAGATCACAG
TP19362_Hit	D+G	1	chr3	.	CAGCATCTCCTAAGTCGCAACCTGTAACCACACGATCATCTCAGAAAGTGAGAGAGAGGATAAG
TP19362_Query	D+G	1	chr3	.	CAGCATCTCCTAAGTCACAACCTGTAACCACACGATCATCTCAGAAAGTGAGAGAGAGGATAAG
TP19454_Hit	D+G	1	chr3	.	CAGCATCTGGATCTCATACTGGTCTCAGGTGAGATTTAGACATGCTAATTTATCAAACTAGAAG
TP19454_Query	D+G	1	chr3	.	CAGCATCTGGATCTCATACTGGTCTCAGGTGAGATTTAGACATGCTAATTTATCAAACTAGAAA
TP19481_Hit	D	1	chr3	.	CAGCATCTGTCGACTTCAAAAATTTCTTCCAACCATGTACAACTCTGCTTATTTTAACTTTCGA
TP19481_Query	D	1	chr3	.	CAGCATCTGTCGACTTCAAAAATTTCCTCCAACCATGTACAACTCTGCTTATTTTAACTTTCGA
TP19651_Hit	D+G	1	chr3	.	CAGCATGAAAAATTCTGCCAGCATCTAGGTGACCATAATCCTTCAAGCCAACAGCCTCAGCAGA
TP19651_Query	D+G	1	chr3	.	CAGCATGAAAAATTCTGCCAGAATCTAGGTGACCATAATCCTTCAAGCCAACAGCCTCAGCAGA
TP2010_Hit	D+G	1	chr3	.	CAGCAAATAAAATCAATGTATGCATAAACTACTACTATTTTATTGCTATGTCACTCACTGCTTC
TP2010_Query	D+G	1	chr3	.	CAGCAAATAAAATCAATCTATGCATAAACTACTACTATTTTATTGCTATGTCACTCACTGCTTC
TP20350_Hit	D+G	1	chr3	.	CAGCATGGAGTGGATGTTAATGCAATGGATAGGACTCTGCTTCAATCTTCAAAGCCATTTCTTC
TP20350_Query	D+G	1	chr3	.	CAGCATGGAGTGGATGTTAATGCAATAGATAGGACTCTGCTTCAATCTTCAAAGCCATTTCTTC
TP20375_Hit	D	1	chr3	.	CAGCATGGCAACATGTGCTCTCCACAATCTGCTTGTATCACATTAAATTTGCACAAGTTAGCTA
TP20375_Query	D	1	chr3	.	CAGCATGGCAACATGTGCTCTCCACAATCTGCTTGCATCACATTAAATTTGCACAAGTTAGCTA
TP20430_Hit	D	1	chr3	.	CAGCATGGGTCCTTGTCAATGTGAAAACAGTGGTGCCTTTTACTTTTATCTAGGCTAGTAGTTT
TP20430_Query	D	1	chr3	.	CAGCATGGGCCCTTGTCAATGTGAAAACAGTGGTGCCTTTTACTTTTATCTAGGCTAGTAGTTT
TP20577_Hit	D	1	chr3	.	CAGCATGTACGCCACAGATGAATTTCGCATGTATTCGTTTAAGATCCTTCTTTGTCATCGTGCA
TP20577_Query	D	1	chr3	.	CAGCATGTACGCCACAGATGAATTTCGCATGTATTCGTTCAAGATCCTTCTTTGTCATCGTGCA
TP2058_Hit	D	1	chr3	.	CAGCAAATAATGTACCAACATGGTTGTAGGTATAAAACCAATTTTGAGGTAAGCTTTTCAGTAA
TP2058_Query	D	1	chr3	.	CAGCAAATAATGTACCAACATGGTTGTAGGTATAAAACCAATTTTGAGGTAAGCTCTTCAGTAA
TP20686_Hit	D+G	1	chr3	.	CAGCTTGTGAAGAATTCTTTTAAATTACCACCATATATTTCATCTTTAGCATCATTTTGGTATG
TP20686_Query	D+G	1	chr3	.	CAGCATGTGAAGAATTCTTTTAAATTACCACCATATATTTCATCTTTAGCATCATTTTGGTATG
TP20754_Hit	D	1	chr3	.	CAGCATGTGTACGATACACTTTACTTCATTGTGGATCTGCATGCGGTATGTCCATTAAATTCCT
TP20754_Query	D	1	chr3	.	CAGCATGTGTACGATACACTTTACTTCATTGTGGATCTACATGCGGTATGTCCATTAAATTCCT
TP20772_Hit	D	1	chr3	.	CAGCATGTGTTTAGATGGCTGGGGTTGGTGATTGTTTTTCCTCTCAATTTGTTTATTCTCTGTG
TP20772_Query	D	1	chr3	.	CAGCATGTGTTTAGATGGCTGGGGTTGGTGATTGTTTTTCCTCTCAATTTATTTATTCTCTGTG
TP20942_Hit	D+G	1	chr3	.	CAGCATTAACTTAGCTTTTTGATGGTTTTTGCTGTTTTGGATTATGCTGTTTGGTGTTTCTGGG
TP20942_Query	D+G	1	chr3	.	CAGCATTAACTTAGCTTTTTGATGGTTTTTGCTGTTTTGGATTATGCTGTTAGGTGTTTCTGGG
TP20978_Hit	D	1	chr3	.	CAGCATTAATGTTAAAATATAAAGTACCATAAGTGCAGAGGAATTGGCTGTCAATGATTCGATA
TP20978_Query	D	1	chr3	.	CAGCATTAATGTTAAAATATAAAGTACCATAAGTGCAGAGGAATTGGCGGTCAATGATTCGATA
TP20987_Hit	D	1	chr3	.	CAGCATTAATTTTCAGACATAAAGTACCATAAGGGCAAAGAAATTGATGGTCAATGATTCGATA
TP20987_Query	D	1	chr3	.	CAGCATTAATTTTCAGACATAAAGTACCATAAGAGCAAAGAAATTGATGGTCAATGATTCGATA
TP21120_Hit	D	1	chr3	.	CAGCATTGGGACCGTATTCTCAAGCAATCAAAGCCAACAACCTTCTATTTGTCTCAGGTGTCCT
TP21120_Query	D	1	chr3	.	CAGCATTAGGACCGTATTCTCAAGCAATCAAAGCCAACAACCTTCTATTTGTCTCAGGTGTCCT
TP21268_Hit	D	1	chr3	.	CAGCATTCAAATCTGACGTTTGTGAAGGGTATAGATTCAAAGCAGTACTTGACTTTTGGAAACT
TP21268_Query	D	1	chr3	.	CAGCATTCAAATCTGACATTTGTGAAGGGTATAGATTCAAAGCAGTACTTGACTTTTGGAAACT
TP2145_Hit	D	1	chr3	.	CAGCAAATATTGTAATAGTACCAAAGGGAATAGAGGTGAAAATGAAAGAACTAACGGTATTTAT
TP2145_Query	D	1	chr3	.	CAGCAAATATTGTAATAGTACCAAAGGGAATAGACGTGAAAATGAAAGAACTAACGGTATTTAT
TP2171_Hit	D	1	chr3	.	CAGCAAATCAGAAGACCTTTCCACCGTTGAGAAGGCTGATAAAACATTAGAGAATTTGGAGAAA
TP2171_Query	D	1	chr3	.	CAGCAAATCAGAAGACCTTTCCACCGTTGAGAAGGCTGATAAAACATTAGAGAATCTGGAGAAA
TP21710_Hit	D	1	chr3	.	CAGCATTGCATCGGAGCTATGTGGTTTCATCACTGTTTTATCTGGAACAACTGTATTGCACAGT
TP21710_Query	D	1	chr3	.	CAGCATTGCATCGGAGCTATGTGGTTTCATCACTGTTTTATCCGGAACAACTGTATTGCACAGT
TP21910_Hit	D+G	1	chr3	.	CAGCATTGTGCTACTGAGTCGTTTCTTATTTAATAACTGGATATACATATTTTTGTAGCCTGTG
TP21910_Query	D+G	1	chr3	.	CAGCATTGTGCTACTGAGTCGTTTCTTATTTAGTAACTGGATATACATATTTTTGTAGCCTGTG

Name	Filter	Nb hit (Mt4.0)	Mt Chr (Mt4.0)	Ms Chr (Li et al., 2014)	Sequence
TP22077_Hit	D	1	chr3	.	CAGCATTTCAACAAGTGAGATTCGGTTCTCTAAGATTGGGATCAATCAATTTGATGCTGACATG
TP22077_Query	D	1	chr3	.	CAGCATTTCAACAAGTGAGATTCGGTTCTCTAAGATTGGGATCAATAAATTTGATGCTGACATG
TP22168_Hit	D	1	chr3	.	CAGCATTTCTGAATACTTGATTGAAACAATACCTGGTTACTGTTTTGAAGACTTTCTTGATGCT
TP22168_Query	D	1	chr3	.	CAGCATTTCTGAATACCTGATTGAAACAATACCTGGTTACTGTTTTGAAGACTTTCTTGATGCT
TP22682_Hit	D	1	chr3	.	CAGCCAAAACAGATGCTTCTTCATCATTGGTGGATTTATATTCCTGAATATATACATGAAATAA
TP22682_Query	D	1	chr3	.	CAGCCAAAACAGATGCTTCTTCATCATAGGTGGATTTATATTCCTGAATATATACATGAAATAA
TP22702_Hit	D	1	chr3	.	CAGCCAAAACTAACACATCAACTACTTTGTCAAAAATAGCATTTGCCACACCAAAACAAAATAA
TP22702_Query	D	1	chr3	.	CAGCCAAAACTAACACATCAACTACTTTGTCAAAAATAGCATTCGCCACACCAAAACAAAATAA
TP22725_Hit	D+G	1	chr3	.	CAGCCAAAAGAATACTGAGATATGTGAAAGGAACTCTGAAGCTTGGTTTACTATTTCCAACTAC
TP22725_Query	D+G	1	chr3	.	CAGCCAAAAGAATACTGAGATATGTGAAAGGAACTCTGAAGCTTGGTTTACTATTTCAAACTAC
TP22773_Hit	D+G	1	chr3	.	CAGCCAAAATAAGAGTAGAAGAGAGATAAAATAAGGAGAAATGAGGTTATCTATACCTGAAGGG
TP22773_Query	D+G	1	chr3	.	CAGCCAAAATAAGAGTAGAAGAGAGATAAAATAAGGAGAAATGAGGTTATCTATACCTGAAGGA
TP22787_Hit	D	1	chr3	.	CAGCCAAAATCAAAATCGCTAAACCAAAACCAGTAAAATGAACATAAACTAGCAACTTAAAATT
TP22787_Query	D	1	chr3	.	CAGCCAAAATCAAAATCACTAAACCAAAACCAGTAAAATGAACATAAACTAGCAACTTAAAATT
TP22805_Hit	D	1	chr3	.	CAGCCAAAATCTGTATGTTCACATTATGGAGAAAGAATTAGAGCTCCTTTAGGATTGGAGTGCA
TP22805_Query	D	1	chr3	.	CAGCCAAAATCTGTATGTTCACATTATAGAGAAAGAATTAGAGCTCCTTTAGGATTGGAGTGCA
TP22999_Hit	D+G	1	chr3	.	CAGCCAAAGCATAACTACAATAAACAAATAAACGAACCAAGCCGAGGCCTAAACCAATAGCCAA
TP22999_Query	D+G	1	chr3	.	CAGCCAAAGCATAACTACAATAAACAAATAAACGAACCAAGCCAAGGCCTAAACCAATAGCCAA
TP23010_Hit	D	1	chr3	.	CAGCCAAAGCGCATATCAGCTTCATGACATCAGGCAACACAAAAGCAACCTTGGACGGCTCGTG
TP23010_Query	D	1	chr3	.	CAGCCAAAGCGCATATCAGCTTCATGACATCAGGCAAAACAAAAGCAACCTTGGACGGCTCGTG
TP23022_Hit	D+G	1	chr3	.	CTGCCAAAGGACACTCCGTTAATTCACAACGCTAAGGCTAGAGAATAGCGGTCAGCCGCTGAAA
TP23022_Query	D+G	1	chr3	.	CAGCCAAAGGACACTCCGTTAATTCACAACGCTAAGGCTAGAGAATAGCGGTCAGCCGCTGAAA
TP23282_Hit	D+G	1	chr3	.	CAGCTAACACACTACTTCCCATTTCTGATTTAGCTGTTTTTGGTTCCTTGGACTGGTTAAGAAC
TP23282_Query	D+G	1	chr3	.	CAGCCAACACACTACTTCCCATTTCTGATTTAGCTGTTTTTGGTTCCTTGGACTGGTTAAGAAC
TP23343_Hit	D	1	chr3	.	CAGCCAACATCATCTGGATGTCAGCTTCTTTTTGAGAGAGCTGACGGGATGAAAGTTGTTCGGT
TP23343_Query	D	1	chr3	.	CAGCCAACATCATCTGGATGTCAGCTTCCTTTTGAGAGAGCTGACGGGATGAAAGTTGTTCGGT
TP23363_Hit	D	1	chr3	.	CAGCCAACGTTGTCTCAGGAAGGATGGCTCTCAACAATGTGGCTTTCCTGCTGAAAAAAAAAAA
TP23363_Query	D	1	chr3	.	CAGCCAACATTGTCTCAGGAAGGATGGCTCTCAACAATGTGGCTTTCCTGCTGAAAAAAAAAAA
TP23462_Hit	D	1	chr3	.	CAGCCAACTAAGAAATTTTCTCCCTCTCCCCACACTGACAGTACCTCCTTCCTTTCCTCATTAC
TP23462_Query	D	1	chr3	.	CAGCCAACTAAGAAATTTTCTCCCTCTCCCCACACTGACAGTACCTCCCTCCTTTCCTCATTAC
TP2376_Hit	D	1	chr3	.	CAGCAAATGGGCCTTGTAACCCAGGAGCCTGTGTTGTTCAATGACACCGTGCGAGCCAACATTG
TP2376_Query	D	1	chr3	.	CAGCAAATGGGCCTGGTAACCCAGGAGCCTGTGTTGTTCAATGACACCGTGCGAGCCAACATTG
TP23783_Hit	D	1	chr3	.	CAGCCCAGTGTAAAATGGTGTTTTCATTGTGATCCACTCCTAGACTTAAGCTATAAAAGATTTC
TP23783_Query	D	1	chr3	.	CAGCCAAGTGTAAAATGGTGTTTTCATTGTGATCCACTCCTAGACTTAAGCTATAAAAGATTTC
TP23839_Hit	D	1	chr3	.	CAGCCAATAATAACTATTTTATTTTCCATTCTCTGAAGCAAAAAGTGTTAATTAAAAGTTAGGC
TP23839_Query	D	1	chr3	.	CAGCCAATAATAACTATTTTATTTTCCATTCTTTGAAGCAAAAAGTGTTAATTAAAAGTTAGGC
TP23984_Hit	D	1	chr3	.	CAGCCAATGACTGAATTATAAGCGCGAATATGAGTCCAATAAGTACCAACCACAGCAACTGAAT
TP23984_Query	D	1	chr3	.	CAGCCAATGACTGAATTATAAGCGCGAATATGAGTCCAATAAGTACCAACCACAACAACTGAAT
TP24128_Hit	D	1	chr3	.	CAGCCAATTGTTAGCATTATGTAATAATAACAACTTGTTGTGTTTATGATTTTGGGTTGTGACA
TP24128_Query	D	1	chr3	.	CAGCCAATTGTTAGCATTATGTAATAATAACAACTTGATGTGTTTATGATTTTGGGTTGTGACA
TP24567_Hit	D	1	chr3	.	CAGCCACATCAATTCTACATGTGCACGGACATGAGACTGGACAAAAACTGAAGCCTGTTTGCAG
TP24567_Query	D	1	chr3	.	CAGCCACATCAATTCTACATGTGCACGGACATGAGACTGAACAAAAACTGAAGCCTGTTTGCAG
TP24784_Hit	D	1	chr3	.	CAGCCACCATATTTCCAAGGCTGACTCAAGAAGGCTCGAAAAATGCACACAACAGGAAAGTTTA
TP24784_Query	D	1	chr3	.	CAGCCACCATATTTCCAAGGCCGACTCAAGAAGGCTCGAAAAATGCACACAACAGGAAAGTTTA
TP25021_Hit	D	1	chr3	.	CAGCCACGACCTTGCAATTGGAATGATGGAATAATAGAACAGCCACGGCAAACACCTGTTAACA
TP25021_Query	D	1	chr3	.	CAGCCACGACCTTGCAATTGGAATGATGGAATAATAGAACAGCCACGACAAACACCTGTTAACA
TP25049_Hit	D	1	chr3	.	CAGCCATGATTGCTTAAGTTTATGGAGGATTCTCGAACACAATTGTTAGAACAGCAACTAGTAA
TP25049_Query	D	1	chr3	.	CAGCCACGATTGCTTAAGTTTATGGAGGATTCTCGAACACAATTGTTAGAACAGCAACTAGTAA
TP25052_Hit	D	1	chr3	.	CAGCCACGCAAAGGCTTCAATTAATTTATTTGTGAATAATGAATATAGATGTATCCGAGGGGGT
TP25052_Query	D	1	chr3	.	CAGCCACGCAAAGGCTTCAATTAATTTATTTGTGAATAATGAATATAGATATATCCGAGGGGGT
TP25078_Hit	D	1	chr3	.	CAGCCACGGACAGAGGCTCTTCAACTTTCTGTTTGTTTTTACTATTCCTGCTATTCTTCCTTTT
TP25078_Query	D	1	chr3	.	CAGCCACGGACAAAGGCTCTTCAACTTTCTGTTTGTTTTTACTATTCCTGCTATTCTTCCTTTT
TP25095_Hit	D	1	chr3	.	CAGCCACGGGTTAAATATATCAGATACCGGTTGGAAAGGCTCGAGAAATAAATAAAAGCAATGG
TP25095_Query	D	1	chr3	.	CAGCCACGGGTTAAACATATCAGATACCGGTTGGAAAGGCTCGAGAAATAAATAAAAGCAATGG

Name	Filter	Nb hit (Mt4.0)	Mt Chr (Mt4.0)	Ms Chr (Li et al., 2014)	Sequence
TP25180_Hit	D+G	1	chr3	.	CAGCCACTGCAGATTCTCTTAGCTTCTTTGACAGGCGCCGGAGAAGGAGATTCTGCGGAATTCG
TP25180_Query	D+G	1	chr3	.	CAGCCACTACAGATTCTCTTAGCTTCTTTGACAGGCGCCGGAGAAGGAGATTCTGCGGAATTCG
TP25218_Hit	D	1	chr3	.	CAGCGACTATATTTGGAATCATATAAACTGCAACAGCCCAATAATAAATTGACTGGGTCCCCCA
TP25218_Query	D	1	chr3	.	CAGCCACTATATTTGGAATCATATAAACTGCAACAGCCCAATAATAAATTGACTGGGTCCCCCA
TP25228_Hit	D	1	chr3	.	CAGCCACTATGCATGTCAACTTTGTGATCATCTTTCAGGTGATCCACCAAGAATTGTACATCAC
TP25228_Query	D	1	chr3	.	CAGCCACTATGCATGTCAACTTTGTGATCATCTTTCAGGTGATCCACCAAGAACTGTACATCAC
TP25247_Hit	D	1	chr3	.	CAGCCACTCCAAGGTACATATTTTTCTCCATTACTGGATATAGAAAAAACTACTTGATCTTGTA
TP25247_Query	D	1	chr3	.	CAGCCACTCCAAGGTACATATTTTTCTCCATTACTGGATATAGAAAAAACTACTCGATCTTGTA
TP25341_Hit	D	1	chr3	.	CAGCCACTGGAAGAGTGACAACCAAAGTAGATGTTTACGCTTTTGGAGTAGTTCTGATGGAACT
TP25341_Query	D	1	chr3	.	CAGCCACTGGAAGAGTGACAACCAAAGTAGATGTTTACGCATTTGGAGTAGTTCTGATGGAACT
TP25346_Hit	D	1	chr3	.	CAGCCACTGGATTTCTGTAAGTGTCACGCCTTGTCTCATCAACCGTGTACTGCTTCTTTCCGTA
TP25346_Query	D	1	chr3	.	CAGCCACTGGATTTCTGTAAGTGTCACGCCTTGTCTCATCAACCGTGTACTGCTTCTTTCCATA
TP25347_Hit	D+G	1	chr3	.	CAGCCACTGGCACTTTTTTCACTCTCATGCTTCTAGGGCTTAAGTACTGCTCCTGCCAGTTTCA
TP25347_Query	D+G	1	chr3	.	CAGCCACTGGCACTTTTTTCACTCTCATGCTTCTAGGGCTCAAGTACTGCTCCTGCCAGTTTCA
TP254_Hit	D	1	chr3	.	CAGCGAAAATCCAGATATTTGCAGGAGACGTGGTTCCCCGAAAAAAGCCTGATCCAGTAAGATT
TP254_Query	D	1	chr3	.	CAGCAAAAATCCAGATATTTGCAGGAGACGTGGTTCCCCGAAAAAAGCCTGATCCAGTAAGATT
TP25547_Hit	D	1	chr3	.	CAGCCAGAGTATATGTTTGCTCTGACATATAAAGTTACCCGGGGTTTTATTACAGGAGTAGACG
TP25547_Query	D	1	chr3	.	CAGCCAGAATATATGTTTGCTCTGACATATAAAGTTACCCGGGGTTTTATTACAGGAGTAGACG
TP25551_Hit	D	1	chr3	.	CAGCCAGAATCCAGCAGAAAATACCAAACTTTAAACATGTTTTCCGAACTCGTTTCAAATCCGA
TP25551_Query	D	1	chr3	.	CAGCCAGAATCCAGCAGAAAATACCAAACTTTAAACATGTTTTCCAAACTCGTTTCAAATCCGA
TP2557_Hit	D	1	chr3	.	CAGCAAATTTACAAAACAAATTTTCATGTGGTCTAGCACTACCAGTACTGGATTCACCGACATC
TP2557_Query	D	1	chr3	.	CAGCAAATTTACAAAACAAATTTTCATGTGGTCTAGCACTACCAGTACTGGATTCACCAACATC
TP25571_Hit	D+G	1	chr3	.	CAGCCAGACCAAAAGCTAAGAACAACACACTCACAATCCCTGCAGTCTCTGCGTGCTGAAAAAA
TP25571_Query	D+G	1	chr3	.	CAGCCAGACCAAAAGCTAAGAACAACACACTCACAATCCCTGCAGTCTCTGCATGCTGAAAAAA
TP25609_Hit	D+G	1	chr3	.	CAGCCAGAGAGGAAGACCAGCTTCTTGGAAAATATCTGGAACAAAAATCTAATGATAGTCAGCA
TP25609_Query	D+G	1	chr3	.	CAGCCAGAGAGGAAGACCAGCTTCTTGGAAAATATCTGGAACAAAAATCTAACGATAGTCAGCA
TP25717_Hit	D+G	1	chr3	.	CAGCCAGCAAACTTTGCTCCGGACTTTGCGCTGTCGTTATCCGAGCTTGCTGAAAAAAAAAAAA
TP25717_Query	D+G	1	chr3	.	CAGCCAGCAAACTTTGCTCCGGACTTTGCGCTGTCGTTATCCGAGCGTGCTGAAAAAAAAAAAA
TP25740_Hit	D	1	chr3	.	CAGCCAGCAATGAAAAATGCAACTCAATTTGAGAGCTACTTCTTAGCAATCCCTAACAAAGAGA
TP25740_Query	D	1	chr3	.	CAGCCAGCAATGAAAAATGCAACTCAATTTGAGAGCTACTTCTTAGCAATCCCAAACAAAGAGA
TP25776_Hit	D	1	chr3	.	CAGCCAGCATAAATGCACAATTGCACATGTATATAAATATGCTGATAGATTGGAACACTAAATT
TP25776_Query	D	1	chr3	.	CAGCCAGCATAAATGCACAATTGCACATGTATATAAATATGCTGAAAGATTGGAACACTAAATT
TP25842_Hit	D	1	chr3	.	CAGCCAGCCTTCTTGCCCAGGTCTTGGGCTAGGCTAGGAGATCGGAAGCCTCAATTCAAGTCGT
TP25842_Query	D	1	chr3	.	CAGCCAGCCTTCTTGCCCAGGTCTTGGGCTAGGCTAGGAGATCGGAAGCCTCAATTCAAGTCGG
TP25905_Hit	D	1	chr3	.	CAGCCAGGAATTGCTATTGGACGATTATGTGAGAAATGTGACGGCAAGTGTGTGATATGCGACT
TP25905_Query	D	1	chr3	.	CAGCCAGGAATTGCTATTGGACGATTATGCGAGAAATGTGACGGCAAGTGTGTGATATGCGACT
TP25972_Hit	D	1	chr3	.	CAGCTAGGGACTTTAATTTGTCACGATCCATGCACAGTTGTGACACACTTTGGATGCACTTCAT
TP25972_Query	D	1	chr3	.	CAGCCAGGGACTTTAATTTGTCACGATCCATGCACAGTTGTGACACACTTTGGATGCACTTCAT
TP25977_Hit	D	1	chr3	.	CAGCTAGGGCACTCAAAACAGTACTTCAAGGAGTCTTACTATCTTCTGAAGGGTAATGATCTCA
TP25977_Query	D	1	chr3	.	CAGCCAGGGCACTCAAAACAGTACTTCAAGGAGTCTTACTATCTTCTGAAGGGTAATGATCTCA
TP25987_Hit	D	1	chr3	.	CAGCCAGGGTAAGAATATCAGCACATGACACAACTCCTGGGCATGCTTTTTCCACATTGGCTTT
TP25987_Query	D	1	chr3	.	CAGCCAGGGTAAGAATATCAGCACAGGACACAACTCCTGGGCATGCTTTTTCCACATTGGCTTT
TP26000_Hit	D+G	1	chr3	.	CAGCCAGGTAGCGTGTCTGTAGGATTTCCAAGTGTGCAAAGCTTAACAGATGTTATTGAGATGG
TP26000_Query	D+G	1	chr3	.	CAGCCAGGTAGCGTGTCTGTAGGATTTCCAACTGTGCAAAGCTTAACAGATGTTATTGAGATGG
TP26113_Hit	D	1	chr3	.	CAGCCAGTGAGAAAGGTGCTTGGTCAGAACTCAGGCGTAAAAAATACTGCTGGTACAGATTCAG
TP26113_Query	D	1	chr3	.	CAGCCAGTGAGAAAGGTGCTTGGTCAGAACTCAGGCGTAAAAAAAACTGCTGGTACAGATTCAG
TP26131_Hit	D	1	chr3	.	CAGCCAGTGCCGAAGTGAATATGTATTGTGTGTATGACAGTCAAATATTGACATTATTTACGTC
TP26131_Query	D	1	chr3	.	CAGCCAGTGCCGAAGTGAATATGTATTGTGTCTATGACAGTCAAATATTGACATTATTTACGTC
TP26289_Hit	D	1	chr3	.	CAGCCATAATGCTCCAACCGAGGCAGGACACCGTATTCATTGCTCATAGACTCGAAGCATTGAG
TP26289_Query	D	1	chr3	.	CAGCCATAATGCTCCAACCGAGGCAGGACACCGTATTCATTGCTCATAGACTCGAAACATTGAG
TP26363_Hit	D	1	chr3	.	CAGCCATACTATTAGAAGAAGATGCCTCCACACTAGGTAAGGGGGCGGATGGAGGTACTGTGTC
TP26363_Query	D	1	chr3	.	CAGCCATACTATTAGAAGAAGATGCCTCCACACTAGATAAGGGGGCGGATGGAGGTACTGTGTC
TP26395_Hit	D	1	chr3	.	CAGCCATAGCGGTTTTATACCGCTATAGCATAATGGAATTTGGAGAAACCATATTGTTCCATAA
TP26395_Query	D	1	chr3	.	CAGCCATAGCGGTTTTATACCGCTATAGCATAATGGAATTTGGAGAAACCACATTGTTCCATAA

Name	Filter	Nb hit (Mt4.0)	Mt Chr (Mt4.0)	Ms Chr (Li et al., 2014)	Sequence
TP26667_Hit	D	1	chr3	.	CAGCCATCCGTTCCTTATTTCATGCTACACTACCCATCAATAAACTTGATCCACACCTCTAATT
TP26667_Query	D	1	chr3	.	CAGCCATCCGTTCCTTATTTCATGCTACACTACCCAGCAATAAACTTGATCCACACCTCTAATT
TP26692_Hit	D	1	chr3	.	CAGCCATCGCCATGGGCATTGCTCTCCTTAGGATGGTTCCTAATCGACCTCTACTATCATGTGC
TP26692_Query	D	1	chr3	.	CAGCCATCGCCATGGGCATTGCTCTCCTTAGGATGGTTCCTAATCGACCTCTACTATCAGGTGC
TP26733_Hit	D	1	chr3	.	CAGCTATCTCCATTCAGAAGTATATTCGTATGTGTCAGATGAGGCGTGCTTACATGACACTGTA
TP26733_Query	D	1	chr3	.	CAGCCATCTCCATTCAGAAGTATATTCGTATGTGTCAGATGAGGCGTGCTTACATGACACTGTA
TP26767_Hit	D	1	chr3	.	CAGCCATCTTTTCCCTGTTGGATGCAAGAGACCCACGTCTGAGCTAAAGGCAATTTGTGCATTT
TP26767_Query	D	1	chr3	.	CAGCCATCTTTTCCCTGTTGGATGCAAGAAACCCACGTCTGAGCTAAAGGCAATTTGTGCATTT
TP26825_Hit	D	1	chr3	.	CAGCCATGAATTGAAGGTTCATACTTTGGGCACCAATGTTTGGAAAAGCATTCAGAAGTTCCCT
TP26825_Query	D	1	chr3	.	CAGCCATGAATTGAAGGTTCATACTTTGGGCACCAATGTTTGGAAAAGCATTCAAAAGTTCCCT
TP27127_Hit	D+G	1	chr3	.	CAGCCATGTAGTCTCCAATAGCTTTAGTAGGGGAAGCAAAAGGGCTATCAGGGAGATTATGCTC
TP27127_Query	D+G	1	chr3	.	CAGCCATGTAGTCACCAATAGCTTTAGTAGGGGAAGCAAAAGGGCTATCAGGGAGATTATGCTC
TP27168_Hit	D	1	chr3	.	CAGCCATGTGTAAACTGGTGTTTTCATTGTTATCCACTTCTAGACTTAAGCTCTGAAAGGTTTC
TP27168_Query	D	1	chr3	.	CAGCCATGTGTAAAATGGTGTTTTCATTGTTATCCACTTCTAGACTTAAGCTCTGAAAGGTTTC
TP27247_Hit	D+G	1	chr3	.	CAGCCATTACCAACATTCTACCATGGAAAAACAAATGAAAGATAGCATTGTGCTTAGATCCATA
TP27247_Query	D+G	1	chr3	.	CAGCCATTACCAACATTCTACCATGGAAAAACAAATGAAAGATAGCATTGTGCTTAGATCCACA
TP2728_Hit	D+G	1	chr3	.	CAGCAACAAACACAAGGTATTGTTGATGCAAATGTTGCTGTTCAGGTACAAGGCATTGTGAAGA
TP2728_Query	D+G	1	chr3	.	CAGCAACAAACACAAGGTATTGTTGACGCAAATGTTGCTGTTCAGGTACAAGGCATTGTGAAGA
TP27490_Hit	D	1	chr3	.	CAGCCATTGTTGAAGTGTTGCTAGTGTCACCCAATTCTAATTTTCTATCGCGGGAGCGCCAACC
TP27490_Query	D	1	chr3	.	CAGCCATTGTTGAAGTGTTGCTAGTGTCACCCAATTCTAATTTTCTATCACGGGAGCGCCAACC
TP27507_Hit	D	1	chr3	.	CAGCCATTTACATGAGAACATGAGTGAGATGGATGATAAAAAGCAAGACCCCCAGAAGATTTAC
TP27507_Query	D	1	chr3	.	CAGCCATTTACATGAGAACATGAGTGAGAGGGATGATAAAAAGCAAGACCCCCAGAAGATTTAC
TP27522_Hit	D	1	chr3	.	CAGCCATTTCAAATTTTTGAAATAAGCCGGATCAATAGCTTCAGTATCATGATATGTAACTTTG
TP27522_Query	D	1	chr3	.	CAGCCATTTCAAATTTTTGAAATAAGCCGGATCAATAGCTTCAATATCATGATATGTAACTTTG
TP28019_Hit	D	1	chr3	.	CAGCCCACTGGATGTCTGTAGGTTGGTTGCCTGTAGTAAGGAGTGATTGTGATGCTTATTTTGA
TP28019_Query	D	1	chr3	.	CAGCCCACTGGATGTCTGTAGGTTGGTTGCCTGTAGTAAAGAGTGATTGTGATGCTTATTTTGA
TP2832_Hit	D	1	chr3	.	CAGCAACTACAAAAGGCTGATTCCACTATCATGATATGGAAAGCTTTGAAGAAAACAGTATACA
TP2832_Query	D	1	chr3	.	CAGCAACAACAAAAGGCTGATTCCACTATCATGATATGGAAAGCTTTGAAGAAAACAGTATACA
TP28402_Hit	D+G	1	chr3	.	CAGCCCCAAGATGACGCAAAGCTACCTCCTCAAGACGGCCATCACGATATGCGTGCTGAAAAAA
TP28402_Query	D+G	1	chr3	.	CAGCCCCAAGATGACGCAAAGCTACCTCCTCAAGACGGCCATCACGATATGCATGCTGAAAAAA
TP28425_Hit	D	1	chr3	.	CAGCCCCAATGCTTCTCCAATATCAACTGAACATAACGGTGTGTGCCACATTCATTGTTTTTGC
TP28425_Query	D	1	chr3	.	CAGCCCCAATGCTTCTCCAATATCAACCGAACATAACGGTGTGTGCCACATTCATTGTTTTTGC
TP28578_Hit	D	1	chr3	.	CAGCCCCCACTCCAAGATATGTGGTGCCCTTACTCAGATTATGACATTTTATTGGATTTATCCA
TP28578_Query	D	1	chr3	.	CAGCCCCCACTCCAAGATATGTGGTGCCCTTACTCAGATTATGACACTTTATTGGATTTATCCA
TP28620_Hit	D	1	chr3	.	CAGCCCCCGCACTCTGTGTTCCTTTATAGAATCCTGTGTACCTGTGTATGTGTAAAAATCAGTA
TP28620_Query	D	1	chr3	.	CAGCCCCCGCACTCTGTATTCCTTTATAGAATCCTGTGTACCTGTGTATGTGTAAAAATCAGTA
TP28991_Hit	D	1	chr3	.	CAGCCCGAGGGAAAAGGCGGTTTGTTCAAATTCCGCCACGCAACAGTGCTATAGCTGAAAAAAA
TP28991_Query	D	1	chr3	.	CAGCCCGAGGGAAAAGGCGGTTTGTTCAAATTCCGCCACGCAACAGTGCTATAGCAGAAAAAAA
TP29087_Hit	D	1	chr3	.	CAGCCCGGCAGGCTTTGCAAAATGTAAGCTGAATAGATATACATCCATATATAAATTCAGCTTC
TP29087_Query	D	1	chr3	.	CAGCCCGGCAGGCTTTGCAAAATGTAAGCTGAATAGATATACATCCATATATAAATTAAGCTTC
TP29418_Hit	D+G	1	chr3	.	CAGCCCTATCCCTTCTCTCTTGAACATCTTGTTCATCGCTGGTATCAGGTGCATCTTTCTCGAA
TP29418_Query	D+G	1	chr3	.	CAGCCCTATCCCTTCTCTCTTGAACATCTTGTTCACCGCTGGTATCAGGTGCATCTTTCTCGAA
TP29634_Hit	D	1	chr3	.	CAGCCCTGTAGCGTAGCAGAATTTGAACAAATTGCTATTTTTCGCTATCTCTGATTGATAACAT
TP29634_Query	D	1	chr3	.	CAGCCCTGTAGCGTAGCAAAATTTGAACAAATTGCTATTTTTCGCTATCTCTGATTGATAACAT
TP29824_Hit	D	1	chr3	.	CAGCCCTTTGGATTACTCCCTGTTATTCAAGATGGTGTCTATACTTTCTATGGTTAGCTCCCTC
TP29824_Query	D	1	chr3	.	CAGCCCTTTGGATTACTCCCTGTTATTCAAGATGGTGACTATACTTTCTATGGTTAGCTCCCTC
TP30056_Hit	D+G	1	chr3	.	CAGCCGACGACGGCTATGTATAATTCGATGATTGCTTTGTATTGTTATCATGGGGATATTGGTG
TP30056_Query	D+G	1	chr3	.	CAGCCGACGACGGCTATGTATAATTCGATGATTGCTTTGTATTGTTATCATGGGGATATCGGTG
TP30109_Hit	D+G	1	chr3	.	CAGCCTAGATCACAGACCTTTTTAAAACCTTGGTTGCACCCATGTTGGTATCCTTAATAATCAT
TP30109_Query	D+G	1	chr3	.	CAGCCGAGATCACAGACCTTTTTAAAACCTTGGTTGCACCCATGTTGGTATCCTTAATAATCAT
TP30223_Hit	D	1	chr3	.	CAGCCGATGATATTCACTCCATTCATGCAGGTAGGTCCATCCATCTCGAGAAAACACGGAGGAA
TP30223_Query	D	1	chr3	.	CAGCCGATGATATTCACTCCATTCATGCAGGTTGGTCCATCCATCTCGAGAAAACACGGAGGAA
TP3046_Hit	D	1	chr3	.	CAGCAACAAGAGCTTCTGAGATTGTCAATGCAATATGGAGGTTCTTCTTCCTCTTCTGACCCTT
TP3046_Query	D	1	chr3	.	CAGCAACAAGAGCTTCTGAGATTGTCAATGCAATATGGAGGTTCTTCTTCCTCTTCCGACCCTT

Name	Filter	Nb hit (Mt4.0)	Mt Chr (Mt4.0)	Ms Chr (Li et al., 2014)	Sequence
TP3058_Hit	D	1	chr3	.	CAGCAACAAGATGATGAATTTATGATGTATACATTCAAAGTGCGGCATTGCGGTTATGTTCATG
TP3058_Query	D	1	chr3	.	CAGCAACAAGATGATGAATTTATGATGTATACATTCAAAGTGCGGCATTGCAGTTATGTTCATG
TP30649_Hit	D	1	chr3	.	CAGCCGCGATGAGTCGGAAGAGTTGAAGAGTGAACGTAAGAGTGATCTTAGGGTTCCAATCAAT
TP30649_Query	D	1	chr3	.	CAGCCGCGATGAGTCGGAAGAGTTGAAGAGTGGACGTAAGAGTGATCTTAGGGTTCCAATCAAT
TP30728_Hit	D	1	chr3	.	CAGCCGCTAAAGTAGCTGGGATGAAGTGTATAGTAACAAAGAGCTGGTATATTTCTATTTACTG
TP30728_Query	D	1	chr3	.	CAGCCGCTAAAGTAGCTGGGATGAAGTGTATAGTAACAAAGAGCGGGTATATTTCTATTTACTG
TP30899_Hit	D	1	chr3	.	CAGCCGGACCAGGTAACTTTTTCCGAACTAGTCCACGTAGTCTTTGGCACTCAGCTTCTAACTT
TP30899_Query	D	1	chr3	.	CAGCCGGACCAGGTAACTTTTTCCGAACTAATCCACGTAGTCTTTGGCACTCAGCTTCTAACTT
TP30913_Hit	D+G	1	chr3	.	CAGCCGGAGCCGAAGCAGGAGCAGGAGCAGAAGTCGTAGCCCTGGAATCCGTAAGATTCGCTCT
TP30913_Query	D+G	1	chr3	.	CAGCCGGAGCCGAAGCAGGAGCAGGAGCAGAAGTCGTAGCCCAGGAATCCGTAAGATTCGCTCT
TP30986_Hit	D+G	1	chr3	.	CAGCCGGCATCATTTTGCGGTGTTGTAGGTTTAAAGCCAACATATGGGCGCGTCTCAAGATTTG
TP30986_Query	D+G	1	chr3	.	CAGCCGGCATCATTTTGCGGTGTTGTAGGTTTGAAGCCAACATATGGGCGCGTCTCAAGATTTG
TP31277_Hit	D	1	chr3	.	CAGCCGTGGTGAATCCCATCTTCTCTTCCCCGAAAACCCTTTGGAGGTAAGGATCACAGATGAA
TP31277_Query	D	1	chr3	.	CAGCCGTAGTGAATCCCATCTTCTCTTCCCCGAAAACCCTTTGGAGGTAAGGATCACAGATGAA
TP31285_Hit	D	1	chr3	.	CAGCCGTATCATATGATTATGGTACTAATGACAAGTCATCTGGTTCACACAAGCCACCTATGTT
TP31285_Query	D	1	chr3	.	CAGCCGTATAATATGATTATGGTACTAATGACAAGTCATCTGGTTCACACAAGCCACCTATGTT
TP31611_Hit	D	1	chr3	.	CAGCCGTTTGTGTGTAAACTTATGTGTATATAGATGTTTGTGACATTTTTATTCTATCTCTTTT
TP31611_Query	D	1	chr3	.	CAGCCGTTTGTGTGTAAACTTATGTGTATATAGATGTTTGTGACATTTTTATTCTATCTCTCTT
TP31669_Hit	D	1	chr3	.	CAGCCTAAAGATGAAAAGATAGGCTACTACAGAAACTCGTATACATTACAAATCAATGTCACAT
TP31669_Query	D	1	chr3	.	CAGCCTAAAGATGAAAAGATAGGCTACTACAGGAACTCGTATACATTACAAATCAATGTCACAT
TP31677_Hit	D	1	chr3	.	CAGCCTAAATCTATAAGAAATCTACGGTCTCATGGGAAATAAGCCTGTTATGGTAAAGTTTATA
TP31677_Query	D	1	chr3	.	CAGCCTAAATCTATAAGAAATCTACGGTCTCATGGGAAATAAGCCTGTTATGGTAAACTTTATA
TP31741_Hit	D	1	chr3	.	CAGCCTAAGAGAGAAAGAAATTTAGAGCGATAAAGAGATTAAAAACTGTCCGAGTGGGATGGTA
TP31741_Query	D	1	chr3	.	CAGCCTAAGAGAGAAAGAAATTTAGAGCGATAAAGAGATTAAAAACTGTCCGAGTGAGATGGTA
TP31988_Hit	D	1	chr3	.	CAGCCTTGTAAATACAAGGAAGCTCACTTGTAAACTTTTCTATGTACTTTTCAAAGGATGACTA
TP31988_Query	D	1	chr3	.	CAGCCTAGTAAATACAAGGAAGCTCACTTGTAAACTTTTCTATGTACTTTTCAAAGGATGACTA
TP32001_Hit	D	1	chr3	.	CAGCCTAGTGCACGAGGCTCTCACTAAGTGGGGTCTAGGGTGTACATGTATGCAACTTGTGCCC
TP32001_Query	D	1	chr3	.	CAGCCTAGTGCACGAGGCTCTCACTAAGTGGGGTCTAGGGTGTACATGTATGCAACTTGCGCCC
TP32025_Hit	D+G	1	chr3	.	CAGCCTATAAATCTGGAACTTCTACTGCCATACAGTTTATGGTGTTCCACAATTTCAATGTCGA
TP32025_Query	D+G	1	chr3	.	CAGCCTATAAATCTGCAACTTCTACTGCCATACAGTTTATGGTGTTCCACAATTTCAATGTCGA
TP32100_Hit	D	1	chr3	.	CAGCCTATCTGATAATTTCTTCTTTATCTGTACTATGTGGGGGATTAAGAATAGGAGAAATCTT
TP32100_Query	D	1	chr3	.	CAGCCTATCTGATAATTTCTTCTTTATCTGTACTATATGGGGGATTAAGAATAGGAGAAATCTT
TP32713_Hit	D	1	chr3	.	CAGCCTCCCTGACAACCCCGCCTATAAGCATTCGCAAACCAATCTCGTTTGAATATGTCATAGG
TP32713_Query	D	1	chr3	.	CAGCCTCCCTGACAACCCCGCCTATAAGCATTCGCAAACCAATCTCGTTCGAATATGTCATAGG
TP32786_Hit	D+G	1	chr3	.	CAGCCTCCTCTATGCCTTTAGTGCCAGCATTAAGCCGCTCTTTCACCGTTTCATAATTTGCATT
TP32786_Query	D+G	1	chr3	.	CAGCCTCCTCTATGCCTTTAGTGCCAGCATTAAGCCGCTCTTTCACCATTTCATAATTTGCATT
TP32947_Hit	D	1	chr3	.	CAGCCTCGTGGAGAAGACTCAGATCGCGTGTCAAAGGATTGATCAATTAGAAGAATTAGAAATT
TP32947_Query	D	1	chr3	.	CAGCCTCGTGGAGAAGACGCAGATCGCGTGTCAAAGGATTGATCAATTAGAAGAATTAGAAATT
TP32996_Hit	D	1	chr3	.	CAGCCTCTATTGCTTCTGCCACTAAATGTTGGTTGTAACATTTATCTATTGACTGAGTGTTAAT
TP32996_Query	D	1	chr3	.	CAGCCTCTATTGCTTCTGCCACTAAATGTTGGTTGTAACATTTATCTATTAACTGAGTGTTAAT
TP33047_Hit	D	1	chr3	.	CAGCCTCTCCTGGTAAACAGAAGTGCTAGATCTTAATACAGATTTTACTTTTGTTTCACTGGCA
TP33047_Query	D	1	chr3	.	CAGCCTCTCCTGGTAAACAGAAGAGCTAGATCTTAATACAGATTTTACTTTTGTTTCACTGGCA
TP33112_Hit	D	1	chr3	.	CAGCCTCTGCTTCCATCAATATAATGCTTGGTGGACAATGATCCCCTTCCCCTACAATTAATAT
TP33112_Query	D	1	chr3	.	CAGCCTCTGCTTCCATCAATATAATGCTTGGTGGACAATGAACCCCTTCCCCTACAATTAATAT
TP33249_Hit	D	1	chr3	.	CAGCCTGAAATGTTTCCCTTCGTGTCAACAGCAAATCTTGTTCTCCTCAAATTTTTCTGCTGTC
TP33249_Query	D	1	chr3	.	CAGCCTGAAATGTTTCCCATCGTGTCAACAGCAAATCTTGTTCTCCTCAAATTTTTCTGCTGTC
TP334_Hit	D	1	chr3	.	CAGCAAAACAACTTAAATCTCCTGCAAATAAAAGTCTAAACTATACAAAACTTTCATTTTCACA
TP334_Query	D	1	chr3	.	CAGCAAAACAACTTAAATCTCCTGCAAATAAAAGTCTAAAATATACAAAACTTTCATTTTCACA
TP33525_Hit	D	1	chr3	.	CAGCCTGCATAAGAGGGTGTGCAAAGTAAGCTCCTAGTGTTAATGCAGTAATTACTAAGTATGG
TP33525_Query	D	1	chr3	.	CAGCCTGCATAAGAGGGTGTGCAAAGTAAGCTCCTAGTGTTAACGCAGTAATTACTAAGTATGG
TP3356_Hit	D	1	chr3	.	CAGCGACACTGAAAGTATAGTAAGTGTGGTTTTTCTCTTTTATTCGAGTTTGAACCGAAACTCC
TP3356_Query	D	1	chr3	.	CAGCAACACTGAAAGTATAGTAAGTGTGGTTTTTCTCTTTTATTCGAGTTTGAACCGAAACTCC
TP33729_Hit	D+G	1	chr3	.	CAGCCTGGGATAGTTCAGCTTGCCACCTACCCTGTTCTGTTCCAAGTGCGGTGCTGAAAAAAAA
TP33729_Query	D+G	1	chr3	.	CAGCCTGGGATAGTTCAGCTTGCCACCTACCCTGTTCTGTTCCAAGTGCCGTGCTGAAAAAAAA

Name	Filter	Nb hit (Mt4.0)	Mt Chr (Mt4.0)	Ms Chr (Li et al., 2014)	Sequence
TP33822_Hit	D+G	1	chr3	.	CAGCCTGTCATCGTACAATGATTCTGGGAATAGGTTTTATTAGGGCAAAATTCTGAACTTTTTT
TP33822_Query	D+G	1	chr3	.	CAGCCTGTCATCGTACAATGATTCTGGGAATAGGTTTTATTAGGACAAAATTCTGAACTTTTTT
TP33854_Hit	D+G	1	chr3	.	CAGCCTGTCTGCACATTATTGAAGCCATATGTTGGGGTGTTTTTCTGATTTGTGGCTATAGCTG
TP33854_Query	D+G	1	chr3	.	CAGCCTGTCTGCACATTATTGAAGCCATATGTTGGGGTGTTTTTCTGATTTGGGGCTATAGCTG
TP33968_Hit	D	1	chr3	.	CAGCCTGTTTCACTAGAGCTTGGTGGAAAGAGCCCGATAGTTGTTTTTGAGGATGTTGACCTTG
TP33968_Query	D	1	chr3	.	CAGCCTGTTTCACTAGAGCTTGGTGGAAAGAGCCCGATAATTGTTTTTGAGGATGTTGACCTTG
TP34022_Hit	D+G	1	chr3	.	CAGCCTTAACGATGCTTTCATCTTCTATACCAATTTGTTTCAATGTGGCTAGACTTTCATCCCA
TP34022_Query	D+G	1	chr3	.	CAGCCTTAACGATGCTTTCATCTTCTATACCAATTTGGTTCAATGTGGCTAGACTTTCATCCCA
TP34044_Hit	D	1	chr3	.	CAGCCTTAATAAGTTGATGAATAAAATTAGATTGATCCTATCCAAAGCACCAACTGGATCATGG
TP34044_Query	D	1	chr3	.	CAGCCTTAATAAGTTGATGAATAAAATTAGATTGATCCTATCCAAAGCACCAACTAGATCATGG
TP34048_Hit	D	1	chr3	.	CAGCCTTAATGGTTTCCTCGTCCTTGCAGAAAGTAAACCTGACTAGATTCTTCCCCTCTTCTGG
TP34048_Query	D	1	chr3	.	CAGCCTTAATAGTTTCCTCGTCCTTGCAGAAAGTAAACCTGACTAGATTCTTCCCCTCTTCTGG
TP34444_Hit	D	1	chr3	.	CAGCCTTCTCCTTTATGTCACTCCTAGGCCTTCATCCAATCAATAAGAAGGTGCAAAATCTAAT
TP34444_Query	D	1	chr3	.	CAGCCTTCTCCTTTATGTCACTCCTAGGCCTTCATCCAATCAATAAAAAGGTGCAAAATCTAAT
TP34460_Hit	D	1	chr3	.	CAGCCTTCTGAGACTGTTGAGGTTTGGTTTCTATTTGTGTGTGCATTTTGTTTGCATATCTGAT
TP34460_Query	D	1	chr3	.	CAGCCTTCTGAGACTGTTGAGGTTTGGTTTCTATTTGTGTGTGAATTTTGTTTGCATATCTGAT
TP34641_Hit	D	1	chr3	.	CAGCCTTGCGTAGAATCCTTCTGACTGCCCTTTCAAATTTCACTTATGAGAATGGTCAAGATAA
TP34641_Query	D	1	chr3	.	CAGCCTTGCGTAGAATCCTTCTGACTGCCCTTTCAAATTCCACTTATGAGAATGGTCAAGATAA
TP34753_Hit	D	1	chr3	.	CAGCCTTGTCTTGCACACATTTTCAACAGTGGTTCCATAGTTTGAACCTATGAGCTCCTAATCT
TP34753_Query	D	1	chr3	.	CAGCCTTGTCTTGCACACATTTTCAACAGTGGTTCCATAGTTCGAACCTATGAGCTCCTAATCT
TP34941_Hit	D	1	chr3	.	CAGCCTTTGAGATGGAGTATGGACATTGGGTTGATGAGCAAAACCGGCAAATCTTGGAAATGAG
TP34941_Query	D	1	chr3	.	CAGCCTTTGAGATGGAGTATGGACATTGGGTTGATGAGCAAAACCGGCAAATCTCGGAAATGAG
TP34971_Hit	D+G	1	chr3	.	CAGCCTTTGCTAATTCTATAGCTTCTTTATCACTTCGATTTGTGGCTTCCAGTTATTGTGATCT
TP34971_Query	D+G	1	chr3	.	CAGCCTTTGCTAATTCTATAGCTTCTATATCACTTCGATTTGTGGCTTCCAGTTATTGTGATCT
TP35069_Hit	D+G	1	chr3	.	CAGCCTTTTCCCTCCAACGCCTCCACTATTCTCATCACCTTCAATCACGACGTATGATTAGACA
TP35069_Query	D+G	1	chr3	.	CAGCCTTTTCCCTCCAACGCCTCCACTATTCTCATCACCTTCAATCACGACATATGATTAGACA
TP35083_Hit	D	1	chr3	.	CAGCCTTTTCTCTATAGAAGACTGTTCTCTCAATGGCTACAACTGGTTGCACCGATGTAGCATT
TP35083_Query	D	1	chr3	.	CAGCCTTTTCTCTATAGAAAACTGTTCTCTCAATGGCTACAACTGGTTGCACCGATGTAGCATT
TP35201_Hit	D	1	chr3	.	CAGCGAAAATTATGATTCAGTTTTGAACTTAAGTTTGTTGAATTGAACGCATTATATGATTAAA
TP35201_Query	D	1	chr3	.	CAGCGAAAATTATGATTCACTTTTGAACTTAAGTTTGTTGAATTGAACGCATTATATGATTAAA
TP35277_Hit	D	1	chr3	.	CAGCGAAAGTATTGAAAACTGTTCTCGAAGGAGATGCTAAGCGCCGTGCACTTGCTTCTATCAA
TP35277_Query	D	1	chr3	.	CAGCGAAAGTATTGAAAACTGTACTCGAAGGAGATGCTAAGCGCCGTGCACTTGCTTCTATCAA
TP35321_Hit	D	1	chr3	.	CAGCGAACAACAAAAATAGTTCCTGTTGAATGTGTGAGTGCAATGCTTGTTGTTTTGAATGCAT
TP35321_Query	D	1	chr3	.	CAGCGAACAACAAAAATAGTTCCTGTTGAACGTGTGAGTGCAATGCTTGTTGTTTTGAATGCAT
TP35450_Hit	D	1	chr3	.	CAGCGAAGCAACTTCTTGATGTTGCTACTCTCCCGAGGGGGAAAAAATGTTTTGTTGCATAAAG
TP35450_Query	D	1	chr3	.	CAGCGAAGCAACTTCTTGATGTTGCTACTCTCCCGAGGGGAAAAAAATGTTTTGTTGCATAAAG
TP35679_Hit	D	1	chr3	.	CAGCGACAACGAGACCATTCACCTGCTAATACTCAAGACTGTTCACCTGAAGCTGGTGATTTGT
TP35679_Query	D	1	chr3	.	CAGCGACAACGAGACCATTCACCTGCTAACACTCAAGACTGTTCACCTGAAGCTGGTGATTTGT
TP35855_Hit	D+G	1	chr3	.	CAGCGACGAAAGCGGCGACAGATCCGCCAACGACGGAGGCTCTGAATGAAGCGGCGGCGCTAGC
TP35855_Query	D+G	1	chr3	.	CAGCGACGAAAGCGGCGACAGATCCGCCAACGACGGAGACTCTGAATGAAGCGGCGGCGCTAGC
TP36090_Hit	D	1	chr3	.	CAGCGAGAATCGGAAATCCTCAACTCCTTCTTCTTCCCTAACTTGTACAGGAAACACCTTAACA
TP36090_Query	D	1	chr3	.	CAGCGAGAATCGGAAATCCTCAACTCCTTCTTCTTCCCTAACTTGCACAGGAAACACCTTAACA
TP36431_Hit	D	1	chr3	.	CAGCGAGTTCACGAGCATTAGCTTGAACTAGAAACCGTCGACCTTCAATAACGTTCTGTTTAAC
TP36431_Query	D	1	chr3	.	CAGCGAGTTCACGAGCATTAGCTTGAACTAGAAACCGCCGACCTTCAATAACGTTCTGTTTAAC
TP36458_Hit	D	1	chr3	.	CAGCGATAAAGACCCCAACGTTACTGTTTTATATCTGCGTTTCAGACTTTTCACTTTTTTACTA
TP36458_Query	D	1	chr3	.	CAGCGATAAAGACCCCAACGTTACTGCTTTATATCTGCGTTTCAGACTTTTCACTTTTTTACTA
TP36515_Hit	D	1	chr3	.	CAGCGATATCAGATTTATGTTATTAGGACCTATTTCCTGCATGCTATAAGGTTTCAATCCTGCT
TP36515_Query	D	1	chr3	.	CAGCGATATCAGATTTATGTTATTAGGACCTATTTCCTGCATGCTATAAGGATTCAATCCTGCT
TP36655_Hit	D	1	chr3	.	CAGCGATGATGCGTGCACAAGAGGCTGATCCAACAAACTTGGAGGTGCTTCTTGCTCTTGGTGT
TP36655_Query	D	1	chr3	.	CAGCGATGATGCGTGCACAAGAGGCTGATCCAGCAAACTTGGAGGTGCTTCTTGCTCTTGGTGT
TP3682_Hit	D	1	chr3	.	CAGCAACATCAATTTGTTCATATAGTTTTTCACAAGAATTATACTTTCCATTTTCCCACTTTGA
TP3682_Query	D	1	chr3	.	CAGCAACATCAACTTGTTCATATAGTTTTTCACAAGAATTATACTTTCCATTTTCCCACTTTGA
TP36859_Hit	D	1	chr3	.	CAGCGATTGCTTCTGTGCAACCAGATGTAACAGTAATTTCCTTATCAGGGTCTACAATTAGTCC
TP36859_Query	D	1	chr3	.	CAGCGATTGCTTCTGTGCAACCAGATGTAACAGTAATTTCCTTATCAGGATCTACAATTAGTCC

Name	Filter	Nb hit (Mt4.0)	Mt Chr (Mt4.0)	Ms Chr (Li et al., 2014)	Sequence
TP36968_Hit	D	1	chr3	.	CAGCGCAAGAAGCCTACAACGAGAAGGAGCATATCCTGAGTCTTTTCTTTAAACAGGCTTCTCA
TP36968_Query	D	1	chr3	.	CAGCGCAAGAAGCCTACAACGAGAAGGAGCATATCATGAGTCTTTTCTTTAAACAGGCTTCTCA
TP36975_Hit	D+G	1	chr3	.	CAGCGCAAGAGTTTGCACCTTTCGGTGTTTCTTTTACATTCAAATTCAACTATGATTATTAACA
TP36975_Query	D+G	1	chr3	.	CAGCGCAAGAGTCTGCACCTTTCGGTGTTTCTTTTACATTCAAATTCAACTATGATTATTAACA
TP3700_Hit	D	1	chr3	.	CAGCAACATCACGCATGGCGGAGGCAGACTGGTCTGATCTCCCGGAGGAGCTTCTGAATCTGAT
TP3700_Query	D	1	chr3	.	CAGCAACATCACGCATGGCGGAGGCACACTGGTCTGATCTCCCGGAGGAGCTTCTGAATCTGAT
TP37064_Hit	D	1	chr3	.	CAGCGCATGGAACAAAATGCCCCAGGCAACAAAATTAATGAAACTATTCATTAGACTGACCGGC
TP37064_Query	D	1	chr3	.	CAGCGCACGGAACAAAATGCCCCAGGCAACAAAATTAATGAAACTATTCATTAGACTGACCGGC
TP37083_Hit	D+G	1	chr3	.	CAGCGCAGAAAAAGGGATCAGAATTGTGTCCTCAAGTTCCTATTAATTCATCGACCAAAGGTTA
TP37083_Query	D+G	1	chr3	.	CAGCGCAGAAAAAGGGATCAGAATTGTGTCCTCAAGTTCCTATCAATTCATCGACCAAAGGTTA
TP37088_Hit	D	1	chr3	.	CAGCGCAGAAAAGGGATCAGAATTGTGTCCTCAAGTTCCTATTAATTCATCGACCAAAGGTTAT
TP37088_Query	D	1	chr3	.	CAGCGCAGAAAAGGGATCAGAATTGTGTCCTCAAGTTCCTATCAATTCATCGACCAAAGGTTAT
TP37256_Hit	D	1	chr3	.	CAGCGCATGCAAGTTCAGGGCACAATTACATCCTGGAGTTCTACTGCAATGAAGAATGGCATTG
TP37256_Query	D	1	chr3	.	CAGCGCATGCAAGTTCAAGGCACAATTACATCCTGGAGTTCTACTGCAATGAAGAATGGCATTG
TP38103_Hit	D	1	chr3	.	CAGCGCTTTTGATGGAAACTAAGGAGTATTCAGAAGCTTTGACTCTTCTTTCTGGGTTGGTCAA
TP38103_Query	D	1	chr3	.	CAGCGCTATTGATGGAAACTAAGGAGTATTCAGAAGCTTTGACTCTTCTTTCTGGGTTGGTCAA
TP38240_Hit	D	1	chr3	.	CAGCGCTTAACGCGCTTCAATACGACGACGTTAGAGTGGTTGTGAGTGAAACGGGTTGGCCTTC
TP38240_Query	D	1	chr3	.	CAGCGCTTAACGCGCTTCAATACAACGACGTTAGAGTGGTTGTGAGTGAAACGGGTTGGCCTTC
TP38287_Hit	D	1	chr3	.	CAGCGCTTGAAACCTCTTCACCGGAAGCTTTGGTGTGGAGTACGGTAATCGATGAATTATCGAA
TP38287_Query	D	1	chr3	.	CAGCGCTTGAAACCTCTTCACCGGAAGCTTTGGTGTGGAGTACGGTAATCGATGAATCATCGAA
TP38724_Hit	D+G	1	chr3	.	CAGCGGATGATCTCCTAGGCATTATCTTCGGTTACATCGTCTTTGATGTTGGTGAACTGAAGCG
TP38724_Query	D+G	1	chr3	.	CAGCGGATGATCTCCCAGGCATTATCTTCGGTTACATCGTCTTTGATGTTGGTGAACTGAAGCG
TP38744_Hit	D	1	chr3	.	CAGCGGATGTCAAAGTGCATAAGATCCCTGTGCAATGCCGATGCCAAAGAAGGTTTCGTGTTAG
TP38744_Query	D	1	chr3	.	CAGCGGATGTCAAAGGGCATAAGATCCCTGTGCAATGCCGATGCCAAAGAAGGTTTCGTGTTAG
TP3877_Hit	D	1	chr3	.	CAGCAACATTTTTATCATCTGGGCTAATACACGAGCGACTCCAATTGGAGGCCACTCTATTTCC
TP3877_Query	D	1	chr3	.	CAGCAACATTTTTATCATCCGGGCTAATACACGAGCGACTCCAATTGGAGGCCACTCTATTTCC
TP3889_Hit	D+G	1	chr3	.	CAGCAACCAAATCATGCTTATTCACAGCCTGTAGGAGCATATCAAAATACAGGTGCTCCTTATC
TP3889_Query	D+G	1	chr3	.	CAGCAACCAAATCATGCTTATTCACAGCCTATAGGAGCATATCAAAATACAGGTGCTCCTTATC
TP39080_Hit	D+G	1	chr3	.	CAGCGGCGAGCATGCCAGGATGGTGATACATCTGTTGTTGTTGAAGCAAAGCTTGCTGAAAAAA
TP39080_Query	D+G	1	chr3	.	CAGCGGCGAGCATGCCAGGATGGTGATACATCTGTTGTTGTTGAAGCAAAGCGTGCTGAAAAAA
TP3919_Hit	D	1	chr3	.	CAGCAACCAAGGCCAGAGGTGCTGACCTTAGGGTTCATTTCAAGGTACTTAATTCTGTTAACTT
TP3919_Query	D	1	chr3	.	CAGCAACCAAGGCCAGAGGTGCTGACCTTAGGGTTCATTTCAAGGTAATTAATTCTGTTAACTT
TP39666_Hit	D	1	chr3	.	CAGCGGTGATGGAGGCCCCAGTTATTTTTATATGCCGGAATAATGGATGGGCTATAAGTACCCC
TP39666_Query	D	1	chr3	.	CAGCGGTAATGGAGGCCCCAGTTATTTTTATATGCCGGAATAATGGATGGGCTATAAGTACCCC
TP39677_Hit	D	1	chr3	.	CAGCGGTACCTAGACTAATTTTAAACCACTCCCATACTTTTAACAAATCCACGCTCTTCTAAAA
TP39677_Query	D	1	chr3	.	CAGCGGTACCTAGACTAATTTTAAACCACTCCCATACTTTTAACAAATCCACACTCTTCTAAAA
TP39848_Hit	D	1	chr3	.	CAGCGGTGCTAATAAATACGGTCCTACAATTGGTTCATCATCTTCGGCTTCAACTCCATCAACT
TP39848_Query	D	1	chr3	.	CAGCGGTGCTAATAAATACGGTCCTACAATTGGTTCATCATCTTCAGCTTCAACTCCATCAACT
TP40083_Hit	D+G	1	chr3	.	CAGCGGTTTCATCAGCTACAAGTGCCAAACACTTTTTATTTTGCCCTTCAAGTGTTGTCCTCCC
TP40083_Query	D+G	1	chr3	.	CAGCGGTTTCATCAGCTACAAGTGCCAAACACTTTTTATTTTGCCCTTCAAGTGTTATCCTCCC
TP40130_Hit	D+G	1	chr3	.	CAGCGTTAATAAAATACCTCAAAACTGGCCAAGTCCAAGGAAACAAGGAGGCCTTCCTGCTTGG
TP40130_Query	D+G	1	chr3	.	CAGCGTAAATAAAATACCTCAAAACTGGCCAAGTCCAAGGAAACAAGGAGGCCTTCCTGCTTGG
TP40335_Hit	D	1	chr3	.	CAGCGTATCCATATAAACATCAGTATCCTTGGGTACAGATTTCAGATGGTGCACTTGGCGGTAA
TP40335_Query	D	1	chr3	.	CAGCGTATCCATATAAACATCAGTATCCTTAGGTACAGATTTCAGATGGTGCACTTGGCGGTAA
TP40345_Hit	D	1	chr3	.	CAGCGTATGAAGAGGCCAAGAGAAAGTTTGCTTTGCAGGAGGAAAAACAACAGCAAGAAAAGGC
TP40345_Query	D	1	chr3	.	CAGCGTATGAAGAGGCCAAGAGAAAGGTTGCTTTGCAGGAGGAAAAACAACAGCAAGAAAAGGC
TP40392_Hit	D	1	chr3	.	CAGCGTATTTTTTAGAACAACGTTCACATTTCCATTTCTTCTCACCGTGCTTTCTACAAAAGTG
TP40392_Query	D	1	chr3	.	CAGCGTATTTTTTAGAACAACGTTCACATTTCCATTTCTTCTAACCGTGCTTTCTACAAAAGTG
TP40546_Hit	D	1	chr3	.	CAGCGTCCTCGAGTTAAAGGACAATTTGTTCGCCAACCGAATCCTGATTCTCTTTCTGGAGAAA
TP40546_Query	D	1	chr3	.	CAGCGTCCTCGAGTTAAAGGACAATTTGTTCGCCAACCGAATCCCGATTCTCTTTCTGGAGAAA
TP40652_Hit	D	1	chr3	.	CAGCGTCTCAAATATGCTACCTGACCCTGCATAAAATCAGACTAGTTACAGTTACTACCTCAGA
TP40652_Query	D	1	chr3	.	CAGCGTCTCAAATATGCTACCCGACCCTGCATAAAATCAGACTAGTTACAGTTACTACCTCAGA
TP40745_Hit	D	1	chr3	.	CAGCGTGAACTGAGTGGGACTTTGCAAACTGACTTGGACGCAAAGACTCAGAAGCAAATATCAA
TP40745_Query	D	1	chr3	.	CAGCGTGAACTCAGTGGGACTTTGCAAACTGACTTGGACGCAAAGACTCAGAAGCAAATATCAA

Name	Filter	Nb hit (Mt4.0)	Mt Chr (Mt4.0)	Ms Chr (Li et al., 2014)	Sequence
TP4081_Hit	D	1	chr3	.	CAGCAACCCTCTAATTGTTGTTTCAATGCTACTGTTCATTTTGATTCCTCAAGTTCATACTCCC
TP4081_Query	D	1	chr3	.	CAGCAACCCTCTAATTGTTGTTTCAATGCTACTGTTCATTTTGATTCATCAAGTTCATACTCCC
TP40812_Hit	D	1	chr3	.	CAGCGTGCGTAGGAGATTCTACAGTAAGCCCATTATAAGTGCTTGCATCTGCATAGTAAAATGA
TP40812_Query	D	1	chr3	.	CAGCGTGAGTAGGAGATTCTACAGTAAGCCCATTATAAGTGCTTGCATCTGCATAGTAAAATGA
TP41080_Hit	D	1	chr3	.	CAGCGTGTAATTTTCAATTCTTTTTTGCTTTTGCAGTTTCTATTGTCCATGCCTTTTTTGCTTT
TP41080_Query	D	1	chr3	.	CAGCGTGTAATTTTCAATTCTTTTTTGCTTTTGCAGTTTCTATTGTCCATGCCTTTTTCGCTTT
TP41148_Hit	D	1	chr3	.	CAGCGTGTGGTTGACAAGCTGAGGTGCTTCTTGGCGAATTTGTGATCGGTGATTAAAGAATTCC
TP41148_Query	D	1	chr3	.	CAGCGTGTGGTTGACAAGCTGAGGTGCTTCTTGACGAATTTGTGATCGGTGATTAAAGAATTCC
TP41166_Hit	D+G	1	chr3	.	CAGCGTGTTACCCAAATCATCGTCATTTGCAGGACCATCTCTTGGTCAAAGGGCTCGTGGTTTA
TP41166_Query	D+G	1	chr3	.	CAGCGTGTTACCCAAATCATCATCATTTGCAGGACCATCTCTTGGTCAAAGGGCTCGTGGTTTA
TP41323_Hit	D	1	chr3	.	CAGCGTTCATGCCAGCAACGATGAGAGATAGGAGAAGAGCTAACTGATTGGAATTAAACCCAGC
TP41323_Query	D	1	chr3	.	CAGCGTTCATGCCAGCAACGATGAGAGATAGAAGAAGAGCTAACTGATTGGAATTAAACCCAGC
TP41515_Hit	D+G	1	chr3	.	CAGCGTTGTTTCCTTCTAATGGTGTTAAGAGAAGCTTTGATTCTGAGTCGGAGAGATAAAACTC
TP41515_Query	D+G	1	chr3	.	CAGCGTTGTTGCCTTCTAATGGTGTTAAGAGAAGCTTTGATTCTGAGTCGGAGAGATAAAACTC
TP41658_Hit	D	1	chr3	.	CAGCGTTTTGCTAATAACTTAGCATCAGCCAACTCATTAACCAGCCTGGAATTGACAATTTCAG
TP41658_Query	D	1	chr3	.	CAGCGTTTTGCTAATAACTTAGCATCAGCCAACTCATTAACCAACCTGGAATTGACAATTTCAG
TP41710_Hit	D+G	1	chr3	.	CAGCTAAAAACTCGCAACATCCATCCACGGCGTGACCGCCTATGCTGACGGCGTGATTCTTTTG
TP41710_Query	D+G	1	chr3	.	CAGCTAAAAACTCGCAACATCCATCCACGGCGTGACCACCTATGCTGACGGCGTGATTCTTTTG
TP41830_Hit	D	1	chr3	.	CAGCTAAAATTTCTGGCATGCTCTTTATAAGAAGCCATCATAATTTAAGGTTGTTCTGAAACTC
TP41830_Query	D	1	chr3	.	CAGCTAAAATTTCTCGCATGCTCTTTATAAGAAGCCATCATAATTTAAGGTTGTTCTGAAACTC
TP41855_Hit	D+G	1	chr3	.	CAGCTAAACAGCAGAATTCTTTGAATTTAAGTTCTTTCTCCATTTTTCATTTAAAGCTATCAAC
TP41855_Query	D+G	1	chr3	.	CAGCTAAACAGCAGAATTCTTTGAATTTAAGTTCTTTCTCCATTTTTCATTTAAAGCAATCAAC
TP42142_Hit	D	1	chr3	.	CAGCTAACAAGGGCTTATTTTGCATTCTTGGAAGTTTTGTTCAACAGCCACATCACTTTCATAT
TP42142_Query	D	1	chr3	.	CAGCTAACAAGGGCTTATTTTGCATTCATGGAAGTTTTGTTCAACAGCCACATCACTTTCATAT
TP4219_Hit	D	1	chr3	.	CAGCAACCTGCTTCAGTTCGTCAGCACTACTCTTTCCATCCCAAAATGATTCCAGGGCAAACTT
TP4219_Query	D	1	chr3	.	CAGCAACCTGCTTCAGTTCCTCAGCACTACTCTTTCCATCCCAAAATGATTCCAGGGCAAACTT
TP42223_Hit	D+G	1	chr3	.	CAGCTAACCATTTGACTATGAACCACAATTGCTATCTCCTGTTGTAGTAAAAACTAGCTTCAGG
TP42223_Query	D+G	1	chr3	.	CAGCTAACCATTTGACTATGAACCACAATTGCTATCTCCTGTTGTAGTAAAAACTAGCTTCACG
TP42365_Hit	D+G	1	chr3	.	CAGCTAAGACCTTGAATCACTTAATTCATAAATCTGATACAGCAACTATCAGTCAGCTTACATC
TP42365_Query	D+G	1	chr3	.	CAGCTAAGACCTTGAATCACTTAATTCATAAATCTGATACAGCAACTATCAGTCAGCTTACAGC
TP42383_Hit	D	1	chr3	.	CAGCTAAGATAACACGGAAACGGAAGGAAAATTAATATAAGAGGACAGTTTTTTAATACAAGAC
TP42383_Query	D	1	chr3	.	CAGCTAAGATAACACGGAAACGGAAGGAAAAATAATATAAGAGGACAGTTTTTTAATACAAGAC
TP42459_Hit	D	1	chr3	.	CAGCTAAGGATCAATTGTTGCTGTGAGATTAACACTCAGTTAATAGATAAATGTTACAACCAAC
TP42459_Query	D	1	chr3	.	CAGCTAAGGATCAATTGTTGCTGTGAGATTAACACTCAGTCAATAGATAAATGTTACAACCAAC
TP42634_Hit	D	1	chr3	.	CAGCTAATATTTGCTAATGCACTATACTTTAAAGGAGAGTGGAATGATAATTTTGATGCTTTGG
TP42634_Query	D	1	chr3	.	CAGCTAATATTTGCTAATGCACTATACTTTAAAGGAGAGTGGAATGATAATTTTGATGCTTCGG
TP42675_Hit	D	1	chr3	.	CAGCTAATGAAAAACTAAGTAAGAGCTAATGACAGAAAAACATAGGTAAATGCGAATAAAATAA
TP42675_Query	D	1	chr3	.	CAGCTAATGAAAAACTAAGTAAAAGCTAATGACAGAAAAACATAGGTAAATGCGAATAAAATAA
TP42731_Hit	D+G	1	chr3	.	CAGCTAATGGAGGGTATTGAGGGAGAAAGACTCGTCTCACTTTCAACTTCTGTTAAGATTTTGT
TP42731_Query	D+G	1	chr3	.	CAGCTAATGGAGGGTATTGAGGGAGAAAGACTCGTCTCACTTTCAACTTCGGTTAAGATTTTGT
TP42801_Hit	D	1	chr3	.	CAGCTAATTCTTCTGAAATGCCTGATGCTACTGATGCTTTACAGGCTCAAAACAATGTTGTCTC
TP42801_Query	D	1	chr3	.	CAGCTAATTCTTCTGAAATGCCTGATGCCACTGATGCTTTACAGGCTCAAAACAATGTTGTCTC
TP42932_Hit	D+G	1	chr3	.	CAGCTACAACCATGCATGAGCATTTTGTTGACATGTTCTACTTTAACCTGCATTCTAATATATT
TP42932_Query	D+G	1	chr3	.	CAGCTACAACCATGCATGAGCATTTTGTTGACATGTTCTACTTTAACCTGCATTATAATATATT
TP43003_Hit	D	1	chr3	.	CAGCTACGATTGTAGTTGTGGATTGCAAATTAAAACCTTGAAGCTTCAAGTTATTAGTTATTTT
TP43003_Query	D	1	chr3	.	CAGCTACAATTGTAGTTGTGGATTGCAAATTAAAACCTTGAAGCTTCAAGTTATTAGTTATTTT
TP43031_Hit	D+G	1	chr3	.	CAGCTTCACCAATGAGTGCCATGGCATTAACCTTAGCACACTACAAAGAATCCATCACTCAATC
TP43031_Query	D+G	1	chr3	.	CAGCTACACCAATGAGTGCCATGGCATTAACCTTAGCACACTACAAAGAATCCATCACTCAATC
TP43066_Hit	D	1	chr3	.	CAGCTACACTTGCCTTTTATAAAGGTCCTCAATTAAAACTTTTAAGTCATCATCATCATTACCT
TP43066_Query	D	1	chr3	.	CAGCTACACTTGCCTTTTATAAAGGTCCTCAATTAAAACTTTCAAGTCATCATCATCATTACCT
TP43191_Hit	D	1	chr3	.	CAGCTACATGGACAGGATCTGATGCCGATGTAATAGCAACCCTGGAAATACATAAAGCTACAGA
TP43191_Query	D	1	chr3	.	CAGCTACATGAACAGGATCTGATGCCGATGTAATAGCAACCCTGGAAATACATAAAGCTACAGA
TP43329_Hit	D	1	chr3	.	CAGCTACCGTGCCAGAATCTATGGCTTTGATTAATGGGTTTTTGGGTTTTTCTCATAATAATTT
TP43329_Query	D	1	chr3	.	CAGCTACCGTGCCAGAATCTATGGCTTTGATTAATGGGTTTTTGGGTTTTTCTAATAATAATTT

Name	Filter	Nb hit (Mt4.0)	Mt Chr (Mt4.0)	Ms Chr (Li et al., 2014)	Sequence
TP43474_Hit	D	1	chr3	.	CAGCTACTAAGAATCCTTTTTTCGTGCAAAGAAGACCAAATTTTCCTATCCTGACAGTAGCATT
TP43474_Query	D	1	chr3	.	CAGCTACTAAGAATCCTTTTTTCGTGCAAAGAAGACCAAATTTTCCGATCCTGACAGTAGCATT
TP43511_Hit	D	1	chr3	.	CAGCTACTATCCAAAAACTGCCGTGCAAAGAGGCTGTTATCGGAAAATGAATCTTTATTAGCAA
TP43511_Query	D	1	chr3	.	CAGCTACTATCCAAAAACTGCCGTGCAAAGAGGCTGTTATCGGAAAATGAATCTTTAGTAGCAA
TP43558_Hit	D	1	chr3	.	CAGCTACTCGCTTCATCGAACACTCTTTGCCTGACCTCACTCCAGGTGGGTTGTTAACTTGGTT
TP43558_Query	D	1	chr3	.	CAGCTACTCGCTTCATCGAACACTCTCTGCCTGACCTCACTCCAGGTGGGTTGTTAACTTGGTT
TP43659_Hit	D+G	1	chr3	.	CAGCTACTTGACCTTCTTCAGAACTGGACTTCAGTAGGCCGAGCAAAGGAGGAATGCATCCTCC
TP43659_Query	D+G	1	chr3	.	CAGCTACTTGACCTTCTTCAGAACTGGACTTCAGTAGACCGAGCAAAGGAGGAATGCATCCTCC
TP43762_Hit	D	1	chr3	.	CAGCTAGAAGGATGGTTTGGGGATTAAGGTTGATTATGGCTCTGGTATGATATGAATAATTACT
TP43762_Query	D	1	chr3	.	CAGCTAGAAGGATGATTTGGGGATTAAGGTTGATTATGGCTCTGGTATGATATGAATAATTACT
TP43930_Hit	D	1	chr3	.	CAGCTAGATGTCAAATGGCTGTCCTCCCATTCCACTCATTGCTAACTTCTGATCACCTTTTTCC
TP43930_Query	D	1	chr3	.	CAGCTAGATGTCAAATGGCTGTCCTCCAATTCCACTCATTGCTAACTTCTGATCACCTTTTTCC
TP44047_Hit	D	1	chr3	.	CAGCTAGCCGAGGAAGAAGGCCAGGAACATGATATAATTTCTGTTCTGAATCTAATGGCAACTA
TP44047_Query	D	1	chr3	.	CAGCTAGCCGAGGAAGAAGGCCACGAACATGATATAATTTCTGTTCTGAATCTAATGGCAACTA
TP44150_Hit	D	1	chr3	.	CAGCTAGGATCGTAAAAAATGAATCGGTCTTTTGTGTCGAAAGTGCGGTCTCTCACTCGATCTC
TP44150_Query	D	1	chr3	.	CAGCTAGGATCGTAAAAAATGAATCGGTCTTTTGTGTCGAAAGTGCGGTCTCGCACTCGATCTC
TP44188_Hit	D	1	chr3	.	CAGCTAGGGAGTACTTTTTCTCAGCTTCAAGCCTTTCTTGTTCTGAAGTAAGATTGAGTGCATT
TP44188_Query	D	1	chr3	.	CAGCTAGGGAGTACTTTTTCTCAGCTTCAAGCCTTTCTTGTTCTGAAGTAAGATTGAGCGCATT
TP44227_Hit	D+G	1	chr3	.	CAGCTAGGTCATCCGCATGGCTTCTTCGGCATGAATCAACACCTGTCGAACCTTGCAATGTCGG
TP44227_Query	D+G	1	chr3	.	CAGCTAGGTCATCCACATGGCTTCTTCGGCATGAATCAACACCTGTCGAACCTTGCAATGTCGG
TP44297_Hit	D	1	chr3	.	CAGCTAGTATATAGATGGCCTGTCACAAAGAGAATAACACAATTGGCCACATGGTAAAAAAGGC
TP44297_Query	D	1	chr3	.	CAGCTAGTATATAGATGGCCTGTCACAAAGAGAATAACACAATTAGCCACATGGTAAAAAAGGC
TP44339_Hit	D+G	1	chr3	.	CAGCTAGTGACAATTTCTCATTGGGAAATAAAATTGGTGCTGGTAGTTTTGGATGTGTTTACAA
TP44339_Query	D+G	1	chr3	.	CAGCTAGTGACAATTTCTCATTGGGAAATAAAATTGGTGCTGGCAGTTTTGGATGTGTTTACAA
TP44362_Hit	D	1	chr3	.	CAGCTAGTGCTCGATTTTAAAGTTGCTAATAAAGCACAGATCCCGAACAGAAAAAGCACATATA
TP44362_Query	D	1	chr3	.	CAGCTAGTGCTAGATTTTAAAGTTGCTAATAAAGCACAGATCCCGAACAGAAAAAGCACATATA
TP44496_Hit	D+G	1	chr3	.	CAGCTATAACAGCGTCTTCACTAGTAAGAAGTGATTCTACAGATGAGGAGGAGGAGGAGTTTGC
TP44496_Query	D+G	1	chr3	.	CAGCTATAACAGCGTCTTCACTAGTAAGAAGTGACTCTACAGATGAGGAGGAGGAGGAGTTTGC
TP44518_Hit	D	1	chr3	.	CAGCTATAAGCGTAGCAAAATTTGAACAAAGTTTTCTTCTTCCGTGATACATTATTTAGTAGGG
TP44518_Query	D	1	chr3	.	CAGCTATAAGCGTAGCAAAATGTGAACAAAGTTTTCTTCTTCCGTGATACATTATTTAGTAGGG
TP44651_Hit	D	1	chr3	.	CAGCTATAGCCCCAAATCAGAAAAACACCCCAACATATGGCTTCAATAATGTGCAGACAGGCTG
TP44651_Query	D	1	chr3	.	CAGCTATAGCCACAAATCAGAAAAACACCCCAACATATGGCTTCAATAATGTGCAGACAGGCTG
TP44658_Hit	D	1	chr3	.	CAGCTATAGCGACACTATAGTGCTATAGTGTAGCGCAATTTGAACAAATTGCTTTTGTTGTTGT
TP44658_Query	D	1	chr3	.	CAGCTATAGCGACACTATAGTACTATAGTGTAGCGCAATTTGAACAAATTGCTTTTGTTGTTGT
TP44669_Hit	D+G	1	chr3	.	CAGCTATAGCTAATGTCCAGCACGGTGATATTTGTCCGGAAAACATAATACGCGTTGTCGAAAA
TP44669_Query	D+G	1	chr3	.	CAGCTATAGCTAATGTCCAGCACGGTGATATTTGTCCGGAAAACATAATACGCGCTGTCGAAAA
TP44833_Hit	D+G	1	chr3	.	CAGCTATCAAATTCTTCTGTTATTAGTATTGCGGTTGACTTGATGTTTCCATGTGTAAATTTTG
TP44833_Query	D+G	1	chr3	.	CAGCTATCAAATTCTTCTGTTATTAGTATGGCGGTTGACTTGATGTTTCCATGTGTAAATTTTG
TP44896_Hit	D	1	chr3	.	CAGCTATCATGCAAATGCTCTGGAGGTGGAGATGCACATGCATCAACAATGGTGTTAGTGAATT
TP44896_Query	D	1	chr3	.	CAGCTATCATGCAAATGCTCTGGAGGTGGAGATGCACATGCATCAACAATGGTGTTACTGAATT
TP45007_Hit	D	1	chr3	.	CAGCTATGAAATTGCAAGATGTAAAAAGATTAGAAGAAGAATTAGAACGACTGACAGAAGAAAT
TP45007_Query	D	1	chr3	.	CAGCTATGAAATTACAAGATGTAAAAAGATTAGAAGAAGAATTAGAACGACTGACAGAAGAAAT
TP45040_Hit	D	1	chr3	.	CAGCTTTGACGATTCAAATGAGAGCAATGCGTGAGCATTTTGAGAGGCTGTTAAGAGAACAAGC
TP45040_Query	D	1	chr3	.	CAGCTATGACGATTCAAATGAGAGCAATGCGTGAGCATTTTGAGAGGCTGTTAAGAGAACAAGC
TP45109_Hit	D+G	1	chr3	.	CAGCTATGCACTCCTCAGCTTTGTGCATCCAATGTGCGTTACTACCATCACTATAGCTGGTGCT
TP45109_Query	D+G	1	chr3	.	CAGCTATGCACTCCTCAGCTTTGTGCATCCAATGTGCGTTACTACCATCACTACAGCTGGTGCT
TP45168_Hit	D	1	chr3	.	CAGCTATGGAGATGCAAGCAGAAGCAGAACGGAAAAAGAGAGCTCAAATTCTTGAGTCCGAAGG
TP45168_Query	D	1	chr3	.	CAGCTATGGAGATGCAAGCAGAAGCAGAACGAAAAAAGAGAGCTCAAATTCTTGAGTCCGAAGG
TP45179_Hit	D	1	chr3	.	CAGCTATGGCACACCAGAGGATTTCGATAAAACTGAATAAGTTTAAAGAAAAATGAAAATATTA
TP45179_Query	D	1	chr3	.	CAGCTATGGCACACCAGAGGATTTCGATAAAACTGAATAAGTTTAAAGAAAAAGGAAAATATTA
TP45205_Hit	D	1	chr3	.	CAGCTATGGGGTGCCAACATAGTTGGGGTGCCATAGCTGTCTTGTGATTCCCTTGCCCTCTTTT
TP45205_Query	D	1	chr3	.	CAGCTATGGGGTGCCAACATAGTTGGGGTGCCATAGCTGTCTTGTGATGCCCTTGCCCTCTTTT
TP45383_Hit	D	1	chr3	.	CAGCTATTATTCTCGTGCGAGTTTCGACCTCAACCCCTAAGAGTCTAGACAATGGTGCTGAGAC
TP45383_Query	D	1	chr3	.	CAGCTATTATTCTCGGGCGAGTTTCGACCTCAACCCCTAAGAGTCTAGACAATGGTGCTGAGAC

Name	Filter	Nb hit (Mt4.0)	Mt Chr (Mt4.0)	Ms Chr (Li et al., 2014)	Sequence
TP45770_Hit	D	1	chr3	.	CAGCTCAAATAAAAACAAATCGAAAATTAAGGCTGAAGGTATAAAGTAGAGTTTTGGTCATGAG
TP45770_Query	D	1	chr3	.	CAGCTCAAATAAAAACAAATCGAAAATTAAGGCAGAAGGTATAAAGTAGAGTTTTGGTCATGAG
TP45771_Hit	D+G	1	chr3	.	CAGCTCAAATATATATCCTCTTCATTGTCTTCTATCCATGTCATCCCTCCAATTCATTGAAGGG
TP45771_Query	D+G	1	chr3	.	CAGCTCAAATACATATCCTCTTCATTGTCTTCTATCCATGTCATCCCTCCAATTCATTGAAGGG
TP45932_Hit	D	1	chr3	.	CAGCTCAAGAATTGGCCATATTTATTAGATCTTTTCCAACAGAAAAAACATAGAAATCTTGTGA
TP45932_Query	D	1	chr3	.	CAGCTCAAGAATTGGCCATATTTATTAGATCTTTTCCAACAGAAAAAACAGAGAAATCTTGTGA
TP45967_Hit	D	1	chr3	.	CAGCTCAGGCCGAGAAAACACGAATATTTTTGCTCTATCTGTCAGATACAAGACTTGGCACAAA
TP45967_Query	D	1	chr3	.	CAGCTCAAGCCGAGAAAACACGAATATTTTTGCTCTATCTGTCAGATACAAGACTTGGCACAAA
TP4606_Hit	D	1	chr3	.	CAGCAACTCAAATTAACAATATGAAGGATCCCATTACAACGGATTTAGGGTCAATTGCCACACC
TP4606_Query	D	1	chr3	.	CAGCAACTCAAATTAACAATATGAAGGATCCCATTACAACGGATTTAGGGTCAATTGCAACACC
TP4614_Hit	D+G	1	chr3	.	CAGCAACTCAAGGCTTCGGATTTTACTGATCAAGAAGAATACAGTGAATGGCAGAAGAGGACTC
TP4614_Query	D+G	1	chr3	.	CAGCAACTCAAGGCTTCGGATTTTACTGATCAACAAGAATACAGTGAATGGCAGAAGAGGACTC
TP46175_Hit	D	1	chr3	.	CAGCTCACCAAACTCCTTCCACTTCATAGCTGGCTTGTTGTAATTCTCTGATCTCCATTCTGAT
TP46175_Query	D	1	chr3	.	CAGCTCACCAAACTCCTTCCACTTCATAGCTGGCTTGTTGTAATTCTCTGATCTCCACTCTGAT
TP46572_Hit	D	1	chr3	.	CAGCTCGGTGCTGTTTACATCAGTCTGCCGCCATATGAAGAAACTACAGCATGGCAACCGCCAG
TP46572_Query	D	1	chr3	.	CAGCTCAGTGCTGTTTACATCAGTCTGCCGCCATATGAAGAAACTACAGCATGGCAACCGCCAG
TP46644_Hit	D	1	chr3	.	CAGCTCATAGCACAAGCGCCTATCATGATAGGCGATTATATATAAGCTATTTCTATAGAAAAAG
TP46644_Query	D	1	chr3	.	CAGCTCATAGCACAAGCGCCTATCATGATAGGTGATTATATATAAGCTATTTCTATAGAAAAAG
TP46712_Hit	D	1	chr3	.	CAGCTCATCCCAATCATTTGAAATCCAATCTGAGTGACCAGAGTTCATCCTACTGTTGGATTCG
TP46712_Query	D	1	chr3	.	CAGCTCATCCCAATCATTTGAAATCCAATCTGAGAGACCAGAGTTCATCCTACTGTTGGATTCG
TP46727_Hit	D	1	chr3	.	CAGCTCATCGCGGTGACACAGATTCTGCCATTGAATGTTTAGCGAATCCATTGGTGGATGTTAA
TP46727_Query	D	1	chr3	.	CAGCTCATCGCGGTGACACAGATTCTGCCATTGAATGTTTAGCGAATCCATTGGTGAATGTTAA
TP46998_Hit	D+G	1	chr3	.	CAGCTCCAACAGTCCAACTGAAGTTTTGGAAAATCCTTTAATCCTGCGAGGTGACTTCGGTGCT
TP46998_Query	D+G	1	chr3	.	CAGCTCCAACAGTCCAACTGAAGGTTTGGAAAATCCTTTAATCCTGCGAGGTGACTTCGGTGCT
TP47190_Hit	D+G	1	chr3	.	CAGCTCCAGCATGCTCAATCTTCTCTACTAGAGGGTATAGAACTTCACCAAGCATCTATGAATC
TP47190_Query	D+G	1	chr3	.	CAGCTCCAGCATGCTCAATCTTCTCTACTAGAGGGTATAAAACTTCACCAAGCATCTATGAATC
TP47270_Hit	D	1	chr3	.	CAGCTCCATCGGATGTTTATAGGAACTTATGGTATGGTGGGCAAATGGTAACTGGTCCTCAGGC
TP47270_Query	D	1	chr3	.	CAGCTCCATCAGATGTTTATAGGAACTTATGGTATGGTGGGCAAATGGTAACTGGTCCTCAGGC
TP4728_Hit	D	1	chr3	.	CAGCAACTGATGTTATGAACAAGCAAATAGATGAACAGCATAATATGGGTATCCATTCAGAGGA
TP4728_Query	D	1	chr3	.	CAGCAACTGATGTTATGAACAAGCAAATAGATGAACAACATAATATGGGTATCCATTCAGAGGA
TP47417_Hit	D	1	chr3	.	CAGCTCCCATTTTGCTGAAGTGTATGAACGTCGAGGATGTTCAAAACCTGGTCTTTACGTTGAA
TP47417_Query	D	1	chr3	.	CAGCTCCCATTTTGCTGAAGCGTATGAACGTCGAGGATGTTCAAAACCTGGTCTTTACGTTGAA
TP47445_Hit	D	1	chr3	.	CAGCTTCCCTCTCTCAAGGAGCTTTTTATTTCAAACTGTAACGGAATAAAGATCATCGGCGAAG
TP47445_Query	D	1	chr3	.	CAGCTCCCCTCTCTCAAGGAGCTTTTTATTTCAAACTGTAACGGAATAAAGATCATCGGCGAAG
TP47477_Hit	D+G	1	chr3	.	CAGCTCCCTATATCATATGTATGAAATTATATAGAATCATAATGTATATTGTTTTTGTATTTGG
TP47477_Query	D+G	1	chr3	.	CAGCTCCCTATATCATATGTATGAAATTAGATAGAATCATAATGTATATTGTTTTTGTATTTGG
TP47530_Hit	D	1	chr3	.	CAGCTCCGAACTTAAACACCTGCCTTATGGGTTAGTATTGACAAATTATTTCACATGTAGCTAT
TP47530_Query	D	1	chr3	.	CAGCTCCGAACTTAAACACCTGCCTTATGGGTTAGTATCGACAAATTATTTCACATGTAGCTAT
TP47720_Hit	D	1	chr3	.	CAGCTCCTATGGCTATCTTCAACCTTATTTCCCATGACAGTGGTTCAGGGCCTGCCAAACAACA
TP47720_Query	D	1	chr3	.	CAGCTCCTATGGCTATCTTCAACCTTATTTCCCAAGACAGTGGTTCAGGGCCTGCCAAACAACA
TP47796_Hit	D	1	chr3	.	CAGCTCCTCGAGTTAAAGCAGAACTCTCCTTGCCCATAATGTATGCTCTTAAGTCTAAATCTTA
TP47796_Query	D	1	chr3	.	CAGCTCCTCGAGTTAAAGCAGAACTCCCCTTGCCCATAATGTATGCTCTTAAGTCTAAATCTTA
TP4783_Hit	D	1	chr3	.	CAGCAACTGTACTTCAGTTGAAAAACATAAAAATGTGTTCTAAGATACTCCTCAAAGAATATTG
TP4783_Query	D	1	chr3	.	CAGCAACTGTACTTCAGTTGAAAAACATAAAAATGAGTTCTAAGATACTCCTCAAAGAATATTG
TP47889_Hit	D	1	chr3	.	CAGCTCCTGCTTTGGCATCACAATCATTCATGAACAATCAAACGCCTGCAGTCCTTGTAGCTAG
TP47889_Query	D	1	chr3	.	CAGCTCCTGCTTTAGCATCACAATCATTCATGAACAATCAAACGCCTGCAGTCCTTGTAGCTAG
TP47903_Hit	D	1	chr3	.	CAGCTCCTGTGGAGAAGGCAATTAGACTTGTTGTGATTAAAGAGATTAAGGGTGCACAATATGG
TP47903_Query	D	1	chr3	.	CAGCTCCTGTGGAGAAGGCAATTAGACTTGTTGTGATTAAAGAGATTAAGGGCGCACAATATGG
TP47930_Hit	D+G	1	chr3	.	CAGCTCCTTCAATCTGTCTCCTTTCTTTGAGTTAGAGTACCTGGACAAACAAAACTTGTGTTTA
TP47930_Query	D+G	1	chr3	.	CAGCTCCTTCAACCTGTCTCCTTTCTTTGAGTTAGAGTACCTGGACAAACAAAACTTGTGTTTA
TP48514_Hit	D	1	chr3	.	CAGCTCGTGTCGTGAGATGTTTGGTCAAGTCCTATAACGAGCGAAACCCTCGTTTTGTGTTGCT
TP48514_Query	D	1	chr3	.	CAGCTCGTGTCGTGAGATGTTTGGTCAAGTCCTATAACGAGCGAAACCCTCGTTTTGTGGTGCT
TP48577_Hit	D	1	chr3	.	CAGCTCTAAATTGGAAAGATTATGTGCTTTGGACATGGTCAAACCTTCTTAAACTCCCCATAAT
TP48577_Query	D	1	chr3	.	CAGCTCTAAATTGGAAAGATTATGTGCTTTAGACATGGTCAAACCTTCTTAAACTCCCCATAAT

Name	Filter	Nb hit (Mt4.0)	Mt Chr (Mt4.0)	Ms Chr (Li et al., 2014)	Sequence
TP48597_Hit	D	1	chr3	.	CAGCTCTAAGCACATACTTGTGTTTATTAGTCAGAATGTATCATGTCTTTGTACGGGTGAAAGT
TP48597_Query	D	1	chr3	.	CAGCTCTAAGCACATACTTGTGTTTATTAGTCAGAATGTATCATGTCTTTGTACGGGCGAAAGT
TP48646_Hit	D	1	chr3	.	CAGCTTTACTATAGTGCAGTTCCTAACATATATAGTGAAAGTCCTCAAGTTATGATCAATCCGA
TP48646_Query	D	1	chr3	.	CAGCTCTACTATAGTGCAGTTCCTAACATATATAGTGAAAGTCCTCAAGTTATGATCAATCCGA
TP48842_Hit	D	1	chr3	.	CAGCTCTCAGTAGACTGACATGTACACTTACATCAACAAGTACCGCATGTAAAGCATCAAAATG
TP48842_Query	D	1	chr3	.	CAGCTCTCAGAAGACTGACATGTACACTTACATCAACAAGTACCGCATGTAAAGCATCAAAATG
TP48909_Hit	D	1	chr3	.	CAGCTCTCCGCCATGATTGATTCTCTGTCTCTAGTCTTGTCAAAAATTGTTCCAACTCCATCCT
TP48909_Query	D	1	chr3	.	CAGCTCTCCGCCATGATTGATTCTCTGTATCTAGTCTTGTCAAAAATTGTTCCAACTCCATCCT
TP49175_Hit	D	1	chr3	.	CAGCTCTGGGAAAGGTTGAGAAGCCAATAACTAAGGTCAAGTCTTTGTCATTTGGGGAGAGGGC
TP49175_Query	D	1	chr3	.	CAGCTCTGGGAAAGGTTGAGAAGCCAATAACTAAGGTCAAGTCTTTGTCATTTGGAGAGAGGGC
TP49179_Hit	D	1	chr3	.	CAGCTCTGGGATTTAAAACCGGATTTATCTCACTCTTTATGTCCTGATTCCAAATGGCAAGTCA
TP49179_Query	D	1	chr3	.	CAGCTCTGGGATTTAAAACCGGATTTATCTCACTCTTTATATCCTGATTCCAAATGGCAAGTCA
TP49283_Hit	D	1	chr3	.	CAGCTCTTACAAAACAACATTGGCATTGTAGCCATGCATATGCAATTGCTACACAACGACCGTG
TP49283_Query	D	1	chr3	.	CAGCTCTTACAAAACAACATTGGCATTGTAGCCATGCATATGCAATTACTACACAACGACCGTG
TP49322_Hit	D	1	chr3	.	CAGCTCTTCAAGGAGCATGGCAACTTTAGCAAGAACAAGAACTTCCAATCACGTATGTCTTCTT
TP49322_Query	D	1	chr3	.	CAGCTCTTCAAGGAGCATGGCAACTTTAGCAAGAACAAGAACTTCCAATCACGTATGCCTTCTT
TP49356_Hit	D+G	1	chr3	.	CAGCTCTTCGAGCTCAAATATCCGATTAACGCCACCAGAAAGCTCTACAAGCTTCCTATTCAAT
TP49356_Query	D+G	1	chr3	.	CAGCTCTTCGAGCTCAAATATCCGATTAACACCACCAGAAAGCTCTACAAGCTTCCTATTCAAT
TP49518_Hit	D+G	1	chr3	.	CAGCTCTTGTTCTAGCTCTAGACCAACGCGAAATGGCAGTTTCGTGTTTCTCAATGTCAAAGAA
TP49518_Query	D+G	1	chr3	.	CAGCTCTTGTTCTAGCTCTAGACCAACGCGAAATGGCAGTTTCATGTTTCTCAATGTCAAAGAA
TP49572_Hit	D	1	chr3	.	CAGCTCTTTGCAAGGGCTCTGGGCTTGTCGGTGGTCTTTACGCACCGAGTTTAATGATTGGTGC
TP49572_Query	D	1	chr3	.	CAGCTCTTTGCAAGGGCTCTGGGCTTGTAGGTGGTCTTTACGCACCGAGTTTAATGATTGGTGC
TP49628_Hit	D+G	1	chr3	.	CAGCTCTTTTTGTCCAATTGGCGCTTTTGTTCAATCAGTGACCTTGAAGATTCAATGATAATCA
TP49628_Query	D+G	1	chr3	.	CAGCTCTTTTTGTCCAACTGGCGCTTTTGTTCAATCAGTGACCTTGAAGATTCAATGATAATCA
TP49931_Hit	D	1	chr3	.	CAGCTGAACCTGGGAAAGCGGATTGGGAATTGGCATTGGTTGAGACAGCCAGCAATTTATCTAG
TP49931_Query	D	1	chr3	.	CAGCTGAACCTGGGAAAGCGGATTGGGAACTGGCATTGGTTGAGACAGCCAGCAATTTATCTAG
TP50113_Hit	D	1	chr3	.	CAGCTGAAGTAGAAGACTCAAAAAGCAATTTACCATCTTCTGATCAAATAGAAGAAATTTCTCA
TP50113_Query	D	1	chr3	.	CAGCTGAAGTAGAAGACTCAAAAAGCAATTTACCATCTTCCGATCAAATAGAAGAAATTTCTCA
TP50122_Hit	D	1	chr3	.	CAGCTGAAGTCATGTAATATTGTGTCACACTGTCAGCTACAGAAGGAGGAACTAATCTTGCGAG
TP50122_Query	D	1	chr3	.	CAGCTGAAGTCATGTAATACTGTGTCACACTGTCAGCTACAGAAGGAGGAACTAATCTTGCGAG
TP50180_Hit	D	1	chr3	.	CAGCTGAATCAATGTTCCTAGTTGTTACAGATACAGTTGTTGATTTACTGTGGTACATCAATTT
TP50180_Query	D	1	chr3	.	CAGCTGAATCAATGTTCCTAGTTGTTACAGATACAGTCGTTGATTTACTGTGGTACATCAATTT
TP50187_Hit	D+G	1	chr3	.	CAGCTGAATCTGGCTTTAAGCTGAAAACATACATAACATATACATTTCTTTTCTTTTACAATTT
TP50187_Query	D+G	1	chr3	.	CAGCTGAATCTGGCTTTAAGCTGAAAACATACATAACATATACATTTCATTTCTTTTACAATTT
TP50378_Hit	D+G	1	chr3	.	CAGCTGATGCAATGAAGTTATTTCTTCCACCTTCCTCTTTTGTTGAAGTTTTTTCCCAGAACTT
TP50378_Query	D+G	1	chr3	.	CAGCTGACGCAATGAAGTTATTTCTTCCACCTTCCTCTTTTGTTGAAGTTTTTTCCCAGAACTT
TP50408_Hit	D	1	chr3	.	CAGCTGACTTCAATATTGAGGCATGATTTGGGTCCAAGAGCTGACCCATGTTCTCACCAAATCT
TP50408_Query	D	1	chr3	.	CAGCTGACTTCAATATTGAGGCATGATTTGGGTCCAAGAGCTGACCCATGTTCTCACCAAATCG
TP50469_Hit	D	1	chr3	.	CAGCTGAGAGAGGAACCTCCGACCAAGGTTTTAACAGATAGGGTTCAAAGGAACATCTTATCTG
TP50469_Query	D	1	chr3	.	CAGCTGAGAGAGGAACCTCCAACCAAGGTTTTAACAGATAGGGTTCAAAGGAACATCTTATCTG
TP50506_Hit	D+G	1	chr3	.	CAGCTGAGCGTAAGTTTGGTCTGCTATCTGAATGTGATCATCCGTTCTGCGTATCGTGTATTAG
TP50506_Query	D+G	1	chr3	.	CAGCTGAGCGTAAGTTTGGTCTGCTATCTGAATGCGATCATCCGTTCTGCGTATCGTGTATTAG
TP50619_Hit	D+G	1	chr3	.	CAGCTGAGTCATCCTAATCTGGTGAGATTGATTGGTTACTGCTTAGAGGATATCCACCCCATTT
TP50619_Query	D+G	1	chr3	.	CAGCTGAGTCATCCTAATCTGGTGAGACTGATTGGTTACTGCTTAGAGGATATCCACCCCATTT
TP5069_Hit	D	1	chr3	.	CAGCAAGAACCCTTTAAAACCATATTAAAAAGGGAATATAGACCTCAGTTGGTGATGGCATTTG
TP5069_Query	D	1	chr3	.	CAGCAAGAACCCTTTAAAACCATATTAAAAAGGGAATATAGACCTCACTTGGTGATGGCATTTG
TP50810_Hit	D	1	chr3	.	CAGCTGATCTTGCAATGGCTTCGATATATTTTGTGGTAACATTAGTTAAGGCTTCAAGAGCATT
TP50810_Query	D	1	chr3	.	CAGCTGATCTTGCAATGGCTTCGATATATTTTGTGGCAACATTAGTTAAGGCTTCAAGAGCATT
TP51037_Hit	D	1	chr3	.	CAGCTGATTTAATAGGTTCTGAATTATTGTTGTTGTTGTCATTTTGAGAGTTGAAGAATTTAGT
TP51037_Query	D	1	chr3	.	CAGCTGATTTAATAGGTTCAGAATTATTGTTGTTGTTGTCATTTTGAGAGTTGAAGAATTTAGT
TP51147_Hit	D+G	1	chr3	.	CAGCTGGAACTAGGATGGGACTGAAGATAACAAGAAGAGGGGTTGCAATGATTAATCCTATGAC
TP51147_Query	D+G	1	chr3	.	CAGCTGGAACTAGGATAGGACTGAAGATAACAAGAAGAGGGGTTGCAATGATTAATCCTATGAC
TP51329_Hit	D	1	chr3	.	CAGCTGGAGTGCCGGACAAATCATATTTGACTGCAACATAATTACCAGTCTTTATTTTTAGTGA
TP51329_Query	D	1	chr3	.	CAGCTGGAGTGACGGACAAATCATATTTGACTGCAACATAATTACCAGTCTTTATTTTTAGTGA

Name	Filter	Nb hit (Mt4.0)	Mt Chr (Mt4.0)	Ms Chr (Li et al., 2014)	Sequence
TP51597_Hit	D+G	1	chr3	.	CAGCTGGGATACAACTTCTATCAAGGGTCATCTTGGTTCAGGGCATTTTCATTTTTGCACTCAT
TP51597_Query	D+G	1	chr3	.	CAGCTGGGATACAACTTCTATCAAGGGTCATCTTGGTTCAGGGCACTTTCATTTTTGCACTCAT
TP51609_Hit	D	1	chr3	.	CAGCTGGGCATATATGAATCATAGTTGCCATTAGATTCAGAACAGAAATTATATCATGTTCGTG
TP51609_Query	D	1	chr3	.	CAGCTGGGCATATATGAATCATAGTTGCCATTAGATTCAGAACAGAAATTATATCATGTTCCTG
TP51886_Hit	D	1	chr3	.	CAGCTGGTGCTGATGGTTCAAAGCAAAGCAGAAGCGAGAAGAAGAGCCGAAAAGCTATGCTTAA
TP51886_Query	D	1	chr3	.	CAGCTGGTGCTGATAGTTCAAAGCAAAGCAGAAGCGAGAAGAAGAGCCGAAAAGCTATGCTTAA
TP52008_Hit	D	1	chr3	.	CAGCTGGTTTTAATGTTTACAAGTATATTAAATTAACTGCTAATTACTATCGATATCTTTCAAG
TP52008_Query	D	1	chr3	.	CAGCTGGTTTTAATGTTTACAAGTATATTAAATTAACTGCTAATTACTATCAATATCTTTCAAG
TP52032_Hit	D	1	chr3	.	CAGCTGTAAACTTGTTACCATATGCTGTTCCTTACCGATTTGTGCCGTTCGGAGATGGCCACAA
TP52032_Query	D	1	chr3	.	CAGCTGTAAACTTGTTACCATATGCTGTTCCTTACCGATTTGTGCCATTCGGAGATGGCCACAA
TP52036_Hit	D	1	chr3	.	CAGCTGTAAATGGTAATCTCAAGGAGGATCAGATGATTAAGAAGATCGTTGTCTTCAGTGATAT
TP52036_Query	D	1	chr3	.	CAGCTGTAAATGGTAACCTCAAGGAGGATCAGATGATTAAGAAGATCGTTGTCTTCAGTGATAT
TP52148_Hit	D	1	chr3	.	CAGCTGTAGAAAATGAGCTGATTGAGTTATTTTGCATATGTAGAGTTAACATGTACGATGCTAC
TP52148_Query	D	1	chr3	.	CAGCTGTAGAAAATGAGCTGATTGAGTTATTTTGCATATGTAGAGTTAACATGTACAATGCTAC
TP52286_Hit	D	1	chr3	.	CAGCTGTCAATGCAAGACAACATGGCCAGTTTTGAAATACAACAAATAATATAAATTTTATTAA
TP52286_Query	D	1	chr3	.	CAGCTGTCAATGCAAGACAACATGACCAGTTTTGAAATACAACAAATAATATAAATTTTATTAA
TP52338_Hit	D	1	chr3	.	CAGCTGTCCAAGGTAATTGATTTCTGTCTACGAAATACAAGGTTAATGTTAGAGCAACAACCCT
TP52338_Query	D	1	chr3	.	CAGCTGTCCAAGGTAATTGATTTCTGTCTACGAAATACAAGGTTAATGTTAAAGCAACAACCCT
TP5239_Hit	D	1	chr3	.	CAGCAAGACTGACCAACCAAGTTTGCGTTTTAGTAGATAAAAAATGAAATTCTTTCAAATAACA
TP5239_Query	D	1	chr3	.	CAGCAAGACTGACCAACCAAGTTTGCATTTTAGTAGATAAAAAATGAAATTCTTTCAAATAACA
TP52466_Hit	D	1	chr3	.	CTGCTGTGACGATAAATAATTAGGTGGACTACATATTACCTGCAAATCGTCTATACTTTATAGC
TP52466_Query	D	1	chr3	.	CAGCTGTGACGATAAATAATTAGGTGGACTACATATTACCTGCAAATCGTCTATACTTTATAGC
TP52529_Hit	D	1	chr3	.	CAGCTGTGCAAATTGGAAAAGCATGTGGGGCTATTGTCATTGCTGTTGCTAGGTACTCTTCTGC
TP52529_Query	D	1	chr3	.	CAGCTGTGCAAATTGGAAAAGCATGTGGGGCTATTGTCATTGCTGTTGCTAGGTACTCTTCTCC
TP52553_Hit	D	1	chr3	.	CAGCTGTGCTAAATGCAATGGTATGCATAAAACATGTTCGGATTACTTGATACATATAATCTTC
TP52553_Query	D	1	chr3	.	CAGCTGTGCTAAATGCAATGGTATGCATAAAACATGTTCAGATTACTTGATACATATAATCTTC
TP526_Hit	D	1	chr3	.	CAGCAAAACTTTTCAACAAAACAGCAAGCAGAACCATATCCTACCTCCAACTTGATTGTCCACA
TP526_Query	D	1	chr3	.	CAGCAAAACTTTTCAACAAAACAGCAAGCAGAACCATATCCTAACTCCAACTTGATTGTCCACA
TP52655_Hit	D+G	1	chr3	.	CAGCTGTGTACAGATCCTGAATTGGTTCGATTACCGGAATCACATATGCATTGTAAGTAGTCTT
TP52655_Query	D+G	1	chr3	.	CAGCTGTGTACAGATCCTGAATTGGTTCGATTACCGGAATCACACATGCATTGTAAGTAGTCTT
TP52669_Hit	D	1	chr3	.	CAGCTGTGTCATCAAATATTGGTGTGAAGATCTTAACATATTGGATGTGTTTCGTTCGTATGTT
TP52669_Query	D	1	chr3	.	CAGCTGTGTCATCAAATATTGCTGTGAAGATCTTAACATATTGGATGTGTTTCGTTCGTATGTT
TP52802_Hit	D	1	chr3	.	CAGCTGTTAGTAAAGACCATGAATCTAATTTAAGGGCGTTTACTGTAAGGAAAACAGAATCATA
TP52802_Query	D	1	chr3	.	CAGCTGTTAGTAAAGACCACGAATCTAATTTAAGGGCGTTTACTGTAAGGAAAACAGAATCATA
TP52826_Hit	D	1	chr3	.	CAGCTGTTATTATTGATTGAAGAAATATTTGCTCTACATCACGATAACTTTGCGATTGTTGAAT
TP52826_Query	D	1	chr3	.	CAGCTGTTATTATTGATGGAAGAAATATTTGCTCTACATCACGATAACTTTGCGATTGTTGAAT
TP52925_Hit	D	1	chr3	.	CAGCTGTTGACAAGCTTTGTCTAAGTAAACTCAATGATAACTTTTTGGCTGTCATAGTTCCCAG
TP52925_Query	D	1	chr3	.	CAGCTGTTGACAAGCTTTGTCTAAGTAAACTCAACGATAACTTTTTGGCTGTCATAGTTCCCAG
TP52957_Hit	D	1	chr3	.	CAGCTGTTGCCACTGTCCTACCATCTGGGCTCTGCGCCATATGAAGAACTCTTGAAGTGTGACC
TP52957_Query	D	1	chr3	.	CAGCTGTTGCCACTGTCCAACCATCTGGGCTCTGCGCCATATGAAGAACTCTTGAAGTGTGACC
TP53144_Hit	D	1	chr3	.	CAGCTGTTTTAAGATCTTCATGAATCACTTCAGTCAGGTATGTGATAAGGTTCATGGTTAATCC
TP53144_Query	D	1	chr3	.	CAGCTGTTTTAAGATCTTCATGAATCACTTCAGTCAGGTACGTGATAAGGTTCATGGTTAATCC
TP5326_Hit	D	1	chr3	.	CAGCAAGAGGAAATAGTATACTAGAGAGGACTTTAGATGACAGTTCCACTCGGAATCAATGCAT
TP5326_Query	D	1	chr3	.	CAGCAAGAGGAAATAGTATACTAGAGAGCACTTTAGATGACAGTTCCACTCGGAATCAATGCAT
TP53377_Hit	D	1	chr3	.	CAGCTTAAGATCATTGGCAATGTAATGCTCAAATGTAACTTCAATACTGTGGACCTAGTTTATC
TP53377_Query	D	1	chr3	.	CAGCTTAAGATCATTGGCAATGTAATGCTCAAATGTAACTTCAATACCGTGGACCTAGTTTATC
TP53632_Hit	D	1	chr3	.	CAGCTTACCCTCAACGACATTACTGCCAAATGGTCATGAAATCCTACCAATTATGGGAAGAAGC
TP53632_Query	D	1	chr3	.	CAGCTTACCCTCAACAACATTACTGCCAAATGGTCATGAAATCCTACCAATTATGGGAAGAAGC
TP53676_Hit	D	1	chr3	.	CAGCTTACGTTAGCGAAGAGGCTTGCTTCTTTGGCAAGTCTTGTGTCGGAACCTGTTCTCACTC
TP53676_Query	D	1	chr3	.	CAGCTTACGTTAGCGAAGAGGCTTGCTTCTTTGGCAAGTCTCGTGTCGGAACCTGTTCTCACTC
TP53695_Hit	D	1	chr3	.	CAGCTTACTGCCTGCAACATATGCACCAGTTGGACCCTCCTTTCGCACATAGTGACCTAAATAC
TP53695_Query	D	1	chr3	.	CAGCTTACTGCCTGCAACATATGCACCAGTTGGACCCTCCTTTCGCACATAGCGACCTAAATAC
TP53864_Hit	D+G	1	chr3	.	CAGCTTAGTTTATTTTAGTTAAAAGGGTAAGCACAATGCATAAGGTTTAAACTTTAAACTGATT
TP53864_Query	D+G	1	chr3	.	CAGCTTAGTTTATTTTAGTTAAAAGGGTAAGCACAATGCATAAGGTTTAAACTTCAAACTGATT

Name	Filter	Nb hit (Mt4.0)	Mt Chr (Mt4.0)	Ms Chr (Li et al., 2014)	Sequence
TP53909_Hit	D	1	chr3	.	CAGCTTATTAGCTAGATAAATGTAGAGATATTTTAAAAGCTTATTCAAAGTGTAGTAGTGGTGA
TP53909_Query	D	1	chr3	.	CAGCTTATAAGCTAGATAAATGTAGAGATATTTTAAAAGCTTATTCAAAGTGTAGTAGTGGTGA
TP54022_Hit	D+G	1	chr3	.	CAGCTTATATTAGGTTAGGCATATTCAATATTTTTCTTTTAAATTGGTTATGACAAGCTGTATT
TP54022_Query	D+G	1	chr3	.	CAGCTTATATTAGGTTAGGCATATTCAATATTTTTCTTTTAAATTGGTTATGACAAACTGTATT
TP54114_Hit	D	1	chr3	.	CAGCTTATGAACTTGAAGCCTAGCTATTGGACTTTGGGTTCTGGTCAGTAGATGATGAGTTCTC
TP54114_Query	D	1	chr3	.	CAGCTTATGAACTTAAAGCCTAGCTATTGGACTTTGGGTTCTGGTCAGTAGATGATGAGTTCTC
TP54116_Hit	D	1	chr3	.	CAGCTTATGAAGCACTGACATAGACACCAGAAATGACACTGAAATACTTTAATGTAACCATATG
TP54116_Query	D	1	chr3	.	CAGCTTATGAAGCACTGACATAGACACCAGAAACGACACTGAAATACTTTAATGTAACCATATG
TP54144_Hit	D+G	1	chr3	.	CAGCTTATGATATTTGTCGATTATGGTGGCTCCATCTTCCCCATCATTTCTCATGCTCCATGGA
TP54144_Query	D+G	1	chr3	.	CAGCTTATGATATTGGTCGATTATGGTGGCTCCATCTTCCCCATCATTTCTCATGCTCCATGGA
TP54150_Hit	D	1	chr3	.	CAGCTTATGATGCAACAACGCTCGTATTTGTTGCTCCCTATTTCCACGCTTTCCTCCCACGTTG
TP54150_Query	D	1	chr3	.	CAGCTTATGATGCAACAACGCTCGTATTTATTGCTCCCTATTTCCACGCTTTCCTCCCACGTTG
TP54200_Hit	D	1	chr3	.	CAGCTTATGTACTATCGTTAACGTGTTGGCTTCACCAGTCGCGTTGTTGCCTTCTGGTGGCAAG
TP54200_Query	D	1	chr3	.	CAGCTTATGTACTATCGTTAACGTGTTGGCTTCACCAGTCACGTTGTTGCCTTCTGGTGGCAAG
TP54455_Hit	D	1	chr3	.	CAGCTTCAACACAGCGTTTCACGATGTTTAGGTTATCGGCATAGGGAAGAAGGTGGTGGCAAGA
TP54455_Query	D	1	chr3	.	CAGCTTCAACACAGCGTTTCACGATGTTTAGGTCATCGGCATAGGGAAGAAGGTGGTGGCAAGA
TP54471_Hit	D	1	chr3	.	CAGCTTCAACATCAGCTAACAGTTCCCTTCCTACCTACTCATAATGGCAGGTAGAGAGCAAGGA
TP54471_Query	D	1	chr3	.	CAGCTTCAACATCAGCTAACAGTTCCCTTCCTACCTACTCATAATGGCAGGGAGAGAGCAAGGA
TP54521_Hit	D+G	1	chr3	.	CAGCTTCAAGAAGCAGGTAATATACCACTTGATATTAAGGTTAATCAGAATGTGAGGGTTAAGC
TP54521_Query	D+G	1	chr3	.	CAGCTTCAAGAAGCAGGTAATATACCACTTGATATTAAGGTTAATCAAAATGTGAGGGTTAAGC
TP54538_Hit	D+G	1	chr3	.	CAGCTTCAAGCCCAATATCTGTTACTCCAGGGCACAGCCCAATTGTGAGCGACTTTAGGTGCTG
TP54538_Query	D+G	1	chr3	.	CAGCTTCAAGCCCAATATCTGTTACTCCAGGGCACAGCCCAATTGTGAGCGACGTTAGGTGCTG
TP54789_Hit	D	1	chr3	.	CAGCTTCAGAAGATCCAACCTTGTCTCCTTTCTTAATGAGTTCCACAGGGGTAATAATTTCCAC
TP54789_Query	D	1	chr3	.	CAGCTTCAGAAGATCCAACCTTGTCTCCTTTCTTAATGAGTTCCACAGGGGAAATAATTTCCAC
TP54869_Hit	D	1	chr3	.	CAGCTTCAGCTTCTCAAGTTGTCAAGAGAATAGGGGAAGGAAAATTGAATGAACAAGAAAAGCC
TP54869_Query	D	1	chr3	.	CAGCTTCAGCTTCTCAAGTTGTCAAGAGAATAGGAGAAGGAAAATTGAATGAACAAGAAAAGCC
TP55165_Hit	D+G	1	chr3	.	CAGCTTCCAAAAGGAAAAGAAAAGCATGGTCAGAGGAAGAGGACAATTTGCTACGGGGTGCTGA
TP55165_Query	D+G	1	chr3	.	CAGCTTCCAAAAGGAAAAGAAAAGCATGGTCAGAGGAAGAGGACAATTTGCTAAGGGGTGCTGA
TP55167_Hit	D	1	chr3	.	CAGCTTCCAAACCCATCTGGTATCTTCCCAGAAAAACTATTCCTTGCCAAGTTAATGCTCCGCA
TP55167_Query	D	1	chr3	.	CAGCTTCCAAACCCATCTGGTATCTTCCCAGAAAAACTATTCCTCGCCAAGTTAATGCTCCGCA
TP55183_Hit	D+G	1	chr3	.	CAGCTTCTAACTTTTGTTTCTTTTTAGGCCTGGTCTTTTTTTCAGAAGCAAGGACATTATGATT
TP55183_Query	D+G	1	chr3	.	CAGCTTCCAACTTTTGTTTCTTTTTAGGCCTGGTCTTTTTTTCAGAAGCAAGGACATTATGATT
TP55239_Hit	D	1	chr3	.	CAGCTTCCAGGTACTTAATCTAGCTAGAACAATCCTTAGTCAAATTTTATTATTTTACCTCCCA
TP55239_Query	D	1	chr3	.	CAGCTTCCAGGTACTTAATCCAGCTAGAACAATCCTTAGTCAAATTTTATTATTTTACCTCCCA
TP55443_Hit	D	1	chr3	.	CAGCTTCCTGATGGTGGTTACAGAGCACATTGCAAGGGCGCCTCTGAAATAATTCTTGCTGAAA
TP55443_Query	D	1	chr3	.	CAGCTTCCTGATGGTGGTTACAGAGCACATTGCAAGGGCGCCTCTGAAATAATTCGTGCTGAAA
TP55502_Hit	D	1	chr3	.	CAGCTTCGAAAAAGACGACGTCATCACCGTCTTCTTCTTGTTGTTCCCCTCTCGACTCTTCCCG
TP55502_Query	D	1	chr3	.	CAGCTTCGAAAAAGACGACGTCATCACCGTCTTCTTCTTGTTGTCCCCCTCTCGACTCTTCCCG
TP55604_Hit	D	1	chr3	.	CAGCTTCGGAGGTACGCTGATGCTCGATCACCTTTTTCAAATACAAAAAATTATACTGTGTTAA
TP55604_Query	D	1	chr3	.	CAGCTTCGGAGGTACGCTGATGCTCGATCACCTTTTTCAAATACAAAAAATTATAATGTGTTAA
TP55694_Hit	D	1	chr3	.	CAGCTTCTAAAAATTTGGCTTTACATAACGGGCAAAATTTTGAGAATAGGTTTGGTGGCGATAA
TP55694_Query	D	1	chr3	.	CAGCTTCTAAAAATTTGGCTTTACATAACAGGCAAAATTTTGAGAATAGGTTTGGTGGCGATAA
TP56570_Hit	D	1	chr3	.	CAGCTTCTTCGATTTTGGTGGAGAAACAATCAACTCTATTCAACTGGCTGGCTTGGGTCTCTAA
TP56570_Query	D	1	chr3	.	CAGCTTCTTCGATTTTGGTGGAGAAACAATCAACTCTATTCAACTGGCTGGCTTGGCTCTCTAA
TP56620_Hit	D	1	chr3	.	CAGCTTCTTGCAGTACGTAATTCTGTTTCTCGTTTTTATTGTTCTTTTTTGTAACAACCTGAGA
TP56620_Query	D	1	chr3	.	CAGCTTCTTGCAGTACGTAATTCTGTTTCTCGTTTTTATTGTCCTTTTTTGTAACAACCTGAGA
TP56820_Hit	D	1	chr3	.	CAGCTTGAACAGCACTATAAATGGCCAGAGCATCATTCCCATCTACACGAATGCTACGGACTCC
TP56820_Query	D	1	chr3	.	CAGCTTGAACAGCACCATAAATGGCCAGAGCATCATTCCCATCTACACGAATGCTACGGACTCC
TP56844_Hit	D	1	chr3	.	CAGCTTGAACTGATTGTGCGAATACAATTGCAGTTATACAGTACTGTCACACTGAGGCAAGGGG
TP56844_Query	D	1	chr3	.	CAGCTTGAACTGATTGTGCAAATACAATTGCAGTTATACAGTACTGTCACACTGAGGCAAGGGG
TP57_Hit	D	1	chr3	.	CAGCAAGAAACTTTTTTCTATTTATAGCTATGGTGATTATGTGCAATGGTTTGCATTTTCTGAC
TP57_Query	D	1	chr3	.	CAGCAAAAAAACTTTTTTCTATTTATAGCTATGGTGATTATGTGCAATGGTTTGCATTTTCTGAC
TP57025_Hit	D+G	1	chr3	.	CAGCTTGACTTTGGAAATCAAGTCCCAAAAGTCTCACATCCCTGAGATTAATCAAAAATCTCAA
TP57025_Query	D+G	1	chr3	.	CAGCTTGACTTTGGAAATCAAGTCCCAAAAGTTTCACATCCCTGAGATTAATCAAAAATCTCAA

Name	Filter	Nb hit (Mt4.0)	Mt Chr (Mt4.0)	Ms Chr (Li et al., 2014)	Sequence
TP57194_Hit	D	1	chr3	.	CAGCTTGATCCCACCAAATCCATTTTGATGATCATCTGGGCTTGCCTCCTCTTTGAAACTTGGT
TP57194_Query	D	1	chr3	.	CAGCTTGATCCCACCAAATCCATTTTGATGATCATCTGGGCTCGCCTCCTCTTTGAAACTTGGT
TP57352_Hit	D	1	chr3	.	CAGCTTGCACTGGGTTTTGATCGCCATTTGAGTTATGGACCATCATTTGCTGGTGCGGAGATCG
TP57352_Query	D	1	chr3	.	CAGCTTGCACTGGGTTTTGATCGCCATTTGAGTTATGGACCATCATTTGCTGGTGCCGAGATCG
TP57409_Hit	D	1	chr3	.	CAGCTTGCATTTTTCCATATAATTCAGAGCTGGTACTTTGTTGTTCCATGCATTCCTTGGCGCT
TP57409_Query	D	1	chr3	.	CAGCTTGCATTTTTCCATATAATTCAGAGCTGGTACTCTGTTGTTCCATGCATTCCTTGGCGCT
TP57491_Hit	D	1	chr3	.	CAGCTTGCGGAGAAGAATCGTGTCCCAAGAACTATTGTGTTCTGCAACAAAGTATTCTGAACTC
TP57491_Query	D	1	chr3	.	CAGCTTGCGGAGAAGAATCGCGTCCCAAGAACTATTGTGTTCTGCAACAAAGTATTCTGAACTC
TP57615_Hit	D+G	1	chr3	.	CAGCTTGGAAAGCCACCTCTTCAGAAGTACGAAATTAATTGATTAATTAATTCACTAGTAATTA
TP57615_Query	D+G	1	chr3	.	CAGCTTGGAAAGCCACCTCTTCAGAAGTACAAAATTAATTGATTAATTAATTCACTAGTAATTA
TP57670_Hit	D+G	1	chr3	.	CAGCTTGGAGAGCCTCCCACAACTTCTCTTTCTGGTTCCCCTTTTTGCAATCCTCACGGTAACC
TP57670_Query	D+G	1	chr3	.	CAGCTTGGAGAGCCTCCCACAACTTCTCTTCCTGGTTCCCCTTTTTGCAATCCTCACGGTAACC
TP57675_Hit	D+G	1	chr3	.	CAGCTTGGAGCATAATCTCCCTGATAGCCCTTTTGCTTCCCCTACTAAAGCTATTGGTGACTAC
TP57675_Query	D+G	1	chr3	.	CAGCTTGGAGCATAATCTCCCTGATAGCCCTTTTGCTTCCCCTACTAAAGCTATTGGAGACTAC
TP5781_Hit	D+G	1	chr3	.	CAGCAAGCGGTAATATCAATGAGGATTTACCGCAATCAGGAGTCTCCGATCCAATGGTTCGCAA
TP5781_Query	D+G	1	chr3	.	CAGCAAGCGGTAACATCAATGAGGATTTACCGCAATCAGGAGTCTCCGATCCAATGGTTCGCAA
TP57844_Hit	D	1	chr3	.	CAGCTTGGTCATTACTATCATTGGTATGTTTTAATGTTGGATACCAAAGTTTGTTTTTGATTTT
TP57844_Query	D	1	chr3	.	CAGCTTGGTCATTACTATCATTGGTATGTTTTAATGTTGGATACCAAAGTTTGTTTGTGATTTT
TP57992_Hit	D	1	chr3	.	CAGCTTGTACTGGACTAAGTGCCTTCATTATAACACTATCGCCTCCAATACGATTTGGTCCAGA
TP57992_Query	D	1	chr3	.	CAGCTTGTACTGGACTAAGTGCCTTCATTATAACACTATCACCTCCAATACGATTTGGTCCAGA
TP58032_Hit	D	1	chr3	.	CAGCTTGTATCTTAGATTGTAAACTACTAATATTTGAACTAGCAAATCAATGATTTAGTACCTG
TP58032_Query	D	1	chr3	.	CAGCTTGTATCTTAGATTGTAAACTACTAATATTTGAACTAGAAAATCAATGATTTAGTACCTG
TP58096_Hit	D	1	chr3	.	CAGCTTGTCCGTACTATTTCAACCGTGACATCTGATAATCATCGGCAACTTCTGGAAAATACTG
TP58096_Query	D	1	chr3	.	CAGCTTGTCCGTACTATTTCAACCGTGACATCTGATAATCATCGGCAACTTATGGAAAATACTG
TP58127_Hit	D	1	chr3	.	CAGCTTGTCTCTCCCCGGTGACCATCCAGCATACCACCTCTGCAAGATTTCATGCATAAGTAAC
TP58127_Query	D	1	chr3	.	CAGCTTGTCTCTCCCCGGTGACCATCCAGCATGCCACCTCTGCAAGATTTCATGCATAAGTAAC
TP58167_Hit	D	1	chr3	.	CAGCTTGTGATGAGATGGTAGTGTGTATTACTTAACGAAAACTTTGATGTTGTGTTTTTTTCTT
TP58167_Query	D	1	chr3	.	CAGCTTGTGATGAGATGGTAGTGTGTATTACTTAACGAAAACTTAGATGTTGTGTTTTTTTCTT
TP582_Hit	D+G	1	chr3	.	CAGCAAAAGACTAGGTATCAGATTAATCAACTCACATAATAATCCTACACTTTTTGTCTTCTTC
TP582_Query	D+G	1	chr3	.	CAGCAAAAGACTAGGTATCAGATTAATCAACTCAAATAATAATCCTACACTTTTTGTCTTCTTC
TP58598_Hit	D	1	chr3	.	CAGCTTTACAGAACTCAAAGAGTTTGTAAATTTTGTATCTCCCGATAATATTATACCAAGTGTG
TP58598_Query	D	1	chr3	.	CAGCTTTACAGAACTCAAAGAGTTTGTAAATTTTGTATCTCCCGATAACATTATACCAAGTGTG
TP58787_Hit	D	1	chr3	.	CAGCTTTATCAGTTCCCATGCTTGGAGTTGAGTTGCCCAGGCTATCAAGCTCAATTTCTGTACC
TP58787_Query	D	1	chr3	.	CAGCTTTATCAGTTCCCATGCTTGGAGTTGAGTTGCCCAGGCCATCAAGCTCAATTTCTGTACC
TP58810_Hit	D	1	chr3	.	CAGCTTTATGCAAAATCCTTCATTATATTGCTTATATTCATGGTGTATATTGAATAAGCCTTAT
TP58810_Query	D	1	chr3	.	CAGCTTTATGCAAAATCCTCCATTATATTGCTTATATTCATGGTGTATATTGAATAAGCCTTAT
TP58823_Hit	D	1	chr3	.	CTGCTTTATGTACCGGCACTTCACATTGAATTCGTATCCAGTGTCTGATATCAACACGACACCG
TP58823_Query	D	1	chr3	.	CAGCTTTATGTACCGGCACTTCACATTGAATTCGTATCCAGTGTCTGATATCAACACGACACCG
TP59018_Hit	D	1	chr3	.	CAGCTTTCATCAATCTATCAAACGAGCTCAATGCAGAAGGGTGCTTTGCCTAGTAATAATGCAT
TP59018_Query	D	1	chr3	.	CAGCTTTCATCAATCTATCAAACGAGCTCAATGCAGAAGGGTGCTTTACCTAGTAATAATGCAT
TP59065_Hit	D	1	chr3	.	CAGCTTTCCAATGCTGGAGGAAGACTTGTTATCAGCAGTGATGGAGTCTGGGATGCTCTAGCTG
TP59065_Query	D	1	chr3	.	CAGCTTTCCAATGCTGGAGGAAGACTTGTTATCAGCAGTGATGGAGTCTGGGATGCTCTAACTG
TP59128_Hit	D	1	chr3	.	CAGCTTTCGCAGAACCATGTTGTCACCTATCTGATCCACACTCGCTTCCAGTTTCACAGATTCA
TP59128_Query	D	1	chr3	.	CAGCTTTCGCAGAACCATGTTGTCACCTATCTGATCCACACTCGCTTCCAGTTCCACAGATTCA
TP59235_Hit	D	1	chr3	.	CAGCTTTCTGTTGCCGAGATCACCAACAGTGCTTTTGAACCTTCTTCCATGATGGCTAAGTGTG
TP59235_Query	D	1	chr3	.	CAGCTTTCTGTTGCCGAGATCACCAACAGTGCTTTTGAACCTTCTTCCATGATGGCCAAGTGTG
TP59402_Hit	D	1	chr3	.	CAGCTTTGAGGTATTTTTGTGATGTTTTGAATTCCCTGGCACAAAGACTGTGAAGGCCAAGTTG
TP59402_Query	D	1	chr3	.	CAGCTTTGAGGTATTTTTGTGATGTTTTGAATTCCCTAGCACAAAGACTGTGAAGGCCAAGTTG
TP5944_Hit	D	1	chr3	.	CAGCAAGGAAGAGAGCCCCTGGATGTTGATTTTTGTTCGATGACAATGGATGGAACACTAGACA
TP5944_Query	D	1	chr3	.	CAGCAAGGAAGAGAGCCACTGGATGTTGATTTTTGTTCGATGACAATGGATGGAACACTAGACA
TP59526_Hit	D	1	chr3	.	CAGCTTTGCTGAAATTTAAGGTGTGGAATTTCCTTGATTCGTCATTGGCCAGCCAAATGAATAA
TP59526_Query	D	1	chr3	.	CAGCTTTGCTGAAATATAAGGTGTGGAATTTCCTTGATTCGTCATTGGCCAGCCAAATGAATAA
TP59675_Hit	D	1	chr3	.	CAGCTTTGTAACACGAGTCAAGTGGAATCAGCGCGTATCCATACATGTTATAACAGAACTCTAA
TP59675_Query	D	1	chr3	.	CAGCTTTGTAACACGAGTCAAGCGGAATCAGCGCGTATCCATACATGTTATAACAGAACTCTAA

Name	Filter	Nb hit (Mt4.0)	Mt Chr (Mt4.0)	Ms Chr (Li et al., 2014)	Sequence
TP59692_Hit	D	1	chr3	.	CAGCTTTGTATGTACAGTCTCCATGCATTCCACGACGGATTTTTTTTTTTGTGCAAAAATCATA
TP59692_Query	D	1	chr3	.	CAGCTTTGTATGTACAGTCTCCATGCATTCCACGACGGATTTTTTTTTTTGTGCAAAAATAATA
TP59814_Hit	D+G	1	chr3	.	CAGCTTTTAACTACCTTGCAAAAGCATTTTTCGGAACAAACCCTACTGAAACAAATCTCAAAAC
TP59814_Query	D+G	1	chr3	.	CAGCTTTTAACTACCTTGCAAAAGCATTTTTCGGAACAAACCCTACCGAAACAAATCTCAAAAC
TP59926_Hit	D	1	chr3	.	CAGCTTTTCAACAAACTTGCTTCCAGATCTGCCAAATACAAACCTGGGGTAAATTTAAACTGTT
TP59926_Query	D	1	chr3	.	CAGCTTTTCAACAAACTTGCTTCCAGATCTGCCAAATACAAACCTGGGGTAAATTTAAACTATT
TP60084_Hit	D+G	1	chr3	.	CAGCTTTTGAGCAAATTCGCCCATAGCATCTGCCACCCCTCTTTCTGAAATTCTATTCATTGCT
TP60084_Query	D+G	1	chr3	.	CAGCTTTTGAGCAAATTCGCCCATAGCATCTGCCACCCCTCTTTCTGAAATTCTATTCATTGCG
TP60192_Hit	D	1	chr3	.	CAGCTTTTGGAGGAAATGGCAACATTACTCGAAGCTTCAGCTATGGAGGTGTCCTTTCTTGTTT
TP60192_Query	D	1	chr3	.	CAGCTTTTGGAGGAAATGGCAACATTACTCGAAGCTTCAGATATGGAGGTGTCCTTTCTTGTTT
TP60203_Hit	D	1	chr3	.	CAGCTTTTGGGATGAGATCCAATCCCATTTAACAATTGTTATAACCGATTTGATTACATTCTGT
TP60203_Query	D	1	chr3	.	CAGCTTTTGGGATGAGATCCAATCCCATTTAACAATTGTTACAACCGATTTGATTACATTCTGT
TP60276_Hit	D	1	chr3	.	CAGCTTTTGTTTGTTCTTTGATTGAATTTAGTAGTTTAATTTTGTCTTTTCATAGGTTTACAAA
TP60276_Query	D	1	chr3	.	CAGCTTTTGTTTGTTCTTTGATTGAATTTAGTAGTTTAATTTTGCCTTTTCATAGGTTTACAAA
TP604_Hit	D	1	chr3	.	CAGCAAAAGAGGTATGTTTTTGTATTTGATGACGTATGGAACATAAGTTTTTGGGATGACATTG
TP604_Query	D	1	chr3	.	CAGCAAAAGAGGTACGTTTTTGTATTTGATGACGTATGGAACATAAGTTTTTGGGATGACATTG
TP61001_Hit	D	1	chr3	.	CTGCAAACAAGAAAACACAATTTGATATTGCATGAATTCATTGAACTGACAGTAATTTGTAGAT
TP61001_Query	D	1	chr3	.	CTGCAAACAAGAAAACACAATTTGATATTGCATGAATTCATCGAACTGACAGTAATTTGTAGAT
TP61109_Hit	D	1	chr3	.	CTGCAAATCTTATTAGAGGCTTATCTTTTGAACCTGATGCTAATATATGGGGTGCACTACTTGG
TP61109_Query	D	1	chr3	.	CTGCAAACCTTATTAGAGGCTTATCTTTTGAACCTGATGCTAATATATGGGGTGCACTACTTGG
TP61169_Hit	D	1	chr3	.	CTGCAAACTGAGCAAACTCTATATCATAGTCCATTCCATCCATCAAATTCGATGACAGTAAAGC
TP61169_Query	D	1	chr3	.	CTGCAAACTGAGCAAACACTATATCATAGTCCATTCCATCCATCAAATTCGATGACAGTAAAGC
TP61185_Hit	D	1	chr3	.	CTGCAAACTTATCCTCGTTTCCTATCTTTTGAGTGTAAACTCTGCCGCCAGGACGGTTCCTACC
TP61185_Query	D	1	chr3	.	CTGCAAACTTATCCTCGTTTCCTATCTTTTGAGTGTAAACTCTGCCGCCAGAACGGTTCCTACC
TP61383_Hit	D+G	1	chr3	.	CTGCGAAGGTAGAAATTGCTGGTTTGAAATAGGACCTGCTAACCCCTGTGATTGAGGTGGTCTC
TP61383_Query	D+G	1	chr3	.	CTGCAAAGGTAGAAATTGCTGGTTTGAAATAGGACCTGCTAACCCCTGTGATTGAGGTGGTCTC
TP61453_Hit	D	1	chr3	.	CTGCAAATAATTTGGCTCCTGCTTCCGAAGACTGTAGCAGTAGCAGTCTTAAAGGAAATTTAGA
TP61453_Query	D	1	chr3	.	CTGCAAATAAATTGGCTCCTGCTTCCGAAGACTGTAGCAGTAGCAGTCTTAAAGGAAATTTAGA
TP61530_Hit	D	1	chr3	.	CTGCAAATATATCAATGACGAAGTCAATAGGAGTATCTATTGGATTGATGTGTTCAACCACTCT
TP61530_Query	D	1	chr3	.	CTGCAAATATATCAACGACGAAGTCAATAGGAGTATCTATTGGATTGATGTGTTCAACCACTCT
TP62208_Hit	D	1	chr3	.	CTGCAACAGTGAGGTGAGAACTTGGACCTAAATTCCATATATGCTTTATAGTAACTGCAATGTC
TP62208_Query	D	1	chr3	.	CTGCAACAGTGAGGTGAGAACTTGGACCTAAATTCCATATATGCTTTATAGTAACTGCAATATC
TP62440_Hit	D	1	chr3	.	CTGCAACCGTAATTTAAAACCATGATACAATCCATTCAAAATAAAAATAGTTCTTACAGGTTTT
TP62440_Query	D	1	chr3	.	CTGCAACCGTAATTTAAAACCATGATACAAGCCATTCAAAATAAAAATAGTTCTTACAGGTTTT
TP62804_Hit	D	1	chr3	.	CTGCAACTGCACTAAACTCTGGTGTTTATCCAAATGGTCCGCCTGTGCCTGTTCGTTCTGCTAA
TP62804_Query	D	1	chr3	.	CTGCAACTGCACTAAACTCTGGTGTTTATCCAAATGGTCCGCCTGTACCTGTTCGTTCTGCTAA
TP6281_Hit	D	1	chr3	.	CAGCAAGTAAACTATATTTTATCATCTAAAACAAAAAGAACTGCCAACAAGGTCAGAATGTCTC
TP6281_Query	D	1	chr3	.	CAGCAAGTAAACTATATTATATCATCTAAAACAAAAAGAACTGCCAACAAGGTCAGAATGTCTC
TP62847_Hit	D+G	1	chr3	.	CTGCTACTGTTGAATATGAGACTGAAACTCGTCATTATGCTCATGTTGATTGCCCCGGTCACGC
TP62847_Query	D+G	1	chr3	.	CTGCAACTGTTGAATATGAGACTGAAACTCGTCATTATGCTCATGTTGATTGCCCCGGTCACGC
TP62973_Hit	D+G	1	chr3	.	CTGCAGGAAAGACCAACCGCCGGAATTAATCTTCTTAAAAGGAGCAACCACAACAAGTCCAGGT
TP62973_Query	D+G	1	chr3	.	CTGCAAGAAAGACCAACCGCCGGAATTAATCTTCTTAAAAGGAGCAACCACAACAAGTCCAGGT
TP62982_Hit	D+G	1	chr3	.	CTGCAAGAAAGGGGCACGCGCGTTCGGCACTGTAGTTTTTTGAGACAGCGTCGTTTTTTTTCCG
TP62982_Query	D+G	1	chr3	.	CTGCAAGAAAGGGCCACGCGCGTTCGGCACTGTAGTTTTTTGAGACAGCGTCGTTTTTTTTCCG
TP63009_Hit	D	1	chr3	.	CTGCAAGAACTGATTTTGTCGCATAATCTGCATCCTGAGAAGCAATACAAGTTCTATTGGAGTG
TP63009_Query	D	1	chr3	.	CTGCAAGAACTGATTTTGTCGCATAATCTGCATCCTGAGAAGCAATACAAGTTCTATTCGAGTG
TP6303_Hit	D	1	chr3	.	CAGCAAGTAAGTCAGATTCTGATGACCTTCCAGCTTTCTTTGTGATAATCCATTCATAAGAGCT
TP6303_Query	D	1	chr3	.	CAGCAAGTAAGTCAGATTCTGATGACCTTCCAGCTTTCTTTGTGACAATCCATTCATAAGAGCT
TP63231_Hit	D	1	chr3	.	CTGCAAGCAACTAATGTTGAGACGGCCTTCATGACTGTTTTGACAGAAATATTTAACATTGTTA
TP63231_Query	D	1	chr3	.	CTGCAAGCAACTAATGTTGAGACGGCCTTCATGACTGTTTTGACAGAAATATTTAACATTGTCA
TP63656_Hit	D	1	chr3	.	CTGCAAGTTATTGGATGAATGAGGAAAAGAGAATGAGAGCTTTGGTGAAAGTTTGATGAATTAT
TP63656_Query	D	1	chr3	.	CTGCAAGTTATTGGATGAATCAGGAAAAGAGAATGAGAGCTTTGGTGAAAGTTTGATGAATTAT
TP63867_Hit	D	1	chr3	.	CTGCAATAGCCGTGGATTTTTGCATATCGGAGATAGCGGTTATTGCTTACATAGCGGAAGTTAT
TP63867_Query	D	1	chr3	.	CTGCAATAGCCGTGAATTTTTGCATATCGGAGATAGCGGTTATTGCTTACATAGCGGAAGTTAT

Name	Filter	Nb hit (Mt4.0)	Mt Chr (Mt4.0)	Ms Chr (Li et al., 2014)	Sequence
TP63959_Hit	D	1	chr3	.	CTGCGATATGCACACATTCACCTGCGAAACCAGTTCTTATATTTGTCTCGTCACGGCGCCAAAC
TP63959_Query	D	1	chr3	.	CTGCAATATGCACACATTCACCTGCGAAACCAGTTCTTATATTTGTCTCGTCACGGCGCCAAAC
TP63999_Hit	D	1	chr3	.	CTGCAATGTTGAGCTCGACATAGGGAGATCGAGTGAGAGACCGCACTTTCGACACAAAAAACCG
TP63999_Query	D	1	chr3	.	CTGCAATATTGAGCTCGACATAGGGAGATCGAGTGAGAGACCGCACTTTCGACACAAAAAACCG
TP64254_Hit	D+G	1	chr3	.	CTGCAATGACAATATCGACGGAGGAGTTCGAGTCTCTAAAACGGAAAGTTGAGGAATCCGATAA
TP64254_Query	D+G	1	chr3	.	CTGCAATGACAATATCGACGGAGGAGTTCGAGTCTCTAAAACGGAAAGTCGAGGAATCCGATAA
TP64386_Hit	D	1	chr3	.	CTGCAATGGATGGTGCCAAAGCCGTGAATATGCATGATTTAGAGTTTGCACGAGACAAGATTAT
TP64386_Query	D	1	chr3	.	CTGCAATGGATGGTGCCAAAGCCGTAAATATGCATGATTTAGAGTTTGCACGAGACAAGATTAT
TP64519_Hit	D	1	chr3	.	CTGCAATGTGTAGTGCGGCATAGAGAGATCGAGTGAGAGACCGCACTTTCGACACAAAAGACCG
TP64519_Query	D	1	chr3	.	CTGCAATGTGTAGCGCGGCATAGAGAGATCGAGTGAGAGACCGCACTTTCGACACAAAAGACCG
TP6463_Hit	D	1	chr3	.	CAGCAAGTGCAGGATTGTCCACAACTTTGACAATAGCACCAGGATTCACTTTGTTGAGGACATT
TP6463_Query	D	1	chr3	.	CAGCAAGTGCAGGATTGTCCACAACTTTGACAACAGCACCAGGATTCACTTTGTTGAGGACATT
TP64723_Hit	D	1	chr3	.	CTGCAATTGAGCTAATTGGGGTAAGCATCTCCGCTTTTTGTTGTTGCTGAACTGTGTTTCAGTG
TP64723_Query	D	1	chr3	.	CTGCAATTGAGCTAATTGGGGTAAGCATCTCCGCTTTTTGGTGTTGCTGAACTGTGTTTCAGTG
TP64892_Hit	D+G	1	chr3	.	CTGCAATTTCTAAAAGATTAAAGGTTGAAGGATATAGGAAAAAGAAAGAGATGAAATTGGAAAG
TP64892_Query	D+G	1	chr3	.	CTGCAATTTCTAAAAGATTAAAGGTTGAAGGATATAGGAAAAAGAAAGAGATGAAATTGAAAAG
TP64918_Hit	D+G	1	chr3	.	CTGCAATTTGCGGTTGTGTACTGTAATTTAAAACCATGCTTGATGGTTCAGTTGAATAGGTGTT
TP64918_Query	D+G	1	chr3	.	CTGCAATTTGCGGTTGCGTACTGTAATTTAAAACCATGCTTGATGGTTCAGTTGAATAGGTGTT
TP64990_Hit	D	1	chr3	.	CTGCACAAAACCGTATTGGCCTTTCAACTCAGATCGATCTGATGGATGATTTCCTCGAAATGGA
TP64990_Query	D	1	chr3	.	CTGCACAAAACCGTATAGGCCTTTCAACTCAGATCGATCTGATGGATGATTTCCTCGAAATGGA
TP65067_Hit	D	1	chr3	.	CTGCACAAATTTCTTGATCTGAGGTTGGATTTCTAGACCTCTGGCATAGATACCTGAACCATTT
TP65067_Query	D	1	chr3	.	CTGCACAAATTTCTTGATCTGAGGCTGGATTTCTAGACCTCTGGCATAGATACCTGAACCATTT
TP65218_Hit	D+G	1	chr3	.	CTGCACAATACGTAATTCACACAGTTAAATGAGCAACATTTTGAAGAGGGGAGTATGAACTTGA
TP65218_Query	D+G	1	chr3	.	CTGCACAATACGTAATTCACACAGTTAAATGAGCAACATTTTGAAGAGGGGAGTATGAACTTAA
TP65527_Hit	D+G	1	chr3	.	CTGCACATAACGTTAGAACAGCAAGAAAAGCTTATAATCTTCAACCAAACGGTGCCGGACAGCT
TP65527_Query	D+G	1	chr3	.	CTGCACATAACGTTAGAACAGCAAGAAAAGCTTATAATCTTCAACCAAACGGTGCCGGAAAGCT
TP65849_Hit	D	1	chr3	.	CTGCACCACGACGTAGAGAATCCCTTCTCTGATACACCACTCAACAAACCATGAAAACTCACCG
TP65849_Query	D	1	chr3	.	CTGCACCACGACGTAGAGAATCCCTTCTCCGATACACCACTCAACAAACCATGAAAACTCACCG
TP66122_Hit	D	1	chr3	.	CTGCACCGGAAAGTGTAGTTGTACCTGAGGTAACGGTTGATACCACAAATGGAGATGTTCCCGC
TP66122_Query	D	1	chr3	.	CTGCACCGGAAAGTGCAGTTGTACCTGAGGTAACGGTTGATACCACAAATGGAGATGTTCCCGC
TP66245_Hit	D	1	chr3	.	CTGCACCTGAAGAGGAGAAGAAAGAAGAGGTAAGTTTGTGTTTTTGTTTCTTTCTACTTTTTAT
TP66245_Query	D	1	chr3	.	CTGCACCTGAAGAGGAGAAGAAAGAAGAGGTAAATTTGTGTTTTTGTTTCTTTCTACTTTTTAT
TP6658_Hit	D	1	chr3	.	CAGCAAGTTTGGTTGGTGTGGATCAACTGGCGAATACTGCGGTGATGGTTGTCAGAGTCAATGT
TP6658_Query	D	1	chr3	.	CAGCAAGTTTGGTTGGTGTGGATCAACCGGCGAATACTGCGGTGATGGTTGTCAGAGTCAATGT
TP66644_Hit	D	1	chr3	.	CTGCACGTGTTAGTAAATACAATTGGTTCAAATTTGATTTATTTACTAAACATGCTCTTATGTT
TP66644_Query	D	1	chr3	.	CTGCACGTGTTAGTAAATACAATTGGTTAAAATTTGATTTATTTACTAAACATGCTCTTATGTT
TP66784_Hit	D	1	chr3	.	CTGCACTAGTTGCTTTACCGGAAGTCTGCACATAATTTCCACAATCAGTTCGAATGGAAGAGTG
TP66784_Query	D	1	chr3	.	CTGCACTAGTTGCTTTACCGGAAGTCTGCACATAATTTCCACAATCAGATCGAATGGAAGAGTG
TP66851_Hit	D	1	chr3	.	CTGCACTCAATGAAGAGCTCTTCTTTAAAATTGCTAACCATCAACTAACTGTTGTTGAGGTTGA
TP66851_Query	D	1	chr3	.	CTGCACTCAATGAAGAGCTCTTCTTTAAAATTGCAAACCATCAACTAACTGTTGTTGAGGTTGA
TP66868_Hit	D	1	chr3	.	CTGCACTCATTAGTGATGTGTGTGCTTGGATTCTCTTGGCTTTAGCCATTGCAATGGCTGAGAA
TP66868_Query	D	1	chr3	.	CTGCACTCATTAGTGATGTGTGTGCTTGGATTCTCTTGGCTTTAGCCATTGCAATGGCTGAAAA
TP67417_Hit	D	1	chr3	.	CTGCACTTTTTGTTACTTTTTATTTTGGTATAATTCTCAAAATATAAATTGCAGATTGAAACAT
TP67417_Query	D	1	chr3	.	CTGCACTTTTTGTTACTTTTTATTTTGGTATAATTCTCAAAAAATAAATTGCAGATTGAAACAT
TP67460_Hit	D	1	chr3	.	CTGCAGAAAAGAGATCACAGTTGGGAACTCTTCTTCCATCTGGACCAAATCGTATTGGAGGTGA
TP67460_Query	D	1	chr3	.	CTGCAGAAAAGAGATCACAGTTGGGAACTCTTCTTCCATCTGGACCAAATCGTATTGGAGGCGA
TP6784_Hit	D	1	chr3	.	CAGCAATAACCTTCATTTATTAAGTGTGTGTTTGGAATGGCTTAATTTTTTCAAAACCCTGGTA
TP6784_Query	D	1	chr3	.	CAGCAATAACCTTCATTTATTAAGTGTGTGTTTGGAATGGCTTAATTTTTTCAAAACCCTGATA
TP67924_Hit	D	1	chr3	.	CTGCTGAGCAATTTGCATACATTGGTTTGAGTTCAAATTTGGTGTTATACCTCACAAAGGCACT
TP67924_Query	D	1	chr3	.	CTGCAGAGCAATTTGCATACATTGGTTTGAGTTCAAATTTGGTGTTATACCTCACAAAGGCACT
TP67956_Hit	D	1	chr3	.	CTGCAGAGGAAGAGGCTTTCTTGGAATGACTTCCAGTGGTAACATCCTCAAAGTAGAATCTTTT
TP67956_Query	D	1	chr3	.	CTGCAGAGGAAGAGGCTTTCTTGGAATGACTTCCAGTGCTAACATCCTCAAAGTAGAATCTTTT
TP68133_Hit	D+G	1	chr3	.	CTGCAGATGAAGAGATTAGAGGGGAGGTTGATTTTCGACATGCCTATATTGATTTCTCCAAACT
TP68133_Query	D+G	1	chr3	.	CTGCAGATGAAGAGATTAAAGGGGAGGTTGATTTTCGACATGCCTATATTGATTTCTCCAAACT

Name	Filter	Nb hit (Mt4.0)	Mt Chr (Mt4.0)	Ms Chr (Li et al., 2014)	Sequence
TP6921_Hit	D	1	chr3	.	CAGCAATACGGAGATATCCTTACCATGTATACTGCTTGCAAGCATCGCGGCTTTGTCATGATTT
TP6921_Query	D	1	chr3	.	CAGCAATACGGAGATATCCGTACCATGTATACTGCTTGCAAGCATCGCGGCTTTGTCATGATTT
TP69215_Hit	D+G	1	chr3	.	CTGCAGGGAAAGAGGGGAACTTGCGTGCTCTACTCTCCACCTTACAATATGTATGTACTAAGTA
TP69215_Query	D+G	1	chr3	.	CTGCAGGGAAAGAGGGGAACTTGCGTGCTCTACTCTCCACCTTACAATATGTATGTACCAAGTA
TP6933_Hit	D	1	chr3	.	CAGCAATACTCATTCAGTCCTACTATAGAGGATACCTGGTAAGTCTTCTTTTATCTGTTTTGGT
TP6933_Query	D	1	chr3	.	CAGCAATACTCATTCAGTCCTACTATAGAGGATACCTAGTAAGTCTTCTTTTATCTGTTTTGGT
TP69711_Hit	D	1	chr3	.	CTGCAGTGCTCTTCATTGGGGTACAAAATGCTACATCGGTGCAACCAGTTGTAGCCATTGAGAG
TP69711_Query	D	1	chr3	.	CTGCAGTCCTCTTCATTGGGGTACAAAATGCTACATCGGTGCAACCAGTTGTAGCCATTGAGAG
TP70046_Hit	D	1	chr3	.	CTGCAGTTCTTATTTCATCTACAAGCGACGAAGGAAGAGCCTTTTGTTCCCCACTAGCTGAAAA
TP70046_Query	D	1	chr3	.	CTGCAGTTCTTATTTCATCTACAAGCGACGAAGGAAGAGCCTTTTGTTCCCCACTAGCAGAAAA
TP70085_Hit	D	1	chr3	.	CTGCAGTTGCCATTCGACTCCGAAGTATCTATAGTATCTCAGACGACTATGTTCTTGAGTTTTT
TP70085_Query	D	1	chr3	.	CTGCAGTTGCCATTCGACTCCGAAGCATCTATAGTATCTCAGACGACTATGTTCTTGAGTTTTT
TP7015_Hit	D+G	1	chr3	.	CAGCAATAGCGGTGCTATAGCACTGTAGTGTAGTAGAATTTGAACAAACCGCTATTCTTCGCAA
TP7015_Query	D+G	1	chr3	.	CAGCAATAGCGGTGCTATAGCACTGTAGTGTAGTAGAATTCGAACAAACCGCTATTCTTCGCAA
TP70189_Hit	D	1	chr3	.	CTGCAGTTTCTCAGAGATGGAAAATGGGTCGGTGTTCAACCAATAGAAGGAGCCATTGTTGCTA
TP70189_Query	D	1	chr3	.	CTGCAGTTTCTCAAAGATGGAAAATGGGTCGGTGTTCAACCAATAGAAGGAGCCATTGTTGCTA
TP70200_Hit	D+G	1	chr3	.	CTGCAGTTTCTGGTGATTTACAATTGGCATTTGATCTTTGTCTTGTTTTGGCTAGAAAAGGACA
TP70200_Query	D+G	1	chr3	.	CTGCAGTTTCTGGTGATTTACAATTGGCATTTGATCTTTGTCTTGTTTTGGCCAGAAAAGGACA
TP70337_Hit	D+G	1	chr3	.	CTGCATAACTAGTCCACCTCCTCCAACCATCTCTTGAGGAAAATTGTTAGGGTAATTCAAAAAA
TP70337_Query	D+G	1	chr3	.	CTGCATAACTAATCCACCTCCTCCAACCATCTCTTGAGGAAAATTGTTAGGGTAATTCAAAAAA
TP70356_Hit	D	1	chr3	.	CTGCATAAGAGAACATTCCAAAGTAATCCTATTTTTGTACTAAGAATAGAGCAGAATCATTAGG
TP70356_Query	D	1	chr3	.	CTGCATAAGAGAACATTCCAAAGTAATCCCATTTTTGTACTAAGAATAGAGCAGAATCATTAGG
TP7049_Hit	D	1	chr3	.	CAGCAATAGTGAAGTGCCATCCGGTGATGAGACCAGCCGCACCGGAGATCAATGGGACAAGGCT
TP7049_Query	D	1	chr3	.	CAGCAATAGTGAAGTGCCATCCGGTGATGAGACCAGCCGCACCGGAGATCAATGGGACAAGGCA
TP7050_Hit	D	1	chr3	.	CAGCAATAGTGAAGTGCCATCGGGCGATGAGACCAGCCGCACCGGAGATCAATGGGACAAGGCT
TP7050_Query	D	1	chr3	.	CAGCAATAGTGAAGTGCCATCGGGCGATGAGACCAGCCGCACCGGAGATCAATGGGACAAGGCA
TP70572_Hit	D	1	chr3	.	CTGCATAGCAATCATGACAATAGCTACCCTCCATGTAACGATTAAGCCAAGTACAAAAGCCAGC
TP70572_Query	D	1	chr3	.	CTGCATAGCAATCATGACAATAGCTACCCTCCATGTAACGATTAAACCAAGTACAAAAGCCAGC
TP70612_Hit	D	1	chr3	.	CTGCATAGTAAGCAACTTGTGAAAGAATAACTATCCCTTAAGCCACGGATTCAATCTCAATTTT
TP70612_Query	D	1	chr3	.	CTGCATAGTAAGCAACTTGTGAAAGAATAACTATCCCTTAAGCCACAGATTCAATCTCAATTTT
TP70778_Hit	D+G	1	chr3	.	CTGCATATGCGGTATGAGGATGAAATATATGTTGTTTCCTATGATCAGATTGCGCACATCGAAC
TP70778_Query	D+G	1	chr3	.	CTGCATATGCGGTATGAGGATGAAATATATGTTGTTTCCTATGATCAGATTGCGCACATCAAAC
TP70862_Hit	D	1	chr3	.	CTGCATATTTTTGGTTTTTTAGCCTTAAGCCATAAGGCGCAAGGTATCTGTATCAAGTATTTCA
TP70862_Query	D	1	chr3	.	CTGCATATTTTTGGTTTTTTAGCCTTAAGCCATAAGGCACAAGGTATCTGTATCAAGTATTTCA
TP70938_Hit	D	1	chr3	.	CTGCATCAAGGGAAGCCTTCCTATCCCCCTTGCTTGCAGATGACATATTGAACTAGAGCTTCCA
TP70938_Query	D	1	chr3	.	CTGCATCAAGGGAAGCCTTCCTATCACCCTTGCTTGCAGATGACATATTGAACTAGAGCTTCCA
TP71014_Hit	D	1	chr3	.	CTGCATCACTGTGTAGCTAGGTTCTATTCTGCCAATAGTAGAGTTACAATTTGCAAAATGGGCT
TP71014_Query	D	1	chr3	.	CTGCATCACTGTGTAGCTAGGTTCCATTCTGCCAATAGTAGAGTTACAATTTGCAAAATGGGCT
TP71052_Hit	D	1	chr3	.	CTGCATCAGGAAGATATTACCATGCAAAAAATATAAAACCAAAACGCACAACTTGTTCATGTGA
TP71052_Query	D	1	chr3	.	CTGCATCAGGAAGATATTACCATGCAAAAAAAATAAAACCAAAACGCACAACTTGTTCATGTGA
TP71063_Hit	D+G	1	chr3	.	CTGCATCAGTCAACAAAACTCTGTTGTTGGATGAAGATCAACTATTTATATTTTTATTTCACCA
TP71063_Query	D+G	1	chr3	.	CTGCATCAGTCAACAAAACTCTGTTGTTGGATGAAGATCAACGATTTATATTTTTATTTCACCA
TP71136_Hit	D+G	1	chr3	.	CTGCATCATTGGCAATGAATGTGGGAGTTGTTGCATAGGACCTGCTCCCCGCCGTCCGCCTGGA
TP71136_Query	D+G	1	chr3	.	CTGCATCATTGGCAATGAATGCGGGAGTTGTTGCATAGGACCTGCTCCCCGCCGTCCGCCTGGA
TP71141_Hit	D	1	chr3	.	CTGCGTCATTTCCTTTGTTATTTTCTCTAGCAAGTAAAAGTGCTGTTTCATTCCTCTCATTTTT
TP71141_Query	D	1	chr3	.	CTGCATCATTTCCTTTGTTATTTTCTCTAGCAAGTAAAAGTGCTGTTTCATTCCTCTCATTTTT
TP71313_Hit	D+G	1	chr3	.	CTGCATCGACTTCGGGTACATCTGTACGCTGTGCAGTTTTTAAAGAAGATGCTCGTGCTGAAAA
TP71313_Query	D+G	1	chr3	.	CTGCATCGACTTCGGGTACATCTGTACGCTGTGCAGTTTTTAAAGAAGATGCTCATGCTGAAAA
TP7148_Hit	D	1	chr3	.	CAGCAATATCCCATCTTCATCACAAGTAAACAAGTTTCAACTTTTAAGTGTCATAATGTAAATA
TP7148_Query	D	1	chr3	.	CAGCAATATCCCATCTTCATCACAAGTAAACAAGTTTCAACTTTTAAGTGTCATAATGCAAATA
TP71709_Hit	D+G	1	chr3	.	CTGCATGAGGACCAAGCCATGGTGTGTGCCGCCACACAAGCTTCCCATTGCAACCTGCATGAAC
TP71709_Query	D+G	1	chr3	.	CTGCATGAGGACCAAGCCAAGGTGTGTGCCGCCACACAAGCTTCCCATTGCAACCTGCATGAAC
TP71948_Hit	D	1	chr3	.	CTGCATGGCCAAACTAATCACCAAAGGTATAAAAATCAAAAACTGGCTTCTTCAAAATCAAAAC
TP71948_Query	D	1	chr3	.	CTGCATGGCCAAACTAATCACCAAAGGTATAAAAAACAAAAACTGGCTTCTTCAAAATCAAAAC

Name	Filter	Nb hit (Mt4.0)	Mt Chr (Mt4.0)	Ms Chr (Li et al., 2014)	Sequence
TP7222_Hit	D	1	chr3	.	CAGCAATATTCACCGGTAATGAATTACAAAAAGATTCAGGAAAAATTCTTATGTTACTATAGAG
TP7222_Query	D	1	chr3	.	CAGCAATATTCACCGGTAATGAATTACAAAAAGATTCAGAAAAAATTCTTATGTTACTATAGAG
TP72222_Hit	D	1	chr3	.	CTGCATTAAAAGAAAAATCTATTCAGAAATCAGTGTCTTCCTTACATAGACTTAATACTCCATA
TP72222_Query	D	1	chr3	.	CTGCATTAAAAGAAAAATCTATTCAGAAATCAGTGTCTTCCTTACATAGACTTAAGACTCCATA
TP7229_Hit	D	1	chr3	.	CAGCAATATTCTGGCAACAGGCATCAAGTATCACATCCTCAAACCCACCAAGCTCGGCTGAAAA
TP7229_Query	D	1	chr3	.	CAGCAATATTCTGGCAACAGGCATCAAGTATCACATCCTCAAACCCACCAAGCTCGGCAGAAAA
TP72609_Hit	D	1	chr3	.	CTGCATTGAGAGGCAAGAAAAGGTCCAATCCAAAAGTTCTTTGTTCTTGAATTAGTTTCATGTT
TP72609_Query	D	1	chr3	.	CTGCATTGAGAGACAAGAAAAGGTCCAATCCAAAAGTTCTTTGTTCTTGAATTAGTTTCATGTT
TP72661_Hit	D	1	chr3	.	CTGCATTGCACCCATAGCAACTGGCAAAGCATCGTCTGTATCCCGTAATGATGAAAGAAAGCGT
TP72661_Query	D	1	chr3	.	CTGCATTGCACCCATAGCAACCGGCAAAGCATCGTCTGTATCCCGTAATGATGAAAGAAAGCGT
TP72742_Hit	D	1	chr3	.	CTGCATTGGTCAGGAAAGTATTTGGTTGAAATCATAAATAGGCAAGATTGTACCTCCTAGACGC
TP72742_Query	D	1	chr3	.	CTGCATTGGTCAGGAAAGTATTTGGATGAAATCATAAATAGGCAAGATTGTACCTCCTAGACGC
TP7283_Hit	D	1	chr3	.	CAGCAATCAACTCCTGAAAAGCAAGAAATTGCAATTGTAAATTATAACTATCATTAGCATGTAA
TP7283_Query	D	1	chr3	.	CAGCAATCAACTCCTGAAAAGCAAGAAATTGCAATTGTAAATTATAACTATCATTAGCAAGTAA
TP72868_Hit	D+G	1	chr3	.	CTGCATTTAGAGTGGTGATTGTTGGAGGGAAGCTGTTTGTGGATTGGTATTATGCTTGTGTTCA
TP72868_Query	D+G	1	chr3	.	CTGCATTTAGAGTGGTGATTGTTGGAGGGAAGTTGTTTGTGGATTGGTATTATGCTTGTGTTCA
TP72972_Hit	D+G	1	chr3	.	CTGCATTTCTTAACAAACTTGATGCAAAACAAAACCCAACTAGCTAATTCTCACTACAAATACA
TP72972_Query	D+G	1	chr3	.	CTGCATTTCTTAACAAACTTAATGCAAAACAAAACCCAACTAGCTAATTCTCACTACAAATACA
TP73058_Hit	D	1	chr3	.	CTGCATTTGGTTATTCATTTGGTTATTAGCTCTACCACAATCCCAATTACTTGGTGGAAAAGGA
TP73058_Query	D	1	chr3	.	CTGCATTTGGTTATTCATTTGGTTATTAGCTCTACCACAATCCCAATTACTCGGTGGAAAAGGA
TP73082_Hit	D	1	chr3	.	CTGCATTTGTGGCTGAAGTCATATGAATAAGAGAATATTGAGGAAAAAAAGAGATAATGATGTG
TP73082_Query	D	1	chr3	.	CTGCATTTGTGGCAGAAGTCATATGAATAAGAGAATATTGAGGAAAAAAAGAGATAATGATGTG
TP7310_Hit	D	1	chr3	.	CAGCAATCACAAATGCCGCTTCATCAGGAGATTCAGCTTCATATGAAACTCTTCCTGTTTCCTC
TP7310_Query	D	1	chr3	.	CAGCAATCACAAATGCCGCTTCATCAGGAGATTCAGCTTCATATGAAACTCTTCCTGTATCCTC
TP73304_Hit	D	1	chr3	.	CTGCCAAAAACTCTAAGACACATGCACTTTTTAATTTCTGAAAAATATCATTATCATTATCAAC
TP73304_Query	D	1	chr3	.	CTGCCAAAAACTCTAAGACACATGCACTTTTTTATTTCTGAAAAATATCATTATCATTATCAAC
TP73339_Hit	D	1	chr3	.	CTGCCAAAAGCAGAAAAGGACCGATTCTTGGATCATCGAGGTACACAGAAAGTCCGCCAACAAC
TP73339_Query	D	1	chr3	.	CTGCCAAAAGCAGAAAAGGACCGATTCTTGGATCATCGAGGTACACAGAAAGTCCACCAACAAC
TP73449_Hit	D	1	chr3	.	CTGCCAAAGGGAATGGCGATGCCATACGCAAAAAGGAAGAGACTCATAGAATGGCTGAAAAAAA
TP73449_Query	D	1	chr3	.	CTGCCAAAGGGAATGGCGATGCCATACGCAAAAAGGAAGAGACTCATAGAATGGCAGAAAAAAA
TP7348_Hit	D	1	chr3	.	CAGCAATCAGGATGAGCTGAATGAACCTACTGAGAGTCAGCATGACAATGGACGGGCACGAACA
TP7348_Query	D	1	chr3	.	CAGCAATCAGGATGAGCTGAATGAACCTACTGAGAGCCAGCATGACAATGGACGGGCACGAACA
TP73721_Hit	D	1	chr3	.	CTGCCAAGCATGAGTACTATGGACAGAGTTCAGAGGTTCTCATCTGGTTGTTGCCGTCCACAAG
TP73721_Query	D	1	chr3	.	CTGCCAAGCATGAGTACCATGGACAGAGTTCAGAGGTTCTCATCTGGTTGTTGCCGTCCACAAG
TP73939_Hit	D	1	chr3	.	CTGCCAATTAACCAAATGTAACAGCTAAATAAGGAAATTTCACAAATTGAGCATCTGGTACCTT
TP73939_Query	D	1	chr3	.	CTGCCAATTAACCAAATGTAACAGCAAAATAAGGAAATTTCACAAATTGAGCATCTGGTACCTT
TP73994_Hit	D	1	chr3	.	CTGCCAATTTTCCCAGCTCAATATTCCCACTGTCCACACAGGCACTGAGTAGCGCTCCCCACAT
TP73994_Query	D	1	chr3	.	CTGCCAATTTTCCCAGATCAATATTCCCACTGTCCACACAGGCACTGAGTAGCGCTCCCCACAT
TP74713_Hit	D	1	chr3	.	CTGCCAGATATGCTTTGATTGCCGCCAGATATGCTTTGTTAGTTGTGTCTATGGCCTCTGTTTT
TP74713_Query	D	1	chr3	.	CTGCCAGATATGCTTCGATTGCCGCCAGATATGCTTTGTTAGTTGTGTCTATGGCCTCTGTTTT
TP7491_Hit	D	1	chr3	.	CAGCAATCTCAGGTAGAATGGCAATTTTATTTACCATACAATTTTATATCTAGAAGAAAATTAT
TP7491_Query	D	1	chr3	.	CAGCAATCTCAGGTAGAATGGCAATTTTATTTACCATAAAATTTTATATCTAGAAGAAAATTAT
TP75125_Hit	D	1	chr3	.	CTGCCATATCCAAGTACCAAGCTGACAATGCAATTCCGGGGAAGAATGATGGAGGCAATCCGAT
TP75125_Query	D	1	chr3	.	CTGCCATATCCAAGTACCAAGCAGACAATGCAATTCCGGGGAAGAATGATGGAGGCAATCCGAT
TP75145_Hit	D+G	1	chr3	.	CTGCCATATTAGTGAAGACAGTCAGTCATCCTTAAGTAAACAATACCGTCGTCTATCAATAGAA
TP75145_Query	D+G	1	chr3	.	CTGCCATATTAGTGAAGACAGTCAGTCATCCTTAAGTAAACAATACCGCCGTCTATCAATAGAA
TP75204_Hit	D	1	chr3	.	CTGCCATCAGCAGTCTCATACTTATACCTTCATATGAGAGATTTATAATCCCAATTCTCCGAGA
TP75204_Query	D	1	chr3	.	CTGCCATCAGCAGTCTCATACTTATACCTTCATATGAGAGATTTATAATCCCAATTCTCCGAAA
TP75509_Hit	D+G	1	chr3	.	CTGCCATGTGCAATGACTGCGCTATGATCTGGCTCGGAACATAAGTCGCCATCACACTTTGCTG
TP75509_Query	D+G	1	chr3	.	CTGCCATGTGCAATGACTGAGCTATGATCTGGCTCGGAACATAAGTCGCCATCACACTTTGCTG
TP75599_Hit	D+G	1	chr3	.	CTGCCATTCTATGAGTCTCTTCCTTTTTGCGTATGGCATCGCCATTCCCTTTGGCTGAAAAAAA
TP75599_Query	D+G	1	chr3	.	CTGCCATTCTATGAGTCTCTTCCTTTTTGCGTATGGCATCGCCATTCCCTTTGGCAGAAAAAAA
TP75643_Hit	D	1	chr3	.	CTGCCATTGCTGTTGGCACACAATCTCGAGGTGGATTAGGGAAGCTTTCAATTCATGGAAGCAA
TP75643_Query	D	1	chr3	.	CTGCCATTGCTGTTGGCACACAATCTCGAGGAGGATTAGGGAAGCTTTCAATTCATGGAAGCAA

Name	Filter	Nb hit (Mt4.0)	Mt Chr (Mt4.0)	Ms Chr (Li et al., 2014)	Sequence
TP75717_Hit	D+G	1	chr3	.	CTGCCATTTCTTTCTGTAACATGCATGTTTGCATGGAAAGCTCAATTACCAATGAAGGTAGACA
TP75717_Query	D+G	1	chr3	.	CTGCCATTTCTTTCTGTAACATGCATGTTTGCATCGAAAGCTCAATTACCAATGAAGGTAGACA
TP75845_Hit	D	1	chr3	.	CTGCCCAAGAAGGATTGCCGTTTGATTGGCCTAGATTCTGTGAGGCGATAGGACTGAAACAAGA
TP75845_Query	D	1	chr3	.	CTGCCCAAGAAGGATTACCGTTTGATTGGCCTAGATTCTGTGAGGCGATAGGACTGAAACAAGA
TP7596_Hit	D	1	chr3	.	CAGCAATGAACTGCATGATCAATAAAAGTCAACAGCAAACAACTTTACTCACAGAATATTCTAT
TP7596_Query	D	1	chr3	.	CAGCAATGAACTGCATGATCAATAAAAATCAACAGCAAACAACTTTACTCACAGAATATTCTAT
TP76008_Hit	D+G	1	chr3	.	CTGCCCAGTCTTTATACACACTACAAAGGCCATATCTCTAGGCAATGCGGGTCTAAACCCACGC
TP76008_Query	D+G	1	chr3	.	CTGCCCAGTCTTTATACACACTACAAAGGCCATATCTCTAGGCAATGCGGGACTAAACCCACGC
TP76204_Hit	D+G	1	chr3	.	CTGCCCCCAACTGCATAAGCCTTTGACATTACAACTTCTGTCATGTCAGCACCGTCAAACTTGG
TP76204_Query	D+G	1	chr3	.	CTGCCCCCAACTGCATAAGCCTTTGACATTACAACTTCTGTCATGTCAGCACCGTCAAACTTAG
TP76300_Hit	D	1	chr3	.	CTGCCCCTAGGTGCTACATGAAACCCTGTTGTTGCATTTTCACACAGTCGTTGTACTGGCCAAA
TP76300_Query	D	1	chr3	.	CTGCCCCTAGGTGCTACATGAAACCCTGTTGTTGCATTTTCACACAGTCGTTGTAATGGCCAAA
TP76347_Hit	D+G	1	chr3	.	CTGCCCCTTATTTAGTTGGTGGATCCTTTGGTTTGATGAAGAAGAAGAGATCAAAGTGGGAAAA
TP76347_Query	D+G	1	chr3	.	CTGCCCCTTATGTAGTTGGTGGATCCTTTGGTTTGATGAAGAAGAAGAGATCAAAGTGGGAAAA
TP7642_Hit	D	1	chr3	.	CAGCAATGACATGATAGCTGGGTCCCAATGTGGGTCATCTGATGGCTGGTCTATGCGCACCTTT
TP7642_Query	D	1	chr3	.	CAGCAATGACATGATAGCTGGATCCCAATGTGGGTCATCTGATGGCTGGTCTATGCGCACCTTT
TP76877_Hit	D+G	1	chr3	.	CTGCCCTTTGGGGAGCTCATGTTAGGCTCTGCTCGCCGAAATGATAACAGTAATAGTGTTACTG
TP76877_Query	D+G	1	chr3	.	CTGCCCTTCGGGGAGCTCATGTTAGGCTCTGCTCGCCGAAATGATAACAGTAATAGTGTTACTG
TP7709_Hit	D	1	chr3	.	CAGCAATGATGCTTAGTACAACTTGGAGCGAAAGTGGCGATAACACGATGTTCTTGTTCCGGTG
TP7709_Query	D	1	chr3	.	CAGCAATGATGCTTAGTACAACTTGGAGCGAAAGTGGCGATAACACCATGTTCTTGTTCCGGTG
TP7722_Hit	D	1	chr3	.	CAGCAATGATTCTTCTTCCACACAAACCCAAAAGTAATAGAAGAAGAACAAACCCTAAATCGTG
TP7722_Query	D	1	chr3	.	CAGCAATGATTCTCCTTCCACACAAACCCAAAAGTAATAGAAGAAGAACAAACCCTAAATCGTG
TP77292_Hit	D+G	1	chr3	.	CTGCGGCATTTGTCTTTAGAGCAGTTCTCGATGCTTTTGACTCTCAGGATGGTTTACAGAAATT
TP77292_Query	D+G	1	chr3	.	CTGCCGCATTTGTCTTTAGAGCAGTTCTCGATGCTTTTGACTCTCAGGATGGTTTACAGAAATT
TP77721_Hit	D	1	chr3	.	CTGCCGGGCTTGTACTTGGTGTCATAGGACTTGTGTTACTCGAGGTAGATTAATTTTGGTCTCA
TP77721_Query	D	1	chr3	.	CTGCCGGGCTTGTACTTGGTGTCATAGGACTTGTGTTACTCGAGGTAGAATAATTTTGGTCTCA
TP77849_Hit	D	1	chr3	.	CTGCCGTAGACAACAATATCTTTCTTTTCCTCATATGGACGAAGTAAGATATTGACAAAGTATG
TP77849_Query	D	1	chr3	.	CTGCCGTAGACAACAATATCTTTCTTTTCCTCATATGGACGAAGTAAGATATTCACAAAGTATG
TP7797_Hit	D+G	1	chr3	.	CAGCAATGCGCGTATTACCGGGCTTCCTCCTAACGAAACAACAATTGCAACAATTGCAGAAAAA
TP7797_Query	D+G	1	chr3	.	CAGCAATGCGCGTATTACCGGGCCTCCTCCTAACGAAACAACAATTGCAACAATTGCAGAAAAA
TP78130_Hit	D+G	1	chr3	.	CTGCCTACAACAATCTCACGTTTCCGCAATGGTGGTGATGACACTACAGCAAATTTATTAGAAA
TP78130_Query	D+G	1	chr3	.	CTGCCTACAACAATCTCACGTTTCCGCAATGGTGGTGATGACACTACAGCAAATTTACTAGAAA
TP78244_Hit	D	1	chr3	.	CTGCCTATACGTGGGTCTTCCTTTTCATTTCAGTTCACTCTCCTATTGCTTCCATTCATCTAAC
TP78244_Query	D	1	chr3	.	CTGCCTATACGTGGGTCTTCCTTTTCATTTCAGTTCAATCTCCTATTGCTTCCATTCATCTAAC
TP78416_Hit	D	1	chr3	.	CTGCCTCATACAATCCTTTAGTTCCCCATTAAGATGACTAACTTGATCTTCAAGAACCGCATTT
TP78416_Query	D	1	chr3	.	CTGCCTCATACAATCCTTTAGTTCCCCATTAAGATGACTAACTTGATCTTCAAGAACCGCATTC
TP7858_Hit	D	1	chr3	.	CAGCGATGGATGGTGCTTCTGATTGACCTTGAGATCCATGTGAAACATCCATTCCAATAACCAA
TP7858_Query	D	1	chr3	.	CAGCAATGGATGGTGCTTCTGATTGACCTTGAGATCCATGTGAAACATCCATTCCAATAACCAA
TP78657_Hit	D+G	1	chr3	.	CTGCCTCTCAGTAAGTTTGATCTTAATGAATCTGGAAAGTTGGTTACTATGGATATCCCTTTGC
TP78657_Query	D+G	1	chr3	.	CTGCCTCTCAGTAAGTTCGATCTTAATGAATCTGGAAAGTTGGTTACTATGGATATCCCTTTGC
TP79158_Hit	D+G	1	chr3	.	CTGCCTTATCGTGCCAATGCTTGTTGGGAGGTTGTCATGTGGAGTAGCTTATATGCGCCTTCAT
TP79158_Query	D+G	1	chr3	.	CTGCCTTATCATGCCAATGCTTGTTGGGAGGTTGTCATGTGGAGTAGCTTATATGCGCCTTCAT
TP79304_Hit	D	1	chr3	.	CTGCCTTCTTGGAAAATAGTGGCCCTAGGGATGTAACAATCCAATTTCAAAATATTTCGTATGA
TP79304_Query	D	1	chr3	.	CTGCCTTCTTGGAAAATAGTGGACCTAGGGATGTAACAATCCAATTTCAAAATATTTCGTATGA
TP7971_Hit	D	1	chr3	.	CAGCAATGTGGAAAGAACACCAGCTGGATTACTCTATGTAAGACAGTGGAACAACATGCAATAT
TP7971_Query	D	1	chr3	.	CAGCAATGTAGAAAGAACACCAGCTGGATTACTCTATGTAAGACAGTGGAACAACATGCAATAT
TP80579_Hit	D	1	chr3	.	CTGCGATGAGAACAAACTCTAGACCGAACCGGTGAACTAAGAAGGGCGGTTCGTCGGCCGAATT
TP80579_Query	D	1	chr3	.	CTGCGATGAGAACAAACTCTAGACCGAACCGGTCAACTAAGAAGGGCGGTTCGTCGGCCGAATT
TP8058_Hit	D	1	chr3	.	CAGCAATGTTAAAATATAAAGTACCGTAAGTGCAGAGGAATTGGCGGTCAATGATTCGATATTA
TP8058_Query	D	1	chr3	.	CAGCAATGTTAAAATATAAAGTACCGTAACTGCAGAGGAATTGGCGGTCAATGATTCGATATTA
TP80712_Hit	D	1	chr3	.	CTGCGATTGGCTAAGCACATGTCTCCTAAGGTGGTGACTCTAGTTGAGCAAGAATTCAACACCA
TP80712_Query	D	1	chr3	.	CTGCGATTGGCTAAGCACATGTCTCCTAAGGTGGTGACTCTAGTGGAGCAAGAATTCAACACCA
TP80749_Hit	D+G	1	chr3	.	CTGCGATTTCTGCGTTGGTTAAGTTACTGAGTGAAAATCCTGCAAGAGCACTTGCTGTTGCTGA
TP80749_Query	D+G	1	chr3	.	CTGCGATTTCTGCGTTAGTTAAGTTACTGAGTGAAAATCCTGCAAGAGCACTTGCTGTTGCTGA

Name	Filter	Nb hit (Mt4.0)	Mt Chr (Mt4.0)	Ms Chr (Li et al., 2014)	Sequence
TP8085_Hit	D+G	1	chr3	.	CAGCAATGTTGGCTGATATCTTTCTGATATTTGTGAATGGAGGGTATATCAGTCCCTTATCATA
TP8085_Query	D+G	1	chr3	.	CAGCAATGTTGGCTGATATCTTTCTGATATTGGTGAATGGAGGGTATATCAGTCCCTTATCATA
TP8118_Hit	D	1	chr3	.	CAGCAATTAAAGCATTGCAAGAGAGTGAAGCAAATAGAAGCAAAAATGAAGTGGACCCGTCCAG
TP8118_Query	D	1	chr3	.	CAGCAATTAAAGCATTGCAAGAGAGTGAAGCAAATAGAAGCAAAAATGAAGTGGACCCATCCAG
TP81184_Hit	D+G	1	chr3	.	CTGCGCCCGAAAGACGAGTTAGGTTTGCTGTTGATGATGATCATGAAAGTGAGACAAGGTCTGA
TP81184_Query	D+G	1	chr3	.	CTGCGCCCGAAAGACGAGTGAGGTTTGCTGTTGATGATGATCATGAAAGTGAGACAAGGTCTGA
TP8125_Hit	D	1	chr3	.	CAGCAATTAAATCTTTAGTATATATAATTGGAATAACTTAGTAATCGTTCATATGCAGGAATTA
TP8125_Query	D	1	chr3	.	CAGCAATTAAATCTTTAGTATATATAATTGGAATAACATAGTAATCGTTCATATGCAGGAATTA
TP81356_Hit	D+G	1	chr3	.	CTGCGCGACGAGGAACAGAGAATCCTCCATGTGTGCTGGTGTCACTGGCTGTCAAAGTCTTGCA
TP81356_Query	D+G	1	chr3	.	CTGCGCGACGAGGAACAGAGAATCCTCCATGTGTACTGGTGTCACTGGCTGTCAAAGTCTTGCA
TP82080_Hit	D	1	chr3	.	CTGCGGCACAGCTAATCAAGGTATTTTTAAGCATGATTATCTACAAATTACTGTCAGTTCGATG
TP82080_Query	D	1	chr3	.	CTGCGGCACAGCTAATCAAGGTATTTTTAAGCATGATTATCTACAAATTACTGTCAGTTCAATG
TP8237_Hit	D	1	chr3	.	CAGCAATTATGGAAGTAATGGTTGCTTGTTCTTGTGGACTAAGCTTTCCATGCGGTGTACCAGG
TP8237_Query	D	1	chr3	.	CAGCAATTATGGAAGTAATGGTTGCTTGTTCTTGTGGACTAAGCTTTCCATGCGGCGTACCAGG
TP8252_Hit	D	1	chr3	.	CAGCGATTCAAAATTGGGCTTTGCAGAGGAGTACCTACGTCATAGAATACAATTCAAATTGCAT
TP8252_Query	D	1	chr3	.	CAGCAATTCAAAATTGGGCTTTGCAGAGGAGTACCTACGTCATAGAATACAATTCAAATTGCAT
TP82581_Hit	D	1	chr3	.	CTGCGGGGTTCATAAACTTTGTTGCCGCAAAACACCCTTGGGGATGGCGAATCTCTCTTGGCTT
TP82581_Query	D	1	chr3	.	CTGCGGGGTTCATAAACTTTGTCGCCGCAAAACACCCTTGGGGATGGCGAATCTCTCTTGGCTT
TP82964_Hit	D+G	1	chr3	.	CTGCTGTTTCCTCAGATTCTCTCGGTCTCTTTTTCGACTGCAATGGAGACAAAGAAATTGTTGT
TP82964_Query	D+G	1	chr3	.	CTGCGGTTTCCTCAGATTCTCTCGGTCTCTTTTTCGACTGCAATGGAGACAAAGAAATTGTTGT
TP8300_Hit	D+G	1	chr3	.	CAGCAATTCCAGCATAGTCAACTCTGAGCATAATGTAGGAAAGGCGTTGCGAGTGACAAGCTAA
TP8300_Query	D+G	1	chr3	.	CAGCAATTCCAGCATAGTCAACTCTCAGCATAATGTAGGAAAGGCGTTGCGAGTGACAAGCTAA
TP83157_Hit	D	1	chr3	.	CTGCGTATGATTCGTTTTTGTGTTGTTTGATTAAGGGGAGTGATGGGATTCGTGAGGCGGTTAA
TP83157_Query	D	1	chr3	.	CTGCGTATGATTCGTTTTTGTGTTGTTTGATTAAGGGGAGTGATGGGATTCGTGAGGCAGTTAA
TP83279_Hit	D	1	chr3	.	CTGCGTCCAGAAGTTGTAAAAATACTTGACGAGGAGTCTTCTTGGACAATTTATGAGGTAGCAT
TP83279_Query	D	1	chr3	.	CTGCGTCCAGAAGTTGTAAAAATACTTGACGAGGAGTCTCCTTGGACAATTTATGAGGTAGCAT
TP8337_Hit	D	1	chr3	.	CAGCAATTCTCGCTTAACTTTCCATTCTTCTTCAGCTATTACCTCCAAAGAAAGTGAAGTTAAG
TP8337_Query	D	1	chr3	.	CAGCAATTCTCGCTTAACTTTCCATTCTTCTTCAGCTATTACCTCCAAACAAAGTGAAGTTAAG
TP83526_Hit	D+G	1	chr3	.	CTGCGTGCCGGACTAATTGTACAAATGATGGCTGTTCTGGCATTGCCTCCTAAGGAGTTATGCA
TP83526_Query	D+G	1	chr3	.	CTGCGTGCAGGACTAATTGTACAAATGATGGCTGTTCTGGCATTGCCTCCTAAGGAGTTATGCA
TP83910_Hit	D	1	chr3	.	CTGCGTTGTTTACATTTTTTAGATCCAATGCAATTTTAATGCTTAATCTTTTTCTTCTTATCCC
TP83910_Query	D	1	chr3	.	CTGCGTTGTCTACATTTTTTAGATCCAATGCAATTTTAATGCTTAATCTTTTTCTTCTTATCCC
TP83984_Hit	D	1	chr3	.	CTGCGTTTGGAGAGGTAGCAAAATGAAGTGTTGGTGTTGTTGATGTTATCAATGATCTCGTTGT
TP83984_Query	D	1	chr3	.	CTGCGTTTGGAGAGGTAGCAAAATCAAGTGTTGGTGTTGTTGATGTTATCAATGATCTCGTTGT
TP84084_Hit	D	1	chr3	.	CTGCTAAAAACGACAGAGTAAAGAAAGGCGGTCCTAATGTAACATCAAATTCAGCCTTGGCTGA
TP84084_Query	D	1	chr3	.	CTGCTAAAAACGACAGAGTAAAGAAAGGCGGTCCTAACGTAACATCAAATTCAGCCTTGGCTGA
TP8431_Hit	D	1	chr3	.	CAGCAATTGCGTTCTCTTGCGTTGTTGAAGTTAGAACAAGATCAATCAACGGTGGCACAGTTCC
TP8431_Query	D	1	chr3	.	CAGCAATTGCGTTCTCTTGCGTTGTTGAAGTTAGAACAAGATCAATCAAAGGTGGCACAGTTCC
TP84358_Hit	D	1	chr3	.	CTGCTAACTATATGGTGCGGAGAACATAGTTCTAAATTGCGGTCTGAAACTGCAAACTGCAAGT
TP84358_Query	D	1	chr3	.	CTGCTAACTATATGGTGCGGAGAACATAGTTCTAAATTGCGGTCTGAAACTGCAAACTGCAAAT
TP8443_Hit	D+G	1	chr3	.	CAGCCATTGCTTTAGCCAAGCAGGAAGGCCTCCTTGTTTCCTTGGACTTGGCCAGTTTTGAGGT
TP8443_Query	D+G	1	chr3	.	CAGCAATTGCTTTAGCCAAGCAGGAAGGCCTCCTTGTTTCCTTGGACTTGGCCAGTTTTGAGGT
TP84478_Hit	D	1	chr3	.	CTGCTAAGGTTAACACAACTTTTGCATGCCATGGATAGCTAGATAGGTAATTCAGTAACACCAT
TP84478_Query	D	1	chr3	.	CTGCTAAGGTTAACACAACTTTTGCATGCCATGGATAGCTAGATAGGTAATTCACTAACACCAT
TP8467_Hit	D	1	chr3	.	CAGCAATTGGGAGGGTTTACTTGGACTTAAGTTTTTGTAAGAGCGTACAAAAAAGTTATGATAT
TP8467_Query	D	1	chr3	.	CAGCAATTGGGAGGGTTTACTTGGACTTAAGTTTTTGCAAGAGCGTACAAAAAAGTTATGATAT
TP84679_Hit	D	1	chr3	.	CTGCTAATTCTGATATTTTTCAGCTTTGTGATGCGGTTGAACAGAATACTCATTTGTTGAAGCT
TP84679_Query	D	1	chr3	.	CTGCTAATTCTGATATTTTTCAGCTTTGTGATGCGGATGAACAGAATACTCATTTGTTGAAGCT
TP84697_Hit	D	1	chr3	.	CTGCTAATTGTTTCATCATCTTCCTTCCTAATTCTCCAAAGCTACCACTACCATCTGGTGTATC
TP84697_Query	D	1	chr3	.	CTGCTAATTGTTTCATCATCTTCCTTCCTAACTCTCCAAAGCTACCACTACCATCTGGTGTATC
TP84768_Hit	D	1	chr3	.	CTGCTACAACTGGAGAGGAGTTGGAAGGAAACACAATTTCCTTCCTACGCACTGCTGAGACATT
TP84768_Query	D	1	chr3	.	CTGCTACAACTGGAGAGGAGTTGGAAGGAAACACAACTTCCTTCCTACGCACTGCTGAGACATT
TP84799_Hit	D	1	chr3	.	CTGCTACACAAGGAACAACAAGACAGACGTCAAAAAAGGAAGTGGTGAGAAGCCAGATGAGTAA
TP84799_Query	D	1	chr3	.	CTGCTACACAAGGAACAACAAGACAGACGTCAAAAAAGGAAGTAGTGAGAAGCCAGATGAGTAA

Name	Filter	Nb hit (Mt4.0)	Mt Chr (Mt4.0)	Ms Chr (Li et al., 2014)	Sequence
TP84855_Hit	D+G	1	chr3	.	CTGCTACAGATACAACGACGATGCAATCCCCTGAAGGAACTGCCTTCACCTTCGCCTTATACCA
TP84855_Query	D+G	1	chr3	.	CTGCTACAGATACAACGACGATACAATCCCCTGAAGGAACTGCCTTCACCTTCGCCTTATACCA
TP85189_Hit	D+G	1	chr3	.	CTGCTACTATACTACTATTTCTTATTGTGTAAAGGGTTGGCTCCTGTAGGTACTCTTCGTTTCT
TP85189_Query	D+G	1	chr3	.	CTGCTACTATACTACTATTTCTTATTGTGTAAAGGGTTGGCTCCTGTAGGCACTCTTCGTTTCT
TP85242_Hit	D	1	chr3	.	CTGCTACTCTTCAGCATATCAACAATCATTGGTATAAGCCCTGCACTGCGGACAACATGTTTAT
TP85242_Query	D	1	chr3	.	CTGCTACTCTTCAGCATATCAACAATCATCGGTATAAGCCCTGCACTGCGGACAACATGTTTAT
TP85493_Hit	D+G	1	chr3	.	CTGCTAGACTAGACCCTAACAACAAGATGGCAAACAAGGTGTTGAGAAAGGCTCGAGCTGTGAC
TP85493_Query	D+G	1	chr3	.	CTGCTAGACTAGACCCTAACAACAAGATGGCAAACAAGGTGTTGAGAAAGGCCCGAGCTGTGAC
TP85646_Hit	D	1	chr3	.	CTGCTAGCGTGGAAACTAAGGTTTTTCCCTCTCCCGTTTTCATCTCAGCAATGGACCCATCGTG
TP85646_Query	D	1	chr3	.	CTGCTAGCGTGGAAACTAAGGTTTTTCCCTCTCCCGTTTTCATCTCAGCAATGGACCCATCATG
TP85695_Hit	D	1	chr3	.	CTGCTAGGAAAACAGCTGAGAATGCGATTAACCTTAAGCGGGTCGAAGACCAGGTTAATAATGC
TP85695_Query	D	1	chr3	.	CTGCTAGGAAAACAGCTGAGAATGCGATTAACCTTAAGCGGGTCGAAGACCAGGTTAACAATGC
TP85848_Hit	D	1	chr3	.	CTGCTAGTCCAGTAACTGATATACAAGAAGAAGGAATGCTACGAATTTGCCTGTTGCACTCGAG
TP85848_Query	D	1	chr3	.	CTGCTAGTCCAGTAACTGATATACAAGAAGAAGGAATGCTACGAATTTGCATGTTGCACTCGAG
TP85898_Hit	D	1	chr3	.	CTGCTAGTGGTTTTATTTGTGGGCTTTCTTGAGATGTAAATGTGATATAAGGGATGTAATTTAT
TP85898_Query	D	1	chr3	.	CTGCTAGTGGTTTTATTTGTGGGCATTCTTGAGATGTAAATGTGATATAAGGGATGTAATTTAT
TP85917_Hit	D	1	chr3	.	CTGCTAGTTCATCATATGAGAATTCAACTGATTTGTCGACTGTTATGCCTGTCATCACACCAGG
TP85917_Query	D	1	chr3	.	CTGCTAGTTCATCATATGAGAATTCAACTGATTTGTCGACTGTTATGCCGGTCATCACACCAGG
TP85961_Hit	D	1	chr3	.	CTGCTATAAACCGAAAGCATGTCATTCATAATTTGGCAATTGATAACACTCAATTTGCAACCCA
TP85961_Query	D	1	chr3	.	CTGCTATAAACCAAAAGCATGTCATTCATAATTTGGCAATTGATAACACTCAATTTGCAACCCA
TP85963_Hit	D	1	chr3	.	CTGCTATAAAGAAGCTTATTGGTTCTGTTAGTCCCAATGCACTTGATTCTATTCTTAAATATAC
TP85963_Query	D	1	chr3	.	CTGCTATAAAGAAGCTTATTGGTTCTGTTAGTCCCAATGCACTCGATTCTATTCTTAAATATAC
TP86014_Hit	D	1	chr3	.	CTGCTATAATTCGAATATTTGGGGTAACGAAGGAAGGTTAGTTAATGTTATTTTTTCTTCAACC
TP86014_Query	D	1	chr3	.	CTGCTATAATTCGAATATTCGGGGTAACGAAGGAAGGTTAGTTAATGTTATTTTTTCTTCAACC
TP86068_Hit	D+G	1	chr3	.	CTGCTATAGCACTGTTGCGTGGCGGAATTTGAACAAACCGCCTTTTCCCTCGTGCTGAAAAAAA
TP86068_Query	D+G	1	chr3	.	CTGCTATAGCACTGTTGCGTGGCGGAATTTGAACAAACCGCCTTTTCCCTCGGGCTGAAAAAAA
TP86108_Hit	D	1	chr3	.	CTGCTATAGGCTATAGCCTCTATTTGACAACAATTTGCATTAAATTTCGTATCGCAGAACAATA
TP86108_Query	D	1	chr3	.	CTGCTATAGGCTATAGCCTCTATTTGACAACAATTTGAATTAAATTTCGTATCGCAGAACAATA
TP86124_Hit	D	1	chr3	.	CTGCTATAGTTCCAGCATATTGCTGATAAATAGGAGGAGAGGTTGAAAGAGAGTATCTTATGAT
TP86124_Query	D	1	chr3	.	CTGCTATAGTTCCAGCATATTGCTGATAAATAGGAGGAGAGGTTGAAAGAGAGTATCTTACGAT
TP8617_Hit	D	1	chr3	.	CAGCAATTTGGGTTGTTGTTTTGCCAGTCTGTTTTAGCAGATCTCTGAAGAATCCAACTGGGGA
TP8617_Query	D	1	chr3	.	CAGCAATTTGGGTTGTTGTTTTGCCAGTCAGTTTTAGCAGATCTCTGAAGAATCCAACTGGGGA
TP86178_Hit	D	1	chr3	.	CTGCTATATTGCTATATATCTACATGACTTATCAGAGACAATTTGCATCCACAGCAACAAAGAA
TP86178_Query	D	1	chr3	.	CTGCTATATTGCTATATATCTACATCACTTATCAGAGACAATTTGCATCCACAGCAACAAAGAA
TP8618_Hit	D	1	chr3	.	CAGCAATTTGGGTTGTTGTTTTGCCGGTCTGTTTTAGCAGTTCTCTGCAGAATCCAACTGGTCT
TP8618_Query	D	1	chr3	.	CAGCAATTTGGGTTGTTGTTTTGCCAGTCTGTTTTAGCAGTTCTCTGCAGAATCCAACTGGTCT
TP866_Hit	D	1	chr3	.	CAGCAAAATCAAACAACTGATTCCGAGCAAAAATGCACCTAAAAATTAGAAAATGTAAGAGATG
TP866_Query	D	1	chr3	.	CAGCAAAATCAAACAACTGATTCCGAGCAAAAATGCACCTAAAAATTAGAAAATGTAAGAAATG
TP86798_Hit	D	1	chr3	.	CTGCTATTGTAGATGAGCGACCTTATGTCGAAATATTCTTATCAACCCAATGCACATTCAGGAT
TP86798_Query	D	1	chr3	.	CTGCTATTGTAGATGAACGACCTTATGTCGAAATATTCTTATCAACCCAATGCACATTCAGGAT
TP87167_Hit	D	1	chr3	.	CTGCTCAAGTTGAGAGATACAGTTCAACAGGAAGTGAGGAATCTTCGGACTCAAGTGAAGAGGA
TP87167_Query	D	1	chr3	.	CTGCTCAAGTTGAGAGATACAGTTCAACAGGAAGTGAGGAATCTTCAGACTCAAGTGAAGAGGA
TP87177_Hit	D	1	chr3	.	CTGCTCAATACTTTGACGGTAAAGAGCCAAAGAGGTTACTCTATTATTAGGGTTTACTATATGG
TP87177_Query	D	1	chr3	.	CTGCTCAATACTTTGACGGTAAAAAGCCAAAGAGGTTACTCTATTATTAGGGTTTACTATATGG
TP87390_Hit	D+G	1	chr3	.	CTGCTCAGTCCAGCATTCTTACCACATATAGGACAAGTTTTCATTGTTGGTTTCCGCCGCTGAA
TP87390_Query	D+G	1	chr3	.	CTGCTCAGTCCAGCATTCTTACCACATATAGGACAAGTTTTCATCGTTGGTTTCCGCCGCTGAA
TP87391_Hit	D	1	chr3	.	CTGCTCAGTCCAGCATTCTTACCACATATAGGACAAGTTTTCATTGTTGGTTTCCGCTGAAAAA
TP87391_Query	D	1	chr3	.	CTGCTCAGTCCAGCATTCTTACCACATATAGGACAAGTTTTCATCGTTGGTTTCCGCTGAAAAA
TP87392_Hit	D	1	chr3	.	CTGCTCAGTCCAGCATTCTTACCACATATAGGACAAGTTTTCATTGTTGGTTTCCGGTGCTGAA
TP87392_Query	D	1	chr3	.	CTGCTCAGTCCAGCATTCTTACCACATATAGGACAAGTTTTCATCGTTGGTTTCCGGTGCTGAA
TP87956_Hit	D	1	chr3	.	CTGCTCCGGCAGGTTACACAGAAAAACCGGTGGACCACGACGGAAGAGATGCGGAAGTGGCCGG
TP87956_Query	D	1	chr3	.	CTGCTCCGGCAGGTTACACAAAAAAACCGGTGGACCACGACGGAAGAGATGCGGAAGTGGCCGG
TP88213_Hit	D	1	chr3	.	CTGCTCCTGGTGGTGTTATTACTCTTCATGGAAGATTTGAGATACTTTCGCTTTCGGGTGCTTT
TP88213_Query	D	1	chr3	.	CTGCTCCTGGTGGTGTTATTACTCTTCATGGAAGATTCGAGATACTTTCGCTTTCGGGTGCTTT

Name	Filter	Nb hit (Mt4.0)	Mt Chr (Mt4.0)	Ms Chr (Li et al., 2014)	Sequence
TP88266_Hit	D	1	chr3	.	CTGCTCCTTTCTCTACGAATGCAAAGACAGGTTGAACATATACTTGGTATGCTCCCACAAGGTG
TP88266_Query	D	1	chr3	.	CTGCTCCTTTCTCTACGAAGGCAAAGACAGGTTGAACATATACTTGGTATGCTCCCACAAGGTG
TP89016_Hit	D	1	chr3	.	CTGCTCTGCAATCCCTCTAGAGTAATACCTCTTACATATAACAATGATGCATAAACCAAAGCCA
TP89016_Query	D	1	chr3	.	CTGCTCTGCAATCCCTCTAGAGTAATACCTCTTACATATAACAATGATGCATAAACCAAAACCA
TP89141_Hit	D+G	1	chr3	.	CTGCTCTTAGGGAACGATGGAACAACCGGAATGCATGCCCATCCATGGCCCGATCGTGGAGGAT
TP89141_Query	D+G	1	chr3	.	CTGCTCTTAGGGAACGATGGAACAACCGGAATGCATGCCCATCCATGGCCAGATCGTGGAGGAT
TP89308_Hit	D	1	chr3	.	CTGCTCTTGTGAGTCAACCAGCTCTTTATAGCAAGGAGCTCATGCATCAACAGCCAGTTGGACC
TP89308_Query	D	1	chr3	.	CTGCTCTTGTGAGTCAACCAGCTCTTTATAACAAGGAGCTCATGCATCAACAGCCAGTTGGACC
TP8932_Hit	D	1	chr3	.	CAGCACAACTCAAAAGGAAACGGTAAAATGATACCTAAACTTGGTCTTGTTACATGAAAAATTT
TP8932_Query	D	1	chr3	.	CAGCACAACTCAAAAGGAAACAGTAAAATGATACCTAAACTTGGTCTTGTTACATGAAAAATTT
TP89577_Hit	D	1	chr3	.	CTGCTGAAGAAGCAAACTCATTGACTTAGGTGAGTAAGGAAAAGGTGAAGTGGCCATAAGCTGA
TP89577_Query	D	1	chr3	.	CTGCTGAAGAAGCAAACTCATTGACTTAGGTGAATAAGGAAAAGGTGAAGTGGCCATAAGCTGA
TP89664_Hit	D+G	1	chr3	.	CTGCTGAAGTTGATGAATTAATCACACCATCTGTGGAAATAAGATACACAAAGAATCTCATTAA
TP89664_Query	D+G	1	chr3	.	CTGCTGAAGTTGATGAATTAATCACACCATCAGTGGAAATAAGATACACAAAGAATCTCATTAA
TP89715_Hit	D+G	1	chr3	.	CTGCTGAATGATAATATACATTGGCTGTTGAAACGATAATAGTTGAATGGAATCGTACTTAACT
TP89715_Query	D+G	1	chr3	.	CTGCTGAATGATAATATACATTGGCTGTTGAAACGATAATAGTTGAATGGAATCATACTTAACT
TP89837_Hit	D	1	chr3	.	CTGCTGACTTTGCTTGCAATCATTTGCCAAAGTTCATTCTCGATGATAAAGATTTCCCAGTGGA
TP89837_Query	D	1	chr3	.	CTGCTGACTTTGCTTGCAATCATTTGCCAAAGTTCATTCTCGAGGATAAAGATTTCCCAGTGGA
TP89923_Hit	D+G	1	chr3	.	CTGCTGAGGAATCAAAAGAAACTATTGCTAATACTCTTCATGGCTCAGATTTAGTGTTTGTAAC
TP89923_Query	D+G	1	chr3	.	CTGCTGAGGAATCAAAAGAAACTATTGCTAATGCTCTTCATGGCTCAGATTTAGTGTTTGTAAC
TP89992_Hit	D	1	chr3	.	CTGCTGAGTGAGCAAGAGCGTGAGAAGCGTAGGGTCTTGAAAGAAGATTGGGACAAGTGGGCCA
TP89992_Query	D	1	chr3	.	CTGCTGAGTGAACAAGAGCGTGAGAAGCGTAGGGTCTTGAAAGAAGATTGGGACAAGTGGGCCA
TP90025_Hit	D	1	chr3	.	CTGCTGAGTTGGCTACACTTGCTTTCTATGTGTTTACGGGGTATAAGTTCAAGCCAGAGGCGCA
TP90025_Query	D	1	chr3	.	CTGCTGAGTTGGCTACACTTGCTTTCTATGTGTTCACGGGGTATAAGTTCAAGCCAGAGGCGCA
TP90082_Hit	D	1	chr3	.	CTGCTGATATCAAGGATATGCTGGGACATGCTGTCTTTGAAGTTCACAATCTGTTTAACCAAAT
TP90082_Query	D	1	chr3	.	CTGCTGATATCAAGGATATGCTGGGACATCCTGTCTTTGAAGTTCACAATCTGTTTAACCAAAT
TP90096_Hit	D+G	1	chr3	.	CTGCTGATATTGAATTAATAGGGTGAGGTCCAGTATCTGATTAAATGGTTAGTCACTCACTCAC
TP90096_Query	D+G	1	chr3	.	CTGCTGATATTGAATTAATAGGGTGAGGTCCAGTACCTGATTAAATGGTTAGTCACTCACTCAC
TP90283_Hit	D	1	chr3	.	CTGCTGATTCCAGAGTAATGGATTTTGGATCAGTTTTCTTATTGTCCTCTGGGAAAGCTGACAG
TP90283_Query	D	1	chr3	.	CTGCTGATTCCAGAGTAATGGATTTTGGATCAGTTTTCCTATTGTCCTCTGGGAAAGCTGACAG
TP90370_Hit	D	1	chr3	.	CTGCTGGAACCACGGCTAAGCCAAGAGAGATTCGCCATCCCCAAGGGTGTTTTGCGGCGACAAA
TP90370_Query	D	1	chr3	.	CTGCTGGAACCACGGCTAAGCCAAGAGAGATTCGCCATCCCCAAGGGTGTTTTGCGGCAACAAA
TP90418_Hit	D	1	chr3	.	CTGCTGGACGATGGTGTGAAGAGTTCGATGAACATCTCTGGCCATGCCCTTGGCATCACCACAT
TP90418_Query	D	1	chr3	.	CTGCTGGACAATGGTGTGAAGAGTTCGATGAACATCTCTGGCCATGCCCTTGGCATCACCACAT
TP90475_Hit	D	1	chr3	.	CTGCTGGAGCTATGGAGAATTATGGGTTGGTTACATATCGGGAGACTGCTTTACTCTATGATGA
TP90475_Query	D	1	chr3	.	CTGCTGGAGCCATGGAGAATTATGGGTTGGTTACATATCGGGAGACTGCTTTACTCTATGATGA
TP90597_Hit	D	1	chr3	.	CTGCTGGCAGGTAATTAGTACTACTAATTTATTTTCTACTGATAATTTGAATTTGAAAAAATGT
TP90597_Query	D	1	chr3	.	CTGCTGGCAGGTAATTAGTACTACTAATTTAGTTTCTACTGATAATTTGAATTTGAAAAAATGT
TP9076_Hit	D	1	chr3	.	CAGCACGAGTGACAGCTCGATAGCGGGAAAGACTCTCTTCAATACTACAATTAATGTTTGATTT
TP9076_Query	D	1	chr3	.	CAGCACAAGTGACAGCTCGATAGCGGGAAAGACTCTCTTCAATACTACAATTAATGTTTGATTT
TP90840_Hit	D+G	1	chr3	.	CTGCTGGGCCAACACACTATGAGGAAATGCCCAACATATCCAAGAGAGTGAGGTTAACATTTGA
TP90840_Query	D+G	1	chr3	.	CTGCTGGGCCAACACACTATGAGGAAATACCCAACATATCCAAGAGAGTGAGGTTAACATTTGA
TP90915_Hit	D+G	1	chr3	.	CTGCTGGGTCCCTTGCCATTCATCTTGCTAATAACGACCCACAAATTGTCATAGAGATTCCACC
TP90915_Query	D+G	1	chr3	.	CTGCTGGGTCCCTTGCCATTCATCTTGCTAATAACAACCCACAAATTGTCATAGAGATTCCACC
TP90930_Hit	D+G	1	chr3	.	CTGCTGGGTTAACTCCGGTGAGTTTTCATGGTTTGTTGAGTGGTGTATCGGAGAAGGGATTCTC
TP90930_Query	D+G	1	chr3	.	CTGCTGGGTTAACTCCGGTGAGTTTTCATGGTTTGTTGAGTGGTGTATCAGAGAAGGGATTCTC
TP91093_Hit	D	1	chr3	.	CTGCTGGTGCATCTGCTGGTGCAGTGACGGATCCAAGTTCACAAGATCCAAATGGTAAAACTGC
TP91093_Query	D	1	chr3	.	CTGCTGGTGCATCTGCTGGAGCAGTGACGGATCCAAGTTCACAAGATCCAAATGGTAAAACTGC
TP91116_Hit	D	1	chr3	.	CTGCTGGTGCTGGTGCTCCTCCTTCTGGTGTGTCTGCAGGCAAATCTTCTGCTGAAAAAAAAAA
TP91116_Query	D	1	chr3	.	CTGCTGGTGCTGGTGCTCCTCCTTCTGGTGTGTCTGCAGGCAAATCTTCTGCAGAAAAAAAAAA
TP91566_Hit	D	1	chr3	.	CTGCTGTCAAAAAAATTACACAGTGAAACAGATTAGAGCAATTGGTTTTCTGACTGTAGTGTGA
TP91566_Query	D	1	chr3	.	CTGCTGTCAAAAAAACTACACAGTGAAACAGATTAGAGCAATTGGTTTTCTGACTGTAGTGTGA
TP9167_Hit	D	1	chr3	.	CAGCACAATGTGTAATTTCTTCTGGATAAACTACACTTTCTAGTAAATTTGCTGTAGTGTCATC
TP9167_Query	D	1	chr3	.	CAGCACAATGTGTAATTTCTTCTGGATAAACTACACTTTCTAATAAATTTGCTGTAGTGTCATC

Name	Filter	Nb hit (Mt4.0)	Mt Chr (Mt4.0)	Ms Chr (Li et al., 2014)	Sequence
TP91898_Hit	D+G	1	chr3	.	CTGCTGTGCAACTCAGGCAACATGTCTCTCATAGGCGTACTTTTTACTATTTGGTGCTGAAAAA
TP91898_Query	D+G	1	chr3	.	CTGCTGTGCAACTCAGGCAACACGTCTCTCATAGGCGTACTTTTTACTATTTGGTGCTGAAAAA
TP91905_Hit	D	1	chr3	.	CTGCTGTGCAGGCGTATCGTCGGGAAGAGGAATCACAATTTTACCGGTCATCCAAAAGAAAAAG
TP91905_Query	D	1	chr3	.	CTGCTGTGCAGGCGTATCGTCGGGAAGAGGAATCACAATTTTACAGGTCATCCAAAAGAAAAAG
TP92009_Hit	D	1	chr3	.	CTGCTGTGGTATTTGTATAGGGCACCACCATTTTCCCGCTATCTTTTCCCAGCAACGCTGAAAA
TP92009_Query	D	1	chr3	.	CTGCTGTGGTATTTGTATAGGGCACCACCATTTTCCCGCTATCTTTTCCCAGCAACGCAGAAAA
TP92041_Hit	D	1	chr3	.	CTGCTGTGTCTGATTGACTTGTTTCAACCGGGCGAGGTTCGGTATGAGTATCAATGGAGGAATT
TP92041_Query	D	1	chr3	.	CTGCTGTGTCTGATTGACTTGTTTCAACCGGGCGAGGTTCGGCATGAGTATCAATGGAGGAATT
TP92373_Hit	D	1	chr3	.	CTGCTGTTGCCCAATAATCCCCTGCAACCCAACACCAGTATTACCACCCTGTTCCCCAATCCCA
TP92373_Query	D	1	chr3	.	CTGCTGTTGCCCAATAATCCCCTGCAACCCAACACCAGTATCACCACCCTGTTCCCCAATCCCA
TP9240_Hit	D+G	1	chr3	.	CAGCACACACCTCTTGAAATGCTGGACAAGAAGCATTTTGCCAAGGGGGCGCGTGGTTTAGAAA
TP9240_Query	D+G	1	chr3	.	CAGCACACACATCTTGAAATGCTGGACAAGAAGCATTTTGCCAAGGGGGCGCGTGGTTTAGAAA
TP92857_Hit	D	1	chr3	.	CTGCTTACGGTGATTTCTCCGATGATGAGATTGAAAGCCCCAAGAAAATGGGTGTTTATGTCAA
TP92857_Query	D	1	chr3	.	CTGCTTACGGTGATTTCTCCGATGATGAGATTGAAAGCCCCAAGAAAATGGGTGGTTATGTCAA
TP93125_Hit	D	1	chr3	.	CTGCTTCAAAAATAGGATTTTATCTAATAAAGGCATCTGCTGTGCAGGAAAGGTGGCCTGAGAA
TP93125_Query	D	1	chr3	.	CTGCTTCAAAAATAGGATTTTATCTAATAAAGGCATCTGCTGTGCAGGAAAGGTGACCTGAGAA
TP9315_Hit	D	1	chr3	.	CAGCACACCAGTTGGGAAACCAGTAAACCAAGTGTATGTTGTGTTTATTCTTCTTGGTGCTTTA
TP9315_Query	D	1	chr3	.	CAGCACACCAGTTGGGAAACCAGTAAACCAAATGTATGTTGTGTTTATTCTTCTTGGTGCTTTA
TP93423_Hit	D+G	1	chr3	.	CTGCTTCCACCGTTTCAAACATCAAATACACAAAACCTGCACTATTTCTGTATACAAAATGAAT
TP93423_Query	D+G	1	chr3	.	CTGCTTCCACCGTTTCAAACATCAAATACACAAAACCTGCACTATTTCTGTATACAAAATGAAA
TP93432_Hit	D	1	chr3	.	CTGCTTCCACTTTCTTCATGATCAAGAAGAACAAGTTCATGTTCTTGTTCTTCTTTTTTGTTCT
TP93432_Query	D	1	chr3	.	CTGCTTCCACTTTCTTCATGATCAAGAAGAACAAGTACATGTTCTTGTTCTTCTTTTTTGTTCT
TP9352_Hit	D	1	chr3	.	CAGCACACGAGGAAGTGGAGCGATTGGAGAGGGTAATAGTGAAGGATCTCCAGAATGAACCAAC
TP9352_Query	D	1	chr3	.	CAGCACACGAGGAAGTGGAGCGATTGGAGAGGGTAATAGTGAAGGATCTCCAAAATGAACCAAC
TP93775_Hit	D	1	chr3	.	CTGCTTCTCGGAACTAGGTTTGTTCTCCACTAAACCAGAAGGCCCGCCAGGCGGACCATTTTGA
TP93775_Query	D	1	chr3	.	CTGCTTCTCGGAACTAGGTTTGTTCTCCACTAAACCAGAAGGCCCGCCAGGAGGACCATTTTGA
TP93853_Hit	D+G	1	chr3	.	CTGCTTCTGCTCCTTCTGCAACACCATCACCATCAGGGAAAGGTTCACCTTCCTATGGCACCCC
TP93853_Query	D+G	1	chr3	.	CTGCTTCTGCTCCTTCTGCAACACCATCACCATCAGGGAAAGGTTCACCTTCCGATGGCACCCC
TP93928_Hit	D	1	chr3	.	CTGCTTCTTCAAGTTCTCTCAACCCTCCCACTTTTTGTTCTATTTCCTCAATCAACTCCTCACG
TP93928_Query	D	1	chr3	.	CTGCTTCTTCAAGTTCTCTCAACCCTCCAACTTTTTGTTCTATTTCCTCAATCAACTCCTCACG
TP940_Hit	D	1	chr3	.	CAGCAAAATCTTTTTCAATTTCCGCAAACAAGTTAGTGTCACCAAGGCGAATTTTGTCGCCAAT
TP940_Query	D	1	chr3	.	CAGCAAAATCTTTTTCAATTTCCGCAAACAAGTTAGTGTCACCAAGACGAATTTTGTCGCCAAT
TP94111_Hit	D	1	chr3	.	CTGCTTGAGATAATTCAACAGTGCATTGAGAACACTCTAGGGAATCTGAAGGAAGAGCTGGGCT
TP94111_Query	D	1	chr3	.	CTGCTTGAGATAATTCAACAGTGCATTGAGAACACTCTAGGGAATCTGAAGGAAGAGCTGGGCC
TP94167_Hit	D	1	chr3	.	CTGCTTGGTCTATATCAGAAACTGAAATTTCCTAATAAAAAAGAAACCACACCTTATCCCATTA
TP94167_Query	D	1	chr3	.	CTGCTTGATCTATATCAGAAACTGAAATTTCCTAATAAAAAAGAAACCACACCTTATCCCATTA
TP9455_Hit	D+G	1	chr3	.	CAGCACAGAAGTCACCTCGCAGGATTAAAGGATTTTCCAAACCTTCAGTTGGACTGTTGGTGCT
TP9455_Query	D+G	1	chr3	.	CAGCACAGAAGTCACCTCGCAGGATTAAAGGATTTTCCAAAACTTCAGTTGGACTGTTGGTGCT
TP94575_Hit	D+G	1	chr3	.	CTGCTTGTGAACTACTATATAATGCCGGTTACACAAACCTGTTTTGGGTTCAAGGAGGCTTTGG
TP94575_Query	D+G	1	chr3	.	CTGCTTGTGAACTACTATATAATGCCGGCTACACAAACCTGTTTTGGGTTCAAGGAGGCTTTGG
TP94770_Hit	D	1	chr3	.	CTGCTTTACCAAAACCTTGTCTGACATGACGACGTTGACCCGAGCATTAGTAGTCACCTGCTTT
TP94770_Query	D	1	chr3	.	CTGCTTTACCAAAACCTTGTCTGACATGACGAGGTTGACCCGAGCATTAGTAGTCACCTGCTTT
TP94913_Hit	D	1	chr3	.	CTGCTTTCACCTCGGTTCTTCGATTTCTTTGAGTAAGTCTCCCATGGCCCATCATCCTGTCCCG
TP94913_Query	D	1	chr3	.	CTGCTTTCACCTCGGTTCTTCGATTTCTTTGAGTAAGTCTCCCATGGCCCATCATCCAGTCCCG
TP94980_Hit	D	1	chr3	.	CTGCTTTCCTTGCTTGTGCCTTTTCATCGGAGAATATCATACACATCAGAAATTGATTAAATAA
TP94980_Query	D	1	chr3	.	CTGCTTTCCTTGCTTGTGCCTTTTCATCAGAGAATATCATACACATCAGAAATTGATTAAATAA
TP94984_Hit	D	1	chr3	.	CTGCTTTTGAAGTTTGAGAACTATTTGATTTTTTTTTTCTCCTGTTCAGTTCCACATTTACCTT
TP94984_Query	D	1	chr3	.	CTGCTTTCGAAGTTTGAGAACTATTTGATTTTTTTTTTCTCCTGTTCAGTTCCACATTTACCTT
TP95140_Hit	D	1	chr3	.	CTGCTTTGACGGCTTCTTCAGGTGCATTGGCTGATCTGGCTTCAGCTTCAGCTGAAAAAAAAAA
TP95140_Query	D	1	chr3	.	CTGCTTTGACGGCTTCTTCAGGTGCATTGGCTGATCTGGCTTCAGCTTCAGCAGAAAAAAAAAA
TP95378_Hit	D+G	1	chr3	.	CTGCTTTGTTTTTCTGTTCCTCTGTTCCGAAAACTAGTCTCCGAGCTAATGACCATGACGTGAA
TP95378_Query	D+G	1	chr3	.	CTGCTTTGTTTTTCTGTTCATCTGTTCCGAAAACTAGTCTCCGAGCTAATGACCATGACGTGAA
TP95402_Hit	D	1	chr3	.	CTGCTTTTAGAGGAAGGGCTTTACCTTCTGGTTTCAATGCTTGGATTTCTATCGCAATATTCGC
TP95402_Query	D	1	chr3	.	CTGCTTTTAGAGGAAGAGCTTTACCTTCTGGTTTCAATGCTTGGATTTCTATCGCAATATTCGC

Name	Filter	Nb hit (Mt4.0)	Mt Chr (Mt4.0)	Ms Chr (Li et al., 2014)	Sequence
TP95438_Hit	D	1	chr3	.	CTGCTTTTTAACTATGTATGTTGGATTTTATGGCATCCATCTTTCTTGATTTTCATCAAATCTT
TP95438_Query	D	1	chr3	.	CTGCTTTTCAACTATGTATGTTGGATTTTATGGCATCCATCTTTCTTGATTTTCATCAAATCTT
TP95463_Hit	D+G	1	chr3	.	CTGCTTTTCCCTGACTTGCCATTTGGAATCAGGATATAAAGAGTGAGATAAATCCGGTTTTAAA
TP95463_Query	D+G	1	chr3	.	CTGCTTTTCCCTGACTTGCCATTTGGAATCAGGACATAAAGAGTGAGATAAATCCGGTTTTAAA
TP95581_Hit	D	1	chr3	.	CTGCTTTTGATTTCAGAGGATAAGGCAAAAGCACCTTGTTTGCGGGATCAAAGTGAGGGTAGGA
TP95581_Query	D	1	chr3	.	CTGCTTTTGATTTCAGAGGATAAGGCAAAAGCACCTTGTTCGCGGGATCAAAGTGAGGGTAGGA
TP95650_Hit	D	1	chr3	.	CTGCTTTTGTTAGCTGATACAATCTGTATACCTGGCAAGTCAATTCAATTTCAAAATGTTACAG
TP95650_Query	D	1	chr3	.	CTGCTTTTGTTAGCTGATACAATCTGTATACCTGGCAAGTCAATTCAATTTCAAAATGTCACAG
TP9586_Hit	D+G	1	chr3	.	CAGCACAGTAGCCTGAGAACAAGTTTCACGAAATTCAGACCTGACCACTTCCCCACCTTTCTCT
TP9586_Query	D+G	1	chr3	.	CAGCACAGTAGCCTGAGAACAAGTTTCACGAAATTCAGACCTGACAACTTCCCCACCTTTCTCT
TP959_Hit	D	1	chr3	.	CAGCAAAATGAGACAGCAATATGACTCCTATCCCAAAATTGAGATTGAATCCGTGGCTTAAGGG
TP959_Query	D	1	chr3	.	CAGCAAAATGAGACAGCAATATGACTCATATCCCAAAATTGAGATTGAATCCGTGGCTTAAGGG
TP9685_Hit	D+G	1	chr3	.	CAGCACATAGTAGTTGGGGAGGGGAAGTGAACTCATGTAGTGAATACATTGTTCTGTGGTGAAG
TP9685_Query	D+G	1	chr3	.	CAGCACATAGTAGTTGGGGAGGGAAAGTGAACTCATGTAGTGAATACATTGTTCTGTGGTGAAG
TP9840_Hit	D	1	chr3	.	CAGCACATGGAGTCCAAAAGATTTTCGGTCTCGCATGTAGAAACCTTACCGGCCAAAACATAAT
TP9840_Query	D	1	chr3	.	CAGCACATGGAGTCCAAAAGATTTTCGGTCTCGCAAGTAGAAACCTTACCGGCCAAAACATAAT
TP9867_Hit	D	1	chr3	.	CAGCACATGTGCTTGTTCCAGGCTTCTCAAAGATTCTAACCTGCATGATGCATTATGTATTTCT
TP9867_Query	D	1	chr3	.	CAGCACATGTGCTTGTTCCAGGCTTCTCAAAGATTCTAACCTGCACGATGCATTATGTATTTCT
TP9877_Hit	D+G	1	chr3	.	CAGCACATGTTGCCAATCTTTTGTCAACAGAGCCTCCAAATAGTATGTTTTCTATTATGCCTCG
TP9877_Query	D+G	1	chr3	.	CAGCACATGTTGCCAATCTTTTGTCAACAGAGCCTCCAAATAGTATGTTTTCTATTATCCCTCG
TP9926_Hit	D	1	chr3	.	CAGCACATTGAAATATCCATAGGAATGTTCATTCTAATGTTTGTAGAAAAACATCTACACCACA
TP9926_Query	D	1	chr3	.	CAGCACATTGAAATATCCATAGAAATGTTCATTCTAATGTTTGTAGAAAAACATCTACACCACA
TP994_Hit	D+G	1	chr3	.	CAGCAAAATGTACTTTCATGTTACCAAAATGATGTCTGGTTTTTCTATTTTATTTTTAAAAATA
TP994_Query	D+G	1	chr3	.	CAGCAAAATGTACTTTCATGTTACCAAAATGATGTCTGATTTTTCTATTTTATTTTTAAAAATA
TP9562_Hit	D	1	chr3	2A	CAGCTCAGGGACTCCTGGAGCTAGTTCCTGGAAAGAGATTTCAGGTACTGGAACTTTTTTGAAT
TP9562_Query	D	1	chr3	2A	CAGCACAGGGACTCCTGGAGCTAGTTCCTGGAAAGAGATTTCAGGTACTGGAACTTTTTTGAAT
TP71996_Hit	D	1	chr3	2D	CTGCATGGTAATATAGTGTTGGATTTAGATTGGAATGTTGAAATTTGAGATTTTTGAAGTTAAT
TP71996_Query	D	1	chr3	2D	CTGCATGGTAATATAGTGTTGGATTTAAATTGGAATGTTGAAATTTGAGATTTTTGAAGTTAAT
TP10402_Hit	D	1	chr3	3A	CAGCTCCATATGTAATGCTTTCAAACGTGTATGCAAGTGCCAGCAGATGGGAAGAGGCTGAAAA
TP10402_Query	D	1	chr3	3A	CAGCACCATATGTAATGCTTTCAAACGTGTATGCAAGTGCCAGCAGATGGGAAGAGGCTGAAAA
TP13270_Hit	D	1	chr3	3A	CAGCAGAATAATATGCTGTCAAACTTTGTTCCTTATCACTATCACTAGATGCATCACCATTTTC
TP13270_Query	D	1	chr3	3A	CAGCAGAATAATATGCAGTCAAACTTTGTTCCTTATCACTATCACTAGATGCATCACCATTTTC
TP15280_Hit	D+G	1	chr3	3A	CAGCAGGTCTTGCTGTGGCTACCCTCCATGCATGCTATGGAAACGAGTTGAAGCAATTCATTTC
TP15280_Query	D+G	1	chr3	3A	CAGCAGGTCTTGCTGTGGCTACCCTCCATGCATGCTATGGAAACGAATTGAAGCAATTCATTTC
TP16701_Hit	D	1	chr3	3A	CAGCAGTTGGAAGAAGTGGTTGTTATGCCAAATTACCACCGATTATTCCCTGGTGGTGCTAGGA
TP16701_Query	D	1	chr3	3A	CAGCAGTTGGAAGAAGTGGTTGTTATGCCAAATTACCACCGATTATTCCCTGGGGGTGCTAGGA
TP17943_Hit	D+G	1	chr3	3A	CAGCATATCTTAATGCCCTGACCTCTGAGGGTGTTCATGGTTTGTGTTATTACCAACACTTCAA
TP17943_Query	D+G	1	chr3	3A	CAGCATATCTTAATGCCCTGACCTCTGAGGGTGTCCATGGTTTGTGTTATTACCAACACTTCAA
TP20106_Hit	D	1	chr3	3A	CAGCATGCATACTATCTGGATTACAAGGTATGTAACCATCATATATCGTCGCTTATTTTTCGAT
TP20106_Query	D	1	chr3	3A	CAGCATGCATACTATCTGGATTACAAGGTATGTAACCATCATATATCATCGCTTATTTTTCGAT
TP20441_Hit	D	1	chr3	3A	CAGCATGGGGTGACTACGAGTATTTTTTATCCTTACCTATTGAAACTTTGACCATAGAAAGTGT
TP20441_Query	D	1	chr3	3A	CAGCATGGGGTGACTACGAGTATTTTTTATCCTTACCTATTGAAACTTTGACCATACAAAGTGT
TP21384_Hit	D	1	chr3	3A	CAGCATTCCAAGACACAAGGTGGTTCAGATATACATTCACATACTTTGCTCTGTCATTCTGTCC
TP21384_Query	D	1	chr3	3A	CAGCATTCCAAGACACAAGGTGGTTCAGAAATACATTCACATACTTTGCTCTGTCATTCTGTCC
TP22304_Hit	D	1	chr3	3A	CAGCATTTGCTGTTATTTTTGGAGAGTTTTGGCTTGTTGCTCAATCAAGTGTTTCAAACACACT
TP22304_Query	D	1	chr3	3A	CAGCATTTGCTGTTATTTTCGGAGAGTTTTGGCTTGTTGCTCAATCAAGTGTTTCAAACACACT
TP32682_Hit	D	1	chr3	3A	CAGCCTCCCAAGTAAAACGACGCCTTCTCCACCTCTGCACATCCACTCCCCACCCTCACCTATG
TP32682_Query	D	1	chr3	3A	CAGCCTCCCAAGTAAAACAACGCCTTCTCCACCTCTGCACATCCACTCCCCACCCTCACCTATG
TP36972_Hit	D	1	chr3	3A	CAGCGCAAGAGAACTCTTCCAAGTAAACAATCCAAACGAAGGACAGTTAAACAGATGAAACAAT
TP36972_Query	D	1	chr3	3A	CAGCGCAAGAGAACTCTTCCAAGTAAACAATCCAAACGAAGGACAGTTAAAAAGATGAAACAAT
TP38571_Hit	D	1	chr3	3A	CAGCGGAGAGCAGTGATCTGCAAAAAAATATTTTTTAAGTATGTAGAACTTTGTTCTTAATAGAT
TP38571_Query	D	1	chr3	3A	CAGCGGAGAGCAGTGATCTGCAAAAAAATATTTTTTAAGCATGTAGAACTTTGTTCTTAATAGAT
TP43446_Hit	D+G	1	chr3	3A	CAGCTACGGTGTGTTTAGCAAACTCGTCTGTAGGGGTTGATCTTGACCCCTGAGAAAATAGAGC
TP43446_Query	D+G	1	chr3	3A	CAGCTACGGTGCGTTTAGCAAACTCGTCTGTAGGGGTTGATCTTGACCCCTGAGAAAATAGAGC

Name	Filter	Nb hit (Mt4.0)	Mt Chr (Mt4.0)	Ms Chr (Li et al., 2014)	Sequence
TP44603_Hit	D	1	chr3	3A	CAGCTATACTATGCAACATATTTTCCAATTTTCCTTTAACATTTTGTGTGACATGCTTTTGCAA
TP44603_Query	D	1	chr3	3A	CAGCTATACTATGCAACATATTTTCCAATTTTCCTTTAACATTTTGTGTGACATGCTTCTGCAA
TP4557_Hit	D	1	chr3	3A	CAGCAACTATAATGTGGCAGTGGCCTTTATTGCTCAAAGGAGAAAATCTAACTGGAAAACATTT
TP4557_Query	D	1	chr3	3A	CAGCAACTATAATGTGGCAGTGGCCTTCATTGCTCAAAGGAGAAAATCTAACTGGAAAACATTT
TP47241_Hit	D	1	chr3	3A	CAGCTCCATACAAAGCCTGACGCTCATCAGTGATATTGTGTTTCTTCTTCAATCTCTTAGACAC
TP47241_Query	D	1	chr3	3A	CAGCTCCATACAAAGCCTCACGCTCATCAGTGATATTGTGTTTCTTCTTCAATCTCTTAGACAC
TP48853_Hit	D	1	chr3	3A	CAGCTCTCAGTTTTGACTCAGCTTTCTCATTTTTTTCTCGGGATGCTTTAAGTTCTTCATATTC
TP48853_Query	D	1	chr3	3A	CAGCTCTCAGTTTTGACTCAGCTTTCTCATTTTTTTCCCGGGATGCTTTAAGTTCTTCATATTC
TP49921_Hit	D	1	chr3	3A	CAGCTGAACCGAGAGATGTCAAGTGAACGCCCAACCGATCCCGATTTGTAAACCTATGAAAAGG
TP49921_Query	D	1	chr3	3A	CAGCTGAACCGAGAGATGTCAAGTGAACGCCCAACCGATCCCGATTTGTAAACCTATGAAAAGA
TP51245_Hit	D+G	1	chr3	3A	CAGCTGGACCTTCAGCCTGACAACAATCACAAATAAATCATCCAAAACCTAGTTACCCAATTTG
TP51245_Query	D+G	1	chr3	3A	CAGCTGGACCTTCAGCCTGACAACAATCACAAATAAATCATCAAAAACCTAGTTACCCAATTTG
TP53435_Hit	D+G	1	chr3	3A	CAGCTTAATATTTGTTCTATTAATCAAGCTGGTTGTGAGTGGAGCATAGCATATTTAATGTCTT
TP53435_Query	D+G	1	chr3	3A	CAGCTTAATATTTGTTCTATTAATCAAGCTGGTTGTGAGTGGAGCATAGCATATTTAATATCTT
TP59947_Hit	D	1	chr3	3A	CAGCTTTTCACATTAAAAGACTTTGTCGGTTCAGGTAGCCAAACAAAAGAATCTGCCTCTTCAA
TP59947_Query	D	1	chr3	3A	CAGCTTTTCACATTAAAAGACTTTGTCGGTTCAGGTAGCCAAACAAAAAAATCTGCCTCTTCAA
TP6065_Hit	D+G	1	chr3	3A	CAGCAAGGCCAAACATGTTTCAAGCTAGATGATTGCCCACTTTGGTTAATTGGGCATGTTTGTT
TP6065_Query	D+G	1	chr3	3A	CAGCAAGGCCAAACATGTTTCAAGCTAGATGATTGCCCACTTTGGTTAATTGGGCATGCTTGTT
TP6330_Hit	D+G	1	chr3	3A	CAGCAAGTACTTGGCGGTGATTGGGATGAAAGTCTAGCCACATTGAACCAAATTGGTATAGAAG
TP6330_Query	D+G	1	chr3	3A	CAGCAAGTACTTGGCGGTGATTGGGATGAAAGTCTAGCCACATTGAAACAAATTGGTATAGAAG
TP63522_Hit	D	1	chr3	3A	CTGCAAGTACCATAACCCGAGCACTCTCTACACAACAACAAAAAAATAACATAAAGCAACAGTC
TP63522_Query	D	1	chr3	3A	CTGCAAGTACCATAACCCGAGCACTCTCTACACAACAACAAAAAAATAACATAAAGCAACAATC
TP64617_Hit	D	1	chr3	3A	CTGCAATTATTAAACTAACATCAGTGAAAAACAAATGTGACTCCACAACAATAGCACAAATGAG
TP64617_Query	D	1	chr3	3A	CTGCAATTATTAAACTAACATCAGTGAAAAACAAATGTGACCCCACAACAATAGCACAAATGAG
TP67927_Hit	D	1	chr3	3A	CTGCAGAGCCATCGAAAGATGTAGAAGAAGGAGAAATTGATTGATGTTACCCCCCATTAATTGT
TP67927_Query	D	1	chr3	3A	CTGCAGAGCCATCGAAAGATGTAGAAGAAGGAGAAATTGATTGATGTTACCCCCCATTAATTGC
TP70891_Hit	D	1	chr3	3A	CTGCATCAACAAAACAGCTACTTTCCACTTGATCAGAGATTACTTGTGCCGAGGTTTTGTTGCA
TP70891_Query	D	1	chr3	3A	CTGCATCAACAAAACAGCTACTTTCCACTTGATCAGAGATTACTTGTGCCGAGGTTTTGTTACA
TP73557_Hit	D	1	chr3	3A	CTGCCAACACGAGAATTCACTAGCAACATTATCAATCTCAACCTTCGACCTCAGCCGGTATCTG
TP73557_Query	D	1	chr3	3A	CTGCCAACACGAGAATTCACTAGCAACATTATCAATCTCAACCTTCGACCTCAACCGGTATCTG
TP75045_Hit	D	1	chr3	3A	CTGCCATACACAACCTGTTAGGAAGTATTAACCATGACAAGCCTGTAGGAAATTAAACTCTTCA
TP75045_Query	D	1	chr3	3A	CTGCCATACACAACCTGTTAGGAAGTATTAACCATGACAAGCCTGTAGGAAATCAAACTCTTCA
TP76918_Hit	D+G	1	chr3	3A	CTGCCCTTGTAAAGTATGAGTATAGCAGGAATTTTGTATTTATTATTGTGTTCTGGGTCTTGGA
TP76918_Query	D+G	1	chr3	3A	CTGCCCTTGTAAACTATGAGTATAGCAGGAATTTTGTATTTATTATTGTGTTCTGGGTCTTGGA
TP77397_Hit	D	1	chr3	3A	CTGCCGCGGACTCTTAGTCAGAAAACAGTTGATGAGGTTTGGAAGGATATTTCTAAGGATTATG
TP77397_Query	D	1	chr3	3A	CTGCCGCGAACTCTTAGTCAGAAAACAGTTGATGAGGTTTGGAAGGATATTTCTAAGGATTATG
TP77920_Hit	D+G	1	chr3	3A	CTGCCGTCGTAATCCACCTTGTGGGAGCATACCAAGTATATGTTCAACCTGTCTTTGCCTTCGT
TP77920_Query	D+G	1	chr3	3A	CTGCCGTCGTAATCCACCTTGTGGGAGCATACCAAGTATATGTTCAACCTGTCTTTGCATTCGT
TP78356_Hit	D	1	chr3	3A	CTGCCTCACCATTCCTTGATCATACATGCCATTCAACAATAAGGGATCAAGCCCACCACCTAAA
TP78356_Query	D	1	chr3	3A	CTGCCTCACCAGTCCTTGATCATACATGCCATTCAACAATAAGGGATCAAGCCCACCACCTAAA
TP78474_Hit	D	1	chr3	3A	CTGCCTCCACCACTTGTCTAAATGGATATGTCACCGGTTTAGTTGGCATTGGTGGATATGTTAG
TP78474_Query	D	1	chr3	3A	CTGCCTCCACCACTTGTCTAAATGGATATGTCACCGGTTTAGGTGGCATTGGTGGATATGTTAG
TP78761_Hit	D	1	chr3	3A	CTGCCTCTTTCTCGACGAAGGCAAATAGGGGTTGGGCATACACTTGGTATGCTCCAAAAAGGTG
TP78761_Query	D	1	chr3	3A	CTGCCTCTTTCTCGACGAAGGCAAATAGCGGTTGGGCATACACTTGGTATGCTCCAAAAAGGTG
TP80689_Hit	D	1	chr3	3A	CTGCGATTCGAAGTGACAATCCTGTGATACTTTTTGAGCATGTTTTGCTTTATAACCTCAAGGA
TP80689_Query	D	1	chr3	3A	CTGCGATTCGAAGTGACAACCCTGTGATACTTTTTGAGCATGTTTTGCTTTATAACCTCAAGGA
TP8432_Hit	D	1	chr3	3A	CAGCAATTGCGTTTCTTCTCTACAAACGTCACAAACGCATATTAGAAGCACAACAACGCCTAGC
TP8432_Query	D	1	chr3	3A	CAGCAATTGCGTTTCTTCTCTACAAACGTCACAAAAGCATATTAGAAGCACAACAACGCCTAGC
TP85002_Hit	D	1	chr3	3A	CTGCTATCATTATTAGGACAAAATTGAACCATATTGGACCTTTTTTAAATAAGGGTACATAAAT
TP85002_Query	D	1	chr3	3A	CTGCTACCATTATTAGGACAAAATTGAACCATATTGGACCTTTTTTAAATAAGGGTACATAAAT
TP86301_Hit	D	1	chr3	3A	CTGCTATCGTTATTCACCTTTTTGGAGCATACCAAGTGTATGCCCAACCGCTATTTGCCTTCGT
TP86301_Query	D	1	chr3	3A	CTGCTATCGTTATTCACCTTTTTGGAGCATACCAAGTGTATGCCCAACCCCTATTTGCCTTCGT
TP91009_Hit	D+G	1	chr3	3A	CTGCTGGTCATGTCTTACTAGAAACCCTAGATACTTCCCTTGTCACTGGCCACCTTGGGCTTCC
TP91009_Query	D+G	1	chr3	3A	CTGCTGGTCATGTCTTACTAGAAACCCTAGACACTTCCCTTGTCACTGGCCACCTTGGGCTTCC

Name	Filter	Nb hit (Mt4.0)	Mt Chr (Mt4.0)	Ms Chr (Li et al., 2014)	Sequence
TP92015_Hit	D	1	chr3	3A	CTGCTGTGGTGATATGAATGACTAAAGCCGAATTTTCAAAAATTGATTACAGGGTTGTTAGTTT
TP92015_Query	D	1	chr3	3A	CTGCTGTGGTGATATGAATGACTAAAGCCGAATTGTCAAAAATTGATTACAGGGTTGTTAGTTT
TP93921_Hit	D+G	1	chr3	3A	CTGCTTCTTATTCATTCTTGGTAATCTTTCACTAACATTGAGACAAACTTAGGGTAAGGCGAGC
TP93921_Query	D+G	1	chr3	3A	CTGCTTCTTATTCATTCTAGGTAATCTTTCACTAACATTGAGACAAACTTAGGGTAAGGCGAGC
TP94228_Hit	D	1	chr3	3A	CTGCTTGCACCAGTGACTGCTCATTTGCAGAAGCATGTCACACAAAATGTTAAAGGAAAATTGG
TP94228_Query	D	1	chr3	3A	CTGCTTGCACCAGTGACTGCTCATTTGCAAAAGCATGTCACACAAAATGTTAAAGGAAAATTGG
TP993_Hit	D	1	chr3	3A	CAGCAAAATGGGGTCTGTTGGATTGGTTAACTCACGGTGGTTCGACTCCCTTAATCGACATGTT
TP993_Query	D	1	chr3	3A	CAGCAAAATGGGGTCTGCTGGATTGGTTAACTCACGGTGGTTCGACTCCCTTAATCGACATGTT
TP11020_Hit	D+G	1	chr3	3B	CAGCACCTTACTTTACTTTTACTGCTTTTTGTTTCTTATTTCTGAGATTTAGTCGTGGAGACCC
TP11020_Query	D+G	1	chr3	3B	CAGCACCTTACGTTACTTTTACTGCTTTTTGTTTCTTATTTCTGAGATTTAGTCGTGGAGACCC
TP11540_Hit	D	1	chr3	3B	CAGCACGGTCAGAGATGGAATAAAATAATGTGAAGTGATAGATAAGACTTTCCTTGGGGCGCAG
TP11540_Query	D	1	chr3	3B	CAGCACGGTCAGAGATGGAATAAAATAATGTGAAGTGATAGATAAGACTTTCCTTAGGGCGCAG
TP14220_Hit	D+G	1	chr3	3B	CAGCAGATGCTGAAACAGCAAGTCTCTGGGCAGATGTAAGGGGAGCTCTAGGTTCTGCGGGAAC
TP14220_Query	D+G	1	chr3	3B	CAGCAGATGCTGAAACAGCAAGTCTCTGGGCAGATGTAAGGGGAGCTCTAGGTTCCGCGGGAAC
TP17975_Hit	D+G	1	chr3	3B	CAGCATATGATTGTCTGGATGAGGCTTCAGACTCATTAAATCTATTTTGCCTGGGATCATTCAA
TP17975_Query	D+G	1	chr3	3B	CAGCATATGATTGTCTGGATGAGGCTTCAGACTCATTAAATCTATTCTGCCTGGGATCATTCAA
TP21110_Hit	D+G	1	chr3	3B	CAGCATTAGCTGGTGGAATTATGGTGGTGGATGCCTTTGAGTCGTTAAGTGGCCGATGAGTTTG
TP21110_Query	D+G	1	chr3	3B	CAGCATTAGCTGGTGGAATTATGGTGGTGGATGCCGTTGAGTCGTTAAGTGGCCGATGAGTTTG
TP22030_Hit	D	1	chr3	3B	CAGCGTTTAGTTGTTGGTGATTGTGAAAATCTGAAATCTCTATTCTCTGTGAAGGAATCTAGAA
TP22030_Query	D	1	chr3	3B	CAGCATTTAGTTGTTGGTGATTGTGAAAATCTGAAATCTCTATTCTCTGTGAAGGAATCTAGAA
TP24772_Hit	D+G	1	chr3	3B	CAGCCACCATAAACTGGTTACCTCTGGTAGCCAGTTTCTGCCTCCAATCACCATCAGCTTTCTT
TP24772_Query	D+G	1	chr3	3B	CAGCCACCATAAACTGGTTACCTCTGGTACCCAGTTTCTGCCTCCAATCACCATCAGCTTTCTT
TP2569_Hit	D	1	chr3	3B	CAGCAAATTTCATCCATGTGTCAAATGAGGTTTCACGTAGTTTTACACCAGCCTTGACAAACCT
TP2569_Query	D	1	chr3	3B	CAGCAAATTTCATCCATGAGTCAAATGAGGTTTCACGTAGTTTTACACCAGCCTTGACAAACCT
TP26854_Hit	D	1	chr3	3B	CAGCCATGAGCAACTGCCTTGAGGCCTACATCAGTCAAGGCACGATCTGGATTGCTTCCATGAA
TP26854_Query	D	1	chr3	3B	CAGCCATGAGCAACTGCCTTGAGACCTACATCAGTCAAGGCACGATCTGGATTGCTTCCATGAA
TP30488_Hit	D+G	1	chr3	3B	CAGCCGCATTGACCACATTTTGCTGTAATATCAAAATTTACCGCGCAAACGTGACCACAGCTGA
TP30488_Query	D+G	1	chr3	3B	CAGCCGCATTGACCACATTTTGCTGTAATATCAAAATTTACCGCGCAAACCTGACCACAGCTGA
TP31668_Hit	D	1	chr3	3B	CAGCCTAAAGATCAAGTTAAGGATGGTGCTACTGAGGTCCTCCTCAATAAAACTGAAACAAAGA
TP31668_Query	D	1	chr3	3B	CAGCCTAAAGATCAAGTTAAGGATGGTGCTACTGAGGTCCTCCCCAATAAAACTGAAACAAAGA
TP34715_Hit	D+G	1	chr3	3B	CAGCCTTGGTGAACGATACCAGCCCATTGTCTGACAGAAATGCACACCTACGAAGCTGACAGTT
TP34715_Query	D+G	1	chr3	3B	CAGCCTTGGTGAACGATACCAGCCCATTGTCTGACAGAAATGCACACCTACGAAGCTGAAAGTT
TP36593_Hit	D	1	chr3	3B	CAGCGATCTCATAGCATCAATTGCATCTTCATCCACTTCAAAATTGATGAAAGCATAGCTTCTC
TP36593_Query	D	1	chr3	3B	CAGCGATCTCATAGCATCAATGGCATCTTCATCCACTTCAAAATTGATGAAAGCATAGCTTCTC
TP38898_Hit	D	1	chr3	3B	CAGCGGCAGTTGACCTGTTTAAAGAAAAAGTTAAGTATGATTCCATTCAACTATTATCGTTTCA
TP38898_Query	D	1	chr3	3B	CAGCGGCAGTTGACCTGTTTAAAGAAAAAGTTAAGTACGATTCCATTCAACTATTATCGTTTCA
TP40723_Hit	D	1	chr3	3B	CAGCGTCTTGGAGGATGACAGTAACGAGCTTAGAGGCGAAAGAGTTGAGATAGTCAGTGGATTC
TP40723_Query	D	1	chr3	3B	CAGCGTCTTGGAGGACGACAGTAACGAGCTTAGAGGCGAAAGAGTTGAGATAGTCAGTGGATTC
TP52635_Hit	D+G	1	chr3	3B	CAGCTGTGGTCAGGTTTGCGCGGTAAATTTTGATATTACAGCAAAATGTGGTCAATGCGGCTGA
TP52635_Query	D+G	1	chr3	3B	CAGCTGTGGTCACGTTTGCGCGGTAAATTTTGATATTACAGCAAAATGTGGTCAATGCGGCTGA
TP63669_Hit	D+G	1	chr3	3B	CTGCAAGTTCTCTCTCACCCCTGCGTTCTTGTTCACTGGAAGGTCCCACTAGAACTTTCTGATC
TP63669_Query	D+G	1	chr3	3B	CTGCAAGTTCTCTCTCACCCCTGCGTTCTTGCTCACTGGAAGGTCCCACTAGAACTTTCTGATC
TP6409_Hit	D+G	1	chr3	3B	CAGCAAGTCGGAACAAAGTAGTAAGGTCTGGTTGACTGCTAGATGTAGTAGCCGAAAGTCCTCG
TP6409_Query	D+G	1	chr3	3B	CAGCAAGTCGGAACAAAGTAGTAAGGTCTGGTTGACGGCTAGATGTAGTAGCCGAAAGTCCTCG
TP71354_Hit	D	1	chr3	3B	CTGCATCGGGGCGTAGAACCTTGACTGCAACAGGTGTATGGTCTAGAAGGCACTTAAAAACTGG
TP71354_Query	D	1	chr3	3B	CTGCATCGGGGCGTAGAACCTTGACTGCAACAGGTGTATGGTCCAGAAGGCACTTAAAAACTGG
TP71850_Hit	D+G	1	chr3	3B	CTGCGTGCCATGTCAACTCCGTCATTGTATATTACATTTTATTTGTGTGTGTGAGAGAGAGAAG
TP71850_Query	D+G	1	chr3	3B	CTGCATGCCATGTCAACTCCGTCATTGTATATTACATTTTATTTGTGTGTGTGAGAGAGAGAAG
TP73446_Hit	D+G	1	chr3	3B	CTGCCAAAGGATGCCATCTCTTGAGTATTGGACTGAACACTAGCTTTTCATTAATTGCCAGGCT
TP73446_Query	D+G	1	chr3	3B	CTGCCAAAGGATGCCATCTCTTGAGTATTGGACTGAACACTAGCTTTTCATTAACTGCCAGGCT
TP74676_Hit	D	1	chr3	3B	CTGCGAGACGTATTTTCAATTCAGGAGACATAGTTGTCTCACAAACAACTCTCATGATGTACTC
TP74676_Query	D	1	chr3	3B	CTGCCAGACGTATTTTCAATTCAGGAGACATAGTTGTCTCACAAACAACTCTCATGATGTACTC
TP7793_Hit	D+G	1	chr3	3B	CAGCAATGCGAGGACTTTCGGCTACTACATCTAGCCGTCAACCAGACCTTACTACTTTGTTCCG
TP7793_Query	D+G	1	chr3	3B	CAGCAATGCGAGGACTTTCGGCTACTACATCTAGCAGTCAACCAGACCTTACTACTTTGTTCCG

Name	Filter	Nb hit (Mt4.0)	Mt Chr (Mt4.0)	Ms Chr (Li et al., 2014)	Sequence
TP82120_Hit	D+G	1	chr3	3B	CTGCGGCATATCTATCCTTAAGATCATTAAGATTTGTCTCTTCTTCTTGAAGCCCTAGTTGAAC
TP82120_Query	D+G	1	chr3	3B	CTGCGGCATATCTATCCTTAAGACCATTAAGATTTGTCTCTTCTTCTTGAAGCCCTAGTTGAAC
TP8589_Hit	D	1	chr3	3B	CAGCAATTTGAAGATTGTTTACTCAGGAAGTCAAGAATTCAAGACAGCCAGTGCTATGAGGGAT
TP8589_Query	D	1	chr3	3B	CAGCAATTTGAAGATTGTTTACTCAGGAACTCAAGAATTCAAGACAGCCAGTGCTATGAGGGAT
TP87612_Hit	D+G	1	chr3	3B	CTGCTCCAACAAATTCTGATATACGTCGTGTCAATATTTTATGTTTTCATTGACTTGGGTTCAA
TP87612_Query	D+G	1	chr3	3B	CTGCTCCAACAAATTCTGATATACATCGTGTCAATATTTTATGTTTTCATTGACTTGGGTTCAA
TP93396_Hit	D+G	1	chr3	3B	CTGCTTCATTGTATTTCTCATTGGTCTTTCTATATATACCCCCACCTAGAGTCTTCTGTGATGG
TP93396_Query	D+G	1	chr3	3B	CTGCTTCATTGTATTTCTCATTGGTCTTTCTATATACACCCCCACCTAGAGTCTTCTGTGATGG
TP12894_Hit	D+G	1	chr3	3C	CAGCAGAAAGGAGAAGCAAGTCCTTTTTTGGCAGAATTGTTACGGTTAGAAGAACAAGCTAAAC
TP12894_Query	D+G	1	chr3	3C	CAGCAGAAAGGAGAAGCAAGTCCCTTTTTGGCAGAATTGTTACGGTTAGAAGAACAAGCTAAAC
TP1631_Hit	D	1	chr3	3C	CAGCGAAGAGACAGATTTTTTCGACAACAACAATACTGGATGTACAGGGCTTGGTATGTTGCAT
TP1631_Query	D	1	chr3	3C	CAGCAAAGAGACAGATTTTTTCGACAACAACAATACTGGATGTACAGGGCTTGGTATGTTGCAT
TP27344_Hit	D	1	chr3	3C	CAGCCATTCGAAAAACATTAAACTCTTCCTCGTCGTCATCGTCATCAGGGCCAAAAGCATCCAT
TP27344_Query	D	1	chr3	3C	CAGCCATTCGAAAAACATTAAACTCTTCCTCATCGTCATCGTCATCAGGGCCAAAAGCATCCAT
TP28255_Hit	D	1	chr3	3C	CAGCCCATGCTTTGTGTCACTGCCGTTAATCCCTTCCTTGTGTCTAAGGTCCCAGCCTTGCTTC
TP28255_Query	D	1	chr3	3C	CAGCCCATGCTTTGTGTCACTGCCGTTAATCCCTTCCTTGTGTCAAAGGTCCCAGCCTTGCTTC
TP44176_Hit	D	1	chr3	3C	CAGCTAGGCTTGGTTGAGATGCATCAGATACACATTTCACGACATGTTCTTTCAGGTGTGATGG
TP44176_Query	D	1	chr3	3C	CAGCTAGGCTTGGTTGAGATGCATCAGATACACATTTCAAGACATGTTCTTTCAGGTGTGATGG
TP46505_Hit	D	1	chr3	3C	CAGCTCAGGAGCTTGCTGATTCAAAAATTATGAAACCCAAACGTGATACAGGTTCTTTTGTATC
TP46505_Query	D	1	chr3	3C	CAGCTCAGGAGCTTGCTGATTCAAAAATTATGAAACCCAAACGCGATACAGGTTCTTTTGTATC
TP57775_Hit	D	1	chr3	3C	CAGCTTGGGACTTAAAGGGCTGTGTACCCTCTGTGTTTTGGGCACTACTTTACCTACTTCACTT
TP57775_Query	D	1	chr3	3C	CAGCTTGGGACTTAAAGGGCTGTGTACCCTCTGAGTTTTGGGCACTACTTTACCTACTTCACTT
TP60664_Hit	D	1	chr3	3C	CTGCAAAACAGTTTGAAACATATCAAGCTCTTGTGCCTGAAGAGTTTCGACAGAAGGGATTTCT
TP60664_Query	D	1	chr3	3C	CTGCAAAACAGTTTGAAACATATCAAGCTCTTGTGCCTGAAGAGTTTCCACAGAAGGGATTTCT
TP63160_Hit	D+G	1	chr3	3C	CTGCAAGAGTCCTTCTCATCAAACATTCCTCTAATATTTTCCTGTCTTCTCCGTCGCAATCCTC
TP63160_Query	D+G	1	chr3	3C	CTGCAAGAGTCCTTCTCATCAAACATTCCTCTAATATTTTCATGTCTTCTCCGTCGCAATCCTC
TP64697_Hit	D	1	chr3	3C	CTGCAATTCTTTGGGAAGGCTCTCCTTGAATTGAGACAATTCGTCGGAGATTGTTTATCATTGA
TP64697_Query	D	1	chr3	3C	CTGCAATTCTTTGGGAAGGCTCTCCTTGAATTGAGACAATTCGTCGGAGATTGTTTATCATCGA
TP69244_Hit	D	1	chr3	3C	CTGCAGGGATGAACCAAGGCTATACTGCCGCTGAGTTGTTGGCTTATCAGCGTCAGCTTGCCTT
TP69244_Query	D	1	chr3	3C	CTGCAGGGATGAACCAAGGCTATACTGCCGCTGAGTTGTTGGCTTATCAGCGCCAGCTTGCCTT
TP70018_Hit	D+G	1	chr3	3C	CTGCAGTTCCATCACACCTGAAAGAACATGTCTTGAAATGTGTATCTGATGCATCTCAACCAAG
TP70018_Query	D+G	1	chr3	3C	CTGCAGTTCCATCACACCTGAAAGAACATGTCGTGAAATGTGTATCTGATGCATCTCAACCAAG
TP70593_Hit	D+G	1	chr3	3C	CTGCATAGCTTGGGTGTTTGTGTTCTTGAAAGTTCCAGAAACCAAAGGCATGCCACTTGAAGTG
TP70593_Query	D+G	1	chr3	3C	CTGCATAGCTTGGGTGTTTGTGTTCTTGAAAGTTCCAGAAACCAAAGGCATGCCACTGGAAGTG
TP75001_Hit	D+G	1	chr3	3C	CTGCCATAACCATATGGAGAGATAACCCCTCCAGTTGACTGCACATATTGCCCTCCATTTGACT
TP75001_Query	D+G	1	chr3	3C	CTGCCATAACCATATGGAGAGATAACCCCACCAGTTGACTGCACATATTGCCCTCCATTTGACT
TP76899_Hit	D	1	chr3	3C	CTGCCCTTGACAGTAAGATTTCAGGGTCCATCTAACCAATATATACTGTCGACAACAACATTAA
TP76899_Query	D	1	chr3	3C	CTGCCCTTGACAGTAAGATTTCAGGGTCCATCTAACCAATATATACTGTCAACAACAACATTAA
TP78109_Hit	D+G	1	chr3	3C	CTGCCTAATACACAAAAAAGTTGTTTATGCTGAGAGTTGATTGCAATATAGAAAATAGAATATT
TP78109_Query	D+G	1	chr3	3C	CTGCCTAATACACAAAAAAGTTGTTTACGCTGAGAGTTGATTGCAATATAGAAAATAGAATATT
TP79420_Hit	D+G	1	chr3	3C	CTGCCTTGTCTTTTTCTCAAACCCAAATCGCGTCCAACCCGACCCATACAAGCCTATATAAGTC
TP79420_Query	D+G	1	chr3	3C	CTGCCTTGTCTTTTTCTCAAACCCAAATCGCGTCCAACCCGACCCATACAAGACTATATAAGTC
TP83742_Hit	D	1	chr3	3C	CTGCGTTGGATGCACTTTTTCTGCTCAGGCAAGCTTGGTCAGCATGCCCAGCAGAAGTTTCAAG
TP83742_Query	D	1	chr3	3C	CTGCGTTAGATGCACTTTTTCTGCTCAGGCAAGCTTGGTCAGCATGCCCAGCAGAAGTTTCAAG
TP84918_Hit	D+G	1	chr3	3C	CTGCTACATCCACTATTTGGCCATTACAACGACTGTGTGAAAATGCAACAACAGGGTTTCATGT
TP84918_Query	D+G	1	chr3	3C	CTGCTACATCCACTATTTGGCCAGTACAACGACTGTGTGAAAATGCAACAACAGGGTTTCATGT
TP93452_Hit	D	1	chr3	3C	CTGCTTCCATCTATTGGCCATGACATTGTGGCTGAACTTGAAAAATGACTCATTCTCTAGGGAG
TP93452_Query	D	1	chr3	3C	CTGCTTCCATCTATTGGCCATGACATTGTGACTGAACTTGAAAAATGACTCATTCTCTAGGGAG
TP12896_Hit	D	1	chr3	3D	CAGCAGAAAGGGATAAGAAACCATACTTAGCCATGCCTTCAGAAAGAACAACGAAAGTATTAGC
TP12896_Query	D	1	chr3	3D	CAGCAGAAAGGGATAAGAAACCATACTTAGCCATGCCTTCAGAAAGAACAACAAAAGTATTAGC
TP17139_Hit	D	1	chr3	3D	CAGCATCAGAGAACACAGAAGAAACAAGTTGAGAGGATAAGGAATTAAGATGATCCGTGGATTT
TP17139_Query	D	1	chr3	3D	CAGCATAAGAGAACACAGAAGAAACAAGTTGAGAGGATAAGGAATTAAGATGATCCGTGGATTT
TP19306_Hit	D	1	chr3	3D	CAGCATCTATCATAGTAATACATAAATTCGTTCAGATTTCAGAGTCAAACCTTTACCTGTTGAC
TP19306_Query	D	1	chr3	3D	CAGCATCTATCATAATAATACATAAATTCGTTCAGATTTCAGAGTCAAACCTTTACCTGTTGAC

Name	Filter	Nb hit (Mt4.0)	Mt Chr (Mt4.0)	Ms Chr (Li et al., 2014)	Sequence
TP33750_Hit	D	1	chr3	3D	CAGCCTGGTAGGATACTGATTGATTTCCTAGGCAATTCATACGAAATATTTTGAAATTGGATTG
TP33750_Query	D	1	chr3	3D	CAGCCTGGTAGGATACTGATTGATTTCCCAGGCAATTCATACGAAATATTTTGAAATTGGATTG
TP36166_Hit	D+G	1	chr3	3D	CAGCGAGATCCTTTTCTGCAGTTTAGTGATCCAGCTGATGCAGTGAGAGATGCTACTAGAGATG
TP36166_Query	D+G	1	chr3	3D	CAGCGAGATCCTTTTCTGCAGTTTAGTGATCCAGCTGATGCAGTGAGAGATGCTACTAGAGAAG
TP427_Hit	D	1	chr3	3D	CAGCAAAACCATCTTCTCTAGTAGCATCTCTCACTGCATCAGCTGGATCACTAAACTGCAGAAA
TP427_Query	D	1	chr3	3D	CAGCAAAACCATCATCTCTAGTAGCATCTCTCACTGCATCAGCTGGATCACTAAACTGCAGAAA
TP44391_Hit	D	1	chr3	3D	CAGCTAGTGTTGCTGAGAATCCTGTAAACCAGGCGACTCCTACTACAGAAAATCCTATAAGCAA
TP44391_Query	D	1	chr3	3D	CAGCTAGTGTTGCTGAGAATCCTGCAAACCAGGCGACTCCTACTACAGAAAATCCTATAAGCAA
TP44928_Hit	D+G	1	chr3	3D	CAGCTATCCTCGGAGGAGAAACATTAAAGTGACTGTTACTCTATTTTGAGATTTATTTGGAACA
TP44928_Query	D+G	1	chr3	3D	CAGCTATCCTCGGAGGAGAAACATTAAAGTGACTGTTACTATATTTTGAGATTTATTTGGAACA
TP4506_Hit	D	1	chr3	3D	CAGCAACTACAATTTCTTTGTCTCCGTCAAAGCGACCGAGTGAATCCGAGGAAGCTGAAAAAAA
TP4506_Query	D	1	chr3	3D	CAGCAACTACAATTTCTTTGTCTCCGTCAAAGCGACCGAGTGAATCCGAGGAAGCAGAAAAAAA
TP54824_Hit	D	1	chr3	3D	CAGCTTCAGATGAATCACATTGTGGTCTGGTCAGATTTCCTTTATATCTAGACTGGGAGAAGAA
TP54824_Query	D	1	chr3	3D	CAGCTTCAGATGAATCACATTGTGGTCTGGCCAGATTTCCTTTATATCTAGACTGGGAGAAGAA
TP75238_Hit	D	1	chr3	3D	CTGCCATCCACGATCCTGCAACTGTTTCAGTTGATGCGAGTGTTTCATTGGCACCAGCTACCAC
TP75238_Query	D	1	chr3	3D	CTGCCATCCACGATCCTGCAACTGTTTCAGTTGATGCAAGTGTTTCATTGGCACCAGCTACCAC
TP84785_Hit	D	1	chr3	3D	CTGCTACAGTGGAAATACTGTGTTAGAGTTGATATCCGTCGTTACCTTTAGATGTTTCAGTCCA
TP84785_Query	D	1	chr3	3D	CTGCTACAATGGAAATACTGTGTTAGAGTTGATATCCGTCGTTACCTTTAGATGTTTCAGTCCA
TP8849_Hit	D	1	chr3	3D	CAGCACAAATGTCATAAAAAAGCTCCATACATACATTGGTGTTCCAAATAAATCTCAAAATATA
TP8849_Query	D	1	chr3	3D	CAGCACAAATGTCATAAAAAAGCTCCATACATACATTGGTGTTCCAAATAAATCTCAAAATAGA
TP28486_Hit	D	1	chr3	8A	CAGCCCCAGACTGGGTCATACAAGAAACATTCACCTGGTGTTTTGAAACCAATGTTTGTCTTCA
TP28486_Query	D	1	chr3	8A	CAGCCCCAGACCGGGTCATACAAGAAACATTCACCTGGTGTTTTGAAACCAATGTTTGTCTTCA
TP94039_Hit	D+G	1	chr3	8A	CTGCTTGAAGACAAACATTGGTTTCAAAACACCAGGTGAATGTTTCTTGTATGACCCGGTCTGG
TP94039_Query	D+G	1	chr3	8A	CTGCTTGAAGACAAACATTGGTTTCAAAACACCAGGTGAATGTTTCTTGTATGACCCAGTCTGG
TP10048_Hit	D	1	chr4	.	CAGCACCAACAATTATTGAAGTATAGGGAACCACTGAAGGATTGACTATTCTCCTAAAGACTTT
TP10048_Query	D	1	chr4	.	CAGCACCAACAATCATTGAAGTATAGGGAACCACTGAAGGATTGACTATTCTCCTAAAGACTTT
TP10195_Hit	D	1	chr4	.	CAGCACCACAGTTTGCCAACTCGAACACAAAACCACCAATGAATGGCATATCCGTAGATAACCG
TP10195_Query	D	1	chr4	.	CAGCACCACAGTTTGCCAACTCGAACACAAAACCACCAATGAATGGCATATCCGTAAATAACCG
TP10296_Hit	D	1	chr4	.	CAGCACCAGAGCTTTTCGGACTTAGTATAGAGAAAAGGCTTAGTCCAAGGGCTTCGGTTATCCA
TP10296_Query	D	1	chr4	.	CAGCACCAGAGCTTTTCGGACTTAGTATAGAGAAAAGGCTTAGTCCAAGGGCTTCAGTTATCCA
TP10364_Hit	D+G	1	chr4	.	CAGCACCAGTGGTGCTTAACTCAATAATTAATGCTCCGGCTGAGGAAACCATACTTCCTCTACC
TP10364_Query	D+G	1	chr4	.	CAGCACCAGTGGTGCTTAACTCAATAATTAATGCTCCGGCGGAGGAAACCATACTTCCTCTACC
TP10388_Hit	D+G	1	chr4	.	CAGCACCATAATACAGCTATAGAAGATCCTTGTCAAATATACGAAATGGAATCGTCTACAATTG
TP10388_Query	D+G	1	chr4	.	CAGCACCATAATACAGCTATAGAAGATCCTTGTCAAATATACGAAATGGAATCGTCTAAAATTG
TP10475_Hit	D+G	1	chr4	.	CAGCGCCATTGAGTTCAATTTCAATGATAAAAGGGAAACAACAAAGCCAGTCTTTGATTCCATT
TP10475_Query	D+G	1	chr4	.	CAGCACCATTGAGTTCAATTTCAATGATAAAAGGGAAACAACAAAGCCAGTCTTTGATTCCATT
TP10536_Hit	D+G	1	chr4	.	CAGCACCCATACTGAAGCCTCCAATCCCAACTTTCACTATATCACAATAAAAATGATGTTTTAG
TP10536_Query	D+G	1	chr4	.	CAGCACCCATACTGAAGCCTCCAATCCCAACTTTCACTATACCACAATAAAAATGATGTTTTAG
TP10545_Hit	D+G	1	chr4	.	CAGCACCCATTACCTGTGCCCCACCATAAACAAAAGGTCCCCTTCCAGGACCCTGGGGAGCTCC
TP10545_Query	D+G	1	chr4	.	CAGCACCCATTACCTGTGCCCCACCATAAACAAAAGGTCCCCTTCCAGGACCCTGGGGAGCACC
TP10653_Hit	D+G	1	chr4	.	CAGCACCCTTTCCATTGCTTTCAAACTTAGGTTGGAGAAGCAATCTTTGTGTTACTTATCAGTT
TP10653_Query	D+G	1	chr4	.	CAGCACCCTTTCCATTGCTTTCAAACTTACGTTGGAGAAGCAATCTTTGTGTTACTTATCAGTT
TP10844_Hit	D	1	chr4	.	CAGCACCTAATGCCTGTGCACAAGTTTCGCGCACTGGAGCAACAACCTGATCAGATACATAATC
TP10844_Query	D	1	chr4	.	CAGCACCTAATGCCTGTGCACAAGTTTCACGCACTGGAGCAACAACCTGATCAGATACATAATC
TP10908_Hit	D	1	chr4	.	CAGCACCTCCAGCATGGTCGTGGCTTTCGAAAACGACAACATCTTGTTCATATCTTGCCAACGG
TP10908_Query	D	1	chr4	.	CAGCACCTCCAGCATGGTCGTGGCTTTCGAAAACGACAACATCTTGTTCATATCTTGCCAACAG
TP11025_Hit	D	1	chr4	.	CAGCACCTTATGTTCCCATTCGACATATGCGATCATCTTGCCAGGGAGTTGCACCTCCAGTTAC
TP11025_Query	D	1	chr4	.	CAGCACCTTATGTTCCCATTCGACATATGCGATCATCATGCCAGGGAGTTGCACCTCCAGTTAC
TP11035_Hit	D	1	chr4	.	CAGCACCTTCACACTTGGGGATGCCCACTTCTTTTCCCAGTGTCTTTGCAAGAACAGCTGAAAA
TP11035_Query	D	1	chr4	.	CAGCACCTTCACACTTGGGGATGCCCACTTCTTTTCCCAGTGTCTTTGCAAGAACAGCAGAAAA
TP11093_Hit	D+G	1	chr4	.	CAGCACCTTTCTTTGATAGGCTTGTGTTTGACAAGGTACTCCATTTTATAACTGTGATTTCGAC
TP11093_Query	D+G	1	chr4	.	CAGCACCTTTCTTTGATAGGCTTGTGTTTGACAAGGTACTCCACTTTATAACTGTGATTTCGAC
TP11170_Hit	D	1	chr4	.	CAGCACGACTTTCAAAGGAGAATGCATGGGACCAAGGGGTTTTCAACGAGGAACTCTTTTATCC
TP11170_Query	D	1	chr4	.	CAGCACGACTTTCAAAGGAGAATGCATGGGACCAAGGCGTTTTCAACGAGGAACTCTTTTATCC

Name	Filter	Nb hit (Mt4.0)	Mt Chr (Mt4.0)	Ms Chr (Li et al., 2014)	Sequence
TP1123_Hit	D	1	chr4	.	CAGCAAACAACATTTTAATCCCAGTTACCGACTTCAAGGTTTATAATTTAGTGTACTTGAATTA
TP1123_Query	D	1	chr4	.	CAGCAAACAACATTTTAATCCCAGTTACCGACTTCAAGGTTTATAATTTAGTGCACTTGAATTA
TP11254_Hit	D	1	chr4	.	CAGCACGATGCTGACTTGCTGATTGAGATCCCTGTTCACTATTTGCCTGCCCTGTAGTTACACG
TP11254_Query	D	1	chr4	.	CAGCACGATGCTGACTTGCTGATTGAGATCCCTGTTCACTATTTGCCCGCCCTGTAGTTACACG
TP11371_Hit	D	1	chr4	.	CAGCACGCCTTCGGCTACCATCTGCAGAACTTGTTCCTGCTCTCGTTGACAATTCTTACTTCCA
TP11371_Query	D	1	chr4	.	CAGCACGCCTTCGGCTACCATCTGCAGAACTTGTTCCTGCTCTCGTTAACAATTCTTACTTCCA
TP11378_Hit	D+G	1	chr4	.	CAGCACGCGAGTCGCTTATTGCTATTTCTAACTCTTCACCAGATAAGATTCTGAATTCAAATTC
TP11378_Query	D+G	1	chr4	.	CAGCACGCGAGTCGCTTATTGCCATTTCTAACTCTTCACCAGATAAGATTCTGAATTCAAATTC
TP11575_Hit	D	1	chr4	.	CAGCACGGTTGTCATGTATACTCAGTTGACGTGTCAGTTTGGAGAGAAGCGAGGATTGTTTCTG
TP11575_Query	D	1	chr4	.	CAGCACGGTTGTCATGTATACTCAGTTGACGTGTCAGTTTGGAGAGAAGCGAAGATTGTTTCTG
TP11581_Hit	D+G	1	chr4	.	CAGCACGTAACCTACCAATAAGAGCATCAAGACTTCCCCAAGCTTGTTTCAAAGGACAAGGACA
TP11581_Query	D+G	1	chr4	.	CAGCACGTAACCTACCAATAAGAGCATCAAGACTACCCCAAGCTTGTTTCAAAGGACAAGGACA
TP11624_Hit	D+G	1	chr4	.	CAGCACGTGCACACGACGTTGCCGCACTGGCACTTCGTGGTAAATCAGCTTGTCTTAATTTTGC
TP11624_Query	D+G	1	chr4	.	CAGCACGTGCACACGACGTTGCCGCACTGGCACTTCGTGGAAAATCAGCTTGTCTTAATTTTGC
TP11722_Hit	D	1	chr4	.	CAGCACTAACCCAAGTTTGTACATCACTCATTGTTAGTTCAAAATTTGATGTACTAAGACGGCC
TP11722_Query	D	1	chr4	.	CAGCACTAACCCAAGTTTGTACATCACTCATTGTTAGATCAAAATTTGATGTACTAAGACGGCC
TP11724_Hit	D	1	chr4	.	CAGCACTAACCTGATCTGCGGAAGACCCAGGAGTCTTCAACTCAAGGTCTGTGGCTACATCATT
TP11724_Query	D	1	chr4	.	CAGCACTAACCTGATCTGCGGAAGACCCAGGAGTCTTCAACTCAAGGTCCGTGGCTACATCATT
TP11803_Hit	D	1	chr4	.	CAGCACTACGACCAGGAACATTGGCCTTCCCCTTCGCCTGCAATTGCTTGGTGTTTGCTCTAAA
TP11803_Query	D	1	chr4	.	CAGCACTACGACCAAGAACATTGGCCTTCCCCTTCGCCTGCAATTGCTTGGTGTTTGCTCTAAA
TP11815_Hit	D	1	chr4	.	CAGCACTACTGGATCCTTTGATTATGATTTGTTGATTATTGGTGCTGGTGTTGGTGGTCACGGT
TP11815_Query	D	1	chr4	.	CAGCACTACTGGATCCTTTGATTATGATTTGTTGATTATCGGTGCTGGTGTTGGTGGTCACGGT
TP12124_Hit	D+G	1	chr4	.	CAGCACTCTGGGAAGCTATAGAAACAGACTGGCCGATCAAAGCAACCCAGGGAATATCAGAGGT
TP12124_Query	D+G	1	chr4	.	CAGCACTCTGGGAAGCTATAGAAACAGACTGGCCAATCAAAGCAACCCAGGGAATATCAGAGGT
TP12148_Hit	D+G	1	chr4	.	CAGCACTGAAAACACAGGCACTGGCCAAAATAATCATAAACTAGATAACAAAAAAAGCTCTGAA
TP12148_Query	D+G	1	chr4	.	CAGCACTGAAAACACAGGCACTAGCCAAAATAATCATAAACTAGATAACAAAAAAAGCTCTGAA
TP12230_Hit	D	1	chr4	.	CAGCACTGCACACATGCTAGCTATAATATTTACACGAAGTCTTTAGCTCGCATGCATTGGTTTC
TP12230_Query	D	1	chr4	.	CAGCACTGCACACATGCTAGCTATAATATTTACACAAAGTCTTTAGCTCGCATGCATTGGTTTC
TP12238_Hit	D+G	1	chr4	.	CAGCATTGCAGGCAACAGGAGAAGCTCAGCGTTCTGCAAAATTTAACTCAGAAAGGATCGGTGA
TP12238_Query	D+G	1	chr4	.	CAGCACTGCAGGCAACAGGAGAAGCTCAGCGTTCTGCAAAATTTAACTCAGAAAGGATCGGTGA
TP12259_Hit	D	1	chr4	.	CAGCACTGCGGCCAGTACTTGTTATTGTCTAGCATGTATGCTGAGAAAGAAAGATGGGATCGTG
TP12259_Query	D	1	chr4	.	CAGCACTGCGGCCAGTACTTGTTATTGTCTAGCATGTATGCTGAGAAAGAAAGATGGGATCATG
TP12317_Hit	D	1	chr4	.	CAGCACTGGTACTCAATGCGTCCAGGCGTTTTAGATATACTCTGGACTTCAAAGGGGAAGAGGA
TP12317_Query	D	1	chr4	.	CAGCACTGGTACTCAATGCATCCAGGCGTTTTAGATATACTCTGGACTTCAAAGGGGAAGAGGA
TP12330_Hit	D+G	1	chr4	.	CAGCACTGTACTGCAATTTTAGACGATTCCATTTCGTATATTTGACAAGGATCTTCTATAGCTG
TP12330_Query	D+G	1	chr4	.	CAGCACTGTACTGCAATTGTAGACGATTCCATTTCGTATATTTGACAAGGATCTTCTATAGCTG
TP12385_Hit	D	1	chr4	.	CAGCACTGTTGAGAATCCTAGCTCACCTCTGTCATTACTGCAACAGATGTAGGATCATGTCCAC
TP12385_Query	D	1	chr4	.	CAGCACTGTTGAGAATCCTAGCTCACCTCTGTCATTACCGCAACAGATGTAGGATCATGTCCAC
TP12391_Hit	D+G	1	chr4	.	CAGCACTGTTTAAAGATGAGAGCAGAAACTTTTTCTTGGTTGCATTGAAGTTTCCAAATAATTA
TP12391_Query	D+G	1	chr4	.	CAGCACTGTTTAAAGATGAGAGCAGAAACTGTTTCTTGGTTGCATTGAAGTTTCCAAATAATTA
TP12526_Hit	D	1	chr4	.	CAGCACTTCGTTCCGTCGGTAACCTCCGCCGTCCTCCTTTTGGCCAATGGCGACCTCACTATGA
TP12526_Query	D	1	chr4	.	CAGCACTTCGCTCCGTCGGTAACCTCCGCCGTCCTCCTTTTGGCCAATGGCGACCTCACTATGA
TP12532_Hit	D	1	chr4	.	CAGCACTTCTATGGTTTGTTTGGCTTCATTATCTACGGTAAGTGTATTCACCATCCGCAGAACT
TP12532_Query	D	1	chr4	.	CAGCACTTCTATGGTTTGTTTGGCTTCATTATCTACAGTAAGTGTATTCACCATCCGCAGAACT
TP12607_Hit	D+G	1	chr4	.	CAGCACTTGCATGTACATCAGATAACACATCTCATGAACAGATTGTGCAATGTGCTATTACCGG
TP12607_Query	D+G	1	chr4	.	CAGCACTTGCATGTACATCAGATAACACATCTCATGAACAGATTATGCAATGTGCTATTACCGG
TP12699_Hit	D	1	chr4	.	CAGCACTTTCTTTGATTCGATCCTTTCTTTTCCAAAATGGCACCATCAACAATCCGCAAAGCAA
TP12699_Query	D	1	chr4	.	CAGCACTTTCTTTGATTCGATCCTTTCTCTTCCAAAATGGCACCATCAACAATCCGCAAAGCAA
TP12728_Hit	D	1	chr4	.	CAGCACTTTTCAAGCAGTTGCAAGCAGTCTTGCGGGCGGGGCCGTTGTTGGCGGCACCGTTAAG
TP12728_Query	D	1	chr4	.	CAGCACTTTTCAAGCAGTTGCAAGCAGTCTTGCGGGCAGGGCCGTTGTTGGCGGCACCGTTAAG
TP12842_Hit	D	1	chr4	.	CAGCAGAAATAGATTCATCAAATGTCTTCAAAGTCCTCCTAGAGACTCAACCTCAGCGCACTTC
TP12842_Query	D	1	chr4	.	CAGCAGAAACAGATTCATCAAATGTCTTCAAAGTCCTCCTAGAGACTCAACCTCAGCGCACTTC
TP1285_Hit	D	1	chr4	.	CAGCAAACATTGGAAAAAAATGATAGACATACCGTAAATGCATTTTCAACCAAATGAAAAACCG
TP1285_Query	D	1	chr4	.	CAGCAAACATTGAAAAAAAATGATAGACATACCGTAAATGCATTTTCAACCAAATGAAAAACCG

Name	Filter	Nb hit (Mt4.0)	Mt Chr (Mt4.0)	Ms Chr (Li et al., 2014)	Sequence
TP12919_Hit	D	1	chr4	.	CAGCAGAAATACGAGGATCATATGCGGCTTTCAACAAATTATCGCAAAATTGAGGCAGAAAAAA
TP12919_Query	D	1	chr4	.	CAGCAGAAATACGAGGATCATATGCGGCTTTCAACAAATTATCGCAAAATCGAGGCAGAAAAAA
TP13078_Hit	D	1	chr4	.	CAGCAGAAGAACCATAATTACTCATATTATTACTTTGTTGTCTTTGAAGTTGTTGAAGTTCCAC
TP13078_Query	D	1	chr4	.	CAGCAGAAGAACCATAATTACTCAAATTATTACTTTGTTGTCTTTGAAGTTGTTGAAGTTCCAC
TP13082_Hit	D	1	chr4	.	CAGCAGAAGAAGAAGAGATGAAACAAATGGTGGGTTCCCCTACATCTTCCCCACCTTCTTCCTC
TP13082_Query	D	1	chr4	.	CAGCAGAAGAAGAAGAGATGAAACAAATGGTGGGTTCCCCTACATCTTCCCCACCTTCCTCCTC
TP13105_Hit	D	1	chr4	.	CAGCAGAAGACAGGGCTAACTATTTTGGTAATCTTCAAAAGGGAGTTCTACCTGAAACTTTGGG
TP13105_Query	D	1	chr4	.	CAGCAGAAGACAGGGCTAACTATTTTGGTAATCTGCAAAAGGGAGTTCTACCTGAAACTTTGGG
TP13120_Hit	D+G	1	chr4	.	CAGCAGAAGATAACTTCAACTACAGTAATTGCGAAGCTGAAGAAGATGGTTCATGTGCTGATGG
TP13120_Query	D+G	1	chr4	.	CAGCAGAAGATAACTTCAACTACAATAATTGCGAAGCTGAAGAAGATGGTTCATGTGCTGATGG
TP13156_Hit	D	1	chr4	.	CAGCAGAAGCAGGTCCAAAGTAAACTGTTTTGCCTGAAGACAAAAGACAGAGATTATGGAAAAG
TP13156_Query	D	1	chr4	.	CAGCAGAAGCAGGTCCAAAGTAAACTGTTTTGCCTGAAGACAAAAGACAAAGATTATGGAAAAG
TP13229_Hit	D	1	chr4	.	CAGCAGAAGTAAAAGCCAAAAGGGTACAAGAAAATCAGTTAAGTTAAACATGAAAGCATGCAAT
TP13229_Query	D	1	chr4	.	CAGCAGAAGTAAAAGCCAAAAGGGTACAAGAAAATCAGTCAAGTTAAACATGAAAGCATGCAAT
TP13238_Hit	D+G	1	chr4	.	CAGCAGAAGTATTGCTAGCAAGTGGACTTCCATACACAGTACAATCACATGAAATCTATTGCTA
TP13238_Query	D+G	1	chr4	.	CAGCAGAAGTATTGCTAGCAAGTGGACTTACATACACAGTACAATCACATGAAATCTATTGCTA
TP13428_Hit	D	1	chr4	.	CAGCAGACACAGCAACCAAACCAAGAGTACCCTTTGGTGTACGCTCATGTACAACACAATCAGC
TP13428_Query	D	1	chr4	.	CAGCAGACACAGCAACCAAACCAACAGTACCCTTTGGTGTACGCTCATGTACAACACAATCAGC
TP13539_Hit	D+G	1	chr4	.	CAGCAGACGACGACTGAAGGAGTTTATACATCGGTGTACCAGCAGATAATCGCGCTTCAAGTCC
TP13539_Query	D+G	1	chr4	.	CAGCAGACGACGACTGAAGGAGTTTATACATCGGTGTACCAGCAGATAATCGCGCCTCAAGTCC
TP1362_Hit	D	1	chr4	.	CAGCAAACCTGAGATGAATAATTAGCAAATGGTTCGAGTTCTGCTGAAGAAAGTAAGTTCTCTT
TP1362_Query	D	1	chr4	.	CAGCAAACCTGAGATGAATAATTAGCAAATGGTTCGAGTTCCGCTGAAGAAAGTAAGTTCTCTT
TP13756_Hit	D+G	1	chr4	.	CAGCAGAGCATAAGCATATCAAAAGCTGGAATTGTCACATCTCTTCAGGCACGCTGTTCTGTCG
TP13756_Query	D+G	1	chr4	.	CAGCAGAGCATAAGCATATCAAAAGCTGGAATTGTCACATCTCTTCAGGCACGCTGTTCTGTCA
TP1389_Hit	D	1	chr4	.	CAGCAAACGGAAATACTTTTGCTCGAGAACTCGAAGCCTTAGGCCTCGGATCTGCTGAAAAAAA
TP1389_Query	D	1	chr4	.	CAGCAAACGGAAATACTTTTGCTCGAGAACTCGAAGCCTTAGGCCTCGGATCTGCAGAAAAAAA
TP13932_Hit	D	1	chr4	.	CAGCAGAGTCCTGATATTGCTCAGGGTGTACATGGTCATCTTACAAAAAGACCTGAGGAGATTG
TP13932_Query	D	1	chr4	.	CAGCAGAGTCCCGATATTGCTCAGGGTGTACATGGTCATCTTACAAAAAGACCTGAGGAGATTG
TP14000_Hit	D	1	chr4	.	CAGCAGATAACAGCAGTGCCCGGCTTGCCAGAACTGCTAGACTCTTGGGTCACAGCTGAAAAAA
TP14000_Query	D	1	chr4	.	CAGCAGATAACAGCAGTGCCCGGCTTGCCAGAACTGCTAGACTCTTGGGTCACAGCAGAAAAAA
TP14441_Hit	D	1	chr4	.	CAGCAGGAAGATTAAGATCAAGAAAGTCCCTAATCTGTGGAACTTGTTCCTCAAGTACAACTCT
TP14441_Query	D	1	chr4	.	CAGCAGGAAGATTAAGATCAAGAAAGTCCCTAATCTGCGGAACTTGTTCCTCAAGTACAACTCT
TP14638_Hit	D	1	chr4	.	CAGCAGGAGGAGGAACAAGTCAAGATAAAAAATTCCTTGTCTGTCAGTGGGATTCTTAAAGGTC
TP14638_Query	D	1	chr4	.	CAGCAGGAGGAGGAACAAGTCAAGATAAAAAATTCCTTGTCAGTCAGTGGGATTCTTAAAGGTC
TP14678_Hit	D	1	chr4	.	CAGCAGGAGTATGAATCTGAAACGTTCAACACTTCCAACAGACCCAACAAGGTTCTTACTTTTA
TP14678_Query	D	1	chr4	.	CAGCAGGAGTATGAATCTGAAACGTTCAACACTTCCAACAGACCCAACAAGGTTCTTAATTTTA
TP14857_Hit	D	1	chr4	.	CAGCAGGCGAAGGCCGACTGTGGAGATCCTGCTGTGCTTCCTGACGAGGCCCAAACGCTGAAAA
TP14857_Query	D	1	chr4	.	CAGCAGGCGAAGGCCGACTGTGGAGATCCTGCTGTGCTTCCTGACGAGGCCCAAACGCAGAAAA
TP14932_Hit	D+G	1	chr4	.	CAGCAGGGAAAGCAAGATTCCCAATGAGATTAAACAAAAGAGAACCCGAATCTTCTTGAGCATT
TP14932_Query	D+G	1	chr4	.	CAGCAGGGAAAGCAAGATTCCCAATAAGATTAAACAAAAGAGAACCCGAATCTTCTTGAGCATT
TP15335_Hit	D+G	1	chr4	.	CAGCAGGTGGCAGTGGTATTCTCTTGAGAACTAATAATGGTGGCAAGTCATGGGTCCGTGACAA
TP15335_Query	D+G	1	chr4	.	CAGCAGGTGGCAGTGGTATTCTCTTGAGAACTAATAATGGTGGCAAGTCATGGATCCGTGACAA
TP15378_Hit	D+G	1	chr4	.	CAGCAGGTGTTTTCACAAACAACGTAAGCACAGCCAACACTTTGATGCGGGCACTGAGAGCTGG
TP15378_Query	D+G	1	chr4	.	CAGCAGGTGTTTTCACAAACAACGTAAGCACAGCCAACACTTTGATGCGGGCACCGAGAGCTGG
TP15558_Hit	D+G	1	chr4	.	CAGCAGTAACTTCATCATCGTCAATATCCATATATCTTTTTCTTCTGAGCGCCTGCCGTTTCTT
TP15558_Query	D+G	1	chr4	.	CAGCAGTAACTTCATCATCATCAATATCCATATATCTTTTTCTTCTGAGCGCCTGCCGTTTCTT
TP15605_Hit	D	1	chr4	.	CAGCAGTAATTTGGTTGGTCCACAAATTTTGGGTCGGTGACCGAATCCACTAGAACCTCACCTG
TP15605_Query	D	1	chr4	.	CAGCAGTAATTTGGTTGGTCCACAAATTTTGGGTCGGTGACCGAACCCACTAGAACCTCACCTG
TP15647_Hit	D	1	chr4	.	CAGCAGTACCTGGTCCCTTGGGTCGTGCTTCTTCAGAACCATCTGGTAATTCTACTGGGCTTTA
TP15647_Query	D	1	chr4	.	CAGCAGTACCTGGTCCCTTGGGTCGTGCTTCTTCAGAACCATCTGGTAATTCTAATGGGCTTTA
TP15666_Hit	D+G	1	chr4	.	CAGCAGTACTTAGGGTGTGTTTGTCATCAGATTTTGACTTGATGACAATTTTGGTGTTTTGCAG
TP15666_Query	D+G	1	chr4	.	CAGCAGTACTTAGGGTGTGTTTGTCACCAGATTTTGACTTGATGACAATTTTGGTGTTTTGCAG
TP15682_Hit	D	1	chr4	.	CAGCAGTAGACGAGGAAAGGTATCAATGATTAGTTGGGAAATTAATCTATTTGTTTCACGCAAA
TP15682_Query	D	1	chr4	.	CAGCAGTAGACGAGGAAAGGTATCAATGATTAGTTGGGAAATTAATCTATTTGTTTCACACAAA

Name	Filter	Nb hit (Mt4.0)	Mt Chr (Mt4.0)	Ms Chr (Li et al., 2014)	Sequence
TP15709_Hit	D	1	chr4	.	CAGCAGTAGCACTAGTGGAGAGAAAACCATGGACCGGAGAGTTGGCCCGCATTTGGAGACAACG
TP15709_Query	D	1	chr4	.	CAGCAGTAGCACTAGTGGAGAGAAAACCATGGACCGGAGAGTTAGCCCGCATTTGGAGACAACG
TP1578_Hit	D	1	chr4	.	CAGCAAAGAAGCACATTGATCAAAGCACAACTATCTTGGATGTCCAAGGAGTGGTATGTATCAA
TP1578_Query	D	1	chr4	.	CAGCAAAGAAGCACATTGATCAAAGCACAACTATCTTGGATGTCCAAGGAGTGGTATATATCAA
TP15837_Hit	D	1	chr4	.	CAGCAGTATGGTACTTTACAGCATATTTGGGGCCAACAGTGTGGATAATTCTCCTGCAGAAAAT
TP15837_Query	D	1	chr4	.	CAGCAGTATGGTACTTTACAGCATATTTAGGGCCAACAGTGTGGATAATTCTCCTGCAGAAAAT
TP15877_Hit	D	1	chr4	.	CAGCAGTCAAAAATTGCACATGCCTTGTGCATTTTATGCGGTCAAGTTCTGTAGTTGATTCATC
TP15877_Query	D	1	chr4	.	CAGCAGTCAAAAATTGCACATGCCTTGTGCATTTTATGCAGTCAAGTTCTGTAGTTGATTCATC
TP1589_Hit	D	1	chr4	.	CAGCAAAGAATCAAGTTGCTATGAATCCACAGAACACCGTCTTTGATGCTAAGCGTTTAATCGG
TP1589_Query	D	1	chr4	.	CAGCAAAGAATCAAGTTGCTATGAATCCACAGAACACCGTCTTCGATGCTAAGCGTTTAATCGG
TP1598_Hit	D	1	chr4	.	CAGCAAAGAATGTTTGTATGTAATCATTATTTAAATTTAGATGTCTTAAGTTTCTAATCCCCTG
TP1598_Query	D	1	chr4	.	CAGCAAAGAATGTTTGTATGTAATCATTATTTAAATTTAGATGTCTTAAATTTCTAATCCCCTG
TP16001_Hit	D	1	chr4	.	CAGCAGTCGAGAAGCTCTTATCCCCATAACCTAAAGTAGTGACCGTGGAACAAACACAATAGAT
TP16001_Query	D	1	chr4	.	CAGCAGTCGAGAAGCTCTTATCCCCATAACCTAAAGTAGTGACCGTGGAACAAACACAATAGAG
TP16020_Hit	D	1	chr4	.	CAGCAGTTGTGTGCAGAACTGGATTTTTAAGAATATTACTCTTAGGTCCTTTACCTAGTGGTGA
TP16020_Query	D	1	chr4	.	CAGCAGTCGTGTGCAGAACTGGATTTTTAAGAATATTACTCTTAGGTCCTTTACCTAGTGGTGA
TP16025_Hit	D+G	1	chr4	.	CAGCAGTCTAGTACAGGAGTGACTGCTGACATACCTCCTGCCACTCCTATGCCTACTAATCCAC
TP16025_Query	D+G	1	chr4	.	CAGCAGTCTAGTACAGGAGTGACTGCTAACATACCTCCTGCCACTCCTATGCCTACTAATCCAC
TP16054_Hit	D+G	1	chr4	.	CAGCAGTCTTCCCAGAACCGGTCTCAGCAACGCCGATAACATCGTGCTGAAAAAAAAAAAAAAA
TP16054_Query	D+G	1	chr4	.	CAGCAGTCTTCCCAGAACCGGTCTCAGCAACGCCGATAACATCGCGCTGAAAAAAAAAAAAAAA
TP16243_Hit	D+G	1	chr4	.	CAGCAGTGGAGCTAGGACTCTTAAAGGGCCGTCATCCTCTTCTCACAGATATCGATCTTACAAT
TP16243_Query	D+G	1	chr4	.	CAGCAGTGGAGCTAGGACTCTTAAAGGGCCGGCATCCTCTTCTCACAGATATCGATCTTACAAT
TP16308_Hit	D	1	chr4	.	CAGCAGTGGTAGACTTGCATATTTCAATTTAGATTCTTAAGTAGTATTACAACTTAATCATGAA
TP16308_Query	D	1	chr4	.	CAGCAGTGGTAGACTTGCATATTTCAAATTAGATTCTTAAGTAGTATTACAACTTAATCATGAA
TP16313_Hit	D	1	chr4	.	CAGCAGTGGTATGGCAATTTCATTCATGATTTCTTGTAATTATCGTTCTGTTTGATTTGTTGAG
TP16313_Query	D	1	chr4	.	CAGCAGTGGTATGGCAATTTCATTCATGATTTCTTGTAATTATCGTTCTGTTTGATCTGTTGAG
TP16429_Hit	D	1	chr4	.	CAGCAGTTAAATTTTGTTTGCCTTGTTCCTTCTGATGTGACCTCTGAATATTTCTTTTTTATGC
TP16429_Query	D	1	chr4	.	CAGCAGTTAAATTTTGTTTGCCTTGTTCCTTCTGATGTGACCTCTGAATATCTCTTTTTTATGC
TP16457_Hit	D	1	chr4	.	CAGCAGTTACCAGAACATTCTTCTGCTCGCCAGTCCCATCAAGCTTAATTCCTCCAATTTCTGT
TP16457_Query	D	1	chr4	.	CAGCAGTTACCAAAACATTCTTCTGCTCGCCAGTCCCATCAAGCTTAATTCCTCCAATTTCTGT
TP16521_Hit	D	1	chr4	.	CAGCAGTTGTTACTCCTACAACACTTGTTAACCTTACTTCTCTAAAACGTAGCAGTTTTCCCAA
TP16521_Query	D	1	chr4	.	CAGCAGTTATTACTCCTACAACACTTGTTAACCTTACTTCTCTAAAACGTAGCAGTTTTCCCAA
TP16530_Hit	D	1	chr4	.	CAGCAGTTATTTGATTATTTGTCCTAGAGCAGACAAGCTACATTTTTGGCCTGTTTTCTGCATG
TP16530_Query	D	1	chr4	.	CAGCAGTTATTTGAGTATTTGTCCTAGAGCAGACAAGCTACATTTTTGGCCTGTTTTCTGCATG
TP16552_Hit	D+G	1	chr4	.	CAGCAGTTCATTAAACAAGATTCAAACTCAGTTGTGATAACAATAGACCAGTGTATGGAGTAAT
TP16552_Query	D+G	1	chr4	.	CAGCAGTTCATTAAACAAGATTCAAACTCAGTTGTGATAACAATAGAACAGTGTATGGAGTAAT
TP16650_Hit	D	1	chr4	.	CAGCAGTTGGGTTTGATCAAGGAGGGAAATTTGGAGAAGGTGGATTGGGCTGGATTGATGTGGA
TP16650_Query	D	1	chr4	.	CAGCAGTTGAGTTTGATCAAGGAGGGAAATTTGGAGAAGGTGGATTGGGCTGGATTGATGTGGA
TP1679_Hit	D	1	chr4	.	CAGCAAAGATCTTTCTGAGAAACTATTATTAAATAATAATAATGTTGTAGTAATGTGTTGTGGA
TP1679_Query	D	1	chr4	.	CAGCAAAGATCTTTCTGAGAAACTATTATTAAATAATAATAATGTTGTAGTAATGTGATGTGGA
TP16821_Hit	D	1	chr4	.	CAGCAGTTTCGATTTTCAAATTGACTCGCGAACAACTTAACACATTGAAAGCTAAGTCCAAAGA
TP16821_Query	D	1	chr4	.	CAGCAGTTTCGATTTTCAAATTGACTCGCGAACAACTCAACACATTGAAAGCTAAGTCCAAAGA
TP16861_Hit	D	1	chr4	.	CAGCAGTTTGTTCAAATTTCGCTACACTTCGTAGTTTGAACTAAATCGCATATGGCGGAACAAA
TP16861_Query	D	1	chr4	.	CAGCAGTTTGTTCAAATTTCGCTACACTTCGTAGTTTGAACTAAATCGCATATGCCGGAACAAA
TP16949_Hit	D+G	1	chr4	.	CAGCATAAAAGAAAGATCGAAGAACTCATCATGTGATGAAAATCCGGTTTGCTACCCATAGGGG
TP16949_Query	D+G	1	chr4	.	CAGCATAAAAGAAAGATCGAAGAACTCATCATGTGATGAAAATCAGGTTTGCTACCCATAGGGG
TP16950_Hit	D	1	chr4	.	CAGCATAAAAGAAAGATCGAAGAACTCATCATGTGATGAAAATCCGGTTTGCTGAAAAAAAAAA
TP16950_Query	D	1	chr4	.	CAGCATAAAAGAAAGATCGAAGAACTCATCATGTGATGAAAATCAGGTTTGCTGAAAAAAAAAA
TP17227_Hit	D	1	chr4	.	CAGCGTAATCAACCCAAACCAGGCCTAATGGCATAGTACCACATCATTTCTGTGAATACATCCA
TP17227_Query	D	1	chr4	.	CAGCATAATCAACCCAAACCAGGCCTAATGGCATAGTACCACATCATTTCTGTGAATACATCCA
TP17394_Hit	D	1	chr4	.	CAGCATACATGTAACTTTCTATGTTGGTCATTGTCATCAATATTTTTCTCATGTGACTTTTTAG
TP17394_Query	D	1	chr4	.	CAGCATACATGTAACTTTCTACGTTGGTCATTGTCATCAATATTTTTCTCATGTGACTTTTTAG
TP17435_Hit	D	1	chr4	.	CAGCATACCGAGCTAGCTCTCCAGCTCTCCAATCCAATAAAACTGGCAACCATGGATAAGCTGA
TP17435_Query	D	1	chr4	.	CAGCATACCGAGCTAGCTCTCCAGCTCTCCAATCCAATAAAACTGGCAACCATGGATAAGCAGA

Name	Filter	Nb hit (Mt4.0)	Mt Chr (Mt4.0)	Ms Chr (Li et al., 2014)	Sequence
TP17574_Hit	D	1	chr4	.	CAGCATAGACATCCTTGTCCACTCTCTTGCCAATGGACCAGAGGTACCGTAAATAGAGTAATAA
TP17574_Query	D	1	chr4	.	CAGCATAGACATCCTTGTCCACTCTCTTGCCAATGGACCAGAGGTACCATAAATAGAGTAATAA
TP17745_Hit	D	1	chr4	.	CAGCATAGTTTAGAGTCGCCAGCTGTGCTGACCACCTACTTCATATGAAGTATGAACTATAGTC
TP17745_Query	D	1	chr4	.	CAGCATAGTTTAGAGTCGCCAGCTGTGCTGACCACCTACTTCATATCAAGTATGAACTATAGTC
TP17843_Hit	D+G	1	chr4	.	CAGCATATATCATCAGATTTCACAACATTTTATCAACAATCACGAAAGAGAAAGGAGAAAACCT
TP17843_Query	D+G	1	chr4	.	CAGCATATATCATCAGATTTCACAACATTTTATCAACAATCACGAAAGAGAAAGGACAAAACCT
TP17928_Hit	D	1	chr4	.	CAGCATATCTATATTCTTTCTCATATCTTCCATCTCTTGTTGCGCTTGGTCATTATCTTTTGTA
TP17928_Query	D	1	chr4	.	CAGCATATCTATATTCTTTCTCATATCTTCCATCTCTTGTTGCGCTTGATCATTATCTTTTGTA
TP18015_Hit	D	1	chr4	.	CAGCATATGGCGTTTGTCAATCACAAGTTCATTGGGAATCATATTGTATCATCGCATTTGCAAG
TP18015_Query	D	1	chr4	.	CAGCATATGGCGTTTGTCAATCACAAGTTCATTGGGAATCATATTGTACCATCGCATTTGCAAG
TP18263_Hit	D+G	1	chr4	.	CAGCATCAACAATCCCGTTATGAGATATTCAACCCTAGGAGGGTTGGCAATCGTGTGTGTATGG
TP18263_Query	D+G	1	chr4	.	CAGCATCAACAATCCCGTGATGAGATATTCAACCCTAGGAGGGTTGGCAATCGTGTGTGTATGG
TP18285_Hit	D	1	chr4	.	CAGCATCAACATCAGACTTTGAAACCTGGAAGGACAAATGGAGATTCAATCACCACGGGTGCTG
TP18285_Query	D	1	chr4	.	CAGCATCAACATCAGACTTTGAAACCTGGAAGGACAAATGGAGATTCAATCACCACGAGTGCTG
TP18324_Hit	D+G	1	chr4	.	CAGCATCAACTCCTCTTTGCATCATAATGTATCCTTCTTCGCCCCAATCGGTTCCCCAAGAGTT
TP18324_Query	D+G	1	chr4	.	CAGCATCAACTCCTCTTTGCATCATAATGTATCCTTCTTCACCCCAATCGGTTCCCCAAGAGTT
TP18410_Hit	D	1	chr4	.	CAGCATCAATGAGTATGCAATCATCAAGATACAACTCTTTCAAAATCGATCCTAATGAATCAGC
TP18410_Query	D	1	chr4	.	CAGCATCAATGAGTATGCAATCATCAAGATACAACTCTTTCAAAATCGATCCAAATGAATCAGC
TP18431_Hit	D	1	chr4	.	CAGCATCAATTGACCTAGATATAGAGATGGAAGCTGACAAAAATATTCATGGTTTAGACCTATG
TP18431_Query	D	1	chr4	.	CAGCATCAATTGACCTAGATATAAAGATGGAAGCTGACAAAAATATTCATGGTTTAGACCTATG
TP18445_Hit	D	1	chr4	.	CAGCATCACAGCCAGGTAAGAGTTCTAGCTTTCCACTATTGAAAGCTTCTATAAAACCATCACA
TP18445_Query	D	1	chr4	.	CAGCATCACAACCAGGTAAGAGTTCTAGCTTTCCACTATTGAAAGCTTCTATAAAACCATCACA
TP18556_Hit	D	1	chr4	.	CAGCATCACTTTTTGTCTTTGGTTTAATCCCACAAATATTTGCCTATGCTATAAACTTTCCAAT
TP18556_Query	D	1	chr4	.	CAGCATCACTTTTTGTCTTTGGTCTAATCCCACAAATATTTGCCTATGCTATAAACTTTCCAAT
TP18581_Hit	D+G	1	chr4	.	CAGCATCAGATAGTGCAGGGATTGCCGTGTTAACATCACCTATGAATGCACAAGTTGGTTATAA
TP18581_Query	D+G	1	chr4	.	CAGCATCAGATAGTGCAGGGATTGCCGTGTTAACATCACCTATGAATGCACAAGTTGGCTATAA
TP18766_Hit	D	1	chr4	.	CAGCATCATGAAAAGCTTAATTTCGTCTCATGAATTCAACCACGATATAGATGCACCGGAATGA
TP18766_Query	D	1	chr4	.	CAGCATCATGAAAAGCTTAATTTCGTCTCATGAATTCAACCACGATATAGATGCACCAGAATGA
TP18792_Hit	D	1	chr4	.	CAGCATCATGTTCAACTTCTTCTCCATATCCATCCCTAGCAATCTCACCAACAGAACCAAGTAC
TP18792_Query	D	1	chr4	.	CAGCATCATGTTCAACTTCTTCACCATATCCATCCCTAGCAATCTCACCAACAGAACCAAGTAC
TP18868_Hit	D	1	chr4	.	CAGCATCCAACACAGCCCTTCTCTGTAGATCAGTAAAATAACACGGAATCCCAATACAACAATC
TP18868_Query	D	1	chr4	.	CAGCATCCAACACAGCCCTTCTCTGCAGATCAGTAAAATAACACGGAATCCCAATACAACAATC
TP18871_Hit	D	1	chr4	.	CAGCATCCAACCTTTTTAACAGCTGAGACATCATCTCTACTGCCAACGTTGATTGTCTGCCCTT
TP18871_Query	D	1	chr4	.	CAGCATCCAACCTTTTTAACAGCTGAGACATCATCTCTACTGCCAACATTGATTGTCTGCCCTT
TP18872_Hit	D	1	chr4	.	CAGCATCCAACGACGGCCCTCGTGGAGCTCTAGGAGGTTTAGCTGAAAAAAAAAAAAAAAAAAA
TP18872_Query	D	1	chr4	.	CAGCATCCAACGACGGCCCTCGTGGAGCTCTAGGAGGTTTAGCAGAAAAAAAAAAAAAAAAAAA
TP19001_Hit	D	1	chr4	.	CAGCATCTGGATGAATCTTAGCAACAATAACCTATGCAACCCAATACCCTACATAAATGTATCA
TP19001_Query	D	1	chr4	.	CAGCATCCGGATGAATCTTAGCAACAATAACCTATGCAACCCAATACCCTACATAAATGTATCA
TP19027_Hit	D	1	chr4	.	CAGCATCCTCACGAACTATCACTTTCAAGTTTAATCCACACAGATAATACAACTTTAAAAGGTA
TP19027_Query	D	1	chr4	.	CAGCATCCTCACGAACTATCACTTTCAAGTTTAATCCAAACAGATAATACAACTTTAAAAGGTA
TP19344_Hit	D	1	chr4	.	CAGCATCTCCAACCAATTTTACACTACCATCTCCAGACTCACCACTAGAACCACTACCACCACC
TP19344_Query	D	1	chr4	.	CAGCATCTCCAACCAATTTTACACTACCATCTCCAGACTCACCACCAGAACCACTACCACCACC
TP19508_Hit	D	1	chr4	.	CAGCATCTTAGTGAAATTGGAAAAGGTAGTTTTTTAGCTCTTGCTTCTTTTTTAGGTGAAACGG
TP19508_Query	D	1	chr4	.	CAGCATCTTAGTGAAATTGGAAAAGGTAGTTTTTTAGCTCTTGCTTCTTTTCTAGGTGAAACGG
TP19512_Hit	D	1	chr4	.	CAGCATCTTATGTTCCACCACCTCCCTCAGAAGAGCAAAAGAAGAAAATTGCTAAGTTGTAAGA
TP19512_Query	D	1	chr4	.	CAGCATCTTATGTCCCACCACCTCCCTCAGAAGAGCAAAAGAAGAAAATTGCTAAGTTGTAAGA
TP19630_Hit	D+G	1	chr4	.	CAGCATCTTTTTATGTTAAAATTAAAACCATAGCAGTTTTATCGAAATAATAATAAATATTTGA
TP19630_Query	D+G	1	chr4	.	CAGCATCTTTTTATGTTAAAATAAAAACCATAGCAGTTTTATCGAAATAATAATAAATATTTGA
TP19631_Hit	D+G	1	chr4	.	CAGCTTCTTTTTATGTTAAAATTAAAACCATAGCAGTTTTATCGAAATAATAAATATTTGATAC
TP19631_Query	D+G	1	chr4	.	CAGCATCTTTTTATGTTAAAATTAAAACCATAGCAGTTTTATCGAAATAATAAATATTTGATAC
TP19663_Hit	D	1	chr4	.	CAGCATGAAAAGGAAGTAAGTCAAGTAACGGTTAACGGTGTACTCATAAATCATAATACTTATC
TP19663_Query	D	1	chr4	.	CAGCATGAAAAGGAAGTAAGTCAAGTAACGGTTAACGGTGTAATCATAAATCATAATACTTATC
TP19716_Hit	D+G	1	chr4	.	CAGCATGAAGAGCTCTTAGTTCCACTGCTTTGGCTATGGCAACCTTAATTTCCTGTCTTGTAGT
TP19716_Query	D+G	1	chr4	.	CAGCATGAAGAGCTCTTAGTTCCACAGCTTTGGCTATGGCAACCTTAATTTCCTGTCTTGTAGT

Name	Filter	Nb hit (Mt4.0)	Mt Chr (Mt4.0)	Ms Chr (Li et al., 2014)	Sequence
TP19800_Hit	D	1	chr4	.	CAGCATGACCTTATCCCTGATATCTTAATTCTATAAATTTGCAACTAGAAAATGTCTAGACTCT
TP19800_Query	D	1	chr4	.	CAGCATGACCTTATCCCTGATATCTTAATTCTATAAATTTGCAACTAGAAAATGCCTAGACTCT
TP19941_Hit	D	1	chr4	.	CAGCATGATCCCATATTTTTTCACGGTATCCTTTGTTGGGGAAACGCAAATATATAGCCCCGTC
TP19941_Query	D	1	chr4	.	CAGCATGATCCCATATTTTTTCACGGTATCCTTTGTTGGGGAAACGCAAATATATAGCCCCATC
TP19955_Hit	D	1	chr4	.	CAGCATGATGAGGTATTATCTCTGCCTCGATTTTGCGATAATTTGTTGAAAGCCGCATATGATC
TP19955_Query	D	1	chr4	.	CAGCATGATGAGGTATTATCTCTGCCTCAATTTTGCGATAATTTGTTGAAAGCCGCATATGATC
TP20013_Hit	D	1	chr4	.	CAGCATGCGAAATGATATTAATATTAATTACCGAAATCTGGTTCAAGTCGAAGAATGGAGTTAC
TP20013_Query	D	1	chr4	.	CAGCATGCAAAATGATATTAATATTAATTACCGAAATCTGGTTCAAGTCGAAGAATGGAGTTAC
TP2002_Hit	D+G	1	chr4	.	CAGCAAAGTTTTGGCTCTGTCTCCGGTGGGAGACCTGAATAGGTCACCTACGGTAGGAGACATG
TP2002_Query	D+G	1	chr4	.	CAGCAAAGTTTTGGCCCTGTCTCCGGTGGGAGACCTGAATAGGTCACCTACGGTAGGAGACATG
TP20028_Hit	D+G	1	chr4	.	CAGCATGCAAATTCCAAAGAAGAAAGTGCACACCTGAGTGCCTTTTAGCTCCTTATTTCCCTGC
TP20028_Query	D+G	1	chr4	.	CAGCATGCAAATTCCAAAGAAGAAAGTGCACACCAGAGTGCCTTTTAGCTCCTTATTTCCCTGC
TP20097_Hit	D	1	chr4	.	CAGCATGCAGTGTATGTGAATTTGATTCAACAAAAAATGAATATTGAGAGATGGTTTATGAAAT
TP20097_Query	D	1	chr4	.	CAGCATGCAGTGTATGTGAATTTGATTCAACAAAAAATGAATATTGAAAGATGGTTTATGAAAT
TP2019_Hit	D	1	chr4	.	CAGCAAATAAAGTTATTCCACCTAGAAAGTTAACTTTTAGGAGAGGAAAGGTTATTGAAATTCA
TP2019_Query	D	1	chr4	.	CAGCAAATAAAGTTATTCCACCTAGAAAGTTAACTTTTAGGAGAGGAAAGGTTATTGAAATCCA
TP20257_Hit	D	1	chr4	.	CAGCATGCTGTTATGCTGGTGTTTCCATGCTTCTGTTGTGATTTTTTAGTTTCTATTAAGTGAT
TP20257_Query	D	1	chr4	.	CAGCATGCTGTTATGCTGGTGTTTCCATGCTTCTGTTGTGACTTTTTAGTTTCTATTAAGTGAT
TP20477_Hit	D	1	chr4	.	CAGCATGGTCTCCATTGTTGTAGGTGCCGTTGTAGTTGTAATTCTGCACCTGTTCAGGAACATT
TP20477_Query	D	1	chr4	.	CAGCATGGTCTCCATTGTTGTAGGTGCCATTGTAGTTGTAATTCTGCACCTGTTCAGGAACATT
TP20522_Hit	D	1	chr4	.	CAGCATGGTTGGGTTATGGAAAAGGTGATAACTAATCTTGCTCTGCGTGTATTATCATTGTATG
TP20522_Query	D	1	chr4	.	CAGCATGGTTGGGTTATGGAAAAGGTGATAACTAATCTTGCTCTGCGTGTATTATCATTGTATA
TP20652_Hit	D	1	chr4	.	CAGCATGTCGAAAACGCAAACTTCAGCTTTCAAATAGAACTTTATATGATCATGTTTCTACATT
TP20652_Query	D	1	chr4	.	CAGCATGTCGAAAACGCAAACTTCAACTTTCAAATAGAACTTTATATGATCATGTTTCTACATT
TP20745_Hit	D	1	chr4	.	CAGCATGTGGTGGGAATGCACAATGTTGTCAAGTGTGTTTCTATTTTCCTTTAGAAGACTGTTT
TP20745_Query	D	1	chr4	.	CAGCATGTGGTGGAAATGCACAATGTTGTCAAGTGTGTTTCTATTTTCCTTTAGAAGACTGTTT
TP20751_Hit	D	1	chr4	.	CAGCATGTGTAACAGTGTAAGTTACCTCTTCATAATCTGTCTTTATGTTGATGTCATTTTTGCA
TP20751_Query	D	1	chr4	.	CAGCATGTGTAACAGTGTAAGTTACCTCTTCATAATCTGTCTTTATGTTAATGTCATTTTTGCA
TP20900_Hit	D+G	1	chr4	.	CAGCATTAAACACCTTTGTTAGGCCTTTTGGTGAAGTAACATAATCCTCGATAATGATAGTAAC
TP20900_Query	D+G	1	chr4	.	CAGCATTAAACAACTTTGTTAGGCCTTTTGGTGAAGTAACATAATCCTCGATAATGATAGTAAC
TP20908_Hit	D+G	1	chr4	.	CAGCATTAAAGCGATATAGCAAAACATATTTTGATGACTCTGCTATGAGAACTTCGACACTGTC
TP20908_Query	D+G	1	chr4	.	CAGCATTAAAGCGATATAGCAAAACATATTTTGATAACTCTGCTATGAGAACTTCGACACTGTC
TP2099_Hit	D	1	chr4	.	CAGCAAATAGTGTTTTTAGAGGACTTTCAAATTGCAACCTAAACAAAGGTGAGCAAGGAGAAAA
TP2099_Query	D	1	chr4	.	CAGCAAATAGTGTTTTTAGAGGACTTTCAAATTGCAACCTAAACAAAGGTGAGCAAAGAGAAAA
TP2103_Hit	D	1	chr4	.	CAGCAAATATACCTTCATAATCAGAATCAGATCTCACAGACGCGACATGATCATCTTCATCTGT
TP2103_Query	D	1	chr4	.	CAGCAAATATACCTTCATAATCAGAATCAGATTTCACAGACGCGACATGATCATCTTCATCTGT
TP21127_Hit	D	1	chr4	.	CAGCATTAGGATGAATTGGTCCGGAATGTGAACCGGCTCCTCCAAAAGATGTTGTTCGTGATGG
TP21127_Query	D	1	chr4	.	CAGCATTAGGATGAATTGGTCCGGAATGTGAACCGGCTCCTCCAAAAGATGGTGTTCGTGATGG
TP21148_Hit	D	1	chr4	.	CAGCATTAGTGGATGACTTAGGAGAAACTGATACCAATGTACTGAATGAGATCATGCGAGAAGT
TP21148_Query	D	1	chr4	.	CAGCATTAGTGGATGACTTAGGAGAAACTGATACCAATATACTGAATGAGATCATGCGAGAAGT
TP21397_Hit	D	1	chr4	.	CAGCATTCCATGTGAGATATATGGCTGTCTTGCAACTTTATAGCACAGTTGGGGAGTTCTTATT
TP21397_Query	D	1	chr4	.	CAGCATTCCATGTGAGATATATGACTGTCTTGCAACTTTATAGCACAGTTGGGGAGTTCTTATT
TP21457_Hit	D+G	1	chr4	.	CAGCATTCGGGGAATCTTGTTTCTTTTGAACAACATTTGATGTGTTGGAAACATTGCTGGTGGA
TP21457_Query	D+G	1	chr4	.	CAGCATTCGGGGAATCTTGTTTCTTTTGAACAACATTTGATGAGTTGGAAACATTGCTGGTGGA
TP21645_Hit	D	1	chr4	.	CAGCATTGAGCCAGGAACTTGGACTCCCGAGAAAAGGAAGACATATAACTTGCTTGATGCTGTG
TP21645_Query	D	1	chr4	.	CAGCATTGAGCCAGGAACTTGGACACCCGAGAAAAGGAAGACATATAACTTGCTTGATGCTGTG
TP21795_Hit	D	1	chr4	.	CAGCATTGGCAATTCGAAGTGGGAATGGCTTATTACTTAAAGGAGGAAAGGAAGCCCGAAGATC
TP21795_Query	D	1	chr4	.	CAGCATTGGCAATTCGAAGTCGGAATGGCTTATTACTTAAAGGAGGAAAGGAAGCCCGAAGATC
TP21819_Hit	D+G	1	chr4	.	CAGCATTGGGAAATCAGCAATCATTTCACCCAGTTGCCGAAGACGTATGCCGAGAGACAAATAA
TP21819_Query	D+G	1	chr4	.	CAGCATTGGGAAATCAGCAATCATTTCACCCAGTTGCCGAAGACGTACGCCGAGAGACAAATAA
TP21820_Hit	D	1	chr4	.	CAGCATTGGGAAATCAGCAATCATTTCACCCAGTTGCCGAAGTCGTATGCCGTCTTCTGCTTGA
TP21820_Query	D	1	chr4	.	CAGCATTGGGAAATCAGCAATCATTTCACCCAGTTGCCGAAGACGTATGCCGTCTTCTGCTTGA
TP21836_Hit	D+G	1	chr4	.	CAGCATTGGTCATCTGACGGCAAAAGTATAGGACGTAGTGTAGCTTCCGGCATCCTTCAGAGAC
TP21836_Query	D+G	1	chr4	.	CAGCATTGGTCATCTGACGGCAAAAGTATAGGACATAGTGTAGCTTCCGGCATCCTTCAGAGAC

Name	Filter	Nb hit (Mt4.0)	Mt Chr (Mt4.0)	Ms Chr (Li et al., 2014)	Sequence
TP21876_Hit	D	1	chr4	.	CAGCATTGTCAATTGAGGCACTATCTCCACCTTTAGACGGCCATCCACTCTCGGACACAACTAT
TP21876_Query	D	1	chr4	.	CAGCATTGTCAATTGAGGCACTATCCCCACCTTTAGACGGCCATCCACTCTCGGACACAACTAT
TP21877_Hit	D	1	chr4	.	CAGCATTGTCGGGCTCCAATTGTTCCACAAGTGGGTAAAGATTCTCACCCAGCATCTGCAAATA
TP21877_Query	D	1	chr4	.	CAGCATTGTCAGGCTCCAATTGTTCCACAAGTGGGTAAAGATTCTCACCCAGCATCTGCAAATA
TP21923_Hit	D	1	chr4	.	CAGCATTGTTGGGTCAGGGAGGTTCCAAAAGATATGGAATAACATGGAGATGGTTAAGATTTTT
TP21923_Query	D	1	chr4	.	CAGCATTGTTAGGTCAGGGAGGTTCCAAAAGATATGGAATAACATGGAGATGGTTAAGATTTTT
TP2193_Hit	D	1	chr4	.	CAGCAAATCCAAAGAAACTAATCAACAAACCAAACACACAAGACAACGAAACCGCCAAAAACGC
TP2193_Query	D	1	chr4	.	CAGCAAATCCAAAGAAACTAATCAACAAACCAAACACACAAGACAACGAAACAGCCAAAAACGC
TP2200_Hit	D	1	chr4	.	CAGCAAATCCAGCATCCTATCATTCTTCTTTTCCATGGCCATAACCAAAGCTTTCTTCACTACC
TP2200_Query	D	1	chr4	.	CAGCAAATCCAGCATCCTATCATTCTTCTTCTCCATGGCCATAACCAAAGCTTTCTTCACTACC
TP22074_Hit	D	1	chr4	.	CAGCATTTCAAAGATTACCTTATTGTGTAGAGACCATGAGACATATCGTGTGGTGCTTACCAAG
TP22074_Query	D	1	chr4	.	CAGCATTTCAAAGATTACCTTATTGTGTAGAGACCACGAGACATATCGTGTGGTGCTTACCAAG
TP22108_Hit	D	1	chr4	.	CAGCATTTCATGTGAGATATATGGCTGTCTTACAACTTTATAGTACAGTTGGGGAGTTTTTATT
TP22108_Query	D	1	chr4	.	CAGCATTTCATGTGAGATATATGGCTGTCTTACAACTTTATAGTACAGTTGGGGAGTTCTTATT
TP22191_Hit	D+G	1	chr4	.	CAGCATTTGAAAGAAGGTTTCAACAGGTTTATGTGGCTGAACCTAGTGTTCCGGATACAATTAG
TP22191_Query	D+G	1	chr4	.	CAGCATTTGAAAGAAGGTTTCAACAGGTTTATGTGGCTGAACCTAGTGTTCCAGATACAATTAG
TP2227_Hit	D	1	chr4	.	CAGCAAATCCTTGGAGTCCCTTTCTAAATCTTCCTTCTCTTTAATAAATCAGAACCTCTCAAGT
TP2227_Query	D	1	chr4	.	CAGCAAATCCTTGAAGTCCCTTTCTAAATCTTCCTTCTCTTTAATAAATCAGAACCTCTCAAGT
TP2228_Hit	D	1	chr4	.	CAGCGAATCCTTTGCATTGTTTTCTCTTTTCATATTTTAAAAGTTATCATGTCATGATTAAAGT
TP2228_Query	D	1	chr4	.	CAGCAAATCCTTTGCATTGTTTTCTCTTTTCATATTTTAAAAGTTATCATGTCATGATTAAAGT
TP22493_Hit	D	1	chr4	.	CAGCATTTTGAGGCACTGGTGGAAGAGCACTTCGAAAAGCGCTCCAAACATATACTTTTGGCTT
TP22493_Query	D	1	chr4	.	CAGCATTTTGAGGCACTGGTGGAAGAGCACTTTGAAAAGCGCTCCAAACATATACTTTTGGCTT
TP22529_Hit	D	1	chr4	.	CAGCATTTTTATAATGAACCAAGAAAGAAAGAAAAAACTAGTCATCAGTACCCTTGTAGAAGTT
TP22529_Query	D	1	chr4	.	CAGCATTTTTATAATGAACCAAGAAAGAAAGAAAAAACTAGTCATCAGTACCCATGTAGAAGTT
TP22608_Hit	D	1	chr4	.	CAGCCAAAAAAAGCTTTCCCATTGTTTCTATGTGTGACAGCGCTTCACTGGTTACCATCTTCTC
TP22608_Query	D	1	chr4	.	CAGCCAAAAAAAGCTTTCCCATTGTTTCTATGTGCGACAGCGCTTCACTGGTTACCATCTTCTC
TP22851_Hit	D+G	1	chr4	.	CAGCCAAACAGTATTTCTGAGAGCTTCATTGTGCCGGACTCATCACCTCTGTCAGACTTTGTCA
TP22851_Query	D+G	1	chr4	.	CAGCCAAACAATATTTCTGAGAGCTTCATTGTGCCGGACTCATCACCTCTGTCAGACTTTGTCA
TP22951_Hit	D	1	chr4	.	CAGCCAAAGAAAAACATTTCCACAATTCTCGTATTAAAATTGGTTTGGAGATCTTGACCAAAAA
TP22951_Query	D	1	chr4	.	CAGCCAAAGAAAAACATTTCCACAATTCTCGAATTAAAATTGGTTTGGAGATCTTGACCAAAAA
TP22954_Hit	D+G	1	chr4	.	CAGCCAAAGAAACCTTTTTTCACAGTCAACTTGGAGGGATGTGCAGTTTTCAGAATGCGATGAT
TP22954_Query	D+G	1	chr4	.	CAGCCAAAGAAACCTTTTTTCACAGTCAACTCGGAGGGATGTGCAGTTTTCAGAATGCGATGAT
TP22974_Hit	D	1	chr4	.	CAGCCAAAGAGGCCCAATTACGTGAGAACAACAGTCAGAAAGTCCACCCTCAACCAATGGAGGA
TP22974_Query	D	1	chr4	.	CAGCCAAAGAGGCCCAATTACGTGAGAACAACAGTCAGAAAGTCCACCCTCAACCAATGGAAGA
TP22995_Hit	D	1	chr4	.	CAGCCAAAGCAATCTGAATCTGGGATGCAAGATGGATCAGAAGGTATTCATCGAGTTGATGTGG
TP22995_Query	D	1	chr4	.	CAGCCAAAGCAATCTGAATCTGGGATGCAAGATGGATCAGAAGGTATTCATCGAGTTGACGTGG
TP22998_Hit	D	1	chr4	.	CAGCCGAAGCAGTGTATCTTTGAGCATATTTACTTTGCTTTGCCTAATTCTGTTGTTTTTGGTA
TP22998_Query	D	1	chr4	.	CAGCCAAAGCAGTGTATCTTTGAGCATATTTACTTTGCTTTGCCTAATTCTGTTGTTTTTGGTA
TP23130_Hit	D	1	chr4	.	CAGCCAAATCTCGACCATTTAGTCTAGGTACAATCTACATCCAATGGTTGAGATTTGCATGCAC
TP23130_Query	D	1	chr4	.	CAGCCAAATCTCGACCATTTAGTCAAGGTACAATCTACATCCAATGGTTGAGATTTGCATGCAC
TP23181_Hit	D	1	chr4	.	CAGCCAAATTCAGTTTTTGGAGATGTTGGAGACAGAAGAGACTACTTGAATTGTTGAATTCACC
TP23181_Query	D	1	chr4	.	CAGCCAAATTCAGTTTTTGGAGATGTTGGAGACAGAAAAGACTACTTGAATTGTTGAATTCACC
TP23200_Hit	D	1	chr4	.	CAGCCAAATTGTTTGGATCATCAGAGACATGGTTTCTTGTCGGAGGTACTACTTGTGGGATCCA
TP23200_Query	D	1	chr4	.	CAGCCAAATTGTTTGGATCATCAAAGACATGGTTTCTTGTCGGAGGTACTACTTGTGGGATCCA
TP23208_Hit	D	1	chr4	.	CAGCCAAATTTGCGTTCAATGACACAAGAATTGTAAGAATAGAAAAGTAAGACATATAGCATGT
TP23208_Query	D	1	chr4	.	CAGCCAAATTTGCGTTCAATGACACAAGAACTGTAAGAATAGAAAAGTAAGACATATAGCATGT
TP23230_Hit	D	1	chr4	.	CAGCCAACAAATAAAAAACAGACATGTGTCATCTATTTCACTCGCATCTTTTGGCAGTTGTCTG
TP23230_Query	D	1	chr4	.	CAGCCAACAAATAAAAAACAGACACGTGTCATCTATTTCACTCGCATCTTTTGGCAGTTGTCTG
TP23240_Hit	D	1	chr4	.	CAGCCAACAACTAGGTGAAACTGCCACCATGATCTGATCTTTCACCTACAAAGGAGGTCAGTTA
TP23240_Query	D	1	chr4	.	CAGCCAACAACTAGGTGAAACTGCCACCATGATCTGATCTTTCACCTACAAAGGAGGCCAGTTA
TP23252_Hit	D+G	1	chr4	.	CAGCCAACAAGGTATAAAGCAAAGTAGTCAATTGATAGTTGGTGAACACTAACTTCTTCATTTG
TP23252_Query	D+G	1	chr4	.	CAGCCAACAAGGTATAAAGCAAAGCAGTCAATTGATAGTTGGTGAACACTAACTTCTTCATTTG
TP23308_Hit	D	1	chr4	.	CAGCCAACACTTTGATGCGGGCACTGAGAGCTGGAACAGTGTGGATTAATTGCTTTGATGTGTT
TP23308_Query	D	1	chr4	.	CAGCCAACACTTTGATGCGAGCACTGAGAGCTGGAACAGTGTGGATTAATTGCTTTGATGTGTT

Name	Filter	Nb hit (Mt4.0)	Mt Chr (Mt4.0)	Ms Chr (Li et al., 2014)	Sequence
TP2353_Hit	D+G	1	chr4	.	CAGCAAATGCTCTGAAGAAGCAAATGTTGGCTGAAGCACAGATAGATAAAATTCGCCTGAAAGA
TP2353_Query	D+G	1	chr4	.	CAGCAAATGCTCTGAAGAAGCAAATGTTGGCAGAAGCACAGATAGATAAAATTCGCCTGAAAGA
TP23703_Hit	D	1	chr4	.	CAGCCAAGGCAGGTCCTGCACCTGAACCACGCTGGTACTTTTTTAAGGGATTTTGAACCAACAC
TP23703_Query	D	1	chr4	.	CAGCCAAGGCAGGTCCTGCACCTGAACCACGCTGGTACTTTTTTAAGGGATTCTGAACCAACAC
TP23727_Hit	D	1	chr4	.	CAGCCAAGGTAGACTTGTCTATTTTATTTGTTCATTTTTCCGAAGTCCATATGTGATAAAATGT
TP23727_Query	D	1	chr4	.	CAGCCAAGGTAGACTTGTCTATTTTATTTGTTCATTTTTCCAAAGTCCATATGTGATAAAATGT
TP23778_Hit	D	1	chr4	.	CAGCCAGGTGCACTAAAGCTCCCGCATACGCAAAGTCCACGGAGGGGTCCCCACCATTTTGGTG
TP23778_Query	D	1	chr4	.	CAGCCAAGTGCACTAAAGCTCCCGCATACGCAAAGTCCACGGAGGGGTCCCCACCATTTTGGTG
TP2390_Hit	D	1	chr4	.	CAGCAAATGTAACGGCATGTTGTTCTGCTATCCCCACGTCAAAGCATCTTGTTGGGAATCGGCG
TP2390_Query	D	1	chr4	.	CAGCAAATGTAACGGCATGCTGTTCTGCTATCCCCACGTCAAAGCATCTTGTTGGGAATCGGCG
TP24042_Hit	D	1	chr4	.	CAGCCAATGGCTATGGTATAGAAGCGGTCACGTGCCATCTTCACGACATTATCAACTCGGTAAC
TP24042_Query	D	1	chr4	.	CAGCCAATGGCTATGGTATAGAAGCGGTCACGTGCCATATTCACGACATTATCAACTCGGTAAC
TP24071_Hit	D	1	chr4	.	CAGCCAATGTTCATGATGTTGTTGAAGGTTTGAATTTTGTTGAGATTGTTTCTTCTAGTAAAGA
TP24071_Query	D	1	chr4	.	CAGCCAATGTTCATGATGTTATTGAAGGTTTGAATTTTGTTGAGATTGTTTCTTCTAGTAAAGA
TP241_Hit	D	1	chr4	.	CAGCAAAGATATGAGATGTGAAATACGGTTTTAGCTTCTTCAAAGTCAAGGTACATTTTAGGAT
TP241_Query	D	1	chr4	.	CAGCAAAAATATGAGATGTGAAATACGGTTTTAGCTTCTTCAAAGTCAAGGTACATTTTAGGAT
TP24250_Hit	D	1	chr4	.	CAGCCACAACAATCACATTGTCGGTGCTTTGTAAGAACCTGCGGTGATTTTTGCTTGACAACCA
TP24250_Query	D	1	chr4	.	CAGCCACAACAATCACATTGTCGGTGCTTTGTAAGAACCTGCGATGATTTTTGCTTGACAACCA
TP24511_Hit	D+G	1	chr4	.	CAGCCACAGGTGTTACTGCTGGTGGTGAGCTGACTGGAGTAGGTGTGGGTGGTGATTTGACAGG
TP24511_Query	D+G	1	chr4	.	CAGCCACAGGTGTTACTGCTGGTGGTGAGCTGACTGGAGCAGGTGTGGGTGGTGATTTGACAGG
TP24588_Hit	D	1	chr4	.	CAGCCACATCCACCAATTTTTGCATAATGCTATTCCTATCACCTAAGAGTGAACTTGAGCCTGG
TP24588_Query	D	1	chr4	.	CAGCCACATCCACCAATTTTTGCATAATGCTATTCCTATCACCTAAGAGTGAACTTGAGCCCGG
TP2461_Hit	D	1	chr4	.	CAGCAAATTATCAATATCTCTATTTTTGAGACGTCCTGAGGATATGCTTATTTGGCATTGGAAG
TP2461_Query	D	1	chr4	.	CAGCAAATTATCAATATCTCTATTTTTGAGACGTCCCGAGGATATGCTTATTTGGCATTGGAAG
TP24610_Hit	D	1	chr4	.	CAGCCACATGTGGTCAACTAGTTGATAGCCTTATTCCATGCATTACTTATCTTCAAGGAGGTGC
TP24610_Query	D	1	chr4	.	CAGCCACATGCGGTCAACTAGTTGATAGCCTTATTCCATGCATTACTTATCTTCAAGGAGGTGC
TP2465_Hit	D+G	1	chr4	.	CAGCAAATTATCTTGATCTCTCCAGATCTATCTTGCAGGTGGCACTAGATAAGTCGACACAGGC
TP2465_Query	D+G	1	chr4	.	CAGCAAATTATCTTGATCTATCCAGATCTATCTTGCAGGTGGCACTAGATAAGTCGACACAGGC
TP24697_Hit	D	1	chr4	.	CAGCCACCAACGAGCATGTTTCCGCCTCCGTAGCAGTTTCTGAGAGAGTCATGTGATTTACTGA
TP24697_Query	D	1	chr4	.	CAGCCACCAACGAGCATGTTTCCGCCTCCGTAGCAGTTTCTGAGAGAGTCATGTGAATTACTGA
TP24706_Hit	D+G	1	chr4	.	CAGCCACCAAGTTCCCTATAACAGAAACTAGGTTGACAGGGTTTGCTACTACTGACAGAAACCT
TP24706_Query	D+G	1	chr4	.	CAGCCACCAAGTTCCCTATAACAGAAACCAGGTTGACAGGGTTTGCTACTACTGACAGAAACCT
TP24949_Hit	D	1	chr4	.	CAGCCACCTTGCTGATATCTGTCATCTGCTTGCAAACAAGTTGGTTAACCACGGTGATATTTAT
TP24949_Query	D	1	chr4	.	CAGCCACCTCGCTGATATCTGTCATCTGCTTGCAAACAAGTTGGTTAACCACGGTGATATTTAT
TP25003_Hit	D	1	chr4	.	CAGCCACGAACACAACAAAAATGACACTCATTTCTTCACTCTGTTCTTTTCCTCCAATTCATTC
TP25003_Query	D	1	chr4	.	CAGCCACGAACACAACAAAAATGACACTCATTTCTTCACTCTGTTCTTTTCCCCCAATTCATTC
TP25111_Hit	D+G	1	chr4	.	CAGCCACGTCACGTTTAAAGTAGTCTTTGTAATCATTCTCAATTAGAGATGTTCTTGCATGGAA
TP25111_Query	D+G	1	chr4	.	CAGCCACGTCACATTTAAAGTAGTCTTTGTAATCATTCTCAATTAGAGATGTTCTTGCATGGAA
TP25195_Hit	D+G	1	chr4	.	CAGCCACTACTGTTCGCCGTGGACATAAGCGGAAGGGACCGAAGCGAACTGCTGAAAAAAAAAA
TP25195_Query	D+G	1	chr4	.	CAGCCACTACTGTTCGCCGTGGACATAAGCGGAAGGGACCGAAGCGAACTGCAGAAAAAAAAAA
TP25198_Hit	D	1	chr4	.	CAGCCACTAGAAGACTTACAGTTGCCAAGTCAACAGATGCAATTCCATGCACATTCAAACGGAC
TP25198_Query	D	1	chr4	.	CAGCCACTAGAAGACTTACAGTTGCCAAGTCAACAGATGCAATTCCATGCACATTCAAACGAAC
TP25217_Hit	D	1	chr4	.	CAGCCACTATATGTTGAATTTTCCTTGCTGATGTCTTTGTTTTCGATGATAAGAAATTTTGGTG
TP25217_Query	D	1	chr4	.	CAGCCACTATATGTTGAATTTTCCTTGCTGATGTCTTTGTCTTCGATGATAAGAAATTTTGGTG
TP25389_Hit	D	1	chr4	.	CAGCCACTTATCATAGTAGTGTAAGAAGGACATAGGAAGTATGCAATGGAGTATCTTTCCACTT
TP25389_Query	D	1	chr4	.	CAGCCACTTATCATAGTAGTATAAGAAGGACATAGGAAGTATGCAATGGAGTATCTTTCCACTT
TP25520_Hit	D	1	chr4	.	CAGCTAGAACTTTGAAGCAATGATAACTTGTCTTCCTTTAGGGAAACAAAGGCCTCATATGTTC
TP25520_Query	D	1	chr4	.	CAGCCAGAACTTTGAAGCAATGATAACTTGTCTTCCTTTAGGGAAACAAAGGCCTCATATGTTC
TP25656_Hit	D	1	chr4	.	CAGCCAGATACACAAGCATGGAAACATTGCTGTATACGAAGAAATGAATCATCATTGATTTACA
TP25656_Query	D	1	chr4	.	CAGCCAGATACACAAGCATGGAAACATTCCTGTATACGAAGAAATGAATCATCATTGATTTACA
TP25697_Hit	D+G	1	chr4	.	CAGCCCGATTGGGATATGTTTCATTCACTACATCCTGCGGCTGAGTATCATGGTTCGGCCAGGG
TP25697_Query	D+G	1	chr4	.	CAGCCAGATTGGGATATGTTTCATTCACTACATCCTGCGGCTGAGTATCATGGTTCGGCCAGGG
TP25843_Hit	D	1	chr4	.	CAGCCAGCCTTGTCTGTAGCATGCAAAGTGCAAACATGATCCAATATAAATTTAGACATTTAGT
TP25843_Query	D	1	chr4	.	CAGCCAGCCTTGTCTGTAGCACGCAAAGTGCAAACATGATCCAATATAAATTTAGACATTTAGT

Name	Filter	Nb hit (Mt4.0)	Mt Chr (Mt4.0)	Ms Chr (Li et al., 2014)	Sequence
TP2614_Hit	D	1	chr4	.	CAGCAAATTTTTACAAACTCTTTTTGTTCCGGCATATGCGATTTAGTTCAAACTACGAAGTGTA
TP2614_Query	D	1	chr4	.	CAGCAAATTTTTACAAACTCTTTTTGTTCCGCCATATGCGATTTAGTTCAAACTACGAAGTGTA
TP2615_Hit	D	1	chr4	.	CAGCAAATTTTTACGAACTCTTTTTGTTCTGCCATATGCGATTTAGTTCAAACTATGAAGTGTT
TP2615_Query	D	1	chr4	.	CAGCAAATTTTTACGAACTCTTTTTGTTCCGCCATATGCGATTTAGTTCAAACTATGAAGTGTT
TP26174_Hit	D+G	1	chr4	.	CAGCCAGTTGAAAGAAAATCTCCCAAATGCTTATGAAGATGAAACAAAGCATAAATGCTACACA
TP26174_Query	D+G	1	chr4	.	CAGCCAGTTGAAAGAAAATCTCCCAAATGCTTATGAAGATGAAACAAAGCATAAATACTACACA
TP2618_Hit	D	1	chr4	.	CAGCAAATTTTTGTCGATGGACAGAAACAGGTTCAAGTGGAGAAGGCAAGTTTGAGGCTTGAGA
TP2618_Query	D	1	chr4	.	CAGCAAATTTTTGTCGATGGACAGAAACAGGTTCAAGTGGAGAAGGCAAGTTTGAGGCTTCAGA
TP26246_Hit	D	1	chr4	.	CAGCCATAGCCGAGCTTCTTGATAATGCAGTTGATGAGGTAATTTTTAAGAATCTGACTTTTAA
TP26246_Query	D	1	chr4	.	CAGCCATAACCGAGCTTCTTGATAATGCAGTTGATGAGGTAATTTTTAAGAATCTGACTTTTAA
TP26373_Hit	D	1	chr4	.	CAGCCATAGACAATTGAAGTCATTTAATTTTAGAAAAGCAACTTTGAGATAGAATTGCTTGAAG
TP26373_Query	D	1	chr4	.	CAGCCATAGACAATTGAAGTCATTTAATTTTAGAAAAGCAACTTTGAGATAGAATTGCTGGAAG
TP26400_Hit	D	1	chr4	.	CAGCCATCGCTTTCAGGCCATACAACTCCTCTTACTCTAATCTTCTGCTAGTTCACGCAAAACA
TP26400_Query	D	1	chr4	.	CAGCCATAGCTTTCAGGCCATACAACTCCTCTTACTCTAATCTTCTGCTAGTTCACGCAAAACA
TP26461_Hit	D	1	chr4	.	CAGCCATATCTTCCTTATGTCATCACCAGTATTGCACAACAATCTAATGCAAAGGACGATCCTG
TP26461_Query	D	1	chr4	.	CAGCCATATCTTCCTTATCTCATCACCAGTATTGCACAACAATCTAATGCAAAGGACGATCCTG
TP26485_Hit	D	1	chr4	.	CAGCCATATTGAAGAAAAGGTCAACAATGTTAGGGCACTTTTTGTAACAAGCTGGTGAGCAAAG
TP26485_Query	D	1	chr4	.	CAGCCATATTGAAGAAAAGGTCAACAATGTTAGGGCACTTTTTGTAACAAGCTGGGGAGCAAAG
TP26738_Hit	D	1	chr4	.	CAGCCATCTGATAAGTTGAGTTTGAGAACCAAATGAGTTTGTGTAAGAAAGAAAAGCTAACAGT
TP26738_Query	D	1	chr4	.	CAGCCATCTGACAAGTTGAGTTTGAGAACCAAATGAGTTTGTGTAAGAAAGAAAAGCTAACAGT
TP26811_Hit	D	1	chr4	.	CAGCCATGAAGTTACGCTTAAAGCCATATCTACCAATTTGACAACATTTTGACAATCCTTCTAT
TP26811_Query	D	1	chr4	.	CAGCCATGAAGTTACGCTTAAAGCCATATCTACCAATTTGACAACATTTTAACAATCCTTCTAT
TP26863_Hit	D	1	chr4	.	CAGCCATGAGGTCCTTATGCTTCCATCTGGATCACCAAAAGGCTTCATGTGACAGCTTTCTGCT
TP26863_Query	D	1	chr4	.	CAGCCATGAGGTCCTTATGCTTCCATCTGGATCACCAAAAGGCTTCATGTGACAGCTTTCTGCA
TP26968_Hit	D	1	chr4	.	CAGCCATGCTAACATGTTCGCCAATCCAAGCATTGACCTTCGGAAATGAAGAAGAGATGTTCTG
TP26968_Query	D	1	chr4	.	CAGCCATGCTAACATGTTCACCAATCCAAGCATTGACCTTCGGAAATGAAGAAGAGATGTTCTG
TP27002_Hit	D	1	chr4	.	CAGCCATGGATAAGATTATCTATATCTTGCCATTTTTGTGCAAAGAAAGAATGCATCAAACCTA
TP27002_Query	D	1	chr4	.	CAGCCATGGATAAGATTATCTATATCTTACCATTTTTGTGCAAAGAAAGAATGCATCAAACCTA
TP27141_Hit	D	1	chr4	.	CAGCCATGTCCTTCACCGTCAACGAAGACCACTTCACCGCCGGCGATGACGACAACTATTCTGG
TP27141_Query	D	1	chr4	.	CAGCCATGTCCTTCACCGTCAACGAAGACCACTTCACCGCCGGCGACGACGACAACTATTCTGG
TP27183_Hit	D	1	chr4	.	CAGCCATGTTGAAAGTTTGGATATTTCCAACAAAATACATGAGTATTCAAAGACAATAGTGTAC
TP27183_Query	D	1	chr4	.	CAGCCATGTTGAAAGTTTGGATATTTCCAACAAAATACATGAGTATTCAAAGACAACAGTGTAC
TP27266_Hit	D	1	chr4	.	CAGCGATTAGCAAGTAATTACTTAGATAGCCTAAACGTGGTTGAAATATGGTGCAATATAGTAA
TP27266_Query	D	1	chr4	.	CAGCCATTAGCAAGTAATTACTTAGATAGCCTAAACGTGGTTGAAATATGGTGCAATATAGTAA
TP27380_Hit	D	1	chr4	.	CAGCCATTGACAACAGGAGAGTATCCACTAAGCATGGTGTCTTTTGCGGGAAATCGTTTACCAA
TP27380_Query	D	1	chr4	.	CAGCCATTGACAACAGGAGAGTATCCACTAAGCATGGTGTCTTTTGCGGGAAATCGCTTACCAA
TP27408_Hit	D+G	1	chr4	.	CAGCGATTGCATCATAAAACTCATCTTGTACTTCATCTTCATATTCAGAACTGGAAATGGAAGC
TP27408_Query	D+G	1	chr4	.	CAGCCATTGCATCATAAAACTCATCTTGTACTTCATCTTCATATTCAGAACTGGAAATGGAAGC
TP27428_Hit	D	1	chr4	.	CAGCCATTGGAAATTTTAATGGAATTCCTCAAAGGTATTCAGTGGCTTGTTGAATGTGCTATAC
TP27428_Query	D	1	chr4	.	CAGCCATTGGAAATTTTAATGGAATTCCTCAAAGGTATTCACTGGCTTGTTGAATGTGCTATAC
TP27829_Hit	D	1	chr4	.	CAGCCCAAGTTCCAACATTTCCAAAGCCCTGTACAATGTCATAAAAAAATAACCATAAAAAATC
TP27829_Query	D	1	chr4	.	CAGCCCAAGTTCCAACATTTCCAAAGCCCTGTACAATGTCATAAAAAAATAAACATAAAAAATC
TP27882_Hit	D	1	chr4	.	CAGCCCAATTCAAAGTACATTAACATCACTAAAGCATGTTAAACAATAGGCATGAAGTTAAAAA
TP27882_Query	D	1	chr4	.	CAGCCCAATTCAAAGTACATTAACATCACTAAAGCATGGTAAACAATAGGCATGAAGTTAAAAA
TP2804_Hit	D	1	chr4	.	CAGCAACAAATGATGATGATGGCCCCTCAACAACAATCTGCAAATCCTTTTGGAAATCCTCAAT
TP2804_Query	D	1	chr4	.	CAGCAACAAATGATGACGATGGCCCCTCAACAACAATCTGCAAATCCTTTTGGAAATCCTCAAT
TP28066_Hit	D	1	chr4	.	CAGCCCAGATGAAATTGAAGAGCGTAACATTATCAACCTTGACGAATTCAGTGTCCCATTTGTT
TP28066_Query	D	1	chr4	.	CAGCCCAGATGAAATTGAAGAGCGTAACATTATCAACCTTGACGAATTCAGTGTCCAATTTGTT
TP28347_Hit	D	1	chr4	.	CAGCCCATTTTCCCCATGGTCTCTGCCTCACTCCTCTGTACTTATTATTCTTAGCTCTCTTTTG
TP28347_Query	D	1	chr4	.	CAGCCCATTTTCCCCATGGCCTCTGCCTCACTCCTCTGTACTTATTATTCTTAGCTCTCTTTTG
TP28423_Hit	D	1	chr4	.	CAGCCCCAATCAGATAAAGCCTTGGTAAAGCGCACTAGGCTTGGAAGTTCACGTATCCTCTTGG
TP28423_Query	D	1	chr4	.	CAGCCCCAATCAGATAAAGCCTTGGTAAAGCGCACTAGGCTTGGAAGTTCACGCATCCTCTTGG
TP28760_Hit	D+G	1	chr4	.	CAGCCCCTATCACTAAACCAAATGTGACTCAACCAAAACAGGTTGAGCCTACTCCTAAACAATC
TP28760_Query	D+G	1	chr4	.	CAGCCCCTAGCACTAAACCAAATGTGACTCAACCAAAACAGGTTGAGCCTACTCCTAAACAATC

Name	Filter	Nb hit (Mt4.0)	Mt Chr (Mt4.0)	Ms Chr (Li et al., 2014)	Sequence
TP28890_Hit	D	1	chr4	.	CAGCCCCTTTGTTGTGCCAACAGCTAAAAGGGTTCCGTACTCCTTGTCATGAAAATTTACGGTG
TP28890_Query	D	1	chr4	.	CAGCCCCTTTGCTGTGCCAACAGCTAAAAGGGTTCCGTACTCCTTGTCATGAAAATTTACGGTG
TP28922_Hit	D	1	chr4	.	CAGCCCGAAAAGGAAATTAATTGGAACAACAAGTTTCTCAACAGGCAAAAGGCTTACAATAGTC
TP28922_Query	D	1	chr4	.	CAGCCCGAAAAGGAAATTAATCGGAACAACAAGTTTCTCAACAGGCAAAAGGCTTACAATAGTC
TP28972_Hit	D	1	chr4	.	CAGCCCGAGAAGGTGCATTTTAGTAACTTCCGTGCCTGACTATTTCTCTTAAGCTAGCATTATG
TP28972_Query	D	1	chr4	.	CAGCCCGAGAAGGTGCATTTTAGTAACTTCCGTGCCTAACTATTTCTCTTAAGCTAGCATTATG
TP28996_Hit	D+G	1	chr4	.	CAGCCCGAGTCCAGGAGTGGTTTTAGTCCCGTGAAGACTGAGGAAACATGATTCGTTATTAGCC
TP28996_Query	D+G	1	chr4	.	CAGCCCGAGTCCAGGAGTGGTTTTAGTCCCGTGAAGACTGAGGAAACATGATTAGTTATTAGCC
TP29071_Hit	D	1	chr4	.	CAGCTCGCTGTTTGCCAACATTCAACATGTTTGGGTTAATGTCACATACATATATCTGAGTTTT
TP29071_Query	D	1	chr4	.	CAGCCCGCTGTTTGCCAACATTCAACATGTTTGGGTTAATGTCACATACATATATCTGAGTTTT
TP29403_Hit	D	1	chr4	.	CAGCCCTAGTGTTTCTGATGAGTGTCCAAGATGTGTTGAAGCAAAGAAAAGAGTTTATTTTACG
TP29403_Query	D	1	chr4	.	CAGCCCTAGTGTTTCTGATGAGTATCCAAGATGTGTTGAAGCAAAGAAAAGAGTTTATTTTACG
TP2942_Hit	D	1	chr4	.	CAGCAACAACAGGTTTGGCAGATGGAACAGATGGCGACTGAAACAGGTTTGCAGAATTTCCACC
TP2942_Query	D	1	chr4	.	CAGCAACAACAGGTTTGGCAGATGGAACAGATGGCGACTGAAACAGGTTTGAAGAATTTCCACC
TP29424_Hit	D	1	chr4	.	CAGCCCTATGCCCTGAGTTTTGATAACATTCTTGTGTGATAGCGCTTAACTTTTAAGCAGTTTT
TP29424_Query	D	1	chr4	.	CAGCCCTATGCCCCGAGTTTTGATAACATTCTTGTGTGATAGCGCTTAACTTTTAAGCAGTTTT
TP29665_Hit	D	1	chr4	.	CAGCCCTTACCAACAAGAACACATGTTTAGAAAGCTTAGATTCTGCTTCGGGTACAATGAAGCA
TP29665_Query	D	1	chr4	.	CAGCCCTTACCAACAAGAACACATGTTTAGAAAGCTTAGATTCTGCTTCAGGTACAATGAAGCA
TP29710_Hit	D+G	1	chr4	.	CAGCCCTTCCACTCCAAGTGATATTCTTGAGTACGGCTCATTGAAATCCACGAGTGACCGTTCC
TP29710_Query	D+G	1	chr4	.	CAGCCCTTCCACTCCAAGTGATATTCTTGAGTACGACTCATTGAAATCCACGAGTGACCGTTCC
TP29794_Hit	D	1	chr4	.	CAGCCCTTTAATTAGTTTTTTTTAATCTGAATCGTTGATTCACATCCAACGACTATTATTGAAA
TP29794_Query	D	1	chr4	.	CAGCCCTTTAATTAGTTTTTTTAAATCTGAATCGTTGATTCACATCCAACGACTATTATTGAAA
TP29869_Hit	D+G	1	chr4	.	CAGCCGAAACAGAAAAGACCATGACATTTTACACATTTCTTGGGCATTGATTGTACAGAGCCCA
TP29869_Query	D+G	1	chr4	.	CAGCCGAAACAGAAAAGACCATGACATTTTACACATTTCTTGGGCATTGATTGTACAGAACCCA
TP30115_Hit	D	1	chr4	.	CAGCCGAGATTTCCTTCAAGTCATTGAAGTTTATCTCTAATAAAAGATGGCTTAGTTCAATACA
TP30115_Query	D	1	chr4	.	CAGCCGAGATTTCCTTCAAGTCATTGAAGTTTATCTCTAACAAAAGATGGCTTAGTTCAATACA
TP3019_Hit	D+G	1	chr4	.	CAGCAACAACTTCCCCTATGGGTAGCAAACCTGATTTTCATCACATGATGAGTTCTTCGATCTT
TP3019_Query	D+G	1	chr4	.	CAGCAACAACTTCCCCTATGGGTAGCAAACCGGATTTTCATCACATGATGAGTTCTTCGATCTT
TP30718_Hit	D	1	chr4	.	CAGCGGCGTTCAAATGCGGGATTTCCTGGTACCCCCATTCGGCTTTTGTGGTGCTGAAAAAAAA
TP30718_Query	D	1	chr4	.	CAGCCGCGTTCAAATGCGGGATTTCCTGGTACCCCCATTCGGCTTTTGTGGTGCTGAAAAAAAA
TP30757_Hit	D	1	chr4	.	CAGCCGCTAGAAGACCTCCACCTCCTCCACCAAAGGAGAAAAAAGATCCCAATCTGGGTGCTGA
TP30757_Query	D	1	chr4	.	CAGCCGCTAGAAGACCTCCACCTCCTCCACCAAAGGAGAAAAAAGATCCCAATCTGAGTGCTGA
TP30822_Hit	D	1	chr4	.	CAGCCGCTGTAACAGCCCTGTGGCCACCGGCATCAACATCACGGCTGAAAAAAAAAAAAAAAAA
TP30822_Query	D	1	chr4	.	CAGCCGCTGTAACAGCCCTGTGGCCACCGGCATCAACATCACGGCAGAAAAAAAAAAAAAAAAA
TP31094_Hit	D	1	chr4	.	CAGCCGGTACACCTTACCACGACGGTCTGTTCTTCTTTGACATTCAATTTCCTGCCGATTATCC
TP31094_Query	D	1	chr4	.	CAGCCGGTACACCTTACCACGACGGTCTGTTCTTCTTTGACATTCAATTTCCTGCCGATTACCC
TP31166_Hit	D+G	1	chr4	.	CAGCCGGTGTACTAGTTGAAAGTGCAAGCTTTAACGGACTGGCTAAAGGATCAGCAAGAGGTGG
TP31166_Query	D+G	1	chr4	.	CAGCCGGTGTACTAGTTGAAAGTGCAAGCTTTAACGGACTGGCTAAAGGATCAGCAAGAGGCGG
TP31233_Hit	D+G	1	chr4	.	CAGCCGTAAGCATGCCATTGCTTTTCATTGATGCTGTTAAGTTTCAACAGTTGAGTTTTTGGTA
TP31233_Query	D+G	1	chr4	.	CAGCCGTAAGCATGCCATTGCTTTTCATTGATGCTGTTAAGTTTCAACAGTTGAGTTTCTGGTA
TP31249_Hit	D+G	1	chr4	.	CAGCCGTACCCCTACTATGCTAATCAACATCTTGCAGTCATCCCTAACCCTTGTTCAACATATG
TP31249_Query	D+G	1	chr4	.	CAGCCGTACCCCTACTATGCTAATCAACATCCTGCAGTCATCCCTAACCCTTGTTCAACATATG
TP3133_Hit	D	1	chr4	.	CAGCGACAATATTGATTATTATATGTGTGTTCTCTGTCGGGACGACTGTTGGATTCCATCATAC
TP3133_Query	D	1	chr4	.	CAGCAACAATATTGATTATTATATGTGTGTTCTCTGTCGGGACGACTGTTGGATTCCATCATAC
TP31370_Hit	D	1	chr4	.	CAGCCGTCTTGCTTGACTATTGCCTCCGTTTTGGCTGTATAATATCTGGGGCTGTGAGAAATTG
TP31370_Query	D	1	chr4	.	CAGCCGTCTTGCTTGACTATTGCCTCCGTTTTGGCTGTATAACATCTGGGGCTGTGAGAAATTG
TP31421_Hit	D	1	chr4	.	CAGCCGTGCTGGGGTGAAACATGTTAGGTGGTACACTGCTATGCTAGTTAACCGGTAGCTTGGT
TP31421_Query	D	1	chr4	.	CAGCCGTGCTGGGGTGAAACATGTTAGGTGGTACACTGCTATGCTAGTTAACAGGTAGCTTGGT
TP31596_Hit	D	1	chr4	.	CAGCCGTTTGCATCTTGAACCTGCAACTTCTCCAAATTTGTAACAACCTCGTCCATGTTTGGTC
TP31596_Query	D	1	chr4	.	CAGCCGTTTACATCTTGAACCTGCAACTTCTCCAAATTTGTAACAACCTCGTCCATGTTTGGTC
TP31649_Hit	D	1	chr4	.	CAGCCTAAACAAAATAATGCTATATTAGAGGCTTTCAAAGTTAACTACATTGGTGACTCTCCAT
TP31649_Query	D	1	chr4	.	CAGCCTAAACAAAATAATGCAATATTAGAGGCTTTCAAAGTTAACTACATTGGTGACTCTCCAT
TP31713_Hit	D	1	chr4	.	CAGCCTAACCATCTGAAAATCACATACCACACCTTAGAGGCCACCCCACACAAAACAAAAAGAT
TP31713_Query	D	1	chr4	.	CAGCCTAACCATCTAAAAATCACATACCACACCTTAGAGGCCACCCCACACAAAACAAAAAGAT

Name	Filter	Nb hit (Mt4.0)	Mt Chr (Mt4.0)	Ms Chr (Li et al., 2014)	Sequence
TP31769_Hit	D	1	chr4	.	CAGCCTAAGTGAGTCTCCACGCTGGGCACCACTCTTCTTATTTATAAAAACTAACACTGGCCTT
TP31769_Query	D	1	chr4	.	CAGCCTAAGTGAGTCTCCACACTGGGCACCACTCTTCTTATTTATAAAAACTAACACTGGCCTT
TP31850_Hit	D	1	chr4	.	CAGCCTACACCACCACTGTTTACAGACCCCTTCTTACGCTTTCTCTGGAATGTGTACTTGATAA
TP31850_Query	D	1	chr4	.	CAGCCTACACCACCACTGCTTACAGACCCCTTCTTACGCTTTCTCTGGAATGTGTACTTGATAA
TP31856_Hit	D	1	chr4	.	CAGCCTACAGATGACAAGAATATCTGTTATTTATTGCAAAAGCTATAGAGATAATAACTGAGTG
TP31856_Query	D	1	chr4	.	CAGCCTACAGATGACAAGAATATCCGTTATTTATTGCAAAAGCTATAGAGATAATAACTGAGTG
TP31864_Hit	D	1	chr4	.	CAGCCTACATCCACCATATACACCGAGATTATTCATTAAAAGACCTTTGGCTCCTCCTTCGTCA
TP31864_Query	D	1	chr4	.	CAGCCTACATCCACCATATACACCGAGATTATTCATTAAAAGACCTTTGGCCCCTCCTTCGTCA
TP32045_Hit	D+G	1	chr4	.	CAGCCTATAGATTGAATGTTAGACCTTTGTTAAGGCTGGCTTGTAGCTCTGTTTTTGGTTCAGC
TP32045_Query	D+G	1	chr4	.	CAGCCTATAGATTGAATGTTAGACCTTTGCTAAGGCTGGCTTGTAGCTCTGTTTTTGGTTCAGC
TP32093_Hit	D	1	chr4	.	CAGCCTATCCTTCAGATTAATATTATCAACACCACAGCACAACTTGCGAAAAATGTGAAGCAAA
TP32093_Query	D	1	chr4	.	CAGCCTATCCTTCAGATTAATATTATCAACACCACAGCACAACTTGCAAAAAATGTGAAGCAAA
TP32154_Hit	D	1	chr4	.	CAGCCTATGGCACCGCCATGGCAGGAATCCCTTCACAAATTGGCTGTGGGAGTTGTCAAAAAAA
TP32154_Query	D	1	chr4	.	CAGCCTATGGCACCGCCATGGCAGGAATCCCTTCACAAATTGGCTATGGGAGTTGTCAAAAAAA
TP32252_Hit	D	1	chr4	.	CAGCCTCAAATGAGGCATCACTTCACAATAATAAATAGTTAAGTACACTACAGAGAAAGAAGTG
TP32252_Query	D	1	chr4	.	CAGCCTCAAATGAGACATCACTTCACAATAATAAATAGTTAAGTACACTACAGAGAAAGAAGTG
TP32426_Hit	D+G	1	chr4	.	CAGCCTCAGCAACCTGGCTATAACAGTTCGCGGGTTTACCTAGATCTTCTTGCTGATCTTCCCT
TP32426_Query	D+G	1	chr4	.	CAGCCTCAGCAACCCGGCTATAACAGTTCGCGGGTTTACCTAGATCTTCTTGCTGATCTTCCCT
TP3245_Hit	D	1	chr4	.	CAGCAACACAGAAGCACTCTTCATTATGTATTTATCGAAGTCGCAAAAAGATAAGGCAAGTTGT
TP3245_Query	D	1	chr4	.	CAGCAACACAGAAGCACTCTTCATTATGTATTTATCGAAGTCGCAAAAAGAGAAGGCAAGTTGT
TP32460_Hit	D	1	chr4	.	CAGCCTCAGGTTTTCGGTCCCCATTAAACCCAAATTTACATCCCACTGAACATGACTTCCATTC
TP32460_Query	D	1	chr4	.	CAGCCTCAGGTTTCCGGTCCCCATTAAACCCAAATTTACATCCCACTGAACATGACTTCCATTC
TP32492_Hit	D+G	1	chr4	.	CAGCCTCATATTCCTTAGGCAATCCTTCCAATATCGCATCAATGTGTTCTTGCAAGTTCACTGG
TP32492_Query	D+G	1	chr4	.	CAGCCTCATATTCCTTAGGCAATCCTTCCAATATCGCATCAATGTGTTCTTGCAAGTTCACCGG
TP32546_Hit	D	1	chr4	.	CAGCCTCATTAATCGAAGGCTGGTACTTAACAACACGTCCATCAAGAGGATTATGGGGATTCTT
TP32546_Query	D	1	chr4	.	CAGCCTCATTAATCGAAGGCTGGTACTTAACAACACGTCCATCAAGAGAATTATGGGGATTCTT
TP32660_Hit	D+G	1	chr4	.	CAGCCTCCATCAATTGTTACGGATGAGATTTGTACTGCATGTGACTTCAACCGTCCCGGAAAGA
TP32660_Query	D+G	1	chr4	.	CAGCCTCCATCAATTGTTACGGATGAGATTTGTACTGCATGTGACTTCAACCGTCCCGAAAAGA
TP33219_Hit	D	1	chr4	.	CAGCCTCTTTTTTGACACTAAAGCTCGAGATTGCTTAGGCTCTGAATGCATTTCCCATAAAAAA
TP33219_Query	D	1	chr4	.	CAGCCTCTTTTTCGACACTAAAGCTCGAGATTGCTTAGGCTCTGAATGCATTTCCCATAAAAAA
TP33258_Hit	D	1	chr4	.	CAGCCTGAACAGGAGAAAACTTGTGATTCTGCTGAAATTGAAAATAAGGAATGCTTTGGTGCTG
TP33258_Query	D	1	chr4	.	CAGCCTGAACAGGAGAAAACTTGTGATTCTGCTGAAATTGAAAATAAGGAATGCTTTGGAGCTG
TP33292_Hit	D	1	chr4	.	CAGCCTGAATGAAAATAAACAGAACATTTCTCAGAGAAAATCAACTTTGCTGAAGAGCAAAAAT
TP33292_Query	D	1	chr4	.	CAGCCTGAATGAAAATAAACAGAACATTTCTCAGAGAAAATCAACTTTGCCGAAGAGCAAAAAT
TP33353_Hit	D	1	chr4	.	CAGCCTGGGATATAAATAGCAACCTGCCTCATCTTTAAATCAAGCACTCGTAATATTTCTGCGG
TP33353_Query	D	1	chr4	.	CAGCCTGAGATATAAATAGCAACCTGCCTCATCTTTAAATCAAGCACTCGTAATATTTCTGCGG
TP33402_Hit	D+G	1	chr4	.	CAGCCTGATAGAACAATTAATTGTTGATTAGAATAGTTAAAGGTGGTTAGAAACATTCAATCAG
TP33402_Query	D+G	1	chr4	.	CAGCCTGATAGAACAATTAATTGTTGATTAGAATAGTCAAAGGTGGTTAGAAACATTCAATCAG
TP33425_Hit	D	1	chr4	.	CAGCCTGATGATTTTCATCTTCATCTTCGTGACGGTGCTCTTCTTGAAGCTGTAACCCCTCACA
TP33425_Query	D	1	chr4	.	CAGCCTGATGATTTTCATCTTCATCTTCGTGATGGTGCTCTTCTTGAAGCTGTAACCCCTCACA
TP33570_Hit	D	1	chr4	.	CAGCCTGCCCTTGCAACTTCCTCATCAAATGTTGATTCAAAGCCCTCAATTTCATGACCTCATC
TP33570_Query	D	1	chr4	.	CAGCCTGCCCTTGCAACTTCCTCATCAAATGCTGATTCAAAGCCCTCAATTTCATGACCTCATC
TP33706_Hit	D+G	1	chr4	.	CAGCCTGGCATGCCTAAGGTCTTTGAGGCTGGTACAAGCTAGAACTGTCTTCATCTGGTAACAT
TP33706_Query	D+G	1	chr4	.	CAGCCTGGCATGCCTAAGGCCTTTGAGGCTGGTACAAGCTAGAACTGTCTTCATCTGGTAACAT
TP3378_Hit	D	1	chr4	.	CAGCAACACTTTTCAGTCTTTCAGTTATCGATGAAAATAAGGTTACAATTGGTTCTTCAGGAGC
TP3378_Query	D	1	chr4	.	CAGCAACACTTTTCAGCCTTTCAGTTATCGATGAAAATAAGGTTACAATTGGTTCTTCAGGAGC
TP3381_Hit	D+G	1	chr4	.	CAGCAACACTTTTTCCAATATTGGGGAACTGATAAGTAACACAAAGATTGCTTCTCCAACGTAA
TP3381_Query	D+G	1	chr4	.	CAGCAACACTTTTTCCAATATTGGGGAACTGATAAGTAACACAAAGATTGCTTCTCCAACCTAA
TP33993_Hit	D	1	chr4	.	CAGCCTTAAAATTATGTTCGATAGCTTTCAATTCAAGGAGGAGTTAACTTATTTTCAGATCTGC
TP33993_Query	D	1	chr4	.	CAGCCTTAAAATTATGTTCAATAGCTTTCAATTCAAGGAGGAGTTAACTTATTTTCAGATCTGC
TP34039_Hit	D	1	chr4	.	CAGCCTTAAGTGCATCAGCTTTGCCAAACTCAGAAGGATTTCCGATTGCAATTCTAGAAATCGG
TP34039_Query	D	1	chr4	.	CAGCCTTAAGTGCATCAGCTTTGCCAAACTCAGAAGGATTTCCAATTGCAATTCTAGAAATCGG
TP34204_Hit	D	1	chr4	.	CAGCCTTATATTAGGGTTGTAGGAATAGAAGATGGAAATGAAGCCAAGTCTCGAGGTCCCACTG
TP34204_Query	D	1	chr4	.	CAGCCTTATATCAGGGTTGTAGGAATAGAAGATGGAAATGAAGCCAAGTCTCGAGGTCCCACTG

Name	Filter	Nb hit (Mt4.0)	Mt Chr (Mt4.0)	Ms Chr (Li et al., 2014)	Sequence
TP34230_Hit	D	1	chr4	.	CAGCCTTATGGCCCAGCGACAAATCTTCAACAGCCCTCTTCTCCTCTCTCCTTTTCCTAATAGT
TP34230_Query	D	1	chr4	.	CAGCCTTATGGCCCAGCGACAAATCTTCAACAGCCCTCTTCTCCTCTCTCCTTTTCCTAACAGT
TP34233_Hit	D	1	chr4	.	CAGCCTTATGTAGGAGTAGGCGATCCATATTTGGATCAGAATCTTTATTGGTCCTTCGCGCTTT
TP34233_Query	D	1	chr4	.	CAGCCTTATGTAGGAGTAGGCGATCCATATTTGGATCAGAATCTTTATTGGGCCTTCGCGCTTT
TP34234_Hit	D+G	1	chr4	.	CAGCCTTATGTATTTAATGAATTGTTCAAGACCTGATATTGCATATGTTGTAAATAGGTTGAGT
TP34234_Query	D+G	1	chr4	.	CAGCCTTATGTATTTAATGAATTGTTCAAGACCTGATATTGCATATGCTGTAAATAGGTTGAGT
TP34328_Hit	D	1	chr4	.	CAGCCTTCATCTCCTTGGCAGTCATCATTGTTAACTTCTTACTTGAAGTAACGTTGGAGGATTC
TP34328_Query	D	1	chr4	.	CAGCCTTCATCTCCTTGGCAGTCATCATTGTTAACTTCATACTTGAAGTAACGTTGGAGGATTC
TP34362_Hit	D	1	chr4	.	CAGCTTTCCCATGATAATTGGATCTTTAATCATGTTTTGTTTCTCAAAGCTTTGAACACGCTTG
TP34362_Query	D	1	chr4	.	CAGCCTTCCCATGATAATTGGATCTTTAATCATGTTTTGTTTCTCAAAGCTTTGAACACGCTTG
TP34562_Hit	D+G	1	chr4	.	CAGCCTTGACTGTGTTTGGATCACCAACATCCCCAATCACTATCTCTACTGACCTTGGAAGCTG
TP34562_Query	D+G	1	chr4	.	CAGCCTTGACTGTGTTTGGATCACCAACATCCCCAATCACTATCTCTACTGACCTTGGAAGCAG
TP34607_Hit	D	1	chr4	.	CAGCCTTGATTTTCAATCTCTCTTTTGTTGGGCCTACAAGCACTTTAGTGTCCTGTTTTTATGT
TP34607_Query	D	1	chr4	.	CAGCCTTGATTTTCAATCTCTCTTTTGTTGGGCCTACAAGCACTTTAGTATCCTGTTTTTATGT
TP34671_Hit	D	1	chr4	.	CAGCCTTGGAGGGTGTTAGAGATATAACCCTTGCTTCTCACCTCATAGGAGATATGAGTGGCTC
TP34671_Query	D	1	chr4	.	CAGCCTTGGAGGGTGTTAGAGATATAACCCTTGCTTCTCACCTCATAGGAGACATGAGTGGCTC
TP34812_Hit	D	1	chr4	.	CAGCCTTTAATTCCTCTAGTCCAATAATCTCCTTAAGTGGAAAAGCAAAACTTCCTGTAGACTT
TP34812_Query	D	1	chr4	.	CAGCCTTTAATTCCTCTAGTCCAATAATCTCCTTAAGTGGAAAAGCAAAACTTCCTATAGACTT
TP34818_Hit	D	1	chr4	.	CAGCCTTTACATAAGCATTGGAAGCAGGTCCTTGTCTATCCTCAAATTTCTCCATCTCTACAAC
TP34818_Query	D	1	chr4	.	CAGCCTTTACATAAGCATTGGAAGCAGGTCCTTGTCTATCCTCAAATTACTCCATCTCTACAAC
TP34826_Hit	D	1	chr4	.	CAGCCTTTACTCAATTTTTCCATTCAGTGCCTGGGAATGGACAGTGGAAGCGCAATGCGGGTGC
TP34826_Query	D	1	chr4	.	CAGCCTTTACTCAATTTTTCCATTCAGCGCCTGGGAATGGACAGTGGAAGCGCAATGCGGGTGC
TP34879_Hit	D	1	chr4	.	CAGCCTTTCCAATCCTTCATCAGTAGAAGATGAAACTAATCACAGCGACCAGACTGATAATCGA
TP34879_Query	D	1	chr4	.	CAGCCTTTCCAATCCGTCATCAGTAGAAGATGAAACTAATCACAGCGACCAGACTGATAATCGA
TP34928_Hit	D	1	chr4	.	CAGCCTTTGAAATTGATCTGAGGGTTACAGTCGTTGGTGCCCATGGATTTCCATTAGTTGCCTC
TP34928_Query	D	1	chr4	.	CAGCCTTTGAAATTGATCTGAGGGTTACAGTAGTTGGTGCCCATGGATTTCCATTAGTTGCCTC
TP35078_Hit	D+G	1	chr4	.	CAGCCTTTTCTTTCAATAAAAGATTTTGCCGAGTGTTATTTGCATTCATTGTTTTAAATGTAAG
TP35078_Query	D+G	1	chr4	.	CAGCCTTTTCGTTCAATAAAAGATTTTGCCGAGTGTTATTTGCATTCATTGTTTTAAATGTAAG
TP35092_Hit	D	1	chr4	.	CAGCCTTTTGAGAACAAAATCAAAAAACCGCACCTAATTCTTTCTTTTTATCTAGTATAGTACT
TP35092_Query	D	1	chr4	.	CAGCCTTTTGAGAACAAAATCAAAAAACCACACCTAATTCTTTCTTTTTATCTAGTATAGTACT
TP35172_Hit	D	1	chr4	.	CAGCGAAAACCGGTACCTGTAAAATGCTAGTAGAACAAGTTTATTCAAATGCTCATAACAATCT
TP35172_Query	D	1	chr4	.	CAGCGAAAACCGGTACCTGCAAAATGCTAGTAGAACAAGTTTATTCAAATGCTCATAACAATCT
TP35322_Hit	D	1	chr4	.	CAGCGAACAACAATAAACATATTCTCCATCACGGTTTGGTGATTCTCACAATTGACTAATTTCC
TP35322_Query	D	1	chr4	.	CAGCGAACAACAATAAACATATTCTCCATCACGGTTTGGCGATTCTCACAATTGACTAATTTCC
TP35399_Hit	D	1	chr4	.	CAGCGAACTGGCCACTGTCTCCCATCAGAGATTTGAAGTTTGATAATTCCTGAAACGCCATTCA
TP35399_Query	D	1	chr4	.	CAGCGAACTGGCCACTGCCTCCCATCAGAGATTTGAAGTTTGATAATTCCTGAAACGCCATTCA
TP35467_Hit	D	1	chr4	.	CAGCGAAGGAAGATTCAGTGTGAAAACAATGGAGGAGTTCCAAAACTCGATCTTGGCTGAAATC
TP35467_Query	D	1	chr4	.	CAGCGAAGGAAGATTCAGTGCGAAAACAATGGAGGAGTTCCAAAACTCGATCTTGGCTGAAATC
TP35490_Hit	D	1	chr4	.	CAGCGAAGGTCAGTGGCAACGAAGGAGAATTGAGGCTTCGCAGAATGTGACGATATAGCGACAG
TP35490_Query	D	1	chr4	.	CAGCGAAGGTCAGTGGCAACGAAGGAGAATTGAGGCTTCGCAGAATGGGACGATATAGCGACAG
TP35573_Hit	D	1	chr4	.	CAGCGAATCGCCATAGCTAGAGCTTTACTTAGGGATCCAAAGGTTCTCCTGCTTGATGAAGCCA
TP35573_Query	D	1	chr4	.	CAGCGAATCGCCATAGCTAGAGCTTTACTTAGGGATCCAAAGGTTCTCCTGCTTGATGAAGCAA
TP35576_Hit	D+G	1	chr4	.	CAGCGAATCTGTTTTCTAAGAGGAGAAGCTCAACTTGTTCACAGATAACAACAGACTTACATTG
TP35576_Query	D+G	1	chr4	.	CAGCGAATCTGTTTTCTAAGAGGAGAAGCTCAACTTGTTCACAGATAACAACAGACTGACATTG
TP35595_Hit	D+G	1	chr4	.	CAGCGAATGCTCAATCATACAAATCCCTAAAAAGGGAAGAAGGTGAAAAACTAGAAAAACTAGA
TP35595_Query	D+G	1	chr4	.	CAGCGAATGCTCAATCATACAAATCCCCAAAAAGGGAAGAAGGTGAAAAACTAGAAAAACTAGA
TP35647_Hit	D	1	chr4	.	CAGCGACAAAACAACATTTGGCATTTGTTTGTCATCACAAAGTCAAATTCCAGAAGAGCAATTA
TP35647_Query	D	1	chr4	.	CAGCGACAAAACAACATTTGGCAGTTGTTTGTCATCACAAAGTCAAATTCCAGAAGAGCAATTA
TP35725_Hit	D	1	chr4	.	CAGCGACACGGTGTAGCTTGTTGTGAAGCCATTAGACCTGAGAGAAATTATAAACACAGATGTG
TP35725_Query	D	1	chr4	.	CAGCGACACGGTGTAGCTTGTTGCGAAGCCATTAGACCTGAGAGAAATTATAAACACAGATGTG
TP35951_Hit	D	1	chr4	.	CAGCGACTAAAAAGCCAATAAACAAATACCAATCTGTACCAAACAAAACCAGCAATGAATGATT
TP35951_Query	D	1	chr4	.	CAGCGACTAAAAAGCCAATAAACAAATACCAATCTGTACCAAACAAAACCAACAATGAATGATT
TP3600_Hit	D+G	1	chr4	.	CAGCAACAGTTAGTTACAGAAGTTGCAAATGTAGCTAAGGGTCCTGTGATTCTTGCCATAATGT
TP3600_Query	D+G	1	chr4	.	CAGCAACAGTTAGTTACAGAAGTTGCAAATGTAGCTAAGGGACCTGTGATTCTTGCCATAATGT

Name	Filter	Nb hit (Mt4.0)	Mt Chr (Mt4.0)	Ms Chr (Li et al., 2014)	Sequence
TP36015_Hit	D	1	chr4	.	CAGCGACTTCAACTGCCACCGGTGACTCATATTCCGTTGGATCTTGTGGCTGAAATCCTGTGCA
TP36015_Query	D	1	chr4	.	CAGCGACTTCAACTGCCACCAGTGACTCATATTCCGTTGGATCTTGTGGCTGAAATCCTGTGCA
TP36038_Hit	D+G	1	chr4	.	CAGCGACTTGGACGTACAACTCGGTAAACACCAGAACTTGCAAGAATATAAACATCTTTGTTGT
TP36038_Query	D+G	1	chr4	.	CAGCGACTTGGACGAACAACTCGGTAAACACCAGAACTTGCAAGAATATAAACATCTTTGTTGT
TP3615_Hit	D	1	chr4	.	CAGCAACATAAAACAAAAGGCAATTAAAAAACCATGAGACATGGTAACCAGCCTACTAAATCTT
TP3615_Query	D	1	chr4	.	CAGCAACATAAAACAAAAGGCAATTAAAAAACCACGAGACATGGTAACCAGCCTACTAAATCTT
TP36293_Hit	D	1	chr4	.	CAGCGAGGATAAGAGTGCACAGGCTCGCAGAACGGTAACAAATGTTGCTTGGTCTGGTAAGATA
TP36293_Query	D	1	chr4	.	CAGCGAGGATAAGAGCGCACAGGCTCGCAGAACGGTAACAAATGTTGCTTGGTCTGGTAAGATA
TP36305_Hit	D	1	chr4	.	CAGCGAGGCCAACTTTCTACAACTATGTCGTCGTAATGTTTATATTCAGTGCAATTACTGTGTT
TP36305_Query	D	1	chr4	.	CAGCGAGGCCAACTTTCTACAACTATGTCGTCGTAATGTTTATATTCAGTGCAATTACTATGTT
TP36372_Hit	D	1	chr4	.	CAGCGAGTAGCACATCTTCATTGCTCCACTCTCCGCTTCTATACCCATTTCTCTTCCCTTCTTT
TP36372_Query	D	1	chr4	.	CAGCGAGTAGCACATCTTCATTGCTCCACTCTCCGCTTCTATACCCATTTCTCTTCCCTTCTTC
TP36605_Hit	D	1	chr4	.	CAGCGATCTTGGTGGTTTTGAATAGTTGCGTAGTGACATATATGCAAAATGTAGAAATTATTAT
TP36605_Query	D	1	chr4	.	CAGCGATCTTGGTGGTTTTGAATAGTTGCGTAGTAACATATATGCAAAATGTAGAAATTATTAT
TP36645_Hit	D	1	chr4	.	CAGCTATGATAAGGATTCAAGGAGAGGTGAAAAATTCAGGTAACATTGCGAAAACTGTAATGCA
TP36645_Query	D	1	chr4	.	CAGCGATGATAAGGATTCAAGGAGAGGTGAAAAATTCAGGTAACATTGCGAAAACTGTAATGCA
TP36654_Hit	D+G	1	chr4	.	CAGCGATGATGCCATGGCCATAACTGAGAGGATAGATAACAAAGACACTATGATAAGGTTGGTT
TP36654_Query	D+G	1	chr4	.	CAGCGATGATGCCATGGCCATAACTGAGAGGATAGATAACAAAGACACTACGATAAGGTTGGTT
TP36656_Hit	D+G	1	chr4	.	CAGCGATGATGCTGAGCACAACTTGGAGAGACAATGGCGAAAACACGATGTTCTTTTCTGATTC
TP36656_Query	D+G	1	chr4	.	CAGCGATGATGCTGAGCACAACTTGAAGAGACAATGGCGAAAACACGATGTTCTTTTCTGATTC
TP36762_Hit	D	1	chr4	.	CAGCGATGTTCCACCAGGGCTGACACGGCGTTCATGTTCTGCTGAAAAAAAAAAAAAAAAAAAA
TP36762_Query	D	1	chr4	.	CAGCGATGTTCCACCAGGGCTGACACGGCGTTCATGTTCCGCTGAAAAAAAAAAAAAAAAAAAA
TP36813_Hit	D	1	chr4	.	CAGCGATTCCCTTTCTTGAGAAAACGCATGGATGAAGTTATTGGGAACTTTTTACGAGAAGGCC
TP36813_Query	D	1	chr4	.	CAGCGATTCCCTTTCTTGAGAAAACGCATGGATGAAGTTATTGGAAACTTTTTACGAGAAGGCC
TP37298_Hit	D	1	chr4	.	CAGCGCCAAAAGGTTCGTGTCTCCCGAAACCGCATTTTGGATTCTGCTGAAAAAAAAAAAAAAA
TP37298_Query	D	1	chr4	.	CAGCGCCAAAAGGTTCGTGTCTCCCGAAACCGCATTTTGGATTCCGCTGAAAAAAAAAAAAAAA
TP37323_Hit	D	1	chr4	.	CAGCGCCAATGCATATTTCAAGCCTCTTTTGCCATGACAAAGGAGAAAAGTTGGAGCTATAAAG
TP37323_Query	D	1	chr4	.	CAGCGCCAATGCATATTTCAAGCCTCTTTTGCCATGACAAAGGAGAAAAGTTGGAGCCATAAAG
TP37420_Hit	D	1	chr4	.	CAGCGGCATTTAAATCCATTAACATGGCCGGAGATTTTGCGACAATTTGCATTGTCAGCTGGAT
TP37420_Query	D	1	chr4	.	CAGCGCCATTTAAATCCATTAACATGGCCGGAGATTTTGCGACAATTTGCATTGTCAGCTGGAT
TP3743_Hit	D	1	chr4	.	CAGCAACATCTATTTCAAGCTTGCAAATTGCAATGTCAAGATCATGCGTTCCTTCATCACAAAA
TP3743_Query	D	1	chr4	.	CAGCAACATCTATTTCAAGCTTGCAAATTGCAATGTCAAGATCATGCATTCCTTCATCACAAAA
TP38123_Hit	D	1	chr4	.	CAGCGCTCAGTTTTATTCAGTGCTTTACATTCTTTAATTTGGGATCAAGTTCCTGCAACTGTTG
TP38123_Query	D	1	chr4	.	CAGCGCTCAGTTTTATTCAGTGCTTTACATTCTTTAATTTGGGATCAAGTTCCTGCAACTGTTA
TP38296_Hit	D	1	chr4	.	CAGCGTTTGAGATCAAAGTTGTTGGCACAAGGGAGCACAAAACTAAAAGCAAAATAAAAAATGT
TP38296_Query	D	1	chr4	.	CAGCGCTTGAGATCAAAGTTGTTGGCACAAGGGAGCACAAAACTAAAAGCAAAATAAAAAATGT
TP38453_Hit	D	1	chr4	.	CAGCGGAATAAGTAGAGGGCACTAAGGGAGAGTTCAGGGTCTCTTAAATACCGTAGAACTACCA
TP38453_Query	D	1	chr4	.	CAGCGGAATAAGTAGAGGGCACCAAGGGAGAGTTCAGGGTCTCTTAAATACCGTAGAACTACCA
TP38459_Hit	D	1	chr4	.	CAGCGGAATCCAAAATGCGGTTTCGGGAGACACGAACCTTTTGGTGCTGAAAAAAAAAAAAAAA
TP38459_Query	D	1	chr4	.	CAGCGGAATCCAAAATGCGGTTTCGGGAGACACGAACCTTTTGGCGCTGAAAAAAAAAAAAAAA
TP38587_Hit	D	1	chr4	.	CAGCGGAGATTTCGAAGAAGATCAAAACAGCAATAACAACAGCAATAGCAATAGCTACAGCGTG
TP38587_Query	D	1	chr4	.	CAGCGGAGATTTCGAAGAAGATCAAAACAGCAATAACAACAGCAATAGCAATAGCTACAGCATG
TP38674_Hit	D	1	chr4	.	CAGCGGATAGCATTTCTATTGCCTGAATAGAAAAGACAACAAATACATAAATAAATTGTACGCA
TP38674_Query	D	1	chr4	.	CAGCGGATAGCATTTCTATTGCCTGAAAAGAAAAGACAACAAATACATAAATAAATTGTACGCA
TP38800_Hit	D+G	1	chr4	.	CAGCGGCCACGTTTTCAAACATCGGTGGCAGATTACTGGTGCCATATTGGTTAGTCATCAAGGA
TP38800_Query	D+G	1	chr4	.	CAGCGGCAACGTTTTCAAACATCGGTGGCAGATTACTGGTGCCATATTGGTTAGTCATCAAGGA
TP39267_Hit	D	1	chr4	.	CAGCGGCTAGAGGAGATGGAAGTGCAGAATATTCCTATTGGAGAAGGGAAGAAACAATTTGAGG
TP39267_Query	D	1	chr4	.	CAGCGGCTAGAGGAGATGGAAGTGCAGAATATTCCAATTGGAGAAGGGAAGAAACAATTTGAGG
TP39647_Hit	D	1	chr4	.	CAGCGGTAACAGAGTAATTCAAAGTTGTAAAAAATTGCAAAAGTTATATGCTAAGTTAAAAGTA
TP39647_Query	D	1	chr4	.	CAGCGGTAACAGAGTAATTCAAAGGTGTAAAAAATTGCAAAAGTTATATGCTAAGTTAAAAGTA
TP39651_Hit	D	1	chr4	.	CAGCGGTAACCCTAGAATTGTGAATAGAAATGCCAGTGTTTTGGTTAGGATCTGTTCTACCTTG
TP39651_Query	D	1	chr4	.	CAGCGGTAACCCTAGAATTGTGAATAGAAATGCCAGTGTTTTGGTTAGGATCTGTTCTACCTGG
TP39662_Hit	D	1	chr4	.	CAGCGGTAATAGCACTGAAAACCGAACATGTCCAATTCCACATTCCGCTACTCCCTAAAGCAGA
TP39662_Query	D	1	chr4	.	CAGCGGTAATAGCACTAAAAACCGAACATGTCCAATTCCACATTCCGCTACTCCCTAAAGCAGA

Name	Filter	Nb hit (Mt4.0)	Mt Chr (Mt4.0)	Ms Chr (Li et al., 2014)	Sequence
TP39733_Hit	D+G	1	chr4	.	CAGCGGTATTGCAGTTCCAAAAAGTGCAAATAAAATAGTAATAATACCCAAGAGCTATGTCCTT
TP39733_Query	D+G	1	chr4	.	CAGCGGTATTGCAGTTCCAAAAAGTGCAAATAAAATAGTAATAATACCCAAGAGCTATGTCATT
TP39755_Hit	D	1	chr4	.	CAGCGGTCCGAAAAACTACATCATTTTAAGAGATAGACAATATTGATGACTCTTGCAAGTAACG
TP39755_Query	D	1	chr4	.	CAGCGGTCCGAAAAACTACATCATTTTAAGAGATAGACAATATTGATGACTCTTGCAAGTAACA
TP3986_Hit	D	1	chr4	.	CAGCAATCAGCAGAGTGGGGACTTGACAAAGTCTGATAGAGCAATTGATGGTATATTTGGATTT
TP3986_Query	D	1	chr4	.	CAGCAACCAGCAGAGTGGGGACTTGACAAAGTCTGATAGAGCAATTGATGGTATATTTGGATTT
TP39901_Hit	D	1	chr4	.	CAGCGGTGGCTGTTAAATTCTTGGCACCCAATATTTTTTTACAGAGAATTAACATGCATTGAAA
TP39901_Query	D	1	chr4	.	CAGCGGTGGCTGTTAAATTCTTGGCACCCAATTTTTTTTTACAGAGAATTAACATGCATTGAAA
TP39995_Hit	D+G	1	chr4	.	CAGCGGTTATCGCTCTTGCATTTGTCACCATCCACACCTTTTCGATGGTCATTGCATTTAATGG
TP39995_Query	D+G	1	chr4	.	CAGCGGTTATCACTCTTGCATTTGTCACCATCCACACCTTTTCGATGGTCATTGCATTTAATGG
TP40010_Hit	D+G	1	chr4	.	CAGCGGTTCAGCTTTGGAGAAGGTCACGATTCTCCGCAGGGGTAAGTCGTTGGATTCGGTGAAG
TP40010_Query	D+G	1	chr4	.	CAGCGGTTCAGCTGTGGAGAAGGTCACGATTCTCCGCAGGGGTAAGTCGTTGGATTCGGTGAAG
TP40057_Hit	D+G	1	chr4	.	CAGCGGTTGGAGTTTTGCCAGGAACCTCCGACTGTTCGTACCTTGGTGTTGATTGTGAGGTAGT
TP40057_Query	D+G	1	chr4	.	CAGCGGTTGGAGTTTTGCCAGGAACCTCCGACTGTTCGTACCTTGGTGTTGATTGTGAGATAGT
TP40298_Hit	D	1	chr4	.	CAGCGTAGTAAGGAAGATAAACTGCGTTAGCGGTTTTAGGATCTTCCGTTAAGCATGGATATTC
TP40298_Query	D	1	chr4	.	CAGCGTAGTAAGGAAGATAAACGGCGTTAGCGGTTTTAGGATCTTCCGTTAAGCATGGATATTC
TP40328_Hit	D+G	1	chr4	.	CAGCGTATTTGCGTTTTTAATTAATGATTATTATTTCAAACAACAAATAGATAATGTGGAGAGC
TP40328_Query	D+G	1	chr4	.	CAGCGTATATGCGTTTTTAATTAATGATTATTATTTCAAACAACAAATAGATAATGTGGAGAGC
TP40413_Hit	D+G	1	chr4	.	CAGCGTCGACATTGAGCTTGTAACAACATTCAACAGGGGCTGACCAGTGGACATTTGAATTGGA
TP40413_Query	D+G	1	chr4	.	CAGCGTCAACATTGAGCTTGTAACAACATTCAACAGGGGCTGACCAGTGGACATTTGAATTGGA
TP40657_Hit	D	1	chr4	.	CAGCGTCTCCATCTGTTTCATGTGATCATAAAGGTCATCAACAACCTATTGTGTCCAATGGAGT
TP40657_Query	D	1	chr4	.	CAGCGTCTCCATCTGTTTCACGTGATCATAAAGGTCATCAACAACCTATTGTGTCCAATGGAGT
TP4070_Hit	D	1	chr4	.	CAGCAACCCCAGAATATAGCACAATCAGAAAAGAAACTTGTGATTCCTTCCATAAATATAAATA
TP4070_Query	D	1	chr4	.	CAGCAACCCCAGAATATAGCACAATCAGAAAAGAAACTTGCGATTCCTTCCATAAATATAAATA
TP40730_Hit	D	1	chr4	.	CAGCGTCTTTCTCGACGTGAACAAATGAGCCTGATTGACTGCTATATTCCCCATTCTTTGTTTG
TP40730_Query	D	1	chr4	.	CAGCGTCTTTCTCGACGTGAACAAATGAGCCTGATTGACTGCTATATTCCCCATTCTTCGTTTG
TP40896_Hit	D	1	chr4	.	CAGCGTGCATTCAAATCTGACATGTTAAACTTATAAATTACATGAGATTCTCTTGCATTTTTTT
TP40896_Query	D	1	chr4	.	CAGCGTGCATTCAAATCTGACATGTGAAACTTATAAATTACATGAGATTCTCTTGCATTTTTTT
TP40921_Hit	D	1	chr4	.	CAGCGTGCCGTTTGATGGATGCATAGTTAATGATCATGACAACAATTGTGAGAGTAATAGGCAT
TP40921_Query	D	1	chr4	.	CAGCGTGCCGTTTGATGGATGCATAATTAATGATCATGACAACAATTGTGAGAGTAATAGGCAT
TP40960_Hit	D	1	chr4	.	CAGCGTGCTGAAAAGAGAGCCAGGGAAGAAAAGGGGCAAAAATTTGCACCAAGATGGTTTGAGT
TP40960_Query	D	1	chr4	.	CAGCGTGCTGAAAAGAGAGCCAGGGAAGAAAAGGGGCAAAAATTCGCACCAAGATGGTTTGAGT
TP41202_Hit	D	1	chr4	.	CAGCGTGTTTTTAATAGAATCAATGTTTGAGGTGTTTCAAGTCTAATATAAACAGATTACAAAT
TP41202_Query	D	1	chr4	.	CAGCGTGTTTTTAATAGAATCAATGTTTGAGCTGTTTCAAGTCTAATATAAACAGATTACAAAT
TP41214_Hit	D	1	chr4	.	CAGCGTTAAAGCTGACACTAATGCTGACAGTGATGAAGCTATCACAGACAGCCAAGATACTCAT
TP41214_Query	D	1	chr4	.	CAGCGTTAAAGCTGACACTAATGCTGACAGTGACGAAGCTATCACAGACAGCCAAGATACTCAT
TP4124_Hit	D+G	1	chr4	.	CAGCAACCGGATATTATGTGGTGGTTCCCGACTTCTTCAACGGCGATCCCTACGATCCTCAGAA
TP4124_Query	D+G	1	chr4	.	CAGCAACCGGATATTATGTGGTGGTTCCCGACTTCTTCAACGGCGATCCCTACGATCCCCAGAA
TP41268_Hit	D	1	chr4	.	CAGCGTTAGGAAGGGGTTCTGAATTTGGTTTCTGTCCATCACGAGGTAATTATACTGACCATTT
TP41268_Query	D	1	chr4	.	CAGCGTTAGGAAGGGGTTCTGAATTTGGTTTCTGTCCATCACAAGGTAATTATACTGACCATTT
TP4142_Hit	D	1	chr4	.	CAGCAACCGTTCCTGCCATAGGGACGACCAATCAAATAAAAAGTGTAAACCACAAAGAGGCAAG
TP4142_Query	D	1	chr4	.	CAGCAACCGTTCCTGCCATAGGGACAACCAATCAAATAAAAAGTGTAAACCACAAAGAGGCAAG
TP41454_Hit	D	1	chr4	.	CAGCGTTGGCCCTGTAATGATCTCGGTTCTCCATGAATATAGGTACAGCGGTTGGCAGGAGTTT
TP41454_Query	D	1	chr4	.	CAGCGTTGGCCCTGTAATGATCTCAGTTCTCCATGAATATAGGTACAGCGGTTGGCAGGAGTTT
TP41459_Hit	D	1	chr4	.	CAGCGTTGGCTTTATTGGTAGCTGATGACATTAGCACTTGTAGTCCAAACGTGCCGCCCGTGGC
TP41459_Query	D	1	chr4	.	CAGCGTTGGCTTTATTGGTAGCTGATGACATTAGCACTTGTAGTCCAAACGTGCCACCCGTGGC
TP41726_Hit	D+G	1	chr4	.	CAGCTTAAAATCAAACATTTTATAAACACACACATCAACTCATAGGTCGCAACATTTTAAATTC
TP41726_Query	D+G	1	chr4	.	CAGCTAAAAATCAAACATTTTATAAACACACACATCAACTCATAGGTCGCAACATTTTAAATTC
TP41810_Hit	D	1	chr4	.	CAGCTAAAATATCCGTCAGCACTTGCATCTTTTGATCAAATAACAAACTTCGCAAAAGGCAAAA
TP41810_Query	D	1	chr4	.	CAGCTAAAATATCCATCAGCACTTGCATCTTTTGATCAAATAACAAACTTCGCAAAAGGCAAAA
TP41856_Hit	D	1	chr4	.	CAGCTAAACAGCATGATTTGGAAAACATGTAACAATCATACTGAAAATCGGCATCTTTGCATGC
TP41856_Query	D	1	chr4	.	CAGCTAAACAGCATGATTTGGAAAACATGCAACAATCATACTGAAAATCGGCATCTTTGCATGC
TP41922_Hit	D	1	chr4	.	CAGCTAAAGATGAGCAAAATTCATTTCCAACGGCTACACAAGAGTTAACTAGGGTATTTTTCAT
TP41922_Query	D	1	chr4	.	CAGCTAAAGATGAGCAAAATTCATTTCCAACGGCTACACAAGAGTCAACTAGGGTATTTTTCAT

Name	Filter	Nb hit (Mt4.0)	Mt Chr (Mt4.0)	Ms Chr (Li et al., 2014)	Sequence
TP41982_Hit	D+G	1	chr4	.	CAGCTAAAGTATTTAAGGAAATGAAAGATTTAGTGGAAATAAACTCGGAATCTCTTGTGGTTCT
TP41982_Query	D+G	1	chr4	.	CAGCTAAAGTATTTAAGGAAATGAAAGATTTAGTGGAAATAAACTCAGAATCTCTTGTGGTTCT
TP41983_Hit	D	1	chr4	.	CAGCTTAAGTATTTTCATATAGAAAATTTTGTGTGGAACATGTGGTTTGGTAGTTATGTTTTTG
TP41983_Query	D	1	chr4	.	CAGCTAAAGTATTTTCATATAGAAAATTTTGTGTGGAACATGTGGTTTGGTAGTTATGTTTTTG
TP42006_Hit	D	1	chr4	.	CAGCTAAATACCAATTTTTCTCATAAATTATCAGCTTATAATATGTATTCAAAAAATAATATTG
TP42006_Query	D	1	chr4	.	CAGCTAAATACCAATTTTTCTCATAAATTATCAGCTTATAATATGTATTCAAAAAAGAATATTG
TP42133_Hit	D+G	1	chr4	.	CAGCTAACAACACAGGGCTGAAGAAAATGAACAGAGGGGTGGTGACAGCGAGGCCGACAACGGT
TP42133_Query	D+G	1	chr4	.	CAGCTAACAACACAGGACTGAAGAAAATGAACAGAGGGGTGGTGACAGCGAGGCCGACAACGGT
TP42164_Hit	D	1	chr4	.	CAGCTGACAGAATGAAAGGTTCAATTCTGCAACTGAGATCAGAGAGGGACTAGTAGCGTAAACT
TP42164_Query	D	1	chr4	.	CAGCTAACAGAATGAAAGGTTCAATTCTGCAACTGAGATCAGAGAGGGACTAGTAGCGTAAACT
TP42190_Hit	D	1	chr4	.	CAGCTAACATCCCCGTGCTCATTTTGTTTCAAAGCTTCTTGTACTTTGTAGTCTTCGTGAAACA
TP42190_Query	D	1	chr4	.	CAGCTAACATCCCCGTGCTCATTTTGTTTCAAAGCTTCTTGTACTTTGTAGTCTTCATGAAACA
TP422_Hit	D+G	1	chr4	.	CAGCAAAACCAGGCTAAACTTGTACATGTAAGTCTGAAAGTAAACCCTGACTATTTATTGTAGA
TP422_Query	D+G	1	chr4	.	CAGCAAAACCAGGCTAAACTTGTACATGTAAGTCTGAAAGTAAACCCTGACTATCTATTGTAGA
TP42311_Hit	D	1	chr4	.	CAGCTAACTTCATAAAAATGCTTGATCTTGCACCAGATGGGAAACAGGAAGTTTTGCTTCCTGA
TP42311_Query	D	1	chr4	.	CAGCTAACTTCATAAAAATGCTTGATCTTGCACCAGACGGGAAACAGGAAGTTTTGCTTCCTGA
TP42454_Hit	D	1	chr4	.	CAGCTAAGGAGAGGAAGTACGGTGGTTTCAATGGGGAGGTTTGAGTTAGAGGTTTTTTTGAAGG
TP42454_Query	D	1	chr4	.	CAGCTAAGGAGAGGAACTACGGTGGTTTCAATGGGGAGGTTTGAGTTAGAGGTTTTTTTGAAGG
TP42462_Hit	D	1	chr4	.	CAGCTAAGGATGTTGTGTTAGCTGAGAAGCCTGTGATAACTGATGACTCAAACAACCTCGATCC
TP42462_Query	D	1	chr4	.	CAGCTAAGGATGTCGTGTTAGCTGAGAAGCCTGTGATAACTGATGACTCAAACAACCTCGATCC
TP42476_Hit	D+G	1	chr4	.	CAGCTAAGGGCTCAGTGTAGGTCAAACACTGCAAAACTGAATTCAGAAAACAAGTGTTTCCAAG
TP42476_Query	D+G	1	chr4	.	CAGCTAAGGGCTCAGTGTACGTCAAACACTGCAAAACTGAATTCAGAAAACAAGTGTTTCCAAG
TP42480_Hit	D	1	chr4	.	CAGCTAAGGTAATAAATTGAAGAGATGGTAGAAATTATAGATAAAATGATAAAAATTGGGAAGA
TP42480_Query	D	1	chr4	.	CAGCTAAGGTAATAAACTGAAGAGATGGTAGAAATTATAGATAAAATGATAAAAATTGGGAAGA
TP42765_Hit	D	1	chr4	.	CAGCTAATTACCTGTATTGATTCCCTAGTGAGCTAGCAGAAGATAGGCCAACAATTTTACAGCG
TP42765_Query	D	1	chr4	.	CAGCTAATTACCTGTATTGATTCCCTAGTGAGGTAGCAGAAGATAGGCCAACAATTTTACAGCG
TP4278_Hit	D	1	chr4	.	CAGCAACGAAATTTTGAGGCATCAATTCCAGTTACTGAGAAAATTTACTGTCCTTATCCAAGAT
TP4278_Query	D	1	chr4	.	CAGCAACGAAATTTGGAGGCATCAATTCCAGTTACTGAGAAAATTTACTGTCCTTATCCAAGAT
TP42789_Hit	D	1	chr4	.	CAGCTAATTCCATGAGTTCCAGACGTTCATCTTCAATAAGGTCCATTGCGTCTTTGACCATTCT
TP42789_Query	D	1	chr4	.	CAGCTAATTCCATGAGTTCCAGACGTTCATCTTCAATAAGGTCCATTGCATCTTTGACCATTCT
TP42988_Hit	D	1	chr4	.	CAGCTACAATCCCAATTATCAATATCAACGATTTCAACAGATTGTCCACAGACTTCAAATGTGG
TP42988_Query	D	1	chr4	.	CAGCTACAATCCCAATTATCAATATCAACAATTTCAACAGATTGTCCACAGACTTCAAATGTGG
TP43028_Hit	D	1	chr4	.	CAGCTACACATTACCTTAATTGTCAATATCTCCAAGGGAGATGGTGATGTGCTGGAAATCATGT
TP43028_Query	D	1	chr4	.	CAGCTACACATTACCTTAATTGTCAATATCTCCAAGGGAGATGGTCATGTGCTGGAAATCATGT
TP43044_Hit	D+G	1	chr4	.	CAGCTACACCTTTGCTCATATGTCCAAGTGCACTACTTGCATTACCCAATATATCGACACCTGG
TP43044_Query	D+G	1	chr4	.	CAGCTACACCTTTGCTCATATGTCCAAGTGCACTACTTGCATTACCCAATATATCGACACCTGA
TP43063_Hit	D	1	chr4	.	CAGCTACACTTCCATATATCAAACCATAAGAATGCTCCATTTTTATTGGATTCAAAGAAAAGCC
TP43063_Query	D	1	chr4	.	CAGCTACACTTCCATATATCAAACCATAAGAATGCTCCATTTTTATTGGATTCAAAGAAAAACC
TP43170_Hit	D+G	1	chr4	.	CAGCTACATCAGTAGCATAAAATATTCCAGGAATGGTCCAATGCCTTGCAGTTTTGTGGCTTAA
TP43170_Query	D+G	1	chr4	.	CAGCTACATCAGTAGCATAAAATATTCCAGGAGTGGTCCAATGCCTTGCAGTTTTGTGGCTTAA
TP43328_Hit	D+G	1	chr4	.	CAGCTACTGTGAGTATGTCCAAAGTTGCAGTTTCATTTACACACATAATCAAAAGTGGAGAACC
TP43328_Query	D+G	1	chr4	.	CAGCTACCGTGAGTATGTCCAAAGTTGCAGTTTCATTTACACACATAATCAAAAGTGGAGAACC
TP43342_Hit	D+G	1	chr4	.	CAGCTACCTCTACTTATATGGAGTATCTTGAGCCAGCTTCTAAGTCTACTTCTTTGCATGTTAT
TP43342_Query	D+G	1	chr4	.	CAGCTACCTCTACTTATATGGAGTATCTTGAGCCAGCTTCTAAGTCTACTTCTTTGCATGTCAT
TP43430_Hit	D+G	1	chr4	.	CAGCTACGGAAGAATTGTCTATGTGCCACTGTGTATAGTGTGTCTTGGAATCCTTCAAAACGGC
TP43430_Query	D+G	1	chr4	.	CAGCTACGGAAGAATTGTCTATGTGCCACTGTGTATAGTGTGTCTTGGAAGCCTTCAAAACGGC
TP43443_Hit	D	1	chr4	.	CAGCTACGGTCTCTTAACGATCTTGACTTGGAGCTCTATGAATATGCGAGAGCCATTTTCAATA
TP43443_Query	D	1	chr4	.	CAGCTACGGTCTCTTAACGATCTTGACTTGGAGCTCTATGAATATGCGAGAGACATTTTCAATA
TP43570_Hit	D	1	chr4	.	CAGCTACTTTGACAATAGAGCTATGCTTGGCACATACTCATAAATCCTAAAATTTGAAATTTTA
TP43570_Query	D	1	chr4	.	CAGCTACTCTGACAATAGAGCTATGCTTGGCACATACTCATAAATCCTAAAATTTGAAATTTTA
TP43621_Hit	D	1	chr4	.	CAGCTACTGGATGATTTTTCTGTGACCTTTTTTGATGAATTTTCTCTCTCCTCTACAAGGATCT
TP43621_Query	D	1	chr4	.	CAGCTACTGGATGATTTTTCTATGACCTTTTTTGATGAATTTTCTCTCTCCTCTACAAGGATCT
TP43669_Hit	D	1	chr4	.	CAGCTACTTGGTGTTCCAATTTTAATGTGACAGGGGAGAGTGGTAGTAAAGGTGGGAGTATCGG
TP43669_Query	D	1	chr4	.	CAGCTACTTGGTGTTCCAATTTTAATGTGACAGGGGAGAGTGGTAATAAAGGTGGGAGTATCGG

Name	Filter	Nb hit (Mt4.0)	Mt Chr (Mt4.0)	Ms Chr (Li et al., 2014)	Sequence
TP43709_Hit	D	1	chr4	.	CAGCTAGAAAATAAAATATATGACAAACAAAATAAAGAGGAAATTATTCAAGTGACAATACATC
TP43709_Query	D	1	chr4	.	CAGCTAGAAAATAAAATATATGACAAACAAAATAAAGAGGAAATTATTCAAATGACAATACATC
TP43879_Hit	D+G	1	chr4	.	CAGCTAGAGTCCAAGCAGACCGTGCTGTGTTTGCCAACTGCCGTTTTGAAGGCTTCCAAGACAC
TP43879_Query	D+G	1	chr4	.	CAGCTAGAGTCCAAGCAGACCGTGCTGTGTTTGCCAACTGCCGTTTTGAAGGATTCCAAGACAC
TP43898_Hit	D	1	chr4	.	CAGCTAGATAAGGACACACACAAGATGGTACAACACATCTATTCTTTGTTGCTTGAAAGGGCCA
TP43898_Query	D	1	chr4	.	CAGCTAGATAAGGACACACACAAGATAGTACAACACATCTATTCTTTGTTGCTTGAAAGGGCCA
TP44018_Hit	D	1	chr4	.	CAGCTAGCATCACTTAATATTCAAATCTCATCTTCATAAGCTTAATTTCTTTTTACTTATTGTT
TP44018_Query	D	1	chr4	.	CAGCTAGCATCACTTAATATTCAAATCTCATCTTCAGAAGCTTAATTTCTTTTTACTTATTGTT
TP44117_Hit	D	1	chr4	.	CAGCTAGGAAACACCAGAATTCCTTATATTAGTTCCCACATACCTTTCACAACCCATAGTGGAC
TP44117_Query	D	1	chr4	.	CAGCTAGGAAACACCAGAATTCCTTATATTAGTTCCCACATACCTTTCACAACCCATAGCGGAC
TP44146_Hit	D	1	chr4	.	CAGCTAGGAGTTGGAGGCCATCAAAGTCCCCAAATGTCAACCGTTAGCTCCTTTACTGCATTGT
TP44146_Query	D	1	chr4	.	CAGCTAGGAGTTGGAGGCCATCAAAGTCCCCAAATGTCAACCGTTAGCTCCTTTACTGCATTGA
TP44167_Hit	D+G	1	chr4	.	CAGCTAGGCGGGAAGAGAATGAAAGAAAAGGTTTCAATTCTTCCTCGGCAAGCATCTCCTTTGT
TP44167_Query	D+G	1	chr4	.	CAGCTAGGCGGGAAGAGAATGAAAGAAAAGGTTTCAATTCTTCCTCGGCAAGCATCTCCTTCGT
TP44224_Hit	D	1	chr4	.	CAGCTAGGTACTCTTACTAGTGATATTGCTGGGCAGTTGAAAGAGCTGAGACAGGGTCGCATTG
TP44224_Query	D	1	chr4	.	CAGCTAGGTACTCTTACTAGTGATATTGCTGGGCAGTTGAAAGAGCTAAGACAGGGTCGCATTG
TP44298_Hit	D	1	chr4	.	CAGCTAGTATATATAAACAGATGATGTACAATCATGGATGGGTTGATTAGTTAACTCATTATAT
TP44298_Query	D	1	chr4	.	CAGCTAGTATATATAAAAAGATGATGTACAATCATGGATGGGTTGATTAGTTAACTCATTATAT
TP44318_Hit	D	1	chr4	.	CAGCTAGTCATCTGGCTTCTGATTCTCAGCATTATGATAATAATGAAAGGAATAAGAAAGGCCA
TP44318_Query	D	1	chr4	.	CAGCTAGTCATCTGGCTTCTGATTCTAAGCATTATGATAATAATGAAAGGAATAAGAAAGGCCA
TP44335_Hit	D	1	chr4	.	CAGCTAGTGAATAGCGTGATATTGTGCTGATATCCTCCTTGGAGGCAATTTGCGCAGGATGTAG
TP44335_Query	D	1	chr4	.	CAGCTAGTGAATAGCGTGATATTGTGCTGACATCCTCCTTGGAGGCAATTTGCGCAGGATGTAG
TP44433_Hit	D	1	chr4	.	CAGCTAGTTGTGTAGGTTATTGTGGTGCTGACCTAAAGGCTCTTTGTACTGAAGCGGCTATTCG
TP44433_Query	D	1	chr4	.	CAGCTAGTTGTGTAGGTTATTGTGGTGCTGACCTAAAGGCTCTCTGTACTGAAGCGGCTATTCG
TP44587_Hit	D+G	1	chr4	.	CTGCTATACCCTTCATTGTCAGAATAAGGGTATCCTCCAAACCAGGAGCATAGTCACTCGTAGG
TP44587_Query	D+G	1	chr4	.	CAGCTATACCCTTCATTGTCAGAATAAGGGTATCCTCCAAACCAGGAGCATAGTCACTCGTAGG
TP44608_Hit	D	1	chr4	.	CAGCTATACTGTCAGTGGTTCCGGTGCATACAAGACACTCCTTTTGAAAACCTGAAAGGATATG
TP44608_Query	D	1	chr4	.	CAGCTATACTGTCAGTGGTTCCGGTGCATACAACACACTCCTTTTGAAAACCTGAAAGGATATG
TP44686_Hit	D	1	chr4	.	CAGCTATAGGTAACTGAAGTTCAGGTACATTGTCCTTCCCTAAGGGCAATTGCAAAGATGTTCC
TP44686_Query	D	1	chr4	.	CAGCTATAGGTAACTGAAGCTCAGGTACATTGTCCTTCCCTAAGGGCAATTGCAAAGATGTTCC
TP44748_Hit	D	1	chr4	.	CAGCTATATCAAAGGCGACTTCCGGAGGAGCAAGAGCAGGCATCTCCACAAAGTGAAGGTTCAT
TP44748_Query	D	1	chr4	.	CAGCTATATCAAAGGCGACTTCAGGAGGAGCAAGAGCAGGCATCTCCACAAAGTGAAGGTTCAT
TP44750_Hit	D	1	chr4	.	CAGCTATGTCAACAGAAAGTACTATATGGAAACAGTCGTGTGTTGAAATAATAAGCCGGCACTA
TP44750_Query	D	1	chr4	.	CAGCTATATCAACAGAAAGTACTATATGGAAACAGTCGTGTGTTGAAATAATAAGCCGGCACTA
TP44908_Hit	D	1	chr4	.	CAGCTATCATTTTTATTTTTTTCACCCCTTTATTCTATTGATTCCTAACTTGTTACAATTACTT
TP44908_Query	D	1	chr4	.	CAGCTATCATTTTTATTTTTTTCACCCCTTTATTCTATTGATTCCTAACTTGTTACAATTACAT
TP4492_Hit	D	1	chr4	.	CAGCAACTAATAATGTGTATCGACAAAGGGCTTGCACAAATCACCTGTGATTCAAGACTTCCAT
TP4492_Query	D	1	chr4	.	CAGCAACTAATAATGTGTATAGACAAAGGGCTTGCACAAATCACCTGTGATTCAAGACTTCCAT
TP45061_Hit	D+G	1	chr4	.	CAGCTATGAGTCTGAGTTTATTGAGAAGATTGTTAGAGACATCTCAGCTAAACTACCTCCCATG
TP45061_Query	D+G	1	chr4	.	CAGCTATGAGTCTGAGTTTATTGAGAAGATTGTGAGAGACATCTCAGCTAAACTACCTCCCATG
TP45110_Hit	D	1	chr4	.	CAGCTATGCAGATGGTGGTTTTCTAGGAGAGAAATTGCGCTTTGATGAATTATCCCTTTTTCAA
TP45110_Query	D	1	chr4	.	CAGCTATGCAGATGGTAGTTTTCTAGGAGAGAAATTGCGCTTTGATGAATTATCCCTTTTTCAA
TP45162_Hit	D+G	1	chr4	.	CAGCTATGGACATATCTTCAGATATCACCGAGCTTGGAAGAACACCAGTAGCTGTTATATGTGC
TP45162_Query	D+G	1	chr4	.	CAGCTATGGACATATCTTCAGATATCACCGAGCTTGGAAGAACACCAGTAGCAGTTATATGTGC
TP45176_Hit	D	1	chr4	.	CAGCTATGGATGGTACTCATGACTGGTATATTGGGCTACCTAATGATGATCCCAAGAAGTGTAA
TP45176_Query	D	1	chr4	.	CAGCTATGGATGGTACTCATGACTGGTACATTGGGCTACCTAATGATGATCCCAAGAAGTGTAA
TP45224_Hit	D+G	1	chr4	.	CAGCTATGGTTTGCACTTGTTATTGCTACCAACAGTTGTGCATGGTTAACAACTGCTATTCTTG
TP45224_Query	D+G	1	chr4	.	CAGCTATGGTTTGCACTTGCTATTGCTACCAACAGTTGTGCATGGTTAACAACTGCTATTCTTG
TP45234_Hit	D	1	chr4	.	CAGCTATGTATGCAAAACATCCGTACGGTTCTCTTGGCAAAAAGGGAATGGTGGCACTTGTAGT
TP45234_Query	D	1	chr4	.	CAGCTATGTATGCAAAACATCCGTACGGTTCTCTTGGCAAAAACGGAATGGTGGCACTTGTAGT
TP45328_Hit	D	1	chr4	.	CAGCTATTAATGGGAACAAGGCCAAGATTCATGAGTGTTCCATTTGTGGTTCAGAGTTCACGTC
TP45328_Query	D	1	chr4	.	CAGCTATTAATGGGAACAAGGCCAAGATTCATGAGTGTTCCATTTGTGGTTCAGAGTTCACATC
TP45370_Hit	D	1	chr4	.	CAGCTATTATAGAAAACTTATGAAAATTAACTAAAAACCGCTTTTGGAGATGTCATAAGTTGTT
TP45370_Query	D	1	chr4	.	CAGCTATTATAGAAAACTTATGAAAATTAACTAAAAACCGCTTTTGGAAATGTCATAAGTTGTT

Name	Filter	Nb hit (Mt4.0)	Mt Chr (Mt4.0)	Ms Chr (Li et al., 2014)	Sequence
TP45471_Hit	D	1	chr4	.	CAGCTATTGCCGTTTAGGCATGTGATGCTGTTGAGAGAATGTTATGCAGGAGTGAGGGTAGACT
TP45471_Query	D	1	chr4	.	CAGCTATTGCCGTTTAGGCATGTGATGCGGTTGAGAGAATGTTATGCAGGAGTGAGGGTAGACT
TP45625_Hit	D	1	chr4	.	CAGCTATTTTATGCAGAGTACTTCCTCATACCTTTAGTTAGATGAGAATGCAGAAAAAAAAAAA
TP45625_Query	D	1	chr4	.	CAGCTATTTTACGCAGAGTACTTCCTCATACCTTTAGTTAGATGAGAATGCAGAAAAAAAAAAA
TP45638_Hit	D	1	chr4	.	CAGCTATTTTCTTTTAGTGCCATTCTGACTGTGTTCTTCTGTGGCATTCTTATGTCACATTATA
TP45638_Query	D	1	chr4	.	CAGCTATTTTCTTTTAGTGCCATTCTGACTGTGTTCTTCTGCGGCATTCTTATGTCACATTATA
TP45661_Hit	D	1	chr4	.	CAGCTATTTTTTTTAAGTTGTTTTTGGTTTAAAAGTTAATTTTGATAAAATTATGTTGACTTAG
TP45661_Query	D	1	chr4	.	CAGCTATTTTTTTTAAGTTGTTTTTGGTTTAAAAGTTAATTTTGATAAAAGTATGTTGACTTAG
TP45701_Hit	D	1	chr4	.	CAGCTCAAAATGAAATAGGCGGCAATTCAAATCACATTACCTACAAATATTTTGGTGCAATTTG
TP45701_Query	D	1	chr4	.	CAGCTCAAAATAAAATAGGCGGCAATTCAAATCACATTACCTACAAATATTTTGGTGCAATTTG
TP45811_Hit	D+G	1	chr4	.	CAGCTCATATTCCCTTGTGAATCCCATTCTTAAAATTGAGTTAAACCAATATACTTAGAGGGAG
TP45811_Query	D+G	1	chr4	.	CAGCTCAAATTCCCTTGTGAATCCCATTCTTAAAATTGAGTTAAACCAATATACTTAGAGGGAG
TP45879_Hit	D	1	chr4	.	CAGCTCAAGCTCTCTGTCAGAGATAATATCCTCCTCCCTAAAAGACAAAATTATCTCGTTCCAT
TP45879_Query	D	1	chr4	.	CAGCTCAACCTCTCTGTCAGAGATAATATCCTCCTCCCTAAAAGACAAAATTATCTCGTTCCAT
TP45892_Hit	D+G	1	chr4	.	CAGCTCAACGTCACGTTCCATCTCAAACCCTTACACAAGAGGTGATATATAGATGACATGTTTA
TP45892_Query	D+G	1	chr4	.	CAGCTCAACGTCACGTTCCATCTCAAACCCTTGCACAAGAGGTGATATATAGATGACATGTTTA
TP46057_Hit	D+G	1	chr4	.	CAGCTCAATCGGGAGCACAGAAGGGTGATGAAGTGAAACGTGTTGTGGAAATATTGATGAGGAC
TP46057_Query	D+G	1	chr4	.	CAGCTCAATCGGGAGCACAGAAGGGTGATGAAGTGAAACGTGTTGTAGAAATATTGATGAGGAC
TP46103_Hit	D	1	chr4	.	CAGCTCAATTGTCTAGTCGGTACATAACCGGTATGTTCTTTATATTCTCTCTTCATGTTTTTGT
TP46103_Query	D	1	chr4	.	CAGCTCAATTGTCTAGTCGGTACATAACCGGTATGTTCTTTATATTCTCTCTTCATGTTTGTGT
TP46330_Hit	D+G	1	chr4	.	CAGCTCAGAAACTCAAAATTGGTTTGTATTTTGCAACATGGTGGGCTTTGAATGTGGTTTTCAA
TP46330_Query	D+G	1	chr4	.	CAGCTCAGAAACTCAAAATTGGATTGTATTTTGCAACATGGTGGGCTTTGAATGTGGTTTTCAA
TP46528_Hit	D	1	chr4	.	CAGCTCAGGTAGGTTGCACATGAGCAGACAAGCTAAATGTGATGGTGATTGGTGGATAGAAGAT
TP46528_Query	D	1	chr4	.	CAGCTCAGGTAGGTTGCACATGAGCAGACAAGCTAAATGTGATGGCGATTGGTGGATAGAAGAT
TP46570_Hit	D+G	1	chr4	.	CAGCTCAGTGCAGAGCCATCACATCTGTGTTTATAATTTCTCTCTCAGTTCTAATGGCTTCACA
TP46570_Query	D+G	1	chr4	.	CAGCTCAGTGCAGAGCCATCACATCTGTGTTTATAATTTCTCTCTCAGGTCTAATGGCTTCACA
TP46622_Hit	D+G	1	chr4	.	CAGCTCATAATGCTCAAGAAGATTCGGGTTCTCTTTTGTTTAATCTTATTGGGAATCTTGCTTT
TP46622_Query	D+G	1	chr4	.	CAGCTCATAATGCTCAAGAAGATTCGGGTTCTCTTTTGTTTAATCTCATTGGGAATCTTGCTTT
TP46746_Hit	D+G	1	chr4	.	CAGCTCATCTTTGTATAGATTTTGGATAAGTTTTAACAATGCAAGATAATGACTTAGTGCCCAT
TP46746_Query	D+G	1	chr4	.	CAGCTCATCTTTGTATAGATTTTGGATAAGTTTTAACAATGCAAGATAATGACTTAGTGCACAT
TP46750_Hit	D	1	chr4	.	CAGCTCATGAAAGCATACGTGGAGGCCATATTGCGTTGAATTCAATTCAACGTCGATGTGCAAG
TP46750_Query	D	1	chr4	.	CAGCTCATGAAAGCATACGCGGAGGCCATATTGCGTTGAATTCAATTCAACGTCGATGTGCAAG
TP46793_Hit	D	1	chr4	.	CAGCTCATGCTCTACAACAACTCTCTTGAAGGTAACCTTCCTCACCAATTGGTAAATGTAGCAA
TP46793_Query	D	1	chr4	.	CAGCTCATGCTCTACAACAACTCTCTTGAAGGTAACCTTCCTCACCAATTGATAAATGTAGCAA
TP46891_Hit	D	1	chr4	.	CAGCTCATTGCTTACTTGGAGACGTGATTCTTATCCTTTACGAAACTATGGGTACTACCTGGTA
TP46891_Query	D	1	chr4	.	CAGCTCATTGCTTACTTGGAGACGTGATTCTTATCCTTTACGAAACTATGAGTACTACCTGGTA
TP46914_Hit	D+G	1	chr4	.	CAGCTCATTTGCTTGTTGTGTCGATGTTGATATGTCTGCTTTCAGAATCAAAAGATGATATGAT
TP46914_Query	D+G	1	chr4	.	CAGCTCATTTGCTTGTTGTGTCGATGTTGATATGTCTGCTTTCAGAATCAAAAGATGATATAAT
TP47040_Hit	D	1	chr4	.	CAGCTCCAATCATTGGACCAACCCAGAATATCCACTGTTCATCACAAAAACAATTCAACAAAAG
TP47040_Query	D	1	chr4	.	CAGCTCCAATCATTGGACCAACCCAGAAAATCCACTGTTCATCACAAAAACAATTCAACAAAAG
TP47103_Hit	D+G	1	chr4	.	CAGCTCCACCAGCTTCTCCAGGATAGCCAACCCACAGAATGCTTGTGATCTTTTTATTAGTTTT
TP47103_Query	D+G	1	chr4	.	CAGCTCCACCAGCTTCTCCAGGATAGCCAACCCACAGAATGCCTGTGATCTTTTTATTAGTTTT
TP47161_Hit	D	1	chr4	.	CAGCTCCAGAAGAATGGGCTTTTCACGATAATTTTGAGCAACAATATGGCAACAAACATCCCTT
TP47161_Query	D	1	chr4	.	CAGCTCCAGAAGAATGGGCTTTTCACGATAATTTTGAGCAACAATATGACAACAAACATCCCTT
TP47176_Hit	D	1	chr4	.	CAGCTCCAGATTCACGAGAAGAGAAGGCGATGCTTTTGGTGAAATGGCTCAACTTGGAGGCTTT
TP47176_Query	D	1	chr4	.	CAGCTCCAGATTCAAGAGAAGAGAAGGCGATGCTTTTGGTGAAATGGCTCAACTTGGAGGCTTT
TP47178_Hit	D	1	chr4	.	CAGCTCCAGATTTCTTCTTTGCTTTCTTCTCGCGATCCTTAAGTTTCTTCTCAGCTTCCTTCTG
TP47178_Query	D	1	chr4	.	CAGCTCCAGATTTCTTCTTTGCCTTCTTCTCGCGATCCTTAAGTTTCTTCTCAGCTTCCTTCTG
TP47202_Hit	D	1	chr4	.	CAGCTCCAGGAATTTCGGTCATGATGGACAGTCTCAGGGTAGTACGCCGTCCCAACCTCATAAG
TP47202_Query	D	1	chr4	.	CAGCTCCAGGAATTTCGGTCATGATGGACAGTCTCAGGGTACTACGCCGTCCCAACCTCATAAG
TP47209_Hit	D+G	1	chr4	.	CAGCTCCAGGGGATGCAGGCAAGCCAGTGGCAACCACTTTGTCCTTGTACTTAGATGGATCCTC
TP47209_Query	D+G	1	chr4	.	CAGCTCCAGGGGAAGCAGGCAAGCCAGTGGCAACCACTTTGTCCTTGTACTTAGATGGATCCTC
TP4764_Hit	D	1	chr4	.	CAGCAACTGGCGCTCCACCCTTTTCGGGATGGATGATTCTTGAAGGAGTGGATAAGGGAGAAGG
TP4764_Query	D	1	chr4	.	CAGCAACTGGCGCTCCACCCTTTACGGGATGGATGATTCTTGAAGGAGTGGATAAGGGAGAAGG

Name	Filter	Nb hit (Mt4.0)	Mt Chr (Mt4.0)	Ms Chr (Li et al., 2014)	Sequence
TP47721_Hit	D	1	chr4	.	CAGCTCCTATGGTTGATATGCTTCTTCATGACTTGGTATTATATACAGCGAGTTTTCTGTTGAT
TP47721_Query	D	1	chr4	.	CAGCTCCTATGGTTGATATGCTTCTTCACGACTTGGTATTATATACAGCGAGTTTTCTGTTGAT
TP47786_Hit	D	1	chr4	.	CAGCTCCTCCTCCGATTGGAAGTGTGTTGCTTTCGAGAATGGCTTTCACGTCATAACGAGTCAT
TP47786_Query	D	1	chr4	.	CAGCTCCTCCTCCAATTGGAAGTGTGTTGCTTTCGAGAATGGCTTTCACGTCATAACGAGTCAT
TP47858_Hit	D+G	1	chr4	.	CAGCTCCTGATAACAACATTCGAACTCGTCCACCCAATGCTTGTTTTATCTATCAGTAAAATCA
TP47858_Query	D+G	1	chr4	.	CAGCTCCTGATAACAACATTCGAACCCGTCCACCCAATGCTTGTTTTATCTATCAGTAAAATCA
TP4799_Hit	D+G	1	chr4	.	CAGCAACTGTGCAATTAATCTCCTTAGCTAGAGACCCCATCACATGCCTCCAAGAGAAAACTCC
TP4799_Query	D+G	1	chr4	.	CAGCAACTGTGCAATTAATCTCCCTAGCTAGAGACCCCATCACATGCCTCCAAGAGAAAACTCC
TP48006_Hit	D+G	1	chr4	.	CAGCTCCTTTTCCAATTAACCACGACGATGAGCAAGAAACCAAAGCAGAACCTGAACCCCATTT
TP48006_Query	D+G	1	chr4	.	CAGCTCCTTTTCCAATTAACCACGACGATGAGCAAGAAACCAAAGCAGAACCTGAACCCCACTT
TP4813_Hit	D	1	chr4	.	CAGCAACTGTTGTTTTCAGTGCTAGTCCATGAACACGTGGTAAAATCACTTGTTTCACCAGCTG
TP4813_Query	D	1	chr4	.	CAGCAACTGTTGTTTTCAGTGCTAGTCCATGAACACGTGGTAAAATAACTTGTTTCACCAGCTG
TP48231_Hit	D	1	chr4	.	CAGCTCGCCAGGCAACACATCTGCTTCTCTTTGCAAAGTCTGAATTTTCAGGATATAAAATACC
TP48231_Query	D	1	chr4	.	CAGCTCGCCAGGCAACACATCTGCTTCTCTTTGCAAAATCTGAATTTTCAGGATATAAAATACC
TP48389_Hit	D	1	chr4	.	CAGCTCGGTACTGTATTTCTACACGTCTTAGGACAAATCTTGGTGGGCCTACCCAAACATTTGC
TP48389_Query	D	1	chr4	.	CAGCTCGGTACTGTATTTCTACACATCTTAGGACAAATCTTGGTGGGCCTACCCAAACATTTGC
TP48431_Hit	D	1	chr4	.	CAGCTCGTAGAACTACGACTCAAAGGAAACCGGTGAAAGAAGCTGAAAGCTCTAAGGTTTCTAC
TP48431_Query	D	1	chr4	.	CAGCTCGTAGAACTACGACTCAAAGGAAACCGGTGAAAGAAGCTGAAAGCTCTAAGGTTTCGAC
TP48590_Hit	D	1	chr4	.	CAGCTCTAACTGGCAACTGGCCAAAAGTGGTACACCTGGTAATATTGGCAGTTTTGAAGATGTT
TP48590_Query	D	1	chr4	.	CAGCTCTAACTGGCAACTGGCCAAAAGCGGTACACCTGGTAATATTGGCAGTTTTGAAGATGTT
TP48610_Hit	D	1	chr4	.	CAGCTCTAATTGAGAAAAAAATTATTTGCATGCATGGGGGTATTGGCCGGTCAATAAATCACTT
TP48610_Query	D	1	chr4	.	CAGCTCTAATTGAGAAAAAAATTATTTGCATGCATGGGGGTATTGGCCGGTCAATAAATCACCT
TP48624_Hit	D	1	chr4	.	CAGCTCTACATAATGCTTCTTTAATGAGAAGTTGTGCAGGGTCTATATCTGGTGATAAAGTGAT
TP48624_Query	D	1	chr4	.	CAGCTCTACATAATGCTTCTTTAATGAGAAGTTGTGCAGGGTCTATATCCGGTGATAAAGTGAT
TP48719_Hit	D	1	chr4	.	CAGCTCTATTTTCAAACAAAAACAACATAAGAGTGTACCAAATAACACTTTGCAGAACAACAAT
TP48719_Query	D	1	chr4	.	CAGCTCTATATTCAAACAAAAACAACATAAGAGTGTACCAAATAACACTTTGCAGAACAACAAT
TP48996_Hit	D+G	1	chr4	.	CAGCTCTCTCTTCATGTGAAACTTTGACAATCATATGATTAAATTGGTTTGTTTCTTAATCAAG
TP48996_Query	D+G	1	chr4	.	CAGCTCTCTCTTCATGTGAAACTTTGACAATCATATGATTAAATTGGTCTGTTTCTTAATCAAG
TP4901_Hit	D	1	chr4	.	CAGCAACTTGATCACTGGACTCCTCTTCCAGTAATTCTTTTCCAACCGACATTGGTTTAGTGTT
TP4901_Query	D	1	chr4	.	CAGCAACTTGATCACCGGACTCCTCTTCCAGTAATTCTTTTCCAACCGACATTGGTTTAGTGTT
TP49061_Hit	D+G	1	chr4	.	CAGCTCTGAGGAGAATGGTTGAAACCATGTCTAGCTTCTGGGGTCCAGTAACATCTACCAAAGG
TP49061_Query	D+G	1	chr4	.	CAGCTCTGAGGAGAATGGTAGAAACCATGTCTAGCTTCTGGGGTCCAGTAACATCTACCAAAGG
TP49157_Hit	D+G	1	chr4	.	CAGCTCTGGATATTCTTCTCCATAAGACTCCCACCAAGCTCCTGGAGTCTTTTTTCCAAGTGCC
TP49157_Query	D+G	1	chr4	.	CAGCTCTGGATATTCTTCTCCATAAGACTCCCACCAAGCTCCTGGAGTCTGTTTTCCAAGTGCC
TP49286_Hit	D+G	1	chr4	.	CAGCTCTTACATCAGAAGGGTTGAAGAAGACGCTGTTGCGAGGACCGGTGCTGAAAAAAAAAAA
TP49286_Query	D+G	1	chr4	.	CAGCTCTTACATCAGAAGGCTTGAAGAAGACGCTGTTGCGAGGACCGGTGCTGAAAAAAAAAAA
TP49304_Hit	D+G	1	chr4	.	CAGCTCTTAGTAGAAAATCTCGATTTGATGTTTTCTGGGATGATGAGATGCTTGGAAGCGGTGA
TP49304_Query	D+G	1	chr4	.	CAGCTCTTAGTAGAAAATCTCGATTTGATGTTTTCTGGGATGATGAGAAGCTTGGAAGCGGTGA
TP49357_Hit	D+G	1	chr4	.	CAGCTCTTTGAGGCTATGTAGCACCAACACTTCAAATGAAAACGTGATATACTTAAACTATTCT
TP49357_Query	D+G	1	chr4	.	CAGCTCTTCGAGGCTATGTAGCACCAACACTTCAAATGAAAACGTGATATACTTAAACTATTCT
TP49464_Hit	D+G	1	chr4	.	CAGCTCTTGGAATTCTAAGGTAATATCACTCATTTATTCTCGTAGCATTTTGAATTACTTTTAA
TP49464_Query	D+G	1	chr4	.	CAGCTCTTGGAATTCTAAGGTAATATCACTCAGTTATTCTCGTAGCATTTTGAATTACTTTTAA
TP49473_Hit	D	1	chr4	.	CAGCTCTTGGCACTGCTGAACCACATGGTGCCAATTATAGTCCAAGCCCAAAGAAATGGATAAA
TP49473_Query	D	1	chr4	.	CAGCTCTTGGCACTGCTGAACCACATGGTACCAATTATAGTCCAAGCCCAAAGAAATGGATAAA
TP49552_Hit	D+G	1	chr4	.	CAGCTCTTTCTCCAGGACCAAACGAAATAGACTCGGACTTTGGAGATTCCTGCACGTCAACTCC
TP49552_Query	D+G	1	chr4	.	CAGCTCTTTCTCCAGGACCAAACGAAATAGACTCGGACTTTGGAGATTCCTGCACGTCAACCCC
TP49666_Hit	D	1	chr4	.	CAGCTGAACAATGAACAAGAAAAGTAAAAACCTTTGTTTCTCCGAAGTAAGTTGTTAACTTGTT
TP49666_Query	D	1	chr4	.	CAGCTGAAAAATGAACAAGAAAAGTAAAAACCTTTGTTTCTCCGAAGTAAGTTGTTAACTTGTT
TP49686_Hit	D+G	1	chr4	.	CAGCTGAAAAGTATGGATTTAATGGAGATGGTGCACTAACTATTCCTTGTAGCCCTAATAAACT
TP49686_Query	D+G	1	chr4	.	CAGCTGAAAAGTATGGACTTAATGGAGATGGTGCACTAACTATTCCTTGTAGCCCTAATAAACT
TP49900_Hit	D	1	chr4	.	CAGCTGAACAGATGCTTCGCTTGCATCCAAATTCTGCTGGAGCTCACATCACTCTGGCTAACAT
TP49900_Query	D	1	chr4	.	CAGCTGAACAGATGCTTCGCTTGCATCCAAACTCTGCTGGAGCTCACATCACTCTGGCTAACAT
TP49941_Hit	D	1	chr4	.	CAGCTGAACTAACTGCTGGTTACTATAATACTAGACATAATGATGGATATCTTCCAATTGCCAA
TP49941_Query	D	1	chr4	.	CAGCTGAACTAACTGCTGGTTACTATAATACTAGAAATAATGATGGATATCTTCCAATTGCCAA

Name	Filter	Nb hit (Mt4.0)	Mt Chr (Mt4.0)	Ms Chr (Li et al., 2014)	Sequence
TP49960_Hit	D	1	chr4	.	CAGCTGAACTTTTGGCTTTACTAGCTCCTGGAGTCATGAAGCCGGATTGAGATAATGCGACAGC
TP49960_Query	D	1	chr4	.	CAGCTGAACTTTTGGCTTTACTAGCTCCTGGAGCCATGAAGCCGGATTGAGATAATGCGACAGC
TP49988_Hit	D	1	chr4	.	CAGCTGAAGAGTATTTCACAAGTCGTTCAATGGTTGATGTTAAAGAGTTGAAGGAGAACAATCC
TP49988_Query	D	1	chr4	.	CAGCTGAAGAGTATTTCACAAGCCGTTCAATGGTTGATGTTAAAGAGTTGAAGGAGAACAATCC
TP5013_Hit	D	1	chr4	.	CAGCAAGAAACTCAGCAACAGTCATATCCTGACCAAGAATCCACTTAGCCATCCTCCTATGGCG
TP5013_Query	D	1	chr4	.	CAGCAAGAAACTCAGCAACAGTCATATCCTGACCAAGAATCCACTTAGCCATCCTCCTATGCCG
TP50148_Hit	D+G	1	chr4	.	CAGCTGAAGTTGAGGTTCCAGTTTTTTGCCAGTGCAATCCAGTTTAATCTTATGCCTGAGGTGC
TP50148_Query	D+G	1	chr4	.	CAGCTGAAGTTGAGGTTCCAGTTTTTTGCCAGTGCAATCCAGTTTAATCTTATGCCCGAGGTGC
TP50178_Hit	D	1	chr4	.	CAGCTGAATATTATTAAGTGATTATGTTGCTGTTTTCAATACTTTTTAGCAATAATAAATTAAG
TP50178_Query	D	1	chr4	.	CAGCTGAATATTATTAAGTGATTATGATGCTGTTTTCAATACTTTTTAGCAATAATAAATTAAG
TP50243_Hit	D+G	1	chr4	.	CAGCTGAATTCCATTCACTTTCAAACAGAAAAACTTTTCAAACGCGAGAATGGGGAAAAAGCAC
TP50243_Query	D+G	1	chr4	.	CAGCTGAATTCCATTCACTTTCAAACAGAAAAACTTTTCAAACGCAAGAATGGGGAAAAAGCAC
TP50299_Hit	D	1	chr4	.	CAGCTGACAATTCATCCTGCTTTCTCTGAATTGCTATCTCAGTATTCACAACGTGGTTACAGGT
TP50299_Query	D	1	chr4	.	CAGCTGACAATTCATCCCGCTTTCTCTGAATTGCTATCTCAGTATTCACAACGTGGTTACAGGT
TP50311_Hit	D	1	chr4	.	CAGCTGACAGGTCATCTCCTCTTTGAAAGGGCTTTGTCCTGTGAGGGTTGTTCCAAGGACTATT
TP50311_Query	D	1	chr4	.	CAGCTGACAGGTCATCTCCTCTTTGAAAGGGCTTTGTCCTCTGAGGGTTGTTCCAAGGACTATT
TP50438_Hit	D	1	chr4	.	CAGCTGAGAAGCGGGCTCAGATAGAGGAGTTAGATGAATAGGTTGTTTGGGCAAATGATAGTAG
TP50438_Query	D	1	chr4	.	CAGCTGAGAAGCGGGCTCAGATAGAGGAGTTAGATGAATAGGTTGTTTGGACAAATGATAGTAG
TP5058_Hit	D+G	1	chr4	.	CAGCAAGAACAGAAGTCCTTGAATTAAGAACGGTTGTTATTCCTGCTTTGGCTATGGATATAGT
TP5058_Query	D+G	1	chr4	.	CAGCAAGAACAGAAGTCCTTGAATTAAGAACGGCTGTTATTCCTGCTTTGGCTATGGATATAGT
TP50666_Hit	D	1	chr4	.	CAGCTGATAAGCCACTTGGACCAGCACCAACTATTCCAATTCTGGTGTTTTCTGGCAAAGAAGG
TP50666_Query	D	1	chr4	.	CAGCTGATAAACCACTTGGACCAGCACCAACTATTCCAATTCTGGTGTTTTCTGGCAAAGAAGG
TP50726_Hit	D	1	chr4	.	CAGCTGATATCATGGATGCTCTGCTTGAGCTCATTGTGTCATTGGTGAATATTTTATTTTGTAT
TP50726_Query	D	1	chr4	.	CAGCTGATATCATGGATGCTCTGCTTGAGCTCATCGTGTCATTGGTGAATATTTTATTTTGTAT
TP50779_Hit	D	1	chr4	.	CAGCTGATCCCATTAGTAGAGTCATCACATTCCCAACTTCCTCTTTATTAAAAGCTTTCTTGTA
TP50779_Query	D	1	chr4	.	CAGCTGATCCCATTAGTAGAGTCATCACATTCCCAACTTCCTCTTTATAAAAAGCTTTCTTGTA
TP50818_Hit	D	1	chr4	.	CAGCTGATGAAATTGGGGAAAAGAACCTCGAATTTGATCATATAAGTTATTCTTTCTGGACATT
TP50818_Query	D	1	chr4	.	CAGCTGATGAAATTGGGGAAAAGAACCTCGAATTTGATCATATAAGTTATACTTTCTGGACATT
TP50865_Hit	D+G	1	chr4	.	CAGCTGATGCACATGATCTCTGCCAGGAAACAAAGGTTTTCGATCCATCAGTTCCATGAAAATA
TP50865_Query	D+G	1	chr4	.	CAGCTGATGCACATGATCTCTGCCAGGAAACAAAGGCTTTCGATCCATCAGTTCCATGAAAATA
TP50946_Hit	D	1	chr4	.	CAGCTGATGTTCCTGGATTTGCAGTTTGCGAGGAAGGAAAGCCGTACTGTTGGTGCGGAGATCG
TP50946_Query	D	1	chr4	.	CAGCTGATGTTCCTGGATTTGCAGTTTGCGAGGAAGGAAAGCCGTACTGTTGGTGCCGAGATCG
TP50947_Hit	D	1	chr4	.	CAGCTGATGTTCCTGGATTTGCAGTTTGCGAGGAAGGAAAGCCGTACTGTTGGTGCTGATATCG
TP50947_Query	D	1	chr4	.	CAGCTGATGTTCCTGGATTTGCAGTTTGCGAGGAAGGAAAGCCGTACTGTTGGTGCTGAAATCG
TP50978_Hit	D	1	chr4	.	CAGCTGATTCAAGTTCTTGGCATATAAACTCGAACGTTGCTTTGCTCATCCGGAAACATCTCCT
TP50978_Query	D	1	chr4	.	CAGCTGATTCAAGTTCTTGGCATATAAACTCGAACGTTGCTTTGCTCATCCGGAAACATCTCCG
TP50986_Hit	D	1	chr4	.	CAGCTGATTTATCATGTAGACTCCTGGTGAATTGATTTTTGGCTGTTTTCTCTTGGACTGAAAC
TP50986_Query	D	1	chr4	.	CAGCTGATTCATCATGTAGACTCCTGGTGAATTGATTTTTGGCTGTTTTCTCTTGGACTGAAAC
TP5100_Hit	D+G	1	chr4	.	CAGCAAGAAGATATCAGAAATTATATTGACAACTCTGTCAAACATGGTGTTCAACTTTCAACAT
TP5100_Query	D+G	1	chr4	.	CAGCAAGAAGATATCAGAAATTATATTGACAACTCAGTCAAACATGGTGTTCAACTTTCAACAT
TP51018_Hit	D	1	chr4	.	CAGCTGATTGAGTCTAGAGTCTCCACTTTTAAATCTGTCTTCCAACTCTCAAGTTTCTTAGACA
TP51018_Query	D	1	chr4	.	CAGCTGATTGAATCTAGAGTCTCCACTTTTAAATCTGTCTTCCAACTCTCAAGTTTCTTAGACA
TP51094_Hit	D	1	chr4	.	CAGCTGGAAAAGTAAAGATAACTTTAAGCAATTACTATAGTGGTTCTTGCGTAATAAGGGTTTA
TP51094_Query	D	1	chr4	.	CAGCTGGAAAAGTAAAGATAACTTTAAGCAATTACTATAGTGGTTCATGCGTAATAAGGGTTTA
TP51149_Hit	D	1	chr4	.	CAGCTGGAACTGAACCAATCATCAATATCAACCTCCATGCAACGTCAGCCTCCGGCGGCACATC
TP51149_Query	D	1	chr4	.	CAGCTGGAACTGAACCAATCATCAATATCAACCTCCATGCAACGTCAGCCTCCGGAGGCACATC
TP51164_Hit	D	1	chr4	.	CAGCTGGAAGAGACAGACTCCTCTGGTTCACATCCTCATCTTGTGGTGGCCAGTAATGAAGCTC
TP51164_Query	D	1	chr4	.	CAGCTGGAAGAGACAGACCCCTCTGGTTCACATCCTCATCTTGTGGTGGCCAGTAATGAAGCTC
TP51190_Hit	D	1	chr4	.	CAGCTGGAATAGCAAAAGCTGGACCAACACCCATGACAGCAGGATCTACACCAACAGCTGAAAA
TP51190_Query	D	1	chr4	.	CAGCTGGAATAGCAAAAGCTGGACCAACACCCATGACAGCAGGATCTACACCAACAGCAGAAAA
TP51281_Hit	D+G	1	chr4	.	CAGCTGGAGAGCACGGAAAGTTAGGGTTTCATTTCTGTTGTGAGAAGGGTATTGGGCTGATTTA
TP51281_Query	D+G	1	chr4	.	CAGCTGGAGAGCACGGAAAGTTAGGGTTTCATTTCTGTTGCGAGAAGGGTATTGGGCTGATTTA
TP51357_Hit	D	1	chr4	.	CAGCTGGATATTATGTGGTAGTTCCTGACTACTTCTACGGTGATCCCTATGATGATGACTGTGT
TP51357_Query	D	1	chr4	.	CAGCTGGATATTATGTGGTAGTTCCTGACTACTTCTACGGCGATCCCTATGATGATGACTGTGT

Name	Filter	Nb hit (Mt4.0)	Mt Chr (Mt4.0)	Ms Chr (Li et al., 2014)	Sequence
TP51384_Hit	D	1	chr4	.	CAGCTGGATGGCGTTGGTGGAGCACAAAGGATAACTACCAAATCACTCTACGTTATCATAAGCT
TP51384_Query	D	1	chr4	.	CAGCTGGATGGCGTTGGTGGAGCACAAAGGATAACTACCAAATCACTCTACGTTATCATAAGCA
TP51459_Hit	D	1	chr4	.	CAGCTGGCCAGCTCCCAGCGCAAAGTCTAGCCACTCTTCAAGCTGAAAAAAAAAAAAAAAAAAA
TP51459_Query	D	1	chr4	.	CAGCTGGCCAGCTCCCAGCGCAAAGTCTAGCCACTCTTCAAGCAGAAAAAAAAAAAAAAAAAAA
TP51626_Hit	D	1	chr4	.	CAGCTGGGTTATGTGCCGATCTTGCATCGGCAGTTATTTCTGGGCGTGCCAAAAATGGATTTGC
TP51626_Query	D	1	chr4	.	CAGCTGGGCTATGTGCCGATCTTGCATCGGCAGTTATTTCTGGGCGTGCCAAAAATGGATTTGC
TP51704_Hit	D+G	1	chr4	.	CAGCTGGGTTTTCTGAGGGGATTGATATTTTGGGCGTTTGTTTTAAGTGCTGTTAGCCCGCTTG
TP51704_Query	D+G	1	chr4	.	CAGCTGGGTTTTCTGAGGGGATTGATATTTTGGGCGTTTGTTTTAAGTGCTGTTAGCCCACTTG
TP51743_Hit	D	1	chr4	.	CAGCTGGTAGGTATCGGACCACGCCACAACCACCAATTCTTCTTAAATCAGTGATCAGTCTCAT
TP51743_Query	D	1	chr4	.	CAGCTGGTAGGAATCGGACCACGCCACAACCACCAATTCTTCTTAAATCAGTGATCAGTCTCAT
TP51785_Hit	D	1	chr4	.	CAGCTGGTCATTTGAGTGCAATGTTAACTTAAATGCCGACAAAGACAATCTTTTAGGTTTCTGC
TP51785_Query	D	1	chr4	.	CAGCTGGTCATTTGAGTGCAATGCTAACTTAAATGCCGACAAAGACAATCTTTTAGGTTTCTGC
TP51786_Hit	D	1	chr4	.	CAGCTGGTCATTTGTGTGCAATGTTAACTTAAATGCCGACAAAGACAATCTGTTTTAGGTTTCT
TP51786_Query	D	1	chr4	.	CAGCTGGTCATTTGAGTGCAATGTTAACTTAAATGCCGACAAAGACAATCTGTTTTAGGTTTCT
TP51848_Hit	D+G	1	chr4	.	CAGCTGGTGAAGTTCATCTTGAGAGATGCATAAAGGATTTGAAGGATAGGTTTGCAAAAGTAAG
TP51848_Query	D+G	1	chr4	.	CAGCTGGTGAAGTTCATCTTGAGAGATGCATAAAAGATTTGAAGGATAGGTTTGCAAAAGTAAG
TP52260_Hit	D	1	chr4	.	CAGCTGTATTTGTTACAATGTCACCTCTTAATTCATCGACATTCAACTCTGCACAAACCTTGGA
TP52260_Query	D	1	chr4	.	CAGCTGTATTTGTTACAATGTCACCTCTTAATTCATCCACATTCAACTCTGCACAAACCTTGGA
TP52383_Hit	D	1	chr4	.	CAGCTGTCGCATTATCTCAATCCGGCTTCATGACTCCAGGAGCTAGTAAAGCCAAAAGTTCAGC
TP52383_Query	D	1	chr4	.	CAGCTGTCGCATTATCTCAATCCGGCTTCATGGCTCCAGGAGCTAGTAAAGCCAAAAGTTCAGC
TP52388_Hit	D+G	1	chr4	.	CAGCTGTCGCTAACTAAAATTTTAAAAAATGCAGGCATCTCAAAACGGTAAAATAGGGATTACC
TP52388_Query	D+G	1	chr4	.	CAGCTGTCGCTAACTAAAATTTTAAAAAATGCAGGCATCTCAAAAAGGTAAAATAGGGATTACC
TP52424_Hit	D	1	chr4	.	CAGCTGTCTTAAATTTCGCAATGCCCTGGAGAAGTAAAGACAGGTTTTCCTTTATTCCTTTACA
TP52424_Query	D	1	chr4	.	CAGCTGTCTTAAATTTCGCAATGCCCTGGAGAAGTAAAAACAGGTTTTCCTTTATTCCTTTACA
TP52594_Hit	D	1	chr4	.	CAGCTGTGGAGGATGGTGTGGATGTAATATCGATATCACTTGGTCTAAGTGAGCCTCCTCCATT
TP52594_Query	D	1	chr4	.	CAGCTGTGGAGGATGGCGTGGATGTAATATCGATATCACTTGGTCTAAGTGAGCCTCCTCCATT
TP52654_Hit	D	1	chr4	.	CAGCTGTGTGATGTTTATAATCACCGGTGGTGGGACAATGAAGCAATTATTCAAGATTCTTTGC
TP52654_Query	D	1	chr4	.	CAGCTGTGTAATGTTTATAATCACCGGTGGTGGGACAATGAAGCAATTATTCAAGATTCTTTGC
TP5266_Hit	D+G	1	chr4	.	CAGCAAGAGAATAGACCAAGTAAAACAACACTTTCGATGGAGGAAATGAGTATCATTGTCATAC
TP5266_Query	D+G	1	chr4	.	CAGCAAGAGAATAGACCAAGTAAAACAACACTTTCCATGGAGGAAATGAGTATCATTGTCATAC
TP52711_Hit	D	1	chr4	.	CAGCTGTGTTTCAAGCTTTGGAGAAGCTACCAGTATTCCTGATATAATTGAGAGCACTGTTGGT
TP52711_Query	D	1	chr4	.	CAGCTGTGTTTCAAGCTTTGGAGAAGCTACCAGCATTCCTGATATAATTGAGAGCACTGTTGGT
TP52736_Hit	D+G	1	chr4	.	CAGCTGTTAAATAGTTGATGCTATTGTTAATGTCTCTTTAATGTTGTTTTTCATGTGCTTCAAC
TP52736_Query	D+G	1	chr4	.	CAGCTGTTAAATAATTGATGCTATTGTTAATGTCTCTTTAATGTTGTTTTTCATGTGCTTCAAC
TP52813_Hit	D	1	chr4	.	CAGCTGTTCTATCTAACAATGCAAAAAGAAATAAAGTTGACATAATGACTGATGCTCATTTCTA
TP52813_Query	D	1	chr4	.	CAGCTGTTATATCTAACAATGCAAAAAGAAATAAAGTTGACATAATGACTGATGCTCATTTCTA
TP52923_Hit	D	1	chr4	.	CAGCTGTTGAATGCTAGAGGAACATTGAAGAGCAAGTTTAAGGATGCCATCCATAGGTTGAAGC
TP52923_Query	D	1	chr4	.	CAGCTGTTGAATGCTAGAGGAACATTGAAGAGCAAGTTTAAAGATGCCATCCATAGGTTGAAGC
TP52930_Hit	D+G	1	chr4	.	CAGCTGTTGAGGAAGAAAACCATGGCAGGATTCTTCAGGCAAATTGAAAGTCCCTATGATGCGT
TP52930_Query	D+G	1	chr4	.	CAGCTGTTGAGGAAGAAAACCATGGCAGGATTCTTCAGGCAAATTGAAAGTCCCTATGATGCGG
TP53024_Hit	D	1	chr4	.	CAGCTGTTGTAATATGTGGTTTAGCAGATGGCTTGGTTGGTGGAAGTTTGATAGGATCAGCAGG
TP53024_Query	D	1	chr4	.	CAGCTGTTGTAATATGTGGTTTAGCAGATGGCTTGGTTGGTGGAAGTCTGATAGGATCAGCAGG
TP53249_Hit	D	1	chr4	.	CAGCTTAAAGAATCCACCACAGTTTCTATACATTTCAAAAACCATACCAATTTGTCCACCATGT
TP53249_Query	D	1	chr4	.	CAGCTTAAAGAAACCACCACAGTTTCTATACATTTCAAAAACCATACCAATTTGTCCACCATGT
TP53251_Hit	D	1	chr4	.	CAGCTTAAAGAGGAACAGAATTGTAAGTGATTCTAAATTTATGATCCCCTTTCTGTAACTTTCT
TP53251_Query	D	1	chr4	.	CAGCTTAAAGAGGAACAGAATTGCAAGTGATTCTAAATTTATGATCCCCTTTCTGTAACTTTCT
TP53322_Hit	D	1	chr4	.	CAGCTTAACATTGGTCGATATCTTGTATGATTCTTTCTTGCACAGTGAATCTTAATACTCGCCT
TP53322_Query	D	1	chr4	.	CAGCTTAACATTGGTCGATATCTTGTATGATTTTTTCTTGCACAGTGAATCTTAATACTCGCCT
TP53510_Hit	D	1	chr4	.	CAGCTTAATTTATTTTTCCTTGTTAAAGGTTTTAGCTTCACAGAACTTTTCCCTAATTAGTGAA
TP53510_Query	D	1	chr4	.	CAGCTTAATTTATTTTTCCTTGTTAAAGGTTTTAACTTCACAGAACTTTTCCCTAATTAGTGAA
TP53520_Hit	D	1	chr4	.	CAGCTTAATTTGCAAGTTAACCGCAACAATCTCTTCATATTTGTCAAGCTACTATTGAAGTATC
TP53520_Query	D	1	chr4	.	CAGCTTAATTTGCAAGTTAACCGCAACAACCTCTTCATATTTGTCAAGCTACTATTGAAGTATC
TP53528_Hit	D	1	chr4	.	CAGCTTACAGAAAGAAATTTCACATACATACAAGAACAAAATTATGATTTCAGCTTCCAACACA
TP53528_Query	D	1	chr4	.	CAGCTTACAAAAAGAAATTTCACATACATACAAGAACAAAATTATGATTTCAGCTTCCAACACA

Name	Filter	Nb hit (Mt4.0)	Mt Chr (Mt4.0)	Ms Chr (Li et al., 2014)	Sequence
TP53762_Hit	D+G	1	chr4	.	CAGCTTAGATTGTTGAACAGAATAGCTACCGTAAAAGACGATGGAACTGTAGAATTTGACGTTC
TP53762_Query	D+G	1	chr4	.	CAGCTTAGATTGTTGAACAGAATAGCTACCGTAAAAGACGACGGAACTGTAGAATTTGACGTTC
TP53766_Hit	D	1	chr4	.	CAGCTTAGCAATTGTCTCTCTTTGAGGCTTTGTACCCTCAAAGTCTTTGATGGGTGAAGTATTT
TP53766_Query	D	1	chr4	.	CAGCTTAGCAATTGTCTCTCTTTGAGGCTTTGTACCCTCAAAGTCTTTGATAGGTGAAGTATTT
TP53798_Hit	D+G	1	chr4	.	CAGCTTAGGTAGGACTAAGCTAACATCACCCAACTCTCCAGAGAAAGCCTCCAAGTTGAGCCAT
TP53798_Query	D+G	1	chr4	.	CAGCTTAGGCAGGACTAAGCTAACATCACCCAACTCTCCAGAGAAAGCCTCCAAGTTGAGCCAT
TP53985_Hit	D	1	chr4	.	CAGCTTATAGTGAAGCTTTATCTCCTTCAGAAAATTCATATGATAAGCGAAGAACTCCTTTTAA
TP53985_Query	D	1	chr4	.	CAGCTTATAGTGAAGCTTTATCTCCTTCAGAAAATTCACATGATAAGCGAAGAACTCCTTTTAA
TP54252_Hit	D	1	chr4	.	CAGCTTATTATGCAGGAAATTAACAGTGCATATTTTTTACACCAGGTAGATATGCGGGGGAACT
TP54252_Query	D	1	chr4	.	CAGCTTATTATGCAGAAAATTAACAGTGCATATTTTTTACACCAGGTAGATATGCGGGGGAACT
TP5436_Hit	D	1	chr4	.	CAGCAAGATATACCTGGAAAGGGAGTGAAACGGCAATTAGAAATTCTGTGTAGTATTTATGCTT
TP5436_Query	D	1	chr4	.	CAGCAAGATATACCTGGAAAGGGAGTGAAACGGCAATTAGAAATTCTGTGTAGCATTTATGCTT
TP54362_Hit	D	1	chr4	.	CAGCTTATTTTTTGTGTGATTATTTGCATGAGTATGTATGTGGACGTGTTTTCTAATCTTCGAA
TP54362_Query	D	1	chr4	.	CAGCTTATTTTTTGTGTGATTATTTGCATGAGTATGTATGTGGACGTGTTTTCTAATCTTCAAA
TP54424_Hit	D	1	chr4	.	CAGCTTCGAATATAGTAATGTCCGTGACAGTGATGGTGACACTCCTTTTTCTTATGCCATTGTT
TP54424_Query	D	1	chr4	.	CAGCTTCAAATATAGTAATGTCCGTGACAGTGATGGTGACACTCCTTTTTCTTATGCCATTGTT
TP54453_Hit	D+G	1	chr4	.	CAGCTTCAACAATTGCAGGACGCAATGTCGTTAATGCTAACTTATTTGGCATGGCCAAAGGGAC
TP54453_Query	D+G	1	chr4	.	CAGCTTCAACAATTGCAGGACGCAATGTCGTTAATGCTAACTTATTTGGCATGGCCAAAGGAAC
TP54467_Hit	D	1	chr4	.	CAGCTTCAACAGTGCATTGTGCAAGCTTCCACTCTTGTTCAGTACCTATTTCGAGAACAGGAAA
TP54467_Query	D	1	chr4	.	CAGCTTCAACAGTGCATTGAGCAAGCTTCCACTCTTGTTCAGTACCTATTTCGAGAACAGGAAA
TP54480_Hit	D	1	chr4	.	CAGCTTCAACTAAAGGATCAACAGTTCCTTCTTGAATTGGCCTTATGTTAGCGGGATCTACCTG
TP54480_Query	D	1	chr4	.	CAGCTTCAACCAAAGGATCAACAGTTCCTTCTTGAATTGGCCTTATGTTAGCGGGATCTACCTG
TP54514_Hit	D	1	chr4	.	CAGCTTCAACTTGGTATGGTGCATGATGGAAAACAATAAGTAAAAAGAAATTAAGCTTCTGAAG
TP54514_Query	D	1	chr4	.	CAGCTTCAACTTGGTATGGTGCATGATGGAAAACAATAAGTAAAAAGAAATTAAGCTTATGAAG
TP54755_Hit	D+G	1	chr4	.	CAGCTTCACTCCTCTCAAATCCTCTTTTTTCATCGACAGGTAGGGTTAATTCTAAGGTTCCTTA
TP54755_Query	D+G	1	chr4	.	CAGCTTCACTCCTCTCAAATCCTATTTTTTCATCGACAGGTAGGGTTAATTCTAAGGTTCCTTA
TP55016_Hit	D	1	chr4	.	CAGCTTCATCAAGTCTTTGCCTAATACTCAAATGGACCCCATGGTTTGTGACTTGAAAGAAGCC
TP55016_Query	D	1	chr4	.	CAGCTTCATCAAGTCTTTGCCTAATACTCAAAGGGACCCCATGGTTTGTGACTTGAAAGAAGCC
TP55069_Hit	D	1	chr4	.	CAGCTTCATGAATCCTGATCAAAATTTTGTGGGGTCTGTCGGTGAGAATGATGATGAATTGGAT
TP55069_Query	D	1	chr4	.	CAGCTTCATGAATCCTGATCAAAATTTTGTGGGGTCTGTCGGTGAGAATGATGAGGAATTGGAT
TP55116_Hit	D	1	chr4	.	CAGCTTCATTCATAGCAAGCGCTTGCCGATCATAGAAAAGAACGTTGCTACCAAAAAATTCTTT
TP55116_Query	D	1	chr4	.	CAGCTTCATTCATAGCAAGCGCTTGCCGATCATAGAAAAGAACATTGCTACCAAAAAATTCTTT
TP55158_Hit	D	1	chr4	.	CAGCTTCATTTTACTACTGTAGGTGATCTGTGTTGAACCAGAACTGTCCTTACTGTTCCTCATG
TP55158_Query	D	1	chr4	.	CAGCTTCATTTTAATACTGTAGGTGATCTGTGTTGAACCAGAACTGTCCTTACTGTTCCTCATG
TP55215_Hit	D+G	1	chr4	.	CAGCTTCCACATTTCTATTTTCTTCTGTTGGTGGTGCACTAAAACTGAGGATGAAAAGTAGAAC
TP55215_Query	D+G	1	chr4	.	CAGCTTCCACATTTCTATTTTCTTCTGTTGGTGGTACACTAAAACTGAGGATGAAAAGTAGAAC
TP55243_Hit	D	1	chr4	.	CAGCTTCCAGTGGTCAAGTCATCGTCGGAGTCGAAAGCCGTTATCGCATCGTTTATCGTTTAGT
TP55243_Query	D	1	chr4	.	CAGCTTCCAGTGGTCAAGTCATCGTCGGAGTCGAAAGCCGTTACCGCATCGTTTATCGTTTAGT
TP5530_Hit	D	1	chr4	.	CAGCAAGATTTGTAACCTCTATTTCCAATTTAGCAGGGGCCTGTAATTTTTTTTTCAACGACCA
TP5530_Query	D	1	chr4	.	CAGCAAGATTTGTAACCTCTATTTCCAATTTAGCAGGGGCCTGTAATTTTTTTTTCAACAACCA
TP55730_Hit	D+G	1	chr4	.	CAGCTTCTAATGGTGATGGATATGCGAGCTACGGTGGTGCTTATGAGGGTTATGGGCAGTATGG
TP55730_Query	D+G	1	chr4	.	CAGCTTCTAATGGTGATGGATATGCGAGCTACGGTGGTGCTTATGAGGATTATGGGCAGTATGG
TP55804_Hit	D+G	1	chr4	.	CAGCTTCTATAATGAACATGATAGATCCAATATAAATAATTTATGCTATGCACATACACGTGTT
TP55804_Query	D+G	1	chr4	.	CAGCTTCTATAATGAACATGATAGATCCAATATAAATAATTTATGCTATGCACATACAAGTGTT
TP55844_Hit	D	1	chr4	.	CAGCTTCTCAGACACAAACTCGCCATTTTCAATTTCATTACTCTGCGGTGGCAGTGGCTGAAAA
TP55844_Query	D	1	chr4	.	CAGCTTCTCAGACACAAACTCGCCATTTTCAATTTCATTACTCTGCGGTGGCAGTGGCAGAAAA
TP55882_Hit	D	1	chr4	.	CAGCTTCTCCATGAAATTATTCTTTGCTTAACCAAAACTTTGTTATCTTTTATCAATTTCATTC
TP55882_Query	D	1	chr4	.	CAGCTTCTCCATGAAATTATTCTTTGCTTAACCAAAACTTTGTCATCTTTTATCAATTTCATTC
TP55934_Hit	D+G	1	chr4	.	CAGCTTCTCTATCATCCTCTTCATCCTCCGGCTCCGGAGGTAGCCACAGCTGAAAAAAAAAAAA
TP55934_Query	D+G	1	chr4	.	CAGCTTCTCTATCATCCTCTTCATCCTCCGGCTCCGGAGGTAGCCACAGCAGAAAAAAAAAAAA
TP5628_Hit	D	1	chr4	.	CAGCAAGCAGAAGAAGGATTCTCAAAACCTGCCACAAATATATTCAAATTAAGTACTTATTGAT
TP5628_Query	D	1	chr4	.	CAGCAAGCAGAAGAAGGATTCTCAAAACCTGCCACAAATACATTCAAATTAAGTACTTATTGAT
TP56362_Hit	D	1	chr4	.	CAGCTTCTGCATGAAATTATTCTTTGCTTAACCAAAACTTTGTATCTTTTATCAATTTCATTCT
TP56362_Query	D	1	chr4	.	CAGCTTCTGCATGAAATTATTCTTTGCTTAACCAAAACTTTGTATCTTTTATCAACTTCATTCT

Name	Filter	Nb hit (Mt4.0)	Mt Chr (Mt4.0)	Ms Chr (Li et al., 2014)	Sequence
TP56405_Hit	D+G	1	chr4	.	CAGCTTCTGGAATCGACGAAGCGGCTCTGTCAGAAGTCGGAACGATTCTAAAGCCTGTCAAAAA
TP56405_Query	D+G	1	chr4	.	CAGCTTCTGGAATCGACGAAGCGGCTCTGTCAGAAGTCGGAACCATTCTAAAGCCTGTCAAAAA
TP5642_Hit	D	1	chr4	.	CAGCAAGCAGGGTTGCAAATTGTGGTCATTCTCTTCACAGAGACTGTTTAAGATTCTATGGGTA
TP5642_Query	D	1	chr4	.	CAGCAAGCAGGGTTGCAAATTGTGGTCATTCTCTTCACAGAGACTGTTTAAGATTCTACGGGTA
TP56501_Hit	D+G	1	chr4	.	CAGCTTCTTATCCCAGTACAAGGCAAAAATTGGGGGACCCTGCTTTCCAACTACTAGTATACTT
TP56501_Query	D+G	1	chr4	.	CAGCTTCTTATCCCAGTACAAGGCAAAAATTGGGGGACCCTGCTTTCCAACTACTAGTATACAT
TP56613_Hit	D	1	chr4	.	CAGCTTCTTGAGTCTTGCATTTTTAGTTGATAGTAACTGTTAAAAGCATATGATATATTACCTT
TP56613_Query	D	1	chr4	.	CAGCTTCTTGAGTCTTGCATTTTTAGTTGATAGTAACTGTTAAAAGCATATGATATATTACCGT
TP56682_Hit	D	1	chr4	.	CAGCTTCTTTGATTGGCCTTGGAAGTATGTCATACTGGGTACTATCTTGAGTTATATGCTAATA
TP56682_Query	D	1	chr4	.	CAGCTTCTTTGATTGGCCTTGGAAGTATGTCATACTGAGTACTATCTTGAGTTATATGCTAATA
TP56702_Hit	D+G	1	chr4	.	CAGCTTCTTTTCCGCCTGCCCTTATCTTTTCCGCCTGTTTCTGTTTTACCTGTTCATCTTTTGG
TP56702_Query	D+G	1	chr4	.	CAGCTTCTTTTCCGCCCGCCCTTATCTTTTCCGCCTGTTTCTGTTTTACCTGTTCATCTTTTGG
TP56706_Hit	D	1	chr4	.	CAGCTTCTTTTGATGAGAATTTGAACTCAAGCAATTTTCTCAAAACTAATAACGGAATTTGATT
TP56706_Query	D	1	chr4	.	CAGCTTCTTTTGATGAGAATTTGAACTCAAGCAATTTTCTCAAAACAAATAACGGAATTTGATT
TP56714_Hit	D	1	chr4	.	CAGCTTCTTTTTCTTGGATGTTTGGTTATCAATGGTGAGTATAACCTTTCCAGGTTCGCCGACT
TP56714_Query	D	1	chr4	.	CAGCTTCTTTTTCTTGGATGTTTGGTTATCAATGGTGAGTATAACCTTTCCAGGTTCACCGACT
TP56777_Hit	D	1	chr4	.	CAGCTTGAAACCCCTTCAGTATCACCACCAGGCCCACAAGTCATAGTTGAACAACCGCAAGATG
TP56777_Query	D	1	chr4	.	CAGCTTGAAACCCCTTCAGTATCACCACCAGGCCCACAAGTCACAGTTGAACAACCGCAAGATG
TP56888_Hit	D	1	chr4	.	CAGCTTGAAGTATCTGACCAGAAGCATTTGCAATATAATCAAGAAGAAATGCCAATAATCCTGC
TP56888_Query	D	1	chr4	.	CAGCTTGAAGTATCTGACCAGAAGCATTTGCAACATAATCAAGAAGAAATGCCAATAATCCTGC
TP5693_Hit	D+G	1	chr4	.	CAGCAAGCATTCAAATCAACAAATAAATGTTAAACCTAATAGATCACCTTGGTGTCACTACATA
TP5693_Query	D+G	1	chr4	.	CAGCAAGCATTCAAATCAACAAATAAATGTTAAACCTAATAAATCACCTTGGTGTCACTACATA
TP57081_Hit	D	1	chr4	.	CAGCTTGAGTCTTGAAGTAGCAAATCAAGCAAAGGTAAATTAAGGATATCGAACTATATAGTAT
TP57081_Query	D	1	chr4	.	CAGCTTGAGCCTTGAAGTAGCAAATCAAGCAAAGGTAAATTAAGGATATCGAACTATATAGTAT
TP57161_Hit	D	1	chr4	.	CAGCTTGATAGATTTTGGTGAGGTTGTTGAGGTCGGTGGTGGTGAAGAGGGGTTCGACACCATG
TP57161_Query	D	1	chr4	.	CAGCTTGATAGATTTTGGTGAGGTTGTTGAGGTCGGTGGTGGTGAAGAGGGGTTCGACACCATC
TP57291_Hit	D	1	chr4	.	CAGCTTGCAAAATCGACACAGATTTGCAGACATTTGATTCAACCCTTCAAACCAGAACAAATCA
TP57291_Query	D	1	chr4	.	CAGCTTGCAAAATCGACACAGATTTGCAGACATTTGATTCAACACTTCAAACCAGAACAAATCA
TP57393_Hit	D	1	chr4	.	CAGCTTGCATATACTGTTTTGGAAGCCTTCCTGCTGATCCTATCAGACTTCCACCAACCAAGCC
TP57393_Query	D	1	chr4	.	CAGCTTGCATATACTGTTTTGGAAGCCTTCCTGCTGATCCTATCAAACTTCCACCAACCAAGCC
TP57619_Hit	D	1	chr4	.	CAGCTTGGAAATTTCAACTGACAGCCTCTGGGAAGAACTGGAAACACTTCAATTCCTGTAAGAT
TP57619_Query	D	1	chr4	.	CAGCTTGGAAATTTCAACTGACAGCCTCTGGGAAGAACTGGAAACACTTCAATTCCAGTAAGAT
TP57654_Hit	D	1	chr4	.	CAGCTTGGACACTTGCCATCAGTTTTGCTATTTCCCTTCCAGAAAATCCCTCGGTGTTAGCCGC
TP57654_Query	D	1	chr4	.	CAGCTTGGACACTTGCCATCAGTTTTGCTATCTCCCTTCCAGAAAATCCCTCGGTGTTAGCCGC
TP57671_Hit	D+G	1	chr4	.	CAGCTTGGAGATTCATTTGTTCATGAGTAAGTTCTTCCAAGTTGATCATTTCACATCTCACATT
TP57671_Query	D+G	1	chr4	.	CAGCTTGGAGATTCACTTGTTCATGAGTAAGTTCTTCCAAGTTGATCATTTCACATCTCACATT
TP57790_Hit	D	1	chr4	.	CAGCTTGGGCAATGTACAAGGCTCAAGAAGAACTTATAAAGGTTGCAAAGGAGTTTGGTGTTAA
TP57790_Query	D	1	chr4	.	CAGCTTGGGCAATGTACAAGGCTCAAGAAGAACTTATAAAGGTTGCAAAGGAGTTCGGTGTTAA
TP58051_Hit	D	1	chr4	.	CAGCTTGTATTAGTGAATCCTCTGGCTGAGGTAATTGAAAAGCTGAAAAAAGCAGATGAAGCTA
TP58051_Query	D	1	chr4	.	CAGCTTGTATTAGTGAATCCTCTAGCTGAGGTAATTGAAAAGCTGAAAAAAGCAGATGAAGCTA
TP5806_Hit	D	1	chr4	.	CAGCAAGCTAGTTGATCAACAAACCATATAGTTACGAAAAATTGCACATATTGGGGACCATATA
TP5806_Query	D	1	chr4	.	CAGCAAGCTAGTTGATCAACAAAACATATAGTTACGAAAAATTGCACATATTGGGGACCATATA
TP58169_Hit	D	1	chr4	.	CAGCTTGTGATGATGTATGTCTTTTTTATTCTTTTGCAATGTACAGTTTGTATGTGCCATATTT
TP58169_Query	D	1	chr4	.	CAGCTTGTGATGATGTATGTCTTTTTTATTCTTTTGCAATGTACAGTTTGTATGTGCCATATGT
TP5822_Hit	D+G	1	chr4	.	CAGCAAGCTCCAAATTACCAAGATGACCAGAAAATATTTGAGTAGACATTGACATCACATAGTT
TP5822_Query	D+G	1	chr4	.	CAGCAAGCTCCAAATTACCAAGATGACCAGAAAATATTTGAGTAGACATTGACATCACATAATT
TP58289_Hit	D	1	chr4	.	CAGCTTGTTACCAATAAATGATAGTTCATCAGCCACCAGAGAAAACGGATCAAGTTCATCCTGA
TP58289_Query	D	1	chr4	.	CAGCTTGTTACCAATAAATGATAGTTCATCAGCCACCAGAGAAAACGGATCAAGTTCATCCTAA
TP58324_Hit	D	1	chr4	.	CAGCTTGTTCATTTTGATTTCAATAGGTGAAGCTTCACTTGCCTTGCTTGTCATTATCTGAGTT
TP58324_Query	D	1	chr4	.	CAGCTTGTTCATTTTGATTTCAATAGGTAAAGCTTCACTTGCCTTGCTTGTCATTATCTGAGTT
TP58518_Hit	D	1	chr4	.	CAGCTTTAACTCTACATACCGCATTGCGACATGAAGGGAATTTTGAGCTCAGCCGAACAAATGC
TP58518_Query	D	1	chr4	.	CAGCTTTAACTCTACACACCGCATTGCGACATGAAGGGAATTTTGAGCTCAGCCGAACAAATGC
TP58557_Hit	D	1	chr4	.	CAGCTTTAATATCTGGACATACTCAAAATGATTGCAGTGATCAGGCCTTAAACTTGTATCGAGA
TP58557_Query	D	1	chr4	.	CAGCTTTAATATCTGGACATACTCAAAATGATTGCAGTGATCAGGCCTTAAACTTGTACCGAGA

Name	Filter	Nb hit (Mt4.0)	Mt Chr (Mt4.0)	Ms Chr (Li et al., 2014)	Sequence
TP58586_Hit	D	1	chr4	.	CAGCTTTACAAACATTATCCTTGGTATAGAATCCATCTTCACCTTTTTGAACTTCCACACCAAC
TP58586_Query	D	1	chr4	.	CAGCTTTACAAACATTATCCTTAGTATAGAATCCATCTTCACCTTTTTGAACTTCCACACCAAC
TP5861_Hit	D	1	chr4	.	CAGCAAGCTTGCCATCATCAACAAGAACCGTGAAAGCTATGAGTGAGGTAGAGGCATTTGCTTT
TP5861_Query	D	1	chr4	.	CAGCAAGCTTACCATCATCAACAAGAACCGTGAAAGCTATGAGTGAGGTAGAGGCATTTGCTTT
TP58710_Hit	D	1	chr4	.	CAGCTTTAGGACTTACTGGAATAGAGGGTAGCTTCAGAGCAAACTGTGAGGCAGTTATTGGCAT
TP58710_Query	D	1	chr4	.	CAGCTTTAGGACTTACTGGAATAGAGGGTAGCTTCAGAGAAAACTGTGAGGCAGTTATTGGCAT
TP58716_Hit	D+G	1	chr4	.	CAGCTTTAGGGGAGTGGTTGGAAGATATATCTCAGAATCAGGTATGCTGACTGGGATAATATTC
TP58716_Query	D+G	1	chr4	.	CAGCTTTAGGGGAGTGGTTGGAAGATATATCTCAGAATCAGGTATACTGACTGGGATAATATTC
TP58757_Hit	D	1	chr4	.	CAGCTTTATAATATGGAGGTTAAGAAAAGCACAATATCCACTAACATGTGCATGAACAAGATGT
TP58757_Query	D	1	chr4	.	CAGCTTTATAATATGGAGGTTAAGAAAAGCACAATATCCACTAACATGTGCATGAACAAGATGC
TP5880_Hit	D	1	chr4	.	CAGCAAGCTTCTCTCTAACAATATGACAGTCAATCTCCAAATGCTTGGTTCTCTCATGAAAGAC
TP5880_Query	D	1	chr4	.	CAGCAAGCTTCTCTCTAACAATATGACAGTCAATCTCCAAATGCTTGGTTCTCTCATAAAAGAC
TP58904_Hit	D	1	chr4	.	CAGCTTTCAAATCTCACACCTACAATAAATAAAAACAAAAGCCACTACACTAAACCCTGTATAT
TP58904_Query	D	1	chr4	.	CAGCTTTCAAATCTCACACCTACAATAAATAAAAACAAAAGCCACTACACAAAACCCTGTATAT
TP58934_Hit	D	1	chr4	.	CAGCTTTCAATATGTGAAGACATGAACCCCCCGACATGAATGGCATCACAACCCATAGATTGTG
TP58934_Query	D	1	chr4	.	CAGCTTTCAATATGTGAAGACATGAACCACCCGACATGAATGGCATCACAACCCATAGATTGTG
TP58947_Hit	D	1	chr4	.	CAGCTTTCAATTTCTCACGGCACATGCACCTCATAAATATGTGTACTGACCGTCATTACACTTC
TP58947_Query	D	1	chr4	.	CAGCTTTCAATTTCTCACGGCACATGCACCTCATAAATATGTGTACTAACCGTCATTACACTTC
TP58986_Hit	D	1	chr4	.	CAGCTTTCAGAATCTGACAAAATAGTAGATGATGATAGAACATCACCACCGGAATCAACCTTGT
TP58986_Query	D	1	chr4	.	CAGCTTTCAGAATCTGACAAAACAGTAGATGATGATAGAACATCACCACCGGAATCAACCTTGT
TP59008_Hit	D+G	1	chr4	.	CAGCTTTCATAACTACAGTATACAATCCCGACAAACCAGATATGGAAGATCCAACTACAAGGCA
TP59008_Query	D+G	1	chr4	.	CAGCTTTCATAACTACAGTATACAATCCCGACAAACCAGATATAGAAGATCCAACTACAAGGCA
TP59027_Hit	D	1	chr4	.	CAGCTTTCATCTTTATTGTTGGTTTTGATGTGTTTTATGTAGTGTATGCTAATTATGATAACTC
TP59027_Query	D	1	chr4	.	CAGCTTTCATCTTTATTGTTGGTTTTGATGTGTTTTATGTAGTATATGCTAATTATGATAACTC
TP59113_Hit	D+G	1	chr4	.	CAGCTTTCCTTTCAATTGTGGACTAGTCAAATGCAAGAGACCTTGAATTTGAAGAAGCATGGAG
TP59113_Query	D+G	1	chr4	.	CAGCTTTCCTTTCAACTGTGGACTAGTCAAATGCAAGAGACCTTGAATTTGAAGAAGCATGGAG
TP59179_Hit	D+G	1	chr4	.	CAGCTTTCTCAGTAGTCATCAAATACATCTGCCTCTTCGCCTCCTGTTTTTCCTGCCATCTGCT
TP59179_Query	D+G	1	chr4	.	CAGCTTTCTCAGTACTCATCAAATACATCTGCCTCTTCGCCTCCTGTTTTTCCTGCCATCTGCT
TP59192_Hit	D	1	chr4	.	CAGCTTTCTCCTTAAGAGCATCAATATGCTGGAGCTCCCTATTCTTCTCTTCCTCAAAAGCTGT
TP59192_Query	D	1	chr4	.	CAGCTTTCTCCTTAAGAGCATCAATATGCTGGAGCTCCCTATTCTTCTCTTCCTCAAAAGCTGA
TP59279_Hit	D+G	1	chr4	.	CAGCTTTCTTTCTATTCGTAGGAACTGAGAACCGTTAGAGTGAGACTTTTGCTATTTTGCCACC
TP59279_Query	D+G	1	chr4	.	CAGCTTTCTTTCTATTCGTAGGAACTGAGAACCGTTAGAGTGAGACGTTTGCTATTTTGCCACC
TP59326_Hit	D	1	chr4	.	CAGCTTTGAAGCAAGGGGATCCTAAAGTTGCAGAAGATGGTGCAAATGATACTGCAAATGAAGC
TP59326_Query	D	1	chr4	.	CAGCTTTGAAGCAAGGGGATCCAAAAGTTGCAGAAGATGGTGCAAATGATACTGCAAATGAAGC
TP59408_Hit	D	1	chr4	.	CAGCTTTGATATTGTCCTTGCAGGAGTGGAAGCAAGGTTTTGGTTTCACTTATACCACTAGCTG
TP59408_Query	D	1	chr4	.	CAGCTTTGATACTGTCCTTGCAGGAGTGGAAGCAAGGTTTTGGTTTCACTTATACCACTAGCTG
TP59508_Hit	D	1	chr4	.	CAGCTTTGCTAAATTACTTTTCTTGTGGATATTTCTCTGAAGATAGACCCTCAGAAACCCACCT
TP59508_Query	D	1	chr4	.	CAGCTTTGCTAAATTACTTTTCTTGTGGATATTTCTCTAAAGATAGACCCTCAGAAACCCACCT
TP59536_Hit	D	1	chr4	.	CAGCTTTGCTTTCCGTTGTCTCAAAAGTGGTTGACTTCACTGCTGTCTCAGAATATATATCAAC
TP59536_Query	D	1	chr4	.	CAGCTTTGCTTTCCGGTGTCTCAAAAGTGGTTGACTTCACTGCTGTCTCAGAATATATATCAAC
TP59625_Hit	D+G	1	chr4	.	CAGCTTTGGGTGTAGCACATAAACTTGCAACACCGTCTTCCAAACCTGCTTCCGCTGAAAAAAA
TP59625_Query	D+G	1	chr4	.	CAGCTTTGGGTGTAGCACATAAACTTGCAACACCGTCTTCCAAACCTGCTTCCGCAGAAAAAAA
TP59701_Hit	D+G	1	chr4	.	CAGCTTTGTCACGGATCCATGACTTGCCACCATTATTAGTTCTCAAGAGAATACCACTGCCACC
TP59701_Query	D+G	1	chr4	.	CAGCTTTGTCACGGACCCATGACTTGCCACCATTATTAGTTCTCAAGAGAATACCACTGCCACC
TP59725_Hit	D	1	chr4	.	CAGCTTTGTGATATCCCAATCTATAGAGTAGTTTTATTAGTTGCTTGGGTAGTTAGCTTGTAAC
TP59725_Query	D	1	chr4	.	CAGCTTTGTGATATCCCAATCTATAGAGTAGTTTTATTAGTTGCCTGGGTAGTTAGCTTGTAAC
TP59744_Hit	D	1	chr4	.	CAGCTTTGTGTTCTTGCAGAAGATTGTGACCAGCCTGACTATGTTAAACTCGTCAAGGCTCTTT
TP59744_Query	D	1	chr4	.	CAGCTTTGTGTTCTTGCAGAAGATTGTGACCAGCCTGACTATGTTAAACTCGTCAAGGCTCTTG
TP59835_Hit	D	1	chr4	.	CAGCTTTTAATGTTTACATTACTGCTTGCTATGTTGATATTGCCTATGATTCAGATCCATTGTA
TP59835_Query	D	1	chr4	.	CAGCTTTTAATGTTTACATTACTGCTTGCTATGTTGATACTGCCTATGATTCAGATCCATTGTA
TP59842_Hit	D+G	1	chr4	.	CAGCTTTTACAGGATTTCTTCAATGGGAAAGAACTTTGCAAGAGTATTAACCCTGATGAAGCTG
TP59842_Query	D+G	1	chr4	.	CAGCTTTTACAGGATTTCTTCAATGGGAAAGAACTTTGCAAGAGTATTAACCCAGATGAAGCTG
TP60363_Hit	D+G	1	chr4	.	CAGCTTTTTCTGACACATAGAACAGCCCTCAGCTCTGTGGATTTTCTCAGGGGTAATAACTTGT
TP60363_Query	D+G	1	chr4	.	CAGCTTTTTCTGACACATAGAACAGCCCTCAGCTCTGTGGATTTGCTCAGGGGTAATAACTTGT

Name	Filter	Nb hit (Mt4.0)	Mt Chr (Mt4.0)	Ms Chr (Li et al., 2014)	Sequence
TP6055_Hit	D	1	chr4	.	CAGCAAGGCAGTACATTACAAAACAAGCTTTGTCATAGGTACATTATGAAATCCATCCCTGCAT
TP6055_Query	D	1	chr4	.	CAGCAAGGCAGTACATTACAAAACAAGCCTTGTCATAGGTACATTATGAAATCCATCCCTGCAT
TP60728_Hit	D+G	1	chr4	.	CTGCAAAAGACATGGAGAAAGTATTAGATGTCTCAATGAGCCAAATAACCGAAGAGAATCCATT
TP60728_Query	D+G	1	chr4	.	CTGCAAAAGACATGGAGAAAGTACTAGATGTCTCAATGAGCCAAATAACCGAAGAGAATCCATT
TP608_Hit	D	1	chr4	.	CAGCAAAAGAGTCAAGGCCTGCATCTGAAACCATGAACCTGTTGCCAACAATTGAAAACACTGA
TP608_Query	D	1	chr4	.	CAGCAAAAGAGTCAAGGCCTGCATCTGAAACCATGAACCTGTTGCCAACAATTGAAAACACAGA
TP6112_Hit	D+G	1	chr4	.	CAGCAAGGGACAAAGTGGAAGTAAGCCAAGGATAAGGAAAAGCATTCAAAACCTTCTTGTTGTA
TP6112_Query	D+G	1	chr4	.	CAGCAAGGGACAAAGTAGAAGTAAGCCAAGGATAAGGAAAAGCATTCAAAACCTTCTTGTTGTA
TP61333_Hit	D	1	chr4	.	CTGCAAAGGTATGCCAAATAAGTCAATCATTTACTTCAATATATAAACTGACAAATAGCTCCCT
TP61333_Query	D	1	chr4	.	CTGCAAAGGAATGCCAAATAAGTCAATCATTTACTTCAATATATAAACTGACAAATAGCTCCCT
TP61410_Hit	D	1	chr4	.	CTGCAAAGTATGGACTTGGAAAATTGTCTTTAAGACTATTTCAGGAAATGGTTGATAGAAGAAT
TP61410_Query	D	1	chr4	.	CTGCAAAGTATGGACTTGGAAAATTGTCTTCAAGACTATTTCAGGAAATGGTTGATAGAAGAAT
TP61415_Hit	D	1	chr4	.	CTGCAAAGTCTGATTTGAAAGTTGTAGGGTTGTTGCTGTATTCTGAAACAATAGAATCGGTAGA
TP61415_Query	D	1	chr4	.	CTGCAAAGTCTGATTTGAAAGTTGTAGGATTGTTGCTGTATTCTGAAACAATAGAATCGGTAGA
TP61469_Hit	D+G	1	chr4	.	CTGCAAATAAGCTGTTTATCCAAACACTCTGATGGGAACTCGTGATTTTTTTAGGGTCTCCACG
TP61469_Query	D+G	1	chr4	.	CTGCAAATAAGCTGTTTATCCAAACACTCTGATGGGAACTCGTGATTTTTTTAGGGTCTCCACA
TP6152_Hit	D+G	1	chr4	.	CAGCAAGGGGTTTCTTTTGAAACCTCTCGCAACTTGTTGGCTGGTACGACTGGAAAAGACGAGG
TP6152_Query	D+G	1	chr4	.	CAGCAAGGGGTTTCTTTTGAAACCTCTCACAACTTGTTGGCTGGTACGACTGGAAAAGACGAGG
TP61714_Hit	D+G	1	chr4	.	CTGCAAATGGATCCTTTTCCACTTCAACCTCGTCCTGTTTCTGTTTCTGTTGAATCTCTTCATT
TP61714_Query	D+G	1	chr4	.	CTGCAAATGGATCCTTTTCCACTTCAACCTCGTCCTGTTTCTGCTTCTGTTGAATCTCTTCATT
TP61731_Hit	D+G	1	chr4	.	CTGCAAATGTAAAATAAAACACACGCATTAACTAGAAATCATATGAGAAGTTCAGCAACCAATC
TP61731_Query	D+G	1	chr4	.	CTGCAAATGTAAAATAAAACACACACATTAACTAGAAATCATATGAGAAGTTCAGCAACCAATC
TP61792_Hit	D	1	chr4	.	CTGCAAATTAGAGCAAAATGTGGATAAATGAGGCCCAAATTGTAGTTGTGATGCCTAGGTGAAG
TP61792_Query	D	1	chr4	.	CTGCAAATTAGAGCAAAATGTGGATAAATGAGGCCCAAATTGTAGTTGTGATGCCGAGGTGAAG
TP6186_Hit	D	1	chr4	.	CAGCAAGGTAGTAAAATTAGGATATAAAAAGGAATAAGGGAGAAAACAAAGAGGCATCAGGACA
TP6186_Query	D	1	chr4	.	CAGCAAGGTAGTAAAATTAGGATATAAAAAGGAATAAGGGAGAAAACAAAGAGGCACCAGGACA
TP61951_Hit	D	1	chr4	.	CTGCAACAAATACTTCAACACAAGTTTTCAAAGACAACAACAGTGGAAACTCAGAAACTACAAG
TP61951_Query	D	1	chr4	.	CTGCAACAAATACTTCAACACAAGTTTTCAAAGACAACAACAATGGAAACTCAGAAACTACAAG
TP61998_Hit	D	1	chr4	.	CTGCAACCAGAGAATTAAAACAAACAGTCAGTGACATAAAGCATGAAGTCCATCACCAACTAGA
TP61998_Query	D	1	chr4	.	CTGCAACAAGAGAATTAAAACAAACAGTCAGTGACATAAAGCATGAAGTCCATCACCAACTAGA
TP62079_Hit	D	1	chr4	.	CTGCAACACAGACTCGTAAAATTGTACTCATATCATATCCAGATCTTGAAACTCGTTCTATAGA
TP62079_Query	D	1	chr4	.	CTGCAACACAGACTCGTAAAATTGTACTCATATCAAATCCAGATCTTGAAACTCGTTCTATAGA
TP62120_Hit	D	1	chr4	.	CTGCAACACTAGAAGACAATATGTCAAAACACCGATAATTGATTGATTTGGGGTTATTATGATA
TP62120_Query	D	1	chr4	.	CTGCAACACTAGAAGACAATAAGTCAAAACACCGATAATTGATTGATTTGGGGTTATTATGATA
TP62183_Hit	D	1	chr4	.	CTGCAACAGGCGGGCCAAGTCCCATTCCAGTACCAACAAATAACCTTCCCAAAAGCATGCCCCA
TP62183_Query	D	1	chr4	.	CTGCAACAGGCGGGCCAAGTCCCAGTCCAGTACCAACAAATAACCTTCCCAAAAGCATGCCCCA
TP62257_Hit	D	1	chr4	.	CTGCAACATCAAGGGAAGGTCTTCCATTTCAGAATAATAATTTGCGTCTCTAAACCTCGGTGAC
TP62257_Query	D	1	chr4	.	CTGCAACATCAAGGGAAGGTCTTCCATTTCAGAATAATAATTTGCATCTCTAAACCTCGGTGAC
TP62602_Hit	D	1	chr4	.	CTGCAACTTTCATATGATTTTGTTTATACTTCTCTTTGGTGTTCTTCCCCCTGCTGTTTCAAGA
TP62602_Query	D	1	chr4	.	CTGCAACGTTCATATGATTTTGTTTATACTTCTCTTTGGTGTTCTTCCCCCTGCTGTTTCAAGA
TP6261_Hit	D+G	1	chr4	.	CAGCAAGGTTTAATCCCCAAGCTGTTGTTCCATGTGTGTCTATGCCCCCAAAAAGAAAAGGTGG
TP6261_Query	D+G	1	chr4	.	CAGCAAGGTTTAATCCCCAAGCTGTTGTTCCATGTGTGTCTACGCCCCCAAAAAGAAAAGGTGG
TP62766_Hit	D+G	1	chr4	.	CTGCAACTCTTAAACATTTGAATGTTCATTGTTTTTCTTATAAATTTGAAAACAGTTTTCAAAT
TP62766_Query	D+G	1	chr4	.	CTGCAACTCTTAAACATTTGAATGTTAATTGTTTTTCTTATAAATTTGAAAACAGTTTTCAAAT
TP62858_Hit	D	1	chr4	.	CTGCAACTGTTTGAATCCTTAGTTAGCCAATTAGTATTATTGCAATAATCTAATGTCAGCCAAT
TP62858_Query	D	1	chr4	.	CTGCAACTGTTTGAATCCTTAGTTAGCCAATTAGTATCATTGCAATAATCTAATGTCAGCCAAT
TP62865_Hit	D+G	1	chr4	.	CTGCAACTTACAGTACCAATTCAAAATGGTGGTTACTGTAATTATACACATCACTTTCATTTTG
TP62865_Query	D+G	1	chr4	.	CTGCAACTTACAGTACCAATTCAAAATGATGGTTACTGTAATTATACACATCACTTTCATTTTG
TP63468_Hit	D	1	chr4	.	CTGCAAGGTCGCCATTCCTGAATGAATCATAAAACTTGCTGTTTGCTCCAAATACTGCTGTGTT
TP63468_Query	D	1	chr4	.	CTGCAAGGTCACCATTCCTGAATGAATCATAAAACTTGCTGTTTGCTCCAAATACTGCTGTGTT
TP63631_Hit	D+G	1	chr4	.	CTGCAAGTGTCTATTCAAGTAGTTGGAGTTTTATATTGGATTTTATGCTCAAAACCATACATTG
TP63631_Query	D+G	1	chr4	.	CTGCAAGTGTCTATTCAAGTAGTTGGAGTTCTATATTGGATTTTATGCTCAAAACCATACATTG
TP63671_Hit	D	1	chr4	.	CTGCAAGTTCTGAAATAGCCCAAGCAACAACTATTTGAACCTTCATGTGACCATCTTTTAGTAC
TP63671_Query	D	1	chr4	.	CTGCAAGTTCTGAAATAGCCCAAGCAACAACTATTTGAACCTTCATGTGACCATCTTTGAGTAC

Name	Filter	Nb hit (Mt4.0)	Mt Chr (Mt4.0)	Ms Chr (Li et al., 2014)	Sequence
TP63699_Hit	D+G	1	chr4	.	CTGCAAGTTTGAAAGTTGTGGGAAGAGTGCTCAAGGGAGCACAGATTTCTGCAAGGCCCATGGT
TP63699_Query	D+G	1	chr4	.	CTGCAAGTTTGAAAGCTGTGGGAAGAGTGCTCAAGGGAGCACAGATTTCTGCAAGGCCCATGGT
TP63767_Hit	D	1	chr4	.	CTGCAATAAGATATCACCTATTTGCAAATCCATGATCTCTCTTGTTAATGTAGCATCAAAAGTT
TP63767_Query	D	1	chr4	.	CTGCAATAAGATATCACCTATGTGCAAATCCATGATCTCTCTTGTTAATGTAGCATCAAAAGTT
TP63782_Hit	D	1	chr4	.	CTGCAATAAGTGATGGTGTTGATGTGCTTTCAGTGTCTTTGGGTAGTAATATTCCCGTTGAATT
TP63782_Query	D	1	chr4	.	CTGCAATAAGTGATGGCGTTGATGTGCTTTCAGTGTCTTTGGGTAGTAATATTCCCGTTGAATT
TP63911_Hit	D	1	chr4	.	CTGCAATATAAATTTTTTGGAGGTCTTCACCTCGGCATCACAACTACAATTTGGGCCTCATTTA
TP63911_Query	D	1	chr4	.	CTGCAATATAAATTTTTTGGAGGTCTTCACCTAGGCATCACAACTACAATTTGGGCCTCATTTA
TP63925_Hit	D+G	1	chr4	.	CTGCAATATATTAAAGCAAATTAATTAAATGTTAATATGTTATCTAAGCACAAAAAAAAGTTCA
TP63925_Query	D+G	1	chr4	.	CTGCAATATATTAAAGCAAATTAATTAAATGTTAATATGTTATCTAAGCACAAAAAAAAGTGCA
TP64214_Hit	D	1	chr4	.	CTGCAATGAAGAAGCGGGAGAAGGCTCGTTGAGCTCGGATTTCCACTCACGAAGCATTTGATGA
TP64214_Query	D	1	chr4	.	CTGCAATGAAGAAGCGGGAGAAGGCTCGTTGAGCTCGGACTTCCACTCACGAAGCATTTGATGA
TP64304_Hit	D	1	chr4	.	CTGCAATGCAAGTTCTCTCGTTGTGTCCTCCCACACCTTTTCATTCCACCACTTCCAAAGGCAC
TP64304_Query	D	1	chr4	.	CTGCAATGCAAGCTCTCTCGTTGTGTCCTCCCACACCTTTTCATTCCACCACTTCCAAAGGCAC
TP64340_Hit	D	1	chr4	.	CTGCAATGCGTGCCTTGTAAGCATTTTTGTTGATTTCACCAGAATGAAGAATCTAATTGATTTG
TP64340_Query	D	1	chr4	.	CTGCAATGCGTGCCTTATAAGCATTTTTGTTGATTTCACCAGAATGAAGAATCTAATTGATTTG
TP644_Hit	D	1	chr4	.	CAGCAAAAGCACTCAAGCTTGGTGATGCCTGTCCTAGAGACCTGTAATTGGAAATAGAAATATA
TP644_Query	D	1	chr4	.	CAGCAAAAGCACTCAAGCTTGGAGATGCCTGTCCTAGAGACCTGTAATTGGAAATAGAAATATA
TP64410_Hit	D+G	1	chr4	.	CTGCAATGGTGATGCTTGACTGGATGGTTGCATCATCTGTAATTGTTGTTGAAGCTGGGAGAAG
TP64410_Query	D+G	1	chr4	.	CTGCAATGGCGATGCTTGACTGGATGGTTGCATCATCTGTAATTGTTGTTGAAGCTGGGAGAAG
TP64414_Hit	D+G	1	chr4	.	CTGCAATGGCTCTTCCCCCTCTATCTGTTCGAGGAGATTTCGGTGTTCTTTACACCGGTGCTGA
TP64414_Query	D+G	1	chr4	.	CTGCAATGGCTCTTCCCCCTCTATCAGTTCGAGGAGATTTCGGTGTTCTTTACACCGGTGCTGA
TP64484_Hit	D	1	chr4	.	CTGCAATGTTTAATTCCTGTTTTTCTTTTCCTTCCACTTCTCCATATTTGGGCTCTGGTTGATG
TP64484_Query	D	1	chr4	.	CTGCAATGTCTAATTCCTGTTTTTCTTTTCCTTCCACTTCTCCATATTTGGGCTCTGGTTGATG
TP64497_Hit	D	1	chr4	.	CTGCAATGTGATAAACAATACAAGTTCATTAATAAACTAAAGTCTTCTTAGCTTTATTTTCGTT
TP64497_Query	D	1	chr4	.	CTGCAATGTGATAAACAATACAAGTTCATTAATAAACTAAAGTCTTCTTAGCCTTATTTTCGTT
TP64504_Hit	D	1	chr4	.	CTGCAATGTGCATAGCACTTTGATTGTCACAGTACAACACAGGAAGTTGAACACACTGAACATG
TP64504_Query	D	1	chr4	.	CTGCAATGTGCATAACACTTTGATTGTCACAGTACAACACAGGAAGTTGAACACACTGAACATG
TP64546_Hit	D+G	1	chr4	.	CTGCAATGTTTGACATGGAGTATGCAAGATGGCTAGAAGAAGATCAACGTCACATGGCGGAGCT
TP64546_Query	D+G	1	chr4	.	CTGCAATGTTTGACATGGAGTATGCAAGATGGCTAGAAGAAGATCAACGACACATGGCGGAGCT
TP64745_Hit	D	1	chr4	.	CTGCAATTGCACTATTGATACTTTCAGTCAGAATCTTCTGCAAAAACAAGCAAGAATTCTCAAG
TP64745_Query	D	1	chr4	.	CTGCAATTGCACTATTGATACTTTCAGTAAGAATCTTCTGCAAAAACAAGCAAGAATTCTCAAG
TP64768_Hit	D	1	chr4	.	CTGCAATTGCGAATAAAGCGTGTAGATCCACTACTGCAGTAGAACTCCTTGGAAAATTTCTGGT
TP64768_Query	D	1	chr4	.	CTGCAATTGCGAATAAAGCGTGTAGATCCACTACTGCAGTAGAACCCCTTGGAAAATTTCTGGT
TP65024_Hit	D	1	chr4	.	CTGCACAAATAAGGTAAAAACAGTGGTAATTTCTCTCCGGATCTGAAACTTGACAAACACGGGA
TP65024_Query	D	1	chr4	.	CTGCACAAAGAAGGTAAAAACAGTGGTAATTTCTCTCCGGATCTGAAACTTGACAAACACGGGA
TP65069_Hit	D	1	chr4	.	CTGCACAAATTTTCGTTTCATGGGTCTCAGTAATTTATGAGTTTCCAGAAAAACCCTTGAATTT
TP65069_Query	D	1	chr4	.	CTGCACAAATTTTCGTTTCATGGGTCTCAATAATTTATGAGTTTCCAGAAAAACCCTTGAATTT
TP65097_Hit	D+G	1	chr4	.	CTGCACAACCCACTGCATGTTAAGACCCTTATGAGGTTGGGACGGCGTAGTACCCTGAGACTGT
TP65097_Query	D+G	1	chr4	.	CTGCACAACCCACTGCATGTTAAGACCCTTATGAGGTTGGGACGGCGTACTACCCTGAGACTGT
TP65115_Hit	D	1	chr4	.	CTGCACAACTCAGTCCACCAATACCACTCCCAATCACAATTACATCTGTTTCTGTTTCATCTAC
TP65115_Query	D	1	chr4	.	CTGCACAACTCAGTCCACCAATACCACTCCCAATCACAATTACATCTGTTTCTGTTCCATCTAC
TP652_Hit	D	1	chr4	.	CAGCAAGAGCATGGTTAGAAATGAGTTTGATCTCTCCAAAGCAAGAAACGGAGCCCATCGGAAT
TP652_Query	D	1	chr4	.	CAGCAAAAGCATGGTTAGAAATGAGTTTGATCTCTCCAAAGCAAGAAACGGAGCCCATCGGAAT
TP65256_Hit	D+G	1	chr4	.	CTGCACAATTTCCCAACCACCGCCGACGATGCTTCTTCCACCACCACCTCCTCCTTCACATGGT
TP65256_Query	D+G	1	chr4	.	CTGCACAATTTCCCAACCACCGCCGACGACGCTTCTTCCACCACCACCTCCTCCTTCACATGGT
TP65329_Hit	D	1	chr4	.	CTGCACACCTATCATCTTAATTTTATTTGCTTCAACTATCAATTTTGATGTGCCAAAGTTGTTA
TP65329_Query	D	1	chr4	.	CTGCACACCTATCATCTTAATTTTATTTGCGTCAACTATCAATTTTGATGTGCCAAAGTTGTTA
TP65378_Hit	D	1	chr4	.	CTGCACAGAAAACCAAAGACTATGCTGGTGATGCTACAGAGAAGAGCAAAGAGTATGCAACTGA
TP65378_Query	D	1	chr4	.	CTGCACAGAAAACCAAAGACTATGCTGGTGATGCTACAGAGAAGAGCAAAGAGAATGCAACTGA
TP6567_Hit	D	1	chr4	.	CAGCAAGTTCATTCACTTGATACTTTATATCCGGCCGAGTTGAATCAAATTTTCGATGACTCAA
TP6567_Query	D	1	chr4	.	CAGCAAGTTCATTCACTTGATACTTTATATCCGGCCGAGTTGAATCAAATTTTCAATGACTCAA
TP65722_Hit	D+G	1	chr4	.	CTGCACATTTGGACACACTGCGGATGCTATTGAAGCTTCCCATCCATTCTCTGATATCTGCTGA
TP65722_Query	D+G	1	chr4	.	CTGCACATTTGGAAACACTGCGGATGCTATTGAAGCTTCCCATCCATTCTCTGATATCTGCTGA

Name	Filter	Nb hit (Mt4.0)	Mt Chr (Mt4.0)	Ms Chr (Li et al., 2014)	Sequence
TP6576_Hit	D	1	chr4	.	CAGCAAGTTCCTGATAGTGTCGTGCCAGCTATAACTGATGGCCAAATACCAGTCACAATGCCAG
TP6576_Query	D	1	chr4	.	CAGCAAGTTCCTGATAGTGTCGTGCCAGCTATAACTGATGGACAAATACCAGTCACAATGCCAG
TP6626_Hit	D	1	chr4	.	CAGCAAGTTGTGAAAAAGGTCAAAACAAAGTACATAGATAAATCTTGAAAAGCAAGAAGAAGAA
TP6626_Query	D	1	chr4	.	CAGCAAGTTGTGAAAAAGGTCAAAACAAAGAACATAGATAAATCTTGAAAAGCAAGAAGAAGAA
TP6648_Hit	D	1	chr4	.	CAGCAAGTTTCTCTACCCTTTTAAGCAGATCACTTTTGGAGAGACGCTTCTTCTTTGATTTGCC
TP6648_Query	D	1	chr4	.	CAGCAAGTTTCTCTACCCTTTTAAGCAGATCACTTTTGGAGAGACGCTTCTTCTTTGATTTACC
TP66585_Hit	D+G	1	chr4	.	CTGCATGGTCCTTTTACAGTTTATGTTTACTTGTATGCTTCTGCCGTTTCTTAGTTATTGATGT
TP66585_Query	D+G	1	chr4	.	CTGCACGGTCCTTTTACAGTTTATGTTTACTTGTATGCTTCTGCCGTTTCTTAGTTATTGATGT
TP6745_Hit	D	1	chr4	.	CAGCAATAAATAAGTGTGTGAACTTGAAAGAATACAAATAGTATTGGTAACAATAGGAGAAAAA
TP6745_Query	D	1	chr4	.	CAGCAATAAATAAGTGTGTGAACTTGAAAGAATACAAATAGTATTGGTAACAATAAGAGAAAAA
TP67486_Hit	D	1	chr4	.	CTGCAGAAACCACCTTCAACTTCAGGCATCAACCTCATTTTCAGATAGTTCAGCCAAAATAGAT
TP67486_Query	D	1	chr4	.	CTGCAGAAACCACCTTCAACTTCAGGCATCAACCTCATTTTCAGATAGTTCAGCCAAAAGAGAT
TP67755_Hit	D+G	1	chr4	.	CTGCAGACACAACAATCTTATTCTTATCGTTGTTGTTATTATTCTCTTGATAAGGTCCAAGTGG
TP67755_Query	D+G	1	chr4	.	CTGCAGACACAACAATCTTATTCTTATCGTTGTTGCTATTATTCTCTTGATAAGGTCCAAGTGG
TP6780_Hit	D	1	chr4	.	CAGCAATAACCCTCATTTCAAATCTTGCTGATTTAAAAGTTGAATGTTGTTTTGGTCGTGCTCC
TP6780_Query	D	1	chr4	.	CAGCAATAACCCTCATTTCAAATCTTGCTGATTTAAAAGTTAAATGTTGTTTTGGTCGTGCTCC
TP68103_Hit	D	1	chr4	.	CTGCAGATCACCCACCAGTACTTCAGAAATTTCAAACAAGTTCTGACAACTGTCTTTCGAACTT
TP68103_Query	D	1	chr4	.	CTGCAGATCACCCACCAGTACTTCAGAAATTTCAAACAAGTTCTGACAACTGTCTTTCAAACTT
TP68124_Hit	D	1	chr4	.	CTGCAGATCGGAAACAGAAGGCGAGCGTCTTCTCAAGGATAAGCTTTCCTTCTGAGGAAGAAGG
TP68124_Query	D	1	chr4	.	CTGCAGATCGGAAACAGAAGGCGAGCGTCTTCTCAAGGATAAGCTTTCCTTCTGAGGAAGAAGC
TP68819_Hit	D	1	chr4	.	CTGCAGATGTTGTCTTTATTTTTAACATGAAATTTCTTTGTAAATGGCATCGTTGATTTTTACT
TP68819_Query	D	1	chr4	.	CTGCAGATGTTGTCTTTATTTTTAACATGAAATTTCTTGGTAAATGGCATCGTTGATTTTTACT
TP69004_Hit	D+G	1	chr4	.	CTGCAGGACAAAAAACACATTGTTGGTATGACAGGTGATGGTGTGAATGATGCACCTGCACTGA
TP69004_Query	D+G	1	chr4	.	CTGCAGGACAAAAAACACATTGTTGGTATGACTGGTGATGGTGTGAATGATGCACCTGCACTGA
TP69109_Hit	D	1	chr4	.	CTGCAGGATTGTTTGCAGGATCAATAGTGCTGATGCGAAATTATGGTGACCTTATGGCAATCTG
TP69109_Query	D	1	chr4	.	CTGCAGGATTGTTTGCAGGATCAATAGTGCTGATGCGAAATTATGGTGACCTCATGGCAATCTG
TP69234_Hit	D+G	1	chr4	.	CTGCAGGGAGCAGTAAGGGGAGAGCTGGGAGAGGAAAGGGAGCTTTGCGATCAAACAGAACTTC
TP69234_Query	D+G	1	chr4	.	CTGCAGGGAGCAGTAAGGGAAGAGCTGGGAGAGGAAAGGGAGCTTTGCGATCAAACAGAACTTC
TP69247_Hit	D	1	chr4	.	CTGCAGGGCAAGCTGTAGAAGGGATTGCGCGACAACCGGAAGCTGAAAAAAAAAAAAAAAAAAA
TP69247_Query	D	1	chr4	.	CTGCAGGGCAAGCTGTAGAAGGGATTGCGCGACAACCGGAAGCAGAAAAAAAAAAAAAAAAAAA
TP69303_Hit	D+G	1	chr4	.	CTGCAGGGTTATTTTGTTGTTGCTGGTCATGAATAGAAACAAGGCCAGCAAAAGAGAATGAATC
TP69303_Query	D+G	1	chr4	.	CTGCAGGGTGATTTTGTTGTTGCTGGTCATGAATAGAAACAAGGCCAGCAAAAGAGAATGAATC
TP69345_Hit	D+G	1	chr4	.	CTGCAGGTACTACATGTGCATTCTTTGGCCCGGTAAAAGCTGAAAAGGCATAACAATCAGCAAA
TP69345_Query	D+G	1	chr4	.	CTGCAGGTACTACATGTGCATTCTTTGGCCCAGTAAAAGCTGAAAAGGCATAACAATCAGCAAA
TP69703_Hit	D	1	chr4	.	CTGCAGTCCACATGGCATATTTCACCTGTCTGATCCAGGTGGTGTGTCTGTAATTCGTAACTGT
TP69703_Query	D	1	chr4	.	CTGCAGTCCACATGGAATATTTCACCTGTCTGATCCAGGTGGTGTGTCTGTAATTCGTAACTGT
TP69837_Hit	D	1	chr4	.	CTGCAGTGCCTGGAATGCAACTCACAAAGCCGGTGTACACGAGTTCGAACTTCAAGTCCTATTT
TP69837_Query	D	1	chr4	.	CTGCAGTGCCTGGAATGCAACTCACAAAGCCGGTGTACACGAGTCCGAACTTCAAGTCCTATTT
TP69897_Hit	D	1	chr4	.	CTGCAGTGTACAAGAAACACCACCCTCCAAGTCTGTCGGATGAAGTTTGGAGACTGGAAAAGAT
TP69897_Query	D	1	chr4	.	CTGCAGTGTACAAGAAACACCACCCTCCAAGTCTGTCGGATGAAATTTGGAGACTGGAAAAGAT
TP7014_Hit	D	1	chr4	.	CAGCAATAGCGGCAACAATTTGCGGTTGAAAAGCAGTGCCATTGCGGCTCAATCCTGCTACTGT
TP7014_Query	D	1	chr4	.	CAGCAATAGCGGCAACAATTTGCGGTTGAAAAGCAGTGCCATTGCGGCGCAATCCTGCTACTGT
TP70152_Hit	D+G	1	chr4	.	CTGCAGTTGTTGCTCCGAGGTGTTTTGTTGAGACATTTGAGTCGCTGAAAGATTTGGTTGCATC
TP70152_Query	D+G	1	chr4	.	CTGCAGTTGTTGCTCCGAGGTGTTTTGTTGAGACATTTGAGTCACTGAAAGATTTGGTTGCATC
TP70201_Hit	D+G	1	chr4	.	CTGCAGTTTCTGTCTTGGATAGTTGTCATAAGACATTGTCTGAATCAGTTTCTGCTAAAGCTGG
TP70201_Query	D+G	1	chr4	.	CTGCAGTTTCTGTCTTAGATAGTTGTCATAAGACATTGTCTGAATCAGTTTCTGCTAAAGCTGG
TP7035_Hit	D+G	1	chr4	.	CAGCAATAGTACTGTTAAGGTCACCGACTTCCCACGTTCACCCCCACATTCTGTAGGGTACAAT
TP7035_Query	D+G	1	chr4	.	CAGCAATAGTACTGTTAAGGTCACCGACTTACCACGTTCACCCCCACATTCTGTAGGGTACAAT
TP70477_Hit	D	1	chr4	.	CTGCGTACAGAGAACCCATGAACGTAAGCGAAAGAGCACCAACTTCATCACAAGTACCACACTC
TP70477_Query	D	1	chr4	.	CTGCATACAGAGAACCCATGAACGTAAGCGAAAGAGCACCAACTTCATCACAAGTACCACACTC
TP70554_Hit	D	1	chr4	.	CTGCATAGACAACTGAGGATAAGGGGCTAGGTTACTGACTTGAGACTGATCTAACCTTGAGGAA
TP70554_Query	D	1	chr4	.	CTGCATAGACAACTGAGGATAAGGGGCTAGGTTACTGACTTGAGACTGATCCAACCTTGAGGAA
TP70615_Hit	D	1	chr4	.	CTGCATAGTATCGCCGTCAGGGGAAGCAAGCATGGAAATTTGCTCAAGTGTTGCATTTGCATGC
TP70615_Query	D	1	chr4	.	CTGCATAGTATCGCCATCAGGGGAAGCAAGCATGGAAATTTGCTCAAGTGTTGCATTTGCATGC

Name	Filter	Nb hit (Mt4.0)	Mt Chr (Mt4.0)	Ms Chr (Li et al., 2014)	Sequence
TP70880_Hit	D+G	1	chr4	.	CTGCATCAAAGGATGAGTTGCAATCTAGGGTTGCACAACTGAAGAAAGAGTTGTTTGAGACAGA
TP70880_Query	D+G	1	chr4	.	CTGCATCAAAGGATGAGTTGCAATCCAGGGTTGCACAACTGAAGAAAGAGTTGTTTGAGACAGA
TP71056_Hit	D	1	chr4	.	CTGCATCAGGGGCTTGATTCGACAGATCAAAATCAGCAATCTTTGCAATGCCGTCCTCAAATAG
TP71056_Query	D	1	chr4	.	CTGCATCAGGGGCTTGATTCGACAGATCAAAATCAGCAATCTTTGCAACGCCGTCCTCAAATAG
TP71174_Hit	D+G	1	chr4	.	CTGCATCCATGACTTTCTCACAGCCATCCGCTCAGCGAAATGGTTTCCAGCTGATAGGATGCCG
TP71174_Query	D+G	1	chr4	.	CTGCATCCACGACTTTCTCACAGCCATCCGCTCAGCGAAATGGTTTCCAGCTGATAGGATGCCG
TP71185_Hit	D	1	chr4	.	CTGCATCCAGGAGCATGTATACTAGTAGTTGGAAAGCAGGGTCCCCCAATTTTTGCCTTGTACT
TP71185_Query	D	1	chr4	.	CTGCATCCAGGAGCAAGTATACTAGTAGTTGGAAAGCAGGGTCCCCCAATTTTTGCCTTGTACT
TP71252_Hit	D	1	chr4	.	CTGCATCCGCAAATCCTTTTATCTTAGCGATTACATGTAATCCAAGCTCGTGTGCCTTCTTTTC
TP71252_Query	D	1	chr4	.	CTGCATCCGCAAATCCTTTTATCTTAGCGATTACATGTAATCCAAGCTCGTATGCCTTCTTTTC
TP71311_Hit	D	1	chr4	.	CTGCATCGACGAATCAGGTGAAGGAAATTAACGGTTTCGTTGGTGAAGATGGTTCTGACTCTTC
TP71311_Query	D	1	chr4	.	CTGCATCGACGAACCAGGTGAAGGAAATTAACGGTTTCGTTGGTGAAGATGGTTCTGACTCTTC
TP71441_Hit	D	1	chr4	.	CTGCATCTCCTCAAATTTCACATAATTCATCTCCCCAAGTTGACCAACAAACTCATCTCCCGTC
TP71441_Query	D	1	chr4	.	CTGCATCTCCCCAAATTTCACATAATTCATCTCCCCAAGTTGACCAACAAACTCATCTCCCGTC
TP7169_Hit	D	1	chr4	.	CAGCAATATGAAGTGCATCAAAACCATTTCTTGCTTTAATTCCAGCATCAGCAAGATCATAATA
TP7169_Query	D	1	chr4	.	CAGCAATATGAAGTGCATCAAAACCATTTCTAGCTTTAATTCCAGCATCAGCAAGATCATAATA
TP71942_Hit	D	1	chr4	.	CTGCATGGCATCAATCATATACAAACCTTTGAGTGAATTTTCACCAACGTTTCTAAGTTCATGC
TP71942_Query	D	1	chr4	.	CTGCATGGCATCAATCATATACAAACCTTTGAGTGAATTTTCACCAACGTTCCTAAGTTCATGC
TP72036_Hit	D+G	1	chr4	.	CTGCATGTAATTTAATATTTCTTTTCTAGCATTTGATAAACCGTTTTCTGTTGAATGTGCATAG
TP72036_Query	D+G	1	chr4	.	CTGCATGTAATTTAATATTTCTTTTCTAGCATTTGATAAAACGTTTTCTGTTGAATGTGCATAG
TP72226_Hit	D	1	chr4	.	CTGCATTAAACATGAAATAGCTCATTAGTTCACTTATCCTTAGGGATGTAGCAAGCATAAACAC
TP72226_Query	D	1	chr4	.	CTGCATTAAACATGAAATAGCTCATTAGTTCACTTATCCTTAGGGATGGAGCAAGCATAAACAC
TP7252_Hit	D	1	chr4	.	CAGCAATATTTTTGTTGGAAAATTTCATCAATCATTTTTCTTCTATAGTGCACTTGACAAAGCC
TP7252_Query	D	1	chr4	.	CAGCAATATTTTGGTTGGAAAATTTCATCAATCATTTTTCTTCTATAGTGCACTTGACAAAGCC
TP72574_Hit	D	1	chr4	.	CTGCATTGAAATTGGACATTCTTCATCTGACAAAAGCTCTTGGAAGCAACATCCAATTAGATGG
TP72574_Query	D	1	chr4	.	CTGCATTGAAACTGGACATTCTTCATCTGACAAAAGCTCTTGGAAGCAACATCCAATTAGATGG
TP72769_Hit	D	1	chr4	.	CTGCATTGTATCGATTCTCGGATGGAGAAACTGCTCTAAACACCGCAGACACCTTAGTGGAACC
TP72769_Query	D	1	chr4	.	CTGCATTGTATCGATTCTCGGATGGAGAAACCGCTCTAAACACCGCAGACACCTTAGTGGAACC
TP72789_Hit	D	1	chr4	.	CTGCATTGTGGGGATTATCACTGTTGGTATTTGAAGGTGCATGAGCAGAAGAATCCATGTTGAA
TP72789_Query	D	1	chr4	.	CTGCATTGTGGGGATTATCACTGTTGGTATTTGAAGGTGCATGAGCAGAAAAAATCCATGTTGAA
TP73005_Hit	D	1	chr4	.	CTGCATTTGAGGATGAGGTTGCTGATAAGGAGGGATTTGAGTATGTTGTTGAGGTTGAGGTTGC
TP73005_Query	D	1	chr4	.	CTGCATTTGAGGATGAGATTGCTGATAAGGAGGGATTTGAGTATGTTGTTGAGGTTGAGGTTGC
TP73056_Hit	D	1	chr4	.	CTGCATTTGGTATAATCATAGTTAAGGCGCTTTGGAATTTGCATAAAAGAAATATGTAAGTGGT
TP73056_Query	D	1	chr4	.	CTGCATTTGGTATAATCATAGTTAACGCGCTTTGGAATTTGCATAAAAGAAATATGTAAGTGGT
TP73520_Hit	D	1	chr4	.	CTGCCAAATTGCGAGATTACATCCACAAGACACCCGTAGCCACCAACGGCAATAACCTGAAACG
TP73520_Query	D	1	chr4	.	CTGCCAAATTGCGAGATTACATCCACAAGACATCCGTAGCCACCAACGGCAATAACCTGAAACG
TP73797_Hit	D+G	1	chr4	.	CTGCCAAGTTACTGAAGAAGTCTTATTTTAGTGGAGGTGTGTGGCTTAGTGGTTACTTGCCTCT
TP73797_Query	D+G	1	chr4	.	CTGCCAAGTTACTGAAGAAGTCTTATTTTAGTGGAGGTGTGTGCCTTAGTGGTTACTTGCCTCT
TP73905_Hit	D+G	1	chr4	.	CTGCCAATGCATTGAAGGCCGGAGATCTTCCTCTCACACGAACACGGTCAGGACTAACAGACGT
TP73905_Query	D+G	1	chr4	.	CTGCCAATGCATTGAAGGCCGGAGATCTTCCTCTCACACGAACACGGTCAGGACTAACAGACAT
TP74007_Hit	D	1	chr4	.	CTGCCACAAACCACGTGAATTTTCAGCCCTGGTTTGAACAGCACGTAGTTTAGACGCTGAAAAA
TP74007_Query	D	1	chr4	.	CTGCCACAAACCACGTGAATTTTCAGCCCTGGTTTGAACAGCACGTAGTTTAGACGCAGAAAAA
TP7407_Hit	D	1	chr4	.	CAGCAATCCATGTATTTTGCATTCAGATGGCAGATAACTCAATTATTCGTTTCCCTACATCATA
TP7407_Query	D	1	chr4	.	CAGCAATCCATATATTTTGCATTCAGATGGCAGATAACTCAATTATTCGTTTCCCTACATCATA
TP74197_Hit	D	1	chr4	.	CTGCCACATGGAAAAGAAAAGATTAATTGGTTGGTTGTGTAATCCTACAAATTAGATGAAAGAT
TP74197_Query	D	1	chr4	.	CTGCCACATGGAAAAGAAAAGATTAATTGGTTGGCTGTGTAATCCTACAAATTAGATGAAAGAT
TP74235_Hit	D	1	chr4	.	CTGCTACCAATGTTATATTTGGCCTTGCCTTGGGATACAAGTCTGTTATCATTCCAATTTTTGC
TP74235_Query	D	1	chr4	.	CTGCCACCAATGTTATATTTGGCCTTGCCTTGGGATACAAGTCTGTTATCATTCCAATTTTTGC
TP744_Hit	D+G	1	chr4	.	CAGCAAAAGTAAGCTTACAATTGCTAAATTTAAATCTGAATCTTCACTGGATGGATGGGAAGTT
TP744_Query	D+G	1	chr4	.	CAGCAAAAGTAAGCTTACAATTGCTAAATTTAAATCTGAATCTTCACTAGATGGATGGGAAGTT
TP74488_Hit	D	1	chr4	.	CTGCCACTCCAACTTCAATCACAACACCTGGTGTCAATCTTAAGTTACATTTCACAAAGATGGA
TP74488_Query	D	1	chr4	.	CTGCCACTCCAACTTCAATCACAACAACTGGTGTCAATCTTAAGTTACATTTCACAAAGATGGA
TP74513_Hit	D	1	chr4	.	CTGCCACTGAGTAAAAGGAGGGATGGCATGAATATTTTAAAGTCAGAGATAGTAGATTTACGGT
TP74513_Query	D	1	chr4	.	CTGCCACTGAGTAAAAGGAGGGATGGCATGAATATTTTAAAATCAGAGATAGTAGATTTACGGT

Name	Filter	Nb hit (Mt4.0)	Mt Chr (Mt4.0)	Ms Chr (Li et al., 2014)	Sequence
TP74683_Hit	D+G	1	chr4	.	CTGCCAGAGAGAGTTTCAAGTACTTATTGGTGTTTTATTCTTCTTAGAACAACACTTGTTGCAT
TP74683_Query	D+G	1	chr4	.	CTGCCAGAGAGAGTTTCAAGTACTTATTGGTGTTTTATTCTTCTTAGAACAACACTGGTTGCAT
TP74721_Hit	D+G	1	chr4	.	CTGCCAGATGCCGGGGCAAGGTGATTGGCTGACATTAGATTATTGCAATGATACTAATTGGCTA
TP74721_Query	D+G	1	chr4	.	CTGCCAGATGCCGGGGCAAGGTGATTGGCTGACATTAGATTATTGCAATAATACTAATTGGCTA
TP74844_Hit	D	1	chr4	.	CTGCCAGGGACTGCATATTTTTGCTGTTTTACTGTCTTACCTCTTAATATGAATTAATAACACT
TP74844_Query	D	1	chr4	.	CTGCCAGGGACTGCATATTTTTGCTGTTTTACTGTCTTAACTCTTAATATGAATTAATAACACT
TP74853_Hit	D+G	1	chr4	.	CTGCCAGGTCACTAGATGCGAACACAATGACTACTTTCCATGATCTTATTCCTACTAAGCTTGC
TP74853_Query	D+G	1	chr4	.	CTGCCAGGTCACTAGATGCGAACACAATGACTACTTTCCATGATCTTATTCCTACTAAGCGTGC
TP7492_Hit	D	1	chr4	.	CAGCAATCTCAGTACACTTGAAGAAGCATTTCTCTGTGATTATGTGAGTCTAAGACATGTTTTA
TP7492_Query	D	1	chr4	.	CAGCAATCTCAGTACACTTCAAGAAGCATTTCTCTGTGATTATGTGAGTCTAAGACATGTTTTA
TP75202_Hit	D+G	1	chr4	.	CTGCTATCAGCAACACCAAAATTTGTACCCTGATTTTCCTGCATCCTAATTTTATCACTGTCCT
TP75202_Query	D+G	1	chr4	.	CTGCCATCAGCAACACCAAAATTTGTACCCTGATTTTCCTGCATCCTAATTTTATCACTGTCCT
TP75241_Hit	D	1	chr4	.	CTGCCGTCCAGTTGTCATTGTCTTCTCTGACGGTCTAATTTGACTGGTCATCACCTTCTCATTC
TP75241_Query	D	1	chr4	.	CTGCCATCCAGTTGTCATTGTCTTCTCTGACGGTCTAATTTGACTGGTCATCACCTTCTCATTC
TP75381_Hit	D	1	chr4	.	CTGCCTTGATCATTTGTGTGCTTTACCTTTACCAGGATGGTTTGCCAACATTTCCTGACGAGGA
TP75381_Query	D	1	chr4	.	CTGCCATGATCATTTGTGTGCTTTACCTTTACCAGGATGGTTTGCCAACATTTCCTGACGAGGA
TP75952_Hit	D	1	chr4	.	CTGCCCACTTCAACATATTTATAGGAAAACCACCTTAAAAGACCACATACTTTGCAACAAATAG
TP75952_Query	D	1	chr4	.	CTGCCCACTTCAACATATTTATAAGAAAACCACCTTAAAAGACCACATACTTTGCAACAAATAG
TP7687_Hit	D	1	chr4	.	CAGCAATGATACTCATCAGCAAATTGTTGGTGTGGCAAATTATCATGTTAGTGCACATATTGAT
TP7687_Query	D	1	chr4	.	CAGCAATGATACTCATAAGCAAATTGTTGGTGTGGCAAATTATCATGTTAGTGCACATATTGAT
TP7690_Hit	D	1	chr4	.	CAGCAATGATAGCTATGCTCTTCTTGGATAGAGAATGATCGTTCTCTCTGCATGGACTTAGGAA
TP7690_Query	D	1	chr4	.	CAGCAATGATAGCTATGCTCTTCTTGGATAGAGAATGATCATTCTCTCTGCATGGACTTAGGAA
TP76933_Hit	D	1	chr4	.	CTGCCCTTTCCCGAGCTATGACTGTGTGTTTTAAATTTATTTAAGAACAATCTATTTTCCATGC
TP76933_Query	D	1	chr4	.	CTGCCCTTTCCCGAGCTATGACTGTATGTTTTAAATTTATTTAAGAACAATCTATTTTCCATGC
TP77317_Hit	D	1	chr4	.	CTGCCGCCCGGGAATTGCATCCCTTAGCAAAGTGTTTTTCTTTGAAACAGCTGATTCAAGTTCT
TP77317_Query	D	1	chr4	.	CTGCCGCCCAGGAATTGCATCCCTTAGCAAAGTGTTTTTCTTTGAAACAGCTGATTCAAGTTCT
TP7746_Hit	D	1	chr4	.	CAGCAATGCACCGGTAAACTTATGAGGAAACATAAAAAGAACTGAAACTGTATACTTCATTACA
TP7746_Query	D	1	chr4	.	CAGCAATGCACCAGTAAACTTATGAGGAAACATAAAAAGAACTGAAACTGTATACTTCATTACA
TP77843_Hit	D	1	chr4	.	CTGCCGTACCTTTAGCATTTCCTAGCACTTCTGCATTGTCGACAAAAGCACCAGCCGCTGTACT
TP77843_Query	D	1	chr4	.	CTGCCGTACCTTTAGCAATTCCTAGCACTTCTGCATTGTCGACAAAAGCACCAGCCGCTGTACT
TP78163_Hit	D	1	chr4	.	CTGCCTACGGATGTTAGTGCCGTATTAATATGGCTCTACATTATTCTGTAATGGAAAAGATTAG
TP78163_Query	D	1	chr4	.	CTGCCTACGGATGTTAGTGCCGTATTAATATGGCTCTACATTATTCTGTAATGGAAAAGATGAG
TP7863_Hit	D+G	1	chr4	.	CAGCAATGGCAAGTTATAGTGCCCATTTCATTAATATTTGTCGATGTGGGTGCATATCTGGCAG
TP7863_Query	D+G	1	chr4	.	CAGCAATGGCAAATTATAGTGCCCATTTCATTAATATTTGTCGATGTGGGTGCATATCTGGCAG
TP78810_Hit	D	1	chr4	.	CTGCCTGACCTCCCGCCACCATGATGTCGTGGATTCCGATTTCTGATCATGTTCCAAGCTGAAA
TP78810_Query	D	1	chr4	.	CTGCCTGACCTCCCGCCACCATGATGTCGTGGATTCCAATTTCTGATCATGTTCCAAGCTGAAA
TP78925_Hit	D	1	chr4	.	CTGCCTGCTATGTACTATTTGTATCCCCTTCCCTAATGGCTATGTGTCTTTGTCTTGAGAACAG
TP78925_Query	D	1	chr4	.	CTGCCTGCTATGTACTATTTGTATCCCCTCCCCTAATGGCTATGTGTCTTTGTCTTGAGAACAG
TP78928_Hit	D+G	1	chr4	.	CTGCCTGCTCCTGCCACTTCTGAGCGCAAGGCTATGTTGAAGCATATAATACAAAGGCGACACT
TP78928_Query	D+G	1	chr4	.	CTGCCTGCTCCTGCCACTTCTGAGCGAAAGGCTATGTTGAAGCATATAATACAAAGGCGACACT
TP78960_Hit	D+G	1	chr4	.	CTGCCTGGATCCCACAAGTAGTACCTCCGACAAGAAACCATGTCTTTGATGATCCAAACAATTT
TP78960_Query	D+G	1	chr4	.	CTGCCTGGATCCCACAAGTAGTACCTCCGACAAGAAACCATGTCTCTGATGATCCAAACAATTT
TP7918_Hit	D	1	chr4	.	CAGCAATGGTAATGGCAAATAAATCATTTGAATTTCTGAACATATCATGCTCATAAACATTTAT
TP7918_Query	D	1	chr4	.	CAGCAATGGTAATGGCAAACAAATCATTTGAATTTCTGAACATATCATGCTCATAAACATTTAT
TP7933_Hit	D	1	chr4	.	CAGCAATGGTGATTGGTGCTGTGTCATACACATTGTATTCAGTTTTACTACTAGGGAGAGAGAG
TP7933_Query	D	1	chr4	.	CAGCAATGGTGATTGGTGCTGTGTCATACACATTGTATTCAGTTTTACTACTAGGGAGAGAGAA
TP79421_Hit	D	1	chr4	.	CTGCCTTGTGAAGAAGTTCAGCCCTCTTCCAAAGTGGGGTCTTTGCCCATGACTTTTGTGCTGA
TP79421_Query	D	1	chr4	.	CTGCCTTGTGAAGAAGTTCAGCCCTCTTCCAAAGTGGGGTCTTTGCCCATGACTTTGGTGCTGA
TP79464_Hit	D	1	chr4	.	CTGCCTTTATAGAGGTGGTAGAGCTAAGTTAAGCCAGGGGTGGTATGAGTTTTCGCTGGAGAAC
TP79464_Query	D	1	chr4	.	CTGCCTTTATAGAGGTGGTAGAGCTAAGTTAAGCCAGGGGTGGTATGAATTTTCGCTGGAGAAC
TP79474_Hit	D	1	chr4	.	CTGCCTTTCTTAGTATATGTTTTTACTATGCTTGTATGTTTCACTCAGGTTCTTGAAGGGGTAA
TP79474_Query	D	1	chr4	.	CTGCCTTTCATAGTATATGTTTTTACTATGCTTGTATGTTTCACTCAGGTTCTTGAAGGGGTAA
TP79580_Hit	D	1	chr4	.	CTGCCTTTTGATGACGCACCAGGGTTAGCTATGGCTAGGGCGTTTGGAGATTTCTGCTTGAAGG
TP79580_Query	D	1	chr4	.	CTGCCTTTTGATGACGCACCAGGGTTAGCAATGGCTAGGGCGTTTGGAGATTTCTGCTTGAAGG

Name	Filter	Nb hit (Mt4.0)	Mt Chr (Mt4.0)	Ms Chr (Li et al., 2014)	Sequence
TP797_Hit	D+G	1	chr4	.	CAGCAAATGTTTTTATGTAGGATTGCTTCCATAGGTCTAAACCATGAATATTTTTGTCAGCTTC
TP797_Query	D+G	1	chr4	.	CAGCAAAAGTTTTTATGTAGGATTGCTTCCATAGGTCTAAACCATGAATATTTTTGTCAGCTTC
TP7984_Hit	D	1	chr4	.	CAGCAATGTATGTGTGTCATCAAGGGCTATGAATTAAGGAATGATAGTGTAAGTGGGTCCTACA
TP7984_Query	D	1	chr4	.	CAGCAATGTATGTGTGTCATCAAGAGCTATGAATTAAGGAATGATAGTGTAAGTGGGTCCTACA
TP79937_Hit	D	1	chr4	.	CTGCGACAAAAACAATAAGGAAGGGAGTACCAAAAATAACTTCCAGCAATTCTATCTCAAAGTT
TP79937_Query	D	1	chr4	.	CTGCGACAAAAACAATAAGGAAGGGAGTACCAAAAATAACTTCAAGCAATTCTATCTCAAAGTT
TP80196_Hit	D	1	chr4	.	CTGCGACTTGCTTGTACCAGGATCACCAACAAGGAGAATATTTATATCACCACGAAAGCTAGCG
TP80196_Query	D	1	chr4	.	CTGCGACTTGCTTGTACCAGGATCACCAACAAGAAGAATATTTATATCACCACGAAAGCTAGCG
TP80319_Hit	D+G	1	chr4	.	CTGCGAGCTCCCGCGAGAGAAGTCTTCCGGAAAGATTTTATATTGTTCAGTGCTTCTGCAACTC
TP80319_Query	D+G	1	chr4	.	CTGCGAGCTCCCACGAGAGAAGTCTTCCGGAAAGATTTTATATTGTTCAGTGCTTCTGCAACTC
TP80377_Hit	D	1	chr4	.	CTGCGAGGTTTTATATTATAGAAGCACCATGGTTTTAAACTGCGGTTGCAGTTTGATGCAAATG
TP80377_Query	D	1	chr4	.	CTGCGAGGTTTTATATTATAGAAACACCATGGTTTTAAACTGCGGTTGCAGTTTGATGCAAATG
TP8045_Hit	D+G	1	chr4	.	CAGCAATGTGTATTTGAATTTGAAAGATCTTACATGTCAAAATCCCTCGAGCAATAAATTTTAG
TP8045_Query	D+G	1	chr4	.	CAGCAATGTGTATTTGAATTTGAAAGATCTTACATGTCAAAATCCCCCGAGCAATAAATTTTAG
TP80452_Hit	D	1	chr4	.	CTGCGATAACTAGTTTTAATTCGAAGCTACCTTATGGTTGTGGACATTGTCTCATGTTAGATAT
TP80452_Query	D	1	chr4	.	CTGCGATAACTAGTTTTAATTCGAAGCTACCTTATGGTTGTGGACATCGTCTCATGTTAGATAT
TP80825_Hit	D	1	chr4	.	CTGCGCAACCAGTTGTCTTGATAATTTGTCCCTCTTTTCTGTTTTAGGTTCAATATGACTTTTC
TP80825_Query	D	1	chr4	.	CTGCGCAACCAGTTGTCTTGATAATTTGCCCCTCTTTTCTGTTTTAGGTTCAATATGACTTTTC
TP81205_Hit	D	1	chr4	.	CTGCGTCGAAAAAACCAGCCCTTACACCGAAGGCTATAATACACCAAAAGTTTGGTAAAAATGC
TP81205_Query	D	1	chr4	.	CTGCGCCGAAAAAACCAGCCCTTACACCGAAGGCTATAATACACCAAAAGTTTGGTAAAAATGC
TP81366_Hit	D	1	chr4	.	CTGCGCGAGATGCGTTTCCTAGAGATTCATCACTTTCACTGAGATCATTTGGCAGTGGCAGTGG
TP81366_Query	D	1	chr4	.	CTGCGCGAGATGCATTTCCTAGAGATTCATCACTTTCACTGAGATCATTTGGCAGTGGCAGTGG
TP81597_Hit	D	1	chr4	.	CTGCGCTGTGACCCTCACGAGCCTCTGGCCCTTCTCCTCTTATAATTGGAGAATCCCAAGTTTG
TP81597_Query	D	1	chr4	.	CTGCGCTATGACCCTCACGAGCCTCTGGCCCTTCTCCTCTTATAATTGGAGAATCCCAAGTTTG
TP81900_Hit	D+G	1	chr4	.	CTGCGGACATTTCTTCTGGAGTGTTGGTGAGTTTTACCTTACACTTTTTCTTTTATGTTGTGGT
TP81900_Query	D+G	1	chr4	.	CTGCGGACATTTCTTCTGGAGTGTTGATGAGTTTTACCTTACACTTTTTCTTTTATGTTGTGGT
TP81954_Hit	D	1	chr4	.	CTGCGGAGGAAGGAGAGGGTTGTTAAGAGAAGAAGTGCTGAAACGACTGCGCAGAGAAGGCTGA
TP81954_Query	D	1	chr4	.	CTGCGGAGGAAGGAGAGGGTTGTTAAGAGAAGAAGTGCTGAAACGACGGCGCAGAGAAGGCTGA
TP81971_Hit	D	1	chr4	.	CTGCGGAGGGTAGAATTGAGCGTAGAGGGTTGCCGGCAAAGGCATTAAAGTTGAAGTTTATGGA
TP81971_Query	D	1	chr4	.	CTGCGGAGGGTAGAATTGAGCGTAGAGGGTTGCCGGCAAAGGCATTAAAGTTCAAGTTTATGGA
TP81994_Hit	D	1	chr4	.	CTGCGGAGTGTGTTCAAATAGCATTGGGTCATTGCTCATTATTAGAAGCTCGTGGTCTAGCTCT
TP81994_Query	D	1	chr4	.	CTGCGGAGTGTGTTCAAATAGCATTGGGCCATTGCTCATTATTAGAAGCTCGTGGTCTAGCTCT
TP82255_Hit	D	1	chr4	.	CTGCGGCGCGCCTGTGAGACTCGACCTAATTTCACATTAAAAGAATTAATTCACCTTTATAATC
TP82255_Query	D	1	chr4	.	CTGCGGCGCGCCTGTGAGACTCGACCTAATTTCACATTAAAAGAATTAATTCACCTATATAATC
TP82264_Hit	D	1	chr4	.	CTGCGGCGGAAACTACAACGCAGATGAATTTTGGATTGGCTGTGATATTTGTGAGAGGTGGTAT
TP82264_Query	D	1	chr4	.	CTGCGGCGGAAACTACAACGCAGATGAATTTTGGATTGGCTGTGATATTTGTGAGAGGGGGTAT
TP82321_Hit	D+G	1	chr4	.	CTGCGGCTAATACGCCTGTGAAGCGTCCAAAATTTGGAATTATCAAACTTCATTTGGGGAACAA
TP82321_Query	D+G	1	chr4	.	CTGCGGCTAAGACGCCTGTGAAGCGTCCAAAATTTGGAATTATCAAACTTCATTTGGGGAACAA
TP82332_Hit	D	1	chr4	.	CTGCGGCTAGTTCACATGCTTCGCATTATTGGTTCTGGTGTGGTGGGTGATAGATGGTTAATCT
TP82332_Query	D	1	chr4	.	CTGCGGCTAGTTCACATGCTTCGCATTATTGGTTCTGGTGTGGTGGGGGATAGATGGTTAATCT
TP82541_Hit	D	1	chr4	.	CTGCGGGGATGGCCTGATGGAAAGCTAAACCGGAAGCATTATTATGAAACTGTTGTTGGGTGAA
TP82541_Query	D	1	chr4	.	CTGCGGGGATGGCCTGATGGAAAGCTAAACCGGAAGCATTATTATGAAACTGTTGTTGGGCGAA
TP8259_Hit	D	1	chr4	.	CAGCAATTCAAGAAGCTGGGAGTGCAACTCTTCATCCCTCAAGGTTTCAATTCTCCTGGAAATA
TP8259_Query	D	1	chr4	.	CAGCAATTCAAGAAGCTGGGAGTGCAACTCTTCATCCCTCAAGGTTTCAATTCTCCTAGAAATA
TP82623_Hit	D	1	chr4	.	CTGCGGGTGTCGTTCAAAGCATATATAGAGAGGCAATCAGAGTTGCATCTTTTGTCTACTATAG
TP82623_Query	D	1	chr4	.	CTGCGGGTGTCATTCAAAGCATATATAGAGAGGCAATCAGAGTTGCATCTTTTGTCTACTATAG
TP82639_Hit	D+G	1	chr4	.	CTGCGGGTTTACCTATAGCCGGTCTCACAGCTCGTGATGCACTCACCGAAATTGGAGGAGTCAA
TP82639_Query	D+G	1	chr4	.	CTGCGGGTTTACCTATAGCCGGTCTCACAGCCCGTGATGCACTCACCGAAATTGGAGGAGTCAA
TP8278_Hit	D+G	1	chr4	.	CAGCAATTCCGTTTGTTAGATCTACCATCATTTTGTTTTGTTTCATTTTTACTTTTGAAAAAAA
TP8278_Query	D+G	1	chr4	.	CAGCAATTCAGTTTGTTAGATCTACCATCATTTTGTTTTGTTTCATTTTTACTTTTGAAAAAAA
TP82878_Hit	D+G	1	chr4	.	CTGCGGTTAATGATTGTTGTATTGGGATTCCGTGTTATTTTACTGATCTGCAGAGAAGGGCTGT
TP82878_Query	D+G	1	chr4	.	CTGCGGTTAATGATTGTTGTATTGGGATTCCGTGTTATTTTACTGATCTACAGAGAAGGGCTGT
TP8297_Hit	D	1	chr4	.	CAGCAATTCCACCTCCTTTACTGCTATCTTCTATTATGTGGCTACCTCCTCTGCAATCGTGGTC
TP8297_Query	D	1	chr4	.	CAGCAATTCCACCTCCTTTACTGCTATCTTCTATTATCTGGCTACCTCCTCTGCAATCGTGGTC

Name	Filter	Nb hit (Mt4.0)	Mt Chr (Mt4.0)	Ms Chr (Li et al., 2014)	Sequence
TP82991_Hit	D	1	chr4	.	CTGCGGTTTTTTCTATGCAAGGGTTTGGTATATTGGCTAGTGCAACTGTTACTATGGTGGTTTG
TP82991_Query	D	1	chr4	.	CTGCGGTTTTTTCTATGCAAGGGTTTGGTATATTGGCTAGTGCAACTGTTACTATGGGGGTTTG
TP83258_Hit	D	1	chr4	.	CTGCGTCATGTGGTTTAATGATCTAATTGACATAAGACAGTTTGACACTGGTGGCCAAGATCTC
TP83258_Query	D	1	chr4	.	CTGCGTCATGTGGTTTAATGATCTAATCGACATAAGACAGTTTGACACTGGTGGCCAAGATCTC
TP83395_Hit	D	1	chr4	.	CTGCGTCTAAACTACGTGCTGTTCAAACCAGGGCTGAAAATTCACGTGGTTTGTGGCTGAAAAA
TP83395_Query	D	1	chr4	.	CTGCGTCTAAACTACGTGCTGTTCAAACCAGGGCTGAAAATTCACGTGGTTTGTGGCAGAAAAA
TP83557_Hit	D+G	1	chr4	.	CTGCGTGCTTTTTTTTGCCACATTCACATAAATTCTCTTTAGAAGGTATTCTTCTACCTGTTTC
TP83557_Query	D+G	1	chr4	.	CTGCGTGCTTTTTTTTGCCACATCCACATAAATTCTCTTTAGAAGGTATTCTTCTACCTGTTTC
TP83785_Hit	D	1	chr4	.	CTGCGTTCAAATGCGGGATTTCCTGGTACCCCCATTCGGCTTTTGTGGTGCGGAGATCGGAAGA
TP83785_Query	D	1	chr4	.	CTGCGTTCAAATGCGGGATTTCCTGGTACCCCCATTCGGCTTTTGTGGTGCCGAGATCGGAAGA
TP83786_Hit	D+G	1	chr4	.	CTGCGTTCAAATGCGGGATTTCTTGGTACCCCCATTCGGCTTTTGTGGTGCTGAAAAAAAAAAA
TP83786_Query	D+G	1	chr4	.	CTGCGTTCAAATGCGGGATTTCCTGGTACCCCCATTCGGCTTTTGTGGTGCTGAAAAAAAAAAA
TP83788_Hit	D	1	chr4	.	CTGCGTTCAAATGCGGGATTTCCTGGTACCCCCATTCGGCTTTTGTGGTGCTGTGATCGGAAGA
TP83788_Query	D	1	chr4	.	CTGCGTTCAAATGCGGGATTTCCTGGTACCCCCATTCGGCTTTTGTGGTGCTGGGATCGGAAGA
TP83864_Hit	D	1	chr4	.	CTGCGTTGCCCCTTTCAATGAATTCTTTGACAATCTGTACATCAGAAACTTCAAATTCTTGCAG
TP83864_Query	D	1	chr4	.	CTGCGTTGCCCCTTTCAATGAATTCTTTGACAATCTGTACATCAGAAACTTCAAATTCCTGCAG
TP8388_Hit	D	1	chr4	.	CAGCAATTGAGCTTAACTCACCAAATATGTTGTCATCATCATTTTCAGTGTAGATACTTGAATC
TP8388_Query	D	1	chr4	.	CAGCAATTGAGCTTAACTCACCAAATATGTTGTCATCATCATTTTCAGCGTAGATACTTGAATC
TP8401_Hit	D	1	chr4	.	CAGCAATTGATGTATGGTCTGTTGGTTGTATTTTCATGGAACTGATGGATCGAAAGCCTTTGTT
TP8401_Query	D	1	chr4	.	CAGCAATTGATGTATGGTCTGTTGGTTGTATTTTCATGGAACTGATGGATCGAAAACCTTTGTT
TP84134_Hit	D	1	chr4	.	CTGCTAAAATGCGCCTGTTGCTCTCAGAATCCATCATAAACTCGGTTTCATCTTCATCTCCGGC
TP84134_Query	D	1	chr4	.	CTGCTAAAATGCGCCTGTTGCTCTCAGAATCCATCATAAACTCAGTTTCATCTTCATCTCCGGC
TP84159_Hit	D	1	chr4	.	CTGCTAAACCTCCTAGAGCTCCACGAGGGCCGTCGTTGGGTGCGGAGATCGGAAGAGCGGTTCA
TP84159_Query	D	1	chr4	.	CTGCTAAACCTCCTAGAGCTCCACGAGGGCCGTCGTTGGGTGCCGAGATCGGAAGAGCGGTTCA
TP84418_Hit	D	1	chr4	.	CTGCTAAGATTCTTGCCAACTTGATTGTAATTGGTGGGAGCATCTTGGCAAGAGGAGTTGTTGC
TP84418_Query	D	1	chr4	.	CTGCTAAGATTCTTGCCAACTTGATTGTAATTGGTGGGAGCATCTTGGCAAGAGGAGTTGGTGC
TP84621_Hit	D	1	chr4	.	CTGCTAATGCTAGTGAGCGAACCTTTAAATCTGATGGGACTGAAAAGAGAACATCTACCAGCTT
TP84621_Query	D	1	chr4	.	CTGCTAATGCTAGTGAGCGAACATTTAAATCTGATGGGACTGAAAAGAGAACATCTACCAGCTT
TP84669_Hit	D	1	chr4	.	CTGCTAGTTCCATCAACCTCTCGTAGTCTATCTCGGTCGACTTAAACTTGAACTTGATGCCAAC
TP84669_Query	D	1	chr4	.	CTGCTAATTCCATCAACCTCTCGTAGTCTATCTCGGTCGACTTAAACTTGAACTTGATGCCAAC
TP84728_Hit	D	1	chr4	.	CTGCTACAAAAATCAAATCTTCATTCCAAAGTGGAGTAGTTGTTCTAGTGGAACAAATCTTGGT
TP84728_Query	D	1	chr4	.	CTGCTACAAAAACCAAATCTTCATTCCAAAGTGGAGTAGTTGTTCTAGTGGAACAAATCTTGGT
TP84784_Hit	D+G	1	chr4	.	CTGCTACAATGCGTCTTCTACAGCTAAACATTAGTGGAATGGTTTATATGCGCTTCATGTAATT
TP84784_Query	D+G	1	chr4	.	CTGCTACAATGCGTCTTCTACAACTAAACATTAGTGGAATGGTTTATATGCGCTTCATGTAATT
TP84850_Hit	D	1	chr4	.	CTGCTACAGAATGAGCAACCTCCATCTCTGTGTCATTCTCGAGAAATACAGCACGCTGAAAAAA
TP84850_Query	D	1	chr4	.	CTGCTACAGAATGAGCAACCTCCATCTCCGTGTCATTCTCGAGAAATACAGCACGCTGAAAAAA
TP85091_Hit	D	1	chr4	.	CTGCTACGCCTCAATCTCCTCCGAGACTCATCATCAAGTTCTTAATGACAACACAATCTCCTCC
TP85091_Query	D	1	chr4	.	CTGCTACGCCTCAATCTCCTCCGAGACTCATCGTCAAGTTCTTAATGACAACACAATCTCCTCC
TP85178_Hit	D	1	chr4	.	CTGCTACTAGGTAAAATTTATACCAGAATCTGAATCTCCTAGCTTGTCTATACGGTCATTATGT
TP85178_Query	D	1	chr4	.	CTGCTACTAGGTAAAATTTATACCAGAATCTGAATCTCCTAGCTTGTCTATACGGTCATTATGC
TP85224_Hit	D+G	1	chr4	.	CTGCTACTCCGCAACTGATAAACTCCTGCCAACCGCTGTACCTATATTCATGGAGAACTGAGAT
TP85224_Query	D+G	1	chr4	.	CTGCTACTCCGCAACTGATAAACTCCTGCCAACCGCTGTACCTATATTCATGGAGAACCGAGAT
TP85317_Hit	D	1	chr4	.	CTGCTACTGTCCTTAGTAAATGCCTATGCAAAACAACCTGGGCATCAAATACACATTAATTTAC
TP85317_Query	D	1	chr4	.	CTGCTACTGTCCTTAGTAAATGCCTATGCAAAACAACCTGGGCATCAAAAACACATTAATTTAC
TP85358_Hit	D+G	1	chr4	.	CTGCTACTTAGGTGCTGGGAGGACTGGATGTTGCAATTCTCTGGCTTACAGGGCCTTGCGCTTC
TP85358_Query	D+G	1	chr4	.	CTGCTACTTAGGTGCTGGGAGGACTGGATGTTGCAATTCTCTGGCTTACAGGGCCTTACGCTTC
TP854_Hit	D+G	1	chr4	.	CAGCAAAATATGTTTGCCAAGCCCATGGCTATCAGGGCACTTGGAAAACAGACTCCAGGAGCTT
TP854_Query	D+G	1	chr4	.	CAGCAAAATATGTTTGCCAAGCCCATGGCTATCAGGGCACTTGGAAAAAAGACTCCAGGAGCTT
TP85510_Hit	D+G	1	chr4	.	CTGCTAGAGATTTGGAGTTCTTACGTGGGCATATCTTGATAGAGCAATTTTCTCATTTAGCCAC
TP85510_Query	D+G	1	chr4	.	CTGCTAGAGATTTGGAGTTCTTACGTGGGCATATCTTGATAGAGCAATTTTCTCAGTTAGCCAC
TP85581_Hit	D	1	chr4	.	CTGCTAGCAAAGAGAAGAAACTTGAGGAAACTAGAAAACTCAATGCGATGTCTAAAGCTACTGA
TP85581_Query	D	1	chr4	.	CTGCTAGCAAAGAGAAGAAACTTGAGGAAACTAGAAAACTCAATGAGATGTCTAAAGCTACTGA
TP85800_Hit	D+G	1	chr4	.	CTGCTAGTAAACTCGCTAATCATGCCATCAAATTAGCTGGTGTAGGCGGCTTTGGAGCTTCTTT
TP85800_Query	D+G	1	chr4	.	CTGCTAGTAAACTCGCTAATCATGCCATCAAATTAGCTGGTGTAGGAGGCTTTGGAGCTTCTTT

Name	Filter	Nb hit (Mt4.0)	Mt Chr (Mt4.0)	Ms Chr (Li et al., 2014)	Sequence
TP85924_Hit	D	1	chr4	.	CTGCTAGTTGAAATCCAAAGATTTCAATTGGTTGACAAATTCAGTAACCGATCTGTTGTTTCCT
TP85924_Query	D	1	chr4	.	CTGCTAGTTGAAATCCAAAGATTTCAATTGGTTGACAAATTCAGTAACCGATCCGTTGTTTCCT
TP86061_Hit	D	1	chr4	.	CTGCTATTGAGAAACTACCAACTTTCAAAAGGATTAAAACTTCTTTTGTTGATGAAATCACTCA
TP86061_Query	D	1	chr4	.	CTGCTATAGAGAAACTACCAACTTTCAAAAGGATTAAAACTTCTTTTGTTGATGAAATCACTCA
TP8620_Hit	D	1	chr4	.	CAGCAATTTGGTTGATGCTGAGTGATTAGTTCTATATCAGTTTGTAAACAAATAATTAATTGAT
TP8620_Query	D	1	chr4	.	CAGCAATTTGGTTGATGCTGAGTGATTAGTTCTATATCAGTTTGTAAACAAATAATTAATTGAA
TP86595_Hit	D	1	chr4	.	CTGCTATTAAAGTTGAGAAACAGAATATTGGAATGTTGTATCAGAATGACGAGGAAATTCAATT
TP86595_Query	D	1	chr4	.	CTGCTATTAAAGTTGAGAAACAGAATATTGGAATGTTGTATCAGAAAGACGAGGAAATTCAATT
TP8664_Hit	D	1	chr4	.	CAGCAATTTTTAGAAGTATTTTACCTGTGCTCTAATAATAGCACTTCTTTTGTCTTGTTCAGCT
TP8664_Query	D	1	chr4	.	CAGCAATTTTTAGAAGTATTTTACCTGTGCTCTAATAATAGCACTCCTTTTGTCTTGTTCAGCT
TP86650_Hit	D	1	chr4	.	CTGCTATTATGAGAAAACTAACCACTTGTCGAGAAATGTTTGAAGACCAAGATGTTTTGGAGAG
TP86650_Query	D	1	chr4	.	CTGCTATTATGAGAAAACTAACCACTTGTCCAGAAATGTTTGAAGACCAAGATGTTTTGGAGAG
TP86809_Hit	D+G	1	chr4	.	CTGCTATTGTGACCCTGCAAATCGATGATAACAATGTTAATGACGAGGCTATTGCAATTGCTTT
TP86809_Query	D+G	1	chr4	.	CTGCTATTGTGACCCTGCAAATCGATGATAACAATGTTAATGACGAGGCTATTGCAATTGCCTT
TP87107_Hit	D	1	chr4	.	CTGCTCAAGAAGCTGAGAGAGCGAAGTTTGTTGTAGAAAAAGCTGAACAAGACAAAAGAAGTGC
TP87107_Query	D	1	chr4	.	CTGCTCAAGAAGCTGAGAGAGCAAAGTTTGTTGTAGAAAAAGCTGAACAAGACAAAAGAAGTGC
TP87271_Hit	D	1	chr4	.	CTGCTCACGACAAAAGCATGGTAAGTCAGTTTCTAGTTCTTATATGACTCGGAATCACTTTTTG
TP87271_Query	D	1	chr4	.	CTGCTCACGACAAAAGCATGGTAAGTCAGTTTCTAGTTCTTATATGACTCAGAATCACTTTTTG
TP87309_Hit	D+G	1	chr4	.	CTGCTCAGAAAGAGAGATATTCTTTCCGAAGGCCTGTGCGTAAGCTGACTCCAAAGCTCCAGTG
TP87309_Query	D+G	1	chr4	.	CTGCTCAGAAAGAGAGATATTCTTTCCAAAGGCCTGTGCGTAAGCTGACTCCAAAGCTCCAGTG
TP87318_Hit	D+G	1	chr4	.	CTGCTCAGAATTTGAGTTGTTAGTAATTGAGGGCGAGGTTGTCCTATTATCCGCAGACGCCGAA
TP87318_Query	D+G	1	chr4	.	CTGCTCAGAATTTGAGTTGTTAGTAATTGACGGCGAGGTTGTCCTATTATCCGCAGACGCCGAA
TP87398_Hit	D	1	chr4	.	CTGCTCAGTGTGCAAAAGTAGCTGAATCAATGGGAGCTAAGAGAGAGGATCTTAGAAGTGTTAT
TP87398_Query	D	1	chr4	.	CTGCTCAGTGTGCAAAAGTAGCTGAATCAATGGGAGCTAAGAAAGAGGATCTTAGAAGTGTTAT
TP87555_Hit	D	1	chr4	.	CTGCTCATTCGTGAGTGATCACAATCTATGGGTTGTGATGCCATTCATGTCGGGTGGTTCATGT
TP87555_Query	D	1	chr4	.	CTGCTCATTCGTGAGTGATCACAATCTATGGGTTGTGATGCCATTCATGTCGGGGGGTTCATGT
TP87974_Hit	D	1	chr4	.	CTGCTCCGGTGACCAGTGAACAGAATGAAGAGATTCAACAGAAGCAGAAACAGGACGAGGTTGA
TP87974_Query	D	1	chr4	.	CTGCTCCGGTGACCAGTGAACAGAATGAAGAGATTCAACAGAAACAGAAACAGGACGAGGTTGA
TP88002_Hit	D	1	chr4	.	CTGCTCCTAACCAGGCAAGAACGATCACAAGAAGCACACCTCCAAGGTATGGAGTTGAGTTGGC
TP88002_Query	D	1	chr4	.	CTGCTCCTAACCAGGCAAGAACAATCACAAGAAGCACACCTCCAAGGTATGGAGTTGAGTTGGC
TP88347_Hit	D	1	chr4	.	CTGCTCGAGCGGCGAAAGAGAGCCAAAGTTTGTCTAATATTAAAACGGACGAGCTTCATGAAAC
TP88347_Query	D	1	chr4	.	CTGCTCGAGCGGCGAAAGAGAGCCAAAGTTTATCTAATATTAAAACGGACGAGCTTCATGAAAC
TP88356_Hit	D	1	chr4	.	CTGCTCGAGGTTTCACAGAGTATCCAAATGGTCATCAATTTCCTAGTGAGTTTGCTATGATAAG
TP88356_Query	D	1	chr4	.	CTGCTCGAGGTTTCACAGAGTATCCAAATGGTCATCAATTTCCAAGTGAGTTTGCTATGATAAG
TP88363_Hit	D	1	chr4	.	CTGCTCGATAAAGAAAGTGTCAACACTGGTGTGGAGCCTAATGAGAAACGGATCTTTATTGATA
TP88363_Query	D	1	chr4	.	CTGCTCGATAAAGAAAGTGTCAACACTGCTGTGGAGCCTAATGAGAAACGGATCTTTATTGATA
TP8848_Hit	D+G	1	chr4	.	CAGCACAAATGTCAATATTAATACACAGTTTTAATCTTTAATATATTTAATTATTTATTAGTAA
TP8848_Query	D+G	1	chr4	.	CAGCACAAATGTCAATATTAATACACAATTTTAATCTTTAATATATTTAATTATTTATTAGTAA
TP88559_Hit	D	1	chr4	.	CTGCTCGGGGCATTGACATTCCATTGCTTGATAATGTTATCAATTGGGACTTTCCTTCTAAGCC
TP88559_Query	D	1	chr4	.	CTGCTCGGGGCATTGACATTCCATTGCTTGATAATGTTATCAATTGGGACTTTCCTCCTAAGCC
TP88968_Hit	D+G	1	chr4	.	CTGCTCTCTTCACTTCCACCATCATGAGCAAGCTACTAGCAAGGCCAAACGTGAAGCTTTTCAA
TP88968_Query	D+G	1	chr4	.	CTGCTCTCTTCACTTCCACAATCATGAGCAAGCTACTAGCAAGGCCAAACGTGAAGCTTTTCAA
TP8945_Hit	D+G	1	chr4	.	CAGCACAGCTTGTTTTCCCAACACCACCTTTCCCACCAAGCATGTAATACCTCCTTTCCTTCTC
TP8945_Query	D+G	1	chr4	.	CAGCACAACTTGTTTTCCCAACACCACCTTTCCCACCAAGCATGTAATACCTCCTTTCCTTCTC
TP89510_Hit	D	1	chr4	.	CTGCTGAAATTCGTGACCCGAGACGTGCCACCCGTGTTTGGCTCGGAACTTTTTCCACGGCGGG
TP89510_Query	D	1	chr4	.	CTGCTGAAATTCGTGACCCGAGACGTGCCACCCGTGTTTGGCTCGGAACTTTTTCCACGGCGGA
TP89518_Hit	D	1	chr4	.	CTGCTGAAATTTTTAGTGAAAGCTTAAGTTACTAATGCATTTCAATATCATTACTTCCTCCCTC
TP89518_Query	D	1	chr4	.	CTGCTGAAATTTTTAGTGAAAGCTTAAGTTACTAATACATTTCAATATCATTACTTCCTCCCTC
TP89609_Hit	D	1	chr4	.	CTGCTGAAGCCTTTTCACCAATGTGCTAAATTTTTTGTAGGTATGACAGTGAGTGAACTAGCAA
TP89609_Query	D	1	chr4	.	CTGCTGAAGCCTTTTCACCAATGTGCTAAATTTTTTGTAGGCATGACAGTGAGTGAACTAGCAA
TP89800_Hit	D+G	1	chr4	.	CTGCTGACGAAAGCTATGCAGATGATGATGATGCAGAGGACAACAGAGACTTTGCTGGTGAAGC
TP89800_Query	D+G	1	chr4	.	CTGCTGACGAAAGCTATGCAGATGATGATGATGCAGAGGACAACAAAGACTTTGCTGGTGAAGC
TP89801_Hit	D	1	chr4	.	CTGCTGACGAAAGCTATGCAGATGATGATGCAGAGGACAACAGAGACTTTGCTGGTGAAGCTAT
TP89801_Query	D	1	chr4	.	CTGCTGACGAAAGCTATGCAGATGATGATGCAGAGGACAACAAAGACTTTGCTGGTGAAGCTAT

Name	Filter	Nb hit (Mt4.0)	Mt Chr (Mt4.0)	Ms Chr (Li et al., 2014)	Sequence
TP89836_Hit	D	1	chr4	.	CTGCTGACTTTCTGCTGGTTGACCACTTGCTGTTTAGGGTGTTTGAACAGGAGGTTTGGCAGGT
TP89836_Query	D	1	chr4	.	CTGCTGACTTTCTGCTGGTTGACCACTTGCTGTTTAGGGTGTTTGAACAGGAGGTCTGGCAGGT
TP90064_Hit	D	1	chr4	.	CTGCTGATACCTTTCATCAGTGATGACGACCTTGTTATTGTTGTTGTTGTTGTTGTTGTTG
TP90064_Query	D	1	chr4	.	CTGCTGATACCTTTCATCAGTGATGACGACCTTGTTATTGTTGTTGTTGTTGTTGTTGTTC
TP90218_Hit	D+G	1	chr4	.	CTGCTGATGCTGTATTAGGAGTAGAGTGGTGGTGTTGGTAGCCAAGACCAAGAGCCTGATCAGG
TP90218_Query	D+G	1	chr4	.	CTGCTGATGCTGTATTAGGAGTAGAGTGGTGGTGGTGGTAGCCAAGACCAAGAGCCTGATCAGG
TP90340_Hit	D+G	1	chr4	.	CTGCTGGAAAAGTACCTACTTTAGTTCAAGATAAGTTGCTTGGTGAATTTGTTGAGGAAATATC
TP90340_Query	D+G	1	chr4	.	CTGCTGGAAAAGTACCTACTTTAGTTCAAGATAAGTTACTTGGTGAATTTGTTGAGGAAATATC
TP90489_Hit	D+G	1	chr4	.	CTGCTGGAGGAAATTCAGGACCTGCTCCTAGTACTGTAACCAACTTGGAACCTTGGACGCTTAC
TP90489_Query	D+G	1	chr4	.	CTGCTGGAGGAAATTCAGGACCTGCTCCTAGTACTATAACCAACTTGGAACCTTGGACGCTTAC
TP9069_Hit	D	1	chr4	.	CAGCACAGGTAATTTGCATACAGTATCTCCCGGGAGTGTAGTTTTTCTCTATGGAACCGTCTGC
TP9069_Query	D	1	chr4	.	CAGCACAAGTAATTTGCATACAGTATCTCCCGGGAGTGTAGTTTTTCTCTATGGAACCGTCTGC
TP911_Hit	D	1	chr4	.	CAGCAAAATCCTTTCAATAAACACAAACATATGTCAAGTTTAACACCAAACTTTAGTAATCTAT
TP911_Query	D	1	chr4	.	CAGCAAAATCCTTTCAATAAAAACAAACATATGTCAAGTTTAACACCAAACTTTAGTAATCTAT
TP91255_Hit	D+G	1	chr4	.	CTGCTGGTTCTGAGAGTAGAGTTGTACTTGAGGAACAAGTTCCGCAGATTAGGGACTTTCTTGA
TP91255_Query	D+G	1	chr4	.	CTGCTGGTTCTGAGAGTAGAGTTGTACTTGAGGAACAAGTTCCACAGATTAGGGACTTTCTTGA
TP91313_Hit	D+G	1	chr4	.	CTGCTGGTTTTAGAGCAAACACCAAGCAATTGCAGGCGAAGGGGAAGGCCAATGTTCTTGGTCG
TP91313_Query	D+G	1	chr4	.	CTGCTGGTTTTAGAGCAAACACCAAGCAATTGCAGGCGAAGGGGAAGGCCAATGTTCCTGGTCG
TP91615_Hit	D+G	1	chr4	.	CTGCTGTCATACTCATGCCTATTACTCTCACAATTGTTGTCATGATCATTAATTATGCATCCAT
TP91615_Query	D+G	1	chr4	.	CTGCTGTCATACTCATGCCTATTACTCTCACAATTGTTGTCATGATCATTAACTATGCATCCAT
TP91778_Hit	D	1	chr4	.	CTGCTGTCTGAAAAGGACAAGGAAAAGAGGAGTACGGCACCAGGAAAGTCCTTGAATGCTTGGA
TP91778_Query	D	1	chr4	.	CTGCTGTCTGAAAAGGACAAGGAAAAGAGGAGTACAGCACCAGGAAAGTCCTTGAATGCTTGGA
TP91852_Hit	D	1	chr4	.	CTGCTGTGACCCAAGAGTCTAGCAGTTCTGGCAAGCCGGCACTGCTGTTATCTGCTGAAAAAAA
TP91852_Query	D	1	chr4	.	CTGCTGTGACCCAAGAGTCTAGCAGTTCTGGAAAGCCGGCACTGCTGTTATCTGCTGAAAAAAA
TP91873_Hit	D	1	chr4	.	CTGCTGTGAGTGATTTTGAGCATGCAAGTGGCAACATAGGAGCATGCCCTGAATTCTTATTGGT
TP91873_Query	D	1	chr4	.	CTGCTGTGAGTGATTTTAAGCATGCAAGTGGCAACATAGGAGCATGCCCTGAATTCTTATTGGT
TP91921_Hit	D+G	1	chr4	.	CTGCTGTGCCTCCAGTGGTTCCAATGTTGCCACCTGTTGCTCCAATATTGCCGTCTGTTGTCCC
TP91921_Query	D+G	1	chr4	.	CTGCTGTGCCTCCAGTGGTTCCAATGTTGCCACCTGTTGCTCCAATATTGCCGTCTATTGTCCC
TP92071_Hit	D+G	1	chr4	.	CTGCTGTGTTGGAGCTCAGTTCTGCTGATTCTCATTCTCATCGTCCTCTGGTTAAGTGTTCCAC
TP92071_Query	D+G	1	chr4	.	CTGCTGTGTTGGAGCTCAGGTCTGCTGATTCTCATTCTCATCGTCCTCTGGTTAAGTGTTCCAC
TP92729_Hit	D	1	chr4	.	CTGCTTAAATGTAATAATCTTGTTGTAGCTTCTGACTTGTTTGTTTGTTTAGGTTTTGCTTAGG
TP92729_Query	D	1	chr4	.	CTGCTTAAATGTAATAATCTTGTTGTAGCTTCTGACTTGTTTGTTTGGTTAGGTTTTGCTTAGG
TP92947_Hit	D	1	chr4	.	CTGCTTAGGTGCTGGGAGGACTGGATGTTGCAATTCTCTGGCTTACAGGGCCTTGCGCTTCTCG
TP92947_Query	D	1	chr4	.	CTGCTTAGGTGCTGGGAGGACTGGATGTTGCAATTCTCTGGCTTACAGGGCCTTACGCTTCTCG
TP93147_Hit	D	1	chr4	.	CTGCTTCGAAGGGAATATATGTTGATTTTTGTTTGGAGTTGCTGGTCAAACATTTTGTGCCTCC
TP93147_Query	D	1	chr4	.	CTGCTTCAAAGGGAATATATGTTGATTTTTGTTTGGAGTTGCTGGTCAAACATTTTGTGCCTCC
TP93537_Hit	D	1	chr4	.	CTGCTTCCGGTTGTCGCGCAATCCCTTCTACAGCTTGCCCTGCTGAAAAAAAAAAAAAAAAAAA
TP93537_Query	D	1	chr4	.	CTGCTTCCGGTTGTCGCGCAATCCCTTCTACAGCTTGCCCTGCAGAAAAAAAAAAAAAAAAAAA
TP93571_Hit	D+G	1	chr4	.	CTGCTTCCTCTCCATTTCTGCTATTTGCACATATGAAGGTGGCGGCACAGACAGTGGTGCTGAA
TP93571_Query	D+G	1	chr4	.	CTGCTTCCTCTCCATTTCTGCTATTTGCACATATGAAGGCGGCGGCACAGACAGTGGTGCTGAA
TP93583_Hit	D	1	chr4	.	CTGCTTCCTTAGGCTCCACAACAGGCTACCGAACACTACAAATTTATGTTTGTCCAGCTGAAAA
TP93583_Query	D	1	chr4	.	CTGCTTCCTTAGGCTCCACAACAGGCTACCAAACACTACAAATTTATGTTTGTCCAGCTGAAAA
TP93698_Hit	D	1	chr4	.	CTGCTTCTAACTGCTCGGTCACCCTTGCCACTTTACGGTTGCTTCTGTCTGCTTCCTCCATCAC
TP93698_Query	D	1	chr4	.	CTGCTTCTAACTGCTCGGTCACCCTCGCCACTTTACGGTTGCTTCTGTCTGCTTCCTCCATCAC
TP93722_Hit	D	1	chr4	.	CTGCTTCTAGAAGATCAAAATGTAAAGCGCAGGAGAGAGCGTTATCAGAAACAATCTTCGCTTC
TP93722_Query	D	1	chr4	.	CTGCTTCTAGAAGATCAAAATGTAAAGCGCAGGAGAGAGCGTTATCAGAAACAATCCTCGCTTC
TP93732_Hit	D	1	chr4	.	CTGCTTCTATATTTCACAAATACTTTCGATGTGCGACATTTGTTTGCAAGCACTATGGACTATG
TP93732_Query	D	1	chr4	.	CTGCTTCTATATTTCACAAATACTTTCGAGGTGCGACATTTGTTTGCAAGCACTATGGACTATG
TP93930_Hit	D	1	chr4	.	CTGCTTCTTCAATATGATTAAGAGCACATAACTCCTGAATAACTTGAGGCCACTGTGCTTCTGG
TP93930_Query	D	1	chr4	.	CTGCTTCTTCAATATGATTAAGAGCACATAACTCCTGAATAACTTGAGGCCACTGAGCTTCTGG
TP94000_Hit	D	1	chr4	.	CTGCTTCTTTGTGGGAAATCAATGATTTGCATCCTTCAAGGGTCAAGAAGGAGTTTGAAGTTGA
TP94000_Query	D	1	chr4	.	CTGCTTCTTTGTGGGAAATCAATGATTTGCATCCTTCAAGGGTCAAGAAGGACTTTGAAGTTGA
TP94009_Hit	D+G	1	chr4	.	CTGCTTCTTTTGGTTCTTCTGCCGGCGGTGGAAATGGAGGTGCTGGAGTTGGAAATGGAGGAGG
TP94009_Query	D+G	1	chr4	.	CTGCTTCTTTTGGTTCTTCTGCCGGCGGTGGAAATGGAGGTACTGGAGTTGGAAATGGAGGAGG

Name	Filter	Nb hit (Mt4.0)	Mt Chr (Mt4.0)	Ms Chr (Li et al., 2014)	Sequence
TP9405_Hit	D	1	chr4	.	CAGCACGCTTAGAATATCAAGCTCTGTCATAAATTCGTGACAGCCTTGACAACCATCAGGATTT
TP9405_Query	D	1	chr4	.	CAGCACACTTAGAATATCAAGCTCTGTCATAAATTCGTGACAGCCTTGACAACCATCAGGATTT
TP94548_Hit	D	1	chr4	.	CTGCTTGTCAGAATTTAAAATTGAAAACCCACAAGCTTCCTTCATTTTCTCAGATGAAAAAGAG
TP94548_Query	D	1	chr4	.	CTGCTTGTCAGAATTTAAAAATGAAAACCCACAAGCTTCCTTCATTTTCTCAGATGAAAAAGAG
TP94565_Hit	D	1	chr4	.	CTGCTTGTCTAAGTCGTTTATGAAGCAAATTATTTGTATTGTTCCATAGCTTTTTGGCTTCTGA
TP94565_Query	D	1	chr4	.	CTGCTTGTCTAAGTCGTTTATGAAGCAAATTATTTGTATTGTTCCACAGCTTTTTGGCTTCTGA
TP946_Hit	D	1	chr4	.	CAGCAAAATGCACAACAGGTAATCTACCCAATTGTTTCGTTTGCGCTACAATATCATAAGGCGC
TP946_Query	D	1	chr4	.	CAGCAAAATGAACAACAGGTAATCTACCCAATTGTTTCGTTTGCGCTACAATATCATAAGGCGC
TP94652_Hit	D	1	chr4	.	CTGCTTGTTCTGGTGTCATGTTATCAAATGGTGTGAGTCCTGTAATTAGCTCCCAAAGAACTAT
TP94652_Query	D	1	chr4	.	CTGCTTGTTCTGGTGTCATGTTATCAAATGGCGTGAGTCCTGTAATTAGCTCCCAAAGAACTAT
TP94670_Hit	D+G	1	chr4	.	CTGCTTGTTGGGTTTGAAGTATCATAGTAAAGAAAAGTTGAATAAACTTGCCATTTCATCAGAT
TP94670_Query	D+G	1	chr4	.	CTGCTTGTTGGGTTTGAAGCATCATAGTAAAGAAAAGTTGAATAAACTTGCCATTTCATCAGAT
TP95199_Hit	D+G	1	chr4	.	CTGCTTTGCCGAAAGGGCAGACAATCAATGTTGGCAGTAGAGATGATGTCTCAGCTGTTAAAAA
TP95199_Query	D+G	1	chr4	.	CTGCTTTGCCGAAAGGGCAGACAATCAACGTTGGCAGTAGAGATGATGTCTCAGCTGTTAAAAA
TP9521_Hit	D+G	1	chr4	.	CAGCACAGCCTATCAGTACCACATCCACCACCACCACCATCACAACAACAAAAACTAATCCTAT
TP9521_Query	D+G	1	chr4	.	CAGCACAGCCTATCAGTACCACATCCACCACCACCACCATCACAACAACAAAAACGAATCCTAT
TP95426_Hit	D	1	chr4	.	CTGCTTTTATTGTTTTATGTGTGTGCTGGTGTTATTTAGCAATCTTCTGCATGATCAAGGTTCT
TP95426_Query	D	1	chr4	.	CTGCTTTTATTGTTTTATGTGCGTGCTGGTGTTATTTAGCAATCTTCTGCATGATCAAGGTTCT
TP95428_Hit	D	1	chr4	.	CTGCTTTTATTTCCAATTGTGGTGCTCGAAATTTCCGGTTGCAAGCTCTCGAAGCCCTAGAAAA
TP95428_Query	D	1	chr4	.	CTGCTTTTATTTCCAATTGTGGTGCTCGAAATTTCCGATTGCAAGCTCTCGAAGCCCTAGAAAA
TP95466_Hit	D	1	chr4	.	CTGCTTTTCCGTGGAATGATTTGTTTGGAATACTTGGTGGTTATTGTCGTGTAATAAAAAGCTT
TP95466_Query	D	1	chr4	.	CTGCTTTTCCGTGGAATGATTTGTTTGGAATACTTGGTGGTTATTGTCGTGTAATAAAAACCTT
TP95605_Hit	D	1	chr4	.	CTGCTTTTGCTTCTGGGTGTCCTAATTTGGTTAAACTCAAGGTGAGAAAGTGTGTGAAAGTTAC
TP95605_Query	D	1	chr4	.	CTGCTTTTGCTTCCGGGTGTCCTAATTTGGTTAAACTCAAGGTGAGAAAGTGTGTGAAAGTTAC
TP9692_Hit	D	1	chr4	.	CAGCACATATATTCCTCAATCAAACCAACCTTATCGTAGTGTCTTTGTTATCTATCCTCTCAGT
TP9692_Query	D	1	chr4	.	CAGCACATATATTCCTCAATCAAACCAACCTTATCATAGTGTCTTTGTTATCTATCCTCTCAGT
TP9710_Hit	D+G	1	chr4	.	CAGCACATATTGCAAATTTGTTGTCGACTGAGCCAGCTGATGGTACTTTCAAGGCTAAATAATA
TP9710_Query	D+G	1	chr4	.	CAGCACATATTGCAAATTTGTTGTCAACTGAGCCAGCTGATGGTACTTTCAAGGCTAAATAATA
TP9769_Hit	D	1	chr4	.	CAGCACATCTTGCTCTAAAGAAGGCCTTTCTTACGTGCACAGAATGTACACATCCAGGTGCTGA
TP9769_Query	D	1	chr4	.	CAGCACATCTTGCTCTAAAGAAGGCCTTTCTTACGTGCACAGAATGTACACATCCAAGTGCTGA
TP9776_Hit	D	1	chr4	.	CAGCACATGAAACAAGCTTCTTTTCGGTTCTGTGCAAATATTAAAAACTAGAAAGTTAACAAAG
TP9776_Query	D	1	chr4	.	CAGCACATGAAACAAGCTTCTTATCGGTTCTGTGCAAATATTAAAAACTAGAAAGTTAACAAAG
TP9778_Hit	D+G	1	chr4	.	CAGCACATGAAACCTGGTTTTGGTTAGTATGACAATGATACTCATTTCCTCCATGGAAAGTGTT
TP9778_Query	D+G	1	chr4	.	CAGCACATGAAACCTGGTTTTGGTTAGTATGACAATGATACTCATTTCCTCCATCGAAAGTGTT
TP9857_Hit	D	1	chr4	.	CAGCACATGTAAGCGGTGTTTCAACCCTGATACCATTGATAGAAGGAAAATCTGACTTATTAAC
TP9857_Query	D	1	chr4	.	CAGCACATGTAAGCGGTGTTTCAACCCTGATACCATTGATAGAAGGAAAATCCGACTTATTAAC
TP66978_Hit	D	1	chr4	1D	CTGCACTGGTTATCATAAAGGGTAGTATATCCATTTGTCTTCTTGACAGGTATGGAAGTAGTTC
TP66978_Query	D	1	chr4	1D	CTGCACTGATTATCATAAAGGGTAGTATATCCATTTGTCTTCTTGACAGGTATGGAAGTAGTTC
TP494_Hit	D	1	chr4	3B	CAGCAAAACTCAGACACACTAAGTGCTGAGAAGTGGGGAATTGCGGGCACAAAACCGCGTCCCT
TP494_Query	D	1	chr4	3B	CAGCAAAACTCAGACACAATAAGTGCTGAGAAGTGGGGAATTGCGGGCACAAAACCGCGTCCCT
TP1592_Hit	D	1	chr4	4A	CAGCAAAGAATCCGAAGAAAGAAGCTCCAAAGCCTCCTACACCAGCTAATTTGATGGCATGATT
TP1592_Query	D	1	chr4	4A	CAGCAAAGAATCCGAAGAAAGAAGCTCCAAAGCCGCCTACACCAGCTAATTTGATGGCATGATT
TP1792_Hit	D	1	chr4	4A	CAGCAAAGCTTTATAGGGCAAAAAGATCCTTGCGAATTTAGCAGGAGATCTACAACACTAGCTG
TP1792_Query	D	1	chr4	4A	CAGCAAAGCTTTATAGGGCAAAAAGATCCTTGAGAATTTAGCAGGAGATCTACAACACTAGCTG
TP2278_Hit	D	1	chr4	4A	CAGCAAATGACATTGCACCTCGTGAGCTGGAAGAATAGCCACTCCTAAAATCTGTGGGGTCGGA
TP2278_Query	D	1	chr4	4A	CAGCAAATGACATTGCACCTCGTGAGCTAGAAGAATAGCCACTCCTAAAATCTGTGGGGTCGGA
TP34951_Hit	D	1	chr4	4A	CAGCCTTTGATGATGCAATCATCTCTATCCAGCCTTCTACAGAATCAGCAACAGATTCTTTACC
TP34951_Query	D	1	chr4	4A	CAGCCTTTGATGATGCAATCATCACTATCCAGCCTTCTACAGAATCAGCAACAGATTCTTTACC
TP42523_Hit	D	1	chr4	4A	CAGCTAAGTTAGTGCTTGTGGACTTGAGAGGTTCTGATTTATTAAAGAGAAGGAAGATTTAGAA
TP42523_Query	D	1	chr4	4A	CAGCTAAGTTAGTACTTGTGGACTTGAGAGGTTCTGATTTATTAAAGAGAAGGAAGATTTAGAA
TP46946_Hit	D+G	1	chr4	4A	CAGCTCCAAAAGTAGCTTCAGCTATGTTATCTCCATCTAGAAAATCCAAAACACCAAAAGCTTT
TP46946_Query	D+G	1	chr4	4A	CAGCTCCAAAAGTAGCTTCAGCTATGTTATCTCCATCTAGAAAATCAAAAACACCAAAAGCTTT
TP48904_Hit	D+G	1	chr4	4A	CAGCTCTCCCTGTAACAGCAAGAACAGAACATCAAAATGAAACAATATATTCAAATGTAATCCT
TP48904_Query	D+G	1	chr4	4A	CAGCTCTCCCTGTAACAGCAAGAACAGAACACCAAAATGAAACAATATATTCAAATGTAATCCT

Name	Filter	Nb hit (Mt4.0)	Mt Chr (Mt4.0)	Ms Chr (Li et al., 2014)	Sequence
TP50814_Hit	D	1	chr4	4A	CAGCTGATGAAACAGTAATTAGCAACACCTTCAAAGTCGGTGAACCTGGAAAGGTTATACTCAC
TP50814_Query	D	1	chr4	4A	CAGCTGATGAAACAGTAATTAGCAACACCTTCAAAGTCGGCGAACCTGGAAAGGTTATACTCAC
TP57631_Hit	D	1	chr4	4A	CAGCTTGGAACGAATAGTTCAACCAGCGAACATGGAAACCTGTCAGGAATGCAACATTGCAGGA
TP57631_Query	D	1	chr4	4A	CAGCTTGGAACGAATAGTTCAACCAGCGAACATGGAAACCTGCCAGGAATGCAACATTGCAGGA
TP6067_Hit	D	1	chr4	4A	CAGCAAGGCCGCAGGCATCTTCCTCATCATGAAGAGGAATGTCTCGACTGAGCTCCTGTTCAGC
TP6067_Query	D	1	chr4	4A	CAGCAAGGCCACAGGCATCTTCCTCATCATGAAGAGGAATGTCTCGACTGAGCTCCTGTTCAGC
TP62231_Hit	D+G	1	chr4	4A	CTGCAACATAGAAATAATGCAGTCCACAGACTAATTTGCTGGATTTTTTTTGAAGATATTTTAC
TP62231_Query	D+G	1	chr4	4A	CTGCAACATAGAAATAATGCAGTCCACAGACTAATTTGCTGGATTTTTTTTGAAGAAATTTTAC
TP7005_Hit	D	1	chr4	4A	CAGCAATAGCCACCAAATTATATCAATTTATTAGGAGCTTGATAGCTAGCCATTGGAACTATTA
TP7005_Query	D	1	chr4	4A	CAGCAATAGCCACCAAATTACATCAATTTATTAGGAGCTTGATAGCTAGCCATTGGAACTATTA
TP76814_Hit	D	1	chr4	4A	CTGCCCTGGGCTTAGGCTTTTCAACAACAATGGCCTGTGTGGTCAAGACCATCCCAGCCACTGA
TP76814_Query	D	1	chr4	4A	CTGCCCTGGGCTTAGGCTTTTCAACAACAATGGCCTGAGTGGTCAAGACCATCCCAGCCACTGA
TP78305_Hit	D	1	chr4	4A	CTGCCTCAACAGCTAGTGTTGTAGATCTCCTGCTAAATTCTCAAGGATCTTTTTGCCCTATAAA
TP78305_Query	D	1	chr4	4A	CTGCCTCAACAGCTAGTGTTGTAGATCTCCTGCTAAATTCGCAAGGATCTTTTTGCCCTATAAA
TP80871_Hit	D+G	1	chr4	4A	CTGCGCAATGATGGTTTTGATGGCTATTCTACCATGGAATATTAGTAGTTGATAAATAGTTATT
TP80871_Query	D+G	1	chr4	4A	CTGCGCAATGATGGTTTTGATGGCTATTCTACCATGGAATATTAGTAGTTGATAAATAGGTATT
TP81929_Hit	D	1	chr4	4A	CTGCGGAGAAGTATAAGAGAAAAGTAGCTTCTGAATTTGGGATTCCTCTCTATGTTCGTAGGGT
TP81929_Query	D	1	chr4	4A	CTGCGGAGAAGTATAAGAGAAAAGTAGCTTCTGAATTTGGGATTCCTCTATATGTTCGTAGGGT
TP92433_Hit	D	1	chr4	4A	CTGCTGTTGGTAAAGAATCTGTTGCTGATTCTGTAGAAGGCTGGATAGTGATGATTGCATCATC
TP92433_Query	D	1	chr4	4A	CTGCTGTTGGTAAAGAATCTGTTGCTGATTCTGTAGAAGGCTGGATAGAGATGATTGCATCATC
TP12575_Hit	D	1	chr4	4B	CAGCATTTGAGACAACATGCTTCTAACCATAGTATACAGAAAGCTTTTGTGGTGATTGGTATTA
TP12575_Query	D	1	chr4	4B	CAGCACTTGAGACAACATGCTTCTAACCATAGTATACAGAAAGCTTTTGTGGTGATTGGTATTA
TP23577_Hit	D	1	chr4	4B	CAGCCAAGAATTAATTACTAGTACTACAACTTCACTTCCACTAGGAAACATTAATGTTACTGTT
TP23577_Query	D	1	chr4	4B	CAGCCAAGAATTAATTACTAGTACTACAACTTCACTTCCACTAGGAAACATTAATGTTAATGTT
TP43699_Hit	D+G	1	chr4	4B	CAGCTACTTTGACAATGGAGCCATGCTTGGCACATACTCGTAATACCAAAAACAAGCATTCTCT
TP43699_Query	D+G	1	chr4	4B	CAGCTACTTTGACAATGGAGCCATGCTTGGCACATACTCGTAAAACCAAAAACAAGCATTCTCT
TP45285_Hit	D+G	1	chr4	4B	CAGCTATGTTATATGGGCTTCTAACTTCCAACTTTTATTTTGAGGTCATGACTATTACAACCAT
TP45285_Query	D+G	1	chr4	4B	CAGCTATGTTATATGGGCTTCTAACTTCCAACTTTTATTTTGAGGTCATGACTATGACAACCAT
TP46835_Hit	D	1	chr4	4B	CAGCTCATGTTTGAGACGCTTAAGCACCGTGCACTCGAGTTTTGTGGACAACATGATGATGACA
TP46835_Query	D	1	chr4	4B	CAGCTCATGTTTGAGACGCTTAAGCACCGTGCACTCGAGTTTTGCGGACAACATGATGATGACA
TP63690_Hit	D	1	chr4	4B	CTGCAAGTTTCACAATTATCGTCTAAATACTCAGCATTCAGCATACTAAGGGGTCCTTTCAGTA
TP63690_Query	D	1	chr4	4B	CTGCAAGTTTCACAATTATCGTCTAAATACTCAGCATTCAGCATACTAAGGGGTCATTTCAGTA
TP73508_Hit	D	1	chr4	4B	CTGCCAAATTAAAGAGGTTTAGATGGGAAATTAGATGATAGATAATGTGTACTAACATATGATA
TP73508_Query	D	1	chr4	4B	CTGCCAAATTAAAGAGGTTTAGATGGGAAATTAGATGATAGATAATGTGTACTAAAATATGATA
TP85553_Hit	D	1	chr4	4B	CTGCTAGATCCGCTATTAGACAACACAAGTAAATGTAGAAATCAATACATGCACAAATTTCTTA
TP85553_Query	D	1	chr4	4B	CTGCTAGATCCGCTATTAGACAACACAAGTAAATGTACAAATCAATACATGCACAAATTTCTTA
TP85834_Hit	D+G	1	chr4	4B	CTGCTAGTATCATATATAGATATCATGTGAAGGTGGTGGAGAATCACCCTGTTGAGCCAAAGAT
TP85834_Query	D+G	1	chr4	4B	CTGCTAGTATCATATATAGATATCATGTGAAGGTGGTGGAGAATCACCCTATTGAGCCAAAGAT
TP88549_Hit	D	1	chr4	4B	CTGCTCGGCTTCGCATTTGCGGCACGGTGATACAAAAGACCTAAACATTTTCTGGCCAAACCAT
TP88549_Query	D	1	chr4	4B	CTGCTCGGCTTCGCATTTGCGGCACGGTGATACAAAAGACCTAAACATATTCTGGCCAAACCAT
TP16597_Hit	D	1	chr4	4C	CAGCAGTTCGTGAGGCTGTTACAAAAGTCATTGATCAGAAGGTATTTGCTGATTCATGATTAAT
TP16597_Query	D	1	chr4	4C	CAGCAGTTCGTGAGGCTGTTACAAAAGTCATTGATCAGAAGGTATTTGCTGATTCATAATTAAT
TP194_Hit	D+G	1	chr4	4C	CAGCAAAAAGATGATTGGCAAAATTGTTCCGTGTGTCTTGGCCTCTAAAGCTCACAAAAACGTC
TP194_Query	D+G	1	chr4	4C	CAGCAAAAAGATGATCGGCAAAATTGTTCCGTGTGTCTTGGCCTCTAAAGCTCACAAAAACGTC
TP2187_Hit	D	1	chr4	4C	CAGCAAATCATGCATGCCAAGCTTGTTTTTGTCATCAACGATTACAAGGCTTCTCTCAACAAGG
TP2187_Query	D	1	chr4	4C	CAGCAAATCATGCATGCCAAGCTTGTTTTTATCATCAACGATTACAAGGCTTCTCTCAACAAGG
TP43945_Hit	D+G	1	chr4	4C	CAGCTAGATTCTTGATTAATTATGAATCAGCAAATACCTTCTGATCAATGACTTTTGTAACAGC
TP43945_Query	D+G	1	chr4	4C	CAGCTAGATTCTTGATTAATCATGAATCAGCAAATACCTTCTGATCAATGACTTTTGTAACAGC
TP62567_Hit	D	1	chr4	4C	CTGCAACGGCTGTGGGCTCAACGGTCCTTCATCCTCTGTAATACGAAATACAGTAACCTGCTGA
TP62567_Query	D	1	chr4	4C	CTGCAACGGCTGTGGGCTCAACGGTCCTTCATCCTCTGTAATACGAAATACAGTAACCTGCAGA
TP66382_Hit	D	1	chr4	4C	CTGCACCTTGTCCTGCTCATGATGGCCTGCAAAACACCAAAATTGTCATCAAGTCAAAATCTGG
TP66382_Query	D	1	chr4	4C	CTGCACCTTGTCCTGCTCATGATGGCCTGCAAAACACCAAAATTGTCATCAAGTCAAAATCTGA
TP81917_Hit	D	1	chr4	4C	CTGCGGACTTTTCGCATAAATTGGAATAAAGGTCCTTGTTGAGAGAAGCCTTGTAATCGTTGAT
TP81917_Query	D	1	chr4	4C	CTGCGGACTTTTCGCAGAAATTGGAATAAAGGTCCTTGTTGAGAGAAGCCTTGTAATCGTTGAT

Name	Filter	Nb hit (Mt4.0)	Mt Chr (Mt4.0)	Ms Chr (Li et al., 2014)	Sequence
TP82319_Hit	D	1	chr4	4C	CTGCGGCTAACACCGAGGGATTTTCTGGAAGGGAGATAGCAAAACTGATGGCAAGTGTCCAAGC
TP82319_Query	D	1	chr4	4C	CTGCGGCTAACACCGAGGGATTTTCTGGAAGGGAAATAGCAAAACTGATGGCAAGTGTCCAAGC
TP93256_Hit	D	1	chr4	4C	CTGCTTCACGATGACCGGTGTCATACAGTATCCATCCTTCATATACAAGCCTTTCATGGTCAGA
TP93256_Query	D	1	chr4	4C	CTGCTTCACGATGACCAGTGTCATACAGTATCCATCCTTCATATACAAGCCTTTCATGGTCAGA
TP95077_Hit	D+G	1	chr4	4C	CTGCTTTCTTGTCGTCACCTTTAGCATCGGCTATCACAGGTACCGTTCTATATGTTGACAATGG
TP95077_Query	D+G	1	chr4	4C	CTGCTTTCTTATCGTCACCTTTAGCATCGGCTATCACAGGTACCGTTCTATATGTTGACAATGG
TP17977_Hit	D+G	1	chr4	4D	CAGCATATGCAATCTGCACAAACAACATTAGGATGATTGGTTTTAGTGCTTGCAATGAATTGTA
TP17977_Query	D+G	1	chr4	4D	CAGCATATGCAATCTGCACAAACAACATTAGAATGATTGGTTTTAGTGCTTGCAATGAATTGTA
TP20268_Hit	D+G	1	chr4	4D	CAGCATGCTTATGGTTATGGATACCATTGCATGGCTTTACTGCTTCATGAACTAACAAAGGGAA
TP20268_Query	D+G	1	chr4	4D	CAGCATGCTTATGGTTATGGATACCATTGCATGGCTTTACTACTTCATGAACTAACAAAGGGAA
TP22363_Hit	D	1	chr4	4D	CAGCATTTGTGACTATGACCTTCACACACACTGTGCAATACCTTCCACAACTCTCTTCCATCCA
TP22363_Query	D	1	chr4	4D	CAGCATTTGTGACTATGACCTTCACACACACTGCGCAATACCTTCCACAACTCTCTTCCATCCA
TP48788_Hit	D	1	chr4	4D	CAGCTCTATTTGTTACAATGTCACCTCTTAATCCATCCACATTCAACTCTGCACAAACCTTGGA
TP48788_Query	D	1	chr4	4D	CAGCTCTATTTGTTACAATGTCACCTCTTAATCCATCCACATTCAACTCTGCACAAACCATGGA
TP49153_Hit	D	1	chr4	4D	CAGCTCTGGAGCAACATACTTTATGGAACCGAAAGAAGCAATAGATTTGAACAACATGGAAGTT
TP49153_Query	D	1	chr4	4D	CAGCTCTGGAGCAACATACTTTATGGAACCGAAAAAAGCAATAGATTTGAACAACATGGAAGTT
TP52769_Hit	D	1	chr4	4D	CAGCTGTTACATCCTGTGGTCCAGTTGTGACAAGCTTATACCCTTGTGTTTCTTACATTATGAC
TP52769_Query	D	1	chr4	4D	CAGCTGTTACATCCTGTGGTCAAGTTGTGACAAGCTTATACCCTTGTGTTTCTTACATTATGAC
TP58317_Hit	D	1	chr4	4D	CAGCTTGTTCACAAGGACAGAAATTTCCACCAGGGACAACAATGTATGCTTTTTTGCCAACTGT
TP58317_Query	D	1	chr4	4D	CAGCTTGTTCACAAGGACAGAAATTTCCACCAGAGACAACAATGTATGCTTTTTTGCCAACTGT
TP60402_Hit	D	1	chr4	4D	CAGCTTTTTGTAACAAAGCTGTAGCTGACATGTTTGCTGAAGATGTTTGATGTGGTATTTGGTG
TP60402_Query	D	1	chr4	4D	CAGCTTTTTGTAACAAAGCTGTAGCTGACATGTTTGCTGAAGATGTTAGATGTGGTATTTGGTG
TP62822_Hit	D	1	chr4	4D	CTGCAACTGGAGCTTTAACACCTTTTTTGGGAGCCTAAGAAATCAAACACGATGAAGATAAACG
TP62822_Query	D	1	chr4	4D	CTGCAACTGGAGCTTTAACACCTTTTTTGGGAGCCTAAGAAATCAAACACAATGAAGATAAACG
TP65658_Hit	D+G	1	chr4	4D	CTGCACATGTCAGGTGACATTGTAGATAAAGAAAAAATATCTAGGTGTCTCCAAACTCTCCAAA
TP65658_Query	D+G	1	chr4	4D	CTGCACATGTCAGGTAACATTGTAGATAAAGAAAAAATATCTAGGTGTCTCCAAACTCTCCAAA
TP68041_Hit	D	1	chr4	4D	CTGCAGAGTTGCCATCAATTGTAGATGATGGACTTATTCCAAACACAGTAGGACTACCCGCAGT
TP68041_Query	D	1	chr4	4D	CTGCAGAGTTGCCATCAATTGTAGATGATGGACTTATTCCAAACACAGTAGGACTACCAGCAGT
TP68158_Hit	D	1	chr4	4D	CTGCAGATGCATGGAGGACTTGGTAAGCACCACACGATATGTCTCGTGGTCTCTACACAATAAG
TP68158_Query	D	1	chr4	4D	CTGCAGATGCATGGAGGACTTGGTAAGCACCACACGATATGTCTCATGGTCTCTACACAATAAG
TP9152_Hit	D	1	chr4	4D	CAGCACAATCTTGAAGATGAAGGGGGTATACAATTCATTGCAAGCACTAAAACCAATCATTCTA
TP9152_Query	D	1	chr4	4D	CAGCACAATCTTGAAGATGAAGGGGGTATACAATTCATTGCAAGCACTAAAACCAATCATCCTA
TP46119_Hit	D+G	1	chr4	6D	CAGCTCACAAATCAGATATTGTCGTCGAATGGCCAACAAGGATGAAAATAGCAATCGGAATCAC
TP46119_Query	D+G	1	chr4	6D	CAGCTCACAAACCAGATATTGTCGTCGAATGGCCAACAAGGATGAAAATAGCAATCGGAATCAC
TP95136_Hit	D+G	1	chr4	7B	CTGCTTTGACCAGCTTGTACCCTAAATTAGTCACTAAAGAGAACTTACTTTCTTCAGCGGAACT
TP95136_Query	D+G	1	chr4	7B	CTGCTTTGACCAGCTTGTACCCTAAATTAGTCACTAAAGAGAACTTACTTTCTTCAGCAGAACT
TP1437_Hit	D	1	chr4	8A	CAGCAAACTATGCCTCAGCCTATCACTACCAATTACATCTCTCTGACATGTGCCAAGCTTCCTG
TP1437_Query	D	1	chr4	8A	CAGCAAACTATGCCTCAGCCTATCACTACCAATTACATCTCTCTGACATGTGACAAGCTTCCTG
TP14609_Hit	D	1	chr4	8A	CAGCAGGAGCGAGCGGATTTTCAGCGGGATATTGAGATGCAAAAAAGGGAGCTAAATAACCTTA
TP14609_Query	D	1	chr4	8A	CAGCAGGAGCGAGCGGATTTTCAGCGGGATATTGAGATGCAAAAAAGGGAGCTAAACAACCTTA
TP20498_Hit	D	1	chr4	8A	CAGCATGGTGGATCCCAAACATAAAATAGTAACATGCAGTTTACCAAAGTTGGTTGCATTATCC
TP20498_Query	D	1	chr4	8A	CAGCATGGTGGATCCCAAACATAAAATAGTAATATGCAGTTTACCAAAGTTGGTTGCATTATCC
TP23530_Hit	D+G	1	chr4	8A	CAGCCAACTTTTTCAAATGAGATGCAAAAGAAATATTGAGTCCTTGTGCAAACTTATGAGCACC
TP23530_Query	D+G	1	chr4	8A	CAGCCAACTTTCTCAAATGAGATGCAAAAGAAATATTGAGTCCTTGTGCAAACTTATGAGCACC
TP25630_Hit	D+G	1	chr4	8A	CAGCCAGAGGGCAAGGTCACTCCACTAATGGACAAGCCATGACTCATGATGGAATTGTAGTTGA
TP25630_Query	D+G	1	chr4	8A	CAGCCAGAGGACAAGGTCACTCCACTAATGGACAAGCCATGACTCATGATGGAATTGTAGTTGA
TP2747_Hit	D	1	chr4	8A	CAGCAACAAACCGTAACGCAAGGTTTGTAGTGAAAGCATCCTTGAAAGAGAAACTTGTGACAGG
TP2747_Query	D	1	chr4	8A	CAGCAACAAACCGCAACGCAAGGTTTGTAGTGAAAGCATCCTTGAAAGAGAAACTTGTGACAGG
TP27644_Hit	D+G	1	chr4	8A	CAGCCCAAAATAACATCTCCTAAAGTTTTGTTCCATTCCATACATCCATTTGGTGAATACCACT
TP27644_Query	D+G	1	chr4	8A	CAGCCCAAAATAACATCTCCTAAAGTTTTGTTCCATTCCACACATCCATTTGGTGAATACCACT
TP49525_Hit	D+G	1	chr4	8A	CAGCTCTTTACTCTTGTTTTCTACATTATTTGCACGACCTGTCAAATGAATTTGGTCAGTTTTA
TP49525_Query	D+G	1	chr4	8A	CAGCTCTTTACTCTTGTTTTCTACATTATTTGCACCACCTGTCAAATGAATTTGGTCAGTTTTA
TP51973_Hit	D	1	chr4	8A	CAGCTGGTTGAATCAAATTTTCCCGGATGCAAAGAGAAACCCACTGACGTGAAAGAGAGGCAAG
TP51973_Query	D	1	chr4	8A	CAGCTGGTTGAATCAAATTTTCCCGGATGCAAAGAGAAACCCACTGACGTGAAAGAGAAGCAAG

Name	Filter	Nb hit (Mt4.0)	Mt Chr (Mt4.0)	Ms Chr (Li et al., 2014)	Sequence
TP55199_Hit	D	1	chr4	8A	CAGCTTCCAATCTAGAATTCACAGGTTTTAAGACAGGAAAAACAGGAAGCTTGTCACATGTCAG
TP55199_Query	D	1	chr4	8A	CAGCTTCCAATCTAGAATTCACAGGTTTTAAGACAGGAAAAACAGGAAGCTTGGCACATGTCAG
TP61723_Hit	D	1	chr4	8A	CTGCAAATGGTCAAACATCATTGCCTGTTGAATCATCAAAACAACCAACTAAGAAAATATCTTC
TP61723_Query	D	1	chr4	8A	CTGCAAATGGTCAAACATCATTGCCTGTTGAATCATCAAAACAACCAACGAAGAAAATATCTTC
TP69941_Hit	D+G	1	chr4	8A	CTGCAGTTAACCCTGTCACAAGTTTCTCTTTCAAGGATGCTTTCACTACAAACCTTGCGTTGCG
TP69941_Query	D+G	1	chr4	8A	CTGCAGTTAACCCTGTCACAAGTTTCTCTTTCAAGGATGCTTTCACTACAAACCTTGCGTTACG
TP76032_Hit	D	1	chr4	8A	CTGCCCGTAGAATTCTCGTTGTCACTATTTGTCTCAGGGTTTGATTTGGAAGTATCTGTGCTCC
TP76032_Query	D	1	chr4	8A	CTGCCCATAGAATTCTCGTTGTCACTATTTGTCTCAGGGTTTGATTTGGAAGTATCTGTGCTCC
TP84342_Hit	D	1	chr4	8A	CTGCTAATGGTCTTTTGGTTCTTTACATTACCATCAACTTGTTCAAGATTTACTATGGTGATGA
TP84342_Query	D	1	chr4	8A	CTGCTAACGGTCTTTTGGTTCTTTACATTACCATCAACTTGTTCAAGATTTACTATGGTGATGA
TP11296_Hit	D	1	chr4	8B	CAGCACGCAATAGAACCGGGGATAGGTGGGTGTGTTGACCTTCATAGCTGGTTAGCACGATCGA
TP11296_Query	D	1	chr4	8B	CAGCACGCAATAGAACCGGGGATAGGTGAGTGTGTTGACCTTCATAGCTGGTTAGCACGATCGA
TP67405_Hit	D	1	chr4	8B	CTGCACTTTTCGCCTTAGTTGAATCAGGTAACAATGACCCGGATTGAGATAGTCCTATAGCTGA
TP67405_Query	D	1	chr4	8B	CTGCACTTTTCGCCTTAGTAGAATCAGGTAACAATGACCCGGATTGAGATAGTCCTATAGCTGA
TP81953_Hit	D	1	chr4	8B	CTGCGGAGGAAGAGGTGATTCTGGATAACAACTTTGGCACATCAAAATTGATAGTTGACGCAAA
TP81953_Query	D	1	chr4	8B	CTGCGGAGGAAGAGGTGATTCTGGATAACAACTTTGGCACATCAAAATTGATAGTTGAAGCAAA
TP88099_Hit	D	1	chr4	8B	CTGCTCCTCGGTAAGGTTCTCGGAATTTACTGGATCATCCGTGCTAGTTTCCAGTGAGCCACTG
TP88099_Query	D	1	chr4	8B	CTGCTCCTCGGTAAGGTTCTCGGAATTTACTGGATCATCCGTGCTAATTTCCAGTGAGCCACTG
TP93907_Hit	D	1	chr4	8B	CTGCTTCTTAACACCCAAAAACAAAAGAGACTGCATACTACACACGGACAACAAATAAAATCTC
TP93907_Query	D	1	chr4	8B	CTGCTTCTTAACACCCAAAAACAAAAGAGACTGCACACTACACACGGACAACAAATAAAATCTC
TP9541_Hit	D	1	chr4	8B	CAGCACAGCTTTTCTGCCTGTAAATTAATCCCGCTTCTAAAATTTACCAAAAATACCATGACAG
TP9541_Query	D	1	chr4	8B	CAGCACAGCTTTTCTGCCTGTAAATTAATCCCGCTTCTAAAATTCACCAAAAATACCATGACAG
TP13544_Hit	D+G	1	chr4	8C	CAGCAGACGATGCTCTGATAGCCTGCCACACTTGATGCTTACAACTTCCAATGAATGCACTGCG
TP13544_Query	D+G	1	chr4	8C	CAGCAGACGATGCTCTGATAGCCTGCCACACTTGATGCTTACAACTTCCAATGAATGAACTGCG
TP18598_Hit	D	1	chr4	8C	CAGCATCAGATTTCTCCCTTGCATCTTTCTCAGCCAAATCTTCCAAAAGTGTCTGAAAGATCAA
TP18598_Query	D	1	chr4	8C	CAGCATCAGATTTCTCCCTTGCATCTTTATCAGCCAAATCTTCCAAAAGTGTCTGAAAGATCAA
TP21066_Hit	D+G	1	chr4	8C	CAGCATTGGAGCACCTTGTTTTAGCAAGCATGGCAATGGTATCAAATGGCCAAGAAGTGGAAGT
TP21066_Query	D+G	1	chr4	8C	CAGCATTAGAGCACCTTGTTTTAGCAAGCATGGCAATGGTATCAAATGGCCAAGAAGTGGAAGT
TP24975_Hit	D	1	chr4	8C	CAGCCACCTTAAGAAGGTTGAAGCTTCTTTGTGGAGGTAGGAGAGGCCATCAATATTTACTTGC
TP24975_Query	D	1	chr4	8C	CAGCCACCTTAAGAAGGTTGAAGCTTCCTTGTGGAGGTAGGAGAGGCCATCAATATTTACTTGC
TP27286_Hit	D	1	chr4	8C	CAGCCATTATCCTAGGACGGGTATCAAACTCAATCCCTAAAACTTTAGCTAACGGTGTTGAAAC
TP27286_Query	D	1	chr4	8C	CAGCCATTATCCTAGGACGGGTATCAAACTCAATCCCTAAAACTTTAGCTAACGGTGTGGAAAC
TP32311_Hit	D	1	chr4	8C	CAGCCTCAAGCATTACTGCTTCATCATGTTCCACTGAGGACATGCCACCCCACTGTAAATATTG
TP32311_Query	D	1	chr4	8C	CAGCCTCAAGCATTACTGCTTCATCATGTTCCACTGAAGACATGCCACCCCACTGTAAATATTG
TP4007_Hit	D	1	chr4	8C	CAGCAACCATATGAGTTGCACCGATCTAGAATGGAAATCCCTCCTCCCTCCCCTTATCTGAACC
TP4007_Query	D	1	chr4	8C	CAGCAACCATATGAGTTGCACCAATCTAGAATGGAAATCCCTCCTCCCTCCCCTTATCTGAACC
TP40406_Hit	D	1	chr4	8C	CAGCGTCAAATCTTCCTCCTTCCATACAAAATGGATTGCCCTTGCATCCCAATCAAACCTTACC
TP40406_Query	D	1	chr4	8C	CAGCGTCAAATCTTCCTCCTTCCAGACAAAATGGATTGCCCTTGCATCCCAATCAAACCTTACC
TP50455_Hit	D	1	chr4	8C	CAGCTGAGACATCCAAAGGCTTCCAGGAACAATTAAGAATCATCTCAATCCATTCATTGTCAAC
TP50455_Query	D	1	chr4	8C	CAGCTGAGACATCCAAAGGCTTCCAAGAACAATTAAGAATCATCTCAATCCATTCATTGTCAAC
TP58179_Hit	D	1	chr4	8C	CAGCTTGTGCACAAACCAGCCTTGGTATCTTGTCAACAAGCCCTAAATCTTTACACATTTTGAA
TP58179_Query	D	1	chr4	8C	CAGCTTGTGCACAAACCAGCCTTGGTATCTTATCAACAAGCCCTAAATCTTTACACATTTTGAA
TP64039_Hit	D+G	1	chr4	8C	CTGCAATCAACAGATGCCACTTCCACTTCTTGGCCATTTGATACCATTGCCATGCTTGCTAAAA
TP64039_Query	D+G	1	chr4	8C	CTGCAATCAACAGACGCCACTTCCACTTCTTGGCCATTTGATACCATTGCCATGCTTGCTAAAA
TP79813_Hit	D	1	chr4	8C	CTGCGAAGCTCCCAGAAAGCATATACACATTGGCGTTTGGTCTACATCATGTTTTAGAAGTTCA
TP79813_Query	D	1	chr4	8C	CTGCGAAGCTCCCAGAAAGCATATACACATTGGCGTTTGGTCTACAACATGTTTTAGAAGTTCA
TP80209_Hit	D+G	1	chr4	8C	CTGCGAGAAATGGTAGTTGCTAAGGTATATCACTGGTCTTTGTTAACTTTCTAGTTTTTAATAT
TP80209_Query	D+G	1	chr4	8C	CTGCGAGAAATGGTAGTTACTAAGGTATATCACTGGTCTTTGTTAACTTTCTAGTTTTTAATAT
TP83077_Hit	D	1	chr4	8C	CTGCGTACCCGAAACGATGCCAAGGTGTTAAATCCACGAAGTTAGCTAGCTCGATGACTGAGAA
TP83077_Query	D	1	chr4	8C	CTGCGTACCCGAAACGATGCCAAGGTGTTAAATCCACGAAGTTAGCTAGCTCAATGACTGAGAA
TP88819_Hit	D	1	chr4	8C	CTGCTCTCATGAGAGCCAATGTTGATCCATCACTTGTGCAAGAAGTGTTTTTTGGGAATGTTCT
TP88819_Query	D	1	chr4	8C	CTGCTCTCAAGAGAGCCAATGTTGATCCATCACTTGTGCAAGAAGTGTTTTTTGGGAATGTTCT
TP89223_Hit	D	1	chr4	8C	CTGCTCTTGAATGTTAGGACTCTCAATAATGGTCATTTCAGTATCACGGTCAACTTCTTTTCCA
TP89223_Query	D	1	chr4	8C	CTGCTCTTGAATGTTAGGACTCTCAATAATGGTCATTTCAGTATCACGATCAACTTCTTTTCCA

Name	Filter	Nb hit (Mt4.0)	Mt Chr (Mt4.0)	Ms Chr (Li et al., 2014)	Sequence
TP91254_Hit	D	1	chr4	8C	CTGCTGGTTCTGAACTTCTAAAACATGTTGTAGACCAAACGCCAATGTGTATATGCTTTCTGGG
TP91254_Query	D	1	chr4	8C	CTGCTGGTTCTGAACTTCTAAAACATGATGTAGACCAAACGCCAATGTGTATATGCTTTCTGGG
TP15990_Hit	D	1	chr4	8D	CAGCAGTCCTCCGCATAATGACAATTCCTTCCCATCTGATGAAGTATAGTCCTTCACTTTATGA
TP15990_Query	D	1	chr4	8D	CAGCAGTCCTCCGCATAATAACAATTCCTTCCCATCTGATGAAGTATAGTCCTTCACTTTATGA
TP5423_Hit	D+G	1	chr4	8D	CAGCGAGATAATGAAGACCACTCCAAGGACTAACATCTCTACTAAGCATAATCAAGCTCATAGC
TP5423_Query	D+G	1	chr4	8D	CAGCAAGATAATGAAGACCACTCCAAGGACTAACATCTCTACTAAGCATAATCAAGCTCATAGC
TP5969_Hit	D	1	chr4	8D	CAGCAAGGACATGAAATGCATTATTATCATTAAGTTTAGGGTGGTTCCCATAAAGATATGCCAA
TP5969_Query	D	1	chr4	8D	CAGCAAGGACACGAAATGCATTATTATCATTAAGTTTAGGGTGGTTCCCATAAAGATATGCCAA
TP6702_Hit	D	1	chr4	8D	CAGCAATAAAACCACTTTGGATATCCTTCATGCTGATTAATGTTGTTTGAATCCTTGGTTATAT
TP6702_Query	D	1	chr4	8D	CAGCAATAAAACCACTTTGGATATCCTTCATGTTGATTAATGTTGTTTGAATCCTTGGTTATAT
TP68030_Hit	D	1	chr4	8D	CTGCAGAGTGCACATTAAATTTTTCCACTCGAGCTTCTGTACAGGATCTTTTATTCGACAATAA
TP68030_Query	D	1	chr4	8D	CTGCAGAGTGCACATTAAATTTTTCCACTCGAGCTTCTGTACAGGATCTGTTATTCGACAATAA
TP90124_Hit	D	1	chr4	8D	CTGCTGATCCCCTTTTTCCCAGATTCTGTGTTGGGGTTAAACTGATCGGCTCCAAATGCTAAGT
TP90124_Query	D	1	chr4	8D	CTGCTGATCCCCTTTTTCCCAGATTCTGTGTTGGGATTAAACTGATCGGCTCCAAATGCTAAGT
TP10055_Hit	D	1	chr5	.	CAGCACCAACAGCAGAAACTGGGGACACTTGGTTGAACCCACCACCAGCAGAAGAATTAGAGAC
TP10055_Query	D	1	chr5	.	CAGCACCAACAGCAGAAACTGGGGACACTTGGTTGAACCCACCACCAGCAGAAGAATTAGAAAC
TP10230_Hit	D	1	chr5	.	CAGCACCACCATCTCCACCACTTGGATTTGAAACATTCTCAGTCCTTTGAACCTTCTTTGGCCT
TP10230_Query	D	1	chr5	.	CAGCACCACCATCACCACCACTTGGATTTGAAACATTCTCAGTCCTTTGAACCTTCTTTGGCCT
TP10690_Hit	D	1	chr5	.	CAGCACCGACGGTGTAAATGACATCAAAGGTCAATTTTAAAAGGATTATTGAGTACATTGTATG
TP10690_Query	D	1	chr5	.	CAGCACCGACGGTGTAAATGACATCAAAGGTCAATTTTAAAAGGATTATTGAGTACAATGTATG
TP1070_Hit	D	1	chr5	.	CAGCAAAATTTGTCGGATGATGGATTCCATGCAAAGCATCCATCATATGACAGTGCGGCAACAC
TP1070_Query	D	1	chr5	.	CAGCAAAATTTGTCGGATGATGGATTCCATGCAAAGCATCCATCATATGACAGTGCGGAAACAC
TP10829_Hit	D	1	chr5	.	CAGCACCGTTTCATGCCTTTTTCCTCTCCTGCTCTTTTTCTTCACTCCTAAACCAGTGATAAAG
TP10829_Query	D	1	chr5	.	CAGCACCGTTTCATGCCTTTTTCCTCTCCTGCTCTTTTTCTTCACTCCTAAACCAGTAATAAAG
TP10927_Hit	D	1	chr5	.	CAGCACCTCTAAAATAACAAGAACCACCATTGTTCTTGAATTTCTGAAAGTAACTGTTGAAGGC
TP10927_Query	D	1	chr5	.	CAGCACCTCTAAAATAACAAGAACCACCATTGTTCTTGAATTTCTGAAAGTAACTGTTAAAGGC
TP11453_Hit	D	1	chr5	.	CAGCACGGATCTTGAAGGAAGTAATAGCGGAGGAGACAGATATCTAGGAATGCTAATATCCCTG
TP11453_Query	D	1	chr5	.	CAGCACGGATCTTGAAGGAAGTAATAGCGGAGGAGACAAATATCTAGGAATGCTAATATCCCTG
TP11567_Hit	D	1	chr5	.	CAGCACGGTTAGTGAGGAAGGAAACATCCTCATCATCCAATTCCAAAGCTTTCGAATAATGCTG
TP11567_Query	D	1	chr5	.	CAGCACGGTTAGTGAGGAAGGAAACATCCTCATCATCCAATTCCAAAGCTTTCGAATAATGCAG
TP11600_Hit	D	1	chr5	.	CAGCACGTCCCCTTGTCACAATCCAAACCCTAGAATCGGAAATGTCCACCGATTCACCAACCAC
TP11600_Query	D	1	chr5	.	CAGCACGTCCCCTCGTCACAATCCAAACCCTAGAATCGGAAATGTCCACCGATTCACCAACCAC
TP1169_Hit	D	1	chr5	.	CAGCAAACACACTTATTGTGATCTAAAGTAAGTGCATTGAAACAGTCAATGACAGTTAGTAGAG
TP1169_Query	D	1	chr5	.	CAGCAAACACACTTATTGTGATCTAAAGTAAGTGCATTGAAACAGTCAATGACAGTTAGCAGAG
TP11730_Hit	D+G	1	chr5	.	CAGCACTAAGAACATCATCTTGCTTACTGAATATGTTCTTCCGTGGAACATGCCACATTTGCGA
TP11730_Query	D+G	1	chr5	.	CAGCACTAAGAACATCATCTTGCTTACTGAATATGTTCTTCCGTGGAACATACCACATTTGCGA
TP11759_Hit	D	1	chr5	.	CAGCACTATTGAGTGAAAACCTTAACAAAGCTAAAGAGACTGAATTTTAACTCATTTGATTGTT
TP11759_Query	D	1	chr5	.	CAGCACTAATGAGTGAAAACCTTAACAAAGCTAAAGAGACTGAATTTTAACTCATTTGATTGTT
TP11766_Hit	D	1	chr5	.	CAGCACTAATTAATGACCCTGATGCAGGAATTGGTTCAGATTGGAGAGACAGGTACCTTGGCAT
TP11766_Query	D	1	chr5	.	CAGCACTAATTAATGACCCTGATGCAGAAATTGGTTCAGATTGGAGAGACAGGTACCTTGGCAT
TP1186_Hit	D+G	1	chr5	.	CAGCATACACCTGATCCATGTTATACACTGTCATCAAGTACACCATGAAATTTGTTTGTATTCC
TP1186_Query	D+G	1	chr5	.	CAGCAAACACCTGATCCATGTTATACACTGTCATCAAGTACACCATGAAATTTGTTTGTATTCC
TP12092_Hit	D	1	chr5	.	CAGCACTCGTACATGAAAGCACATGAACCTGCATCTGATTATGTAGATGGTCGAAACAAGGCTG
TP12092_Query	D	1	chr5	.	CAGCACTCGTACATGAAAGCACATGAACCTGCATCTGATTATGTAGATGATCGAAACAAGGCTG
TP12113_Hit	D	1	chr5	.	CAGCACTCTCCAAACATGCAAGCTAAGTACTTGGAGCTCATGCTTGCAATGAATGAGAATGCCA
TP12113_Query	D	1	chr5	.	CAGCACTCTCCAAACATGCAAGCTAAGTACTTGGAGCTCATGCTTGCAATGAATGAAAATGCCA
TP12204_Hit	D	1	chr5	.	CAGCACTGATAGAACACCCAAAGACACTAATATTTGGTGCACATACGTTGCATTCATATAATAT
TP12204_Query	D	1	chr5	.	CAGCACTGATAGAACACCCAAAGACACTAATATTTGGTGCACATACATTGCATTCATATAATAT
TP12329_Hit	D	1	chr5	.	CAGCACTGTACAAACTCCCCCTTTTTGTTTAATACATATTAATTTTGACTACATTCTGAGATCA
TP12329_Query	D	1	chr5	.	CAGCACTGTACAAACTCCCCCTTTTTGCTTAATACATATTAATTTTGACTACATTCTGAGATCA
TP12478_Hit	D	1	chr5	.	CAGCACTTCAAAGGATCCTCGTGGGTCGTCTTTGACAAAGGAGGGGAAATCTTCACGACGGGAC
TP12478_Query	D	1	chr5	.	CAGCACTTCAAAGGATCCTCGTGGGCCGTCTTTGACAAAGGAGGGGAAATCTTCACGACGGGAC
TP12669_Hit	D	1	chr5	.	CAGCACTTTACCTTGTCAAAAACTTGCTTCAGTATTACATTTTTGCATATGTAGATGCTCCTGG
TP12669_Query	D	1	chr5	.	CAGCACTTTACCTTGTCAAAAACTTGCTTCAGTATTACATCTTTGCATATGTAGATGCTCCTGG

Name	Filter	Nb hit (Mt4.0)	Mt Chr (Mt4.0)	Ms Chr (Li et al., 2014)	Sequence
TP1271_Hit	D	1	chr5	.	CAGCAAACATGATTTTACCATTAAATTGCATAAACCTACAACCAGGGAAGGAAACTATGCTCCA
TP1271_Query	D	1	chr5	.	CAGCAAACATGATTTTACCACTAAATTGCATAAACCTACAACCAGGGAAGGAAACTATGCTCCA
TP128_Hit	D	1	chr5	.	CAGCAAAAACAGATAAAAAAAATGACTCTAATGCTCAAAAGTAGACACCACAAAGCCGCAGGAA
TP128_Query	D	1	chr5	.	CAGCAAAAACAGATAAAAAAAAAGACTCTAATGCTCAAAAGTAGACACCACAAAGCCGCAGGAA
TP12865_Hit	D	1	chr5	.	CAGCAGAAACGTAAGGAAAAAACGTGTTGGGCTCTACGAACCGGGTCGGGTCAAAATAAAAGAA
TP12865_Query	D	1	chr5	.	CAGCAGAAACGTAAGGAAAAAACGTGCTGGGCTCTACGAACCGGGTCGGGTCAAAATAAAAGAA
TP12870_Hit	D+G	1	chr5	.	CAGCAGAAACTCTGAGTGCAACAAATAGCTCTTGTTCGGATTTTTAGTTCTTGATCATTCAAAA
TP12870_Query	D+G	1	chr5	.	CAGCAGAAACTCTGAGTGCAACAAATAGCTCTTGTTCGGATTTCTAGTTCTTGATCATTCAAAA
TP12914_Hit	D	1	chr5	.	CAGCAGAGAGTTTAACAAATTCCCAAAGCCCAGAAAAAGCCTCCATAGAAAACCCATTCCAAGT
TP12914_Query	D	1	chr5	.	CAGCAGAAAGTTTAACAAATTCCCAAAGCCCAGAAAAAGCCTCCATAGAAAACCCATTCCAAGT
TP12947_Hit	D	1	chr5	.	CAGCAGAAATGTTGAAATTTGGCTGAGAACACCCATCAGTCAGTTTCATATTTGTTTCATGAAA
TP12947_Query	D	1	chr5	.	CAGCAGAAATGTTGAAATTTGGCCGAGAACACCCATCAGTCAGTTTCATATTTGTTTCATGAAA
TP13019_Hit	D	1	chr5	.	CAGCTGAACCATAAACAGAACTATATTGTCCTCCATACTGAGCAACAGAAGCATGAGCCCTACT
TP13019_Query	D	1	chr5	.	CAGCAGAACCATAAACAGAACTATATTGTCCTCCATACTGAGCAACAGAAGCATGAGCCCTACT
TP13211_Hit	D	1	chr5	.	CAGCAGAAGGCGCACGAGGGTAAAGAAAGATTTCAATACGTTCGTTTACTACTTGATGTGCATC
TP13211_Query	D	1	chr5	.	CAGCAGAAGGCGCACGAGGGTAAAGAAAGATTTCAATACGGTCGTTTACTACTTGATGTGCATC
TP13376_Hit	D+G	1	chr5	.	CAGCCGAATTCTTTTTAATAATGACAGATTGGACAGCCTTATATTCGACTCGATTGTCAAGTTG
TP13376_Query	D+G	1	chr5	.	CAGCAGAATTCTTTTTAATAATGACAGATTGGACAGCCTTATATTCGACTCGATTGTCAAGTTG
TP13411_Hit	D	1	chr5	.	CAGCAGACAACTGATATCTACCACATAACTTTGCCGGGCAATGTGATTTACCAGCTTCATGCAG
TP13411_Query	D	1	chr5	.	CAGCAGACAACTGATATCTACCACATAACTTTGCCGGGCAATGTGATCTACCAGCTTCATGCAG
TP13434_Hit	D	1	chr5	.	CAGCAGAGACTTTGTTAAATCCATGGAAACAAGTTTGATACTGCACATGAAATGCTCTAACTAA
TP13434_Query	D	1	chr5	.	CAGCAGACACTTTGTTAAATCCATGGAAACAAGTTTGATACTGCACATGAAATGCTCTAACTAA
TP1359_Hit	D+G	1	chr5	.	CAGCAAACCTCAACCTTTGATGTTTTCTGACCAGCAGAACGTTATAGCTGGTGGTCAACGGAGT
TP1359_Query	D+G	1	chr5	.	CAGCAAACCTCAACCTTTGAAGTTTTCTGACCAGCAGAACGTTATAGCTGGTGGTCAACGGAGT
TP13642_Hit	D+G	1	chr5	.	CAGCAGAGAAAGTTGCTCCTTCGATTGGTGTAGTAGGGTAGTATAGATAGGATGGTCCAGCTTG
TP13642_Query	D+G	1	chr5	.	CAGCAGAGAAAGTTGCTCCTTCCATTGGTGTAGTAGGGTAGTATAGATAGGATGGTCCAGCTTG
TP13737_Hit	D	1	chr5	.	CAGCAGAGCAATGGGTTTCTCCAGGGCATGGAAATGATTGGAAGCAAGGATATGATGTCGCTGA
TP13737_Query	D	1	chr5	.	CAGCAGAGCAATGGGTTTCTCCAGGGCATGGAAATGATTGGAAGCAAGGATATGATGTCACTGA
TP13742_Hit	D	1	chr5	.	CAGCAGAGCACAGCTCCACTCGACACCATGATTTGACAATTGCGCGCTAAATCGCCATGATTCA
TP13742_Query	D	1	chr5	.	CAGCAGAGCACAGCTCCACTCGACACCATGATTTGACAATTGCGCGCTAAATCGCCACGATTCA
TP13774_Hit	D	1	chr5	.	CAGCAGAGCCACCATCCACGTGTAAATAATAATACATGAAGGAGTCAGGACTCACATGCGTCAG
TP13774_Query	D	1	chr5	.	CAGCAGAGCCACCATCCACGTGTAAATAATAATACATGAAGGAATCAGGACTCACATGCGTCAG
TP14075_Hit	D	1	chr5	.	CAGCAGATATGTCAAAATTTCAGCTCCCATTTCTCTCTTTTCTGATACCATGTAATCTGGCAAC
TP14075_Query	D	1	chr5	.	CAGCAGATATGTCAAAATTTCAGCTCCCATCTCTCTCTTTTCTGATACCATGTAATCTGGCAAC
TP14119_Hit	D	1	chr5	.	CAGCAGATCCAACTTTCCATTTTTCAAAAAATTTGTGCTGGACAGCCAAAAAAGAACTTGACTC
TP14119_Query	D	1	chr5	.	CAGCAGATCCAACTTTCCATTTTTCAAAAAATTTGTGCTGGACAGCCAAAAAAGAACATGACTC
TP14172_Hit	D	1	chr5	.	CAGCAGATGAAGCAAGTTCATTCAAACAAGAATGTTCTGAGCTCAAATCCAAAACAGAAAAACT
TP14172_Query	D	1	chr5	.	CAGCAGATGAAGCAAGTTCATTCAAACAAGAATGTTCTGAGCTCAAATCAAAAACAGAAAAACT
TP14231_Hit	D	1	chr5	.	CAGCAGATGGATTGCTGGTTATCAAGGAATTCAAATTAGTTGCTGGATTTTGCTGTGGTCCTGA
TP14231_Query	D	1	chr5	.	CAGCAGATGGATTGCTGGTTATCAAGGAATCCAAATTAGTTGCTGGATTTTGCTGTGGTCCTGA
TP14303_Hit	D	1	chr5	.	CAGCAGATTCGCGGTTCTTCGCCATTCTCTTCTGCCTCCTCTCAATAGTCTTTTCCATCATCTC
TP14303_Query	D	1	chr5	.	CAGCAGATTCGCGGTTCTTCGCCATTCGCTTCTGCCTCCTCTCAATAGTCTTTTCCATCATCTC
TP14425_Hit	D+G	1	chr5	.	CAGCAGGAACCCAAGTACTCTTAACATGAGTTTGACAATCAAAACCTCTACTTTTACAACAAGT
TP14425_Query	D+G	1	chr5	.	CAGCAGGAACCCAAGTACTCTTAACATGAGTTTGACAATCAAAACCTCTACTCTTACAACAAGT
TP14461_Hit	D	1	chr5	.	CAGCAGGAAGGGGTCAGACAAACTCTCGGTATTAAAACACAAACCTGCTGTATGATGCGATTTA
TP14461_Query	D	1	chr5	.	CAGCAGGAAGGGGTCAGACAAACTCTCGGTATTAAAACACAAACCTGCTGTACGATGCGATTTA
TP14494_Hit	D	1	chr5	.	CAGCAGGAGTTATTTATGGCTCAGAAGGAATGAAACCACTCCAAAAGAAGTTCCCTAATTTGTT
TP14494_Query	D	1	chr5	.	CAGCAGGAATTATTTATGGCTCAGAAGGAATGAAACCACTCCAAAAGAAGTTCCCTAATTTGTT
TP14582_Hit	D	1	chr5	.	CAGCAGGAGATGGTTCAACATGTGGTGATGGATCAACAGGCTTTCCAAGTAAATCAGCTGGTAA
TP14582_Query	D	1	chr5	.	CAGCAGGAGATGGTTCAACATGTGGTGAAGGATCAACAGGCTTTCCAAGTAAATCAGCTGGTAA
TP1469_Hit	D	1	chr5	.	CAGCAAACTCTGCAGACATGGAACCATTTGAAACACTTAATTGATCATGTTCAGGGTTTACCTA
TP1469_Query	D	1	chr5	.	CAGCAAACTCTGCAGACATGGAACCACTTGAAACACTTAATTGATCATGTTCAGGGTTTACCTA
TP14903_Hit	D+G	1	chr5	.	CAGCAGGCTGTGAAAAATGGGGTTGATTTTGTTTTGCCGGTGGAGAATTCTTTGGTTCATATGT
TP14903_Query	D+G	1	chr5	.	CAGCAGGCTCTGAAAAATGGGGTTGATTTTGTTTTGCCGGTGGAGAATTCTTTGGTTCATATGT

Name	Filter	Nb hit (Mt4.0)	Mt Chr (Mt4.0)	Ms Chr (Li et al., 2014)	Sequence
TP1509_Hit	D+G	1	chr5	.	CAGCAAACTTCATGCCTGAATCTATGAAATCTGGTTTTGAGTATGACGGTGAAGGCAAACTAGA
TP1509_Query	D+G	1	chr5	.	CAGCAAACTTCATGCCTGAATCTATGAAATCTGGTTTTGAGTATGACGGTGAAGGCAAACAAGA
TP15178_Hit	D	1	chr5	.	CAGCAGGTAATGTTTTAATCAAAAAATATTGATTGGTGTTCAAATGTTAGCTTTATTACTTTAA
TP15178_Query	D	1	chr5	.	CAGCAGGTAATGTTTTAATCAAAAAATATCGATTGGTGTTCAAATGTTAGCTTTATTACTTTAA
TP15190_Hit	D	1	chr5	.	CAGCCGGTACCCAAGTACTCTTAACATGAGTAGGGCAATCAAAACCTCTACTTTTACAACAAGT
TP15190_Query	D	1	chr5	.	CAGCAGGTACCCAAGTACTCTTAACATGAGTAGGGCAATCAAAACCTCTACTTTTACAACAAGT
TP15286_Hit	D+G	1	chr5	.	CAGCAGGTGAAGGATGAAGGATTTCTTAAGGATGTGGATGAATTGTTTGAAGGGATTTGCCCGG
TP15286_Query	D+G	1	chr5	.	CAGCAGGTGAAGGATGAAGGAATTCTTAAGGATGTGGATGAATTGTTTGAAGGGATTTGCCCGG
TP15526_Hit	D+G	1	chr5	.	CAGCAGTAACACTAACAACAACACCAACAATAAAAGTAACCGTAAATGCAAAGGTAAAGGTGGA
TP15526_Query	D+G	1	chr5	.	CAGCAGTAACACCAACAACAACACCAACAATAAAAGTAACCGTAAATGCAAAGGTAAAGGTGGA
TP15754_Hit	D+G	1	chr5	.	CAGCAGTAGGGTCACTTATCAGAAGAGTCCTGTTGGTATAAGCATCGGTGGAATATTCTACCAC
TP15754_Query	D+G	1	chr5	.	CAGCAGTAGGGTCACTTATCAAAAGAGTCCTGTTGGTATAAGCATCGGTGGAATATTCTACCAC
TP16131_Hit	D	1	chr5	.	CAGCAGTGAGAGTCATTAGATCACCTGGCGTTTGAATATCGATCGCAGAATTAACAGTAGAAGT
TP16131_Query	D	1	chr5	.	CAGCAGTGAGAGTCATTAGATCACCTGGCGTTTGAATATCGACCGCAGAATTAACAGTAGAAGT
TP16342_Hit	D	1	chr5	.	CAGCAGTGTAGAGAGGGGAAGCCAAACCAATCATCATCATCATGGGTTCCTCTTCATTACCATC
TP16342_Query	D	1	chr5	.	CAGCAGTGTAGAGAGGGGAACCCAAACCAATCATCATCATCATGGGTTCCTCTTCATTACCATC
TP16474_Hit	D	1	chr5	.	CAGCAGTTACTTCACTAAAACAAACCCATGCCTGTCCTCGAAGCTTAGGTGTCTTTAGAGCAAT
TP16474_Query	D	1	chr5	.	CAGCAGTTACTTCACTAAAACAAACCCATGCCTGTCCTCGAAACTTAGGTGTCTTTAGAGCAAT
TP16560_Hit	D	1	chr5	.	CAGCAGTTCCAATTACACCAAATTGAAACTTAAAGACAAACAACCAGCTCACAAAAACATGAAC
TP16560_Query	D	1	chr5	.	CAGCAGTTCCAATTACACCAAATTGAAACTTAAACACAAACAACCAGCTCACAAAAACATGAAC
TP16698_Hit	D	1	chr5	.	CAGCAGTTGCTTCTCTACCAAGTGCCACCAAGGCAGATTTATGCTCTTTCAACTTTTTTTCTGC
TP16698_Query	D	1	chr5	.	CAGCAGTTGCTTCTCTACCAAGTGCCACCAAGGCAGATTTATGCTCTTTCAACTTTTTTTCTGA
TP16800_Hit	D	1	chr5	.	CAGCAGTTTCACAACAGATTCTGGGAGTTCAGAGCTCAAACTAGAAGTTTTGTTGCTTACTTCC
TP16800_Query	D	1	chr5	.	CAGCAGTTTCACAACAGATTCTGGGAGTTCAGTGCTCAAACTAGAAGTTTTGTTGCTTACTTCC
TP16880_Hit	D	1	chr5	.	CAGCAGTTTTGTGAAATTCAAGCATGTGTTGAGTGTTCATCGTATAAGACATTCGAGGTATCTT
TP16880_Query	D	1	chr5	.	CAGCAGTTTTGCGAAATTCAAGCATGTGTTGAGTGTTCATCGTATAAGACATTCGAGGTATCTT
TP16922_Hit	D	1	chr5	.	CAGCATAAAAAATGGCATACCTCCTCATATGTATAATCACGGTCACTCCCTTCCCAAGGATAAC
TP16922_Query	D	1	chr5	.	CAGCATAAAAAATGGCATACCTCCTCATATGTATAATCACGATCACTCCCTTCCCAAGGATAAC
TP17046_Hit	D+G	1	chr5	.	CAGCATAAATTTGAAAGTGACTTTTAATAGTGTAATTTTCCCATTTTGGTATAGTAACTTTAGA
TP17046_Query	D+G	1	chr5	.	CAGCATAAATTTGAAAGTGACTTTTAATAGTGTAATTTCCCCATTTTGGTATAGTAACTTTAGA
TP17133_Hit	D	1	chr5	.	CAGCATAAGAAACTGTCGCCATTGCTCTCCCGAAATACTCTCCGATCATCTTCATGAAGCTCTG
TP17133_Query	D	1	chr5	.	CAGCATAAGAAACTGTCGCCATTGCTCTCCCGAAATACTCTCCGATCATCTTCATGAAGCTCTA
TP17138_Hit	D	1	chr5	.	CAGCATAAGGCTTCCAAAAGATCAAATACGAGATATCAGAAGTGGAATGACTGAACCCTCTTCC
TP17138_Query	D	1	chr5	.	CAGCATAAGACTTCCAAAAGATCAAATACGAGATATCAGAAGTGGAATGACTGAACCCTCTTCC
TP17197_Hit	D	1	chr5	.	CAGCATAATAACCCTTTTGGTAATTCTCTTTACCCTTATAATGGTGGTGTTCCCGCGGTAACCG
TP17197_Query	D	1	chr5	.	CAGCATAATAACCCTTTTGGTAATTCTCTTTACCCTTATAATGGTGGTGTACCCGCGGTAACCG
TP17216_Hit	D	1	chr5	.	CAGCATAATATACACAGCAATGAACATGTGGATATGAACAAGCATGCAATAGCACCCTTGAGGA
TP17216_Query	D	1	chr5	.	CAGCATAATATACACAGCAATGAACACGTGGATATGAACAAGCATGCAATAGCACCCTTGAGGA
TP17267_Hit	D	1	chr5	.	CAGCATAATGGTGGCTGGACAGTGGTTTTATCGTCCTGAAGAAGCTGAAAAAAAAGGCGGTGGA
TP17267_Query	D	1	chr5	.	CAGCATAATGGTGGCTGGACAGTGGTTTTATCGTCCTGAAGAAGCTGAAAAAAAAGGCGGGGGA
TP17383_Hit	D	1	chr5	.	CAGCATACAGTTCAGTGTACGACAAGAACGCTGATGATCATATCCACTCTGAGCCTGTTCCTGA
TP17383_Query	D	1	chr5	.	CAGCATACAGTTCAGTGTACGACAAGAACGCTGATGATCAGATCCACTCTGAGCCTGTTCCTGA
TP17587_Hit	D	1	chr5	.	CAGCATAGAGCTGATTGGGGGGAACCTCGCGAAGATAATCATTTGCCACATTCAACAGGTGTGA
TP17587_Query	D	1	chr5	.	CAGCATAGAGCTGATTGGGGGGAACCTCGCGAAGATAATCATTTGCCACATTCAACAGGTGAGA
TP17641_Hit	D	1	chr5	.	CAGCATAGCTCAGACAAACATGTCAATAATAACTCGGTTACATTTCTATATTGAACCAAAATAA
TP17641_Query	D	1	chr5	.	CAGCATAGCTCAGACAAACATGTCAATAATAACTCGGTTACATTTCTATATTAAACCAAAATAA
TP17657_Hit	D	1	chr5	.	CAGCATAGGAGGTGAAAAAGGTGAGACAAAGGTGAGACTCTTCTAGTAAACTGAGGTAAACTGT
TP17657_Query	D	1	chr5	.	CAGCATAGGAGGTGAAAAAGGTGAGACAAAAGTGAGACTCTTCTAGTAAACTGAGGTAAACTGT
TP17782_Hit	D	1	chr5	.	CAGCATATACAACTCCGCAGGCCACAACCTCACTCTTAAATCTAACACACAATGAAGTGCGCAA
TP17782_Query	D	1	chr5	.	CAGCATATACAACTCCACAGGCCACAACCTCACTCTTAAATCTAACACACAATGAAGTGCGCAA
TP17795_Hit	D+G	1	chr5	.	CAGCATATACCTCAAACCATGATGATGCTGAACCAATGTTTGTGAATGAGGATGAGAAACGAGC
TP17795_Query	D+G	1	chr5	.	CAGCATATACCTCAAACCATGATGATGCTGAACCAATGTTTGTGAATGAGGAAGAGAAACGAGC
TP17891_Hit	D	1	chr5	.	CAGCATATCATCATAAAATCCAAATGAGGTACCTTGTTCAAGTCCTCAACTAGGGAAGAGCATG
TP17891_Query	D	1	chr5	.	CAGCATATCATCATAAAATCCAAAAGAGGTACCTTGTTCAAGTCCTCAACTAGGGAAGAGCATG

Name	Filter	Nb hit (Mt4.0)	Mt Chr (Mt4.0)	Ms Chr (Li et al., 2014)	Sequence
TP17968_Hit	D	1	chr5	.	CAGCATATGAGTGGACAACCAAATATTGTCGAGTTTAAAGGTGCGTACGAGGATAAAAATTCAG
TP17968_Query	D	1	chr5	.	CAGCATATGAGTGGACAACCAAATATTGTCGAGTTTAAAGGTGCGTACAAGGATAAAAATTCAG
TP1815_Hit	D	1	chr5	.	CAGCAAAGGATTCAAGAGTCTAAGGATATACCAAGACTGGTCAGTTATAGTCATTAAAATGGTG
TP1815_Query	D	1	chr5	.	CAGCAAAGGACTCAAGAGTCTAAGGATATACCAAGACTGGTCAGTTATAGTCATTAAAATGGTG
TP18243_Hit	D	1	chr5	.	CAGCATCAAATTCACCTTATTGCATGTATTTTATTTGAGGAAGGTCTTGGGGCACCAGGAGGTG
TP18243_Query	D	1	chr5	.	CAGCATCAAATTCACCTTATTGCATGTATTTTATCTGAGGAAGGTCTTGGGGCACCAGGAGGTG
TP18293_Hit	D+G	1	chr5	.	CAGCCTCAACATTACATTTTACCTCTCCTTCCGTTGGTGGTAGCCATCGAATTTGGGTGTTGTT
TP18293_Query	D+G	1	chr5	.	CAGCATCAACATTACATTTTACCTCTCCTTCCGTTGGTGGTAGCCATCGAATTTGGGTGTTGTT
TP18425_Hit	D	1	chr5	.	CAGCATCAATTAACTTGCCTCATCAGAGTAATGCTTTGAAAGTGGGATACATAGCACTAAGTGG
TP18425_Query	D	1	chr5	.	CAGCATCAATTAACTTGCCCCATCAGAGTAATGCTTTGAAAGTGGGATACATAGCACTAAGTGG
TP18697_Hit	D+G	1	chr5	.	CAGCATCATAAGCTATTTGCAGAATTTTCATCCCGATGATCATGGACCTTTTGGAATTACAGCC
TP18697_Query	D+G	1	chr5	.	CAGCATCATAAGCTATTTGCAGAATTTTCATCCCGATGATCATGGACCTTCTGGAATTACAGCC
TP18719_Hit	D	1	chr5	.	CAGCATCATATTCTTATAGGATCCCCTAGCAAGAAGCTTGATGATATTCTTATGTTTGATTGAA
TP18719_Query	D	1	chr5	.	CAGCATCATATTCTTATAGGATCCCCAAGCAAGAAGCTTGATGATATTCTTATGTTTGATTGAA
TP18733_Hit	D	1	chr5	.	CAGCATCATCACCTTTAAAAGAAGCTTTATAAACAGAACCTTTAATCTTATTCTCTTCACTGAA
TP18733_Query	D	1	chr5	.	CAGCATCATCACCTTTAAAAGAAGCTCTATAAACAGAACCTTTAATCTTATTCTCTTCACTGAA
TP1889_Hit	D	1	chr5	.	CAGCAAAGGTCATCCACTAGACATCAGCAAAGGTTCATCACTAAAACTACATAAGTTTGATTGT
TP1889_Query	D	1	chr5	.	CAGCAAAGGTCATCCACCAGACATCAGCAAAGGTTCATCACTAAAACTACATAAGTTTGATTGT
TP19052_Hit	D	1	chr5	.	CAGCATCCTGAAGACGGGGTTGAGGAATTCCCTGTGATATAGTTAAGACAAGGTGATAGGCTGA
TP19052_Query	D	1	chr5	.	CAGCATCCTGAAGACGGGGTTGAGGAACTCCCTGTGATATAGTTAAGACAAGGTGATAGGCTGA
TP19264_Hit	D	1	chr5	.	CAGCATCTACAGGAGACAGAGTCGTAGCTTTGTTGGCCAACTTGAATAAAGCCGCAGAAAAAAA
TP19264_Query	D	1	chr5	.	CAGCATCTACAGGAGACAGAGTCATAGCTTTGTTGGCCAACTTGAATAAAGCCGCAGAAAAAAA
TP19310_Hit	D	1	chr5	.	CAGCATCTATGAACCGGTTACCTCCACATCCGGTAACTCTTGAACATCACTATATCTATGTTTT
TP19310_Query	D	1	chr5	.	CAGCATCTATGAACCGGTTACCTCCACATCCGGTAACTCTTGAACATCACTAGATCTATGTTTT
TP1933_Hit	D+G	1	chr5	.	CAGCAAAGTCAGCATCTATGGCATCATCTAGCAATGTTTTTCCGTGATGAGCTCCTGTCTTGGT
TP1933_Query	D+G	1	chr5	.	CAGCAAAGTCAGCATCTATGGCATCATCTAGCAATGTTTTTCCGTGATGAGCTACTGTCTTGGT
TP19437_Hit	D	1	chr5	.	CAGCATCTGCCTCCAAGCACCCTCATCATCTGCCTGGCCGTAATGTGTTCGATGACATTGCTGA
TP19437_Query	D	1	chr5	.	CAGCATCTGCCTCCAAGCACCCTCATCACCTGCCTGGCCGTAATGTGTTCGATGACATTGCTGA
TP19527_Hit	D	1	chr5	.	CAGCATCTTCGGCAGTTGAAAAAGTACCAAGCCATTTACGAGTACGTTTACGTGGTTCACGAAT
TP19527_Query	D	1	chr5	.	CAGCATCTTCAGCAGTTGAAAAAGTACCAAGCCATTTACGAGTACGTTTACGTGGTTCACGAAT
TP19595_Hit	D	1	chr5	.	CAGCATCTTTACGCTCAGCGCCACTCTTAAACTCAGCAAGATACCTATGATAATCACCTTTCAT
TP19595_Query	D	1	chr5	.	CAGCATCTTTACGCTCAGCACCACTCTTAAACTCAGCAAGATACCTATGATAATCACCTTTCAT
TP19649_Hit	D	1	chr5	.	CAGCATGAAAACTATGTGAATCAATTAAGATTTAGGGACGTTATGAGAAGAGCAACATCTATAC
TP19649_Query	D	1	chr5	.	CAGCATGAAAAATATGTGAATCAATTAAGATTTAGGGACGTTATGAGAAGAGCAACATCTATAC
TP19900_Hit	D	1	chr5	.	CAGCATGATAAAGTTCAAGAGCAGGTAAAGTATCAACAAGACGACGATCAGTTTCAACTTCGGG
TP19900_Query	D	1	chr5	.	CAGCATGATAAAGTTCAAGAGCAGGTAAAGTATCAACAAGACGACGATCAGTTTCAACTTCAGG
TP19934_Hit	D	1	chr5	.	CAGCATGATCAGGGTAACATGCCAACCACTCAACAAATACCAAAATGCCTGGTAAAAGATAGCT
TP19934_Query	D	1	chr5	.	CAGCATGATCAGGGTAACATGCCAACCACTCAACAAATACCAAAATGCCTGGTAAAAGATAACT
TP19965_Hit	D	1	chr5	.	CAGCATGATGATCCCTCAGTGCTACACAAAACTCCATCCGATTGCAGTTTCGAGGTGCTGAAAA
TP19965_Query	D	1	chr5	.	CAGCATGATGATCCCTCAGTGCTACACAAAACTCCATCCCATTGCAGTTTCGAGGTGCTGAAAA
TP19998_Hit	D+G	1	chr5	.	CAGCATGATTGAGGATTCCATAAGGGTGTTTAATCAAGTTGCTTATGCAAGCCATGTGAGTTGA
TP19998_Query	D+G	1	chr5	.	CAGCATGATTGAGGATTCCATAAAGGTGTTTAATCAAGTTGCTTATGCAAGCCATGTGAGTTGA
TP20_Hit	D	1	chr5	.	CAGCAAAAAAACAAAACCATCAAATTCGACTTTGAACCAATTGCTATAATACAATAAAGTAAGA
TP20_Query	D	1	chr5	.	CAGCAAAAAAACAAAACCATCAAATTCGACTTTGAACCAATTGCTATAATAAAATAAAGTAAGA
TP20115_Hit	D	1	chr5	.	CAGCATGCATCAAACTATTTGAAGAATCCAAGTCCTATGAAGGACTATATACTTTTTCTTGGAT
TP20115_Query	D	1	chr5	.	CAGCATGCATCAAACTATTTGAAGAATCCAAGTCCTATGAAGGACTATATACTTTTACTTGGAT
TP20518_Hit	D	1	chr5	.	CAGCATGGTTGAGGATTCCATAAAGGTGTTCAATCAACTTGCTTATGCAAGCCATGTGACTTGG
TP20518_Query	D	1	chr5	.	CAGCATGGTTGAGGATTCCATAAAGGTGTTCAGTCAACTTGCTTATGCAAGCCATGTGACTTGG
TP20802_Hit	D+G	1	chr5	.	CAGCATGTTCATAGAAAAGAGGCAAATTTGCACTCCCGTGTGGGCTTCCCATCATCGGTGGTGA
TP20802_Query	D+G	1	chr5	.	CAGCATGTTCATAGAAAAGAGGCAAATTTACACTCCCGTGTGGGCTTCCCATCATCGGTGGTGA
TP20853_Hit	D	1	chr5	.	CAGCATGTTGTTTTGCTTCAATCTTTATCCCTTTGTTTAAGACACCAACAATCATTTCTAATCC
TP20853_Query	D	1	chr5	.	CAGCATGTTGTTTTGCTTCAATCTTTATCCCTTTGTTTAAGACACCAACAATCATTTCTAAGCC
TP20937_Hit	D+G	1	chr5	.	CAGCATTAACTATCAGCACTGCGGAGCGTCAAAAACTTGGTATGGTATCCCTGGTCACGCTGAA
TP20937_Query	D+G	1	chr5	.	CAGCATTAACTATCAGCACTGCGGAGCGTCAAAAACTTGGTATGGTATCCCTGGTCACGCAGAA

Name	Filter	Nb hit (Mt4.0)	Mt Chr (Mt4.0)	Ms Chr (Li et al., 2014)	Sequence
TP21045_Hit	D	1	chr5	.	CAGCATTACTTCCGATTCTTCGCCAACCGCCTTTATCAGACACTCATTCTCCACGTGCTGAAAA
TP21045_Query	D	1	chr5	.	CAGCATTACTTCCGATTCTTCGCCAACCGCCTTTATCAGACACTCATTCTCCACATGCTGAAAA
TP21050_Hit	D	1	chr5	.	CAGCATTATTTGGAGAAGGGAAAGATCCAGCGTTATCTTCTAAAAGCGTCCAAGGATCAATTTC
TP21050_Query	D	1	chr5	.	CAGCATTACTTGGAGAAGGGAAAGATCCAGCGTTATCTTCTAAAAGCGTCCAAGGATCAATTTC
TP21055_Hit	D	1	chr5	.	CAGCATTAGAAATTGGGCAAAATTTCTCATTTTTAAAAATCAGCTGGTTTCTTCCTTCCCAAAG
TP21055_Query	D	1	chr5	.	CAGCATTAGAAATTGGGCAAAATTTCTCATTTTTAAAAATCAGCTGGATTCTTCCTTCCCAAAG
TP21651_Hit	D	1	chr5	.	CAGCGTTGAGTTTAACAGTTAGCTCTCGCTTCTCGTATTGAAGTTCTTTATTTTTCCTCTTAAG
TP21651_Query	D	1	chr5	.	CAGCATTGAGTTTAACAGTTAGCTCTCGCTTCTCGTATTGAAGTTCTTTATTTTTCCTCTTAAG
TP2174_Hit	D	1	chr5	.	CAGCAAATCAGATGAAAAAGAAAATGAACCTGTTTCTCAAGTTGATGATAAAAAAGTTACTAGT
TP2174_Query	D	1	chr5	.	CAGCAAATCAGATGAAAAAGAAAATGAACCTGTTTCTCAAGTTGATGATAAAAAAGTTACCAGT
TP21814_Hit	D	1	chr5	.	CAGCATTGGCTCGTCAGCGAGCTCTTGACACGAAAAGGCAGTCACCATTTTCAGCTGAAAAAAA
TP21814_Query	D	1	chr5	.	CAGCATTGGCTCGTCAGCGAGCTCTTGACACAAAAAGGCAGTCACCATTTTCAGCTGAAAAAAA
TP21830_Hit	D	1	chr5	.	CAGCATTGGTACTATCAATGATGTGTAGACTCGACCTTAACTAAGTAAATATTGGGTTACGCTA
TP21830_Query	D	1	chr5	.	CAGCATTGGTACTATCAATGATGTGTAGACTCGACCTTAACTAAGTAAATATTCGGTTACGCTA
TP21913_Hit	D	1	chr5	.	CAGCATTGTGGTCAAAATTGATTAGAATGTCTGATTGAGTTGCATCACCAAATAATAGGTAACC
TP21913_Query	D	1	chr5	.	CAGCATTGTGGTCAAAATTGATTAGAATGTCTGATTGAGTTGAATCACCAAATAATAGGTAACC
TP22000_Hit	D	1	chr5	.	CAGCATTTACATGGAAGAGGTTCACGTTAAGGTTTGGTTTGGTTAATCTCTAAAATCTAAGTTT
TP22000_Query	D	1	chr5	.	CAGCATTTACATGGAAGAGGTTCACGTTAAGGTTTGGTTTCGTTAATCTCTAAAATCTAAGTTT
TP22019_Hit	D+G	1	chr5	.	CAGCATTTAGCTCGGCAAGAGTGAACTCTTCTGACCTATCTGGATGTTTTGTTGACGATGTTCC
TP22019_Query	D+G	1	chr5	.	CAGCATTTAGCTCAGCAAGAGTGAACTCTTCTGACCTATCTGGATGTTTTGTTGACGATGTTCC
TP22020_Hit	D	1	chr5	.	CAGCATTTAGCTGTGCAATAGAATTTAACTAAAATAAGTTGACTTCCTGCTTCATTTAGTTAAA
TP22020_Query	D	1	chr5	.	CAGCATTTAGCTGTGCAATAGAATTTAACTAAAATAAGTCGACTTCCTGCTTCATTTAGTTAAA
TP22031_Hit	D	1	chr5	.	CAGCATTTATAGCAAGAAGAAATAGATAAATAAATAAAATTATAGCATACACAAACTTAAGAGT
TP22031_Query	D	1	chr5	.	CAGCATTTATAGCAAGAAGAAATAGACAAATAAATAAAATTATAGCATACACAAACTTAAGAGT
TP2206_Hit	D	1	chr5	.	CAGCAAATCCATTTATATGCTTATTGTAAGAGTACATCACTGTTTCCTTTGCCTTCTCATGGCT
TP2206_Query	D	1	chr5	.	CAGCAAATCCATTGATATGCTTATTGTAAGAGTACATCACTGTTTCCTTTGCCTTCTCATGGCT
TP22085_Hit	D+G	1	chr5	.	CTGCATTTCAAGCAAAATTGATGGCTGATGAAGCCTTGATTTCATCTGGCTATGAAAATACCAG
TP22085_Query	D+G	1	chr5	.	CAGCATTTCAAGCAAAATTGATGGCTGATGAAGCCTTGATTTCATCTGGCTATGAAAATACCAG
TP22244_Hit	D	1	chr5	.	CAGCATTTGATGCACGTTTAAAAGCAATCTGAAGTTCATCCTTGATTGTTACCTTATCCTTAAA
TP22244_Query	D	1	chr5	.	CAGCATTTGATGCACATTTAAAAGCAATCTGAAGTTCATCCTTGATTGTTACCTTATCCTTAAA
TP22364_Hit	D	1	chr5	.	CAGCATTTGTGAGGGCCAATGGACGGGTTTTCTGCAACAGACCACGTCTACACAATCCCTCACG
TP22364_Query	D	1	chr5	.	CAGCATTTGTGAGGGCCAATGGACGGGTTTTCTGCAACAGACCACGTCTACACAATCCCCCACG
TP2251_Hit	D	1	chr5	.	CAGCAAATCTCAGAAAATTTCTCTACCAGTATCCTATCAAGAGAAAGCTTCAAGTTTTTTTTTT
TP2251_Query	D	1	chr5	.	CAGCAAATCTCAGAAAATTTATCTACCAGTATCCTATCAAGAGAAAGCTTCAAGTTTTTTTTTT
TP22525_Hit	D	1	chr5	.	CAGCTTTTTTAAGATTAAACAATGGTCATAAATTAAGAGTTTATTTTATTATGCAAAACCTTTT
TP22525_Query	D	1	chr5	.	CAGCATTTTTAAGATTAAACAATGGTCATAAATTAAGAGTTTATTTTATTATGCAAAACCTTTT
TP22552_Hit	D+G	1	chr5	.	CAGCATTTTTCTCCCAGACGTTGGCAGTAAATGATGCCACAGTGAAATTTGAGATTTGGGACAC
TP22552_Query	D+G	1	chr5	.	CAGCATTTTTCTCCCAGACGTTGGCAGTAAATGACGCCACAGTGAAATTTGAGATTTGGGACAC
TP22753_Hit	D	1	chr5	.	CAGCCAAAAGGGAGTACTCAAACAAAAGGGCATGCTCTCGGATAGAAGGGAATATGTATGTGGT
TP22753_Query	D	1	chr5	.	CAGCCAAAAGGGAGTACTCAAACAAAAGGGCATGCTCCCGGATAGAAGGGAATATGTATGTGGT
TP22888_Hit	D+G	1	chr5	.	CAGCCAAACCAAAGAGTAACACGCCTAAAGGAAAAGAATCAACAAGTCCAAGCAGGGGTGCTGA
TP22888_Query	D+G	1	chr5	.	CAGCCAAACCAAAGAGTAACACGCCTAAAGGAAAAGAATCAACAAGTCCAAACAGGGGTGCTGA
TP23019_Hit	D	1	chr5	.	CAGCCAAAGGAACTGATTTTTGAAGAATATTGTAAGAATGATGAGAAGATAAGGAGCATGAAAA
TP23019_Query	D	1	chr5	.	CAGCCAAAGGAACTGATTTTTGAAGAATATTGTAAGAATGATGAGAAGATAAGGAGCAAGAAAA
TP23047_Hit	D+G	1	chr5	.	CAGCCAAAGTGAAAGTGCTTCATCTAATGCTAGGATCATATCTGAGAGTAGTGACTCGGAGAAT
TP23047_Query	D+G	1	chr5	.	CAGCCAAAGTGAAAGTGCTTCATCTAATGCTAGGATCATATCTGAGAGTAGTGACTCAGAGAAT
TP23068_Hit	D	1	chr5	.	CAGCCAAATAACAACACCAAGACTCCTATCAAAATATAACACCAACACTAGTACCAATTACACA
TP23068_Query	D	1	chr5	.	CAGCCAAATAACAACACCAAGACCCCTATCAAAATATAACACCAACACTAGTACCAATTACACA
TP23219_Hit	D	1	chr5	.	CAGCCAACAAAAACTCACCTTCACAACACACACAGGTTCCTACCCTATTACCTCAATTGACTAT
TP23219_Query	D	1	chr5	.	CAGCCAACAAAAACTCACCTTCACAACACACACAGGCTCCTACCCTATTACCTCAATTGACTAT
TP23228_Hit	D+G	1	chr5	.	CAGCCAACAAAGGTCCTAAATTTGCGCCTCTTGCCAAGCCTCTAGAGTCCATGGCATTCATTCC
TP23228_Query	D+G	1	chr5	.	CAGCCAACAAAGGTCCTAAATTTGCGCCTCTTGCCAAGCCTCTAGAGTCAATGGCATTCATTCC
TP23284_Hit	D	1	chr5	.	CAGCCAACACAGTATCAGAGACACCTCTCCAGCATTCATTCAACACTTCTTCAATATGGTAATA
TP23284_Query	D	1	chr5	.	CAGCCAACACAGTATCAGAGACACCTCTCCAGCATTCATTCAACACTTCATCAATATGGTAATA

Name	Filter	Nb hit (Mt4.0)	Mt Chr (Mt4.0)	Ms Chr (Li et al., 2014)	Sequence
TP23593_Hit	D+G	1	chr5	.	CAGCCAAGACTCGATCCAACTTTGCTCGGAAATGATCATAATCAGCAATACCAAGCCATGTTGG
TP23593_Query	D+G	1	chr5	.	CAGCCAAGACTCGATCCAACTTTGCTAGGAAATGATCATAATCAGCAATACCAAGCCATGTTGG
TP23655_Hit	D	1	chr5	.	CAGCCAAGCCACTGAAACTAGTGGTCATAAAACGAAGTGGTGTAGAAATCACAGCACTAGCTGA
TP23655_Query	D	1	chr5	.	CAGCCAAGCCACTGAAACTAGTGGTCATAAAACGAAGTGGTGTAGAAATCACAGCACTAGCAGA
TP23680_Hit	D+G	1	chr5	.	CAGCCAAGGAAGCTGGTGGCATTACTCAGCATCTAGGTGCCTTTGTGGTGGGCATGTCATCAGG
TP23680_Query	D+G	1	chr5	.	CAGCCAAGGAAGCTGGTGGCATTACTCAGCATCTAGGTGCCTTTGTGGTGGCCATGTCATCAGG
TP24096_Hit	D	1	chr5	.	CAGCCAATTCAGAAAATAACTCTCTACACTAGCGTAACCGAATATTTACTTAGTTAAGGTCGAG
TP24096_Query	D	1	chr5	.	CAGCCAATTCAGAAAATAACTCTCTACACTAGCGTAACCCAATATTTACTTAGTTAAGGTCGAG
TP24258_Hit	D+G	1	chr5	.	CAGCCACAACAGATCCGTCATTCAAGCATGCCTTGTAAACTATTCCAAAACCACCTCTTCCTAG
TP24258_Query	D+G	1	chr5	.	CAGCCACAACAGATCCGTCATTCAAGCATGCCTTGTAAACTATTCCAAAACCACCTCTTCCTAA
TP24262_Hit	D	1	chr5	.	CAGCCACAACATGGGACTGGGTAATTCAAGGTCTCTATGTTCACATTTGTTTGCCATACAGATT
TP24262_Query	D	1	chr5	.	CAGCCACAACATGGGACTGGGTAATTCAAGGTCTCGATGTTCACATTTGTTTGCCATACAGATT
TP24377_Hit	D+G	1	chr5	.	CAGCTACACAAATCAACGAACGTGCAATGTTTACTTTAGATCAGCAATTTTGCAGATAATATCA
TP24377_Query	D+G	1	chr5	.	CAGCCACACAAATCAACGAACGTGCAATGTTTACTTTAGATCAGCAATTTTGCAGATAATATCA
TP24574_Hit	D+G	1	chr5	.	CAGCCATATCACTCAAACATCAGTAAGTACAAAACCAAGCCGAAATAGTAGTAAATGACAGAAC
TP24574_Query	D+G	1	chr5	.	CAGCCACATCACTCAAACATCAGTAAGTACAAAACCAAGCCGAAATAGTAGTAAATGACAGAAC
TP24642_Hit	D	1	chr5	.	CAGCCACATTGAATTCTGTTTTTGTTCCAAATTGTTTGATTCTTGTCAGCTACCAAAGAACCGT
TP24642_Query	D	1	chr5	.	CAGCCACATTGAATTCTGTTTTTGTCCCAAATTGTTTGATTCTTGTCAGCTACCAAAGAACCGT
TP25085_Hit	D+G	1	chr5	.	CAGCCATGGCATCAGCATAGCTCTTTCTTTGATGTCTTACATCTGATGGATCAACATGGAAAAA
TP25085_Query	D+G	1	chr5	.	CAGCCACGGCATCAGCATAGCTCTTTCTTTGATGTCTTACATCTGATGGATCAACATGGAAAAA
TP25116_Hit	D	1	chr5	.	CAGCCACGTCAGGGTGGCAGACTCTTGCAAGTCGCCGGTACGCTGAAAAAAAAAAAAAAAAAAA
TP25116_Query	D	1	chr5	.	CAGCCACGTCAGGGTGGCAGACTCTTACAAGTCGCCGGTACGCTGAAAAAAAAAAAAAAAAAAA
TP25123_Hit	D	1	chr5	.	CAGCCACGTGAACAAAAAATTCATGTACATTTGTCATGACTTTGGGAAGAGATTGCAATAACGA
TP25123_Query	D	1	chr5	.	CAGCCACGTGAACAAAAAAGTCATGTACATTTGTCATGACTTTGGGAAGAGATTGCAATAACGA
TP25279_Hit	D	1	chr5	.	CAGCCACTCTCGTCGTATATATGTGTTTCTTAGTCGGTTTATAGTGTAAATTAATAGTGCTAAC
TP25279_Query	D	1	chr5	.	CAGCCACTCTCGTCGTATATATGTGTTTCTTAGTCAGTTTATAGTGTAAATTAATAGTGCTAAC
TP25433_Hit	D	1	chr5	.	CAGCCACTTGGACAAGAACACGCCCAAAATGCTTTCCCTTGGAATCTTTAACTTCAATGAGCAA
TP25433_Query	D	1	chr5	.	CAGCCACTTGGACAAGAACACGCCCAAAATGCTTTCCCTTGGAATCTTGAACTTCAATGAGCAA
TP25508_Hit	D+G	1	chr5	.	CAGCCAGAAATGATTAGAAATAGTTAATAGCAGGGCATATTGGTAGTCATAGATCCGTTCAAGT
TP25508_Query	D+G	1	chr5	.	CAGCCAGAAATGATTAGAAATAGTTAATAGCAGGGCATATTAGTAGTCATAGATCCGTTCAAGT
TP25685_Hit	D	1	chr5	.	CAGCCAGATGTTATTTTGATATGATGCCTGGTAAAAATATTGTTACTTGGAATGAAATGATACA
TP25685_Query	D	1	chr5	.	CAGCCAGATGTTATTTTGATATGATGCCTGGTAAAAATATTGTCACTTGGAATGAAATGATACA
TP25751_Hit	D+G	1	chr5	.	CAGCCAGCACGGCCGCGGGAAGGCCCGTGCAAGGTGCTAATGTCTTTGGTAATGCTAATGGTAC
TP25751_Query	D+G	1	chr5	.	CAGCCAGCACGACCGCGGGAAGGCCCGTGCAAGGTGCTAATGTCTTTGGTAATGCTAATGGTAC
TP25896_Hit	D	1	chr5	.	CAGCCTGGAAAGCTCATACCATTTACTAATTGTCGAATGGGTACTATTGGAAAATGGTTACACT
TP25896_Query	D	1	chr5	.	CAGCCAGGAAAGCTCATACCATTTACTAATTGTCGAATGGGTACTATTGGAAAATGGTTACACT
TP25933_Hit	D	1	chr5	.	CAGCCAGGAGGTGGACGCTTAAAAACCTTCTTTTGCTCCAAGGTTGGAACCATTGAATGCTGAA
TP25933_Query	D	1	chr5	.	CAGCCAGGAGGTGGACGCTTAAAAACCTTCTTTTGCTCCAAGGTTGGAACCATTGAATGCAGAA
TP26079_Hit	D+G	1	chr5	.	CAGCCAGTCCATCGGCTTTGATAACAATTGGTGCTCCTTCTTCTTGAATATATTGCCTTGCTGA
TP26079_Query	D+G	1	chr5	.	CAGCCAGTCCATCGGCTTTGATAACAATTGGTGCTCCTTCTTCTTGAATATATTGCCGTGCTGA
TP2611_Hit	D	1	chr5	.	CAGCAAATTTTGCTGGTTCAGCAAAATCCAAGAAGGTAATTAGAAACGATGTCATGCAACTGCT
TP2611_Query	D	1	chr5	.	CAGCAAATTTTGCTGGTTCAGCAAAATCCAAGAAGGTAATTAGAAACCATGTCATGCAACTGCT
TP26316_Hit	D	1	chr5	.	CAGCCATGCACATGCAGTTCCCTATCAAGCACAGAACCCCAAGATGAAAATGGTCAAACCCAAG
TP26316_Query	D	1	chr5	.	CAGCCATACACATGCAGTTCCCTATCAAGCACAGAACCCCAAGATGAAAATGGTCAAACCCAAG
TP26369_Hit	D	1	chr5	.	CAGCCATTGAAATTCTTCCAATATCACAGAACATGTTTACTGCATTATCCAAGCAGGATATAGC
TP26369_Query	D	1	chr5	.	CAGCCATAGAAATTCTTCCAATATCACAGAACATGTTTACTGCATTATCCAAGCAGGATATAGC
TP26408_Hit	D+G	1	chr5	.	CAGCCATAGGGGCCATGGGCATCCCAAGTGGTCTTCTCCAAGACCAAGACAGTAGCTCATCACG
TP26408_Query	D+G	1	chr5	.	CAGCCATAGGGGCCATGGGCATCCCAAGTGGTCTTCTCCAAGACCAAGACAGGAGCTCATCACG
TP26514_Hit	D+G	1	chr5	.	CAGCCGTCAAAGTTAAAACCGTTCATTAATTGAAACTTAGCTTATACTGCAACAAATCCATTAA
TP26514_Query	D+G	1	chr5	.	CAGCCATCAAAGTTAAAACCGTTCATTAATTGAAACTTAGCTTATACTGCAACAAATCCATTAA
TP26766_Hit	D+G	1	chr5	.	CAGCCATCTTTGTGCGGTTGTCTTTTTCTGTACAGCATGGGCGAATGTCTGTGGTCGTCTTGTG
TP26766_Query	D+G	1	chr5	.	CAGCCATCTTTGTGCGGTTGTCTTTTTCTGTACAGCATGGGCAAATGTCTGTGGTCGTCTTGTG
TP26822_Hit	D	1	chr5	.	CAGCCATGAGTGCTCCTTTTTGTGGATATTGACCTTCTAATTTTGAATCCATGATCCGAAATAG
TP26822_Query	D	1	chr5	.	CAGCCATGAATGCTCCTTTTTGTGGATATTGACCTTCTAATTTTGAATCCATGATCCGAAATAG

Name	Filter	Nb hit (Mt4.0)	Mt Chr (Mt4.0)	Ms Chr (Li et al., 2014)	Sequence
TP26836_Hit	D	1	chr5	.	CAGCCATGACCAAGATGCTATCTACGTTCGCAGTGTGAACGAGATTGAAGTCAAGCCAAAACCC
TP26836_Query	D	1	chr5	.	CAGCCATGACCAAGATGCCATCTACGTTCGCAGTGTGAACGAGATTGAAGTCAAGCCAAAACCC
TP26883_Hit	D+G	1	chr5	.	CAGCCATGATTGATGTTGCCAAAGAGTTCTCCATCCCTACACTCGTCTTCTTCACTTCCAGTGT
TP26883_Query	D+G	1	chr5	.	CAGCCATGATTGATGTTGCCAAAGAGTTCTCCGTCCCTACACTCGTCTTCTTCACTTCCAGTGT
TP26985_Hit	D	1	chr5	.	CAGCCATGGAAAGTGAACATGGAATTTTCAGCAAGTGAAGACTGTTTCTTCCAAAGTTTCTGTA
TP26985_Query	D	1	chr5	.	CAGCCATGGAAAGTGAACATGGAATTTTCAGCAAGTGAAGACTGTTTCTTCCAAAGTTTCCGTA
TP26989_Hit	D	1	chr5	.	CAGCCATGGAAGTAGTTCAGATTTGAATCTTTCTTATTTCACTTTTCACTGATTTTCAGGTACA
TP26989_Query	D	1	chr5	.	CAGCCATGGAAGTAGTTCAGATTTGAATCTTTCTTATTACACTTTTCACTGATTTTCAGGTACA
TP27132_Hit	D	1	chr5	.	CAGCCATGTATTTAAAAGGAAAATAGTCTAAAACAAAAAATGAGGCTTCATGATGTGGCCTGTA
TP27132_Query	D	1	chr5	.	CAGCCATGTATTTAAAAGGAAAATAGTCTAAAACAAAAAAGGAGGCTTCATGATGTGGCCTGTA
TP27331_Hit	D	1	chr5	.	CAGCCATTCCAACCTGCAAAAGGAACTTTGATATGATCATCCTTGCCACGCCAGCTTCTTTTCC
TP27331_Query	D	1	chr5	.	CAGCCATTCCAACCTGCAAAAGGAACTTTGATATGATCATCCGTGCCACGCCAGCTTCTTTTCC
TP2778_Hit	D	1	chr5	.	CAGCAACAAAGGAGTAAAAATATTCTTCTTCCATTTTTTAGAATAAGATAAGAAAATTCATCAA
TP2778_Query	D	1	chr5	.	CAGCAACAAAGGAGTAAAAATATTCTTCTCCCATTTTTTAGAATAAGATAAGAAAATTCATCAA
TP27887_Hit	D	1	chr5	.	CAGCCCAATTCTCGCATTAAATCCGGTGACAACTTTTGTTTGACTTCAGTTTCATACTTGAGCT
TP27887_Query	D	1	chr5	.	CAGCCCAATTCTCGCATTAAATCCAGTGACAACTTTTGTTTGACTTCAGTTTCATACTTGAGCT
TP28007_Hit	D+G	1	chr5	.	CAGCCCACTGAATCTCACTTAACCTGAAATGTAACAGGAACACAATGCTAAACAGCCATGTTCA
TP28007_Query	D+G	1	chr5	.	CAGCCCACTGAATCTCACTCAACCTGAAATGTAACAGGAACACAATGCTAAACAGCCATGTTCA
TP28100_Hit	D	1	chr5	.	CAGCCCAGGCTGATTTACAAAGAAAAAATATGTATGGTAAGCAATATCAACAGTTAAAGAAAGA
TP28100_Query	D	1	chr5	.	CAGCCCAGGCTGATTTACAAAGAAAAAAAATGTATGGTAAGCAATATCAACAGTTAAAGAAAGA
TP28330_Hit	D	1	chr5	.	CAGCCCATTTAACATATGCTTTCCCTGGTTCTTTGAAATTCCACATGATATCTCCATATTCATT
TP28330_Query	D	1	chr5	.	CAGCCCATTTAACATATGCTTTCCCTGGTTCCTTGAAATTCCACATGATATCTCCATATTCATT
TP28463_Hit	D	1	chr5	.	CAGCCCCACCTTTGGTCATTTCTTTGTTGGGCGGAGGGATATTCCCGGACCAAGTTCTTTCGAC
TP28463_Query	D	1	chr5	.	CAGCCCCACCTTTGGTCATTTCTTTGTTGGGCGGAGGGATATTCCCCGACCAAGTTCTTTCGAC
TP288_Hit	D	1	chr5	.	CAGCAAAAATTCTCTTAAAAATGTGTAAAATATAGTGATAAAATGACTACATATGATCGTGTGT
TP288_Query	D	1	chr5	.	CAGCAAAAATTCTCTGAAAAATGTGTAAAATATAGTGATAAAATGACTACATATGATCGTGTGT
TP2881_Hit	D	1	chr5	.	CAGCAACAATAAGTGAGGAGAACATTCCAACTGATCCATCACCACTGCCTAGTCTCATATTCTC
TP2881_Query	D	1	chr5	.	CAGCAACAACAAGTGAGGAGAACATTCCAACTGATCCATCACCACTGCCTAGTCTCATATTCTC
TP28920_Hit	D+G	1	chr5	.	CAGCCCGAAAACAAAAACAAAACTCAAACCCGGAATGACATAAGTTATGAACCCTAAACCCTGC
TP28920_Query	D+G	1	chr5	.	CAGCCCGAAAACAAAAACAAAACTCAAACCCGGAATGACATAAGTTATGAACCCTAAACCCTAC
TP29442_Hit	D	1	chr5	.	CAGCCCTCAAAGGGTAATTTCTCATACTGAAAACTGCATATATTCTCTTTCTTTAACTGTTGAT
TP29442_Query	D	1	chr5	.	CAGCCCTCAAAGGGTAATTTATCATACTGAAAACTGCATATATTCTCTTTCTTTAACTGTTGAT
TP29834_Hit	D+G	1	chr5	.	CAGCCCTTTTCTTTATTTTCATCTTCAGTCTCTTAGCAATCTCTTCCAGCCTTGAAATAATGGT
TP29834_Query	D+G	1	chr5	.	CAGCCCTTTTCTTTATATTCATCTTCAGTCTCTTAGCAATCTCTTCCAGCCTTGAAATAATGGT
TP29948_Hit	D+G	1	chr5	.	CAGCCGAAGCATTCGATTGACCAATTGCTAAGATTTGTTCGTTCGGGACAGTTATAACAACTTG
TP29948_Query	D+G	1	chr5	.	CAGCCGAAGCATTCGATTGACCAATTGCTAAGATTTGTTCATTCGGGACAGTTATAACAACTTG
TP30172_Hit	D	1	chr5	.	CAGCTGAGTCCATTTCTTCACTGCAGGTGAATTTTATGATCACGAGCTTTTCGATACATGATAT
TP30172_Query	D	1	chr5	.	CAGCCGAGTCCATTTCTTCACTGCAGGTGAATTTTATGATCACGAGCTTTTCGATACATGATAT
TP30577_Hit	D	1	chr5	.	CAGCCGCCGCGGCAACGGCAAAAGCCACAGTCAGAAACCTGTTAAGCATCAAAAGCTGAAAAAA
TP30577_Query	D	1	chr5	.	CAGCCGCCGCGGCAACGGCAAAAGCCACAGTCAGAAACCTGTTAAGCATCAAAAGCAGAAAAAA
TP30659_Hit	D	1	chr5	.	CAGCCGCGCCACCTGCTATAGCGGAGCGTAGCGGAAAAAAATCAGCCCACTGCTCCAAGCCGCGA
TP30659_Query	D	1	chr5	.	CAGCCGCGCCACCCGCTATAGCGGAGCGTAGCGGAAAAAAATCAGCCCACTGCTCCAAGCCGCGA
TP3069_Hit	D+G	1	chr5	.	CAGCAACAAGCTTTTGACAAAAGAAAAAACAACATCCCAGATTTTGAGGCCAAAACACGTGAGG
TP3069_Query	D+G	1	chr5	.	CAGCAACAAGCTTTTAACAAAAGAAAAAACAACATCCCAGATTTTGAGGCCAAAACACGTGAGG
TP30701_Hit	D+G	1	chr5	.	CAGCCGCGGTAAACGCCCTCTTCCTGAAGACAACGATGATTCCACCAAAAATCCACCAGCTAAG
TP30701_Query	D+G	1	chr5	.	CAGCCGCGGTAAACGCCCTCTTCCTGAAGACAACGACGATTCCACCAAAAATCCACCAGCTAAG
TP30975_Hit	D+G	1	chr5	.	CAGCCGGCAATATTAGAAGCATTGGCATACCATTCTGCGCAGAAGGCAAACTTGCAGGTGCTGA
TP30975_Query	D+G	1	chr5	.	CAGCCGGCAATATTAAAAGCATTGGCATACCATTCTGCGCAGAAGGCAAACTTGCAGGTGCTGA
TP31097_Hit	D	1	chr5	.	CAGCCGGTACTTCTAAACTCTGATGTTGTGCATCAGAAGCATTACATGCATCGCTATAGCAATC
TP31097_Query	D	1	chr5	.	CAGCCGGTACATCTAAACTCTGATGTTGTGCATCAGAAGCATTACATGCATCGCTATAGCAATC
TP31281_Hit	D	1	chr5	.	CAGCCGTAGTTGGATGGATCCAATATGCATTTAGGGGTGTATTAGATTGAAATTTTAAGAAACT
TP31281_Query	D	1	chr5	.	CAGCCGTAGTTGGATGGATCCAATATGCATTTAGGAGTGTATTAGATTGAAATTTTAAGAAACT
TP31633_Hit	D	1	chr5	.	CAGCCTAAAACAATCAAGAAAGCAGGAATAGTTGTCATATCAAACACCATCTGCATTTTTCTCT
TP31633_Query	D	1	chr5	.	CAGCCTAAAACAATCAAAAAAGCAGGAATAGTTGTCATATCAAACACCATCTGCATTTTTCTCT

Name	Filter	Nb hit (Mt4.0)	Mt Chr (Mt4.0)	Ms Chr (Li et al., 2014)	Sequence
TP31837_Hit	D+G	1	chr5	.	CAGCCTACAAAGTTTGTTGGAAAGGTGGTTGCTCAGATGCAGATGTCTTAGCTGGTTTTGAAGC
TP31837_Query	D+G	1	chr5	.	CAGCCTACAAAGTGTGTTGGAAAGGTGGTTGCTCAGATGCAGATGTCTTAGCTGGTTTTGAAGC
TP31868_Hit	D	1	chr5	.	CAGCCTACATTCATATCAATTACCAATAAATGTTTTAAGTTAAGCAGAAAACCTTTTTGTATAG
TP31868_Query	D	1	chr5	.	CAGCCTACATTCATATCAATTACCAATAAATGTTTTAAGTTAAGCAGAAAACCTTTATGTATAG
TP31914_Hit	D	1	chr5	.	CAGCCTACTCTATCAAATCACAACAACAATAAAGTTAAAATTGTAATCACAAACTATATCCTTG
TP31914_Query	D	1	chr5	.	CAGCCTACTCTATCAAATCACAACAACAATAAAGTTAAAATTGTAATCACAAACTATATCCCTG
TP31924_Hit	D	1	chr5	.	CAGCCTACTGTGGCTGTATGACATTGTGGATCTTGTCATTAGGTATGCGCCAAATTATGATTCT
TP31924_Query	D	1	chr5	.	CAGCCTACTGTGGCTGTATGACATTGTGGATCTTGTCATTAGATATGCGCCAAATTATGATTCT
TP32147_Hit	D+G	1	chr5	.	CAGCCTATGCGGTACGCGGGAGTCGGTGGAGAAAGTTCTTGCATTTATTCAGAGTGATGATCGT
TP32147_Query	D+G	1	chr5	.	CAGCCTATGCGGCACGCGGGAGTCGGTGGAGAAAGTTCTTGCATTTATTCAGAGTGATGATCGT
TP32243_Hit	D	1	chr5	.	CAGCCTCAAAGCTAATGGGTTACAACTATGCTATGTTTGGTTTCACTCCTTATGGTCCTTATTG
TP32243_Query	D	1	chr5	.	CAGCCTCAAAGCTAATGGGTTACAACTATGCTATGTTTGGTTTCACTCCTTACGGTCCTTATTG
TP32295_Hit	D	1	chr5	.	CAGCCTCAATTGTCACTCAGTAATTGGTGGATTTGTTCCTATTCTGATTGCATTTCTCTTTGCC
TP32295_Query	D	1	chr5	.	CAGCCTCAACTGTCACTCAGTAATTGGTGGATTTGTTCCTATTCTGATTGCATTTCTCTTTGCC
TP32404_Hit	D	1	chr5	.	CAGCCTCACTATATATAGGAACAATGCTGATGTCTTTTTGTGTTGTATTCTTCACTACTTGGAG
TP32404_Query	D	1	chr5	.	CAGCCTCACTATATATAGGAACAACGCTGATGTCTTTTTGTGTTGTATTCTTCACTACTTGGAG
TP32443_Hit	D	1	chr5	.	CAGCCTCAGGAACTGCTGGAAGCCCCCACCTTTCACAATAGAATTCCTTCCAAGCATGATGCTC
TP32443_Query	D	1	chr5	.	CAGCCTCAGGAACTACTGGAAGCCCCCACCTTTCACAATAGAATTCCTTCCAAGCATGATGCTC
TP32502_Hit	D	1	chr5	.	CAGCCTCATCGCTTGTAATAAGACCTTTTGCCCAATAGACCCTATTTTTAGCATTGGCACCAGG
TP32502_Query	D	1	chr5	.	CAGCCTCATCACTTGTAATAAGACCTTTTGCCCAATAGACCCTATTTTTAGCATTGGCACCAGG
TP3283_Hit	D+G	1	chr5	.	CTGCAACACCAACAGCAGAAACTGGGGACACTTGGTTGAACCCACCACCAGCAGAAGAATTAGA
TP3283_Query	D+G	1	chr5	.	CAGCAACACCAACAGCAGAAACTGGGGACACTTGGTTGAACCCACCACCAGCAGAAGAATTAGA
TP33056_Hit	D	1	chr5	.	CAGCCTCTCTCCATAGCCTCATAGCTTTGATCTCCTCTCTAGCATTGTAAGCATCAGCGCGCCT
TP33056_Query	D	1	chr5	.	CAGCCTCTCTCCATAGCCTCATAGCTTTGATCTCCTCTCTAGCATTATAAGCATCAGCGCGCCT
TP33075_Hit	D	1	chr5	.	CAGCCTCTGACTTTAGCTTGTTCCAAAATGAATCTGTTAGTTTGGGGAATGCACATAAGCAAAT
TP33075_Query	D	1	chr5	.	CAGCCTCTGACTTTAGCTTGTTCCAAAATGAATCTGTTAGTTTGGGAAATGCACATAAGCAAAT
TP3311_Hit	D+G	1	chr5	.	CAGCAACACCCCATGTTGGTGTTTGATCAAGTGTTCTTGAACTGTTCCTACTATCACTTGCAGG
TP3311_Query	D+G	1	chr5	.	CAGCAACACCCCATGTCGGTGTTTGATCAAGTGTTCTTGAACTGTTCCTACTATCACTTGCAGG
TP33159_Hit	D+G	1	chr5	.	CAGCCTCTTCAGACTCGAATTCCATACCCTCGGAAGGTTCCAGATCTCCGGCCTCGGGAAAATA
TP33159_Query	D+G	1	chr5	.	CAGCCTCTTCAGACTCGAATTCCATACCCTCAGAAGGTTCCAGATCTCCGGCCTCGGGAAAATA
TP33246_Hit	D	1	chr5	.	CAGCCTGAAATGCAATATCCAGGACCAAACATCATTGTTGCTATCCAGTTAAACAGGTGGGCTA
TP33246_Query	D	1	chr5	.	CAGCCTGAAATGCAATATCCAGGACCAAACATCATTGTTACTATCCAGTTAAACAGGTGGGCTA
TP33488_Hit	D	1	chr5	.	CAGCCTGCAAATTTCTGAAAAGAAGATGCATACCAAATTGTATATTTGCGCCATACTTTCGCTC
TP33488_Query	D	1	chr5	.	CAGCCTGCAAATTTCTCAAAAGAAGATGCATACCAAATTGTATATTTGCGCCATACTTTCGCTC
TP33622_Hit	D	1	chr5	.	CAGCCTGCTGAATAGCTCCAAAAACCTGTGACAGTTAGATGTAGCATCAGCATGGATTTCTGGG
TP33622_Query	D	1	chr5	.	CAGCCTGCTGAATAGCTCCAAAAACCTGTGACAGTTAGATGTAGCATCAGCATAGATTTCTGGG
TP33687_Hit	D	1	chr5	.	CAGCCTGGATCCAGTGAAGTCCATATGTTGTAAGTTATTTTATTTTCAGTCTTTATAATATAAT
TP33687_Query	D	1	chr5	.	CAGCCTGGATCCAGTGAAGTCCATATGTTGTAAGTTATTTTATTTTCAGTCTTTAAAATATAAT
TP33859_Hit	D	1	chr5	.	CAGCCTGTGAAGGTGCTGAAAAGGTATTGGCCGATACTCGTAAAGCTTACTGCTTTGGAACTCG
TP33859_Query	D	1	chr5	.	CAGCCTGTGAAGGTGCTGAAAAAGTATTGGCCGATACTCGTAAAGCTTACTGCTTTGGAACTCG
TP33881_Hit	D	1	chr5	.	CAGCCTGTGCACAGTTAGGTGATCTTCCTGTTGGGAAGTCATCTCATGCTTATGTGTTCAGGAA
TP33881_Query	D	1	chr5	.	CAGCCTGTGCACAGTTAGGTGATCTTCATGTTGGGAAGTCATCTCATGCTTATGTGTTCAGGAA
TP33891_Hit	D	1	chr5	.	CTGCCTGTGGCCTTAGACCTGAAGTTCCCAAGGACTGCCAACCAAATTTAAAATACATTATGAC
TP33891_Query	D	1	chr5	.	CAGCCTGTGGCCTTAGACCTGAAGTTCCCAAGGACTGCCAACCAAATTTAAAATACATTATGAC
TP3399_Hit	D	1	chr5	.	CAGCAACAGACGCAACAGCAACAAATGCAACAACACCAGCAGATGCAATCGCAACAGCTGAAAA
TP3399_Query	D	1	chr5	.	CAGCAACAGACGCAACAGCAACAAATGCAACAACACCAGCAGATGCAATCGCAACAGCAGAAAA
TP34239_Hit	D	1	chr5	.	CAGCCTTATTCTCAACATGACTGTCAACAATCTTGTTTGCATGATTGTATGTGTTCTGTTGTTG
TP34239_Query	D	1	chr5	.	CAGCCTTATTCTCAACAGGACTGTCAACAATCTTGTTTGCATGATTGTATGTGTTCTGTTGTTG
TP34270_Hit	D	1	chr5	.	CAGCCTTCAACGCTCAATTGTTCAGAATATTCTTTAGCAACCTGAACAAACATAAGTAAGCTTA
TP34270_Query	D	1	chr5	.	CAGCCTTCAACGCTCAATTGTTCAGAATATTCTTTAGCAACCTGAACAAACATAAGCAAGCTTA
TP34330_Hit	D	1	chr5	.	CAGCCTTCATGAAACAACAGCCGGGTGCTCCCATACCTCAACACTCGTCTGACTAAAGCTAAGC
TP34330_Query	D	1	chr5	.	CAGCCTTCATGAAACAACAGCCGGGTGCTCCCATAACTCAACACTCGTCTGACTAAAGCTAAGC
TP34793_Hit	D+G	1	chr5	.	CAGCCTTGTTTCGACCATCTACATAATCAGATGCAGGTTCATGTGCTTTCATGTATGGGTGCTG
TP34793_Query	D+G	1	chr5	.	CAGCCTTGTTTCGACCATCTACATAATCAGATGCAGGTTCATGTGCTTTCATGTACGGGTGCTG

Name	Filter	Nb hit (Mt4.0)	Mt Chr (Mt4.0)	Ms Chr (Li et al., 2014)	Sequence
TP3485_Hit	D	1	chr5	.	CAGCAACAGCCGGATTCGACAAATCATTGACGTCCTCCTCAGCAATTACTTTCTCCACTATTAC
TP3485_Query	D	1	chr5	.	CAGCAACAGCCGGATTCGACAAATCATTGACATCCTCCTCAGCAATTACTTTCTCCACTATTAC
TP35111_Hit	D	1	chr5	.	CAGCCTTTTGTGCAAAGAAGAAAACTTCCTGGACCTTCAACAAGGAAACTTAATTAGAATTGTG
TP35111_Query	D	1	chr5	.	CAGCCTTTTGTGCAAAGAAGAAAACTTCCCGGACCTTCAACAAGGAAACTTAATTAGAATTGTG
TP35178_Hit	D	1	chr5	.	CAGCGAAAAGAACCAAGTGTCCCCATTTCCAGTGACTGTGTCCGTAATAGAATCCAAACCCTGA
TP35178_Query	D	1	chr5	.	CAGCGAAAAGAACCAAGTGTCCCCATTTCCAGTGACTGTGTCCGTAATAGAATCCAAACCCGGA
TP35221_Hit	D+G	1	chr5	.	CAGCGAAACATGTTTCCCCAAACACTTTCGTTTTGGCATTTCCAGTTGCATTTGTGAACAGGGC
TP35221_Query	D+G	1	chr5	.	CAGCGAAACATGTTTCCCCAAACACTTTCGTTTTGGCATTTCCAGTCGCATTTGTGAACAGGGC
TP3535_Hit	D	1	chr5	.	CAGCAACAGGTGTAGGAGTTTTCTTCTGTCTCCCGGCTGGATTCTTCAAAGTCACAGGCATCTT
TP3535_Query	D	1	chr5	.	CAGCAACAGGGGTAGGAGTTTTCTTCTGTCTCCCGGCTGGATTCTTCAAAGTCACAGGCATCTT
TP35564_Hit	D	1	chr5	.	CAGCGAATATTCAGATTGCAGTGAGGCATCCGCGGCAATGTCTTCAGCTCTCGACCATGTATAC
TP35564_Query	D	1	chr5	.	CAGCGAATATTCAGATTGCAGTGAGGCATCCGTGGCAATGTCTTCAGCTCTCGACCATGTATAC
TP35607_Hit	D	1	chr5	.	CAGCGAATGTGAAACATCTGATGAAGTTTATTTGGTGTCATTTGACTTATACAATGAGGTATTT
TP35607_Query	D	1	chr5	.	CAGCGAATGTGAAACATCTGATGAAGTTTATTTGGTGTCATTTGACTTATACAATGAGATATTT
TP35664_Hit	D	1	chr5	.	CAGCGACAAATGTAATCATTTTTAGGAATGGAAACTAGATAATGTGATAAACAGCCTTCTCATA
TP35664_Query	D	1	chr5	.	CAGCGACAAATGTAATCATTTTTAGGAATGAAAACTAGATAATGTGATAAACAGCCTTCTCATA
TP35881_Hit	D	1	chr5	.	CAGCGACGAGTCATCTTCATTGGCATATATGTGCAAAAATGGTTTGCAGTCACAGTTACTCAGT
TP35881_Query	D	1	chr5	.	CAGCGACGAGTCATCTTCATTGGCATATATGTGCAAAAATGGTTTGCAGTCACAATTACTCAGT
TP36023_Hit	D	1	chr5	.	CAGCGACTTCTAAAACCTTATATTGCGGCCGCAATTGTGATTGTGGATCTCAATTTAAATTTAA
TP36023_Query	D	1	chr5	.	CAGCGACTTCTAAAACCTTATATTGCGGACGCAATTGTGATTGTGGATCTCAATTTAAATTTAA
TP36091_Hit	D	1	chr5	.	CAGCGAGAATGGCAATCAAACAGAGAGAACCGACCATAGTTCCTGCTACTATTTCTAGAGCCAA
TP36091_Query	D	1	chr5	.	CAGCGAGAATGGCAATCAAACAGAGAGAACCGACCATAGTTCCTGCTACTATCTCTAGAGCCAA
TP36100_Hit	D	1	chr5	.	CAGCGAGACAATGTGCATGATATTATTTTATACTGGTAATATGTAACTCAAAGGCTTACAATAC
TP36100_Query	D	1	chr5	.	CAGCGAGACAATGTGCATGATATTATTTTATACTGGTAATATCTAACTCAAAGGCTTACAATAC
TP3617_Hit	D	1	chr5	.	CAGCAACATAAAATCTTGAGGGAAAAAAAAACTCACCCTAAAGATGCAGAAAGCTCTTCTGAAG
TP3617_Query	D	1	chr5	.	CAGCAACATAAAATCTCGAGGGAAAAAAAAACTCACCCTAAAGATGCAGAAAGCTCTTCTGAAG
TP3618_Hit	D	1	chr5	.	CAGCAACATAAAATCTTGAGGGAAAAAAAACTCACCCTAAAGATGCAGAAAGCTCTTCTGAAGG
TP3618_Query	D	1	chr5	.	CAGCAACATAAAATCTCGAGGGAAAAAAAACTCACCCTAAAGATGCAGAAAGCTCTTCTGAAGG
TP3626_Hit	D	1	chr5	.	CAGCAACATAACAGCCTCCTTGAAGAGGAGTATTCTCATAATTATGGAACAGCTAAGTTCTCAT
TP3626_Query	D	1	chr5	.	CAGCAACATAACAGCCTCCTTGAAGAGGAATATTCTCATAATTATGGAACAGCTAAGTTCTCAT
TP36297_Hit	D	1	chr5	.	CAGCGAGGATGAGTATAGGCGCTTAGATGACATAATTCATCCATGCAAAGCTGAAGCCACTATC
TP36297_Query	D	1	chr5	.	CAGCGAGGATGAGTATAGGCGCTCAGATGACATAATTCATCCATGCAAAGCTGAAGCCACTATC
TP36386_Hit	D+G	1	chr5	.	CAGCGAGTCTCTCCATGTTGGTGCCGTGAGTGGAGGACACTAAGTCCTTGAGCCGAATCAATAT
TP36386_Query	D+G	1	chr5	.	CAGCGAGTCTCTCCATGTTGGTGCCGTGAGTGGAGGACAATAAGTCCTTGAGCCGAATCAATAT
TP36548_Hit	D	1	chr5	.	CAGCGATCAGCCCAACCGTTGATTCTTCTGCTTTCCCCATCATTCCACCGCCGTTAACCACCAC
TP36548_Query	D	1	chr5	.	CAGCGATCAGCCCAACCGTTGATTCTTCTGCTTTCCCCATCATTCCACCGCCATTAACCACCAC
TP36558_Hit	D	1	chr5	.	CAGCGATCCAAAGCCGGAATCCATCCGCTTTGCCACCATACCCAACGTGGCTCCACCGGAGCGG
TP36558_Query	D	1	chr5	.	CAGCGATCCAAAGCCGGAATCCATCCGCTTCGCCACCATACCCAACGTGGCTCCACCGGAGCGG
TP36709_Hit	D	1	chr5	.	CAGCGATGCTGAAAGCTCCCTTCTGTGCATCCCCAGAGGAAAAATAAAAGTTGACAATCAGTTC
TP36709_Query	D	1	chr5	.	CAGCGATGCTGAAAGCTCCCTTCTGTACATCCCCAGAGGAAAAATAAAAGTTGACAATCAGTTC
TP36783_Hit	D	1	chr5	.	CAGCGATTAATTCTTCGATTACATAATTAGGGCTGTGATCCTAGTCACATACTAAAATTAGTAA
TP36783_Query	D	1	chr5	.	CAGCGATTAATTCTTCGATTACATAATTAGGGCTGTGATCCTAGTCACATACTAAAACTAGTAA
TP36933_Hit	D+G	1	chr5	.	CAGCGCAACAAGATAGGGGAATGCATCTTCTCTTGGCATATAGCACATAGGTCACCGGCTGAAA
TP36933_Query	D+G	1	chr5	.	CAGCGCAACAAGATAGGGGAATGCATCTTCTCTTGGCATATAGCACATAGGTCACCGGCAGAAA
TP36944_Hit	D	1	chr5	.	CAGCGCAACCACCAACAACCTGAACCCATGGTAGAGGTCACAAATGGTGCGGAGATCGGAAGAG
TP36944_Query	D	1	chr5	.	CAGCGCAACCACCAACAACCTGAACCCATGGTAGAGGTCACAAATGGTGCCGAGATCGGAAGAG
TP37087_Hit	D	1	chr5	.	CAGCGCAGAAAATAGAAGTCTAAGTATCTTCTTCCTAATTTTTTTTTTACAGTGTCTGATTACC
TP37087_Query	D	1	chr5	.	CAGCGCAGAAAACAGAAGTCTAAGTATCTTCTTCCTAATTTTTTTTTTACAGTGTCTGATTACC
TP37194_Hit	D+G	1	chr5	.	CAGCGCAGTTGCTACCTACCGATTGCACATAACCCCAATCCCTTAATTGTACTGAGCTAGTTTG
TP37194_Query	D+G	1	chr5	.	CAGCGCAGTTGCTACCTACCGACTGCACATAACCCCAATCCCTTAATTGTACTGAGCTAGTTTG
TP37241_Hit	D	1	chr5	.	CAGCGCATCTATGTTCTATTTTGTTCTTCATTGGTTAAAAGCTGGCCTGTGTGCCACATGGTTT
TP37241_Query	D	1	chr5	.	CAGCGCATCTATGTCCTATTTTGTTCTTCATTGGTTAAAAGCTGGCCTGTGTGCCACATGGTTT
TP37347_Hit	D	1	chr5	.	CAGCGCCATGGCAGGTATCCCTTCACAAATTTCTTGTGCTGATTGTCAGCTATTCTGCCATGGC
TP37347_Query	D	1	chr5	.	CAGCGCCACGGCAGGTATCCCTTCACAAATTTCTTGTGCTGATTGTCAGCTATTCTGCCATGGC

Name	Filter	Nb hit (Mt4.0)	Mt Chr (Mt4.0)	Ms Chr (Li et al., 2014)	Sequence
TP37385_Hit	D	1	chr5	.	CAGCGCCAGTTTTGGATTGGATATGAATGATTCTATGTTGTATGCAACTGAACAGCTAGAATCT
TP37385_Query	D	1	chr5	.	CAGCGCCAGTTTTGGATTGGATATGAATGATTCTATGTTGTACGCAACTGAACAGCTAGAATCT
TP37595_Hit	D	1	chr5	.	CAGCGCCGTTTACCTTTAATTTTTTTCTTCCCTTTTGGCTCTTTATGTTTTTAAATTAAAATTG
TP37595_Query	D	1	chr5	.	CAGCGCCGTTTACCTTTAATTTTTTTCTTCCCTTTTGGCTCTTTATGTTCTTAAATTAAAATTG
TP37658_Hit	D	1	chr5	.	CAGCGCCTGCGTTGAAACCTGCCGTTGAGCTTTATAGCCTTCATCATGATATCTCAACTCCTGA
TP37658_Query	D	1	chr5	.	CAGCGCCTGCGTTGAAACCTGCCGTTGAGCTTTACAGCCTTCATCATGATATCTCAACTCCTGA
TP37699_Hit	D	1	chr5	.	CAGCGCGAAGGGATTAGAAAGGAGTGTTCGTTCGCTGAAAAAAAAAAAAAAAAAAAAAAAAAAA
TP37699_Query	D	1	chr5	.	CAGCGCGAAGGGATTAGAAAGGAGTGTTCGTTCGCAGAAAAAAAAAAAAAAAAAAAAAAAAAAA
TP3790_Hit	D	1	chr5	.	CAGCAACATGTAAAGCACTATCTTTGTTTTTGTTAAATGTGAATAAAAGTTTTGCATCACGTTC
TP3790_Query	D	1	chr5	.	CAGCAACATGTAAAGCACTATCTTTGTTTTTGTTAAATGTGAACAAAAGTTTTGCATCACGTTC
TP37949_Hit	D+G	1	chr5	.	CAGCGCGTATTGAGTGGCTCAAGGCTCATAAAAACGTGTGAAATTAGTTGGTGGGTCAGAGTAG
TP37949_Query	D+G	1	chr5	.	CAGCGCGTATTGAGTGGCTCAAGGCTCAGAAAAACGTGTGAAATTAGTTGGTGGGTCAGAGTAG
TP38100_Hit	D	1	chr5	.	CAGCGCTATGGAGGGAGTTGAGGATTGGCCATGAAATTTGTTTGGGCATCAATGGAAGCAAGTC
TP38100_Query	D	1	chr5	.	CAGCGCTATGGAGGGAGTTGAGGATTGGCCATGAAATTCGTTTGGGCATCAATGGAAGCAAGTC
TP3829_Hit	D+G	1	chr5	.	CAGCAACATTATCTAAGGATGGAGCACAACACAAAACTACTTTGGGGAGAACCAAGCAAGACCA
TP3829_Query	D+G	1	chr5	.	CAGCAACATTATCCAAGGATGGAGCACAACACAAAACTACTTTGGGGAGAACCAAGCAAGACCA
TP38528_Hit	D	1	chr5	.	CAGCGGATGTTTAAAGCTGGCCCAGAAACTGTGTGATGAACTTGGTGCTTTGGCATCACCTGTT
TP38528_Query	D	1	chr5	.	CAGCGGACGTTTAAAGCTGGCCCAGAAACTGTGTGATGAACTTGGTGCTTTGGCATCACCTGTT
TP38598_Hit	D	1	chr5	.	CAGCGGAGCGATCACTACCTTCGGGAAACGACTCATCAACCAGATCTGGAAGAACAACAACTCC
TP38598_Query	D	1	chr5	.	CAGCGGAGCGATCACTACCTTCGGGAAACGACTCATCAACCAGATCTGGAACAACAACAACTCC
TP38753_Hit	D	1	chr5	.	CAGCGGATTCATTCTTTCTGGAAGATGTTTTACTCCTTTCACTCATTCTGTTCTCATCTTCAGA
TP38753_Query	D	1	chr5	.	CAGCGGATTCATTCTTTCTGGAAGATGTTTTACTCCTTTCACTCATTATGTTCTCATCTTCAGA
TP39166_Hit	D	1	chr5	.	CAGCGGCGGCATAGAGCTGATTGGGGGGAACCTCGCGAAGATAATCATTTGCCACATTCAACAG
TP39166_Query	D	1	chr5	.	CAGCGGCGGCATAGAGCTGATTGGGGGGAACCTCGCGAAGATAATCATTAGCCACATTCAACAG
TP3937_Hit	D	1	chr5	.	CAGCAATCACAACAATGGAAATGGCGCACTTTCTGTGGGTAGTGTTGGTAGTAGCATTCAGAAA
TP3937_Query	D	1	chr5	.	CAGCAACCACAACAATGGAAATGGCGCACTTTCTGTGGGTAGTGTTGGTAGTAGCATTCAGAAA
TP39855_Hit	D+G	1	chr5	.	CAGCGGTGCTGTTTATTTTCCCGAGGCCGGAGATCTGGAACCTTCTGAGGGTATGGAATTCGAG
TP39855_Query	D+G	1	chr5	.	CAGCGGTGCTGTTTATTTTCCCGAGGCCGGAGATCTGGAACCTTCCGAGGGTATGGAATTCGAG
TP40210_Hit	D	1	chr5	.	CAGCGTACACATCTGGTTTGATATTTCAATGAAGATTCCCTTCGTTGTTCAGGACAGGACATGT
TP40210_Query	D	1	chr5	.	CAGCGTACACATCTGGTTTGATATTTCAATGAAGATTCCCTTCGTTGTTCAGGACAAGACATGT
TP40359_Hit	D	1	chr5	.	CAGCGTATGGTTTGAAGCCCCCAAAGAGGGAGAAGTATGTGAGAAAAGGCAGTAGAAACCAGTC
TP40359_Query	D	1	chr5	.	CAGCGTATGGTTTGAAGCCCCCAAAGAGGGAGAAGTATGTGAGAAAAGGCAGTAGAAACCAGCC
TP40399_Hit	D	1	chr5	.	CAGCGTCAAAATGATGATAAATAAAAGAAATAAGAGGACATAGGATTTAGTAATATACTTGATA
TP40399_Query	D	1	chr5	.	CAGCGTCAAAATGATGATAAAGAAAAGAAATAAGAGGACATAGGATTTAGTAATATACTTGATA
TP40442_Hit	D	1	chr5	.	CAGCGTCACGACATAATAAAACAAAAAAAAGGAAGGAGTGACGCTTTGGTTACCGGCAAACGCT
TP40442_Query	D	1	chr5	.	CAGCGTCACGACATAATAAAACAAAAAAAAGGAAGGAGTGACGCTTTGGTTACCGGAAAACGCT
TP40538_Hit	D	1	chr5	.	CAGCGTCCGGAATGGCTGTGGACGATGAATGCAAACTGAAGTTCCAAGAGCTTAAAGCGAAGAG
TP40538_Query	D	1	chr5	.	CAGCGTCCGGAATGGCTGTGGACGACGAATGCAAACTGAAGTTCCAAGAGCTTAAAGCGAAGAG
TP40629_Hit	D	1	chr5	.	CAGCGTCGTTAAGAGTTGCTTCAGAAGAATTCTTTGTCTTATCTACAACAGTATCACTGTTCTG
TP40629_Query	D	1	chr5	.	CAGCGTCGTTAAGAGTTGCTTCAGAAGAATTCGTTGTCTTATCTACAACAGTATCACTGTTCTG
TP40778_Hit	D+G	1	chr5	.	CAGCGTGACCAGGAGTGTCGAGAAATGTGATTGATGCTCCTGATGACATGGCCACCACAAAGGC
TP40778_Query	D+G	1	chr5	.	CAGCGTGACCAGGAGTGTCGAGAAATGTGATTGATGCTCCTGATGACATGCCCACCACAAAGGC
TP4082_Hit	D	1	chr5	.	CAGCAACCCTCTGTTACTGGAAAGCATGAAGAATGAGCCCATACAAGCTCTAAGCAACCAAAGT
TP4082_Query	D	1	chr5	.	CAGCAACCCTCTGTTACCGGAAAGCATGAAGAATGAGCCCATACAAGCTCTAAGCAACCAAAGT
TP41024_Hit	D	1	chr5	.	CAGCGTGGCGAAGATTCAACAACGCGATCACACGGTAACAAAAATGGATGCTGAAGCTGAAAAA
TP41024_Query	D	1	chr5	.	CAGCGTGGCGAAGATTCAACAACGCGATCACACGGTAACAAAAATGGATGCTGAAGCAGAAAAA
TP41090_Hit	D	1	chr5	.	CAGCGTGTAGATCTGCGAGTTGAGCTTTAATTCTGTAAGCCTGAGAAAGTTGGTCTTGTAGATT
TP41090_Query	D	1	chr5	.	CAGCGTGTAGATCTGCGAGCTGAGCTTTAATTCTGTAAGCCTGAGAAAGTTGGTCTTGTAGATT
TP41105_Hit	D	1	chr5	.	CAGCGTGTCCCCAATAATACTCATGCTGTACCACCTGCCATTGCTGGAAAGACATCCCCCAAAC
TP41105_Query	D	1	chr5	.	CAGCGTGTCCCCAATAATACTCATGCTGTACCGCCTGCCATTGCTGGAAAGACATCCCCCAAAC
TP4131_Hit	D	1	chr5	.	CAGCAACCGGTTATCCTTGCGAGTATGCGGCGATGAATAATTTATCACCGAGAGGGCGAAGCTG
TP4131_Query	D	1	chr5	.	CAGCAACCGGTTATCCTTGCGAGTATGCGGCGATGAATAATTTATCACCGAGAGGGCGAAGCAG
TP41447_Hit	D+G	1	chr5	.	CAGCGTTGGACCTACCCTCACCAATGGGTGGAATAGAAGGGCTGGAAACTCTCGGTACAAATTG
TP41447_Query	D+G	1	chr5	.	CAGCGTTGGACCAACCCTCACCAATGGGTGGAATAGAAGGGCTGGAAACTCTCGGTACAAATTG

Name	Filter	Nb hit (Mt4.0)	Mt Chr (Mt4.0)	Ms Chr (Li et al., 2014)	Sequence
TP41823_Hit	D+G	1	chr5	.	CAGCTAAAATTGCTGAGCTTCATGTAGAGAAAGCGGAGATTCTTTCGGCTGAGCTGATACGTTT
TP41823_Query	D+G	1	chr5	.	CAGCTAAAATTGCTGAGCTTCATGTAGAGAAAGCGGAGATTCTTTCGGCTGAACTGATACGTTT
TP42028_Hit	D	1	chr5	.	CAGCTAAATCAGACATGTTGCATGCATGAAAATCCATATTAAACTAAAGAAAAGATTGAAAAGA
TP42028_Query	D	1	chr5	.	CAGCTAAATCAGAAATGTTGCATGCATGAAAATCCATATTAAACTAAAGAAAAGATTGAAAAGA
TP42043_Hit	D	1	chr5	.	CAGCTAAATGAATGGCATGATGCATGGAATGAAAAGGATAAGGATTTTGAGAATCAAGGAATTA
TP42043_Query	D	1	chr5	.	CAGCTAAATGAATGGCATGATGCATGGAATGAAAAGGATAAGGATTTTGAGAATCAAGGAATCA
TP42125_Hit	D	1	chr5	.	CAGCTAACAAATTCACCCTCCAGAAAAGATGGTATAGATGAAGCCACTGAAACAACCCTTAGAA
TP42125_Query	D	1	chr5	.	CAGCTAACAAATTCACCCTCCAGAAAAGATGGTATAGATGAAGCCACGGAAACAACCCTTAGAA
TP4226_Hit	D	1	chr5	.	CAGCAACCTGTCAACATTTCAAACTCACCAGAAATACCTCCTCCTGTTTACACCAAAGAAGAAT
TP4226_Query	D	1	chr5	.	CAGCAACCTGTCAACATTTCAAACTCACAAGAAATACCTCCTCCTGTTTACACCAAAGAAGAAT
TP42271_Hit	D+G	1	chr5	.	CAGCTAACTACTCTGACCCACCAACTAATTTCACACGTTTTTCTGAGCCTTGAGCCACTCAATA
TP42271_Query	D+G	1	chr5	.	CAGCTAACTACTCTGACCCACCAACTAATTTCACACGTTTTTATGAGCCTTGAGCCACTCAATA
TP4233_Hit	D+G	1	chr5	.	CAGCAACCTGTTAGTCAGGGTAGAAAATAAAATTCGTTTCATGCATAAAAATACTGATTTTGAA
TP4233_Query	D+G	1	chr5	.	CAGCAACCTGTTAGTCAGGGTAGAAAATAAAATTCGTTTCATGCATAAAAATACAGATTTTGAA
TP42333_Hit	D	1	chr5	.	CAGCTAAGAAAATTCCAACTAAAATTTTTCTTGGAAAATTTTCCATTGCAAGGGCTATACTCAT
TP42333_Query	D	1	chr5	.	CAGCTAAGAAAATTCCAACTAAAATTTTTCTAGGAAAATTTTCCATTGCAAGGGCTATACTCAT
TP42388_Hit	D+G	1	chr5	.	CAGCTAAGATCATTGGAGTGATTTGTGATGGTTACCAAAATTTCAAGTCCATTGAATCTGAACT
TP42388_Query	D+G	1	chr5	.	CAGCTAAGATCATTGGAGTGATTTGTGATGGTTACCAAAATTTCAACTCCATTGAATCTGAACT
TP42392_Hit	D	1	chr5	.	CTGCTAAGATGGCTGAATCTTATGCGCGTAGTGTGCTAGATGAGTGCAGAATAAAGGTTGAGGA
TP42392_Query	D	1	chr5	.	CAGCTAAGATGGCTGAATCTTATGCGCGTAGTGTGCTAGATGAGTGCAGAATAAAGGTTGAGGA
TP42442_Hit	D+G	1	chr5	.	CAGCTAAGGAACCAATAGCATTGATAAAGAATGAGCATCAGAATTATTTCGCTCACCTCAAAAA
TP42442_Query	D+G	1	chr5	.	CAGCTAAGGAACCAATAGCATTGACAAAGAATGAGCATCAGAATTATTTCGCTCACCTCAAAAA
TP42558_Hit	D	1	chr5	.	CAGCTAATAATAACGATGTAAGGTCATCTGGTCGTTCGTGTTTGTGCAAGGACTTCCTTAGCTG
TP42558_Query	D	1	chr5	.	CAGCTAATAATAACGATGTAAGGTCATCTGGTCGTTCGTGTTTGTGCAAGGACTTCCTTAGCAG
TP42577_Hit	D	1	chr5	.	CAGCTAATACTAATGGACATCAAGAAAAGGAGGTTGCTGAGGCAGAAACAGAAACAGAGGAAAA
TP42577_Query	D	1	chr5	.	CAGCTAATACTAATGGACATCAAGAAAAGGAAGTTGCTGAGGCAGAAACAGAAACAGAGGAAAA
TP42623_Hit	D	1	chr5	.	CAGCTAATATGCCATAGAGATCTGAAGCTTGAAAACACACTCTTAGATGGAAGCACCGCACCAC
TP42623_Query	D	1	chr5	.	CAGCTAATATGCCATAGAGATCTGAAGCTTGAAAACACACTCTTAGACGGAAGCACCGCACCAC
TP42739_Hit	D	1	chr5	.	CAGCTAATGGGTCACACGAAACCTATTGTAACATTATTGATACTGTCCTTAGAGGAAGCTTCCC
TP42739_Query	D	1	chr5	.	CAGCTAATGGGTCACACGAAACCTATTGAAACATTATTGATACTGTCCTTAGAGGAAGCTTCCC
TP42758_Hit	D	1	chr5	.	CAGCTAATTGAACTTTGGATGGTGGTCTTTTGCCCAAAAAAGAGGTAGAAAATAAAAAGGTTGG
TP42758_Query	D	1	chr5	.	CAGCTAATTAAACTTTGGATGGTGGTCTTTTGCCCAAAAAAGAGGTAGAAAATAAAAAGGTTGG
TP4284_Hit	D	1	chr5	.	CAGCAACGAAGACAGGGAGAATATCAACAGCGCTATGGAGGGAGTTGAGGATTGGCCATGAAAT
TP4284_Query	D	1	chr5	.	CAGCAACGAAGACAGGGAGAATATCAACAGCGCTATGGAGGAAGTTGAGGATTGGCCATGAAAT
TP42927_Hit	D	1	chr5	.	CAGCTACAACATAATTGGCAATACATGAGCAATTTGTTATCTCTTAAAAGGATAACTGGAGGTA
TP42927_Query	D	1	chr5	.	CAGCTACAACATAATTGGCAATACATGAGCAATTCGTTATCTCTTAAAAGGATAACTGGAGGTA
TP42959_Hit	D+G	1	chr5	.	CAGCTACAAGCAATGATTACCTGTAACTTAGTCTCCCTCGGTAAAAGTTTAAGGGCCTGAGCTC
TP42959_Query	D+G	1	chr5	.	CAGCTACAAGCAATAATTACCTGTAACTTAGTCTCCCTCGGTAAAAGTTTAAGGGCCTGAGCTC
TP4303_Hit	D+G	1	chr5	.	CAGCAACGACTGGAAGGGTATGTTAGATCCCTTGGATGACAACCTCCGGCGAGAGGTTGTTAGA
TP4303_Query	D+G	1	chr5	.	CAGCAACGACTGGAAGGGGATGTTAGATCCCTTGGATGACAACCTCCGGCGAGAGGTTGTTAGA
TP43196_Hit	D+G	1	chr5	.	CAGCTACATGCACATCTAGGCCATTTCGTGCAAATGCTTCCTGCAAACGTGCTGAAAAAAAAAA
TP43196_Query	D+G	1	chr5	.	CAGCTACATGCACATCTAGGCCATTTCGTGCAAATGCTTCCTGCAAACATGCTGAAAAAAAAAA
TP43220_Hit	D	1	chr5	.	CAGCTACATTGTGATCCTTGATATTGTGAAAAAATGCGACCGATGCAACTACAATTACACCCAC
TP43220_Query	D	1	chr5	.	CAGCTACATTGTGATCCTTGATATTGTGAAAAAATGCGACCGATGCAACCACAATTACACCCAC
TP43319_Hit	D+G	1	chr5	.	CAGCTACCGCCTTCTCAGATCAATTGGAATTCTAATTTTTAGGATGGGAAACATTTTCTTGGTG
TP43319_Query	D+G	1	chr5	.	CAGCTACCGCCTTCTCAGATCAATTGGAATTCTAATTTTTAGGATGGGAAACATTTTCGTGGTG
TP43460_Hit	D+G	1	chr5	.	CAGCTACGTCTAGTGCTTGGGAGGATGTTGGATCCATCTCGTTTCCGGAGTTATTAGCTAGAAA
TP43460_Query	D+G	1	chr5	.	CAGCTACGTCTAGTGCTTGGGAGGATGTTGGATCCACCTCGTTTCCGGAGTTATTAGCTAGAAA
TP43475_Hit	D	1	chr5	.	CAGCTACTAAGAGATTCTGATGTTGAAACAAGATGTGAGATTGAAGGTGAGACTCTTGAGAACA
TP43475_Query	D	1	chr5	.	CAGCTACTAAGAGATTCTGATGTTGAAACAAGATGTGAAATTGAAGGTGAGACTCTTGAGAACA
TP43537_Hit	D+G	1	chr5	.	CAGCTACTCAGACATTTTTATGTAGCATTTTAGTTGGCTACGGCAACTATACTATAGTTAGGTT
TP43537_Query	D+G	1	chr5	.	CAGCTACTCAGACATTTTTATGTAGCATTTTAGTTGGCTACGGCAACTATACTATAGCTAGGTT
TP43619_Hit	D+G	1	chr5	.	CAGCTACTGGAATACTCACCCACAACTCATCTGGGGATTACTAGGCTGGTGGGGAACCTTGGGC
TP43619_Query	D+G	1	chr5	.	CAGCTACTGGAATACTCACCCACAACTCATCTGGGGATTACTAGGCTGGTGGGGAACCTTAGGC

Name	Filter	Nb hit (Mt4.0)	Mt Chr (Mt4.0)	Ms Chr (Li et al., 2014)	Sequence
TP43626_Hit	D	1	chr5	.	CAGCTACTGTACATTCTCTGGAATTCTTTTTCCATCCACGGGAATAAGATCTGCAGGTCCCTTT
TP43626_Query	D	1	chr5	.	CAGCTACTGTACATTCTCTGGAATTCTTTTTCCATCCACGGGAATAAGATCTGCAGATCCCTTT
TP43978_Hit	D	1	chr5	.	CAGCTAGCAATAACCTTTCCATATGTAAATTTTATTAGTATACTGTTAAGTTCAATACAATCTT
TP43978_Query	D	1	chr5	.	CAGCTAGCAATAACCTGTCCATATGTAAATTTTATTAGTATACTGTTAAGTTCAATACAATCTT
TP44082_Hit	D+G	1	chr5	.	CAGCTAGTTATTGAGAACCAGAGCAGGACGCAGAAATAGAAAATCATTAAGATCCACCTATGTA
TP44082_Query	D+G	1	chr5	.	CAGCTAGCTATTGAGAACCAGAGCAGGACGCAGAAATAGAAAATCATTAAGATCCACCTATGTA
TP44116_Hit	D	1	chr5	.	CAGCTAGGAAAAGTTACATTGTCCTGATAACCTTTATTTATATAAATGCGCCCTCTTTGGAGGC
TP44116_Query	D	1	chr5	.	CAGCTAGGAAAAGTTACATTGTCATGATAACCTTTATTTATATAAATGCGCCCTCTTTGGAGGC
TP4416_Hit	D	1	chr5	.	CAGCAACGTCATCTTCTGGCCTATTTGCATGGTTATTTTTCTTTTCTGCTGGCCCAACTAGGAA
TP4416_Query	D	1	chr5	.	CAGCAACGTCATCTTCTGGCCTATTTGCATGGTTATTTCTCTTTTCTGCTGGCCCAACTAGGAA
TP44175_Hit	D	1	chr5	.	CAGCTAGGCTTCACATTGTTGATACTGGTTTACTTGATTATGTGCTGAAATCACTTAACAATGT
TP44175_Query	D	1	chr5	.	CAGCTAGGCTTCACATTGGTGATACTGGTTTACTTGATTATGTGCTGAAATCACTTAACAATGT
TP44277_Hit	D	1	chr5	.	CAGCTAGTACTAGTGGACTCCATGTAGGGACCTACAACACAAGACAAGATGGAAATTTTATCAT
TP44277_Query	D	1	chr5	.	CAGCTAGTACTAGCGGACTCCATGTAGGGACCTACAACACAAGACAAGATGGAAATTTTATCAT
TP44300_Hit	D	1	chr5	.	CAGCTAGTATATTAGGTTGGCATTTCGAACACGGGTGTGTAGCTCATTACTCCTTTTTACTAAT
TP44300_Query	D	1	chr5	.	CAGCTAGTATATTAGGTTGGCATTTCGAACACGGGTGTGTAGCTCATTACTCCTTTTTACTAAG
TP44358_Hit	D+G	1	chr5	.	CAGCTAGTGCATTCTCTTATCTAGAGAAACGAGCTTTTGGAATAGCCGATGACATGTGAGTTGT
TP44358_Query	D+G	1	chr5	.	CAGCTAGTGCATTCTCTTATCTAGAGAAAAGAGCTTTTGGAATAGCCGATGACATGTGAGTTGT
TP44385_Hit	D	1	chr5	.	CAGCTAGTGTGATTTTGCCTCACCTACCTCCTCTTATATATAATAACGAAAATTGAAATTGTCA
TP44385_Query	D	1	chr5	.	CAGCTAGTGTGATTTCGCCTCACCTACCTCCTCTTATATATAATAACGAAAATTGAAATTGTCA
TP44418_Hit	D	1	chr5	.	CAGCTAGTTCTAAGCCAAACTTTGACCATGAGGATTGGGATCAAATTTCATCTCAAAAAGTTTT
TP44418_Query	D	1	chr5	.	CAGCTAGTTCTAAGCCAAACTTTGACCATGAGGATTGGGATCAAATTTCATCTCAAAAAGTTCT
TP44442_Hit	D	1	chr5	.	CAGCTAGTTTATCCGCCATTTGCTACTTTTTACCAAAGAGAGTCTAAATCTTGGTTGCCTTTTA
TP44442_Query	D	1	chr5	.	CAGCTAGTTTATCCACCATTTGCTACTTTTTACCAAAGAGAGTCTAAATCTTGGTTGCCTTTTA
TP4448_Hit	D+G	1	chr5	.	CAGCAACGTTTCTGCTGTTAATGAAGCCCTGAACGAGATATATGTTGAGGAGGAAGATTATGAT
TP4448_Query	D+G	1	chr5	.	CAGCAACGTTTCTGCCGTTAATGAAGCCCTGAACGAGATATATGTTGAGGAGGAAGATTATGAT
TP44602_Hit	D	1	chr5	.	CAGCTATACTATATACATTGACATATATACCATTGCATGTTGAAACATATCTGGAGAAATCTAA
TP44602_Query	D	1	chr5	.	CAGCTATACTATATACATTGACATATATACCATTGCATGTTGAAACATATCAGGAGAAATCTAA
TP44630_Hit	D	1	chr5	.	CAGCTCTAGATGCGCTATAGCACTGTAGTGAAGCAGAATTTGAACAAACCATTGTTTTCCGTGA
TP44630_Query	D	1	chr5	.	CAGCTATAGATGCGCTATAGCACTGTAGTGAAGCAGAATTTGAACAAACCATTGTTTTCCGTGA
TP4495_Hit	D+G	1	chr5	.	CAGCAACTAATGATGGATGAGATATCCGGTCTTTCCTCTCCTTCCAACTGGAATCATTGCATGC
TP4495_Query	D+G	1	chr5	.	CAGCAACTAATGATGGATGAGATATCCGGTCTTTCCTCTCCTTCCAACTGGAATCATTCCATGC
TP45309_Hit	D+G	1	chr5	.	CAGCTATTAAACTTCAGTCTGCTTGCAGAGGCTATCAGGTAATACTCCCCTGTAATTTTACATT
TP45309_Query	D+G	1	chr5	.	CAGCTATTAAACTTCAGTCTGCTTGCAGAGGCTATAAGGTAATACTCCCCTGTAATTTTACATT
TP45355_Hit	D	1	chr5	.	CAGCTATTAGAGTATTATATGTAATCTGATCTGGATGCACACCCCTTTTTTCCATATCCTCAAA
TP45355_Query	D	1	chr5	.	CAGCTATTAGAGTATTATATGTAATCTGATCTGGATGCACACCCATTTTTTCCATATCCTCAAA
TP4538_Hit	D	1	chr5	.	CAGCAACTAGAAGTATAATGTTTTCGTACAATATAAAAATTAAAAAGGAATATAATTTTTAGAA
TP4538_Query	D	1	chr5	.	CAGCAACTAGAAGTAGAATGTTTTCGTACAATATAAAAATTAAAAAGGAATATAATTTTTAGAA
TP45514_Hit	D+G	1	chr5	.	CAGCTATTGTCGAAGGGTTTCTGCCATATTGCAACATACCCATCTTTTTTCAAAAACTTCCAGC
TP45514_Query	D+G	1	chr5	.	CAGCTATTGTCAAAGGGTTTCTGCCATATTGCAACATACCCATCTTTTTTCAAAAACTTCCAGC
TP45550_Hit	D	1	chr5	.	CAGCTATTTATACAAGTAATGGCCAGTTGGCACTCTTAACAAAATTTGCCTAGTCAACAATAAC
TP45550_Query	D	1	chr5	.	CAGCTATTTATACAAGTAATGGCCACTTGGCACTCTTAACAAAATTTGCCTAGTCAACAATAAC
TP46008_Hit	D	1	chr5	.	CAGCTCAAGTATCTTAACATTGAAGGCAACAATCTTGTTGGAGAAATCCCTTGTGAACTTGGAA
TP46008_Query	D	1	chr5	.	CAGCTCAAGTATCCTAACATTGAAGGCAACAATCTTGTTGGAGAAATCCCTTGTGAACTTGGAA
TP46014_Hit	D+G	1	chr5	.	CAGCTCAAGTGATTGTGGAAGCATTAAAAGAGGACAAAGCTATGAAACCAGGCTCTAGTGGAAT
TP46014_Query	D+G	1	chr5	.	CAGCTCAAGTGATTGTGGAAGCATTAAAAGAGGACAAAGCTATGAAACCAGGCTCTAGTGGAAC
TP46284_Hit	D	1	chr5	.	CAGCTCACTATCTACAATCACAACGGTGGTCCTTGCTATCGATGCCTCTTTCCTACTCCACCAC
TP46284_Query	D	1	chr5	.	CAGCTCACTATCTACAATCACAACGGTGGTCCTTGCTATCGATGCCTCTTTCCAACTCCACCAC
TP4644_Hit	D+G	1	chr5	.	CAGCAACTCCAAAAGCAAGTAAACTGATTGGTCTCTGAACAAACACAATTGTCAAGAATCAAAA
TP4644_Query	D+G	1	chr5	.	CAGCAACTCCAAAAGCAAGGAAACTGATTGGTCTCTGAACAAACACAATTGTCAAGAATCAAAA
TP46529_Hit	D	1	chr5	.	CAGCTCAGGTATAATTTTACCACAAAAATGTGTGCTGAAAGAAGTACCAGCTTTTCGTCAGCAC
TP46529_Query	D	1	chr5	.	CAGCTCAGGTATAATTTTACCACAAAAATGTGTGCTGAAAGAAGTACCAACTTTTCGTCAGCAC
TP46575_Hit	D+G	1	chr5	.	CAGCTCAGTGTCATTTACATGGTTTGGTACATCGCGACATGAAGCCAGAGGTGAGGTCAAGTTT
TP46575_Query	D+G	1	chr5	.	CAGCTCAGTGTCATTTACATGGTTTGGTACATCGCGACATGAAGCCAGAGGTGAGGTCAAGTTC

Name	Filter	Nb hit (Mt4.0)	Mt Chr (Mt4.0)	Ms Chr (Li et al., 2014)	Sequence
TP46623_Hit	D	1	chr5	.	CAGCTCATAATGTGCTTGTGAAGATTTACAATAATGTGGATAATCATGACAATAATGCCGATAT
TP46623_Query	D	1	chr5	.	CAGCTCATAATGTGCTTGTGAAGATTTACAATAATGTGAATAATCATGACAATAATGCCGATAT
TP4665_Hit	D+G	1	chr5	.	CAGCTACTCGAAGATTTTTGAAGGGATAGTAACTCAGCACCTGATCAGTATTGGACCTGAAAAT
TP4665_Query	D+G	1	chr5	.	CAGCAACTCGAAGATTTTTGAAGGGATAGTAACTCAGCACCTGATCAGTATTGGACCTGAAAAT
TP46876_Hit	D	1	chr5	.	CAGCTCATTCTGAATGGGCTTAGCAGGCATTCCATTCTCCTTGTCATCCAAATCACCAGCACTT
TP46876_Query	D	1	chr5	.	CAGCTCATTCTGAATGGGCTTAGCAGGCATTCCATTCTCCTTATCATCCAAATCACCAGCACTT
TP46975_Hit	D+G	1	chr5	.	CAGCTCCAAATGATCAAAGAATTGCACTTGTGTCAAAGGGGTTCTGCTCTAAGCCAACTTCATT
TP46975_Query	D+G	1	chr5	.	CAGCTCCAAATGATCAAAGAATTACACTTGTGTCAAAGGGGTTCTGCTCTAAGCCAACTTCATT
TP47033_Hit	D	1	chr5	.	CAGCTCCAATACAGTATTGTTTTCACTAGCCTCGGTTTCCAGCTTTGAATCTAGTAAGCCCTTC
TP47033_Query	D	1	chr5	.	CAGCTCCAATACAGTATTGTTTTCACTAGCCTCGATTTCCAGCTTTGAATCTAGTAAGCCCTTC
TP47073_Hit	D	1	chr5	.	CAGCTCCACAAACAACCTGCTGATTTATTGTTAGGACATCACCAGGATGTGACTTCAACAATGC
TP47073_Query	D	1	chr5	.	CAGCTCCACAAACAACCTGCTGATTTATTGTTAGGACATCACCAGGATGTGACCTCAACAATGC
TP47117_Hit	D	1	chr5	.	CAGCTCCACCTTACAGGGAATTGAAAACGCATAAGAAAATAGAAGAGGTAGAAAAGTTTAATTA
TP47117_Query	D	1	chr5	.	CAGCTCCACCTTACAGGGAATTGAAAACGCATAAGAAAATAGAAGAGGTAGAAAAGCTTAATTA
TP47206_Hit	D	1	chr5	.	CAGCTCCAGGCTATGAGCCTCTCCCAAAACCTTGTAGCCTTCCATACGTTGATCAGTATGATGG
TP47206_Query	D	1	chr5	.	CAGCTCCAGGCTATGAGCCTCTCCCAAAACCTTGTAGCCTTCCATACATTGATCAGTATGATGG
TP4721_Hit	D	1	chr5	.	CAGCAACTGAGTTGAATCTATTTGTTCAAACAGATTGTGAAGATAAAGAGTCAAACCAAGGAAA
TP4721_Query	D	1	chr5	.	CAGCAACTGAGTTGAATCTACTTGTTCAAACAGATTGTGAAGATAAAGAGTCAAACCAAGGAAA
TP47396_Hit	D	1	chr5	.	CAGCTCCCAGGTAGCCGTTTGAAGAGTGCTTTGAGTGCGAGGGATCTCGATCTTGAGATGGAAT
TP47396_Query	D	1	chr5	.	CAGCTCCCAGGTAGCCGTTTGAAGAGTGCTTTGAGTGCAAGGGATCTCGATCTTGAGATGGAAT
TP47738_Hit	D	1	chr5	.	CAGCTCCTCAATGCCCTTTTCCTGATGCTTGGATAGGAAATGCCCTAAAAACAGGTCTTTTTGA
TP47738_Query	D	1	chr5	.	CAGCTCCTCAATGCCCTTTCCCTGATGCTTGGATAGGAAATGCCCTAAAAACAGGTCTTTTTGA
TP47810_Hit	D+G	1	chr5	.	CAGCTCCTCTCCCCAAACTAGCTCAGTACAATTAAGGGATTGGGGTTATGTGCAGTCGGTAGGT
TP47810_Query	D+G	1	chr5	.	CAGCTCCTCTCCCCAAACTAGCTCAGTACAATTAAGGGATTGGGGTTATGTGCAATCGGTAGGT
TP47928_Hit	D	1	chr5	.	CAGCTCCTTCAAATGTTATTTATCTGTCAAGTATTTTGGGCCATGATGGTCCAAGCCCTGTTCA
TP47928_Query	D	1	chr5	.	CAGCTCCTTCAAATGTTATTTATCTGTCAAGTATTTTGGGCCATGATGGACCAAGCCCTGTTCA
TP47973_Hit	D	1	chr5	.	CAGCTCCTTGAGGTTGACATTCAATTTCTCAAAACAACCAGGAAACCCACAAACAACAGTTATC
TP47973_Query	D	1	chr5	.	CAGCTCCTTGAGGGTGACATTCAATTTCTCAAAACAACCAGGAAACCCACAAACAACAGTTATC
TP48117_Hit	D	1	chr5	.	CAGCTCGAGCCCATGACGTTGCGGCCCAAGCCATCAAAGGCTGAAAAAAAAAAAAAAAAAAAAA
TP48117_Query	D	1	chr5	.	CAGCTCGAGCCCATGACGTTGCGGCCCAAGCCATCAAAGGCAGAAAAAAAAAAAAAAAAAAAAA
TP48305_Hit	D+G	1	chr5	.	CAGCTCGCTTCAATCCATGATCTTCAATCCATTTCCTCACTTTATCAAGGCCATTTAACGGCTT
TP48305_Query	D+G	1	chr5	.	CAGCTCGCTTCAATCCACGATCTTCAATCCATTTCCTCACTTTATCAAGGCCATTTAACGGCTT
TP48705_Hit	D	1	chr5	.	CAGCTCTATACAAATTTCTCTCCCTCTCTCTACACAGAACAATCATCAGATTTACTTGAACGGA
TP48705_Query	D	1	chr5	.	CAGCTCTATACAAATCTCTCTCCCTCTCTCTACACAGAACAATCATCAGATTTACTTGAACGGA
TP4875_Hit	D	1	chr5	.	CAGCAACTTCGCCTGATCTCAGATATTGAAGATACAGAGGATCGATCAAGGGAAGCTGATGAGC
TP4875_Query	D	1	chr5	.	CAGCAACTTCGCCTGATCTCAGATATTGAAGATACAAAGGATCGATCAAGGGAAGCTGATGAGC
TP49018_Hit	D	1	chr5	.	CAGCTCTCTTATTTAGACACCATATCATCAACAAGGTCCCAATGTATCTGAGACAAGAAAGTTC
TP49018_Query	D	1	chr5	.	CAGCTCTCTTATTTAGACACCATATCATCAACAAGGTCCCAATATATCTGAGACAAGAAAGTTC
TP49298_Hit	D	1	chr5	.	CAGCTCTTAGCATCTTATTGTGTTGAAATGGCAGAGTTAGCAGGTGCTGATCATGACCGTGTCG
TP49298_Query	D	1	chr5	.	CAGCTCTTAGCATCTTATTGTGTTGAAATGGCAGAGTTAGCAGGTGCTGATCATGACCGTGTCA
TP49305_Hit	D+G	1	chr5	.	CAGCTCTTATAAATGTCGGAGAACCAACAAAAGATGGGATTCTTGTAACTACAAGTGCTCCTGT
TP49305_Query	D+G	1	chr5	.	CAGCTCTTATAAATGTCGGAGAACCAACAAAAGATGGGATTCTTGGAACTACAAGTGCTCCTGT
TP49403_Hit	D	1	chr5	.	CAGCTCTTCTTTTTCAGGATCATATAACTTGAAAAAAAGTAATATGTCATGCTTTCTCTTGGTG
TP49403_Query	D	1	chr5	.	CAGCTCTTCTTTTTCAGGATCATATAACTTGAAAAAAAGTAATATGTCATGCTCTCTCTTGGTG
TP49513_Hit	D+G	1	chr5	.	CAGCTCTTGTGGATTTCATACCATTCCTTGAGTGTAAGTGCATTTCTCTTGAGATGTATTTAAA
TP49513_Query	D+G	1	chr5	.	CAGCTCTTGTGGATTTCATACCATTCCTTGAGTGTAAGTGCATTCCTCTTGAGATGTATTTAAA
TP49596_Hit	D	1	chr5	.	CAGCTCTTTTAAACCTCTCAAAATATGCCAAGAGTAGAAGTGAAATGGTTGAGAATTGGGGCTT
TP49596_Query	D	1	chr5	.	CAGCTCTTTTAAACCTCTCAAAATATGCCAAGAGTAGAAGTGAAATGGTTGAGAATTGGGGATT
TP49641_Hit	D	1	chr5	.	CAGCTGAAAAAAAACGTAGTGCCAACATATCATCTACTGGTAAGCAGAATAATGCTTCTGCCGG
TP49641_Query	D	1	chr5	.	CAGCTGAAAAAAAACGTAGTGCCAACATATCATCTACTGGTAAGCAGAATAATGCTTCTGCAGG
TP49902_Hit	D	1	chr5	.	CAGCTGAACATGCATATAATTACCTACTTGATGCTTGACTGAGTAACTAACGAGTTATTTGTAC
TP49902_Query	D	1	chr5	.	CAGCTGAACATGCATATAATTACCTACTTGATGCATGACTGAGTAACTAACGAGTTATTTGTAC
TP50335_Hit	D	1	chr5	.	CAGCTGGCCAGAAAATTCACTTGTCATGTGGTTCTGTCATTAATCTTATATTACTTTGTTGCGA
TP50335_Query	D	1	chr5	.	CAGCTGACCAGAAAATTCACTTGTCATGTGGTTCTGTCATTAATCTTATATTACTTTGTTGCGA

Name	Filter	Nb hit (Mt4.0)	Mt Chr (Mt4.0)	Ms Chr (Li et al., 2014)	Sequence
TP504_Hit	D	1	chr5	.	CAGCAAAACTGAAATTTATGATAGTCAAATGCAGAGAAAAAGGAAGTACAAAGTAGGAAGTGAT
TP504_Query	D	1	chr5	.	CAGCAAAACTGAAATTTATGATAGTCAAATGCAGAGAAAAAGGAAGAACAAAGTAGGAAGTGAT
TP50481_Hit	D+G	1	chr5	.	CAGCTGAGCAACTGAAGCCGTTAAATGGCCTTGATAAAGTGAGGAAATGGATTGAAGATCGTGG
TP50481_Query	D+G	1	chr5	.	CAGCTGAGCAACTGAAGCCGTTAAATGGCCTTGATAAAGTGAGGAAATGGATTGAAGATCATGG
TP50529_Hit	D+G	1	chr5	.	CAGCTGAGGAAGGCACTCATGGAGTATTTCAAACCATCTTCTTTTGTTGGAGAGAACACTGGAA
TP50529_Query	D+G	1	chr5	.	CAGCTGAGGAAGGCACTCATGGAGTATTTCAAACCATCCTCTTTTGTTGGAGAGAACACTGGAA
TP50657_Hit	D	1	chr5	.	CAGCTGAGTTTTTACGAGAAGCAAAGCCTTTCATTGAAGATGGTGTTCACTCTCAAAATTTAAT
TP50657_Query	D	1	chr5	.	CAGCTGAGTTTTTAAGAGAAGCAAAGCCTTTCATTGAAGATGGTGTTCACTCTCAAAATTTAAT
TP50894_Hit	D+G	1	chr5	.	CAGCTGATGGAGCTGATAACTGGTCAAAATTCTTCCCTCCACAGATAGCACTTTCTATGAAATC
TP50894_Query	D+G	1	chr5	.	CAGCTGATGGAACTGATAACTGGTCAAAATTCTTCCCTCCACAGATAGCACTTTCTATGAAATC
TP51123_Hit	D	1	chr5	.	CAGCTGGAAATTTTGTTGTTTACAACTCTCCTTCGACTGTTAGTTTCTCGCCTAACGATAGTCC
TP51123_Query	D	1	chr5	.	CAGCTGGAAATTTTGTTGTTTACAACTCTCCTTCGACTGTTAGTTTCTCGCCTAACAATAGTCC
TP51259_Hit	D	1	chr5	.	CAGCTGGGCTCCAATCAGGATGGACAGATTTAAGTAGCCCAACCAGTCCAGCAACATGAGGACA
TP51259_Query	D	1	chr5	.	CAGCTGGACTCCAATCAGGATGGACAGATTTAAGTAGCCCAACCAGTCCAGCAACATGAGGACA
TP51299_Hit	D	1	chr5	.	CAGCTGGAGCTACAGCAAGTAGCCAACAGGCCAGAGTTATGGCTTCATCACATGTTGCCAGACA
TP51299_Query	D	1	chr5	.	CAGCTGGAGCTACAGCAAGTAGCCAACAGGCCAGAGTGATGGCTTCATCACATGTTGCCAGACA
TP51335_Hit	D	1	chr5	.	CAGCTGGAGTTCTTTTCCCTTCTACAGGCTTTCGGTTGCTGAAAAAAAAAAAAAAAAAAAAAAA
TP51335_Query	D	1	chr5	.	CAGCTGGAGTTCTTTTCCCTTCTACAGGCTTTCGGTTGCAGAAAAAAAAAAAAAAAAAAAAAAA
TP51336_Hit	D	1	chr5	.	CAGCTGGAGTTCTTTTCCCTTCTACAGGCTTTCGGTTGCCGCCTTGGATCGCTGGTGCGGAGAT
TP51336_Query	D	1	chr5	.	CAGCTGGAGTTCTTTTCCCTTCTACAGGCTTTCGGTTGCCGCCTTGGATCGCTGGTGCCGAGAT
TP5140_Hit	D	1	chr5	.	CAGCAAGAATAAGAAATTAATGTCCTCTAAGTAAAGTTGTTACTTGCAAGTATTAAATTGATAT
TP5140_Query	D	1	chr5	.	CAGCAAGAATAAGAAATTAATGTCCTCCAAGTAAAGTTGTTACTTGCAAGTATTAAATTGATAT
TP51613_Hit	D	1	chr5	.	CTGCTGGGCATTTAAATTTCATGGCAGTTGGTGGCTTCAGAGCTGTTGCAACAAGGGGGACAAC
TP51613_Query	D	1	chr5	.	CAGCTGGGCATTTAAATTTCATGGCAGTTGGTGGCTTCAGAGCTGTTGCAACAAGGGGGACAAC
TP52005_Hit	D+G	1	chr5	.	CAGCTGGTTTGTCCAAATTAGGCATCCAGAAAGGCGATGTAATTATGATTCTTCTACAAAACTC
TP52005_Query	D+G	1	chr5	.	CAGCTGGTTTGTCCAAATTAGGCATCCAGAAAGGCGATGTAATCATGATTCTTCTACAAAACTC
TP52102_Hit	D	1	chr5	.	CAGCTGTACAACAATGATGGAGGCCAAGCAACTCAATTCTCGGCTCCAACTTCTTCTGACCTTT
TP52102_Query	D	1	chr5	.	CAGCTGTACAACAATGATGGAGCCCAAGCAACTCAATTCTCGGCTCCAACTTCTTCTGACCTTT
TP52140_Hit	D	1	chr5	.	CAGCTGTACTAATAACTATGCTAGCTGGGATTCACGTTGGCTCCTTACCCTTCTATAGAGACAA
TP52140_Query	D	1	chr5	.	CAGCTGTACTAATAACTATGCTAGCTGGGATTCACGTTGGCTCCTTACCCTGCTATAGAGACAA
TP5218_Hit	D	1	chr5	.	CAGCAAGACCAATTTGGGCGCCTTCTACCAACATTCGTCCAGGTTATCAATTTTACGGAGCAAG
TP5218_Query	D	1	chr5	.	CAGCAAGACCAATTTGGGCGCCTTCTACCAACATTCGTCCAGGTTATCAATTTTACCGAGCAAG
TP52205_Hit	D	1	chr5	.	CAGCTGTAGGTTGAATCACAGCTGATGGTGGAGTCGTAGTCTTCCTCGACGCTGAAAAAAAAAA
TP52205_Query	D	1	chr5	.	CAGCTGTAGGTTGAATCACAGCTGATGGTGGAGTCGTAGTCTTCCTCGACGCAGAAAAAAAAAA
TP52219_Hit	D+G	1	chr5	.	CAGCTGTAGTGTTCAGCCAAGGGTTCAATATCAAAATCTTCAATCATGCATTTAAATAAATCCA
TP52219_Query	D+G	1	chr5	.	CAGCTGTAGTGTTCAGCCAAGGGTTCAATAGCAAAATCTTCAATCATGCATTTAAATAAATCCA
TP52249_Hit	D	1	chr5	.	CAGCTGTATGCTTGATAAATTCTCAAATGGAGAATGGTGACTTTCCGCAAGAGGTATTGTTGAA
TP52249_Query	D	1	chr5	.	CAGCTGTATGCTTGATAAATTCTCAAATGGAGAATGGTGACTTTCCGCAACAGGTATTGTTGAA
TP52371_Hit	D	1	chr5	.	CAGCTGTCCTTGTATTTTCACAAGATATGAATGTTACTTAAGAACAACAGTGAACTGATTATTT
TP52371_Query	D	1	chr5	.	CAGCTGTCCTTATATTTTCACAAGATATGAATGTTACTTAAGAACAACAGTGAACTGATTATTT
TP52633_Hit	D	1	chr5	.	CAGCTTTGGTATGCATATGCAAAGACATTGGGAGAAAGAGAGGCTTGGAGGATTGCAGAGGAAA
TP52633_Query	D	1	chr5	.	CAGCTGTGGTATGCATATGCAAAGACATTGGGAGAAAGAGAGGCTTGGAGGATTGCAGAGGAAA
TP52676_Hit	D+G	1	chr5	.	CAGCTGTGTCTCTTCTGCAACAAGAGATCTGTGGGAGCGTCTTTTCAATGAAGGGTATAAAGCC
TP52676_Query	D+G	1	chr5	.	CAGCTGTGTCTCTTCCGCAACAAGAGATCTGTGGGAGCGTCTTTTCAATGAAGGGTATAAAGCC
TP52721_Hit	D	1	chr5	.	CAGCTGTGTTTGTAGAGGTAAGAGATTGATGAGTTTGGGGTTGGCAATTTTGGTTCGACTTGAT
TP52721_Query	D	1	chr5	.	CAGCTGTGTTTGTAGAGGTAAGAGAGTGATGAGTTTGGGGTTGGCAATTTTGGTTCGACTTGAT
TP52750_Hit	D	1	chr5	.	CAGCTGTTAAGTCCTTTACCAATTTCAGTCCTACTTGATGAGTCTTGTATGCAACAGGATGCGG
TP52750_Query	D	1	chr5	.	CAGCTGTTAAGTCCTTTACCAACTTCAGTCCTACTTGATGAGTCTTGTATGCAACAGGATGCGG
TP52804_Hit	D	1	chr5	.	CAGCTGTTAGTTCAACCTACAAAATATAATTGACTTTGGTAAGATATAACAGAACCTTCATCAA
TP52804_Query	D	1	chr5	.	CAGCTGTTAGTTCAACCTACAAAATATAATTGACTTTGGTAAGATATAACAAAACCTTCATCAA
TP5298_Hit	D	1	chr5	.	CAGCAAGAGCAGAAGACTTTGCATTTTCCATTATGTTTTTCCAAGTCGAAAGCAAACCTTCCAA
TP5298_Query	D	1	chr5	.	CAGCAAGAGCAGAAGAATTTGCATTTTCCATTATGTTTTTCCAAGTCGAAAGCAAACCTTCCAA
TP53248_Hit	D+G	1	chr5	.	CAGCTTAAACTTTTTGATCTTGGAAGCATTTTCAACCTGAAAGATGATCTCAGTACTTGATTTT
TP53248_Query	D+G	1	chr5	.	CAGCTTAAACTTTTTGATCTTGGAAGCATTTCCAACCTGAAAGATGATCTCAGTACTTGATTTT

Name	Filter	Nb hit (Mt4.0)	Mt Chr (Mt4.0)	Ms Chr (Li et al., 2014)	Sequence
TP53584_Hit	D	1	chr5	.	CAGCTTACAGCTTTTAAACAAGTGGAGAATAAATTTGTCATCGATGTAGGAATGATAAGAGAGA
TP53584_Query	D	1	chr5	.	CAGCTTACAGCTTTTAAACAAGTGGAGAATAAATTTGTCATCGATGTAGGAATGACAAGAGAGA
TP53788_Hit	D+G	1	chr5	.	CAGCTTGGCTGTTAGATCTAATAGTGCATTGAATCTGATGGTTTTGTAATAGTTGGATTGTGCA
TP53788_Query	D+G	1	chr5	.	CAGCTTAGCTGTTAGATCTAATAGTGCATTGAATCTGATGGTTTTGTAATAGTTGGATTGTGCA
TP5386_Hit	D	1	chr5	.	CAGCAAGAGTCTCTCCAGCTTGATGCATAGCCTTAAGAACCAAATTAAAACTCGCTGTATTCGG
TP5386_Query	D	1	chr5	.	CAGCAAGAGTCTCTCCAGCCTGATGCATAGCCTTAAGAACCAAATTAAAACTCGCTGTATTCGG
TP5391_Hit	D+G	1	chr5	.	CAGCAAGAGTGGAAGTGGTCCAACAAGACTGCAATTCTTTATGTTAACACCCATATCACCGCGA
TP5391_Query	D+G	1	chr5	.	CAGCAAGAGTGGAAGTGCTCCAACAAGACTGCAATTCTTTATGTTAACACCCATATCACCGCGA
TP53940_Hit	D+G	1	chr5	.	CAGCTTATACGTTTGCAATCTTTTCTAGATATCAGTAACAACCACAATACCTGCATATTTGACG
TP53940_Query	D+G	1	chr5	.	CAGCTTATACGTGTGCAATCTTTTCTAGATATCAGTAACAACCACAATACCTGCATATTTGACG
TP5398_Hit	D+G	1	chr5	.	CAGCAAGAGTGTTGGGTATAAACGAGGCACCTGAGGCAAGAGGCTGTGATCCAGCAAATGACAC
TP5398_Query	D+G	1	chr5	.	CAGCAAGAGTGTTGGGTATAAAAGAGGCACCTGAGGCAAGAGGCTGTGATCCAGCAAATGACAC
TP54228_Hit	D	1	chr5	.	CAGCTTATTAATACAACAATTCAGTCAAACAACATAGGAAGGTCAAAACGTAAATGACATGAAA
TP54228_Query	D	1	chr5	.	CAGCTTATTAATACAACAATTCAATCAAACAACATAGGAAGGTCAAAACGTAAATGACATGAAA
TP54279_Hit	D	1	chr5	.	CAGCTTATTCTTCAATGGTTGACTGGGATAGCTGTGCTTATCTGCTGTTTTCTCAAGAGGAAGG
TP54279_Query	D	1	chr5	.	CAGCTTATTCTTCAATGGTTGACTGGGATAGCTGTGCTTATATGCTGTTTTCTCAAGAGGAAGG
TP54343_Hit	D	1	chr5	.	CAGCTTATTTGCTGGAAGTTTGACTATTGACAATGGGCTAACTTTTAATTTATCCATCATTATA
TP54343_Query	D	1	chr5	.	CAGCTTATTTGCTGGAAGTTTGACTATTGACAATGGGCTAACTTTTAACTTATCCATCATTATA
TP54373_Hit	D+G	1	chr5	.	CAGCTTCAAAACCAGCTAAGACATCTGCATCTGAGCAACCACCTTTCCAACACACTTTGTAGGC
TP54373_Query	D+G	1	chr5	.	CAGCTTCAAAACCAGCTAAGACATCTGCATCTGAGCAACCACCTTTCCAACAAACTTTGTAGGC
TP54382_Hit	D	1	chr5	.	CAGCTTCAAAAGTGGACCAACCCCACCTTCTTCAATTATAAGCTTCCCGTATCGGTCACTACCT
TP54382_Query	D	1	chr5	.	CAGCTTCAAAAGTGGACCAACCCCACCTTCTTCAATTATAAGCTTCCCATATCGGTCACTACCT
TP54430_Hit	D+G	1	chr5	.	CAGCTTCAAATCCCGCACCCAACAAAACGCGAGCCACTACCTTCTGAGAGAGCTTCGCTGAAAA
TP54430_Query	D+G	1	chr5	.	CAGCTTCAAATCCCACACCCAACAAAACGCGAGCCACTACCTTCTGAGAGAGCTTCGCTGAAAA
TP5447_Hit	D	1	chr5	.	CAGCAAGATCAACTATTGGCCTGATTTTGATCAACTCCTTCTCTATCGGTATCCTAGACTTTGC
TP5447_Query	D	1	chr5	.	CAGCAAGATCAACTATTGGCCTGATTTTGATCAACTCCTTCTCTATCGGTATCCTAGACTTAGC
TP54559_Hit	D	1	chr5	.	CAGCTTCAAGTGGTGGTGGAGGGTTACTTGGAGGTTCAGCTTATGGTAATCAAATAAATTGAAC
TP54559_Query	D	1	chr5	.	CAGCTTCAAGTGGTGGTGGAGGATTACTTGGAGGTTCAGCTTATGGTAATCAAATAAATTGAAC
TP54727_Hit	D+G	1	chr5	.	CAGCTTCACCTGTGATGGTGACTCGTCCAAGGTAAATATAAGATTTTTCCAATTTTTCTATATT
TP54727_Query	D+G	1	chr5	.	CAGCTTCACCTGTGATGGTGACTCGTCCAAGGTAAATATAAGATTTTTCCAAATTTTCTATATT
TP54728_Hit	D+G	1	chr5	.	CAGCTTCACCTGTGGACAGCACGCCTGCTACTACGACAGTGATACGTTCAAAGGTCTATCATGC
TP54728_Query	D+G	1	chr5	.	CAGCTTCACCTGTGGACAGCACGCCTGCTACTACAACAGTGATACGTTCAAAGGTCTATCATGC
TP54735_Hit	D	1	chr5	.	CAGCTTCACGATCTTCACCTCGTATAATTGTTGGTGACCACCAGCCAACGTGCTGAAAAAAAAA
TP54735_Query	D	1	chr5	.	CAGCTTCACGATCTTCACCTCGTATAATTGTTGGTGACCACCAGCCAACATGCTGAAAAAAAAA
TP54743_Hit	D	1	chr5	.	CAGCTTCGCGTAGTAGGCGCTTTACTCTGGTATGTTTTCTCAACCAAGTAAAACGGCGCCTGGC
TP54743_Query	D	1	chr5	.	CAGCTTCACGTAGTAGGCGCTTTACTCTGGTATGTTTTCTCAACCAAGTAAAACGGCGCCTGGC
TP54853_Hit	D	1	chr5	.	CAGCTTCGGCAGTTTCATAAGTTCCAAGCCAAACTCTCATTCTATTCTGTGGAAGTCTAATCTC
TP54853_Query	D	1	chr5	.	CAGCTTCAGCAGTTTCATAAGTTCCAAGCCAAACTCTCATTCTATTCTGTGGAAGTCTAATCTC
TP55271_Hit	D	1	chr5	.	CAGCTTCCATGCTGGAAGAATTTGATCCCATCGAAAGTTTCAGCTCCATACTTTGAATATTCGG
TP55271_Query	D	1	chr5	.	CAGCTTCCATGCTGGAAGAATTTGATCCCATCGAAAGTTTCAGCACCATACTTTGAATATTCGG
TP55446_Hit	D	1	chr5	.	CAGCTTCCTGCCTGGCTCTTTCCTCTTCCATTGAAACTCTTAAGGCAAGAGCCAATTCAGGATC
TP55446_Query	D	1	chr5	.	CAGCTTCCTGCCTAGCTCTTTCCTCTTCCATTGAAACTCTTAAGGCAAGAGCCAATTCAGGATC
TP5551_Hit	D	1	chr5	.	CAGCAAGCAATGGAATAGCTGTTAATCTTCTCACTGACTTGAACTTGAAAACATCAATCATTGA
TP5551_Query	D	1	chr5	.	CAGCAAGCAAAGGAATAGCTGTTAATCTTCTCACTGACTTGAACTTGAAAACATCAATCATTGA
TP55898_Hit	D+G	1	chr5	.	CAGCTTCTCCTCTTCCTTCAATTGAAGTTGTTCTTCCCGATATTTTAGCAACTGCACGCTTTGC
TP55898_Query	D+G	1	chr5	.	CAGCTTCTCCTCTTCCTTCAATTGAAGCTGTTCTTCCCGATATTTTAGCAACTGCACGCTTTGC
TP55958_Hit	D	1	chr5	.	CAGCTTCTCTTTCATGCATCTTAATTTGTCTTGCTGACCATACTCCTTTCCGTTTTTTTCTTGC
TP55958_Query	D	1	chr5	.	CAGCTTCTCTTTCATGCATCTTAATTTGTCTTGCTGACCATACTCCTCTCCGTTTTTTTCTTGC
TP56380_Hit	D	1	chr5	.	CAGCTTCTGCTCTGTTCCTTCTCGACATCAAAGGCCGCATCCTCGTCTGGCGTGACTACCGCGG
TP56380_Query	D	1	chr5	.	CAGCTTCTGCTCTGTTCCTCCTCGACATCAAAGGCCGCATCCTCGTCTGGCGTGACTACCGCGG
TP56649_Hit	D	1	chr5	.	CAGCTTCTTTAAAGAGCTCTACCCTTCTTCTTATCTGTCACATGTGGTTGTTCGTCGTACAAGC
TP56649_Query	D	1	chr5	.	CAGCTTCTTTAAAGAGCTCTACCCTTCCTCTTATCTGTCACATGTGGTTGTTCGTCGTACAAGC
TP57223_Hit	D	1	chr5	.	CAGCTTGATGGTTTGAAACATTGGATGGTTTAAGTTAGGGCCTTTAAGTTATTACCAAGTTAGT
TP57223_Query	D	1	chr5	.	CAGCTTGATGGTTTGAAACATTAGATGGTTTAAGTTAGGGCCTTTAAGTTATTACCAAGTTAGT

Name	Filter	Nb hit (Mt4.0)	Mt Chr (Mt4.0)	Ms Chr (Li et al., 2014)	Sequence
TP57283_Hit	D	1	chr5	.	CAGCTTGATTTTGCTATCAATGCTCTTATGTCCATGAACATGGTGGTTACACTGTTGGTGGCAT
TP57283_Query	D	1	chr5	.	CAGCTTGATTTTGCTATCAACGCTCTTATGTCCATGAACATGGTGGTTACACTGTTGGTGGCAT
TP57339_Hit	D	1	chr5	.	CAGCTTGTACAACCCTTACTTTGTCCAAGAGACTTCCAGTTCCATCACATTGAATTGTACCGAG
TP57339_Query	D	1	chr5	.	CAGCTTGCACAACCCTTACTTTGTCCAAGAGACTTCCAGTTCCATCACATTGAATTGTACCGAG
TP57369_Hit	D	1	chr5	.	CAGCTTGCAGGAATCAAACCATTTACATTAAGAATCTCAATGAAAATATCAAGAAAGATGAGTT
TP57369_Query	D	1	chr5	.	CAGCTTGCAGGAATCAAACCATTTACATTAAGAATCTCAATGAAAATATCAAGAAAGACGAGTT
TP5740_Hit	D	1	chr5	.	CAGCAAGCCTTAAGAGGGGATTACAGATAATTGATTCTCATCAGAGGAACTCATTACTGAACAA
TP5740_Query	D	1	chr5	.	CAGCAAGCCTTAAGAGGGGATTACAAATAATTGATTCTCATCAGAGGAACTCATTACTGAACAA
TP57494_Hit	D	1	chr5	.	CAGCTTGCGGAGCATCTTTAGCCATGGTTTTATCTGCAGAAAAAAAAAAAAAAAAAAAAAAAAA
TP57494_Query	D	1	chr5	.	CAGCTTGCGGAGCATCTTTAGCCATGGTTTAATCTGCAGAAAAAAAAAAAAAAAAAAAAAAAAA
TP57604_Hit	D	1	chr5	.	CAGCTTGGAAGCAAAAATTGTCTGTACAAGAAACCCTAGCTTCCAGCACAGTAAGCCTCATCTC
TP57604_Query	D	1	chr5	.	CAGCTTGGAAACAAAAATTGTCTGTACAAGAAACCCTAGCTTCCAGCACAGTAAGCCTCATCTC
TP57730_Hit	D	1	chr5	.	CAGCTTGGCATTTTAAAGTTTAGTCCAACACTTGCCAGTACTATTGAGGCCAGTGGAGAAATAT
TP57730_Query	D	1	chr5	.	CAGCTTGGCATTTTAAAGTTCAGTCCAACACTTGCCAGTACTATTGAGGCCAGTGGAGAAATAT
TP57755_Hit	D	1	chr5	.	CAGCTTGGGTCGTTAGTTAATATTTCATTCTTCTCTTTTTGAAGCCCATCATTCATGAATGATT
TP57755_Query	D	1	chr5	.	CAGCTTGGCTCGTTAGTTAATATTTCATTCTTCTCTTTTTGAAGCCCATCATTCATGAATGATT
TP58208_Hit	D	1	chr5	.	CAGCTTGTGGAATCGCTTCCATGCACCGTCATCTGGAAATGCAATAACTACCTGATAATAACAT
TP58208_Query	D	1	chr5	.	CAGCTTGTGGAATCGCTTCCATGCACCGTCATCTGGAAATGCAATAACTACCTGATAATAACAA
TP58310_Hit	D	1	chr5	.	CAGCTTGTTATTTTGATCACATTTTGCCCTTTCAACATCATATACCGGTCAAGTCGTTTTTTCT
TP58310_Query	D	1	chr5	.	CAGCTTGTTATTTTGATCACATTTTGCCCTTTCAACATCATATACCGGTCAAGTCGTTTCTTCT
TP58385_Hit	D	1	chr5	.	CAGCTTGTTGTTGCTCAGGTCGACAAATTGTAATGAGGTTAGATTATTCCAACAATTAGGCAAC
TP58385_Query	D	1	chr5	.	CAGCTTGTTGTTGCTCAGGTCAACAAATTGTAATGAGGTTAGATTATTCCAACAATTAGGCAAC
TP58451_Hit	D+G	1	chr5	.	CAGCTTTAAAAATAAATAGCTTTCTCTCAGATCAGCTATCAACATGTTTTTTAATTCTACATCT
TP58451_Query	D+G	1	chr5	.	CAGCTTTAAAAATAAATAGCTTTCTCTCAGATCAGCTATCAACATGGTTTTTAATTCTACATCT
TP58524_Hit	D+G	1	chr5	.	CAGCTTTAAGAAAATAATCATCAAGCTCCTTAATAATTTCAACCAGATCTTTACTGTTTCTTGA
TP58524_Query	D+G	1	chr5	.	CAGCTTTAAGAAAATAATCATCAAGCTCCTTAATAACTTCAACCAGATCTTTACTGTTTCTTGA
TP58803_Hit	D+G	1	chr5	.	CAGCTTTATCTATACATCGTTGTATTTCTTCCCGACGATGCCTGGGATATAGTTTCCTAAATGT
TP58803_Query	D+G	1	chr5	.	CAGCTTTATCTATACAGCGTTGTATTTCTTCCCGACGATGCCTGGGATATAGTTTCCTAAATGT
TP58845_Hit	D	1	chr5	.	CAGCTTTATTCTCAAGTGTGTCCATTTCCTTCTTTGAAATGCCTCCAAGCTTGTAGAACAAATG
TP58845_Query	D	1	chr5	.	CAGCTTTATTCTCAAGTGTGTCCATTACCTTCTTTGAAATGCCTCCAAGCTTGTAGAACAAATG
TP59117_Hit	D	1	chr5	.	CAGCTTTCGAACTGATGAGTATTATAATAGAGAGATGCATCCAGTTGCAGGATCCTACTTGCAG
TP59117_Query	D	1	chr5	.	CAGCTTTCGAACTGATGAGTATTATAATAGAGAGATGCATCCAGTTGCAGAATCCTACTTGCAG
TP59172_Hit	D+G	1	chr5	.	CAGCTTTCTATAAGTCACCTGCTTCTTGGGTAGGGGGTGGGCTTTCAGGTTGAGAGAGTTGGTG
TP59172_Query	D+G	1	chr5	.	CAGCTTTCTATAAGTCACCTCCTTCTTGGGTAGGGGGTGGGCTTTCAGGTTGAGAGAGTTGGTG
TP5922_Hit	D	1	chr5	.	CAGCAAGGAAATCATTGTTCGCTATGCATCTGGCTCCATCCAAGCCACATACATCCACCTTTAT
TP5922_Query	D	1	chr5	.	CAGCAAGGAAATCATTGTTCGCTATGCATCTGGCTCCATCCAAGCCACATACATCCACCTTGAT
TP59247_Hit	D	1	chr5	.	CAGCTTTCTTATGGCGTTTGCTGGTAAGTCTTACATTATTATTGAATCATTTGATTCTGGGTTT
TP59247_Query	D	1	chr5	.	CAGCTTTCTTATGGCGTTTGCTGGTAAGTCTTGCATTATTATTGAATCATTTGATTCTGGGTTT
TP59276_Hit	D+G	1	chr5	.	CAGCTTTCTTTCGATTACTTGAGAGATCATGACGAAAAAATACCAAGGATGTGCTGTGGGAATA
TP59276_Query	D+G	1	chr5	.	CAGCTTTCTTTCGATTACTTGAGAGATCATGACGAAAAAATACCAAGGATGTGCCGTGGGAATA
TP5935_Hit	D	1	chr5	.	CAGCAAGGAACGAACAGAGAGAATCAAACCTTTGATAAAATCTTCAGGGCACTCATCATCTAGC
TP5935_Query	D	1	chr5	.	CAGCAAGGAACGAACAGAAAGAATCAAACCTTTGATAAAATCTTCAGGGCACTCATCATCTAGC
TP5942_Hit	D	1	chr5	.	CAGCAAGGAACTCTGGGTCAATATCCCCAGAGTTCTGAGATTCGACATTTGGTGGCTGAGCCAC
TP5942_Query	D	1	chr5	.	CAGCAAGGAACTCTGAGTCAATATCCCCAGAGTTCTGAGATTCGACATTTGGTGGCTGAGCCAC
TP5956_Hit	D	1	chr5	.	CAGCAAGGAATAGTAGGAACTCAACAAGGAAAAGGGACGAAAATATGCCACCATCATTTGTCAT
TP5956_Query	D	1	chr5	.	CAGCAAGGAATAGTAGGAACTCAACAAGGAAAAGGGAAGAAAATATGCCACCATCATTTGTCAT
TP59581_Hit	D	1	chr5	.	CAGCTTTGGATCTTGAATCCGTGAAGAGGACTTGGTCTGAGGTGAATAAACCCTTTCCCTTTTG
TP59581_Query	D	1	chr5	.	CAGCTTTGGATCTTGAATCCGTGAAGAGGACTTGGTCTGAGGTGAATAAACCCTTCCCCTTTTG
TP59634_Hit	D	1	chr5	.	CAGCTTTGGTAGACATGTATGCAAAGTGTGGAGACCTTCAAACAGCCCGAAAAGTTTTCGATAG
TP59634_Query	D	1	chr5	.	CAGCTTTGGTAGACATGTACGCAAAGTGTGGAGACCTTCAAACAGCCCGAAAAGTTTTCGATAG
TP59651_Hit	D	1	chr5	.	CAGCTTTGGTTCCATGTTCTCAATTGGAGGATCATAGAAAACACCACTTGTCTCCTCTTTATCA
TP59651_Query	D	1	chr5	.	CAGCTTTGGTTCCATGTTCTCAATTGGAGGATCATAGAAAACACCACTTGTATCCTCTTTATCA
TP59806_Hit	D	1	chr5	.	CAGCTTTTAAAGAGATGCCGTAAGATTAGACATTTGAACCTAACCAACTGTAAAAGTTTCAAGT
TP59806_Query	D	1	chr5	.	CAGCTTTTAAAGAGATGCCATAAGATTAGACATTTGAACCTAACCAACTGTAAAAGTTTCAAGT

Name	Filter	Nb hit (Mt4.0)	Mt Chr (Mt4.0)	Ms Chr (Li et al., 2014)	Sequence
TP59868_Hit	D	1	chr5	.	CAGCTTTTAGAACAACTGCATGGTTAATTAATGATCTAGAATATTCTAATGACACTTGTAGAGT
TP59868_Query	D	1	chr5	.	CAGCTTTTAGAACAACTGCACGGTTAATTAATGATCTAGAATATTCTAATGACACTTGTAGAGT
TP5989_Hit	D	1	chr5	.	CAGCAAGGAGAATAAAATAGAGAATAAGGCATAAATTCATTTAGAGTTTAAGTCAGAAGTTCAG
TP5989_Query	D	1	chr5	.	CAGCAAGGAGAATAAAATAGAGAATAAGGCATAAATTCATTTAGAATTTAAGTCAGAAGTTCAG
TP60037_Hit	D	1	chr5	.	CAGCTTTTCTTCTGAAATTAGTATTGATCACAACGAAAATTCAGTACAGCATATAATGTGAAGG
TP60037_Query	D	1	chr5	.	CAGCTTTTCTTCTGAAATAAGTATTGATCACAACGAAAATTCAGTACAGCATATAATGTGAAGG
TP60340_Hit	D	1	chr5	.	CAGCTTTTTCCATAAACTTCACGAGGACCTCTTGAATCTACAAACCCAAATCGAATTCCTCTAA
TP60340_Query	D	1	chr5	.	CAGCTTTTTCCATAAACTTCACAAGGACCTCTTGAATCTACAAACCCAAATCGAATTCCTCTAA
TP6039_Hit	D	1	chr5	.	CAGCAAGGATTCTTCAATGGGGAAATTGATTTCTGATGTTAATGATTAATTTGATATAGGCCTT
TP6039_Query	D	1	chr5	.	CAGCAAGGATTCTTCAATGGGGAAATTGATTTCTCATGTTAATGATTAATTTGATATAGGCCTT
TP60501_Hit	D	1	chr5	.	CTGCAAAAAACATAAAAGTAAATTATAAGGGTCAGCTTCAAAGATTTAAAGTTTAAATGGCTAT
TP60501_Query	D	1	chr5	.	CTGCAAAAAACATAAAAGTAAATTATAAGGGTCAGCTTCAAAGATTTAAAGTTTAAACGGCTAT
TP60526_Hit	D	1	chr5	.	CTGCAAATAATTGGACCTGTTGTTAGAACTAAATCCTCATAAAGAATTGTAGGAATTGATGCCA
TP60526_Query	D	1	chr5	.	CTGCAAAAAAATTGGACCTGTTGTTAGAACTAAATCCTCATAAAGAATTGTAGGAATTGATGCCA
TP60683_Hit	D+G	1	chr5	.	CTGCAAAACCTTCCAAGACAACAAGCAAGAGCAGAAATTACTATTATGATGGCTAAGACAACAA
TP60683_Query	D+G	1	chr5	.	CTGCAAAACCTTCCAAGACAACAAGCAACAGCAGAAATTACTATTATGATGGCTAAGACAACAA
TP60795_Hit	D	1	chr5	.	CTGCAAAAGGGTAGAAGGTGAAGTTACTGTCTGCCCTGGTTGATAGATACCGGGAAATGCTCCG
TP60795_Query	D	1	chr5	.	CTGCAAAAGGGTAGAAGGTGAAGCTACTGTCTGCCCTGGTTGATAGATACCGGGAAATGCTCCG
TP61142_Hit	D	1	chr5	.	CTGCAAACGTTTCCTTTGAGACCTTAAGCTCCATTGTACGAAGTATTAGCTGAGACTTTGCACA
TP61142_Query	D	1	chr5	.	CTGCAAACGTTTCCTTTGAGACCTTAAGCTCCATTGTACGAAGTATTAGCTGAGACTTGGCACA
TP61413_Hit	D	1	chr5	.	CTGCAAAGTCAAGGGCACAACTACTTCAAGCATTGTCTGATGCTAAGCTTGAGCTTTCTACACT
TP61413_Query	D	1	chr5	.	CTGCAAAGTCAAGGGCACAACTACTTCAAGCATTGTCTGATGCTAAACTTGAGCTTTCTACACT
TP61646_Hit	D	1	chr5	.	CTGCAAATGAAGGACTAAAAGATGAAAGTAGCTTGTTGACTTACATTGTTCACGTTGAAAAGCC
TP61646_Query	D	1	chr5	.	CTGCAAATGAAGGACTAAAAGATGAAAGTAGCTTGTTGACTTACATTGTTCAAGTTGAAAAGCC
TP6167_Hit	D	1	chr5	.	CAGCAAGGGTTTCGGTAGCAAAAACAACCTTGACAAGACCTCTTTGAAACAACTCTTCTATGAA
TP6167_Query	D	1	chr5	.	CAGCAAGGGTTTCAGTAGCAAAAACAACCTTGACAAGACCTCTTTGAAACAACTCTTCTATGAA
TP6172_Hit	D+G	1	chr5	.	CAGCAAGGTAAGCTAAAATGCAACATCGATGCGGCGTTTTTGGAAGATTTAAATCGTGTTGGTT
TP6172_Query	D+G	1	chr5	.	CAGCAAGGTAAGCTAAAATGCAACATCGATGCGGCGTTTTCGGAAGATTTAAATCGTGTTGGTT
TP61800_Hit	D	1	chr5	.	CTGCAAATTATCACTGAAAGTATGCAGAAGATATTGGAGAATAATACCTTGCTGGAGAAGTCCC
TP61800_Query	D	1	chr5	.	CTGCAAATTATCACTGAAAGTATGCAGAAGATATTGGAGAATAATACCTTGCTGGAAAAGTCCC
TP61808_Hit	D	1	chr5	.	CTGCAAATTCAATAAACCCTTCATCAGATCAACTCTATCTTCTCCTAAATGCTCTTGTCCCTCT
TP61808_Query	D	1	chr5	.	CTGCAAATTCAATAAACCCTTCATCAGATCAACTCTATCTTCTCCTAAATGCCCTTGTCCCTCT
TP61915_Hit	D	1	chr5	.	CTGCAAATTTTTGAGGCTCATGTGGTGAAAAATAAGGAGAGAAAGGGCATTCCTCAGCACATCT
TP61915_Query	D	1	chr5	.	CTGCAAATTTTTGAGGCTCATGTGGTGAAAAATAAGGAGAGAAAGGACATTCCTCAGCACATCT
TP61976_Hit	D	1	chr5	.	CTGCAACAACCATCACCGGCCCGGACGCAATCAACGACCCGATAACCAGTCCTCCAACAACCTG
TP61976_Query	D	1	chr5	.	CTGCAACAACCATCACCGGCCCCGACGCAATCAACGACCCGATAACCAGTCCTCCAACAACCTG
TP62422_Hit	D	1	chr5	.	CTGCAACCGACCATTTCAACTCAAAGAACATTCTAGGAAGAGGTGGTTTTGGAATAGTTTACAA
TP62422_Query	D	1	chr5	.	CTGCAACCGACCATTTCAACTCAAAGAACATTTTAGGAAGAGGTGGTTTTGGAATAGTTTACAA
TP62443_Hit	D	1	chr5	.	CTGCAATCGTGACAACTGACCGAGTTCTTGAGGGATATTTCCAAAGAAACTATTTTTTGCAACG
TP62443_Query	D	1	chr5	.	CTGCAACCGTGACAACTGACCGAGTTCTTGAGGGATATTTCCAAAGAAACTATTTTTTGCAACG
TP62497_Hit	D+G	1	chr5	.	CTGCAACCTTTGTTCGGAGAGAGGAGCATGATCGGTGGATGTTGGTACAGATGATAAATGTTCG
TP62497_Query	D+G	1	chr5	.	CTGCAACCTTTGTTCGGAGAGAGGAGCATGATCAGTGGATGTTGGTACAGATGATAAATGTTCG
TP6257_Hit	D	1	chr5	.	CAGCAAGGTTGATCCAAGAAGTCACTGCTTCTGCGAATAATGTAGAATAAGATGCCTTATACTA
TP6257_Query	D	1	chr5	.	CAGCAAGGTTGATCCAAGAAGTCACTGCTTCGGCGAATAATGTAGAATAAGATGCCTTATACTA
TP62755_Hit	D	1	chr5	.	CTGCAACTCTCCCTGTTCCTTCTCAACAATCTTAGCCAAACAAAGCATCCCTTTCGTCTCGGTT
TP62755_Query	D	1	chr5	.	CTGCAACTCTCCCTGTTCCTTCTCAACAATCTTAGCCAAACAAAGCATCCCTTTCGTCTCAGTT
TP62799_Hit	D+G	1	chr5	.	CTGCAACTGCAAGTGATAGTAGGAACAGTTCAAGAACACTTGATCAAACACCGACATGGGGTGG
TP62799_Query	D+G	1	chr5	.	CTGCAACTGCAAGTGATAGTAGGAACAGTTCAAGAACACTTGATCAAACACCAACATGGGGTGG
TP62914_Hit	D	1	chr5	.	CTGCAACTTGCAAGGATTTGCGTGAGCTCCGAGTTTTTCCTGTGGATGCAAGGGAGGAGACTGA
TP62914_Query	D	1	chr5	.	CTGCAACTTGCAAGGACTTGCGTGAGCTCCGAGTTTTTCCTGTGGATGCAAGGGAGGAGACTGA
TP62969_Hit	D	1	chr5	.	CTGCAAGAAACCAAACCTTTAATGAGCACCACACATCAGTCAATGAAAATAAGGCAAGCTTCAC
TP62969_Query	D	1	chr5	.	CTGCAAGAAACCAAACCTTTAATGAGCACCACACATCAGTCAATGAAAATAAGGCAAGCTTCAA
TP63022_Hit	D	1	chr5	.	CTGCTAGAAGCACCTTGCGTCCACCATCATTGCAAACTCTTCTAGAGGATACACCTATTTCAGC
TP63022_Query	D	1	chr5	.	CTGCAAGAAGCACCTTGCGTCCACCATCATTGCAAACTCTTCTAGAGGATACACCTATTTCAGC

Name	Filter	Nb hit (Mt4.0)	Mt Chr (Mt4.0)	Ms Chr (Li et al., 2014)	Sequence
TP63225_Hit	D	1	chr5	.	CTGCAAGCAAAACTCAGATTCCACAAAAAGTTCTATAATTGATTCTGGCTCATTACATTTAGCA
TP63225_Query	D	1	chr5	.	CTGCAAGCAAAACTCAGATACCACAAAAAGTTCTATAATTGATTCTGGCTCATTACATTTAGCA
TP63240_Hit	D+G	1	chr5	.	CTGCAAGCAAGTTTTGGAGCATAGTTTCCTTCCCTGGTTGTAGGTTTATGCAATTTAGTGGTAA
TP63240_Query	D+G	1	chr5	.	CTGCAAGCAAGTTTTGGAGCATAGTTTCCTTCCCTGGTTGTAGGTTTATGCAATTTAATGGTAA
TP63456_Hit	D	1	chr5	.	CTGCAAGGGGAATTGGCGCATGAAATAAAGCAGTAGACACTTGGAGTGGATTCCGAAGGTTGTT
TP63456_Query	D	1	chr5	.	CTGCAAGGGGAATTGGCGCATGAAATAAAGCAGTAGACACTTGGAGTGGATTCCGAAGGTAGTT
TP63904_Hit	D	1	chr5	.	CTGCAATAGTTTATGATGCTAATGCTATCTCGGCACCTGGTAATGGAAACATAATAATGTCTGA
TP63904_Query	D	1	chr5	.	CTGCAATAGTTTATGATGCTAACGCTATCTCGGCACCTGGTAATGGAAACATAATAATGTCTGA
TP63988_Hit	D	1	chr5	.	CTGCAATATGTTGTGCATTTGGACTTTCTTCTTCAATTTCGAAATCAACGCGATCAGTACTGAT
TP63988_Query	D	1	chr5	.	CTGCAATATGTTGTGCATTTGGACTTTCTTCTTCAATTTCGAAATCAACGCGATCAGCACTGAT
TP6435_Hit	D+G	1	chr5	.	CAGCAAGTGAATAGTGATGCTGGATCAGGAGCAATTGATCCTGCTTTTCTAGATGCTCTTCCTG
TP6435_Query	D+G	1	chr5	.	CAGCAAGTGAATAGTGATGCTGGATCAGGAGCAATTGATCCTGCTTTGCTAGATGCTCTTCCTG
TP64390_Hit	D	1	chr5	.	CTGCAATGGCAGAATGCTATACCAGAATTTGATGTTCGGCATCTGAGGGACTTAAATTAGTTGA
TP64390_Query	D	1	chr5	.	CTGCAATGGCAAAATGCTATACCAGAATTTGATGTTCGGCATCTGAGGGACTTAAATTAGTTGA
TP64485_Hit	D	1	chr5	.	CTGCAATGTCTTGAACCCTACTCAACATATAGGCCATGCATGGTTGATATTGTCCGCGAGTTGG
TP64485_Query	D	1	chr5	.	CTGCAATGTCTAGAACCCTACTCAACATATAGGCCATGCATGGTTGATATTGTCCGCGAGTTGG
TP64761_Hit	D	1	chr5	.	CTGCAATTGCCCTCTTATGCAATATCTGCACAAACAATACCATGCATTTCAGAGTTCATTGATC
TP64761_Query	D	1	chr5	.	CTGCAATTGCCCTCTTATGCAACATCTGCACAAACAATACCATGCATTTCAGAGTTCATTGATC
TP64807_Hit	D	1	chr5	.	CTGCAATTGGTACTTTGGATGGTATTATTGACACCGTTTCAGCTGTTCATCCTCTCTTACCAAT
TP64807_Query	D	1	chr5	.	CTGCAATTGGTACTTTGGATGGTATTATTGACACCGTTTCAGCAGTTCATCCTCTCTTACCAAT
TP6508_Hit	D	1	chr5	.	CAGCTAGTGTCACTATTCCTGCATTTAACAACATGCAAGTGAAAACCAAAAGCAATTGCAAAGA
TP6508_Query	D	1	chr5	.	CAGCAAGTGTCACTATTCCTGCATTTAACAACATGCAAGTGAAAACCAAAAGCAATTGCAAAGA
TP65707_Hit	D	1	chr5	.	CTGCACATTGCGCCACCAACGACCTCGCTTTGTTCCCAAGTCTACCCATCAAAGTCCACTTATC
TP65707_Query	D	1	chr5	.	CTGCACATTGCGCCACCAACGACCTCGCCTTGTTCCCAAGTCTACCCATCAAAGTCCACTTATC
TP65752_Hit	D	1	chr5	.	CTGCACCAACACAACCACCATCAGCAACCCCAACTCCACCAACCCCAGCACCAAAATCTCCAGC
TP65752_Query	D	1	chr5	.	CTGCACCAACACAACCACCATCAGCAACCCCAACACCACCAACCCCAGCACCAAAATCTCCAGC
TP658_Hit	D	1	chr5	.	CAGCAAAAGCCTTTGTTAGGGGTAATAAAAACTATAAACATTGTTTTTTTTTTTTTATTTATTC
TP658_Query	D	1	chr5	.	CAGCAAAAGCCTTTGTTAGGGGTAATAAAAACTATAAACATTGTTTTTTTTTTCTTATTTATTC
TP66193_Hit	D	1	chr5	.	CTGCACCTATTTATTTCTAGTAGAACATTCATTTTGATGAACTGATTGTCAACTTTTATTTTTC
TP66193_Query	D	1	chr5	.	CTGCACCTATCTATTTCTAGTAGAACATTCATTTTGATGAACTGATTGTCAACTTTTATTTTTC
TP66247_Hit	D+G	1	chr5	.	CTGCACCTGAGCCGTTGGATACAATACTGCATCTGAATCCAACGACATTGCAACAGCTGAAAAA
TP66247_Query	D+G	1	chr5	.	CTGCACCTGAGCCGTTGGATACAATACTGCACCTGAATCCAACGACATTGCAACAGCTGAAAAA
TP66762_Hit	D+G	1	chr5	.	CTGCACTAGAGAATATGAGACTAGGCAGTGGTGATGGATCAGTTGGAATGTTCTCCTCACTTGT
TP66762_Query	D+G	1	chr5	.	CTGCACTAGAGAATATGAGACTAGGCAGTGGTGATGGATCAGTTGGAATGTTCTCCTCACTTAT
TP66990_Hit	D+G	1	chr5	.	CTGCACTGCACAGAAAGATCTGAGCAACTAGACTGGGATTCCCGGTTGAATATAATCATGGGTG
TP66990_Query	D+G	1	chr5	.	CTGCACTGCACAGAAAGATCTGAGCAACTAGACTGGGATTCACGGTTGAATATAATCATGGGTG
TP67118_Hit	D+G	1	chr5	.	CTGCACTTATTGCTTTGATCCGGTCAAAGCTTCTTGAAGAGTTCCAATAAATACTTGTTTCTGT
TP67118_Query	D+G	1	chr5	.	CTGCACTTATTGCTTTGATCCGGTCAAAGCTTCTTGAAGAGTTCCAATAAATACTCGTTTCTGT
TP67284_Hit	D+G	1	chr5	.	CTGCACTTTCTGTTGTTATGCCTGAACCACCTGCCGAATCACTAACATGGGTTCGTGGTGCTGA
TP67284_Query	D+G	1	chr5	.	CTGCACTTTCTGTTGTTATGCCTGAACCACCTGCCGAATCACTAACATGGGTCCGTGGTGCTGA
TP67397_Hit	D+G	1	chr5	.	CTGCACTTTTAATTCCTGATCTTCACCTTGAGTAGTATCTAGGTTGTTTTCATGCACATCAATT
TP67397_Query	D+G	1	chr5	.	CTGCACTTTTAATTCCTGATCTTCACCTTGAGTAGTATCTAGGTTGTTTTCATGCACATCAATC
TP67400_Hit	D	1	chr5	.	CTGCACTTTTTAGGCCAAAATTAATAGTTGCCGGTGCTAGTGCTTATGCCCGTCTTTATGATTA
TP67400_Query	D	1	chr5	.	CTGCACTTTTCAGGCCAAAATTAATAGTTGCCGGTGCTAGTGCTTATGCCCGTCTTTATGATTA
TP67512_Hit	D	1	chr5	.	CTGCAGAAAGTATGGTTCCTAAAGGACCTGAATGGGGCGGTGGAAACTGGGAAATCAAAACGCT
TP67512_Query	D	1	chr5	.	CTGCAGAAAGTATGGTTCCTAAAGGACCTGAATGGGGCGGTGGAAACTGGGAAATCAAAACCCT
TP67539_Hit	D	1	chr5	.	CTGCAGAACAAGCCAAGAAAAGAGAACAGGAGCGACAGAGGCAAGAAAAGCTTGCTGAAACTAG
TP67539_Query	D	1	chr5	.	CTGCAGAACAAGCCAAGAAAAGAGAACAGGAGCGACAGAGGCAAGAAAAACTTGCTGAAACTAG
TP67637_Hit	D	1	chr5	.	CTGCGGAAGGCGGGTATATGCGTGCTATGTACAATATATCCTTGTGCTACTCTTTTGGGGAAGG
TP67637_Query	D	1	chr5	.	CTGCAGAAGGCGGGTATATGCGTGCTATGTACAATATATCCTTGTGCTACTCTTTTGGGGAAGG
TP67689_Hit	D+G	1	chr5	.	CTGCAGAATCAACCATTCCAAATTCCGAATATTCAAAGTATGGTGCTGAAACTTTCGATGGGAT
TP67689_Query	D+G	1	chr5	.	CTGCAGAATCAACCATTCCAAATTCCGAATATTCAAAGTATGGAGCTGAAACTTTCGATGGGAT
TP67713_Hit	D+G	1	chr5	.	CTGCAGAATGGTGATTGTTCGAATATAGTGTTCATTGAGTTCTCAAAGGATTTACATGTAAGAA
TP67713_Query	D+G	1	chr5	.	CTGCAGAATGGTGATTGTTCGAATATAGTGTTCATCGAGTTCTCAAAGGATTTACATGTAAGAA

Name	Filter	Nb hit (Mt4.0)	Mt Chr (Mt4.0)	Ms Chr (Li et al., 2014)	Sequence
TP68089_Hit	D+G	1	chr5	.	CTGCAGATAGTTCCATATAATTCTTGGTTTCTACAATGCCATGTCGTTTATGTGGATATCATTT
TP68089_Query	D+G	1	chr5	.	CTGCAGATAGTTCCATATAATTCTTGGTTTATACAATGCCATGTCGTTTATGTGGATATCATTT
TP6814_Hit	D	1	chr5	.	CAGCAATAAGATGTGTTTATAATGGTTGGCTTTGTATGCAGGTATTGGTTGATTTCCAGGAAAA
TP6814_Query	D	1	chr5	.	CAGCAATAAGATGTGTTTATAATGGTTGGCTTTGCATGCAGGTATTGGTTGATTTCCAGGAAAA
TP6837_Hit	D	1	chr5	.	CAGCAATAATCAAGGCTCGGTCTTTGAAGAAGGCTAGTGAGGCTCGTGCATGGAAATTGACTTC
TP6837_Query	D	1	chr5	.	CAGCAATAATCAAGGCTCGGTCTTTGAAGAAGGCTAGCGAGGCTCGTGCATGGAAATTGACTTC
TP6874_Hit	D+G	1	chr5	.	CAGCAGTACAAGTTTTTCTCATCTTAGGTCTTCTAGCCACCTCTTGTTTTGCACAAGCACCAGG
TP6874_Query	D+G	1	chr5	.	CAGCAATACAAGTTTTTCTCATCTTAGGTCTTCTAGCCACCTCTTGTTTTGCACAAGCACCAGG
TP6882_Hit	D+G	1	chr5	.	CAGCAATACAGAAGATATTGTATGCTACTGATGACAATGGATTTGAGTTGGAAGAAGCTCAAGC
TP6882_Query	D+G	1	chr5	.	CAGCAATACAGAAGATATTGTATGCTACTGATGACAATGGATCTGAGTTGGAAGAAGCTCAAGC
TP6980_Hit	D	1	chr5	.	CAGCAATAGCAAGCATTTTAGCCGGAAGAGGCTGAGGTCGCAAGTTTTTAACAAAGGCTGAAAA
TP6980_Query	D	1	chr5	.	CAGCAATAGCAAGCATTTTAGCAGGAAGAGGCTGAGGTCGCAAGTTTTTAACAAAGGCTGAAAA
TP69903_Hit	D	1	chr5	.	CTGCAGTGTCTCAGTCTCCGCATGTTTCGTTTGTTTCAACTTCTGGCTATGACATACCTATAGA
TP69903_Query	D	1	chr5	.	CTGCAGTGTCTCAGTCACCGCATGTTTCGTTTGTTTCAACTTCTGGCTATGACATACCTATAGA
TP70183_Hit	D	1	chr5	.	CTGCAGTTTCCTTTTCTGATCTTCAACACTAAACTGATGAGATTCTTGTGGCGTAGCATCCTTC
TP70183_Query	D	1	chr5	.	CTGCAGTTTCCTTTTCTGATCTTCAACACTAAACTGATGAGATTCTTGCGGCGTAGCATCCTTC
TP70812_Hit	D	1	chr5	.	CTGCATATTATGTTCTTTCAAATGAGCCTGTTTTAAACATGGCTGTCGTTGAAGATAGTGCTTG
TP70812_Query	D	1	chr5	.	CTGCATATTATGTTCTTTCAAATGAGCCTGTTTTAAACATGGCTGTCGTTGAAGATAATGCTTG
TP7082_Hit	D+G	1	chr5	.	CAGCAATATAAAGCAATCTCTTTTAGGAATTTCTTGACGTACGTGACTTAGTGTTTTTTGGCTA
TP7082_Query	D+G	1	chr5	.	CAGCAATATAAAACAATCTCTTTTAGGAATTTCTTGACGTACGTGACTTAGTGTTTTTTGGCTA
TP7083_Hit	D	1	chr5	.	CAGCAATATAAAAGATTTTGAGATCAACCGCAGTTTTGAATACATCGACTGCATTTAACCGCGA
TP7083_Query	D	1	chr5	.	CAGCAATATAAAAGATTTCGAGATCAACCGCAGTTTTGAATACATCGACTGCATTTAACCGCGA
TP71089_Hit	D+G	1	chr5	.	CTGCATCATATGTTTTCCATGAGTGTACCTCAAAATAATGTGATATAATCAAAGACATAAAGGG
TP71089_Query	D+G	1	chr5	.	CTGCATCATATGTTTTCCATGAGTATACCTCAAAATAATGTGATATAATCAAAGACATAAAGGG
TP71280_Hit	D	1	chr5	.	CTGCATCCTCGCAATCTGCGTCTCAAGATCCGCCGCCCTAGCTGAAAAAAAAAAAAAAAAAAAA
TP71280_Query	D	1	chr5	.	CTGCATCCTCGCAATCTGCGTCTCAAGATCCGCCGCCCTAGCAGAAAAAAAAAAAAAAAAAAAA
TP71488_Hit	D	1	chr5	.	CTGCATCTGCTATAATAAACACATCAAATGTGATGTCGTGCAAGAATAAGGGAACTCCATTGTC
TP71488_Query	D	1	chr5	.	CTGCATCTGCTATAATAAACACATCAAATGTGATGTCGTGCAAGAACAAGGGAACTCCATTGTC
TP71501_Hit	D	1	chr5	.	CTGCATCTGGGGTTAAGGTTGATCCAACAACTATAGGAAAATATGCTCGTGGTGGTCAGCGGAT
TP71501_Query	D	1	chr5	.	CTGCATCTGGGGTTAAGGTTGATCCAACAACTATAGGAAAATATGCTCGTGGTGGTCAGCGAAT
TP71641_Hit	D	1	chr5	.	CTGCATGAATGCATGAATGGTTACATACCTTCTAGAGAACTTGAAAGGAGAAAGGACTCCAGAT
TP71641_Query	D	1	chr5	.	CTGCATGAATACATGAATGGTTACATACCTTCTAGAGAACTTGAAAGGAGAAAGGACTCCAGAT
TP72303_Hit	D+G	1	chr5	.	CTGCATTACTCACACTTCTTTGTCATTTTCCACAAAATATCAGGAATTATCACAAATATAGTTT
TP72303_Query	D+G	1	chr5	.	CTGCATTACTCACACTTCTTTGTAATTTTCCACAAAATATCAGGAATTATCACAAATATAGTTT
TP72381_Hit	D	1	chr5	.	CTGCATTATCCTTGTTTGTTCTTCCCTCACCAATTGCAATTCCTTCTCAGCATTTTCAAGAAAG
TP72381_Query	D	1	chr5	.	CTGCATTATCCTTATTTGTTCTTCCCTCACCAATTGCAATTCCTTCTCAGCATTTTCAAGAAAG
TP72527_Hit	D+G	1	chr5	.	CTGCATTTGTCATCGTCTGGCAGAAAAACAAAATCGACTCCAATTTTCTACAACCCTGGGAAAT
TP72527_Query	D+G	1	chr5	.	CTGCATTCGTCATCGTCTGGCAGAAAAACAAAATCGACTCCAATTTTCTACAACCCTGGGAAAT
TP72690_Hit	D	1	chr5	.	CTGCATTGCGTATCCAACGTGCCCGGAGAAGAAGAGATGATCCGGATGAAGATGTGGGTGCTGA
TP72690_Query	D	1	chr5	.	CTGCATTGCGTATCCAACGCGCCCGGAGAAGAAGAGATGATCCGGATGAAGATGTGGGTGCTGA
TP7309_Hit	D+G	1	chr5	.	CAGCAATCACAAAGACCGGTGGACCATTCAACTAGAGGTTTAGGCGGAGGAGGAGTAGGGGCGT
TP7309_Query	D+G	1	chr5	.	CAGCAATCACAAAGACCGGTGGACCATTCAACTAGAGGTTTAGGCGGAGGAGGAGGAGGGGCGT
TP73173_Hit	D	1	chr5	.	CTGCATTTTCTTTCTTTTTTGAAAGAGAGAGAGAGAAAAGGAACGTGCTGAAACGGCGACGGTG
TP73173_Query	D	1	chr5	.	CTGCATTTTCTTTCTTTTTTGAAAGAGAGAGAGAAAAAAGGAACGTGCTGAAACGGCGACGGTG
TP7336_Hit	D	1	chr5	.	CAGCAATCATAGAGGGTGACATAAATGTCCCTTGCCACATTTTTAGATGATAAAAAGGTAAAAG
TP7336_Query	D	1	chr5	.	CAGCAATCAGAGAGGGTGACATAAATGTCCCTTGCCACATTTTTAGATGATAAAAAGGTAAAAG
TP73788_Hit	D+G	1	chr5	.	CTGCCAAGTCCCTAGACGCAAACACAATGACTACATTCCGTGATCTTGTTCCTACAAAGCTTGC
TP73788_Query	D+G	1	chr5	.	CTGCCAAGTCCCTAGACGCAAACACAATGACTACATTCCGTGATCTTGTTCCTACAAAGCGTGC
TP73924_Hit	D	1	chr5	.	CTGCCAATGGTGAGCCAGATCTCACGGATTCTGCAGATGATTTTGTTGTCAATATATGTCGCCA
TP73924_Query	D	1	chr5	.	CTGCCAATGGCGAGCCAGATCTCACGGATTCTGCAGATGATTTTGTTGTCAATATATGTCGCCA
TP74073_Hit	D	1	chr5	.	CTGCCACAATGTTTTCCAAATCAAACTCACTGTCAATGTTGACAAACACGCCTGCTTCGGCTGA
TP74073_Query	D	1	chr5	.	CTGCCACAATGTTTTCCAAATCAAACTCACTGTCAATGTTGACAAACACGCCTGCTTCGGCAGA
TP7423_Hit	D	1	chr5	.	CAGCAATCCGTGAAGAAATAAATGATGTTGAGATAATTTACAAACCCCTTTCGGAAATGCTCGA
TP7423_Query	D	1	chr5	.	CAGCAATCCGTGAAGAAATAAATGATATTGAGATAATTTACAAACCCCTTTCGGAAATGCTCGA

Name	Filter	Nb hit (Mt4.0)	Mt Chr (Mt4.0)	Ms Chr (Li et al., 2014)	Sequence
TP74341_Hit	D	1	chr5	.	CTGCCGCCTACAGATTCCAGGTTAAGACCAGATCAGAGGTATTTGGAAAATGGGGAGTATGAGA
TP74341_Query	D	1	chr5	.	CTGCCACCTACAGATTCCAGGTTAAGACCAGATCAGAGGTATTTGGAAAATGGGGAGTATGAGA
TP74734_Hit	D	1	chr5	.	CTGCCAGCAACAAACTGTTGTTGGGGTTGATATTGAAGTTGGTGATACATTTGATTAGAACTAA
TP74734_Query	D	1	chr5	.	CTGCCAGCAACAAACTGTTGTTGAGGTTGATATTGAAGTTGGTGATACATTTGATTAGAACTAA
TP74765_Hit	D+G	1	chr5	.	CTGCCAGCATGTAGTGCAAGGTAGCAAAGGAGAGTTAGCGCATGATGCATCTGGTTAACTTCGT
TP74765_Query	D+G	1	chr5	.	CTGCCAGCATGTAGTGCAAGGTAGCAAAGGAGAATTAGCGCATGATGCATCTGGTTAACTTCGT
TP74857_Hit	D	1	chr5	.	CTGCCAGGTGCAACAAGTTATTGCCAAACGAGTCTATACGATGTCTAAGTATCTCCTTCCGTCC
TP74857_Query	D	1	chr5	.	CTGCCAGGTGCAACAAATTATTGCCAAACGAGTCTATACGATGTCTAAGTATCTCCTTCCGTCC
TP75027_Hit	D	1	chr5	.	CTGCCATAATATACAACAGAGACCATGCTTGGGATGACCATGTATGTCCTTTCACTTTGTTATG
TP75027_Query	D	1	chr5	.	CTGCCATAATATACAACAGAGACCATGCTTGGGATGACCATGTATGTCCTTCCACTTTGTTATG
TP75028_Hit	D	1	chr5	.	CTGCCATAATCATAACATGTCTTGCTCCGATTCGCCTTGCATTCTCAGCAATTCCTATTAGCCT
TP75028_Query	D	1	chr5	.	CTGCCATAATCATAACATGTCTTGCTCCCATTCGCCTTGCATTCTCAGCAATTCCTATTAGCCT
TP75076_Hit	D	1	chr5	.	CTGCCATAGATTTTACAATTCGCGTTGTGTCAGCTACAGAAACTTTGGAAGAAACAGTCTTCAC
TP75076_Query	D	1	chr5	.	CTGCCATAGATTTTACAATTCGCGTTGTGTCAGCTACAAAAACTTTGGAAGAAACAGTCTTCAC
TP75441_Hit	D	1	chr5	.	CTGCCATGGCAGAATAGCTGACAATCAGCACAAGAAATTTGTGAAGGGATACCTGCCGTGGTGC
TP75441_Query	D	1	chr5	.	CTGCCATGGCAGAATAGCTGACAATCAGCACAAGAAATTTGTGAAGGGATACCTGCCATGGTGC
TP76503_Hit	D	1	chr5	.	CTGCCCGGTCCATCGGTATACTTCTCTTGAGCACCATGGTACTTCTCAAGAGGTGGCTGTGTTG
TP76503_Query	D	1	chr5	.	CTGCCCGGTCCATCGGTATACTTCTCTTGAGCACCATGGTACTTCTCAAGAGGTGGCTATGTTG
TP76612_Hit	D	1	chr5	.	CTGCCCTTGAAGTGGAAGGCACTGAGCCAGAATCAACAACACCAAGCTCATCTACAGACATGCT
TP76612_Query	D	1	chr5	.	CTGCCCTAGAAGTGGAAGGCACTGAGCCAGAATCAACAACACCAAGCTCATCTACAGACATGCT
TP76959_Hit	D	1	chr5	.	CTGCCCTTTTGATCTTGCTTCGAGAAGCTTTGCTTTCAAAGTATTGTCTTTTGATCCTGAATGA
TP76959_Query	D	1	chr5	.	CTGCCCTTTTGATCTTGCTTCAAGAAGCTTTGCTTTCAAAGTATTGTCTTTTGATCCTGAATGA
TP77008_Hit	D	1	chr5	.	CTGCCGAAGCAGGCGTGTTTGTCAACATTGACAGTGAGTTTGATTTGGAAAACATTGTGGCTGA
TP77008_Query	D	1	chr5	.	CTGCCGAAGCAGGCGTGTTTGTCAACATTGACAGTGAGTTTGATTTGGAAAACATTGTGGCAGA
TP77100_Hit	D+G	1	chr5	.	CTGCCGAGATGAACTATACTAGAACTAGGGACAAGCCGAGTTTGATTAAGCGACATTACCGGCC
TP77100_Query	D+G	1	chr5	.	CTGCCGAGATGAACTATACTAGAACTAGGGACAAGCCGAGTTTGACTAAGCGACATTACCGGCC
TP7742_Hit	D	1	chr5	.	CAGCAATGCACAACAGAAAGTCTGATGTATACAGCTACGGAGTTGTTTTGCTCGAGCTAATAAC
TP7742_Query	D	1	chr5	.	CAGCAATGCACAACAGAAAGTCTGATGTATACAGCTACGGAGTTGTTTTACTCGAGCTAATAAC
TP77576_Hit	D	1	chr5	.	CTGCCGGACAAAATGTCAGTGCTTTGAATCTGCTAAGGAAGTCCTTGCACAAACACGAACGACC
TP77576_Query	D	1	chr5	.	CTGCCGGACAAAATGCCAGTGCTTTGAATCTGCTAAGGAAGTCCTTGCACAAACACGAACGACC
TP7759_Hit	D	1	chr5	.	CAGCAATGCATATTCAATTGTTCTAATTTTTCCATGTTTTGTGTTGTATGTCTTCTCTGAAGTT
TP7759_Query	D	1	chr5	.	CAGCAATGCATATTCAATTGTTCTAATTTTTCCAAGTTTTGTGTTGTATGTCTTCTCTGAAGTT
TP77985_Hit	D	1	chr5	.	CTGCCGTGTGTCAGACACTATCTCCCATTCTCCCTCTACTCGAACTTGTTTGTGTTGTGGTGGT
TP77985_Query	D	1	chr5	.	CTGCCGTGTGTCAGACACCATCTCCCATTCTCCCTCTACTCGAACTTGTTTGTGTTGTGGTGGT
TP7813_Hit	D+G	1	chr5	.	CAGCAATGCTGAAAGCGCCCTTCTGTACATCCCCAGAGGAAAAATAAAAGTTGACAATCAGTTC
TP7813_Query	D+G	1	chr5	.	CAGCAATGCTGAAAGCGCCCTTCTGCACATCCCCAGAGGAAAAATAAAAGTTGACAATCAGTTC
TP78157_Hit	D+G	1	chr5	.	CTGCCTACCTCAATCAACAGTAGCACTTCACAGTAGAGAGGTCATGATGGGGACGGTGCTGAAA
TP78157_Query	D+G	1	chr5	.	CTGCCTACCGCAATCAACAGTAGCACTTCACAGTAGAGAGGTCATGATGGGGACGGTGCTGAAA
TP78333_Hit	D+G	1	chr5	.	CTGCCTCAATAGCAATGTCTGTGCCAGCACCTATTGCCATTCCCACGTCTGCTGAAAAAAAAAA
TP78333_Query	D+G	1	chr5	.	CTGCCTCAATAGCAATGTCTGTGCCAGCACCTATTGCCATTCCCACATCTGCTGAAAAAAAAAA
TP78527_Hit	D+G	1	chr5	.	CTGCCTCCGGTGGTGTTTTTGATGGTATTGACGGTGGTGGTGGTTCTCCACCAGGTGCTGAAAA
TP78527_Query	D+G	1	chr5	.	CTGCCTCCGGTGGTGTTTCTGATGGTATTGACGGTGGTGGTGGTTCTCCACCAGGTGCTGAAAA
TP7887_Hit	D	1	chr5	.	CAGCAATGGCGGCAATGTCAGAACCTTTTGGCTCCTTCAACTTGACAATCGAGTCGAATATAAG
TP7887_Query	D	1	chr5	.	CAGCAATGGCGGCAATGTCAGAACCCTTTGGCTCCTTCAACTTGACAATCGAGTCGAATATAAG
TP7912_Hit	D	1	chr5	.	CAGCAATGGGTTGAATTATGGTATAACACTACTTTTCATGTCACTGCTCACTAGTATACAACAT
TP7912_Query	D	1	chr5	.	CAGCAATGGGTTGAATTATGGTATAACACTACTTTTCATGTCACTACTCACTAGTATACAACAT
TP79288_Hit	D	1	chr5	.	CTGCCTTCTGGGATTTCGAGAATGTGTGGGCTCATGGCGGAGTCGGAGGAGTGATTGATTATGA
TP79288_Query	D	1	chr5	.	CTGCCTTCTGGGATTTCGAGAATGTGTGGGCTCATGGCGGAGTCGGAGGAGGGATTGATTATGA
TP79493_Hit	D+G	1	chr5	.	CTGCCTTTCCTAGTTGGGCCAGCAGAAAAGAGAAATAACCATGCAAATAGGCCAGAAGATGACG
TP79493_Query	D+G	1	chr5	.	CTGCCTTTCCTAGTTGGGCCAGCAGAAAAGAAAAATAACCATGCAAATAGGCCAGAAGATGACG
TP79506_Hit	D	1	chr5	.	CTGCCTTTCTTTTTGATCCACGGCCCTTCTTTTTTGCAACCGGTTTACTCTCTTCTTCATCCGG
TP79506_Query	D	1	chr5	.	CTGCCTTTCTTTTTGATCCACGGCCCTTCTTTTTTGCAACCAGTTTACTCTCTTCTTCATCCGG
TP79667_Hit	D+G	1	chr5	.	CTGCGAAACTAACAAGTGTTCCAGGGTGGTCGGGTCGGATATGGGCAAGGACTGGTTGCACGTT
TP79667_Query	D+G	1	chr5	.	CTGCGAAACTAACAAGTGTTCCAGGGTGGTCGGGTCGGATATGGGCAAGGACTGGTTGCACATT

Name	Filter	Nb hit (Mt4.0)	Mt Chr (Mt4.0)	Ms Chr (Li et al., 2014)	Sequence
TP79847_Hit	D	1	chr5	.	CTGCGAAGTCTGGAGTTGTGGATAATCTCCAGGGCAATCTAGATGCATTGGCTTGGGGAAATTG
TP79847_Query	D	1	chr5	.	CTGCGAAGTCTGGAGTTGTGGATAATCTCCAGGGCAATCTAGATGCATTGGCTTGGGGAAACTG
TP80032_Hit	D	1	chr5	.	CTGCGACATTGGACTATTGTTAGGCGAGAAACTAACAGTCGAAGGAGAGTTGTAAACAACAAAA
TP80032_Query	D	1	chr5	.	CTGCGACATTGGACTATCGTTAGGCGAGAAACTAACAGTCGAAGGAGAGTTGTAAACAACAAAA
TP80080_Hit	D	1	chr5	.	CTGCGACGAACCACGCCTCTCTCATTCCTCGTCCTCCTCCACCTCAACCACCGCCTTCGAAGAC
TP80080_Query	D	1	chr5	.	CTGCGACGAACCACGCCTCTCTCATTCCTCGTCCTCCTCCACCTCAACCACCGCCCTCGAAGAC
TP8010_Hit	D	1	chr5	.	CAGCAATGTCTTAGCAAGTCCAAAATCAGCAACACGAGGAACAAAATCATGATCAAGCAATATG
TP8010_Query	D	1	chr5	.	CAGCAATGTCTTAGCAAGTCCAAAATCAGCAACACGAGGAACAAAATCATAATCAAGCAATATG
TP80114_Hit	D	1	chr5	.	CTGCGACGGAAGGACCAAACAGTTAATTTTGATTCTTGGGTTTTCTTTCACCCATTACAGTATT
TP80114_Query	D	1	chr5	.	CTGCGACGGAAGGACCAAACAGTTAATTTTGATTCTTGAGTTTTCTTTCACCCATTACAGTATT
TP80910_Hit	D	1	chr5	.	CTGCGCACTGTCCAAGCTTACAAGCACTACTACTTAGGCCATGTCTAGGTCATGTGGGACTAAA
TP80910_Query	D	1	chr5	.	CTGCGCACTGTCCAAACTTACAAGCACTACTACTTAGGCCATGTCTAGGTCATGTGGGACTAAA
TP81339_Hit	D	1	chr5	.	CTGCGCGAACCACCGCTCTCGATGAGAGTACTCTCTTGCAAGCTTCCAAATAGGTTGACAATAG
TP81339_Query	D	1	chr5	.	CTGCGCGAACCACCGCTCTCGATGAGAGTACTCTCTTGCAAACTTCCAAATAGGTTGACAATAG
TP81467_Hit	D	1	chr5	.	CTGCGCGGCTGTTATTGCTTTTGGAGGTGTTTTCTTTGCTTTAGGGAGTTTTTATTCTCTGTTA
TP81467_Query	D	1	chr5	.	CTGCGCGGCTGTTATTGCTTTTGGAGGTGTTTTCTTTGCGTTAGGGAGTTTTTATTCTCTGTTA
TP81800_Hit	D	1	chr5	.	CTGCGCTTTGATGATTGTGCTTATCAATACGAAAGTGGATTTCCACTCGGTAAATAAAACAAAT
TP81800_Query	D	1	chr5	.	CTGCGCTTTGATGAGTGTGCTTATCAATACGAAAGTGGATTTCCACTCGGTAAATAAAACAAAT
TP81867_Hit	D+G	1	chr5	.	CTGCGGAAGTTGTCAATGTTATAGCGGGCATACTTCCTGCCCTGGTTCAGTGGAACACGATTGA
TP81867_Query	D+G	1	chr5	.	CTGCGGAAGTTGTCAATCTTATAGCGGGCATACTTCCTGCCCTGGTTCAGTGGAACACGATTGA
TP81890_Hit	D	1	chr5	.	CTGCGGACAAAATGTAAAGGATTTATAAGGTTTTGCAACCACTTAGTGGGTGTAATTGTGGTTG
TP81890_Query	D	1	chr5	.	CTGCGGACAAAATGTAAAGGATTTATAAGGTTTTGCAACCACTTAGTGGGTGTAATTGTAGTTG
TP81956_Hit	D+G	1	chr5	.	CTGCGGAGGAATCAAAGGAAGCTATTGCTGATGCCCTTAAAGGATCAGATTTGGTGTTCATAAC
TP81956_Query	D+G	1	chr5	.	CTGCGGAGGAATCAAAAGAAGCTATTGCTGATGCCCTTAAAGGATCAGATTTGGTGTTCATAAC
TP82206_Hit	D	1	chr5	.	CTGCGGCCTGTTTTTTCCTTATTGAAGATTTAGTCCTCTTAACTTGTGTAATTGGTACTAGTGT
TP82206_Query	D	1	chr5	.	CTGCGGCCTGTTTTTTCCTTACTGAAGATTTAGTCCTCTTAACTTGTGTAATTGGTACTAGTGT
TP82714_Hit	D	1	chr5	.	CTGCGGTCATTTGGAAGAGATTTCTATTGAGTATTTTGCCACAGATGAGCTCCTTGAATGCATA
TP82714_Query	D	1	chr5	.	CTGCGGTCATTTAGAAGAGATTTCTATTGAGTATTTTGCCACAGATGAGCTCCTTGAATGCATA
TP83107_Hit	D	1	chr5	.	CTGCGTATCATAATTTGAATGAACCGCTATTTTCCGCAATTGACAACAATGTAGCTATTACTAT
TP83107_Query	D	1	chr5	.	CTGCGTAGCATAATTTGAATGAACCGCTATTTTCCGCAATTGACAACAATGTAGCTATTACTAT
TP83202_Hit	D	1	chr5	.	CTGCGTCAACATGATAGAAACGCCTTGCCAGAACCATTCATACATCATCACTGCCTCATTCAAT
TP83202_Query	D	1	chr5	.	CTGCGTCAACATGATAGAAACGCCTTGCCAGAACCATCCATACATCATCACTGCCTCATTCAAT
TP83238_Hit	D	1	chr5	.	CTGCGTCAGGAACTGGTGAGGATTTACAAAATATACCTTGTATATAACTTTTATTTCTCAATCT
TP83238_Query	D	1	chr5	.	CTGCGTCAGGAACTGGTGAGGATTTACAAAATATACCTTGTAGATAACTTTTATTTCTCAATCT
TP83271_Hit	D	1	chr5	.	CTGCTTCCAACAAATTTGAAGAGGCAATAGAGCAAAGGCTTTTAATGAAGGAAAACTTGACAGA
TP83271_Query	D	1	chr5	.	CTGCGTCCAACAAATTTGAAGAGGCAATAGAGCAAAGGCTTTTAATGAAGGAAAACTTGACAGA
TP83316_Hit	D	1	chr5	.	CTGCGTCGAAGGGTTCGGCTCTTGGTATGGTGGATGCTGGGCTTATTCATTGGGAGAAAGGAGA
TP83316_Query	D	1	chr5	.	CTGCGTCGAAGGGTTCGGCTCTTGCTATGGTGGATGCTGGGCTTATTCATTGGGAGAAAGGAGA
TP83717_Hit	D+G	1	chr5	.	CTGCGTTAACCATGTAATAAATATCACGCGCATTTGGGAATAGTGGCATACGGTGTTTGTACGA
TP83717_Query	D+G	1	chr5	.	CTGCGTTAACCATGTAATAAATATCACGCACATTTGGGAATAGTGGCATACGGTGTTTGTACGA
TP83904_Hit	D+G	1	chr5	.	CTGCGTTGTCAATTACGAATCACGGAAAACAATGGTTTGTTCAAATTCTGCTTCACTACAGTGC
TP83904_Query	D+G	1	chr5	.	CTGCGTTGTCAATTACAAATCACGGAAAACAATGGTTTGTTCAAATTCTGCTTCACTACAGTGC
TP83905_Hit	D	1	chr5	.	CTGCGTTGTCAATTGCGAATCACTGAAAACAATGGTTTGTTCAAATTCTGCTTCGCTACAGTGC
TP83905_Query	D	1	chr5	.	CTGCGTTGTCAATTGCGAATCACGGAAAACAATGGTTTGTTCAAATTCTGCTTCGCTACAGTGC
TP84546_Hit	D+G	1	chr5	.	CTGCTAATATGGAGTTCACAGGAAGCATCCCACAGGGTTTCCAAGTTGATTGGGAAGCCATTTT
TP84546_Query	D+G	1	chr5	.	CTGCTAATATGGAGTTCACAGGAAGCACCCCACAGGGTTTCCAAGTTGATTGGGAAGCCATTTT
TP84588_Hit	D	1	chr5	.	CTGCTAATCTCAAAGATCCTATTGTCAACCTATTTGGAAGTTTGCAAGAGAGTACTCTCATCGA
TP84588_Query	D	1	chr5	.	CTGCTAATCTCAAAGATCCTATTGTCAACCTATTTGGAAGCTTGCAAGAGAGTACTCTCATCGA
TP84827_Hit	D	1	chr5	.	CTGCTACACCTTCACTCTTGGCACTCATTCGACCACCTCCAAAGCCACCATGGAGTTTCTTTTT
TP84827_Query	D	1	chr5	.	CTGCTACACCTTCACTCTAGGCACTCATTCGACCACCTCCAAAGCCACCATGGAGTTTCTTTTT
TP84873_Hit	D	1	chr5	.	CTGCTACAGGACTTCCAAGCGTTGCATTTTTCAACACAATCGCAGAAGTCAAGGTGATCTTGCT
TP84873_Query	D	1	chr5	.	CTGCTACAGGACTTCCAAGCGTTGCATTTTTCAACACAATCGCAGAAGTCAAGGTGATCTTGAT
TP84900_Hit	D	1	chr5	.	CTGCTACATACATTATTTGAGTGGTGAAGTTCACTCTCATTATCTTTTCAATCTTTTCTTTAGT
TP84900_Query	D	1	chr5	.	CTGCTACATACATTATTTGAGTGGCGAAGTTCACTCTCATTATCTTTTCAATCTTTTCTTTAGT

Name	Filter	Nb hit (Mt4.0)	Mt Chr (Mt4.0)	Ms Chr (Li et al., 2014)	Sequence
TP84999_Hit	D	1	chr5	.	CTGCTACCATGTATTTGCCACTTGCACGGTTGGTTTTGAGAAGTACATTTGTTGTTTGTCCTGG
TP84999_Query	D	1	chr5	.	CTGCTACCATGTATTTGCCACTTGCACGGTTGGCTTTGAGAAGTACATTTGTTGTTTGTCCTGG
TP85273_Hit	D	1	chr5	.	CTGCTACTGCGGCTGATGAATCAAATCCATATGGTGAGGGCTTCCTCACTGGAGAGGAAGGCAT
TP85273_Query	D	1	chr5	.	CTGCTACTGCGGCTGACGAATCAAATCCATATGGTGAGGGCTTCCTCACTGGAGAGGAAGGCAT
TP85386_Hit	D	1	chr5	.	CTGCTACTTGAATAGACAATAAAAGACACATTTTATCTCTCCTTTGTTTGTTCTCCTCTGTTTT
TP85386_Query	D	1	chr5	.	CTGCTACTTGAATAGACAATAAAAGACACATTTTATCTCTCATTTGTTTGTTCTCCTCTGTTTT
TP85416_Hit	D	1	chr5	.	CTGCTACTTTGAATTTCTCTTGGTGGGCCCTTACTGTTGGGCTTTTTTGTTACACTGTTTATGG
TP85416_Query	D	1	chr5	.	CTGCTACTTTGAATTTCTCTTGGTGGGCCCTTACTGTAGGGCTTTTTTGTTACACTGTTTATGG
TP85427_Hit	D	1	chr5	.	CTGCTAGAAACAGACAATGAGAAAAACCTAAATACATATAATTACAATGAGTCCCCAAAAAACT
TP85427_Query	D	1	chr5	.	CTGCTAGAAACAGACAATGAGAAAAACCTAAATACACATAATTACAATGAGTCCCCAAAAAACT
TP8551_Hit	D+G	1	chr5	.	CAGCTATTTCATTCCGTGGGGTCAATGCCATTACAAACTTCCCAATACCTTACAATGCTTCTAC
TP8551_Query	D+G	1	chr5	.	CAGCAATTTCATTCCGTGGGGTCAATGCCATTACAAACTTCCCAATACCTTACAATGCTTCTAC
TP85561_Hit	D	1	chr5	.	CTGCTAGATGATGAGTGCCCTGAAGATTTTATCAAAGGTTTGATTCTTTCTGTTCGTTCCTTGC
TP85561_Query	D	1	chr5	.	CTGCTAGATGATGAGTGCCCTGAAGATTTTATCAAAGGTTTGATTCTTTCTGTTCGTTCCGTGC
TP85689_Hit	D	1	chr5	.	CTGCTAGCTTGGTTCAAGGAGTTTATGTTCTAGAAAAAGACCGACAAGAACAGCGGAAAGGTCC
TP85689_Query	D	1	chr5	.	CTGCTAGCTTGGTTCAAGGAGTTTATGTTCTAGAAAAAGACCGACAAGAACAGAGGAAAGGTCC
TP857_Hit	D	1	chr5	.	CAGCAAAATATTATTACTTTATGCTGGTGAATGTTTTCTTGGGAAGCATTATAACTGGAACTGC
TP857_Query	D	1	chr5	.	CAGCAAAATATTATTACTTTATGCTGGTGAATGTTTTCTTGGGAAGCATAATAACTGGAACTGC
TP85720_Hit	D	1	chr5	.	CTGCTAGGCATAGAAGGGAAAGGATAAGTGAGAGGATAAGGATATTGCAAAGATTAGTACCTGG
TP85720_Query	D	1	chr5	.	CTGCTAGGCATAGAAGGGAAAGGATAAGTGAGAGGATAAGGATATTACAAAGATTAGTACCTGG
TP85736_Hit	D	1	chr5	.	CTGCTAGGGAGAAGATTAAGCGCCGTGGTGACACCCAGCCGCTAGCTGAAAAAAAAAAAAAAAA
TP85736_Query	D	1	chr5	.	CTGCTAGGGAGAAGATTAAGCGCCGTGGTGACACCCAGCCGCTAGCAGAAAAAAAAAAAAAAAA
TP8580_Hit	D+G	1	chr5	.	CAGCAATTTCTGATTGACCAAGAGTTTCGTGAATTTAGTATTGGACGGTAGACAGATATACATT
TP8580_Query	D+G	1	chr5	.	CAGCAATTTCTGAGTGACCAAGAGTTTCGTGAATTTAGTATTGGACGGTAGACAGATATACATT
TP86016_Hit	D+G	1	chr5	.	CTGCTATAATTGGAACAGTTGGAGCTGGTTTTTTTGTTGAGCTAGTTGGTTCATTCCGAGGATT
TP86016_Query	D+G	1	chr5	.	CTGCTATAATTGGAACAGTTGGAGCTGGATTTTTTGTTGAGCTAGTTGGTTCATTCCGAGGATT
TP86060_Hit	D	1	chr5	.	CTGCTATAGAATCTAGTTTATTTTAATATACATTCACTGTGTTGCATGTGGTACATGCTCTTAT
TP86060_Query	D	1	chr5	.	CTGCTATAGAATCTAGTTTATTTCAATATACATTCACTGTGTTGCATGTGGTACATGCTCTTAT
TP8635_Hit	D	1	chr5	.	CAGCAATTTTAACAACTCAATTATTATGTAGGATCTGGGATTTATGGTAAGGTATTGAATGTTT
TP8635_Query	D	1	chr5	.	CAGCAATTTTAACAACTCAATTATTATGTAGGATCTGGGATTTATGGGAAGGTATTGAATGTTT
TP86768_Hit	D+G	1	chr5	.	CTGCTATTGCTCCGCTTTAAAGGAACAACCGTGTATGAGGCATGTATTTCATTTCTTTGTTTCA
TP86768_Query	D+G	1	chr5	.	CTGCTATTGCTCCGCTTTAAAGGAACAACCGTGTACGAGGCATGTATTTCATTTCTTTGTTTCA
TP86772_Hit	D+G	1	chr5	.	CTGCTATTGCTGGAATTGTTGTGTTGCTTTTTGGAAACATCAGTGATGCTGGTGGTGCTGGTGC
TP86772_Query	D+G	1	chr5	.	CTGCTATTGCTGGAATTGTTGTGTTGCTATTTGGAAACATCAGTGATGCTGGTGGTGCTGGTGC
TP86776_Hit	D	1	chr5	.	CTGCTATTGGAATGCCAAGCAAATTATAACCCAACGCCCAAACATAATTGAGGCGTATACGAAA
TP86776_Query	D	1	chr5	.	CTGCTATTGGAATGCCAAGCAAATTATAACCCAAAGCCCAAACATAATTGAGGCGTATACGAAA
TP88212_Hit	D	1	chr5	.	CTGCTCCTGGTGCTCCTGTCCCCACACCCCCACCTTGATATGTACCCCTCCCTGCTGAAAAAAA
TP88212_Query	D	1	chr5	.	CTGCTCCTGGTGCTCCTGTCCCCACACCCCCACCTTGATATGTACCCCTCCCTGCAGAAAAAAA
TP8874_Hit	D	1	chr5	.	CAGCACAACAACCTGTCGTCTTGCCATTGTTGTGTTTCTCAGTACAGTTGATGATGTCGAAGAA
TP8874_Query	D	1	chr5	.	CAGCACAACAACCTGTCATCTTGCCATTGTTGTGTTTCTCAGTACAGTTGATGATGTCGAAGAA
TP88748_Hit	D	1	chr5	.	CTGCTCTAGCCGTAATTTTTCTGAATTTCCCATCTCATACTCCCCATTTTCCAAATACCTTTGA
TP88748_Query	D	1	chr5	.	CTGCTCTAGCCGTAATTTTTCTGAATTTCCCATCTCATACTCCCCATTTTCCAAATACCTCTGA
TP88876_Hit	D	1	chr5	.	CTGCTCTCCGCAGAAACTTGGCTTCTGAACCAGCTAAAGGTAAACAATTTGAACCAGGTACTAG
TP88876_Query	D	1	chr5	.	CTGCTCTCCGCAGAAACTTGGCTTCTGAACCAGCTAAAGGTAAACAATTTGAACCAGGTAATAG
TP89145_Hit	D+G	1	chr5	.	CTGCTCTTATAAATGTTGGAGAACCGACAAATGATGGGATTCTTGGAACTACAAGTGCTCCTAT
TP89145_Query	D+G	1	chr5	.	CTGCTCTTATAAATGTTGGAGAACCGACAAATGATAGGATTCTTGGAACTACAAGTGCTCCTAT
TP89409_Hit	D	1	chr5	.	CTGCTGAAAAAGATAATGCGTTAGAAAGTGATAAAGCACCAACCAGTTCGCCTCAAGTACCTGC
TP89409_Query	D	1	chr5	.	CTGCTGAAAAAGATAATGCATTAGAAAGTGATAAAGCACCAACCAGTTCGCCTCAAGTACCTGC
TP90268_Hit	D+G	1	chr5	.	CTGCTGATGTTGGTAGTTATAGTAGGGCTCATGCTTCTGTTGCTCAGTATGGAGGACAATATAG
TP90268_Query	D+G	1	chr5	.	CTGCTGATGTTGGTAGTTACAGTAGGGCTCATGCTTCTGTTGCTCAGTATGGAGGACAATATAG
TP9032_Hit	D	1	chr5	.	CAGCACAAGCTCGTAGTACCCCTGTAAGGAAGTTTTCAGGGGCTTCAAAGTCTCCTTCAAGCAG
TP9032_Query	D	1	chr5	.	CAGCACAAGCTCGCAGTACCCCTGTAAGGAAGTTTTCAGGGGCTTCAAAGTCTCCTTCAAGCAG
TP90378_Hit	D	1	chr5	.	CTGCTGGAACTGTTTTGGGAATTTAATGAATGGTTTGTAGTCAGAGGAGCTGACTGGGAAGAAT
TP90378_Query	D	1	chr5	.	CTGCTGGAACTGTTTTGGGAATTTAATGAATGGTTTGTAGTCAAAGGAGCTGACTGGGAAGAAT

Name	Filter	Nb hit (Mt4.0)	Mt Chr (Mt4.0)	Ms Chr (Li et al., 2014)	Sequence
TP90449_Hit	D	1	chr5	.	CTGCTGGTCTGAACCCTATTGCTGGATTAGTGTTTGAGATTATCATAACATTTGGATTGGTTTA
TP90449_Query	D	1	chr5	.	CTGCTGGACTGAACCCTATTGCTGGATTAGTGTTTGAGATTATCATAACATTTGGATTGGTTTA
TP90522_Hit	D+G	1	chr5	.	CTGCTGGAGTTGGTGCGGGAACTGTCGGTGCTTTGGGAGCATTAGCATTAGGAGCTACAGGTCC
TP90522_Query	D+G	1	chr5	.	CTGCTGGAGTTGGCGCGGGAACTGTCGGTGCTTTGGGAGCATTAGCATTAGGAGCTACAGGTCC
TP90814_Hit	D+G	1	chr5	.	CTGCTGGGACTATTGGCACAATGGAGAAAGTGAGGAGAGAGATACAAGTGACCATGCTCATCCT
TP90814_Query	D+G	1	chr5	.	CTGCTGGGACTATTGGCACAATGGAGAAAGTGAGGACAGAGATACAAGTGACCATGCTCATCCT
TP91111_Hit	D	1	chr5	.	CTGCTGGTGCTCGTGCTGGTGAAGATTTTCATAGTGGCTGTGATTCATCTACATCTGTTGTTGA
TP91111_Query	D	1	chr5	.	CTGCTGGTGCTCGTGCTGGTGAAGATTTTCACAGTGGCTGTGATTCATCTACATCTGTTGTTGA
TP9125_Hit	D	1	chr5	.	CAGCACAATCAACAAAACAGCAATAACCCAATATACCCATTTCCTGTTCCTCTTCTTGATCTGG
TP9125_Query	D	1	chr5	.	CAGCACAATCAACAAAACAGCAATAACCCAATAGACCCATTTCCTGTTCCTCTTCTTGATCTGG
TP91371_Hit	D+G	1	chr5	.	CTGCTGTAACTGAAGAAAGGACATTCGCTGACCACCACGAGCATATTTTCCTATAGTTGTTGGA
TP91371_Query	D+G	1	chr5	.	CTGCTGTAACTGAAGAAAGGACATCCGCTGACCACCACGAGCATATTTTCCTATAGTTGTTGGA
TP91735_Hit	D	1	chr5	.	CTGCTTTCGCCGCTGAGTTTCAACCCTAAGCATTGCAATATCTGAATTAATATCTCTAGCGGTC
TP91735_Query	D	1	chr5	.	CTGCTGTCGCCGCTGAGTTTCAACCCTAAGCATTGCAATATCTGAATTAATATCTCTAGCGGTC
TP9187_Hit	D	1	chr5	.	CAGCACAATTCTTTAGGATTGGATGGAAAATGGTTATGCATGGTGATGATACGTGGCTGAAGTT
TP9187_Query	D	1	chr5	.	CAGCACAATTCTTTAAGATTGGATGGAAAATGGTTATGCATGGTGATGATACGTGGCTGAAGTT
TP91878_Hit	D	1	chr5	.	CTGCTGTGATAACGACCATTTCCTATTTATTCTTTTCCAGGCTAAATCAAATGCCATGAAGGGT
TP91878_Query	D	1	chr5	.	CTGCTGTGATAACGACCATTTCCTATTTATTCTTTTCCAGGCTAAATCAAATGCCATCAAGGGT
TP92045_Hit	D+G	1	chr5	.	CTGCTGTGTGATTCACAACATCTGTTTCCTTCTCAAAAGGACGTCAATAGGACAATCCAAGGTT
TP92045_Query	D+G	1	chr5	.	CTGCTGTGTGATTCACAACATCTGTTCCCTTCTCAAAAGGACGTCAATAGGACAATCCAAGGTT
TP9218_Hit	D	1	chr5	.	CAGCACACAACGTGCTTGAAGCTTGTGCTCAAACAGAAACAATAGATAAAGTTGTGTTCACATC
TP9218_Query	D	1	chr5	.	CAGCACACAACGTGCTTGAAGCTTGTGCTCAAACAGAAACAATAGATAAAGTCGTGTTCACATC
TP92327_Hit	D+G	1	chr5	.	CTGCTGTTGACGACGAGGACTATCCAGACCGAGCAATTCCATCTCAAGATCGAGATCCCTTGCA
TP92327_Query	D+G	1	chr5	.	CTGCTGTTGACGACGAGGACTATCCAGACCGAGCAATTCCATCTCAAGATCGAGATCCCTCGCA
TP92395_Hit	D	1	chr5	.	CTGCTGTTGCTCCTCCTGCCATTTGTGGTGGCCCTCCTGCCGGAACACCGCCGTACGCCGCTGA
TP92395_Query	D	1	chr5	.	CTGCTGTTGCTCCTCCTGCCATTTGTGGTGGCCCTCCTGCCGGAACACCACCGTACGCCGCTGA
TP92464_Hit	D+G	1	chr5	.	CTGCTGTTGTCATTCTTGTTTCATTTACTTCAATGATGTTACGGACCTAATAAAGCTCTTTTAT
TP92464_Query	D+G	1	chr5	.	CTGCTGTTGTCATTCTTCTTTCATTTACTTCAATGATGTTACGGACCTAATAAAGCTCTTTTAT
TP9279_Hit	D	1	chr5	.	CAGCACACCTCTGCTACATCATCATCTACACTTCCATGCAATGATTCTGATAAGGTTAATTGAG
TP9279_Query	D	1	chr5	.	CAGCACACATCTGCTACATCATCATCTACACTTCCATGCAATGATTCTGATAAGGTTAATTGAG
TP92899_Hit	D	1	chr5	.	CTGCTTAGACAGCAAAAAGTCACAAGCTTTACGGATGCTTAAGCAATTACTGAAACTCTTCCCA
TP92899_Query	D	1	chr5	.	CTGCTTAGACAGCAAAAAGTCACAAGCTTTACGGATGCTTAAGCAATTACTGAAACTCTTACCA
TP93419_Hit	D	1	chr5	.	CTGCTTCCACAAGAGAAACTCTTAATCTACAAGACCAACTTTCTCAGGCTTACAGAATTAAAGC
TP93419_Query	D	1	chr5	.	CTGCTTCCACAACAGAAACTCTTAATCTACAAGACCAACTTTCTCAGGCTTACAGAATTAAAGC
TP93578_Hit	D	1	chr5	.	CTGCTTCCTGAATCAATCATTTGGATTTTCTGTATTGTTAGAAAGCCCATTAGCTAAAATGAAG
TP93578_Query	D	1	chr5	.	CTGCTTCCTGAATCAATCACTTGGATTTTCTGTATTGTTAGAAAGCCCATTAGCTAAAATGAAG
TP93595_Hit	D	1	chr5	.	CTGCTTCCTTGGATGATCTCGAGGAGAATGCGATAAATCGTTGATTGTTCAGGATCATGTCATC
TP93595_Query	D	1	chr5	.	CTGCTTCCTTGGAAGATCTCGAGGAGAATGCGATAAATCGTTGATTGTTCAGGATCATGTCATC
TP93633_Hit	D	1	chr5	.	CTGCTTCGCCCTCTCGGTGATAAATTATTCATCGCTGAAAAAAAAAAAAAAAAAAAAAAAAAAA
TP93633_Query	D	1	chr5	.	CTGCTTCGCCCTCTCGGTGATAAATTATTCATCGCAGAAAAAAAAAAAAAAAAAAAAAAAAAAA
TP93750_Hit	D+G	1	chr5	.	CTGCTTCTCAAGTGGGACAGTTCATGAAGTTTGAAGACAAGTCTGAAACTTCATCTAGCCATGG
TP93750_Query	D+G	1	chr5	.	CTGCTTCTCAAGTGGGACAGTTCATGAAGTTTGAAGACAAGTCTGAAACTTCATCCAGCCATGG
TP93768_Hit	D	1	chr5	.	CTGCTTCTCCGGTCCAGTAGACCGTTGAAAACAAACCGTATCACCGGCTTTCAGATTTTTCTCC
TP93768_Query	D	1	chr5	.	CTGCTTCTCCGGTCCAGTAGACCGTTGAAAACAAACCGTATCACCGGCTTTCAGATTCTTCTCC
TP9390_Hit	D	1	chr5	.	CAGCACACTTGGTTTTACTTTTGCTGTTTTGATGATTGATACAAGCACTCGACTCAAATAGAAG
TP9390_Query	D	1	chr5	.	CAGCACACTCGGTTTTACTTTTGCTGTTTTGATGATTGATACAAGCACTCGACTCAAATAGAAG
TP93987_Hit	D	1	chr5	.	CTGCTTCTTTAGTTTCTCTTTTTGAGGTATGCCCTTAAATCCTTAGCAAAATTAATTAGAGTTT
TP93987_Query	D	1	chr5	.	CTGCTTCTTTAGTTTCTCTTTTTGAGGTATGCCCTTAAATACTTAGCAAAATTAATTAGAGTTT
TP93994_Hit	D	1	chr5	.	CTGCTTCTTTGACCGGTTTTCGGTAGTAGTTCTATGTGGATTTTAGCATTGTTTCAGAATTGTG
TP93994_Query	D	1	chr5	.	CTGCTTCTTTGACCGGTTCTCGGTAGTAGTTCTATGTGGATTTTAGCATTGTTTCAGAATTGTG
TP94007_Hit	D+G	1	chr5	.	CTGCTTCTTTTCCTGCTAATCTTCCCGCTAGAAAACTCATTTTTCCAGATTATTATTGTTTGTT
TP94007_Query	D+G	1	chr5	.	CTGCTTCTTTTCCTGCTAACCTTCCCGCTAGAAAACTCATTTTTCCAGATTATTATTGTTTGTT
TP9429_Hit	D	1	chr5	.	CAGCACTGAAACAACGACTCTAGCGTGTAAACACGACTGTTACTTCAACTTCCGGCGCCGATTA
TP9429_Query	D	1	chr5	.	CAGCACAGAAACAACGACTCTAGCGTGTAAACACGACTGTTACTTCAACTTCCGGCGCCGATTA

Name	Filter	Nb hit (Mt4.0)	Mt Chr (Mt4.0)	Ms Chr (Li et al., 2014)	Sequence
TP94314_Hit	D	1	chr5	.	CTGCTTGCGTCCAAATGGGTACCCATGAAAGCTTTAACTATGCTCTCAATGCTTTCAAGGAAGA
TP94314_Query	D	1	chr5	.	CTGCTTGCGTCCAAATGGGTACCCATGAAAGCTTAAACTATGCTCTCAATGCTTTCAAGGAAGA
TP94455_Hit	D	1	chr5	.	CTGCTTGGTGCAACGGATTCTGTGACTGAGCTGACAGAAGAGAGAGAGCATGGCTAGATAATTC
TP94455_Query	D	1	chr5	.	CTGCTTGGTGCAACGGATTCTGTGACTGAGCTGACAGAAGAGAGAGAGCATGGCTAGATAACTC
TP94603_Hit	D	1	chr5	.	CTGCTTGTGGCCTTAGACCTGAAGTTCCCAAGGACTGCCAACCAAATCTAAAATACATTATGAC
TP94603_Query	D	1	chr5	.	CTGCTTGTGGCCTTAGACCTGAAGTTCCCAAGGACTGCCAACCAAACCTAAAATACATTATGAC
TP94699_Hit	D	1	chr5	.	CTGCTTGTTTGTCGGATATTGGTGAAAAAAAGACAAAATATACTCGGAGAAGAAAGGGATATGA
TP94699_Query	D	1	chr5	.	CTGCTTGTTTGTCGGATATTGGTGAAAAAAACACAAAATATACTCGGAGAAGAAAGGGATATGA
TP94851_Hit	D	1	chr5	.	CTGCTTTATCCGGGTAAATTTTACATTGAAAGTAAAAACAAATTACTTGAATTACTCGCATGGT
TP94851_Query	D	1	chr5	.	CTGCTTTATCCGGGTAAAATTTACATTGAAAGTAAAAACAAATTACTTGAATTACTCGCATGGT
TP95425_Hit	D	1	chr5	.	CTGCTTTTATTGTTGTTATTTTTATTAGCTTTAAGATTTTCTGTTTTCTTTACTCAGAACTACA
TP95425_Query	D	1	chr5	.	CTGCTTTTATTGTTGTTATTTTTATTAGCTTTAAGATTTTCCGTTTTCTTTACTCAGAACTACA
TP9543_Hit	D	1	chr5	.	CAGCATAGGAACCTATTTCATGAAAGTCAAGTGCAATTATGATGAAAAAATTGATATGCATATG
TP9543_Query	D	1	chr5	.	CAGCACAGGAACCTATTTCATGAAAGTCAAGTGCAATTATGATGAAAAAATTGATATGCATATG
TP9639_Hit	D+G	1	chr5	.	CAGCACATAAATACAAACACAAATTTTGTGCACCTACGAGGAAACTCACTTCCAAAGAGTTTCA
TP9639_Query	D+G	1	chr5	.	CAGCACATAAATACAAACACAAATTTTGTGCACCTACGAGGAAACTCACTTCCAAAGAGTTTAA
TP9670_Hit	D+G	1	chr5	.	CAGCACATACTTGTCTTCCCACTCGTTCCGGCTTAATCCAAAAAGGTTTCCCTTGCTCCGATAC
TP9670_Query	D+G	1	chr5	.	CAGCACATACTTGTCTTCCCACTCGTTCCGGCTTAATCCAAAAAGGTGTCCCTTGCTCCGATAC
TP9697_Hit	D	1	chr5	.	CAGCACATATCGATTATAGAGATTAAGTATGGATCAAATAACATCACATGGACATTCACTTCCT
TP9697_Query	D	1	chr5	.	CAGCACATATCGATTATAGAGATCAAGTATGGATCAAATAACATCACATGGACATTCACTTCCT
TP9812_Hit	D+G	1	chr5	.	CAGCACATGCAAGGTGTAAATCTTCATCTTGACGAATTTCCATTCCCCCAGTAAAGCTAACAAT
TP9812_Query	D+G	1	chr5	.	CAGCACATGCAAGGTGTAAATCTTCATCTTGAAGAATTTCCATTCCCCCAGTAAAGCTAACAAT
TP9862_Hit	D+G	1	chr5	.	CAGCACATGTTTATAAACGCTATCTTAGACCATTCTACATGAACCCACAATTACCACAAATGCC
TP9862_Query	D+G	1	chr5	.	CAGCACATGTATATAAACGCTATCTTAGACCATTCTACATGAACCCACAATTACCACAAATGCC
TP27446_Hit	D+G	1	chr5	1A	CAGCCATTGGTAAGGGAACATGCATTCCATTTTCAATTTCTTTTAAGTGGCACACATGACATAT
TP27446_Query	D+G	1	chr5	1A	CAGCCATTGGTAAGGGAACATGCATTCCATTTTCAATTTCTTTAAAGTGGCACACATGACATAT
TP9669_Hit	D+G	1	chr5	3A	CAGCACATACTTGTCTTCCCACCCGCTCCGGCTTAATCCAAAAAGGTGTCCCTTGCTCCGATAC
TP9669_Query	D+G	1	chr5	3A	CAGCACATACTTGCCTTCCCACCCGCTCCGGCTTAATCCAAAAAGGTGTCCCTTGCTCCGATAC
TP80728_Hit	D+G	1	chr5	3B	CTGCGATTTATTTGAGGCGTAGAGGGAAGAAATGGGAGAGCCAGAGGAAACCTATACAAATAAC
TP80728_Query	D+G	1	chr5	3B	CTGCGATTTATTTGAGGCATAGAGGGAAGAAATGGGAGAGCCAGAGGAAACCTATACAAATAAC
TP14563_Hit	D	1	chr5	5A	CAGCAGGAGAAAGCGTCCGCATGCTTGGACTAGCACTTCCACCTCTAGCTGGTGATGCCAAAGG
TP14563_Query	D	1	chr5	5A	CAGCAGGAGAAAGCGTCCGCATGCTTGGACTAGCACTTCCACCTCTAGATGGTGATGCCAAAGG
TP19391_Hit	D	1	chr5	5A	CAGCATCTCTTGGATTCAATAAGCTTTTCCTACTTACAACACGTACAGCAGATTGGTAAGCAAC
TP19391_Query	D	1	chr5	5A	CAGCATCTCTTGGATTCAATAAGCTTTTCCTACTTACAACACGGACAGCAGATTGGTAAGCAAC
TP20075_Hit	D	1	chr5	5A	CAGCATGCACTATATTTTTTCTCTAAAACCTCTGTGATCTGATCTTCCAGCTTCTTTTCCTGTT
TP20075_Query	D	1	chr5	5A	CAGCATGCACTATATTTTTTCTCTAAAACCTCTGTGATCTGATCTTCCAGCTTCTTTTCCCGTT
TP27548_Hit	D+G	1	chr5	5A	CAGCCATTTGATGATTTTATGAATGATAGATCATGGCATTATCAGGATCCATCTGGGAAGGTTC
TP27548_Query	D+G	1	chr5	5A	CAGCCATTTGATGATTTTATGAATGATAGATCATGGCACTATCAGGATCCATCTGGGAAGGTTC
TP3134_Hit	D+G	1	chr5	5A	CAGCAACAATCAAACGAGCTTGAACTTGAAGTTCACAAGGTTTTTCTGATAATGGTGAAGTGGG
TP3134_Query	D+G	1	chr5	5A	CAGCAACAATCAAACGAGCTTGAACTTGAAGTTCACAAGGTTTCTCTGATAATGGTGAAGTGGG
TP31839_Hit	D	1	chr5	5A	CAGCCTACAAATTATTTTTGCAAGACATTGACGGCGAGTGATACTAGCACACATGGAGGGTTCT
TP31839_Query	D	1	chr5	5A	CAGCCTACAAATTATTTTTGCAAGACATTGACGGCGAGTGATACAAGCACACATGGAGGGTTCT
TP37735_Hit	D+G	1	chr5	5A	CAGCGCGAGCGAGTGGCTATCACACAAAGCAACTGATGTATGTATGGTCTACTGTGTTTAGAGT
TP37735_Query	D+G	1	chr5	5A	CAGCGCGAGCGAGTGGCTATCACACAAAGCAACTGATGTATGTATGGTCTACTGTATTTAGAGT
TP44449_Hit	D	1	chr5	5A	CAGCTAGTTTGAGCATGTTGTTTGCATGGTTGTGTTTGTGTTTGGAGCCTAAGAAGAGACGAGG
TP44449_Query	D	1	chr5	5A	CAGCTAGTTTGAGCATGTTGTTTGCATGGTTGTGTTTGTGTTTGGAACCTAAGAAGAGACGAGG
TP51948_Hit	D	1	chr5	5A	CAGCTGGTTACAAAAAATAGAATATATAATGGATTAGCAGGTAGAAGACTGATCAACGTTTGAG
TP51948_Query	D	1	chr5	5A	CAGCTGGTTACAAAAAATAGAATATATAATGGATTAGCAGGTAGAAGACTGATCAACGTTTAAG
TP5966_Hit	D+G	1	chr5	5A	CAGCGAGGACAAGAATACGATCCTCTGATTTCGAGGTAAGTCCATCCCAATTGGACATGAATTC
TP5966_Query	D+G	1	chr5	5A	CAGCAAGGACAAGAATACGATCCTCTGATTTCGAGGTAAGTCCATCCCAATTGGACATGAATTC
TP63453_Hit	D+G	1	chr5	5A	CTGCAAGGGCCGCCCTTGCTCGTACAGCCTTTGTTAAAAACTTGCGACCTCAGCCTCTTCCTGC
TP63453_Query	D+G	1	chr5	5A	CTGCAAGGGCCGCCCTTGCTCGTACAGCCTTTGTTAAAAACTTGCGACCTCAGCCTCTTCCGGC
TP67547_Hit	D	1	chr5	5A	CTGCAGAACATTAGGAATATTAGAATCTAAAACCAAGTTTATGGCAATCTCACTATGTTTCTTT
TP67547_Query	D	1	chr5	5A	CTGCAGAACATTAGGAATATTAGAATCTAAAACCAAGTTTATGGCAATCTCACTATGTTACTTT

Name	Filter	Nb hit (Mt4.0)	Mt Chr (Mt4.0)	Ms Chr (Li et al., 2014)	Sequence
TP67666_Hit	D	1	chr5	5A	CTGCAGAAGTTGAATACTTCACAAAAATTTCATTGTTCAAATTGATTCATGTAGAAGTTAACTT
TP67666_Query	D	1	chr5	5A	CTGCAGAAGTTGAATACTTCACAAAAATTTCATTGCTCAAATTGATTCATGTAGAAGTTAACTT
TP6981_Hit	D	1	chr5	5A	CAGCAATAGCAAGCATTTTAGCCGGAAGAGGCTGAGGTCGCAAGTTTTTAACAAAGGCTGTACG
TP6981_Query	D	1	chr5	5A	CAGCAATAGCAAGCATTTTAGCAGGAAGAGGCTGAGGTCGCAAGTTTTTAACAAAGGCTGTACG
TP71899_Hit	D+G	1	chr5	5A	CTGCATGCTTGTCTGTCTGTGCTAAGTTTTACAATTTTGTATACAGGGTGATACACCGCATGAA
TP71899_Query	D+G	1	chr5	5A	CTGCATGCTTGTCTGTCTGTGCTAAGTTTTACAATTTTGTATACAGGGTGATACACCACATGAA
TP85922_Hit	D	1	chr5	5A	CTGCTAGTTCTGTTATTTACACAAAGATTTCAGTATAAGATTCAGATTTTTCCCCGTGTACATT
TP85922_Query	D	1	chr5	5A	CTGCTAGTTCTGTTATTTACACAAAGATTTCAGTATAAGATTCAGATTTTTCCCAGTGTACATT
TP86611_Hit	D	1	chr5	5A	CTGCTATTACAACCGCCCTTGAAAGTGAGTGAACTTATAATTTAAGTCCACAGTGAATACTGAA
TP86611_Query	D	1	chr5	5A	CTGCTATTACAACCGCCCTTGAAAGTGAGTGAACTTATAATTTAAGTCCACAGTGAAAACTGAA
TP89337_Hit	D+G	1	chr5	5A	CTGCTCTTTCCACAAGCAGAAAATGGATCTCACTCTCTATTGAGCCAGTATTTGCAGAGTAATG
TP89337_Query	D+G	1	chr5	5A	CTGCTCTTTCCAAAAGCAGAAAATGGATCTCACTCTCTATTGAGCCAGTATTTGCAGAGTAATG
TP90325_Hit	D	1	chr5	5A	CTGCTGATTTGCTTGATTTGTTAGCTCAGATGGAACAATTTGATATTTTGCCGAATACAGCGAG
TP90325_Query	D	1	chr5	5A	CTGCTGATTTGCTTGATTTGTTAGCTCAGATGGAACAATTTGATATTTCGCCGAATACAGCGAG
TP16683_Hit	D+G	1	chr5	5B	CAGCAGTTGCTAACAAATGTGGAGGAGGCCCTGTTTGCCACTTTTGTTGATCAGACATTGATAA
TP16683_Query	D+G	1	chr5	5B	CAGCAGTTGCTAACAAATGTGGAGGAGGCCCTGATTGCCACTTTTGTTGATCAGACATTGATAA
TP39014_Hit	D	1	chr5	5B	CAGCGGTCGTGATATCAATGCCTCTCCCATCCCACAGTTGCCCAAATTGATGGACAATGTCTGC
TP39014_Query	D	1	chr5	5B	CAGCGGCCGTGATATCAATGCCTCTCCCATCCCACAGTTGCCCAAATTGATGGACAATGTCTGC
TP45045_Hit	D	1	chr5	5B	CAGCTATGACTGCAGTTGTAGTGGAGATCTTCTGTATATCAATGACCATGATACCTGTATAAGT
TP45045_Query	D	1	chr5	5B	CAGCTATGACTGCAGTTGTAGTGGAGATCTTCTGTATATCAAGGACCATGATACCTGTATAAGT
TP53529_Hit	D	1	chr5	5B	CAGCTTATAAAAATGAAATGCAAATTTTTGTAACCAAGATCATTAATGTGGCAAAAGAAGCAAA
TP53529_Query	D	1	chr5	5B	CAGCTTACAAAAATGAAATGCAAATTTTTGTAACCAAGATCATTAATGTGGCAAAAGAAGCAAA
TP56666_Hit	D	1	chr5	5B	CAGCTTCTTTCCAATCTGGAAGCTTTGGCTCAAATTTTATTACATTTGCTTTCAGTACCCCAAG
TP56666_Query	D	1	chr5	5B	CAGCTTCTTTCCAATCTGGAAGCTTTGGCTCAAATTTGATTACATTTGCTTTCAGTACCCCAAG
TP64208_Hit	D+G	1	chr5	5B	CTGCAATGAACAAGGTCAAAGATTCAGAGGTAAAGGAGTTCATTGAGAGGTGTCTTGCTCAGCC
TP64208_Query	D+G	1	chr5	5B	CTGCAATGAACAAGGTCAAAGATTCAGAGGTAAAGGAATTCATTGAGAGGTGTCTTGCTCAGCC
TP67619_Hit	D+G	1	chr5	5B	CTGCAGAAGCTGAAATGGAAGAGTTAAGGGCCCAGTTTGATGCAAATGAAAGGTTTGTGTTTAT
TP67619_Query	D+G	1	chr5	5B	CTGCAGAAGCTGAAATAGAAGAGTTAAGGGCCCAGTTTGATGCAAATGAAAGGTTTGTGTTTAT
TP72031_Hit	D	1	chr5	5B	CTGCATGTAAGCTATTGAGAAGAAGATGTGCTGAGGAATGTCCTTTCTCTCCTTATTTTTCACC
TP72031_Query	D	1	chr5	5B	CTGCATGTAAGCTATTGAGAAGAAGATGTGCTGAGGAATGCCCTTTCTCTCCTTATTTTTCACC
TP7480_Hit	D	1	chr5	5B	CAGCAATCTAGACCAAATGAGCATGAGAAAATTCAACTTACCCACCACATTAGCCCGACACTAT
TP7480_Query	D	1	chr5	5B	CAGCAATCTAGACCAAATGAGCATGAGAAAATTCAACTTACCCACCACATTAGCCCGACACTAA
TP7848_Hit	D	1	chr5	5B	CAGCAATGGAGGCCGAATTGGAAGTGATTTAACATTATCAATGTCTGATCAACAAAAGTGGCAA
TP7848_Query	D	1	chr5	5B	CAGCAATGGAGGCCGAATCGGAAGTGATTTAACATTATCAATGTCTGATCAACAAAAGTGGCAA
TP8368_Hit	D	1	chr5	5B	CAGCAATTGAATCAATTGATGTCCCACGTATCCTTTCTCAACAAGGTGGAGAAGCAGTGTATCC
TP8368_Query	D	1	chr5	5B	CAGCAATTGAATCAATTGATATCCCACGTATCCTTTCTCAACAAGGTGGAGAAGCAGTGTATCC
TP84217_Hit	D	1	chr5	5B	CTGCTAAATAATTATGTGTTTGTTAATTATTCTCATTTGTTCAATCTATTTTTCCAATTTGTTA
TP84217_Query	D	1	chr5	5B	CTGCTAAATAATTATGTGTTTGTTAACTATTCTCATTTGTTCAATCTATTTTTCCAATTTGTTA
TP86252_Hit	D	1	chr5	5B	CTGCTATCATTGCTGTACCTCTTGGGGTACTGAAAGCAAATGTAATCAAATTTGAGCCAAAGCT
TP86252_Query	D	1	chr5	5B	CTGCTATCATTGCTGTACCTCTTGGGGTACTGAAAGCAAATGTAATAAAATTTGAGCCAAAGCT
TP89214_Hit	D+G	1	chr5	5B	CTGCTCTTCTTTCGGGTGCTTTGAATGTGCCAATGGTTCTCACTGGTCATTCACTTGGAAGAAA
TP89214_Query	D+G	1	chr5	5B	CTGCTCTTCTTTCAGGTGCTTTGAATGTGCCAATGGTTCTCACTGGTCATTCACTTGGAAGAAA
TP16423_Hit	D+G	1	chr5	5C	CAGCAGTGTTTTTCTCATCCTCATTGTATGTTACTTCAATTTCAACTACAAGAGTATCATTCAC
TP16423_Query	D+G	1	chr5	5C	CAGCAGTGTTTTTCTCATCCTCATTGTACGTTACTTCAATTTCAACTACAAGAGTATCATTCAC
TP2722_Hit	D	1	chr5	5C	CAGCAACAAACAAAAGATCTTCATTCCATTGTGGGTTTAGAGTTCTAGCAGGAACTGTTTTTGT
TP2722_Query	D	1	chr5	5C	CAGCAACAAACAAAAGATCTTCATTCCATTGTGGGTTTAGAGTTCTAGCAGGAACTGTCTTTGT
TP42082_Hit	D	1	chr5	5C	CAGCTAAATTCTCTGTTATTGTTGCAATATGTCTCGCTTTGTGCCTGCACATAATGGATTGCAA
TP42082_Query	D	1	chr5	5C	CAGCTAAATTCTCTGTTATTGTTGCAATATGTCTCGCGTTGTGCCTGCACATAATGGATTGCAA
TP57862_Hit	D	1	chr5	5C	CAGCTTGGTGATGTCTGCCTAGGTCAGGTAGTAAATCAAATTTCAACCCACAAAACCTGCAAGC
TP57862_Query	D	1	chr5	5C	CAGCTTGGTGATGTCTGCCTAAGTCAGGTAGTAAATCAAATTTCAACCCACAAAACCTGCAAGC
TP58157_Hit	D	1	chr5	5C	CAGCTTGTGAGACAATGGGATCAGCCACAACTATATGTAGTGACAAAACTGGGACACTTACAAC
TP58157_Query	D	1	chr5	5C	CAGCTTGTGAGACAATGGGATCAGCCACAACTATATGTAGTGACAAAACTGGGACACTAACAAC
TP59527_Hit	D+G	1	chr5	5C	CAGCTTTGCTGTTTCACTTTTGCTTATGGTTGGGAATTGTTGGATCACAACCTCGAAGCTTGGG
TP59527_Query	D+G	1	chr5	5C	CAGCTTTGCTGTTTCACTTTTGCTTATGGTTGGGAATTGTTGGATCACAACCCCGAAGCTTGGG

Name	Filter	Nb hit (Mt4.0)	Mt Chr (Mt4.0)	Ms Chr (Li et al., 2014)	Sequence
TP61352_Hit	D+G	1	chr5	5C	CTGCAAAGGCAATTTTTGAGAGAGGTGGTAAGGTGGTTGCAGTGAGTGATATCAATGGTGCTAT
TP61352_Query	D+G	1	chr5	5C	CTGCAAAGGCAATTTTTGAGAGAGGTGGTAAGGTGGTTGCAGTGAGTGACATCAATGGTGCTAT
TP69350_Hit	D+G	1	chr5	5C	CTGCAGGTAGCGCTCATCAGCTTCCCTTGATCGATCCTTTGTATCTTCAATATCTGAGATCAGG
TP69350_Query	D+G	1	chr5	5C	CTGCAGGTAGCGCTCATCAGCTTCCCTTGATCGATCCTCTGTATCTTCAATATCTGAGATCAGG
TP7131_Hit	D	1	chr5	5C	CAGCAATATCAATCCCATTTGGATTACTAATAGCACCATTGATGTCACTCACTGCAACCACCTT
TP7131_Query	D	1	chr5	5C	CAGCAATATCAATCCCATTTGGATTACTAATAGCACCATTGATATCACTCACTGCAACCACCTT
TP72545_Hit	D	1	chr5	5C	CTGCATTCTCTACACTCGCACCCACTTCATTACCATCACCTTTTGAAGGCCAACCCGTTTCCGA
TP72545_Query	D	1	chr5	5C	CTGCATTCTCTACACTCGCACCCACTTCATTACCATCACCTTTCGAAGGCCAACCCGTTTCCGA
TP75160_Hit	D	1	chr5	5C	CTGCCCTCAAATCTTTCTGCTGGTAGAAACCCAAGTCTGGATTATGGTTTTAAGGCCTCTGAAG
TP75160_Query	D	1	chr5	5C	CTGCCATCAAATCTTTCTGCTGGTAGAAACCCAAGTCTGGATTATGGTTTTAAGGCCTCTGAAG
TP89307_Hit	D+G	1	chr5	5C	CTGCTCTTGTCGCTTCTATTGGGGCGTCGTGGCTAACTCGAGTGCTTGGAAGGCGTATCACCAT
TP89307_Query	D+G	1	chr5	5C	CTGCTCTTGTCGCTTCTATCGGGGCGTCGTGGCTAACTCGAGTGCTTGGAAGGCGTATCACCAT
TP19263_Hit	D+G	1	chr5	5D	CAGCATCTACAGGAGACAGAGTCGTAGCTTTGTTGGCCAACTTGAATAAAGCCACAGCACCATC
TP19263_Query	D+G	1	chr5	5D	CAGCATCTACAGGAGACAGAGTCATAGCTTTGTTGGCCAACTTGAATAAAGCCACAGCACCATC
TP2311_Hit	D	1	chr5	5D	CAGCAAATGATTAATTTGCACTTCAAAAGATACCTGACTCGGAACCACGCCGCTTTTTAAAAAA
TP2311_Query	D	1	chr5	5D	CAGCAAATGATTAATTTGCACTTCAAAAGATACCTGACTCGGAACCACACCGCTTTTTAAAAAA
TP27143_Hit	D	1	chr5	5D	CAGCCATGTCGCTCATCTTTTTCTGCCTCCCATATGCACTGATTAGACAGGTATATGAATTGGC
TP27143_Query	D	1	chr5	5D	CAGCCATGTCGCTCATCTTTTTCTGCCTCCCATATGCACTGATTAGACAGGTATATGAAGTGGC
TP39813_Hit	D	1	chr5	5D	CAGCGGTGACTGTTCTTGTGTACATACAAGATAACCAAGGTAGAGGTTGGGGTTACGGTATTTG
TP39813_Query	D	1	chr5	5D	CAGCGGTGACTGTTCTTGTGTACATACAAGATAACCAAGGTAGAGGTTAGGGTTACGGTATTTG
TP55481_Hit	D+G	1	chr5	5D	CAGCTTCCTTGATAGCTTCTGTTGCACTAGGTAAACCCTGAACATGATCAATTAAGTGTTTCAA
TP55481_Query	D+G	1	chr5	5D	CAGCTTCCTTGATAGCTTCAGTTGCACTAGGTAAACCCTGAACATGATCAATTAAGTGTTTCAA
TP59948_Hit	D	1	chr5	5D	CAGCTTTTCACCTCAAAGGACGCGACGCAAGGCTCAATTTTCCAGATATGATTGAGAAGCTTCC
TP59948_Query	D	1	chr5	5D	CAGCTTTTCACCTCAAAGGACGCGACCCAAGGCTCAATTTTCCAGATATGATTGAGAAGCTTCC
TP63144_Hit	D	1	chr5	5D	CTGCAAGAGGCACTCCCCCACACTTTTTTACTATCTCTTTCCCTATGATGACAAGATCGGGTCG
TP63144_Query	D	1	chr5	5D	CTGCAAGAGGCACTCCCCCACACTTTTTTACTATCTCTTTCCCTATGATCACAAGATCGGGTCG
TP71720_Hit	D	1	chr5	5D	CTGCATGAGTGGTATGTGACATTCTTTGACTGCAATGCAACTTTCAATATATGTGGTGATCATG
TP71720_Query	D	1	chr5	5D	CTGCATGAGTGGTATGTGACATTCTTTGACTGCAATGCAACTTTCAATATACGTGGTGATCATG
TP10028_Hit	D	1	chr6	.	CAGCACCAAATCAGAAAATTGTAGTCAGGATGCAAACTGGAAATCATAGATATTATAGCATAAA
TP10028_Query	D	1	chr6	.	CAGCACCAAATCAGAAAATTGTAGTCAGGATGCAAACTGGAAATCATAGATATCATAGCATAAA
TP10270_Hit	D	1	chr6	.	CAGCACCACTGCGTACTTCTGGTTGGGTGTATTGAGGAAACAAACCATGCTCGTCTCCGGTGTT
TP10270_Query	D	1	chr6	.	CAGCACCACTGCGTACTTCTGGTTGAGTGTATTGAGGAAACAAACCATGCTCGTCTCCGGTGTT
TP10284_Hit	D	1	chr6	.	CAGCACCAGAAGACGAAGGTACATGCCGAAACAACGGCAAAGAAACCCTAAACCTTGTCCTCAC
TP10284_Query	D	1	chr6	.	CAGCACCAGAAGACAAAGGTACATGCCGAAACAACGGCAAAGAAACCCTAAACCTTGTCCTCAC
TP10853_Hit	D	1	chr6	.	CAGCACCTACACACTTTTTTAACAAAGATAGCATATCATATTCATGAGTATGGTTCATGATTCA
TP10853_Query	D	1	chr6	.	CAGCACCTACACACTTTTTTAACAAAGACAGCATATCATATTCATGAGTATGGTTCATGATTCA
TP12028_Hit	D+G	1	chr6	.	CAGCACTCATATATGAATATCTTGTGATCCCAACTGAGCCACCTCATGCACACCAGCCTCTTGC
TP12028_Query	D+G	1	chr6	.	CAGCACTCATATATGAATATCTTGTGATCCCAACTGAGCCACCTCATGCACACCAGCCACTTGC
TP12432_Hit	D	1	chr6	.	CAGCACTTACATCAAGCCTTGACAACGCCTCTATTTCCGGATATTCTGATGCTGGACTTCCCAC
TP12432_Query	D	1	chr6	.	CAGCACTTACATCAAGCCTTGACAACGCCTCTATTTCCGGATATTCTGATACTGGACTTCCCAC
TP12992_Hit	D	1	chr6	.	CAGCAGAACAGAAGTAAGTAATACAACACATCTAACTAATGAAAATAACGGTAAGAGATTACAG
TP12992_Query	D	1	chr6	.	CAGCAGAACAGAAGTAAGTAATACAACACATCTAACTAATGAAAATAACGGCAAGAGATTACAG
TP13008_Hit	D	1	chr6	.	CAGCAGAACGTTTCTTTTACTATTTCTGTTTGTGCTCACTTTTTCCATCATCTTTCTTGATTGG
TP13008_Query	D	1	chr6	.	CAGCAGAACATTTCTTTTACTATTTCTGTTTGTGCTCACTTTTTCCATCATCTTTCTTGATTGG
TP13091_Hit	D	1	chr6	.	CAGCAGAAGAATAACGAAATGATGATAGTTGATGCTTATTTATCTCCAAAGATAGTCTTGTCCA
TP13091_Query	D	1	chr6	.	CAGCAGAAGAATAACGAAATGATGATAGTTGATGCGTATTTATCTCCAAAGATAGTCTTGTCCA
TP13315_Hit	D	1	chr6	.	CAGCAGAATCTCAACAAGGGAAGGAAAAAGAAAATTTTTATATCTATTTCTGACATTATTATGA
TP13315_Query	D	1	chr6	.	CAGCAGAATCCCAACAAGGGAAGGAAAAAGAAAATTTTTATATCTATTTCTGACATTATTATGA
TP13978_Hit	D	1	chr6	.	CAGCAGAGTTTGTTATTGAGCAAAGTGATTTCAGTGATGCAGAAAAGCAACACCACGGCTGACA
TP13978_Query	D	1	chr6	.	CAGCAGAGTTTGTTATTGAGCAAAGTGATTTCAGTGATGCAGAAAAGCAACACCACGACTGACA
TP14196_Hit	D	1	chr6	.	CAGCAGATGATGCTCGGTGTTCTGCAGTTGGTGTTTCAATGCTTAGGCTTGGTGGACATGCTGT
TP14196_Query	D	1	chr6	.	CAGCAGATGATGCTCGGTGTTCTGCAGTTGGTGTCTCAATGCTTAGGCTTGGTGGACATGCTGT
TP14299_Hit	D+G	1	chr6	.	CAGCTGATTCCTCCCCAGGAACCACCAACATCACTGAACCACCGTATGAACCTAACTTCACCAT
TP14299_Query	D+G	1	chr6	.	CAGCAGATTCCTCCCCAGGAACCACCAACATCACTGAACCACCGTATGAACCTAACTTCACCAT

Name	Filter	Nb hit (Mt4.0)	Mt Chr (Mt4.0)	Ms Chr (Li et al., 2014)	Sequence
TP14817_Hit	D	1	chr6	.	CAGCAGGCCACCCTTGAAACAACGGAAAAACTCTTCAAATTGGCAGAGGTCAGTTCTCTCATTT
TP14817_Query	D	1	chr6	.	CAGCAGGCCACCCTTGAAACAACGGAAAAACTCCTCAAATTGGCAGAGGTCAGTTCTCTCATTT
TP15244_Hit	D+G	1	chr6	.	CAGCAGGTCATGTACTGATGCAGATTGCGGAATTATGACAGAGCAGAAATAACCTGTTGTCGAA
TP15244_Query	D+G	1	chr6	.	CAGCAGGTCATGTACTGATGCAGATTGCGGAATTATGACAGAGCAGAAATAACCTATTGTCGAA
TP15613_Hit	D	1	chr6	.	CAGCAGTACACAAGGAACATAGCAAATTTCTACTCCACGGCATTTGCGCTTGTGAGATGTTGAA
TP15613_Query	D	1	chr6	.	CAGCAGTACACAAGGAACATAGCAAATTTCTACTCCAAGGCATTTGCGCTTGTGAGATGTTGAA
TP16225_Hit	D	1	chr6	.	CAGCAGTGCTGTTGGGAATCCATTTCTAAGCTCCTGCCGTGGCGTTTCTCTTACTGTACCATCG
TP16225_Query	D	1	chr6	.	CAGCAGTGCTGTTGGGAATCCATTTCTAAGCTCCTGCCGTGGCGTTTCTCTGACTGTACCATCG
TP17228_Hit	D	1	chr6	.	CAGCATTATCACCTGGCCTCGACAATGGCATCCACGGTCTTCTGCTCCTGTGGACTGAGCCTCC
TP17228_Query	D	1	chr6	.	CAGCATAATCACCTGGCCTCGACAATGGCATCCACGGTCTTCTGCTCCTGTGGACTGAGCCTCC
TP17248_Hit	D	1	chr6	.	CAGCATAATGAAGCATGTAAATGATGCCATTCATCTCTCCACATTAGACAGAGTATATGCTTTG
TP17248_Query	D	1	chr6	.	CAGCATAATGAAGCATGTAAATGATCCCATTCATCTCTCCACATTAGACAGAGTATATGCTTTG
TP17428_Hit	D	1	chr6	.	CAGCATACCCGAGGATGTTTCCGACGGCCATGAAGAAAGAAAAGAAACCATTTGCTGTTCTCAT
TP17428_Query	D	1	chr6	.	CAGCATACCCGAGGATGTTTCCGACAGCCATGAAGAAAGAAAAGAAACCATTTGCTGTTCTCAT
TP17494_Hit	D	1	chr6	.	CAGCATACTTATGAAGGTCAGGCATGATCAACAACTCCATCACTGACCTTGACGTGTTGCAGTA
TP17494_Query	D	1	chr6	.	CAGCATACTCATGAAGGTCAGGCATGATCAACAACTCCATCACTGACCTTGACGTGTTGCAGTA
TP17618_Hit	D	1	chr6	.	CAGCATAGCAGAACATGTTTGTTTCAGCAAAACCAGAAAATAACTCTACAAAATCATCGAGGAA
TP17618_Query	D	1	chr6	.	CAGCATAGCAGAACATGTTTGTTTCAACAAAACCAGAAAATAACTCTACAAAATCATCGAGGAA
TP18277_Hit	D	1	chr6	.	CAGCATCAACAGCGTGTCCACCAAGCTTAAGCATTGAAACACCGACTGCAGAACACCGAGCATC
TP18277_Query	D	1	chr6	.	CAGCATCAACAGCGTGTCCACCAAGCTTAAGCATTGAAACACCAACTGCAGAACACCGAGCATC
TP18573_Hit	D	1	chr6	.	CAGCATCAGAGAGTTCTCTGCAAGATGTTGTATCACGATATAGTTTCATGGATCTATGGCCGTG
TP18573_Query	D	1	chr6	.	CAGCATCAGAGAGTTCTCTGCAAGATGTTGTATCACGATATAGTTTCATGGATCTATGGCCATG
TP18648_Hit	D	1	chr6	.	CAGCATCAGGATCAAATTTTATTGGTGTAGAAGCAGATATTAATGTTTGGAATCCAAAGGTTGA
TP18648_Query	D	1	chr6	.	CAGCATCAGGATCAAATTTTATTGGTGCAGAAGCAGATATTAATGTTTGGAATCCAAAGGTTGA
TP18755_Hit	D	1	chr6	.	CAGCATCATCGCGGAGTCCATCTCCTTGTGTTGAATGCCTCTTACGAGTTAAATCATTTGAATC
TP18755_Query	D	1	chr6	.	CAGCATCATCGCGGAGTCCATCTCCTTGTGTTGAATGCCTCTTACGAGTAAAATCATTTGAATC
TP18874_Hit	D	1	chr6	.	CAGCATCCAAGAAGTTTGACAAGATTACGGTGTTGAAGTTTGGCAATCAACGCCGCTTCATTTT
TP18874_Query	D	1	chr6	.	CAGCATCCAAGAAGTTTGACAAGATTACGATGTTGAAGTTTGGCAATCAACGCCGCTTCATTTT
TP19402_Hit	D	1	chr6	.	CAGCATCTGAACTTTCATCGCACTTAGGAGGGGAATCTAAATCTTTGGTAGCAACTTCGTTGGT
TP19402_Query	D	1	chr6	.	CAGCATCTGAACTTTCATCGCACTTAGGAGGGGAATCTAAATCGTTGGTAGCAACTTCGTTGGT
TP19516_Hit	D+G	1	chr6	.	CAGCATCTTCAAACTTGGAGTTGTGTGCACTTCAACACTACCAGCTAGCAGACCATCAATGAAG
TP19516_Query	D+G	1	chr6	.	CAGCATCTTCAAACTTGGAGTTATGTGCACTTCAACACTACCAGCTAGCAGACCATCAATGAAG
TP19539_Hit	D	1	chr6	.	CAGCGTCTTCCCATTGGACTTATCTCATTCATATGTTCGATTATGAGTAGGAGGATTAGTGGTG
TP19539_Query	D	1	chr6	.	CAGCATCTTCCCATTGGACTTATCTCATTCATATGTTCGATTATGAGTAGGAGGATTAGTGGTG
TP19596_Hit	D	1	chr6	.	CAGCATCTTTAGCAAACTCCGTGTCCCCTGCAGGGACAAGCCTGCAGACAAAAATAAGTAGGAT
TP19596_Query	D	1	chr6	.	CAGCATCTTTAGCAAACTCCGTGTCCCCTGCAGGAACAAGCCTGCAGACAAAAATAAGTAGGAT
TP19979_Hit	D	1	chr6	.	CAGCATGATGGTGATGATGCTCATGGTGATTGTGATCATGACAATCACTATGGTCATGGTGCCC
TP19979_Query	D	1	chr6	.	CAGCATGATGGTGATGATGCTCATGGTGATTGTGATCATGACAATCACTATGGTCATGATGCCC
TP20232_Hit	D+G	1	chr6	.	CAGCATGCTCCACAACCTTGCCTTACAATAAGGACAAACCTCTGTCGGATGGTGCTGAAAAAAA
TP20232_Query	D+G	1	chr6	.	CAGCATGCTCCACAACCTCGCCTTACAATAAGGACAAACCTCTGTCGGATGGTGCTGAAAAAAA
TP20931_Hit	D	1	chr6	.	CAGCATTAACATTTTTAATAGGTGCATTATTAATGGGATTAGCACCATCATTTGCATTCTTAAT
TP20931_Query	D	1	chr6	.	CAGCATTAACATTTTTAATAGGTGCATTATTAATGGGATTAGCACCATCATTTACATTCTTAAT
TP21407_Hit	D	1	chr6	.	CAGCATTCCCCCATCAAAGAATAATTTCAGGGCAAAGGTGCCAGTGGAACCAGTACTACCTGCT
TP21407_Query	D	1	chr6	.	CAGCATTCCCCCATCAAAGAATAATTTCAGGGCAAAGGTGCCAGTGGAACCAGTACTACCCGCT
TP21455_Hit	D	1	chr6	.	CAGCATTCGGCATAGAAACATTGTGAAGTTGTTATGTTGTATCTCTAACGATGATACAATGCTA
TP21455_Query	D	1	chr6	.	CAGCATTCGGCATAGAAACATCGTGAAGTTGTTATGTTGTATCTCTAACGATGATACAATGCTA
TP22100_Hit	D	1	chr6	.	CAGCATTTCAGTGCATCCACAACATTGCTTTTGTCATTCATCTCATTTCTTCCACAATCAATTT
TP22100_Query	D	1	chr6	.	CAGCATTTCAGTGCATCCACAACATTGCTTTTGTCATTCATCTCATTCCTTCCACAATCAATTT
TP22286_Hit	D	1	chr6	.	CAGCATTTGCCGCTTGCTGAAGATACACATTGTTATTTTGATCTCCACGAAACAAAGCCTGTCT
TP22286_Query	D	1	chr6	.	CAGCATTTGCCGCTTGCTGAAGATACACATTGTTATTTTGATCCCCACGAAACAAAGCCTGTCT
TP22287_Hit	D+G	1	chr6	.	CAGCATTTGCCTTCTACTAAGATGGGGTGCGATGGATGCAGAAGCATACCCAGAAGCATCAAAG
TP22287_Query	D+G	1	chr6	.	CAGCATTTGCCTTCTACTAAGATGGGGTGCGATGGACGCAGAAGCATACCCAGAAGCATCAAAG
TP22476_Hit	D	1	chr6	.	CAGCATTTTCTGAGCAGACTAACCGATGTCTTTCTCCAAATTCTTGCACCACCGAGATTCACCC
TP22476_Query	D	1	chr6	.	CAGCATTTTCTGAGCAGACTAACAGATGTCTTTCTCCAAATTCTTGCACCACCGAGATTCACCC

Name	Filter	Nb hit (Mt4.0)	Mt Chr (Mt4.0)	Ms Chr (Li et al., 2014)	Sequence
TP22826_Hit	D	1	chr6	.	CAGCCAAAATTATCGCAAACTACTTAATATGACCAATATATGATGAGGAGATTCACCTTGCAAC
TP22826_Query	D	1	chr6	.	CAGCCAAAATTATCGCAAACGACTTAATATGACCAATATATGATGAGGAGATTCACCTTGCAAC
TP22861_Hit	D	1	chr6	.	CAGCCAAACACAGGAAACAACTTAAAATTAAACAGATAGTATTTCAAATATAGGCTACACACAA
TP22861_Query	D	1	chr6	.	CAGCCAAACACAGGAAACAACTTAAAATTAAACAGATAGTACTTCAAATATAGGCTACACACAA
TP2344_Hit	D	1	chr6	.	CAGCAAATGCTCGTTTTTCAGTGCGACGCAGGAGGCGTTCTGCATCTTCCTCAGCTTCCATTTG
TP2344_Query	D	1	chr6	.	CAGCAAATGCCCGTTTTTCAGTGCGACGCAGGAGGCGTTCTGCATCTTCCTCAGCTTCCATTTG
TP23443_Hit	D	1	chr6	.	CAGCCAACGGATGATCATCCGGCAACAATCCCTTCCCCATTGGAGTCGGAAGAAAGGGAATCCC
TP23443_Query	D	1	chr6	.	CAGCCAACGGATGATCATCCGGCAACAATCCCTTCCCCATTGGAGTCGGAAGAAACGGAATCCC
TP23736_Hit	D	1	chr6	.	CAGCCAAGGTTGCTTATACAACTTTGAAAGAACTAGTTATGTCTATAATATTAGTGTCTCAATG
TP23736_Query	D	1	chr6	.	CAGCCAAGGTTGCTTATACAACTTTGAAAGAACTAGGTATGTCTATAATATTAGTGTCTCAATG
TP24002_Hit	D	1	chr6	.	CAGCCAATGATCCACTTGTTCTTGTTCATTCTTCTAGACATGTTTGTCATAATTTTCTTGCTCT
TP24002_Query	D	1	chr6	.	CAGCCAATGATCCACTTGTTCTTGTTCATCCTTCTAGACATGTTTGTCATAATTTTCTTGCTCT
TP2454_Hit	D	1	chr6	.	CAGCAAATTAGTACTCTGGCGGGAAATTTGACACCAAGCTATTTTATTTGGGAAGTCCTTTTTA
TP2454_Query	D	1	chr6	.	CAGCAAATTAGTACTCTGGCAGGAAATTTGACACCAAGCTATTTTATTTGGGAAGTCCTTTTTA
TP25076_Hit	D	1	chr6	.	CAGCCACGGAAGTAAAAACTAAGCAACGGAAGAATGTCAGTTGCAATATTCTCAACACATAGTG
TP25076_Query	D	1	chr6	.	CAGCCACGGAAGTAAAAACTAAGCAACGGAAGAATGTCAGTTGCAATATTCTCAACACATAATG
TP25730_Hit	D	1	chr6	.	CAGCCAGCAAGCATTGTAAAATGGATAAAACCAGGTCTAGGTCGTTATAAGTGTAATATTGACG
TP25730_Query	D	1	chr6	.	CAGCCAGCAAGCATTGCAAAATGGATAAAACCAGGTCTAGGTCGTTATAAGTGTAATATTGACG
TP25813_Hit	D	1	chr6	.	CAGCCAGCCAGACATGGAACCAAGCCATTGGCCAATGAAATGCATTAGCAAGGCAACAGAAACA
TP25813_Query	D	1	chr6	.	CAGCCAGCCAGACATCGAACCAAGCCATTGGCCAATGAAATGCATTAGCAAGGCAACAGAAACA
TP27174_Hit	D	1	chr6	.	CAGCCATGTTAACACTTTCTTCTTGCAAGAGAAATAAGGTAGCAATAGCTTCATCTGGTGCAGT
TP27174_Query	D	1	chr6	.	CAGCCATGTTAACACTTTCTTCTTGCAAGAGAAATAAGGTAGCAATAGCTTCAGCTGGTGCAGT
TP27395_Hit	D	1	chr6	.	CAGCCATTGATGAATAGTTCCTGCGCATATGTGTGGATCGTTTATTGCAGTCACATAGCAAAAC
TP27395_Query	D	1	chr6	.	CAGCCATTGATGAATAGTTCCTGCGCATATGTGTGGATCGTTTATTGCAGTCACATAGAAAAAC
TP27861_Hit	D+G	1	chr6	.	CAGCCCAATCGGCTTTGCACCTCCAACAACTATCAATTGGGTTGGTTGCTTCACATGGTCCATC
TP27861_Query	D+G	1	chr6	.	CAGCCCAATCGGCTTTGCACCTCCAACAACTATCAATTGGGTTCGTTGCTTCACATGGTCCATC
TP28069_Hit	D	1	chr6	.	CAGCCCGGATTGTATGAGCAGTCGAGCATATGCTTCAACGATCAAAGAGTTATGCTTGGCTAAT
TP28069_Query	D	1	chr6	.	CAGCCCAGATTGTATGAGCAGTCGAGCATATGCTTCAACGATCAAAGAGTTATGCTTGGCTAAT
TP29102_Hit	D	1	chr6	.	CAGCCTGGCTGGAGAACTGGACTTTTCCTGGATCTACTCTACCCTCTGGATTGGAGATTTTATT
TP29102_Query	D	1	chr6	.	CAGCCCGGCTGGAGAACTGGACTTTTCCTGGATCTACTCTACCCTCTGGATTGGAGATTTTATT
TP29588_Hit	D+G	1	chr6	.	CAGCCCTGCATATACGTATTGGTGTTTCCTTCTTTGGTCATGAGCAGACCTTTAGATTATTTTG
TP29588_Query	D+G	1	chr6	.	CAGCCCTGCATATACATATTGGTGTTTCCTTCTTTGGTCATGAGCAGACCTTTAGATTATTTTG
TP29799_Hit	D+G	1	chr6	.	CAGCCCTTTATCGCTCCAGGATCTTGTCAGCTGGAAAATGGCCCTAGAGAAAAGACAGCTTCCT
TP29799_Query	D+G	1	chr6	.	CAGCCCTTTATCGCGCCAGGATCTTGTCAGCTGGAAAATGGCCCTAGAGAAAAGACAGCTTCCT
TP30226_Hit	D+G	1	chr6	.	CAGCTGATGATGCTTGGTGTTCTGTTGGTGTTTCAATGCTTAGGCTTGGTGGACATGTTGTTGG
TP30226_Query	D+G	1	chr6	.	CAGCCGATGATGCTTGGTGTTCTGTTGGTGTTTCAATGCTTAGGCTTGGTGGACATGTTGTTGG
TP3071_Hit	D	1	chr6	.	CAGCAACAAGGAAACCAAACACATGAAAATTTTGAAGGTCAGCAAGAAGTTAGACAGGTTGATC
TP3071_Query	D	1	chr6	.	CAGCAACAAGGAAACCAAACACATGAAAATTTTGAAGGTCAGCAAGAAGTTAGACAAGTTGATC
TP30719_Hit	D	1	chr6	.	CAGCCGTGTTGCAGAGACATCATAAGTTTTTATGTTGCATTCCAAATCAGGGCCGGGGACTTAC
TP30719_Query	D	1	chr6	.	CAGCCGCGTTGCAGAGACATCATAAGTTTTTATGTTGCATTCCAAATCAGGGCCGGGGACTTAC
TP30721_Hit	D+G	1	chr6	.	CAGCCGCGTTGCAGAGACATCATAAGTTTTTATGTTGTGTTCCAAATCAGGGCCGGGGACTTGC
TP30721_Query	D+G	1	chr6	.	CAGCCGCGTTGCAGAGACATCATAAGTTTTTATGTTGCGTTCCAAATCAGGGCCGGGGACTTGC
TP30799_Hit	D	1	chr6	.	CAGCCGCTCGCGTAATTGGCCTAACTCTGATAAAATGTTACGCTTTTTTCTGTTTGCATTGTTA
TP30799_Query	D	1	chr6	.	CAGCCGCTCGCGTAATTGGCCTAACTCTGATAAAATATTACGCTTTTTTCTGTTTGCATTGTTA
TP3104_Hit	D+G	1	chr6	.	CAGCAACAAGTTTGGCATTCATATCAGCAACAAGTTCATAAGTAGGAAAGCTAGAAGCTGTTTG
TP3104_Query	D+G	1	chr6	.	CAGCAACAAGTTTGGCATTCATATCAGCAACAAGTTCATAAGTAGGAAAACTAGAAGCTGTTTG
TP31199_Hit	D	1	chr6	.	CAGCCGGTTGTTTGTGGGCAGTTTCCGAGCTCACATAGGCCCATAATTGGCATGACTGATGCAA
TP31199_Query	D	1	chr6	.	CAGCCGGTTGTTTGTGGGCAGTTTCCAAGCTCACATAGGCCCATAATTGGCATGACTGATGCAA
TP31448_Hit	D	1	chr6	.	CAGCCGTGGTGCCGATTTATCCCCCTTGGAGGTAATCATATATACTTATATGTATTTTGGCTAA
TP31448_Query	D	1	chr6	.	CAGCCGTGGTGCCGATTTATCCCCCTTGGAGGTAATCATATATACTTATAGGTATTTTGGCTAA
TP31939_Hit	D	1	chr6	.	CAGCCTAGAGCTATAAAGCTCCCACGTGCTGGGTCGAGGGGAGGGGCACAGATCCATTGAGGAT
TP31939_Query	D	1	chr6	.	CAGCCTAGAGCTATAAAGCTCCCACGTGCTGGGTCAAGGGGAGGGGCACAGATCCATTGAGGAT
TP32028_Hit	D	1	chr6	.	CAGCCTATAACAGTGAATCCGAAACCTGCCTCCTGTTTCCAACAGAACGCATCTGCTACATCGG
TP32028_Query	D	1	chr6	.	CAGCCTATAACAGTGAATCCGAAACCTGCCTCCTGTTTCCAACAGAACGCATCTGCTACATCGA

Name	Filter	Nb hit (Mt4.0)	Mt Chr (Mt4.0)	Ms Chr (Li et al., 2014)	Sequence
TP337_Hit	D+G	1	chr6	.	CAGCAAAACAAGGAAAGAAATCCGATACATTCTCGCTTGCTCGCAACTCACAGACGCCTTTGCT
TP337_Query	D+G	1	chr6	.	CAGCAAAACAAGGAAAGAAATCCGATACATTCTCGCTTGCTCGCAACTCACAGACGCCCTTGCT
TP33702_Hit	D	1	chr6	.	CAGCCTGGCAGACCAAAGAAGAATAGAAGAAAGGATGCTGATGAAAAAAGGGATGAACAATAGT
TP33702_Query	D	1	chr6	.	CAGCCTGGCAGACCAAAGAAGAATAGAAGAAAGGATGCTGATGAAAAAAGGGATGAACAACAGT
TP33918_Hit	D	1	chr6	.	CAGCCTGTTAATCTATCCGGCAAACGCAAGCGCAAGCTCTTCAAACAATGGCGTCGTGTCAGTA
TP33918_Query	D	1	chr6	.	CAGCCTGTTAAGCTATCCGGCAAACGCAAGCGCAAGCTCTTCAAACAATGGCGTCGTGTCAGTA
TP34019_Hit	D+G	1	chr6	.	CAGCCTTAACCGGGAAGTCGAGGAGGCAGTTTCTTAGATTTGAGTATTGCCTCATAACATGTCT
TP34019_Query	D+G	1	chr6	.	CAGCCTTAACCGGGAAGTCGAGGAGGCAATTTCTTAGATTTGAGTATTGCCTCATAACATGTCT
TP34474_Hit	D	1	chr6	.	CAGCCTTCTTAACACGAGGATAACCTTCAACAAAATTAAGACTTTGCACCTAACACCAACAACA
TP34474_Query	D	1	chr6	.	CAGCCTTCTTAACACGAGGATAACCTTCAACAAAATTAAGACTCTGCACCTAACACCAACAACA
TP34805_Hit	D	1	chr6	.	CAGCCTTTAACAGCCTTGAAATGTTAAAAGTCCTAGAACCAGCGTATGCGGGAGCTTTAGTGCA
TP34805_Query	D	1	chr6	.	CAGCCTTTAACAGCCTTGAAATGTTAAAAGTCCTAGAACCAGCGTATGCAGGAGCTTTAGTGCA
TP35235_Hit	D+G	1	chr6	.	CAGCGAAACTAGTGCGATCATTGATAATGAAGTGCGAGATTGGGTGAAGAAAGCATACAAACAT
TP35235_Query	D+G	1	chr6	.	CAGCGAAACTAATGCGATCATTGATAATGAAGTGCGAGATTGGGTGAAGAAAGCATACAAACAT
TP35567_Hit	D	1	chr6	.	CAGCGAATCAACCTATGCAAAATACCAGAGGATGCATCCTCCATTTCATGTTTAGGCAAAGATT
TP35567_Query	D	1	chr6	.	CAGCGAATCAACCTATGCAAAATACCAGAGGATGCATCCTCCATTTCATGGTTAGGCAAAGATT
TP3602_Hit	D	1	chr6	.	CAGCAACAGTTCCATCTCTGCCTCCTGCTTGGCCTTGTGGACGTCCATAGCCAACTTTTATACC
TP3602_Query	D	1	chr6	.	CAGCAACAGTTCCATCTCTGCCTCCTGCTTGGCCTTGTGGACGTCCATACCCAACTTTTATACC
TP36358_Hit	D	1	chr6	.	CAGCGAGGTTTTTGATAAAGATATGGAGTTAACAAGTAATAATGAGGGGGAGATGGTTAAGCTT
TP36358_Query	D	1	chr6	.	CAGCGAGGTTTTTGATAAAGATATGGAGTTAACAAGTAATAATGAGGGGGAGATGGTTAAACTT
TP36504_Hit	D	1	chr6	.	CAGCGATAGTCACTGTTGAAGGACTAACAACAGTTTTGGAGGGTCCAACTAGTCTTCTGGCCGT
TP36504_Query	D	1	chr6	.	CAGCGATAGTAACTGTTGAAGGACTAACAACAGTTTTGGAGGGTCCAACTAGTCTTCTGGCCGT
TP36810_Hit	D	1	chr6	.	CAGCGATTCATCAATCCATTTTTTCATTAGGTTCAATACGGTTGAATCAACTTCAAAAGGAACC
TP36810_Query	D	1	chr6	.	CAGCGATTCATCAATCCATTTTCTCATTAGGTTCAATACGGTTGAATCAACTTCAAAAGGAACC
TP37289_Hit	D+G	1	chr6	.	CAGCGCATTGTACTCTGCTTCCTCTTCTGTCATTTTCTCAGGCAGTTCGACGTTCCTCCCAACT
TP37289_Query	D+G	1	chr6	.	CAGCGCATTGTACTCTGCTTCCTCTGCTGTCATTTTCTCAGGCAGTTCGACGTTCCTCCCAACT
TP3742_Hit	D	1	chr6	.	CAGCAACATCTATCAATCCATTAGTAATCTCTTCTAAAGGGATTCCAGGTTTTTTCTTCCCGAA
TP3742_Query	D	1	chr6	.	CAGCAACATCTATCAATCCATTAGCAATCTCTTCTAAAGGGATTCCAGGTTTTTTCTTCCCGAA
TP37490_Hit	D	1	chr6	.	CAGCGCCGAACAGGAGGGTTGGTTACATGGAGAGCGGGAAGTGTTATGATCGTGTATCGTGGTA
TP37490_Query	D	1	chr6	.	CAGCGCCGAACAGGAGGGTTGGTTACATGGAGAGCAGGAAGTGTTATGATCGTGTATCGTGGTA
TP37571_Hit	D+G	1	chr6	.	CAGCGCCGGTATGTTCTGTTGGGCTGATATGAGTGGTTTAATCAGACCTTATAGTGAGAAAGGA
TP37571_Query	D+G	1	chr6	.	CAGCGCCGGTATGTTCTGTTGGGCTGATATGAGTGGTTTAATCAGACCTTATAGCGAGAAAGGA
TP37763_Hit	D+G	1	chr6	.	CAGCGCGATGTTATCGGTGTTGCTGAGACTGGTTCTGGGAAGACTGCTGAAAAAAAAAAAAAAA
TP37763_Query	D+G	1	chr6	.	CAGCGCGATGTTATCGGTGTTGCTGAGACGGGTTCTGGGAAGACTGCTGAAAAAAAAAAAAAAA
TP38172_Hit	D	1	chr6	.	CAGCGCTGACAGATCACATTTAAAAAGGAAATTAGATCTAGAGTTCCAGTAACCAGAATACTCT
TP38172_Query	D	1	chr6	.	CAGCGCTGACAGATCACATTTAAAAAGGAAATTAGATCTAGAGTTCCAGTAACCAGAATACTAT
TP3859_Hit	D	1	chr6	.	CAGCAACATTTATCCATTCCTTATTTTAACAACTGTTTCATTTCTTCCGCACACTAAAAGCCCC
TP3859_Query	D	1	chr6	.	CAGCAACATTTATCCATTCCTTATTTTAACAACTGTTTCATTTCTTCCGCACACTAAAAGACCC
TP39489_Hit	D	1	chr6	.	CAGCGGGGTTTGGAGGCATCATCCGAAATCATGAAGGTCGATTCATGCTTGGGTTTAGTGGATC
TP39489_Query	D	1	chr6	.	CAGCGGGCTTTGGAGGCATCATCCGAAATCATGAAGGTCGATTCATGCTTGGGTTTAGTGGATC
TP40424_Hit	D	1	chr6	.	CAGCGTCAATATTACACTTATAACGACCTAGACCTGGTTTTATCCATTTTGCAATGCTTGCTGG
TP40424_Query	D	1	chr6	.	CAGCGTCAATATTACACTTATAACGACCTAGACCTGGTTTTATCCATTTTACAATGCTTGCTGG
TP41350_Hit	D	1	chr6	.	CAGCGTTCGCGTTTCAAATGAACTCGGTGCTGGTAATCCACAGGCTGAAAAAAAAAAAAAAAAA
TP41350_Query	D	1	chr6	.	CAGCGTTCGCGTTTCAAACGAACTCGGTGCTGGTAATCCACAGGCTGAAAAAAAAAAAAAAAAA
TP41490_Hit	D	1	chr6	.	CAGCGTTGTCCAACTCTTTCTGTAGTTGAAGGGTGTTTAGAGTCACATCTACATGTGTTGGGGG
TP41490_Query	D	1	chr6	.	CAGCGTTGTCCAACTCTTTCTGTAGTTGAAGGGCGTTTAGAGTCACATCTACATGTGTTGGGGG
TP41768_Hit	D	1	chr6	.	CAGCTAAAACTGATGCTGACAAACCAAGCGCCTCTGAAATGGCAACATAATTAACAGCTACACA
TP41768_Query	D	1	chr6	.	CAGCTAAAACTGATGCAGACAAACCAAGCGCCTCTGAAATGGCAACATAATTAACAGCTACACA
TP41869_Hit	D	1	chr6	.	CAGCTAAACCCAATCCTTCTAAGCCTTGCCTTTTGAAGAACATCTAATGCCAAATTCCCAAAAT
TP41869_Query	D	1	chr6	.	CAGCTAAACCAAATCCTTCTAAGCCTTGCCTTTTGAAGAACATCTAATGCCAAATTCCCAAAAT
TP42050_Hit	D	1	chr6	.	CAGCTAAATGACTCGTCAGGTTTTCCACATTTTCCCTTGAAAATTGATTGTGAAGTTTGCTGAA
TP42050_Query	D	1	chr6	.	CAGCTAAATGACTCGTCAGGTTTTCCACACTTTCCCTTGAAAATTGATTGTGAAGTTTGCTGAA
TP42260_Hit	D	1	chr6	.	CAGCTAACGTAAAGCACAGTGCTTGCATTGTTGATCTTTTAGGACGTTCAGGAAGGCTGGAGGA
TP42260_Query	D	1	chr6	.	CAGCTAACGTAAAGCACAGTGCTTGCATTGTTGATCTTTTAGGACGTGCAGGAAGGCTGGAGGA

Name	Filter	Nb hit (Mt4.0)	Mt Chr (Mt4.0)	Ms Chr (Li et al., 2014)	Sequence
TP42757_Hit	D+G	1	chr6	.	CAGCTAATGTTGGAGGTTATGCTGTTGATGGTTGTAGAGAGTTCATGCCAAGTAGTGAACAAGG
TP42757_Query	D+G	1	chr6	.	CAGCTAATGTTGGAGGTTATGCTGTTGATGGTTGTAGAGAGTTCATGCCAAGTAATGAACAAGG
TP42977_Hit	D+G	1	chr6	.	CAGCTACAAGTTCTTCCTTTCCATTGTCAGTTTCTGATAGTTTTTCATCTTCCGCAACAATTTG
TP42977_Query	D+G	1	chr6	.	CAGCTACAAGTTCTTCCTTTCCATTGTCAGTTTCTGATAGTTTTTCATATTCCGCAACAATTTG
TP43515_Hit	D+G	1	chr6	.	CAGCTACTATGAAGGCTATGCAGAAAGCAACGTATGTATTGCTATTATATATGATATATGATTC
TP43515_Query	D+G	1	chr6	.	CAGCTACTATGAAGGCTATGCAGAAAGCAACGTATGTATTGCTATTATATACGATATATGATTC
TP43650_Hit	D	1	chr6	.	CAGCTACTTCAGTGATAGCGTTTTGAACGGTGGTTTCTCCATTGATCTTTGGATGTTCAAGATT
TP43650_Query	D	1	chr6	.	CAGCTACTTCAGTGATAGCGTTTTGAACGGTAGTTTCTCCATTGATCTTTGGATGTTCAAGATT
TP43916_Hit	D	1	chr6	.	CAGCTAGATGAAACGGCTTCACCATCTGAAAAATCACGTGATACTGAAGTCACCAGATGCCTAG
TP43916_Query	D	1	chr6	.	CAGCTAGATGAAACGGCTTCACCATCTGAAAAATCACGCGATACTGAAGTCACCAGATGCCTAG
TP44876_Hit	D	1	chr6	.	CAGCTATCAGAAGTTTCGCTCCATTCAGGGAATGCTTCTAGTCCCATCAACTTTGCCACAACAC
TP44876_Query	D	1	chr6	.	CAGCTATCAGAAGTTTCGCTCCAGTCAGGGAATGCTTCTAGTCCCATCAACTTTGCCACAACAC
TP45043_Hit	D	1	chr6	.	CTGCTATGACGTTGATTCTAATATGTTCTTATCATTTCTTATTAAATGTACTTGTAGATAAGTT
TP45043_Query	D	1	chr6	.	CAGCTATGACGTTGATTCTAATATGTTCTTATCATTTCTTATTAAATGTACTTGTAGATAAGTT
TP45221_Hit	D	1	chr6	.	CAGCTATGGTTATGATCCTTTTGGTCTAAGCAAGAAGCCCGAAGACTTCGCCAAGTATGTCTAA
TP45221_Query	D	1	chr6	.	CAGCTATGGTTACGATCCTTTTGGTCTAAGCAAGAAGCCCGAAGACTTCGCCAAGTATGTCTAA
TP45390_Hit	D+G	1	chr6	.	CAGCTATTCAAATGGTGAACTACCTGATGAAGTGTTTAGATCCACGGAGTATTTTCTCGGAGGT
TP45390_Query	D+G	1	chr6	.	CAGCTATTCAAATGGTGAACTACCTGATGAAGTGTTTAGATCCAAGGAGTATTTTCTCGGAGGT
TP4580_Hit	D	1	chr6	.	CAGCAACTATTGGAACTGATGTATTGCCGAAATCTCAGGATGGAACCTTGGTGAATGAATCAGT
TP4580_Query	D	1	chr6	.	CAGCAACTATTAGAACTGATGTATTGCCGAAATCTCAGGATGGAACCTTGGTGAATGAATCAGT
TP47819_Hit	D	1	chr6	.	CAGCTCCTCTGCTACCTAATCCAGATTTGTATACCATTATCCAACATAGAGGAAGATGAAGTAA
TP47819_Query	D	1	chr6	.	CAGCTCCTCTGCTACCTAATCCAGACTTGTATACCATTATCCAACATAGAGGAAGATGAAGTAA
TP47969_Hit	D	1	chr6	.	CAGCTCCTTGAGCCTTTGCTTGCATCTGTTGACCAGTCTACACATAATAAAAAAAAATATAAGC
TP47969_Query	D	1	chr6	.	CAGCTCCTTGAGCCTTTGCTTGCATCTGTTGACCAGTCTACACATAATAAAAAAAAACATAAGC
TP48426_Hit	D	1	chr6	.	CAGCTCGTAATGAGATTCCATCAGTTTGGTGGAGAGCCTTTGGAGAGGAATTACCGGTGCTGAA
TP48426_Query	D	1	chr6	.	CAGCTCGTAATGAGATTCCATCAGTTTGGTGGAGAGCCTTTGGAGAGGAACTACCGGTGCTGAA
TP48943_Hit	D	1	chr6	.	CAGCTCTGGCTTCAAGAAAGTCCTCTCCAAGAGTGACTAACGAGGCTGTTCAGAAGGCTGTGAG
TP48943_Query	D	1	chr6	.	CAGCTCTCGCTTCAAGAAAGTCCTCTCCAAGAGTGACTAACGAGGCTGTTCAGAAGGCTGTGAG
TP49483_Hit	D+G	1	chr6	.	CAGCTCTTGGGTTTGCCCAGTTTGTTCCAAATGACGACCATCTCATGACAAAGGTGCTGAAAAA
TP49483_Query	D+G	1	chr6	.	CAGCTCTTGGGTTTGCCCAGTTTGTTCCAAATGACGACAATCTCATGACAAAGGTGCTGAAAAA
TP49521_Hit	D+G	1	chr6	.	CAGCTCTTGTTGCCAATGTGATGTAGTGTTGGGGATTGGGTGGTTTGGTTTCTCAAGTCTTAAA
TP49521_Query	D+G	1	chr6	.	CAGCTCTTGTTGCCAATGTGATGTAGTGTTGGGGATTGGGTGGTTTGGTTTCTCAAGTCCTAAA
TP49806_Hit	D	1	chr6	.	CAGCTGAAAGCTGGTAGCAGACAAGGAGAACGCGAATTTCAAGCTGAAGTTGGGATTATTAGCC
TP49806_Query	D	1	chr6	.	CAGCTGAAAGCAGGTAGCAGACAAGGAGAACGCGAATTTCAAGCTGAAGTTGGGATTATTAGCC
TP50086_Hit	D	1	chr6	.	CAGCTGAAGTGAAATCCTTATTACTGAACAAGGTAAAAATTGAAAACTAATTTGTCTGTCTTTG
TP50086_Query	D	1	chr6	.	CAGCTGAAGGGAAATCCTTATTACTGAACAAGGTAAAAATTGAAAACTAATTTGTCTGTCTTTG
TP5050_Hit	D	1	chr6	.	CAGCAAGAACAAGAATTTGTTCATTAGGTCCTGATAATAGTCCATCCCAATGTGTCATGAATTC
TP5050_Query	D	1	chr6	.	CAGCAAGAACAAGAATTTGTTCATTAGGTCCAGATAATAGTCCATCCCAATGTGTCATGAATTC
TP50568_Hit	D+G	1	chr6	.	CAGCTGAGGCCATCAACTAAAAGATATTTAAAAAGTGAATCATGAACCATACTCGTGCTTATGA
TP50568_Query	D+G	1	chr6	.	CAGCTGAGGCCATCAACTAAAAGATATTTAAAAAGTGAATCATGAACCATACTCGTGCATATGA
TP5096_Hit	D+G	1	chr6	.	CAGCAAGAAGAGAGGGAAGCTGGTGATGAAGTAGCTTTTGAACTGTTTCGGTGCGTATTTATAC
TP5096_Query	D+G	1	chr6	.	CAGCAAGAAGAGAGGGAAGCTGGTGATGAAGTAGCATTTGAACTGTTTCGGTGCGTATTTATAC
TP51052_Hit	D	1	chr6	.	CAGCTGATTTGATACGACGTAGGTTATGTCATGAAAAGGCCCTTCTGATTCTTGATAATGTGGA
TP51052_Query	D	1	chr6	.	CAGCTGATTTGATACGACGTAGGTTACGTCATGAAAAGGCCCTTCTGATTCTTGATAATGTGGA
TP51756_Hit	D	1	chr6	.	CAGCTGGTATTAGTGCTTCTGGAGAAGCTTTTGCAAACTCATCTTCTCTGAGTTTGGAATGGGA
TP51756_Query	D	1	chr6	.	CAGCTGGTATTAGTGCTTCTGGAGAAGCTTTTGCAAACTCATCTTCTCTGAGTTTGAAATGGGA
TP52659_Hit	D	1	chr6	.	CAGCTGTGTACTTTCAACAAGCTGTTGATGAGGTACAGTTTTAGTTCAGTATTGATGAATGTAT
TP52659_Query	D	1	chr6	.	CAGCTGTGTACTTTCAACAAGCTGTTGATGAGGTACAGTTTTAGTTCAGCATTGATGAATGTAT
TP52933_Hit	D+G	1	chr6	.	CAGCTGTTGATAGGTTATCCAACTCAACAAACTTTCTTAACTTTTTGGCACAACATGCACTCAT
TP52933_Query	D+G	1	chr6	.	CAGCTGTTGATAGGTTATCCAACTCAACAAACTTTCTTAACTTTTTAGCACAACATGCACTCAT
TP53577_Hit	D	1	chr6	.	CAGCTTACAGATAGTGAGACTAGCGTCTTGTCCTTGCATCTTGTACGTGAGATTCCGTCAATTG
TP53577_Query	D	1	chr6	.	CAGCTTACAGACAGTGAGACTAGCGTCTTGTCCTTGCATCTTGTACGTGAGATTCCGTCAATTG
TP53811_Hit	D	1	chr6	.	CAGCTTAGGGTTTATGGCGTGCTTTTTTGTAATCAAGTACATCACTAGATGACAAGTTTGCAAC
TP53811_Query	D	1	chr6	.	CAGCTTAGGGTTTATAGCGTGCTTTTTTGTAATCAAGTACATCACTAGATGACAAGTTTGCAAC

Name	Filter	Nb hit (Mt4.0)	Mt Chr (Mt4.0)	Ms Chr (Li et al., 2014)	Sequence
TP54680_Hit	D+G	1	chr6	.	CAGCTTCACACTCGAATGCTTTTCATTCTCACACGGTGTACTAGATTGTTGCAATTTCACAAGG
TP54680_Query	D+G	1	chr6	.	CAGCTTCACACTCGAATGCTTTTCATTCTCACACGGTGCACTAGATTGTTGCAATTTCACAAGG
TP54866_Hit	D	1	chr6	.	CAGCTTCAGCTGTTTCAAAGGTGCCAAGCCACACTCTTGCTCCTTTTTTAGGATCTCTTATTTC
TP54866_Query	D	1	chr6	.	CAGCTTCAGCTGTTTCAAAGGTGCCAAGCCACACTCTTGCTCCTTTTTTAGGATCTCGTATTTC
TP55085_Hit	D	1	chr6	.	CAGCTTCATGGTGTGGATGATAACATAGGCAAGAGCAAGAAAATTATGACAAACATGTCTAGAA
TP55085_Query	D	1	chr6	.	CAGCTTCATGGCGTGGATGATAACATAGGCAAGAGCAAGAAAATTATGACAAACATGTCTAGAA
TP5515_Hit	D	1	chr6	.	CAGCAAGATTCTCTCTATTCTCTGACTCCATTTTCAAATTTCAATTACTTTATATTTCATTCTT
TP5515_Query	D	1	chr6	.	CAGCAAGATTCTCTCTATTCTCTGACTCCATTTTCAAATTTCAATTACTTTATATTTCATTCAT
TP55308_Hit	D	1	chr6	.	CAGCTTCCCATCCCAGGCCTGTAATGCTGGGAGCCAGTTTTTGATTATGATGTGTGTTCTGCAG
TP55308_Query	D	1	chr6	.	CAGCTTCCCATCCCAGGCCCGTAATGCTGGGAGCCAGTTTTTGATTATGATGTGTGTTCTGCAG
TP5533_Hit	D	1	chr6	.	CAGCAAGATTTTCTGATCTACCAGAGTTACGTGACCTTCGGCAAATATTTCAGGAGAGATACGG
TP5533_Query	D	1	chr6	.	CAGCAAGATTTTCTGATCTACCAGAGTTACGCGACCTTCGGCAAATATTTCAGGAGAGATACGG
TP5550_Hit	D+G	1	chr6	.	CAGCGAGCAAAGCGGATTCATTGACGATGTTTGCAAGATCAGCACCTACGAGACCATCAGTAAC
TP5550_Query	D+G	1	chr6	.	CAGCAAGCAAAGCGGATTCATTGACGATGTTTGCAAGATCAGCACCTACGAGACCATCAGTAAC
TP55722_Hit	D	1	chr6	.	CAGCTTCTAAGGGTAGCGAATTAAGTGGTTTTTCTGATAAAAATAATTTGCCTGCTTCTGTAAG
TP55722_Query	D	1	chr6	.	CAGCTTCTAAGGGTAGCGAATTAAGTGGTTTTTCAGATAAAAATAATTTGCCTGCTTCTGTAAG
TP56381_Hit	D	1	chr6	.	CAGCTTCTGCTGAGAAATCTCAGCTTTAAGCTTCCCAAGTTCAGCATTTAAATTCTCCAAACTC
TP56381_Query	D	1	chr6	.	CAGCTTCTGCTGAGAAATCTCACCTTTAAGCTTCCCAAGTTCAGCATTTAAATTCTCCAAACTC
TP56385_Hit	D	1	chr6	.	CAGCTTCTGCTGTGTCAAATGTTCCTAGCCATACTCTTTGTGCTTTGTGTGGATCTCTTATTTC
TP56385_Query	D	1	chr6	.	CAGCTTCTGCTGTATCAAATGTTCCTAGCCATACTCTTTGTGCTTTGTGTGGATCTCTTATTTC
TP56845_Hit	D+G	1	chr6	.	CAGCTTGAACTTAACAATTTCCGGTAACGGAACAGAGACAATAAACAATGTATGCGGCGGAGTG
TP56845_Query	D+G	1	chr6	.	CAGCTTGAACTTAACAATTTCCGGTAACGGAACAGAAACAATAAACAATGTATGCGGCGGAGTG
TP57257_Hit	D+G	1	chr6	.	CAGCTTGATTGAGTTGCTGAGAAACTGTTGTTATGGCTTTAAATAAGACAGGAAAATTGATAGG
TP57257_Query	D+G	1	chr6	.	CAGCTTGATTGAGTTGCTGAGAAACTGTTGCTATGGCTTTAAATAAGACAGGAAAATTGATAGG
TP57261_Hit	D	1	chr6	.	CAGCTTGATTGGTAGTATCAAAAGAAGTTTGTTGTTGAGTATCAGTCTTGTTATTATTATTATT
TP57261_Query	D	1	chr6	.	CAGCTTGATTGGTAGTATCAAAAGAAGTTTGTTGTTGAGCATCAGTCTTGTTATTATTATTATT
TP57288_Hit	D	1	chr6	.	CAGCTTGCAAAAGCAGACAGTGAAAACTTCGACAAGCTTATTAGTGTCAACAACTACAGCTTGT
TP57288_Query	D	1	chr6	.	CAGCTTGCAAAAGCAGACAGTGAAAAATTCGACAAGCTTATTAGTGTCAACAACTACAGCTTGT
TP57617_Hit	D	1	chr6	.	CAGCTTGGAAATGCATCCAGCTTCGCAGTACAAGGGTCTTACTTCATCTAATTGTCTTCCATCA
TP57617_Query	D	1	chr6	.	CAGCTTGGAAATACATCCAGCTTCGCAGTACAAGGGTCTTACTTCATCTAATTGTCTTCCATCA
TP58537_Hit	D	1	chr6	.	CAGCTTTGAGGACTAAAATGGTTGATTCACTCCTTATCATTTGATTAATTATGCTTAATAGGCA
TP58537_Query	D	1	chr6	.	CAGCTTTAAGGACTAAAATGGTTGATTCACTCCTTATCATTTGATTAATTATGCTTAATAGGCA
TP58599_Hit	D	1	chr6	.	CAGCTTTACAGCACTGTTACTCTAAGGCAAGGTGAGAAGAAGCATTACAATCATAAAGTGGATG
TP58599_Query	D	1	chr6	.	CAGCTTTACAGCACTGTTACTCTAAGACAAGGTGAGAAGAAGCATTACAATCATAAAGTGGATG
TP58917_Hit	D+G	1	chr6	.	CAGCTTTCAACATGGTCAGGAAGAGAAGAACTGATGGACCCAGTGGTGCTGAAGGTTCCGAAGG
TP58917_Query	D+G	1	chr6	.	CAGCTTTCAACATGGTCAGGAAGAGAAGAACTGATGGACCCAGTGGTGATGAAGGTTCCGAAGG
TP58972_Hit	D	1	chr6	.	CAGCTTTCACGTCCTTAATGGGAGCGCTTGTTCTTTCTCTCAATTGTTTGATCAGATTAATTTC
TP58972_Query	D	1	chr6	.	CAGCTTTCACGTCCTGAATGGGAGCGCTTGTTCTTTCTCTCAATTGTTTGATCAGATTAATTTC
TP59088_Hit	D	1	chr6	.	CAGCTTTCCCGCTTTCCGACTAACGGCTATCCCCAGTTTTAGCGTCGGCTAATCCGTGCCGCTA
TP59088_Query	D	1	chr6	.	CAGCTTTCCCGCTTTCCGACTAACGGCTATCCCCAGTTTTAGCGTCGGCCAATCCGTGCCGCTA
TP59283_Hit	D+G	1	chr6	.	CAGCTTTCTTTGATGTGCATTGGCCAAGATGTTCATGGTAACATTAAATTATCCCTCAAAGCGG
TP59283_Query	D+G	1	chr6	.	CAGCTTTCTTTGATGTGCATTGGCCAAGATGTTCATGGTAACATTAAATTATCACTCAAAGCGG
TP59363_Hit	D	1	chr6	.	CAGCTTTGACATATAAGCAGAAGTATGCCAACTGCATGCTCTTTTCCTAGTGAAGTTCCATCTT
TP59363_Query	D	1	chr6	.	CAGCTTTGACATATAAGCAGAAGTATGCCAACTGAATGCTCTTTTCCTAGTGAAGTTCCATCTT
TP5971_Hit	D	1	chr6	.	CAGCAAGGACATATATCATATCAACTTGTCTGTGTGGTTTTAGCTTTTAAGAGGCTTTTACGAT
TP5971_Query	D	1	chr6	.	CAGCAAGGACATATATCATATCAACTTGTCCGTGTGGTTTTAGCTTTTAAGAGGCTTTTACGAT
TP59784_Hit	D	1	chr6	.	CAGCTTTGTTTAAAATTACTAAATTGAGTTGAAATGCTGATATTTTTTATGGTCGGGGCATGTG
TP59784_Query	D	1	chr6	.	CAGCTTTGTTTAAAATTACTAAATTGAGTTGAAATGCTGATATTTTTTATGATCGGGGCATGTG
TP5982_Hit	D	1	chr6	.	CAGCAAGGACTATTACTTGACAGATTCTGATGATGATAAAGTTAAAGACTTTGAATTGTTTTGC
TP5982_Query	D	1	chr6	.	CAGCAAGGACTATTACTTGACAGATTCTGATGATGATAAAGTTAAAGACTTTGAATTGTTCTGC
TP59850_Hit	D	1	chr6	.	CAGCTTTTACGGGGAAAGTAAAATCTATCCAACCGTTTGGTGCGTTTGTTGATTTCGGAGCTTT
TP59850_Query	D	1	chr6	.	CAGCTTTTACGGGGAAAGTAAAATCTATCCAACCATTTGGTGCGTTTGTTGATTTCGGAGCTTT
TP60809_Hit	D	1	chr6	.	CTGCAAAAGTCTTTTATACGACTCGGTCACCAAATCAAAAGAATCAACACTATGAGTATAATCC
TP60809_Query	D	1	chr6	.	CTGCAAAAGTCGTTTATACGACTCGGTCACCAAATCAAAAGAATCAACACTATGAGTATAATCC

Name	Filter	Nb hit (Mt4.0)	Mt Chr (Mt4.0)	Ms Chr (Li et al., 2014)	Sequence
TP61168_Hit	D	1	chr6	.	CTGCAAACTGACTCTGTGGGCTTTGATTTACAAATGAAGTTAAAGGGCCTGTAGTGTATAGAGA
TP61168_Query	D	1	chr6	.	CTGCAAACTGACTCTGTGGGCTTTGATTTACAAATGAAGTTAAAGGGCCTATAGTGTATAGAGA
TP61408_Hit	D+G	1	chr6	.	CTGCAAAGTAGATAGCTCCCATAATATGATCCCAAAGCTATAAACATCACACCTGGATAATTGA
TP61408_Query	D+G	1	chr6	.	CTGCAAAGTAGATAGCTCCCATAATATGATCCCAAAGCTATAAACATCACACCTGGATAATGGA
TP61513_Hit	D+G	1	chr6	.	CTGCAAATAGAAGCAACACAGCAGGAAATTCCAACAAATCATCTTTGGGAGAAGAAAGAGATGG
TP61513_Query	D+G	1	chr6	.	CTGCAAATAGAAGCAACAAAGCAGGAAATTCCAACAAATCATCTTTGGGAGAAGAAAGAGATGG
TP61798_Hit	D	1	chr6	.	CTGCAAATTATATAACAACGATTTTGGAAGGCGATAGTTTTTCTACAAATTCAGTTTACTACTA
TP61798_Query	D	1	chr6	.	CTGCAAATTATATAACAACGATTCTGGAAGGCGATAGTTTTTCTACAAATTCAGTTTACTACTA
TP62263_Hit	D	1	chr6	.	CTGCAACATCAGAAGCAATTCCAGCACTGCGGAAGACAGAAGTTGAATAATACACCACAGCATT
TP62263_Query	D	1	chr6	.	CTGCAACATCAGAAGCAATTCCAGCACTACGGAAGACAGAAGTTGAATAATACACCACAGCATT
TP62716_Hit	D	1	chr6	.	CTGCAACTCCATCATTGACTTGTGGATATCACCGCTGATATAAAATTTATTGCCATCATTATGA
TP62716_Query	D	1	chr6	.	CTGCAACTCCATCAGTGACTTGTGGATATCACCGCTGATATAAAATTTATTGCCATCATTATGA
TP62954_Hit	D	1	chr6	.	CTGCAAGAAAAAAGACCTTCGACTAGAGGCACCGTATGAGTGAAGCATTGCATGATTGAATTTA
TP62954_Query	D	1	chr6	.	CTGCAAGAAAAAAGACCTTCGACTAGAGGCACCGTATGAGTGAAGCATTGCATGATTGAATTGA
TP63187_Hit	D+G	1	chr6	.	CTGCAAGATCGTATAAGTCCATTTTCCACAGAAGTTGCTTTCAACATGATTGAACAAGAACTTG
TP63187_Query	D+G	1	chr6	.	CTGCAAGATCGAATAAGTCCATTTTCCACAGAAGTTGCTTTCAACATGATTGAACAAGAACTTG
TP63563_Hit	D	1	chr6	.	CTGCAAGTCAGTGCAGAGTTCTGCTATATTAGGAAATCAAATAATCAAACTAAAAGATTCATTT
TP63563_Query	D	1	chr6	.	CTGCAAGTCAGTGCAGAGTTCTGATATATTAGGAAATCAAATAATCAAACTAAAAGATTCATTT
TP64750_Hit	D	1	chr6	.	CTGCAATTGCAGTTGCATTTGTACTTTGCGCCTCAGATTCAAATTGGGTTTTGGGATAGTGGTG
TP64750_Query	D	1	chr6	.	CTGCAATTGCAGTTGCATTTGTACTTTGCGCCTCAGATGCAAATTGGGTTTTGGGATAGTGGTG
TP65253_Hit	D+G	1	chr6	.	CTGCACAATTTAAGGTTTCAAGTTCAACTCTTCCATATACTCCACAAAACTGTGGCAATCAATT
TP65253_Query	D+G	1	chr6	.	CTGCACAATTTAAGGTTTCAAGTTCAACTCTTCCATATACTCCACAAAACAGTGGCAATCAATT
TP6562_Hit	D	1	chr6	.	CAGCAAGTTCAGTTGAACCAACAAAATGACCAATAAAAGCTTGAGTTATTATGTATGTGCCAAA
TP6562_Query	D	1	chr6	.	CAGCAAGTTCAGTTGAACCAACAAAATGACCAATAAAAGCTTGAGTTATTACGTATGTGCCAAA
TP6563_Hit	D	1	chr6	.	CAGCAAGTTCAGTTGAACCAACAACATGACCAATAAAAGCTTGCGTTATTATATATGTGCCAAA
TP6563_Query	D	1	chr6	.	CAGCAAGTTCAGTTGAACCAACAAAATGACCAATAAAAGCTTGCGTTATTATATATGTGCCAAA
TP67894_Hit	D	1	chr6	.	CTGCAGAGAAGCTATGGTCTGGACTGGAAAATTTTGTTTTTGATTGGCTTATAAATGCTGACAG
TP67894_Query	D	1	chr6	.	CTGCAGAGAAGCTATGGTCTGGACTGGAAAATTTTGTTTTTGACTGGCTTATAAATGCTGACAG
TP67970_Hit	D	1	chr6	.	CTGCAGAGGATCCGGGCCAGAACGAAAAGGAATATGTAATCTATGTTTACGGGATTAGTCTTTT
TP67970_Query	D	1	chr6	.	CTGCAGAGGATCCAGGCCAGAACGAAAAGGAATATGTAATCTATGTTTACGGGATTAGTCTTTT
TP6901_Hit	D	1	chr6	.	CAGCAATACCAAGCATGATTCTCCAATTCAAACCAATTGGAAGAGAAGAAAGAGCATAGTTTGA
TP6901_Query	D	1	chr6	.	CAGCAATACCAAGCATGATTCTCCAATTCAAACCAATAGGAAGAGAAGAAAGAGCATAGTTTGA
TP69117_Hit	D	1	chr6	.	CTGCAGGCAAAACTGTTGGACGGTACTCAATATTTGAATTGAGACCAGGAAACACCCTTGACAC
TP69117_Query	D	1	chr6	.	CTGCAGGCAAAACTGGTGGACGGTACTCAATATTTGAATTGAGACCAGGAAACACCCTTGACAC
TP69632_Hit	D	1	chr6	.	CTGCAGTATCCAACAGGAGAGACAAGATGTCTGTGATGAACACGGCTAATAATCCCAACTTCAG
TP69632_Query	D	1	chr6	.	CTGCAGTATCCAACAAGAGAGACAAGATGTCTGTGATGAACACGGCTAATAATCCCAACTTCAG
TP69697_Hit	D+G	1	chr6	.	CTGCAGTCATGGAAGAATTTTCATCAAATTGCCCTTGTTGATTTGCCGATGGTACAGTCAGAGA
TP69697_Query	D+G	1	chr6	.	CTGCAGTCATGGAAGAATTTTCATCAAATTGCCCTTGTTGATTTGCCGATGGTACAGTAAGAGA
TP7022_Hit	D	1	chr6	.	CAGCAATAGGAGATTTTGTGTTGACTTTTACATGGATGTTTATATCTTCAACACTTGGATTAGT
TP7022_Query	D	1	chr6	.	CAGCAATAGGAGATTTTGTGTTGACTTTCACATGGATGTTTATATCTTCAACACTTGGATTAGT
TP70479_Hit	D	1	chr6	.	CTGCATACAGAGATGCAGTGTACATGTTGCCAACTTGCTTTGGGATTAATGTGCTCGGTTGTAC
TP70479_Query	D	1	chr6	.	CTGCATACAGAGATGCAGTGTACATGTTGCCAACTTGCTTCGGGATTAATGTGCTCGGTTGTAC
TP70747_Hit	D+G	1	chr6	.	CTGCATATGAAACTTTGCAGGTAGCGAAAGTGAATTTGCATGTTGAATTGAATATTGATGTAGC
TP70747_Query	D+G	1	chr6	.	CTGCATATGAAACTTTGCAGGTAGCGAAAGTGAATTTGCATGTTGAATTGAATATTGATATAGC
TP71139_Hit	D+G	1	chr6	.	CTGCATCATTGTTTAACCCTCAACTTGAAAACATGCTTCTAGGACTTAACAGGAAACTTGGCAG
TP71139_Query	D+G	1	chr6	.	CTGCATCATTGTTTAACCCTCAACTTGAAAACATGCTTCTAGGACTTAACAAGAAACTTGGCAG
TP71305_Hit	D	1	chr6	.	CTGCATCGACAATTTCAGGAGTCAAACATATCTGTTCATGGACTGCAGATAGCTTGCCATCTGC
TP71305_Query	D	1	chr6	.	CTGCATCGACAATTTCAGGAGTCAAACATATCTGTTCATGGACTGCAGAGAGCTTGCCATCTGC
TP728_Hit	D	1	chr6	.	CAGCAAAAGGTCTGGGAATTGATTTGCAGATTTTCTTCTCCTACTCACCTGAGTTGACCAATGA
TP728_Query	D	1	chr6	.	CAGCAAAAGGTCTGGGAATTGATTTGCAAATTTTCTTCTCCTACTCACCTGAGTTGACCAATGA
TP72992_Hit	D	1	chr6	.	CTGCATTTGAAATAAGTTCTCAGGATTCAAATGATTTAACTCGTAAGAGGCATTCAACACAAGG
TP72992_Query	D	1	chr6	.	CTGCATTTGAAATAAGTTCTCAGGATTCAAATGATTTAACTCGCAAGAGGCATTCAACACAAGG
TP73686_Hit	D	1	chr6	.	CTGCCAAGAAGAAACTCTGATTTGAACAATGAGCATTTTACCTCAGCATCAGATTTTATAGTTA
TP73686_Query	D	1	chr6	.	CTGCCAAGAAGAAACTCTAATTTGAACAATGAGCATTTTACCTCAGCATCAGATTTTATAGTTA

Name	Filter	Nb hit (Mt4.0)	Mt Chr (Mt4.0)	Ms Chr (Li et al., 2014)	Sequence
TP75736_Hit	D+G	1	chr6	.	CTGCCATTTGTGGTTCTTCATAGGCCTCTAACCATATTTTGCTTTTCTTCCCTTGATCCTTTAT
TP75736_Query	D+G	1	chr6	.	CTGCCATTTGTGGTTCTTCATAGACCTCTAACCATATTTTGCTTTTCTTCCCTTGATCCTTTAT
TP75876_Hit	D+G	1	chr6	.	CTGCCCAAGTTTTATAAAACACTACAAGGCCATATCTCTAAACAATGACGGACTAAATCCACCC
TP75876_Query	D+G	1	chr6	.	CTGCCCAAGTTTTATAAAACACTACAAGGCCATATCTCTAAACAATGACGGACTAAATCCAACC
TP7677_Hit	D	1	chr6	.	CAGCGATGAGTCATTCTCATAGTCAAATCCTGGGCTTGCCAATTGTTCCAAACAATGTTTCTGC
TP7677_Query	D	1	chr6	.	CAGCAATGAGTCATTCTCATAGTCAAATCCTGGGCTTGCCAATTGTTCCAAACAATGTTTCTGC
TP76942_Hit	D	1	chr6	.	CTGCCCTTTGAATGTCTTTTGCCTCAGCGGTGGCAGGAATAGGAAGCCTCCACGCCGAGTCTGC
TP76942_Query	D	1	chr6	.	CTGCCCTTTGAATGTCTTTTGCCTCAGCGGTGGCAGGAATAGGAAGCCTCCACACCGAGTCTGC
TP7724_Hit	D	1	chr6	.	CAGCAATGATTGTCCATACAAAATAAAACAATCGTAAAAGCCTCTTAAAAGCTAAAACCACACG
TP7724_Query	D	1	chr6	.	CAGCAATGATTGTCCATACAAAATAAAACAATCGTAAAAGCCTCTTAAAAGCTAAAACCACACA
TP77514_Hit	D	1	chr6	.	CTGCCGCTGGGATGTTGATGGGGAAAAGGACTAGGCTGAAAAAAAAAAAAAAAAAAAAAAAAAA
TP77514_Query	D	1	chr6	.	CTGCCGCTGGGATGTTGATGGCGAAAAGGACTAGGCTGAAAAAAAAAAAAAAAAAAAAAAAAAA
TP7756_Hit	D	1	chr6	.	CAGCAATGCAGTATGCCAATCATTTGCTATGAAGACCAGATTTCCATCTCCATAGCAAACGCCG
TP7756_Query	D	1	chr6	.	CAGCAATGCAGTATGCCAATCATTTGCTATGAAGACCAGATTTCCATCTCCATAACAAACGCCG
TP7783_Hit	D	1	chr6	.	CAGCAATGCCCGCCGCAAGTGCTTCAGCCCCTGAATCAAAAGACTCATAAGGGATGTACAGTCC
TP7783_Query	D	1	chr6	.	CAGCAATGCCCGCCGCAAGTGCTTCAGCCCCTGAATCAAAAGACTCATAAGGGATGGACAGTCC
TP77878_Hit	D	1	chr6	.	CTGCCGTCACCTCGTTGGTCCACGTGAGACCTTTACTCTCAATTTCCTCTTTCTCCTCTCAACT
TP77878_Query	D	1	chr6	.	CTGCCGTCACCTCGTTGGTCCACCTGAGACCTTTACTCTCAATTTCCTCTTTCTCCTCTCAACT
TP78148_Hit	D	1	chr6	.	CTGCCTACCAACTCACTCAAATCTGACTGCACGTTCAATGCCCTGTAGCGTGCGAATTGTGTAA
TP78148_Query	D	1	chr6	.	CTGCCTACCAACTCACTCAAATCTGACTGCACGTTCAATGCCCTGTAGCGTGCAAATTGTGTAA
TP7843_Hit	D	1	chr6	.	CAGCAATGGACTTGATTAATAAACCAAAAACTCCATCATCCAGAGAGTTGGTCATTTCCCCTGT
TP7843_Query	D	1	chr6	.	CAGCAATGGACTTGATTAATAAACCAAAAACTCCATCATCCAGAGAGTTAGTCATTTCCCCTGT
TP7941_Hit	D	1	chr6	.	CAGCAATGGTGGCAGATAGAAGAAGAACACCAACTGCTAAACCAGCTATTTCACTTGCTGTTAT
TP7941_Query	D	1	chr6	.	CAGCAATGGTGGCAGAAAGAAGAAGAACACCAACTGCTAAACCAGCTATTTCACTTGCTGTTAT
TP79568_Hit	D	1	chr6	.	CTGCCTTTTCCGATTACTTTGACAGTCTCAAGGTCATCCAATGTGAAGTCAAAGTCGAGATCCT
TP79568_Query	D	1	chr6	.	CTGCCTTTTCCGATTACTTTGACAGTCTCAAGGTCATCCAACGTGAAGTCAAAGTCGAGATCCT
TP80248_Hit	D+G	1	chr6	.	CTGCGAGAGCTTTTTTATTATGTCTGGTTGGTTGCACGCTATTTAGTGACAAGAGTGCATTCGC
TP80248_Query	D+G	1	chr6	.	CTGCGAGAGCTTTTTTATTATGTCTGGTTGGGTGCACGCTATTTAGTGACAAGAGTGCATTCGC
TP80530_Hit	D+G	1	chr6	.	CTGCGATCCGGAGCACAAAAACAAGGTTTTTCATGTCTCAGCCATCCGAACACAATTGGTGCTG
TP80530_Query	D+G	1	chr6	.	CTGCGATCCGGACCACAAAAACAAGGTTTTTCATGTCTCAGCCATCCGAACACAATTGGTGCTG
TP80674_Hit	D+G	1	chr6	.	CTGCGATTATGGAAATATTGAGGTCTCAACAGCAATTTCGACTGCATCAACCGAACTTTCATTA
TP80674_Query	D+G	1	chr6	.	CTGCGATTATGGAAATATTGAGGTCTCAACAGCAATTTCGACCGCATCAACCGAACTTTCATTA
TP81049_Hit	D	1	chr6	.	CTGCGCCAACGGTTTGTGGTATTGTTATTGGGGCAATGGCTGTTGCACAGGTGTTTTCTTCTGT
TP81049_Query	D	1	chr6	.	CTGCGCCAACGGTTTGCGGTATTGTTATTGGGGCAATGGCTGTTGCACAGGTGTTTTCTTCTGT
TP81998_Hit	D	1	chr6	.	CTGCGGATAAAATTCACGGTGAGGTTTTAAAACCTGCTCGGGAGTTGCACGCATATACTTTGAT
TP81998_Query	D	1	chr6	.	CTGCGGATAAAATTCACGGCGAGGTTTTAAAACCTGCTCGGGAGTTGCACGCATATACTTTGAT
TP82000_Hit	D	1	chr6	.	CTGCGGATAAGTGCTACAAACCCCTTTTTTGGCTTCAATCATCAAGTCCTTACAGCTAATTATT
TP82000_Query	D	1	chr6	.	CTGCGGATAAGTGCTACAAACCCCTTTTTTGGCTTCAATCATCAACTCCTTACAGCTAATTATT
TP82410_Hit	D	1	chr6	.	CTGCGGCTTGAATATCTCTTGGCGAGTTAGAAGCTGGTCGAGGAAGTGAAGCTGAAAGTTCAGG
TP82410_Query	D	1	chr6	.	CTGCGGCTTGAATATCTCTTGGCGAGTTAGAAGCTGGTCGAGGAAGGGAAGCTGAAAGTTCAGG
TP83095_Hit	D+G	1	chr6	.	CTGCGTAGAATATACCAAAATGGTGGGTCCCCTTCCCAGACCCTGCGTATGTGGGAGCTTTAGT
TP83095_Query	D+G	1	chr6	.	CTGCGTAGAATATACCAAAATGGTGGGTCCCCTTCCCAGACCCTACGTATGTGGGAGCTTTAGT
TP83325_Hit	D	1	chr6	.	CTGCGTTGAGGCCTGAGGGAACGATGATGGCACCGTTGATGGTTCAGCCGGTGCTGAAAAAAAA
TP83325_Query	D	1	chr6	.	CTGCGTCGAGGCCTGAGGGAACGATGATGGCACCGTTGATGGTTCAGCCGGTGCTGAAAAAAAA
TP83433_Hit	D	1	chr6	.	CTGCGTCTTGGTTCCCAAGTCTATGGCATGCCACTTCTTTTTGTAGTACTTCCAAAGCTCGGTT
TP83433_Query	D	1	chr6	.	CTGCGTCTTGGTTCCCAAGTCTATGACATGCCACTTCTTTTTGTAGTACTTCCAAAGCTCGGTT
TP83739_Hit	D+G	1	chr6	.	CTGCGTTAGACATGTTTGGTCATTGTCAGTCACATGTTACTGTGTTTGGAAGGTTTTTCTTCAT
TP83739_Query	D+G	1	chr6	.	CTGCGTTAGACATGTTTGGTCATTGTCAGTCACATGTTACTGTGTTAGGAAGGTTTTTCTTCAT
TP83755_Hit	D	1	chr6	.	CTGCGTTAGGGACAAAAATCCCTACGATACGTCTGTTATCTGATGTAGTTTCCACACGAACAAC
TP83755_Query	D	1	chr6	.	CTGCGTTAGGGACAAAAATCCCTACAATACGTCTGTTATCTGATGTAGTTTCCACACGAACAAC
TP84064_Hit	D	1	chr6	.	CTGCGTTTTTTCTGTCTCATTAGCGAGGCTGGTTGGAGTATATTGTCATCGTCTCAAGCTTGGT
TP84064_Query	D	1	chr6	.	CTGCGTTTTTTCTGTCTCATTAGCGAGGCTGGTTGGAGTATATGGTCATCGTCTCAAGCTTGGT
TP8423_Hit	D	1	chr6	.	CAGCAATTGCCAAACCTTAACACACTTATCAACCCCATTTCCACCAAATGGAAAACACTCTATC
TP8423_Query	D	1	chr6	.	CAGCAATTGCCAAACCTTAACACACTTATCAACCCCATTTCCACCAAACGGAAAACACTCTATC

Name	Filter	Nb hit (Mt4.0)	Mt Chr (Mt4.0)	Ms Chr (Li et al., 2014)	Sequence
TP84505_Hit	D	1	chr6	.	CTGCTAAGTTCCCATTTCAAACTCAGAGAAGATGAGTTTGCAAAAGCTTCTCCAGAAGCACTAA
TP84505_Query	D	1	chr6	.	CTGCTAAGTTCCCATTCCAAACTCAGAGAAGATGAGTTTGCAAAAGCTTCTCCAGAAGCACTAA
TP8566_Hit	D	1	chr6	.	CAGCAATTTCCCTGCGAATAGATATTACATGCACATGCATGAAATAAACATGCTCTCAAAAGGA
TP8566_Query	D	1	chr6	.	CAGCAATTTCCCTGCGAATAGATATTACATGCACATGCATGAAATAAACATGCTCTCAAAAGAA
TP8579_Hit	D	1	chr6	.	CAGCAATTTCTGAAAGATATACTGAAAGATCTTTGTGTTTACAATAACAAAGGAACTAATCAAG
TP8579_Query	D	1	chr6	.	CAGCAATTTCTGAAAGATATACTGAAAGATCTTTGTGTCTACAATAACAAAGGAACTAATCAAG
TP8598_Hit	D	1	chr6	.	CAGCAATTTGCAAGCGAAACTTGTCTCGTCTCGATGGAGAATTAACCGGTGCTGAAAAAAAAAA
TP8598_Query	D	1	chr6	.	CAGCAATTTGCAAGCGAAACTTGTCTCATCTCGATGGAGAATTAACCGGTGCTGAAAAAAAAAA
TP87087_Hit	D	1	chr6	.	CTGCTCAACGGCGTACGGCTGACGACAACGCAAGGCCGTGTACCGAAAAGCTGTGATCGGAAGA
TP87087_Query	D	1	chr6	.	CTGCTCAACGGCGTACGGCTGACGACAACGCAAGGCCGTGTACCGAAAAGCTGGGATCGGAAGA
TP88269_Hit	D+G	1	chr6	.	CTGCTCCTTTGATTGATTTGTAGAAATGGAATCATTCCCATCATTTTTGGCTACAGGATCAACT
TP88269_Query	D+G	1	chr6	.	CTGCTCCTTTGATTGATTTGTAGAAATGGAATCACTCCCATCATTTTTGGCTACAGGATCAACT
TP88707_Hit	D	1	chr6	.	CTGCTCTAATCGAGCTGTGAAATCGGTGTGGTGAGGTATTGGAAGTGTTCAGCGAATATAGATA
TP88707_Query	D	1	chr6	.	CTGCTCTAATCGAGCTGTGAAATCGGTGTGGTGAGGTATTGGAAGTGTTCAACGAATATAGATA
TP89675_Hit	D+G	1	chr6	.	CTGCTGAATACAAGAGGCCTAAAGTATGATTTGTTCGACCATGCACTAAAATACACAGAAGAAA
TP89675_Query	D+G	1	chr6	.	CTGCTGAATACAAGAGGCCTAAAGTACGATTTGTTCGACCATGCACTAAAATACACAGAAGAAA
TP917_Hit	D	1	chr6	.	CAGCAAAATCTAAGAAAAATGAAAGTTCCATTTCTTGTTCTACACGGCACTGGTGATTGTGTGA
TP917_Query	D	1	chr6	.	CAGCAAAATCTAAGAAAAATGAAAGTTCCATTTCTTGTTCTACACGGCACCGGTGATTGTGTGA
TP9277_Hit	D	1	chr6	.	CAGCACACATCATTACACCTCCATAAGCAACAGATTGAACTGCCGAATCAAGAAACACAGAAGG
TP9277_Query	D	1	chr6	.	CAGCACACATCATTACACCTCCATAAACAACAGATTGAACTGCCGAATCAAGAAACACAGAAGG
TP93339_Hit	D	1	chr6	.	CTGCTTCATCCAGAAACCCTTCTTCCTGATGACCATGAGACGGTTGATTACATGTTATTTTGGT
TP93339_Query	D	1	chr6	.	CTGCTTCATCCAGAAACCCTTCTTCCTGATGACCATGAGACGCTTGATTACATGTTATTTTGGT
TP9334_Hit	D+G	1	chr6	.	CAGCACACCCTTTGATGCTTCTGGGTATGCTTCTGCGTCCATCGCACCCCATCTTAGTAGAAGG
TP9334_Query	D+G	1	chr6	.	CAGCACACCCTTTGATGCTTCTGGGTATGCTTCTGCATCCATCGCACCCCATCTTAGTAGAAGG
TP93844_Hit	D	1	chr6	.	CTGCTTCTGCAATATGCTTAGCCTTCAACACAACACCCTTCAGATTACCCTTAGAACTTACGAT
TP93844_Query	D	1	chr6	.	CTGCTTCTGCAATATGCCTAGCCTTCAACACAACACCCTTCAGATTACCCTTAGAACTTACGAT
TP94125_Hit	D+G	1	chr6	.	CTGCTTGAGCCAGACGGGTTTAAACCGTATTCACTTGTAGCTCCACTGTACTCGCTGAAAAAAA
TP94125_Query	D+G	1	chr6	.	CTGCTTGAGCCAGACGGGTTTAAACCGTATTCACTAGTAGCTCCACTGTACTCGCTGAAAAAAA
TP94506_Hit	D	1	chr6	.	CTGCTTGTAGATTAGACATGCCTTCAAAGGGCAACTTGTTATGGATGAGCTCCCACAACACAAT
TP94506_Query	D	1	chr6	.	CTGCTTGTACATTAGACATGCCTTCAAAGGGCAACTTGTTATGGATGAGCTCCCACAACACAAT
TP9485_Hit	D+G	1	chr6	.	CAGCACAGATGGTTCTACCGGTGTTCTATGATGTTGATCCCTCGGAGGTACGTCACCAAACAGG
TP9485_Query	D+G	1	chr6	.	CAGCACAGATAGTTCTACCGGTGTTCTATGATGTTGATCCCTCGGAGGTACGTCACCAAACAGG
TP9884_Hit	D	1	chr6	.	CAGCACATTAAGAACCTTTTGTGTCCATCAACTCCATTGTTTTTCAATACCCTTTATGATCCTT
TP9884_Query	D	1	chr6	.	CAGCACATTAAGAACCTTTTGTGTCCATCAACTCCATTGTTTTTCAACACCCTTTATGATCCTT
TP81579_Hit	D+G	1	chr6	2D	CTGCGTTAGGAACAAAAATCCCTACGGTACGTCTGTTATCTGATGTAGTTTCCACACGAACAAC
TP81579_Query	D+G	1	chr6	2D	CTGCGCTAGGAACAAAAATCCCTACGGTACGTCTGTTATCTGATGTAGTTTCCACACGAACAAC
TP3726_Hit	D+G	1	chr6	4A	CAGCAACATCCACAAGCTTCTTATTAGCCAGTAGTGATAAAAGAACAGTGTAAGTGCTTAGGCC
TP3726_Query	D+G	1	chr6	4A	CAGCAACATCCACAAGCTTCTTATTAGCCAGTAGAGATAAAAGAACAGTGTAAGTGCTTAGGCC
TP5060_Hit	D+G	1	chr6	6A	CAGCAAGAACAGGGTTGAAGTTGTTCATGTTCATGAGATCTTCGATGGAAGGAGATGGATTGTT
TP5060_Query	D+G	1	chr6	6A	CAGCAAGAACAGGGTTGAAGTTGTTCATGTTCATGAGATCGTCGATGGAAGGAGATGGATTGTT
TP5909_Hit	D	1	chr6	6A	CAGCAAGGAAAAGTTAAGGAATCTCTTTCCGCACTTAGGCCTCATTTTAACCATGAAAGTCCAG
TP5909_Query	D	1	chr6	6A	CAGCAAGGAAAAGTTAAGGAATCTCTTGCCGCACTTAGGCCTCATTTTAACCATGAAAGTCCAG
TP59250_Hit	D	1	chr6	6A	CAGCTTTCTTCAAACCCTTACAATTCGGATTACCGCATTTGTTACTTGTGCAAACAATAACACC
TP59250_Query	D	1	chr6	6A	CAGCTTTCTTCAAACCCTTACAATTCGGATTACCACATTTGTTACTTGTGCAAACAATAACACC
TP63484_Hit	D+G	1	chr6	6A	CTGCAAGGTTGAAGCGTGATGCTGTGATTTGTAGCCAGGAAAAGTTCTGGTTACTCCAGGATTT
TP63484_Query	D+G	1	chr6	6A	CTGCAAGGTTGAAGCGTGATGCTGTGATTTGTAGCCAGGAAAAGTTCTGGGTACTCCAGGATTT
TP83933_Hit	D	1	chr6	6A	CTGCGTTTACCTTCAAAATAAAATCGTACTCAACAATTTGCAGAAATGGAAAATGATCAACTGT
TP83933_Query	D	1	chr6	6A	CTGCGTTTACCTTCAAAATAAAATCATACTCAACAATTTGCAGAAATGGAAAATGATCAACTGT
TP94751_Hit	D+G	1	chr6	6A	CTGCTTTAATTCCAAAAGCTAAAAGTGTTGATCCAAGGATAAAAAGGTCTGCGTCGTTATATGC
TP94751_Query	D+G	1	chr6	6A	CTGCTTTAATTCCAAAAGCTAAAAGTGTCGATCCAAGGATAAAAAGGTCTGCGTCGTTATATGC
TP95252_Hit	D+G	1	chr6	6A	CTGCTTTGGAGGAACACTTTTTACCATACGGTGAACTTTCCGCTGTAGAGCTAGAAGATGTCCA
TP95252_Query	D+G	1	chr6	6A	CTGCTTTGGAGGAACACTTCTTACCATACGGTGAACTTTCCGCTGTAGAGCTAGAAGATGTCCA
TP14712_Hit	D+G	1	chr6	6B	CAGCAGGATCAAGCAGTAGAAGATGAAATGAAGGTAAAGTTTGTTTCCAACTCAAAAAAGGAAA
TP14712_Query	D+G	1	chr6	6B	CAGCAGGATCAAGCAGTAGAAGACGAAATGAAGGTAAAGTTTGTTTCCAACTCAAAAAAGGAAA

Name	Filter	Nb hit (Mt4.0)	Mt Chr (Mt4.0)	Ms Chr (Li et al., 2014)	Sequence
TP4959_Hit	D	1	chr6	6B	CAGCAACTTTGGCTTTAGCACCGAGCTGAATAAATTCCTGAATAATTCTTCCATCTTCCCCAAT
TP4959_Query	D	1	chr6	6B	CAGCAACTTTGGCTTTAGCACCGAGCTGAATAAATTCCTGAATAATTCTTCCATCCTCCCCAAT
TP49853_Hit	D+G	1	chr6	6B	CAGCTGAAATCTTCCCTAATCACCACTCGGAGGACAGCGATGTGTTTTGCGTCTGCTGGCATTG
TP49853_Query	D+G	1	chr6	6B	CAGCTGAAATCTTCCCTAATCACCACTCGGAGGACAGCGATGTGTTTTGCGTCCGCTGGCATTG
TP55076_Hit	D+G	1	chr6	6B	CAGCTTCATGCAGATCGACAAAGATAAGGAACGACATACAATGAAAGCTGATTCACAGCAATTT
TP55076_Query	D+G	1	chr6	6B	CAGCTTCATGCAGATCGACAAAGATAAGGAACGACATACAATGAAAGCTGACTCACAGCAATTT
TP67390_Hit	D	1	chr6	6B	CTGCACTTTGTGTGCTTAAAAATGTGTCAAGTTCAAATTGGACGAAATTCTTTTTCTTGCAATT
TP67390_Query	D	1	chr6	6B	CTGCACTTTGTGAGCTTAAAAATGTGTCAAGTTCAAATTGGACGAAATTCTTTTTCTTGCAATT
TP87823_Hit	D	1	chr6	6B	CTGCTCCCATGATTGCATTACGCGACTTCTGCAGAAAGCTAGTACTCTGGGACTTTTCCTTTTT
TP87823_Query	D	1	chr6	6B	CTGCTCCCATGATTGCATTACGCGACCTCTGCAGAAAGCTAGTACTCTGGGACTTTTCCTTTTT
TP18266_Hit	D	1	chr6	6C	CAGCATCAACAATGTTTCATAATTTTTGCTTCAACATGGCAAAAGACGTTGAAGCCATTGTTGC
TP18266_Query	D	1	chr6	6C	CAGCATCAACAATGTTTCATAATTTTTGCCTCAACATGGCAAAAGACGTTGAAGCCATTGTTGC
TP28166_Hit	D+G	1	chr6	6C	CAGCCCATACTTGCTTTTCACCAAAAGAAGTTTCAGTTCGATCCTTGTAAACATCATCAAAATC
TP28166_Query	D+G	1	chr6	6C	CAGCCCATACTTGCTTTTCACCAAAAGAAGTTTCAGTTCGATCCTTGTAAAAATCATCAAAATC
TP42031_Hit	D	1	chr6	6C	CAGCTAAATCATCATAATCGGGTAAGTTGGGATGCACATGTCGGGCTTCTTTTGATCTATCTGG
TP42031_Query	D	1	chr6	6C	CAGCTAAATCATCATAATCGGGTAAGTTGGGATGCACATGCCGGGCTTCTTTTGATCTATCTGG
TP76084_Hit	D	1	chr6	6C	CTGCCCATTCTTGTGCTTTCACCCAGTTGCCTAATCCCAATGCCTCAGGTACCAAAATCCCTGG
TP76084_Query	D	1	chr6	6C	CTGCCCATTCTTGCGCTTTCACCCAGTTGCCTAATCCCAATGCCTCAGGTACCAAAATCCCTGG
TP17847_Hit	D	1	chr6	6D	CAGCATATATGAAGAAAACCATGGTATGATATTTGATATACTGTGGGAAATGGTTGTTAGCTCT
TP17847_Query	D	1	chr6	6D	CAGCATATATGAAGAAAACCATAGTATGATATTTGATATACTGTGGGAAATGGTTGTTAGCTCT
TP41018_Hit	D	1	chr6	6D	CAGCGTTGCACCATGGCCGGCTATAAGGGATATATGTCCACATAACTTCTGCAAAATTATGGGG
TP41018_Query	D	1	chr6	6D	CAGCGTGGCACCATGGCCGGCTATAAGGGATATATGTCCACATAACTTCTGCAAAATTATGGGG
TP59267_Hit	D	1	chr6	6D	CAGCTTTCTTGTATCCTTGAGTTTGGTGGTGTATCCATGGGGGTTGAGTATGGTGATCTAAACA
TP59267_Query	D	1	chr6	6D	CAGCTTTCTTGTATCCTTGAGTTTGGCGGTGTATCCATGGGGGTTGAGTATGGTGATCTAAACA
TP10051_Hit	D	1	chr7	.	CAGCACCAACACCTTCATCGCGTAGGATTCGTCATAAAAGTGTGCAGTTTGATGACCATATTTT
TP10051_Query	D	1	chr7	.	CAGCACCAACACCTTCATCGCGTAGGATTCGTCATAAAAGTGTGCAGTTTGATGACCAGATTTT
TP10075_Hit	D	1	chr7	.	CAGCACCAACGAAAAGACCTTCGTTGTCATGTCCACACCCTAAAGCCACACGCGCCACGCGCGT
TP10075_Query	D	1	chr7	.	CAGCACCAACGAAAAGACCTTCGTTATCATGTCCACACCCTAAAGCCACACGCGCCACGCGCGT
TP10198_Hit	D	1	chr7	.	CAGCACCACATAGAAGAGCTTGACAGGGAGAAACTAGAGGAGCAGGCAAGAGCGTTGGAGAAAG
TP10198_Query	D	1	chr7	.	CAGCACCACATAGAAGAGCTTGACAAGGAGAAACTAGAGGAGCAGGCAAGAGCGTTGGAGAAAG
TP10313_Hit	D	1	chr7	.	CAGCGCCAGCAATATTCACAAGATTTTCAACTTTTGGTATTCAAATTATTAGTCAAGCTTTTGT
TP10313_Query	D	1	chr7	.	CAGCACCAGCAATATTCACAAGATTTTCAACTTTTGGTATTCAAATTATTAGTCAAGCTTTTGT
TP10368_Hit	D	1	chr7	.	CAGCACCAGTTCAAGGGTAAGGAAGATTAAGGACAAAACTGAGAACAACCTCAAAATTCAAGGA
TP10368_Query	D	1	chr7	.	CAGCACCAGTTCAAGGGTAAAGAAGATTAAGGACAAAACTGAGAACAACCTCAAAATTCAAGGA
TP10456_Hit	D+G	1	chr7	.	CAGCACCATGTCCCTCTTCTTTGATGATCTCGGGCGCTAAGTATTCATGTGTACCAACAAATGA
TP10456_Query	D+G	1	chr7	.	CAGCACCATGTCCCTCTTCTTTGATGATCTCGGGCGCCAAGTATTCATGTGTACCAACAAATGA
TP10597_Hit	D	1	chr7	.	CAGCACCCGGGACAATTTGAATACTATAAGAACTATGATGACCTTGAGGTAAGAAATATTTGTC
TP10597_Query	D	1	chr7	.	CAGCACCCGGGACAATTTGAATACTATAAGAACTACGATGACCTTGAGGTAAGAAATATTTGTC
TP1072_Hit	D	1	chr7	.	CAGCAAAATTTTAACAAACCGCTTTTTTCTACGATTCACGGATGGCAACAATGCATAAATGTAT
TP1072_Query	D	1	chr7	.	CAGCAAAATTTTAACAAACCGCTTTTTTCTACGATTCACGAATGGCAACAATGCATAAATGTAT
TP10876_Hit	D	1	chr7	.	CAGCACCTATCTCCGCATTCAACTCCCACCCATTTTTCACCATGTAACCATGAACTGATTTTCC
TP10876_Query	D	1	chr7	.	CAGCACCTATCTCCGCATTCAACTCCCACCCATTTTTCACAATGTAACCATGAACTGATTTTCC
TP11036_Hit	D	1	chr7	.	CAGCACCTTCTGCATTCATTGCATGTTTGTTAAAGGAATTGATTGCAAATTCAATCACGGCCAA
TP11036_Query	D	1	chr7	.	CAGCACCTTCAGCATTCATTGCATGTTTGTTAAAGGAATTGATTGCAAATTCAATCACGGCCAA
TP11122_Hit	D	1	chr7	.	CAGCACGAACAATATCTTCAATCTGTACATCCACATATACCAGCAAGCAAAGAGATATAATGAG
TP11122_Query	D	1	chr7	.	CAGCACGAACAATATCTTCAATCTGTACATACACATATACCAGCAAGCAAAGAGATATAATGAG
TP11136_Hit	D	1	chr7	.	CAGCACGAAGCTCATCTAAGGAATGGCGGTCCCAGTTAACTCCTCTACAAGATGTCCCATTATG
TP11136_Query	D	1	chr7	.	CAGCACGAAGCTCATCTAAAGAATGGCGGTCCCAGTTAACTCCTCTACAAGATGTCCCATTATG
TP11245_Hit	D	1	chr7	.	CAGCACGATGAACACATAACCTCTTCCCTTGTTAGATCTGTGAAGAAGAATTGGTTTGTAAGGG
TP11245_Query	D	1	chr7	.	CAGCACGATGAACACATAACCTCTTCCCTTGTTAGATCTGTGAAAAAGAATTGGTTTGTAAGGG
TP11278_Hit	D+G	1	chr7	.	CAGCACGCAAACTATTTGGCATTCTTTTCCAGCATAGCCTTCCTTCCTCAAGTTTGTCTGAACA
TP11278_Query	D+G	1	chr7	.	CAGCACGCAAACTATTTGACATTCTTTTCCAGCATAGCCTTCCTTCCTCAAGTTTGTCTGAACA
TP11380_Hit	D	1	chr7	.	CAGCGCGCGATGCAGGAACGGAGAAACCTGCTTACACTAAATGGGAAGAACAAGATTCTTTGCT
TP11380_Query	D	1	chr7	.	CAGCACGCGATGCAGGAACGGAGAAACCTGCTTACACTAAATGGGAAGAACAAGATTCTTTGCT

Name	Filter	Nb hit (Mt4.0)	Mt Chr (Mt4.0)	Ms Chr (Li et al., 2014)	Sequence
TP11401_Hit	D	1	chr7	.	CAGCACGCTATAGCACTGTTGTGTAACGAATTCAAACAAACCGCTATTTTCTGTGGTTTGTGTT
TP11401_Query	D	1	chr7	.	CAGCACGCTATAGCACTGTTGTGTAACAAATTCAAACAAACCGCTATTTTCTGTGGTTTGTGTT
TP1145_Hit	D	1	chr7	.	CAGCAAACAATCTTATGAGTTACCACATCATCACACATTCAAAATTATGAAACTTAGATACCCA
TP1145_Query	D	1	chr7	.	CAGCAAACAATCTTATGAGTTACCACATCATCACACATTCAAAATTATGAAACTTAGATACCAA
TP1156_Hit	D	1	chr7	.	CAGCAAACAATTGAAACAAATTTTCCTCTAATGTTGAAACAAATTTTCCTGTATATACTTTCTA
TP1156_Query	D	1	chr7	.	CAGCAAACAATTGAAACAAAGTTTCCTCTAATGTTGAAACAAATTTTCCTGTATATACTTTCTA
TP1157_Hit	D	1	chr7	.	CAGCAAACAATTGAAAGAAACTTTCCTCTATTGTTGAAACCAATTTTCCTGTATATACTTTCTA
TP1157_Query	D	1	chr7	.	CAGCAAACAATTGAAAGAAACTTTCCTCTAATGTTGAAACCAATTTTCCTGTATATACTTTCTA
TP11741_Hit	D+G	1	chr7	.	CAGCACTAAGGAACTTTTTATTCTTTAGATTACTACTAGTTTGCTCCTTAGATACAATTCTAAA
TP11741_Query	D+G	1	chr7	.	CAGCACTAAGGAACTTTTTATTCTTTAGATTACTACTAGTTTGCTCCTTAGATACAACTCTAAA
TP11903_Hit	D	1	chr7	.	CAGCACTATAGCACAATAGCATAGTAGAATTTGAACAAATTGCTACTGTTCTGCTATCTGCTAT
TP11903_Query	D	1	chr7	.	CAGCACTATAGCACAATAGCATAGTAGAATTTGAACAAATTGCTACTGTTCTGCTATCCGCTAT
TP11927_Hit	D	1	chr7	.	CAGCACTATCCAAGTCTGTCGGAGAGTACTAAGGCAGAGAGAAAGCAGAAGAAGCAGGAAAACA
TP11927_Query	D	1	chr7	.	CAGCACTATCCAAGTCTGTCGGAGAGCACTAAGGCAGAGAGAAAGCAGAAGAAGCAGGAAAACA
TP11959_Hit	D+G	1	chr7	.	CAGCACTATTATGTGCATTGATATGTGCTCTAGGGCTAAACACCATAGCTCGTTGCGCCATGCG
TP11959_Query	D+G	1	chr7	.	CAGCACTATTATGTGCATTGATATGCGCTCTAGGGCTAAACACCATAGCTCGTTGCGCCATGCG
TP12057_Hit	D	1	chr7	.	CAGCACTCCAGAGGATCAGATTCGTGCTTTTAGCACTTGGTTCCTCTCCATCAGTCCAAGAGAG
TP12057_Query	D	1	chr7	.	CAGCACTCCAGAGGATCAGATTCGTGCTTTTAGCACTTGGTTCCTCGCCATCAGTCCAAGAGAG
TP12144_Hit	D	1	chr7	.	CAGCACTCTTTCCCTTCTTTTTCTGTAATTTCTTCATGTAAAACTCCAGCTCCTTACCTTCAAG
TP12144_Query	D	1	chr7	.	CAGCACTCTTTCCCTTCTTTTTCTGTAATTTCTTCATGTAAAACTCCAACTCCTTACCTTCAAG
TP12224_Hit	D	1	chr7	.	CAGCACTGCAAATGTTTGATTGCAGTGGATTAAACTACTCATTGTAATGAGAAGCTACTCATTA
TP12224_Query	D	1	chr7	.	CAGCACTGCAAATGTTTGATTGAAGTGGATTAAACTACTCATTGTAATGAGAAGCTACTCATTA
TP1224_Hit	D	1	chr7	.	CAGCAAACAGCACCCCCTGTTGCTCGTCCCAACTTCCTCCATTTGTTAATTGACATTAATTGAG
TP1224_Query	D	1	chr7	.	CAGCAAACAGCACCCCCTGTTGCTCGTCCCAACTTCCTCCATTTGTTAATTGACATTAATTAAG
TP1233_Hit	D	1	chr7	.	CAGCAAACAGTTGGCCACGTTCGAATCATGACTCAATTAGAGGCCGAATCAATTATCATCACGT
TP1233_Query	D	1	chr7	.	CAGCAAACAGTTGGCCACGTTCGAATCATGACTCAATTAGAGGCCAAATCAATTATCATCACGT
TP12450_Hit	D+G	1	chr7	.	CAGCACTTAGACCAGAGTAAGGGCCTGAAGAAAACACTGAAGATGTTCCATCAAACTCAAAAGT
TP12450_Query	D+G	1	chr7	.	CAGCACTTAGACCAGAGTAAGGGCCTGAAGAAAACACCGAAGATGTTCCATCAAACTCAAAAGT
TP12953_Hit	D	1	chr7	.	CAGCAGAAATTCGTGACCCAACAAGAAAAGGCTCTCGTATTTGGCTTGGAACATTTGATACAGC
TP12953_Query	D	1	chr7	.	CAGCAGAAATTCGTGACCCAACAAGAAAAGGCTCTCGCATTTGGCTTGGAACATTTGATACAGC
TP13067_Hit	D	1	chr7	.	CAGCAGAAGAAACATTAGCAGACGATTCGGTCAGAACGCCCACTACTGCCTGATCCTGACTAGT
TP13067_Query	D	1	chr7	.	CAGCAGAAGAAACATTAGCAGACGACTCGGTCAGAACGCCCACTACTGCCTGATCCTGACTAGT
TP13101_Hit	D+G	1	chr7	.	CAGCAGAAGAATTAGATGAGCAGACAAAAAATATTAGAGTTCACCTCAAACTTTCTATCTCTGT
TP13101_Query	D+G	1	chr7	.	CAGCAGAAGAATTAGATGAGCAGACAAAAAAAATTAGAGTTCACCTCAAACTTTCTATCTCTGT
TP13222_Hit	D+G	1	chr7	.	CAGCAGATGGTGATGTCACGGAGAAACCTGTAGACCCTATCAAGATATACTCCGATAAGGAGTT
TP13222_Query	D+G	1	chr7	.	CAGCAGAAGGTGATGTCACGGAGAAACCTGTAGACCCTATCAAGATATACTCCGATAAGGAGTT
TP13237_Hit	D	1	chr7	.	CAGCAGAAGTATTATACCAATCTGTCACCTCTTCTAAGTTTTGCCATCTTTGTCTAGTTATCGT
TP13237_Query	D	1	chr7	.	CAGCAGAAGTATTATACCAATCTGTCACCTCTTCTAAGTTTCGCCATCTTTGTCTAGTTATCGT
TP13463_Hit	D	1	chr7	.	CAGCAGACATGCACGAAGTGTTTCACGTTCTCCAGGCAGGTCTCGTTCTCCAGCCAGGTCTCGT
TP13463_Query	D	1	chr7	.	CAGCAGACATGCACGAAGTGTTTCACGTTCTCCAGGCAGGTCACGTTCTCCAGCCAGGTCTCGT
TP14100_Hit	D	1	chr7	.	CAGCAGTTCAGAGCTTCGCTTCTGCACCGACATTGACAACACAGGTAGGATACTAAATTTAGTT
TP14100_Query	D	1	chr7	.	CAGCAGATCAGAGCTTCGCTTCTGCACCGACATTGACAACACAGGTAGGATACTAAATTTAGTT
TP14179_Hit	D+G	1	chr7	.	CAGCAGATGACTGAGGGGCCAAATCCACAGTTTCACCGGTAACAAAGGGGTGCTGAAAAAAAAA
TP14179_Query	D+G	1	chr7	.	CAGCAGATGACCGAGGGGCCAAATCCACAGTTTCACCGGTAACAAAGGGGTGCTGAAAAAAAAA
TP14181_Hit	D	1	chr7	.	CAGCAGATGACTGAGGGGCCAAATCCACAGTTTCACCGGTAACAAAGGGGTGCGGAGATCGGAA
TP14181_Query	D	1	chr7	.	CAGCAGATGACTGAGGGGCCAAATCCACAGTTTCACCGGTAACAAAGGGGTGCCGAGATCGGAA
TP14483_Hit	D+G	1	chr7	.	CAGCAGGAATGAAAGCAAAACTTATAGTGGAAGTAACATCATCTCCTGCATCTTCATCGACGAT
TP14483_Query	D+G	1	chr7	.	CAGCAGGAATGAAAGCAAAACTTATAGTGGAAGTAACATCATCTCCTGCATCTTCATCAACGAT
TP14781_Hit	D+G	1	chr7	.	CAGCAGGCACAGGAGGAATTAAGCCCAGCATCACCACACTTGGAGCCGACCAATTTGATGAGTT
TP14781_Query	D+G	1	chr7	.	CAGCAGGCACAGGAGGAATTAAGCCCAGCATCACCACACTTGGAGCAGACCAATTTGATGAGTT
TP14787_Hit	D	1	chr7	.	CAGCAGGCACGTTATTTAGAGGCAGTTCACCTTTGTTTGCAATGACTTCTGATGATTTGTTACC
TP14787_Query	D	1	chr7	.	CAGCAGGCACGTTATTTAGAGGCAATTCACCTTTGTTTGCAATGACTTCTGATGATTTGTTACC
TP14791_Hit	D	1	chr7	.	CAGCAGGCACTGGGGGGATTAAGCCCAACATCTCCACACTTGGAGCCGACCAATTTGACGAGTT
TP14791_Query	D	1	chr7	.	CAGCAGGCACTGGGGGGATTAAGCCCAACATCTCCACACTTGGAGCCGACCAATTTGACGAGCT

Name	Filter	Nb hit (Mt4.0)	Mt Chr (Mt4.0)	Ms Chr (Li et al., 2014)	Sequence
TP15070_Hit	D	1	chr7	.	CAGCAGGTGCATCTGGCAATGTAACACCAGTACTGTCTGTCATTTGTGCTTCACTAGAATGCAC
TP15070_Query	D	1	chr7	.	CAGCAGGGGCATCTGGCAATGTAACACCAGTACTGTCTGTCATTTGTGCTTCACTAGAATGCAC
TP15542_Hit	D+G	1	chr7	.	CAGCAGTAACGAAAGGGTGACGAATTTTGAGGAGGTAGTTTTTTCTGAGCATAACCTTTAACTG
TP15542_Query	D+G	1	chr7	.	CAGCAGTAACGAAAGGGTGACGAATTTTGAGAAGGTAGTTTTTTCTGAGCATAACCTTTAACTG
TP15658_Hit	D	1	chr7	.	CAGCAGTACTAGTAGCACTCAAGATGAACATTACAGTAGGGATCAAAAGGAAATAAGGAATTTA
TP15658_Query	D	1	chr7	.	CAGCAGTACTAGTAGCACTCAAGATGAACATTACAGTACGGATCAAAAGGAAATAAGGAATTTA
TP15768_Hit	D	1	chr7	.	CAGCAGTAGTAGTAATAAGATGGTAGTGAAGCGCAGATGGAGCTTTCGAAAACTAACAAGTGGA
TP15768_Query	D	1	chr7	.	CAGCAGTAGTAGTAATAAGATGGTAGTGAAGCGCAGATGGAGCTTCCGAAAACTAACAAGTGGA
TP1577_Hit	D	1	chr7	.	CAGCAAAGAAGATTTTTCATGCTCACCTTCGAGCTTATGTCTACCAAATGTTCATCAAGGTGTT
TP1577_Query	D	1	chr7	.	CAGCAAAGAAGATATTTCATGCTCACCTTCGAGCTTATGTCTACCAAATGTTCATCAAGGTGTT
TP16134_Hit	D	1	chr7	.	CAGCAGTGAGGGAGGGCCATCTTGATATTCTTGAGACCTTAATTAATGCAGGGGCATCCCAGCC
TP16134_Query	D	1	chr7	.	CAGCAGTGAGGGAGGGCCATCTTGATATTCTTGAGACCTTAATCAATGCAGGGGCATCCCAGCC
TP16406_Hit	D	1	chr7	.	CAGCAGTGTTGATATTAATAAGTTTGAGGTTACTAAAATTCAAGAGCTTGAGAATATGTTTAAA
TP16406_Query	D	1	chr7	.	CAGCAGTGTTGATATTAACAAGTTTGAGGTTACTAAAATTCAAGAGCTTGAGAATATGTTTAAA
TP16775_Hit	D	1	chr7	.	CAGCAGTTTAACATAAGTAGAATACCTTTTTGGATCATTGTACCGAGGGAAGCGAGTCAGTTTG
TP16775_Query	D	1	chr7	.	CAGCAGTTTAACATAAGTAGAATACCTTTTTGGATCATTATACCGAGGGAAGCGAGTCAGTTTG
TP17067_Hit	D	1	chr7	.	CAGCATAACACAACTGTTCCAGAATAGGACGGAGCAAAAGGGTCAGCAAATTCATATGAGGCAA
TP17067_Query	D	1	chr7	.	CAGCATAACACAACTGTTCCAGAATAGGACAGAGCAAAAGGGTCAGCAAATTCATATGAGGCAA
TP17289_Hit	D+G	1	chr7	.	CAGCGTAATTCCAAAGTTGAACCAGTAATTGAGAATGGGAACACTTATCCAGAGACAAAAACTA
TP17289_Query	D+G	1	chr7	.	CAGCATAATTCCAAAGTTGAACCAGTAATTGAGAATGGGAACACTTATCCAGAGACAAAAACTA
TP17299_Hit	D	1	chr7	.	CAGCCTAATTTAAACAAACCGTTATTGCTTTGCGATGTGTGATTTAATACAAAGGGTTGTCAAA
TP17299_Query	D	1	chr7	.	CAGCATAATTTAAACAAACCGTTATTGCTTTGCGATGTGTGATTTAATACAAAGGGTTGTCAAA
TP17458_Hit	D	1	chr7	.	CAGCATACGACTGCCAAGACACCATTGATTGATCGAAGAATGGAAGCAGAACACATATCAGTAA
TP17458_Query	D	1	chr7	.	CAGCATACGACTGCCAAGAAACCATTGATTGATCGAAGAATGGAAGCAGAACACATATCAGTAA
TP17773_Hit	D	1	chr7	.	CAGCATATAATATTTTCTCAGTATGCCGGATCAACAGACGTCTGGTAAAAGGAAAAGATTTTAT
TP17773_Query	D	1	chr7	.	CAGCATATAATATTTTCTCAGTATGCCAGATCAACAGACGTCTGGTAAAAGGAAAAGATTTTAT
TP17950_Hit	D	1	chr7	.	CAGCATATCTTTGGTTGAAATTTTTGATTTGATTTGATAACCATGCAAAAGTGTATTTGCACTA
TP17950_Query	D	1	chr7	.	CAGCATATCTTTGGTTGAAACTTTTGATTTGATTTGATAACCATGCAAAAGTGTATTTGCACTA
TP17978_Hit	D	1	chr7	.	CAGCATATGCACCTAGTAGTGCATTGTATGAGACTATATTTGGTTTCAGCCCTTCTGCAAGCAT
TP17978_Query	D	1	chr7	.	CAGCATATGCACCTAGTAGTGCATTGTATGAGACTACATTTGGTTTCAGCCCTTCTGCAAGCAT
TP1817_Hit	D	1	chr7	.	CAGCAAAGGAGATGCAACCTCTACGTTTCGTTCATCTAAATTGCGATCCCTGCGTTGATATGGT
TP1817_Query	D	1	chr7	.	CAGCAAAGGAGATGCAACCTCTACGTTTCGTTCATCTAAACTGCGATCCCTGCGTTGATATGGT
TP18261_Hit	D	1	chr7	.	CAGCATCAACAAGAAGAGCTTTATGAGTGATGTTACCGTAAAGAGATCTTCTATAATCAGCATA
TP18261_Query	D	1	chr7	.	CAGCATCAACAAGAAGAGCTTTATGAGTGATATTACCGTAAAGAGATCTTCTATAATCAGCATA
TP18302_Hit	D	1	chr7	.	CAGCATCAACCATGGAGGTTCTCAGCCTTTGTTCACTACAAAAGAATTTTGAGCAGACTCCTCT
TP18302_Query	D	1	chr7	.	CAGCATCAACCATAGAGGTTCTCAGCCTTTGTTCACTACAAAAGAATTTTGAGCAGACTCCTCT
TP18401_Hit	D	1	chr7	.	CAGCATCAATATTTTCAGCTCAGAGTGGTGAGACCGTTGCATTCTCTAGGAATGCAATGTTTTT
TP18401_Query	D	1	chr7	.	CAGCATCAATATTTTCAGCTCAGAGTGGTGAGACCGTGGCATTCTCTAGGAATGCAATGTTTTT
TP1845_Hit	D	1	chr7	.	CAGCAAAGGCCCTCAGAAATGCTTAAAAGTCACCGATAATGTCACTTTTTCGAGGGTGACTCTC
TP1845_Query	D	1	chr7	.	CAGCAAAGGCCCTCAGAAATGCTTAAAAGTCACCGATAATGTCACTTTTTCGAGGGTGACCCTC
TP18476_Hit	D	1	chr7	.	CAGCATCACCAACCAACCTTTGGGATCCAGTAAAAGCAACACAAGAAGGAGTGGTTCTGTTTCC
TP18476_Query	D	1	chr7	.	CAGCATCACCAACCAACCTTTGGGAGCCAGTAAAAGCAACACAAGAAGGAGTGGTTCTGTTTCC
TP18817_Hit	D+G	1	chr7	.	CAGCATCATTCTGGTGTATCTTTGACATCTTAGCTCGATCGGTCATTGATACTGGTCTTCTAGA
TP18817_Query	D+G	1	chr7	.	CAGCATCATTCTGGTGTATCTTTGACATCTTAGCTCGATCGATCATTGATACTGGTCTTCTAGA
TP18959_Hit	D+G	1	chr7	.	CAGCATCCCCAAAAATCAATTCAAGACAATAAGTGGAGTTTCTGTCACAATTGAATCTTGATGT
TP18959_Query	D+G	1	chr7	.	CAGCATCCCCAAAAATCAATTCAAGACAATAAGTGGAGTTTCTGCCACAATTGAATCTTGATGT
TP1900_Hit	D	1	chr7	.	CAGCAAAGGTTGTGGATCTTCTACAGATGGTTCAAAGGGTCATACATAGTGAATGTTGTGTCTC
TP1900_Query	D	1	chr7	.	CAGCAAAGGTTGTGGATCTTCTACAGATGGTTCAAAGGGTCATACATAGTAAATGTTGTGTCTC
TP19007_Hit	D	1	chr7	.	CAGCATCCGTGGGTTTTTGGTTTGTTGTTTTCACTACAAACAATGTCTCAATCATTTCCTCATT
TP19007_Query	D	1	chr7	.	CAGCATCCGTGGGTTTTTGGTTTGCTGTTTTCACTACAAACAATGTCTCAATCATTTCCTCATT
TP19015_Hit	D	1	chr7	.	CAGCATCCTAATCTTTCGCTAGAAGGGACGGAATTTATTCCTTGTGTGTAGAGTTCCTCAGCTT
TP19015_Query	D	1	chr7	.	CAGCATCCTAATCTTTCACTAGAAGGGACGGAATTTATTCCTTGTGTGTAGAGTTCCTCAGCTT
TP1904_Hit	D	1	chr7	.	CAGCAAAGTAAAGCTAAGAAATTTAAAGTCAAAGATCTTGTCAACTCGGGTCAGCTCGAATTCA
TP1904_Query	D	1	chr7	.	CAGCAAAGTAAAGCTAAGAAACTTAAAGTCAAAGATCTTGTCAACTCGGGTCAGCTCGAATTCA

Name	Filter	Nb hit (Mt4.0)	Mt Chr (Mt4.0)	Ms Chr (Li et al., 2014)	Sequence
TP19102_Hit	D	1	chr7	.	CAGCATCGAACCTTTGGAATCGAATAATGAATGTCAAATTTCCTGCGGCTGACGAGTTGATTCT
TP19102_Query	D	1	chr7	.	CAGCATCGAACCTTTGGAATCGAATAATGAATGTCAAATTTCCTGCGGCTGAAGAGTTGATTCT
TP19209_Hit	D	1	chr7	.	CAGCATCGTATAAAGGAAAAATTGTTGTACTGCAAAATCAACATCAAATCAAATCAAATAATGG
TP19209_Query	D	1	chr7	.	CAGCATCGTATAAAGGAAAAATTGTTGTACTGCAAAATCAACATCAAATCAAATCAAATAATGA
TP1921_Hit	D	1	chr7	.	CAGCAAAGTAGTTAATCTTTTCTCAATAGTCTTGCTTTGCATTACAATAGCACAAGGAAGAAAA
TP1921_Query	D	1	chr7	.	CAGCAAAGTAGTTAATCTTTTATCAATAGTCTTGCTTTGCATTACAATAGCACAAGGAAGAAAA
TP19212_Hit	D	1	chr7	.	CAGCATCGTATCCACCACCGCCTACATTACGAGTAGGCGGTGTAGTGGACCCATACTGCATGAT
TP19212_Query	D	1	chr7	.	CAGCATCGTATCCACCACCACCTACATTACGAGTAGGCGGTGTAGTGGACCCATACTGCATGAT
TP19401_Hit	D	1	chr7	.	CAGCATCTGAACTAGACGAACCTTGTGATGTTTCATTCTCAGGAACACCACTTGAATTAACCTT
TP19401_Query	D	1	chr7	.	CAGCATCTGAACTAGACGAACCTTGTGATGTTTCATTATCAGGAACACCACTTGAATTAACCTT
TP19412_Hit	D	1	chr7	.	CAGCATCTGAGAGTCGTTTTATTTCCTCCTGAGCCTCAGACTGCGCTCTATGAACAGCTGAAAA
TP19412_Query	D	1	chr7	.	CAGCATCTGAGAGTCGTTTTATTTCCTCCTGAGCCTCAGACTGCGCTCTATGAACAGCAGAAAA
TP19547_Hit	D+G	1	chr7	.	CAGCATCTTTGGGTTCCTTGTATGACATATTTCAATGTAAGCAGGAATAATATATCTGGCATGC
TP19547_Query	D+G	1	chr7	.	CAGCATCTTCGGGTTCCTTGTATGACATATTTCAATGTAAGCAGGAATAATATATCTGGCATGC
TP1959_Hit	D+G	1	chr7	.	CAGCAAAGTGGTTATGGTTTTGTATTAAGAATGGATAAATAATGTATCAATAATTGATTTTGTT
TP1959_Query	D+G	1	chr7	.	CAGCAAAGTGGTTATGGTTTTGTATTAAGAATGGATAAATAATGTATAAATAATTGATTTTGTT
TP19669_Hit	D	1	chr7	.	CAGCATGAAACCGTTGGATAGGCTTCACCGTAGGTAAAGGTATGTTACAACGCCGATGATGATT
TP19669_Query	D	1	chr7	.	CAGCATGAAACCGTTGGATAGGCTTCACCGTAGGTAAAGGTATGTTACAACGACGATGATGATT
TP19771_Hit	D+G	1	chr7	.	CAGCATGAATTTTGGAATTCCCATGTTTTAGAGTTACATACTAATAGTAATAACAGTGATGCAA
TP19771_Query	D+G	1	chr7	.	CAGCATGAATTTTGGAATTCCCATGTTTTAGAGTTACATACTAATAGCAATAACAGTGATGCAA
TP19928_Hit	D	1	chr7	.	CAGCATGATCAACAGACTCCATTTGTGTGCTCATATTCATTCCCTCACCCGAGGAACCAGCTGG
TP19928_Query	D	1	chr7	.	CAGCATGATCAACAGACTCCATTTGTGTGCTCATATTCATTCCCTCACCCGAGAAACCAGCTGG
TP20005_Hit	D	1	chr7	.	CAGCATGATTTCCAACTTGGGGAGCACAATCTACCACACAGCAGGCACAATGCAGGATCATCAA
TP20005_Query	D	1	chr7	.	CAGCATGATTTCCAACTTGGGGAGCACAATCTACCACACAGAAGGCACAATGCAGGATCATCAA
TP20058_Hit	D	1	chr7	.	CAGCTTGCACAAAGTCTTCACACCGGGTGATCCCACCGGACAAAACCTCCGGCAGTACCCCAAC
TP20058_Query	D	1	chr7	.	CAGCATGCACAAAGTCTTCACACCGGGTGATCCCACCGGACAAAACCTCCGGCAGTACCCCAAC
TP20118_Hit	D	1	chr7	.	CAGCATGCATCCAGGTCCTATTTTCAGCTGTCTATCATCTACGATCTGCAGTTCTTCCTTATGC
TP20118_Query	D	1	chr7	.	CAGCATGCATCCAGGTCCTATTTTCAGCTGTCTATCATCTACGATCTGCAGTTCTTCCATATGC
TP20264_Hit	D	1	chr7	.	CAGCATGCTTAGCACCCAAGAAAGCTCCATAAACATTAACGTCAAATACCCTTTTGAAAGCTTC
TP20264_Query	D	1	chr7	.	CAGCATGCTTAGCACCCAAGAAAGCTCCATAAACATTAACATCAAATACCCTTTTGAAAGCTTC
TP20322_Hit	D	1	chr7	.	CAGCATGGACCGGATCACAAATTAAAGTACCAGTGAAGTTCATCATAGGTGATCTGGATTTAGT
TP20322_Query	D	1	chr7	.	CAGCATGGACCGGAGCACAAATTAAAGTACCAGTGAAGTTCATCATAGGTGATCTGGATTTAGT
TP20353_Hit	D+G	1	chr7	.	CAGCATGGAGTGTTACTTTGACCGATAATAAACAAGTCATATGGCTGATATGATATCCCGATGT
TP20353_Query	D+G	1	chr7	.	CAGCATGGAGTGTTACTTTGACCGATAATAAACAAGTCATAGGGCTGATATGATATCCCGATGT
TP20499_Hit	D	1	chr7	.	CAGCATGGTGGCACATTAGTAACTTATGTTTATAATAACTGCACGCATTCTTTTTAGTGCCACA
TP20499_Query	D	1	chr7	.	CAGCATGGTGGCACAGTAGTAACTTATGTTTATAATAACTGCACGCATTCTTTTTAGTGCCACA
TP20524_Hit	D	1	chr7	.	CAGCATGTTTTAAATTGAGAATACGTTGAAGAGCTAATTCACATGTTTCTCGAACCTCTTGAGC
TP20524_Query	D	1	chr7	.	CAGCATGGTTTAAATTGAGAATACGTTGAAGAGCTAATTCACATGTTTCTCGAACCTCTTGAGC
TP20897_Hit	D	1	chr7	.	CAGCATTAAAATGTAACACAGTGAAACTTAAATAGCATCAGGAATTAGAAATTTCTTACCCAGG
TP20897_Query	D	1	chr7	.	CAGCATTAAAATGTAACACAGTGAAACTCAAATAGCATCAGGAATTAGAAATTTCTTACCCAGG
TP20947_Hit	D+G	1	chr7	.	CAGCATTAAGATTAAGGAGTGGAAGTTTAAGGAGAAAAGGGTTTGACATGTACAATGAGTTTGA
TP20947_Query	D+G	1	chr7	.	CAGCATTAAGATTAAGGAGTGGAAGTTTAAGGAGAAAAGGGTATGACATGTACAATGAGTTTGA
TP21015_Hit	D+G	1	chr7	.	CAGCATTACCAGCCAAAGAGATACCTCTAAAACCACACTGTTGAAGCTTTTCTCTCCAATTATG
TP21015_Query	D+G	1	chr7	.	CAGCATTACCAGCCAAAGAGATACCTCTAAAACCACAATGTTGAAGCTTTTCTCTCCAATTATG
TP21097_Hit	D+G	1	chr7	.	CAGCATTAGCCCAGTGGACTGGTCAGAGGACTGTGCCAAATGTTTTTATTGGCGGAAACCACAT
TP21097_Query	D+G	1	chr7	.	CAGCATTAGCCCAGTGGACTGGTCAGAGGACCGTGCCAAATGTTTTTATTGGCGGAAACCACAT
TP21203_Hit	D	1	chr7	.	CAGCATTATGAATCATATAATCAACCCCAGAATCTGGGAAAAGGGATTCTGCTTCATCGACGAC
TP21203_Query	D	1	chr7	.	CAGCATTATGAATCATATAATCAACCCCAGAATCTGGGAAAAGGGATTCTGCTTCATCGACAAC
TP21360_Hit	D	1	chr7	.	CAGCATTCATGGAAGATGTATTGGGAATATTTGAAATTTTCCCCTTCTCACTTCTCTTTTTCTT
TP21360_Query	D	1	chr7	.	CAGCATTCATGGAAGATGTATTGGGAATATTTGAAAATTTCCCCTTCTCACTTCTCTTTTTCTT
TP21618_Hit	D	1	chr7	.	CAGCATTGACAGATGTTATTCGTAAACAGAGATGGAAACTCAAATCTGAAATTGCAGTTGATCC
TP21618_Query	D	1	chr7	.	CAGCATTGACAGATGTTATTCCTAAACAGAGATGGAAACTCAAATCTGAAATTGCAGTTGATCC
TP21630_Hit	D	1	chr7	.	CAGCATTGACTCTCAGCATATTGCAAAAGTTGGGTGTCGCATCAGAAATCTTTCATCTTTGGTT
TP21630_Query	D	1	chr7	.	CAGCATTGACTCTCAGCATATTGCAAAAGTTGGGTGTCGCATCAGAAATCTTCCATCTTTGGTT

Name	Filter	Nb hit (Mt4.0)	Mt Chr (Mt4.0)	Ms Chr (Li et al., 2014)	Sequence
TP22062_Hit	D	1	chr7	.	CAGCATTTATTGTTTCTTGCACCTGTCCAGTTTTGATAGTTTAAATTAAACCTATGACTGTAAT
TP22062_Query	D	1	chr7	.	CAGCATTTATTGTTTCTTGCACCTGTCCAATTTTGATAGTTTAAATTAAACCTATGACTGTAAT
TP22192_Hit	D+G	1	chr7	.	CTGCATTTGAAAGGAGGTTTCAACAGGTTCATGTTTCTGAACCTAGTGTGCCTGATACCATTAG
TP22192_Query	D+G	1	chr7	.	CAGCATTTGAAAGGAGGTTTCAACAGGTTCATGTTTCTGAACCTAGTGTGCCTGATACCATTAG
TP22276_Hit	D+G	1	chr7	.	CAGCATTTGCATCTCTTTTTTTTGGCATATGCCTAGCTTTAATGCTTTTTGGATCGGTCACTTT
TP22276_Query	D+G	1	chr7	.	CAGCATTTGCATCTCTTTTTTTTGGCATATGCCTAGCTTTAATGCTTTTTGGATCGGTCAATTT
TP22284_Hit	D	1	chr7	.	CAGCATTTGCTCGCCACGGAGATGGTTTTAAGGCACTTCAATTTTACGAAGAGATGAGAGTGGA
TP22284_Query	D	1	chr7	.	CAGCATTTGCCCGCCACGGAGATGGTTTTAAGGCACTTCAATTTTACGAAGAGATGAGAGTGGA
TP22341_Hit	D	1	chr7	.	CAGCATTTGGTGTTTTTGATTCCATTAGGGATAAGAATTTGGTTTCTTGGAATGCAATAATTGG
TP22341_Query	D	1	chr7	.	CAGCATTTGGTGTTTTTGAGTCCATTAGGGATAAGAATTTGGTTTCTTGGAATGCAATAATTGG
TP22490_Hit	D	1	chr7	.	CAGCATTTTGACGATACCCGAAAAATACCAAATACTACAGTTTGGTATGGTTGCTGAGGTTTCT
TP22490_Query	D	1	chr7	.	CAGCATTTTGACAATACCCGAAAAATACCAAATACTACAGTTTGGTATGGTTGCTGAGGTTTCT
TP22512_Hit	D	1	chr7	.	CAGCATTTTGGATAATCTGGCTGGGTCATCTGATCTTCCAAAGGTTAAAAAGTCAGCAAAAGGG
TP22512_Query	D	1	chr7	.	CAGCATTTTGGATAATCTGGCCGGGTCATCTGATCTTCCAAAGGTTAAAAAGTCAGCAAAAGGG
TP2262_Hit	D	1	chr7	.	CAGCAAATCTTGAAGCCAAAACCGTAGCACCAAAATTATTAACATTTTTACCACTCCTTGATGT
TP2262_Query	D	1	chr7	.	CAGCAAATCTTGAAGCCAAAACAGTAGCACCAAAATTATTAACATTTTTACCACTCCTTGATGT
TP22685_Hit	D	1	chr7	.	CAGCCAAAACATGGGGCTTCTTCCAAATCATAAACCACAACGTAAACGTGTCGGTTCTCGACAA
TP22685_Query	D	1	chr7	.	CAGCCAAAACATGGGGCTTCTTCCAAATCATAAACCACAACGTAAAAGTGTCGGTTCTCGACAA
TP2274_Hit	D+G	1	chr7	.	CAGCAAATGAAGTATGTGATAGATTTGCGGGTGCTGGATTTCACAGTAACTTGATAAGTTACCT
TP2274_Query	D+G	1	chr7	.	CAGCAAATGAAGTATGTGATAGATTTGCGGGCGCTGGATTTCACAGTAACTTGATAAGTTACCT
TP2284_Hit	D	1	chr7	.	CAGCAAGTGACTCTTTAGCCAGCTTAAGAGTTTCCAACTCCCTCTGTAAATAGTCTTCTGTGGC
TP2284_Query	D	1	chr7	.	CAGCAAATGACTCTTTAGCCAGCTTAAGAGTTTCCAACTCCCTCTGTAAATAGTCTTCTGTGGC
TP22852_Hit	D	1	chr7	.	CAGCCAAACAATCAAAACCCGATGAAACAGCGGTTACTTGACAACCCAGCTTCTCAAGCTGAAA
TP22852_Query	D	1	chr7	.	CAGCCAAACAATCAAAACCCGATGAAACAGCGGTTACTTGACAACCCAGCTTCTCAAGCAGAAA
TP22899_Hit	D	1	chr7	.	CAGCCAACCCCAAATGTGGGATAGCAGGGATAGCGTATAGCGGTAATTGCAAATAACTAAGAAG
TP22899_Query	D	1	chr7	.	CAGCCAAACCCAAATGTGGGATAGCAGGGATAGCGTATAGCGGTAATTGCAAATAACTAAGAAG
TP23096_Hit	D+G	1	chr7	.	CAGCCAAATATTGTTGGTATGTGTTCTACATAAAAATCAATATAAGTACACATGACTGAACAAC
TP23096_Query	D+G	1	chr7	.	CAGCCAAATATTGTTGGTATGTGTTCTACATAAAAATCAATATAAGTACACATGACCGAACAAC
TP23165_Hit	D	1	chr7	.	CAGCCAAATGTGCCTGAGTTTGAAAAGGAAGAGAGAGTTACTCATGGTATTTCGTCGTTTCAAA
TP23165_Query	D	1	chr7	.	CAGCCAAATGTGCCTGAGTTTGAAAAGGAAGAGAGAGTCACTCATGGTATTTCGTCGTTTCAAA
TP2341_Hit	D	1	chr7	.	CAGCAAATGCCATAACGTACAATAACTTATCAAAGAGCTCGATAAAAAAAATAAACCTTAGCAA
TP2341_Query	D	1	chr7	.	CAGCAAATGCCATAAAGTACAATAACTTATCAAAGAGCTCGATAAAAAAAATAAACCTTAGCAA
TP23671_Hit	D+G	1	chr7	.	CAGCCAAGCTTGAGCCTAGACAAAGATGTCTGTTGTTGCCTATGAACATGGTTGAGGTTAGTGT
TP23671_Query	D+G	1	chr7	.	CAGCCAAGCTTGAGCCTAGACAAAGATGTCTGTTGTTGCCCATGAACATGGTTGAGGTTAGTGT
TP23672_Hit	D	1	chr7	.	CAGCCAAGCTTTCTGATTTTGGGTTGGCTAAAGCAGGGCCTCAGGGCGATGAAACCCATGTATC
TP23672_Query	D	1	chr7	.	CAGCCAAGCTTTCTGATTTTGGGTTGGCTAAAGCAGGGCCTCAAGGCGATGAAACCCATGTATC
TP23728_Hit	D	1	chr7	.	CAGCCAAGGTATGTGGTTAATCAAGTAACAGCTGGTGACTATGATTGTCCAAGGAATGTTTTAA
TP23728_Query	D	1	chr7	.	CAGCCAAGGTATGTGGTTAATCAAGTAACAGCTGGGGACTATGATTGTCCAAGGAATGTTTTAA
TP23927_Hit	D+G	1	chr7	.	CAGCCAATCACCACATATCCCAGCCCTTCCAAAGGGATCAAAGATGCAAGGAACTTCAGGTGTG
TP23927_Query	D+G	1	chr7	.	CAGCCAATCACCACATATCCCAGCCCTTCCAAAGGGATCAAAGATGCAAGGAACTCCAGGTGTG
TP23955_Hit	D+G	1	chr7	.	CAGCCAATCGTGGCTGTTCCTTCTTTCATTTGTGCGGATGCATTCAGCAAGTTTCTTCCATTCT
TP23955_Query	D+G	1	chr7	.	CAGCCAATCGTAGCTGTTCCTTCTTTCATTTGTGCGGATGCATTCAGCAAGTTTCTTCCATTCT
TP24000_Hit	D	1	chr7	.	CAGCCAATGATCAAGCACCACTTGTCTACCTCCTGTTCTAAGTGATGTTCATCGTCAATTCTAT
TP24000_Query	D	1	chr7	.	CAGCCAATGATCAAGCACCACTTGTCTACCTCCTGTTCTAAGTGATGTTCATCATCAATTCTAT
TP24157_Hit	D	1	chr7	.	CAGCCAATTTGTGCGATTGGATATATTCAATTTCTACTGCAACTTTACACAATTATTTACTTCA
TP24157_Query	D	1	chr7	.	CAGCCAATTTGTGCGATTGGATATATTCAATTTCTACTGCAACTTTACACAATCATTTACTTCA
TP24167_Hit	D	1	chr7	.	CAGCCAATTTTCTTTCACTAGACATATAAGCACCTCTAACCAACTTAAATCCCATTGGTATCCC
TP24167_Query	D	1	chr7	.	CAGCCAATTTTCTTTCACTAGACATATAAGCACCTCTAACCAACTTAAACCCCATTGGTATCCC
TP24188_Hit	D	1	chr7	.	CAGCCACAAAAGGGACTCCAGACCCTGGTGCATTTGGTGGTATTTTTTGTGATCATTATGAGAT
TP24188_Query	D	1	chr7	.	CAGCCACAAAAGGGACTCCAGAACCTGGTGCATTTGGTGGTATTTTTTGTGATCATTATGAGAT
TP24286_Hit	D	1	chr7	.	CAGCCACAAGAAATTTCTGACCGGACAATGTCTTGGGGGAAGGTGGTTTTGGTTCAGTTTCTAA
TP24286_Query	D	1	chr7	.	CAGCCACAAGAAATTTCTGACCGGACAATGTCTTAGGGGAAGGTGGTTTTGGTTCAGTTTCTAA
TP24492_Hit	D	1	chr7	.	CAGCCGCAGGATCCATCTCATGAATCATCTCATTGGAAACAAAAATTATATCCCTTAAATGACA
TP24492_Query	D	1	chr7	.	CAGCCACAGGATCCATCTCATGAATCATCTCATTGGAAACAAAAATTATATCCCTTAAATGACA

Name	Filter	Nb hit (Mt4.0)	Mt Chr (Mt4.0)	Ms Chr (Li et al., 2014)	Sequence
TP24512_Hit	D	1	chr7	.	CAGCCACAGGTGTTGATGTTCCAAGCCTTGCTATAGGTAATTAATCTTAGTCACCACTACATTT
TP24512_Query	D	1	chr7	.	CAGCCACAGGTGTTGATGTACCAAGCCTTGCTATAGGTAATTAATCTTAGTCACCACTACATTT
TP24607_Hit	D	1	chr7	.	CAGCCACATGCACATGTTGGGCAGGGTACAATGGTGTCGTTATAGAACGATGAGAGGGAGACAC
TP24607_Query	D	1	chr7	.	CAGCCACATGCACATGTTGGGCAGGGTACAATGGTGTCGTTATAGAAAGATGAGAGGGAGACAC
TP24683_Hit	D	1	chr7	.	CAGCCACCAAAGTTTGGTTGGTCTCCGGTACCCTCACCTTCAGCCTCCCCATCACCCTCACCTG
TP24683_Query	D	1	chr7	.	CAGCCACCAAAGTTTGGTTGGTCTCCAGTACCCTCACCTTCAGCCTCCCCATCACCCTCACCTG
TP24689_Hit	D	1	chr7	.	CAGCCACCAACACAGACAAACAATATAAAAGAGAAAGACATAACATTGAGATAATCGGAACCCG
TP24689_Query	D	1	chr7	.	CAGCCACCAACACAGACAAACAATATAAAAGAGAAAGACACAACATTGAGATAATCGGAACCCG
TP24832_Hit	D	1	chr7	.	CAGCCACCCCAGTTCTCCACTCAAAATCGATGGTCACAGTTATCGGGGGTTTTTGATCCAGCAA
TP24832_Query	D	1	chr7	.	CAGCCACCCCAGTTCTCCACTCAAAATCAATGGTCACAGTTATCGGGGGTTTTTGATCCAGCAA
TP24937_Hit	D	1	chr7	.	CAGCCACCTCAAGAGGATTGCGGGGAATAGCATTGGTAAAACTATGACGGCATTCTGGGAGAGA
TP24937_Query	D	1	chr7	.	CAGCCACCTCAAGAGGATTGCGGGGAATAGCATTGGTAAAACTATGACGGCATACTGGGAGAGA
TP2499_Hit	D	1	chr7	.	CAGCAAATTCGCTAGCAATAATCAATCCAAGCAATTTAGCCTCAAAAATATATATGTCCCCCAA
TP2499_Query	D	1	chr7	.	CAGCAAATTCGCTAGCAATAATCAATCCAAGCAATTCAGCCTCAAAAATATATATGTCCCCCAA
TP25004_Hit	D	1	chr7	.	CAGCCACGAAGAATACCAAGTGTGATAACAAAATTAGAATGGCTATAAATCCGTTTGCAAAGAT
TP25004_Query	D	1	chr7	.	CAGCCACGAAGAATACCAAGTGTGACAACAAAATTAGAATGGCTATAAATCCGTTTGCAAAGAT
TP25024_Hit	D	1	chr7	.	CAGCCACGACCTTGCAATTGGAATGGAATAGGACAGCCACGGAAAACACCTGTTAATGATGGAA
TP25024_Query	D	1	chr7	.	CAGCCACGACCTTGCAATTGGAATGGAATAGGACAGCCACGGAAAACAACTGTTAATGATGGAA
TP25093_Hit	D+G	1	chr7	.	CAGCCACGGGTCTTTATGGCAATGGCTAGGGATGGTTTGTTGCCTGCCTTCTTCTCAGATATCC
TP25093_Query	D+G	1	chr7	.	CAGCCACGGGTCTTTATGGCAATGGCTAGGGATGGTTTGTTGCCCGCCTTCTTCTCAGATATCC
TP25159_Hit	D	1	chr7	.	CAGCCACTATTGTTCCTCCACCAACCATAAGAACAATTCCTCCAGGACCTAATTTTTTCTTCTT
TP25159_Query	D	1	chr7	.	CAGCCACTAATGTTCCTCCACCAACCATAAGAACAATTCCTCCAGGACCTAATTTTTTCTTCTT
TP25239_Hit	D	1	chr7	.	CAGCCACTCAAATCTAAAGCTCCTTTAGTCAAAGCCAAAGTGACTTTAGAAAAGTCAACTACAA
TP25239_Query	D	1	chr7	.	CAGCCACTCAAATCTAAAGCTCCTTTAGTCAAAGCCAAAGTGACTTCAGAAAAGTCAACTACAA
TP25333_Hit	D	1	chr7	.	CAGCCACTGCGGTGATGACATGAATCTATTATGGAAATGTGAAGGAACAAAGGCACGTGCTGAA
TP25333_Query	D	1	chr7	.	CAGCCACTGCGGTGATGACATGAATCTATTATGGAAATGTGAAGGAACAAAGGCACGAGCTGAA
TP25467_Hit	D	1	chr7	.	CAGCCACTTTCCGAATGCTAACAAGTGAATCGGAACAAGCCATACCCATTGTCTTTAGCACCAC
TP25467_Query	D	1	chr7	.	CAGCCACTTTCCGAATGCTAACAAGTGAATCGGAACAAGCCATACCCATTGTCTTAAGCACCAC
TP25475_Hit	D	1	chr7	.	CAGCCACTTTTCAGGTAGGCTATCAGGATTTGTGCCAGCAGGAAGAAGACGCCATTTATGACAT
TP25475_Query	D	1	chr7	.	CAGCCACTTTTCAGGTAGGCTATCAGGATTTGTGCCAGCAGGAAGAAGACGCCATTTATGACAC
TP25559_Hit	D	1	chr7	.	CAGCCAGAATTGGCTATTTAAGCTAAAGTCACTCTCAAACATAAGGGTTACGATTTTGGAGAGC
TP25559_Query	D	1	chr7	.	CAGCCAGAATTGGCTATTTAAGCTAAAGCCACTCTCAAACATAAGGGTTACGATTTTGGAGAGC
TP25710_Hit	D	1	chr7	.	CAGCCAGCAAAACACACCAAATGAAGGGGTTGAGTATTACTACTATATCCCTCCATGTATGTTA
TP25710_Query	D	1	chr7	.	CAGCCAGCAAAACACACCAAATGAAGGGGTCGAGTATTACTACTATATCCCTCCATGTATGTTA
TP25834_Hit	D	1	chr7	.	CAGCCAGCCGTGTCAGTGTCCGTGTCGTGTTCGGTGTCGGTGTCCGTATCCGTGCTTCATAGGT
TP25834_Query	D	1	chr7	.	CAGCCAGCCGTGTCAATGTCCGTGTCGTGTTCGGTGTCGGTGTCCGTATCCGTGCTTCATAGGT
TP25880_Hit	D	1	chr7	.	CAGCCAGCTCTGTGCAGTCCTGAATTACCGGTTCCATCTGAGTCTGCACAAGTTAACCATTTCA
TP25880_Query	D	1	chr7	.	CAGCCAGCTCTGTGCAGTCCTGAATTACCGGTTCCATCGGAGTCTGCACAAGTTAACCATTTCA
TP25909_Hit	D	1	chr7	.	CAGCCAGGACTCATAAGACTCTGAGAGAAGTGAGTTCACCATCATAAATTGGACGGTTTCTTCT
TP25909_Query	D	1	chr7	.	CAGCCAGGACTCATAAGACTCTGAGAGAAGTGAATTCACCATCATAAATTGGACGGTTTCTTCT
TP25992_Hit	D	1	chr7	.	CAGCCAGGGTTGGGGAGCAAAATCGTGCCGGTGAAGGTAACAGTAGTAGTACGCGAGATCAGTC
TP25992_Query	D	1	chr7	.	CAGCCAGGGTTGGGGAGCAAAATCGTGCCGGTGAAGGTAACAGTAGTAGTACGCAAGATCAGTC
TP26156_Hit	D+G	1	chr7	.	CAGCCAGTTACGGTGCAGTTTTCGTGGTATACTGCTGTCTTGAGAAACCATTTGCCACCATCCA
TP26156_Query	D+G	1	chr7	.	CAGCCAGTTACGGTGCAGTTTTCGTGGTATACTGCTGTCTTAAGAAACCATTTGCCACCATCCA
TP26299_Hit	D	1	chr7	.	CAGCCATATTTCATAAATATCTTTGTAAGCAACCTGTTCTCTGCTCGGATGAACTTCATGAAGC
TP26299_Query	D	1	chr7	.	CAGCCATAATTCATAAATATCTTTGTAAGCAACCTGTTCTCTGCTCGGATGAACTTCATGAAGC
TP26333_Hit	D+G	1	chr7	.	CAGCCATACCAAGCAATAGGAAACTAGGAATAGAAGTGAAAGACATGAAACTATCTATACAGAA
TP26333_Query	D+G	1	chr7	.	CAGCCATACCAAGCAATAAGAAACTAGGAATAGAAGTGAAAGACATGAAACTATCTATACAGAA
TP26398_Hit	D+G	1	chr7	.	CAGCCATAGCTTCATATCTTCCTCAAAGAACAGATAATGATATAAAGAATTATTGGAACACACA
TP26398_Query	D+G	1	chr7	.	CAGCCATAGCTTCATACCTTCCTCAAAGAACAGATAATGATATAAAGAATTATTGGAACACACA
TP26435_Hit	D	1	chr7	.	CAGCCCTATACGAGGAATAGAGTTTTATCAGGCGGGTCTTCATCTGTTCCCTTGAGAATTCTTG
TP26435_Query	D	1	chr7	.	CAGCCATATACGAGGAATAGAGTTTTATCAGGCGGGTCTTCATCTGTTCCCTTGAGAATTCTTG
TP26632_Hit	D+G	1	chr7	.	CAGCCATCATTGCTTCTTCAATGTCTTCTTCGCGAGTCACAAAGATTGTTGTTGATGCTTCATT
TP26632_Query	D+G	1	chr7	.	CAGCCATCATTGCTTCTTCAATGTCTTCTTCGCAAGTCACAAAGATTGTTGTTGATGCTTCATT

Name	Filter	Nb hit (Mt4.0)	Mt Chr (Mt4.0)	Ms Chr (Li et al., 2014)	Sequence
TP26917_Hit	D	1	chr7	.	CAGCCATGCATGGTAGCTCCGACGAAAATAATTGCTGGTCTGGGCTTGTCACTTGGTATTGATA
TP26917_Query	D	1	chr7	.	CAGCCATGCATGGTAGCTCCAACGAAAATAATTGCTGGTCTGGGCTTGTCACTTGGTATTGATA
TP27192_Hit	D	1	chr7	.	CAGCCATGTTTCATAAATGTGTTAAAATCTAATTTGGCGTGACATCTATCATGTAAAACAATTT
TP27192_Query	D	1	chr7	.	CAGCCATGTTTCATAAATGCGTTAAAATCTAATTTGGCGTGACATCTATCATGTAAAACAATTT
TP27271_Hit	D+G	1	chr7	.	CAGCCATTAGCTGTTGTTGTTGTGTCATCTGCTGAGCCTGCATGTAGTGTGCTCCTCCCTGGTG
TP27271_Query	D+G	1	chr7	.	CAGCCATTAGCTGTTGTTGTTGTGTCATCTGCTGAGCCTGCATGTAGTGTGCTCCTCCCTGCTG
TP27334_Hit	D+G	1	chr7	.	CAGCCATTCCATGGTGCATGACCAATCGCTGGCCATTCTCCTCCAGCATCATCAACCAAGATCA
TP27334_Query	D+G	1	chr7	.	CAGCCATTCCATGGTGCATGACCAATCGCTGGCCATTCTCCTCCAGCATCATCAACCAAAATCA
TP27536_Hit	D	1	chr7	.	CAGCCATTTCTGATAAGGGCGAGGTGTAAGTGATATGAAGGATTTTCTAGATGGAGATATTTAT
TP27536_Query	D	1	chr7	.	CAGCCATTTCTGATAAGGGCGAGGTGTAAGTGATATAAAGGATTTTCTAGATGGAGATATTTAT
TP27600_Hit	D	1	chr7	.	CAGCCATTTTGTTATCAAACTTGCCATTTGAGAGTGTTGGGAAAACAGTAGAAATAGAACAATT
TP27600_Query	D	1	chr7	.	CAGCCATTTTGTTATCAAACTTGCCATTTGAGTGTGTTGGGAAAACAGTAGAAATAGAACAATT
TP27785_Hit	D	1	chr7	.	CAGCCCAAGACCCTACATTTCCAAAACCCTGAACAAATACACAGTATGGTATCAAAATACTAAA
TP27785_Query	D	1	chr7	.	CAGCCCAAGACCCTACATTTCCAAAACCCTGAACAAATACACAATATGGTATCAAAATACTAAA
TP27896_Hit	D	1	chr7	.	CAGCCCAATTTGTTGGTGTATCAGGATTAATTGGAACATGATGATGAGTTGTTTGTGTGTAGGG
TP27896_Query	D	1	chr7	.	CAGCCCAATTTGTTAGTGTATCAGGATTAATTGGAACATGATGATGAGTTGTTTGTGTGTAGGG
TP28325_Hit	D	1	chr7	.	CAGCCCATTGTTCAGTCAATTTCTGCTCTTTGGTTTCTGCATCTCTTCTCAGCCCTGCTAACTG
TP28325_Query	D	1	chr7	.	CAGCCCATTGTTCAGTCAATTTCTGCTCTTTGGTTTCAGCATCTCTTCTCAGCCCTGCTAACTG
TP28596_Hit	D	1	chr7	.	CAGCCCCCCAAACGCGACGACACATTCCAGAAGTGAGTGGCGGCCATCCCTGCCGCTTTTGTGG
TP28596_Query	D	1	chr7	.	CAGCCCCCCAAACGCGACAACACATTCCAGAAGTGAGTGGCGGCCATCCCTGCCGCTTTTGTGG
TP2877_Hit	D	1	chr7	.	CAGCAACAACAAGAACGTTACGTGTTCTTCCACCAAGTGTGGCCATTTCAGCTTTGAGAGTTTT
TP2877_Query	D	1	chr7	.	CAGCAACAACAAGAACGTTACGTGTTCTTCCACCAAGTGTGGCCATTTCAGCTCTGAGAGTTTT
TP29005_Hit	D	1	chr7	.	CAGCCCGATCGTAAAGCAGATGAGACATGGAGTCCACATGCCTTCCAATCTTCAAAGTTTGCTC
TP29005_Query	D	1	chr7	.	CAGCCCGATCCTAAAGCAGATGAGACATGGAGTCCACATGCCTTCCAATCTTCAAAGTTTGCTC
TP29044_Hit	D	1	chr7	.	CAGCCCGCCCTTTTCACCGCCAACAATAAGTGAGAGCAAGTCATTTGGCCTAGGTGTCAAGTCA
TP29044_Query	D	1	chr7	.	CAGCCCGCCCTTTTCACCGCCAACAATAAGTGAAAGCAAGTCATTTGGCCTAGGTGTCAAGTCA
TP29147_Hit	D	1	chr7	.	CAGCCCGGTGCACTAAAGCTTCTGCATACACAGGGTCGGGGAAGGTGTTCCACCAATTGGTGTA
TP29147_Query	D	1	chr7	.	CAGCCCGGTGCACTAAAGCTTCTGCATACACAGGGTCCGGGAAGGTGTTCCACCAATTGGTGTA
TP29169_Hit	D+G	1	chr7	.	CAGCCCGTAAATTATTGTTAAACCCAATACTTCGGGGAGTTATTGATTGCTAAATTATAAGTGT
TP29169_Query	D+G	1	chr7	.	CAGCCCGTAAATTATTGTTAAACCCAATACTTCGGGGAGTTATGGATTGCTAAATTATAAGTGT
TP29354_Hit	D+G	1	chr7	.	CAGCCCTACACATCAAGATTCAATTGTGGCAGAAACTCCACTTATTGTCTTGAATTGATTTTTG
TP29354_Query	D+G	1	chr7	.	CAGCCCTACACATCAAGATTCAATTGTGACAGAAACTCCACTTATTGTCTTGAATTGATTTTTG
TP29469_Hit	D	1	chr7	.	CAGCCCTCATAAAGTACTAGCTAGGTTCTTTGCCTGTTTGATTCAACCAACATGTAAGTTGGAA
TP29469_Query	D	1	chr7	.	CAGCCCTCATAAAGTACTAGCTAGGTTCATTGCCTGTTTGATTCAACCAACATGTAAGTTGGAA
TP29616_Hit	D	1	chr7	.	CAGCCCTGGATTAGCCAGACGACCAACATCTTCAACAGATTCTTCTCCACCTTCTCCTGCTGAA
TP29616_Query	D	1	chr7	.	CAGCCCTGGATTAGCCAGACGACCAACATCTTCAACAGATTCTTCTCCACCTTCTCCTGCAGAA
TP29773_Hit	D	1	chr7	.	CAGCCCTTGGTTCCAAATCAGCCCACATTCCTTACAGGTAATTCTCTTTGGTGTCATCCATAAC
TP29773_Query	D	1	chr7	.	CAGCCCTTGCTTCCAAATCAGCCCACATTCCTTACAGGTAATTCTCTTTGGTGTCATCCATAAC
TP29815_Hit	D+G	1	chr7	.	CAGCCCTTTCTGAGCTCTTGACAGCACACGAGACAGAGATCATATGAGCAACCGGTGTTGGTGC
TP29815_Query	D+G	1	chr7	.	CAGCCCTTTCTGAGCTCTTGACAACACACGAGACAGAGATCATATGAGCAACCGGTGTTGGTGC
TP29917_Hit	D+G	1	chr7	.	CAGCCGAACCCCACAAACGTCGTAGGGAGATGGTGGATAACTGGATCCCAGCTATTTCAAAAAT
TP29917_Query	D+G	1	chr7	.	CAGCCGAACCCCACAAACGTCGTAGGGAGATAGTGGATAACTGGATCCCAGCTATTTCAAAAAT
TP29932_Hit	D	1	chr7	.	CAGCCGAAGAAAAGATGTTCCTCGCTCTAATTATTGTCGGAACAGAACCCAAATGATGAATTAT
TP29932_Query	D	1	chr7	.	CAGCCGAAGAAAAGATGTTCCTCACTCTAATTATTGTCGGAACAGAACCCAAATGATGAATTAT
TP30257_Hit	D	1	chr7	.	CAGCCGATTCAAAAGCTTTTTCAGATTCACAGGTATGTTCCACAACTTCTGCTTGGATTTTTTA
TP30257_Query	D	1	chr7	.	CAGCCGATTCAAAAGCTTTTTCAGATTCACAGGTATGTTCCACAACTTCTGCTTGGATCTTTTA
TP30350_Hit	D+G	1	chr7	.	CAGCCGCAATGCGATTGCAGTTGAGGCCACATCACATTCAACTGCGATTCTCCGCAATACTAAG
TP30350_Query	D+G	1	chr7	.	CAGCCGCAATGCGATTGCAGTTGAGGCCACATCAAATTCAACTGCGATTCTCCGCAATACTAAG
TP30411_Hit	D	1	chr7	.	CAGCCGCAGACCAGACCCTGAGATTTTGGGAAGTCTTTGGAACCCCCCCAGCTGAAAAAAAAAA
TP30411_Query	D	1	chr7	.	CAGCCGCAGACCAGACCCTGAGATTTTGGGAAGTCTTTGGAACCCCCCCAGCAGAAAAAAAAAA
TP30502_Hit	D	1	chr7	.	CAGCCGCCAAAGTAAGCAAAATACCCGTATTTCCACTTGACCGGTGACTATTGAGAACCAAATT
TP30502_Query	D	1	chr7	.	CAGCCGCCAAAGTAAGCAAAATACCCGTATTTCCACTTGACCGGTGACTATTGAGAACAAAATT
TP30509_Hit	D+G	1	chr7	.	CAGCCGCCAATGTGGTTTCCGCCAATAAAAACATTTGGCACGGTCCTCTGACCAGTCCACTGGG
TP30509_Query	D+G	1	chr7	.	CAGCCGCCAATGTGGTTTCCGCCAATAAAAACATTTGGCACAGTCCTCTGACCAGTCCACTGGG

Name	Filter	Nb hit (Mt4.0)	Mt Chr (Mt4.0)	Ms Chr (Li et al., 2014)	Sequence
TP30734_Hit	D	1	chr7	.	CAGCCGCTAAGGTAAGTGACAATCCTGCTAATGGTAATGGTAACTCCATGAACTACAAGACTCT
TP30734_Query	D	1	chr7	.	CAGCCGCTAAGGTAAGTGACAATCCTGCTAATGGTAATGGTAACTCCATGAACTACAAAACTCT
TP30878_Hit	D	1	chr7	.	CAGCCGGAAGACTCCGACGTCTCGCAACAAGCGCGAGCTGTTGCTCTCATCGATCTCGTCTCCG
TP30878_Query	D	1	chr7	.	CAGCCGGAAGACTCCGACGTCTCGCAACAAGCGCGAGCTATTGCTCTCATCGATCTCGTCTCCG
TP30915_Hit	D+G	1	chr7	.	CAGCCGGAGCCTGAATTTCGACCACAAATGTCAGAGGTGGTTCAAGCACTGGTACGATTGGTGC
TP30915_Query	D+G	1	chr7	.	CAGCCGGAGCCTGAATTTCGACCACAAATGTCAGAGGTGGTTCAAGCACTGGTACGATTAGTGC
TP3106_Hit	D	1	chr7	.	CAGCAACAATAAAAAGCAACCATTAATAACCAAAATAAGGGACTGTTTGTATTGACTTATTCGA
TP3106_Query	D	1	chr7	.	CAGCAACAATAAAAAGCAACCATCAATAACCAAAATAAGGGACTGTTTGTATTGACTTATTCGA
TP31192_Hit	D	1	chr7	.	CAGCCGGTTGAGGAAGATAGCGACGAAGAGCTCGAATATGCTGATAGTACTAGTGGTATTGCCG
TP31192_Query	D	1	chr7	.	CAGCCGGTTGAGGAAGATAGCGACGAAGAGCTCGAATATGCCGATAGTACTAGTGGTATTGCCG
TP31352_Hit	D	1	chr7	.	CAGCCGTCGTCTATTCTCTCAAGATTCTGTAACCACCACCACTTTCAAAGAAGAACGCCTTGAC
TP31352_Query	D	1	chr7	.	CAGCCGTCGTCTATTCTCTCAAGATTCTGTAATCACCACCACTTTCAAAGAAGAACGCCTTGAC
TP31423_Hit	D	1	chr7	.	CAGCCGTGCTTCTGATTTTGGATTAGTGAAGGTTGATGAAAGAGGACGGATATGCCAGTTCATG
TP31423_Query	D	1	chr7	.	CAGCCGTGCTTCTGATTTTGGATTAGTGAAGGTTGATGAAAGAGGACGGATACGCCAGTTCATG
TP31490_Hit	D+G	1	chr7	.	CAGCCGTTAAACAGAAACAGACAGAAATACCGCCCAAATAGGTAGGAACCTTGCACTGAGAACC
TP31490_Query	D+G	1	chr7	.	CAGCCGTTAAACAGAAACAGACAGAAATACCACCCAAATAGGTAGGAACCTTGCACTGAGAACC
TP32056_Hit	D	1	chr7	.	CAGCGTATAGGTAACTTTCATATATGTCCTGTAAATTGAGCATGCTATTATGTTACAGAATCTA
TP32056_Query	D	1	chr7	.	CAGCCTATAGGTAACTTTCATATATGTCCTGTAAATTGAGCATGCTATTATGTTACAGAATCTA
TP32187_Hit	D	1	chr7	.	CAGCCTATTATTTTTAGTTTTTAGTCATAGAAGCTTGAAAGTTTGCATATGAAGCAGGAAATTG
TP32187_Query	D	1	chr7	.	CAGCCTATTATTTTTAGTTTTTAGTCATAGAAGCTTGAAAGTTAGCATATGAAGCAGGAAATTG
TP32444_Hit	D	1	chr7	.	CAGCCTGAGGAAGAATTTCTATAAGAAGTCATGTCCTCAAGCCGAGGAGATTGTTAAGAACATA
TP32444_Query	D	1	chr7	.	CAGCCTCAGGAAGAATTTCTATAAGAAGTCATGTCCTCAAGCCGAGGAGATTGTTAAGAACATA
TP32445_Hit	D+G	1	chr7	.	CAGCCTCAGGAAGAATTTTTATAAGAAGTCATGTCCTCAAGCTGAGGAGATTGTTAAGAACGTA
TP32445_Query	D+G	1	chr7	.	CAGCCTCAGGAAGAATTTCTATAAGAAGTCATGTCCTCAAGCTGAGGAGATTGTTAAGAACGTA
TP32634_Hit	D	1	chr7	.	CAGCCTCCATGGCAGGCTGTGTACAGCGGATACACGAACAAAGACAACCAGCGGTAAGTAACTT
TP32634_Query	D	1	chr7	.	CAGCCTCCACGGCAGGCTGTGTACAGCGGATACACGAACAAAGACAACCAGCGGTAAGTAACTT
TP3287_Hit	D+G	1	chr7	.	CAGCAACATCAAGGTATTTGAAGTCTTCAGGGCCGCAATCAAGGCTATATTTGACCATGATTCT
TP3287_Query	D+G	1	chr7	.	CAGCAACACCAAGGTATTTGAAGTCTTCAGGGCCGCAATCAAGGCTATATTTGACCATGATTCT
TP33003_Hit	D	1	chr7	.	CAGCCTCTCAAAAGAAATATGATATAGCCCCAAACTCAAGCAAGTAAGGTATAGTCCAAGTACA
TP33003_Query	D	1	chr7	.	CAGCCTCTCAAAACAAATATGATATAGCCCCAAACTCAAGCAAGTAAGGTATAGTCCAAGTACA
TP33041_Hit	D	1	chr7	.	CAGCCTCTCCCTCATAACTCGTTCCGACAAATTCAACTGAGGACCCAGCTGGGCTGACAACAAG
TP33041_Query	D	1	chr7	.	CAGCCTCTCCCTCATAACTCGTTCCGACAAATTCAACCGAGGACCCAGCTGGGCTGACAACAAG
TP33414_Hit	D	1	chr7	.	CAGCCTGATCTCACTTTACAATACATCTCAAATCTTTCATGTCTAAAAACCATATTCATCAACA
TP33414_Query	D	1	chr7	.	CAGCCTGATCTCACTTTACAATACATCTCAAATCTTCCATGTCTAAAAACCATATTCATCAACA
TP33490_Hit	D	1	chr7	.	CAGCCTGCAACCACAAGGGATCGTTGTTTGTAGACATCGGGATATCATATCAGCCATATGACTT
TP33490_Query	D	1	chr7	.	CAGCCTGCAACCACAAGGGATCATTGTTTGTAGACATCGGGATATCATATCAGCCATATGACTT
TP336_Hit	D+G	1	chr7	.	CAGCAAAACAAGCTTATGCAGAGAGAGAGAGCACATAGTTACACTTATTTTTAAACAGGCTTCACA
TP336_Query	D+G	1	chr7	.	CAGCAAAACAAGCTTATGCAGAGAGAGAGAGCACGTAGTTACACTTATTTTTAAACAGGCTTCACA
TP33655_Hit	D+G	1	chr7	.	CAGCCTGCTTTCCAGCAGTCAAGCTCAGCATCTGCATATGTTTCAGAGTCTGGGCCTGAGGCTG
TP33655_Query	D+G	1	chr7	.	CAGCCTGCTTTCCAGCAGTCAAGCTCAGCATCTGCATATGTTTCAGAGTCTGGGCCTGAGGCAG
TP33820_Hit	D+G	1	chr7	.	CAGCCTGTCACCAATGGAGACTCAGGTTCTGTCCATGAATAAATTCAGCTCTTTTAATTAGTTT
TP33820_Query	D+G	1	chr7	.	CAGCCTGTCACCAATGGAGACTCAGGTTCTGTTCATGAATAAATTCAGCTCTTTTAATTAGTTT
TP33885_Hit	D	1	chr7	.	CAGCCTGTGCTTGCTGTCATGTATCTTGTTCAATACATGCCAACTATTGGTTTTTGGCTCATGG
TP33885_Query	D	1	chr7	.	CAGCCTGTGCTTGCTGTCATGTATCTTGTTCAATACATGCCAACTATTGGTTATTGGCTCATGG
TP34020_Hit	D	1	chr7	.	CAGCCTTAACCGTTTCCTCAGCGCTATCATGCGCCGAGTCAACGATCCCCAATCTCATCGCCTC
TP34020_Query	D	1	chr7	.	CAGCCTTAACCGTTTCCTCAGCGCTATCATGCGCCGAGTCAACGATCCCCAATCTCACCGCCTC
TP34145_Hit	D+G	1	chr7	.	CAGCCTTACTTTGTAGCAGAGACTACAATGCCTGGGAAGAGTGGGTTCGATTTCAGTGGTTCTT
TP34145_Query	D+G	1	chr7	.	CAGCCTTACTTTGTAGCAGAGACTACAATGCCTGGGAAGAGTGGGTTCGATTTCAGCGGTTCTT
TP34153_Hit	D	1	chr7	.	CAGCCTTAGAGTGTTCCATCCTCAACTCTTCAAATGTATTCTGACCAACAAAAAAAAAGAATGT
TP34153_Query	D	1	chr7	.	CAGCCTTAGAGTGTTCCATCCTCAACTCTTCAAATGTATTCTGACCAACAAAAAAAAAGAATGG
TP34307_Hit	D	1	chr7	.	CAGCCTTCGGCTCTCTCCTAACACAACCTTCCCAAAAGACTCAATTGTTGACTCATCCAGACCC
TP34307_Query	D	1	chr7	.	CAGCCTTCAGCTCTCTCCTAACACAACCTTCCCAAAAGACTCAATTGTTGACTCATCCAGACCC
TP3459_Hit	D	1	chr7	.	CAGCAACAGCAGGTATTGACATGGGACAAGCATTGAAAGGACTAGCCTCACTTGTTAATTCAGC
TP3459_Query	D	1	chr7	.	CAGCAACAGCAGGTATTGACATGGGACAAGCATTGAAAGGACTAGCCTCACTTGCTAATTCAGC

Name	Filter	Nb hit (Mt4.0)	Mt Chr (Mt4.0)	Ms Chr (Li et al., 2014)	Sequence
TP34862_Hit	D	1	chr7	.	CAGCCTTTCAACATAGTAAATTCGCCTAATATTTATAATTGTGATGTAGGCTGTGAATCAAATT
TP34862_Query	D	1	chr7	.	CAGCCTTTCAACATAGTAAATTCGCCTAATATTTATAATTGTGATGTAGGCGGTGAATCAAATT
TP3491_Hit	D	1	chr7	.	CAGCAACAGCCTTCAACCGTGCCGCCGAGAATACAAACCTGCACCACAGGTAAGATGGGGGTTA
TP3491_Query	D	1	chr7	.	CAGCAACAGCCTTCAACCGTGCCGCCGAGAATACAAACCTGCACCACAGGTAACATGGGGGTTA
TP34997_Hit	D	1	chr7	.	CAGCCTTTGGTTGAAGGCCAAAACCTTTCTTTTCAATTTTGGTTTCTCTACCTTCTCCCCAGAT
TP34997_Query	D	1	chr7	.	CAGCCTTTGGTTGAAGGCCAAAACCTTTCTTTTCAATTTTGGTTTCTCTACCTTCTCCCCAGAC
TP3517_Hit	D	1	chr7	.	CAGCAACAGGAGCAACTGCTGAAGAGGAAGAACCTTCTGACAGAATCTGTAGACCGGACGGTGA
TP3517_Query	D	1	chr7	.	CAGCAACAGGAGCAACTGCTGAAGAGGAAGAACCTTCCGACAGAATCTGTAGACCGGACGGTGA
TP35711_Hit	D	1	chr7	.	CAGCGACACATCTCTTCAATACATCCTTCTGGTTCTCTTCAGTTCCATGCTGTAGTTCAAATTT
TP35711_Query	D	1	chr7	.	CAGCGACACATCTCTTCAATACATCCTTCTGGTTCTCCTCAGTTCCATGCTGTAGTTCAAATTT
TP3581_Hit	D	1	chr7	.	CAGCGACAGTCCAATAACCATCCCTAATTTCATCTCTCAATTGTTCTTTCTCCCACCCACAATA
TP3581_Query	D	1	chr7	.	CAGCAACAGTCCAATAACCATCCCTAATTTCATCTCTCAATTGTTCTTTCTCCCACCCACAATA
TP36049_Hit	D	1	chr7	.	CAGCGACTTTGTTGTTGGGTCCACACAATGAGTTAAGGAGCCTTGTCGAAGAAAACACCGCTGA
TP36049_Query	D	1	chr7	.	CAGCGACTTTGCTGTTGGGTCCACACAATGAGTTAAGGAGCCTTGTCGAAGAAAACACCGCTGA
TP36207_Hit	D	1	chr7	.	CAGCGAGCATGGCTACTGATGCTTATGGATTGTGGATTCACGGTCGAGGTTTGTAAAGTGAAAC
TP36207_Query	D	1	chr7	.	CAGCGAGCATGGCTACTGATGCTTATGGATTGTGGATTCACGGTCGAGGTTTGTAAAGGGAAAC
TP36443_Hit	D	1	chr7	.	CAGCGAGTTGGGAAGTCTTGATATCTAAAGCGTTTTGATACAACAGACAAGGAGAACAACTCCA
TP36443_Query	D	1	chr7	.	CAGCGAGTTGGGAAGTCTTGATATCTAAAACGTTTTGATACAACAGACAAGGAGAACAACTCCA
TP36809_Hit	D+G	1	chr7	.	CAGCGATTCAGCATAATATCATAGTGGAAGGTTTGACCTTGCTAGTCATGACTCATGAGAGAAG
TP36809_Query	D+G	1	chr7	.	CAGCGATTCAGCATAATATCATAGTCGAAGGTTTGACCTTGCTAGTCATGACTCATGAGAGAAG
TP36919_Hit	D	1	chr7	.	CAGCGCAAACGGTTGACAATTGCAGTTGAGTTGGTAGCCAACCCTTCTATAATCTTCATGGATG
TP36919_Query	D	1	chr7	.	CAGCGCAAACGGTTGACAATTGCAGTTGAGTTGGTAGCCAACCCATCTATAATCTTCATGGATG
TP37098_Hit	D+G	1	chr7	.	CAGCGCAGAGGATACCAGAACAGGCATACTCCTGATAACATTGACAGTGATGATGAAGAATCGG
TP37098_Query	D+G	1	chr7	.	CAGCGCAGAGGATACCAGAACAGGCATACTCCTGATAACATCGACAGTGATGATGAAGAATCGG
TP37120_Hit	D	1	chr7	.	CAGCGCAGGCAATATCTTCCCACCCATCAAGTGCCTCCAATTGATATGTGCTAGTGGCTGAAAA
TP37120_Query	D	1	chr7	.	CAGCGCAGGCAATATCTTCCCACCCATCAAGTGCCTCCAATTGATATGTGCTAGTGGCAGAAAA
TP37300_Hit	D	1	chr7	.	CAGCGCCAAAGAGGGTTTCAGATCTGGAAAATCACTCCTTTTATTAGGAATGGGTGATGGAGAC
TP37300_Query	D	1	chr7	.	CAGCGCCAAAGAGGGTTTCAGATCCGGAAAATCACTCCTTTTATTAGGAATGGGTGATGGAGAC
TP37577_Hit	D	1	chr7	.	CAGCGCCGTACTTTTACTTATTAGACCTAGAGCCAACCTTCAGAAGTTTATGAGCAAAAATCTA
TP37577_Query	D	1	chr7	.	CAGCGCCGTACTTTTACTTATTAGACCTAGAGCCAACCTTAAGAAGTTTATGAGCAAAAATCTA
TP37614_Hit	D+G	1	chr7	.	CAGCGCCTATCTCACTATTGAAGCTCACCGGGGCTCACTCACGGCTTTAGCAGTACATAGACAC
TP37614_Query	D+G	1	chr7	.	CAGCGCCTATCTCACTATTGAAGCTCACCGGGGCTCACTCACGGCATTAGCAGTACATAGACAC
TP37928_Hit	D	1	chr7	.	CAGCGCGGTCTGTTGCATGCAGAGCTATGATACATCCATCTTTCAAAAGTTCAGTACATTTTCC
TP37928_Query	D	1	chr7	.	CAGCGCGGTCTGTTGCATGCAGAGCTATGATACACCCATCTTTCAAAAGTTCAGTACATTTTCC
TP37996_Hit	D	1	chr7	.	CAGCGCGTGTTTCCCACGTTCCCCTTGTGAAGAAAGTGTCGAGTGATGCAGTTAGAATCAGTTG
TP37996_Query	D	1	chr7	.	CAGCGCGTGTTTCCCACGTTCCCCCTGTGAAGAAAGTGTCGAGTGATGCAGTTAGAATCAGTTG
TP38465_Hit	D	1	chr7	.	CAGCGGAATCTCTGGTTTCCCTTTTGGAGAAAGCGGAGGGTGTTGTTCCACGGGAATTGCGATC
TP38465_Query	D	1	chr7	.	CAGCGGAATCTCTGGTTTCCCTTTTGGAAAAAGCGGAGGGTGTTGTTCCACGGGAATTGCGATC
TP38486_Hit	D	1	chr7	.	CAGCGGACAACTCTCCTCCATGGCCTGCTCCGTCGGAGACATTATCCGTTGAACATAATACTAG
TP38486_Query	D	1	chr7	.	CAGCGGACAACTCTCCGCCATGGCCTGCTCCGTCGGAGACATTATCCGTTGAACATAATACTAG
TP38559_Hit	D	1	chr7	.	CAGCGGAGAATCATCATCGTCGTTGTAACATACCTTTACCTACGGTGAAGCCTATCCAACGGTT
TP38559_Query	D	1	chr7	.	CAGCGGAGAATCATCATCGGCGTTGTAACATACCTTTACCTACGGTGAAGCCTATCCAACGGTT
TP38590_Hit	D	1	chr7	.	CAGCGGAGCAAGAAATTAGGGTTAAAGAAAAAGACAAGTTGCAAAGAGGATTATGAACAAATGA
TP38590_Query	D	1	chr7	.	CAGCGGAGCAAGAAAGTAGGGTTAAAGAAAAAGACAAGTTGCAAAGAGGATTATGAACAAATGA
TP38669_Hit	D	1	chr7	.	CAGCGGATACAAAGCACAGGCTCTGAGTATCAAGCATCGAGAAGATCTGCACATCATAACACTT
TP38669_Query	D	1	chr7	.	CAGCGGATACAAAGCACAGGCTCTGAGTATCAAGCATCGAGAAGATCTGCACATCATAACACCT
TP38685_Hit	D	1	chr7	.	CAGCGGATATTTAGTCCTGGTGGAACTTCCAAAACTGATTTCATTTTAGATAAGCTGAATCAAC
TP38685_Query	D	1	chr7	.	CAGCGGATATTTAGTCCTGGTGGAACTTCCAAAACTGATTTCATTCTAGATAAGCTGAATCAAC
TP38786_Hit	D+G	1	chr7	.	CAGCGGCAACAACAGCATTCATCAGTATTCCACGGAAACCCGCTTTTTCCAACTCATGAACGCC
TP38786_Query	D+G	1	chr7	.	CAGCGGCAACAACAGCATTCATCAGTATTCCACGGAAACCCGCTTTTTCCAACTCATGAACACC
TP38969_Hit	D	1	chr7	.	CAGCGGCCATTCCTGACGTGTTTGAGCGTAGTGATCGACACACACAAGAGGATGATGAATATCA
TP38969_Query	D	1	chr7	.	CAGCGGCCATTCCGGACGTGTTTGAGCGTAGTGATCGACACACACAAGAGGATGATGAATATCA
TP39037_Hit	D+G	1	chr7	.	CAGCGGCCTTCTTTGATTTCTTTTGCGGTCTGCACAAAAGATATTCATTTAATCAATCATGCAT
TP39037_Query	D+G	1	chr7	.	CAGCGGCCTTCTTTGATTTCTTTTGCGGTCTGCACAAAAGATATTCATTTAAACAATCATGCAT

Name	Filter	Nb hit (Mt4.0)	Mt Chr (Mt4.0)	Ms Chr (Li et al., 2014)	Sequence
TP39051_Hit	D+G	1	chr7	.	CAGCGGCGAATATTTCACTCGCATCGGTGTTGGAACTCCAGCTAGGTATGTTTTCATGGTTCTC
TP39051_Query	D+G	1	chr7	.	CAGCGGCGAATATTTCACTCGCATCGGCGTTGGAACTCCAGCTAGGTATGTTTTCATGGTTCTC
TP39171_Hit	D	1	chr7	.	CAGCGGCGGCCAAGGCGGCTAGTATGATGAAGGCTTCGTTGGATGGGAAGGGTTGCATTGTGTC
TP39171_Query	D	1	chr7	.	CAGCGGCGGCCAAGGCGGCTAGTATGATGAAGGCTTCGTTGGATGAGAAGGGTTGCATTGTGTC
TP3927_Hit	D	1	chr7	.	CAGCAACCAATGTTTGTGAGATCTCATCAGAACAATTACCAATGTTTCCTAATTTACCAATCAA
TP3927_Query	D	1	chr7	.	CAGCAACCAATGTTTGTGAGATCTCATCAGAACAATTACCAATGTTTCCTAATTTAACAATCAA
TP39277_Hit	D	1	chr7	.	CAGCGGCTCCAGTGGTGCCGTTGCCTCCAAGACATCCAGGATCAACGTCAAAAGCGAGTTTACA
TP39277_Query	D	1	chr7	.	CAGCGGCTCCAGTGGTGCCATTGCCTCCAAGACATCCAGGATCAACGTCAAAAGCGAGTTTACA
TP3929_Hit	D	1	chr7	.	CAGCAACCAATTCTCGACCATGGAAATTTCACGTGTTCTTGAGTTTCAGAGGAGTAGAAACTCG
TP3929_Query	D	1	chr7	.	CAGCAACCAATTCTCCACCATGGAAATTTCACGTGTTCTTGAGTTTCAGAGGAGTAGAAACTCG
TP39355_Hit	D	1	chr7	.	CAGCGGCTTTTTACCGATTGAGAGGCGCCAATGTGCTTCTGCCATTTGCCTTTCATTGCACCGG
TP39355_Query	D	1	chr7	.	CAGCGGCTTTTTACCGATTGAGAGGCGCCAATGTGCTTCTGCCATTTGCCTTTCATTGCACAGG
TP39532_Hit	D	1	chr7	.	CAGCGGGGGCTTTAGGTGTTTTGAATAGGATGAAAGATGAGGGGGTGAAGATGGATACGGTTAC
TP39532_Query	D	1	chr7	.	CAGCGGGGGCGTTAGGTGTTTTGAATAGGATGAAAGATGAGGGGGTGAAGATGGATACGGTTAC
TP39779_Hit	D	1	chr7	.	CAGCGGTCTATAGAATTTGCTTTGTGTTATTTGGAGAAATCTGGATTTGATAATGATGATCTAG
TP39779_Query	D	1	chr7	.	CAGCGGTCTATAGAATTTACTTTGTGTTATTTGGAGAAATCTGGATTTGATAATGATGATCTAG
TP39850_Hit	D	1	chr7	.	CAGCGGTGCTACGTCTGGTTTAACCTCAAGTCTTGCATTTACTCCAGTGCAGGTTAGTTTCACT
TP39850_Query	D	1	chr7	.	CAGCGGTGCTACATCTGGTTTAACCTCAAGTCTTGCATTTACTCCAGTGCAGGTTAGTTTCACT
TP39887_Hit	D	1	chr7	.	CAGCGGTGGCATGTGTAGTGTATGTGCCCGGCACATGCGATTACATTCAATTATGTCATTTTCT
TP39887_Query	D	1	chr7	.	CAGCGGTGGCATGTGTAGTGTATGTGCCCGGCACATGCGATTACATTCAATTATGTCATTTTAT
TP39909_Hit	D	1	chr7	.	CAGCGGTGGGGGTTGGGGGTTTTGGGCTTCCTACTGTGTGTGAATGTGGCCTGCTGGTGTCTTG
TP39909_Query	D	1	chr7	.	CAGCGGTGGGGGTTGGGGGTTTTGGGCTTCCTACTGTGTGTAAATGTGGCCTGCTGGTGTCTTG
TP39957_Hit	D+G	1	chr7	.	CAGCGGTGTGGAATCCGTGGGAGAAGAAATCAAAGTCGATGACGGACTTTGGTGATGAGGAGTA
TP39957_Query	D+G	1	chr7	.	CAGCGGTGTGGAATCCGTGGGAGAAGAAATCAAAGTCGATGACAGACTTTGGTGATGAGGAGTA
TP40106_Hit	D	1	chr7	.	CAGCGTAAAAAGCCACAATCAATACGATGTTTTGTTCAATAGTTAATTCTGACTTCGTGTGTTA
TP40106_Query	D	1	chr7	.	CAGCGTAAAAAACCACAATCAATACGATGTTTTGTTCAATAGTTAATTCTGACTTCGTGTGTTA
TP40142_Hit	D	1	chr7	.	CAGCGTAACAGTTGGTGTTTCCCACTTTGAAAGATGTGCCCTTTGGGTTCAAGTGTTGTATTAC
TP40142_Query	D	1	chr7	.	CAGCGTAACAGTTGGTGTTTCCCACTTTGAAAGATGCGCCCTTTGGGTTCAAGTGTTGTATTAC
TP40416_Hit	D	1	chr7	.	CAGCGTCAAGAAAGAGAATTGGCTAAGTTTGCCCAAATGTATCCTTTTGCCTTTTAAATTATTT
TP40416_Query	D	1	chr7	.	CAGCGTCAAGAAAGAGAATTGGCTAAGTTTGCCCAAATGTATCCTTTTGCCTGTTAAATTATTT
TP40472_Hit	D	1	chr7	.	CAGCGTCAGTTGGGAGTTATGGAGCTTATTTGGCTGATGGATCAGAGTACAGGTGAAATTTTCT
TP40472_Query	D	1	chr7	.	CAGCGTCAGTCGGGAGTTATGGAGCTTATTTGGCTGATGGATCAGAGTACAGGTGAAATTTTCT
TP40551_Hit	D	1	chr7	.	CAGCGTCGAACCATGGCCAGGGCTGTATCGGCATATATATCCTCTTCTACATCAGATGTCAGAC
TP40551_Query	D	1	chr7	.	CAGCGTCGAACCATGGCCAGGGCTGTATCAGCATATATATCCTCTTCTACATCAGATGTCAGAC
TP40645_Hit	D	1	chr7	.	CAGCGTCTACTTTCAAATCCTCAATTTCCATATCCTTCGCTGAAAAAAAAAAAAAAAAAAAAAA
TP40645_Query	D	1	chr7	.	CAGCGTCTACCTTCAAATCCTCAATTTCCATATCCTTCGCTGAAAAAAAAAAAAAAAAAAAAAA
TP40844_Hit	D	1	chr7	.	CAGCGTGATCATCACGCGCCACATCAAAATCCTTAAAATTAAACTCCCAAATATAACTATTTTC
TP40844_Query	D	1	chr7	.	CAGCGTGATCATCACGAGCCACATCAAAATCCTTAAAATTAAACTCCCAAATATAACTATTTTC
TP41421_Hit	D	1	chr7	.	CAGCGTTGCAAGGAAGAGAGACAAAAACGTGCAAGGAAGTGCATCAGTCGTTGAACTCGAATCT
TP41421_Query	D	1	chr7	.	CAGCGTTGCAAGGAAGAGAAACAAAAACGTGCAAGGAAGTGCATCAGTCGTTGAACTCGAATCT
TP41542_Hit	D	1	chr7	.	CAGCGTTTCAAGGAAGAGAGACAAAAACGTGCAAGGAAGTGAATTGCCCTCAATCTTTCTCTCC
TP41542_Query	D	1	chr7	.	CAGCGTTTCAAGGAAGAGAGACAAAAACGTGCAAGGAAGTGAATCGCCCTCAATCTTTCTCTCC
TP4198_Hit	D	1	chr7	.	CAGCAACCTCCTCCTGAAGAACTCCACACGCGCGTCACATTATTGATTCGCTCCGGCAACCTCG
TP4198_Query	D	1	chr7	.	CAGCAACCTCCTCCTGAAGAACTCCACACGCGCGTCACATCATTGATTCGCTCCGGCAACCTCG
TP42040_Hit	D	1	chr7	.	CAGCTAAATCTTCAACAGAGTGCTTGGGCATTCCGGCATATTGCATAATACAGTGAAAGGTTGC
TP42040_Query	D	1	chr7	.	CAGCTAAATCTTCAACAGAGTGCTTGGGAATTCCGGCATATTGCATAATACAGTGAAAGGTTGC
TP42078_Hit	D	1	chr7	.	CAGCTAAATTGTGTATCTTTTTGTACATGAATCTGTTTTGATTATGGTTATATTTGATCTGAAA
TP42078_Query	D	1	chr7	.	CAGCTAAATTATGTATCTTTTTGTACATGAATCTGTTTTGATTATGGTTATATTTGATCTGAAA
TP42083_Hit	D	1	chr7	.	CAGCTAAATTCTGATAAGATTGTCCAAAAAAATTAAGGATATTGATCCTAATGTTGGCTTGCCA
TP42083_Query	D	1	chr7	.	CAGCTAAATTCTGATAAGATTGTCCAAAAAAATTAACGATATTGATCCTAATGTTGGCTTGCCA
TP42279_Hit	D	1	chr7	.	CAGCTAACTCTATCTGTGTCTGGTAGATAAATTCCAAAACTGCTGAAATATCTTTCTCAAACTT
TP42279_Query	D	1	chr7	.	CAGCTAACTCAATCTGTGTCTGGTAGATAAATTCCAAAACTGCTGAAATATCTTTCTCAAACTT
TP42517_Hit	D	1	chr7	.	CAGCTAAGTGGTTGAGTGGAGACACCCTGTTGAGAATGAGGTCTAGGCTCCCCTACAAACATAA
TP42517_Query	D	1	chr7	.	CAGCTAAGTGGTTGAGCGGAGACACCCTGTTGAGAATGAGGTCTAGGCTCCCCTACAAACATAA

Name	Filter	Nb hit (Mt4.0)	Mt Chr (Mt4.0)	Ms Chr (Li et al., 2014)	Sequence
TP42520_Hit	D	1	chr7	.	CAGCTAAGTGTTGTCCTTTATAGTATGGTGCAACATGAGGAATTCTTCCATCCTTGCTTTTAGG
TP42520_Query	D	1	chr7	.	CAGCTAAGTGTTGTCCTTTATAGTATGGTGCAACATGAGGAATACTTCCATCCTTGCTTTTAGG
TP42555_Hit	D+G	1	chr7	.	CAGCTAATAAGCCTATAACTTCAAGCTTGGTGTTGTTATTTCCTCTAATTCTTGTGGCTTTTGC
TP42555_Query	D+G	1	chr7	.	CAGCTAATAAGCCTATAACTTCAAGCTTGGTGTTGTTATTTCCTCTAATTCTTGTGGCTTTTAC
TP42556_Hit	D	1	chr7	.	CAGCTAATAAGGTCTGTCAATTTTTATCCCAGCCTCTTGAGAGTCACTGGACAGCTGTATAGAG
TP42556_Query	D	1	chr7	.	CAGCTAATAAGGCCTGTCAATTTTTATCCCAGCCTCTTGAGAGTCACTGGACAGCTGTATAGAG
TP42687_Hit	D	1	chr7	.	CAGCTAATGACAACAAGTCTTCAATGTCAATCACACACCTACACAGTTGAAATGAGCATAAGAC
TP42687_Query	D	1	chr7	.	CAGCTAATGACAACAAGTCTTCAATGTCAATCACACACCTACACAGTTGAAATGAGCATAAAAC
TP42736_Hit	D+G	1	chr7	.	CAGCTAATGGCTTCTTTCTGTATTGCAGACCTTCAAGTGGCAAGACTACATGGACTGGCAGAGA
TP42736_Query	D+G	1	chr7	.	CAGCTAATGGCTTCTTTCTGTATCGCAGACCTTCAAGTGGCAAGACTACATGGACTGGCAGAGA
TP42861_Hit	D	1	chr7	.	CAGCTACAAAACCAAATTATACATCAGTAAAAATCACTGTCATAGAGAAATGGAAATGGTAAAT
TP42861_Query	D	1	chr7	.	CAGCTACAAAACCAAATTATACATCAATAAAAATCACTGTCATAGAGAAATGGAAATGGTAAAT
TP43269_Hit	D	1	chr7	.	CAGCTACCAGAATGTCGTCTAGCTCTGGGATTCTGGGGTTGCCAACTAGAGACTGTGAGTTTTT
TP43269_Query	D	1	chr7	.	CAGCTACCAGAATGTCGTCTAGCTCTGGGATTCAGGGGTTGCCAACTAGAGACTGTGAGTTTTT
TP4328_Hit	D	1	chr7	.	CAGCAACGATCTCAACAACCTTAGTTCTTTTCATCAAAAAAGGTAAAAAGGGTGAAGGAGAAGA
TP4328_Query	D	1	chr7	.	CAGCAACGATCTCAACAACCTTAGTTCTTTTCATCAAAAAAGGCAAAAAGGGTGAAGGAGAAGA
TP4342_Hit	D+G	1	chr7	.	CAGCAACGCAATGTCATCAACAACCGGCGCAACACCGCGAAGACCAATCCAAATCACCATACCA
TP4342_Query	D+G	1	chr7	.	CAGCAACGCAATGTCATCAACAACCGGCGCAACACCGCGAAGACCAATCCAAATCAACATACCA
TP43479_Hit	D	1	chr7	.	CAGCTACTAATACATCCTCTTCTTGTGGGGGAATTTTCAGGAATAAAGACTCTATGTTCCTCCT
TP43479_Query	D	1	chr7	.	CAGCTACTAATACATCCTCTTCTTGTGGGCGAATTTTCAGGAATAAAGACTCTATGTTCCTCCT
TP43510_Hit	D	1	chr7	.	CAGCTACTATCATTGTTTGTTTAAATATCCTTTAACTATTAAAACCATCCCCAAGTTTGATTCT
TP43510_Query	D	1	chr7	.	CAGCTACTATCATTGTTTGTTTAAATATCCTTTAACTATTAAAACCATCCCCAAGATTGATTCT
TP43566_Hit	D	1	chr7	.	CAGCTACTCTGGGAACTCTCTGTTATTGGGTGATTGCTCACCGCCTCGAAGCCTCCAGATTACG
TP43566_Query	D	1	chr7	.	CAGCTACTCTAGGAACTCTCTGTTATTGGGTGATTGCTCACCGCCTCGAAGCCTCCAGATTACG
TP43615_Hit	D	1	chr7	.	CAGCTACTGCTAAACTATTTGTTCAACATGGTGCTAAAGTTGTGATTTCAGATATTCAAGATGA
TP43615_Query	D	1	chr7	.	CAGCTACTGCTAAACTATTTGTTCAACATGGTGCTAAAGTTGTGATTGCAGATATTCAAGATGA
TP43687_Hit	D+G	1	chr7	.	CAGCTACTTTAAATGAATGATCCAATTTTTTGGCTATGCATGGATTGACTTGTGTCGTGAGCTG
TP43687_Query	D+G	1	chr7	.	CAGCTACTTTAAATGAATGATCCAATTTTTTGGCTATGCATGGATTGACTTGTGTCGTGAGCTA
TP43707_Hit	D	1	chr7	.	CAGCTACTTTTTGACCGTGGACTACAAGATAAAGCATTCAAAATTACTGAGTTAATTTATAAGA
TP43707_Query	D	1	chr7	.	CAGCTACTTTTTGACCGTGGACTACAAGATAAAGCATTCAAAATTACTGAGCTAATTTATAAGA
TP43731_Hit	D	1	chr7	.	CAGCTAGAACAACAGATGATGCTATTAACTCCAAATCCTTCTGACTCAGATCTTTGTTGAAGCT
TP43731_Query	D	1	chr7	.	CAGCTAGAACAACAGATGATGCTATTAACTCCAAATCCTTCTGACTCAGATCTTTATTGAAGCT
TP43765_Hit	D	1	chr7	.	CAGCTAGAAGGTCTTTAAATGCAAAGAAAGATGTTAATGACTTCAGTTTAATGATGGAAGATTG
TP43765_Query	D	1	chr7	.	CAGCTAGAAGGTCTTTAAATGCAAAGAAAGATGTTAATGACTTCAGTTTAACGATGGAAGATTG
TP43849_Hit	D	1	chr7	.	CAGCTAGAGCTTATGATGAAGCCGCGTGCCTTCTTCGAGGTTCGAACACTCGAACCAACTTCAT
TP43849_Query	D	1	chr7	.	CAGCTAGAGCTTATGATGAAGCCGCGTGCCTTCTTCGAGGATCGAACACTCGAACCAACTTCAT
TP44012_Hit	D	1	chr7	.	CAGCTAGCATAAATATCTCTCATTTTCTTTACCCCGAGAGAACTGGAAAACAATTAGCAATGTC
TP44012_Query	D	1	chr7	.	CAGCTAGCATAAATATCTCTCATTTTCTTTACCCCAAGAGAACTGGAAAACAATTAGCAATGTC
TP44086_Hit	D	1	chr7	.	CAGCTAGCTCGGTAGATAATCTCTCATTATGGTTCATGAGTTTGGCAACTTCTTCTGACAGTGC
TP44086_Query	D	1	chr7	.	CAGCTAGCTCGGCAGATAATCTCTCATTATGGTTCATGAGTTTGGCAACTTCTTCTGACAGTGC
TP44159_Hit	D	1	chr7	.	CAGCTAGGCCATATTAATCTTCACATCTCAGCACAAAGTGGCAACATGACCCATTGCAGGCATT
TP44159_Query	D	1	chr7	.	CAGCTAGGCAATATTAATCTTCACATCTCAGCACAAAGTGGCAACATGACCCATTGCAGGCATT
TP44523_Hit	D	1	chr7	.	CAGCTATAAGGAATTCGATGCCAACAGCATTCTTTAAAGCAAATACATATTCTTCAAATGGCAT
TP44523_Query	D	1	chr7	.	CAGCTATAAGGAAGTCGATGCCAACAGCATTCTTTAAAGCAAATACATATTCTTCAAATGGCAT
TP44559_Hit	D+G	1	chr7	.	CAGCTATAATTGAAAATGCTGAAAATTCAATAGAGAAGCAAAGTCCATGGAAGCTAACAACTGT
TP44559_Query	D+G	1	chr7	.	CAGCTATAATTGAAAATGCTGAAAATTCAATAGAGAAGCAAAGTCCATGGAAGCTAACAACTAT
TP44576_Hit	D	1	chr7	.	CAGCTTTACAGCCAAGAAAAACAATGAATTAAAGGGGAATAATTTCATGAAATGTGATTTTATG
TP44576_Query	D	1	chr7	.	CAGCTATACAGCCAAGAAAAACAATGAATTAAAGGGGAATAATTTCATGAAATGTGATTTTATG
TP44684_Hit	D	1	chr7	.	CAGCTATAGGGCTATGGCATAGCAGAATTTAAACAAAACTGCTACTGGTCGGCGGTACGCTAAT
TP44684_Query	D	1	chr7	.	CAGCTATAGGGCTATGGCATAGCAGAATTTAAACAAAACCGCTACTGGTCGGCGGTACGCTAAT
TP45079_Hit	D+G	1	chr7	.	CAGCTATGATTGGCCTTTTTGGACAAGCTACAGACAGGCTCTGCCAGAAGCTAGATACTGCTGA
TP45079_Query	D+G	1	chr7	.	CAGCTATGATCGGCCTTTTTGGACAAGCTACAGACAGGCTCTGCCAGAAGCTAGATACTGCTGA
TP45143_Hit	D	1	chr7	.	CAGCTATGCTTAACAATGTAGCAAATGGGAAATTCCTTCTGTCAAATCCAGTGAACTGTAACTC
TP45143_Query	D	1	chr7	.	CAGCTATGCTTAACAATGTAGCAAATGGGAAATTCCTTCTGTCAAATCCAGTGAACCGTAACTC

Name	Filter	Nb hit (Mt4.0)	Mt Chr (Mt4.0)	Ms Chr (Li et al., 2014)	Sequence
TP45166_Hit	D	1	chr7	.	CAGCTATGGAGAAAGGTATTGTTGTTTCGTCTTCTGCAGGTAACTCGGGACCTAAGCATGGAAC
TP45166_Query	D	1	chr7	.	CAGCTATGGAGAAAGGTATTGTTGTTTCATCTTCTGCAGGTAACTCGGGACCTAAGCATGGAAC
TP45277_Hit	D	1	chr7	.	CAGCTATGTGTTCCTCAAACCTGTGAAGCCATCGGGTAGGTGAATTTGAAGAAACCACAGATCT
TP45277_Query	D	1	chr7	.	CAGCTATGTGTTCCTCAAACCTGTGAAGCCATCGGGTAGGTGAATCTGAAGAAACCACAGATCT
TP45297_Hit	D	1	chr7	.	CAGCTGTGTTGTTCTTTAGTGGCATCTTGTAAATAAAGGTGGTCAAATTTGTGGTTTGTATGTG
TP45297_Query	D	1	chr7	.	CAGCTATGTTGTTCTTTAGTGGCATCTTGTAAATAAAGGTGGTCAAATTTGTGGTTTGTATGTG
TP45418_Hit	D	1	chr7	.	CAGCTATTCTAACTCTTCAGCCTTAAGGTTGACATCCACCCCTCGAGTGTCATGAGCAGAAAAA
TP45418_Query	D	1	chr7	.	CAGCTATTCTAACTCTTCAGCCTTAAGGTTGACATCCACCCCTCGAGTGTCAAGAGCAGAAAAA
TP45463_Hit	D	1	chr7	.	CAGCTATTGCGGGACTTTTTTAAGGGGAAGGATCTTTACAGTAGCATCAATCCAGATGAGGCTG
TP45463_Query	D	1	chr7	.	CAGCTATTGCAGGACTTTTTTAAGGGGAAGGATCTTTACAGTAGCATCAATCCAGATGAGGCTG
TP45475_Hit	D	1	chr7	.	CAGCTATTGCGTATGGACTGGACAAGAGAACTAATTGTGTGGAGGAGCGTAGTATTTTCATCTT
TP45475_Query	D	1	chr7	.	CAGCTATTGCGTATGGACTGGACAAGAGAACTAATTGTGTGGAGGAGCGTAATATTTTCATCTT
TP45480_Hit	D	1	chr7	.	CAGCTATTGCTTTATGGTTAAACTACTTTGTGACTTATGCCGGACTTGAATAAAGTTAAACTAC
TP45480_Query	D	1	chr7	.	CAGCTATTGCTTTATGGTTAAACTACTTTGTGACTTATGCCGGACTTGAATAAAGTTAAACGAC
TP45497_Hit	D	1	chr7	.	CAGCTATTGGGTACTTATTGGTGCAAATCTTTGGGATCTAGGTAATGAATTGCCTTATCTTATC
TP45497_Query	D	1	chr7	.	CAGCTATTGGGTACTTATTGGTGCAAATCTTTGGGATCCAGGTAATGAATTGCCTTATCTTATC
TP45693_Hit	D	1	chr7	.	CAGCTCAAAAGAGAAAAGCTAACTCCTTTGAATTTGGAAGTAGTAGTTGTGTTAAAGTTGGGAA
TP45693_Query	D	1	chr7	.	CAGCTCAAAAGAGAAAAGCTAACTCCTTTGAATTTCGAAGTAGTAGTTGTGTTAAAGTTGGGAA
TP4572_Hit	D	1	chr7	.	CAGCAACTATGGTTACAGGAATAGTTGGTTCCGAGAGGATTGTTTTAAGAGGTAATTGTGTTAG
TP4572_Query	D	1	chr7	.	CAGCAACTATGGTTACAGGAATAGTTGGTTACGAGAGGATTGTTTTAAGAGGTAATTGTGTTAG
TP4586_Hit	D+G	1	chr7	.	CAGCAACTATTGCAGAACTTCTTCAAGGGGAAGGAATTATGCAAGAGTATCAACCCTGATGAGG
TP4586_Query	D+G	1	chr7	.	CAGCAACTATTGCAGAACTTCTTCAAGGGAAAGGAATTATGCAAGAGTATCAACCCTGATGAGG
TP4588_Hit	D+G	1	chr7	.	CAGCAACTATTGCAGGATTTCTTCAAGGGGAAGAAGTTATGCAAGAGTATCAACCCTGATGAGG
TP4588_Query	D+G	1	chr7	.	CAGCAACTATTGCAGGATTTCTTCAAGGGGAAGAAATTATGCAAGAGTATCAACCCTGATGAGG
TP45981_Hit	D	1	chr7	.	CAGCTCAAGCTTATGGAAGATTAGGCCCTAAAGATGTGTGTAAACCAGCTCATCAAGAGCTGGC
TP45981_Query	D	1	chr7	.	CAGCTCAAGCTTATGGAAGATTAGGCCCTAAAGATGTGTGTAAACCAGCTCATCAAGAGCTAGC
TP46032_Hit	D	1	chr7	.	CAGCTCAATATCTGCTATTCTATCTACGATCATCGGATTGCATTATGGACATGTACTCATAGAC
TP46032_Query	D	1	chr7	.	CAGCTCAATATCTGCTATTCTATCTACGATCATCGGATTGCATTATGGACATGTACTCATACAC
TP46354_Hit	D+G	1	chr7	.	CAGCTCCGAATCCAATTCCAGAAACAGCATTTTACAATAACCGAAGGAATAGAAGCATTCAGCC
TP46354_Query	D+G	1	chr7	.	CAGCTCAGAATCCAATTCCAGAAACAGCATTTTACAATAACCGAAGGAATAGAAGCATTCAGCC
TP46360_Hit	D	1	chr7	.	CAGCTCAGACAAATCTTTGCTAGTAAAAAGCTTACTCTTCATGACTTGGTTGTATTATCAGGTA
TP46360_Query	D	1	chr7	.	CAGCTCAGACAAATCTTTGCTAGCAAAAAGCTTACTCTTCATGACTTGGTTGTATTATCAGGTA
TP46468_Hit	D+G	1	chr7	.	CAGCTCAGCCAGTGCTCCTGAGTCCAATGTTCATGATGGTGATGCTGTCGGTTTGGCTGAAAAA
TP46468_Query	D+G	1	chr7	.	CAGCTCAGCCAGTGCTCCTGAGTCCAATGTTCATGATGGTGATGCTGTCGGTTTGGCAGAAAAA
TP46499_Hit	D	1	chr7	.	CAGCTCAGGAACTAGTGACAGCACAAGACAAGAGACATCTGAACCTGTTCCTGGATTTGGTGAA
TP46499_Query	D	1	chr7	.	CAGCTCAGGAACTAGTGACAGCACAAGACAAGAGACATCTGAACCTGTTCCTGGATTTGGAGAA
TP46538_Hit	D	1	chr7	.	CAGCTCAGGTGTCAAGGAGCCTGAACCACCATTTATATTACTTCTCTCAACACATGGTTCATTT
TP46538_Query	D	1	chr7	.	CAGCTCAGGTGTCAAGGAGCCTGAACCACCATTTATAGTACTTCTCTCAACACATGGTTCATTT
TP46710_Hit	D	1	chr7	.	CAGCTCATCCAGTTACACCACTAGTATCCCTACATCACTTGGACCTAATTGAGCCCATATTTCC
TP46710_Query	D	1	chr7	.	CAGCTCATCCAGTTACACCACTAGTATCCCTACATCACTTGGACCTAATTGAACCCATATTTCC
TP46847_Hit	D+G	1	chr7	.	CAGCTCATTACTCATCAAGAGGTCCATCACATGGCTTTCCTTGGATTTTGAAGCCTGACATAAT
TP46847_Query	D+G	1	chr7	.	CAGCTCATTACTCATCAAGAGGTCCATCACATGGCTTTCCATGGATTTTGAAGCCTGACATAAT
TP46867_Hit	D	1	chr7	.	CAGCTCATTCATCCAAAAAAAAAAAAAATGAATGGTTTCTATAATTCTATAGATATTACTTAATA
TP46867_Query	D	1	chr7	.	CAGCTCATTCATCCAAAAAAAAAAAAAATGAATGGTTTCTATAATTCTATACATATTACTTAATA
TP46868_Hit	D	1	chr7	.	CAGCTCATTCATCCAAAAAAAAAAAATGAATGGTTTCTATAATTCTATAGATATTACTTAATATG
TP46868_Query	D	1	chr7	.	CAGCTCATTCATCCAAAAAAAAAAAATGAATGGTTTCTATAATTCTATACATATTACTTAATATG
TP46869_Hit	D	1	chr7	.	CAGCTCATTCATCCAAAAAAAAAAATGAATGGTTTCTATAATTCTATAGATATTACTTAATATGT
TP46869_Query	D	1	chr7	.	CAGCTCATTCATCCAAAAAAAAAAATGAATGGTTTCTATAATTCTATACATATTACTTAATATGT
TP46918_Hit	D	1	chr7	.	CAGCTCATTTGTAAAAGTGACCAAACGGCTTGTTGATCTTGATCTGTCTATTTCCTCCATCGTT
TP46918_Query	D	1	chr7	.	CAGCTCATTTGTAAAAGTGACCAAACGGCTTGTTGATCTTGATCTGTCTATTCCCTCCATCGTT
TP46961_Hit	D	1	chr7	.	CAGCTCCAAACGTAATTGCCGCAAAACCAAAACGTAAATAACACTAGTTCCGCAGTCCCGCCGC
TP46961_Query	D	1	chr7	.	CAGCTCCAAACGTAATTGCCGCAAAACCAAAACGTAAATAACACTAGTTCCGCAGTCCCACCGC
TP471_Hit	D	1	chr7	.	CAGCAAAACGGCAATCGTATGACCCCTTGGCAATAAAGGATTTGATACCATGTGTCAAAAACCA
TP471_Query	D	1	chr7	.	CAGCAAAACGCCAATCGTATGACCCCTTGGCAATAAAGGATTTGATACCATGTGTCAAAAACCA

Name	Filter	Nb hit (Mt4.0)	Mt Chr (Mt4.0)	Ms Chr (Li et al., 2014)	Sequence
TP47158_Hit	D	1	chr7	.	CAGCTCCAGAAAGGGGAATCTATTTCACTCCAACTTCGACAGGCAATTCAACACTCACGTGTTT
TP47158_Query	D	1	chr7	.	CAGCTCCAGAAAGGGGAATCTATTTCACTCCAACTTCGACAGGCAATTCAACAATCACGTGTTT
TP47281_Hit	D	1	chr7	.	CAGCTCCATCGATCCACAATTTCCAAACTATTATATACAAATTTAGGTCTAAGATATATGAGTT
TP47281_Query	D	1	chr7	.	CAGCTCCATCGATCCACAATTTCCAAACTATTATATACAAATTTAGGTCTAAGATATATGAGTG
TP47293_Hit	D+G	1	chr7	.	CAGCTCCATCTTCAACGGTTCCGCCTTCTTCAACTCCGCCTCCATCTTTGCAATCTTATCGATC
TP47293_Query	D+G	1	chr7	.	CAGCTCCATCTTCAACGGTTCCGCCTTCTTCAACTCCGCCTCCATCTTTGCAATCTTATCAATC
TP4731_Hit	D+G	1	chr7	.	CAGCAACTGCAACTGGTGGCCGGCTTTTTCTAGCAGGGGAAAGCCACAAATCAGGAGGACAAAA
TP4731_Query	D+G	1	chr7	.	CAGCAACTGCAACTGGTGGCCGGCTTTTTCTAGCAGGAGAAAGCCACAAATCAGGAGGACAAAA
TP47665_Hit	D	1	chr7	.	CAGCTCCGTTTCTTACCGCGACTGCTTGGTGTTTGCTTGGTCCAGCCGTGTTGCGAAATGTTAT
TP47665_Query	D	1	chr7	.	CAGCTCCGTTTCTTACCGCGACGGCTTGGTGTTTGCTTGGTCCAGCCGTGTTGCGAAATGTTAT
TP47718_Hit	D	1	chr7	.	CAGCTCCTATGAAAGTTTTCACAATAAAGCAAAACCATTTATGTTCAAGTAAAGGTTACTGAAG
TP47718_Query	D	1	chr7	.	CAGCTCCTATGAAAGTTTTCACAATAAAGCAAAACCATTTATGTTCAAGTAAAGGTTACGGAAG
TP4789_Hit	D	1	chr7	.	CAGCAACTGTGAAAATTGAAGCTGGTGGAAGTTCAAACCTTTCACCTATCTTTCTGTTCATTAT
TP4789_Query	D	1	chr7	.	CAGCAACTGTGAAAATTGAAGCTGGTGGAAGTTCAAACCTTTCACCTATCTTTCTGTTCATTAC
TP47905_Hit	D	1	chr7	.	CAGCTCCTGTTAGGCCAAATTCACCATCAGTGACACTTAGCCTATCTCCCTCGACAGTAGCTGA
TP47905_Query	D	1	chr7	.	CAGCTCCTGTTAGGCCAAATTCACCATCAGTGACACTTAGCCTATCTCCCTCGACAGTAGCAGA
TP48014_Hit	D	1	chr7	.	CAGCTCGAAAATATTTTAAGAAGTCAGCTAAGTCCTCTCCAGACTTGAATTGTGACATTGATGA
TP48014_Query	D	1	chr7	.	CAGCTCGAAAATATTTTAAGAAGTCAGCTAAGTCCTCTCCAGACTTAAATTGTGACATTGATGA
TP4808_Hit	D	1	chr7	.	CAGCAACTGTTATTACCTATTAGTGATAAAGGGTGAGTTATGTTAATTATTCTTGCTATCTACT
TP4808_Query	D	1	chr7	.	CAGCAACTGTTATTACCTATTAGTGATAAAGGGCGAGTTATGTTAATTATTCTTGCTATCTACT
TP48099_Hit	D	1	chr7	.	CAGCTTGAGAACGAATTAGCAACTCTTCAATCTGAGCTAGATAGGGTATCCTACCTGTTGAAGA
TP48099_Query	D	1	chr7	.	CAGCTCGAGAACGAATTAGCAACTCTTCAATCTGAGCTAGATAGGGTATCCTACCTGTTGAAGA
TP48122_Hit	D	1	chr7	.	CAGCTCGAGGACCAAAATGAACAAGAAATTCTCTGCACAGACCAGCATCCCTAATAGAACTATA
TP48122_Query	D	1	chr7	.	CAGCTCGAGGACCAAAATGAACAAGAAAGTCTCTGCACAGACCAGCATCCCTAATAGAACTATA
TP48672_Hit	D	1	chr7	.	CAGCTCTAGCAACAAAACCACCCATAGAGTGACCAACCAATATCACACTTTTGGGCAAGCTACC
TP48672_Query	D	1	chr7	.	CAGCTCTAGCAACAAAACCACCCATAGAGTGACCAACCAATATCACACTCTTGGGCAAGCTACC
TP48751_Hit	D	1	chr7	.	CAGCTCTATGTGCTATTAAAACAGTGGTCTTGTTTCCCATTACTAGTGTATCTAGAGCTTCTTG
TP48751_Query	D	1	chr7	.	CAGCTCTATGCGCTATTAAAACAGTGGTCTTGTTTCCCATTACTAGTGTATCTAGAGCTTCTTG
TP48834_Hit	D+G	1	chr7	.	CAGCTCTCACTACTTTGTTTGGGAAGCGTTTGCAAACAGGTCATTGGAATGTTACTGATGTTTG
TP48834_Query	D+G	1	chr7	.	CAGCTCTCACTACTTTGTTTGGGAAACGTTTGCAAACAGGTCATTGGAATGTTACTGATGTTTG
TP48897_Hit	D	1	chr7	.	CAGCTCTCCCACCCATGTAAGCAAGATATCGCATGAGTAACTTCGTAGTTTCTGTTTTACCTGC
TP48897_Query	D	1	chr7	.	CAGCTCTCCCACCCATGTAAGCAAGATATCGCATGAGTAACTTAGTAGTTTCTGTTTTACCTGC
TP49066_Hit	D+G	1	chr7	.	CAGCTCTGAGGTCACTATTGATATTGTGCGATCATTTCTGGTTAGTATCCCATCTAGATGACAG
TP49066_Query	D+G	1	chr7	.	CAGCTCTGAGGTCACTATTGATATTGTGCGATCATTTCTGGTTAGTATCCCATCTAAATGACAG
TP49086_Hit	D	1	chr7	.	CAGCTCTGCAAAATGATGAAAGGGCAAACTATCAACTTGATGTCAAGTTTCAGGAATCTTATCT
TP49086_Query	D	1	chr7	.	CAGCTCTGCAAAATGATGAAAGGGCAAACTATCAACTTGATGCCAAGTTTCAGGAATCTTATCT
TP49269_Hit	D+G	1	chr7	.	CAGCTCTTAACAAATGATATTGAGCTTAGAGATGGATCTTCATAGCCAGTTATGTACCTGGTTT
TP49269_Query	D+G	1	chr7	.	CAGCTCTTAACAAATGATATTGAGCTTAGAGATGGATCTTCATAGCCAGTTAAGTACCTGGTTT
TP4934_Hit	D	1	chr7	.	CAGCAACTTTACCAGAAAGCACTTACTATTAAGTCAGCAATTCCCCATCCAAGGATAATGGGGA
TP4934_Query	D	1	chr7	.	CAGCAACTTTACCAGAAAGCACTTACTATCAAGTCAGCAATTCCCCATCCAAGGATAATGGGGA
TP49376_Hit	D+G	1	chr7	.	CAGCTCTTCTCCAAGGACGAGCTATTGTCCCCATGCAATTAATTGCACGTATTCCTGCTGAAAA
TP49376_Query	D+G	1	chr7	.	CAGCTCTTCTCCAAGGACGAGCTATTGTCCCCATGCAATTAATTGCACGCATTCCTGCTGAAAA
TP49414_Hit	D	1	chr7	.	CAGCTCTTGAATATTCCTTCCATGGTCTCGCCAAGTAAGACTTTACTGTCAACCTATGTGGTAA
TP49414_Query	D	1	chr7	.	CAGCTCTTGAATATTCCTTCCATGGTCTCCCCAAGTAAGACTTTACTGTCAACCTATGTGGTAA
TP49480_Hit	D+G	1	chr7	.	CAGCTCTTGGGTTTGCCCAGTATGTTCGAAATGAGAACCATCCCATGACAAAGGTGCTGAAAAA
TP49480_Query	D+G	1	chr7	.	CAGCTCTTGGGTTTGCCCAGTATGTTCCAAATGAGAACCATCCCATGACAAAGGTGCTGAAAAA
TP49969_Hit	D	1	chr7	.	CAGCTGAAGAACAAGCACTCAAAGTCTCCTATGAAGACAACTCGGGCGGCAGAGGTAGGGGACG
TP49969_Query	D	1	chr7	.	CAGCTGAAGAACAAGCACTCAAAGTCTCCTATGAAGACAACTCAGGCGGCAGAGGTAGGGGACG
TP50143_Hit	D	1	chr7	.	CAGCTGAAGTTCAGTGTCCTACAGTTGCTGTCATTCCTCATTAAGGTATACCGGTGTACAAGGT
TP50143_Query	D	1	chr7	.	CAGCTGAAGTTCAGTGTCCTACAGTTGCTGTCATTCCTCATTAAGGTATACCGGTGTACAAGGA
TP50232_Hit	D+G	1	chr7	.	CAGCTGAATTATCAGTTCCTGCTCCCACCATTGAAGCTTCATTGGATGCTAGGTTCCTTAGTGG
TP50232_Query	D+G	1	chr7	.	CAGCTGAATTATCAGTTCCTGCTCCCACCATTGAAGCTTCATTGGATGCTAGGTTCCTCAGTGG
TP503_Hit	D	1	chr7	.	CAGCAAAACTGAAATGATATAAAGTTTGAGACAAATATATGCGCCAACGACACATGAAGGATAT
TP503_Query	D	1	chr7	.	CAGCAAAACTGAAATGATATAAAGTTTGAGACAAATATACGCGCCAACGACACATGAAGGATAT

Name	Filter	Nb hit (Mt4.0)	Mt Chr (Mt4.0)	Ms Chr (Li et al., 2014)	Sequence
TP50316_Hit	D	1	chr7	.	CAGCTGACATAAGAAGGCTTCTTCTCGTTAGTGGACAAATCAAGGTGACAATTTCAATAATACT
TP50316_Query	D	1	chr7	.	CAGCTGACATAAGAAGGCTTCTTCTCGTTAGTGGACAAATCAAGGTGACAATTGCAATAATACT
TP50329_Hit	D	1	chr7	.	CAGCTGACATTTCAGAAGAGACACAACTTTACTCAGATAAGATTCCTGAAACTTGGCATCAAGT
TP50329_Query	D	1	chr7	.	CAGCTGACATTTCAGAAGAGACACAACTTTACTCAGATAAGATTCCTGAAACTTGACATCAAGT
TP50416_Hit	D	1	chr7	.	CAGCTGACTTGGACTCTTCTGTTATTGAGGAGAGAATGAAAAAAAAGAAAAAAAATCTAAACTT
TP50416_Query	D	1	chr7	.	CAGCTGACTTGGACTCTTCTGTTATTGAGGAGAGAAAGAAAAAAAAGAAAAAAAATCTAAACTT
TP50422_Hit	D	1	chr7	.	CAGCTGACTTTGAGAGAGATATAATTGTGGAGACTCAATCAGGTTCGTTGAAACGTGTCTCATT
TP50422_Query	D	1	chr7	.	CAGCTGACTTTGAGAGAGATATAATTGTGGAGACTCAATCAGGTTCGTTGAAACGTGTCTCAGT
TP50434_Hit	D+G	1	chr7	.	CAGCTGAGAAGGACCGGTTGAAAAGGGAATTGGAAATCGCAGAAAGCAAGAACGGTGCTGAAAA
TP50434_Query	D+G	1	chr7	.	CAGCTGAGAAGAACCGGTTGAAAAGGGAATTGGAAATCGCAGAAAGCAAGAACGGTGCTGAAAA
TP50508_Hit	D	1	chr7	.	CAGCTGAGCGTTATTTATTTCAGAATAGGGCTCCACCTGTGCCTGTGATGCCTCGCAGTTTAGA
TP50508_Query	D	1	chr7	.	CAGCTGAGCGTTATTTATTTCAGAATAGGGCTCCACCTGTGCCTGTGATACCTCGCAGTTTAGA
TP50530_Hit	D	1	chr7	.	CAGCTGAGGAAGGCTACCGATTTGAAGAAGGTTTGATTCATCTTCTTAGTATGAATTTTTTATT
TP50530_Query	D	1	chr7	.	CAGCTGAGGAAGGCTACCGATTTGAAGAAGGTTTGATTCATCTTCTTAATATGAATTTTTTATT
TP50563_Hit	D	1	chr7	.	CAGCTGAGGCATGGATTGAAGCGCTCAAGGCGAGTGAGAAGGAAATACTACTGGAAACAAAAAT
TP50563_Query	D	1	chr7	.	CAGCTGAGGCATGGATTGAAGCGCTCAAGGCGAGCGAGAAGGAAATACTACTGGAAACAAAAAT
TP50733_Hit	D	1	chr7	.	CAGCTGATATGGCCTCAATTGAGGTTGTGTTGCGATTGTGGAAGCATAAAAAACTTTGATGATA
TP50733_Query	D	1	chr7	.	CAGCTGATATGGCCTCAATTGAGGTTGTGTTGTGATTGTGGAAGCATAAAAAACTTTGATGATA
TP50796_Hit	D	1	chr7	.	CAGCTGATCTATTTGTATTGGAAAAAGATAAAGAATACGAAAACTACCAAGTGCGAGGATAGCT
TP50796_Query	D	1	chr7	.	CAGCTGATCTATTTATATTGGAAAAAGATAAAGAATACGAAAACTACCAAGTGCGAGGATAGCT
TP50850_Hit	D	1	chr7	.	CAGCTGATGAGTAGTATAGTTGTAAAGAAATAGAGGTAAGCCCGGTGTAATTGCCCTGACAGAA
TP50850_Query	D	1	chr7	.	CAGCTGATGAGTAGTATAGTTGTAAAGAAATAGAGGTAAGCCCGGTGTAATTGCCCTAACAGAA
TP50873_Hit	D	1	chr7	.	CAGCTGATGCATTGTATACTTCTTTATTATTTAGGTTGACTTAAACATCAATCCTCACCTCTCT
TP50873_Query	D	1	chr7	.	CAGCTGATGCATTGTATACTTATTTATTATTTAGGTTGACTTAAACATCAATCCTCACCTCTCT
TP50970_Hit	D	1	chr7	.	CAGCTGATTACTAATGTATTTTCTTTGTGGCTTCTGGACTCTACAGCATCAATTCGTTGCAGGT
TP50970_Query	D	1	chr7	.	CAGCTGATTACTAATGTATTTTCTTTGTGGCTTCTGGACTCTACAGCATCAATTCGTTGCAGGG
TP51111_Hit	D+G	1	chr7	.	CAGCTGGAAATATCTGTTGTGGTCTCTTTTAATCTTCCAGATGTTGGTATGCGTATCTACTAGT
TP51111_Query	D+G	1	chr7	.	CAGCTGGAAATATCTGTTGTGGTCTCATTTAATCTTCCAGATGTTGGTATGCGTATCTACTAGT
TP513_Hit	D	1	chr7	.	CAGCAAAACTGGCTTATGCCCATGACTTTATATCTTCACTTCGTAATGGCTATGAAACCCTTGT
TP513_Query	D	1	chr7	.	CAGCAAAACTGGCTTATGCCCATGACTTTATATCTTCACTTCCTAATGGCTATGAAACCCTTGT
TP51584_Hit	D	1	chr7	.	CAGCTGGGAGGATAATAACATTGCCTGAGGTCAATACCATTGCAGACGGGCTCCGAGCATGTCT
TP51584_Query	D	1	chr7	.	CAGCTGGGAGGATAATAACATTGCCTGAGGTCAATACCATAGCAGACGGGCTCCGAGCATGTCT
TP51636_Hit	D	1	chr7	.	CAGCTGGTGACTATGATTGTCCAAGGAATGTTTTAAAGTTTTTAACAGAACTTCATGCCGCATT
TP51636_Query	D	1	chr7	.	CAGCTGGGGACTATGATTGTCCAAGGAATGTTTTAAAGTTTTTAACAGAACTTCATGCCGCATT
TP5186_Hit	D	1	chr7	.	CAGCAAGAATTTCCAATGGTGTAATTGTCGATGCAGTTGCTACGGGTTCTGCATCGTCATATGC
TP5186_Query	D	1	chr7	.	CAGCAAGAATTTCCAATGGTGTAATTGTCGATGCAGTTGCTACGAGTTCTGCATCGTCATATGC
TP51884_Hit	D	1	chr7	.	CTGCTGGTGCTCAGTTTATTCAATTTGATGAACCCACACTTGTCCTGGATCTTGAAGCTCACAA
TP51884_Query	D	1	chr7	.	CAGCTGGTGCTCAGTTTATTCAATTTGATGAACCCACACTTGTCCTGGATCTTGAAGCTCACAA
TP52071_Hit	D+G	1	chr7	.	CAGCTGTAAGGGTTTAAAAACTTTCGTAGGGGGGAGATCAGTTCCTGTTCAATTTTTTGGCACC
TP52071_Query	D+G	1	chr7	.	CAGCTGTAAGGGTTTAAAAACTTTCGTAGGGGGGAGATCAGTTCCTGTCCAATTTTTTGGCACC
TP52248_Hit	D	1	chr7	.	CAGCTGTATGCTCCCGACCGCATGCAATATCCAACCAGCGTGTATTGAGACCAGCCGGACATCC
TP52248_Query	D	1	chr7	.	CAGCTGTATGCTCCCGACCACATGCAATATCCAACCAGCGTGTATTGAGACCAGCCGGACATCC
TP52264_Hit	D	1	chr7	.	CAGCTGTGAAAAAAAATTAAAGGCATCTTACCATTCTTCAATTCGAAAACCTTAACATGAAGTC
TP52264_Query	D	1	chr7	.	CAGCTGTCAAAAAAAATTAAAGGCATCTTACCATTCTTCAATTCGAAAACCTTAACATGAAGTC
TP5259_Hit	D	1	chr7	.	CAGCAAGAGAACTGTCGAACGAATGCATCAGAACTTCCGTCTGACCACACTCGTATTTGGTCAG
TP5259_Query	D	1	chr7	.	CAGCAAGAGAACTGCCGAACGAATGCATCAGAACTTCCGTCTGACCACACTCGTATTTGGTCAG
TP52642_Hit	D+G	1	chr7	.	CAGCTGTGGTGGCTGAAGAACAACATGATAACAGCACTTATTTGAAAGATTATGCTGATTATAG
TP52642_Query	D+G	1	chr7	.	CAGCTGTGGTGGCTGAAGAACAACATGATAACAGCACTTACTTGAAAGATTATGCTGATTATAG
TP52684_Hit	D	1	chr7	.	CAGCTGTGTGTAAAGCAGTTGAATTGGACAAATCTGTAGTCATGGCCAAGTTGGGAAAAGGGTG
TP52684_Query	D	1	chr7	.	CAGCTGTGTGTAAAGCAGTTGAATTGGACAAATCTGTAGTCATGGCCAAGTTGGGAAAAGAGTG
TP52858_Hit	D+G	1	chr7	.	CAGCTGTTCATGCACTCACAGCGTTTATAAAATGCTTTATTTCTTCTGATGTTAAGAATGACGG
TP52858_Query	D+G	1	chr7	.	CAGCTGTTCATGCACTCACAGCGTTTATAAAATGCTTTATATCTTCTGATGTTAAGAATGACGG
TP52940_Hit	D	1	chr7	.	CAGCTGTTGCGACTCTTGTTGCTACAATCATTTCCATCGTAATCACTATTCCCGTGGCTCGGAT
TP52940_Query	D	1	chr7	.	CAGCTGTTGCAACTCTTGTTGCTACAATCATTTCCATCGTAATCACTATTCCCGTGGCTCGGAT

Name	Filter	Nb hit (Mt4.0)	Mt Chr (Mt4.0)	Ms Chr (Li et al., 2014)	Sequence
TP52984_Hit	D	1	chr7	.	CAGCTGTTGGACGAAACCTTGGGGATTGAGTATAGAACAAGCCTTGTATTTGTAATTTTGGAGA
TP52984_Query	D	1	chr7	.	CAGCTGTTGGACGAAACCTTGGGGACTGAGTATAGAACAAGCCTTGTATTTGTAATTTTGGAGA
TP53089_Hit	D+G	1	chr7	.	CAGCTGTTTATACACTATTCTTGCATTTGTTCGGCGCAAACCATGTTATTTCGAGCAGGTGCTG
TP53089_Query	D+G	1	chr7	.	CAGCTGTTTATACACTATTCTTACATTTGTTCGGCGCAAACCATGTTATTTCGAGCAGGTGCTG
TP53190_Hit	D+G	1	chr7	.	CAGCTTAAAAAGAAATATTACGAGATTTTGCTCTTCTAAAATGGGAGAAAAGAGAGTGCAAATT
TP53190_Query	D+G	1	chr7	.	CAGCTTAAAAAGAAATATTACGAGATTTTGCTCTTCAAAAATGGGAGAAAAGAGAGTGCAAATT
TP53491_Hit	D	1	chr7	.	CAGCTTAATTCTCATATTCTTCAGGTAACCATGATCAACGTTTGGATTTCAACAATCTATACAC
TP53491_Query	D	1	chr7	.	CAGCTTAATTCTCATATTCTTCAGGTAACCATGACCAACGTTTGGATTTCAACAATCTATACAC
TP53683_Hit	D+G	1	chr7	.	CAGCTTACTATAGAATTGATGATGAACATCACTTAGAACAGGAGGTAGACAAGTGGTGCTTGAT
TP53683_Query	D+G	1	chr7	.	CAGCTTACTATAGAATTGACGATGAACATCACTTAGAACAGGAGGTAGACAAGTGGTGCTTGAT
TP54018_Hit	D	1	chr7	.	CAGCTTATATGGTGGCGTCAACCGTGACTTCTGTGGTTGCATCTGGAGAGAGGGAAAGGCAGGG
TP54018_Query	D	1	chr7	.	CAGCTTATATGGTGGCGTCAACCGTGACTTCTGTGGTTGCATCTGGAGAGAGGGAAAGGCAGGA
TP54075_Hit	D	1	chr7	.	CAGCTTATCGAAGGATGCTAAAATATGTAACTACTAGCTTTGGTTCTTAATTCTAGACATCAAT
TP54075_Query	D	1	chr7	.	CAGCTTATCGAAGGAAGCTAAAATATGTAACTACTAGCTTTGGTTCTTAATTCTAGACATCAAT
TP54076_Hit	D	1	chr7	.	CAGCTTATTGAAGGAAGCTAAAATATGTAACTATTAGCTTCCATTCTTAATTCTAGACATCAAT
TP54076_Query	D	1	chr7	.	CAGCTTATCGAAGGAAGCTAAAATATGTAACTATTAGCTTCCATTCTTAATTCTAGACATCAAT
TP54147_Hit	D+G	1	chr7	.	CAGCTTATGATTTTGGTTGATGATGCTGGAGGAGAATGGCCAGCGATTGGTCATGCACCATGGA
TP54147_Query	D+G	1	chr7	.	CAGCTTATGATCTTGGTTGATGATGCTGGAGGAGAATGGCCAGCGATTGGTCATGCACCATGGA
TP54397_Hit	D	1	chr7	.	CAGCTTCAAACCCTCTTCATCATGCACGACCATACTCTAGGCTATATGGTCTGATCTCCATCTT
TP54397_Query	D	1	chr7	.	CAGCTTCAAACACTCTTCATCATGCACGACCATACTCTAGGCTATATGGTCTGATCTCCATCTT
TP54529_Hit	D	1	chr7	.	CAGCTTCAAGAGATGTTCCATTAGAACCATATGGAATAACTGAACGTTATTCCAGAGACTATTC
TP54529_Query	D	1	chr7	.	CAGCTTCAAGAGATGTTCCATTAGAACCATATGGAATAACAGAACGTTATTCCAGAGACTATTC
TP54547_Hit	D+G	1	chr7	.	CAGCTTCAAGGGCTAGCTCTTGATGAGCTGGTTTACACACATCTTTAGGGCCTAATCTTCCATA
TP54547_Query	D+G	1	chr7	.	CAGCTTCAAGGGCCAGCTCTTGATGAGCTGGTTTACACACATCTTTAGGGCCTAATCTTCCATA
TP54590_Hit	D	1	chr7	.	CAGCTTCAATCATATTGAAGCTAATCATTGTGTTGTTATACATAATGACAGAATGATTGGATTG
TP54590_Query	D	1	chr7	.	CAGCTTCAATCATATTAAAGCTAATCATTGTGTTGTTATACATAATGACAGAATGATTGGATTG
TP54607_Hit	D	1	chr7	.	CAGCTTCAATGAGATTTTTGTTTTCCCGTATGATATATTCTTGTGTGTTTGAAGATCGACATAC
TP54607_Query	D	1	chr7	.	CAGCTTCAATGAGATTTTTGTTTTCCCGTATGATATATTCTTATGTGTTTGAAGATCGACATAC
TP5461_Hit	D	1	chr7	.	CAGCAAGATCCCTTGAATCTTCAGCTACATACTCTCCTATAATCGAAGAATACGCTTCTTTTAC
TP5461_Query	D	1	chr7	.	CAGCAAGATCCCTTGAATCTTCAGCTACATACTCTCCTATAATCGAAGAATACACTTCTTTTAC
TP54734_Hit	D	1	chr7	.	CAGCTTCACGAGAGAGTAAAATGGAGGACATAGAATCATTGAAACTGTAAGTAGAATCAGTATC
TP54734_Query	D	1	chr7	.	CAGCTTCACGAGAGAGTAAAATGGAGGACATAGAATCATTGAAACGGTAAGTAGAATCAGTATC
TP55002_Hit	D	1	chr7	.	CAGCTTCATATGAAAGTTCCCGTTCTTTACTCAAGGCTAGTTCTCTCTTGACATCAGAGTTTGA
TP55002_Query	D	1	chr7	.	CAGCTTCATATGAAAGTTCCCGGTCTTTACTCAAGGCTAGTTCTCTCTTGACATCAGAGTTTGA
TP55080_Hit	D+G	1	chr7	.	CAGCTTCATGCTGGGTCCGGGCAACACTCACAGGCATCTAAAAAGCGACGTGGAGGTTATCTCT
TP55080_Query	D+G	1	chr7	.	CAGCTTCATGCTGGGTCCGGACAACACTCACAGGCATCTAAAAAGCGACGTGGAGGTTATCTCT
TP55228_Hit	D	1	chr7	.	CAGCTTCCACTTCTGCGATATTGAGGATCTGATATGAGAAGTCAAAGAGCTCAAAATGTAGCTG
TP55228_Query	D	1	chr7	.	CAGCTTCCACTTCTGCGATATTGAGGATCTGATACGAGAAGTCAAAGAGCTCAAAATGTAGCTG
TP55437_Hit	D	1	chr7	.	CAGCTTCCTCTTTCTCCTGTGCAGTGTTGTAGCCCCAATTCCCTGATCCAAACAGTCATCATTA
TP55437_Query	D	1	chr7	.	CAGCTTCCTCTTTCTCCTGTGCAGTGTTGTAACCCCAATTCCCTGATCCAAACAGTCATCATTA
TP55501_Hit	D	1	chr7	.	CAGCTTCCTTTTTCTATGTATTTTACATCACAGGCTCCTTGGGAGCTCAAATCTGCCTTGAAAG
TP55501_Query	D	1	chr7	.	CAGCTTCCTTTTTCTATGTATTTTACATCACAGGCTCCTTCGGAGCTCAAATCTGCCTTGAAAG
TP55617_Hit	D	1	chr7	.	CAGCTTCGGCTCATTTCATGAAACTATTGCACATCTTTACAAAATGAACTGCTAATACACGTAA
TP55617_Query	D	1	chr7	.	CAGCTTCGGCTCATTTCATGAAACTATTGCACATCTTTACAAAATGAACTGCTAATACACATAA
TP55636_Hit	D	1	chr7	.	CAGCTTCGGTTATGGAAAGGTTTGAGATTGTTGCTTTGGAGAAAGATACTTGTCCTGAACATGT
TP55636_Query	D	1	chr7	.	CAGCTTCGGTTATCGAAAGGTTTGAGATTGTTGCTTTGGAGAAAGATACTTGTCCTGAACATGT
TP55855_Hit	D	1	chr7	.	CAGCTTCTCATTTTTCCTTAAATCTTCACAAGGTGTTTGTTTCATTGATGTTGGCAGACCTGAT
TP55855_Query	D	1	chr7	.	CAGCTTCTCATCTTTCCTTAAATCTTCACAAGGTGTTTGTTTCATTGATGTTGGCAGACCTGAT
TP55875_Hit	D+G	1	chr7	.	CAGCTTCTCCACCCTTTACCCCACTTATACTTTCAGCCTTTTCCCGCTCCTCCAAAAGACGTTG
TP55875_Query	D+G	1	chr7	.	CAGCTTCTCCACCCTTTACCCCACTTATACTATCAGCCTTTTCCCGCTCCTCCAAAAGACGTTG
TP56001_Hit	D	1	chr7	.	CAGCTTCTGACAGCTCTTCGGACTCATCACCTTCACGCCTTTCATTGGGGTTTTGAACAGCTTC
TP56001_Query	D	1	chr7	.	CAGCTTCTGACAGCTCTTCGGACTCATCACCTTCACGCCTTTCACTGGGGTTTTGAACAGCTTC
TP56014_Hit	D	1	chr7	.	CAGCTTCTGAGTTCTACAAGGAGGACAAATCATACGATTTGAACTTCAAGGAAGATGTGAGCTC
TP56014_Query	D	1	chr7	.	CAGCTTCTGAGCTCTACAAGGAGGACAAATCATACGATTTGAACTTCAAGGAAGATGTGAGCTC

Name	Filter	Nb hit (Mt4.0)	Mt Chr (Mt4.0)	Ms Chr (Li et al., 2014)	Sequence
TP56395_Hit	D	1	chr7	.	CAGCTTCTGCTTGTTTTGCCATGATGTGGAAAGAAGCAGGTGATGGGCAACCAGGATATGAAAC
TP56395_Query	D	1	chr7	.	CAGCTTCTGCTTGTTTTGACATGATGTGGAAAGAAGCAGGTGATGGGCAACCAGGATATGAAAC
TP56420_Hit	D	1	chr7	.	CAGCTTCTGGCAGTTAGGTGATGAGCTTCGCGGGCACTCAAAAGCCTCTGAGGATCACAAGTGG
TP56420_Query	D	1	chr7	.	CAGCTTCTGGCAGTTAGGTGATGAGCTTCGCGGACACTCAAAAGCCTCTGAGGATCACAAGTGG
TP56532_Hit	D+G	1	chr7	.	CAGCTTCTTCAGCAGTGTCGAATGTTCCTAACCAGTGTCTTTCTTTTGTTGAAGGATCACGAAT
TP56532_Query	D+G	1	chr7	.	CAGCTTCTTCAGCAGTGTCGAATGTTCCTAACCAGTGTCGTTCTTTTGTTGAAGGATCACGAAT
TP56608_Hit	D	1	chr7	.	CAGCTTCTTGACAGACTGGGTTTTCCTCATAATTCAGAGAACTTTCGTTTGCATCGACAGGTGC
TP56608_Query	D	1	chr7	.	CAGCTTCTTGACAGACTGGGTTTTCCTCATAATTCAGAGAACTTTCGTTTGCATCGACAAGTGC
TP5662_Hit	D	1	chr7	.	CAGCAAGCATATTCTTGGGATAATAATCTACAGATACCGTCAATGCTGACTGCTGTGGAATCCA
TP5662_Query	D	1	chr7	.	CAGCAAGCATATTCTTGGGATAATAATCTACAGATACCGTCAATGCTAACTGCTGTGGAATCCA
TP56639_Hit	D	1	chr7	.	CAGCTTCTTGTAAGTCATTTGGAGCCTTTAACTGAACTACAGTTGATGGGTATCTACAACTTGC
TP56639_Query	D	1	chr7	.	CAGCTTCTTGTAAGTCATTTGGAGCCTTTAACTGAACAACAGTTGATGGGTATCTACAACTTGC
TP5682_Hit	D	1	chr7	.	CAGCAAGCATGCTTTCAAATTCCTTGGAAGTCCTTTCACCCACCAGTACCACTTGCTTACGGGA
TP5682_Query	D	1	chr7	.	CAGCAAGCATGCTTTCAAATTCCTCGGAAGTCCTTTCACCCACCAGTACCACTTGCTTACGGGA
TP57039_Hit	D	1	chr7	.	CAGCTTGAGAATCCTCATGTTCCTTAGGCACTCGGATGTTTTTCTCATCCTTTCATCCTTAGCT
TP57039_Query	D	1	chr7	.	CAGCTTGAGAATCCTCATGTTCCTTAGGCACTCGGATGTTTTTCTCATCCTTTCATCCTTAGCA
TP57101_Hit	D	1	chr7	.	CAGCTTGAGGATATGGGGTTCGACCGAGATCTTGTGTTGGAGGTGTTCTTTGCTTGCAATAAAA
TP57101_Query	D	1	chr7	.	CAGCTTGAGGATATGGGGTTCGACCGAGATCTTGTGTTGGAGGTGTTCTTTGCGTGCAATAAAA
TP57134_Hit	D+G	1	chr7	.	CAGCTTGAGTCTTAACAACTGCTGTCTGAACAGAACGAACAGGTTCCTCGACATCCACCACAGT
TP57134_Query	D+G	1	chr7	.	CAGCTTGAGTCTTAACAACTGCTGTCTGAACAGAACGAACAGGTTCCTCGACATCCACCACAGC
TP57225_Hit	D	1	chr7	.	CAGCTTGATGTATCTCTTCCTCACACTCAATAGAGGATGATTCTCGATAGCCGATTCATTGCAA
TP57225_Query	D	1	chr7	.	CAGCTTGATGTATCTCTTCCTCACACCCAATAGAGGATGATTCTCGATAGCCGATTCATTGCAA
TP57315_Hit	D+G	1	chr7	.	CAGCTTGCAAGAAAGACGATATAGCAGTGGATCGGATCGGATCCTTACCAAAGCATTGAGATTG
TP57315_Query	D+G	1	chr7	.	CAGCTTGCAAGAAAGACGATATAGCAGTGGATCAGATCGGATCCTTACCAAAGCATTGAGATTG
TP57435_Hit	D	1	chr7	.	CAGCTTGCCCTGAGTGATTCTCTCTAAACATCTCAAGTGTATTTGCATGGCTTTTCACGACATT
TP57435_Query	D	1	chr7	.	CAGCTTGCCCTGAGTGATTCTCTCTAAACATCTCAAGTGTATTTGCATGGCTTTTCACAACATT
TP57528_Hit	D	1	chr7	.	CAGCTTGCTATATACTTGTAAGAGTCACCTGCCTTTCTCAATAGATTTCTTTATCTGTTGAAAG
TP57528_Query	D	1	chr7	.	CAGCTTGCTATATACTTGTAAGAGTCACCTGCCTTTCTCAATAGATTTCTTTATCTGTCGAAAG
TP57540_Hit	D	1	chr7	.	CAGCTTGCTCAGATTGACTTGTGATATATGTATCATGTGTCTATTATGTAAATATTTTACCCTT
TP57540_Query	D	1	chr7	.	CAGCTTGCTCAGATTGACTTGTGATATATGTATCATGTATCTATTATGTAAATATTTTACCCTT
TP57566_Hit	D+G	1	chr7	.	CAGCTTGGTGTAGCACGCAATTGCAGAATTGAATCGCGGGAGCTATGCTCGTCTGCGACATAAT
TP57566_Query	D+G	1	chr7	.	CAGCTTGCTGTAGCACGCAATTGCAGAATTGAATCGCGGGAGCTATGCTCGTCTGCGACATAAT
TP57647_Hit	D+G	1	chr7	.	CAGCTTGGACAAACTGTATAATTTTGTTACTCTTATCAGCCTTCCCCATACATTTGGTTTGCAT
TP57647_Query	D+G	1	chr7	.	CAGCTTGGACAAACTGTATAATTTTGTTACTCTTATCAGCCTTCCCCATACATTTGGTTTACAT
TP5767_Hit	D	1	chr7	.	CAGCAAGCGATCGAAATTCTTGACTGGTAAGATCTACCTTAACTGCGTCACGCTCAAAAGTACC
TP5767_Query	D	1	chr7	.	CAGCAAGCGATCGAAATTCTTGACTGGTAAGATCTACCTTAACTGCATCACGCTCAAAAGTACC
TP5785_Hit	D	1	chr7	.	CAGCAAGCGTGTCAAAATTGAAAGAGACAGAGTAATATAAGTATATGGTGATTAAATTACCTTA
TP5785_Query	D	1	chr7	.	CAGCAAGCGTATCAAAATTGAAAGAGACAGAGTAATATAAGTATATGGTGATTAAATTACCTTA
TP5788_Hit	D	1	chr7	.	CAGCAAGCTAAAACAAAAAGCAGTTCAACTTATCATATCATCTCAGTTGTTTTCTTTTGTAATG
TP5788_Query	D	1	chr7	.	CAGCAAGCTAAAAAAAAAAGCAGTTCAACTTATCATATCATCTCAGTTGTTTTCTTTTGTAATG
TP57973_Hit	D	1	chr7	.	CAGCTTGTACAAGGGTGTAATCATACTGAATGTTCAACTTCCGTATGAGCCCCTTGCTGAGGGC
TP57973_Query	D	1	chr7	.	CAGCTTGTACAAGGGCGTAATCATACTGAATGTTCAACTTCCGTATGAGCCCCTTGCTGAGGGC
TP58122_Hit	D	1	chr7	.	CAGCTTGTCGTTGTTTTGATAGATGGAAATCAAAGACTATTTAGCCACCTTTATTAGTTGCCTT
TP58122_Query	D	1	chr7	.	CAGCTTGTCGTTGTTTTGATAAATGGAAATCAAAGACTATTTAGCCACCTTTATTAGTTGCCTT
TP58183_Hit	D	1	chr7	.	CAGCTTGTGCATCACTCTTGTTCAAGAAAACAACACGACAAGTTGATGACTTCCAAAATATGCG
TP58183_Query	D	1	chr7	.	CAGCTTGTGCATCACTCTTGTACAAGAAAACAACACGACAAGTTGATGACTTCCAAAATATGCG
TP58184_Hit	D	1	chr7	.	CAGCTTGTGCATCATAAATGCTATTAAATGACCAAACTCTTAACCTTTTGCATATATTAGTATT
TP58184_Query	D	1	chr7	.	CAGCTTGTGCATCATAAATGCTATTAAATGACCAAACTCTTAACCCTTTGCATATATTAGTATT
TP58205_Hit	D+G	1	chr7	.	CAGCTTGTGGAAATCTCCACTCTTCACCAGATGAGACCGGTGCTTTGAACATCCGCATAAGTGC
TP58205_Query	D+G	1	chr7	.	CAGCTTGTGGAAATCTCCACTCTTCACCAGATGAGACAGGTGCTTTGAACATCCGCATAAGTGC
TP58263_Hit	D	1	chr7	.	CAGCTTGTGTTTCAGGAGCTCAGTTATCTAGCTATGCAAGCTTTATAAGCAAAGGGGATGTAGC
TP58263_Query	D	1	chr7	.	CAGCTTGTGTTTCAGGAGCTCAGTTATCTAGCTATGCAAGCTTTATAAGCAAAGGAGATGTAGC
TP58343_Hit	D+G	1	chr7	.	CAGCTTGTTCTTCCCTCGATAGCTAATAGCAATATTTAAATCATTCATGTCTTCACCATATAGC
TP58343_Query	D+G	1	chr7	.	CAGCTTGTTCTTCCCTCGATAACTAATAGCAATATTTAAATCATTCATGTCTTCACCATATAGC

Name	Filter	Nb hit (Mt4.0)	Mt Chr (Mt4.0)	Ms Chr (Li et al., 2014)	Sequence
TP58441_Hit	D+G	1	chr7	.	CAGCTTGTTTTTCCTTGCTGGATCAAAAACCCCCGATAACTGTGACCATTGATTTTGAGTGGAG
TP58441_Query	D+G	1	chr7	.	CAGCTTGTTTTTCCTTGCTGGATCAAAAACCCCCGATAACTGTGACCATCGATTTTGAGTGGAG
TP58490_Hit	D+G	1	chr7	.	CAGCTTTAACAACATCAGTTTTCCGAATGTCTTTTCCTTCTCCAGCTACAGATTTGTTTTCCCG
TP58490_Query	D+G	1	chr7	.	CAGCTTTAACAACATCAGTTTTCCGAATGCCTTTTCCTTCTCCAGCTACAGATTTGTTTTCCCG
TP58827_Hit	D	1	chr7	.	CAGCTTTATGTGAGTCTCTCTTACTCTTCTTCCTTTTGTATTATTGTGTTGCGTGTGAGGTGTT
TP58827_Query	D	1	chr7	.	CAGCTTTATGTGAGTCTCTCTTACTCTTCTTCCTTTTGTATCATTGTGTTGCGTGTGAGGTGTT
TP58855_Hit	D+G	1	chr7	.	CAGCTTTATTGGCAATATGGCCGAGTCTTCAATACCGATAGTTTTCAAGACTGTTGTGCGGGCC
TP58855_Query	D+G	1	chr7	.	CAGCTTTATTGGCAATATGGCCGAGTCTTCAATACCGACAGTTTTCAAGACTGTTGTGCGGGCC
TP59144_Hit	D	1	chr7	.	CAGCTTTCGTAAGCCTATCAATAACATACAAACTTGACAAAGGCGATGGGAGATTCCTCAATCT
TP59144_Query	D	1	chr7	.	CAGCTTTCGTAAGCCTATCAATAACATACAAACTTGACAAAGGCAATGGGAGATTCCTCAATCT
TP59233_Hit	D	1	chr7	.	CAGCTTTCTGTACAACCCTCGTCAACTCTCCTTTTTAAATGACAGTGAGAGCCTTAGTTAGAGG
TP59233_Query	D	1	chr7	.	CAGCTTTCTGTACAACCCTCGTCAACTCTCCTTTTTAAATGACAATGAGAGCCTTAGTTAGAGG
TP59354_Hit	D	1	chr7	.	CAGCTTTGACAAAATAGAAAAATCAAAATGCTTAAATAGGTATCTCTCTTTAGCAAAAAGATCT
TP59354_Query	D	1	chr7	.	CAGCTTTGACAAAATAGAAAAATCAAAATGCTTAAATAGCTATCTCTCTTTAGCAAAAAGATCT
TP59413_Hit	D	1	chr7	.	CAGCTTTGATCAGTATTGCATATGACGATGCAGAACTCGTAGCAACTGCATCGACAATTACACC
TP59413_Query	D	1	chr7	.	CAGCTTTGATCAGTATTGCATATGACGATGCAGAACCCGTAGCAACTGCATCGACAATTACACC
TP59481_Hit	D	1	chr7	.	CAGCTTTGCCACACTATGTTGATAAATTCTTTTAATCAAATTTACATTTGCAAACTTGTGAGTA
TP59481_Query	D	1	chr7	.	CAGCTTTGCCACACTATGTTGATAAATTCTTTTAATCAAATTTACATTTGCAAACTTGCGAGTA
TP59510_Hit	D	1	chr7	.	CAGCTTTGCTACTACTTTTTCCACTGATTATTTCAAGCATCAGAATCCCAAAACTATATACATC
TP59510_Query	D	1	chr7	.	CAGCTTTGCTACTACTTTTACCACTGATTATTTCAAGCATCAGAATCCCAAAACTATATACATC
TP5961_Hit	D	1	chr7	.	CAGCAAGGAATGCAATAATGGCCCAAGGATTACTGCAGTAAGTGTGAAAACCCAGTGCTATCCA
TP5961_Query	D	1	chr7	.	CAGCAAGGAATGCAATAATGGCCCAAGGATTACTGAAGTAAGTGTGAAAACCCAGTGCTATCCA
TP60032_Hit	D	1	chr7	.	CAGCTTTTCTTCAAGGCTACGAAAGCCTGAAAAAGACCATTCGGTTGAAGCTAAAACATCGTCG
TP60032_Query	D	1	chr7	.	CAGCTTTTCTTCAAGGCTACGAAAGCCTGAAAAAGACCATCCGGTTGAAGCTAAAACATCGTCG
TP60134_Hit	D	1	chr7	.	CAGCTTTTGCATAATCCACAGCCGTTGCGGTTTCACCTACGTGTTTTAAGACACGGAGACAAGA
TP60134_Query	D	1	chr7	.	CAGCTTTTGCATAATCCACAGCCGTTGCGGTTTCACCTACGCGTTTTAAGACACGGAGACAAGA
TP60178_Hit	D	1	chr7	.	CAGCTTTTGCTTTGCCCTTCCAAGCCAAGTACTTTCTATTTTCTTGCTTGTATCTTTTCCATTC
TP60178_Query	D	1	chr7	.	CAGCTTTTGCTTTGCCCTTCCAAGCCAAGTACTTCCTATTTTCTTGCTTGTATCTTTTCCATTC
TP60326_Hit	D	1	chr7	.	CAGCTTTTTCATAAGAGCGAGCTTGCTCCAGGAATGCATCGACATTTAGTTGATCATCCAAAGA
TP60326_Query	D	1	chr7	.	CAGCTTTTTCATAAGAGCGAGCTTGCTCCAGGAATGCATCGACATTTAGTTGATCAGCCAAAGA
TP60414_Hit	D+G	1	chr7	.	CAGCTTTTTTACAGTCTCCGGAATCTGCAGAGTGAAGAGAATGGTAATATTGCTGTCCTGGAGA
TP60414_Query	D+G	1	chr7	.	CAGCTTTTTTACAGTCTCCGGAATCTGCAGAGTGAAGAGAATGGCAATATTGCTGTCCTGGAGA
TP60437_Hit	D+G	1	chr7	.	CAGCTTTTTTGCAGAAATACAAGAAGCTACGCACCACTTACCACAAGAGAACTCTTGTACATCT
TP60437_Query	D+G	1	chr7	.	CAGCTTTTTTGCAGAAATACAAGAAGCTACGCACCACTTACCACAAGAGAACTCTTGTACATCG
TP60451_Hit	D	1	chr7	.	CAGCTTTTTTTATATGCACCATGTGTTTTAATGCTTCATTGAACCTTCGATGTGGGATTCATAT
TP60451_Query	D	1	chr7	.	CAGCTTTTTTTATATGCACCATGTGTTGTAATGCTTCATTGAACCTTCGATGTGGGATTCATAT
TP60538_Hit	D	1	chr7	.	CTGCAAAAACACTGACTGATATAGAAACACAAATCCATCCAAGAACTTGGACACGAATCGGACC
TP60538_Query	D	1	chr7	.	CTGCAAAAACACTGACTGATATAGAAACACAAATCCATCCAAGAACTTGGACACGAACCGGACC
TP60907_Hit	D	1	chr7	.	CTGCAAAATGCAAATGCTACTGAGAATTTAGATCAATGACAAATCATACATCTCAATATTGAAA
TP60907_Query	D	1	chr7	.	CTGCAAAATGCAAATGCCACTGAGAATTTAGATCAATGACAAATCATACATCTCAATATTGAAA
TP6122_Hit	D	1	chr7	.	CAGCAAGGGGTGAAAATTCATGCGAAACTAATAAAAAACTCACTCTACTTGGATGTCTTTGTAG
TP6122_Query	D	1	chr7	.	CAGCAAGGGATGAAAATTCATGCGAAACTAATAAAAAACTCACTCTACTTGGATGTCTTTGTAG
TP61265_Hit	D	1	chr7	.	CTGCAAAGAGGATCCTGATAGTTTCTACTGTACTTGTCTTCATGGAAGGCTTGCAGATGGAAGC
TP61265_Query	D	1	chr7	.	CTGCAAAGAGGATCCTGATAGTTTCTACTGTACTTGTCTTCACGGAAGGCTTGCAGATGGAAGC
TP61313_Hit	D+G	1	chr7	.	CTGCGAAGCCCAAGGCGAAGGCTCCTGTTGCTAAGGCCAATGCTGTACCTGTAGCTGAAAAAAA
TP61313_Query	D+G	1	chr7	.	CTGCAAAGCCCAAGGCGAAGGCTCCTGTTGCTAAGGCCAATGCTGTACCTGTAGCTGAAAAAAA
TP61452_Hit	D	1	chr7	.	CTGCAAATAAATTACTTGAACAAAGCAGTCATGAAAATGGAGTCGAAGCAAGGATGCTCCAATG
TP61452_Query	D	1	chr7	.	CTGCAAATAAATTACTTGAACAAAACAGTCATGAAAATGGAGTCGAAGCAAGGATGCTCCAATG
TP61744_Hit	D	1	chr7	.	CTGCAAATGTGGCCATGCCGGTTCCAAACACGGCTCCTCCTCTTCCGGATCGGCTGAAAAAAAA
TP61744_Query	D	1	chr7	.	CTGCAAATGTGGCCATGCCGGTTCCAAACACGGCTCCTCCTCTTCCGGATCGGCAGAAAAAAAA
TP61793_Hit	D	1	chr7	.	CTGCAAATTAGATAAATTTATTAATTAAAAGTAGCCCTGCACAAATTTGTGCCTCTCTATCTTA
TP61793_Query	D	1	chr7	.	CTGCAAATTAGATAAATTTATTAATTAAAAATAGCCCTGCACAAATTTGTGCCTCTCTATCTTA
TP61834_Hit	D+G	1	chr7	.	CTGCAAATTCTGGTAGCTTGCCCAAGAGTGTGATATTGGTTGGTCACTCTATGGGTGGTTTTGT
TP61834_Query	D+G	1	chr7	.	CTGCAAATTCTGGTAGCTTGCCCAAAAGTGTGATATTGGTTGGTCACTCTATGGGTGGTTTTGT

Name	Filter	Nb hit (Mt4.0)	Mt Chr (Mt4.0)	Ms Chr (Li et al., 2014)	Sequence
TP6187_Hit	D	1	chr7	.	CAGCAAGGTAGTTCTTCTTCTTAATTGCTTCTTTTGCAAACCCTTTCTCAATGTCACCAAATAG
TP6187_Query	D	1	chr7	.	CAGCAAGGTAGTTCTTCTTCTTAATTGCTTCCTTTGCAAACCCTTTCTCAATGTCACCAAATAG
TP61946_Hit	D	1	chr7	.	CTGCAACAAAGCCAACCCAGACCCAGTGAGTATTATAATTTATTCTTGCATTGTATTTTACTAC
TP61946_Query	D	1	chr7	.	CTGCAACAAAGCCAACCCAGACCCAGTGAGTATTATAATTTATTCTTGCAATGTATTTTACTAC
TP62089_Hit	D	1	chr7	.	CTGCAACACATCCTCCAACCAATACTGATTGAAGCAAGTTACTGACACGTTGTTCCTTTACCTC
TP62089_Query	D	1	chr7	.	CTGCAACACATCCTCCAACCAATACTGATTGAAGCAAGTTACTGACACGTTGTTCCTTAACCTC
TP6221_Hit	D+G	1	chr7	.	CAGCAAGGTGATAGGAGCAAAATACTTCAACCTCGACCCATCAGGTCCAACCATCGATAACCCA
TP6221_Query	D+G	1	chr7	.	CAGCAAGGTGATAGGAGCAAAATACTTCAACCTCGACCCATCAGGTCCAACCATCGAGAACCCA
TP62276_Hit	D	1	chr7	.	CTGCAACATCTGAAGAAATTCCAGCACTGCGAAAGACCGAAGTTGAATAATAAACCACAGCATT
TP62276_Query	D	1	chr7	.	CTGCAACATCTGAAGAAATTCCAGCACTACGAAAGACCGAAGTTGAATAATAAACCACAGCATT
TP623_Hit	D+G	1	chr7	.	CAGCAAAAGATGAAAATGCTAGTGTGTCACAAAATTGTTGTGCTCAGGTTAAGAAACTTGGTCA
TP623_Query	D+G	1	chr7	.	CAGCAAAAGATGAAAATGCTAGTGTGTCACAAAATTGTTGTGCTCAGGTTAAGAAACTCGGTCA
TP6242_Hit	D	1	chr7	.	CAGCAAGGTTACCTGGACGAACAGATAACGAGATTAAAAATGTATGGCACACCCATTTGAAGAA
TP6242_Query	D	1	chr7	.	CAGCAAGGTTACCTGGACGAACAGATAACGAGATAAAAAATGTATGGCACACCCATTTGAAGAA
TP62626_Hit	D	1	chr7	.	CTGCAACTACAAATTTATTTTGGAAAAAGCAAAACTATCAAAAACATCCAATGGTCTTTTTTGG
TP62626_Query	D	1	chr7	.	CTGCAACTACAAATTTATTTTGGAAAAAGCAAAACTATCAAAAACATCCAATGGTCTTCTTTGG
TP63096_Hit	D+G	1	chr7	.	CTGCAAGACTTTTCTCTGTTCCCTTTGAAGTCTCCAATTTGTCATCCTCAGGGTCAACAGGACT
TP63096_Query	D+G	1	chr7	.	CTGCAAGACTTTTCTCTGTTCCCTTTGAAGTATCCAATTTGTCATCCTCAGGGTCAACAGGACT
TP63113_Hit	D+G	1	chr7	.	CTGCAAGAGATAACAAGAAGAACAGAATTAACCCTAGACACGTGTGCCTTGCTGTGAGGAACGA
TP63113_Query	D+G	1	chr7	.	CTGCAAGAGATAACAAAAAGAACAGAATTAACCCTAGACACGTGTGCCTTGCTGTGAGGAACGA
TP63115_Hit	D	1	chr7	.	CTGCAAGAGATGCAACAGAGTTACTGGAGAAACTTTCATCCTCTGATCAGAATGTTATACAGAT
TP63115_Query	D	1	chr7	.	CTGCAAGAGATGCAACAGAGTTACTGGAGAAACTTTCATCCTCTGATCAGAACGTTATACAGAT
TP63218_Hit	D+G	1	chr7	.	CTGCAAGATTGTGTCAATCTGTTGTTTCTGTCATCAACATGAAGAAACATCCACTCATCTTTTC
TP63218_Query	D+G	1	chr7	.	CTGCAAGATTGTGTCAATCTGCTGTTTCTGTCATCAACATGAAGAAACATCCACTCATCTTTTC
TP63260_Hit	D+G	1	chr7	.	CTGCAAGCATGGAGTTAGAGCCGTGCTCGCGGAAGCTTAGGCTACGAAATTGGTGCTGAAAAAA
TP63260_Query	D+G	1	chr7	.	CTGCAAGCATAGAGTTAGAGCCGTGCTCGCGGAAGCTTAGGCTACGAAATTGGTGCTGAAAAAA
TP63261_Hit	D	1	chr7	.	CTGCAAGCATGGAGTTAGAGCCGTGCTCGCGGAAGCTTAGGCTGAAAAAAAAAAAAAAAAAAAA
TP63261_Query	D	1	chr7	.	CTGCAAGCATAGAGTTAGAGCCGTGCTCGCGGAAGCTTAGGCTGAAAAAAAAAAAAAAAAAAAA
TP63389_Hit	D	1	chr7	.	CTGCAAGGACCACCCCAAAAGGTCTTCACTGTCTTTCTATGCAACTAACTGCTGATTACTTTGC
TP63389_Query	D	1	chr7	.	CTGCAAGGACCAACCCAAAAGGTCTTCACTGTCTTTCTATGCAACTAACTGCTGATTACTTTGC
TP64089_Hit	D	1	chr7	.	CTGCAATCCATGTCAGGGCTAATCTCATAGCCCAAGTCAACACCACAGTCTTTAGGCATGGCTG
TP64089_Query	D	1	chr7	.	CTGCAATCCATGTCAGGGCTAATCTCATAGCCCAAGTCAACACCACAGTCTTTAGGCATGGCAG
TP64218_Hit	D	1	chr7	.	CTGCAATGACGATAAGGTATCATAATCAACCAAAGTCCAAACCTGCTTGTTTTGACGGCTGATC
TP64218_Query	D	1	chr7	.	CTGCAATGAAGATAAGGTATCATAATCAACCAAAGTCCAAACCTGCTTGTTTTGACGGCTGATC
TP64238_Hit	D	1	chr7	.	CTGCAATGAAGTTTCGGCGTTCCTTCTAGCCGAAGATGATGGTGAACTTGAAGAAGACGAAGAA
TP64238_Query	D	1	chr7	.	CTGCAATGAAGTTTCGGCGTTCCTTCTAGCCGAAGATGAAGGTGAACTTGAAGAAGACGAAGAA
TP64312_Hit	D	1	chr7	.	CTGCAATGCGTCCATTCCTTGGGAAAGAGCGTCTTCAGCTTGTTGAGATGACTGTTGCAAGTTG
TP64312_Query	D	1	chr7	.	CTGCAATGCATCCATTCCTTGGGAAAGAGCGTCTTCAGCTTGTTGAGATGACTGTTGCAAGTTG
TP64454_Hit	D	1	chr7	.	CTGCAATGGTTTCATATCCTGGTTGCCCATCACCTGCTTCTTTCCACATCATGTCAAAACAAGC
TP64454_Query	D	1	chr7	.	CTGCAATGGTTTCATATCCTGGTTGCCCATCACCTGCTTCTTTCCACATCATGGCAAAACAAGC
TP64455_Hit	D	1	chr7	.	CTGCAATGGTTTGACCGAAAAAATCAACCAGCTTGATGCTGAAAAAAAAAAAAAAAAAAAAAAA
TP64455_Query	D	1	chr7	.	CTGCAATGGTTTGACAGAAAAAATCAACCAGCTTGATGCTGAAAAAAAAAAAAAAAAAAAAAAA
TP64456_Hit	D	1	chr7	.	CTGCAATGGTTTGACAGAAAAAATCAACCAGCTTGATGTTGCGGTTAACACTTTGAAGAGTGGT
TP64456_Query	D	1	chr7	.	CTGCAATGGTTTGACAGAAAAAATCAACCAGCTTGATGTTGCAGTTAACACTTTGAAGAGTGGT
TP64488_Hit	D+G	1	chr7	.	CTGCGATGTCTGTATTTGAGTCTAAATACAAGGAAAGCTTAAGTGTAAGTAAACATATTAAGGC
TP64488_Query	D+G	1	chr7	.	CTGCAATGTCTGTATTTGAGTCTAAATACAAGGAAAGCTTAAGTGTAAGTAAACATATTAAGGC
TP64635_Hit	D	1	chr7	.	CTGCAATTCACCAGCAATTTCTTCAATTTTCTTGAGTTGTTTTGGTTGCAGTATTTTCCCATTG
TP64635_Query	D	1	chr7	.	CTGCAATTCACCAGCAATTTCTTCAATTCTCTTGAGTTGTTTTGGTTGCAGTATTTTCCCATTG
TP64894_Hit	D+G	1	chr7	.	CTGCAATTTCTTTGGTGATTTCAACAATTGCAAGAACCAGTGGAAATAAAACAGCACTATTGAT
TP64894_Query	D+G	1	chr7	.	CTGCAATTTCTATGGTGATTTCAACAATTGCAAGAACCAGTGGAAATAAAACAGCACTATTGAT
TP64910_Hit	D+G	1	chr7	.	CTGCAATTTGCAAATGGTAAATTTTGATAGAATAATGTCAGATTATAGGACCAATATCCAGACC
TP64910_Query	D+G	1	chr7	.	CTGCAATTTGCAAATGGTAAATTTTGATAGAATAATGTCAAATTATAGGACCAATATCCAGACC
TP65070_Hit	D	1	chr7	.	CTGCACAAATTTTGAGCAGACTGAGATGCAACGATCTCCGGTTCAGTTTTCTTATCATTGGAAT
TP65070_Query	D	1	chr7	.	CTGCACAAATTTTGAGCAGACTGAGATGCAACGATATCCGGTTCAGTTTTCTTATCATTGGAAT

Name	Filter	Nb hit (Mt4.0)	Mt Chr (Mt4.0)	Ms Chr (Li et al., 2014)	Sequence
TP65434_Hit	D+G	1	chr7	.	CTGCACTGCATGTCCCAAGAAGAGCGGTCTTGGTGTATGTATCTAAACTAAACTTCTTTAATAT
TP65434_Query	D+G	1	chr7	.	CTGCACAGCATGTCCCAAGAAGAGCGGTCTTGGTGTATGTATCTAAACTAAACTTCTTTAATAT
TP65565_Hit	D	1	chr7	.	CTGCACATATGCTATATGATCTTGAGGGCGTGAAGAAGACATCTTCAATTGAACCAACCCTTCT
TP65565_Query	D	1	chr7	.	CTGCACATATGCTATATGATCTTGAGGGCGTGAAGAAGACATCTTCAACTGAACCAACCCTTCT
TP65575_Hit	D	1	chr7	.	CTGCACATCAGCATGAACCCTCTGAATCTCCTGATTTAAATGTTGAGCTTTTGCCATAAGCTCC
TP65575_Query	D	1	chr7	.	CTGCACATCAGCATGAACCCTCTGAATCTCCTGATTTAAATGTTGAGCTTTCGCCATAAGCTCC
TP65739_Hit	D+G	1	chr7	.	CTGCACCAAAGGCATATGGACTTGGTCGTCATATGGTCCTTCCAGTCTCTTTACATGGTCTTCC
TP65739_Query	D+G	1	chr7	.	CTGCACCAAAGGCATATGGACTTGGTCGTCATATGGTCCTTCCAGTCTCTTTACACGGTCTTCC
TP65850_Hit	D	1	chr7	.	CTGCACCACGAGGAAGACCGTGTAAAGAGACTGGAAGGACCATATGACGACCAAGTCCATATGC
TP65850_Query	D	1	chr7	.	CTGCACCACGAGGAAGACCATGTAAAGAGACTGGAAGGACCATATGACGACCAAGTCCATATGC
TP65880_Hit	D	1	chr7	.	CTGCACCAGACTCCCAACATAACAGCAAAGACTCCCAACACAACAGCATAGTTATTAAATAATG
TP65880_Query	D	1	chr7	.	CTGCACCAGACTCCCAACACAACAGCAAAGACTCCCAACACAACAGCATAGTTATTAAATAATG
TP65921_Hit	D	1	chr7	.	CTGCACCGGTTAACCATGACTCAATATCATAGTCCAACACTGAAACTTTTTCAAGATGATGGTC
TP65921_Query	D	1	chr7	.	CTGCACCAGTTAACCATGACTCAATATCATAGTCCAACACTGAAACTTTTTCAAGATGATGGTC
TP65971_Hit	D	1	chr7	.	CTGCACCATCCATATTTTCAGAAAACTTGTGAGATTTGGTTATCTGAATCATATGAGAGAATTC
TP65971_Query	D	1	chr7	.	CTGCACCATCCATATTTGCAGAAAACTTGTGAGATTTGGTTATCTGAATCATATGAGAGAATTC
TP66004_Hit	D	1	chr7	.	CTGCACCATTTCTAATGAATGACATCATTCTGAAAACACTAACCTCAACCATGTTCATGGGCAA
TP66004_Query	D	1	chr7	.	CTGCACCATTTCTAATGAATGACATCATTCTGAAAACACTAACCTCAACCATGTTCATAGGCAA
TP661_Hit	D	1	chr7	.	CAGCAAAAGCTAACCATGACTTAATTTCATCATCCAACTTGGTCTCGTTAACAAGATCCACAGC
TP661_Query	D	1	chr7	.	CAGCAAAAGCTAACCATGACTTAATTTCATCATCCAACTTGGTCTCATTAACAAGATCCACAGC
TP6620_Hit	D+G	1	chr7	.	CAGCAAGTTGGTCAACAGCAGACATAGGTGGCTCCAATGAAAAGGATTTGATTTGGCCACCAGA
TP6620_Query	D+G	1	chr7	.	CAGCAAGTTGGTCAACAACAGACATAGGTGGCTCCAATGAAAAGGATTTGATTTGGCCACCAGA
TP66210_Hit	D	1	chr7	.	CTGCACCTCATTGACTTGATGATAGCTTTTGACAAAAAGATTATGTCTTTGGTAGAAAATTCTG
TP66210_Query	D	1	chr7	.	CTGCACCTCATTGACTTGATGATAGCTTTTGACAAAAAGATTACGTCTTTGGTAGAAAATTCTG
TP66317_Hit	D	1	chr7	.	CTGCACCTTCCCCATTTACCTCGTCTCACAATTCAGGTATTGCATTGTGCACCGAAAACTATTA
TP66317_Query	D	1	chr7	.	CTGCACCTTCCCCATTTACCTCGTCTCACAATTCAGGTATTACATTGTGCACCGAAAACTATTA
TP66675_Hit	D	1	chr7	.	CTGCACTAAAACATGATAAATAAAACCAACAAACTATAGCTAAAAATTAAGACCTGAATTTGTG
TP66675_Query	D	1	chr7	.	CTGCACTAAAACATGATAAATAAAACCAACAAACTATAGCTAAAAATGAAGACCTGAATTTGTG
TP66790_Hit	D	1	chr7	.	CTGCACTATACATTGACAATGCACGGTGGGATGGTGTACCGTTTTTGGTTAAAACAGGCTTGGG
TP66790_Query	D	1	chr7	.	CTGCACTATACATTGACAATGCACGATGGGATGGTGTACCGTTTTTGGTTAAAACAGGCTTGGG
TP67277_Hit	D	1	chr7	.	CTGCACTTTCCACTCCTGAATATTTCTGCACATGATTAGTTTGGACTTTGGATAATTAAGCTAT
TP67277_Query	D	1	chr7	.	CTGCACTTTCCACTCCTGAATATTTATGCACATGATTAGTTTGGACTTTGGATAATTAAGCTAT
TP68086_Hit	D	1	chr7	.	CTGCAGATAGGCCTACTATGGCGACAGTAATTTCTATGCTTATCAGTGAGGATGCATTTCTTCC
TP68086_Query	D	1	chr7	.	CTGCAGATAGGCCTACTATGGCGACAGTAATTTCTATGCTTATCAGTGAGAATGCATTTCTTCC
TP68131_Hit	D	1	chr7	.	CTGCAGATGAAACTCTTATATTTTGGAATGACTTTGGGAGCCCAGCAGAAGCATGCAAAGCCGC
TP68131_Query	D	1	chr7	.	CTGCAGATGAAACTCTTATATTTTGGAATGACTTTGGGAGCCCAGCAGAAGCATGCAAAGCCAC
TP68164_Hit	D	1	chr7	.	CTGCAGATGCTACAGGACAGGGTGAAATTGCCCGTGAAGCTCTGAAGAGGTTAGCTGAAAAAAA
TP68164_Query	D	1	chr7	.	CTGCAGATGCTACAGCACAGGGTGAAATTGCCCGTGAAGCTCTGAAGAGGTTAGCTGAAAAAAA
TP68892_Hit	D+G	1	chr7	.	CTGCAGATTGCTTCAGGCATACCATATTCTCATAGGAAGTCAGCGAGGGGGTATCCTATCCTCC
TP68892_Query	D+G	1	chr7	.	CTGCAGATTGCTTCAGGCATACCATATTCTCATAGGAAGTCAGCGAGGGGGTATCATATCCTCC
TP6897_Hit	D+G	1	chr7	.	CAGCAATTCCAAAGCATTGTTGGGTGAAAGATTCATGGAAGTCAATGAGTTATGTTTTGAGAGA
TP6897_Query	D+G	1	chr7	.	CAGCAATACCAAAGCATTGTTGGGTGAAAGATTCATGGAAGTCAATGAGTTATGTTTTGAGAGA
TP69039_Hit	D	1	chr7	.	CTGCCGGAGAATTTTCCAGCACCAACAGCTCACCCCATTTTGACCTATTTGTTACCAAAGATTT
TP69039_Query	D	1	chr7	.	CTGCAGGAGAATTTTCCAGCACCAACAGCTCACCCCATTTTGACCTATTTGTTACCAAAGATTT
TP69548_Hit	D	1	chr7	.	CTGCAGTAATAGATGTTGGCATGAATTCTGTTGATGACCCAACTAGGAAGGCAGGTTATAGGCT
TP69548_Query	D	1	chr7	.	CTGCAGTAATAGATGTCGGCATGAATTCTGTTGATGACCCAACTAGGAAGGCAGGTTATAGGCT
TP69574_Hit	D+G	1	chr7	.	CTGCAGTACTCAATTTTCCTTTTTGTGTACCTGTTTCATCACCCGCCCTCTCTGCAAATGCGAC
TP69574_Query	D+G	1	chr7	.	CTGCAGTACTCAATTTTCCTTTTTGTGTACCTGTTTCATCACCCACCCTCTCTGCAAATGCGAC
TP69817_Hit	D	1	chr7	.	CTGCAGTGTTTATATACCTAAGCAGTGACTACTATTAGAAACTCAAAGAAGTTGATGAATTCAT
TP69817_Query	D	1	chr7	.	CTGCAGTGATTATATACCTAAGCAGTGACTACTATTAGAAACTCAAAGAAGTTGATGAATTCAT
TP6982_Hit	D+G	1	chr7	.	CAGCAATGGCAAGGAAATACAAATAAGCAACCCTATGAAACTGAATAAATAAATTCTTTGGCAA
TP6982_Query	D+G	1	chr7	.	CAGCAATAGCAAGGAAATACAAATAAGCAACCCTATGAAACTGAATAAATAAATTCTTTGGCAA
TP69968_Hit	D+G	1	chr7	.	CTGCAGTTACTTGTGTTGGGGGTTCTTGTTTGGCCTCTTTTTGCCTGCTTCTTTGTGAAGACTA
TP69968_Query	D+G	1	chr7	.	CTGCAGTTACTTGTGTTGGGGGTTCTTGTTTGGCCTCTTTTTGCCTGCTTCTTTGGGAAGACTA

Name	Filter	Nb hit (Mt4.0)	Mt Chr (Mt4.0)	Ms Chr (Li et al., 2014)	Sequence
TP7017_Hit	D	1	chr7	.	CAGCAATAGCTGACAAGCGATATTCACCAGGAAGAACCTAGACATTAAACCAATAAGATAGAAT
TP7017_Query	D	1	chr7	.	CAGCAATAGCTGACAAGCAATATTCACCAGGAAGAACCTAGACATTAAACCAATAAGATAGAAT
TP70333_Hit	D	1	chr7	.	CTGCATAACCTTCTACAACCATTCATAATGATACTGCTTCTTCCCTTTCCAGTTGCCTTGAAAA
TP70333_Query	D	1	chr7	.	CTGCATAACCTTCTACAACCATTCAGAATGATACTGCTTCTTCCCTTTCCAGTTGCCTTGAAAA
TP70587_Hit	D+G	1	chr7	.	CTGCATAGCGCTCATACATCCCTTTTTCCTCATCGAGAAGTAAATCCCTAATATTATTAATTTA
TP70587_Query	D+G	1	chr7	.	CTGCATAGCGCTCATACATCCCCTTTTCCTCATCGAGAAGTAAATCCCTAATATTATTAATTTA
TP7064_Hit	D	1	chr7	.	CAGCAATAGTGTCTTCAAGTTGAGACTCAGCGTCCAATATATTTTCTTTGCAATCAGCCAAACC
TP7064_Query	D	1	chr7	.	CAGCAATAGTGTCTTCAAGTTGAGACTCAGCATCCAATATATTTTCTTTGCAATCAGCCAAACC
TP70777_Hit	D+G	1	chr7	.	CTGCATATGCGGCTTTTAACAGCAGATGGCGCAGAACCATTGGGCGCATGTGCATTTCGTGGTG
TP70777_Query	D+G	1	chr7	.	CTGCATATGCGGCTTTTAACAGCAGATGGCGCAGAACCATTGGGCGCATGTGCATTTCGTGGGG
TP7085_Hit	D	1	chr7	.	CAGCAATATAAACAAGCGGATAATAGTGTTGTGGTTGAGCATGCCAAGCATCGAGTGGAAGTTG
TP7085_Query	D	1	chr7	.	CAGCAATATAAACAAGCGGATAATAGTGTTGTGGTTGAGCATACCAAGCATCGAGTGGAAGTTG
TP70881_Hit	D+G	1	chr7	.	CTGCATCAAAGGGACCAACTATTTTGGTTTTGATGTCTGGTGGGCCTGTGGACATTACTTTTGC
TP70881_Query	D+G	1	chr7	.	CTGCATCAAAGGGACCAACTATATTGGTTTTGATGTCTGGTGGGCCTGTGGACATTACTTTTGC
TP70932_Hit	D+G	1	chr7	.	CTGCATCAACTTGCAGAGCATTGATTATTGAGCATGATCTAATTCTCAGATGCAATCGCCGAAG
TP70932_Query	D+G	1	chr7	.	CTGCATCAACTTGCAGACCATTGATTATTGAGCATGATCTAATTCTCAGATGCAATCGCCGAAG
TP71290_Hit	D	1	chr7	.	CTGCATCCTTATACAATCCTCAACTTGTTGACATGATCGGATCACTAAACCAAGAGATTGGCTC
TP71290_Query	D	1	chr7	.	CTGCATCCTTATACAATCCTCAACTTGTTGACATGATCAGATCACTAAACCAAGAGATTGGCTC
TP7154_Hit	D	1	chr7	.	CAGCAATATCTCATTTCCAATTGCTAATAATGTAATTTAATAATAATTGGGCACAGTAAAAAGC
TP7154_Query	D	1	chr7	.	CAGCAATATCTCATTTCCAATTGCTAATAATGTAATTCAATAATAATTGGGCACAGTAAAAAGC
TP72093_Hit	D	1	chr7	.	CTGCATGTCTCAGAAGTTCAGCTCTTCCTTCAGCACACTGACAAAGATTCTCCAACAAAACCAA
TP72093_Query	D	1	chr7	.	CTGCATGTCTCAAAAGTTCAGCTCTTCCTTCAGCACACTGACAAAGATTCTCCAACAAAACCAA
TP72264_Hit	D	1	chr7	.	CTGCATTAAGCGAATTGATAGCAGAAAACCGAGCATCGATAATGGCTAGGCAATACGTCTTAGA
TP72264_Query	D	1	chr7	.	CTGCATTAAGCGAATTGATAGCAGAAAACCGAGCATCGATAATGGCTAGGCAATACGTCTCAGA
TP72302_Hit	D+G	1	chr7	.	CTGCATTACTAAAATGAAATTCACCAAACAAATGGAAGGGAAGAAAATGAGAAGAAATAAAAGG
TP72302_Query	D+G	1	chr7	.	CTGCATTACTAAAATGAAATTCACCAAACAAATGGAAAGGAAGAAAATGAGAAGAAATAAAAGG
TP72439_Hit	D	1	chr7	.	CTGCATTCAAGGCCATGTTTGATTCACCATCTTACGCAGTTGACCCTGTTGGTTCAACTGTTCC
TP72439_Query	D	1	chr7	.	CTGCATTCAAGGCCATGTTTGATTCACCATCGTACGCAGTTGACCCTGTTGGTTCAACTGTTCC
TP72442_Hit	D+G	1	chr7	.	CTGCATTCAATATGATGCTTGCAGAAGGGCTGAAACCAAATGTAGTCTCATACAATGCACTACT
TP72442_Query	D+G	1	chr7	.	CTGCATTCAATATGATGCTTGCAGAAGGGCTGAAACCAAATATAGTCTCATACAATGCACTACT
TP72514_Hit	D	1	chr7	.	CTGCATTCCTTCCATTGGTTAATGCGTGAGTAGCTTTCTGGGGTCAGCTTGGTTGGTTAATTGG
TP72514_Query	D	1	chr7	.	CTGCATTCCTTCCATTGGTTAATGCGTGAGTAGCTTTCTGGGGTCAGCTTGGTTGGTTAATTGA
TP7278_Hit	D+G	1	chr7	.	CAGCAATCAACCAATTCAGACATTACCATGCTTTCGCCGTAACTTAGAACAGCGCCATGTGGAG
TP7278_Query	D+G	1	chr7	.	CAGCAATCAACCAACTCAGACATTACCATGCTTTCGCCGTAACTTAGAACAGCGCCATGTGGAG
TP72944_Hit	D	1	chr7	.	CTGCATTTCGTTCATCTAGGCACAATGAAGCTTACATTTTCATAAATGATAAGTACGTGCTATT
TP72944_Query	D	1	chr7	.	CTGCATTTCGTTCATCCAGGCACAATGAAGCTTACATTTTCATAAATGATAAGTACGTGCTATT
TP72946_Hit	D	1	chr7	.	CTGCATTTCGTTCATCTAGGCACAATGAAGCTTACATTTTCATCAATGATGATATGTACGTGCT
TP72946_Query	D	1	chr7	.	CTGCATTTCGTTCATCTAGGCACAATGAAGCTTACATTTTCATCAATGATGATAAGTACGTGCT
TP73149_Hit	D	1	chr7	.	CTGCATTTTATCCTCAGAATAACTTATCATGCAGTAAAGCTTCTCATAGTTCTATTCATATTCC
TP73149_Query	D	1	chr7	.	CTGCATTTTATCCTCAGAATAACTTATCACGCAGTAAAGCTTCTCATAGTTCTATTCATATTCC
TP73181_Hit	D	1	chr7	.	CTGCATTTTGAACTTCATGCCAAATTCCGGCGGAATTCCAAACTTGGATAGCAAAAGAACATTC
TP73181_Query	D	1	chr7	.	CTGCATTTTGAACTTCAGGCCAAATTCCGGCGGAATTCCAAACTTGGATAGCAAAAGAACATTC
TP73404_Hit	D	1	chr7	.	CTGCCAAACTCACTCTGATACCAAACAAGCTTTAAGTTCTCTCGTGCAAGGTCTTTTGTTAACC
TP73404_Query	D	1	chr7	.	CTGCCAAACTCACTCTGATACCAAACAAGCTTTAAGTTCTCTAGTGCAAGGTCTTTTGTTAACC
TP73742_Hit	D	1	chr7	.	CTGCCAAGGAAACAGAGAGAACATTTTCACGCCTAGACATGTGGACATCATCAGAAAGTATTCC
TP73742_Query	D	1	chr7	.	CTGCCAAGGAAACAGAGAGAACATTTTCACGCCTAGACATGTGGACATAATCAGAAAGTATTCC
TP73759_Hit	D	1	chr7	.	CTGCCAAGGCCTCCAAGCGAGGATCGAGGATGGGAGGATACTTCAAATTAATGGAATGATAGAG
TP73759_Query	D	1	chr7	.	CTGCCAAGGCCTCCAAGCGAGGATCAAGGATGGGAGGATACTTCAAATTAATGGAATGATAGAG
TP741_Hit	D	1	chr7	.	CAGCAAAAGTAAACTGAAAATAAACAAGTGAAGCCATAGGAAAAAAAGGTTCTTCCATTGGTGT
TP741_Query	D	1	chr7	.	CAGCAAAAGTAAACTGAAAATAAACAAGTGAAGCCATAGGAAAAAAAGGTTCTTCCATAGGTGT
TP74139_Hit	D	1	chr7	.	CTGCCACAGCCGCCAACAACGGCATGGCGGTGGATGCGTTGGTTCCCTGCTCCAATACTACTAA
TP74139_Query	D	1	chr7	.	CTGCCACAGCCGCCAACAACGGCATGACGGTGGATGCGTTGGTTCCCTGCTCCAATACTACTAA
TP74411_Hit	D	1	chr7	.	CTGCCGCGTAACCTATGTTTGTCTAGGAGGTCGTAGGTCCATGTTTTGGAATCAGCCTCTTGCA
TP74411_Query	D	1	chr7	.	CTGCCACGTAACCTATGTTTGTCTAGGAGGTCGTAGGTCCATGTTTTGGAATCAGCCTCTTGCA

Name	Filter	Nb hit (Mt4.0)	Mt Chr (Mt4.0)	Ms Chr (Li et al., 2014)	Sequence
TP74966_Hit	D	1	chr7	.	CTGCCAGTTTCAGAAACAAGCTCTTCTGTCTCCTTGGTTTCGGTAACTTCTTCACGACCTTCTG
TP74966_Query	D	1	chr7	.	CTGCCAGTTTCAGAAACAAGCTCTTCTGTCTCCTTGGTTTCGATAACTTCTTCACGACCTTCTG
TP7528_Hit	D	1	chr7	.	CAGCAATCTGGTCATTACTGACAGTCTCGTCATGTTGGGTAGAAATGAGGACAGTGTGGACACG
TP7528_Query	D	1	chr7	.	CAGCAATCTGGTCATTACTGACAGTCTCGTCATGTTGAGTAGAAATGAGGACAGTGTGGACACG
TP75567_Hit	D	1	chr7	.	CTGCCATTATTGATTACTTTTCTTCTGAAGTTACTCGTGGAGTATGGAAACAGGTTCCAATGAA
TP75567_Query	D	1	chr7	.	CTGCCATTATTGATTACTTTTCTTCAGAAGTTACTCGTGGAGTATGGAAACAGGTTCCAATGAA
TP7575_Hit	D	1	chr7	.	CAGCAATGAAACATCTTGATCTTCTGCTTCATATTTCTTACTATACTTTTTCAAGTTCTTTGTG
TP7575_Query	D	1	chr7	.	CAGCAATGAAACATCTTGATCTTCTGCTTCATATTTCTTACTATACTTTTTCAAGTTCTTTGCG
TP75863_Hit	D	1	chr7	.	CTGCCCAAGCTTTATATACACTACACATGACCTATCTCTAAGCAGTGTGGGAGTAAATCCACCC
TP75863_Query	D	1	chr7	.	CTGCCCAAGCTTTATATACACTACACATGACCTATCTCTAAGCAGTGTGGGACTAAATCCACCC
TP75871_Hit	D+G	1	chr7	.	CTGCCCAAGTGAGTTGATACTCATCATCCTCTTGTGTGTGCCGATCACCTCGCTCGAACACGTC
TP75871_Query	D+G	1	chr7	.	CTGCCCAAGTGAGTTGATACTCATCATCCTCTTGTGTGTGCCGATCACCCCGCTCGAACACGTC
TP76020_Hit	D	1	chr7	.	CTGCCCATAAAATCCAATGCTAGGTGGCCTGAAATATCCTCCTGGTGATGACTGCTGGTGCTGA
TP76020_Query	D	1	chr7	.	CTGCCCATAAAATCCAATGCCAGGTGGCCTGAAATATCCTCCTGGTGATGACTGCTGGTGCTGA
TP76790_Hit	D	1	chr7	.	CTGCCCTGAGTGCAAACAACGTCTTGATGATCCTGCATTGTGCCTTCTGTGTGGTAGATTGTGC
TP76790_Query	D	1	chr7	.	CTGCCCTGAGTGCAAACAACGTCTTGATGATCCTGCATTGTGCCTGCTGTGTGGTAGATTGTGC
TP7711_Hit	D	1	chr7	.	CAGCAATGATGGCAATGATGAACTGGAGATTCTTTTTGCATACCTTTCATTGTATCCACAAAGA
TP7711_Query	D	1	chr7	.	CAGCAATGATGGCAATGATGAACTGGAGATTCTTTTGGCATACCTTTCATTGTATCCACAAAGA
TP77183_Hit	D	1	chr7	.	CTGCCGATCATAGTTCACGTCTTCAGACAAGAAGAATCGAGAGGCAGTTAGGAGATACCACCAG
TP77183_Query	D	1	chr7	.	CTGCCGATCATAGTTCACGTCTTCAAACAAGAAGAATCGAGAGGCAGTTAGGAGATACCACCAG
TP77565_Hit	D	1	chr7	.	CTGCCGTAACTGAAGGATTACCATTTTGAAGGGATATATGCTTTATCTTCCATGTTAAAATAAA
TP77565_Query	D	1	chr7	.	CTGCCGGAACTGAAGGATTACCATTTTGAAGGGATATATGCTTTATCTTCCATGTTAAAATAAA
TP777_Hit	D	1	chr7	.	CAGCAAAAGTGGTCCGTGGGATATGATCTGGTATGAAAATAGAGCAGAAGACTGATTCTCAAGA
TP777_Query	D	1	chr7	.	CAGCAAAAGTGGTCCATGGGATATGATCTGGTATGAAAATAGAGCAGAAGACTGATTCTCAAGA
TP7771_Hit	D+G	1	chr7	.	CAGCAATGCCAAATTACAACAAAATTAAGAAAAAATGATATCTATTGCCACAACACATTCAACA
TP7771_Query	D+G	1	chr7	.	CAGCAATGCCAAATTACAACAAAATTAAGAAAAAATGATATCTAATGCCACAACACATTCAACA
TP77835_Hit	D+G	1	chr7	.	CTGCCGTACAAAGCTAGAGAAGTTGCTATGCTTGAAGAGTGTTGGAAGGATATCACGAGCAAAC
TP77835_Query	D+G	1	chr7	.	CTGCCGTACAAAACTAGAGAAGTTGCTATGCTTGAAGAGTGTTGGAAGGATATCACGAGCAAAC
TP78686_Hit	D	1	chr7	.	CTGCCTTTGAGAAATGTTTACCCAGAAAGCAACCTCTAGTGGACCTTGCTCAATTTTACCTCTG
TP78686_Query	D	1	chr7	.	CTGCCTCTGAGAAATGTTTACCCAGAAAGCAACCTCTAGTGGACCTTGCTCAATTTTACCTCTG
TP78693_Hit	D	1	chr7	.	CTGCCTCTGATTCAATTGGGTAAAAGATCCAAGCAGAAGTTGTGGAACATACCTGTGAATCTGA
TP78693_Query	D	1	chr7	.	CTGCCTCTGATTCAATTGGGTAAAAAATCCAAGCAGAAGTTGTGGAACATACCTGTGAATCTGA
TP79720_Hit	D	1	chr7	.	CTGCGAAATTGTGATTATTTTTCTCTCTGCCTATTGTTCAAATTTTTAAAACAAACTAGCTAGG
TP79720_Query	D	1	chr7	.	CTGCGAAATTGTGATTATTTTTCTCTCCGCCTATTGTTCAAATTTTTAAAACAAACTAGCTAGG
TP79796_Hit	D+G	1	chr7	.	CTGCGAAGATCGCTTCGACCTAATTCCCGACCTAATCGAAATCCTCAAGTCTCTTCACCTAAAA
TP79796_Query	D+G	1	chr7	.	CTGCGAAGATCGCTCCGACCTAATTCCCGACCTAATCGAAATCCTCAAGTCTCTTCACCTAAAA
TP80033_Hit	D+G	1	chr7	.	CTGCGACATTGTACAATCCTCAACTACAACACATGATACAAGGACTCAACAAGAAAATTGGCAA
TP80033_Query	D+G	1	chr7	.	CTGCGACATTGTACAATCCTCAAATACAACACATGATACAAGGACTCAACAAGAAAATTGGCAA
TP80347_Hit	D	1	chr7	.	CTGCGAGGATAAGAAATCAGTCTTCTATGCCGCATACAACAATAGGAATGTCCAAAATTACGGG
TP80347_Query	D	1	chr7	.	CTGCGAGGATAAGAAATCAGTCTTCTATGCCGCATACAACAATAGGAATGTCCAAAATTACCGG
TP80438_Hit	D	1	chr7	.	CTGCGAGTTTAGGTCTTCTAGATGGGGTGTGTGTTGAGCTTGTAATAATTGCAAAATTTATGTT
TP80438_Query	D	1	chr7	.	CTGCGAGTTTAGGTCTTCTAGATGAGGTGTGTGTTGAGCTTGTAATAATTGCAAAATTTATGTT
TP80454_Hit	D	1	chr7	.	CTGCGATAAGAGAATGGAAAAGATACAAGCAAGAAAATAGGAAGTACTTGGCTTGGAAGGGCAA
TP80454_Query	D	1	chr7	.	CTGCGATAAGAGAATGGAAAAGATACAAGCAAGAAAATAGAAAGTACTTGGCTTGGAAGGGCAA
TP80875_Hit	D	1	chr7	.	CTGCGCACAAAGTGTTTGGGGCTAGCAATGTATCCAAACTCCTTCTTGGCATTCCTGCTAGTAG
TP80875_Query	D	1	chr7	.	CTGCGCACAAAGTGTTTGGGGCTAGCAATGTATCCAAACTCCTTCTTGGCATTCCAGCTAGTAG
TP8131_Hit	D+G	1	chr7	.	CAGCAATTAACTAATGCTCAATTTTTGCATTCAGAAGAAGGAAGCAGTAGAAGAACCTGAAATG
TP8131_Query	D+G	1	chr7	.	CAGCAATTAACAAATGCTCAATTTTTGCATTCAGAAGAAGGAAGCAGTAGAAGAACCTGAAATG
TP81999_Hit	D	1	chr7	.	CTGCGGATAAGGCGAAGCAGGCTAAGGATGCGACAGTGCAGAAGGCTGGAGAAGCTAAAGATGC
TP81999_Query	D	1	chr7	.	CTGCGGATAAGGCGAAGCAGGCGAAGGATGCGACAGTGCAGAAGGCTGGAGAAGCTAAAGATGC
TP8229_Hit	D	1	chr7	.	CAGCAATTATCATTCAAAGTCAACTTTGGTACTTAAAATGCATTCCTTCAACAGCGAGAGATTT
TP8229_Query	D	1	chr7	.	CAGCAATTATCATTCAAAGTCAACTTTGGTACTTAAAATGCATTCCTTCAACAACGAGAGATTT
TP82834_Hit	D	1	chr7	.	CTGCGGTGGCCTGGGAACCCAACAAACCCTTAACAATTGAAGATGTTGAGGTCGCTCCTCCGCA
TP82834_Query	D	1	chr7	.	CTGCGGTGGCCTGGGAACCCAACAAACCCTTAACAATCGAAGATGTTGAGGTCGCTCCTCCGCA

Name	Filter	Nb hit (Mt4.0)	Mt Chr (Mt4.0)	Ms Chr (Li et al., 2014)	Sequence
TP8303_Hit	D+G	1	chr7	.	CAGCAATTCCAGTTGTCCACTTTGTCGGCGCTCCCTCTCAGAATCTGAGTTATTCTCAGCTCCC
TP8303_Query	D+G	1	chr7	.	CAGCAATTCCAGTTGTCCACTTTGTCGGCGCTCCCTCTCAGAATCTGAGTTATTCTCAGCACCC
TP83100_Hit	D+G	1	chr7	.	CTGCGTAGATGTGATCGGTGAATTTGTTGCGAGTTTCTACTCCTCTGAAACTCAAGAACACGTG
TP83100_Query	D+G	1	chr7	.	CTGCGTAGAGGTGATCGGTGAATTTGTTGCGAGTTTCTACTCCTCTGAAACTCAAGAACACGTG
TP83154_Hit	D+G	1	chr7	.	CTGCGTATCTTCCCCATGGTCTTCTTCGTACTCCTAGGTATCTTCCTCCCCATGCTGTTTGTTG
TP83154_Query	D+G	1	chr7	.	CTGCGTATCTTCCCCATGGTCTTCTTCGTACTCCTAGGTATCTTCCTCCCCATGCTGTTTGCTG
TP8320_Hit	D+G	1	chr7	.	CAGCAATTCGCTGGAATAAATGCTGTGGTTTATTATTCAACTTCGGTCTTTCGTAGTGCTGGAA
TP8320_Query	D+G	1	chr7	.	CAGCAATTCGCTGGAATAAATGCTGTGGTTTATTATTCAACTTCGGTCTTTCGCAGTGCTGGAA
TP8360_Hit	D	1	chr7	.	CAGCAATTGAAGCTTTTGTATGGAAAACTAACTAACATGACCTCAGTTGCCTATAAAAATTGTC
TP8360_Query	D	1	chr7	.	CAGCAATTGAAGCCTTTGTATGGAAAACTAACTAACATGACCTCAGTTGCCTATAAAAATTGTC
TP83949_Hit	D+G	1	chr7	.	CTGCGTTTCATGGTTTTGAACTGTGCACACCACCTGTTCGAGAAAAGTACCGTTTGTGTCTGGA
TP83949_Query	D+G	1	chr7	.	CTGCGTTTCATGGTTTTGAACTGTGCACACCACCTGTTCGAGAAAAGTACCGTTTGTGTATGGA
TP8406_Hit	D	1	chr7	.	CAGCAATTGCAACACAATGTGCATGGAGAGGAAAAGTTGCCCGGCGAGAACTGCGGAAACTCAA
TP8406_Query	D	1	chr7	.	CAGCAATTGCAACACAATGTGCATGGAGAGGAAAAGTTGCCCGGCGACAACTGCGGAAACTCAA
TP8408_Hit	D+G	1	chr7	.	CAGCAATTGCAACTGATGCAACAGCGCAATGCACAATTACAACGAAGGGATCCTAATCATCCTG
TP8408_Query	D+G	1	chr7	.	CAGCAATTGCAACTGATGCAACAGCGCAATGCACAATTACAACGAAGAGATCCTAATCATCCTG
TP84181_Hit	D+G	1	chr7	.	CTGCTAAAGATTCAAAAGAAATAACAAAATAAAGTTGAAACTTCAAAACTAGGGTTATGAAAAT
TP84181_Query	D+G	1	chr7	.	CTGCTAAAGATTCAAAAGAAATAACAAAATAAAATTGAAACTTCAAAACTAGGGTTATGAAAAT
TP8427_Hit	D	1	chr7	.	CAGCGATTGCCCTCAAAGCTGTTTGCAAAGTAAGCACTGATGCATGGAGTTTATTGTAGGATTT
TP8427_Query	D	1	chr7	.	CAGCAATTGCCCTCAAAGCTGTTTGCAAAGTAAGCACTGATGCATGGAGTTTATTGTAGGATTT
TP84347_Hit	D+G	1	chr7	.	CTGCTAACTAAAATTTTGTTTGGTGGACATACATACCTTCTTTTAGAAGATTTCTCAATGTCTG
TP84347_Query	D+G	1	chr7	.	CTGCTAACTAAAATTTTGTTTGGAGGACATACATACCTTCTTTTAGAAGATTTCTCAATGTCTG
TP84605_Hit	D+G	1	chr7	.	CTGCTAATGACTTTGGCAACATACATCATTTTCCTCCTCTAGCAGTGTTACATCCAAAAACTGT
TP84605_Query	D+G	1	chr7	.	CTGCTAATGACTTTGGCAACATACACCATTTTCCTCCTCTAGCAGTGTTACATCCAAAAACTGT
TP85073_Hit	D	1	chr7	.	CTGCTACCTTTGCCATGTCGTCAAAGTTGTAACCTCCAGCCAAAGATGGATCCACAAGCTGAAA
TP85073_Query	D	1	chr7	.	CTGCTACCTTTGCCATGTCATCAAAGTTGTAACCTCCAGCCAAAGATGGATCCACAAGCTGAAA
TP85122_Hit	D	1	chr7	.	CTGCTACGTCGAATGGTGGTGGTTGGGATGATAATTGGGATAATGATGATGGTGATAGTTATGG
TP85122_Query	D	1	chr7	.	CTGCTACGTCGAATGGTGGCGGTTGGGATGATAATTGGGATAATGATGATGGTGATAGTTATGG
TP85139_Hit	D	1	chr7	.	CTGCTACTAAATATGGACAATTAGATTTGTCTTCTTCGAGGTTTCCTTTCTATGACTAATTAAA
TP85139_Query	D	1	chr7	.	CTGCTACTAAATATGGACAATAAGATTTGTCTTCTTCGAGGTTTCCTTTCTATGACTAATTAAA
TP85436_Hit	D	1	chr7	.	CTGCTAGAAATGCTTCTGCAATGTTAAACGATGTGAAATCAATGATTGATGATGCTAATTTAGA
TP85436_Query	D	1	chr7	.	CTGCTAGAAATGCTTCTGCAATGTTAAACGACGTGAAATCAATGATTGATGATGCTAATTTAGA
TP85502_Hit	D	1	chr7	.	CTGCTAGAGACTATGAAAGGTTGCAAATGGAGGGAGGCAAGTGCCAAAGTAGTGAGAAAGTGAA
TP85502_Query	D	1	chr7	.	CTGCTAGAGACTATGAAAGGTTGCAAATGGAGGGAGGCAAGTGCCAAAGTAGCGAGAAAGTGAA
TP85569_Hit	D+G	1	chr7	.	CTGCTAGATTACTGGTTGAGTCAGCTGTTCTTTCTTGGAAGACAAAATTCCCTTACTGTAAGAT
TP85569_Query	D+G	1	chr7	.	CTGCTAGATTACTGGTTGAGTCAGCTATTCTTTCTTGGAAGACAAAATTCCCTTACTGTAAGAT
TP85586_Hit	D+G	1	chr7	.	CTGCTAGTACCACTAGAGGAGTGTAATGGAACAAAGGTGTTAGGAATAATAATGTCAACATCAC
TP85586_Query	D+G	1	chr7	.	CTGCTAGCACCACTAGAGGAGTGTAATGGAACAAAGGTGTTAGGAATAATAATGTCAACATCAC
TP8565_Hit	D	1	chr7	.	CAGCAATTTCCCATGGTTTAGAAGAGATCTGGGTTTGGAATTTGGATCTGAGATGAGAATTTCC
TP8565_Query	D	1	chr7	.	CAGCAATTTCCCATGGTTTACAAGAGATCTGGGTTTGGAATTTGGATCTGAGATGAGAATTTCC
TP85663_Hit	D	1	chr7	.	CTGCTAGCTCCTGTTGTCGTTTCTCTTCTTCAAGAGACCATGAACAATTGCCCGACTACAGTGA
TP85663_Query	D	1	chr7	.	CTGCTAGCTCCTGTCGTCGTTTCTCTTCTTCAAGAGACCATGAACAATTGCCCGACTACAGTGA
TP86123_Hit	D	1	chr7	.	CTGCTATAGTTATCGGGAATGGTCTGGCTTTATCATCCACCGCCGTTGTTCTGCAGGTTAATAA
TP86123_Query	D	1	chr7	.	CTGCTATAGTTATCGGGAATGGCCTGGCTTTATCATCCACCGCCGTTGTTCTGCAGGTTAATAA
TP86220_Hit	D	1	chr7	.	CTGCTATCACATTCTAATAGTTAAGACTTGGTATCTCCCCCAACTACTAATACAAACAAAAAAC
TP86220_Query	D	1	chr7	.	CTGCTATCACATTCTAATAGTTAAGACTTGGTATCTCCCACAACTACTAATACAAACAAAAAAC
TP86313_Hit	D+G	1	chr7	.	CTGCTATCTCCTAACTCAGACAAACAAGATTTTACCTTTTCTCGATCAGCTGGCGCAGGAAACA
TP86313_Query	D+G	1	chr7	.	CTGCTATCTCCCAACTCAGACAAACAAGATTTTACCTTTTCTCGATCAGCTGGCGCAGGAAACA
TP86453_Hit	D	1	chr7	.	CTGCTATGCTCGTTGTTTCAAGTAAACCTGAAGTAATTCTTCTGCCAAAGTGTTTATACAATTC
TP86453_Query	D	1	chr7	.	CTGCTATGCTCGTTGTTTCAAGCAAACCTGAAGTAATTCTTCTGCCAAAGTGTTTATACAATTC
TP868_Hit	D	1	chr7	.	CAGCAAAATCAAGCTCGGTACTCATGCAAATATTAGAATGGTTGGCTCCACTTGCGCACAACAC
TP868_Query	D	1	chr7	.	CAGCAAAATCAAGCTCGGTACTCATGCAAATATTAGAATGGTTGGCTCCACTTGCACACAACAC
TP86824_Hit	D	1	chr7	.	CTGCTATTGTTGCTAAATGCAAGGTTCAGAGCTCTAAAAGCCAAACCCCTGCACTGATAGCAAC
TP86824_Query	D	1	chr7	.	CTGCTATTGTTGCTAAATGCAAGGTTCAGAGCTCTAAAAGCCAAACCCCTGCACTGATACCAAC

Name	Filter	Nb hit (Mt4.0)	Mt Chr (Mt4.0)	Ms Chr (Li et al., 2014)	Sequence
TP86833_Hit	D	1	chr7	.	CTGCTATTTAACAACAACGATTTTATGAGAATGAAGTTTTATGATTTAGCACTGTTTTCACTAT
TP86833_Query	D	1	chr7	.	CTGCTATTTAACAACAAAGATTTTATGAGAATGAAGTTTTATGATTTAGCACTGTTTTCACTAT
TP86878_Hit	D+G	1	chr7	.	CTGCTATTTGACAACACTGAAATCAATAGATTGCAAGATCACTGTAATTATTGCAAATTGTTCC
TP86878_Query	D+G	1	chr7	.	CTGCTATTTGACAACACTGAAATCAATAGATTGCAAGATCACTGTAACTATTGCAAATTGTTCC
TP86996_Hit	D+G	1	chr7	.	CTGCTGAAAATGGTCATCTTGATATAGTCAAGGAATTGATCAAATATCATGATATTGGGTTGGC
TP86996_Query	D+G	1	chr7	.	CTGCTCAAAATGGTCATCTTGATATAGTCAAGGAATTGATCAAATATCATGATATTGGGTTGGC
TP88209_Hit	D	1	chr7	.	CTGCTCCTGGCTTGGGAATGATGACTCATGAGGTAATTGGTCTTAAATTCACCACATCCTTTTG
TP88209_Query	D	1	chr7	.	CTGCTCCTGGCTTGGGAATGATGACTCATGAGGAAATTGGTCTTAAATTCACCACATCCTTTTG
TP88291_Hit	D	1	chr7	.	CTGCTCGAACGAAAACAATTTTGTTGTGGGCGAGGTGTTGGTGGTCATTGGGGTACATGAGAGA
TP88291_Query	D	1	chr7	.	CTGCTCGAACGAAAACAAATTTGTTGTGGGCGAGGTGTTGGTGGTCATTGGGGTACATGAGAGA
TP8838_Hit	D	1	chr7	.	CAGCACAAATCCAATTCCAATGCCAAAGGAAAAGAAATTTGAGGACCGATATAGTGCCCCTGTG
TP8838_Query	D	1	chr7	.	CAGCACAAATCCAATTCCAATGCCAAAGGAAAAGAAATTTGAGGACCGATATAGTGCCCCTATG
TP8843_Hit	D	1	chr7	.	CAGCACAGATGATGTTTTAACTATTAAGGAACATTAGATGAAACAAAAGGGATTTTCACAACAT
TP8843_Query	D	1	chr7	.	CAGCACAAATGATGTTTTAACTATTAAGGAACATTAGATGAAACAAAAGGGATTTTCACAACAT
TP8940_Hit	D	1	chr7	.	CAGCACAACTTGAAAGGAGTCATGGTTCCAGGGAAACTCTTGATTCATTACTTCGCAAAGCAGT
TP8940_Query	D	1	chr7	.	CAGCACAACTTGAAAGGAGTCATGGTACCAGGGAAACTCTTGATTCATTACTTCGCAAAGCAGT
TP89502_Hit	D	1	chr7	.	CTGCTGAAATGGTGAAGAGGAATGTGGTTGAGGTTGGTGATTTTAGATTCTTTGATGGGTATTG
TP89502_Query	D	1	chr7	.	CTGCTGAAATGGTAAAGAGGAATGTGGTTGAGGTTGGTGATTTTAGATTCTTTGATGGGTATTG
TP89504_Hit	D+G	1	chr7	.	CTGCTGAAATGTGTTGTTGTTTCCATGAAAAAATGGACTAGAGTTTATATAATCAGACCATTTA
TP89504_Query	D+G	1	chr7	.	CTGCTGAAATGTGTTGTTGTTTCCAAGAAAAAATGGACTAGAGTTTATATAATCAGACCATTTA
TP89541_Hit	D	1	chr7	.	CTGCTGAACCAATTTATAACAACCCTTCTAATGGTCAGAACCCAAATCGTTATTCTGTCTTGCC
TP89541_Query	D	1	chr7	.	CTGCTGAACCAATTTATAACAACCCTTCTAATGGTCAGAACCCAAATCGTTATTCTATCTTGCC
TP89576_Hit	D	1	chr7	.	CTGCTGAAGAAATTTCATCTATGGTGCTCATGAAGATGCGTGAGATTGCTGAAGCTTATCTTGG
TP89576_Query	D	1	chr7	.	CTGCTGAAGAAATTTCATCTATGGTGCTCATGAAGATGCGTGAGATTGCCGAAGCTTATCTTGG
TP89599_Hit	D	1	chr7	.	CTGCTGAAGCAATGGGAGCTGAGCGCGACCACCTTGCATCGGTGGTCAGCTCAGCTGTGAATGT
TP89599_Query	D	1	chr7	.	CTGCTGAAGCAATGGGAGCTGAGCACGACCACCTTGCATCGGTGGTCAGCTCAGCTGTGAATGT
TP89729_Hit	D	1	chr7	.	CTGCTGAATTAGCAAGTGAGGCTAGTCCTTTCAATGCTTGTCCCATGTCAATACCTGCTGGTGC
TP89729_Query	D	1	chr7	.	CTGCTGAATTAACAAGTGAGGCTAGTCCTTTCAATGCTTGTCCCATGTCAATACCTGCTGGTGC
TP89841_Hit	D	1	chr7	.	CTGCTGAGAAAATGGGGATACCAATGGGGTTTAAGTTGGTTAGAGGTGCTTATATGTCTAGTGA
TP89841_Query	D	1	chr7	.	CTGCTGAGAAAATGGGGATACCAATGGGATTTAAGTTGGTTAGAGGTGCTTATATGTCTAGTGA
TP8998_Hit	D	1	chr7	.	CAGCACAAGATTTTATCCGGAGAGGTGGCACTTCATTCAAGTTCATTAAAGAGGCTGTCTGAGG
TP8998_Query	D	1	chr7	.	CAGCACAAGATTTTATCCGGAGAAGTGGCACTTCATTCAAGTTCATTAAAGAGGCTGTCTGAGG
TP90088_Hit	D	1	chr7	.	CTGCTGATATGGGTGGGATGGGTTTTATCCAATCCAATCATTCTGTCATTATGTATAACAACAC
TP90088_Query	D	1	chr7	.	CTGCTGATATGGGTGGGATGGGTTTTATCCAATCCAATCACTCTGTCATTATGTATAACAACAC
TP90404_Hit	D+G	1	chr7	.	CTGCTGGAATGTTGGAAATGGTGAAAGCTTCCCTCCTCAGACCCCCCCGTGCTCATGCGATCAA
TP90404_Query	D+G	1	chr7	.	CTGCTGGAATGTTGGAAATGATGAAAGCTTCCCTCCTCAGACCCCCCCGTGCTCATGCGATCAA
TP9047_Hit	D	1	chr7	.	CAGCACAAGGCCAATTCCTAGTCTTTGTAGCATTGTGATCCCTCTGGGGTTCTTTGTGTACCGC
TP9047_Query	D	1	chr7	.	CAGCACAAGGCCAATTCCTAGTCTTTGTAGCATCGTGATCCCTCTGGGGTTCTTTGTGTACCGC
TP90474_Hit	D+G	1	chr7	.	CTGCTGGAGCATAATCATAGCCACCGTCGTCATCGTCGTCTCCTTCGTCGCAAACTGTAGGAGG
TP90474_Query	D+G	1	chr7	.	CTGCTGGAGCATAATCATAGCCACCGTCGTCATCATCGTCTCCTTCGTCGCAAACTGTAGGAGG
TP90481_Hit	D+G	1	chr7	.	CTGCTGGAGCTGATGATGATGATCTTTACAATTAGGTCAAACTTGTATGTGTGTAGTTCATTTT
TP90481_Query	D+G	1	chr7	.	CTGCTGGAGCTGATGATGACGATCTTTACAATTAGGTCAAACTTGTATGTGTGTAGTTCATTTT
TP9049_Hit	D+G	1	chr7	.	CAGCACAAGGGAACCCTCTTTGTTTAGTTCTGTCACCGAGGAGTGAGGAGCTTCCGAACTAATT
TP9049_Query	D+G	1	chr7	.	CAGCACAAGGGAACCCTCTCTGTTTAGTTCTGTCACCGAGGAGTGAGGAGCTTCCGAACTAATT
TP90594_Hit	D	1	chr7	.	CTGCTGGCAGAGGCAATGGCAGTGGCAGAGCCTCCACATTAGGTTCTTTACTGGAAGGTAGGCA
TP90594_Query	D	1	chr7	.	CTGCTGGCAGAGGCAATGGCAGTGGCAGAGCCTCCACATTAGGTTCTTTACTGGAAGGCAGGCA
TP91146_Hit	D+G	1	chr7	.	CTGCTGGTGGTGGCCATTCAGCTGTGTTGACTGACGCTTTTTCGTTAAAGGAGTTGTGTGAGTT
TP91146_Query	D+G	1	chr7	.	CTGCTGGTGGCGGCCATTCAGCTGTGTTGACTGACGCTTTTTCGTTAAAGGAGTTGTGTGAGTT
TP91238_Hit	D	1	chr7	.	CTGCTGGTTAAGGTATTATTTCTTTCTGCTAAAAACACTGTTTCTCCCCACTATGCACTTATTC
TP91238_Query	D	1	chr7	.	CTGCTGGTTAAGGTATTATTTCTTTCTGCTAATAACACTGTTTCTCCCCACTATGCACTTATTC
TP91692_Hit	D	1	chr7	.	CTGCTGTCCGGTAATTTTGGACATTCCTATTGTTGTATGCGGCATAGAAGACTGATTTCTTATC
TP91692_Query	D	1	chr7	.	CTGCTGTCCCGTAATTTTGGACATTCCTATTGTTGTATGCGGCATAGAAGACTGATTTCTTATC
TP91797_Hit	D	1	chr7	.	CTGCTGTCTGTGTGCTTGTGAGTTGCGACGTGTTTGGAAGTGAATTTGTGAATGAGGTTGGTGA
TP91797_Query	D	1	chr7	.	CTGCTGTCTGTGTGCTTGTGAGTTGCGACGTGTTTGGAAGAGAATTTGTGAATGAGGTTGGTGA

Name	Filter	Nb hit (Mt4.0)	Mt Chr (Mt4.0)	Ms Chr (Li et al., 2014)	Sequence
TP9184_Hit	D	1	chr7	.	CAGCACAATTCAGATCTTGACGTTCAGTATCCTTTAACGGACCCAAATCGGTGAAGGAAGTGTT
TP9184_Query	D	1	chr7	.	CAGCACAATTCAGATCTTGACGTTCAGTATCCTTTAACGGACCCAAATCGGTGAAGGAAGGGTT
TP92007_Hit	D	1	chr7	.	CTGCTGTGGTACCTACTAAACTTTTCTTGGTAGAAAGTACAGTTGTTTTAAACTATAAATGGGT
TP92007_Query	D	1	chr7	.	CTGCTGTGGTACCTACTAAACTTTTCTTGGTAGAAAATACAGTTGTTTTAAACTATAAATGGGT
TP92215_Hit	D	1	chr7	.	CTGCTGTTCATAGAGCGCAGTCTGAGGCTCAGGAGGAAATAAAACGACTCTCAGGTGCGGAGAT
TP92215_Query	D	1	chr7	.	CTGCTGTTCATAGAGCGCAGTCTGAGGCTCAGGAGGAAATAAAACGACTCTCAGGTGCCGAGAT
TP92233_Hit	D	1	chr7	.	CTGCTGTTCCGAGAGTCGATCCTTGGGACCTTTCACTTCAACAAGCTTAGCTTCACCACTGTAT
TP92233_Query	D	1	chr7	.	CTGCTGTTCCGAGAGTCGATCCTTGGGACCTTTCACTTCAACAAGCTTAGCGTCACCACTGTAT
TP923_Hit	D	1	chr7	.	CAGCAAAATCTACCTGCATTTGTTCTGATGGAATACCTTCTCTAGGAGGCAAACTGGTTAAGAC
TP923_Query	D	1	chr7	.	CAGCAAAATCTACCTGCATTTGTTCTGATGGAATACCTTCTCTAGGAGGCAAACTGGTCAAGAC
TP92422_Hit	D	1	chr7	.	CTGCTGTTGGAGATGGGTTTATAGCTCAGGACATTAGGTTCCAAAACACGGCAGGCCCAGAGAA
TP92422_Query	D	1	chr7	.	CTGCTGTTGGAGATGGGTTTATAGCTCAGGACATTAGGTTCCAAAACACAGCAGGCCCAGAGAA
TP92686_Hit	D+G	1	chr7	.	CTGCTGTTTTTGTACCTTTTTATGCTGGATTTGACATTGCACGTTATCTTTGGGGGTATAATAT
TP92686_Query	D+G	1	chr7	.	CTGCTGTTTTTGTACCTTTTTATGCTGGATTTGACATTGCACGGTATCTTTGGGGGTATAATAT
TP92711_Hit	D	1	chr7	.	CTGCTTAAACACGATGGTGGAGCAAATTCATGGCCTGCTGATGGGTAATGGAAGTTTGGTAAGC
TP92711_Query	D	1	chr7	.	CTGCTTAAACACGACGGTGGAGCAAATTCATGGCCTGCTGATGGGTAATGGAAGTTTGGTAAGC
TP92754_Hit	D+G	1	chr7	.	CTGCTTAAGACTGATCTTGCGTACTACTACTGTTACCTTCACCGGCACGATTTTGCTCCCCAAC
TP92754_Query	D+G	1	chr7	.	CTGCTTAAGACTGATCTCGCGTACTACTACTGTTACCTTCACCGGCACGATTTTGCTCCCCAAC
TP92805_Hit	D	1	chr7	.	CTGCTTACAAGAGCATGCAAGAGAAAGCGGCAAAAAGACAGGAAAATAGCACTGATAATGACGA
TP92805_Query	D	1	chr7	.	CTGCTTACAAGAGCATGAAAGAGAAAGCGGCAAAAAGACAGGAAAATAGCACTGATAATGACGA
TP92816_Hit	D+G	1	chr7	.	CTGCTTACAGAAAAACCGGGGTAATGCTAAATTGTCCATTCTTTCTGTTTTGACTTTTCATACT
TP92816_Query	D+G	1	chr7	.	CTGCTTACAGAAAAACCGGGGTAATGCTAAATTGTCCATTCTTTCTGTTTTCACTTTTCATACT
TP93088_Hit	D	1	chr7	.	CTGCTTATTCCAAATTGGCATCACTAACAGGGTACCCTGAAGACACACGTTATGAAGTTTTTAA
TP93088_Query	D	1	chr7	.	CTGCTTATTCCAAATTGGCATCACTAACAGGGTACCCTGAAGACACACGTTATGAAATTTTTAA
TP93403_Hit	D+G	1	chr7	.	CTGCTTCATTTTATACTTCATATTCTGGTTTTGATTTGTCGGGAAACTAGGAAGAGGTTTATGA
TP93403_Query	D+G	1	chr7	.	CTGCTTCATTTTATACTTCATATTCTGGTTTTGATTTGTCGGGAAACTACGAAGAGGTTTATGA
TP93600_Hit	D	1	chr7	.	CTGCTTCCTTTGGCTTTGTTTGTTGTTGGACAGTAGAAGTGGGACTCCAAGGATTCAGGCTCAT
TP93600_Query	D	1	chr7	.	CTGCTTCCTTTGGCTTTGTTTGTTGGTGGACAGTAGAAGTGGGACTCCAAGGATTCAGGCTCAT
TP9365_Hit	D	1	chr7	.	CAGCACACGTGTCTGTGCACATGCAGGAGAGGGAAAGAAGTCAAAGAAGAGAGGGCTTACATTA
TP9365_Query	D	1	chr7	.	CAGCACACGTGTCTGTGCACATGCAGGAGAGGGAAAGAAGTCAAAGAAGAGAGGGCTTACATGA
TP93720_Hit	D	1	chr7	.	CTGCTTCTAGTTCAGAAGATTGGATCTGGTAACTTTCTTATGATATGAATATTTATCTGATGAT
TP93720_Query	D	1	chr7	.	CTGCTTCTACTTCAGAAGATTGGATCTGGTAACTTTCTTATGATATGAATATTTATCTGATGAT
TP93967_Hit	D+G	1	chr7	.	CTGCTTCTTGAAGGCAATAATAAGAGGAAGGTTTTAGAAAGACTTGATTCAGATGCTCAAAAGT
TP93967_Query	D+G	1	chr7	.	CTGCTTCTTGAAGGCAATAATAAGAGAAAGGTTTTAGAAAGACTTGATTCAGATGCTCAAAAGT
TP9435_Hit	D	1	chr7	.	CAGCACAGAGAGGATTGCCTGTATGCCGCCACCTCTTTCTCCATTACCCTAATGATGAACATGT
TP9435_Query	D	1	chr7	.	CAGCACAGAAAGGATTGCCTGTATGCCGCCACCTCTTTCTCCATTACCCTAATGATGAACATGT
TP9438_Hit	D+G	1	chr7	.	CAGCACAGAAATGTCCAAGATGTGAATCAACAAACACAAAGTTCTGTTACTACAACAACTACAA
TP9438_Query	D+G	1	chr7	.	CAGCACAGAAATGTCCAAGATGTGAATCAACAAACACAAAGTTCTGCTACTACAACAACTACAA
TP94486_Hit	D	1	chr7	.	CTGCTTGTAAAACTCATAATAATGTTGACATTGCAGAGCGAGGTGCAGGAAATATACTAAAGCT
TP94486_Query	D	1	chr7	.	CTGCTTGTAAAACTCATAATAATGTTGACATTGCAGAGCGAGGCGCAGGAAATATACTAAAGCT
TP94562_Hit	D	1	chr7	.	CTGCTTGTCGCGTTGTGCCAGCATTGACATCCTTGAGCCCATTGGAGAGATAGGTTCCACGTTT
TP94562_Query	D	1	chr7	.	CTGCTTGTCGCGTTGTGCCAGCATTGACATCCTTGAGCCCATCGGAGAGATAGGTTCCACGTTT
TP94803_Hit	D	1	chr7	.	CTGCTTTAGCCATAAAGGGTCATTCAGCTTACCTCAATTTCCCTAATTTGGCTCAAAATCTTCC
TP94803_Query	D	1	chr7	.	CTGCTTTAGCCATAAAGGGTCATTCAGCTTACCTAAATTTCCCTAATTTGGCTCAAAATCTTCC
TP94813_Hit	D	1	chr7	.	CTGCTTTCGCTTCCATTGTGGCCACAACAGCAACCTCGTCAACACAAATCAAGGATAAAATGAT
TP94813_Query	D	1	chr7	.	CTGCTTTAGCTTCCATTGTGGCCACAACAGCAACCTCGTCAACACAAATCAAGGATAAAATGAT
TP94879_Hit	D	1	chr7	.	CTGCTTTATTGTTTATAGAAGTTGTCCTTGACTGCTTTCAGCTATCTGTGCCAACTTTGCACCA
TP94879_Query	D	1	chr7	.	CTGCTTTATTGTTTATACAAGTTGTCCTTGACTGCTTTCAGCTATCTGTGCCAACTTTGCACCA
TP94888_Hit	D	1	chr7	.	CTGCTTTCAAACTCAATGTTGTTATGCTCTGGCCAAAGGTCTGACTCTGTTGGTGCCCTCCGTG
TP94888_Query	D	1	chr7	.	CTGCTTTCAAACTCAATGTTGTTATGCTCTGGCCAAAGGTCCGACTCTGTTGGTGCCCTCCGTG
TP94933_Hit	D+G	1	chr7	.	CTGCTTTCATCAAGATAAGTGGCTTAGCCACTTCTATTTTGTTGGATCACTTGTATTTTGCAAT
TP94933_Query	D+G	1	chr7	.	CTGCTTTCATCAAGATAAGTGGCTTAGCCACTTCTATTTTGTTGGATCACTTGCATTTTGCAAT
TP94975_Hit	D	1	chr7	.	CTGCTTTCCTAAGCAGTCAAACAAATTTTGTTCCTTTTTTGTTTCTTTTTGTTGTACTTGAGGA
TP94975_Query	D	1	chr7	.	CTGCTTTCCTAAGCAGTCAAACAAATTTTGTTCCCTTTTTGTTTCTTTTTGTTGTACTTGAGGA

Name	Filter	Nb hit (Mt4.0)	Mt Chr (Mt4.0)	Ms Chr (Li et al., 2014)	Sequence
TP95187_Hit	D	1	chr7	.	CTGCTTTGCAGTCAGACAGGTTTTTCAATGCTCTGAAGGGTCATGGTGCTCTAAGTCGGCTGGT
TP95187_Query	D	1	chr7	.	CTGCTTTGCAGTCAGACAGGTTTTTCAATGCTCTGAAGGGCCATGGTGCTCTAAGTCGGCTGGT
TP95244_Hit	D	1	chr7	.	CTGCTTTGGAATCACAAGTCGATTCAGCTTCCCTACGTCACTAGGTGTCACCGCTTTATCAAAC
TP95244_Query	D	1	chr7	.	CTGCTTTGGAATCACAAGTCGATTCAGCTTCCCTACGTCACTAGGTGTCACCACTTTATCAAAC
TP95290_Hit	D	1	chr7	.	CTGCTTTGGTAAGGCGGAGGTTGAGGCTGGGGTATGCGGTGTCTGCTGTCAGCTGTTTCCTGCT
TP95290_Query	D	1	chr7	.	CTGCTTTGGTAAGGCGGAGGTTGAGGCTGGGGTATGCGATGTCTGCTGTCAGCTGTTTCCTGCT
TP95301_Hit	D	1	chr7	.	CTGCTTTGGTGTTGGATATGGATGATGCGTTGTTGTTGATTGAGTATGGATTGGAGGGGATGGC
TP95301_Query	D	1	chr7	.	CTGCTTTGGTGTTGGATATGGATGATGCGTTGTTGTTGATTGAGTATGGATTGGAGGAGATGGC
TP95495_Hit	D	1	chr7	.	CTGCTTTTCTGTGCCGGTGACTGCATCATGGTGTTGAGCAATTGCTAAAGCATCTGCTAATGAG
TP95495_Query	D	1	chr7	.	CTGCTTTTCTGTGCCGGTGACTGCATCATGGTGTTGAGCAATTGCTAAAGCATCAGCTAATGAG
TP95652_Hit	D+G	1	chr7	.	CTGCTTTTGTTCCTTATAAACCAACAGCTAGAATTGGTTTTGATGTGCTCTTATCAAGTGACTA
TP95652_Query	D+G	1	chr7	.	CTGCTTTTGTTCCTTATAAACCAACAGCTAGAATTGGTTTCGATGTGCTCTTATCAAGTGACTA
TP95725_Hit	D	1	chr7	.	CTGCTTTTTGTAGTAAAGCGGTTGCCGACATTTGAGCCATGCCAGCAATTAGTCCTCCATTTTT
TP95725_Query	D	1	chr7	.	CTGCTTTTTGTAGTAAAGCAGTTGCCGACATTTGAGCCATGCCAGCAATTAGTCCTCCATTTTT
TP9604_Hit	D	1	chr7	.	CAGCACAGTGAAGAGCGGTGCTCTTGTCAAGGCCACAAGGCCTGTTGATATCAACATCCGATAA
TP9604_Query	D	1	chr7	.	CAGCACAGTGAAGAGCAGTGCTCTTGTCAAGGCCACAAGGCCTGTTGATATCAACATCCGATAA
TP9777_Hit	D	1	chr7	.	CAGCACATGAAACCACCAAATGACAAATCAATAAATGCATGTTATTGCTAATATAGAGAATGCC
TP9777_Query	D	1	chr7	.	CAGCACATGAAACCACCAAATGACAAATCAATAAATGCATGTTATTACTAATATAGAGAATGCC
TP9987_Hit	D+G	1	chr7	.	CAGCACCAAAAAGGGCAATTAGGGAGAGGACTGAAACTTGGCCTATAAATGTAGCCATTGCTTC
TP9987_Query	D+G	1	chr7	.	CAGCACCAAAAAGGACAATTAGGGAGAGGACTGAAACTTGGCCTATAAATGTAGCCATTGCTTC
TP11169_Hit	D	1	chr7	7A	CAGCACGACTTGTTCAGGACCTAAAACTGTAAGAAGATGGTTGAAGGAAAGAGGAAAGCAAAAT
TP11169_Query	D	1	chr7	7A	CAGCACGACTTGTTCAGGACCTAAAACTGTAAGAAGATGGTTGAAAGAAAGAGGAAAGCAAAAT
TP16776_Hit	D+G	1	chr7	7A	CAGCAGTTTAACATAAGTAGAATACCTTTTTTGGATCATTGTACCGAGGGAAGCGAGTCAGTTT
TP16776_Query	D+G	1	chr7	7A	CAGCAGTTTAACATAAGTAGAATACCTTTTTTGGATCATTATACCGAGGGAAGCGAGTCAGTTT
TP17479_Hit	D	1	chr7	7A	CAGCATACTATAAACTAGACCATGAAATAAAATCTACTCACATCTACCGGGTTGATTCCGACAT
TP17479_Query	D	1	chr7	7A	CAGCATACTATAAACTAGACCATGAAATAAAATCTACTCACATCTACCGGGTTGATTCCAACAT
TP1840_Hit	D	1	chr7	7A	CAGCAAAGGCAGTGGCCCTTGTCCTCCCATCACTTAAAGGCAAGCTCAACGGTATTGCACTTCG
TP1840_Query	D	1	chr7	7A	CAGCAAAGGCAGTGGCCCTTGTCCTCCCATCACTCAAAGGCAAGCTCAACGGTATTGCACTTCG
TP19604_Hit	D	1	chr7	7A	CAGCATCTTTCTTCCCCCTGAGAGTTCCAGAAGCTAGAAGTTCCACCAAAGCTTTAACAGCACC
TP19604_Query	D	1	chr7	7A	CAGCATCTTTCTTCCCCCTAAGAGTTCCAGAAGCTAGAAGTTCCACCAAAGCTTTAACAGCACC
TP22886_Hit	D+G	1	chr7	7A	CAGCCAAACCAAAGACAATAACAACATCTCTCAAAACATAACTCATTGACTTCCATGAATCTTT
TP22886_Query	D+G	1	chr7	7A	CAGCCAAACCAAAAACAATAACAACATCTCTCAAAACATAACTCATTGACTTCCATGAATCTTT
TP27564_Hit	D+G	1	chr7	7A	CAGCCATTTGTTAGCTTGCTACACGATGACATGAAGAGTAATCTGAATGATGACTTCAAGAGTA
TP27564_Query	D+G	1	chr7	7A	CAGCCATTTGTTAGCTTGCTACACGATGACATGAAGAGTAATCTGAATGATGACTTCAAGAATA
TP27582_Hit	D+G	1	chr7	7A	CAGCCATTTTCAAACGAAGTGTGAACCAACCCATCAAAAAGTTGTGCCTAAGTGTAGGGAGTTA
TP27582_Query	D+G	1	chr7	7A	CAGCCATTTTCAAACGAAGTGTAAACCAACCCATCAAAAAGTTGTGCCTAAGTGTAGGGAGTTA
TP54294_Hit	D	1	chr7	7A	CAGCTTATTGCTAATCAGATACCTTTGCCTTCCTCTGATTTAGAGTGGATCATGGGCAAGACTG
TP54294_Query	D	1	chr7	7A	CAGCTTATTGCTAATCAGATACCTTTGCCTTCTTCTGATTTAGAGTGGATCATGGGCAAGACTG
TP60774_Hit	D	1	chr7	7A	CTGCAAAAGGAATTACAACTGAAGAAATGGTGACCCTTTTGGGAGCACATACTGTTGGTGTTGC
TP60774_Query	D	1	chr7	7A	CTGCAAAAGGAATTACAACCGAAGAAATGGTGACCCTTTTGGGAGCACATACTGTTGGTGTTGC
TP69402_Hit	D	1	chr7	7A	CTGCAGGTGACTGATTATACGAGAGATCTCGAAGAAATGCAGAACGTTTCGAGAGAAGAATACC
TP69402_Query	D	1	chr7	7A	CTGCAGGTGACTGATTATACAAGAGATCTCGAAGAAATGCAGAACGTTTCGAGAGAAGAATACC
TP75693_Hit	D	1	chr7	7A	CTGCCATTTACGGATTTTCTCATCTTGCATTCAAAAGAGCAAGCCAACCATTTTGGATTCATAT
TP75693_Query	D	1	chr7	7A	CTGCCATTTACGGATTTTCTCATCTTGCATTCAAAAGAACAAGCCAACCATTTTGGATTCATAT
TP76164_Hit	D	1	chr7	7A	CTGCCCCACTTTTATTCTTGAAGTCATCATTCAGATTACTCTTCATGTCATCGTGTAGCAAGCT
TP76164_Query	D	1	chr7	7A	CTGCCCCACTTTTACTCTTGAAGTCATCATTCAGATTACTCTTCATGTCATCGTGTAGCAAGCT
TP86390_Hit	D	1	chr7	7A	CTGCTATGACACATTTTATGTTCATGAAACCTAAATCTGTGTTCATTCAAGTTGTTCCTCTTGG
TP86390_Query	D	1	chr7	7A	CTGCTATGACACATTTTATGTTCATGAAACCTAAATCTGTGTTCATTCAAGTTGTTCCGCTTGG
TP89882_Hit	D	1	chr7	7A	CTGCTGAGCATTATGTATTTTGCTTTCCTCTTTCTTTCAACCATCTTCTTACAGTTTTAGGTCC
TP89882_Query	D	1	chr7	7A	CTGCTGAGCATTATGTATTTTGCTTTCCTCTTTCCTTCAACCATCTTCTTACAGTTTTAGGTCC
TP18516_Hit	D+G	1	chr7	7B	CAGCATCCCCTTTTGAACACATAACTATCAGGCGACCTCTACGGGCATGAAGTTGCTGAATAAC
TP18516_Query	D+G	1	chr7	7B	CAGCATCACCTTTTGAACACATAACTATCAGGCGACCTCTACGGGCATGAAGTTGCTGAATAAC
TP19483_Hit	D	1	chr7	7B	CAGCATCTGTCTTTTTTCTGCAGAATTCGGCTGTTGAAGTGTTGTTTAAATCTCTATCGTTAGT
TP19483_Query	D	1	chr7	7B	CAGCATCTGTCTTTTTTCGGCAGAATTCGGCTGTTGAAGTGTTGTTTAAATCTCTATCGTTAGT

Name	Filter	Nb hit (Mt4.0)	Mt Chr (Mt4.0)	Ms Chr (Li et al., 2014)	Sequence
TP19593_Hit	D	1	chr7	7B	CAGCATCTTTAAGAAGCAATGCAGACGTAATTTCTGTCACTGTAGTCGGGCAATTGTTCATGGT
TP19593_Query	D	1	chr7	7B	CAGCATCTTTAAGAAGCAATGCAGAAGTAATTTCTGTCACTGTAGTCGGGCAATTGTTCATGGT
TP29006_Hit	D	1	chr7	7B	CAGCCCGATCGGGTTGTGGTTGCTAATGGACCTGCATTTGGATGCATTTTGATGAAGGATTTCT
TP29006_Query	D	1	chr7	7B	CAGCCCGATCGGGTTGTGGTTGCTAATGGACCTGCATTTGGATGCATTTTGATGAAGGATTTCC
TP41590_Hit	D	1	chr7	7B	CAGCGTTTGAGGAACCTCTTCCTTTTCTAGATAATACAGAAGTTCCTTTTGAAGGAGCTTTTGT
TP41590_Query	D	1	chr7	7B	CAGCGTTTGAGGAACCTCTTCCTTTTCTAGAGAATACAGAAGTTCCTTTTGAAGGAGCTTTTGT
TP42497_Hit	D	1	chr7	7B	CAGCTAAGTCGATTGGAGAAATTGAGCTTGAGGCAAAGTTTGAAGAGGCAGTGTCCAAAATCAA
TP42497_Query	D	1	chr7	7B	CAGCTAAGTCAATTGGAGAAATTGAGCTTGAGGCAAAGTTTGAAGAGGCAGTGTCCAAAATCAA
TP4775_Hit	D+G	1	chr7	7B	CAGCAACTGTAAAATGAAATGTGTGTATTGGCTATTTGCAAATACCAAATAAATGGCGTGCTAG
TP4775_Query	D+G	1	chr7	7B	CAGCAACTGTAAAATGAAATGTATGTATTGGCTATTTGCAAATACCAAATAAATGGCGTGCTAG
TP49861_Hit	D+G	1	chr7	7B	CAGCTGAAATGGAAAGTGGGTTTATAGCGAGAAAATCAGTCATAGTGAGGGAGAGCTGAACACC
TP49861_Query	D+G	1	chr7	7B	CAGCTGAAATGGAAAGTGGGTTTATAGCGAGAAAATCAGTCATAGTGAAGGAGAGCTGAACACC
TP49989_Hit	D	1	chr7	7B	CAGCTGAAGAGTTGATCAGCGCATTTTCATCATTTATATTTTCGTTTGCCATGGGTAGAGCTTC
TP49989_Query	D	1	chr7	7B	CAGCTGAAGAGTTGATCAGCGCATTTTCATCATTTATATTTTCGTCTGCCATGGGTAGAGCTTC
TP52024_Hit	D	1	chr7	7B	CAGCTGTAGAAGGTGGACAAATCAGCTCCGTTTCTTACCGCGACTGCTTGGTGTTTGCTTGGTC
TP52024_Query	D	1	chr7	7B	CAGCTGTAAAAGGTGGACAAATCAGCTCCGTTTCTTACCGCGACTGCTTGGTGTTTGCTTGGTC
TP52182_Hit	D+G	1	chr7	7B	CAGCTGTAGCTCCAACAAGCTCCTTTCTTTCTTTTCTCCAACCATCCCACAATGTACGATCAAT
TP52182_Query	D+G	1	chr7	7B	CAGCTGTAGCTCCAACAAGCTCCTTTCTTTCCTTTCTCCAACCATCCCACAATGTACGATCAAT
TP522_Hit	D	1	chr7	7B	CAGCAAAATTTGATAGTCTCTAATGTTATGGAGAAAATAGCAACAGATTCTGAGTATTTTCTTA
TP522_Query	D	1	chr7	7B	CAGCAAAACTTGATAGTCTCTAATGTTATGGAGAAAATAGCAACAGATTCTGAGTATTTTCTTA
TP8578_Hit	D+G	1	chr7	7B	CAGCAATTTCTCTAGCACTGTTTTGTTTAGCCTCAAGCATTTTAACAAGGAGAGGAATGCAACC
TP8578_Query	D+G	1	chr7	7B	CAGCAATTTCTCTAGCACTGTTTTGTTTAGCCTCAAGCATCTTAACAAGGAGAGGAATGCAACC
TP88842_Hit	D	1	chr7	7B	CTGCTCTCATATCACTTGTCATATACTTGGCTCTCTCCTCCAATGGAAGATCGAAGAATCTTCC
TP88842_Query	D	1	chr7	7B	CTGCTCTCATATCACTTGTCATATACTTAGCTCTCTCCTCCAATGGAAGATCGAAGAATCTTCC
TP18758_Hit	D	1	chr7	7C	CAGCATCATCTACAAGCAACCACACAGCCGATCAGGGTACACAAAGATAAGAACATTCATTGGA
TP18758_Query	D	1	chr7	7C	CAGCATCATCTACAAGCAACCACACACCCGATCAGGGTACACAAAGATAAGAACATTCATTGGA
TP20326_Hit	D	1	chr7	7C	CAGCATGGACTGGAGCACAAATTAAAGTGCCAGTAAAGTTCATAACTGGTGATTCAGATTTAGT
TP20326_Query	D	1	chr7	7C	CAGCATGGACTGGAGCACAAATTAAAGTGCCAGTAAAGTTCATAACTGGTGAGTCAGATTTAGT
TP27350_Hit	D+G	1	chr7	7C	CAGCCATTCGTACGTACCTTCTTGAGAGATCTCGGGTTTGCCAAGTTAATGACCCAGAACGTAA
TP27350_Query	D+G	1	chr7	7C	CAGCCATTCGTACGTACCTTCTCGAGAGATCTCGGGTTTGCCAAGTTAATGACCCAGAACGTAA
TP29020_Hit	D	1	chr7	7C	CAGCCCGCAATCGACGGTCAGCCTTTCAAATGCAAATCAATGCAATGTAAGGAAGGACGAACGG
TP29020_Query	D	1	chr7	7C	CAGCCCGCAATCGACGGTCAACCTTTCAAATGCAAATCAATGCAATGTAAGGAAGGACGAACGG
TP34644_Hit	D	1	chr7	7C	CAGCCTTGCGTTTGTTGCTGGCATGTTATCCTGAAACCTTTCCCCCTTGACATTTGCTTCAGGA
TP34644_Query	D	1	chr7	7C	CAGCCTTGCGTTTGTTGCTGGCATGTTATCCTGAAACCTTTCCCCCTTGACATTTGCTCCAGGA
TP43370_Hit	D+G	1	chr7	7C	CAGCTACCTTGGTTTGCTTCTCAATGCTCAGAACGTTCATGATGACACAAAAGCATGGCGATGC
TP43370_Query	D+G	1	chr7	7C	CAGCTACCTTGGTTTGCTTCTCAATGCTCAGAACGTTCATGATGACACAAAAGCATGGCAATGC
TP43372_Hit	D	1	chr7	7C	CAGCTACCTTGTGAAATAGATAAAGAATTAAATTCCAAAATAACAATTATCCAAGTACATAAAA
TP43372_Query	D	1	chr7	7C	CAGCTACCTTGTGAAATAGATAAAGAATTAAATTCCAAAATAACAATTATCCAAGTACACAAAA
TP4803_Hit	D+G	1	chr7	7C	CAGCAACTGTGGCAACTGGGATTATAGCTAGGAGTTCCACATCCTCCAAGGAAATGACTTCTAG
TP4803_Query	D+G	1	chr7	7C	CAGCAACTGTGGCAACTGGGATTATAGCTAGGAGTTCCACATCCTCCAAGGAAATCACTTCTAG
TP48932_Hit	D+G	1	chr7	7C	CAGCTCTCGAATGGTTCTCAGTGCAAGGTTCCTACCTATTTGGGTGGTATTTCTGTCTGTTTCT
TP48932_Query	D+G	1	chr7	7C	CAGCTCTCGAATGGTTCTCAGTGCAAGGTTCCTACCTATTTGGGCGGTATTTCTGTCTGTTTCT
TP59148_Hit	D	1	chr7	7C	CAGCTTTCGTGTTCAGAGACTGATACTTAGAATTTAATGCCTCGGCCTTAGTGATTGCCATGCA
TP59148_Query	D	1	chr7	7C	CAGCTTTCGTGTTCAGAGACCGATACTTAGAATTTAATGCCTCGGCCTTAGTGATTGCCATGCA
TP61236_Hit	D	1	chr7	7C	CTGCAAAGACGATGTCCCTCTTGATTTTGGACACTGCCTCTTCAAACTTTGCCTCAAGCTCAAT
TP61236_Query	D	1	chr7	7C	CTGCAAAGACAATGTCCCTCTTGATTTTGGACACTGCCTCTTCAAACTTTGCCTCAAGCTCAAT
TP83639_Hit	D	1	chr7	7C	CTGCGTGTAGATCTCATTTGAGTACCTCATAAACTCCTAATGTGTGTCTGTTTGTTTGATTTAG
TP83639_Query	D	1	chr7	7C	CTGCGTGTAGATCTCATTTGAGTACCTCATAAACTCCTAATGTGTGTCTGTTTGTTTGACTTAG
TP83827_Hit	D	1	chr7	7C	CTGCGTTCTTCTAGTCCACAACCTTTACCGCTTCCTTCTCCTCAGGGAGGTAGTTCACTGAAAG
TP83827_Query	D	1	chr7	7C	CTGCGTTCTTCTAGTCCACAACCTTTACCACTTCCTTCTCCTCAGGGAGGTAGTTCACTGAAAG
TP87313_Hit	D	1	chr7	7C	CTGCTCAGAACTCCCAGCCACAACCAGGCAAGCCATTGGCACTGGAAGCACCTCCTACATCCAC
TP87313_Query	D	1	chr7	7C	CTGCTCAGAACTCCCAGCCACAACCAGGCAAGCCATTGGCACTGGAAGCACCTCCTACAACCAC
TP87710_Hit	D	1	chr7	7C	CTGCTCCAGAGCGTTCCACATTACTATTATCAGACTTAAGAGTTTCAAACAACCATGGGACAAG
TP87710_Query	D	1	chr7	7C	CTGCTCCAGAGCGTTCAACATTACTATTATCAGACTTAAGAGTTTCAAACAACCATGGGACAAG

Name	Filter	Nb hit (Mt4.0)	Mt Chr (Mt4.0)	Ms Chr (Li et al., 2014)	Sequence
TP95488_Hit	D+G	1	chr7	7C	CTGCTTTTTTGAGTTAAACTTCCATCCACATCATGCTTTCTCCTACTGCTTTTGTGGTCATTTT
TP95488_Query	D+G	1	chr7	7C	CTGCTTTTCTGAGTTAAACTTCCATCCACATCATGCTTTCTCCTACTGCTTTTGTGGTCATTTT
TP14317_Hit	D	1	chr7	7D	CAGCAGATTGAGCAGGCCCTGATTTTTTTGCTTGTTGAGCTCGTCTCTGCTTTTCCTACAATAG
TP14317_Query	D	1	chr7	7D	CAGCAGATTGAGCAGGCCCTGATTTTTTTGCTTGTTGAGCTCGCCTCTGCTTTTCCTACAATAG
TP18511_Hit	D	1	chr7	7D	CAGCATCACCTTCAATTGTTTGTTCTTGCATACTTGTCTTGGGTCAAATATATCATTGTAATGA
TP18511_Query	D	1	chr7	7D	CAGCATCACCTTCAATTGTTTGTTCTTGCATACTTGTCCTGGGTCAAATATATCATTGTAATGA
TP19506_Hit	D	1	chr7	7D	CAGCATCTTACGGAATTGTGAAAATACAACTGATTTTGTAGCTGGATTTTGATCCCTTGATTCC
TP19506_Query	D	1	chr7	7D	CAGCATCTTACGGAATTGAGAAAATACAACTGATTTTGTAGCTGGATTTTGATCCCTTGATTCC
TP19978_Hit	D	1	chr7	7D	CAGCATGATGGTGAAAATTGTGATGATAATCACTGCTAGAGGCGAGGAACCTGGATGGATTCCT
TP19978_Query	D	1	chr7	7D	CAGCATGATGGTGAAAATTGTGATGATAATCACTGCTAGAGGCGAGGAACCTGGATGGATTCCG
TP41996_Hit	D	1	chr7	7D	CAGCTAAATTTTGATCAACTCCAAGAAAACCCATTTGAGAAGAAACTGAAAACAAACCATCCAT
TP41996_Query	D	1	chr7	7D	CAGCTAAAGTTTGATCAACTCCAAGAAAACCCATTTGAGAAGAAACTGAAAACAAACCATCCAT
TP48442_Hit	D	1	chr7	7D	CAGCTCGTCCAAGATAAGCTATCCTTCCAGAGCATCTTGACAGAGCTAAAATTATCTTGTAAGC
TP48442_Query	D	1	chr7	7D	CAGCTCGTCAAAGATAAGCTATCCTTCCAGAGCATCTTGACAGAGCTAAAATTATCTTGTAAGC
TP50478_Hit	D+G	1	chr7	7D	CAGCTGAGAGTGCTGTGCAATTATTTTCAAAATTAGAAAAAAGTTACGGAGAAGAGGTTTCAAG
TP50478_Query	D+G	1	chr7	7D	CAGCTGAGAGTGCTGTGCAATTATTTTCAAAATTAAAAAAAAGTTACGGAGAAGAGGTTTCAAG
TP58383_Hit	D	1	chr7	7D	CAGCTTGTTGTTCACGTAAAAACTTTTGGAAAACCCATTCTGAATCACTCCGATTCATCTTTGT
TP58383_Query	D	1	chr7	7D	CAGCTTGTTGTTCACGTAAAAACTTTTGGAAAGCCCATTCTGAATCACTCCGATTCATCTTTGT
TP63701_Hit	D+G	1	chr7	7D	CTGCAAGTTTGACCATCCAATGGGAATCTTCACCTATAATGTATCTGCATCACCTTTAGCTGAA
TP63701_Query	D+G	1	chr7	7D	CTGCAAGTTTGACCATCCAATGGGAATCTTCACCTATAATGTATCCGCATCACCTTTAGCTGAA
TP64278_Hit	D+G	1	chr7	7D	CTGCAATGAGGTAGAAAAACCTGTCCATGTGCCCAACATTCAAGTTATTCGGAATCCATCCAGG
TP64278_Query	D+G	1	chr7	7D	CTGCAATGAGGTAGAAAAACCTGTCCATGTGCCCAACATTCAAGTTATTAGGAATCCATCCAGG
TP83152_Hit	D	1	chr7	7D	CTGCGTATCTGATGAAACCAATTGAGTCAGGATCTGTAGATTTCCCCGAGTTTGAACGGGGGCT
TP83152_Query	D	1	chr7	7D	CTGCGTATCTGATGAAACCAATTGAGTCAGGATCCGTAGATTTCCCCGAGTTTGAACGGGGGCT
TP95117_Hit	D	1	chr7	7D	CTGCTTTGAACATTGAACCAAGAACACCTGGTTTCTGTTCTTCTTGTTGATACCCTGTAGCATT
TP95117_Query	D	1	chr7	7D	CTGCTTTGAACATTGAACCAAGAACACCTGGTTTCTGTTCTTCTTGTTGATACCCTGAAGCATT
TP7937_Hit	D	1	chr7	8C	CAGCAATGGTGCATGAGCCACATCCACCTCCAGAGATTTTTTCTGCAACTCATCTGGAGGCCGG
TP7937_Query	D	1	chr7	8C	CAGCAATGGTGCATGAGCCACATCCACCTCCAGAGATTTTTTCTGCAACTCATCTGGAGGCCAG
TP10034_Hit	D	1	chr8	.	CAGCACCAAATTATGCTCTAACAAAACATCTCGACAAGTTTTCCACAAGAATAGATTACTCAAA
TP10034_Query	D	1	chr8	.	CAGCACCAAATTATGCTCTAACAAAACATCTCGAAAAGTTTTCCACAAGAATAGATTACTCAAA
TP10047_Hit	D	1	chr8	.	CAGCACCAACAAGGCAGAAGAGATTGTGACGATAATGGATGGTGTCAGCATCCCCACCACTGAC
TP10047_Query	D	1	chr8	.	CAGCACCAACAAGGCAGAAGAGATTGCGACGATAATGGATGGTGTCAGCATCCCCACCACTGAC
TP10217_Hit	D	1	chr8	.	CAGCACCACCACCGCTTTCAGCCACCGCAAGGGAGTTTCTGCAAACACACAACCAAGCAAGAGC
TP10217_Query	D	1	chr8	.	CAGCACCACCACCGCTTTCAGCCACCGCAAGGGAGTTTCTACAAACACACAACCAAGCAAGAGC
TP10251_Hit	D	1	chr8	.	CAGCACCACCTGTTTCCAATCAAGATCCGCAAACAAGTGACTTGTTTCCCATTCAGAGTCGCTA
TP10251_Query	D	1	chr8	.	CAGCACCACCTGTTTCCAATCAAGATCCGCAAACAAATGACTTGTTTCCCATTCAGAGTCGCTA
TP10491_Hit	D	1	chr8	.	CAGCACCATTTTCAACAGATAATATACCTGTGTTTCTATTCATATCTGTTTTAACAAAACCCGG
TP10491_Query	D	1	chr8	.	CAGCACCATTTTCAACAGATAATATACCTGTGTTTCTATTCATATCTGTTTTAACAAAACCAGG
TP10527_Hit	D	1	chr8	.	CAGCACCCACGGATCAGCAGAAGATTGTGTTGGATCATCCAGCAAATGTTCATGGTGCGAGGGT
TP10527_Query	D	1	chr8	.	CAGCACCCACGGATCAGCAGAAGATTGCGTTGGATCATCCAGCAAATGTTCATGGTGCGAGGGT
TP10598_Hit	D	1	chr8	.	CAGCACCGGGTGGATACATTCACATTCCAAGGAGGAGAATTTTGAAGAATCTTGAAATCAATGG
TP10598_Query	D	1	chr8	.	CAGCACCCGGTGGATACATTCACATTCCAAGGAGGAGAATTTTGAAGAATCTTGAAATCAATGG
TP1064_Hit	D	1	chr8	.	CAGCAAAATTTGAATATAGCTGTGTAGAGGAGGTGCCTCTTGAACCCCGCATTCATTAACAAAG
TP1064_Query	D	1	chr8	.	CAGCAAAATTTGAATATAGCTGTGTAGAGGAGGTGCCTCTTGAACCCCACATTCATTAACAAAG
TP10705_Hit	D+G	1	chr8	.	CAGCACCGATCTGAGCAGAAACCATAGATGATGTTCCCTGAGGACCAGAATGCTGTGCTAGATT
TP10705_Query	D+G	1	chr8	.	CAGCACCGATCTGAGCAGAAACCATAGATGATGGTCCCTGAGGACCAGAATGCTGTGCTAGATT
TP10811_Hit	D	1	chr8	.	CAGCACCGTCACACCAAAACAAATGTGAAAGCCTTCCTTTATCATCAGCCTTATGCCGCCAAAA
TP10811_Query	D	1	chr8	.	CAGCACCGTCACACCAAAACAAATGTGAAAGCCTTCCTTTATCATCAACCTTATGCCGCCAAAA
TP10867_Hit	D	1	chr8	.	CAGCACCTAGCATTTCTATGACTTCAAAAGGGGACCAAATTCCTCATAAAGATTTCTTCGGTAT
TP10867_Query	D	1	chr8	.	CAGCACCTAGCATTTCTATGACTTCAAAAGGGGACCAAATTCCTCATAAAGACTTCTTCGGTAT
TP10874_Hit	D	1	chr8	.	CAGCACCTATCAATATCCACCTGTTTTTACCTGCATTGAATATACACAATAATTTTAATTGTGA
TP10874_Query	D	1	chr8	.	CAGCACCTATCAATATCCACCTGTTTTTACCTGCATTGAATATACACAACAATTTTAATTGTGA
TP10921_Hit	D	1	chr8	.	CAGCACCTCGAGCCATGGAGAAATGTAAAAGTCGCAACTAGTGCGATGCAATGTGCTAGCTGTA
TP10921_Query	D	1	chr8	.	CAGCACCTCGAGCCATGGAGAAATGTAAAAGTCGCAACTAGTGCGATGCAATGTGCTAGCTGAA

Name	Filter	Nb hit (Mt4.0)	Mt Chr (Mt4.0)	Ms Chr (Li et al., 2014)	Sequence
TP11004_Hit	D	1	chr8	.	CAGCACCTGTCACACACACCACTTTTCCTTCCCCACTCATAATCCTTTTTCTCTTATTCCTTTA
TP11004_Query	D	1	chr8	.	CAGCACCTGTCACACACACCACTTTTCCTTCCCCACTCATAATCCTTTTTCTCTTACTCCTTTA
TP11094_Hit	D	1	chr8	.	CAGCACCTTTGAAATCTGTCAAAGATGCTGTGTCCAAGTTTGGAGGAATAGTGGATTGGAAAAC
TP11094_Query	D	1	chr8	.	CAGCACCTTTGAAATCTGTCAAAGATGCTGTGTCCAAGTTTGGAGGAATAGTGGACTGGAAAAC
TP11134_Hit	D	1	chr8	.	CAGCTCGAAGATTTGAATCTTCAGGTGGAAGGAGCTTGCACAATGACAAAGCTTCTTCGAAGTT
TP11134_Query	D	1	chr8	.	CAGCACGAAGATTTGAATCTTCAGGTGGAAGGAGCTTGCACAATGACAAAGCTTCTTCGAAGTT
TP11184_Hit	D	1	chr8	.	CAGCACGAGCTGTACTTCTAGGGAGATCAGAGTAGTCAGATCCACGAGGTGGATAATCCCTATA
TP11184_Query	D	1	chr8	.	CAGCACGAGCAGTACTTCTAGGGAGATCAGAGTAGTCAGATCCACGAGGTGGATAATCCCTATA
TP11441_Hit	D	1	chr8	.	CAGCACGGACCCACATGTAGACACATTCTCCGGGAAAACTGTAAACATCGGAGGTGCGGAGATC
TP11441_Query	D	1	chr8	.	CAGCACGGACCCACATGTAGACACATTCTCCGGGAAAACTGTAAACATCGGAGGTGCCGAGATC
TP11781_Hit	D	1	chr8	.	CAGCACTACAGGGTAGTGAAAGTTGAATAAACTTCTATTTTCTGCAATCCATGGTCGACAACAC
TP11781_Query	D	1	chr8	.	CAGCACTACAGGGTAGTGAAAATTGAATAAACTTCTATTTTCTGCAATCCATGGTCGACAACAC
TP11946_Hit	D	1	chr8	.	CAGCTCTATGCAAGCCTTGGTCTTGATGATATCATTTATTTAAATCCTGTTTTTGTAAACCACT
TP11946_Query	D	1	chr8	.	CAGCACTATGCAAGCCTTGGTCTTGATGATATCATTTATTTAAATCCTGTTTTTGTAAACCACT
TP1244_Hit	D	1	chr8	.	CAGCAAACATAGGCAATGACATTTGAAGTCGAAGTATTATGTAGTCTAATCTGCCTTGAATATT
TP1244_Query	D	1	chr8	.	CAGCAAACATAGGCAATGACATTTGAAGTCGAAGTATTATGCAGTCTAATCTGCCTTGAATATT
TP12447_Hit	D	1	chr8	.	CAGCACTTACTGTTGCTTCTTCACCATAAGGTCCACCAGATACTTCAAAGTAGATGAGAAAGAT
TP12447_Query	D	1	chr8	.	CAGCACTTACTGTTGCTTCTTCACCATAAGGTCCACCAGATACCTCAAAGTAGATGAGAAAGAT
TP12527_Hit	D	1	chr8	.	CAGCACTTCGTTTGGATGATGGTGATAAAGATGATATTGAGAAAACATTAAGAGTTGCATTGCT
TP12527_Query	D	1	chr8	.	CAGCACTTCGTTTGGATGATGGTGATAAAGATGATATTGAGAAAACATTAAAAGTTGCATTGCT
TP12573_Hit	D	1	chr8	.	CAGCACTTGACTTTGTTCAGGTCAATACTCTTCAATTGTCCTTTTCAAAATTAATGTATAACAC
TP12573_Query	D	1	chr8	.	CAGCACTTGACTTTGTTCAGGTCAATACTCTTTAATTGTCCTTTTCAAAATTAATGTATAACAC
TP12576_Hit	D	1	chr8	.	CAGCTCTTGAGACTTGGGATAATGGGAAACCATATGAACAATCTGCTCAGATTGAAATTCCAAT
TP12576_Query	D	1	chr8	.	CAGCACTTGAGACTTGGGATAATGGGAAACCATATGAACAATCTGCTCAGATTGAAATTCCAAT
TP1258_Hit	D	1	chr8	.	CAGCAAACATCCACGCCGCAACAAACACCCAGGGAGAAGAAAAATGCAAGTCGGTGCGGAGATC
TP1258_Query	D	1	chr8	.	CAGCAAACATCCACGCCGCAACAAACACCCAGGGAGAAGAAAAATGCAAGTCGGTGCCGAGATC
TP12605_Hit	D	1	chr8	.	CAGCACTTGCAGGATCAATTAATGTCTCGCTTATGTCCCTATTCTGCTCTAGTTATGTCTTTGA
TP12605_Query	D	1	chr8	.	CAGCACTTGCAGGATCAATTAATGTCCCGCTTATGTCCCTATTCTGCTCTAGTTATGTCTTTGA
TP12632_Hit	D+G	1	chr8	.	CAGCACTTGGTGAGGGAGCATTTCGAGCCGGTGGTTGTGTTGGGGAGTGTGCCGGAGAAGCACT
TP12632_Query	D+G	1	chr8	.	CAGCACTTGGTGAGGGAGCATTTCGAACCGGTGGTTGTGTTGGGGAGTGTGCCGGAGAAGCACT
TP12646_Hit	D	1	chr8	.	CAGCACTTGTGAATTTCCTGATCCATTCCATATCATCCCCTCCATACAAGCATATGTATTTGCC
TP12646_Query	D	1	chr8	.	CAGCACTTGTGAATTTCCTGATCCATCCCATATCATCCCCTCCATACAAGCATATGTATTTGCC
TP12726_Hit	D	1	chr8	.	CAGCACTTTTGCTTGGTATCTTTGACATAGAAAAAACGAAATGATTTTTTGAATACTCTCCACC
TP12726_Query	D	1	chr8	.	CAGCACTTTTACTTGGTATCTTTGACATAGAAAAAACGAAATGATTTTTTGAATACTCTCCACC
TP12736_Hit	D+G	1	chr8	.	CAGCACTTTTTAATAGAGTGATGGTATCTGACTGTATAAGAGAACCTATTCGGCTTTCAATCTC
TP12736_Query	D+G	1	chr8	.	CAGCACTTTTTAATAGAGTGATAGTATCTGACTGTATAAGAGAACCTATTCGGCTTTCAATCTC
TP12818_Hit	D	1	chr8	.	CAGCAGAAAATTACCGGGAGAGGGCATTCCCATTTTATCTTAGGAATGTGGGAGAATTGAACCT
TP12818_Query	D	1	chr8	.	CAGCAGAAAATTACCGGGAGAGGGCATTCCCATTTTATCTTAGGAATGTCGGAGAATTGAACCT
TP12934_Hit	D	1	chr8	.	CAGCAGAAATCGCACAATAGATGACCTTGCTAACCAATGACCACAAATGGGCATAATAGGGCCC
TP12934_Query	D	1	chr8	.	CAGCAGAAATCGCACAATAGACGACCTTGCTAACCAATGACCACAAATGGGCATAATAGGGCCC
TP13043_Hit	D+G	1	chr8	.	CAGCAGAACGTTGCCCGGACTAGATTGTTTTTCAAGCTCATGGAGGAATGCTTGCTGAAAAAAA
TP13043_Query	D+G	1	chr8	.	CAGCAGAACGTTGCCCGGACTAGATTGTTTTTCAAGCTCATGGAGGAATGCGTGCTGAAAAAAA
TP13049_Hit	D	1	chr8	.	CAGCAGAACTATCTGAGGACTCAATGTTGTTATATCTTCAAACCGAAAAACATATGTAGGATCA
TP13049_Query	D	1	chr8	.	CAGCAGAACTATCTGAGGACTCAATGTTGTTATATCTTCAAACCGAAAAACATATCTAGGATCA
TP13141_Hit	D+G	1	chr8	.	CAGCAGAAGCAAGACGAGGACCAACCAAATCACAAGCTGTTCCCATTGCAATTCTTGCAAAAAC
TP13141_Query	D+G	1	chr8	.	CAGCAGAAGCAAGACGAGGACCAACCAAATCACAAGCAGTTCCCATTGCAATTCTTGCAAAAAC
TP13212_Hit	D+G	1	chr8	.	CAGCAGAAGGCTTCGTCAGAGGAAGGTCTTATAACATTAGACTTGATGATCAAGTTTTGCTTAA
TP13212_Query	D+G	1	chr8	.	CAGCAGAAGGCTTCGTCAGAGGAAGGTCTTATAACATCAGACTTGATGATCAAGTTTTGCTTAA
TP13413_Hit	D	1	chr8	.	CAGCAGACAAGTGAAATATTGGCATTTATTTGCCCCTATTATTTTGGGTACAACAATGATATCA
TP13413_Query	D	1	chr8	.	CAGCAGACAAGTGAAATATTGGCATTTATTTGCCCCTACTATTTTGGGTACAACAATGATATCA
TP13688_Hit	D	1	chr8	.	CAGCAGAGACGAAAGTAAAAATATCATGGTACCAGCTCTGAAGAAATGAGCGAGGGAGAAAAGG
TP13688_Query	D	1	chr8	.	CAGCAGAGACGAAAGTAAAAATATCATGGTACCAGCTATGAAGAAATGAGCGAGGGAGAAAAGG
TP137_Hit	D	1	chr8	.	CAGCAAAAACCATACAGAAGGAGGAGATCTGAGTAAACACATTCAAACAGAGCCTGAGAAATGA
TP137_Query	D	1	chr8	.	CAGCAAAAACCATACAGAAGAAGGAGATCTGAGTAAACACATTCAAACAGAGCCTGAGAAATGA

Name	Filter	Nb hit (Mt4.0)	Mt Chr (Mt4.0)	Ms Chr (Li et al., 2014)	Sequence
TP13731_Hit	D	1	chr8	.	CAGCAGAGCAGACACCATGTGGGCTGGAGATAAAGGAAACGGGCAACTGGGGTTTGTTTGAGG
TP13731_Query	D	1	chr8	.	CAGCAGAGCAAACACCATGTGGGCTGGAGATAAAGGAAACGGGCAACTGGGGTTTGTTTGAGG
TP13792_Hit	D	1	chr8	.	CAGCAGAGCTCCATTAACATGGAACGAAGGGGAAAATAAAAAGATCAGGCTTCTGTATATTTA
TP13792_Query	D	1	chr8	.	CAGCAGAGCTCCATTAACATGGAACGAAGGGGAAAATAAAAAGATCAGGCTTCTGTACATTTA
TP13885_Hit	D+G	1	chr8	.	CAGCAGAGGTGTGAGGAGGTGCATCCTGGATATGGGGATGAAGGGAATCTGAGGGTGCTTGGAT
TP13885_Query	D+G	1	chr8	.	CAGCAGAGGTGTCAGGAGGTGCATCCTGGATATGGGGATGAAGGGAATCTGAGGGTGCTTGGAT
TP13968_Hit	D+G	1	chr8	.	CAGCGGAGTTGAAAGGGCACATGTGACTCACTTCCTTCAGATTAACATTACTATAAAGTAGTTT
TP13968_Query	D+G	1	chr8	.	CAGCAGAGTTGAAAGGGCACATGTGACTCACTTCCTTCAGATTAACATTACTATAAAGTAGTTT
TP14107_Hit	D	1	chr8	.	CAGCAGATCATGCCTAATGCACTTCCATTTGCTAGAAGGTTAGTTTTTTAAAACAAAACCCTTT
TP14107_Query	D	1	chr8	.	CAGCAGATCATGCCTAATACACTTCCATTTGCTAGAAGGTTAGTTTTTTAAAACAAAACCCTTT
TP14218_Hit	D+G	1	chr8	.	CAGCAGATGCTTTCATTCTTGGTCATGGTCGTGCAATGCCCTGGATTCTTGGTTCAGCTGAAAA
TP14218_Query	D+G	1	chr8	.	CAGCAGATGCTCTCATTCTTGGTCATGGTCGTGCAATGCCCTGGATTCTTGGTTCAGCTGAAAA
TP1432_Hit	D+G	1	chr8	.	CAGCAAGCTATCACAAGCTAAAGGGGGTGTGACAGTAGCAACAGCCACAGCCAATGTATTCAGA
TP1432_Query	D+G	1	chr8	.	CAGCAAACTATCACAAGCTAAAGGGGGTGTGACAGTAGCAACAGCCACAGCCAATGTATTCAGA
TP14351_Hit	D	1	chr8	.	CAGCTGATTTAGCCAAAATGGTAGCAAACTCATTGAAACTAATAACACCATCACCATTGCTATC
TP14351_Query	D	1	chr8	.	CAGCAGATTTAGCCAAAATGGTAGCAAACTCATTGAAACTAATAACACCATCACCATTGCTATC
TP14479_Hit	D	1	chr8	.	CAGCAGGAATCAGTTTCTCTAGTTCCATAAGTTCAGTCATACTGAGGTGCTCAAGTTCAAAAAC
TP14479_Query	D	1	chr8	.	CAGCAGGAATCAGTTTCTCTAGTTCCATAAGTTCAGTCACACTGAGGTGCTCAAGTTCAAAAAC
TP14507_Hit	D	1	chr8	.	CTGCAGGACAATTTCAAGGGACTATTGCTAATTCACAATAATTACCGCAGGAGGCCGAGGAGGC
TP14507_Query	D	1	chr8	.	CAGCAGGACAATTTCAAGGGACTATTGCTAATTCACAATAATTACCGCAGGAGGCCGAGGAGGC
TP14557_Hit	D	1	chr8	.	CAGCAGGACTTATCAGAAGTATTTTCTGAACGATTTATCTAACACCGGAAAACATTCTGACATT
TP14557_Query	D	1	chr8	.	CAGCAGGACTTATCAGAAGTATTTACTGAACGATTTATCTAACACCGGAAAACATTCTGACATT
TP14578_Hit	D	1	chr8	.	CAGCAGGAGAGGGTGCCAAGCTGTCCTTCCGACAAGATGTCGACAAATTAGCTGAAAAAAAAAA
TP14578_Query	D	1	chr8	.	CAGCAGGAGAGGGTGCCAAGCTGTCCTTCCGACAAGATGTCGACAAATTAGCAGAAAAAAAAAA
TP14701_Hit	D	1	chr8	.	CAGCAGGATATATTGCAGGTGTACTGTGTGCAATTGTTTCACACCCTGCTGATAACCTTGTTTC
TP14701_Query	D	1	chr8	.	CAGCAGGATATATTGCAGGTGTACTGTGTGCAATTGTTTCACACCCTGCTGATAACCTCGTTTC
TP14923_Hit	D	1	chr8	.	CAGCAGGCTTTACAAAACTAAAAGAACCCAACCATGAACATCACCTACTCCCCCAACAGCAGTA
TP14923_Query	D	1	chr8	.	CAGCAGGCTTTACAAAACTAAAAGAACACAACCATGAACATCACCTACTCCCCCAACAGCAGTA
TP14942_Hit	D	1	chr8	.	CAGCGGGGAATCAGGTTAATGGTGCATCATTCTTCGGTTATGCAAATGGAACAGCAAGAGGAAT
TP14942_Query	D	1	chr8	.	CAGCAGGGAATCAGGTTAATGGTGCATCATTCTTCGGTTATGCAAATGGAACAGCAAGAGGAAT
TP15105_Hit	D	1	chr8	.	CAGCAGGGGGTTTTCACGAAGAACATAGACACTGCAAATACTTTGACAAGAGCATTGAGAGTTGG
TP15105_Query	D	1	chr8	.	CAGCAGGGGGTTTTCACAAAGAACATAGACACTGCAAATACTTTGACAAGAGCATTGAGAGTTGG
TP15121_Hit	D	1	chr8	.	CAGCAGGGTGAATTGGGGACTGGTGGAATTTTTGGTTAAGATTGGAGTTATTTAAACCTATGTC
TP15121_Query	D	1	chr8	.	CAGCAGGGTGAATTGGGGACTGGTGGAATTTTTGGTTAAGATTGAAGTTATTTAAACCTATGTC
TP15213_Hit	D+G	1	chr8	.	CAGCAGGTATATCTTTAACAGAAAGAGGCAAGACAACCAAAGAAGGTACATAGAACAAAGGGAG
TP15213_Query	D+G	1	chr8	.	CAGCAGGTATATCTTTAACAGAAAGAGGCAAGACAACCAAAGAAGGAACATAGAACAAAGGGAG
TP15248_Hit	D	1	chr8	.	CAGCAGGTCCAATGTTGCTCAATATTCTCTGGAGATCTGCCAATTTAACTGGACCAGAAGATGT
TP15248_Query	D	1	chr8	.	CAGCAGGTCCAATGTTGCTCAATATTCTCTGGAGATCTGCCAATTTAACTGGACCACAAGATGT
TP15459_Hit	D	1	chr8	.	CAGCAGGTTTATATTTACTCTTCATTTGTTTAGACCTGGATTCTAGAGGATTCCATGCATATTC
TP15459_Query	D	1	chr8	.	CAGCAGGTTTATATTTACTCTTCATTTGTTTAGACCTGGATTCTAGAGGATTCCACGCATATTC
TP15671_Hit	D	1	chr8	.	CAGCAGTACTTTTGTCCTCTTGTTGCAGAAGAAATATACTAGTGCATCAATTTCTGGTCTCACC
TP15671_Query	D	1	chr8	.	CAGCAGTACTTTTGTCCTCTTGTTGCAGAAGAAATATACTACTGCATCAATTTCTGGTCTCACC
TP15927_Hit	D	1	chr8	.	CAGCAGTCATCAATGAGCTCTCCATTCCCATCTCTGAGCCTGCAAGGTTGTCCTGGAGTTGATC
TP15927_Query	D	1	chr8	.	CAGCAGTCATCAATGAGCTCTCCATTCCCATCTCTGAGCCTGCAAGGTTGTCCCGGAGTTGATC
TP16_Hit	D	1	chr8	.	CAGCAAAAAAAAGTTCTTTTGATTCTAGATGATGTTGACAAAATAGAACAGCTAGATGCCTTGG
TP16_Query	D	1	chr8	.	CAGCAAAAAAAAGTTCTTTTGATTCTAGATGATGTTGACAAAATAGAACAGCTAGAGGCCTTGG
TP16007_Hit	D	1	chr8	.	CAGCAGTCGATGTGATTCGGTAACCAGACTCCCACCGCTTGTGAATCCCTTCACTAGGGTACAG
TP16007_Query	D	1	chr8	.	CAGCAGTCGATGTGATTCGGTAACCAGACTCCCACCGCTTGTGAATCCCTTCACTAGGATACAG
TP16089_Hit	D	1	chr8	.	CAGCAGTGAACACTCATCCCAATGTTCAGCTACAGAATTTGCATCAATACATGCCGGCAACAAC
TP16089_Query	D	1	chr8	.	CAGCAGTGAACACTCATCCCAATGTTCAGCCACAGAATTTGCATCAATACATGCCGGCAACAAC
TP16545_Hit	D	1	chr8	.	CAGCAGTTCATACCTCATGATTTTGATGCAAGTGACTATGGGACATCTGGCTTCCCTGTTACTG
TP16545_Query	D	1	chr8	.	CAGCAGTTCATACCTCATGATTTTGATGCAAGTGACTATGGAACATCTGGCTTCCCTGTTACTG
TP1660_Hit	D	1	chr8	.	CAGCAAAGAGGTAGAGAACTATGTCAACACTAGGATTCAAGTAGGTGGATAAAATGGAGAGTTT
TP1660_Query	D	1	chr8	.	CAGCAAAGAGGTAGAGAACTATGTCAACACTAGCATTCAAGTAGGTGGATAAAATGGAGAGTTT

Name	Filter	Nb hit (Mt4.0)	Mt Chr (Mt4.0)	Ms Chr (Li et al., 2014)	Sequence
TP16736_Hit	D	1	chr8	.	CAGCAGTTGTACCTCCACAAAATGGAGTGATTGCTTTAACACGCCTTTCTTTTTTACTTGAGAA
TP16736_Query	D	1	chr8	.	CAGCAGTTGTACCTCCACAAAATGGAGTGATTGCTTTAACACGCCTTTCTTTTTTACTTAAGAA
TP16827_Hit	D	1	chr8	.	CAGCAGTTTCTTTGGCTTCTCAGAACAGCTTCTTTGTGAAGCCAAAGACTGTTAAAAGGAGAAA
TP16827_Query	D	1	chr8	.	CAGCAGTTTCTTTGGCTTCTCAGAACAGCTTCTATGTGAAGCCAAAGACTGTTAAAAGGAGAAA
TP16829_Hit	D	1	chr8	.	CAGCAGTTTGACTCTCGCGGCTACTTTCAAGTGAACGAATTACAACCCAATGATCAGTATGCCA
TP16829_Query	D	1	chr8	.	CAGCAGTTTGACTCTCGCGGCTACTTCCAAGTGAACGAATTACAACCCAATGATCAGTATGCCA
TP1684_Hit	D+G	1	chr8	.	CAGCAAAGATGGAGGAACCCCTGGTTCCTTCTCTCTTGGACTTTCAGGTATACATTCATTATTT
TP1684_Query	D+G	1	chr8	.	CAGCAAAGATGGAGGAACCACTGGTTCCTTCTCTCTTGGACTTTCAGGTATACATTCATTATTT
TP17095_Hit	D+G	1	chr8	.	CAGCATAACCCGCCGTGGCTATGCCACCGAACCGGTTCCTGAACGCAAGGTGGCCATTCTCGGT
TP17095_Query	D+G	1	chr8	.	CAGCATAACCCGCCGTGGCTATGCCACCGAACCGGTGCCTGAACGCAAGGTGGCCATTCTCGGT
TP17307_Hit	D	1	chr8	.	CAGCATAATTTGCATTCTCTCCAAGAAACCAAACCTAAAGGCATAAGTAGTCAGTAAGAAAAGA
TP17307_Query	D	1	chr8	.	CAGCATAATTTGCATTCTCTCCAAGAAACCAAACCTAAAAGCATAAGTAGTCAGTAAGAAAAGA
TP1731_Hit	D	1	chr8	.	CAGCAAGGCAGGCCCAATAATTTTTAATAGCAAGTGAGGCGTAAAGGAGATTTTAAAATATGGG
TP1731_Query	D	1	chr8	.	CAGCAAAGCAGGCCCAATAATTTTTAATAGCAAGTGAGGCGTAAAGGAGATTTTAAAATATGGG
TP17724_Hit	D	1	chr8	.	CAGCATAGTGTGAGAGGACTTAGAAGCCAAGGACTGACTCCACAAATCTTGGCTTGCCGCTGAA
TP17724_Query	D	1	chr8	.	CAGCATAGTGTGAGAGGACTTAGAAGCCAAGGACTGACTCCACAAATCTTGGCTTGCCGCAGAA
TP17807_Hit	D	1	chr8	.	CAGCATATAGGAAGGACTCGGATTTTAGTGGTAATTTAAGAGAAGCAATAGCATTATCAGGAAA
TP17807_Query	D	1	chr8	.	CAGCATATAGAAAGGACTCGGATTTTAGTGGTAATTTAAGAGAAGCAATAGCATTATCAGGAAA
TP17901_Hit	D	1	chr8	.	CAGCATATCCATCATATTCAGCTTCGAAGAATTGGCGTTCTGCCTCTGCAATTGCCAACAGTCG
TP17901_Query	D	1	chr8	.	CAGCATATCCATCATATTCAGCTTCGAAGAATTGGCGTTCTGCCTCTGCAATTGCCAACAATCG
TP17912_Hit	D+G	1	chr8	.	CAGCATATCCTGAGTGTTGTCAGACAGCATATTCGTCTGAGATTCGCCTTCCTTAGGTGCTGAA
TP17912_Query	D+G	1	chr8	.	CAGCATATCCTGAGTGTTGTCAGACAGCATATTCGTCTGAGATTCGCCTTCCATAGGTGCTGAA
TP17933_Hit	D+G	1	chr8	.	CAGCATATCTCAGAAAGTTTCCTGAACAAATTGGGAGTACCTTTTATAGCTCAGTACCTGGTGA
TP17933_Query	D+G	1	chr8	.	CAGCATATCTCAGAAAGTTTCCTAAACAAATTGGGAGTACCTTTTATAGCTCAGTACCTGGTGA
TP17966_Hit	D	1	chr8	.	CAGCATATGACTCACAACGTAGGGAGTTGCCACCACCAATATTGGTGTGGGAATGGGGATGGAC
TP17966_Query	D	1	chr8	.	CAGCATATGACTCACAACGTAGGGAGTTGCCACCACCAATATTGGTGTGAGAATGGGGATGGAC
TP17971_Hit	D	1	chr8	.	CAGCATATGATAGTCATCATGCTTCTCAGGGAGCAATTCATCCAATACAAGAGCCTGGCGGAAT
TP17971_Query	D	1	chr8	.	CAGCATATGATAGTCATCATGCTTCTCAGGGAGCAATTCATCCAATACAAGAGCCTGACGGAAT
TP18185_Hit	D	1	chr8	.	CAGCATCAAAATGCTGAAGAGGAAAGGGTAATAGATAAATTACTGATTCATTATAGCAAAAAGC
TP18185_Query	D	1	chr8	.	CAGCATCAAAATGCTGAAGAGGAAAGGGTAATAGATAAATTACTGATTCATTATAGCAAAAAAC
TP184_Hit	D+G	1	chr8	.	CAGCAAAAAGATAAGAGTGGTCCTATTGAGATAGTAGGGCAATCAAGTAGCCTGGATATTATGA
TP184_Query	D+G	1	chr8	.	CAGCAAAAAGATAAGAGTGGTCCTATTGAGATAGTAGGGCAATCAAGTAACCTGGATATTATGA
TP18544_Hit	D+G	1	chr8	.	CAGCATCACTGAGAGCTGGCACTGATGAAAATATTTGAGGAATCAGCCCATCATTTGTCAAACC
TP18544_Query	D+G	1	chr8	.	CAGCATCACTGAGAGCTGGCACAGATGAAAATATTTGAGGAATCAGCCCATCATTTGTCAAACC
TP18572_Hit	D	1	chr8	.	CAGCATCAGAGAGGAAGACCGGTGGAATTTGCGGCGAAACGACGTAACGCACCATCTTCGTTCC
TP18572_Query	D	1	chr8	.	CAGCATCAGAGAGGAAAACCGGTGGAATTTGCGGCGAAACGACGTAACGCACCATCTTCGTTCC
TP18577_Hit	D	1	chr8	.	CAGCATCAGATAATCTAGGTTGATTCTCTGCAATATTATTGACCTTAGCATCCATATTCTCAGT
TP18577_Query	D	1	chr8	.	CAGCATCAGATAATCTAGGCTGATTCTCTGCAATATTATTGACCTTAGCATCCATATTCTCAGT
TP18582_Hit	D	1	chr8	.	CAGCATCAGATATAATGAATATAGTTAAAGGGACGAACCGTAAGCTTTCCAAGGGACTTGCTGA
TP18582_Query	D	1	chr8	.	CAGCATCAGATATAATGAATATAGTTAAAGGGACGAACCGTAAGCTTTCCAAGGGACTTGCGGA
TP18668_Hit	D	1	chr8	.	CAGCATCAGTAGTATTTTACAACAAGTGTTCCCACCCAGTGTGGCCAGCAATCCAACCCGGTGC
TP18668_Query	D	1	chr8	.	CAGCATCAGTAGTATTTTACAACAAGTGTCCCCACCCAGTGTGGCCAGCAATCCAACCCGGTGC
TP1877_Hit	D	1	chr8	.	CAGCAAAGGGTCAAGTTCCTTTTGGTTTCGTAACAAGTTCCCTGCTATAGCACTTTGTCTTGTT
TP1877_Query	D	1	chr8	.	CAGCAAAGGGTCAAATTCCTTTTGGTTTCGTAACAAGTTCCCTGCTATAGCACTTTGTCTTGTT
TP18860_Hit	D	1	chr8	.	CAGCATCCAAAGCTAGTGAATCATTCACACCGTCTCCTAAGAAACCAACAGCATGGTTTCCTTC
TP18860_Query	D	1	chr8	.	CAGCATCCAAAGCTAGTGAATCATTCACACCGTCTCCTAAGAAACCAACAACATGGTTTCCTTC
TP18929_Hit	D	1	chr8	.	CAGCATCCATAATTTGTTCAAACATATCTAATGCCGATGGATGGCGAAGCTGTTTGATAAAAAC
TP18929_Query	D	1	chr8	.	CAGCATCCATAATTTGTTCAAACATATCTAATGCCGATGGATGGCGAAGCTGTTCGATAAAAAC
TP18946_Hit	D+G	1	chr8	.	CAGCATCCATTTGAGCTCTCTCCTAAGCATCTTGGAGATCTGCAATAGAGGATGAAGTCCCTAG
TP18946_Query	D+G	1	chr8	.	CAGCATCCATTTGAGCTCTATCCTAAGCATCTTGGAGATCTGCAATAGAGGATGAAGTCCCTAG
TP18956_Hit	D+G	1	chr8	.	CAGCATCCCATCTTCTTCATCCAATTCTACCACTGTTTCGTGTTTTTCTTGGAATATCAGGAAA
TP18956_Query	D+G	1	chr8	.	CAGCATCCCATCTTCTTCATCCAATTCTACCACTGTTTCCTGTTTTTCTTGGAATATCAGGAAA
TP18991_Hit	D	1	chr8	.	CAGCATCTGATACGGTCCTGCAAATTGGCCTGTACAAAACCACACAAACAATGTTGCACATTTA
TP18991_Query	D	1	chr8	.	CAGCATCCGATACGGTCCTGCAAATTGGCCTGTACAAAACCACACAAACAATGTTGCACATTTA

Name	Filter	Nb hit (Mt4.0)	Mt Chr (Mt4.0)	Ms Chr (Li et al., 2014)	Sequence
TP19031_Hit	D	1	chr8	.	CAGCATCCTCATCTTGCCATCAACAGAATTGCATGGTAATAGAGTATATGGAGACAAAAGAAAG
TP19031_Query	D	1	chr8	.	CAGCATCCTCATCTTGCCATCAACAGAATTGCATGCTAATAGAGTATATGGAGACAAAAGAAAG
TP19326_Hit	D	1	chr8	.	CAGCATCTCAAATCATACAAAGTTAAAATTTTCAAGAATCATATAACAAGTGCCATAAATTCTA
TP19326_Query	D	1	chr8	.	CAGCATCTCAAATCATACAAAGTAAAAATTTTCAAGAATCATATAACAAGTGCCATAAATTCTA
TP19443_Hit	D	1	chr8	.	CAGCATCTGCTGAAAATCCAATTCCAGGACTACACCAAACATCTTGAAAATCAACACAAGTAGC
TP19443_Query	D	1	chr8	.	CAGCATCTGCTGAAAATCCAATTCCAGGACCACACCAAACATCTTGAAAATCAACACAAGTAGC
TP19472_Hit	D+G	1	chr8	.	CAGCATCTGGTTTCTCTACCCAAGTCCTTACCACTAGTAGACAATGCACCTGCTTATTCGTCTT
TP19472_Query	D+G	1	chr8	.	CAGCATCTGGTTTCTCTACCCAAGTCCTTACCACTAGCAGACAATGCACCTGCTTATTCGTCTT
TP19525_Hit	D	1	chr8	.	CAGCATCTTCAGCATACTCATAAGTCCCAAGCCACACCCTCGCGCCATCTCAGCTGAAAAAAAA
TP19525_Query	D	1	chr8	.	CAGCATCTTCAGCAGACTCATAAGTCCCAAGCCACACCCTCGCGCCATCTCAGCTGAAAAAAAA
TP19526_Hit	D	1	chr8	.	CAGCATCTTCAGCATACTCATAAGTCCCAAGCCACACCCTCGCGCCGTTCTTATTCGGATCTCT
TP19526_Query	D	1	chr8	.	CAGCATCTTCAGCAGACTCATAAGTCCCAAGCCACACCCTCGCGCCGTTCTTATTCGGATCTCT
TP19607_Hit	D+G	1	chr8	.	CAGCATCTTTGGAATCTTATTCATACTTCTGAAAACAAGTGCAATATCGTTTTTGGAAATGACT
TP19607_Query	D+G	1	chr8	.	CAGCATCTTTGAAATCTTATTCATACTTCTGAAAACAAGTGCAATATCGTTTTTGGAAATGACT
TP19935_Hit	D	1	chr8	.	CAGCATGATCATATTTCATACAGCCCTGGGTCAGGGGGACATTTTGGTGTTGGTTTTGGTTCAA
TP19935_Query	D	1	chr8	.	CAGCATGATCATATTTCATACAGCCCTGGGTCAGGGGGACATTCTGGTGTTGGTTTTGGTTCAA
TP20019_Hit	D	1	chr8	.	CAGCATGCAAAGGCCTATGGAGTTGTTTATCCCACTTCTCATTAATGATATCCCATAGCGCCTT
TP20019_Query	D	1	chr8	.	CAGCATGCAAAGGCCTATGGAGTTGTTTATCCCACTTCTCATTAATGATATCCCATAGCACCTT
TP20053_Hit	D	1	chr8	.	CAGCATGCAATTTGATAAACAATGAAATGAAGCAATTTGTTATCAGTTCAGAAACTGATGCAAT
TP20053_Query	D	1	chr8	.	CAGCATGCAATTTGATAAACAATGAAATGAAGCAATTCGTTATCAGTTCAGAAACTGATGCAAT
TP20113_Hit	D	1	chr8	.	CAGCATGCATATTGGATGCGATATTGGGGACACTGATTGTATGTTTCTCACTGTTGTTTTCATT
TP20113_Query	D	1	chr8	.	CAGCATGCATATTGGATGCGATATTGGGGACACTGATTGTATGTTTCTCACTGTCGTTTTCATT
TP20169_Hit	D	1	chr8	.	CAGCATGCCTGTGCGGTCCTTATCGGTATCAGTCCAAATATGGCTGTTTCAGGTTTATGAAAAT
TP20169_Query	D	1	chr8	.	CAGCATGCCTGTACGGTCCTTATCGGTATCAGTCCAAATATGGCTGTTTCAGGTTTATGAAAAT
TP20174_Hit	D	1	chr8	.	CAGCATGCTTTATTCTCTAATTATTCCAACTCCAAATAACTTACTAAATGACCATGGGAGTACC
TP20174_Query	D	1	chr8	.	CAGCATGCCTTATTCTCTAATTATTCCAACTCCAAATAACTTACTAAATGACCATGGGAGTACC
TP20250_Hit	D+G	1	chr8	.	CAGCATGCTGAGTTACTGAAGAAGGTGATTCGGCACGAGAGGGACCTTTCTTGACGGGTGGCTT
TP20250_Query	D+G	1	chr8	.	CAGCATGCTGAGTTACTGAAGAAGGTGATTCGGCACGAGAGGGACCTTTCTTGACGGGTAGCTT
TP20267_Hit	D+G	1	chr8	.	CAGCATGTTTATCATCCCAACTAAACCATGATACACTTCCTACACTACCAAATTCATTCTCATT
TP20267_Query	D+G	1	chr8	.	CAGCATGCTTATCATCCCAACTAAACCATGATACACTTCCTACACTACCAAATTCATTCTCATT
TP20407_Hit	D+G	1	chr8	.	CAGCATGGCTCACTGGATCAGGTTCTGTGCCTCTTTACAATCTCATGGAAGGTGCTGAAAAAAA
TP20407_Query	D+G	1	chr8	.	CAGCATGGCTCACTGGATCAGGTTCTGTGCCACTTTACAATCTCATGGAAGGTGCTGAAAAAAA
TP2074_Hit	D	1	chr8	.	CAGCAAATACCAGGATTGTTGACGCAAAATTGAGCAATGTAGCATGGAGAGAAAGAGTTGACGG
TP2074_Query	D	1	chr8	.	CAGCAAATACCAGGATTGTTGACGCAAAATTGGGCAATGTAGCATGGAGAGAAAGAGTTGACGG
TP20886_Hit	D	1	chr8	.	CAGCATTAAAAAATAGTATATGGCCCAACTTTAAAACCTTTATCTGGTCTTAAAACAAAAGAAA
TP20886_Query	D	1	chr8	.	CAGCATTAAAAAATAGTATATGGCCCAACTTTAAAACCTTTATCTAGTCTTAAAACAAAAGAAA
TP20935_Hit	D	1	chr8	.	CAGCATTAACTAATATAATCCATAGAAATGTCACTACTACCACTAAATAAAACAGTAGCTATCA
TP20935_Query	D	1	chr8	.	CAGCATTAACTAATATAATCCATAAAAATGTCACTACTACCACTAAATAAAACAGTAGCTATCA
TP21009_Hit	D	1	chr8	.	CAGCATTACATCATTTATATGTGTCTGAAGAGCTACACAAATTTCTTCACCCTACAACGAAGCG
TP21009_Query	D	1	chr8	.	CAGCATTACATCATTTATATGTGTCTGAAGAGCTACACAAATTTCTTCACCCTACAACAAAGCG
TP21030_Hit	D	1	chr8	.	CAGCATTACTACTAGACCATTCTGGTGCACTGAAAATAGGATCCAATATTGGTGAACCTATAAT
TP21030_Query	D	1	chr8	.	CAGCATTACTACTAGACCATTCTGGTGCACTGAAAATAAGATCCAATATTGGTGAACCTATAAT
TP21053_Hit	D	1	chr8	.	CAGCATTAGAAAGATTTCCATGTTCATCGACTTTAACTTCCTCACCGAGCATGGAAAGACATTG
TP21053_Query	D	1	chr8	.	CAGCATTAGAAAGATTTCCATGTTCATCGACTTTAACCTCCTCACCGAGCATGGAAAGACATTG
TP21071_Hit	D	1	chr8	.	CAGCATTAGAGTGCATGTACTTATGCACAAGTTTTAGTGAGATGAATTCCAAACGATGGTGATG
TP21071_Query	D	1	chr8	.	CAGCATTAGAGTGCATGTACGTATGCACAAGTTTTAGTGAGATGAATTCCAAACGATGGTGATG
TP21096_Hit	D	1	chr8	.	CAGCATTAGCCACATTTGACTATGATTTTAAATCACACTGGTTGCTATTTAAAACCCTGGTAGA
TP21096_Query	D	1	chr8	.	CAGCATTAGCCACATTTGACTATGATTTTAAATCACACTGGTTGCTATTTAAAACCCTAGTAGA
TP21150_Hit	D+G	1	chr8	.	CAGCATTAGTGTTCACTTTATCCCAATGCAAAGGCTTCAATTTCACTTGATCATTGTTGGATTC
TP21150_Query	D+G	1	chr8	.	CAGCATTAGTGTTCACTTTATCCCAATGCAAAGGCTTCAATTTCACTTGATCATTATTGGATTC
TP21170_Hit	D+G	1	chr8	.	CAGCATTATAGCTTGTAAACTTGAAATCATCGTCTTCAGCACTCACTTTCAATGCTGTTGTTAT
TP21170_Query	D+G	1	chr8	.	CAGCATTATAGCTTGTAAACTTGAAATCATCGTCTTCAGCACTCACTTTCAACGCTGTTGTTAT
TP2121_Hit	D	1	chr8	.	CAGCAAATATGGATTATAATAGTTTAATTGGTTCAATTTCAGCACTATCTGTTAAGCTAATTGA
TP2121_Query	D	1	chr8	.	CAGCAAATATGGATTATAATAGTTTAATTGGTTCAATTTCAGCACTATCTGTTAAACTAATTGA

Name	Filter	Nb hit (Mt4.0)	Mt Chr (Mt4.0)	Ms Chr (Li et al., 2014)	Sequence
TP21265_Hit	D	1	chr8	.	CAGCATTCAAAGGGGCTTAATATACCTATTTGTAATTCAATTTTAAGAACCATTGGTTGGCACA
TP21265_Query	D	1	chr8	.	CAGCATTCAAAGGGGCTTAATATACCTATTTGTAATTCAATTTTAAGAACCATTGGTTGCCACA
TP21565_Hit	D+G	1	chr8	.	CAGCATTCTTTTCTGGCTTCATCTTCCAGCCGTCAACTCTTTCTCTCCATGCTACATTGCTCAA
TP21565_Query	D+G	1	chr8	.	CAGCATTCTTTTCTGGCTTCATCTTCCAGCCGTCAACTCTTTCTCTCCATGCTACATTGCCCAA
TP21593_Hit	D	1	chr8	.	CAGCATTGAACAAAGGCATAATTCCACGTGACTCGGCGCGGTTTTGGTTGTTTTGATTATTTGC
TP21593_Query	D	1	chr8	.	CAGCATTGAACAAAGGCATAATTCCACGTGACTCGGCGCGGTTTTGGTTGTTTTGATTATTTCC
TP21601_Hit	D	1	chr8	.	CAGCATTGAAGAAAGCAATAAAAGAAGGGTAATTGAGATCCAATGAAGGTTTAGAACAATCATT
TP21601_Query	D	1	chr8	.	CAGCATTGAAGAAAGCAATAAAAGAAGGGTAATTGAGATCCAACGAAGGTTTAGAACAATCATT
TP21844_Hit	D	1	chr8	.	CAGCATTGGTGTGCCAACTGTGAATGGGTCGGTTCCTGTTCAGCAGGTTTTTAGCATGCCTTTT
TP21844_Query	D	1	chr8	.	CAGCATTGGTGTGCCAACTGTGAATGGGTCGGTTCCTGTTCAGCAGGCTTTTAGCATGCCTTTT
TP21928_Hit	D	1	chr8	.	CAGCATTGTTCGGATTATCCCTTCACGATTCCCAAATTAACCAAACCCGCATTCTACCTTTACC
TP21928_Query	D	1	chr8	.	CAGCATTGTTCGGACTATCCCTTCACGATTCCCAAATTAACCAAACCCGCATTCTACCTTTACC
TP21989_Hit	D	1	chr8	.	CAGCATTTGATTAACATTCTTTATTGAATACACAAATCTAACTAGCTTGCTCCATATTTTTAGA
TP21989_Query	D	1	chr8	.	CAGCATTTAATTAACATTCTTTATTGAATACACAAATCTAACTAGCTTGCTCCATATTTTTAGA
TP22029_Hit	D	1	chr8	.	CAGCATTTGGTTGGACTTGAAGAATTGAGCTTTTGTTACTGTCGAGATCTCGAGACTATTCCAT
TP22029_Query	D	1	chr8	.	CAGCATTTAGTTGGACTTGAAGAATTGAGCTTTTGTTACTGTCGAGATCTCGAGACTATTCCAT
TP22137_Hit	D	1	chr8	.	CAGCATTTCCTCTCACGTGGCATCCCACAAAGTATATAATGGACTGACATTGCACAAAATAAAA
TP22137_Query	D	1	chr8	.	CAGCATTTCCTCTCACGTGGCATCCCACAAAGTATATAACGGACTGACATTGCACAAAATAAAA
TP2238_Hit	D+G	1	chr8	.	CAGCAAATCGGCATTTTTAAAATGCAGGTTGGCCCTGAATGGGGTGAAGGGAAACCTGTGATTC
TP2238_Query	D+G	1	chr8	.	CAGCAAATCGGCATTTTTAAAATGCAGGTTGGCCCTGAATGGGGTGAAGGGAAACCTATGATTC
TP22384_Hit	D	1	chr8	.	CAGCATTTGTTGCAAAAGAACAAGCATGGCTTTCTATGATGAGTCTCTGAACATCGATAATCAC
TP22384_Query	D	1	chr8	.	CAGCATTTGTTGCAAAAGAACAAGCATGGCTTTCTATGATGAGTCTCTGAACAACGATAATCAC
TP22469_Hit	D	1	chr8	.	CAGCATTTTCGCGACAATAATTCCTTCCACAATCACAATCATTTAAATATAAAATTACACGACG
TP22469_Query	D	1	chr8	.	CAGCATTTTCGCGACAATAATTCCTCCCACAATCACAATCATTTAAATATAAAATTACACGACG
TP22503_Hit	D	1	chr8	.	CAGCATTTTGCACCGCACGGGTACCCTAATAATTTCTACCATTCTCAGAGTGGGATTTCTCTAG
TP22503_Query	D	1	chr8	.	CAGCATTTTGCACCGCACGGGTACCCTAATAATTTCTACCATTCTCAGAGTGGAATTTCTCTAG
TP22613_Hit	D	1	chr8	.	CAGCCAAAAAAATTGCATTTGACACCCTCTTCCATGACTTGAGAGATTTCAATTTCTTATCAAG
TP22613_Query	D	1	chr8	.	CAGCCAAAAAAATTGCATTCGACACCCTCTTCCATGACTTGAGAGATTTCAATTTCTTATCAAG
TP22683_Hit	D	1	chr8	.	CAGCCAAAACATTGAATATCATAAACAGGTCAAATAAAGATAGCTCACCACATTGTCAGAAAAT
TP22683_Query	D	1	chr8	.	CAGCCAAAACATAGAATATCATAAACAGGTCAAATAAAGATAGCTCACCACATTGTCAGAAAAT
TP2269_Hit	D	1	chr8	.	CAGCAAATGAACGTATCTTAGTTCGATGCACGGCACCTGTGACGACATTCTGCATTGTCCTTAA
TP2269_Query	D	1	chr8	.	CAGCAAATGAACGTATCTTAGTTCGATGCACGGCACCTGTGACAACATTCTGCATTGTCCTTAA
TP22766_Hit	D	1	chr8	.	CAGCCAAAAGTTGAGCCAAGAATGTGGAGTGGATGGTTACCCTTTGATGTGAAGGGTGATCCGA
TP22766_Query	D	1	chr8	.	CAGCCAAAAGTTGAGCCAAGAATGTGGAGTGGATGGTTACCCTTTGATGTGAAGGGGGATCCGA
TP22942_Hit	D	1	chr8	.	CAGCCAAACTTGAAAATTTCATCCGCGGGTCAAGAACCGCTCCCATTGCAAGAACAACACTATA
TP22942_Query	D	1	chr8	.	CAGCCAAACTTGAAAATTTCATCCACGGGTCAAGAACCGCTCCCATTGCAAGAACAACACTATA
TP23009_Hit	D	1	chr8	.	CAGCCAAAGCCTTTTCCCACTATCTGGGTCCTTAGATACTTTAAACAAACACGTTCAATTATCT
TP23009_Query	D	1	chr8	.	CAGCCAAAGCCTTTTCCCACTATCTGGGTCCTTAGATACTTTAAACAAACACATTCAATTATCT
TP23028_Hit	D	1	chr8	.	CAGCCAAAGGCCGGTCACCGCCAAATCCTGCTCCACAGCCAATGTAAACCTTATCCCTTCGCTT
TP23028_Query	D	1	chr8	.	CAGCCAAAGGCCGGTCACCGCCAAATCCTACTCCACAGCCAATGTAAACCTTATCCCTTCGCTT
TP2321_Hit	D	1	chr8	.	CAGCAAATGCAACACTACCTAAATTTTCATTCCTCGAAAGTGAAGACTTTGTGGCTAGAAATGA
TP2321_Query	D	1	chr8	.	CAGCAAATGCAACACTACCTAAATTTTCATTCCTCGAAAGTGAAGACTTTGTAGCTAGAAATGA
TP2340_Hit	D	1	chr8	.	CAGCAAATGCATTTTAGCTCAGCGTGTTGGTATATACTACAACATAAAGCTTTAACCAACAAAT
TP2340_Query	D	1	chr8	.	CAGCAAATGCATTTTAGCTCAGCGTGTTGGTATATACTACAACATAAAGCTTGAACCAACAAAT
TP23424_Hit	D	1	chr8	.	CAGCCAACCTCTCCATAAGAGAACATCACTACTCAGCCCTAAGCCCTTAACCAATAACCGTTTA
TP23424_Query	D	1	chr8	.	CAGCCAACCTCTCCATAACAGAACATCACTACTCAGCCCTAAGCCCTTAACCAATAACCGTTTA
TP23504_Hit	D+G	1	chr8	.	CAGCCAACTGGGGTTCGTTAAAGGTCCTGCGCAGATACTCGGTAAAGTTTGGCGTGAAGCATTC
TP23504_Query	D+G	1	chr8	.	CAGCCAACTGGGGTTCGTTAAAAGTCCTGCGCAGATACTCGGTAAAGTTTGGCGTGAAGCATTC
TP23541_Hit	D	1	chr8	.	CAGCCAAGAAAAGAGAGATGAGGTACTCTGATCTGAAGAAAATTGACCGAGGTGTTCGTCGTCA
TP23541_Query	D	1	chr8	.	CAGCCAAGAAAAGAGAGATGAGGTACTCTGATCTGAAGAAAATTGACCGAGGTGTCCGTCGTCA
TP23565_Hit	D	1	chr8	.	CAGCCAAGAAGAAAAACAATCAATACACAGATGGACTTAAATCCATATAGAAAGGGAAAAATCA
TP23565_Query	D	1	chr8	.	CAGCCAAGAAGAAAAACAATCAATACACAGACGGACTTAAATCCATATAGAAAGGGAAAAATCA
TP23647_Hit	D	1	chr8	.	CAGCCAAGCATGAAGGAGCAAGTAAGAACAAAACCTTGGTCATCACCTTATCAAGTGTACTTGT
TP23647_Query	D	1	chr8	.	CAGCCAAGCATGAAGGAGCAAGTAAGAACAAAACCTTGATCATCACCTTATCAAGTGTACTTGT

Name	Filter	Nb hit (Mt4.0)	Mt Chr (Mt4.0)	Ms Chr (Li et al., 2014)	Sequence
TP24074_Hit	D	1	chr8	.	CAGCCAATGTTTTGCATTTGATTTCTCCATCTCTGAATCAATTTGATAATGCACCAGACATCAT
TP24074_Query	D	1	chr8	.	CAGCCAATGTTTTGCATCTGATTTCTCCATCTCTGAATCAATTTGATAATGCACCAGACATCAT
TP24319_Hit	D	1	chr8	.	CAGCCACAATAAAGAATTTACATGCCAACTTGACATTTCTTTTCACTCCAATCCCTTTGAGATA
TP24319_Query	D	1	chr8	.	CAGCCACAATAAAGAATTTACATGCCAACTTGACATCTCTTTTCACTCCAATCCCTTTGAGATA
TP24477_Hit	D+G	1	chr8	.	CAGCCACAGGCACAACCCCAGGAAGATGGTTTGACAAATGATGGGCTGATTCCTCAAATATTTT
TP24477_Query	D+G	1	chr8	.	CAGCCACAGCCACAACCCCAGGAAGATGGTTTGACAAATGATGGGCTGATTCCTCAAATATTTT
TP24590_Hit	D	1	chr8	.	CAGCCACATCGATCGCGTTTGATCATGCTTTTTCACGATATCAAGAATCACAACAAAATCAACC
TP24590_Query	D	1	chr8	.	CAGCCACATCGATCACGTTTGATCATGCTTTTTCACGATATCAAGAATCACAACAAAATCAACC
TP24669_Hit	D	1	chr8	.	CAGCCACCAAAAGAACTTTTATTGTTGGATTTGTCAATGCACTATGAAAATTCAAAATATGTTA
TP24669_Query	D	1	chr8	.	CAGCCACCAAAAGAACTTTTATTGTTGGATTTGTCAATGCACTATGAAAATGCAAAATATGTTA
TP2467_Hit	D	1	chr8	.	CAGCAAATTATGATAATGCAGAATAGATAGAAAGAAGCTACATGAAATAGACAATTAGCTATGC
TP2467_Query	D	1	chr8	.	CAGCAAATTATGATAATGCAGAATAGATAGAAAGAAGCTACATGAAATAGACAATTAACTATGC
TP24722_Hit	D	1	chr8	.	CAGCCACCACAAGTAGAGGTGTTAACAGCAAAAGTAAGGAGCAATAAAAAAGAAAGACAAAACT
TP24722_Query	D	1	chr8	.	CAGCCACCACAAGTAGAGGTGCTAACAGCAAAAGTAAGGAGCAATAAAAAAGAAAGACAAAACT
TP24738_Hit	D	1	chr8	.	CAGCCACCACCCTTCCTAGCTCTTGAGCCTGAGGACCAGTCACCACCCTTCCTAGCTCTTGATG
TP24738_Query	D	1	chr8	.	CAGCCACCACCCTTCCTAGCTCTTGAGCCTGAGGACCAGTCACCACCCTTCCTAGCTCTTGAGG
TP24896_Hit	D	1	chr8	.	CAGCCACTGGTGTTGTGGAGACCCTAACCTTCGATGACCCCTTCACTTCCTTGTCATCCTGATC
TP24896_Query	D	1	chr8	.	CAGCCACCGGTGTTGTGGAGACCCTAACCTTCGATGACCCCTTCACTTCCTTGTCATCCTGATC
TP25351_Hit	D	1	chr8	.	CAGCCACTGGTGCAGAATTTGCAGAACATGATATTCGTTATTGGTCTTTTCATTCTGAGTTGAG
TP25351_Query	D	1	chr8	.	CAGCCACTGGTGCAGAATTCGCAGAACATGATATTCGTTATTGGTCTTTTCATTCTGAGTTGAG
TP25614_Hit	D	1	chr8	.	CAGCCAGAGATAAAACTATTCAAAGTGGACCAACCAGTGAAGTCTGGCATAGGAATGCTCGGTG
TP25614_Query	D	1	chr8	.	CAGCCAGAGATAAAACTATTCAAAGTGAACCAACCAGTGAAGTCTGGCATAGGAATGCTCGGTG
TP25784_Hit	D	1	chr8	.	CAGCCAGCATCACCTCCCAGAGCTTCTGCTATGGAGGTAGACTCTAGTGGTGCGGAGATCGGAA
TP25784_Query	D	1	chr8	.	CAGCCAGCATCACCTCCCAGAGCTTCTGCTATGGAGGTAGACTCTAGTGGTGCCGAGATCGGAA
TP25808_Hit	D	1	chr8	.	CAGCCAGCCAATAAACTTGCATATAATCACACACATTGTTACATTAATATATAGAACAATCAAC
TP25808_Query	D	1	chr8	.	CAGCCAGCCAATAAACTTGCATACAATCACACACATTGTTACATTAATATATAGAACAATCAAC
TP2594_Hit	D+G	1	chr8	.	CAGCAAATTTGGTGACTGTGTCGCTTGGTACTGAAACCGACGGCTCCATCTTATGCCCTTCAAG
TP2594_Query	D+G	1	chr8	.	CAGCAAATTTGGTGACTGTGTCACTTGGTACTGAAACCGACGGCTCCATCTTATGCCCTTCAAG
TP26235_Hit	D	1	chr8	.	CAGCCATAAATCCTGTGTGACAAGAAAACCATAATATTAAAAGAGAGGAACAAATAAAATGGAA
TP26235_Query	D	1	chr8	.	CAGCCATAAATCCTGTGTGACAAGAAAAACATAATATTAAAAGAGAGGAACAAATAAAATGGAA
TP26371_Hit	D	1	chr8	.	CAGCCATAGATGTATCAGGTGCTAAACCAGCTAGTTGTCCAACAGAAAATGAACTCAAAACAAG
TP26371_Query	D	1	chr8	.	CAGCCATAGAAGTATCAGGTGCTAAACCAGCTAGTTGTCCAACAGAAAATGAACTCAAAACAAG
TP26375_Hit	D+G	1	chr8	.	CAGCCATAGAGGAAGAAATGTAGAAACATGAGACTAAACACACTGCAAAAACAAAATAGAAACA
TP26375_Query	D+G	1	chr8	.	CAGCCATAGAGGAAGAAATGTAGAAACATGAGACTAAACACACTGCAAAAACAAAACAGAAACA
TP26515_Hit	D	1	chr8	.	CAGCCATCAAATGAAGAAGACTTTGAATGGTTGGGATCTTATTTGGTTCGGAATCGGTGCCGTC
TP26515_Query	D	1	chr8	.	CAGCCATCAAATGAAGAAGACTTTGAATGGTTGGGATCTTATTTGGTTCGGAATCGGCGCCGTC
TP26675_Hit	D	1	chr8	.	CAGCCATCCTCATCTTCTCAATCAAAGCTTCAGTATTTACCTGTTCTGACAAAATCTCCTCTAG
TP26675_Query	D	1	chr8	.	CAGCCATCCTCATCTTCTCAATCAAAGCTCCAGTATTTACCTGTTCTGACAAAATCTCCTCTAG
TP2670_Hit	D	1	chr8	.	CAGCAACAAAACATGATTTTAGTTAAAAGCGAATTGAATATAAAGTGATTTATATTTGGATATA
TP2670_Query	D	1	chr8	.	CAGCAACAAAACATGATTTTAGTTAAAAGCGAATTGAATATAAAGTGATTTATATTTGGACATA
TP26739_Hit	D	1	chr8	.	CAGCCATCTGATCATCGCCTCTACTCGGTCTTAAAAGGACTACACATTCTGCCTCAGCTAGCAT
TP26739_Query	D	1	chr8	.	CAGCCATCTGATCATCGCCTCTACTCGGTCTTAAAAGGACTACACATCCTGCCTCAGCTAGCAT
TP26882_Hit	D	1	chr8	.	CAGCCATGATTGATGAGCACTGCGATGTAGACTCTTTAAGGGATGGAGATAACAATTTGTCCGG
TP26882_Query	D	1	chr8	.	CAGCCATGATTGAAGAGCACTGCGATGTAGACTCTTTAAGGGATGGAGATAACAATTTGTCCGG
TP26986_Hit	D+G	1	chr8	.	CAGCCATGGAAGACTGAAGAACATTAAGGTGCAAAGTTTCACTCCTTCAACACCACCACCAACA
TP26986_Query	D+G	1	chr8	.	CAGCCATGGAAGACTGAAGAACATTAAGGTGCAAAGCTTCACTCCTTCAACACCACCACCAACA
TP27121_Hit	D	1	chr8	.	CAGCCATGTACTGTGTTCTCCCGGCGTCGATCTGGCCGGCGGGAATGAACATGAACCCGACTTG
TP27121_Query	D	1	chr8	.	CAGCCATGTACTGTGTTCTCCCAGCGTCGATCTGGCCGGCGGGAATGAACATGAACCCGACTTG
TP27200_Hit	D	1	chr8	.	CAGCTATTAAAAGTTTTAACTTTACTGATGAAAGAATAATGAATGGTAACTGGGTTAATGAGCC
TP27200_Query	D	1	chr8	.	CAGCCATTAAAAGTTTTAACTTTACTGATGAAAGAATAATGAATGGTAACTGGGTTAATGAGCC
TP27255_Hit	D	1	chr8	.	CAGCCATTACTGGCATAATGCCTCCGCCGCTCATGGAACAACAGGTAAAATATTAATTATTTTA
TP27255_Query	D	1	chr8	.	CAGCCATTACTGGCATAATGCCTCCACCGCTCATGGAACAACAGGTAAAATATTAATTATTTTA
TP27365_Hit	D	1	chr8	.	CAGCCATTCTTAGGGTGTTCCAATCTGTTGTACAAAATTCTTTGGCAAAAATGTTGTAATCAAC
TP27365_Query	D	1	chr8	.	CAGCCATTCTTAGGGTGTTCCAATCTGTTGTACAAAATTCTTTGGCAAAAATGTTGCAATCAAC

Name	Filter	Nb hit (Mt4.0)	Mt Chr (Mt4.0)	Ms Chr (Li et al., 2014)	Sequence
TP27560_Hit	D	1	chr8	.	CAGCCATTTGTGCTCGCTCCCTGTCCCTCGGGTAGGTACAATAAAGGAATGAATAGATGAAGCT
TP27560_Query	D	1	chr8	.	CAGCCATTTGTGCTCGCTCCCTGTCCCTCGGGTAGGTACAATAAAGGAATGAATAGATGAAGCA
TP27576_Hit	D	1	chr8	.	CAGCCATTTTACGAGGGATGATGGCACATTAATAGCATGTGCATTATCAGGATCGATTTTCACA
TP27576_Query	D	1	chr8	.	CAGCCATTTTACGAGGGATGACGGCACATTAATAGCATGTGCATTATCAGGATCGATTTTCACA
TP27595_Hit	D	1	chr8	.	CAGCCATTTTCTTGTAAGCTTTCTGTCAGACATTTTAATTTGCAGGTTGAATGGCAGATACAAT
TP27595_Query	D	1	chr8	.	CAGCCATTTTCTTGTAAGCTTTCTCTCAGACATTTTAATTTGCAGGTTGAATGGCAGATACAAT
TP27598_Hit	D+G	1	chr8	.	CAGCCATTTTGTTAATTGTGTTAGTTCCAGAAAGACTGGCTTCACACTGTTTCTTGTATATATA
TP27598_Query	D+G	1	chr8	.	CAGCCATTTTGTTAATTGTGTTAGTTCCAGAAAGACTAGCTTCACACTGTTTCTTGTATATATA
TP27599_Hit	D	1	chr8	.	CAGCCATTTTGTTAATTGTGTTAGTTCCAGAAAGACTGGCTTCACACATTTTCTTGCATATATA
TP27599_Query	D	1	chr8	.	CAGCCATTTTGTTAATTGTGTTAGTTCCAGAAAGACTGGCTTCACACAGTTTCTTGCATATATA
TP27611_Hit	D+G	1	chr8	.	CAGCCATTTTTGGTGAAATTGAAACTGAGGTGAGAACTGACCATCCTGAGATTTATTGTTGTTG
TP27611_Query	D+G	1	chr8	.	CAGCCATTTTTGGTGAAATTGAAACTGAGGTGAGAACTGACCATCCTGAGATTTATTGTTATTG
TP27804_Hit	D	1	chr8	.	CAGCCCAAGCTTTGCAAACATACCTTGCAGAGTTAATCAGGCAGTTAAGAGGAACCAACCTAAG
TP27804_Query	D	1	chr8	.	CAGCCCAAGCTTTACAAACATACCTTGCAGAGTTAATCAGGCAGTTAAGAGGAACCAACCTAAG
TP2789_Hit	D	1	chr8	.	CAGCAACAAAGTTTCAGAAAAGGTAATATTTTAAGTTATCTCTAGTTAAATGGTTTTCTCCAAT
TP2789_Query	D	1	chr8	.	CAGCAACAAAGTTTCAGAAAAGGTAATATTTTAAGTTATCTCTAGTAAAATGGTTTTCTCCAAT
TP28289_Hit	D	1	chr8	.	CAGCCCATTAATCAATGCAACGATAATAGACCCAAGGCGGCGAGTAAGTCTTGCATAGACATAA
TP28289_Query	D	1	chr8	.	CAGCCCATTAATCAATGCAACGATAATAGACCCAAGGCGGCGAGTAAGTCTTGCATAGACACAA
TP28344_Hit	D	1	chr8	.	CAGCCCATTTGCCCCATGGCCTCATACGAACACCGCGGTATTTGGCACAAGTTCGTCTCCCTGT
TP28344_Query	D	1	chr8	.	CAGCCCATTTGCCCCATGGCCTCATACGAACACCGCGGTATTTGGCACAAGATCGTCTCCCTGT
TP28406_Hit	D+G	1	chr8	.	CAGCCCCAAGCAGTTCTTCAATGGCCATGTACTATGTCCTAAATTTGCTTCCTTGACGTGCTGA
TP28406_Query	D+G	1	chr8	.	CAGCCCCAAGCAGTTCTTCAATGGCCATGTACTATGTCCTAAATTTGCTTCCTTGACATGCTGA
TP28704_Hit	D	1	chr8	.	CAGCCCCGCTCCAATCTTGCTTGTCAACGGCAAAGTACCGGAGATAGGATCAGACCATTTTTTC
TP28704_Query	D	1	chr8	.	CAGCCCCGCTCCAATCTTACTTGTCAACGGCAAAGTACCGGAGATAGGATCAGACCATTTTTTC
TP28762_Hit	D+G	1	chr8	.	CAGCCCCTAGGTGCTCAGAAATTCCAGAACTTGTATCACTAAAGAAAATCTTTGAGAAGAAATA
TP28762_Query	D+G	1	chr8	.	CAGCCCCTAGGTGCTCAGAAATTCCAGAACTTGTATCACTAAAAAAAATCTTTGAGAAGAAATA
TP28929_Hit	D	1	chr8	.	CAGCCTGAAATTCCTTCCACCAATTCATCAATGGTATCCTCACCAACTATAGTTCTTTCTGGCT
TP28929_Query	D	1	chr8	.	CAGCCCGAAATTCCTTCCACCAATTCATCAATGGTATCCTCACCAACTATAGTTCTTTCTGGCT
TP29163_Hit	D+G	1	chr8	.	CAGCCCGGTTCGGCCTTGAACCGTTTGGATATTTTCGATCCCTTGGAGAGAAAAAATACCACTC
TP29163_Query	D+G	1	chr8	.	CAGCCCGGTTCGGCCTTGAACCGTTTGGATATTTACGATCCCTTGGAGAGAAAAAATACCACTC
TP29228_Hit	D+G	1	chr8	.	CAGCCCGTCCTGTGTTTGATTCGGTAGGGTTTTGCGGTGGTTGGTGTTCCTGGTGCTGAAAAAA
TP29228_Query	D+G	1	chr8	.	CAGCCCGTCCTGTGTTTGATTCGGTAGGGTTTTGCGGTGGTGGGTGTTCCTGGTGCTGAAAAAA
TP29344_Hit	D+G	1	chr8	.	CAGCCCTGATGCCAAAGTGTGTGAAAATCGAAGCCATTTCTTGTTCTGGGATCAATATCATCCC
TP29344_Query	D+G	1	chr8	.	CAGCCCTAATGCCAAAGTGTGTGAAAATCGAAGCCATTTCTTGTTCTGGGATCAATATCATCCC
TP29626_Hit	D	1	chr8	.	CAGCCCTGGGTCAGGGGGACATTTTGGTGTTGGTTTTGGTTCAACTGGTTATTCTCCTCAAACA
TP29626_Query	D	1	chr8	.	CAGCCCTGGGTCAGGGGGACATTTTGGTGTTGGTTTTGGTTCAACTGGTTATTCTCCACAAACA
TP29817_Hit	D+G	1	chr8	.	CAGCCCTTTCTGCTCGTTCGCGGCCTTCAGCATATGCTCTATCACGAGCCTCAAAAGTTGCTCT
TP29817_Query	D+G	1	chr8	.	CAGCCCTTTCTGCTCGTTCGCGACCTTCAGCATATGCTCTATCACGAGCCTCAAAAGTTGCTCT
TP3008_Hit	D	1	chr8	.	CAGCAACAGCTATGTCAGAATTAGGAAGGAATGGTAATTCACATGCAAAATGGAATAAAATATG
TP3008_Query	D	1	chr8	.	CAGCAACAACTATGTCAGAATTAGGAAGGAATGGTAATTCACATGCAAAATGGAATAAAATATG
TP30165_Hit	D	1	chr8	.	CAGCCGAGGTGGAGGCGGAGATTCTGATCATTCAGATCTAGATGTTTCTGCTGTGAAGGAAGGA
TP30165_Query	D	1	chr8	.	CAGCCGAGGTGGAGGCGGAGATTCTGATCATTCAGATCTAGATGTTTCTGCTGTCAAGGAAGGA
TP30217_Hit	D	1	chr8	.	CAGCCGATCTTTTGAAACAATACCAGAGAAAGTTCCAAGGAGGGACTTTATCAGTGACATGGAA
TP30217_Query	D	1	chr8	.	CAGCCGATCTTTTGAAACAATACCAGAGAAAGTTCCAAGGAGGGACTTTAGCAGTGACATGGAA
TP30364_Hit	D+G	1	chr8	.	CAGCCGCAATTTCTACCGCTGTTAACCTTGATTTTCCTTGACCCTAGAGAAAATGAGATTAAGT
TP30364_Query	D+G	1	chr8	.	CAGCCGCAATTTCTACCGCTGTTAACCTTGATTTACCTTGACCCTAGAGAAAATGAGATTAAGT
TP30375_Hit	D+G	1	chr8	.	CAGCCGCACAAGAGACAAACGAAAAGTAAACTTTAGAAAATAGAAGAGTGAATAAAAAAAGGGT
TP30375_Query	D+G	1	chr8	.	CAGCCGCACAAGAGACAAACGAAAAGTAAACTTGAGAAAATAGAAGAGTGAATAAAAAAAGGGT
TP31601_Hit	D+G	1	chr8	.	CAGCTGTTTCATTTTGTTTAAAACATCCTTTGATTATGAATATTTTCAAGCAATTTCTGATTCA
TP31601_Query	D+G	1	chr8	.	CAGCCGTTTCATTTTGTTTAAAACATCCTTTGATTATGAATATTTTCAAGCAATTTCTGATTCA
TP31659_Hit	D	1	chr8	.	CAGCCTAAACCATCTAATGGATATGGTCGTCGGAAATCTGAAAAAGACGGGTCATCTAAATCTG
TP31659_Query	D	1	chr8	.	CAGCCTAAACCATCTAATGGATATGGTCGTCGGAAATCTGAAAAAGACGGGGCATCTAAATCTG
TP31921_Hit	D	1	chr8	.	CAGCCTACTGCTGTTGGGGGAGTAGGTGATGTTCATGGTTGTGTTCTTTTAGTTTTGTAAAGCC
TP31921_Query	D	1	chr8	.	CAGCCTACTGCTGTTGGGGGAGTAGGTGATGTTCATGGTTGGGTTCTTTTAGTTTTGTAAAGCC

Name	Filter	Nb hit (Mt4.0)	Mt Chr (Mt4.0)	Ms Chr (Li et al., 2014)	Sequence
TP31991_Hit	D	1	chr8	.	CAGCCTAGTCAAGTTCAAAACCTTTTCCATTCCGTACAACTAAATGTGAACAATCCACATTTCC
TP31991_Query	D	1	chr8	.	CAGCCTAGTCAAGTTCAAAACCTTTTCCATTCCGTACAACTAAATGTGAACAACCCACATTTCC
TP32004_Hit	D	1	chr8	.	CAGCCTAGTGGCAAGTGGCTCTAAAATTCTTAGCCTAAATTTGTAATTGATTAGATATTGTTGT
TP32004_Query	D	1	chr8	.	CAGCCTAGTGGCAAGTGGCTCTAAAATTCTTAGCCTAAATTTGTAATTGATTAGATATTGTAGT
TP32097_Hit	D	1	chr8	.	CAGCCTATCTATATTCGGTTCTGCAAATTCGTACTCTTCAGCCTTGTAACGAACATGAAACTTA
TP32097_Query	D	1	chr8	.	CAGCCTATCTATATTCGGTTCTGCAAATTCGTACTCCTCAGCCTTGTAACGAACATGAAACTTA
TP32280_Hit	D	1	chr8	.	CAGCCTCAACCACGTTCGCGTTGTGGTCACAAAGACATCAAAAACTTTTTTTTCGCTGAAAAAA
TP32280_Query	D	1	chr8	.	CAGCCTCAACCACGTTCGCGTTGTGGTCACAAAGACATCAAAAACTTTTTTTTCGCAGAAAAAA
TP32325_Hit	D	1	chr8	.	CAGCCTCAATATAAAAGATTTTGAGGTCTCTGCAATGACATCGCAACTGCGCATTTTGCCCGCA
TP32325_Query	D	1	chr8	.	CAGCCTCAATATAAAAGATTTTGAGGTCTCTGCAATGACATCACAACTGCGCATTTTGCCCGCA
TP32516_Hit	D	1	chr8	.	CAGCCTCATCCTTACTAATAACACCTTTACTCCATTTTGCCCTAGCTTGAGTATTAGCACCAAG
TP32516_Query	D	1	chr8	.	CAGCCTCATCCTTACTAATAACACCTTTACTCCATTTAGCCCTAGCTTGAGTATTAGCACCAAG
TP32622_Hit	D+G	1	chr8	.	CAGCCTCCACTGTCAGATTTGAGTTTCCAAACACAATGCACCTCTCAGGTATGAAGTTCAAAAG
TP32622_Query	D+G	1	chr8	.	CAGCCTCCACAGTCAGATTTGAGTTTCCAAACACAATGCACCTCTCAGGTATGAAGTTCAAAAG
TP32721_Hit	D	1	chr8	.	CAGCCTCTGAAGATGCTTGTGTTGATTCTAAAAAGTCTTTTTCATCTGATCTTATGACACAAGC
TP32721_Query	D	1	chr8	.	CAGCCTCCGAAGATGCTTGTGTTGATTCTAAAAAGTCTTTTTCATCTGATCTTATGACACAAGC
TP32809_Hit	D	1	chr8	.	CAGCCTCCTTAATCTTCTCAATATACCCATCAATAGGTGGCGTAAGTGTCGCCATAAACCTATT
TP32809_Query	D	1	chr8	.	CAGCCTCCTTAATCTTCTCAATATACCCATCAATAGGTGGCGTAAGCGTCGCCATAAACCTATT
TP3304_Hit	D+G	1	chr8	.	CAGCAACACCAGGGGTACCAGCAATATCATAAAGAGAAAGGGTTGAAACGAGAGGGTTGAGCTT
TP3304_Query	D+G	1	chr8	.	CAGCAACACCAGGGGTACCAGCAATATCATAAAGAGAAAGGGTTGAAACGAGAGGATTGAGCTT
TP33163_Hit	D+G	1	chr8	.	CAGCCTCTTCCGACTAAACTTGTTTTAAGAAAAAAGAGGGCTAAAGAGGGAAGATCGGGCGATG
TP33163_Query	D+G	1	chr8	.	CAGCCTCTTCCAACTAAACTTGTTTTAAGAAAAAAGAGGGCTAAAGAGGGAAGATCGGGCGATG
TP33198_Hit	D	1	chr8	.	CAGCCTCTTTACACTCTGCAACTCGCCTATTATATCCGGGGTTGGTAGTCAACGCTTGCTTCAA
TP33198_Query	D	1	chr8	.	CAGCCTCTTTACACTCTGCAACTCGCCTATTATATCCGGGGTTGGTAGTCAAAGCTTGCTTCAA
TP33491_Hit	D+G	1	chr8	.	CAGCCTGCAACTAATTTAGTTTTGTTTTTATCCATTATTTAGTTTCAAGGTGCTATGGGATATC
TP33491_Query	D+G	1	chr8	.	CAGCCTGCAACTAATTTAGTTTTGTTTTTATCCATTATTTAGTTTCAAGGCGCTATGGGATATC
TP33614_Hit	D	1	chr8	.	CAGCCTGCTAGTTTCAACTCTGCAGGAATGAGAACCCGTGCCCAAAAGGGTTTCTCCAAAGTGG
TP33614_Query	D	1	chr8	.	CAGCCTGCTAGTTTCAACTCAGCAGGAATGAGAACCCGTGCCCAAAAGGGTTTCTCCAAAGTGG
TP33735_Hit	D	1	chr8	.	CAGCCTGGGCTCATCCAAAACACAGATCCGAGCTATTAAGTTCCTCCCAAAACGGATCAGACGA
TP33735_Query	D	1	chr8	.	CAGCCTGGGCTCATCCAAAACACAGATCCGAGCTATTAAGTTCATCCCAAAACGGATCAGACGA
TP33746_Hit	D	1	chr8	.	CAGCCTGGTAAATTTTTGTTTGGAGAAGTAAGCTGATTTTGTACGCAAGGGATGCTTAGTCACA
TP33746_Query	D	1	chr8	.	CAGCCTGGTAAATTTTTGTGTGGAGAAGTAAGCTGATTTTGTACGCAAGGGATGCTTAGTCACA
TP34666_Hit	D	1	chr8	.	CAGCCTTGGAATGTGACCGTATATGGCAAAGTTGGCATCCTGACAATGAAGCCTCAGTAATATG
TP34666_Query	D	1	chr8	.	CAGCCTTGGAATGTGACCGTATATGGCAAAGTTGGCATACTGACAATGAAGCCTCAGTAATATG
TP34855_Hit	D	1	chr8	.	CAGCCTTTATGACTCTATCTTGGTCTGTGGTTATCGAAACGGGAGGCTGACCATTCATTGCAGA
TP34855_Query	D	1	chr8	.	CAGCCTTTATGACTCTATCTTGGTCTGTGGTTATCGAAACGGGAGGCTGACCATTCATAGCAGA
TP3493_Hit	D	1	chr8	.	CAGCAACAGCGAGCGAGAAGGGTGGTTCTGGTTCGGTGGCACCTTTCTTGAATAAATGTTACGA
TP3493_Query	D	1	chr8	.	CAGCAACAGCGAGCGAGAAGGGTGGTTCTGGTTCGGTGGCACCTTTCTTAAATAAATGTTACGA
TP35017_Hit	D	1	chr8	.	CAGCCTTTGTGTAGAACACATCATACATAAAACCAAGCTCAATTTCCATAACTTCAAACCCTTT
TP35017_Query	D	1	chr8	.	CAGCCTTTGTGTAGAACACATCATACATAAAACCAAGCTCAATTTCCACAACTTCAAACCCTTT
TP35052_Hit	D	1	chr8	.	CAGCCTTTTCAAACTACAACAATTTCACAGTTCCCGGCTCTGCTGAGTCGGAAACTGTGTACGG
TP35052_Query	D	1	chr8	.	CAGCCTTTTCAAACTACAACAATTTCACAGTTCCCGGCTCTGCGGAGTCGGAAACTGTGTACGG
TP35181_Hit	D+G	1	chr8	.	CAGCGAAAAGCACATACTTCAATTCATTGAGTTTTTGTTCCTTTGTTTTCGATCCACTGCTTTT
TP35181_Query	D+G	1	chr8	.	CAGCGAAAAGCACATACATCAATTCATTGAGTTTTTGTTCCTTTGTTTTCGATCCACTGCTTTT
TP35205_Hit	D	1	chr8	.	CAGCGAAAATTGATGCAGAGACGAAAGTTATCGCGACGCAGAGGGCTGGAGAGGCTGAGAAGGA
TP35205_Query	D	1	chr8	.	CAGCGAAAATTGATGCAGAGACGAAAGTTATCGCGACACAGAGGGCTGGAGAGGCTGAGAAGGA
TP35390_Hit	D+G	1	chr8	.	CAGCGAACGTAGTAGCGAAGGCCGTTATCGAGAGTGCCGAAGTCGACGCCGACTGGTTGGTCAG
TP35390_Query	D+G	1	chr8	.	CAGCGAACGTAGTAGCGAAGGCCGTTATCGAGAGTGCCGAAGTCGACGCCGACGGGTTGGTCAG
TP35443_Hit	D	1	chr8	.	CAGCGAAGAGTGGCTTTATTATCGTAGAAAGCATAAACTTGATGAGACATGGATCTATGCTTTG
TP35443_Query	D	1	chr8	.	CAGCGAAGAGTGGCTTTATTATCGTAGAAAGCATAAACTTGACGAGACATGGATCTATGCTTTG
TP3560_Hit	D	1	chr8	.	CAGCAACAGTAAGTATCCAAGGTGCAAGGTTAGTAGCAGAACCAGGATTTCCTTCATTTCCAGC
TP3560_Query	D	1	chr8	.	CAGCAACAGTAAGCATCCAAGGTGCAAGGTTAGTAGCAGAACCAGGATTTCCTTCATTTCCAGC
TP35656_Hit	D	1	chr8	.	CAGCGACAAACTTCGTCATCATGCCTATACTTCCTCTACCCGCTCCTCCCACTGATGGAAATCT
TP35656_Query	D	1	chr8	.	CAGCGACAAACTTCGTCATCATGCCTATACTTCCTCAACCCGCTCCTCCCACTGATGGAAATCT

Name	Filter	Nb hit (Mt4.0)	Mt Chr (Mt4.0)	Ms Chr (Li et al., 2014)	Sequence
TP35724_Hit	D+G	1	chr8	.	CAGCGACACGGCGAGGTTGAGTGACGGCGATTTTGGAGTAACCGCGGCGGTGAAGCATCTGAGA
TP35724_Query	D+G	1	chr8	.	CAGCGACACGGCGAGGTTGAGTGACAGCGATTTTGGAGTAACCGCGGCGGTGAAGCATCTGAGA
TP35775_Hit	D	1	chr8	.	CAGCGACATCTGTCAAACTCTTGCTTTCTACTGCATAATCAAACACTTCATAAGCATCCGCAAT
TP35775_Query	D	1	chr8	.	CAGCGACATCTGTCAAACTCTTGCTTTCAACTGCATAATCAAACACTTCATAAGCATCCGCAAT
TP35861_Hit	D	1	chr8	.	CAGCGACGGAGAAACTACCGACGAAGGGGAAAGATTCAGCGCATTACATTGATGAAAAATCGAA
TP35861_Query	D	1	chr8	.	CAGCGACGAAGAAACTACCGACGAAGGGGAAAGATTCAGCGCATTACATTGATGAAAAATCGAA
TP35996_Hit	D	1	chr8	.	CAGCGACTGCAAGCATGAGAAGAAGGAAGACACCCGGTGGTGGGGCATCTGGAGGTGCGGAGAT
TP35996_Query	D	1	chr8	.	CAGCGACTGCAAGCATGAGAAGAAGGAAGACACCCGGTGGTGGGGCATCTGGAGGTGCCGAGAT
TP3601_Hit	D+G	1	chr8	.	CAGCAACAGTTCAACCACTCACACCAAATCCATCTTCACAAGGAACAATCCCTGCCTTTCCTGA
TP3601_Query	D+G	1	chr8	.	CAGCAACAGTTCAACCACTCACACCAAATCCATCTTCACAAGGAACAATACCTGCCTTTCCTGA
TP36259_Hit	D+G	1	chr8	.	CAGCGAGCTGAAGTCTGGAAATCCAGATCGTTTTGCGATTGCTGACACAACATATACGTACGGA
TP36259_Query	D+G	1	chr8	.	CAGCGAGCTGAAGTCTGGAAATCCAGATCGTTTTGCGATTGCTGACACAACATATACGTACCGA
TP36387_Hit	D	1	chr8	.	CAGCGAGTCTGAAGATGCCGGCGGAGGGAATCCGCATTCGAATTCGCATCATCTGAAACGGCAT
TP36387_Query	D	1	chr8	.	CAGCGAGTCTGAAGATGCCGGCGGAGGGAATCCGCATTCGAATTCGCATCATCGGAAACGGCAT
TP36427_Hit	D	1	chr8	.	CAGCGAGTTAATATTGTTGAACTATCGGAAAATGAAAAGCTTGCCTTCTTCTTGAATTTGTATA
TP36427_Query	D	1	chr8	.	CAGCGAGTTAATATCGTTGAACTATCGGAAAATGAAAAGCTTGCCTTCTTCTTGAATTTGTATA
TP36562_Hit	D+G	1	chr8	.	CAGCGATCCATAATGAAATATCAAGGTGTGCAACAGCTCTGAAGAAAGAAAGACGAAAGCACAG
TP36562_Query	D+G	1	chr8	.	CAGCGATCCATAATGAAATATCAAGGTGTGCAACAACTCTGAAGAAAGAAAGACGAAAGCACAG
TP36598_Hit	D	1	chr8	.	CAGCGATCTGCCTCGTGATTTCTATGATCCTACCACCGATCCTAAAAACTACAATCTTAACATC
TP36598_Query	D	1	chr8	.	CAGCGATCTGACTCGTGATTTCTATGATCCTACCACCGATCCTAAAAACTACAATCTTAACATC
TP36659_Hit	D	1	chr8	.	CAGCGATGATGGCGTTGCACGTGAACACCGTAGGCCTGGTTGATGAGAACACAGAGTGACGGGC
TP36659_Query	D	1	chr8	.	CAGCGATGATGGCGTTGCACGTGAACACCGTAGGCCTGGTTGATGAGAACACAGAGTGACGAGC
TP36855_Hit	D	1	chr8	.	CAGCGATTGCGTCCATGTGCGTTCAACCAGAAGTATCTCAACGACCTTTCATGGGAGAAGTTGT
TP36855_Query	D	1	chr8	.	CAGCGATTGCGTCCATGTGCGTTCAACCAGAAGTATCTCAACGACCATTCATGGGAGAAGTTGT
TP36994_Hit	D	1	chr8	.	CAGCGCAAGTTTCCTGCTTCCAGAGTCCAAACTACAAATGCAATTTAGTTGTAGTTTGGACTAT
TP36994_Query	D	1	chr8	.	CAGCGCAAGTTTCCTGCTTCCAGAGTCCAAACTACAAATGCAATTTAATTGTAGTTTGGACTAT
TP37104_Hit	D	1	chr8	.	CAGCGCAGATCCAAGCAACGCAGATCGGATGATGTCACCTCTGTAGTAGCTCCAGCGCCGGTGG
TP37104_Query	D	1	chr8	.	CAGCGCAGATCCAAGCAACGCAGATCGGACGATGTCACCTCTGTAGTAGCTCCAGCGCCGGTGG
TP37766_Hit	D	1	chr8	.	CAGCGCGATTGTGATTTAAAACAATGCTTGGAAGAAATTGAGAATAAAGTTGAGCATGTTATTA
TP37766_Query	D	1	chr8	.	CAGCGCGATTGTGATTTAAAACAATGCTTGGAAGAAATCGAGAATAAAGTTGAGCATGTTATTA
TP38118_Hit	D	1	chr8	.	CAGCGCTCAATACAGTCTCTTTGGAAGCGCAACAGCAACAACCGCCCATCGTGCCTTATCTCCA
TP38118_Query	D	1	chr8	.	CAGCGCTCAATACAGTCTCTTTGGAAGCACAACAGCAACAACCGCCCATCGTGCCTTATCTCCA
TP38253_Hit	D	1	chr8	.	CTGCGCTTATCGCAAAAACAGAGTCTGAAGTATCCTATTGATGGCAACCAAGCTGTTTTCATAT
TP38253_Query	D	1	chr8	.	CAGCGCTTATCGCAAAAACAGAGTCTGAAGTATCCTATTGATGGCAACCAAGCTGTTTTCATAT
TP38255_Hit	D	1	chr8	.	CAGCGCTTATTAGTGGAGAATATGATGTGGTAGAGTTACAAAGTAGTTGGTTTGTTTGGGTGTG
TP38255_Query	D	1	chr8	.	CAGCGCTTATTAGTGGAGAATATGATGTGGTAGAGTTAAAAAGTAGTTGGTTTGTTTGGGTGTG
TP38414_Hit	D	1	chr8	.	CAGCGGAAGAGCTTCAGATGGAGTTGGAAAGTTCTCATTTGATAGCCGTTGAGACAAGGTTGTT
TP38414_Query	D	1	chr8	.	CAGCGGAAGAGCTTCAGATGGAGTTGGAAAGTTCTCATTCGATAGCCGTTGAGACAAGGTTGTT
TP38417_Hit	D	1	chr8	.	CAGCGGAAGATAAGGTTGAGGAACATAACCATGTTTTCTCAACTCAAGAAGAAGATTCTTGAGA
TP38417_Query	D	1	chr8	.	CAGCGGAAGATAAGGTTGAGGAACATAACCATGTTTTCTCAACTCAAGAAGAAGATTCTTAAGA
TP38629_Hit	D	1	chr8	.	CAGCGGAGGCTGGACACTCTCTGGCACGCCTTTGGACTTGCGTGATCCTCGATTGTTGGCAATT
TP38629_Query	D	1	chr8	.	CAGCGGAGGCTGGACACTCTCTGGCACGCCTTTGGACTTGCGTGATCCTCGACTGTTGGCAATT
TP38781_Hit	D	1	chr8	.	CAGCGGCAAATGCAGGAACTGATAACCAGGTAACTTTTCATCTTATTTTTTCTCCGTTGGGGGC
TP38781_Query	D	1	chr8	.	CAGCGGCAAATGCAGGAACTGATAACCAGGTAACTTTTCATCTTATTTTTTCTCCGTTGGGAGC
TP38836_Hit	D	1	chr8	.	CAGCGGCACAGAACATTCCAACTCTAATCATTGGTAGAGTCTTCCTTGGTGGAGGCATTGGCTT
TP38836_Query	D	1	chr8	.	CAGCGGCACAGAACATTCCAACTCTAATCATTGGTAGAATCTTCCTTGGTGGAGGCATTGGCTT
TP39035_Hit	D	1	chr8	.	CAGCGGCCTTATAATTATTATTACTATTTTCTTCTGAGAAGATGGCAGAGACAGTCTCCTGAGC
TP39035_Query	D	1	chr8	.	CAGCGGCCTTATAATTATTATTACTATTTTCTTCTGAGAAGATGGCAGAGACAGCCTCCTGAGC
TP39149_Hit	D+G	1	chr8	.	CAGCGGCGGAGAAGAAGCGTCATCCAGGTGAGTCAAAGGGGTTTGTGGAGGAGATGAGGTTTGT
TP39149_Query	D+G	1	chr8	.	CAGCGGCGGAGAAGAAGCGTCATCCAGGTGAGTCAAAGGGGTTTGTGGAGGAAATGAGGTTTGT
TP39155_Hit	D	1	chr8	.	CAGCGGCGGAGGGGAAAGCATTCGCCCAAGGATTAGTGGTGGAGATTCTAGACGCAACTCCTGG
TP39155_Query	D	1	chr8	.	CAGCGGCGGAGGGGAAAGCATTCGCCCAAGGATTAGTGGTGGAGATTCTAGACGCAACTCCAGG
TP39196_Hit	D	1	chr8	.	CAGCGGCGGCGTCGTTTCGGCGTGGTGTTGTGAATAGTTTGGAGTTTTACTCGATGATGCTGGA
TP39196_Query	D	1	chr8	.	CAGCGGCGGCGTCGTTTCGGCGTGGGGTTGTGAATAGTTTGGAGTTTTACTCGATGATGCTGGA

Name	Filter	Nb hit (Mt4.0)	Mt Chr (Mt4.0)	Ms Chr (Li et al., 2014)	Sequence
TP39760_Hit	D	1	chr8	.	CAGCGGTCCTGGGATTCTTGTAGGCGATAGAGGCCTGGGGTCCCAGCAGGCGGTGGCGGCCCTG
TP39760_Query	D	1	chr8	.	CAGCGGTCCTGGGATTCTTGTAGGCGATAGAGGCCTGGGGTCCCAGCAGGCGGGGGCGGCCCTG
TP3979_Hit	D	1	chr8	.	CTGCAACCAGAGCTTTGTGAAGAGAATTCATGGCAGGAACAAGAACGGGAGCAACATTTGGGAT
TP3979_Query	D	1	chr8	.	CAGCAACCAGAGCTTTGTGAAGAGAATTCATGGCAGGAACAAGAACGGGAGCAACATTTGGGAT
TP39989_Hit	D	1	chr8	.	CAGCGGTTAGGAGGAGTGCAAGGAAGAGACTCCCTAACCAATTGATGGATGATTTTGTGTGCAA
TP39989_Query	D	1	chr8	.	CAGCGGTTAGGAGGAGTGCAAGGAAGAGACTCCCTAACCAAATGATGGATGATTTTGTGTGCAA
TP4002_Hit	D	1	chr8	.	CAGCAACCATAAGTGCAGAATATGCCCAGCAACACATATAGACTGAACTTGTGATTTTGACGTA
TP4002_Query	D	1	chr8	.	CAGCAACCATAAGTGCAGAATATGCCCAGCAACACAGATAGACTGAACTTGTGATTTTGACGTA
TP40032_Hit	D	1	chr8	.	CAGCGGTTCTTGGCGATGGATTCATGGCAAAGGATCTAACGATCGAAAACACAGCAGGTCCTGA
TP40032_Query	D	1	chr8	.	CAGCGGTTCTTGGCGATGGATTCATGGCAAAAGATCTAACGATCGAAAACACAGCAGGTCCTGA
TP40164_Hit	D+G	1	chr8	.	CAGCGTACGAACGTTTTGAGCCAGATAGAGAATCATAATCACGAGGGCGTCCTTCACGATAACT
TP40164_Query	D+G	1	chr8	.	CAGCGTAAGAACGTTTTGAGCCAGATAGAGAATCATAATCACGAGGGCGTCCTTCACGATAACT
TP40395_Hit	D	1	chr8	.	CAGCGTCGAAAACCTTCAAACAAATCGTATCGGTCAAGCTCGACGATACGAACTACCTTCAGTG
TP40395_Query	D	1	chr8	.	CAGCGTCAAAAACCTTCAAACAAATCGTATCGGTCAAGCTCGACGATACGAACTACCTTCAGTG
TP40526_Hit	D	1	chr8	.	CAGCGTCCCCTTTGAGGTCCATTGAACAATTTGACGAAAGGATCCTATAAAGAACATCTACTGC
TP40526_Query	D	1	chr8	.	CAGCGTCCCCTTTGAGGTCCATTGAACAATTTGACGAAAGGATCCTATAAAGAACATCTACTGA
TP40542_Hit	D+G	1	chr8	.	CAGCGTCCTAGGTTGGTTTTGGATCCAAGGAAAGGTGATGGTTCTGTCAATGAATCTCTGGTGG
TP40542_Query	D+G	1	chr8	.	CAGCGTCCTAGGTTGGTTTTGGATCCAAGGAAAGGTGATGGTTCTGTCAATGAATCTCCGGTGG
TP40887_Hit	D+G	1	chr8	.	CAGCGTGCAGGTCAGCGCCTCATACAAGGTATATGTTGATAACTTGTTTGAACAAAATACTCAA
TP40887_Query	D+G	1	chr8	.	CAGCGTGCAGGTCAGCGCCTCATACAAGGTACATGTTGATAACTTGTTTGAACAAAATACTCAA
TP409_Hit	D	1	chr8	.	CAGCAAAACCAATACAAACAATCCACTCTCTCCAATTCAACCTCTCAGTATTAGCAAATTTCTT
TP409_Query	D	1	chr8	.	CAGCAAAACCAATACAAACAATCCACTCTCTCCAATTCAACCTCTCAGTATTAGCAAACTTCTT
TP41079_Hit	D	1	chr8	.	CAGCGTGTAATGAATAGATAATGAAATCAGTTGGTTAGAATTCAAACTGCTGTTGTAAAAACAA
TP41079_Query	D	1	chr8	.	CAGCGTGTAATGAATAGATAATGAAATAAGTTGGTTAGAATTCAAACTGCTGTTGTAAAAACAA
TP41128_Hit	D	1	chr8	.	CAGCGTGTGATTACGTGGAGAATCTGTCGAATGGTTCGGCTCTTGAAGTGTTGCCATCAGCTAA
TP41128_Query	D	1	chr8	.	CAGCGTGTGATTACGTGGAGAATCTGTCGAATGGTTCAGCTCTTGAAGTGTTGCCATCAGCTAA
TP41289_Hit	D	1	chr8	.	CAGCGTTATGTCTCGCTGAATTTTCTAAAAGCTCGGTTAGTCTCAGCAGACCGATTCCGAAGCA
TP41289_Query	D	1	chr8	.	CAGCGTTATGTCTCGCTGAATTTTCTAAAAGCTCGGTTAGTCTCAGCAGACCGATTCCAAAGCA
TP41300_Hit	D	1	chr8	.	CAGCGTTCCACAAGGAAGTATAGCTTCTTCATACTTTTGGTCATGAATACACTGCATGTGAAAA
TP41300_Query	D	1	chr8	.	CAGCGTTCAACAAGGAAGTATAGCTTCTTCATACTTTTGGTCATGAATACACTGCATGTGAAAA
TP41384_Hit	D	1	chr8	.	CAGCGTTGAATTCAGTTTTGTAGCCAAACAAGAATTTAAATGATGAAACAAGACCATGACCATG
TP41384_Query	D	1	chr8	.	CAGCGTTGAACTCAGTTTTGTAGCCAAACAAGAATTTAAATGATGAAACAAGACCATGACCATG
TP4166_Hit	D+G	1	chr8	.	CAGCAACCTCAACAACTATATTGATTATCTCAGGTTTATCTTTACTCAAAATCGGGGGAAGAAT
TP4166_Query	D+G	1	chr8	.	CAGCAACCTCAACAACTATATTGATTATCTCAGGTTTATCTTTACTAAAAATCGGGGGAAGAAT
TP41842_Hit	D	1	chr8	.	CAGCTAAACAAGGGCTAACCTTGACAAAGAACAGGGAGGTGGGAAAGTTCCAAGGAATAATGTG
TP41842_Query	D	1	chr8	.	CAGCTAAACAAGGGCTAACCTTGACAAAGAACAGGGAAGTGGGAAAGTTCCAAGGAATAATGTG
TP41951_Hit	D	1	chr8	.	CAGCTAAAGGAAGAGATGTGTTCAGTGAAAAACCTTCCAGTGATGAACTTTTGTCTGTTTCAAG
TP41951_Query	D	1	chr8	.	CAGCTAAAGGAAGAGATGTGTTCAGTGAAAAACCTTCCAGTGATGAACTTTTGGCTGTTTCAAG
TP42003_Hit	D	1	chr8	.	CAGCTAAATAATCGGTCATTCAACCTTCTAATTATGATTCCATTCCCATTTATTCTTCTGGGTC
TP42003_Query	D	1	chr8	.	CAGCTAAATAATCGGTCATTCAACCTTCTAATTATGATCCCATTCCCATTTATTCTTCTGGGTC
TP42421_Hit	D	1	chr8	.	CAGCTAAGCTTTTTTTTTCTTTCCCCATGATGTTTCTTCTACAGGGAGTTGGAAATACACTCAA
TP42421_Query	D	1	chr8	.	CAGCTAAGCCTTTTTTTTCTTTCCCCATGATGTTTCTTCTACAGGGAGTTGGAAATACACTCAA
TP42482_Hit	D	1	chr8	.	CAGCTAAGGTATCTTTGAGTGAGGTCCGATCCTCACTGAAAGCAATATTTTCCTTGGAGACATT
TP42482_Query	D	1	chr8	.	CAGCTAAGGTATCTTTGAGTGAGGTCCGATCCTCACTGAAAGCAATATTTTCCTTGGAGACATG
TP42712_Hit	D+G	1	chr8	.	CAGCTCATGCATTATTGTTGCAGGCGAGGTCATTAGTGAGAGAGATAGATGCCCTCAATGCAAG
TP42712_Query	D+G	1	chr8	.	CAGCTAATGCATTATTGTTGCAGGCGAGGTCATTAGTGAGAGAGATAGATGCCCTCAATGCAAG
TP42729_Hit	D	1	chr8	.	CAGCTAATGGAGATAATGCTGGTGATTCTAAATCTCATCTGGGATATAATGGAGCACCATTGCC
TP42729_Query	D	1	chr8	.	CAGCTAATGGAGATAATGCCGGTGATTCTAAATCTCATCTGGGATATAATGGAGCACCATTGCC
TP42805_Hit	D	1	chr8	.	CAGCTAATTGAAGACACTAACATAATTCTATTTATAAAAGTTAATTAAGCTTGATTCATGAAAT
TP42805_Query	D	1	chr8	.	CAGCTAATTGAAGACACTAACATAATTCTATTTATAAAAGTTAAATAAGCTTGATTCATGAAAT
TP43050_Hit	D	1	chr8	.	CAGCTACACGGGCAGAGAGAGAGGGCATATCCTGGAAATCTCTACACAATCCCGCATTTCGAAA
TP43050_Query	D	1	chr8	.	CAGCTACACGGGCAGAGAGAGAGGGCATATCCTGGAAATCTCTACACAATCCCGCATATCGAAA
TP43064_Hit	D+G	1	chr8	.	CAGCTACACTTCCGGTGAATGGAAAGAAAACAGCGGGAGCATTATTGAAACACATGTTTCCTGG
TP43064_Query	D+G	1	chr8	.	CAGCTACACTTCCGGTGAATGGAAAGAAAACAGCAGGAGCATTATTGAAACACATGTTTCCTGG

Name	Filter	Nb hit (Mt4.0)	Mt Chr (Mt4.0)	Ms Chr (Li et al., 2014)	Sequence
TP43098_Hit	D	1	chr8	.	CAGCTACAGCAGTGATGAACTTCTTTGAGCCTGCTTTTATGTTCATTCAGAGATGGCTCCCTTT
TP43098_Query	D	1	chr8	.	CAGCTACAGCAGTGATGAACTTCTTTGAGCCTGCGTTTATGTTCATTCAGAGATGGCTCCCTTT
TP43133_Hit	D	1	chr8	.	CAGCTACAGTTTTATCATCATTAACTCGAACATAGAATGGTTTAGCTTCTTTCGGATAACTGTG
TP43133_Query	D	1	chr8	.	CAGCTACAGTTTTATCATCATTAACACGAACATAGAATGGTTTAGCTTCTTTCGGATAACTGTG
TP43139_Hit	D	1	chr8	.	CAGCTACATAACAAAAACCTCATTTTATAAATTACTTAAGCTAGAAGAAAAATCACAATTTACA
TP43139_Query	D	1	chr8	.	CAGCTACATAACAAAAACCTCATTTTATAAATTACTTAAGCTAGAAGAAAAATCACAAGTTACA
TP43149_Hit	D	1	chr8	.	CAGCTACATAGATAGATAGATAGATAACATGCATAAAGGTACTTAACTTAACAAGTGATGCTTA
TP43149_Query	D	1	chr8	.	CAGCTACATAGATAGATAGATAGATAACATGCATAAAGGTACTTAACATAACAAGTGATGCTTA
TP43229_Hit	D	1	chr8	.	CAGCTACATTTTGAAGTGTACGTCGAAGAAGGCGAATGTTGGAAAGATAACTCCAATCTTTGAC
TP43229_Query	D	1	chr8	.	CAGCTACATTTTGAAGTGTACGTCGAAGAAGACGAATGTTGGAAAGATAACTCCAATCTTTGAC
TP43258_Hit	D	1	chr8	.	CAGCTACCACCGGATTCAACAGGAGAGCATATAATACTAACTGTTTTCTCCCTTATCCTTGTTG
TP43258_Query	D	1	chr8	.	CAGCTACCACCGGATTCAACAAGAGAGCATATAATACTAACTGTTTTCTCCCTTATCCTTGTTG
TP43297_Hit	D	1	chr8	.	CAGCTATCCCATTAATATATTATTTGGCTACACCAAACAATTTTGTTGTTTTAAAGGGAGATGT
TP43297_Query	D	1	chr8	.	CAGCTACCCCATTAATATATTATTTGGCTACACCAAACAATTTTGTTGTTTTAAAGGGAGATGT
TP43310_Hit	D	1	chr8	.	CAGCTACCCTCAGAGAAATTGGAGTCACGGGGCATTTATTTGGAACTTCTACTCCCAAACCACC
TP43310_Query	D	1	chr8	.	CAGCTACCCTCAGAGAAATTGGAGTCACGGGACATTTATTTGGAACTTCTACTCCCAAACCACC
TP43341_Hit	D	1	chr8	.	CAGCTACCTCTAATCTGAAACTGTGCTAGACACAACTTACAAGCTATAACTCACTGTTTTGCCC
TP43341_Query	D	1	chr8	.	CAGCTACCTCTAATCTGAAACTGTGCTAGACACAACTTACAAGCTATAACTCACTGTTTTGCAC
TP43516_Hit	D	1	chr8	.	CAGCTACTATGAATTATCTCTACTGTGCTGACCACCGATTTCCACTGTCAGGCGTATAAGCATA
TP43516_Query	D	1	chr8	.	CAGCTACTATGAATTATCTCTAATGTGCTGACCACCGATTTCCACTGTCAGGCGTATAAGCATA
TP43613_Hit	D	1	chr8	.	CAGCTACTGCCGGTCATGGACTCATAACCGGCATGACAGGTGAATCTGTCACGGCCGCTGAAAA
TP43613_Query	D	1	chr8	.	CAGCTACTGCCAGTCATGGACTCATAACCGGCATGACAGGTGAATCTGTCACGGCCGCTGAAAA
TP43643_Hit	D	1	chr8	.	CAGCTACTTACGATGCAATGGGAAGGTATAATTCAATTTCCTTTCTCTTTTTAACAATCTTATG
TP43643_Query	D	1	chr8	.	CAGCTACTTACGATGCAATGGGAAGGTATAATTCAATTCCCTTTCTCTTTTTAACAATCTTATG
TP43660_Hit	D	1	chr8	.	CAGCTACTTGACGGCCGACTCTGAAATCAAGAATAAAACAACTTACCTCTTGTGGAAGTAGTGG
TP43660_Query	D	1	chr8	.	CAGCTACTTGACGGCCGACTCTGAAATCAAGAATAAAACAACTTACCTCTTGTGGAAGCAGTGG
TP43786_Hit	D	1	chr8	.	CAGCTAGAATTGAGCTACCAGATTCCTTCTACAAACGTTCAGCTGAAGAGGTGAGAAGAGAAGC
TP43786_Query	D	1	chr8	.	CAGCTAGAATTGAGCTACCAGATTCCTTCTACAAACGTTCAGCTGAAGAGGTGAGAAAAGAAGC
TP43824_Hit	D	1	chr8	.	CAGCTAGAGAACAGCCTCCACCGCCACCACCTTCGGCAAAGCTCCCGCCAGCTGAAAAAAAAAA
TP43824_Query	D	1	chr8	.	CAGCTAGAGAACAGCCTCCACCGCCACCACCTTCGGCAAAGCTCCCGCCAGCAGAAAAAAAAAA
TP43873_Hit	D+G	1	chr8	.	CAGCTAGAGTACAAGCAAATTCTGAGGGATGATATTGATCCCAGAACAAGAAATGGCTTCGATT
TP43873_Query	D+G	1	chr8	.	CAGCTAGAGTACAAGCAAACTCTGAGGGATGATATTGATCCCAGAACAAGAAATGGCTTCGATT
TP44074_Hit	D	1	chr8	.	CAGCTAGCTAGCTGGCCTAATATCGTAGAACAGCGAAAGTCATTTAGTACTGTTTTCTTCTTAC
TP44074_Query	D	1	chr8	.	CAGCTAGCTAGCTGACCTAATATCGTAGAACAGCGAAAGTCATTTAGTACTGTTTTCTTCTTAC
TP44135_Hit	D	1	chr8	.	CAGCTAGGAGAAGACTTGGATAATCATGTACAAAATTTGTGACCATTCTTACAAACACTACGTG
TP44135_Query	D	1	chr8	.	CAGCTAGGAGAAGACTTGGATAATCATGTACAAAATTTGGGACCATTCTTACAAACACTACGTG
TP44141_Hit	D	1	chr8	.	CAGCTAGGAGTAAGAATTCTGAGGTATTCAACATGATTCTTGATTTTGCTCTCTTGGGAAAAGA
TP44141_Query	D	1	chr8	.	CAGCTAGGAGTAAGAATTCTGAGGTATTCAACATGATTCTTGATTTTGCTCTCTTAGGAAAAGA
TP44183_Hit	D	1	chr8	.	CAGCTAGGGTCACAAGAATACAATGCAAATAGCCAATACACCAGAAAGCAATTAGCCAAATCCA
TP44183_Query	D	1	chr8	.	CAGCTAGGGACACAAGAATACAATGCAAATAGCCAATACACCAGAAAGCAATTAGCCAAATCCA
TP44259_Hit	D	1	chr8	.	CAGCTAGTAAAAGGTTGCAAGGACATTTGATATGTCAGGACGCAAATTCGTATCCGCATGTCCC
TP44259_Query	D	1	chr8	.	CAGCTAGTAAAAGGTTGCAAGGACATTTGATATGTCAGGACGCAAATTCATATCCGCATGTCCC
TP44414_Hit	D	1	chr8	.	CAGCTAGTTCCTAGATGAAACAACAAACATTATCAACATGAAACTCTACATGTCCGTTTTCTTT
TP44414_Query	D	1	chr8	.	CAGCTAGTTCCTAGATGAAACAACAAACATTATCAACATGAAACTCTACATGTCCATTTTCTTT
TP44697_Hit	D	1	chr8	.	CAGCTATAGTAGTTCACCTTGTGGGAGCATATCAAGTATTTTCCCAACCAATCTTTGCCTTTGT
TP44697_Query	D	1	chr8	.	CAGCTATAGTAGTTCACCTTGTGGGAGCATATCAAGTATTTTCCCAACCAATCTTTGCCTTTAT
TP447_Hit	D	1	chr8	.	CAGCAAAACCTCGGGTGAGTTAGCTAGAGTCCTGGCAAGTGCAACTCTTTGAGCCTGACCCACA
TP447_Query	D	1	chr8	.	CAGCAAAACCTCGGGTGAGTTAGCTAGAGTCCTGGCAAGTGCAACTCTTTGAGCCTGACCAACA
TP44736_Hit	D	1	chr8	.	CAGCTATATATAAACTATATACGCACACCCTCCCCGATTATTCATTCATATTTATATATCATGC
TP44736_Query	D	1	chr8	.	CAGCTATATATAAACTATATACGCACACCCTCCCCCATTATTCATTCATATTTATATATCATGC
TP44913_Hit	D+G	1	chr8	.	CAGCTATCCAGGATTGACAACCAAGTTTAAAACCACCATTTTCGCCATGCACTGTTATTTCTTT
TP44913_Query	D+G	1	chr8	.	CAGCTATCCAGGATTGACAACCAAGTTTAAAACCACCATTTTCGCCATGCACTGTTACTTCTTT
TP44922_Hit	D	1	chr8	.	CAGCTATCCCGGGTTCAGTAACAAATGTAAGAGCTTACCGTCTTTGAAAATTATTATTGAACTG
TP44922_Query	D	1	chr8	.	CAGCTATCCCGGGTTCAGCAACAAATGTAAGAGCTTACCGTCTTTGAAAATTATTATTGAACTG

Name	Filter	Nb hit (Mt4.0)	Mt Chr (Mt4.0)	Ms Chr (Li et al., 2014)	Sequence
TP44960_Hit	D	1	chr8	.	CAGCTATCTATTTGATGCCATCTACAAAAAACCAGTTATAATTCACAGTTATCCAAAAGAAGCC
TP44960_Query	D	1	chr8	.	CAGCTATCTAGTTGATGCCATCTACAAAAAACCAGTTATAATTCACAGTTATCCAAAAGAAGCC
TP45103_Hit	D	1	chr8	.	CAGCTATGCAACTTCTCACTGCCAAAGGCGTGTTAGATAGTTGCTTCTATGCTTTGAAAATAAG
TP45103_Query	D	1	chr8	.	CAGCTATGCAACTTCTCACTGCCAAAGGCATGTTAGATAGTTGCTTCTATGCTTTGAAAATAAG
TP45131_Hit	D+G	1	chr8	.	CAGCTATGCCTTCCACTCCCTTGGATTATCACATCTCAGTATCATTTCTTGGCCAATACCTCCC
TP45131_Query	D+G	1	chr8	.	CAGCTATGCCTTCCACTCCCTTGGATTATCACATCTCAGTATCATTTCTTAGCCAATACCTCCC
TP45158_Hit	D	1	chr8	.	CAGCTATGGAAGTTATGGAGAGAGCAGTAACATGTTTGCTTATTATTCACCTATCGCTCCATCT
TP45158_Query	D	1	chr8	.	CAGCTATGGAAGCTATGGAGAGAGCAGTAACATGTTTGCTTATTATTCACCTATCGCTCCATCT
TP45377_Hit	D	1	chr8	.	CAGCTATTATCGCTGGTGCAGGTCAAGGACACACCTCCTTTTGTTTTGACCCCGGTGCTGAAAA
TP45377_Query	D	1	chr8	.	CAGCTATTATCGCTGGTGCAGGTCAAGGACACGCCTCCTTTTGTTTTGACCCCGGTGCTGAAAA
TP45393_Hit	D	1	chr8	.	CAGCTATTCAATATCCGAAGAACAGTGAGGAGTTGGGTCGTTGTAGAGGGCCTGAAGTTATTTC
TP45393_Query	D	1	chr8	.	CAGCTATTCAATATCCGAAGAACAGTGAGGAGTTGGGTCGTTGTAGAGGGCCCGAAGTTATTTC
TP45466_Hit	D+G	1	chr8	.	CAGCTATTGCATATGGATCTCTTCTTCTTAAAGGTCAGCTCATGTTCTGCATTCACTTTAGGAC
TP45466_Query	D+G	1	chr8	.	CAGCTATTGCATATGGATCTCTTCTTCTTAAAGGTCAGCTCATGTTCTGCATTCACTTTAGAAC
TP45499_Hit	D	1	chr8	.	CAGCTATTGGGTTTGGTCTTCCAAAGGACGCATTCAGGTCTCTTATGAAGCTGGTGAGTGAGCA
TP45499_Query	D	1	chr8	.	CAGCTATTGGGTTTGGTCTTCCAAAGGACGCATTCACGTCTCTTATGAAGCTGGTGAGTGAGCA
TP45652_Hit	D	1	chr8	.	CAGCTATTTTTCTTGCATCAGATTTGTTTAAGTACTAGTCAATGTGCATCTTGCCAGTATTGTA
TP45652_Query	D	1	chr8	.	CAGCTATTTTTCTTGCATCAGATTTGGTTAAGTACTAGTCAATGTGCATCTTGCCAGTATTGTA
TP45786_Hit	D+G	1	chr8	.	CAGCTCAAATATTCCTCGGGAAGTAGAAACTTTGAAAGGGTTGAGAGCAACCAGGGTTTCATGT
TP45786_Query	D+G	1	chr8	.	CAGCTCAAATATTCCCCGGGAAGTAGAAACTTTGAAAGGGTTGAGAGCAACCAGGGTTTCATGT
TP46062_Hit	D+G	1	chr8	.	CAGCTCAATCTTCTCACATGCAGTTTCCCGGCATGTACCACACTCCTCCACAACAAGCTCCTGT
TP46062_Query	D+G	1	chr8	.	CAGCTCAATCTTCTCACATGCAGTTTCCCGGCATGTACCACACTCCTCCACAACAAGCTCCTAT
TP4624_Hit	D	1	chr8	.	CAGCAACTCACGCAGATAAAGCTTGCTTTAATTCATCCTCAGTGCAGTTTGGACCAAGATTTGC
TP4624_Query	D	1	chr8	.	CAGCAACTCACGCAGATAAAGCTTGCTTTAATTCATCCTCAGTGCAATTTGGACCAAGATTTGC
TP46278_Hit	D	1	chr8	.	CAGCTCATTACAGAAATTTGATGAATCTAACTGAAGCCGTTTCTTGTTCTTGTATGTTATATCG
TP46278_Query	D	1	chr8	.	CAGCTCACTACAGAAATTTGATGAATCTAACTGAAGCCGTTTCTTGTTCTTGTATGTTATATCG
TP4657_Hit	D	1	chr8	.	CAGCAACTCCGGCTTTCTGGGATATACTACTAGGCCCTGAAGTGAACTAAATTCAATAAAACGC
TP4657_Query	D	1	chr8	.	CAGCAACTCCGGCTTTCTGGGATATACTACTAGGCCCTGAAGTGAACTAAATTCAATAAAACAC
TP46584_Hit	D+G	1	chr8	.	CAGCTCAGTTCTCCCTGAACAAAGTTAGGGCATGTGTTGTGTCGTACATTATCATACCGAAGAA
TP46584_Query	D+G	1	chr8	.	CAGCTCAGTTCTCCCCGAACAAAGTTAGGGCATGTGTTGTGTCGTACATTATCATACCGAAGAA
TP46646_Hit	D	1	chr8	.	CAGCTCATAGTCCTAGCTCAAATGCAAGTCCATCACCCAACCCTCACAATCAACTCAGAGACAC
TP46646_Query	D	1	chr8	.	CAGCTCATAGTACTAGCTCAAATGCAAGTCCATCACCCAACCCTCACAATCAACTCAGAGACAC
TP46648_Hit	D	1	chr8	.	CAGCTCATAGTCATATATTTCTTATGCTCATGCTCTTCAACGAAGTTTGCTTGAAGCTTATTTT
TP46648_Query	D	1	chr8	.	CAGCTCATAGTCATATATTTCTTATGCTCAGGCTCTTCAACGAAGTTTGCTTGAAGCTTATTTT
TP46753_Hit	D	1	chr8	.	CAGCTCATGTACTCCTACTTTTGCATCATTATTGTTTTTGGTGATATCATGATAATTTCTTAAT
TP46753_Query	D	1	chr8	.	CAGCTCATGAACTCCTACTTTTGCATCATTATTGTTTTTGGTGATATCATGATAATTTCTTAAT
TP47163_Hit	D	1	chr8	.	CAGCTCCAGAAGGAACTTATGATGCAGTTATAGTGGATTCATCTGATCCTATTGGTATGCTACA
TP47163_Query	D	1	chr8	.	CAGCTCCAGAAGGAACTTATGATGCAGTTATAGTGGATTCATCTGACCCTATTGGTATGCTACA
TP47262_Hit	D	1	chr8	.	CAGCTCCATCAAATGTATATAGAAAGCTGGGCCCCATTAAATTAAGTTCAAGAGCCCCGGATTT
TP47262_Query	D	1	chr8	.	CAGCTCCATCAAATGTATATAGAAAGCTGGGCCCCATTAAATTAAGTTCAAGAGCCCCGAATTT
TP47319_Hit	D+G	1	chr8	.	CAGCTCCATTAATGGACAAACTTGTATTCAGTAAGGTGATTGTAGTTTCACATATTTTAATTCG
TP47319_Query	D+G	1	chr8	.	CAGCTCCATTAATGGACAAACTTGTATTCAGTAAGGTGATTGTAGTTTCACATATTTTAATTCA
TP47343_Hit	D	1	chr8	.	CAGCTCCATTCTAAGAATCGTCCTCGGTTGAAATATGTATTTGGCACCTTCAACCTACAGTTTA
TP47343_Query	D	1	chr8	.	CAGCTCCATTCTAAGAATCGTCCTCGGTTGAAATATGTATTTCGCACCTTCAACCTACAGTTTA
TP47365_Hit	D	1	chr8	.	CAGCTCCCAAATCATTCTTTTCACTCTCGACTTTCCGAAGCAAGGGAAGTGACTCAAAACCCAG
TP47365_Query	D	1	chr8	.	CAGCTCCCAAATCATTCTTTTCACTCTCAACTTTCCGAAGCAAGGGAAGTGACTCAAAACCCAG
TP47485_Hit	D	1	chr8	.	CAGCTCCCTCATTACCAAAAGTCCCAATTCCTTGATGCCTACACATTCAGTTACGTGTCAAGGT
TP47485_Query	D	1	chr8	.	CAGCTCCCTCATTACCAAAAGTCCCAATTACTTGATGCCTACACATTCAGTTACGTGTCAAGGT
TP47504_Hit	D	1	chr8	.	CAGCTCCCTGGGAATTCACATGCCCCCATCTGTGAACCTTGGTGATTGGTTGGCTATTATGTGG
TP47504_Query	D	1	chr8	.	CAGCTCCCTGGGAATTCACATGCCCCCATCTATGAACCTTGGTGATTGGTTGGCTATTATGTGG
TP47743_Hit	D	1	chr8	.	CAGCTCCTCGCAAATTGTTGCCATAACCTGCGTAAAACATGAAGCTCAATGTATGAAATAACAA
TP47743_Query	D	1	chr8	.	CAGCTCCTCACAAATTGTTGCCATAACCTGCGTAAAACATGAAGCTCAATGTATGAAATAACAA
TP47808_Hit	D	1	chr8	.	CAGCTCCTTTCATATTCTTAATCGTTCTCGCTTTTCAATTTGCTTCTTATTGGATTGATAAATT
TP47808_Query	D	1	chr8	.	CAGCTCCTCTCATATTCTTAATCGTTCTCGCTTTTCAATTTGCTTCTTATTGGATTGATAAATT

Name	Filter	Nb hit (Mt4.0)	Mt Chr (Mt4.0)	Ms Chr (Li et al., 2014)	Sequence
TP47910_Hit	D+G	1	chr8	.	CAGCTCCTGTTGTTTTATTAATTAAATTTCAGCGCATAACGAAGCATGAAAGCAATATTGCCTT
TP47910_Query	D+G	1	chr8	.	CAGCTCCTGTTGTTTTATTAATTAAATTTCAGCACATAACGAAGCATGAAAGCAATATTGCCTT
TP47912_Hit	D+G	1	chr8	.	CAGCTCCTTAAATTTTAGAATGTTTAATACTTGTCTTAAGAAATCACATAATCAGCGTGATTAC
TP47912_Query	D+G	1	chr8	.	CAGCTCCTTAAATTTTAGAATGTTTAATACTTGTCTTAAGAAATCACATAATCAGAGTGATTAC
TP47926_Hit	D+G	1	chr8	.	CAGCTCCTTATTAGATTTTCATGTACTGGGTCGGAGATCTACCTATTTCTCCGTGTTGAATTTT
TP47926_Query	D+G	1	chr8	.	CAGCTCCTTATTAGATTTTCATGTACTGGGCCGGAGATCTACCTATTTCTCCGTGTTGAATTTT
TP47976_Hit	D	1	chr8	.	CAGCTCCTTGTGAAACACGTGATGGCATTGTCTTAACAGCACCATCCTTTGTTTCCTTCTTCCT
TP47976_Query	D	1	chr8	.	CAGCTCCTTGCGAAACACGTGATGGCATTGTCTTAACAGCACCATCCTTTGTTTCCTTCTTCCT
TP47986_Hit	D	1	chr8	.	CAGCTCCTTGTTACCTTAGAAGAAGAGACATTAAACCACTGAATCCAAGAATACATTGGAATGT
TP47986_Query	D	1	chr8	.	CAGCTCCTTGTTACCTTAGAAGAAGAGACATTAAACCACTAAATCCAAGAATACATTGGAATGT
TP48021_Hit	D	1	chr8	.	CAGCTCGAAAGCGGCTGGTTGATGAACTAAATAAGCCAGCTAGGGGAAAAAGGTACAAATTAGT
TP48021_Query	D	1	chr8	.	CAGCTCGAAAGCGGCTGGTTGATGAACTAAATAAGCCAGCTAGGGGAAAAAGGGACAAATTAGT
TP48067_Hit	D+G	1	chr8	.	CAGCTCGAATTTTCTGCAAGTGTATTCTAACTAACTGCCTCCATTGTCATGCTGATGTTGATCC
TP48067_Query	D+G	1	chr8	.	CAGCTCGAATTTTCTGCAAGTGTATTATAACTAACTGCCTCCATTGTCATGCTGATGTTGATCC
TP48138_Hit	D	1	chr8	.	CAGCTCGATAATTATGAAAAGATAATGAGTGTGCTGGACAGAGTTGGAGAAGAGAACGCCTTAG
TP48138_Query	D	1	chr8	.	CAGCTCGATAATTATGAAAAGATAATGAGTGTGCTGGACAGAGTTGGAGAAGAGAACACCTTAG
TP4819_Hit	D	1	chr8	.	CAGCAACTGTTTTGGGCTCACTTTGTAAAGAAAATGAACTGAGGGTGAAAGTCTTGCTTGGTGG
TP4819_Query	D	1	chr8	.	CAGCAACTGTTTTGGGCTCACTTTGTAAAAAAAATGAACTGAGGGTGAAAGTCTTGCTTGGTGG
TP48666_Hit	D	1	chr8	.	CTGCTCTAGATAGCCGGTAATTGAACCATTCCTCAAATTCACCAAATTCTTCAGCCCCTTGAAT
TP48666_Query	D	1	chr8	.	CAGCTCTAGATAGCCGGTAATTGAACCATTCCTCAAATTCACCAAATTCTTCAGCCCCTTGAAT
TP48795_Hit	D	1	chr8	.	CAGCTCTATTTTGTTTCTGATGCGCATCAACATGAGGTCACATGTGAACTTATGTTGGTGTATG
TP48795_Query	D	1	chr8	.	CAGCTCTATTTTGTTTCTGATGCGCATCAACATGAGGTCACATGTGAACTTATGTTGGCGTATG
TP48875_Hit	D	1	chr8	.	CAGCTCTCATTGCTCCCAACACTTATCGGGGGGGAAAAAATAAGACATGCTCTGAACCATAGAG
TP48875_Query	D	1	chr8	.	CAGCTCTCATTGCTCCCAACACTTATCGGGGGGAAAAAAATAAGACATGCTCTGAACCATAGAG
TP48964_Hit	D	1	chr8	.	CAGCTCTCTCAACCTTTACTTTTTCCATTGCACGCTCTCGAGCCTCTGCAGTTGCTCTTTCTAC
TP48964_Query	D	1	chr8	.	CAGCTCTCTCAACCTTTACTTTTTCCATTGCACGCTCTCGAGCCTCGGCAGTTGCTCTTTCTAC
TP49199_Hit	D	1	chr8	.	CAGCTCTGTACAAAGCTGAACCCCAGTGATTAAAACTCCATGATGCTTCTCTCTTAGTAAGCCA
TP49199_Query	D	1	chr8	.	CAGCTCTGTACAAAGCTGAACCCCAGTGATCAAAACTCCATGATGCTTCTCTCTTAGTAAGCCA
TP49204_Hit	D	1	chr8	.	CAGCTCTGTATGCTGATTTCTTCTACTATTACTTCATCAGGTAAACAATATTTATTTGTTGAAG
TP49204_Query	D	1	chr8	.	CAGCTCTGTATGCTGATTTCTTCTACTATTACTTCATCAGGTAAACAATATTTATTTGTGGAAG
TP49236_Hit	D	1	chr8	.	CAGCTTTGTGTTTAATGTAATTTTTCTAAAGTTATCTTATCTTGGCATTTCTATTCCTGTCACA
TP49236_Query	D	1	chr8	.	CAGCTCTGTGTTTAATGTAATTTTTCTAAAGTTATCTTATCTTGGCATTTCTATTCCTGTCACA
TP49500_Hit	D+G	1	chr8	.	CAGCTCTTGTAATAAAAGTTGACGAGCAAGGTAACCATCATGAAGACAGTCTTAATATCTACCC
TP49500_Query	D+G	1	chr8	.	CAGCTCTTGTAATAAAAGTTGACGAGCAAGGTAACCATCATGAAGACAGTCTCAATATCTACCC
TP49505_Hit	D+G	1	chr8	.	CAGCTCTTGTCACCCTGATCAACATCTTGAACCCCACCTTTGACCAAACAACCAACCTGAGCAC
TP49505_Query	D+G	1	chr8	.	CAGCTCTTGTCACCCTGATCAACATCCTGAACCCCACCTTTGACCAAACAACCAACCTGAGCAC
TP49610_Hit	D	1	chr8	.	CAGCTCTTTTCTCGTCTTTTATTACCAATGGAGCTCTCCTTGTTGCTACTGTCATTTCAATGTT
TP49610_Query	D	1	chr8	.	CAGCTCTTTTCTCATCTTTTATTACCAATGGAGCTCTCCTTGTTGCTACTGTCATTTCAATGTT
TP49910_Hit	D+G	1	chr8	.	CAGCTGAACCAAGAATCCAGGGCATTGCACGACCATGACCAAGAATGAGAGCATCTGCTGAAAA
TP49910_Query	D+G	1	chr8	.	CAGCTGAACCAAGAATCCAGGGCATTGCACGACCATGACCAAGAATGAAAGCATCTGCTGAAAA
TP50138_Hit	D	1	chr8	.	CAGCTGAAGTTAGGCTTCCTCCTTCTATTTCCAGGGATGAAAAAAAGAAGAGGGTTCACGAGCT
TP50138_Query	D	1	chr8	.	CAGCTGAAGTTAGGCTTCCTCCTTCTATTTCCAGGGACGAAAAAAAGAAGAGGGTTCACGAGCT
TP50220_Hit	D	1	chr8	.	CAGCTGAATGGTGCACCCCAAAGTTATTCGTTTAACCAATCTCTTGAAGAAACCAAAGAGAGAG
TP50220_Query	D	1	chr8	.	CAGCTGAATGGAGCACCCCAAAGTTATTCGTTTAACCAATCTCTTGAAGAAACCAAAGAGAGAG
TP50289_Hit	D	1	chr8	.	CAGCTGACAACCTACTTCAATTGGCTCCCGAGCAATCAGGTTACTATGTTTTGTTATCTAATAT
TP50289_Query	D	1	chr8	.	CAGCTGACAACCTACTTCAATTGGCTCCCGAGCAATCAGGTTACTATGTGTTGTTATCTAATAT
TP50298_Hit	D	1	chr8	.	CAGCTGACAATGTTTCCCAGCAGAGTTTGGAGAAAGACTCTGTTGCCTCGTTTCCTTCAACTGC
TP50298_Query	D	1	chr8	.	CAGCTGACAATGTTTCCCAGCAGAGTTTGGAGAAAGACTCTGTTGCCTCATTTCCTTCAACTGC
TP50494_Hit	D	1	chr8	.	CAGCTGAGCTGACTTTCTTGTTAAATCCTTGTGCATCAGTGTTGATTCTTGCGACACAACCATT
TP50494_Query	D	1	chr8	.	CAGCTGAGCCGACTTTCTTGTTAAATCCTTGTGCATCAGTGTTGATTCTTGCGACACAACCATT
TP50767_Hit	D+G	1	chr8	.	CAGCTGATCCAAAGAAAGGTGCAATAGGCACAGTTGCACCCATTGCCATTGGTTTCATTGTTGG
TP50767_Query	D+G	1	chr8	.	CAGCTGATCCAAAGAAAGGGGCAATAGGCACAGTTGCACCCATTGCCATTGGTTTCATTGTTGG
TP50778_Hit	D	1	chr8	.	CAGCTGATCCATTGTCTGGCTTGAAACTTGTTTGGGAGTGTGATGCTATAGCTCACTGTGTAAT
TP50778_Query	D	1	chr8	.	CAGCTGATCCATTGTCTGGCTTGAAACTTGTTTGGGAGTGTGATGCTATAGCTCACTGCGTAAT

Name	Filter	Nb hit (Mt4.0)	Mt Chr (Mt4.0)	Ms Chr (Li et al., 2014)	Sequence
TP51535_Hit	D	1	chr8	.	CAGCTGGCTTCATCTGGTCAACTGCAACAAAACTCTATGACCCTGAACCAACAACAGTTGTCCC
TP51535_Query	D	1	chr8	.	CAGCTGGCTTCATCTGGTCAACTGCAACAAAACTCTATGACCCTGAACCAACAACAATTGTCCC
TP51853_Hit	D	1	chr8	.	CAGCTGGTGAGATTATATGGGAAGACAAAACCAATGTGATTGACTTCTAGAAAATACTGAATGT
TP51853_Query	D	1	chr8	.	CAGCTGGTGAGATTATATGGGAAGACAAAACCAATGCGATTGACTTCTAGAAAATACTGAATGT
TP51902_Hit	D	1	chr8	.	CAGCTGGTGGAGGTAGCGAAAATTCAGACAATGCGTCAAATGTCACCATGTTGAATAATGAAAC
TP51902_Query	D	1	chr8	.	CAGCTGGTGGAGGTAGCAAAAATTCAGACAATGCGTCAAATGTCACCATGTTGAATAATGAAAC
TP51995_Hit	D	1	chr8	.	CAGCTGGTTTACAATTTTTAACGTGTAAGTTGCATACACGGTTCTGTTTGCTCCACAATTTTTT
TP51995_Query	D	1	chr8	.	CAGCTGGTTTACAATTTTTAACGTGTAAGTTGCATACACGGTTCTGTTTGCTCCACAATATTTT
TP52037_Hit	D+G	1	chr8	.	CAGCTGTAAATTCTCATGCAAGTGATACCGAACTTCGCATGATTGTTGATGGTGTAGTGGCACA
TP52037_Query	D+G	1	chr8	.	CAGCTGTAAATTCTCATGCAAGTGATACCGAACTTCGCATGATTGTTGATGGTGTAGTCGCACA
TP52067_Hit	D	1	chr8	.	CAGCTGTAAGATCTGTTGGAGTTGGCAGGAACTCACCTTCGAACTCTGGTGTTGAAGATACGGG
TP52067_Query	D	1	chr8	.	CAGCTGTAAGATCTATTGGAGTTGGCAGGAACTCACCTTCGAACTCTGGTGTTGAAGATACGGG
TP52343_Hit	D+G	1	chr8	.	CAGCTGTCCACGAAATGATAGTCATTTCCAAAAACGATATTGCACTTGTTTTCAGAAGTATGAA
TP52343_Query	D+G	1	chr8	.	CAGCTGTCCACCAAATGATAGTCATTTCCAAAAACGATATTGCACTTGTTTTCAGAAGTATGAA
TP52591_Hit	D	1	chr8	.	CAGCTGTGGAGATTAATCCTTCAAGGTAACTAGATAAATTTAAGGGGTTAACCTCCGCCGGCAC
TP52591_Query	D	1	chr8	.	CAGCTGTGGAGATTAATCCTTCAAGGTAACTAGATAAATTTAAGGGGTTAACCTCCGCCAGCAC
TP52593_Hit	D	1	chr8	.	CAGCTGTGGAGGAGGGTATTGTTCCAGGTAACTCAATTTTGTCATTTCTGATGGTATAACAAGT
TP52593_Query	D	1	chr8	.	CAGCTGTGGAGGAGGGTATTGTTCCAGGTAACTCAATTTTGTCATTTCTGATGGGATAACAAGT
TP52850_Hit	D	1	chr8	.	CAGCTGTTCAGATTTTATTGATTCAGGTAAAATTCGTACTCAACTCCTCCAGAATTTGTCAAAA
TP52850_Query	D	1	chr8	.	CAGCTGTTCAGATTTTATTGATTCAGGTAAAATTCGTACTCAACTCCTCCAAAATTTGTCAAAA
TP5287_Hit	D+G	1	chr8	.	CAGCAAGAGAGTATCAGGAATTGTTGGAGGATATGCGCGAACAGAAACTTGCGCCGAATTTGCC
TP5287_Query	D+G	1	chr8	.	CAGCAAGAGAGTATCAGGAATTGTTGGAAGATATGCGCGAACAGAAACTTGCGCCGAATTTGCC
TP52916_Hit	D+G	1	chr8	.	CAGCTGTTGAAGCATGTTCTTCATTTCTAGCTACAAAGTCTTCACTTTCGAGGAATGAAAATTT
TP52916_Query	D+G	1	chr8	.	CAGCTGTTGAAGCATGTTCTTCATTTCTAGCCACAAAGTCTTCACTTTCGAGGAATGAAAATTT
TP52918_Hit	D	1	chr8	.	CAGCTGTTGAAGGTATTGACAAACACTGGGGAGCATCTGCCTCTGGTTTGTAGATTAATTTCTT
TP52918_Query	D	1	chr8	.	CAGCTGTTGAAGGTATCGACAAACACTGGGGAGCATCTGCCTCTGGTTTGTAGATTAATTTCTT
TP53046_Hit	D	1	chr8	.	CAGCTGTTGTGGAAGTGAGTAGTAAATTTGGTGCAAAAGGCCATACGTCACAACCAGAAGAAGG
TP53046_Query	D	1	chr8	.	CAGCTGTTGTGGAAGTGAGTAGTAAATTTGGTGCAAAAGGCCAGACGTCACAACCAGAAGAAGG
TP5307_Hit	D+G	1	chr8	.	CAGCAAGAGCATGATCGGCACCACGCTCAACATCAGGAACATGGATCGCGCGTTCGAGAACAGG
TP5307_Query	D+G	1	chr8	.	CAGCAAGAGCATGATCAGCACCACGCTCAACATCAGGAACATGGATCGCGCGTTCGAGAACAGG
TP53072_Hit	D	1	chr8	.	CAGCTGTTGTTTCCCTTTGGTCTTTATCTCATAATTGTTTTCACGTTTATGAATTTGGCTTCCT
TP53072_Query	D	1	chr8	.	CAGCTGTTGTTTCCCTTTGGTCTTTATCTCATAATTGTTTTCACGTTTATGAATTAGGCTTCCT
TP53239_Hit	D	1	chr8	.	CAGCTTAAACCAACCATGTAGTGACAAAATAAAGTCTCTTTGAAATTTTATGATCCGGATAAAA
TP53239_Query	D	1	chr8	.	CAGCTTAAACCAACCACGTAGTGACAAAATAAAGTCTCTTTGAAATTTTATGATCCGGATAAAA
TP53488_Hit	D	1	chr8	.	CAGCTTAATTATTTCCGATGGGACAAAAAGGAAGAATCCTCGTCAGACTTCTTCAGATTTTGCC
TP53488_Query	D	1	chr8	.	CAGCTTAATTATTTCCAATGGGACAAAAAGGAAGAATCCTCGTCAGACTTCTTCAGATTTTGCC
TP53666_Hit	D	1	chr8	.	CAGCTTACGGACCTAATGAAGAACATATTTGCATTGAAAATCGCGAATATGATATTGCAGAACT
TP53666_Query	D	1	chr8	.	CAGCTTACGGACCTAATGAAGAACATATTTGCGTTGAAAATCGCGAATATGATATTGCAGAACT
TP53915_Hit	D	1	chr8	.	CAGCTTATAATCACTTCGTTGATGCAGTAACTTTTGACACAACGTATCGGACAAATCATTACCA
TP53915_Query	D	1	chr8	.	CAGCTTATAATCACTTCGGTGATGCAGTAACTTTTGACACAACGTATCGGACAAATCATTACCA
TP53942_Hit	D	1	chr8	.	CAGCTTATACGTTTTTCTCTCAACATACTACATGAAAGTGTAGCAAGTTGGTGAAGTTACTTTA
TP53942_Query	D	1	chr8	.	CAGCTTATACGTTTTTCTCTCAACATACTACATGAAAGTGTAGCAAGCTGGTGAAGTTACTTTA
TP54262_Hit	D	1	chr8	.	CAGCTTATTCCCTGTTATTTTGATTGTAGGGAACCCCCAAAATCACACAATTTGAGCAAATAAA
TP54262_Query	D	1	chr8	.	CAGCTTATTCCCTATTATTTTGATTGTAGGGAACCCCCAAAATCACACAATTTGAGCAAATAAA
TP54306_Hit	D	1	chr8	.	CAGCTTATTGTCTTCAGTACATGCACCATGATCTTAATCCACCAGTCTCTCATTCCAACCTTAA
TP54306_Query	D	1	chr8	.	CAGCTTATTGTCTTCAGTACATGCACCATGATCTCAATCCACCAGTCTCTCATTCCAACCTTAA
TP54339_Hit	D+G	1	chr8	.	CAGCTTATTTCTGAAGCTTATGATGTTTTGAAGCATGTTGGTGGGTTGTCCAATTCTGAACTTG
TP54339_Query	D+G	1	chr8	.	CAGCTTATTTCTGAAGCTTATGATGTTTTGAAGCATGTTGGTGGATTGTCCAATTCTGAACTTG
TP54398_Hit	D	1	chr8	.	CAGCTTCAAACATAGATTTGTATTTGATCCATTGTGTTTTTGCCCTTTCTGTGATCAGCCACTG
TP54398_Query	D	1	chr8	.	CAGCTTCAAACATAGATTTGTACTTGATCCATTGTGTTTTTGCCCTTTCTGTGATCAGCCACTG
TP54463_Hit	D	1	chr8	.	CAGCTTCAACAGAGACGTGAAAATCATTTGGATGATCGTCAGAAGACATCTGTTGGTTTATCTA
TP54463_Query	D	1	chr8	.	CAGCTTCAACAGAGACGTGAAAATCATTTGGATGATCGTCAGAAGACATCTGTTGGTTCATCTA
TP54464_Hit	D	1	chr8	.	CAGCTTCAACAGATAATAGCAAGACTGGTAGAAGCCATAAATACTTATGACGGGCTTTTGAATC
TP54464_Query	D	1	chr8	.	CAGCTTCAACAGATAATAGCAAGACTGGTAGAAGCCATAAATACTTATGACGGGCATTTGAATC

Name	Filter	Nb hit (Mt4.0)	Mt Chr (Mt4.0)	Ms Chr (Li et al., 2014)	Sequence
TP54491_Hit	D	1	chr8	.	CAGCTTCAACCCACGAGTTCAAAGAAGCATTGTATGAATAAACTTCTTCAGTAGCATCTTCAGA
TP54491_Query	D	1	chr8	.	CAGCTTCAACCCACGAGTTCAAAGAAGCATTGTATGAATAAACTTCGTCAGTAGCATCTTCAGA
TP54535_Hit	D	1	chr8	.	CAGCTTCAAGATCAAATTTCACAAATGAGTGTATACGAGAATCAAAATGTGATCATAGCACAAC
TP54535_Query	D	1	chr8	.	CAGCTTCAAGATCAAATATCACAAATGAGTGTATACGAGAATCAAAATGTGATCATAGCACAAC
TP54574_Hit	D	1	chr8	.	CAGCTTCAATAGCAAATATGTCTTTCAAGTTAACATTTTTTGGTACTAAACCAGCACCTAGTGT
TP54574_Query	D	1	chr8	.	CAGCTTCAATAGCAAATATGTCTTTCAAGTTAACATTTTATGGTACTAAACCAGCACCTAGTGT
TP54667_Hit	D	1	chr8	.	CAGCTTCACAAGATTGATGAAGGGAGAGAATATGCTGAATTTATGCATGCTCCAAGGAAAAGAT
TP54667_Query	D	1	chr8	.	CAGCTTCACAAGATTGATGAAGGAAGAGAATATGCTGAATTTATGCATGCTCCAAGGAAAAGAT
TP55160_Hit	D+G	1	chr8	.	CAGCTTCATTTTCAATAGGATTCATTGAGCACGTTGAATAAACCATTCTTCCCCCGATTTTTAG
TP55160_Query	D+G	1	chr8	.	CAGCTTCATTTTCAATAGGATTCATTGAGCACGTTGAATAAACCATTCTTCCCCCGATTTTGAG
TP55318_Hit	D	1	chr8	.	CAGCTTCTCCGAGGAAGTAGCAGTCCTTGATAGCTACTTTGCGAAACTTGGATCAGGAAGCGTG
TP55318_Query	D	1	chr8	.	CAGCTTCCCCGAGGAAGTAGCAGTCCTTGATAGCTACTTTGCGAAACTTGGATCAGGAAGCGTG
TP55323_Hit	D	1	chr8	.	CAGCTTCCCGGATAAAAAAGAGCTGTCTTTGTTTGGCAACAAATGCTGTCTCTGTGGATCAGTG
TP55323_Query	D	1	chr8	.	CAGCTTCCCGGATAAAAAAGAGCTGTCTTTGTTTGGCAACAAATGCCGTCTCTGTGGATCAGTG
TP55378_Hit	D+G	1	chr8	.	CAGCTTCCGGGTTGGTTTTCCGGCGCCGTTCATTGTGTCCAGCCAACCGCCTGCGGCTGAAAAA
TP55378_Query	D+G	1	chr8	.	CAGCTTCCGGGTTGGTTTTCCGGCGCCGTTCATTGTGTCCAGCCAACCGCCTGCGGCAGAAAAA
TP55763_Hit	D	1	chr8	.	CAGCTTCTACGAGTTGAACAATTTGAGAAAGGGCAGTGTCTGCTCCAACATGTGTAGCCTTAAC
TP55763_Query	D	1	chr8	.	CAGCTTCTACGAGTTGAACAATTTGAGAAAGGGCAGTGTCTGATCCAACATGTGTAGCCTTAAC
TP55963_Hit	D	1	chr8	.	CAGCTTTTCTTTTTCCTTTTCCTCTGCCTGCAGAATTCGGTGGAACTCAAGTTGTTCTTTTATC
TP55963_Query	D	1	chr8	.	CAGCTTCTCTTTTTCCTTTTCCTCTGCCTGCAGAATTCGGTGGAACTCAAGTTGTTCTTTTATC
TP56433_Hit	D+G	1	chr8	.	CAGCTTCTGGTGCTAAAGAAGTTTCAGCACCTTCAAAGGCCAAGCCTGCAACAGGTGCTGAAAA
TP56433_Query	D+G	1	chr8	.	CAGCTTCTGGTGCTAAAGAAGTTTCAGCACCTTCAAAGGCCAAACCTGCAACAGGTGCTGAAAA
TP56445_Hit	D+G	1	chr8	.	CAGCTTCTGTAACTGTAATTTGTGGAGTGTGCACCTTATCGTCAACATCATTCTCAGAGATAGA
TP56445_Query	D+G	1	chr8	.	CAGCTTCTGTAACTGTAATTTGTGGAGTGTGCACCTTATCGTCAACATCATCCTCAGAGATAGA
TP56657_Hit	D	1	chr8	.	CAGCTTCTTTATCGAAAACTATGCTACATCTTGGGCAAAGCATGGCATGTGCATTGCAAATTTT
TP56657_Query	D	1	chr8	.	CAGCTTCTTTATCGAAAACTACGCTACATCTTGGGCAAAGCATGGCATGTGCATTGCAAATTTT
TP56814_Hit	D	1	chr8	.	CAGCTTGAACAATATGCAGGGTCAAGGTACCCTGGTGTTCCTTTGATTTGGCTACTGACATGTG
TP56814_Query	D	1	chr8	.	CAGCTTGAACAATATGCAGGGTCAAGGTACCCTGGTGTCCCTTTGATTTGGCTACTGACATGTG
TP57183_Hit	D+G	1	chr8	.	CAGCTTGATCAGTGTTAGCGGTGCGGTGAGTGTAAAGATGATCAACATTCGATGATGTTAGCGG
TP57183_Query	D+G	1	chr8	.	CAGCTTGATCAGTGTTAGCCGTGCGGTGAGTGTAAAGATGATCAACATTCGATGATGTTAGCGG
TP57332_Hit	D	1	chr8	.	CAGCTTGCAATATCACTCTGGAAATCTCAACAACACCACCCTCTTTGGCAATTTCGTTCGGCCA
TP57332_Query	D	1	chr8	.	CAGCTTGCAATATCACTCTAGAAATCTCAACAACACCACCCTCTTTGGCAATTTCGTTCGGCCA
TP57417_Hit	D	1	chr8	.	CAGCTTGCCAATTCTGGATTGTAATTGGAATATCCCCATGTATCTGATACAAAAGTCTGAAGAG
TP57417_Query	D	1	chr8	.	CAGCTTGCCAATTCTGGATTGTAATTGGAATATCCACATGTATCTGATACAAAAGTCTGAAGAG
TP57784_Hit	D	1	chr8	.	CAGCTTGGGATGATGTCAGCCAACATCTTTCAAAATGCAGTATGCTTCAGCAAGGATAAACCAG
TP57784_Query	D	1	chr8	.	CAGCTTGGGATGATGTCAGCCAACATCTTTCAAAATGCAGTATGCTTCAGCAAGGATAAAACAG
TP58155_Hit	D	1	chr8	.	CAGCTTGTGACCATTGTTGTGGGATGTAAATGTCTGGAAGGACAAAACCAGATGAACCCATTGC
TP58155_Query	D	1	chr8	.	CAGCTTGTGACCATTGTTGTGGGATGTAAATGTCTGGAAGGACAAAACCAGATGAACCCATGGC
TP58174_Hit	D	1	chr8	.	CAGCTTGTGATTATGCCAAGGGTAAGTGGGTTGCAGATAGCAGGAGACCGCTCTATTCTGGTTT
TP58174_Query	D	1	chr8	.	CAGCTTGTGATTACGCCAAGGGTAAGTGGGTTGCAGATAGCAGGAGACCGCTCTATTCTGGTTT
TP58382_Hit	D	1	chr8	.	CAGCTTGTTGTGTTGGCTTCACACATGGTATTGCTTTGGCTTTTCATGTGCTTGAATTTTTATA
TP58382_Query	D	1	chr8	.	CAGCTTGTTGTGTTGGCTTCACACATGGTATTGCTTTGGCTTTTCATGTGCTTAAATTTTTATA
TP58459_Hit	D	1	chr8	.	CAGCTTTAAAATATATCAGATTAGCAAGCAGAATAAACATATAAAACATATTGCAACATTGGTT
TP58459_Query	D	1	chr8	.	CAGCTTTAAAATATATCAGATTAGCAAGCAGAATAAACATATAAAACAGATTGCAACATTGGTT
TP58672_Hit	D	1	chr8	.	CAGCTTTAGAGTAAGCAAATAATTTTGGTAATGTGTCCAAGCACCATATGAATGCTACTTTGGA
TP58672_Query	D	1	chr8	.	CAGCTTTAGAGTAAGCAAATAATTTTGGTAATGTGTCCAAGCACCAAATGAATGCTACTTTGGA
TP58786_Hit	D	1	chr8	.	CAGCTTTATCAGGTTCAACTCCAGCCTCTTGCATCTTTTCAAAAATCTCAAGAGCTTCTTTGCA
TP58786_Query	D	1	chr8	.	CAGCTTTATCAGGTTCAACTCCAGCCTCTTGCATCTTCTCAAAAATCTCAAGAGCTTCTTTGCA
TP58801_Hit	D	1	chr8	.	CAGCTTTATCTAATCCATGTTCCTTATGACCATCATCTCCATCTATTTATTTGCAATTTAAGTG
TP58801_Query	D	1	chr8	.	CAGCTTTATCTAATCCATGTTCCTTATAACCATCATCTCCATCTATTTATTTGCAATTTAAGTG
TP58818_Hit	D	1	chr8	.	CAGCTTTATGCTCTATAAGAATCATAAAGAAAGTCCCAGACCTGGCTGAAAATTTTGTAAATCC
TP58818_Query	D	1	chr8	.	CAGCTTTATGCTCTATAAGAATCATAAAGAAAGTCCCAGACCTGGCAGAAAATTTTGTAAATCC
TP5898_Hit	D+G	1	chr8	.	CAGCAAGCTTTTCTAGTATTGGGACGCCCAACAGAAATGGAAGCTGGAAAATATGCACATTAAA
TP5898_Query	D+G	1	chr8	.	CAGCAAGCTTTTCTAGTATTGGGACGCCCAACAGAAATGGAAGCTGGAAAATATGCACAATAAA

Name	Filter	Nb hit (Mt4.0)	Mt Chr (Mt4.0)	Ms Chr (Li et al., 2014)	Sequence
TP5907_Hit	D	1	chr8	.	CAGCAAGGAAAACAAAAATCTCAAACGAGGTGATCAACGTTCTCTTAAAATCTTGTTTTAAATT
TP5907_Query	D	1	chr8	.	CAGCAAGGAAAACAAAAATCTCAAACGAGGTAATCAACGTTCTCTTAAAATCTTGTTTTAAATT
TP59152_Hit	D+G	1	chr8	.	CAGCTTTCGTTGAGAAAAAAGCATCGAGAAGGTATGGGTTCTACATTTTTTGATGCAAATTTGT
TP59152_Query	D+G	1	chr8	.	CAGCTTTCGTTGAGAAAAAAACATCGAGAAGGTATGGGTTCTACATTTTTTGATGCAAATTTGT
TP59213_Hit	D	1	chr8	.	CAGCTTTCTCTTTAGTGTCTTCTGATTCACCAGAACAACTGCCGTTATTAGAAACAGAATCATA
TP59213_Query	D	1	chr8	.	CAGCTTTCTCTTTAGTGTCTTCCGATTCACCAGAACAACTGCCGTTATTAGAAACAGAATCATA
TP5945_Hit	D	1	chr8	.	CAGCAAGGAAGAGATCATTGAAATAACGAAGACTTTCAACTTAGCGAAACAAATTGTACTATAC
TP5945_Query	D	1	chr8	.	CAGCAAGGAAGAGATAATTGAAATAACGAAGACTTTCAACTTAGCGAAACAAATTGTACTATAC
TP59587_Hit	D	1	chr8	.	CAGCTTTGGATTCGCCCGGTTGTAGGGCGAAGCCTGTTGTTGCTAGCTGTGCAGTTCCGGCACC
TP59587_Query	D	1	chr8	.	CAGCTTTGGATTCGCCCGGTTGTAGGGCAAAGCCTGTTGTTGCTAGCTGTGCAGTTCCGGCACC
TP59590_Hit	D	1	chr8	.	CAGCTTTGGCAAAATCACATGGCAGTAGAATTAAATTTGTTGACAATCTGTGTTAATTTACCCT
TP59590_Query	D	1	chr8	.	CAGCTTTGGCAAAATCACATAGCAGTAGAATTAAATTTGTTGACAATCTGTGTTAATTTACCCT
TP59648_Hit	D+G	1	chr8	.	CAGCTTTGGTGTTGTTATGCTTGAGCTTCTCACTGGAAGGAGACCTATGGATGTATGCAAGCCT
TP59648_Query	D+G	1	chr8	.	CAGCTTTGGTGTTGTAATGCTTGAGCTTCTCACTGGAAGGAGACCTATGGATGTATGCAAGCCT
TP59664_Hit	D	1	chr8	.	CAGCTTTGGTTGTGGTTCTGTGTTTCCTTGCATTGATTTTTAAGGAGACGGTAATGGGAGGTAA
TP59664_Query	D	1	chr8	.	CAGCTTTGGTTGTGGTTCTGTGTTTCCTTGCATTGATTTTTAAGGAGACAGTAATGGGAGGTAA
TP59697_Hit	D	1	chr8	.	CAGCTTTGTATTAGTTCTTCTCACTTTTATTTTGTGCAATGTCAGTCCGTTATATACTTTGTGG
TP59697_Query	D	1	chr8	.	CAGCTTTGTATTAGTTCTTCTCACTTTTATTTTGTGCAATGTCAGTCCATTATATACTTTGTGG
TP59755_Hit	D	1	chr8	.	CAGCTTTGTTACCTTCTCCGATGTCTTTGATATGTTCCTCAGCATTATCAAATATGGTTGATGT
TP59755_Query	D	1	chr8	.	CAGCTTTGTTACCTTCTCCAATGTCTTTGATATGTTCCTCAGCATTATCAAATATGGTTGATGT
TP59819_Hit	D	1	chr8	.	CAGCTTTTGAGTATGCAAATAATTTTGGCAATGAGTCCAAGCACCATATGAATGCTACTTTGAA
TP59819_Query	D	1	chr8	.	CAGCTTTTAAGTATGCAAATAATTTTGGCAATGAGTCCAAGCACCATATGAATGCTACTTTGAA
TP60062_Hit	D	1	chr8	.	CAGCTTTTGAATGCACCTGCATCAACCTCAACCGCTGAAAAAAAAAAAAAAAAAAAAAAAAAAA
TP60062_Query	D	1	chr8	.	CAGCTTTTGAACGCACCTGCATCAACCTCAACCGCTGAAAAAAAAAAAAAAAAAAAAAAAAAAA
TP60250_Hit	D	1	chr8	.	CAGCTTTTGTGATATAATTTTTATTGGAAACAGGTTACAGTTCATGTCAAAGGAAGAACAGAGT
TP60250_Query	D	1	chr8	.	CAGCTTTTGTGATATAATTTTTATTGGAAACAGGTAACAGTTCATGTCAAAGGAAGAACAGAGT
TP60277_Hit	D+G	1	chr8	.	CTGCTTTTGTTTTCAGTATTTCATATCAGGAGGTGGCAGGAAAATCCCCTTGCACAGGTAAAAT
TP60277_Query	D+G	1	chr8	.	CAGCTTTTGTTTTCAGTATTTCATATCAGGAGGTGGCAGGAAAATCCCCTTGCACAGGTAAAAT
TP60322_Hit	D	1	chr8	.	CAGCTTTTTCACCGAAGTTGAATGTCACTGTGCCAGAACCACCACGACCGCGGCCACCGCCACG
TP60322_Query	D	1	chr8	.	CAGCTTTTTCACCAAAGTTGAATGTCACTGTGCCAGAACCACCACGACCGCGGCCACCGCCACG
TP60401_Hit	D	1	chr8	.	CAGCTTTTTGGTAATGGTGATGGGTTTGGTAATTTCCTGGAGCACAACACTATTGGTGGTGGAT
TP60401_Query	D	1	chr8	.	CAGCTTTTTGGTAATGGTGATGGGTTTGGTAATTTCCTGGAGCACAACACTATTGGTGGTAGAT
TP60428_Hit	D	1	chr8	.	CAGCTTTTTTCATTTGAACTCCATTCTGCAGTTTCAATTTTGGATTTCATTACTCACTAGCTCT
TP60428_Query	D	1	chr8	.	CAGCTTTTTTCATTTGAACTCCATTCTGCAGTTTCAATTTTGGATTTCATTACTCACTAGCACT
TP60596_Hit	D+G	1	chr8	.	CTGCAAAAATCAACAAGATCAAGATTAGTATCAATGCAACAAGATCTTATGTTGAACTATTGTA
TP60596_Query	D+G	1	chr8	.	CTGCAAAAATCAACAAGATCAAGATTAGTATCAACGCAACAAGATCTTATGTTGAACTATTGTA
TP60641_Hit	D	1	chr8	.	CTGCAAAACAAGTCAAGAATATAAAAAAATGACTTTTCAATATTTGAGCTCTACACAGTATATG
TP60641_Query	D	1	chr8	.	CTGCAAAACAAGTCAAGAATATAAAAAAATGACTTTTCAATATTTGAGCTCTACACAGCATATG
TP61018_Hit	D+G	1	chr8	.	CTGCAAACACCTTGGGCCTCTCGTTTCATGAGGCCTTCGACAAGCAACCAATTGAGCTACTACA
TP61018_Query	D+G	1	chr8	.	CTGCAAACACCTTAGGCCTCTCGTTTCATGAGGCCTTCGACAAGCAACCAATTGAGCTACTACA
TP61025_Hit	D	1	chr8	.	CTGCAAACACTTAATTAAGTTGTTTATCCAAATAGACCTTAGACTATCAAAAGTAGAGAACACT
TP61025_Query	D	1	chr8	.	CTGCAAACACTTAATTAAATTGTTTATCCAAATAGACCTTAGACTATCAAAAGTAGAGAACACT
TP61093_Hit	D+G	1	chr8	.	CTGCGAACCGCGGCGTGCTTGTTGTTTCATCTGCTGGAAATGAAGGAAATCCTGGTTCTGCTAC
TP61093_Query	D+G	1	chr8	.	CTGCAAACCGCGGCGTGCTTGTTGTTTCATCTGCTGGAAATGAAGGAAATCCTGGTTCTGCTAC
TP61259_Hit	D+G	1	chr8	.	CTGCAAAGAGCGAGATCAGTGATTGGTGATGCGTGACCACACAACACTCCCACCGCTTTAACTT
TP61259_Query	D+G	1	chr8	.	CTGCAAAGAGCGAGATCAGTGATTGGTGATGCGTGACCACACAACACTCCCACCGCTTTAACGT
TP61796_Hit	D	1	chr8	.	CTGCAAATTATGCAGATCTCTCTCCTTTTAGTAATCAATACTTGATGTTCTCATCAAACTCTTG
TP61796_Query	D	1	chr8	.	CTGCAAATTATACAGATCTCTCTCCTTTTAGTAATCAATACTTGATGTTCTCATCAAACTCTTG
TP61807_Hit	D	1	chr8	.	CTGCAAATTCAAGTGGATCCCAAAACTGTTGTTGGAATTTCACTCATCTCCAGAGTGGTCACTC
TP61807_Query	D	1	chr8	.	CTGCAAATTCAAGTGGATCCCAAAACTATTGTTGGAATTTCACTCATCTCCAGAGTGGTCACTC
TP62067_Hit	D	1	chr8	.	CTGCAACACAATCTGTCTCTTGTAAGAAGAAAACAGATGTCTCTATGAGGTTTACAGATGCATC
TP62067_Query	D	1	chr8	.	CTGCAACACAATCTGTCTCTTGTAAGAAGAAAACAGATGTCTCTATGAGGTTTACAAATGCATC
TP6244_Hit	D	1	chr8	.	CAGCAAGGTTATAAAACCTCTATTCATATCTGAAATGATTATTTATTTATTACTCCCTACTAGC
TP6244_Query	D	1	chr8	.	CAGCAAGGTTATAAAACCTCTATTCATATCTGAAATGATTATTTATTTACTACTCCCTACTAGC

Name	Filter	Nb hit (Mt4.0)	Mt Chr (Mt4.0)	Ms Chr (Li et al., 2014)	Sequence
TP6249_Hit	D	1	chr8	.	CAGCAAGGTTCGACATAGTAAACCGATGCTCCTACACCGTGTGGCCAGCAGGCATTCCCAGGGG
TP6249_Query	D	1	chr8	.	CAGCAAGGTTCGACATAGTAAACCGATGCCCCTACACCGTGTGGCCAGCAGGCATTCCCAGGGG
TP62547_Hit	D	1	chr8	.	CTGCAACGCCTTTCAGTCTAAAAATCTCATCATAATGTGCCAATATACCATCAACAATCATGCG
TP62547_Query	D	1	chr8	.	CTGCAACGCCTTTCAGCCTAAAAATCTCATCATAATGTGCCAATATACCATCAACAATCATGCG
TP62657_Hit	D	1	chr8	.	CTGCAACTAGGGTTGATGCTTATGTGTTGGAGGGTGATTTCCCGTGCTTGGCTCGCCTGATAAC
TP62657_Query	D	1	chr8	.	CTGCAACTAGGGTTGATGCTTATGTGTTGGAGGGTGATTTCCCATGCTTGGCTCGCCTGATAAC
TP63067_Hit	D	1	chr8	.	CTGCAAGGCAGAAGAACTCAGGCCTTGAAGACCGAGTTAGTCACCTTGATGGGGCCCTCAAAGA
TP63067_Query	D	1	chr8	.	CTGCAAGACAGAAGAACTCAGGCCTTGAAGACCGAGTTAGTCACCTTGATGGGGCCCTCAAAGA
TP63161_Hit	D	1	chr8	.	CTGCAAGAGTCGATGACTCATCTGGTAGGCACTCATCTCATCATGCTGTTGGCGGTGCTGAAAA
TP63161_Query	D	1	chr8	.	CTGCAAGAGTCGATGACTCATCTGGTAGGCACTCATCTCATCATGCTGTTGGCAGTGCTGAAAA
TP63222_Hit	D	1	chr8	.	CTGCAAGATTTGTGTGTGTCTACATCATATTACATTGCATCAATTGTTACATTTTTTTACAACA
TP63222_Query	D	1	chr8	.	CTGCAAGATTTGTGTGTGTCTACATCATATTACATAGCATCAATTGTTACATTTTTTTACAACA
TP63376_Hit	D+G	1	chr8	.	CTGCAAGGAAGCGCCATTTCGCTTATGGCCCATCCACTGCTTCTTCTAATGCTTCCGCCAATGC
TP63376_Query	D+G	1	chr8	.	CTGCAAGGAAGCGCCATTTCGCTTATGGCCCATCCACTGCTTCTTCTAATGCTTCCGCCAACGC
TP63659_Hit	D	1	chr8	.	CTGCAAGTTCACGATAGGCGAGTATAAGGGTTCTTAGACCTGCATCGGCGTATTCATGCACATG
TP63659_Query	D	1	chr8	.	CTGCAAGTTCACGATAGGCGAGTATAAGGGTTCTTAGACCTGCATCGGCATATTCATGCACATG
TP63660_Hit	D	1	chr8	.	CTGCAAGTTCCGAAGAAAAACCTTCTGAGGATGCTCTTAATGCTTGGGATACTGAATTTCTCAA
TP63660_Query	D	1	chr8	.	CTGCAAGTTCAGAAGAAAAACCTTCTGAGGATGCTCTTAATGCTTGGGATACTGAATTTCTCAA
TP63675_Hit	D	1	chr8	.	CTGCAAGTTCTTTAGCCAAAGCCATATTTCTCTCTTGCAATTTAAAACGTTCCAAATTTGACTT
TP63675_Query	D	1	chr8	.	CTGCAAGTTCTTTAGCCAAAGCCATATTTCTCTCTTGCAATTTAAAACGGTCCAAATTTGACTT
TP63716_Hit	D+G	1	chr8	.	CTGCAATAAAAATCTGATTTACGATTCAGAAAATAGTTCTTCTGATTCACAATATGAATGATTT
TP63716_Query	D+G	1	chr8	.	CTGCAATAAAAATCCGATTTACGATTCAGAAAATAGTTCTTCTGATTCACAATATGAATGATTT
TP63880_Hit	D	1	chr8	.	CTGCAATAGGAGGAACAAGCCAAGTGTTCGATCTGAAGCCATATGGGAGATTTCCAAACTGAAG
TP63880_Query	D	1	chr8	.	CTGCAATAGGAGGAACAAGCCAAGTGTTCGATCTGAAGCCAAATGGGAGATTTCCAAACTGAAG
TP6392_Hit	D+G	1	chr8	.	CAGCAAGTCCACGGGGGTCTTGGCCTTTATTCCGAAAGAGGGAAAGAGGGGTCATCCAAAACCA
TP6392_Query	D+G	1	chr8	.	CAGCAAGTCCACGGGGGTCTTAGCCTTTATTCCGAAAGAGGGAAAGAGGGGTCATCCAAAACCA
TP63955_Hit	D	1	chr8	.	CTGCAATATGATATACATATACACATGCAATATCTTCTGGTAACAGCAACTGTGATAATGTGTC
TP63955_Query	D	1	chr8	.	CTGCAATATGATATACATATACACATGCAATATCTTCTGGTAACAGCAACTGTGATAACGTGTC
TP64299_Hit	D	1	chr8	.	CTGCAATGCAAACAGAAAGAGGAACAAGAGCATTCAAAAGAAGATACAACGAGTCAGATGATGG
TP64299_Query	D	1	chr8	.	CTGCAATGCAAACAAAAAGAGGAACAAGAGCATTCAAAAGAAGATACAACGAGTCAGATGATGG
TP64353_Hit	D+G	1	chr8	.	CTGCAATGCTTGAACAACTTCTCCCATGAATGGTCGTTGAGATACTTCTGGTTGAACGCACATG
TP64353_Query	D+G	1	chr8	.	CTGCAATGCTTGAACAACTTCTCCCATGAAAGGTCGTTGAGATACTTCTGGTTGAACGCACATG
TP64537_Hit	D	1	chr8	.	CTGCAATGTTGGATGTTGAATCAAGCTCCTTTTCTTGGGATGGGCTCTCTTCCCTGCACCATAC
TP64537_Query	D	1	chr8	.	CTGCAATGTTGGATGTTGAATCAAGCTCCTTTTCATGGGATGGGCTCTCTTCCCTGCACCATAC
TP64652_Hit	D	1	chr8	.	CTGCTATTCCAGAAATGTGAGGGCATGCCATGGAAGTGCCAGACATTAGTGTAAATTCTGAAAA
TP64652_Query	D	1	chr8	.	CTGCAATTCCAGAAATGTGAGGGCATGCCATGGAAGTGCCAGACATTAGTGTAAATTCTGAAAA
TP6484_Hit	D+G	1	chr8	.	CAGCAAGTGCTAACAATGAAACTGGAATGGGACTGGATGCAAACCATCCACTTACAAGTACCAT
TP6484_Query	D+G	1	chr8	.	CAGCAAGTGCTAACAATGAAACTGGAATGGGACTGGACGCAAACCATCCACTTACAAGTACCAT
TP64938_Hit	D	1	chr8	.	CTGCAATTTTCACAATCATGTAAGGACCCTGCCACTCCCAATCCCATGTGCATCGCCACACAGG
TP64938_Query	D	1	chr8	.	CTGCAATTTTCACAATCATGGAAGGACCCTGCCACTCCCAATCCCATGTGCATCGCCACACAGG
TP65305_Hit	D+G	1	chr8	.	CTGCACACATGACACAGTTAAGCACCATGGATAAGGTTGTTGAAGTCTTTGAGCAAGCTGTGTC
TP65305_Query	D+G	1	chr8	.	CTGCACACATGACACAGTTAAGCACCATGGATAAGGTTGTTGAAGTCTTTGAACAAGCTGTGTC
TP6543_Hit	D+G	1	chr8	.	CAGCAAGTTAGCTATACCATTTTTTTTAATAGGAAAATTATGTGTATGCGTATATGTTTTTAAG
TP6543_Query	D+G	1	chr8	.	CAGCAAGTTAGCTATACCATTTTTTTTAATAGGAAAATTATGTGTATGCGTATATGTTTTTAAA
TP65431_Hit	D	1	chr8	.	CTGCACAGCAGAAAATGTAATAAAACTGAAACAACTTGTACTAGATTGTGTAACGTTGACCCAA
TP65431_Query	D	1	chr8	.	CTGCACAGCAGAAAATGTAATAAAACTGAAACAACTTGTACTAGATTGTGTAACGTCGACCCAA
TP65549_Hit	D+G	1	chr8	.	CTGCACATACTCTGGCAGAGGGAGGGAGTTGAAGTTCAAAGGGCTAGGTGGAGGTTGTGGTTGG
TP65549_Query	D+G	1	chr8	.	CTGCACATACTCTGGCAGAGGGAGGGAGTTGAAGTTCAAAGGGATAGGTGGAGGTTGTGGTTGG
TP65621_Hit	D+G	1	chr8	.	CTGCACATGATGCACGGATGAAGTGTGTTGCTGTTGCTAGCAAGCATCCTGTGTATGAGCTTGG
TP65621_Query	D+G	1	chr8	.	CTGCACATGATGCACAGATGAAGTGTGTTGCTGTTGCTAGCAAGCATCCTGTGTATGAGCTTGG
TP65746_Hit	D	1	chr8	.	CTGCACCAAATGGATTCGGCTTATTCGTCTTCACCACCGGAGATTCATTGACAGAACCATCACC
TP65746_Query	D	1	chr8	.	CTGCACCAAATGGATTCGGCTTATTCGTCTTCACCACCAGAGATTCATTGACAGAACCATCACC
TP65766_Hit	D	1	chr8	.	CTGCACCAACTTATCTACCACCCTATTACTTTGCAAATGATGGTGCTGAATTCAATTTGGTTGA
TP65766_Query	D	1	chr8	.	CTGCACCAACTTATCTACCACCCTATTACTTTGAAAATGATGGTGCTGAATTCAATTTGGTTGA

Name	Filter	Nb hit (Mt4.0)	Mt Chr (Mt4.0)	Ms Chr (Li et al., 2014)	Sequence
TP65775_Hit	D	1	chr8	.	CTGCACCAAGCCAGTGGGATCTTGTTTCTGACAAGCAAATGATGCAGGAGGTGCTGAAAAAAAA
TP65775_Query	D	1	chr8	.	CTGCACCAAGCCAGTGGGATCTTGTTTCTGACAAGCAAATGATGCAAGAGGTGCTGAAAAAAAA
TP66021_Hit	D	1	chr8	.	CTGCACCCACAAATCATGAGCAATAATTGAATGGAAACTAATTGATTTATTTGATATTCTCATT
TP66021_Query	D	1	chr8	.	CTGCACCCACAAAGCATGAGCAATAATTGAATGGAAACTAATTGATTTATTTGATATTCTCATT
TP66069_Hit	D	1	chr8	.	CTGCACCCTCAACGTCGTTGGCTCGTTCTTTTAAAGACACAAGGGTGGCAACAGTAGCCGGCAT
TP66069_Query	D	1	chr8	.	CTGCACCCTCAACGTCGTTGGCTCGTTCTTTTAAAGACACAAGGGGGGCAACAGTAGCCGGCAT
TP6611_Hit	D	1	chr8	.	CAGCAAGTTGCTCAGTCACAAGTTTCTTGTATTAAGACTGCCTTTTCGTTGATTAAAGGGGAGG
TP6611_Query	D	1	chr8	.	CAGCAAGTTGCTCAGTCACAAGTTTCTTGTATTAAGACCGCCTTTTCGTTGATTAAAGGGGAGG
TP66457_Hit	D+G	1	chr8	.	CTGCACGATGCGTTGAACCACTAGTCTCTCTCCTTGCAACCGAGTTCAGTCCTGCTCATCATTC
TP66457_Query	D+G	1	chr8	.	CTGCACGATGCGTTGAACCACTAGTCTCTCTCCTTGCAACCGAGTTCAATCCTGCTCATCATTC
TP67429_Hit	D	1	chr8	.	CTGCAGAAAAACCATGTTCACAGTTCAATTCCTCTCATAGTTAAAAGCTCAAAATTGAAAATAA
TP67429_Query	D	1	chr8	.	CTGCAGAAAAACCATGTTCACAGTTCAATTCCTCTCATAGTTAAAAGCTCAAAATTAAAAATAA
TP67492_Hit	D	1	chr8	.	CTGCAGAAACTAGGAATGGTGATTTCTATGATGGGATTCCACGCTATGCTGATTCTTTTTCACA
TP67492_Query	D	1	chr8	.	CTGCAGAAACTAGGAATGATGATTTCTATGATGGGATTCCACGCTATGCTGATTCTTTTTCACA
TP67858_Hit	D	1	chr8	.	CTGCAGACTTGGTTGTGAGGCGTCTGGACGAGCTTTCAGTGGTTGATTTGAAGAATTTGGCTGA
TP67858_Query	D	1	chr8	.	CTGCAGACTTGGTTGTGAGGCGTCTGGACGAGCTTTCAGTGGTTGATTTGAAGAATTTGGCAGA
TP68099_Hit	D	1	chr8	.	CTGCAGATATTATGGTGCATGATCAAGGGCTTCATTTCAGCCCCCGCAAGGTCACTGTTTCAAC
TP68099_Query	D	1	chr8	.	CTGCAGATATTATGGTGCATGACCAAGGGCTTCATTTCAGCCCCCGCAAGGTCACTGTTTCAAC
TP689_Hit	D	1	chr8	.	CAGCAAAAGGAGCTGATATAGTTATTCCGATTGATGTTCTTGCTTGGGGTGGATTTGGAATTGT
TP689_Query	D	1	chr8	.	CAGCAAAAGGAGCTGATATAGTTATTCCGATTGATGTTCTTGCTTGGGGTGGATTTGGAATCGT
TP69142_Hit	D	1	chr8	.	CTGCAGGTAGTAGTCACGGTAATGATGCTGAGAGTGGAAATGGTCTTCCCGATGATGAGTTAGA
TP69142_Query	D	1	chr8	.	CTGCAGGCAGTAGTCACGGTAATGATGCTGAGAGTGGAAATGGTCTTCCCGATGATGAGTTAGA
TP69188_Hit	D	1	chr8	.	CTGCAGGTGGTTGAGTGGTGGTTGGTTGTTTGCTAGACAAGGGGGAAGTTGCTACCACAGTAGG
TP69188_Query	D	1	chr8	.	CTGCAGGCGGTTGAGTGGTGGTTGGTTGTTTGCTAGACAAGGGGGAAGTTGCTACCACAGTAGG
TP69250_Hit	D+G	1	chr8	.	CTGCAGGGCAGTCTTATACTGGGGCAGTACTTGTTAGTCCAGATGGAGCATTTCCAAATGAAAG
TP69250_Query	D+G	1	chr8	.	CTGCAGGGCAGTCTTATACTGGGGCAGTACTTGTTAGTCCAGATGGAGCATATCCAAATGAAAG
TP69353_Hit	D	1	chr8	.	CTGCAGGTAGCTTCTTTGATGTTTTAAATTAAATATGTTCCCTCTGTGTGTGTTTGTGTTTGCG
TP69353_Query	D	1	chr8	.	CTGCAGGTAGCTTCTTTGATGTTTTAAATTAAATATGTTCCCTCTGTGTGTGTTTATGTTTGCG
TP69358_Hit	D+G	1	chr8	.	CTGCAGGTATATTGAATTTTATGATGCGATGTCAGTGCCAATGGCAATTGCTCTCTCTGGCCAA
TP69358_Query	D+G	1	chr8	.	CTGCAGGTATATTGAATTTTATGATGCGATGTCAGTGCCAATGGCAATTGCGCTCTCTGGCCAA
TP6952_Hit	D	1	chr8	.	CAGCAATAGAAGATTCACTTGGAGAAGCTGAGCTCAAATATATTGGATTTGTTTGATTTGCTAA
TP6952_Query	D	1	chr8	.	CAGCAATAGAAGATTCACTTGGAGAAGCTGAGCTCAAATATATTGGATTTGTTTGATATGCTAA
TP69743_Hit	D	1	chr8	.	CTGCAGTCTCCTAGGATGGCAGGACCTACAGGTCAAAAGTCAATAAGTTTAACAGGATCGCTGA
TP69743_Query	D	1	chr8	.	CTGCAGTCTCCTAGGATGGCAGGACCTACAGGTCAAAAGTCAATAAGTTTAACAGGATCGCAGA
TP70294_Hit	D	1	chr8	.	CTGCATAAACTGGGACAATTGTTGTTGGTTCAGGGTCATAGAGTTTTGTTGCAGTTGACCAGAT
TP70294_Query	D	1	chr8	.	CTGCATAAACTGGGACAACTGTTGTTGGTTCAGGGTCATAGAGTTTTGTTGCAGTTGACCAGAT
TP70556_Hit	D	1	chr8	.	CTGCATAGACCTGCCCTATCCTCAGTTTGCTAATTTGAGCAACGGAGGATAGATCTCCTGCTCT
TP70556_Query	D	1	chr8	.	CTGCATAGACCTGCCCTATCATCAGTTTGCTAATTTGAGCAACGGAGGATAGATCTCCTGCTCT
TP70801_Hit	D	1	chr8	.	CTGCATATGTTTCTGATGAAGGGCCTGCTTCTCTTTCCAAGATGGTTGCAGTGCTTTGTGAACA
TP70801_Query	D	1	chr8	.	CTGCATATGTTTCCGATGAAGGGCCTGCTTCTCTTTCCAAGATGGTTGCAGTGCTTTGTGAACA
TP70983_Hit	D	1	chr8	.	CTGCATCACACTACATTACTAACACTGCATTGCTATTTCACAGTATTTTGTACTAAATAGCTTA
TP70983_Query	D	1	chr8	.	CTGCATCACACTACATTACTAACACTGCATTGTTATTTCACAGTATTTTGTACTAAATAGCTTA
TP70984_Hit	D	1	chr8	.	CTGCATCACACTGCATTACTAAGACTTCATTGCTAAGAAAGGATTCATGTCGACCACACGATCA
TP70984_Query	D	1	chr8	.	CTGCATCACACTACATTACTAAGACTTCATTGCTAAGAAAGGATTCATGTCGACCACACGATCA
TP7118_Hit	D	1	chr8	.	CAGCAATATATTTACAGTGATTACTCTCTTTCCAATTCCCCTTTTCTTTACAGATCTTACTCTT
TP7118_Query	D	1	chr8	.	CAGCAATATATTTACAGTGATTACTCTCTTTCCAATTCCCCTTTGCTTTACAGATCTTACTCTT
TP71234_Hit	D	1	chr8	.	CTGCATCCCTAGGTGGACATGCACTTGATGGATGTGGTGAATTCATGTCACCATCATCTCTCAA
TP71234_Query	D	1	chr8	.	CTGCATCCCTAGGTGGACATGCACTTGATGGATGTGGTGAATTCATGCCACCATCATCTCTCAA
TP71265_Hit	D+G	1	chr8	.	CTGCATCCGTAGAAAGATATATGATGGGTGCATCGGCCCTCTCAATCACCCTCAAGATGCAATC
TP71265_Query	D+G	1	chr8	.	CTGCATCCGTAGAAAGATATATGATGGGTGCATCGGCCCTCTCAATCACCCTCAAAATGCAATC
TP71278_Hit	D+G	1	chr8	.	CTGCATCCTCCCAGTCATCAGGTTCGGCTTTATTGTGACCACTTTTCTCGCTTACTGCTGAAAA
TP71278_Query	D+G	1	chr8	.	CTGCATCCTCCCAGTCATCAGGTTCGGCTTTATTGTGACCACTTTTCTCGCTTACTGCAGAAAA
TP71349_Hit	D	1	chr8	.	CTGCATCGGCGTTATCTAAAGAAAACCCCATAACCCCCTTTATCTGACTCCTCTCTAATGTTAA
TP71349_Query	D	1	chr8	.	CTGCATCGGCGTTATCCAAAGAAAACCCCATAACCCCCTTTATCTGACTCCTCTCTAATGTTAA

Name	Filter	Nb hit (Mt4.0)	Mt Chr (Mt4.0)	Ms Chr (Li et al., 2014)	Sequence
TP71756_Hit	D	1	chr8	.	CTGCATGATGGATCATGACGCGGTAGTGGATCGGGACCGTGGGATTGACTTGATGATTAGGTTG
TP71756_Query	D	1	chr8	.	CTGCATGATGGACCATGACGCGGTAGTGGATCGGGACCGTGGGATTGACTTGATGATTAGGTTG
TP71855_Hit	D	1	chr8	.	CTGCATGCCTACTGGAACTTTGATTAATAAATTTGACTGGTTGCAGAGAGTTGGCTGTACCCTG
TP71855_Query	D	1	chr8	.	CTGCATGCCTACTGGAACTCTGATTAATAAATTTGACTGGTTGCAGAGAGTTGGCTGTACCCTG
TP71950_Hit	D	1	chr8	.	CTGCATGGCCATGTCACTGGAGTGACCAGCATTTGTTGTTTGGCCAAGATGGCTGGGTGGAACA
TP71950_Query	D	1	chr8	.	CTGCATGGCCATGTCACTGGAGTGACCAGCATTTGTTGTTGGGCCAAGATGGCTGGGTGGAACA
TP72379_Hit	D	1	chr8	.	CTGCATTATCAGTTTCTAAACATGTGGCACTACAAGATCCTCAAATAAATATCAGTTTTCTACG
TP72379_Query	D	1	chr8	.	CTGCATTATCAGTTTCTAAACATGTAGCACTACAAGATCCTCAAATAAATATCAGTTTTCTACG
TP72679_Hit	D+G	1	chr8	.	CTGCATTGCCTGCAAAGAACTCCTCTAAAAATCCCATTAGCTGAACACCGTAGAGAAGCCAAGG
TP72679_Query	D+G	1	chr8	.	CTGCATTGCCTGCAAAGAACTCCTCTAAAAATCCCATTAGCTGAACACCATAGAGAAGCCAAGG
TP72681_Hit	D	1	chr8	.	CTGCATTGCCTTGTCGCCTCTAACAGTCACATAGCAAGCAATCTTTTCATTTCTTCTAATACCA
TP72681_Query	D	1	chr8	.	CTGCATTGCCTTGTCACCTCTAACAGTCACATAGCAAGCAATCTTTTCATTTCTTCTAATACCA
TP73159_Hit	D	1	chr8	.	CTGCATTTTCAATATCAACCTTTCTGCATTATCTGTCTCACCATTCTGAGCAAACGCCCTCGCC
TP73159_Query	D	1	chr8	.	CTGCATTTTCAATATCAACCTTTCCGCATTATCTGTCTCACCATTCTGAGCAAACGCCCTCGCC
TP73189_Hit	D	1	chr8	.	CTGCATTTTGATGCTTCAACAAAATTAAGGTGCTATCGTGGTTTCGTCAAATTCGCTGAAAAAA
TP73189_Query	D	1	chr8	.	CTGCATTTTGATGCTTCAACAAAATTAAGATGCTATCGTGGTTTCGTCAAATTCGCTGAAAAAA
TP73505_Hit	D	1	chr8	.	CTGCCAAATGTGGTGGTCTGCTTGCAAAGACAAGATCATGGGTTTTGAGGAAAAGTTCAGCTGA
TP73505_Query	D	1	chr8	.	CTGCCAAATGTGGTGGTCTACTTGCAAAGACAAGATCATGGGTTTTGAGGAAAAGTTCAGCTGA
TP73519_Hit	D	1	chr8	.	CTGCCAAATTCTTCAAATCAACCACTGAAAGCTCGTCCAGACGCCTCACAACCAAGTCTGCTGA
TP73519_Query	D	1	chr8	.	CTGCCAAATTCTTCAAATCAACCACTGAAAGCTCGTCCAGACGCCTCACAACCAAGTCTGCAGA
TP73714_Hit	D+G	1	chr8	.	CTGCCAAGCAACTACAATACAAATTCTCCCCTGGGCAAATACCATCTGAGTTTAAACTTGTGGG
TP73714_Query	D+G	1	chr8	.	CTGCCAAGCAACTACAATACAAATTCTCCCCTGGGCAAATACCATCTGAGTTTAAACTTGTAGG
TP7372_Hit	D	1	chr8	.	CAGCAATCATGGAACATGGATTTCATTAGATGGTTGCAGAATCTGAGCTTGTGGAGCTTTTTGA
TP7372_Query	D	1	chr8	.	CAGCAATCATGGAACATGAATTTCATTAGATGGTTGCAGAATCTGAGCTTGTGGAGCTTTTTGA
TP7393_Hit	D+G	1	chr8	.	CAGCAATCCAAGAGTTAGAAATGCTGGCAAGTTTGGACCTTAAAGCACCATTAAGAGATGAAAC
TP7393_Query	D+G	1	chr8	.	CAGCAATCCAAGAGTTAGAAATGCTGGCAACTTTGGACCTTAAAGCACCATTAAGAGATGAAAC
TP73973_Hit	D	1	chr8	.	CTGCCAATTGCTTTACCGTAAGATTACAAAAGTACATAATACAGACAGAATACGAGTGATCGTA
TP73973_Query	D	1	chr8	.	CTGCCAATTGCTTTAACGTAAGATTACAAAAGTACATAATACAGACAGAATACGAGTGATCGTA
TP7414_Hit	D	1	chr8	.	CAGCAATCCCCATTCTCTGTTTCTTATTTCTTCAATGATCTTTTCAGATTCTTCAGACCGTTTA
TP7414_Query	D	1	chr8	.	CAGCAATCCCCATTCTCTGTTTCTTATTTCTTCAATGATCTTCTCAGATTCTTCAGACCGTTTA
TP74147_Hit	D	1	chr8	.	CTGCCACAGGTTTTCCGGTTAGCTACAACAGCGCTACATCGGGGTACTCCGGTGCCTCATCGGA
TP74147_Query	D	1	chr8	.	CTGCCACAGGTTTTCCGGTTAGCTACAACAGCGCTACATCGGGGTACTCCGGTGCCTCATCAGA
TP74455_Hit	D	1	chr8	.	CTGCCACTGCTTCCACAAGAGGTAAGTTGTTTTATTCTTGATTTCAGAGTCGGCCGTCAAGTAG
TP74455_Query	D	1	chr8	.	CTGCCACTACTTCCACAAGAGGTAAGTTGTTTTATTCTTGATTTCAGAGTCGGCCGTCAAGTAG
TP74456_Hit	D	1	chr8	.	CTGCCACTGCTTCCACAAGAGGTAAGTTGTTTTGTTCTTGATTTTAGAGTCGGTGCTGTTAAGT
TP74456_Query	D	1	chr8	.	CTGCCACTACTTCCACAAGAGGTAAGTTGTTTTGTTCTTGATTTTAGAGTCGGTGCTGTTAAGT
TP7446_Hit	D	1	chr8	.	CAGCAATCGAATATAATGCAAAAAGTCTTCAGTATTTTGGTTGTTTTATCGAAGGAGGAGCGGT
TP7446_Query	D	1	chr8	.	CAGCAATCGAATATAACGCAAAAAGTCTTCAGTATTTTGGTTGTTTTATCGAAGGAGGAGCGGT
TP74603_Hit	D	1	chr8	.	CTGCCACTTTTCTGGTGCGACTCAACACTGAGCGCCACCACCATACCGCCGCGTAATCCTCTGG
TP74603_Query	D	1	chr8	.	CTGCCACTTTTCTGGTGCGACTCAACACCGAGCGCCACCACCATACCGCCGCGTAATCCTCTGG
TP74628_Hit	D	1	chr8	.	CTGCCAGAACCAAGCCAATCAAATCCTCCAACCAAGGCCTCTAGCTGTTCTATTTTGTCAACAT
TP74628_Query	D	1	chr8	.	CTGCCAGAACCAAGCCAATCAAATCCTCCAACCAAGGCATCTAGCTGTTCTATTTTGTCAACAT
TP74807_Hit	D	1	chr8	.	CTGCCAGCTCTCGGATCTACCAACCACATCGGGACGAACAATCAACCAAGCTCCTGGGTTGGAT
TP74807_Query	D	1	chr8	.	CTGCCAGCTCTCGGAGCTACCAACCACATCGGGACGAACAATCAACCAAGCTCCTGGGTTGGAT
TP74944_Hit	D	1	chr8	.	CTGCCAGTGTCATCCTGTCGGTCACGTGACTGTAAGATGGTTGGCATCTGGCCTGAAAACGTCC
TP74944_Query	D	1	chr8	.	CTGCCAGTGTCATCCTGTCGGTCACGCGACTGTAAGATGGTTGGCATCTGGCCTGAAAACGTCC
TP74949_Hit	D	1	chr8	.	CTGCCAGTTAACAATAACCACCACCATCTGTTGATGTTTCTTTTGCTTTTACCACCACTTTCAA
TP74949_Query	D	1	chr8	.	CTGCCAGTTAACAATAACCACCACCATCTGTTGATGTTTCTTTTGCTTTTACCACCACTATCAA
TP75040_Hit	D	1	chr8	.	CTGCCATAATTTTACCAAGAAGCATCAACCTGATGTGGAGGGACATCAAAGACTATACACTTGG
TP75040_Query	D	1	chr8	.	CTGCCATAATTTTACCAAGAAGCATCAACCTGACGTGGAGGGACATCAAAGACTATACACTTGG
TP75224_Hit	D	1	chr8	.	CTGCCATCATCCACCTCGATAACACGATCAACATTGCTCTCAGCCTCCCCCTCAGCTGAAAAAA
TP75224_Query	D	1	chr8	.	CTGCCATCATCCACCTCGATAACACGATCAACATTGCTCTCAGCCTCCCCCTCAGCAGAAAAAA
TP75538_Hit	D+G	1	chr8	.	CTGCCTTTAATGATGCAAACCGATGAGTGAGATTATGATGAGTGTCATTTAAGTATAATCAACC
TP75538_Query	D+G	1	chr8	.	CTGCCATTAATGATGCAAACCGATGAGTGAGATTATGATGAGTGTCATTTAAGTATAATCAACC

Name	Filter	Nb hit (Mt4.0)	Mt Chr (Mt4.0)	Ms Chr (Li et al., 2014)	Sequence
TP75562_Hit	D	1	chr8	.	CTGCCATTATGACTCTTATTTTCCAATGCCGTGACACAAGAGAAAACAGACACTCCTACTAAGG
TP75562_Query	D	1	chr8	.	CTGCCATTATGAATCTTATTTTCCAATGCCGTGACACAAGAGAAAACAGACACTCCTACTAAGG
TP75642_Hit	D	1	chr8	.	CTGCCATTGCTGTTGCTCATTCACGTTTCTAATTTCAATTACTTTTTTATTTCATTCACGCTAG
TP75642_Query	D	1	chr8	.	CTGCCATTGCTGTTGCTCATTCACGTTTCTAATTTCAATTAATTTTTTATTTCATTCACGCTAG
TP75818_Hit	D	1	chr8	.	CTGCCCAAATTCGTGCCATTGTTGCCTCTGTTGATTATCGTCTCTCGCCTGAGCATCGCCTCCC
TP75818_Query	D	1	chr8	.	CTGCCCAAATTCCTGCCATTGTTGCCTCTGTTGATTATCGTCTCTCGCCTGAGCATCGCCTCCC
TP75965_Hit	D	1	chr8	.	CTGCCCAGCAGAAATCACCTAAAGCTCCATCTGAAGGATCATCTAACGGGGTTGAAATAGAAGG
TP75965_Query	D	1	chr8	.	CTGCCCAGCAGAAATCACCTAAAGCTCCATCTGAAGGATCATCTAACGGGGTCGAAATAGAAGG
TP76115_Hit	D	1	chr8	.	CTGCCCCAAATCATCACCACCGATCGCCATTTGAACTCCAGGAGTTGCATGAATGAAACAAGGC
TP76115_Query	D	1	chr8	.	CTGCCCCAAACCATCACCACCGATCGCCATTTGAACTCCAGGAGTTGCATGAATGAAACAAGGC
TP76364_Hit	D	1	chr8	.	CTGCTCCTTTACTTCCAAGCTTAAATTCAAAAACCAAAAGCCAACACAAAAGGGTGTGAAGCAA
TP76364_Query	D	1	chr8	.	CTGCCCCTTTACTTCCAAGCTTAAATTCAAAAACCAAAAGCCAACACAAAAGGGTGTGAAGCAA
TP765_Hit	D+G	1	chr8	.	CAGCAAAAGTGCAGGCGATCCAGAGTTAGAAAAAAATTCATGGAAGATTTCAAGACTGACTCTGA
TP765_Query	D+G	1	chr8	.	CAGCAAAAGTGCAGGCAATCCAGAGTTAGAAAAAAATTCATGGAAGATTTCAAGACTGACTCTGA
TP7683_Hit	D+G	1	chr8	.	CAGCAATGAGTTTAAGGAATATGTCTTCGTCGTCTTCGGCTTTCTTTTCAGCCAATCAGTCACC
TP7683_Query	D+G	1	chr8	.	CAGCAATGAGTTTAAGGAATATGTCTTCGTCGTCTTCGGCATTCTTTTCAGCCAATCAGTCACC
TP76838_Hit	D	1	chr8	.	CTGCCCTGTTGCCAATAACTTCACTAATATGTCCACTGCTCCGGCTTGAATTGTTGCATTCCAG
TP76838_Query	D	1	chr8	.	CTGCCCTGTTGCCAATAACTTCACTAATATGTCCACTCCTCCGGCTTGAATTGTTGCATTCCAG
TP76865_Hit	D	1	chr8	.	CTGCCCTTCCATGGACAATAGTAGAATTGGCTACGTGCACGTGTAGTTTGAACGACGCTGAAAA
TP76865_Query	D	1	chr8	.	CTGCCCTTCCATGGACAATAGTAGAATTGGCTACGTGCACGTGTAGTTTGAACGACGCAGAAAA
TP77157_Hit	D+G	1	chr8	.	CTGCCGATAAATTTGAAGTTGCTTCATGCATGAGATATTGTAGTCGGTTGTTGCGAAATATGCA
TP77157_Query	D+G	1	chr8	.	CTGCCGATAAATTTGAAGTTGCTTCATGCATGAGACATTGTAGTCGGTTGTTGCGAAATATGCA
TP77211_Hit	D	1	chr8	.	CTGCCGATGGATGTCACCCACAATCCGTTGAGATCTATTTGATTCTTAAGAATCTTCTTCTTGA
TP77211_Query	D	1	chr8	.	CTGCCGATGGATGTCACCCACAATCCGTTGAGATCTATTTGATTCTCAAGAATCTTCTTCTTGA
TP77470_Hit	D	1	chr8	.	CTGCCGCTAGATCTTTTAATGGATTTGGTACCACTGGTGATGATGCCACCCGTAAGAAGGAACT
TP77470_Query	D	1	chr8	.	CTGCCGCTAGATCTTTTAATGGATTTGGTACCACTGGTGATGATGCCACCCGTAAGAAGGAACG
TP77673_Hit	D	1	chr8	.	CTGCTGGCTCAAAAACCATAGTTGATGTCTATTATTGAACTGCGTGAATAGAAAGTATGACTAG
TP77673_Query	D	1	chr8	.	CTGCCGGCTCAAAAACCATAGTTGATGTCTATTATTGAACTGCGTGAATAGAAAGTATGACTAG
TP77694_Hit	D+G	1	chr8	.	CTGCCGGGACAACTTCTGATCTTTATAATGCATATAAAGGACCTGAGGAAAAGAAAGATACTGG
TP77694_Query	D+G	1	chr8	.	CTGCCGGGACAACTTCTGATCTTTATAATGCATATAAAGGACCTGAGGAAAAGAAAGAAACTGG
TP78200_Hit	D+G	1	chr8	.	CTGCCTAGCATCCCCACCAGTGGTTGATCTCCTGGTGTTATTTTGGGGAGCTCCAGCTGAAAAA
TP78200_Query	D+G	1	chr8	.	CTGCCTAGCATCCCCACCAGTGGTTGATCTCCTGCTGTTATTTTGGGGAGCTCCAGCTGAAAAA
TP78204_Hit	D	1	chr8	.	CTGCCTAGCCTCACTGTTTGAACCTTCCGCCGTCTGAATATGGAGACCTTGCGGCGGTGCTGAA
TP78204_Query	D	1	chr8	.	CTGCCTAGCCTCACCGTTTGAACCTTCCGCCGTCTGAATATGGAGACCTTGCGGCGGTGCTGAA
TP78460_Hit	D	1	chr8	.	CTGCCTCATTTTCTCCTGGTACAAAGAAGATTCCAACTTGTCAAAAACCAGGTTTAAATGTACA
TP78460_Query	D	1	chr8	.	CTGCCTCATTTTCTCCTGGTACAAAGAAGATTCCAACTTGTCAAAAACCAGGTTTAAATATACA
TP7860_Hit	D	1	chr8	.	CAGCAATGGATTTAACAGGATGGGATTGAGAAGGTACATTAGGATGTGTCCTAGCACCAGATGA
TP7860_Query	D	1	chr8	.	CAGCAATGGATTTAACAGGATGGGATTGAGAAGGTACATTAGGATGCGTCCTAGCACCAGATGA
TP78692_Hit	D	1	chr8	.	CTGCCTCTGATGTTGTTGAAGACGTAGCGGGGAGGCGGGGCCAGACATTGCTGGCATTTGCTTA
TP78692_Query	D	1	chr8	.	CTGCCTCTGATGTTGTTGAAGACGTAGCGGGGAGGCAGGGCCAGACATTGCTGGCATTTGCTTA
TP78833_Hit	D	1	chr8	.	CTGCCTGAGTGGACTGATCTTGTCAAAACTGCAAAATTCAAAGAATTGGCTCCCTATGATCCTG
TP78833_Query	D	1	chr8	.	CTGCCTGAGTGGACTGATCTTGTCAAAACTGCGAAATTCAAAGAATTGGCTCCCTATGATCCTG
TP78948_Hit	D+G	1	chr8	.	CTGCCTGGAAAACAAAATCTGTATATGCATTAGAGGACAAATTCATAAAGGTAGCTTGGATATT
TP78948_Query	D+G	1	chr8	.	CTGCCTGGAAAACAAAATCTGTATATGCATTAGAGGACAAATTCATAAAGATAGCTTGGATATT
TP78968_Hit	D	1	chr8	.	CTGCCTGGCCGCCTGGTGGGAGGCATTTTGGGAGGAAGTTCGACTGAAGCTGAAAAAAAAAAAA
TP78968_Query	D	1	chr8	.	CTGCCTGGCCGCCTGGTGGGAGGCATTTTGGGAGGAAGTTCGACTGAAGCAGAAAAAAAAAAAA
TP79309_Hit	D	1	chr8	.	CTGCCTTCTTTTATGCATGCCAAGAACCTCTCCGGTAGGTGCCCTCCCTTATGCTTCAGTTCCC
TP79309_Query	D	1	chr8	.	CTGCCTTCTTTTATGCATGCCAAGAACCTCTCCGGTAGGTGCCCTCCCGTATGCTTCAGTTCCC
TP79359_Hit	D+G	1	chr8	.	CTGCCTTGCACCTTGAGTCCGTTCTCTGCTTTCTTTTGTCTCGCAATCACAGCCATCTGCCGAT
TP79359_Query	D+G	1	chr8	.	CTGCCTTGCACCTTGAGTCCGTTCTCTGCTTTCTTTTGTCCCGCAATCACAGCCATCTGCCGAT
TP79414_Hit	D	1	chr8	.	CTGCCTTGTCACTTCCTGGGGGACCACGCCAATCAAGCTCGGGTTCGTTAGCTGAAAAAAAAAA
TP79414_Query	D	1	chr8	.	CTGCCTTGTCACTTCCTGGGGGACCACACCAATCAAGCTCGGGTTCGTTAGCTGAAAAAAAAAA
TP79422_Hit	D	1	chr8	.	CTGCCTTGTGATGTAAGATATGTTATTGGTGCAGAATGGTTTAGTGTATAAATTTTTGGAAGGA
TP79422_Query	D	1	chr8	.	CTGCCTTGTGATGTAAGATATGTTATTGGTGCAGAATGGTTTAGCGTATAAATTTTTGGAAGGA

Name	Filter	Nb hit (Mt4.0)	Mt Chr (Mt4.0)	Ms Chr (Li et al., 2014)	Sequence
TP79566_Hit	D+G	1	chr8	.	CTGCCTTTTCCCCATTGATCATAGAATAGCATACCAGATCCGGATGCTTGCATGCTCCTCTTTG
TP79566_Query	D+G	1	chr8	.	CTGCCTTTTCCCCAATGATCATAGAATAGCATACCAGATCCGGATGCTTGCATGCTCCTCTTTG
TP7981_Hit	D+G	1	chr8	.	CAGCAGTGTATATTCAATTATTTGCGGTGTGATCCTGAGGATCATTATTTTCTTTTGACCGAGA
TP7981_Query	D+G	1	chr8	.	CAGCAATGTATATTCAATTATTTGCGGTGTGATCCTGAGGATCATTATTTTCTTTTGACCGAGA
TP79973_Hit	D	1	chr8	.	CTGCGACACCACAAACCGAGCAACGATGGAGGGGAGTACGTGGTGAGGCTGTTGCACTTAAATT
TP79973_Query	D	1	chr8	.	CTGCGACACCACAAACCGAGCAACGATGGAGGGGAGTACGTGGTGAGGCTGTTGCACCTAAATT
TP80054_Hit	D	1	chr8	.	CTGCGACCATTTTTGTCTAGTGCTACTTTTCCTTTCCTTCCTTTCTTGTCCATCTCTGTCCAGT
TP80054_Query	D	1	chr8	.	CTGCGACCATTTTTGTCTAGTGCTACTTTTCCTTTCCATCCTTTCTTGTCCATCTCTGTCCAGT
TP80280_Hit	D	1	chr8	.	CTGCGAGCAAATAATCAGGTGCTTCATGTAAGATGTCGAGTATACTTTCAATGATTCCAGCTTT
TP80280_Query	D	1	chr8	.	CTGCGAGCAAATAATCAGGTGCTTCATGTAAGATGTCGAGTATACTTTCAATGACTCCAGCTTT
TP80443_Hit	D+G	1	chr8	.	CTGCGATAAAAATCCGATTCACGATTCAGAAAATAGTTCTTCTGATTCACAATATGAATGATTC
TP80443_Query	D+G	1	chr8	.	CTGCGATAAAAATCCGATTCACGATTCAAAAAATAGTTCTTCTGATTCACAATATGAATGATTC
TP80532_Hit	D	1	chr8	.	CTGCGATCCTGACAACTATTGACCAAAATATATGACCTTCTCAAAGCATCTTCTAGTTGTTCTA
TP80532_Query	D	1	chr8	.	CTGCGATCCTGACAACTATTGACCAAAATATATGACCTTCTCAAAGCATCTTCTAATTGTTCTA
TP80533_Hit	D	1	chr8	.	CTGCGATCCTGTTAAACTTATTGACTTTTGACCTGTAGGTCCTGCCATCCTAGGAGACTGCTGA
TP80533_Query	D	1	chr8	.	CTGCGATCCTGTTAAACTTATTGACTTTTGACCTGTAGGTCCTGCCATCCTAGGAGACTGCAGA
TP80650_Hit	D+G	1	chr8	.	CTGCGATGTGGGGAATGTGGAAGAGGCAGACACACTTGTTGTCAAAGATATGTCTCCTGCTGAA
TP80650_Query	D+G	1	chr8	.	CTGCGATGTGAGGAATGTGGAAGAGGCAGACACACTTGTTGTCAAAGATATGTCTCCTGCTGAA
TP80744_Hit	D+G	1	chr8	.	CTGCGATTTCGGCCGATATCCAAACAACACAAACACATCCCTATGAGTTTGCTATAGCAGTCGG
TP80744_Query	D+G	1	chr8	.	CTGCGATTTCGGCCGATATCCAAACAACACAAACACATCCCTATGAGTTTGCTATAGCAGTCAG
TP81829_Hit	D	1	chr8	.	CTGCGGAACAAGCCCACTGGTTGAAACAGTGACCTTGCGGGGGCTGAAATGAAGCCCTTGGTCA
TP81829_Query	D	1	chr8	.	CTGCGGAACAAGCCCACTGGTTGAAACAGTGACCTTGCGGGGGCTGAAATGAAGCCCTTGATCA
TP81963_Hit	D	1	chr8	.	CTGCGGAGGAGCTCCCTGGAGAATATAGGGACCGTCATCAGGTGAATACTTTAGTGTCTCTTGC
TP81963_Query	D	1	chr8	.	CTGCGGAGGAGCTCCCTGAAGAATATAGGGACCGTCATCAGGTGAATACTTTAGTGTCTCTTGC
TP82174_Hit	D+G	1	chr8	.	CTGCGGCCCTTCAGCGATCCCAGATTCTGTCAAAGCATCACGCATAAAATGGCCAGGAAGCTGA
TP82174_Query	D+G	1	chr8	.	CTGCGGCCCTTCAGCGATCCCAGATTCTGTCAAAGCATCACGCATAAAATGCCCAGGAAGCTGA
TP82475_Hit	D	1	chr8	.	CTGCGGGATGGGAAGCTACTCCTCGCCAGTCGTATGGGTTTTTCAGAATAGAGATTCTCGGTGC
TP82475_Query	D	1	chr8	.	CTGCGGGATGGGAAGCTACTCCTCGCCAGTCGTACGGGTTTTTCAGAATAGAGATTCTCGGTGC
TP82610_Hit	D	1	chr8	.	CTGCGGGTGAGTGCACACTTGACTGGCCTTCGAGACTCAAAATCGCAATTGGTGCGGAGATCGG
TP82610_Query	D	1	chr8	.	CTGCGGGTGAGTGCACACTTGACTGGCCTTCGAGACTCAAAATCGCAATTGGTGCCGAGATCGG
TP8265_Hit	D	1	chr8	.	CAGCAATTCAATATTCATGGTAGAGAGTCTCAAGCTCCTCCGAGGCAACAAGTAAAAGCTGGAA
TP8265_Query	D	1	chr8	.	CAGCAATTCAATATTCATGGTAGAGAGTCTCAAGCTCCTCCGAGGCAACAAGTAAAAGCTGAAA
TP83401_Hit	D	1	chr8	.	CTGCGTCTCAGGACTTTTCTTGATACGTTTTGAGGAAGCAACTGGAGCCTCTTCAGGGAAATTC
TP83401_Query	D	1	chr8	.	CTGCGTCTCAGGACTTTTCTTGAGACGTTTTGAGGAAGCAACTGGAGCCTCTTCAGGGAAATTC
TP83553_Hit	D+G	1	chr8	.	CTGCGTGCTTTAACATTAGAGAGGAGTCAGATAAAGGGGGTTATGGGGTTTTCTTTGGATAACG
TP83553_Query	D+G	1	chr8	.	CTGCGTGCTTTAACATTAGAGAGGAGTCAGATAAAGGGGGTTATGGGGTTTTCTTTAGATAACG
TP83695_Hit	D+G	1	chr8	.	CTGCGTGTTGGTTAAGGCTACACATGTTGGATCAGACACTGCCCTTTCTCAAATTGTTCAACTC
TP83695_Query	D+G	1	chr8	.	CTGCGTGTTGGTTAAGGCTACACATGTTGGAGCAGACACTGCCCTTTCTCAAATTGTTCAACTC
TP83758_Hit	D	1	chr8	.	CTGCGTTAGGTTCCCTCACTCTTTTCCGATCCCTTGTTCAATCGTTCTGTTACCCTCTGGCTGA
TP83758_Query	D	1	chr8	.	CTGCGTTAGGTTCCCTCACTCTTTTCCGATCCCTTGTTCAATCATTCTGTTACCCTCTGGCTGA
TP83878_Hit	D	1	chr8	.	CTGCGTTGGAGTTGGAGGGTAACTTAACTGATGGTGTGGACACCGCCTCGGTGGATACAAGAAA
TP83878_Query	D	1	chr8	.	CTGCGTTGGAGTTGGAGGGTAACTTAACTGATGATGTGGACACCGCCTCGGTGGATACAAGAAA
TP83961_Hit	D	1	chr8	.	CTGCGTTTCGCCGTCTTGAAGCGGAGGAAGATATTCCAAAAGTGCAAAGCATCAGTCATGCAAC
TP83961_Query	D	1	chr8	.	CTGCGTTTCGCCGTCTTGAAGCGGAGGAAAATATTCCAAAAGTGCAAAGCATCAGTCATGCAAC
TP83976_Hit	D	1	chr8	.	CTGCGTTTGATAGAGCAACAGCTGAAGCTCGACAAAGAGCACTGGCTGAGGCCAGGGAAAGACT
TP83976_Query	D	1	chr8	.	CTGCGTTTGATAGAGCAACAGCTGAAGCTCGACAAAGAGCACTGGCTGAGGCCAGGGAAAAACT
TP84026_Hit	D	1	chr8	.	CTGCGTTTTGGTTTTGATTCAGAGTCATCACCGACACTGTCCTTCATTATATCTTTATGATTCT
TP84026_Query	D	1	chr8	.	CTGCGTTTTGGTTTTGATTCAGAATCATCACCGACACTGTCCTTCATTATATCTTTATGATTCT
TP8414_Hit	D	1	chr8	.	CAGCAATTGTAGATTCAACCTGCATAAGTTCAAACCACTCATCAAATCATGAATACAAAAGAAA
TP8414_Query	D	1	chr8	.	CAGCAATTGCAGATTCAACCTGCATAAGTTCAAACCACTCATCAAATCATGAATACAAAAGAAA
TP84187_Hit	D	1	chr8	.	CTGCTAAAGCTAAAACTACTTTTGGTGAGATGGTGATTCGTGTTGGTTGGAGTCAAGATAATGT
TP84187_Query	D	1	chr8	.	CTGCTAAAGCTAAAACTACTTTTGGTGAGATGGTGATCCGTGTTGGTTGGAGTCAAGATAATGT
TP84252_Hit	D	1	chr8	.	CTGCTAAATTGGCACACCTCATAACGAGATCAAAGGTTAAAAAATTCCAAAAACACTTGTAGAA
TP84252_Query	D	1	chr8	.	CTGCTAAATTGCCACACCTCATAACGAGATCAAAGGTTAAAAAATTCCAAAAACACTTGTAGAA

Name	Filter	Nb hit (Mt4.0)	Mt Chr (Mt4.0)	Ms Chr (Li et al., 2014)	Sequence
TP84395_Hit	D	1	chr8	.	CTGCTAAGAACGATGGAACAGTTGCTTTGGTTAAATCTGGCTCCGAAATTGTCAAGACTACCCC
TP84395_Query	D	1	chr8	.	CTGCTAAGAACGATGGAACAGTTGCTTTGGTTAAATCTGGCTCCGAAATTGTCAAGACCACCCC
TP84552_Hit	D+G	1	chr8	.	CTGCTAATATTGCAACTGATAATGAATTAGGAGGCTTAGCCAAGTGAGTGAGTGTGATAGTTTT
TP84552_Query	D+G	1	chr8	.	CTGCTAATATTGCAACTGATAATGAATTAGGAGGCTTAGCCAAGTGAGTGAGTATGATAGTTTT
TP84572_Hit	D+G	1	chr8	.	CTGCTAATCCATACGCAGGAACCTATGGAACTTCTATCACTCAAAGCCCAGCTGGAATAGGAAT
TP84572_Query	D+G	1	chr8	.	CTGCTAATCCATACGCAGGAACCTATGGAACCTCTATCACTCAAAGCCCAGCTGGAATAGGAAT
TP84607_Hit	D+G	1	chr8	.	CTGCTATTGAGTCTACAGTCAATAATGGTTGATCAATCTGACTTGTCCCTTCCCCTTTTCCAAT
TP84607_Query	D+G	1	chr8	.	CTGCTAATGAGTCTACAGTCAATAATGGTTGATCAATCTGACTTGTCCCTTCCCCTTTTCCAAT
TP84989_Hit	D+G	1	chr8	.	CTGCTACCATAGTTCATAAAGCCATTGGGGATAGGCTTCATTGTGTTTTTGTTGACAATGGCTT
TP84989_Query	D+G	1	chr8	.	CTGCTACCATAGTTCATAAAGCCATTGGCGATAGGCTTCATTGTGTTTTTGTTGACAATGGCTT
TP8505_Hit	D+G	1	chr8	.	CAGCAATTGTTTTTGCCTTTGCTAAGGGCGTTTGAGATGTTCCTTCCATCATGCCCCTTGCTGA
TP8505_Query	D+G	1	chr8	.	CAGCAATTGTTTTTGCCTTTGCTAAGGGCGTTTGAGATGTTCCTTCCATCATGCCCCGTGCTGA
TP85162_Hit	D+G	1	chr8	.	CTGCTACTACTAATTCGATTGGTGTTGGAGATGGAGTCATCGGAAGCGGGTAAAGGTAGAATGC
TP85162_Query	D+G	1	chr8	.	CTGCTACTACTAATTCGATTGATGTTGGAGATGGAGTCATCGGAAGCGGGTAAAGGTAGAATGC
TP85210_Hit	D	1	chr8	.	CTGCTACTCAACAACATCATTTAATGATGTGTGTAAGTTTCCCATAGGCTCGTGGGCTTGGGGT
TP85210_Query	D	1	chr8	.	CTGCTACTCAACAACATCATTTAATGATGTGTGTAAGTTTCCCATAGGCTCGTGGGCTTGGGAT
TP8538_Hit	D	1	chr8	.	CAGCAATTTATTTGGTTCATGGTTGTTTAAATTGTTTACTTTTGAATTGATCACTCTGAGAGGG
TP8538_Query	D	1	chr8	.	CAGCAATTTATTTGGTTCATGGCTGTTTAAATTGTTTACTTTTGAATTGATCACTCTGAGAGGG
TP85389_Hit	D	1	chr8	.	CTGCTACTTGAGTAACCTGCAGGAGCCGAATCATTTGCTGAAAAAAAAAAAAAAAAAAAAAAAA
TP85389_Query	D	1	chr8	.	CTGCTACTTGAGTAACCTGCAGGAGCCGAATCAATTGCTGAAAAAAAAAAAAAAAAAAAAAAAA
TP85390_Hit	D+G	1	chr8	.	CTGCTACTTGAGTAACCTGCAGGAGCCGAATCATTTGCTGTGATTATGAATTGCCTACTGCATA
TP85390_Query	D+G	1	chr8	.	CTGCTACTTGAGTAACCTGCAGGAGCCGAATCAATTGCTGTGATTATGAATTGCCTACTGCATA
TP8542_Hit	D	1	chr8	.	CAGCAATTTCAATGTAATACTTCAAAATTTTAAATTCTGAATCACATTAGTTATTTTAGACAGT
TP8542_Query	D	1	chr8	.	CAGCAATTTCAATGTAATACTTCAAAATTTAAAATTCTGAATCACATTAGTTATTTTAGACAGT
TP85451_Hit	D	1	chr8	.	CTGCTAGAAGAAATTGAGTTATTTGGTCTGAGTTTTGCAAACTGGATTAACAGCCACACCTCAT
TP85451_Query	D	1	chr8	.	CTGCTAGAAGAAATCGAGTTATTTGGTCTGAGTTTTGCAAACTGGATTAACAGCCACACCTCAT
TP85668_Hit	D	1	chr8	.	CTGCTAGCTCTTCTATGTTTTCATCAATTAGGTCCGCAAATTTCATCATAAGTTTTGCTCTTTC
TP85668_Query	D	1	chr8	.	CTGCTAGCTCTTCTATGTTTTCATCAATTAGGTCCGCAAATTTCATCATAAGTTTTGCCCTTTC
TP85698_Hit	D+G	1	chr8	.	CTGCTAGGAACAAGCGGCGGAGATTTGAGTATACTGAGTCTTTTGATGTGCCGGCTAAGGAAGT
TP85698_Query	D+G	1	chr8	.	CTGCTAGGAACAAGCGGAGGAGATTTGAGTATACTGAGTCTTTTGATGTGCCGGCTAAGGAAGT
TP86125_Hit	D	1	chr8	.	CTGCTATAGTTCTATACATGTCAGATTTCTTGTGGTTTTAACTCTGATTTGGAATGTCCTTTAC
TP86125_Query	D	1	chr8	.	CTGCTATAGTTCTATACATGTCAGATTTCTCGTGGTTTTAACTCTGATTTGGAATGTCCTTTAC
TP8626_Hit	D+G	1	chr8	.	CAGCAATTTGTCAACAGCCAAGTGGAACATGGTCTGGACCTTGTGTCGGCTCTAACATATGCAA
TP8626_Query	D+G	1	chr8	.	CAGCAATTTGTCAACAGCCAAGTGGAACATGGTCTGGACCATGTGTCGGCTCTAACATATGCAA
TP86502_Hit	D+G	1	chr8	.	CTGCTATGGGTGGCAGTGCTTCCAATTTGTCGCGATCGGCTTTGATTGGACAGAGTGGTCATTT
TP86502_Query	D+G	1	chr8	.	CTGCTATGGGTGGCAGTGCTTCCAATTTGTCGCGATCGGCGTTGATTGGACAGAGTGGTCATTT
TP8679_Hit	D	1	chr8	.	CAGCAATTTTTTTTTCCGCTTCCTGGTATTCTTTCACAGCATCCATCATTCTTTGCCGAATTTT
TP8679_Query	D	1	chr8	.	CAGCAATTTTTTTTTCCACTTCCTGGTATTCTTTCACAGCATCCATCATTCTTTGCCGAATTTT
TP86857_Hit	D+G	1	chr8	.	CTGCTATTTCCCTAAGAGTGAAACTAGGATAGCATCGCGGTTTTCGTAGCGCATTCATTAAAAT
TP86857_Query	D+G	1	chr8	.	CTGCTATTTCCCTAAGAGTGAAACTAGGATAGCATCGCGGTTTTCGTAGCGCATTCATCAAAAT
TP86986_Hit	D	1	chr8	.	CTGCTCAAAAGGGCTGGAAAGTATTTCAGTTAGATGTCAAATCAGCTTTTTTAAATGGAGTATT
TP86986_Query	D	1	chr8	.	CTGCTCAAAAGGGCTGGAAAGTATTTCAGTTAGATGTCAAATCAGCTTTTTTAAATGGAGTAAT
TP87061_Hit	D	1	chr8	.	CTGCTCAGCACAACAGACATTCACTTTCGTTGGTTAGTATAAAAACAAATTAATAGGAGAGAGT
TP87061_Query	D	1	chr8	.	CTGCTCAACACAACAGACATTCACTTTCGTTGGTTAGTATAAAAACAAATTAATAGGAGAGAGT
TP8715_Hit	D	1	chr8	.	CAGCACAAAAATTTCAAACGAAGAGAGGGAAACAGTCTCCACTCACCTCTCATCATCTGGTTAT
TP8715_Query	D	1	chr8	.	CAGCACAAAAATTTCAAACGAAGAGAAGGAAACAGTCTCCACTCACCTCTCATCATCTGGTTAT
TP87160_Hit	D+G	1	chr8	.	CTGCTCAAGTCCAGTGATGAAAGTGGTTGATGAGGATCAAAATGCATGGACATGTTAGAGGTAC
TP87160_Query	D+G	1	chr8	.	CTGCTCAAGTCCAATGATGAAAGTGGTTGATGAGGATCAAAATGCATGGACATGTTAGAGGTAC
TP87163_Hit	D	1	chr8	.	CTGCTTAAGTCTCATTGAATTTTCTTTGTCCTCTGATTCAATTGAAGGGTTGGATTTAAGCAAC
TP87163_Query	D	1	chr8	.	CTGCTCAAGTCTCATTGAATTTTCTTTGTCCTCTGATTCAATTGAAGGGTTGGATTTAAGCAAC
TP8728_Hit	D	1	chr8	.	CAGCACAAAAGAAACGATAAACAAGTAAGGTATCAACTTAAATGCACACATTGCATGAGCTTTG
TP8728_Query	D	1	chr8	.	CAGCACAAAAGAAACGATAAACAAGTAAGGTATCAACTTAAATGCACACATTGCACGAGCTTTG
TP87528_Hit	D	1	chr8	.	CTGCTCATGGTGCAATCCCATGCCTTATTTATGACCAGACATACCTTTGCATGGTATAAGACTA
TP87528_Query	D	1	chr8	.	CTGCTCATGGTGCAATCCCATGCCTTATTTATGACCAGACACACCTTTGCATGGTATAAGACTA

Name	Filter	Nb hit (Mt4.0)	Mt Chr (Mt4.0)	Ms Chr (Li et al., 2014)	Sequence
TP8758_Hit	D	1	chr8	.	CAGCACAAAATTTCCCACCGCACTTTGATACTCGAATGGCTGGAGCAGATAACAACCGCTTTCC
TP8758_Query	D	1	chr8	.	CAGCACAAAATTTCCCACCGCACTTTGATACTCGAATGGCTGGAGCAGATAAAAACCGCTTTCC
TP87669_Hit	D	1	chr8	.	CTGCTCCACATATATCTCTTCTTGTAAAACTCCATTCAAAAAAGCTGATTTGACATCTAACTGG
TP87669_Query	D	1	chr8	.	CTGCTCCACATATATCTCTTCCTGTAAAACTCCATTCAAAAAAGCTGATTTGACATCTAACTGG
TP87787_Hit	D	1	chr8	.	CTGCTCCATTCTCGAAAATCTGGAAATTTCATTTAGTCCTGAATCTTTGGCCATAATTTGGGTG
TP87787_Query	D	1	chr8	.	CTGCTCCATTCTCGAAAATCTGGAAATTTCATTTAGTCCTGAATCTTTGGCCATAATTCGGGTG
TP88732_Hit	D+G	1	chr8	.	CTGCTCTCCGTAAGATTTGTCCTATGGTTAGCAGTGAGATCACGTGTGAAGCATCTGCTGAAAA
TP88732_Query	D+G	1	chr8	.	CTGCTCTACGTAAGATTTGTCCTATGGTTAGCAGTGAGATCACGTGTGAAGCATCTGCTGAAAA
TP88788_Hit	D	1	chr8	.	CTGCTCTATCTGCTAGTAATGTTGGAAAGGAGGATAAGGCGAGGCAATCTAGAGACAATAAGGT
TP88788_Query	D	1	chr8	.	CTGCTCTATCTGCTAGTAATGTTGGAAAGGAGGATAAGGCGAGGCAATCAAGAGACAATAAGGT
TP89011_Hit	D	1	chr8	.	CTGCTCTGATCTTGCAACGATGGGACTGCTGAGAAGTGTTTCACAATGGACCTACAATCTGTTC
TP89011_Query	D	1	chr8	.	CTGCTCTGATCTTGCAACGATGGGACTGCTGAGAAGGGTTTCACAATGGACCTACAATCTGTTC
TP89803_Hit	D+G	1	chr8	.	CTGCTGACGAGCTGGAACGGAGAAAACAGATTAATTATATACAAAGTTTACTTATCCAATGACC
TP89803_Query	D+G	1	chr8	.	CTGCTGACGAGCTGGAACAGAGAAAACAGATTAATTATATACAAAGTTTACTTATCCAATGACC
TP89941_Hit	D	1	chr8	.	CTGCTGAGGGAATGTCAAGAGCCTTACACATATGGTACAACTTTCCTGCATCAATGGCATCAGG
TP89941_Query	D	1	chr8	.	CTGCTGAGGGAATGTCAAGAGCCTTACACATATGGTACAACTTTCCTGCATCAATGGCATCAAG
TP89977_Hit	D+G	1	chr8	.	CTGCTGAGTATCGCTTTAGATATGGATATGAGATGCCTGTGGATGTGCTGTCTAAATGGTAGGT
TP89977_Query	D+G	1	chr8	.	CTGCTGAGTATCGCTTTAGATATGGATATGAGATGCCTGTGGATGTACTGTCTAAATGGTAGGT
TP89987_Hit	D+G	1	chr8	.	CTGCTGAGTCTGCTTCTGCCGATTCTCTTGGTTCTGCCTCTTCCCCTGGCCCTGCTGAAAAAAA
TP89987_Query	D+G	1	chr8	.	CTGCTGAGTCTGCTTCTGCCGATTCTCTTGGTTCTGACTCTTCCCCTGGCCCTGCTGAAAAAAA
TP90035_Hit	D	1	chr8	.	CTGCTGATAAAATTCATGGTCAGGTATTAAAATCTTCTGGCCAATTCCATGCATACACACTGAT
TP90035_Query	D	1	chr8	.	CTGCTGATAAAATTCATGGTCAGGTATTAAAATCTTCTGGCCAATTCCATGCATACACACTAAT
TP90079_Hit	D	1	chr8	.	CTGCTGATATAGTTCTGATCAAAAGTAACTTGGAAGATGTGATTACAGCCATAGATCTATCTAG
TP90079_Query	D	1	chr8	.	CTGCTGATATAGTTCTGATCAAAAGTAACTTGGAAGATGTGATTACAGCCATAGATCTATCCAG
TP90805_Hit	D	1	chr8	.	CTGCTGGGAAGAAATCACTGCAACATTTTGATTCAGAGGAGTCTGATCCTGAAGTAATTTGAAG
TP90805_Query	D	1	chr8	.	CTGCTGGGAAGAAATCACTGCAACATTTTGATTCAGAGGAGTCTGATCCTGAAGTAATTTAAAG
TP90959_Hit	D+G	1	chr8	.	CTGCTGGTAACTTCGAAGAAGCTTTGTCATTGTGCAAGCTCCTTCCACCTGAAGATTCAAATCT
TP90959_Query	D+G	1	chr8	.	CTGCTGGTAACTTCGAAGAAGCTTTGTCAATGTGCAAGCTCCTTCCACCTGAAGATTCAAATCT
TP90968_Hit	D+G	1	chr8	.	CTGCTGGTACTGGCCATGGTGGTGAACTTGGCTATGGCGAAGAGTGAAGCGTAGATACGGTGGT
TP90968_Query	D+G	1	chr8	.	CTGCTGGTACTGGCCATGGTCGTGAACTTGGCTATGGCGAAGAGTGAAGCGTAGATACGGTGGT
TP9102_Hit	D+G	1	chr8	.	CAGCACAATACAGAATACAAACACGGTATACTTGTTGATAACATAACTAACACTGAATGTCGTA
TP9102_Query	D+G	1	chr8	.	CAGCACAATACAGAATACAAACACGGTATACTTATTGATAACATAACTAACACTGAATGTCGTA
TP91442_Hit	D	1	chr8	.	CTGCTGTACTAGTTTGTTGGGTTTTGTGGGCCCATCAAATGGCATTTGGTGTCAAGCTTTTGCC
TP91442_Query	D	1	chr8	.	CTGCTGTACTAGTTTGTTGGGTTTTATGGGCCCATCAAATGGCATTTGGTGTCAAGCTTTTGCC
TP91504_Hit	D+G	1	chr8	.	CTGCTGTAGTAAGTAATTTGGATTAACTCCGTGATGGTTTGGGTGCTCCCATTGTGCCTGCTGA
TP91504_Query	D+G	1	chr8	.	CTGCTGTAGTAAGTAATTTGGATCAACTCCGTGATGGTTTGGGTGCTCCCATTGTGCCTGCTGA
TP91581_Hit	D	1	chr8	.	CTGCTGTCAATGAAGCGAGTGAAATGGTCTCTTGTCAACTTGAAGAGCTAGAAATTCCAGATAT
TP91581_Query	D	1	chr8	.	CTGCTGTCAATGAAGCCAGTGAAATGGTCTCTTGTCAACTTGAAGAGCTAGAAATTCCAGATAT
TP9161_Hit	D	1	chr8	.	CAGCACAATGCATGCGTTCCATTGAAGAGTTGTTGAATAAGGAAACTAAACCTGAAAAGGTACA
TP9161_Query	D	1	chr8	.	CAGCACAATGCATGCGTTCCATTGAAGAGTTGTTGAATAAGGAAACTAAACCTGAAAAGGTAAA
TP91757_Hit	D+G	1	chr8	.	CTGCTGTCGTGTTTGTGGTTGAGGAAATGAGATTACCCAATTTCAAGTTTCCCTTCGTGCTTTT
TP91757_Query	D+G	1	chr8	.	CTGCTGTCGTGTTTGTGGTTGAGGAAATGAGATAACCCAATTTCAAGTTTCCCTTCGTGCTTTT
TP92328_Hit	D+G	1	chr8	.	CTGCTGTTGACTAGCTAGTCAAACAGCTTCTCTGCTTCCATAGTCCAAACTACAATTAAATTGC
TP92328_Query	D+G	1	chr8	.	CTGCTGTTGACTAGCTAGTCAAACAGCTTCTCTGCTTCCATAGTCCAAACTACAACTAAATTGC
TP92922_Hit	D	1	chr8	.	CTGCTTAGCCCAGCATTTTTCCCACAGATGGGACAAACTTTCATTGTTGGTTTTCGCTGAAAAA
TP92922_Query	D	1	chr8	.	CTGCTTAGCCCAGCATTTTTCCCACAGATGGGACAAACTTTCATCGTTGGTTTTCGCTGAAAAA
TP92923_Hit	D+G	1	chr8	.	CTGCTTAGCCCAGCATTTTTCCCACAGATGGGACAAACTTTCATTGTTGGTTTTCGGTGCTGAA
TP92923_Query	D+G	1	chr8	.	CTGCTTAGCCCAGCATTTTTCCCACAGATGGGACAAACTTTCATCGTTGGTTTTCGGTGCTGAA
TP93286_Hit	D	1	chr8	.	CTGCTTCAGCAGTGCTCAACTTCAGTGAGAATTGCACACCTGAAATTTTAACGCCATACTTGGA
TP93286_Query	D	1	chr8	.	CTGCTTCAGCAGTGCTCAACTTCAGCGAGAATTGCACACCTGAAATTTTAACGCCATACTTGGA
TP93351_Hit	D	1	chr8	.	CTGCTTCATCTATTCATTCCTTTATTGCACCTACCCGAGGGACAGGGAGCGAGCACGAATGGCT
TP93351_Query	D	1	chr8	.	CTGCTTCATCTATTCATTCCTTTATTGCACCTACCCGAGGGACAGAGAGCGAGCACGAATGGCT
TP93422_Hit	D	1	chr8	.	CTGCTTCCACCATGTTCTCAAAATCTTTCGTTCCATAAGGATGAGAAAACCAGACGCTGTCACG
TP93422_Query	D	1	chr8	.	CTGCTTCCACCATGTTCTCAAAATCTTTCGTTCCATAAGGATGAGAAAACCAGACGCCGTCACG

Name	Filter	Nb hit (Mt4.0)	Mt Chr (Mt4.0)	Ms Chr (Li et al., 2014)	Sequence
TP9351_Hit	D+G	1	chr8	.	CAGCACACGAGAAGGAGATCTCAGTGATGATGGAGTGAAAGTTTTTTGAAACTTAGAATCTAGG
TP9351_Query	D+G	1	chr8	.	CAGCACACGAGAAGGAGATCTCAGTGACGATGGAGTGAAAGTTTTTTGAAACTTAGAATCTAGG
TP93557_Hit	D	1	chr8	.	CTGCTTCCTCGATCACCTCCTCCCCAAGATTTCCATCAGTGGGAGGAGCGGGTTGAGGAAGTAT
TP93557_Query	D	1	chr8	.	CTGCTTCCTCGATCACCTCCTCCCCAAGATTTCCATCAGTGGGAGGAGCGGGTAGAGGAAGTAT
TP93559_Hit	D	1	chr8	.	CTGCTTCCTCGATGGAGAGAGGACCAAGATTTCCATCAGTGGGAGGAGCGGGTAGAGGAAGTAT
TP93559_Query	D	1	chr8	.	CTGCTTCCTCGATGGAGAGAGGACCAAGATTTCCATCAGTGGGAGGAGCAGGTAGAGGAAGTAT
TP93897_Hit	D	1	chr8	.	CTGCTTCTGTTCATCCGTCGGCTCGTCAGTGGAATTAATCAAATTCCCATCATCAAGCTGAAAA
TP93897_Query	D	1	chr8	.	CTGCTTCTGTTCATCCGTCGGCTCGTCAGTGGAATTAATCAAACTCCCATCATCAAGCTGAAAA
TP93918_Hit	D	1	chr8	.	CTGCTTCTTATCTTTCACAAACAACATCATACTTAGCTGGTTGTTTCTCCGACTATTCAGGCAA
TP93918_Query	D	1	chr8	.	CTGCTTCTTATCTTTCACAAACAACATCATACTTAGCCGGTTGTTTCTCCGACTATTCAGGCAA
TP94203_Hit	D	1	chr8	.	CTGCTTGATTTCATATTTGAAGACGGTAAGACTACACCGGAAGTAAACGAAAGCATTCCATCGT
TP94203_Query	D	1	chr8	.	CTGCTTGATTTCATATTTGAAGACGGTAAGACTACACCGGAAGTAAAGAAAGCATTCCATCGT
TP94433_Hit	D	1	chr8	.	CTGCTTGGGTTCAATCCTTCTACTCAGAAACTGAGAAGGAGCAAAACAAGCACGCCATTGCTGT
TP94433_Query	D	1	chr8	.	CTGCTTGGGTTCAATCCTTCTACTCAGAAACTGAGAAGGAGCAAAACAAGCACGCCATTGCGGT
TP94495_Hit	D+G	1	chr8	.	CTGCTTGTAAGAAATGGCCTCTATAGTCTTGAATGCACATACCTATTCCAAAGAACCTCTACTC
TP94495_Query	D+G	1	chr8	.	CTGCTTGTAAGAAATGGCCTCTATAGTCTCGAATGCACATACCTATTCCAAAGAACCTCTACTC
TP94534_Hit	D	1	chr8	.	CTGCTTGTATGACATGGGAGTTGGCAGAGTCAGGCAGTAGTTTTATTACTTCTATCATAAATGG
TP94534_Query	D	1	chr8	.	CTGCTTGTATGACATGGGAGTTGGCAGAGTCAGGCAATAGTTTTATTACTTCTATCATAAATGG
TP9494_Hit	D	1	chr8	.	CAGCACAGATTCGCAGACTCGCCTCCGCTGTCTTCATTAGTTTTGATCTCGCTTTCTGGCGTGC
TP9494_Query	D	1	chr8	.	CAGCACAGATTCGCAGACTCGCCTCCGCTGTCTTCATTAGTTTTGATCCCGCTTTCTGGCGTGC
TP95044_Hit	D	1	chr8	.	CTGCTTTCTCTCACTACGACAGCTCTCTGGAAATCCTCTACTCCGGTAACCAAGTCGGGTTCAT
TP95044_Query	D	1	chr8	.	CTGCTTTCTCTCACTACGACAGCTCTCTCGAAATCCTCTACTCCGGTAACCAAGTCGGGTTCAT
TP95075_Hit	D+G	1	chr8	.	CTGCTTTCTTATTGGAAAGGGTTGGTGATGTTGGTAGTGCTCTTTCACTTACACTTTCTGATCT
TP95075_Query	D+G	1	chr8	.	CTGCTTTCTTACTGGAAAGGGTTGGTGATGTTGGTAGTGCTCTTTCACTTACACTTTCTGATCT
TP95310_Hit	D	1	chr8	.	CTGCTTTGTAAATAGTGAACAATTGCTAGTAGAATATGCTTGAGCTTTTAACTTGATTTTCAAG
TP95310_Query	D	1	chr8	.	CTGCTTTGTAAATAGTGAACAATTGCTAGTAGAACATGCTTGAGCTTTTAACTTGATTTTCAAG
TP95450_Hit	D	1	chr8	.	CTGCTTTTCATATGTTTGTACTGCACCAGTTGAATCGGATGACAGTCTTTCCATTGTCACAGGA
TP95450_Query	D	1	chr8	.	CTGCTTTTCATATGTTTGTACCGCACCAGTTGAATCGGATGACAGTCTTTCCATTGTCACAGGA
TP95586_Hit	D+G	1	chr8	.	CTGCTTTTGCACCATTTCTGAAGGATTAGTAACCCTCGCACCATGAACATTTGCTGGATGATCC
TP95586_Query	D+G	1	chr8	.	CTGCTTTTGCACCATTTCAGAAGGATTAGTAACCCTCGCACCATGAACATTTGCTGGATGATCC
TP9642_Hit	D+G	1	chr8	.	CAGCACATAAATGTCACAAATGAACAATATATTCACAAACACCATAGAAAAAAAGTCTCTTACC
TP9642_Query	D+G	1	chr8	.	CAGCACATAAATGTCACAAATGAACAATATATTCACAAAAACCATAGAAAAAAAGTCTCTTACC
TP9839_Hit	D	1	chr8	.	CAGCACATGGAGAAGCCTCATTCAAAGTATTCAAGTAGGACGCGAAGAAGATGAAAACAGAATT
TP9839_Query	D	1	chr8	.	CAGCACATGGAGAACCCTCATTCAAAGTATTCAAGTAGGACGCGAAGAAGATGAAAACAGAATT
TP988_Hit	D	1	chr8	.	CAGCAGAATGGATTATTGGTAAGTGGTGTGAGAAGAAGAACAAAAGAGGGAGGGGTTTGGTGGA
TP988_Query	D	1	chr8	.	CAGCAAAATGGATTATTGGTAAGTGGTGTGAGAAGAAGAACAAAAGAGGGAGGGGTTTGGTGGA
TP9916_Hit	D	1	chr8	.	CAGCACATTCATTATTCAAGCTCACCTTTCTTTCACCCCTGAGGTGCTCATTAGGCGGTCGAAA
TP9916_Query	D	1	chr8	.	CAGCACATTCATTATTCAAGCTCACCTTTCTTTCACCCCTGAGGTGCTCATTACGCGGTCGAAA
TP9921_Hit	D	1	chr8	.	CAGCACATTCTAACACTCCTCCTTGATGCATATTTCGGTAACTCCAAGCAAACTTCGTTGAAGA
TP9921_Query	D	1	chr8	.	CAGCACATTCTAACACTCCTCCTTGATGCATATTTCGGTAACTCCAAGCAAACTTCATTGAAGA
TP3082_Hit	D+G	1	chr8	3B	CAGCAACAAGGCAAGCAACACAATAGAAAGAAAGAATTTACACCATCGTCGCCGTTACGCCGGC
TP3082_Query	D+G	1	chr8	3B	CAGCAACAAGGCAAGCAACACAATAGAAAGAAAGAATGTACACCATCGTCGCCGTTACGCCGGC
TP14244_Hit	D	1	chr8	4A	CAGCAGATGGTGCCATCGGAGCCTGGCTAAGTGTTCCCATTGAAGTTGTCGCTGAAAAAAAAAA
TP14244_Query	D	1	chr8	4A	CAGCAGATGGTGCCATCGGAGCCTGGCTAAGTGTTCCCATTGAAGTTGTCGCAGAAAAAAAAAA
TP35948_Hit	D	1	chr8	4A	CAGCGACGTTTCCGCTGTACCTTTACACTTACAAAGAGGATCGCCAAGATGCATTCTGCTCTTG
TP35948_Query	D	1	chr8	4A	CAGCGACGTTTCCACTGTACCTTTACACTTACAAAGAGGATCGCCAAGATGCATTCTGCTCTTG
TP46881_Hit	D+G	1	chr8	4A	CAGCTCATTGAGCGCTTTCATCTTCTTTCGAGTGGTCTCTAACTTCTCAAGTGGACAGAGACCA
TP46881_Query	D+G	1	chr8	4A	CAGCTCATTGAGCGCTTTCATCTTCTTTCGAGTGGTCTCTAACTTCTCAAGTGGACAGAGAACA
TP49759_Hit	D	1	chr8	4A	CAGCTGAAACCATTCCCTGCTTTATTGCGGCAACAATCCCAATTGAAGCAACTGATATGATCTG
TP49759_Query	D	1	chr8	4A	CAGCTGAAACCATTCCCTGCTTTATTGCGGAAACAATCCCAATTGAAGCAACTGATATGATCTG
TP6031_Hit	D+G	1	chr8	4A	CAGCAAGGATGTTCTCTGTCCACTTGAGAAGTTAGAGACCACTCGAAAGAAGATGAAAGCGCTC
TP6031_Query	D+G	1	chr8	4A	CAGCAAGGATGGTCTCTGTCCACTTGAGAAGTTAGAGACCACTCGAAAGAAGATGAAAGCGCTC
TP60762_Hit	D	1	chr8	4A	CTGCAAAGGCCGCTTTTGAAGCAACTTTTAAGGGTTTGGTGAAAAGTCATGAGAAGAGAGCATC
TP60762_Query	D	1	chr8	4A	CTGCAAAAGCCGCTTTTGAAGCAACTTTTAAGGGTTTGGTGAAAAGTCATGAGAAGAGAGCATC

Name	Filter	Nb hit (Mt4.0)	Mt Chr (Mt4.0)	Ms Chr (Li et al., 2014)	Sequence
TP68107_Hit	D	1	chr8	4A	CTGCAGATCATATCAGTTGCTTCAATTGGGATTGTTTCCGCAATAAAGCAGGGAATGGTTTCAG
TP68107_Query	D	1	chr8	4A	CTGCAGATCATATCAGTTGCTTCAATTGGGATTGTTGCCGCAATAAAGCAGGGAATGGTTTCAG
TP87114_Hit	D+G	1	chr8	4A	CTGCTCAAGACCTTCCTTCTTCCATGAACGGACTAAGACTAGCCGTTTGCGTTAGGAAGAAGGA
TP87114_Query	D+G	1	chr8	4A	CTGCTCAAGACCTTCCTACTTCCATGAACGGACTAAGACTAGCCGTTTGCGTTAGGAAGAAGGA
TP92726_Hit	D	1	chr8	4A	CTGCTTAAAGTGCCAATGGCTGGTAATCTAAACTGCCAAGCTGTGCCTTTCAAACTGTTCTGGC
TP92726_Query	D	1	chr8	4A	CTGCTTAAAGTGCCAATGGCTGGTAATCTAAACTACCAAGCTGTGCCTTTCAAACTGTTCTGGC
TP14366_Hit	D	1	chr8	4B	CAGCAGATTTTACGTTCATTACAAATTGTTTATTAAAATGAACATAGTTGCTCCTGGGTTCTAG
TP14366_Query	D	1	chr8	4B	CAGCAGATTTTACGTTCATTACAAATTGTGTATTAAAATGAACATAGTTGCTCCTGGGTTCTAG
TP27676_Hit	D	1	chr8	4B	CAGCCCAAAGAAAAGCACCTAGAGCAATAACATGTGCACGATTGTGTCGCGTGGCGAGGTAAGC
TP27676_Query	D	1	chr8	4B	CAGCCCAAAGAAAAGCACCTAGAGCAATAACATGTGCACGATTGTATCGCGTGGCGAGGTAAGC
TP61943_Hit	D	1	chr8	4B	CTGCAACAAACTTTTTAAGCCGAGAGCCACTGAAATTACTGAATAACTGAAACTCACTCTTAAT
TP61943_Query	D	1	chr8	4B	CTGCAACAAACTTTTTAAGCCGAGAGCCACTGAAATTACTGAATAACTGAAACTCACTCTAAAT
TP63937_Hit	D	1	chr8	4B	CTGCATTATCTCAGAAGTCTGCTTTGCACATAACTCTTCCAGAGCCATTTCTCTCCTTATGGAT
TP63937_Query	D	1	chr8	4B	CTGCAATATCTCAGAAGTCTGCTTTGCACATAACTCTTCCAGAGCCATTTCTCTCCTTATGGAT
TP91876_Hit	D	1	chr8	4B	CTGCTGTGAGTTTTGTAACTACACCTATCCTTATTTTTTTGTATTTTGTAGTATTTCTATTTCC
TP91876_Query	D	1	chr8	4B	CTGCTGTGAGTTTTGTAACTACACCTATCCTTATTTATTTGTATTTTGTAGTATTTCTATTTCC
TP63942_Hit	D+G	1	chr8	4C	CTGCAATATCTTTTACTTTAGTTGTTTGGACATGATGTAATGTGACTATGCTCACATCAGGAAT
TP63942_Query	D+G	1	chr8	4C	CTGCAATATCTTTTACTTTAGTTGTTTGGACATGATGTAATGTGACCATGCTCACATCAGGAAT
TP93539_Hit	D+G	1	chr8	4C	CTGCTTCCGTATTTGCTTCTTCCTTTGCGGCGGTTAAATTTCGGCCTTCCTTCACCGGCATCGG
TP93539_Query	D+G	1	chr8	4C	CTGCTTCCGTATTTGCTTCTTCCTCTGCGGCGGTTAAATTTCGGCCTTCCTTCACCGGCATCGG
TP19060_Hit	D	1	chr8	4D	CAGCATCCTGGAGCCATCTTTCTTACTCGCAAGCATATCTACATTTACATATGTGGCACCATTA
TP19060_Query	D	1	chr8	4D	CAGCATCCTGGAGCCATCTTTCTTACTCGCAAGCATATCTACATTTACATACGTGGCACCATTA
TP28059_Hit	D+G	1	chr8	4D	CAGCCCAGAGCAATTCGTCCTGCGCGCACAATTACCGACATTCAATTATTACCATTCAAGCAAT
TP28059_Query	D+G	1	chr8	4D	CAGCCCAGAGCAATTCGTCCTGCGCGCACAATTACCGACATTCAATTATTAACATTCAAGCAAT
TP3222_Hit	D	1	chr8	4D	CAGCAACACAAGCAGGGATGTCTTGTGTGGTTACCTACACATCTTCCACTGCTGAGCAGGTGAG
TP3222_Query	D	1	chr8	4D	CAGCAACACAAGCAGGGATGTCTTGTGTGGTTACCTACACATCTTCCACCGCTGAGCAGGTGAG
TP34683_Hit	D	1	chr8	4D	CAGCCTTGGCAGTGACAGTGAAGTTGGTATCAACACCAGCCAACATCTTAAGAAATCCTTGGAA
TP34683_Query	D	1	chr8	4D	CAGCCTTGGCAGTCACAGTGAAGTTGGTATCAACACCAGCCAACATCTTAAGAAATCCTTGGAA
TP42343_Hit	D	1	chr8	4D	CAGCTAAGAAATCACCCTTTGTTGGAACTTGGTCATCCCTAGATTTGATAACTTCTAAGTAAGA
TP42343_Query	D	1	chr8	4D	CAGCTAAGAAATCACCCTTTGTTGGAACTTGGTCATCCCTAGATTTGATAACTTCCAAGTAAGA
TP44931_Hit	D+G	1	chr8	4D	CAGCTATCCTTGTAACATTTTGTGGAGTGTCTTACTTGGAAGTTATCAAATCTAGGGATGACCA
TP44931_Query	D+G	1	chr8	4D	CAGCTATCCTTGTAACATTTTGTGGAGTGTCTTACTTAGAAGTTATCAAATCTAGGGATGACCA
TP47167_Hit	D+G	1	chr8	4D	CAGCTCCAGACAAAACAATGCGTACATTACCCCCTAAAGCTTGTTTTACCTGATCATGTTGCAA
TP47167_Query	D+G	1	chr8	4D	CAGCTCCAGACAAAACAATACGTACATTACCCCCTAAAGCTTGTTTTACCTGATCATGTTGCAA
TP54949_Hit	D	1	chr8	4D	CAGCTTCAGTTTTGAATTGTTCTTCCAACTGCAAAACAAAATTCCTGATGTTACCTATTTGTTG
TP54949_Query	D	1	chr8	4D	CAGCTTCAGTTTTGAATTGTTCTTCCAACTGCAAAACAAAATTCCTGATGTTACATATTTGTTG
TP61063_Hit	D+G	1	chr8	4D	CTGCAAACATTTTCACAATCTTTGAATGACTGTTGTGAATAGTGATTTGTTATTTCATACATTG
TP61063_Query	D+G	1	chr8	4D	CTGCAAACATTTTCACAATCTTCGAATGACTGTTGTGAATAGTGATTTGTTATTTCATACATTG
TP6945_Hit	D	1	chr8	4D	CAGCAATACTTGATGGAAGACAAAAGTTACCAAAAGATTTCTACAAAGAGTTCCTTCGATTACC
TP6945_Query	D	1	chr8	4D	CAGCAATACTTGATGGAAGACAAAAGTTACCAAAAGATTTCTACAAAGAGTTCATTCGATTACC
TP70311_Hit	D	1	chr8	4D	CTGCATAAATACTCTCTAGTGTCTTGTTTTCTTATGTAAGAACTTGTTTTCAGGCTGGCGCTTT
TP70311_Query	D	1	chr8	4D	CTGCATAAATACTCTCTAGTGTCTTGTTTTCTTATGTAAGAACTTGTTTTCAGGCCGGCGCTTT
TP73356_Hit	D	1	chr8	4D	CTGCCAAAGTGATTATCAGATGAGAGGAGCTACGCCAAAAATACAGTAAAGTTATATTATGTCT
TP73356_Query	D	1	chr8	4D	CTGCCAAAATGATTATCAGATGAGAGGAGCTACGCCAAAAATACAGTAAAGTTATATTATGTCT
TP78995_Hit	D+G	1	chr8	4D	CTGCCTGGGGATTAAATCATCATCTCATAAGTGCCTGGAGAAAGGTTTAGAGATGAATTGCCCA
TP78995_Query	D+G	1	chr8	4D	CTGCCTGGGGATTAAATCATCATCTCATAAGTGCCTAGAGAAAGGTTTAGAGATGAATTGCCCA
TP83227_Hit	D	1	chr8	4D	CTGCGTCACTACAGCGAGAGATGAATGTTAGGAACAAGAGAACTCTAAATAGGGACCTTCCAAA
TP83227_Query	D	1	chr8	4D	CTGCGTCACTACAGCGAGAGATGAATGTTAGGAACAAGAGAACTCTAAATAGGGACCTTCAAAA
TP85027_Hit	D+G	1	chr8	4D	CTGCTACTGAGTCTAGCATTAAGATCTGGGATTTGGAGAGCAAGAGCATTGTTGAGGATTTGAA
TP85027_Query	D+G	1	chr8	4D	CTGCTACCGAGTCTAGCATTAAGATCTGGGATTTGGAGAGCAAGAGCATTGTTGAGGATTTGAA
TP49499_Hit	D+G	1	chr8	5A	CAGCTCTTGTACGGTCTTTGCCTGTAGAAGAAGAACCAAAGCAAATTATTGTTACCCGTAAAGG
TP49499_Query	D+G	1	chr8	5A	CAGCTCTTGTAAGGTCTTTGCCTGTAGAAGAAGAACCAAAGCAAATTATTGTTACCCGTAAAGG
TP22526_Hit	D	1	chr8	8A	CAGCATTTTTAATACGTTTCTCCTCTGATTCCATGGCATAATCCTGCAAATATATGAGTAAATT
TP22526_Query	D	1	chr8	8A	CAGCATTTTTAATACGTTTCTCCTCTGATTCCATGGCATAATCCTGCAAATATATGAATAAATT

Name	Filter	Nb hit (Mt4.0)	Mt Chr (Mt4.0)	Ms Chr (Li et al., 2014)	Sequence
TP34052_Hit	D	1	chr8	8A	CAGCCTTAATCTGCAATAGCCTGTTCTCAAATTGATCTTTGTTATTTAATTTATTCATATATTT
TP34052_Query	D	1	chr8	8A	CAGCCTTAATCTGCAATAGCCTGTTCTCAAATTGATCTTTGTTATTTAATTTACTCATATATTT
TP48031_Hit	D	1	chr8	8A	CAGCTTGAACATTAGTATGGTCACCACCACCTATGCTAGCAATATTACTTGCAGAAGGGCAAGA
TP48031_Query	D	1	chr8	8A	CAGCTCGAACATTAGTATGGTCACCACCACCTATGCTAGCAATATTACTTGCAGAAGGGCAAGA
TP55813_Hit	D+G	1	chr8	8A	CAGCTTCTATCAATGCTATCTTAGTTGAACCAAACCATGTTCCAAGCAAGATCTTGATGGGTTC
TP55813_Query	D+G	1	chr8	8A	CAGCTTCTATCAATGCTATCCTAGTTGAACCAAACCATGTTCCAAGCAAGATCTTGATGGGTTC
TP57208_Hit	D	1	chr8	8A	CAGCTTGATGATTGGGTTTTGTGTCGAATATACAAGAAAAACTCAAGCTCGCAAAAGGAAATTT
TP57208_Query	D	1	chr8	8A	CAGCTTGATGATTGGGTTTTGTGTCGAATATACAAGAAAAACTCAAGCTCGCAAAAGGAAATTC
TP78770_Hit	D	1	chr8	8A	CTGCCTGAAAAAGCATGCTACTGACATCCGTGGACCAATTTTTGGTGCCAGAACTCTGTGTTTA
TP78770_Query	D	1	chr8	8A	CTGCCTGAAAAAGCATGCTACTGACACCCGTGGACCAATTTTTGGTGCCAGAACTCTGTGTTTA
TP85659_Hit	D	1	chr8	8A	CTGCTAGCTCCGGCGTACGGTTGCCGCCGGTGAAGGATGATGTTCCTGTTGTTACTCGTAGCAT
TP85659_Query	D	1	chr8	8A	CTGCTAGCTCCGGCGTAAGGTTGCCGCCGGTGAAGGATGATGTTCCTGTTGTTACTCGTAGCAT
TP95237_Hit	D	1	chr8	8A	CTGCTTTGGAAAATACAACTAAGTTGAAGAAGGCATTGTCATATTTGGAGAGGGCTAAGTTGTC
TP95237_Query	D	1	chr8	8A	CTGCTTTGGAAAATACAACTAAGTTGAAGAAAGCATTGTCATATTTGGAGAGGGCTAAGTTGTC
TP28481_Hit	D	1	chr8	8B	CAGCCCCAGAAACTTTTAACAATATTGTGTTGCAAATAAACCCCATATGCAGATATAGGTATCA
TP28481_Query	D	1	chr8	8B	CAGCCCCAGAAACTTTTAACAATATTGTGTTGCAAATAAACCCCATATACAGATATAGGTATCA
TP30434_Hit	D	1	chr8	8B	CAGCCGCAGTCATTCGGGTAATTCATTTGCAGAACCAACTCATTTGAACCAACCAGCTCAAACT
TP30434_Query	D	1	chr8	8B	CAGCCGCAGTCATTCGGCTAATTCATTTGCAGAACCAACTCATTTGAACCAACCAGCTCAAACT
TP34967_Hit	D	1	chr8	8B	CAGCCTTTGCATCATTTGCTAGACGGGTACGAGCATAGTCGAGGGAGTAGACAAAGAAAAGGGA
TP34967_Query	D	1	chr8	8B	CAGCCTTTGCATCATTTGCTAGACGGGTACGAGCATAGTCAAGGGAGTAGACAAAGAAAAGGGA
TP3662_Hit	D	1	chr8	8B	CAGCAATATATGGCTTAATATTCAAGTGATATAATAGGGTGGGTATCTAGGGTAAAAGACTCGT
TP3662_Query	D	1	chr8	8B	CAGCAACATATGGCTTAATATTCAAGTGATATAATAGGGTGGGTATCTAGGGTAAAAGACTCGT
TP42185_Hit	D	1	chr8	8B	CAGCTAACATATGTAAAATGGGTGCTTGCAAAATTATAAGTGGTTACCTTTTTGTTGCATCCTA
TP42185_Query	D	1	chr8	8B	CAGCTAACATATGTAAAATGGGTGCTTACAAAATTATAAGTGGTTACCTTTTTGTTGCATCCTA
TP52053_Hit	D+G	1	chr8	8B	CAGCTGTAACGAGACTCTTTTCTTCTGCCTTTTGTTTGAGAAAAAGGTGAATCTGAGCCTTCCT
TP52053_Query	D+G	1	chr8	8B	CAGCTGTAACGAGACTCTTTTCTTCTGCCTCTTGTTTGAGAAAAAGGTGAATCTGAGCCTTCCT
TP58079_Hit	D+G	1	chr8	8B	CAGCTTGTCATGATTCTTCACGGTGGAGTTTGCAATCATATTACCTCATCGTTATCTCAAGATT
TP58079_Query	D+G	1	chr8	8B	CAGCTTGTCATGATTCTTCACGATGGAGTTTGCAATCATATTACCTCATCGTTATCTCAAGATT
TP90454_Hit	D+G	1	chr8	8B	CTGCTGGACTTGGAGCTCTAGCTCCAACTTTGGGTACTCTGATTCCTGTAATCGGTGCCGGTGG
TP90454_Query	D+G	1	chr8	8B	CTGCTGGACTTGGAGCTCTAGCTCCAACTCTGGGTACTCTGATTCCTGTAATCGGTGCCGGTGG
TP91131_Hit	D+G	1	chr8	8B	CTGCTGGTGCTTCATCCCTTTTCTTTGTCTACTCCCTTGACTATGCTCGTACCCGTCTAGCAAA
TP91131_Query	D+G	1	chr8	8B	CTGCTGGTGCTTCATCCCTTTTCTTTGTCTACTCCCTCGACTATGCTCGTACCCGTCTAGCAAA
TP95346_Hit	D	1	chr8	8B	CTGCTTTGTGGGGATGTGTTTGTATGCAATGGATCAATAGGTTGCACTAACAACAATTATGCAT
TP95346_Query	D	1	chr8	8B	CTGCTTTGTGGGGATGTGTTTGTATGCAATGGAACAATAGGTTGCACTAACAACAATTATGCAT
TP14082_Hit	D	1	chr8	8C	CAGCAGATATTTGGTATCTACTACATAAATTTTCTGGGCAATAGGATTTATCGGCACTCCGCAA
TP14082_Query	D	1	chr8	8C	CAGCAGATATTTGGTATCTACTACATAAATTTTCTGGGCAATAGGATTTACCGGCACTCCGCAA
TP16971_Hit	D	1	chr8	8C	CAGCATAAAATTAGAAAATAAAAACTGTGCCTAATCTACTGATTGTCTTCTTATGCCGTGGAAG
TP16971_Query	D	1	chr8	8C	CAGCATAAAATTAGAAAATAAAAACTGTGACTAATCTACTGATTGTCTTCTTATGCCGTGGAAG
TP38584_Hit	D	1	chr8	8C	CAGCGGAGATTCATTTTTACGATTACTTTCGGAGATATGGTATGTATGCTTTACAACTGAAAAA
TP38584_Query	D	1	chr8	8C	CAGCGGAGATTCATTTTTACGATTACTTTCGGAGATATGGTATGTACGCTTTACAACTGAAAAA
TP71637_Hit	D	1	chr8	8C	CTGCATGAAGTGTGCGAGGCTGAACTTCATAACGTGCCACAAACAACCTTCACAACAACTTCTC
TP71637_Query	D	1	chr8	8C	CTGCATGAAGTGTGCGAGGCTGAACTTCATAATGTGCCACAAACAACCTTCACAACAACTTCTC
TP72968_Hit	D+G	1	chr8	8C	CTGCATTTCTGTGGTGGACCCCTTTATACAAAACTTCTTCTCTGCAGACCTTAAAGAATGCTTT
TP72968_Query	D+G	1	chr8	8C	CTGCATTTCTGTGGTGGACCCCTTTATACAAAACTTATTCTCTGCAGACCTTAAAGAATGCTTT
TP73588_Hit	D	1	chr8	8C	CTGCCAACCACTTCCTGTCAGAACTGCTATATAGTTTTGTTATGGGTGGCTCAGTAAGAGGTGA
TP73588_Query	D	1	chr8	8C	CTGCCAACCACTTCCTGTCAGAACTGCTATATAGTTTTGTCATGGGTGGCTCAGTAAGAGGTGA
TP7466_Hit	D+G	1	chr8	8C	CAGCAATTGGCGCTGGTGGAGGACTTGGTGTTTCTTCTGGGAGTACATAGAGTTCTGGGGGGCA
TP7466_Query	D+G	1	chr8	8C	CAGCAATCGGCGCTGGTGGAGGACTTGGTGTTTCTTCTGGGAGTACATAGAGTTCTGGGGGGCA
TP78326_Hit	D+G	1	chr8	8C	CTGCCTCGAGTACTGAAGATAGAGCACATGAGCCTGCTGAGACTGATCACTTATCATCTGTTAA
TP78326_Query	D+G	1	chr8	8C	CTGCCTCAAGTACTGAAGATAGAGCACATGAGCCTGCTGAGACTGATCACTTATCATCTGTTAA
TP8461_Hit	D	1	chr8	8C	CAGCAATTGGCATTCCTAAAATGTTGTAACCAAGAGCCCAAATGTAGTTCAGCCGGATACGAGA
TP8461_Query	D	1	chr8	8C	CAGCAATTGGCATGCCTAAAATGTTGTAACCAAGAGCCCAAATGTAGTTCAGCCGGATACGAGA
TP13322_Hit	D	1	chr8	8D	CAGCAGAATCGGTTATAGTTAGTTAGCTGAAAAACTAAATGTAATTGGAATCATAAATTTTGAG
TP13322_Query	D	1	chr8	8D	CAGCAGAATCGGTTATAGTTAGTTAGCTAAAAAACTAAATGTAATTGGAATCATAAATTTTGAG

Name	Filter	Nb hit (Mt4.0)	Mt Chr (Mt4.0)	Ms Chr (Li et al., 2014)	Sequence
TP30215_Hit	D	1	chr8	8D	CAGCCGATCTCCCCCACCTCGTGATAGGTCGCGTTCTTGAGGAGTAGCTGAATAGGAACTGTAT
TP30215_Query	D	1	chr8	8D	CAGCCGATCTCCCCCACCTCGTGATAGGTCGCGTTCTTGAGGAGTAGCTGAATAGAAACTGTAT
TP59498_Hit	D	1	chr8	8D	CAGCTTTGCCTTCATTTGTAGTAGCGTTTTGTATTTTAACAATGCCTGAGTCACCAAGGTGGTT
TP59498_Query	D	1	chr8	8D	CAGCTTTGCCTTCATTTGTAGTAGCGTTTTGTATTTTAACAATGCCTGAGTCACCAAGATGGTT
TP71524_Hit	D	1	chr8	8D	CTGCATCTGTTGCAGGTTTGGCCTTTGAAGGTGCTGAAACTTCTTTAGCACCAGAAGCTGAAAA
TP71524_Query	D	1	chr8	8D	CTGCATCTGTTGCAGGCTTGGCCTTTGAAGGTGCTGAAACTTCTTTAGCACCAGAAGCTGAAAA
TP91982_Hit	D+G	1	chr8	8D	CTGCTGTGGCATAAACTGTGTATACCAAACCAAATGTTATGATTATTTCAGTAACAACTCCTCC
TP91982_Query	D+G	1	chr8	8D	CTGCTGTGGCATAAACTGTGTATACCAAACCAAATGTTATGATGATTTCAGTAACAACTCCTCC
TP93202_Hit	D+G	1	chr8	8D	CTGCTTCAAGTGCCAGAACCTTGTTGCTTTCCATGATGATATTGTTTCCAAAGATTTTCAGGTG
TP93202_Query	D+G	1	chr8	8D	CTGCTTCAAGTGCCAGAACCTCGTTGCTTTCCATGATGATATTGTTTCCAAAGATTTTCAGGTG
TP11047_Hit	D	0	.	1A	CAGCACCTTCTATTTTTGGCACATCCACTTCTTATCCATTTTGCACATGTTGCCATTTTCACTA
TP11047_Query	D	0	.	1A	CAGCACCTTCTATTTTTGGCACATCCACGTCTTATCCATTTTGCACATGTTGCCATTTTCACTA
TP14049_Hit	D	2	.	1A	CAGCAGATAGCTTCTCAGATATATCCCTTGACCCGTCAGTTAGATTTTCTTCCTTTCCATTCGG
TP14049_Query	D	2	.	1A	CAGCAGATAGCTTCTCAGATATATCCCTCGACCCGTCAGTTAGATTTTCTTCCTTTCCATTCGG
TP16646_Hit	D+G	0	.	1A	CAGCAGTTGAGTCGGCCAAAAAGGAACTTGAAGAAGTAAAGCTTAGCATAGAGAAAGCTACTTC
TP16646_Query	D+G	0	.	1A	CAGCAGTTGAGTCGGCCAAAAAGGAACTTGAAGAAGTAAAGCTTAACATAGAGAAAGCTACTTC
TP26794_Hit	D	0	.	1A	CAGCCATGAACAAGCAATTAGATACTCTAACTAAGGAGTTTCACAGTTTCACTATGGATAACGA
TP26794_Query	D	0	.	1A	CAGCCATGAACAAGCAATTAGAGACTCTAACTAAGGAGTTTCACAGTTTCACTATGGATAACGA
TP42643_Hit	D	0	.	1A	CAGCTAATCAATATCCACCTCCTCGCCGATTAAGCACAAATGAAATCCCCATGCTTGTCAATGA
TP42643_Query	D	0	.	1A	CAGCTAATCAATATCCACCTCCTCGCCGATTAAGCACAAATGAAATCCACATGCTTGTCAATGA
TP44007_Hit	D	0	.	1A	CAGCTAGCAGATAATAAGTGATGTCTCGACCCTGCAACCAGCTAGGAGCTAGGTGGAGAAAGAA
TP44007_Query	D	0	.	1A	CAGCTAGCAGATAATAAGTGATGTCTCGACCCTGCAACCAGCTAGGAGCTAGGTGAAGAAAGAA
TP44015_Hit	D	0	.	1A	CAGCTAGCATATTTGGTGACAGATATACAACAACAGCCAATATAAACATTGAAGGCGAATGAGA
TP44015_Query	D	0	.	1A	CAGCTAGCATATTTGGGGACAGATATACAACAACAGCCAATATAAACATTGAAGGCGAATGAGA
TP45983_Hit	D	0	.	1A	CAGCTCAAGCTTCGCCACAGGACAGGTAACAACTCTTCCATGCTATATGGACGAAATTTGATTG
TP45983_Query	D	0	.	1A	CAGCTCAAGCTTCGCCACAGGACAGGTAACAACACTTCCATGCTATATGGACGAAATTTGATTG
TP48783_Hit	D+G	2	.	1A	CAGCTCTATTTATGTGCAATCTGTATCGATTCCTCTTCTCTGTATTAGTGCTCTGGATGATCCG
TP48783_Query	D+G	2	.	1A	CAGCTCTATTTATGTGCAATCTGCATCGATTCCTCTTCTCTGTATTAGTGCTCTGGATGATCCG
TP55009_Hit	D+G	0	.	1A	CAGCTTCATCAAAATAAAAAAGCTTTCCCGTTGCCAAGAAACATAAGTTTGAGTCGCAAGACAA
TP55009_Query	D+G	0	.	1A	CAGCTTCATCAAAACAAAAAAGCTTTCCCGTTGCCAAGAAACATAAGTTTGAGTCGCAAGACAA
TP63352_Hit	D	0	.	1A	CTGCAAGCTTCTGCAGTAACGCACCAGAAGCAGAATGGCCGGGAGAACCTCCAAAAAACGAACA
TP63352_Query	D	0	.	1A	CTGCAAGCTTCTGCAGTAACGCACCAGAAACAGAATGGCCGGGAGAACCTCCAAAAAACGAACA
TP69136_Hit	D+G	0	.	1A	CTGCAGGCACGTCTCCGGCAAGCCACTTTTCCTTGGCAAGCATCAGCCAAAGAACTTCAACTTG
TP69136_Query	D+G	0	.	1A	CTGCAGGCACGTCTCCGGCAAGCCACTTTTCCTTGGCAAGCATCAGCCAAAGAACTTCAACCTG
TP69884_Hit	D	0	.	1A	CTGCAGTGGTCGAAGTGTCTTGTTACCAAAGATAACAACATTTCCCAATTCAATATCATCTAGC
TP69884_Query	D	0	.	1A	CTGCAGTGGTCGAAGTGTCTTATTACCAAAGATAACAACATTTCCCAATTCAATATCATCTAGC
TP70708_Hit	D	0	.	1A	CTGCATATCAGAAGGATACAACGGAACATTATCCCGCTTACACCTTTTTTTTCCATGTATTCCA
TP70708_Query	D	0	.	1A	CTGCATATCAGAAGGATACAACGGAACATTATCCCGCTTACACCTTTTTTTACCATGTATTCCA
TP74392_Hit	D	0	.	1A	CTGCCACGCCTAGCCTAACATGAAGTAAAAGACTATTTTTTTGTTCAAATAGTCAAAAGCAGAT
TP74392_Query	D	0	.	1A	CTGCCACGCCTAGCCTAACATGAAGTAAAAGAATATTTTTTTGTTCAAATAGTCAAAAGCAGAT
TP75625_Hit	D	0	.	1A	CTGCCATTGATGCAAATGTTCATGCGCCAAATGAAACGATATTATAGCACTTGTATCTTTCTCC
TP75625_Query	D	0	.	1A	CTGCCATTGATGCAAATGTTCATGCGCCAAATGAAACGATATTATAACACTTGTATCTTTCTCC
TP80534_Hit	D	3	.	1A	CTGCGATCGAAAGCATGCCGTTGGCTTTAGAGAATGTTGTGAATGCAGTTTTCAATGGATCCAA
TP80534_Query	D	3	.	1A	CTGCGATCGAAAGCATGCCGTTGGCTTTAGAGAATGTTGTAAATGCAGTTTTCAATGGATCCAA
TP8370_Hit	D+G	0	.	1A	CAGCAATTGAATTGTCCTACTGTTTTTGCCTTGAAATCCATCAAGGCAAGTATCTTGATCTGTG
TP8370_Query	D+G	0	.	1A	CAGCAATTGAATTGTCCTACCGTTTTTGCCTTGAAATCCATCAAGGCAAGTATCTTGATCTGTG
TP86817_Hit	D+G	0	.	1A	CTGCTATTGTTAATATACCGAATGGAAAGGAAGAAAATCTAACTGACGGGTCGAGGGATATATC
TP86817_Query	D+G	0	.	1A	CTGCTATTGTTAATATACCGAATGGAAAGGAAGAAAATCTAACTGACGGGTCAAGGGATATATC
TP92800_Hit	D	2	.	1A	CTGCTTACAAAGGTTTTTCTGATTTAATTCGTCTTTTGTTATTTCTTGATGCACATCGGGGGCG
TP92800_Query	D	2	.	1A	CTGCTTACAAAGGTTTTTCTGATTTAATTCGTCTTTTGTTATTTCTTGATGCAAATCGGGGGCG
TP94863_Hit	D	0	.	1A	CTGCTTTATGGACATGAAAATAATTTGTTGGGATTTTTTCGGCTAGAATAATTAGTTTAGATTT
TP94863_Query	D	0	.	1A	CTGCTTTATGGACATGAAAATAATTTGTTGAGATTTTTTCGGCTAGAATAATTAGTTTAGATTT
TP18685_Hit	D	0	.	1B	CAGCATCAGTTGGTCTACTGGGTAAATATGTCATGAAAAATACTGTTGGAACTGTTTTATTTGA
TP18685_Query	D	0	.	1B	CAGCATCAGTTGGTCTACTGGGTAAATATGTCATGAAAAATACAGTTGGAACTGTTTTATTTGA

Name	Filter	Nb hit (Mt4.0)	Mt Chr (Mt4.0)	Ms Chr (Li et al., 2014)	Sequence
TP40394_Hit	D	0	.	1B	CAGCGTCAAAAAGAACAGTGTTTCTCCCTCCATCTGCACCAAGAGGGTAAATTCACACTTTTTA
TP40394_Query	D	0	.	1B	CAGCGTCAAAAAAAACAGTGTTTCTCCCTCCATCTGCACCAAGAGGGTAAATTCACACTTTTTA
TP40680_Hit	D+G	0	.	1B	CAGCGTCTGATTCGGCTCCAGTTCCCTTGGCAAGGCTCTATGAAATTTACGGCGAAGCTGAAAA
TP40680_Query	D+G	0	.	1B	CAGCGTCTGATTCGGCTCCAGTTCCCTTGGCAAGGCTCTATGAAATTTACGACGAAGCTGAAAA
TP55572_Hit	D+G	0	.	1B	CAGCTTCGTCGTAAATTTCATAGAGCCTTGCCAAGGGAACTGGAGCCGAATCAGACGCTGAAAA
TP55572_Query	D+G	0	.	1B	CAGCTTCGCCGTAAATTTCATAGAGCCTTGCCAAGGGAACTGGAGCCGAATCAGACGCTGAAAA
TP57234_Hit	D+G	0	.	1B	CAGCTTGATTAGCTTCTCCAAACACATGAGACCAATAGAAGTGACCTCCTTTTGTTTGGAAGAT
TP57234_Query	D+G	0	.	1B	CAGCTTGATTAGCTTCTCCAAAAACATGAGACCAATAGAAGTGACCTCCTTTTGTTTGGAAGAT
TP66810_Hit	D+G	0	.	1B	CTGCACTATGCACCCAAACCATCTTATCTGGAAGGGGATCCTTGGGGAGAGTAATCCTTTTAGT
TP66810_Query	D+G	0	.	1B	CTGCACTATGCACCCAAACCATCTTATCTGGAAGGGGATCCTTGGGGAGAGTAATCCGTTTAGT
TP69560_Hit	D	9	.	1B	CTGCAGTACATAAAAAAAGGGCAACCCGGTGCACTAAAGCTCTCGCATATGCAGGGTCCGGGAA
TP69560_Query	D	9	.	1B	CTGCAGTACATAAAAAAAGGGCAACCCGGTGCACTAAAGCTCTCGCATACGCAGGGTCCGGGAA
TP931_Hit	D	0	.	1B	CAGCAAAATCTGAGAAAATTCCTCAGGAAGTCGAAAGTAAAGAGAAGCAAGAAGAAACCAAGGA
TP931_Query	D	0	.	1B	CAGCAAAATCTGAGAAAATTCCTCAGGAAGTCGAAAGTAAAGAGAAACAAGAAGAAACCAAGGA
TP95467_Hit	D	0	.	1B	CTGCTTTTCCTCTATTATTTATGTAGTCCACAAAAGTTCAATTTATCAACACCATATAAAAGAA
TP95467_Query	D	0	.	1B	CTGCTTTTCCTCTATTATTTATGTAGTCCACAAAAGTCCAATTTATCAACACCATATAAAAGAA
TP12042_Hit	D	0	.	1C	CAGCACTCATTCGATTGGTCTCGGTTGCTCGTTGGATGGAGCAAAAGGTTGGTGATTTGAAAAG
TP12042_Query	D	0	.	1C	CAGCACTCATTCGATTGGTCTCGGTTGCTCGTTGGATGGAGCAAAAGGTTGGTGATTTGAAAAA
TP3424_Hit	D	0	.	1C	CAGCAACAGATGCACGGTCTTTTGCAAACATGTTATAGTTTTCCAACCTTGCCATTTAGAACAC
TP3424_Query	D	0	.	1C	CAGCAACAGATGCACAGTCTTTTGCAAACATGTTATAGTTTTCCAACCTTGCCATTTAGAACAC
TP70532_Hit	D	2	.	1C	CTGCATACTGGACAAACACAGCCTGTTCCAAAACAGGGATTTCTGGTTCCAATTTCAGAATGTG
TP70532_Query	D	2	.	1C	CTGCATACTGGACAAACACAGCCTGTTCCAAAGCAGGGATTTCTGGTTCCAATTTCAGAATGTG
TP125_Hit	D	0	.	1D	CAGCAAAAGCACCAACTGGTAAAATAAGCCGAAACACCCAGAAATGGTTGTGTAAACACGAGTC
TP125_Query	D	0	.	1D	CAGCAAAAACACCAACTGGTAAAATAAGCCGAAACACCCAGAAATGGTTGTGTAAACACGAGTC
TP21302_Hit	D	2	.	1D	CAGCATTCACATCCTTGGGAACAACATCGCCTCGGTACATCAAACAGCAAGCCATGTACTTTCC
TP21302_Query	D	2	.	1D	CAGCATTCACATCCTTGGGAACAACATCACCTCGGTACATCAAACAGCAAGCCATGTACTTTCC
TP39950_Hit	D	0	.	1D	CAGCGGTGTCAACGGGCACGTCATGAGCAGGGCCAGCAGAGTTACACGATGTTGAAAAACTATG
TP39950_Query	D	0	.	1D	CAGCGGTGTCAACAGGCACGTCATGAGCAGGGCCAGCAGAGTTACACGATGTTGAAAAACTATG
TP50847_Hit	D	0	.	1D	CTGCTGATGAGCCCAAGCCATCTCTTAATATGACCTCACAATAGCATGTCTTTTGATAAGGCTA
TP50847_Query	D	0	.	1D	CAGCTGATGAGCCCAAGCCATCTCTTAATATGACCTCACAATAGCATGTCTTTTGATAAGGCTA
TP54787_Hit	D	2	.	1D	CAGCTTCAGCACCACCCAACAAGTGAGGAATGATAAATTTATCAGCCAAATCCTTATTCAACAT
TP54787_Query	D	2	.	1D	CAGCTTCAGAACCACCCAACAAGTGAGGAATGATAAATTTATCAGCCAAATCCTTATTCAACAT
TP61071_Hit	D	0	.	1D	CTGCAAACCACAACCAAAACACACCCGAATAGCATTGGTGTGCCGCTACTGGACCGATTGTTTG
TP61071_Query	D	0	.	1D	CTGCAAACCACAACCAAAACACACCCGAATAGCATTGGTGTGCCGCAACTGGACCGATTGTTTG
TP65970_Hit	D	0	.	1D	CTGCACCGTCCAATAGCCAACCAGACAATTGTTCTTTTTGGCCTTCAATTCCTACAATTTCTGT
TP65970_Query	D	0	.	1D	CTGCACCATCCAATAGCCAACCAGACAATTGTTCTTTTTGGCCTTCAATTCCTACAATTTCTGT
TP66846_Hit	D	2	.	1D	CTGCACTCAACAAGCTGTCTAAGTCTGATATGGACTGGTATGGAGTTCTTCAGACTGAAAAGTT
TP66846_Query	D	2	.	1D	CTGCACTCAACAAGCTGTCTAAGTCTGATATGGACTGGTATGGAGTTCTTCAGACTGAAAAATT
TP82891_Hit	D	0	.	1D	CTGCGGTTATGTCTGCTGTGCTGGTTTTTGTGTTGTTTGTAGGTTCTGTTTGCTGTTTTGGTGG
TP82891_Query	D	0	.	1D	CTGCGGTTATGTCTGCTGTGCTGGTTTTTGTGTTGTTCGTAGGTTCTGTTTGCTGTTTTGGTGG
TP84756_Hit	D	0	.	1D	CTGCTACAAATGATAAGCAAAATTAGAGAAGAGTGTAAAAATGTACTTTTCTACTGCTTATCAT
TP84756_Query	D	0	.	1D	CTGCTACAAATGATAAGCAAAATGAGAGAAGAGTGTAAAAATGTACTTTTCTACTGCTTATCAT
TP16884_Hit	D	2	.	2A	CAGCAGTTTTGTCTTCGTCGTTGGAGATGCAGAGGAGGCCGTTACATGAACCAAGGAGAGAGAT
TP16884_Query	D	2	.	2A	CAGCAGTTTTGTCTTCGTCCTTGGAGATGCAGAGGAGGCCGTTACATGAACCAAGGAGAGAGAT
TP20633_Hit	D	0	.	2A	CAGCATGTCACGTACCTGTGTCCTGGCAAATTCGCGGAGTTTCTCAGTTGAAAAGAAGTTAGGT
TP20633_Query	D	0	.	2A	CAGCATGTCACGTACCTGTGTCCTGGCAAACTCGCGGAGTTTCTCAGTTGAAAAGAAGTTAGGT
TP21912_Hit	D	0	.	2A	CAGCATTGTGGAGAACAGTGTTACTTTCTTTGTCTATTTTTCCAGCAAGATGTCTCACCATTTT
TP21912_Query	D	0	.	2A	CAGCATTGTGGAGAACAGTGTTACTTTCGTTGTCTATTTTTCCAGCAAGATGTCTCACCATTTT
TP24356_Hit	D+G	0	.	2A	CAGCCACAATTGATGCTCTTTTGGTCATAATGTAATCATCTGTGTATTTGTTTTGGATACTTGT
TP24356_Query	D+G	0	.	2A	CAGCCACAATTGATGCTCTTTCGGTCATAATGTAATCATCTGTGTATTTGTTTTGGATACTTGT
TP43279_Hit	D	2	.	2A	CAGCTACCATGAACATGTGTTCCAAGTGCCACAAAGACTTGATGCTGAAACAGGAGCAGGCCAC
TP43279_Query	D	2	.	2A	CAGCTACCATGAACATGTGTTCCAAGTGCCACAAAGACATGATGCTGAAACAGGAGCAGGCCAC
TP46005_Hit	D+G	3	.	2A	CAGCTCAAGTAGACAAGACCTGTTGAACGCTGTTGGTTCAATGTATACCGCTGTTCTTTTCCTT
TP46005_Query	D+G	3	.	2A	CAGCTCAAGTAGACAAGACCTGTTGAACGCTGTTGGTTCAATGTATACCGCTGTTCTCTTCCTT

Name	Filter	Nb hit (Mt4.0)	Mt Chr (Mt4.0)	Ms Chr (Li et al., 2014)	Sequence
TP46045_Hit	D	0	.	2A	CAGCTCAATCAAAATAATTTGCAATTTGGAATTATAATTTGATGGAAGTAAGAATTGAAATTGG
TP46045_Query	D	0	.	2A	CAGCTCAATCAAAATAATTTGCAATTTGGAATTATAATTTGATGGAAGAAAGAATTGAAATTGG
TP49526_Hit	D	0	.	2A	CAGCTCTTTAGATTCTCTTTGGATGCTATATTTTAAACTCTATTCTCTCTATTGATATGTGTGT
TP49526_Query	D	0	.	2A	CAGCTCTTTAGAGTCTCTTTGGATGCTATATTTTAAACTCTATTCTCTCTATTGATATGTGTGT
TP61047_Hit	D+G	0	.	2A	CTGCAAACATCCCAAAACACCATCTTTTTCCTTGGATAACGGCAGAAACATATCATCCTCAAAC
TP61047_Query	D+G	0	.	2A	CTGCAAACATCCCAAAACACCATCTTTTTCCTTGGATAACGGCAGAAACATATCACCCTCAAAC
TP61377_Hit	D	0	.	2A	CTGCAAAGGGTTTCTCGAATCTCTCGAGAGTGAATTGGATGATACATCCAACGCTTGCAATGGT
TP61377_Query	D	0	.	2A	CTGCAAAGGGTCTCTCGAATCTCTCGAGAGTGAATTGGATGATACATCCAACGCTTGCAATGGT
TP65102_Hit	D	0	.	2A	CTGCACAACCTGGAAAATGGTCACCACCTCCGGAGAATTTCTTAAAGATCAAGGTTGATGCAGG
TP65102_Query	D	0	.	2A	CTGCACAACCTGGAAAATGGTCACCACCTCCGGAGAATTTCATAAAGATCAAGGTTGATGCAGG
TP6805_Hit	D+G	0	.	2A	CAGCAATAAGAAACACTATAATATTGAATTGCATAGCAGGAACAGCAACCGCAAAACCTCAAGT
TP6805_Query	D+G	0	.	2A	CAGCAATAAGAAACACTATAATATTGAATTGCATAGCAGGAACAGCAACCACAAAACCTCAAGT
TP70546_Hit	D+G	0	.	2A	CTGCATAGAAACGCCATTGAACACAACACACTGCAAGATAAAGCACATAGATCCAGTTACATTC
TP70546_Query	D+G	0	.	2A	CTGCATAGAAACGCCATTGAACACAACACACTGCAACATAAAGCACATAGATCCAGTTACATTC
TP74670_Hit	D+G	4	.	2A	CTGCCAGACCAGGTGTTAAGTAGACTGAGGCATACTTTGCCACAGCCATACAAATTAGGGTTGA
TP74670_Query	D+G	4	.	2A	CTGCCAGACCAGGTGTTAAGTAGACTAAGGCATACTTTGCCACAGCCATACAAATTAGGGTTGA
TP81293_Hit	D	2	.	2A	CTGCGCCTCCTTATTCCAATTAAGAACCACTTGAGGTTTTGTGGTTGCTGTTCCTGCTATGCAA
TP81293_Query	D	2	.	2A	CTGCGCCTCCTTATTCCAATTAAGAACCACTTGAGGTTTTGCGGTTGCTGTTCCTGCTATGCAA
TP85959_Hit	D	0	.	2A	CTGCTATAAAAGGTGCAGTGTTGTATCATAGTCACCACAATAATTGGGATCAAGATGAAGTGGC
TP85959_Query	D	0	.	2A	CTGCTATAAAAAGTGCAGTGTTGTATCATAGTCACCACAATAATTGGGATCAAGATGAAGTGGC
TP92439_Hit	D	3	.	2A	CTGCTGTTGGTGAAGATTTGGTTGCCATGACTAATTGTCACCTTCAGGATAGAAATTTCATCCC
TP92439_Query	D	3	.	2A	CTGCTGTTGGTGAAGATTTGGTTGCCATGACTAATAGTCACCTTCAGGATAGAAATTTCATCCC
TP94925_Hit	D	0	.	2A	CTGCTTTCAGCGTCTCCAATGCAGGACCAGCCTGATACTGGGTATGGAAACCAGATTATACGCA
TP94925_Query	D	0	.	2A	CTGCTTTCAGCGTCTCCAATGCAGGACCAGCCTGATACTGGGTATGGAAAACAGATTATACGCA
TP94971_Hit	D+G	0	.	2A	CTGCTTTCCGACTACATGAGTTGTTAAAAGCAGGACAATTGTGAATTGTGATGGCCTTTTCTCC
TP94971_Query	D+G	0	.	2A	CTGCTTTCCGACTACATGAGTTGTTAAAAGCAGGACAATAGTGAATTGTGATGGCCTTTTCTCC
TP25501_Hit	D	2	.	2B	CAGCCAGAAAGAAAAGCAAAACCAAGCTAAACTAAATGAGTACTCACAAATATTTCTAATTTTC
TP25501_Query	D	2	.	2B	CAGCCAGAAAGAAAAGCAAAACCAAACTAAACTAAATGAGTACTCACAAATATTTCTAATTTTC
TP28807_Hit	D+G	0	.	2B	CAGCCCCTGCATAATTACAGTTCATTTGACAGCACCTGTGTTTGTTGTTCTACCTGCCTGTTAA
TP28807_Query	D+G	0	.	2B	CAGCCCCTGCATAATTACAGTTCATTTGACAGCACCTGTGTTTGTTGATCTACCTGCCTGTTAA
TP38390_Hit	D+G	0	.	2B	CAGCGGAAATAAGATGCTCAAGTTGTTTCTTGTGATGGAATTTGTCGCCTTGGTTTGTGGAAAA
TP38390_Query	D+G	0	.	2B	CAGCGGAAATAAGATGCTCAAATTGTTTCTTGTGATGGAATTTGTCGCCTTGGTTTGTGGAAAA
TP46314_Hit	D+G	3	.	2B	CAGCTCACTTCCTTTGATAACAATATTGGGGAAATCCAGTAAGTGAACCTCCAATGGGTCCAAC
TP46314_Query	D+G	3	.	2B	CAGCTCACTTCCTTTGATAACAATATTGGGGAAATCCAGCAAGTGAACCTCCAATGGGTCCAAC
TP54469_Hit	D	0	.	2B	CAGCTTCAGCATCAATAATTCAGCAGAAACAATCATTAACAGTTTCCACACCGCAAACAATTCT
TP54469_Query	D	0	.	2B	CAGCTTCAACATCAATAATTCAGCAGAAACAATCATTAACAGTTTCCACACCGCAAACAATTCT
TP60504_Hit	D	0	.	2B	CTGCAAAAAACTGCCAAGATATATATACCTCGGTTTGAAAGGACTCGGCAATGAGGGATCTGCG
TP60504_Query	D	0	.	2B	CTGCAAAAAACTGCCAAGATATATATACCTCGGTTTGAAAGGACTCGGCAATGAGGAATCTGCG
TP64248_Hit	D	2	.	2B	CTGCAATGAATGTGGAATTTCCAACACACACGCTCACGCTCGGTCCCAATGAGCCTGAAGCCTG
TP64248_Query	D	2	.	2B	CTGCAATGAATGTGGAATTTCCAACACACACCCTCACGCTCGGTCCCAATGAGCCTGAAGCCTG
TP73640_Hit	D	0	.	2B	CTGCCAACGTCATTGCTAGCCATTATATTTATATCTTGATTCCAATATGTTGCTTTTATCTGCA
TP73640_Query	D	0	.	2B	CTGCCAACGTCATTGCTAACCATTATATTTATATCTTGATTCCAATATGTTGCTTTTATCTGCA
TP78129_Hit	D	0	.	2B	CTGCCTACAAACACAATAATAACAAAAAGATAACTATTTACAAAATAAGATTTGGATATGGGGG
TP78129_Query	D	0	.	2B	CTGCCTACAAACACAATAATAACAAAAAGATAACTATTTACAAAATAAGATTTGGAGATGGGGG
TP8851_Hit	D	0	.	2B	CAGCACAAATGTTATACTGAATTGATGTATTAATTACAACCAGTGTTTAAATTAAGGTTGCTGT
TP8851_Query	D	0	.	2B	CAGCACAAATGTTATACTGAATTGATGTATTAATTACAACCAGTGTGTAAATTAAGGTTGCTGT
TP92440_Hit	D	3	.	2B	CTGCTGTTGGTGAAGATTTGGTTGCTATGACTAATTGTCGCCTTCAGGCTAGAAATTTCATCAC
TP92440_Query	D	3	.	2B	CTGCTGTTGGTGAAGATTTGGTTGCCATGACTAATTGTCGCCTTCAGGCTAGAAATTTCATCAC
TP18358_Hit	D	0	.	2C	CAGCATCAAGCAGAGTTTGAACCCTTGTGATCCAAGTAAGTGTTGAATGACCTGCTTGTAAGAC
TP18358_Query	D	0	.	2C	CAGCATCAAGCAGAGTTTGAACCCTTGTGATCCAAGTAAGTGTTGAATGACCTGCTTGCAAGAC
TP26195_Hit	D+G	0	.	2C	CAGCCCGTTTTGCTAATTCGGGTCAACCCGTTATCACTCCGGTTGAAACACCGTCATTATCGGA
TP26195_Query	D+G	0	.	2C	CAGCCAGTTTTGCTAATTCGGGTCAACCCGTTATCACTCCGGTTGAAACACCGTCATTATCGGA
TP27703_Hit	D	0	.	2C	CAGCCCAAATTCTGAGCACTAGTCCATATGCAGTTGACCAAGTGCTTAATGTGGAAGATGTTAC
TP27703_Query	D	0	.	2C	CAGCCCAAATTCTGAGCACCAGTCCATATGCAGTTGACCAAGTGCTTAATGTGGAAGATGTTAC

Name	Filter	Nb hit (Mt4.0)	Mt Chr (Mt4.0)	Ms Chr (Li et al., 2014)	Sequence
TP3798_Hit	D	0	.	2C	CAGCAACATGTCGCTCTCTTCTACATTATCTTCCAGTTTGTGGTCAACTGTTAGGACATAAACT
TP3798_Query	D	0	.	2C	CAGCAACATGTCGCTCTCTTCTACATTATCTTCCAATTTGTGGTCAACTGTTAGGACATAAACT
TP39396_Hit	D+G	3	.	2C	CAGCGGGAGCATAGTCGTAACCACCATCATCGTCATCATCGCCTTCCATGTATGAGTCTCGATC
TP39396_Query	D+G	3	.	2C	CAGCGGGAGCATAGTCGTAACCACCATCATCGTCATCATCGCCTTCCATGTATGAGCCTCGATC
TP41040_Hit	D+G	0	.	2C	CAGCGTGGCTCTTCCAACCGCTTTGAATTGCCGTGCTACTCTCTTTAGTAGTGTTGCCGTGGCT
TP41040_Query	D+G	0	.	2C	CAGCGTGGCTCTTCCAACCACTTTGAATTGCCGTGCTACTCTCTTTAGTAGTGTTGCCGTGGCT
TP51050_Hit	D	0	.	2C	CAGCTGATTTCTTTGAGGGATTGGTTGCAGACTTTTGCTTCCTAAATGGTTTTGATGCTAGTTT
TP51050_Query	D	0	.	2C	CAGCTGATTTCTTTGAGGGATTGATTGCAGACTTTTGCTTCCTAAATGGTTTTGATGCTAGTTT
TP56346_Hit	D	0	.	2C	CAGCTTCTGCAAATTTGCAGACAGCTCTGCTATACTTTATTTCTCAATCAAAGACTTCGTTTTG
TP56346_Query	D	0	.	2C	CAGCTTCTGCAAATTTGCAGACAGCTCTGCCATACTTTATTTCTCAATCAAAGACTTCGTTTTG
TP60076_Hit	D	2	.	2C	CAGCTTTTGAATTTAGCTTAGCAAAATCACCAATCCTTCCTCTCCCCCATAAAAAAAAGAAGAG
TP60076_Query	D	2	.	2C	CAGCTTTTGAATTTAGCTTAGCAAAATCACCAACCCTTCCTCTCCCCCATAAAAAAAAGAAGAG
TP67383_Hit	D+G	0	.	2C	CTGCACTTTGGAGTCACTTGTGTTGGTTCGATCTGCTTCTGTTCCGATCGGATTCGTCGCTGTT
TP67383_Query	D+G	0	.	2C	CTGCACTTTGGAGTCACTTGTGTTGGTTCGATTTGCTTCTGTTCCGATCGGATTCGTCGCTGTT
TP69738_Hit	D	0	.	2C	CTGCAGTCTAGAGGTTTATCTGTAACAGACGACGCTTTGAACTCTAGGGTTTTAGGTGTTAAGG
TP69738_Query	D	0	.	2C	CTGCAGTCTAGAGGTTTATCTGTAACAGACGACACTTTGAACTCTAGGGTTTTAGGTGTTAAGG
TP73739_Hit	D	0	.	2C	CTGCCAAGCTTGCCTTAACACCTAAAACCCTAGAGTTCAAAGTGTCGTCTGTTACAGATAAACC
TP73739_Query	D	0	.	2C	CTGCCAAGCTTGCCTTAACACCTAAAACCCTAGAGTTCAAAGCGTCGTCTGTTACAGATAAACC
TP82700_Hit	D+G	0	.	2C	CTGCGGTATCTTAACCACTTGAACCGCTGTTTCTGAAGTTCAAAAGCCCTGGCTTGGAAGTCAA
TP82700_Query	D+G	0	.	2C	CTGCGGTATCTTAACCACTTAAACCGCTGTTTCTGAAGTTCAAAAGCCCTGGCTTGGAAGTCAA
TP10517_Hit	D	0	.	2D	CAGCACCCACAATATAAATATACTCATACATGGGCTTTGAAGCGAGCTTACAAAGCTTCTAGGC
TP10517_Query	D	0	.	2D	CAGCACCCACAAGATAAATATACTCATACATGGGCTTTGAAGCGAGCTTACAAAGCTTCTAGGC
TP17767_Hit	D	0	.	2D	CAGCATATAAGATGTTTGAGACCATTAAAAGAAAACCAAACATTGATGCTTATGACACAAGTGG
TP17767_Query	D	0	.	2D	CAGCATATAAGATGTTTGAGACAATTAAAAGAAAACCAAACATTGATGCTTATGACACAAGTGG
TP18468_Hit	D	0	.	2D	CAGCATCACATATTTGTCTTGCTATTTGTTGGGTTGGCTTATGGCTTCATGATGCTTTTGTTCA
TP18468_Query	D	0	.	2D	CAGCATCACATATTTGTCTTGCTATTTGTTGGGTTGGCTTATAGCTTCATGATGCTTTTGTTCA
TP2702_Hit	D	2	.	2D	CAGCAACAAAAGCTTGGGTAGTAGCATCAAGCATAGGAGCCGTGGAGGCCTTAAAAGACCAACT
TP2702_Query	D	2	.	2D	CAGCAACAAAAGCTTGGGTAGTAGCATCAAGCATAGGAGCAGTGGAGGCCTTAAAAGACCAACT
TP50427_Hit	D+G	0	.	2D	CAGCTGAGAAAGCCAAGTCCGCGGCTGTTCAGGCTAAGGATGTTACTGTGGAGACGGGGAAGAC
TP50427_Query	D+G	0	.	2D	CAGCTGAGAAAGCCAAGTCCGCGGCTGTTCAGGCTAAGGATGTTACTGTGGAGACAGGGAAGAC
TP53888_Hit	D	2	.	2D	CAGCTTATAACACATAAGCTATTTCGATACGAAAACATAAAATAAAGTTAAATTGTTTTTGTGT
TP53888_Query	D	2	.	2D	CAGCTTATAACACATAAGCTATTTCGATACAAAAACATAAAATAAAGTTAAATTGTTTTTGTGT
TP12652_Hit	D	2	.	3A	CAGCACTTGTGTTTAGGACTGGTTGCAAGAGAGGCCCTATGACCATTGCTGGGTTAATATTAAC
TP12652_Query	D	2	.	3A	CAGCACTTGTGTTTAGGACTGGTTGCAAGAGAGGCCCTATGACCATTGCTGGGTTAATAGTAAC
TP16198_Hit	D+G	0	.	3A	CAGCAGTGCCTCCAAATTTAATTCTAATTCCTTATATGACATGAGTTGAGGCACCCGTATCAAT
TP16198_Query	D+G	0	.	3A	CAGCAGTGCCTCCAAATTTAATTCTAATTCCTTATATGACATGAGCTGAGGCACCCGTATCAAT
TP25211_Hit	D	0	.	3A	CTGCCACTAGTTTATGCGGGATATGTCTTGTTTTTGTGAAATTAGACTCTGAAATTTTCAAAAA
TP25211_Query	D	0	.	3A	CAGCCACTAGTTTATGCGGGATATGTCTTGTTTTTGTGAAATTAGACTCTGAAATTTTCAAAAA
TP29972_Hit	D	0	.	3A	CAGCCGAATGCGTTCTTACATGCCTGCAAGCGTTAACACTTTCTTACCTCCTAAGTACAAGCCC
TP29972_Query	D	0	.	3A	CAGCCGAATACGTTCTTACATGCCTGCAAGCGTTAACACTTTCTTACCTCCTAAGTACAAGCCC
TP39565_Hit	D+G	0	.	3A	CAGCGGGTCCCTTTTACTCTCATCAAGCCATGTTTTTAGACAAATGGCAAAGAATCTAATGGTT
TP39565_Query	D+G	0	.	3A	CAGCGGGTCCCTTTTACTCTCATCAAGCCATGTTTTTAGACAAATGGCAAACAATCTAATGGTT
TP48028_Hit	D	0	.	3A	CAGCTCGAACAGCTTTGGGATACAAACAGAATAAACACAACATAAGATTTTCTATAGTAGGCTC
TP48028_Query	D	0	.	3A	CAGCTCGAACAGCTTTGGGATACAAACAGAATAAACACAACATAAGATTTTATATAGTAGGCTC
TP60391_Hit	D	0	.	3A	CAGCTTTTTGCTAAGAACAACAATGATACTAAGGAAACACTTGTGACATTGTGGTGGGAATGTC
TP60391_Query	D	0	.	3A	CAGCTTTTTGCTAAGAACAACAATGATACTAAGGAAACACTTCTGACATTGTGGTGGGAATGTC
TP68_Hit	D	0	.	3A	CAGCAAAAAAGTACAAGCTTGGAGTTGGGACAACTGTCGTTGGCAGAGGAGTATAGTTGTATCA
TP68_Query	D	0	.	3A	CAGCAAAAAAGTACAAGCTTGGAGTTGGGACAACTGTCGTTGGCAGAGGAGTATAGTTATATCA
TP75491_Hit	D+G	0	.	3A	CTGCCATGTATTTCGTGTCTAAGAGATTGAAGAAGAAACACAATATCACTGATGAGCGTGAGGC
TP75491_Query	D+G	0	.	3A	CTGCCATGTATTTCGTGTCTAAGAGATTGAAGAAGAAACACAATATCACTGATGAGCGTCAGGC
TP79920_Hit	D	0	.	3A	CTGCGAATTTGGCGGAGTTTCATTCCAAAATTCCGATCAAAGATGCAGTGATTGCTGTTCCGCC
TP79920_Query	D	0	.	3A	CTGCGAATTTGGCGGAGTTTCATTCCAAAATTCCGATCAAAGACGCAGTGATTGCTGTTCCGCC
TP8437_Hit	D	2	.	3A	CAGCAATTGCTCGAAGATAGCCTGCACGAACAGAACCAGAAGCAGTGGACCAATCTCTACCTTT
TP8437_Query	D	2	.	3A	CAGCAATTGCTCGAAGATAGCCAGCACGAACAGAACCAGAAGCAGTGGACCAATCTCTACCTTT

Name	Filter	Nb hit (Mt4.0)	Mt Chr (Mt4.0)	Ms Chr (Li et al., 2014)	Sequence
TP85596_Hit	D+G	2	.	3A	CTGCTAGCAGGTGCTGTACAGATTTTTGGATTACTACAGCAAATTTGATTGGAAGAAGAATTAT
TP85596_Query	D+G	2	.	3A	CTGCTAGCAGGTGCTGTACAGATTTTTGGATTACTACAGCAAATTTGACTGGAAGAAGAATTAT
TP91088_Hit	D	0	.	3A	CTGCTGGTGCTGGTGCTGGTGCTGAAGGTCCACGGTTCCTCAGATGAGGAGGAACGTAAACAGG
TP91088_Query	D	0	.	3A	CTGCTGGTGCAGGTGCTGGTGCTGAAGGTCCACGGTTCCTCAGATGAGGAGGAACGTAAACAGG
TP93544_Hit	D+G	0	.	3A	CTGCTTCCTATGGGAGTATTGATATCCTAAAGCTTATACTCTCGTATCCTGAGGCTGATGTTAA
TP93544_Query	D+G	0	.	3A	CTGCTTCCTATGGGAGTATTGATATCCTAAAGCTTATACTCTCATATCCTGAGGCTGATGTTAA
TP95131_Hit	D+G	0	.	3A	CTGCTTTGAATTTGACTTTTCCCAATTCATTCCATTATTCCGGTTTTGCATGATTTGGCTTTAA
TP95131_Query	D+G	0	.	3A	CTGCTTTGAATTTGACTTTTCCCAATTCATTCCATTATTCCGGTTTCGCATGATTTGGCTTTAA
TP16348_Hit	D	3	.	3B	CAGCAGTGTATACCATATATGACTTTTTCTCTTTCAGATGACGACAGGTATCTAACACAAATCT
TP16348_Query	D	3	.	3B	CAGCAGTGTATACCATATATGACTTTTTCTCTTTCAGATGACGACAGGTATCTAACACAAAACT
TP22860_Hit	D	2	.	3B	CAGCCAAACACAGATTACTCTCCTTCTGAGCCATTATCTCAAATAACTTCTTTCCTGTTGGATT
TP22860_Query	D	2	.	3B	CAGCCAAACACAGATTACTCTCCTTCTGAGCCATTATCTCAAATAACTTCTTTCCTGTAGGATT
TP27279_Hit	D	0	.	3B	CAGCCGTTAGTTGTTGCTGTGTCATCTGTTAAGCTTGTTGATCCTGCATGAAGTATCCTCCCAG
TP27279_Query	D	0	.	3B	CAGCCATTAGTTGTTGCTGTGTCATCTGTTAAGCTTGTTGATCCTGCATGAAGTATCCTCCCAG
TP3429_Hit	D	2	.	3B	CAGCAACAGATTAAATTCTGTGAGAAACAACTTTTGCCTTTGTATACTCAGATAGCTATCAAGT
TP3429_Query	D	2	.	3B	CAGCAACAGATTAAATTCCGTGAGAAACAACTTTTGCCTTTGTATACTCAGATAGCTATCAAGT
TP3549_Hit	D+G	2	.	3B	CAGCAACAGGTGGCTGGATTAATGATGCTTTTGGACGTAAGAAGGCCACACTTAGTGCTGATGT
TP3549_Query	D+G	2	.	3B	CAGCAACAGGTGACTGGATTAATGATGCTTTTGGACGTAAGAAGGCCACACTTAGTGCTGATGT
TP36050_Hit	D+G	0	.	3B	CAGCGACTTTGTCCTCTTTCTTTGTTTCAGTTTTATTGGGGAGGACCTCAGTAGCACCATCCTT
TP36050_Query	D+G	0	.	3B	CAGCGACTTTGTCCTCTTTCTTTGTTTCAGTTTTATTGAGGAGGACCTCAGTAGCACCATCCTT
TP40628_Hit	D	0	.	3B	CAGCGTCGTTAAATGGTAAACTATATAGTGAAATATGAGAGAGACTTCAACGTTATGAACTAGT
TP40628_Query	D	0	.	3B	CAGCGTCGTTAAATGGTAAACTATATAGTGAAATATGAGAGAGAATTCAACGTTATGAACTAGT
TP50114_Hit	D	0	.	3B	CAGCTGAAGTAGATACTCACATTATTGAAAGGATGTCGATAATTTGAATACGCTCGAATGAGGA
TP50114_Query	D	0	.	3B	CAGCTGAAGTAGATACTCACATTATTAAAAGGATGTCGATAATTTGAATACGCTCGAATGAGGA
TP507_Hit	D	0	.	3B	CAGCAGAACTGATCTCAAGTGTTATAGGCTGAATTTTCAATATCGAGGTTGTGATTTTGCTCGG
TP507_Query	D	0	.	3B	CAGCAAAACTGATCTCAAGTGTTATAGGCTGAATTTTCAATATCGAGGTTGTGATTTTGCTCGG
TP51774_Hit	D	0	.	3B	CAGCTGGTCAATTCTGGCCAATTGGAGTTCATGTATTCATTCATCTGACTTTCCATTATTACAT
TP51774_Query	D	0	.	3B	CAGCTGGTCAATTCTGGCCAATTGGAGTTCATGTATTCATCCATCTGACTTTCCATTATTACAT
TP59191_Hit	D	2	.	3B	CAGCTTTCTCCTCTTTTTGTTTCCTGTTTAAAGCATGACAAATTATAAATTTATCAAATCGTAT
TP59191_Query	D	2	.	3B	CAGCTTTCTCCTCTTTTTGTTTCCTGTTTAAAGCATGACAAATTATAAATTTATCAAATCATAT
TP65153_Hit	D	0	.	3B	CTGCACAAGCACTTGGTCAATCCAAATATTCCTCCAAGGTTTCTCTCCGCCCCGAGCAAAATCA
TP65153_Query	D	0	.	3B	CTGCACAAGCACTTGGTCAATCCAAACATTCCTCCAAGGTTTCTCTCCGCCCCGAGCAAAATCA
TP77832_Hit	D+G	0	.	3B	CTGCCGTAATGGTGATGGCAGGTGTGGACGAATAAAAGGTCCAGCTGTAACGGGCGAGTTCACC
TP77832_Query	D+G	0	.	3B	CTGCCGTAATGGTGATGGCAGGTGTCGACGAATAAAAGGTCCAGCTGTAACGGGCGAGTTCACC
TP8386_Hit	D	0	.	3B	CAGCAATTGAGAGGGTTTACTTGGACTTAAGTTTTTGTAAGAGCATACAAAAAAGTTATGATAT
TP8386_Query	D	0	.	3B	CAGCAATTGAGAGGGTTTACTTGGACTAAAGTTTTTGTAAGAGCATACAAAAAAGTTATGATAT
TP84182_Hit	D+G	2	.	3B	CTGCTAAAGCAAAAACAAGAGGAGGATCCCCGTCTTTGTCCAATACATCAACATTTGCCTCTGG
TP84182_Query	D+G	2	.	3B	CTGCTAAAGCAAAAACAAGAGGAGGATCCCCGTCTTTGTCCAAGACATCAACATTTGCCTCTGG
TP86792_Hit	D	2	.	3B	CTGCTATTGGTTCTAAATATTGGCAACTTGTTTGAGTACCATAAAGATTGATGTTCCTGTTGCA
TP86792_Query	D	2	.	3B	CTGCTATTGGTTCTAAATATTGGCAACTTGTTTGAGTAACATAAAGATTGATGTTCCTGTTGCA
TP8984_Hit	D+G	4	.	3B	CAGCACAAGAGGATGATGATGATGCTGTACCAGATCTTGTCCCCGGGGAGACCTTTGAAACTGC
TP8984_Query	D+G	4	.	3B	CAGCACAAGAGGACGATGATGATGCTGTACCAGATCTTGTCCCCGGGGAGACCTTTGAAACTGC
TP91010_Hit	D	0	.	3B	CTGCTGGTCATGTTTAAGCTCTTGAACACCAATTGTAACAATTCCAATACTTAGACTCAATTTG
TP91010_Query	D	0	.	3B	CTGCTGGTCATGTTTAAGCACTTGAACACCAATTGTAACAATTCCAATACTTAGACTCAATTTG
TP9278_Hit	D	0	.	3B	CAGCACACATCCAGGTGAACTCGCCCGTTACAGCTGGACCTTTTATTCGTCGACACCTGCCATC
TP9278_Query	D	0	.	3B	CAGCACACATCCAGGTGAACTCGCCCGTTACAGCTGGACCTTTTATTCGTCCACACCTGCCATC
TP94620_Hit	D	2	.	3B	CTGCTTGTGTGTCATTATAGTTGGGAAGATAAGGTAATGAAGGAGTTGAAAGTGGTGTATAACT
TP94620_Query	D	2	.	3B	CTGCTTGTGTGTCATTATAGTTGGGAAGAAAAGGTAATGAAGGAGTTGAAAGTGGTGTATAACT
TP13962_Hit	D	2	.	3C	CAGCAGAGTTAGGGATTCCATTAACTCATCGCGGTATTGCAAATAGTGTGAGATTCCTCACCGG
TP13962_Query	D	2	.	3C	CAGCAGAGTTAGGGATTCCATTAACTCACCGCGGTATTGCAAATAGTGTGAGATTCCTCACCGG
TP21893_Hit	D	0	.	3C	CAGCATTGTCGTTGTTTTGATACGGTGCCACAGAGACTCTGTTCCAAAATTGCATCCCATATTT
TP21893_Query	D	0	.	3C	CAGCATTGTCGTTGTTTTGATACGGTGCCACAGAGACTCTATTCCAAAATTGCATCCCATATTT
TP25046_Hit	D+G	0	.	3C	CAGCCACGATTCCAGAAGACAACAGAGGGTACGGATATCAACCTAACCGAAGCAGATTCGTTTG
TP25046_Query	D+G	0	.	3C	CAGCCACGATTCCAGAAGACAACAGAGGGTACGGATATCAACCTAACCGAAGCAAATTCGTTTG

Name	Filter	Nb hit (Mt4.0)	Mt Chr (Mt4.0)	Ms Chr (Li et al., 2014)	Sequence
TP39938_Hit	D	0	.	3C	CAGCGGTGGTGGTGGTGACCTTGGTAGCCTCATCTCCAGGGACACCTTCAACAATATGCTCAAG
TP39938_Query	D	0	.	3C	CAGCGGTGGTGGTGGTGACCTTGGTAGCCTAATCTCCAGGGACACCTTCAACAATATGCTCAAG
TP45295_Hit	D+G	0	.	3C	CAGCTATGTTGGGTTGATATCCCTTTCACCACAAACGAATTTGCTTCGGTTAGGTTGATATCCG
TP45295_Query	D+G	0	.	3C	CAGCTATGTTGGGTTGATATCCCTTTCACCACAAACGAATCTGCTTCGGTTAGGTTGATATCCG
TP4666_Hit	D+G	4	.	3C	CAGCAACTCGATTATCAGATAAAACACCCTTATACACAGTTCCTCCGGCACCTCTTCCGATCTC
TP4666_Query	D+G	4	.	3C	CAGCAACTCGATTATCAGATAAAACACCCTTATACACAGTTCCTCCGGCACCTCGTCCGATCTC
TP53763_Hit	D	0	.	3C	CAGCTTAGCAAACTATCCGACACAGAGAATAATTAACAGGTTTCAAACCTGATATTAGTAATAA
TP53763_Query	D	0	.	3C	CAGCTTAGCAAACTATCCGACACAGAGAATAATTAACAGGTTTCAAACCTGATATTAGTAACAA
TP53851_Hit	D	0	.	3C	CAGCTTAGTTCATCGTACTTTAAACCGTAATTAGTCCACTTCATGACTTTTAAGTTCCTCAAAA
TP53851_Query	D	0	.	3C	CAGCTTAGTTCATCATACTTTAAACCGTAATTAGTCCACTTCATGACTTTTAAGTTCCTCAAAA
TP56938_Hit	D	0	.	3C	CAGCTTGAATTTTTGCTTTGATCAAATTATATCAATTATAGTCACACGACTCATCTATCAAAGT
TP56938_Query	D	0	.	3C	CAGCTTGAATTTTTGCCTTGATCAAATTATATCAATTATAGTCACACGACTCATCTATCAAAGT
TP7178_Hit	D	3	.	3C	CAGCAATATGCAAAGGAGTTTCAACAAATGGTATTAAATCTGTCATCTCCAAAATGAATGGATC
TP7178_Query	D	3	.	3C	CAGCAATATGCAAAGGAGTTTCAACAAATGGTATTAAATCTATCATCTCCAAAATGAATGGATC
TP74291_Hit	D+G	2	.	3C	CTGCCACCCCACCCTCCACCACCACTCTCCTGGGTGCACTCCCTGGCCATGTGACCTGACTCAC
TP74291_Query	D+G	2	.	3C	CTGCCACCCCACCCTCCACCACCACTCTCCTGGGTGCACTCCCTAGCCATGTGACCTGACTCAC
TP82327_Hit	D	0	.	3C	CTGCGGCTACAGTGTTGTTGATTGGTGACCAGTCTGACTGCAGTAAATGCAGTGTTATTTATAG
TP82327_Query	D	0	.	3C	CTGCGGCTACAGTGTTGTTGATTGGTGACCAGTCTGACTGCAGTAAATGCAGTGCTATTTATAG
TP86889_Hit	D	0	.	3C	CTGCTATTTGACAATATTTTGTATTGAATAGCATATCTCAGAATAATAGTGGTTTGTTCAAATA
TP86889_Query	D	0	.	3C	CTGCTATTTGACAATATTTTGTACTGAATAGCATATCTCAGAATAATAGTGGTTTGTTCAAATA
TP20332_Hit	D	2	.	3D	CAGCATGGAGCTAAGGGAAATTATGGGTGGGTTTGCAAGTGATACCTCACTTGGTTCTGTCTTG
TP20332_Query	D	2	.	3D	CAGCATGGAGCTAAGGGAAATTATGGGTGGGTTTGCAAGTGATACCTCACTTGGTTCAGTCTTG
TP27681_Hit	D	0	.	3D	CAGCCCAAAGAATCACCCAGAATTCAAAAGTGAAACACGATGAATTCAAAATGCCACATGCTAC
TP27681_Query	D	0	.	3D	CAGCCCAAAGAATCACCCAGAATTCAAAAGTGAAACACGATGAATTCAAAATGCAACATGCTAC
TP44586_Hit	D	0	.	3D	CAGCTATACCATATTGTTAAGGAACTGAAGCGTCAACTACTTATATCAAAGGCAAAACATAGAT
TP44586_Query	D	0	.	3D	CAGCTATACCATATTGTTAAGGAACTGAAGCATCAACTACTTATATCAAAGGCAAAACATAGAT
TP48819_Hit	D+G	0	.	3D	CAGCTCTCAATATTAGCCATAACATCACCGCCACCTACACTCTCATTCAACCATTCACTTCACG
TP48819_Query	D+G	0	.	3D	CAGCTCTCAATATTAACCATAACATCACCGCCACCTACACTCTCATTCAACCATTCACTTCACG
TP67100_Hit	D	0	.	3D	CTGCACTTACTTTCGAATTTATGCAGGGAATAGGCTTTTCTTCTCCCAGTCTAGATATAAAGGA
TP67100_Query	D	0	.	3D	CTGCACTTACTTTCAAATTTATGCAGGGAATAGGCTTTTCTTCTCCCAGTCTAGATATAAAGGA
TP7062_Hit	D	0	.	3D	CAGCAATAGTGGCAGATATAAGATAACAAAAATAGGGTGTGTGGAAGTGGAATTGGAGAAGACG
TP7062_Query	D	0	.	3D	CAGCAATAGTGGCAGATAAAAGATAACAAAAATAGGGTGTGTGGAAGTGGAATTGGAGAAGACG
TP72936_Hit	D	0	.	3D	CTGCATTTCCTTCAACAGTTTTCCGTCCCACAAGTAAATTCGTCTTCTCCAATTCCACTTCCAC
TP72936_Query	D	0	.	3D	CTGCATTTCCTTCAACAGTTTTCCATCCCACAAGTAAATTCGTCTTCTCCAATTCCACTTCCAC
TP27675_Hit	D	3	.	4A	CAGCCCAAAGAAAAGCACCGAGAGCAATGACATGTGCACGATTGTGTCGTGTGGCAAGGTAAGC
TP27675_Query	D	3	.	4A	CAGCCCAAAGAAAAGCACCAAGAGCAATGACATGTGCACGATTGTGTCGTGTGGCAAGGTAAGC
TP31775_Hit	D+G	0	.	4A	CAGCCTAATAACCTCCACTGCTCGGTATCTTTCACCCTTGCAGTTTGACACCCTCCACTGTCAC
TP31775_Query	D+G	0	.	4A	CAGCCTAATAACCTCCACTGCTCGGCATCTTTCACCCTTGCAGTTTGACACCCTCCACTGTCAC
TP32434_Hit	D+G	0	.	4A	CAGCCTCCGCCACCTTCGCCTTATCCATAGGCTTATTATGACCTGCAGATCCTTTCCCTAAACT
TP32434_Query	D+G	0	.	4A	CAGCCTCAGCCACCTTCGCCTTATCCATAGGCTTATTATGACCTGCAGATCCTTTCCCTAAACT
TP40731_Hit	D	0	.	4A	CAGCGTTTTTGAGGATGATGGAAACGAGCTGAGAAGCGAATAAGTTGAGATGATCGATGGATTT
TP40731_Query	D	0	.	4A	CAGCGTCTTTGAGGATGATGGAAACGAGCTGAGAAGCGAATAAGTTGAGATGATCGATGGATTT
TP43364_Hit	D	0	.	4A	CAGCTACCTTGTATTTTCTGCCTCATAATACAACTCATTCCCAGCTGTGTGCATCAAAATGTCC
TP43364_Query	D	0	.	4A	CAGCTACCTTCTATTTTCTGCCTCATAATACAACTCATTCCCAGCTGTGTGCATCAAAATGTCC
TP44853_Hit	D	3	.	4A	CAGCTATCAATGTGTTCTCAACACCACCTCCAAAAACCTCCCCATCAGGTTTAGCCAATGACAC
TP44853_Query	D	3	.	4A	CAGCTATCAATGTGTTCTCAACACCACCTCCAAAAACCTCCCCATCAGGTTTAGCCAATGAAAC
TP49280_Hit	D	0	.	4A	CAGCTCTTAATGCTTTTGAGTATGCCTCTATTCTCCCATCTAAGAGTAGCACCTTAAACACCCC
TP49280_Query	D	0	.	4A	CAGCTCTTAATGCTTTTGAGTATGCCTCTATTCTCCCATCTAAGACTAGCACCTTAAACACCCC
TP55566_Hit	D	0	.	4A	CAGCTTCGCCAAGCCAGAGAAGTGCAAGAGCAAAAGATCTATGAAGCTGTGGCAAATAACTCAC
TP55566_Query	D	0	.	4A	CAGCTTCGCCAAGCCAGAGAAGTGCAAGAGCAAAAGATCCATGAAGCTGTGGCAAATAACTCAC
TP62392_Hit	D	0	.	4A	CTGCAACCCAAATGAATTCTCACCTTTCCATTGAATTGTATGAGCTCTTCCTGGGCAAGATTAA
TP62392_Query	D	0	.	4A	CTGCAACCCAAATGAATTCTCACCTTTCCATTGAATTGTATGAGCTCTTCCTGGGCAAGATCAA
TP66480_Hit	D	0	.	4A	CTGCGCGCATAAGTGGATGTGCAAAGTAAGCACCTAATGTTAAAGCAGTGATTGTAAAATATGG
TP66480_Query	D	0	.	4A	CTGCACGCATAAGTGGATGTGCAAAGTAAGCACCTAATGTTAAAGCAGTGATTGTAAAATATGG

Name	Filter	Nb hit (Mt4.0)	Mt Chr (Mt4.0)	Ms Chr (Li et al., 2014)	Sequence
TP73993_Hit	D	2	.	4A	CTGCCAATTTTATGACTTGAACAAATATCATACGGAACATTGTAGCATTTTAAGCTTTTTGTCT
TP73993_Query	D	2	.	4A	CTGCCAATTTTATGACTTGAACAAATATCATACAGAACATTGTAGCATTTTAAGCTTTTTGTCT
TP77035_Hit	D+G	0	.	4A	CTGCCGAATGTGACAGGGTCGAATTTCCTTAGCCTTTCAGCTTCTTGTGGATTGATCACGCACT
TP77035_Query	D+G	0	.	4A	CTGCCGAATGTGACAGGGTCGAATTTCCTTAGCCTTTCAGCTTCTTGTGGATCGATCACGCACT
TP90678_Hit	D+G	0	.	4A	CTGCTGGCGCACTTGTGACCACCTTTCCATTGTCGTCACTCGTGATCCTCACCATTGCCACCAG
TP90678_Query	D+G	0	.	4A	CTGCTGGCGCACTTGTGACCACCTTTCCATTGTCGTCACTCGTGACCCTCACCATTGCCACCAG
TP11171_Hit	D+G	2	.	4B	CAGCACGACTTTCGAAGGAAAAAGCATGGGACCAAGTCGTTTTCAACGAGGAACTCTTTCATCC
TP11171_Query	D+G	2	.	4B	CAGCACGACTTTCCAAGGAAAAAGCATGGGACCAAGTCGTTTTCAACGAGGAACTCTTTCATCC
TP11236_Hit	D	0	.	4B	CAGCACGATCAATATTGATATGAACGCATCCTGGCAGAGGCAGGTTCCACTTGATACTCATACA
TP11236_Query	D	0	.	4B	CAGCACGATCAATATCGATATGAACGCATCCTGGCAGAGGCAGGTTCCACTTGATACTCATACA
TP1496_Hit	D+G	0	.	4B	CAGCAAACTGTAATGCATTATGAAGGAGAGAACAAAAAACACATGTTTACAACTATGTGGAGAC
TP1496_Query	D+G	0	.	4B	CAGCAAACTGTAATGCATTATGAAGGAGAGAACAAAAAACACATGTTTACAACTATGCGGAGAC
TP23595_Hit	D	0	.	4B	CAGCCAAGACTGCGTAATGGTTCTCGCTCACAGTCTATTCTGTAGTTTCTTGCACACGTTGCCA
TP23595_Query	D	0	.	4B	CAGCCAAGACTGCGTAATGGTTCTCGCTCACAGTCTATTCTGTAGTTTCTTGCACACCTTGCCA
TP3029_Hit	D	0	.	4B	CAGCAACAAGAAAACTTATGGAATTGAAACACGTATCCTAGCAGATGTGCAAAACAGCAACTCA
TP3029_Query	D	0	.	4B	CAGCAACAAGAAAACTTATGGAATTGAAACACGTATCCTAACAGATGTGCAAAACAGCAACTCA
TP3143_Hit	D	0	.	4B	CAGCAACAATCATGTCTATGGGTAAACCAAGAAAAAATCATGTTTGTCAGAGAGTAAGCGTAAA
TP3143_Query	D	0	.	4B	CAGCAACAATCATGTCTATGGGTAAACCAAGAAAAAATCATGTTCGTCAGAGAGTAAGCGTAAA
TP4272_Hit	D	0	.	4B	CAGCAACCTTTTTTTGTGCATACAAGTACAATTCAGTGACCGCTTTTTTAAATGCATATGCAAC
TP4272_Query	D	0	.	4B	CAGCAACCTTTTTATGTGCATACAAGTACAATTCAGTGACCGCTTTTTTAAATGCATATGCAAC
TP44966_Hit	D	0	.	4B	CAGCTATCTCATGGAGAATTTAGAAGATGAGGAATAGGAAGTTGTGGCAAGGTGTGCAAGAAAC
TP44966_Query	D	0	.	4B	CAGCTATCTCATGGAGAATTTAGAAGATGAGGAATAGGAAGTTGTGGCAACGTGTGCAAGAAAC
TP45026_Hit	D	0	.	4B	CAGCTATGAATGTGAAAACAGGACTCATGTGGCCACCAGTTAGAGGAACTGTCACAATTAGGAA
TP45026_Query	D	0	.	4B	CAGCTATGAATATGAAAACAGGACTCATGTGGCCACCAGTTAGAGGAACTGTCACAATTAGGAA
TP60347_Hit	D+G	2	.	4B	CAGCTTTTTCCTCCTCACCGAGAACTTTCCCAGAGCCACTGCTAAATAGCCTTCTAGATGCAAA
TP60347_Query	D+G	2	.	4B	CAGCTTTTTCCTCCTCACCAAGAACTTTCCCAGAGCCACTGCTAAATAGCCTTCTAGATGCAAA
TP61499_Hit	D	0	.	4B	CTGCAAATACCAAGAGTCTTGCTTTTGATGGCGTCTCCATTACACTCTACAACATAGCACTTGA
TP61499_Query	D	0	.	4B	CTGCAAATACCAAGAGTCTTGCTTTCGATGGCGTCTCCATTACACTCTACAACATAGCACTTGA
TP73809_Hit	D+G	0	.	4B	CTGCCAATAACGGTACTGTAGATTCACCTCAGAAAGAAGCTCCTGCCACTGAGGAGCAGTCTGC
TP73809_Query	D+G	0	.	4B	CTGCCAATAACGGTACTGTAGATTCACCTCAGAAAGAAGCTCCTGCCACCGAGGAGCAGTCTGC
TP17331_Hit	D	0	.	4C	CAGCATACAAGACAAGCGCTACCACTAATTTTTTATGAATCACCGTGGAAATGTTCTCGGTCGT
TP17331_Query	D	0	.	4C	CAGCATACAAGACAAGCGCTACCACTAATTTTTTATGAATCACCATGGAAATGTTCTCGGTCGT
TP20792_Hit	D	0	.	4C	CAGCATGTTATGATAAAGTACTCACATCACATTAATACGTAGTTATCGGTAATAATTTAAGCAA
TP20792_Query	D	0	.	4C	CAGCATGTTATGATAAAGTACTCACATCACATTAATACGTAGTTATCGGCAATAATTTAAGCAA
TP34849_Hit	D	2	.	4C	CAGCCTTTATATCAAGTTTGTTATCTGAGAGTGGCACAAAATAGTTAATAACCAATGTTATGCC
TP34849_Query	D	2	.	4C	CAGCCTTTATATCAAGTTTGTTATCTGAGAGTGGCACAAAATAGTTAATAACCAATGTTATACC
TP38425_Hit	D	0	.	4C	CAGCGGAAGCCTTGGCGATGCAAGTGACCGAGGAGAACTACAAAAAGGGCTTGACTTATCCACC
TP38425_Query	D	0	.	4C	CAGCGGAAGCCTTGGCGAAGCAAGTGACCGAGGAGAACTACAAAAAGGGCTTGACTTATCCACC
TP40585_Hit	D	0	.	4C	CAGCGTCGCAAGCTGAATTTTTTGCTATGACTTTATGGAGCATTTGGAAGAGTAAAAATCTTAA
TP40585_Query	D	0	.	4C	CAGCGTCGCAAGCTGAATTTTTTGCTACGACTTTATGGAGCATTTGGAAGAGTAAAAATCTTAA
TP55701_Hit	D	0	.	4C	CAGCTTCTATAGATGTCTGTCAATCTCAATGGATTAGCATGTTTTTTTCTAGAAATGAATCTGA
TP55701_Query	D	0	.	4C	CAGCTTCTAAAGATGTCTGTCAATCTCAATGGATTAGCATGTTTTTTTCTAGAAATGAATCTGA
TP85755_Hit	D+G	0	.	4C	CTGCTAGGGGTTCTCCAGGTATCTGTTTTCAAAGACAGTAGAGGTACGTTTATTTGTTGTTTTG
TP85755_Query	D+G	0	.	4C	CTGCTAGGGGTTCTCCACGTATCTGTTTTCAAAGACAGTAGAGGTACGTTTATTTGTTGTTTTG
TP11344_Hit	D	0	.	4D	CAGCACGCCAACCATCAATCAAGTTTCTTGCTTGCACTGCCACCTGCATCGCAATTTCAAGAAT
TP11344_Query	D	0	.	4D	CAGCACGCCAACCATCAAGCAAGTTTCTTGCTTGCACTGCCACCTGCATCGCAATTTCAAGAAT
TP16845_Hit	D	0	.	4D	CAGCGGTTTGGCGAGCTTTGCTATCTGCTTGTGCATACCATGGTGCGGTTGATAAAGCTCAGGT
TP16845_Query	D	0	.	4D	CAGCAGTTTGGCGAGCTTTGCTATCTGCTTGTGCATACCATGGTGCGGTTGATAAAGCTCAGGT
TP22071_Hit	D+G	0	.	4D	CAGCATTTCAAAATGTTCTTGTAATCGGAACTGATGCTCTTTCGAGATATATCGATTGGACCGA
TP22071_Query	D+G	0	.	4D	CAGCATTTCAAAATGTTCTTGTAATCGGAACTGATGCTCTTTCAAGATATATCGATTGGACCGA
TP25789_Hit	D+G	0	.	4D	CAGCCAGTATCACCTCTTAGAGCTTCTGCTATGGAAGTAGACTCTAGTGTTGCTGAGCTGGTCA
TP25789_Query	D+G	0	.	4D	CAGCCAGCATCACCTCTTAGAGCTTCTGCTATGGAAGTAGACTCTAGTGTTGCTGAGCTGGTCA
TP30031_Hit	D	0	.	4D	CAGCTGACATTGCCATACGACCAACATCGGTTGTGAGAAGTTTGAGTTCAGCTAGGATTCGAGC
TP30031_Query	D	0	.	4D	CAGCCGACATTGCCATACGACCAACATCGGTTGTGAGAAGTTTGAGTTCAGCTAGGATTCGAGC

Name	Filter	Nb hit (Mt4.0)	Mt Chr (Mt4.0)	Ms Chr (Li et al., 2014)	Sequence
TP30188_Hit	D	0	.	4D	CAGCCGATAACATATTAGCAACAATAACAAAAGCAGAATCATCATGAGGTTCTAGTTCCAATAC
TP30188_Query	D	0	.	4D	CAGCCGATAACATATTAGCAACAATAACAAAAGCAAAATCATCATGAGGTTCTAGTTCCAATAC
TP36543_Hit	D	0	.	4D	CAGCGATCAGAACCCTCCTCTCATTTCAGTAGTTGTTTTTGCCCTGAAAAACCTACAAAATACC
TP36543_Query	D	0	.	4D	CAGCGATCAGAACCCTCCTCTCATTTCAGTAGTTGTTTTTGCCCTAAAAAACCTACAAAATACC
TP48792_Hit	D	2	.	4D	CAGCTCTATTTTGCTTACGTAGTTTAACATACTTTGAATGAATTTAATTCTGTTTCATTTTGAT
TP48792_Query	D	2	.	4D	CAGCTCTATTTTGCTTACGCAGTTTAACATACTTTGAATGAATTTAATTCTGTTTCATTTTGAT
TP50276_Hit	D+G	0	.	4D	CTGCTGAATTTGTTTTGGCACAATTGGGAAACAGCGCGTATAACTGAAGCAGATGACAGTTGTG
TP50276_Query	D+G	0	.	4D	CAGCTGAATTTGTTTTGGCACAATTGGGAAACAGCGCGTATAACTGAAGCAGATGACAGTTGTG
TP56974_Hit	D+G	0	.	4D	CAGCTTGACATTGGACATTGAATATCTTCTCAAAACAAAACCATTTTTTTTTCAAAAGAAGTAA
TP56974_Query	D+G	0	.	4D	CAGCTTGACATTGGACAATGAATATCTTCTCAAAACAAAACCATTTTTTTTTCAAAAGAAGTAA
TP62843_Hit	D+G	0	.	4D	CTGCAACTGTTGTTGAAATTGTGGAAAAAAGCAATTGTTGTGAAGATTTGAGAGAGGTTCTTTG
TP62843_Query	D+G	0	.	4D	CTGCAACTGTTATTGAAATTGTGGAAAAAAGCAATTGTTGTGAAGATTTGAGAGAGGTTCTTTG
TP73064_Hit	D	3	.	4D	CTGCATTTGTAGGGTAATATGTGTTTGACTGTATTGGTAACCTCATTTGTAACATGAGCTAGTA
TP73064_Query	D	3	.	4D	CTGCATTTGTAGGGTAATATGCGTTTGACTGTATTGGTAACCTCATTTGTAACATGAGCTAGTA
TP74770_Hit	D	5	.	4D	CTGCCAGCCACATGATGTACACATGTGGGTTTCAAAGGCATTTGTAGATTCTGCTCTCCTAAGA
TP74770_Query	D	5	.	4D	CTGCCAGCCACATAATGTACACATGTGGGTTTCAAAGGCATTTGTAGATTCTGCTCTCCTAAGA
TP7755_Hit	D	0	.	4D	CAGCAATGCAGGAACTCCATCAACTGGTTCACCGGGTTCAACTCCGGGCACCGGAACCGGAACT
TP7755_Query	D	0	.	4D	CAGCAATGCAGGAACTCCATCAACTGGTTCACCGGGTTCAACTCCGGGCACCGGAACCGGAACC
TP80625_Hit	D	0	.	4D	CTGCGATGGGCAGGCCAGTTTTGAGGCCTATGTCTGATGCGTGGATGTTACATGGTGACTCTCA
TP80625_Query	D	0	.	4D	CTGCGATGGGCAGGCCAGTTTTGAGGCCTATGTCTGATGCGTGGATGTTACATGATGACTCTCA
TP86023_Hit	D	0	.	4D	CTGCTATACAGTGGTGGAAATTCTGATTTCTTGGACATTTTTCAAACGCGTAAGAAAAGTTAAA
TP86023_Query	D	0	.	4D	CTGCTATACAGTGGTGGAAATTCTGATTTCTTGGACATTTTTCAAACGCGCAAGAAAAGTTAAA
TP90977_Hit	D	0	.	4D	CTGCTGGTAGTGATAATAGGGGTCAGCTTCTGTTGTTTGTGGTAGCACCAGCTATTCTTTGGGT
TP90977_Query	D	0	.	4D	CTGCTGGTAGTGATAATAGGGGTCAGCTTCTCTTGTTTGTGGTAGCACCAGCTATTCTTTGGGT
TP92917_Hit	D+G	0	.	4D	CTGCTTAGCAAGGTTTAATGGATTGCTTTTCTCATCTTTGAAATGTCTCTGTTTTGTGAATATA
TP92917_Query	D+G	0	.	4D	CTGCTTAGCAAGGTTTAATGGATTGCTTTTCTCATCTTTAAAATGTCTCTGTTTTGTGAATATA
TP17532_Hit	D	2	.	5A	CAGCATACTTCAACTTCTTTACCTGGGTCAGAAGAGAGGAATTTCCCTCGCATGCCTCAATCAA
TP17532_Query	D	2	.	5A	CAGCATACTTCAACTTCTTTACCTGGGTCAGAAGAGAGGAATTTCCCTCACATGCCTCAATCAA
TP29755_Hit	D	0	.	5A	CAGCCCTTGATGGAACTAAAACTAATGCTTTAACAGTTTGCCACCATGATACAAGAGGTATGAA
TP29755_Query	D	0	.	5A	CAGCCCTTGATGGAACTAAAACTAAGGCTTTAACAGTTTGCCACCATGATACAAGAGGTATGAA
TP32604_Hit	D	0	.	5A	CAGCCTCCAACATCTCATTGTGTTGGGTTTGTATGGGTGGATTTAGTCCCAGGTTGCTTAGTGA
TP32604_Query	D	0	.	5A	CAGCCTCCAACATCTCATTGTGTTGGGTTTGCATGGGTGGATTTAGTCCCAGGTTGCTTAGTGA
TP51388_Hit	D	3	.	5A	CAGCTGGATGGTCTACTGCATGAAGAGAGCTGGTTGGAGTCCAGGATGAAAGTATTGATTGAGG
TP51388_Query	D	3	.	5A	CAGCTGGATGGTCTACTGCATGAAGAGAACTGGTTGGAGTCCAGGATGAAAGTATTGATTGAGG
TP70329_Hit	D	3	.	5A	CTGCATAACAGTATTGCAGTCATTTACTGTGGCTTGAAATCTGATTCCACTGGCTTTTACCATA
TP70329_Query	D	3	.	5A	CTGCATAACAGTATTGCAGTCATTTACTGTGGCTTGAAATCTGATTCCACTGGCTTTGACCATA
TP75898_Hit	D	0	.	5A	CTGCCCAATTTGCAAAACGAACATACCAGGAAGCAACCCTTTTTCCATCACGGGAGAACGAGCA
TP75898_Query	D	0	.	5A	CTGCCCAATTTGCAAAACGAACATACCAGGAAGCAACCCTTTTCCCATCACGGGAGAACGAGCA
TP78131_Hit	D	11	.	5A	CTGCCTACAACACACCAATTGGTGGGACCCCTTCCTGGACCCTGTGTATGCGGGAGCATTAGTG
TP78131_Query	D	11	.	5A	CTGCCTACAACACACCAATTGGTGGGACCCCTTCCTGGACCCTGCGTATGCGGGAGCATTAGTG
TP79972_Hit	D	0	.	5A	CTGCGACACCAAGGTTTTTGATGTCTCTGCAGTCGCGATTGAGGCCACATTAGCCACATTAATT
TP79972_Query	D	0	.	5A	CTGCGACACCAAGGTTTTTGATGTCTCTGCAGTCGCGATTGAGGCCACATCAGCCACATTAATT
TP8173_Hit	D	0	.	5A	CAGCAATTACTATTTTCTGGGCATTGAGCTTATTCGCACAGCAGGTGGCCTAATTCTCCTAGCA
TP8173_Query	D	0	.	5A	CAGCAATTACTATTTTCTGGGCATTGAGCTTATTCGCACAGCAGGTGGCCTAATTCTCCCAGCA
TP86684_Hit	D+G	0	.	5A	CTGCTATTCCTATTAAATATATTTTTTCCATTCATTGTGCTCAACTAAGAAGATTCTAATTCTT
TP86684_Query	D+G	0	.	5A	CTGCTATTCCTATTAAATATATTTTTTCCATTCACTGTGCTCAACTAAGAAGATTCTAATTCTT
TP87447_Hit	D	0	.	5A	CTGCTCATATGTAAATTGAAAAGAGTGTAATTAAGAATGTGTACTCTGCGGGTACGTATTTGCT
TP87447_Query	D	0	.	5A	CTGCTCATATGTAAATTGAAAAGAGTGTAATTAAGAATGTGTACTCTGCGGGTACGTATGTGCT
TP87938_Hit	D	0	.	5A	CTGCTCCGATTGTACGATTTTGACCCAGATAGTGAAGGGAAATCTCTCGTATTTTAACTACGAC
TP87938_Query	D	0	.	5A	CTGCTCCGATTGTACGATTTTGACCCAGATAGTGAAGGGAAATCTCTCGTATTTTAAATACGAC
TP14022_Hit	D	0	.	5B	CAGCAGATACACAGTGTAGAGAGCAGTATCAGTCCATCAAAAAACGGAATTACATATCATAATT
TP14022_Query	D	0	.	5B	CAGCAGATACACAGTGTAGAGAGCAGTATCAGTCCATCAAAAAAAGGAATTACATATCATAATT
TP20133_Hit	D	2	.	5B	CAGCATGTATTCCCTACTACTATAGAAGAACTTGAGGGGAAAAGAAGGATTGATGTTGAGGCTC
TP20133_Query	D	2	.	5B	CAGCATGCATTCCCTACTACTATAGAAGAACTTGAGGGGAAAAGAAGGATTGATGTTGAGGCTC

Name	Filter	Nb hit (Mt4.0)	Mt Chr (Mt4.0)	Ms Chr (Li et al., 2014)	Sequence
TP36251_Hit	D	0	.	5C	CAGCGAGCGTGAAGATTTACACGAGTTTGGTCTCTTTGGAGCGGAATAGATCTAAATCCAAAAT
TP36251_Query	D	0	.	5C	CAGCGAGCGTGAAGATTTACACGAGTTTGGTCTCTCTGGAGCGGAATAGATCTAAATCCAAAAT
TP43904_Hit	D	0	.	5C	CAGCTAGATAGTAGAGCTTACTTCTCTAGACATCAAACTAAGTTCAGTAACAAGTGCTATTGCT
TP43904_Query	D	0	.	5C	CAGCTAGATAGTAGAGCTTACTTCTCTAGACATCAAACTAAGTTCAGAAACAAGTGCTATTGCT
TP50693_Hit	D+G	0	.	5C	CAGCTGATACATGAGTGACACGTCAGAATTCAACTGAGACACAAAGAAACCCATTTTATCAGAA
TP50693_Query	D+G	0	.	5C	CAGCTGATACATGAGTGACACGTCAGAATTCAACTGAGACACAAAGAAACCCATTATATCAGAA
TP52590_Hit	D+G	0	.	5C	CAGCTGTGGAGATAAATTTAGATGGACATGATGTTGTGCTCAATTTTCTGCATATGTATCAGTT
TP52590_Query	D+G	0	.	5C	CAGCTGTGGAGATAAATTTAGATGGACAAGATGTTGTGCTCAATTTTCTGCATATGTATCAGTT
TP60523_Hit	D	0	.	5C	CTGCAAAAAATGTTTTTTTAAAGAAAGCTATTTAAATTAGTGTTCTTGCTAATTGATCGTGTTT
TP60523_Query	D	0	.	5C	CTGCAAAAAATGTTTTTTTAAAGAAAGCTATTTAAATTAGTGTTCTCGCTAATTGATCGTGTTT
TP76530_Hit	D	0	.	5C	CTGCCCGTCGCCGTCCTTTTATTAAATCACAATGTCGTCACTCTCCCCTATTCTTCTTCCTCTC
TP76530_Query	D	0	.	5C	CTGCCCGTCGCCGTCCTTTCATTAAATCACAATGTCGTCACTCTCCCCTATTCTTCTTCCTCTC
TP78054_Hit	D+G	0	.	5C	CTGCCGTTTGAACCATAATTTTTCAGTCAGCCGTTTAAACTGAGTTTGCTCCGTTGCTCTTATA
TP78054_Query	D+G	0	.	5C	CTGCCGTTTGAACCATAATTTTTCAGTCAGCCGTTCAAACTGAGTTTGCTCCGTTGCTCTTATA
TP87104_Hit	D	0	.	5C	CTGCTCAAGAACTAACATATCTAGTGGACACGCAGAAACACGATCAATTAGCGAGAACACTAAT
TP87104_Query	D	0	.	5C	CTGCTCAAGAACTAACATATCTAGTGGACACGCAGAAACACGATCAATTAGCAAGAACACTAAT
TP95617_Hit	D	2	.	5C	CTGCTTTTGGCCGAAATACTATACAAAAAGTGGTGATATCGTTGGAACAAACATGTATAGTTTA
TP95617_Query	D	2	.	5C	CTGCTTTTGGCCGAAATACTATACAAAAAGTGGTGATATCGTTGGAACAAACATGCATAGTTTA
TP43152_Hit	D+G	0	.	5D	CAGCTACATATCAACAAAACAGAGATAAGCATATCTTCATTAAGTTTCAAACACAATGAAATAC
TP43152_Query	D+G	0	.	5D	CAGCTACATATCAACAAAACAGAGATAAGCATATCTTCAATAAGTTTCAAACACAATGAAATAC
TP4904_Hit	D	2	.	5D	CAGCAACTTGATGGTGCTGTGGCTTTATTCAAGTTGGCCAACAAAGCTATGACTCTGTCTCCTG
TP4904_Query	D	2	.	5D	CAGCAACTTGATGGTGCTGTGGCTTTATTCAAGTTGGCCAACAAAGCTACGACTCTGTCTCCTG
TP56503_Hit	D	2	.	5D	CAGCTTCTTATCTTAACTCCTTGGTGAACAACAAGTTCCCTTACACAACTGATCAAGTCAGAGA
TP56503_Query	D	2	.	5D	CAGCTTCTTATCTTAACTCCTTAGTGAACAACAAGTTCCCTTACACAACTGATCAAGTCAGAGA
TP66326_Hit	D	0	.	5D	CTGCACCTTCTGTAAATAGTCAACCTTGATAAGTTGACACTATTAGAATGAGCACACATCATTC
TP66326_Query	D	0	.	5D	CTGCACCTTCTGTAAATAGTCAACCTTGATAAGTTGACACTATTAGAATAAGCACACATCATTC
TP72327_Hit	D	0	.	5D	CTGCATTAGCATCGCTTCCCCTCATAGACTTGTGCAATGCACTAATCAAATTATAGTGCTCTTC
TP72327_Query	D	0	.	5D	CTGCATTAGCATCACTTCCCCTCATAGACTTGTGCAATGCACTAATCAAATTATAGTGCTCTTC
TP7665_Hit	D	0	.	5D	CAGCAATGAGATGAATCAAGTGTTAAACCATCATAAAAACATCAAAATCCCTTGGCAGATACTT
TP7665_Query	D	0	.	5D	CAGCAATGAGATGAATCAAGTGTTAAACCATCATAAAAACATCAAAATCCCTTAGCAGATACTT
TP77276_Hit	D	0	.	5D	CTGCCGCAGGTTTTACAATTCGCATTGTGTCAGCTACAGAAGCTTGGCGAGAAATGGTCTTTCC
TP77276_Query	D	0	.	5D	CTGCCGCAGGTTTTACAATTCGCATTGGGTCAGCTACAGAAGCTTGGCGAGAAATGGTCTTTCC
TP80587_Hit	D	0	.	5D	CTGCGATGATCTTCATTTCTGCAAAACAACAAGTTATTAGCAACACGAATCAACAACGTACGGA
TP80587_Query	D	0	.	5D	CTGCGATGATCTTCATTTCTGAAAAACAACAAGTTATTAGCAACACGAATCAACAACGTACGGA
TP16830_Hit	D	4	.	6A	CAGCAGTTTGAGCTTGAAATGGAACAAAAGCGGAAATCTCTCGCTGAGGAGTTTAGCTGAAAAA
TP16830_Query	D	4	.	6A	CAGCAGTTTGAGCTTGAAATGGAACAAAAGCGGAAATCTCTCGCTGAGGAGTTTAGCAGAAAAA
TP19816_Hit	D+G	0	.	6A	CAGCATGACTGGTTGGTTTCTGGTTTTCTTGTTGTCCGTCTTGGTGTTGTCTGCTGAAAAAAAA
TP19816_Query	D+G	0	.	6A	CAGCATGACTGGTTGGTTTCTGGTTTTCTTGCTGTCCGTCTTGGTGTTGTCTGCTGAAAAAAAA
TP25370_Hit	D	0	.	6A	CAGCCACTGTTTCACATCTTTTTTAAAACTCGGGTTCATTGGTTCATTACAATCTGCATGAGTG
TP25370_Query	D	0	.	6A	CAGCCACTGTTTCACACCTTTTTTAAAACTCGGGTTCATTGGTTCATTACAATCTGCATGAGTG
TP35424_Hit	D	0	.	6A	CAGCGAAGAATTCTCTAGAATCACAAGCACTAAATACTTGGCATTCTAAAGACAGCATTTTGTT
TP35424_Query	D	0	.	6A	CAGCGAAGAATTCTCTAGAATCACAAGCACTAAATACTTGGCATTCCAAAGACAGCATTTTGTT
TP44389_Hit	D	0	.	6A	CAGCTAGTGTTCAATTTTTTATTCGGCAAATTTGTCACTGTTCAATTTCTGTCGAAGCAATTTT
TP44389_Query	D	0	.	6A	CAGCTAGTGTTCAATTTTTTATTCGGCAAATTTGTCACTGTTCAATTTCTGTCAAAGCAATTTT
TP67260_Hit	D	0	.	6A	CTGCACTTTAACTTCACTTGTGAATTGATTTTGTCTTGACCGTATCACATACTGTTGCGGTGTG
TP67260_Query	D	0	.	6A	CTGCACTTTAACTTCACTTGTGAATTGATTTTGTCTTAACCGTATCACATACTGTTGCGGTGTG
TP68076_Hit	D	9	.	6A	CTGCAGATACTCGATTCAACATATACATAAACAAAAGAGACATTCATCAAGGAAACCTACCAGC
TP68076_Query	D	9	.	6A	CTGCAGATACTCGATTCAACATATACATAAACAAAAGAGACATTCATCAAGGAAACCTACCACC
TP72906_Hit	D+G	0	.	6A	CTGCATTTCACTTTTGGATTCCTTTCCTGCCTATTACTGCTATGCAAATAGCTCGTTGTAGGTG
TP72906_Query	D+G	0	.	6A	CTGCATTTCACTTTTGGATTCCTTTCCTGCCTATTACTACTATGCAAATAGCTCGTTGTAGGTG
TP86302_Hit	D	0	.	6A	CTGCTATTTAACAACACTGTCTAAACTAGAGGAAACCAGAATGGCTTTGGTCAGATATTTTTCA
TP86302_Query	D	0	.	6A	CTGCTATCTAACAACACTGTCTAAACTAGAGGAAACCAGAATGGCTTTGGTCAGATATTTTTCA
TP92083_Hit	D+G	0	.	6A	CTGCTGTGTTGTGTTGATAATTTCTCATATTGCCCGTGTCACTGTTTACTTCCAATTCCAATTG
TP92083_Query	D+G	0	.	6A	CTGCTGTGTTGTGTTGATAATTTCTCATATTGCCCGTATCACTGTTTACTTCCAATTCCAATTG

Name	Filter	Nb hit (Mt4.0)	Mt Chr (Mt4.0)	Ms Chr (Li et al., 2014)	Sequence
TP14087_Hit	D	0	.	6B	CAGCAGATCAAGATCAATGGAGGAGACACTATAGAATAGTCTCATCTTCATCATGACAAAATGG
TP14087_Query	D	0	.	6B	CAGCAGATCAAGATCAATGGAGGAGACACTATAGAATAGTCGCATCTTCATCATGACAAAATGG
TP17905_Hit	D	0	.	6B	CAGCATATCTCATGTTAAAGTTGTTGGACAACCAATTTCTCCAGAATTTTTTGTGAGGATGCAT
TP17905_Query	D	0	.	6B	CAGCATATCCCATGTTAAAGTTGTTGGACAACCAATTTCTCCAGAATTTTTTGTGAGGATGCAT
TP48455_Hit	D	2	.	6B	CAGCTCGTCCTTTTGTAACTCATCACAATGAACTTGACATGAAATTATTCATGAGAATTGCTCC
TP48455_Query	D	2	.	6B	CAGCTCGTCCATTTGTAACTCATCACAATGAACTTGACATGAAATTATTCATGAGAATTGCTCC
TP51208_Hit	D	0	.	6B	CAGCTGGAATTCCAGGGGAGCCAATGGATATTATGACTGCAGTTCAGCTTGTGTTGAGAAAGTC
TP51208_Query	D	0	.	6B	CAGCTGGAATTCCAAGGGAGCCAATGGATATTATGACTGCAGTTCAGCTTGTGTTGAGAAAGTC
TP57243_Hit	D	0	.	6B	CAGCTTGATTCATCAATTCTACAAGCATACAAAATAATTTAAACCAGAATTCTAGTATTTTCTA
TP57243_Query	D	0	.	6B	CAGCTTGATTCATCAATTCTACAAGCATACAAAATAATTAAAACCAGAATTCTAGTATTTTCTA
TP77125_Hit	D	5	.	6B	CTGCCGAGGCTTTCAGACCAGACAAGACTTTAACGACTAATGACACTGAAACCGCTGTAGCTGA
TP77125_Query	D	5	.	6B	CTGCCGAGGCTTTCAGACCAGACAAGACTTTAACCACTAATGACACTGAAACCGCTGTAGCTGA
TP91578_Hit	D	0	.	6B	CTGCTGTCAAGTTTTGTTTGTGTTAATGTCTAGTAATTGATCAGTTTTTTGGGTGGGGTTTATT
TP91578_Query	D	0	.	6B	CTGCTGTCAAGTTTTGTTTGTGTTAATGTCTAGTAATTGATCAGTTTTTTGGGTGGGGTTTACT
TP22969_Hit	D+G	0	.	6C	CAGCCAAAGACGGTATGGAAGGGTCAGTGATGGCCCACACCGCAAGCTTCTTCAAGTGTTTAAT
TP22969_Query	D+G	0	.	6C	CAGCCAAAGACGGTATGGAAGGGTCAGTGATGGCCCACACCGCAAGCTTCTTCAAGTGTTCAAT
TP26618_Hit	D	0	.	6C	CAGCCATCATCCTTTAGCTTTGGAAGTTATGCTTTTGAGCTTGAGTGGAAATTCTACATGCAAG
TP26618_Query	D	0	.	6C	CAGCCATCATCCTTTAGCTTTGGAAGTTAAGCTTTTGAGCTTGAGTGGAAATTCTACATGCAAG
TP35122_Hit	D+G	0	.	6C	CAGCCTTTTTCATGTTCATCAGAATAAAGAATGAAATCATTAGCGGTGTTCATAAGAAATGGCT
TP35122_Query	D+G	0	.	6C	CAGCCTTTTTCATGTTCATCAGAATAAAGAACGAAATCATTAGCGGTGTTCATAAGAAATGGCT
TP35328_Hit	D	0	.	6C	CAGCGAACAAGGTTGCTCCATAGCCATTTCTTATGAACACCGCTAATGATTTCGTTCTTTATTC
TP35328_Query	D	0	.	6C	CAGCGAACAAGGTTGCTCCATAGCCATTTCTTATGAACACCGCTAATGATTTCATTCTTTATTC
TP51590_Hit	D+G	0	.	6C	CAGCTGGGAGTCTGTGTTCCGGTGCAAGACGGTACTCTATAGACGCAACAATGGCTTCAACGTC
TP51590_Query	D+G	0	.	6C	CAGCTGGGAGTCTATGTTCCGGTGCAAGACGGTACTCTATAGACGCAACAATGGCTTCAACGTC
TP59229_Hit	D	2	.	6C	CAGCTTTCTGCTTTCGAACTTTAGCGGCTTCCTGCTTCTGCTTTCGAACTTCAGCGGCTTTCTG
TP59229_Query	D	2	.	6C	CAGCTTTCTGCTTTCGAACTTCAGCGGCTTCCTGCTTCTGCTTTCGAACTTCAGCGGCTTTCTG
TP70680_Hit	D	0	.	6C	CTGCATATATACGGAACACCTAAAAACCAAAACCAAGTGATGGCCTCAAAAACACAATGAGGTC
TP70680_Query	D	0	.	6C	CTGCATATATACGGAACACCTAAAAACCAAAACCAAGTGATGGCCTCAAAAACACAATGAGGCC
TP42154_Hit	D	0	.	6D	CAGCTAACACATCCAATGCGCACAACTTCATAAGTTCTCTTCCAAATGGTGATGACACCTCTGT
TP42154_Query	D	0	.	6D	CAGCTAACACATCCAATGCGCACAACTTCATAAGTTCTCTTCCAAACGGTGATGACACCTCTGT
TP54638_Hit	D+G	0	.	6D	CAGCTTCAATTGATGCAAATTATCTGATGTAGTTTCAATTTTGGGAGTGTTCGTGCAGTGATTT
TP54638_Query	D+G	0	.	6D	CAGCTTCAATTGATGCAAATTAGCTGATGTAGTTTCAATTTTGGGAGTGTTCGTGCAGTGATTT
TP71606_Hit	D	0	.	6D	CTGCATGAAATAAGCAAACACTGTGAGGCAAGATCTCACTTTATCAAAGAACATGTGAACAAAA
TP71606_Query	D	0	.	6D	CTGCATGAAATAAGCAAACACAGTGAGGCAAGATCTCACTTTATCAAAGAACATGTGAACAAAA
TP85246_Hit	D	2	.	6D	CTGCTACTGAAATACCAAAAAGTCACTTTCAATTTGTGCCTTTTCTAGATAATTCATTGCACAT
TP85246_Query	D	2	.	6D	CTGCTACTGAAATACCAAAAAGTCACTTTCAATTTGTGCCTTTTCTAGAGAATTCATTGCACAT
TP22903_Hit	D+G	0	.	7A	CAGCCAAACCCGTCTCCACTCAGCTACGATAGGGATTTTTATTCATTGAAGGTTATTGACTTGC
TP22903_Query	D+G	0	.	7A	CAGCCAAACCCCTCTCCACTCAGCTACGATAGGGATTTTTATTCATTGAAGGTTATTGACTTGC
TP24265_Hit	D	0	.	7A	CAGCCACAGCCAACGCAAATAACCAAGTGATACATCAAAACAGGTCTCAAAATCCTCTTCAAAA
TP24265_Query	D	0	.	7A	CAGCCACAACCAACGCAAATAACCAAGTGATACATCAAAACAGGTCTCAAAATCCTCTTCAAAA
TP28960_Hit	D	0	.	7A	CAGCCCGATCCTCGTGATACTCATTTGAGTATTCTTCATAGTTGGGTTCATTCTGTGTCAAGTT
TP28960_Query	D	0	.	7A	CAGCCCGACCCTCGTGATACTCATTTGAGTATTCTTCATAGTTGGGTTCATTCTGTGTCAAGTT
TP50312_Hit	D	0	.	7A	CAGCTGACAGTAGCTCTTATCCTTTGTTTTGTTTTGTTAGCATGGAGTTTTTGCCGTTCCACCC
TP50312_Query	D	0	.	7A	CAGCTGACAGTAGCTCTTATCCTTTGTTTTGTTTTGTTAGCAGGGAGTTTTTGCCGTTCCACCC
TP6060_Hit	D	0	.	7A	CAGCAAGGCATAAACATTGAAGGGTACTTTCTTACAATGATATTCACACACCTTCATGTCAAAT
TP6060_Query	D	0	.	7A	CAGCAAGGCATAAACACTGAAGGGTACTTTCTTACAATGATATTCACACACCTTCATGTCAAAT
TP61476_Hit	D	0	.	7A	CTGCAAATAAGTTGTTCATCCAATTGTAATTGTAGAAATTTGGTTAATATTTTATGTTTTGTTT
TP61476_Query	D	0	.	7A	CTGCAAATAAGTTGTTCATCCAATTGTAATTGTAAAAATTTGGTTAATATTTTATGTTTTGTTT
TP64276_Hit	D	2	.	7A	CTGCAATGAGGAAGAGATAGAAAAGGGATCAAATGGACATGATTTATCTGACGATCATCGCCAA
TP64276_Query	D	2	.	7A	CTGCAATGAGGAAGAGATAGAAAAGGGATCAAATGGACATGATTTATCTGACGATCATCACCAA
TP70678_Hit	D	0	.	7A	CTGCATATAGTTGCTTGAAGCTACAAAAGATGTTTTCTCATCTCAAACTGATTGAGGACATTTT
TP70678_Query	D	0	.	7A	CTGCATATAGTTGCTTGAAGCTACAAAAGATGTTTTCTCATCTCAAACTGATTGAAGACATTTT
TP7417_Hit	D+G	0	.	7A	CAGCAATCCCTGCACCTTTTTCCAATACGAGCCTATCATTCTCCGACATGTGTCCATGTTTGAC
TP7417_Query	D+G	0	.	7A	CAGCAATCCCTGCACCTTTTTCCAATACGAGCCTATCATTCTCCGACACGTGTCCATGTTTGAC

Name	Filter	Nb hit (Mt4.0)	Mt Chr (Mt4.0)	Ms Chr (Li et al., 2014)	Sequence
TP76680_Hit	D	2	.	7A	CTGCCCTCCCTAAAGTCTGTGGTGCTAACATAGGCTATGAGATTAGCCCTGACATGGACTGCTG
TP76680_Query	D	2	.	7A	CTGCCCTCCCTAAAGTCTGTGGTGCTAACATAGGCTATGAGATTAGCCCTGACATGGACTGCAG
TP77993_Hit	D+G	0	.	7A	CTGCCGTGTTGAATTTTTGGTGTTCCTTTTCTCATTGTTTGCGGTGGTTTCAATTTATTTTTTC
TP77993_Query	D+G	0	.	7A	CTGCCGTGTTGAATTGTTGGTGTTCCTTTTCTCATTGTTTGCGGTGGTTTCAATTTATTTTTTC
TP89159_Hit	D	0	.	7A	CTGCTTTTCAACATCTCGATATCAGGCAAGAGTTCTTTGTGGTCTGGAAAAGAGGCTTTTCCTT
TP89159_Query	D	0	.	7A	CTGCTCTTCAACATCTCGATATCAGGCAAGAGTTCTTTGTGGTCTGGAAAAGAGGCTTTTCCTT
TP36878_Hit	D+G	0	.	7B	CAGCGATTGTGAGACCAAATCAACTACTCAGAAGCCTCTGCGAAGGACTAGAGCTAAGACATTC
TP36878_Query	D+G	0	.	7B	CAGCGATTGTGAGACCAAATCAACTACTCAAAAGCCTCTGCGAAGGACTAGAGCTAAGACATTC
TP50102_Hit	D+G	0	.	7B	CAGCTGAAGGTGATCCTTCCCACAAATTGGTTGAGGGCTTTTCTTCACAACACCAAATGAGGTC
TP50102_Query	D+G	0	.	7B	CAGCTGAAGGTGATCCTTCCCACAAATTGGTTGAGGGCTTTTCTTCACAACACCAAATGAGGCC
TP62899_Hit	D	0	.	7B	CTGCAACTTCTCTCCCCTATGAATGTCTTAGCTCTAGTCCTTCGCAGAGGCTTTTGAGTAGTTG
TP62899_Query	D	0	.	7B	CTGCAACTTCTCTCCCCTATGAATGTCTTAGCTCTAGTCCTTCGCAGAGGCTTCTGAGTAGTTG
TP80562_Hit	D	0	.	7B	CTGCGATGAAAATTTTGAATTCTTGTCGTAGACAATTTTGACGAATACTATATATAAAATGTCC
TP80562_Query	D	0	.	7B	CTGCGATGAAAATTTTGAATTCTTGTCGTAGACAATTTTGACGAATACTACATATAAAATGTCC
TP10246_Hit	D	0	.	7C	CAGCACCACCTCACACAAACAGGCCACCAAAACAAGCAACAAATCCTAGACCAAACCGCAAACA
TP10246_Query	D	0	.	7C	CAGCACCACCTCACACAAACAGGCCACCAAAACAAGCAACAAAGCCTAGACCAAACCGCAAACA
TP11833_Hit	D	0	.	7C	CAGCACTACTTTTTAGAATTGCACCATCAAATTATCTTGATATTCAAATATTTTTTTAAAAAGT
TP11833_Query	D	0	.	7C	CAGCACTACTTTTTAGAATTGCACCATCAAATTATCTTGATATTCAAATATCTTTTTAAAAAGT
TP16728_Hit	D	0	.	7C	CAGCAGTTGGTTTCTAAGTATGAGGAGTTGGAAGCTTCTCAAAGTTCAGAGGCACAAAGTGTTG
TP16728_Query	D	0	.	7C	CAGCAGTTGGTTTCTAAGTATGAGGAGTTGAAAGCTTCTCAAAGTTCAGAGGCACAAAGTGTTG
TP19041_Hit	D	4	.	7C	CAGCATCCTCCCTACCTCTGCCCCTACCACGTCCCACACCAGGTGGCTTGCGATCTGAAAAGGG
TP19041_Query	D	4	.	7C	CAGCATCCTCCCTACCTCTACCCCTACCACGTCCCACACCAGGTGGCTTGCGATCTGAAAAGGG
TP27244_Hit	D	0	.	7C	CAGCCATTTCACTTGAAAAACAGTAACAAGAAAATTATGCCTCTAACAATATTGAGAGTTCAAG
TP27244_Query	D	0	.	7C	CAGCCATTACACTTGAAAAACAGTAACAAGAAAATTATGCCTCTAACAATATTGAGAGTTCAAG
TP28361_Hit	D	0	.	7C	CAGCCCCAAAATTGCAAACAAAGTTACGGAGAAAATTTTCAACCATACAGTGTGAAACAAACAC
TP28361_Query	D	0	.	7C	CAGCCCCAAAATTGCAAACAAAGTTACGGAGAAAATTTTCAACCATACAGTATGAAACAAACAC
TP34354_Hit	D+G	4	.	7C	CAGCCTTCCAGTTATATAAAAAATAAAGTTATCTAATCAATGTTACCCTTTTCAGATCGCAAGC
TP34354_Query	D+G	4	.	7C	CAGCCTTCCAGTTATATAAAAAATAAAGTTATCTAATCAATGTCACCCTTTTCAGATCGCAAGC
TP36807_Hit	D+G	0	.	7C	CAGCGATTCAAGCAGGTATATACAAAACCCTAATTAATATTCTTTCATTATTTGATTTGATTTG
TP36807_Query	D+G	0	.	7C	CAGCGATTCAAGCAGGTATATACAAAACCCTAATTAATATTCTTCCATTATTTGATTTGATTTG
TP4400_Hit	D	2	.	7C	CAGCAACGGTTTTCTTTGGCAACAACACAGGGTTAATATTTGGCAAAACTCCACCGCTAGCAAT
TP4400_Query	D	2	.	7C	CAGCAACGGTTTTCTTTGGCAACAACACAGGGTTAATATTTGGCAAAACTCCACCACTAGCAAT
TP53005_Hit	D+G	0	.	7C	CAGCTGTTGGGAGTGTTTTTTAGAACCTGGTGCAGTGCTGTTTTTGCTGTTGGTTTTTCTGCGG
TP53005_Query	D+G	0	.	7C	CAGCTGTTGGGAGTGTTTTTTAGAACCTGGTGCAGTGCTGTTTTTGCGGTTGGTTTTTCTGCGG
TP53754_Hit	D+G	0	.	7C	CAGCTTAGAGGTTTGTGTTATCTCCAAGGCACTGTTTACCTAAATTCCAGTCATTTTCAGATTA
TP53754_Query	D+G	0	.	7C	CAGCTTAGAGGTTTGTGTTATCTCCAAGGCACTGTTTACCTAAATTCCAGTCATTTTCAAATTA
TP71510_Hit	D	0	.	7C	CTGCGTCTGTAATTTTGTATTTCTCTAAACCCATGTATTTCCTCACCAGAAAAACAGTTTTCCT
TP71510_Query	D	0	.	7C	CTGCATCTGTAATTTTGTATTTCTCTAAACCCATGTATTTCCTCACCAGAAAAACAGTTTTCCT
TP8152_Hit	D	0	.	7C	CAGCAATTAATTGCCTTTGATACTTTTAAGATCGGCTAAACCGACTTAAAAATTTATTAACCCG
TP8152_Query	D	0	.	7C	CAGCAATTAATTGCCTTTGATACTTTTAAGATCGGCTAAACCGACTTAAAAAGTTATTAACCCG
TP84253_Hit	D	2	.	7C	CTGCTAAATTGCGGAGCCTTCTTTTTCGTCATGAGATGAAGGCAAAACATGTTAAAAAGATTAA
TP84253_Query	D	2	.	7C	CTGCTAAATTGCGGAGCCTTCTTTTTCGTCATGAGATGAAGGCAAAACATGTTAAAAAGATAAA
TP85316_Hit	D	3	.	7C	CTGCTACTGTCCATGGTTTTCCTTTTTCTAGTCAGTTCCTCGTGTCTTTGAAAATGTTCAGTCA
TP85316_Query	D	3	.	7C	CTGCTACTGTCCATGGTATTCCTTTTTCTAGTCAGTTCCTCGTGTCTTTGAAAATGTTCAGTCA
TP91933_Hit	D+G	0	.	7C	CTGCTGTGCGGTTGTGTGTTTGCGGTTTGGTCTAGGCTTTGTTGCTTGTTTTGGTGGCCTGTTT
TP91933_Query	D+G	0	.	7C	CTGCTGTGCGGTTGTGTGTTTGCGGTTTGGTCTAGGATTTGTTGCTTGTTTTGGTGGCCTGTTT
TP93272_Hit	D	0	.	7C	CTGCTTCAGAACTCGAAACGAAACAATGAAATAAAATGAAAGTGCTTAAACCCTAATTATTTGT
TP93272_Query	D	0	.	7C	CTGCTTCAGAACACGAAACGAAACAATGAAATAAAATGAAAGTGCTTAAACCCTAATTATTTGT
TP93821_Hit	D	0	.	7C	CTGCTTCTGAGCCCAAAACTTGTTTGGTTACTCATTGAGTCATGACTGCTTCAAGAATCAAGAG
TP93821_Query	D	0	.	7C	CTGCTTCTGAGCCCAAAACTTGTTTGGTTACTCATCGAGTCATGACTGCTTCAAGAATCAAGAG
TP9499_Hit	D	0	.	7C	CAGCACAGCAACAACACAACCGCAGAAAAACCAACCGCAAAAACAGCACTGCACCAGGTTCTAA
TP9499_Query	D	0	.	7C	CAGCACAGCAACAACACAACCGCAGAAAAACCAACAGCAAAAACAGCACTGCACCAGGTTCTAA
TP12529_Hit	D	0	.	7D	CAGCACTTCTATGAACGATCTGTTTTCCCTTTTCCTTTGAGGAACCAAACCTTGCCGGTTAACA
TP12529_Query	D	0	.	7D	CAGCACTTCTACGAACGATCTGTTTTCCCTTTTCCTTTGAGGAACCAAACCTTGCCGGTTAACA

Name	Filter	Nb hit (Mt4.0)	Mt Chr (Mt4.0)	Ms Chr (Li et al., 2014)	Sequence
TP33876_Hit	D	2	.	7D	CAGCCTGTGATGTGGTGGATTTTACAGCTGATGAACCAAATTTTGAACCATTATCAGTGGTCCA
TP33876_Query	D	2	.	7D	CAGCCTGTGATGTGGTGGATTTTACAGCAGATGAACCAAATTTTGAACCATTATCAGTGGTCCA
TP34007_Hit	D+G	0	.	7D	CAGCCTTAACAACAACATTTTCTGATTCAACAGACCCATTATTATCTAAAACTTTCTCCACCAT
TP34007_Query	D+G	0	.	7D	CAGCCTTAACAACAACATTTTCTGATTCAACAGAACCATTATTATCTAAAACTTTCTCCACCAT
TP38578_Hit	D	2	.	7D	CAGCGGAGATAAGAGACCCAAAAAAGAATGGTGCTAGGGTTTGGCTTGGTACTTATGTGACTGA
TP38578_Query	D	2	.	7D	CAGCGGAGATAAGAGACCCAAAAAAGAATGGTGCTAGGGTTTGGCTTGGTACTTATGCGACTGA
TP42358_Hit	D+G	0	.	7D	CAGCTAAGAAGGAATTTTATGACGCCATTGTGAGTAAATGTATGGAGGATGTACATGAAGCAAG
TP42358_Query	D+G	0	.	7D	CAGCTAAGAAGGAATTTTATGACGCCATTGTGAATAAATGTATGGAGGATGTACATGAAGCAAG
TP49342_Hit	D	0	.	7D	CAGCTCTTCCAACTCTGACCGCCTACACAGGACAGAACCCACAGGCCCCTGCTGAAAAAAAAAA
TP49342_Query	D	0	.	7D	CAGCTCTTCCAACTCTGACCGCCTACACAAGACAGAACCCACAGGCCCCTGCTGAAAAAAAAAA
TP55411_Hit	D+G	3	.	7D	CAGCTTCCTCAGCAGTGTTAAATGTACCAAGCCAAACTCGAGCACCATGCCTAGCCGAATCTCG
TP55411_Query	D+G	3	.	7D	CAGCTTCCTCAGCAGTGTTAAATGTACCAAGCCAAACACGAGCACCATGCCTAGCCGAATCTCG
TP62157_Hit	D+G	0	.	7D	CTGCAACAGCAGATTCATAACGCTTTCTTGCTTCATGTACATCCTCCATACATTTATTCACAAT
TP62157_Query	D+G	0	.	7D	CTGCAACAGCAGATTCATAACGCTTTCTTGCTTCATGTACATCCTCCATACATTTACTCACAAT
TP86600_Hit	D+G	2	.	7D	CTGCTATTGAGCAAGCCATTAGTGATGGTGTTGATATAATCTCACTTTCTCTTGGAAGCAGTAC
TP86600_Query	D+G	2	.	7D	CTGCTATTAAGCAAGCCATTAGTGATGGTGTTGATATAATCTCACTTTCTCTTGGAAGCAGTAC
TP87697_Hit	D+G	0	.	7D	CTGCTCCGCTCAACCCGCACCCGACTAAACCGATGGCTTGTTTGGTCGGAAGTGGATGCATATT
TP87697_Query	D+G	0	.	7D	CTGCTCCACTCAACCCGCACCCGACTAAACCGATGGCTTGTTTGGTCGGAAGTGGATGCATATT
TP89953_Hit	D+G	0	.	7D	CTGCTGAGGTATTTATTTTTTCATTTAACGTTTCTTGGAACAATTGTCAGAAGTGCTTCTAAAT
TP89953_Query	D+G	0	.	7D	CTGCTGAGGTATTTATTTTTTCATTTAACGCTTCTTGGAACAATTGTCAGAAGTGCTTCTAAAT
TP11078_Hit	D+G	0	.	8A	CAGCACCTTGTTGCTAAGCGAAACAGCTATCCGACAGTGGTATTCACCAAATGGATGTGTGGAA
TP11078_Query	D+G	0	.	8A	CAGCACCTTGTTGCTAAGCGAAACAGCTATCCGACAGTGGTATTCACCAAATGGATGTATGGAA
TP1155_Hit	D	0	.	8A	CAGCAAACAATTCCTTTACACGATGCCAAAAGTACAACATCTTCGGGAAGAAAAGCAAAAACCT
TP1155_Query	D	0	.	8A	CAGCAAACAATTCCTTTACACGATGCCAAAACTACAACATCTTCGGGAAGAAAAGCAAAAACCT
TP1374_Hit	D+G	0	.	8A	CAGCAAACGACAGTAGGGCGGTCGTTGGGAGAAGAGGCCGCTTGCCTTTTTGGAGAGCTTAAAG
TP1374_Query	D+G	0	.	8A	CAGCAAACGACAGTAGGGCAGTCGTTGGGAGAAGAGGCCGCTTGCCTTTTTGGAGAGCTTAAAG
TP22003_Hit	D	3	.	8A	CAGCATTTACCTCTGTTTTATACATTTCTGGTTTAGTTGCATCCTTGGTGGCATCTACAGTTAC
TP22003_Query	D	3	.	8A	CAGCATTTACCTCTGTTTTATACATTTCTGGTTTAGTTGCATCCTTGGTGGCATCTACAATTAC
TP24985_Hit	D	0	.	8A	CAGCCACCTTGATGATGAAGTCGAACTGCTCCTTATGTTTGGTGGCATCGATATTGAATTAAGT
TP24985_Query	D	0	.	8A	CAGCCACCTTGATGATGAAGTCGAACTGCTCCTTATGTTTGGTGGCATCGATATTGAATTAAGA
TP31604_Hit	D	0	.	8A	CAGCCGTTTGAGTTAAAAATAAAACCTTTAAAATTTCTGTTTGTTGTCATCTTCACTCATTTTC
TP31604_Query	D	0	.	8A	CAGCCGTTTGAGTTAAAAATAAAACCTTTAAAATTTCTGTTTGTTGTCATATTCACTCATTTTC
TP3638_Hit	D	2	.	8A	CAGCAACATAATGAAGAAGTCAAAGAAGGATTTTCTTGATAGTTTTGAGGTCAAAAATATCACA
TP3638_Query	D	2	.	8A	CAGCAACATAATGAAGAAGTCAAAGAAGGATTTTCTTAATAGTTTTGAGGTCAAAAATATCACA
TP36642_Hit	D	0	.	8A	CAGCGATGAGGGAAATGATATTTAGAATAGCAGTTATGCAACCTATACATATCGATCCCGAATC
TP36642_Query	D	0	.	8A	CAGCGATGAGGGAAATGATATTTAGAATAGCAGTGATGCAACCTATACATATCGATCCCGAATC
TP36763_Hit	D+G	0	.	8A	CAGCGATGTTCCACCGGGGCTGACGCGGCGTTCATGTTCTGCTGAAAAAAAAAAAAAAAAAAAA
TP36763_Query	D+G	0	.	8A	CAGCGATGTTCCACCGGGGCTGACGCGGCGTTCATGTTCCGCTGAAAAAAAAAAAAAAAAAAAA
TP3886_Hit	D	0	.	8A	CAGCAACCAAAATATCACAAGTAAGCGCAATCTCAAATCCAGCTGTAACAGCGAATCCTTTAAT
TP3886_Query	D	0	.	8A	CAGCAACCAAAATATCACAAGCAAGCGCAATCTCAAATCCAGCTGTAACAGCGAATCCTTTAAT
TP43069_Hit	D	0	.	8A	CAGCTACACTTTGTGGATCCTTTGATATCTTCACATTCCTTCTCTTCGGTGGCCTAACGGATTC
TP43069_Query	D	0	.	8A	CAGCTACACTTTGTGGATCCTTTGATATCTTCACATTCCTTCTCTTCGGCGGCCTAACGGATTC
TP43755_Hit	D	0	.	8A	CAGCTAGAAGATCCTGGCATTGACATTCACAGGGAAGGGAAATACTTGATGCTTGCTGTTCAGG
TP43755_Query	D	0	.	8A	CAGCTAGAAGATCCTGGCATTGACATTCACAAGGAAGGGAAATACTTGATGCTTGCTGTTCAGG
TP44459_Hit	D+G	0	.	8A	CAGCTAGTTTTTCATAATGCAAAACTTGCAAATGCGATGGTACAATATGATTCCCAATGAACTT
TP44459_Query	D+G	0	.	8A	CAGCTAGTTTTTCATAATGCAAAACTTGCAAATGCGATGATACAATATGATTCCCAATGAACTT
TP46787_Hit	D	0	.	8A	CAGCTCATGCCAAAAATTGGGCATCTTTTCTTGATCTAAATTTGCGTCATGATATTCAGGAGAT
TP46787_Query	D	0	.	8A	CAGCTCATGCCAAAAATTGGGCATCTTTTCTTGATCTAAATCTGCGTCATGATATTCAGGAGAT
TP48235_Hit	D+G	0	.	8A	CAGCTCGCCATTCCTCAAGCAGGTGCATTGCACACTCAACAACCTGAGTACTTGTTTCATTTTC
TP48235_Query	D+G	0	.	8A	CAGCTCGCCATTCCTCAAGCAGGTGCATTGCACACTCAAAAACCTGAGTACTTGTTTCATTTTC
TP61099_Hit	D	0	.	8A	CTGCAAACCTAGAGTACATAACAGGTGCCCTACTTGTAGGAATAAGCTGGGAAACATTAGATTT
TP61099_Query	D	0	.	8A	CTGCAAACCTAGAGTACATAACAGGTGCCCTACTTGTAGGAATAAGCTGGGAAACATTAGATGT
TP63926_Hit	D	0	.	8A	CTGCAATATCAAAGTTCGCACCATAACCATAACCGTAATTTAAAACCATAGTACTACTCATACA
TP63926_Query	D	0	.	8A	CTGCAATATCAAAGTTCGCACCATAACCACAACCGTAATTTAAAACCATAGTACTACTCATACA

Name	Filter	Nb hit (Mt4.0)	Mt Chr (Mt4.0)	Ms Chr (Li et al., 2014)	Sequence
TP71195_Hit	D	0	.	8A	CTGCATCCATATATTTTATCTACCCTTGAATCACTGCATAATTTACAGCTTGAAAAATGTTTGT
TP71195_Query	D	0	.	8A	CTGCATCCATATATTTTATCTACCCTTGAATCACTGCATAATTTACAGCTTAAAAAATGTTTGT
TP72128_Hit	D	0	.	8A	CTGCATGTGCATTGTGCAATGTGCATAGAAAACTGAAATATGAGAGCCTATTTGAATCTCCATG
TP72128_Query	D	0	.	8A	CTGCATGTGCATTGTGCAATGTGCATAGAAAATTGAAATATGAGAGCCTATTTGAATCTCCATG
TP80642_Hit	D+G	0	.	8A	CTGCGATGTAATTGAATAAACTTCTATTGTTTTATAAGATTTAGGGTTCATCTCCCGCGTGTTG
TP80642_Query	D+G	0	.	8A	CTGCGATGTAATTGAATAAACTTCTATTGTTTTATAAGATTTAGGGTTCATCGCCCGCGTGTTG
TP81344_Hit	D+G	0	.	8A	CTGCGCGAATTCCTTGAAAACAACTGCGAAATCATTGAGTGATAGCTTGTTTTTGAAGGAATCG
TP81344_Query	D+G	0	.	8A	CTGCGCGAATTCCTTGAAAACAACTGCGAAATCATTGAGTGATAGCTTGTTTTTGAAGGAATCA
TP84208_Hit	D+G	0	.	8A	CTGCTAAAGTAGTTCACCACAAGGTTTTTGCTTTTCTTCCCGAAGATGTTGTAGTTTTGGCATC
TP84208_Query	D+G	0	.	8A	CTGCTAAAGTAGTTCACCACAAGGTTTTTGCTTTTCTTCCCGAAGATGTTGTACTTTTGGCATC
TP85458_Hit	D	2	.	8A	CTGCTAGAAGCCATAACTTTGCAGATGAGATCTTCTTGAGGGCCATCAGGTTGAAACACCTCCT
TP85458_Query	D	2	.	8A	CTGCTAGAAGCCATAACTTTGCAGATGAGATCTTCTTGAGAGCCATCAGGTTGAAACACCTCCT
TP86676_Hit	D+G	0	.	8A	CTGCTATTCCAGATTTGCAGTAAACCTGTTTTGTTGATGTAAAGATGGTATTTTACATTGCCAC
TP86676_Query	D+G	0	.	8A	CTGCTATTCCAGATTTGCAGTAAACCTGTTTTGTTGATGTAAAGATGATATTTTACATTGCCAC
TP95569_Hit	D	2	.	8A	CTGCTTTTGATCGACGAGGACCAGAAAGGCATGGTAGACAACTGGTTAGTGAACTGGATATCTT
TP95569_Query	D	2	.	8A	CTGCTTTTGATCGACAAGGACCAGAAAGGCATGGTAGACAACTGGTTAGTGAACTGGATATCTT
TP20309_Hit	D	0	.	8B	CAGCATGGAATACTGTGAGTTGTTATGCATCATCATAGTTTTCTTTATACTTGAAAAATAGTTG
TP20309_Query	D	0	.	8B	CAGCATGGAATACTGTGAGTTGTTATGCATCATCATAGTTTTCTTTATACTTAAAAAATAGTTG
TP2198_Hit	D	0	.	8B	CAGCAAATCCACCGGCACCGATTACAGGAATCAGAGTACCCAGAGTTGGAGCTAGAGCTCCAAG
TP2198_Query	D	0	.	8B	CAGCAAATCCACCGGCACCGATTACAGGAATCAGAGTACCCAAAGTTGGAGCTAGAGCTCCAAG
TP33753_Hit	D	0	.	8B	CAGCCTGGTATTTTGATAGGTTCTGGTAAATCTCTGTACTCACATGATGTTTCCTCGTCCAACT
TP33753_Query	D	0	.	8B	CAGCCTGGTATTTCGATAGGTTCTGGTAAATCTCTGTACTCACATGATGTTTCCTCGTCCAACT
TP35437_Hit	D	0	.	8B	CAGCGAAGAGATCGCCGGAGTTGGGATCTTCTAACCGTATCACACAGCGGTCACGGCAGGGCAC
TP35437_Query	D	0	.	8B	CAGCGAAGAGATCGCCGGAGTTGGGATCTTCTAACCGTATCACACAGCGGTCACGGCAGGACAC
TP50008_Hit	D	0	.	8B	CAGCTGAAGCAAATGAGGAGGGGGACAAATTGTCGCAATATTTTGTTTTATTTTTTGTGTCTCA
TP50008_Query	D	0	.	8B	CAGCTGAAGCAAATGAGGAGGGGGACAAATTGTCACAATATTTTGTTTTATTTTTTGTGTCTCA
TP62402_Hit	D	0	.	8B	CTGCAACCTCACCGAGCTCCTTTCTGGAATATTTTTCATAAGCCACTCTTAACTTTCCCTTCCC
TP62402_Query	D	0	.	8B	CTGCAACCCCACCGAGCTCCTTTCTGGAATATTTTTCATAAGCCACTCTTAACTTTCCCTTCCC
TP66048_Hit	D	0	.	8B	CTGCACCCCTGCACCTTTCAGACCCTAAGCCTGAAACTTTAAGCTCTCCAAAAAGGCAAGCGGC
TP66048_Query	D	0	.	8B	CTGCACCCCTGCACCTTTCAAACCCTAAGCCTGAAACTTTAAGCTCTCCAAAAAGGCAAGCGGC
TP67839_Hit	D	0	.	8B	CTGCAGACTCCTCGATCGTGCTAACCAGCTATGAAGGTCAACACACTCACCTATCCCCGGTTCT
TP67839_Query	D	0	.	8B	CTGCAGACTCCTCGATCGTGCTAACCAGCTATGAAGGTCAACACACCCACCTATCCCCGGTTCT
TP69824_Hit	D	0	.	8B	CTGCAGTGCACCAAAATGGGTTTCTTAGACTGATAGAAATTCATGTTAGCTAGGCATTGCATCT
TP69824_Query	D	0	.	8B	CTGCAGTGCACCAAAATGGGTTTCTTAGACCGATAGAAATTCATGTTAGCTAGGCATTGCATCT
TP7026_Hit	D+G	3	.	8B	CAGCAATAGGCACAGTGCCAGTTCGATCAAACCCGAGGATAATCCACTCTTCACCACAAACATG
TP7026_Query	D+G	3	.	8B	CAGCAATAGGCACAGTGCCAGTTCGATCAAACCAGAGGATAATCCACTCTTCACCACAAACATG
TP79065_Hit	D	2	.	8B	CTGCCTGTGTGTTGCCTTCTTTGCGCTAGGTTGTTGTTTAAGCGGTTTGGCCTTGATTGGCTTT
TP79065_Query	D	2	.	8B	CTGCCTGTGTGTTGCCTTCTTTGCGCTAGGTTGTTGTTTAAGCGGTTCGGCCTTGATTGGCTTT
TP84407_Hit	D	0	.	8B	CTGCTAAGAGATGCAATGCCTAGCTAACATGAATTTCTATCGGTCTAAGAAACCCATTTTGGTG
TP84407_Query	D	0	.	8B	CTGCTAAGAGATGCAATGCCTAGCTAACATGAATTTCTATCAGTCTAAGAAACCCATTTTGGTG
TP85745_Hit	D+G	0	.	8B	CTGCTAGGGCCTACATGCTCCTTCTAGTTGGGTGTACTGTTTTTACGGACAAGAGTTTTACTCT
TP85745_Query	D+G	0	.	8B	CTGCTAGGGCCTACATGCTCCTTCTAGTTGGGTGTACTATTTTTACGGACAAGAGTTTTACTCT
TP90156_Hit	D	0	.	8B	CTGCTGATCTTTATGATTGGTTGCGAGGAAAAAATGTTAACGAAGCATCCATAGTTGGTGTTGG
TP90156_Query	D	0	.	8B	CTGCTGATCTTTATGAGTGGTTGCGAGGAAAAAATGTTAACGAAGCATCCATAGTTGGTGTTGG
TP15377_Hit	D+G	0	.	8C	CAGCAGGTGTTTTACAGCCAAAACTTTGATGCAGGCCTGAGAGCTGGAACAATGTGATTAATTG
TP15377_Query	D+G	0	.	8C	CAGCAGGTGTTTTACAGACAAAACTTTGATGCAGGCCTGAGAGCTGGAACAATGTGATTAATTG
TP18832_Hit	D	0	.	8C	CAGCATCATTTAAGCAAAACTAGTTCTCAAATCAACAATATCACTGCATTCTCATCTAACTAAA
TP18832_Query	D	0	.	8C	CAGCATCATTTAAGCAAAACTAGTTCTCAAATCAACAATACCACTGCATTCTCATCTAACTAAA
TP20798_Hit	D	0	.	8C	CAGCATGTTCAACTGCTAGTGTCACAGATCTGTTGCGGAGTGCCGGTAAATCCTATTGCCCAGA
TP20798_Query	D	0	.	8C	CAGCATGTTCAACTGCTAGTGTCACAGATCTGTTGCGGAGTGCCGATAAATCCTATTGCCCAGA
TP23826_Hit	D	0	.	8C	CAGCCAATAACTGTGATTGAACGATAAAACCAACAGGAAAAGAAACAATCTGATGCAAAAGAAC
TP23826_Query	D	0	.	8C	CAGCCAATAACAGTGATTGAACGATAAAACCAACAGGAAAAGAAACAATCTGATGCAAAAGAAC
TP24946_Hit	D	2	.	8C	CAGCCACCTCCCATACCGGTGCAGGTATTTTGGCCTCCCTTTCCTGCTCCAGTAGTAGGGAGGA
TP24946_Query	D	2	.	8C	CAGCCACCTCCCATACCGGTGCAGGTATTTTGGCCTCCCTTTCCTGCACCAGTAGTAGGGAGGA

Name	Filter	Nb hit (Mt4.0)	Mt Chr (Mt4.0)	Ms Chr (Li et al., 2014)	Sequence
TP49243_Hit	D	0	.	8C	CAGCTCTGTTCAAACTCGGTGACGAGCTCCAACGCTAATACAAGAAGCCTTAGAAAAACTACAG
TP49243_Query	D	0	.	8C	CAGCTCTGTTCAAACTCGGTGACGAGCTCCAATGCTAATACAAGAAGCCTTAGAAAAACTACAG
TP51928_Hit	D	2	.	8C	CAGCTGGTGTCATTGTTAACAATGCAAGTTATTATGGTTTAGCAAAAGGAACAGCAAGAGGTGG
TP51928_Query	D	2	.	8C	CAGCTGGTGTCAATGTTAACAATGCAAGTTATTATGGTTTAGCAAAAGGAACAGCAAGAGGTGG
TP63059_Hit	D+G	0	.	8C	CTGCAAGACAGGCACCAAGAGAACCAAGATCCATTCCTTCGATACACTGACAAACAACCATAGC
TP63059_Query	D+G	0	.	8C	CTGCAAGACAAGCACCAAGAGAACCAAGATCCATTCCTTCGATACACTGACAAACAACCATAGC
TP63129_Hit	D	2	.	8C	CTGCACGAGCTTATGATGCTGAAGCTAGGAGAATCCGTGGCAAGAAAGCCAAGGTGAATTTCCC
TP63129_Query	D	2	.	8C	CTGCAAGAGCTTATGATGCTGAAGCTAGGAGAATCCGTGGCAAGAAAGCCAAGGTGAATTTCCC
TP64583_Hit	D	0	.	8C	CTGCAATTACCATAGAAAATTCAACAAACCCTACCACCTCCCCTGCAAGTCAACTTGTGGAAAA
TP64583_Query	D	0	.	8C	CTGCAATTACCATAGAAAATTCAACAAACCCTACAACCTCCCCTGCAAGTCAACTTGTGGAAAA
TP69105_Hit	D	0	.	8C	CTGCAGGATTATCATCACAAACACTAGGAGCATCAGATACACGGAGAATATCTGGAATTCTTTC
TP69105_Query	D	0	.	8C	CTGCAGGATTATCATCACAAACACTAGGAGCATCAGATACACGGAGAATATCCGGAATTCTTTC
TP78270_Hit	D+G	2	.	8C	CTGCCTATGCAAAATGATTCTGCAAAGACCCTCTCTGAGGAGCTTGCAGATATCGAATCCCAGC
TP78270_Query	D+G	2	.	8C	CTGCCTATGCAAAATGATTCTGCAAAGACCCTCTCTGAGGAGCTCGCAGATATCGAATCCCAGC
TP85598_Hit	D	2	.	8C	CTGCTAGCAGTCACTCCTTTGCCAAGTCCACTAACAACTCCACCAGTCACCAAAACATACTTCA
TP85598_Query	D	2	.	8C	CTGCTAGCAGTCACTCCTTTCCCAAGTCCACTAACAACTCCACCAGTCACCAAAACATACTTCA
TP94240_Hit	D+G	0	.	8C	CTGCTTGCAGAGCAAAAATATGGAGCAGGAGAACGAGGACCGATTATCGAATCTACTGAAAGTC
TP94240_Query	D+G	0	.	8C	CTGCTTGCAGAGCAAAAATATGGAGCAGGAGAACGAGGACCGATTATCGAATCTACCGAAAGTC
TP18029_Hit	D	0	.	8D	CAGCATATGTAACAAATTTAGTTGGAAAAAGGCTGATAAAATAGCCCTCTATTAAAGGGGAAAA
TP18029_Query	D	0	.	8D	CAGCATATGTAACAAATTTAGTTGGAAAAAGGTTGATAAAATAGCCCTCTATTAAAGGGGAAAA
TP22647_Hit	D	0	.	8D	CAGCCAAAAAGCACGAAGGGAAACTTGAAATTGGGTTATCTCATTTCCTCAACCACAAACACGA
TP22647_Query	D	0	.	8D	CAGCCAAAAAGCACGAAGGGAAACTTGAAATTGGGTAATCTCATTTCCTCAACCACAAACACGA
TP25724_Hit	D	0	.	8D	CAGCCAGCAACAGTAGCCTAACCAGAAGTAAACAAAGCCGCAAAAACACAAAAATCAGAATGGA
TP25724_Query	D	0	.	8D	CAGCCAGCAACAGTAGCCTAACCAGAAGTAAACAAAGCCGCAAAAACACAAAAAACAGAATGGA
TP32695_Hit	D	0	.	8D	CAGCCTCCCCCAACAGCACCTTCTCTTTCTTTGCACTGGTCATTGCATATGTTAGAGCCGACAC
TP32695_Query	D	0	.	8D	CAGCCTCCCCCAACAGCACCCTCTCTTTCTTTGCACTGGTCATTGCATATGTTAGAGCCGACAC
TP45906_Hit	D	0	.	8D	CAGCTCAACTGGCTTATCACTTAGGTTCCATATCCTTGTCCCGAATCCCGAGCCTCGTGCTTGG
TP45906_Query	D	0	.	8D	CAGCTCAACTGGCTTATCACTTAGGTTCCATATCCTTGTCCCAAATCCCGAGCCTCGTGCTTGG
TP48399_Hit	D+G	2	.	8D	CAGCTCGGTGCTTGAAAATCATATCCCACCCTATGTTTGCATCAGGAAAGCTTCAAACGTGAAT
TP48399_Query	D+G	2	.	8D	CAGCTCGGTGCTTGAAAATCATATCCCACCCCATGTTTGCATCAGGAAAGCTTCAAACGTGAAT
TP52672_Hit	D	0	.	8D	CAGCTGTGTCCTTGTTCTTGTATGCTATATATCAAGATGTTCAAGACTTCAAGGATCGATGCAA
TP52672_Query	D	0	.	8D	CAGCTGTGTCCTTGTTCTTGTATACTATATATCAAGATGTTCAAGACTTCAAGGATCGATGCAA
TP69389_Hit	D+G	0	.	8D	CTGCAGGTCGGTCATCATGTTTTCTTGCCTTCTGGTCTGCCTCTGGTGCAGTGGTACTGCTGAA
TP69389_Query	D+G	0	.	8D	CTGCAGGTCGGTCATCATGTTTTCTTGCCTTCTGGTCTGCCTCTGGTGCAGTGGTACCGCTGAA
TP70148_Hit	D	0	.	8D	CTGCAGTTGTTGAGAAGAAGCGTCGTGACTTGGCTTTGAAACATAACTCGTTTGTTTCGCCCGT
TP70148_Query	D	0	.	8D	CTGCAGTTGTTGAGAAGAAGCGTCGTGACTCGGCTTTGAAACATAACTCGTTTGTTTCGCCCGT
TP75859_Hit	D+G	4	.	8D	CTGCCCAAGCTTTATAAACACTACACATGCCATATATCTATGCAATGTGGGACTAAATCCACCC
TP75859_Query	D+G	4	.	8D	CTGCCCAAGCTTTATAAACACTACACATGCCATATATCTATGCAATGTGGGACAAAATCCACCC
TP78127_Hit	D+G	2	.	8D	CTGCCTAATTTTTGAAGGAACTGTACCTCTTCCATGGTCCAACAGGGTGAAGATTGCACTTGGT
TP78127_Query	D+G	2	.	8D	CTGCCTAATTTTTGAAGGAACTGTACCACTTCCATGGTCCAACAGGGTGAAGATTGCACTTGGT
TP82647_Hit	D+G	0	.	8D	CTGCGGTAAAAGAAATCGATTCCTTTCTTTTTCTTTGTTTGCCCGCTAGTTGTTTGTGGCAGGG
TP82647_Query	D+G	0	.	8D	CTGCGGTAAAAGAAATCGATTCCTTTCTTTTTCTTCGTTTGCCCGCTAGTTGTTTGTGGCAGGG
TP8545_Hit	D	0	.	8D	CAGCAATTTCACATACAGTTTCTATTCAGCTACTCCTCAAGAACGCGACCTATCACGAGGTGGG
TP8545_Query	D	0	.	8D	CAGCAATTTCACATACAGTTCCTATTCAGCTACTCCTCAAGAACGCGACCTATCACGAGGTGGG
TP11280_Hit	D	1	scaffold	.	CAGCACGCAAATGTCTACGATTACCCTTGACATTGTTACCCAAACCATGCTGGTTCCACCGGTG
TP11280_Query	D	1	scaffold	.	CAGCACGCAAATGTCTACGATTACCCTTGACATTGTCACCCAAACCATGCTGGTTCCACCGGTG
TP11432_Hit	D	1	scaffold	.	CAGCACGGAATATCCCGTTGGCCTTCTCGAAAGATCAGAAAGGTTAACCGTTCGCTGTCCAAGC
TP11432_Query	D	1	scaffold	.	CAGCACGGAATATCCCGTTGGCCTTCTCGAAAGATCAAAAAGGTTAACCGTTCGCTGTCCAAGC
TP13310_Hit	D	1	scaffold	.	CAGCAGAATCAGTCTATCAAGGCACTTAAGGAACACGTTGTCGATAGCGCCGTGCATGCTGATT
TP13310_Query	D	1	scaffold	.	CAGCAGAATCAGTCTATCAAGGCACTTAAGGAACACGTTGTCGATAGCGCAGTGCATGCTGATT
TP15780_Hit	D	1	scaffold	.	CAGCAGTAGTGTCTTATTTTAGGACTTGTTTTTATGACTTTTTGGATATTTGTAGTATTTTCCT
TP15780_Query	D	1	scaffold	.	CAGCAGTAGTGTCTTATTTTAGGACTTGTTTTTATGACTTTTTGGATATTTGTAGTATTTTCCA
TP17106_Hit	D	1	scaffold	.	CAGCATAATGAATTTTACCTTAGAACTTCCTTTCTTTTTAGAATTTCTGTCCAGTTGTAATCTA
TP17106_Query	D	1	scaffold	.	CAGCATAACGAATTTTACCTTAGAACTTCCTTTCTTTTTAGAATTTCTGTCCAGTTGTAATCTA

Name	Filter	Nb hit (Mt4.0)	Mt Chr (Mt4.0)	Ms Chr (Li et al., 2014)	Sequence
TP17661_Hit	D	1	scaffold	.	CAGCATAGGCCAATGGCGCCTAAGCAGAAAATGGGGAACCCTGGAAGTGGTGGCGGCGAAGATG
TP17661_Query	D	1	scaffold	.	CAGCATAGGCCAATGGCGCCTAAGCAGAAAACGGGGAACCCTGGAAGTGGTGGCGGCGAAGATG
TP18075_Hit	D+G	1	scaffold	.	CAGCATATTATGGGTACTTAATAGGTTGTGACAATATTTTGGCTCAACTCCACACGAAATACCC
TP18075_Query	D+G	1	scaffold	.	CAGCATATTATGGGTACTTAATAGGTTGTGACAATATTTTGGCTCAACTCCACACGAAACACCC
TP19268_Hit	D+G	1	scaffold	.	CAGCATCTACATGAATCGGCGTATCCCATTCTATCTCCTTATTCGTAAGAAGTTTGTTGGGAAG
TP19268_Query	D+G	1	scaffold	.	CAGCATCTACATGAATCGGCGTATCCCATTCTATCTCCTTATTCGTAAAAAGTTTGTTGGGAAG
TP19844_Hit	D	1	scaffold	.	CAGCATGAGATTCAATTTCTATGGTTATTGTTATCTCTTCATTTCCACCATGCAGATAATCTTG
TP19844_Query	D	1	scaffold	.	CAGCATGAGATTCAATTTCTATGGTTATTGTCATCTCTTCATTTCCACCATGCAGATAATCTTG
TP20713_Hit	D	1	scaffold	.	CAGCATGTGCCATTCCAGCAAAGATAGGAATTATATCAGATAGTTGTGACAATAAGAACGAACA
TP20713_Query	D	1	scaffold	.	CAGCATGTGCCATTCCAGCAAAGATAGGAATTATATCAGATAGTTGTGACAATAAGAACAAACA
TP2109_Hit	D	1	scaffold	.	CAGCAAATATATTGCTAGATGATTATTGTGAGGCTGTAGTAGGAGACTTTGGATTAGCAAAATT
TP2109_Query	D	1	scaffold	.	CAGCAAATATATTGCTAGATGATTATTGTGAGGCTGTAGTAGGAGACTTTGGATTAGCAAAACT
TP21280_Hit	D	1	scaffold	.	CAGCATTCAAGAATCTTGAGGATCCTAACAATGTTATCAGTAAGGCACACTTTTCGCATAAAGA
TP21280_Query	D	1	scaffold	.	CAGCATTCAAGAATCTTGACGATCCTAACAATGTTATCAGTAAGGCACACTTTTCGCATAAAGA
TP23169_Hit	D	1	scaffold	.	CAGCCAAATTAATGTTGTTCAACCAACATCTACAGACTAACAACCTTGCTTGCAAAGTCTTCCG
TP23169_Query	D	1	scaffold	.	CAGCCAAATTAATGTTGTTCAACCAACATCTACAGACTAACAACCTTACTTGCAAAGTCTTCCG
TP23264_Hit	D	1	scaffold	.	CAGCCAACAATTTCCTCATAAATCGTACTACCTACCAATTTCACAAATGATGGACGTTACATCT
TP23264_Query	D	1	scaffold	.	CAGCCAACAATTTCCTCATAAATCATACTACCTACCAATTTCACAAATGATGGACGTTACATCT
TP23980_Hit	D	1	scaffold	.	CAGCCAATGAACGGGGCACAATTTCAGGAGGAATGCCAAATATATCATTTCCACCGATCTCCTT
TP23980_Query	D	1	scaffold	.	CAGCCAATGAACGGGGCACAATTTCAGAAGGAATGCCAAATATATCATTTCCACCGATCTCCTT
TP24616_Hit	D	1	scaffold	.	CAGCCACATGTATAAGTTCACTCATAGTATTTCATTTAGATCATAAAAAGTCACCTTTTATAGC
TP24616_Query	D	1	scaffold	.	CAGCCACATGTATAAGTTCACTCAAAGTATTTCATTTAGATCATAAAAAGTCACCTTTTATAGC
TP2482_Hit	D	1	scaffold	.	CAGCAAATTCAGATAAAAATATTAGTTCTCAAGAGCACAAACAATATGATAAATTTTTTGTTCC
TP2482_Query	D	1	scaffold	.	CAGCAAATTCAGATAAAAATATTACTTCTCAAGAGCACAAACAATATGATAAATTTTTTGTTCC
TP26691_Hit	D	1	scaffold	.	CAGCCGTCGCAAGCAACCTGCCGCTGTGAGCACAACAAAAGTTTATCAGCAAGAGACTTTTCAT
TP26691_Query	D	1	scaffold	.	CAGCCATCGCAAGCAACCTGCCGCTGTGAGCACAACAAAAGTTTATCAGCAAGAGACTTTTCAT
TP27388_Hit	D	1	scaffold	.	CAGCCATTGATAGGGTCATTGGTGGTTTAGAGAAGAAGAACATGGTAATAAGTAAGCAACAAAG
TP27388_Query	D	1	scaffold	.	CAGCCATTGATAGGGTCATTGGCGGTTTAGAGAAGAAGAACATGGTAATAAGTAAGCAACAAAG
TP29421_Hit	D	1	scaffold	.	CAGCCTTATCTTCTATCCTTAAGCTTAATGGGGATGATATAATTGGTGATTCCATATTGTTTCT
TP29421_Query	D	1	scaffold	.	CAGCCCTATCTTCTATCCTTAAGCTTAATGGGGATGATATAATTGGTGATTCCATATTGTTTCT
TP29576_Hit	D	1	scaffold	.	CAGCCCTGATGTTCTCTATATAATCCTGCACCAAGAAATTAAAATCTGGCAATTACAGTAGTTT
TP29576_Query	D	1	scaffold	.	CAGCCCTGATGTTCTCTATATAATCCTGCAACAAGAAATTAAAATCTGGCAATTACAGTAGTTT
TP30883_Hit	D+G	1	scaffold	.	CAGCCGGAAGCATGGCGAGCTTCATGGCTAAGCCGAAGCAGAAAAGCCTCAGATAGGAGCTAAA
TP30883_Query	D+G	1	scaffold	.	CAGCCGGAAGCAGGGCGAGCTTCATGGCTAAGCCGAAGCAGAAAAGCCTCAGATAGGAGCTAAA
TP32477_Hit	D	1	scaffold	.	CAGCCTCATAAAGTATTTCATTATTTGGCTGACAACTAAAACTGAATATTTTATTAGAATCATT
TP32477_Query	D	1	scaffold	.	CAGCCTCATAAAGTAGTTCATTATTTGGCTGACAACTAAAACTGAATATTTTATTAGAATCATT
TP33211_Hit	D	1	scaffold	.	CAGCCTCTTTGAGAAAAATGGTGCAGAGCACCAGCACTCTGAGACAAGCCTCTCGAATCATTGG
TP33211_Query	D	1	scaffold	.	CAGCCTCTTTGAGAAAAATGGTGCAGAGCACCAACACTCTGAGACAAGCCTCTCGAATCATTGG
TP34332_Hit	D	1	scaffold	.	CAGCCTTCATGCACCTGCAACAGTTGATACTAAACAACCATAGATGGCGCCACCTCCATTACAT
TP34332_Query	D	1	scaffold	.	CAGCCTTCATGCACCTGCAACAGTTGATACTAAACAACCATAGATGGAGCCACCTCCATTACAT
TP34515_Hit	D	1	scaffold	.	CAGCCTTGAAAAGCTGATCAATTATAGCCTGCTGAAACTGATGTGCCGACACATCTTCGCCGCC
TP34515_Query	D	1	scaffold	.	CAGCCTTGAAAAGCTGATCAATTATAGCCTGCTGAAACTGATGCGCCGACACATCTTCGCCGCC
TP34548_Hit	D	1	scaffold	.	CAGCCTTGAATCCCTTCTCATACATGCCCTTGTCTAAATAATTCTTCCCAAAAACCATTCTACA
TP34548_Query	D	1	scaffold	.	CAGCCTTGAATCCCTTCTCATACATGCCCTTGTCCAAATAATTCTTCCCAAAAACCATTCTACA
TP35634_Hit	D	1	scaffold	.	CAGCGAATTGCGGAGGAATACCAGGGAAGAAACAGTGTTCCTCTGGACCGTGCAATTGACAAAG
TP35634_Query	D	1	scaffold	.	CAGCGAATTGCGGAGGAATACCAGAGAAGAAACAGTGTTCCTCTGGACCGTGCAATTGACAAAG
TP36818_Hit	D	1	scaffold	.	CAGCGATTCGGAAGTAGCGAATCGTCTTCTTTGCAGTTTTTCAGGGGAGTGGCTATCAAGCCCA
TP36818_Query	D	1	scaffold	.	CAGCGATTCGGAAGTAGCGAATCGTCTTCTTTGCAGTTTTTCAGGGGAGGGGCTATCAAGCCCA
TP36863_Hit	D	1	scaffold	.	CAGCGATTGGCCACCGGAACCCTAACATACCCACCGTCGGAGCTTCCATCTCTTCCTGTTGATC
TP36863_Query	D	1	scaffold	.	CAGCGATTGGCCACCGGAACCCTAACATACCCACCGCCGGAGCTTCCATCTCTTCCTGTTGATC
TP37304_Hit	D+G	1	scaffold	.	CAGCGCCAGAGTGGCCATATATTTAGGGAGAGAAAATATCTGTATCAAAGAGCCCCTAACAGCT
TP37304_Query	D+G	1	scaffold	.	CAGCGCCAAAGTGGCCATATATTTAGGGAGAGAAAATATCTGTATCAAAGAGCCCCTAACAGCT
TP37449_Hit	D	1	scaffold	.	CAGCGCCCATTCGCTTCGCGCGCGGGAAGGCTACGAAGTTCGGTTCATGAGACCGCTGAAAAAA
TP37449_Query	D	1	scaffold	.	CAGCGCCCATTCGCTTCGCGCGCGGGAAGGCTACGAAGTTCGGTTCATGAGACCGCAGAAAAAA

Name	Filter	Nb hit (Mt4.0)	Mt Chr (Mt4.0)	Ms Chr (Li et al., 2014)	Sequence
TP37993_Hit	D	1	scaffold	.	CAGCGCGTGTGCTTTCTTACGTCTCAGTTTGATAATAGTTAATTGAGCAATTATTTTAACTTCG
TP37993_Query	D	1	scaffold	.	CAGCGCGTGTGCTTTCTTACGTCTCAGTTTGATAATAGTTAATTGAGCAATTATTTTAACTTCA
TP38180_Hit	D+G	1	scaffold	.	CAGCGCTGATAGCTTGTAGTGAACAGCCCCCTTCTTGACCAACCGAAAGCTGAAAAAAAAAAAA
TP38180_Query	D+G	1	scaffold	.	CAGCGCTGATAGCTTGTAGTGAACAGCCCCCTTCTTGACCAACCGAAAGCAGAAAAAAAAAAAA
TP38223_Hit	D	1	scaffold	.	CAGCGCTGTCTTAGATTGAACGTCGACTTATCTTATTGCCGTTCAATCTCTAAGGCGGGTTTAC
TP38223_Query	D	1	scaffold	.	CAGCGCTGTCTTAGATTGAACGTCGACTTATCTTATTGCCGTTCAATCTCTAAGGCGGGGTTAC
TP38310_Hit	D	1	scaffold	.	CAGCGCTTGGGATGATTTTAGGTGCGGCGTATTCCCTTTGGCTATATAATCGTGTGGTTTCTGG
TP38310_Query	D	1	scaffold	.	CAGCGCTTGGGATGATTTTAGGCGCGGCGTATTCCCTTTGGCTATATAATCGTGTGGTTTCTGG
TP38370_Hit	D	1	scaffold	.	CAGCGGAAAATGTGACAGTTGCAGAATCAGGTTGTTGGTCTGGTTTGAGTTTGAAGTGGAATTT
TP38370_Query	D	1	scaffold	.	CAGCGGAAAATGTGACAGTTGCAGAATCAGGTTGTTGGTCTGGTTTGAGTTTGAAGTAGAATTT
TP38490_Hit	D	1	scaffold	.	CAGCGGACAAGGATATCAGTGGCGCAGAAAAAGGGCCAGCTTGCAGGGTTGTATAATTGAATCA
TP38490_Query	D	1	scaffold	.	CAGCGGACAAGGATATCAATGGCGCAGAAAAAGGGCCAGCTTGCAGGGTTGTATAATTGAATCA
TP38564_Hit	D	1	scaffold	.	CAGCGGAGAATGCTACAAAAGAGATGAATCAGTTGAAGGGTGAATATGAAAAGTGGAAATCCAA
TP38564_Query	D	1	scaffold	.	CAGCGGAGAATGCTACAAAAGAGATGAATCAGTTGAAGGGTGAATATGAAAACTGGAAATCCAA
TP39931_Hit	D	1	scaffold	.	CAGCGGTGGTGGCAAGGCCGGATGAGTTTTGGGGGGAGACGCCGTGTGCGTTTGTGAGTTTGAA
TP39931_Query	D	1	scaffold	.	CAGCGGTGGTGGCAAGGCCAGATGAGTTTTGGGGGGAGACGCCGTGTGCGTTTGTGAGTTTGAA
TP39981_Hit	D+G	1	scaffold	.	CAGCGGTTAATTAGATGGCTGAACCATTATATAAGAGTTTAGTCTTAAACATTAACCGAGACTA
TP39981_Query	D+G	1	scaffold	.	CAGCGGTTAATTAGATGGCGGAACCATTATATAAGAGTTTAGTCTTAAACATTAACCGAGACTA
TP4005_Hit	D	1	scaffold	.	CAGCAACCATAGCAGTCAGAGCATTCCTCTCCTGCGCCAATGCGTTGACCCCAATTTCACTTTC
TP4005_Query	D	1	scaffold	.	CAGCAACCATAGCAGTCAGAGCATCCCTCTCCTGCGCCAATGCGTTGACCCCAATTTCACTTTC
TP41330_Hit	D	1	scaffold	.	CAGCGTTCCCTTCTTTTCGGCGGCATCCTTCCGCATTGGCGGCGAGTGGAGTGCCACAATCCCA
TP41330_Query	D	1	scaffold	.	CAGCGTTCCCTTCTTTTCGGCGGCATCCTTCCGCATTGGCGGCGAGTGGAGGGCCACAATCCCA
TP43915_Hit	D	1	scaffold	.	CAGCTGGATCTTTGATCTTTGAATGCTAGTAAACAATAAATCAAAAGTTTTATTAATTTTTAAA
TP43915_Query	D	1	scaffold	.	CAGCTAGATCTTTGATCTTTGAATGCTAGTAAACAATAAATCAAAAGTTTTATTAATTTTTAAA
TP43997_Hit	D	1	scaffold	.	CAGCTAGCACTTTGTCGTTTCTCTTTCTCTTTCTCTTGTCACAATCTATGCAATGAAGCCCATC
TP43997_Query	D	1	scaffold	.	CAGCTAGCACTTTGGCGTTTCTCTTTCTCTTTCTCTTGTCACAATCTATGCAATGAAGCCCATC
TP44030_Hit	D+G	1	scaffold	.	CAGCTAGCATTGATGCTTGTCCTAGAATTGAAGGAGCATCCAGTATACGAGAAACTTATAAATA
TP44030_Query	D+G	1	scaffold	.	CAGCTAGCATTGATGCTAGTCCTAGAATTGAAGGAGCATCCAGTATACGAGAAACTTATAAATA
TP44294_Hit	D	1	scaffold	.	CAGCTAGTAGTTCCTTGAAGGGTCAAAGTGACAAGGAAAAGTACGAGAAGGCTAGGTTGTTGAA
TP44294_Query	D	1	scaffold	.	CAGCTAGTAGTTCCTTGAAGGGTCAAAGTGACAAGGAAAAATACGAGAAGGCTAGGTTGTTGAA
TP44482_Hit	D	1	scaffold	.	CAGCTATAAAGGATTCTACTGAAAAAATGCATCAGGTGATTTCAATTGTTTCCAACTAAATGCT
TP44482_Query	D	1	scaffold	.	CAGCTATAAAGGATTCTACTGAAAAAATCCATCAGGTGATTTCAATTGTTTCCAACTAAATGCT
TP44935_Hit	D	1	scaffold	.	CAGCTATCGACTACAAAGCCAGCATATTATTTGTATAGGAGAATTTAATCCCCCTATGAAGTTA
TP44935_Query	D	1	scaffold	.	CAGCTATCGACTACAAAGCCAGCATATTATTTGTATAGGAGAATTTAACCCCCCTATGAAGTTA
TP45451_Hit	D	1	scaffold	.	CAGCTATTGATGGAAAATTTCTATCTAGTGAGACATCGGGATCACTGTCCACTTTATTTGATGT
TP45451_Query	D	1	scaffold	.	CAGCTATTGATGGAAAATTTCTATCTAGTGAGACATCGGGATCACTGTCAACTTTATTTGATGT
TP45498_Hit	D+G	1	scaffold	.	CAGCTATTGGGTCTATTTTCTTTGGAATTACAACTAGAGATGACTTGGATGACGACGGTGTGAT
TP45498_Query	D+G	1	scaffold	.	CAGCTATTGGGTCTATTTTCTTTGGAATTACAACTAGAGATGACTTAGATGACGACGGTGTGAT
TP46117_Hit	D+G	1	scaffold	.	CAGCTCACAAACATAATTGATGAAGAACTAACGTGGCATCATTTCAAATTTTGGCTGATGTATA
TP46117_Query	D+G	1	scaffold	.	CAGCTCACAAACATAATTGATGAAGAACTAACGTGGCATCATTTCAAATTTTCGCTGATGTATA
TP47333_Hit	D	1	scaffold	.	CAGCTCCATTCATTACCAGTTCAACTTCTCACAAACCAAGCAGACTAATTCCATTACAAGAAAC
TP47333_Query	D	1	scaffold	.	CAGCTCCATTCATTACCAATTCAACTTCTCACAAACCAAGCAGACTAATTCCATTACAAGAAAC
TP4746_Hit	D	1	scaffold	.	CAGCAATTGCGGTGGCCCAGCAATTCCTGGACAATTTTCACCGGAGGACCACCAGCTTAGGGTT
TP4746_Query	D	1	scaffold	.	CAGCAACTGCGGTGGCCCAGCAATTCCTGGACAATTTTCACCGGAGGACCACCAGCTTAGGGTT
TP47551_Hit	D+G	1	scaffold	.	CAGCTCTGACTCCCGGTTTTTTAATTGTTGAAGAGTTTCTTCCAAATTAGTTTGAGCTTCACTA
TP47551_Query	D+G	1	scaffold	.	CAGCTCCGACTCCCGGTTTTTTAATTGTTGAAGAGTTTCTTCCAAATTAGTTTGAGCTTCACTA
TP4815_Hit	D	1	scaffold	.	CAGCAACTGTTTCAAAACAAAAGGAAATTTAACAGTGGGAACAGCAACTACACATTGCTATGCA
TP4815_Query	D	1	scaffold	.	CAGCAACTGTTTCAAAACAAAAGGAAATTTAACAGTGGGAACAGCAACAACACATTGCTATGCA
TP49723_Hit	D+G	1	scaffold	.	CAGCTGAAACAGAATACTCAAATTGTTTCTTGTAATGGAATTAGTCTGCTTGGTTTGTGAGAAG
TP49723_Query	D+G	1	scaffold	.	CAGCTGAAACAAAATACTCAAATTGTTTCTTGTAATGGAATTAGTCTGCTTGGTTTGTGAGAAG
TP50042_Hit	D	1	scaffold	.	CAGCTGAAGCGGCCGCTATTTGATTCCGCGCCGCTACGACGATTAGTGTAGTGGTTCGTCGCCG
TP50042_Query	D	1	scaffold	.	CAGCTGAAGCGGCCGCTATTTGATTCCGCGCCGCTACGACGATTAGTGTAGCGGTTCGTCGCCG
TP50653_Hit	D	1	scaffold	.	CAGCTGAGTTTTCCAATTCAGCTCATATAACTACCGGTATTATTAGCATTACAGACATTACTTA
TP50653_Query	D	1	scaffold	.	CAGCTGAGTTTTCCAATTCAGCTCATATAACTACCGGTATCATTAGCATTACAGACATTACTTA

Name	Filter	Nb hit (Mt4.0)	Mt Chr (Mt4.0)	Ms Chr (Li et al., 2014)	Sequence
TP50654_Hit	D	1	scaffold	.	CAGCTGAGTTTTCCAATTCAGCTCATATAACTACTGGTGTGATCACCATTACAGACATTACTTA
TP50654_Query	D	1	scaffold	.	CAGCTGAGTTTTCCAATTCAGCTCATATAACTACCGGTGTGATCACCATTACAGACATTACTTA
TP50753_Hit	D	1	scaffold	.	CAGCTGATCAGAAAAGGGTCACGTTTTTAGCATCAGATTGTTTACCTTTAGTTTACGATTCAGC
TP50753_Query	D	1	scaffold	.	CAGCTGATCAGAAAAGGGTCACGTTTTTAGCATCACATTGTTTACCTTTAGTTTACGATTCAGC
TP51988_Hit	D+G	1	scaffold	.	CAGCTGGTTGTCAGGAAATGCCTCGGAATAAGATAGATGGGAAGTTGGAAGATACATACAGTTC
TP51988_Query	D+G	1	scaffold	.	CAGCTGGTTGTCAGGAAATGCCTCGGAATAAGATAGATGGGAAGTTGGAAGATACAGACAGTTC
TP52823_Hit	D	1	scaffold	.	CAGCTGTTATGGCTCTAGCATTTAAGCGGTCAGAAATGTGGCCTGCTAGAATTCCTCCGAGCAC
TP52823_Query	D	1	scaffold	.	CAGCTGTTATGGCTCTAGCATTTAAGCGGTCAGAAATGTGGCCTGCTAGAATTCCTCCAAGCAC
TP57521_Hit	D	1	scaffold	.	CAGCTTGCTACTGAACCTGCTCTGGTGCTACTGCATTAGTTGTCTGCACAGAATTTACATCCAG
TP57521_Query	D	1	scaffold	.	CAGCTTGCTACTGAACCTGCTCTGGCGCTACTGCATTAGTTGTCTGCACAGAATTTACATCCAG
TP57798_Hit	D	1	scaffold	.	CAGCTTGGGCTGATGTAACAATGAAATCGTCAGGGTTTGACACACCTCGGAAATTTTTTGTGGG
TP57798_Query	D	1	scaffold	.	CAGCTTGGGCTGATGTAACAATGAAATCGTCAGGGTTTGACACACCTCGGAAATTTTTTGTGGA
TP58666_Hit	D	1	scaffold	.	CAGCTTTAGACTATACTCGTAGGCTTGTAGCTTTCTAAACAAGCAATAAACAATAGTAAGGGAG
TP58666_Query	D	1	scaffold	.	CAGCTTTAGACTATACTCGTAGGCTTGTAGCTTTCTAAACAAGCAATAAACAATAGTAAGGAAG
TP59348_Hit	D	1	scaffold	.	CAGCTTTGAATTCCCATGGTGTAATTGGGCCTCTCATTATCATATCGGCATCAAGAATCACTAT
TP59348_Query	D	1	scaffold	.	CAGCTTTGAATTCCCATGGTGTAATTGGGCCTCTCATTATCATATCAGCATCAAGAATCACTAT
TP60271_Hit	D+G	1	scaffold	.	CAGCTTTTGTTGTTGATGCCAAACGCCGCCTTTAAGGAGTCGTTGTCACCACCTACTGCTGAAA
TP60271_Query	D+G	1	scaffold	.	CAGCTTTTGTTGTTGATGCCAAACGCCGCCTTTAAGGAGTCGTTGTCACCACCTACTGCAGAAA
TP60902_Hit	D+G	1	scaffold	.	CTGCAAAATGAAGAGGTGAATTAAGACCACCAAAAGTTGAGTATTTTGCAAGACAGGGATTGAA
TP60902_Query	D+G	1	scaffold	.	CTGCAAAATGAAGAGGTGAATTAAGACCACCAAAAGTTGAGTATTTTGCAAGACAAGGATTGAA
TP61281_Hit	D+G	1	scaffold	.	CTGCAAAGATTATTTCTTATTTAGCATTCTTTGAATGGTGCAGAGAAAATGTCAACCAGTGTGA
TP61281_Query	D+G	1	scaffold	.	CTGCAAAGATGATTTCTTATTTAGCATTCTTTGAATGGTGCAGAGAAAATGTCAACCAGTGTGA
TP63957_Hit	D	1	scaffold	.	CTGCAATATGCAAATCAAATACAATTTGTGAGCTATTAACATACAGCTTTCACGGTCTTCTACA
TP63957_Query	D	1	scaffold	.	CTGCAATATGCAAATCAAATACAATTAGTGAGCTATTAACATACAGCTTTCACGGTCTTCTACA
TP67226_Hit	D+G	1	scaffold	.	CTGCACTTGGATTCATGTCACCGGCCTCCCGTGGAACACTTATCACAGGTATGCTGTTTTTCTA
TP67226_Query	D+G	1	scaffold	.	CTGCACTTGGATTCATGTCACCGGCCTCCCGCGGAACACTTATCACAGGTATGCTGTTTTTCTA
TP6902_Hit	D	1	scaffold	.	CAGCAATACCAAGTATCATGTAGAAAAACAGCATACCTGTGATAAGTGTTCCGCGGGAGGCCGG
TP6902_Query	D	1	scaffold	.	CAGCAATACCAAGTATCATGTAGAAAAACAGCATACCTGTGATAAGTGTTCCACGGGAGGCCGG
TP69447_Hit	D	1	scaffold	.	CTGCAGGTGTCACCGGAAGACCCAATGTTGATTGGTGCGAAATTCATAAAACAGAAACCATCCG
TP69447_Query	D	1	scaffold	.	CTGCAGGTGTCACCGGAAGACCCAATGTTGATTGGTGCGAAACTCATAAAACAGAAACCATCCG
TP7040_Hit	D	1	scaffold	.	CAGCAATAGTATTATACTTTTGCAAGATTAATAGGTTGCAAAGCCAAGTGATATTTGGTTTTGT
TP7040_Query	D	1	scaffold	.	CAGCAATAGTATTATACTTTTGCAAGATTAATAGGTTGCAAAGCCAAATGATATTTGGTTTTGT
TP73912_Hit	D+G	1	scaffold	.	CTGCCAATGCGGAAGGATGCCGCCGAAAATAAGGGAACGCTGAAAAAAAAAAAAAAAAAAAAAA
TP73912_Query	D+G	1	scaffold	.	CTGCCAATGCGGAAGGATGCCGCCGAAAAGAAGGGAACGCTGAAAAAAAAAAAAAAAAAAAAAA
TP75691_Hit	D	1	scaffold	.	CTGCCATTTAAGAGAATAGCTGAACAACTCAACCTGCCCTGACAAAGAAATCTTTTCTCAATTA
TP75691_Query	D	1	scaffold	.	CTGCCATTTAAGAGAATAGCTGAACAACTCAACCTCCCCTGACAAAGAAATCTTTTCTCAATTA
TP80981_Hit	D	1	scaffold	.	CTGCGCGTAGCGTAGCGGCAACGAACCGCTACGGTAATCGTCGTAGCGGCGCGAAATCAAATAG
TP80981_Query	D	1	scaffold	.	CTGCGCATAGCGTAGCGGCAACGAACCGCTACGGTAATCGTCGTAGCGGCGCGAAATCAAATAG
TP81285_Hit	D	1	scaffold	.	CTGCGCCTCATTCACGTTCCCTTCAAGATCACCAATGTACAATGAAGCATTCTCAAATCTTCCA
TP81285_Query	D	1	scaffold	.	CTGCGCCTCATTCACGTTCCCTTCAAGATCACCAACGTACAATGAAGCATTCTCAAATCTTCCA
TP83833_Hit	D	1	scaffold	.	CTGCGTTGAACGGGTATTTCGATTGTGTACAACTGCTACTTGATCTTAATGCAAATGGATCAGC
TP83833_Query	D	1	scaffold	.	CTGCGTTGAACGGGTATTTCGATTGTGTACAACTGCTACTTGATCTTAATGCAAATGCATCAGC
TP85190_Hit	D	1	scaffold	.	CTGCTACTATACTGTATGTGACTCCATTCCAAGTTGTGGCCCTTCTGACCGGAATTTATGTGTT
TP85190_Query	D	1	scaffold	.	CTGCTACTATACTGTATGTGACTCCATTCCAAGTTGTAGCCCTTCTGACCGGAATTTATGTGTT
TP91531_Hit	D	1	scaffold	.	CTGCTGTATCACCCAACCAAACATCTGGTGCCACCTCTCCCTCTAAAGTCTCATATGATGGACG
TP91531_Query	D	1	scaffold	.	CTGCTGTATCACCCAACCAAACATCTGGTGCCACCTCTCCCTCAAAAGTCTCATATGATGGACG
TP92326_Hit	D	1	scaffold	.	CTGCTGTTGATCAGTATGAAGTGGAGCATACGAGTAAGCCTGACATCGTGGACACATCCCAAGG
TP92326_Query	D	1	scaffold	.	CTGCTGTTGACCAGTATGAAGTGGAGCATACGAGTAAGCCTGACATCGTGGACACATCCCAAGG
TP9858_Hit	D+G	1	scaffold	.	CAGCACATGTAAGTGCTTCTTCCCCTGAACATGCCATGAATTCCATACATCCATCAACAGCATA
TP9858_Query	D+G	1	scaffold	.	CAGCACATGTAAGTGCTTCTTCCCCTGAACATGCCATGAATTCCATACAGCCATCAACAGCATA
TP1069_Hit	D	1	scaffold	1A	CAGCAAAATTTGGTCCCTAAGACTAATGTTTTGCTTTTGCAGACTTTGTTTTTTGCAAACAAAG
TP1069_Query	D	1	scaffold	1A	CAGCAAAATTTGGTCCCTAAGACCAATGTTTTGCTTTTGCAGACTTTGTTTTTTGCAAACAAAG
TP32489_Hit	D	1	scaffold	4A	CAGCCTCATATTCATTCCACTTAATTTTCAGTCACTATGCACATGCAATGTCAAGTTGTACTTA
TP32489_Query	D	1	scaffold	4A	CAGCCTCATATTCATACCACTTAATTTTCAGTCACTATGCACATGCAATGTCAAGTTGTACTTA

Name	Filter	Nb hit (Mt4.0)	Mt Chr (Mt4.0)	Ms Chr (Li et al., 2014)	Sequence
TP4921_Hit	D	1	scaffold	5A	CAGCGACTTGGTCAATAACTTGAGGTGGTTTCACACAACCACATACCCTAGGCTCAGCTGGTTC
TP4921_Query	D	1	scaffold	5A	CAGCAACTTGGTCAATAACTTGAGGTGGTTTCACACAACCACATACCCTAGGCTCAGCTGGTTC
TP78643_Hit	D	1	scaffold	5B	CTGCGTCTAGCGGAACCTCTGAGCAAGCATTTGCTCTAATTACTGATGGAAAATCACTTGCTTA
TP78643_Query	D	1	scaffold	5B	CTGCCTCTAGCGGAACCTCTGAGCAAGCATTTGCTCTAATTACTGATGGAAAATCACTTGCTTA
