# Supplementary figures and images for: Validation of Genotyping-By-Sequencing Analysis in Populations of Tetraploid Alfalfa by 454 Sequencing
Source: PLoS One. 2015 Jun 26;10(6):e0131918. doi: 10.1371/journal.pone.0131918 (PMC4482585; doi:10.1371/journal.pone.0131918)

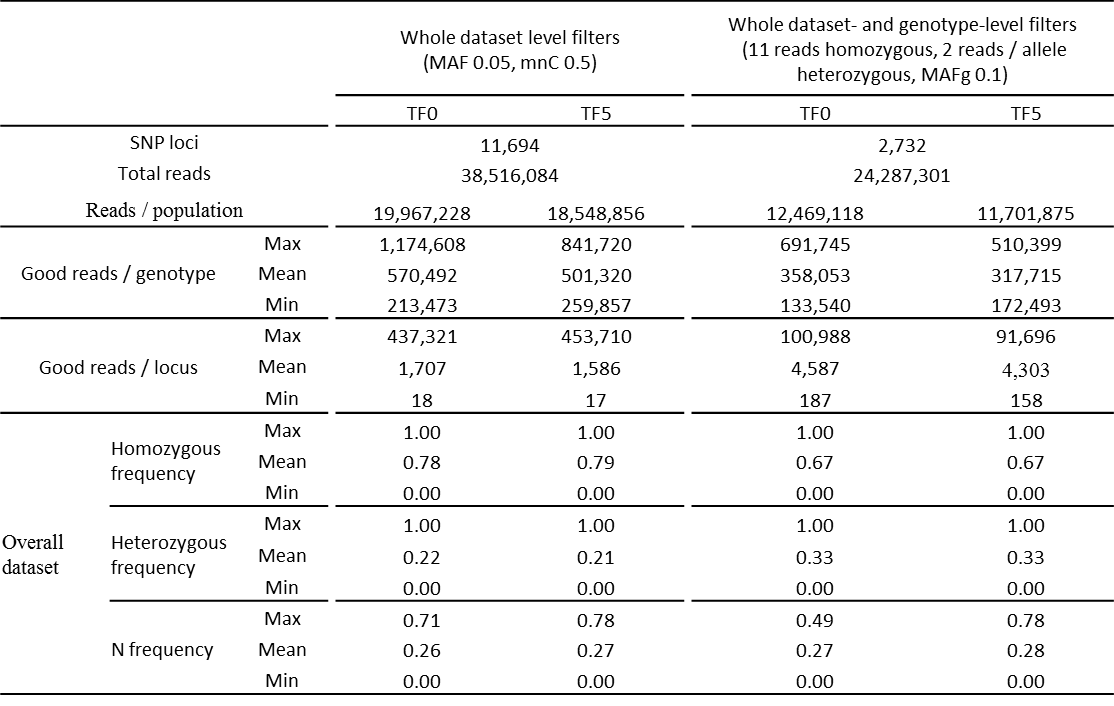

Supplement: S2 Table — The total number of good reads in ATF0 and ATF5 populations, and the maximum, mean and minimum counts of good reads per sample and SNP loci are reported. Homozygote, heterozygotes and missing genotypes frequencies were calculated for each population. (TIF) [file pone.0131918.s005.tif]
