# Supplementary material for: Validation of Genotyping-By-Sequencing Analysis in Populations of Tetraploid Alfalfa by 454 Sequencing
Source: PLoS One. 2015 Jun 26;10(6):e0131918. doi: 10.1371/journal.pone.0131918 (PMC4482585; doi:10.1371/journal.pone.0131918)
Supplement: S3 Table — Haplotypes defined with 454 sequences with perfect and imperfect match with GBS 64bp sequence are listed separately. Position of SNPs is based on location on M. truncatula reference sequence. SNPs included in UNEAK TP are highlighted in bold. RC of GBS alleles (A1 and A2) and 454 sequences covering each haplotype in the eight genotyped plant samples are indicated. Cumulative number of A1-like, A2-like and total reads with perfect or imperfect match is also presented. Haplotypes with frequency <5% in all individual plant samples are not indicated but total read counts supporting those other haplotypes are reported. (PDF) [file pone.0131918.s006.pdf]

TP67636

Haplotypes with perfect match with GBS TP

		Position	26	**74**	167	275	276	316	336	368	369	370	371	373	376	377	378	379	380	381	383	384	385	386	387	388	389	390	392	393	394	395	398
	Mt sequence		T	**T**	A	G	G	G	C	T	G	G	T	C	G	A	A	A	G	C	C	C	C	A	T	T	C	C	C	C	T	T	A
	Ms Sanger consensus		A	**N**	A	A	G	G	C	T	G	G	T	C	G	A	A	A	G	C	C	C	C	A	T	T	T	C	C	C	T	T	A
GBS	A1		-	**T**	-	-	-	-	-	-	-	-	-	-	-	-	-	-	-	-	-	-	-	-	-	-	-	-	-	-	-	-	-
GBS	A2		-	**C**	-	-	-	-	-	-	-	-	-	-	-	-	-	-	-	-	-	-	-	-	-	-	-	-	-	-	-	-	-
454	Haplotype 1	A1-like	T	**T**	A	A	G	G	C	T	G	G	T	C	G	A	A	A	G	C	C	C	C	A	T	T	T	C	C	C	T	T	A
454	Haplotype 2	A1-like	T	**T**	A	A	G	G	T	T	G	G	T	C	G	A	A	A	G	C	C	C	C	A	T	T	T	C	C	C	T	T	A
454	Haplotype 3	A1-like	T	**T**	A	G	C	G	C	C	C	C	A	T	C	C	C	T	T	T	A	G	A	G	A	C	A	T	A	A	A	A	G
454	Haplotype 4	A1-like	T	**T**	A	A	G	A	C	T	G	G	T	C	G	A	A	A	G	C	C	C	C	A	T	T	T	C	C	C	T	T	A
454	Haplotype 5	A1-like	T	**T**	G	A	G	G	C	T	G	G	T	C	G	A	A	A	G	C	C	C	C	A	T	T	T	C	C	C	T	T	A
454	Haplotype 6	A2-like	A	**C**	A	A	G	G	C	T	G	G	T	C	G	A	A	A	G	C	C	C	C	A	T	T	T	C	C	C	T	T	A
454	Haplotype 7	A2-like	A	**C**	A	A	G	G	T	T	G	G	T	C	G	A	A	A	G	C	C	C	C	A	T	T	T	C	C	C	T	T	A
454	TP67636	A1-like		**T**																													
454	TP67636	A2-like		**C**																													
454	TP67636	Other (<5%) A1		**T**																													
454	TP67636	Other (<5%) A2		**C**																													

			Read counts							
			TF0-17	TF0-20	TF0-36	TF0-38	TF5-5	TF5-12	TF5-20	TF5-28
GBS	A1		4	5	25	1	40	9	6	3
GBS	A2		0	110	49	37	175	0	224	94
454	Haplotype 1	A1-like	314	132	200	33	81	370	265	183
454	Haplotype 2	A1-like	0	96	3	100	358	0	196	6
454	Haplotype 3	A1-like	0	0	69	63	0	78	0	0
454	Haplotype 4	A1-like	1	0	0	0	0	154	1	1
454	Haplotype 5	A1-like	1	1	97	3	0	1	0	0
454	Haplotype 6	A2-like	158	38	39	28	125	0	183	471
454	Haplotype 7	A2-like	0	13	3	15	91	0	56	7
454	TP67636	A1-like	316	229	369	199	439	603	462	190
454	TP67636	A2-like	158	51	42	43	216	0	239	478
454	TP67636	Other (<5%) A1	60	116	134	154	74	144	99	26
454	TP67636	Other (<5%) A2	40	53	59	62	72	5	86	103

Haplotypes with imperfect match with GBS TP

		26	74	167	275	276	316	336	368	369	370	371	373	376	377	378	379	380	381	383	384	385	386	387	388	389	390	392	393	394	395	398
454	Haplotype 1[b]	T	**T**	A	A	G	G	C	T	G	G	T	C	G	A	A	A	G	C	C	C	C	A	T	T	T	C	C	C	T	T	A
454	Haplotype 2[b]	T	**T**	A	A	G	G	T	T	G	G	T	C	G	A	A	A	G	C	C	C	C	A	T	T	T	C	C	C	T	T	A
454	Haplotype 3[b]	T	**T**	A	G	C	G	C	C	C	C	A	T	C	C	C	T	T	T	A	G	A	G	A	C	A	T	A	A	A	A	G
454	Haplotype 4[b]	T	**T**	A	A	G	A	C	T	G	G	T	C	G	A	A	A	G	C	C	C	C	A	T	T	T	C	C	C	T	T	A
454	Haplotype 5[b]	T	**T**	G	A	G	G	C	T	G	G	T	C	G	A	A	A	G	C	C	C	C	A	T	T	T	C	C	C	T	T	A
454	Haplotype 6[b]	A	**C**	A	A	G	G	C	T	G	G	T	C	G	A	A	A	G	C	C	C	C	A	T	T	T	C	C	C	T	T	A
454	Haplotype 8[a]	T	**T**	A	A	G	G	C	T	G	G	T	C	G	A	A	A	G	C	C	T	C	A	T	T	T	C	C	C	T	T	A

		TF0-17	TF0-20	TF0-36	TF0-38	TF5-5	TF5-12	TF5-20	TF5-28
454	Haplotype 1[b]	413	213	238	52	107	375	315	311
454	Haplotype 2[b]	1	134	4	165	428	0	245	13
454	Haplotype 3[b]	0	0	75	83	0	74	0	0
454	Haplotype 4[b]	0	0	0	0	0	105	0	2
454	Haplotype 5[b]	0	0	83	2	0	0	0	0
454	Haplotype 6[b]	28	21	10	0	14	0	32	66
454	Haplotype 8[a]	1	132	0	161	1	0	0	0
	Total	443	500	410	463	550	554	592	392
	Other (<5%)	98	429	244	557	157	235	128	99

[a] contains a 3bp (TCT) indel within GBS TP in position 360

[b] Haplotype containing GBS allele with sequencing errors in the 64 bp of the GBS TP

TP7278

Haplotypes with perfect match with GBS TP

		25	26	32	35	41	47	53[a]	80[a]	92	95	125	128	131[a]	**137**	**140**	**145**	**146**
	Mt sequence	T	T	C	T	T	C	C	C	G	C	C	C	G	A	A	A	T
	Ms Sanger consensus	T	T	T	T	T	C	C	C	R	T	C	C	G	A	A	A	C
GBS	A1	-	-	-	-	-	-	-	-	-	-	-	-	-	-	-	-	C
GBS	A2	-	-	-	-	-	-	-	-	-	-	-	-	-	-	-	-	T
454	Haplotype 1 A1-like	T	T	C	C	T	T	C	T	A	T	C	C	A	A	A	A	C
454	Haplotype 2 A1-like	G	A	T	C	T	C	T	C	A	T	C	C	G	A	A	A	C
454	Haplotype 3 A1-like	T	T	T	T	T	C	C	C	A	T	C	C	G	A	A	A	C
454	Haplotype 4 A1-like	T	T	T	T	T	C	T	A	G	C	C	C	G	A	A	A	C
454	Haplotype 5 A2-like	T	C	T	T	T	C	C	C	A	T	C	C	G	A	A	A	T
TP7278	A1-like																	C
TP7278	A2-like																	T
TP7278	Other (<5%) A1																	C
TP7278	Other (<5%) A2																	T

		**167**	**170**	**185**	**200**	**218**	**221**	**227**	**235**	**239**	**242[a]**	260	261[a]	269	281	287	299	320	323[a]	326	338	341	365	368	383	389	397
	Mt sequence	G	G	G	G	A	G	C	G	T	T	C	G	T	T	T	T	T	T	A	C	T	A	T	G	A	A
	Ms Sanger consensus	G	G	G	G	C	G	C	G	C	N	C	G	T	T	A	T	A	T	A	N	T	A	A	G	C	A
GBS	A1	-	-	-	-	-	-	-	-	-	-	-	-	-	-	-	-	-	-	-	-	-	-	-	-	-	-
GBS	A2	-	-	-	-	-	-	-	-	-	-	-	-	-	-	-	-	-	-	-	-	-	-	-	-	-	-
454	Haplotype 1 A1-like	G	G	G	G	C	G	T	G	C	C	C	T	T	T	A	T	A	T	A	C	T	A	A	G	C	A
454	Haplotype 2 A1-like	G	G	G	G	C	G	C	G	C	T	C	T	T	T	A	T	A	T	A	C	T	A	A	G	C	A
454	Haplotype 3 A1-like	G	G	G	G	C	G	C	G	C	T	C	T	T	T	A	C	T	G	A	T	A	T	T	A	A	G
454	Haplotype 4 A1-like	G	G	G	G	C	G	C	G	A	C	C	T	T	T	A	T	A	T	A	T	T	A	A	G	C	A
454	Haplotype 5 A2-like	G	G	G	G	A	G	T	G	T	T	C	T	T	T	A	T	A	T	A	C	T	A	A	G	C	A

		Read counts: TF0-17	TF0-20	TF0-36	TF0-38	TF5-5	TF5-12	TF5-20	TF5-28
GBS	A1	11	251	500	24	55	133	43	186
GBS	A2	0	0	342	0	1	0	0	0
454	Haplotype 1 A1-like	214	0	0	268	0	247	0	0
454	Haplotype 2 A1-like	0	0	258	5	0	245	0	5
454	Haplotype 3 A1-like	0	35	31	1	80	0	3	43
454	Haplotype 4 A1-like	32	0	0	0	0	0	78	65
454	Haplotype 5 A2-like	0	0	44	0	0	0	0	0
TP7278	A1-like	246	35	289	274	80	492	81	113
TP7278	A2-like	0	0	44	0	0	0	0	0
TP7278	Other (<5%) A1	259	157	298	368	112	503	161	287
TP7278	Other (<5%) A2	2	0	54	3	1	0	0	0

Haplotypes with imperfect match with GBS TP

		25	26	32	35	41	47	53[a]	80[a]	92	95	125	128	131[a]	**137**	**140**	**145**	**146**
454	Haplotype 6	T	C	C	T	C	C	C	C	A	T	T	C	G	G	A	G	C
454	Haplotype 7	G	A	T	C	T	C	T	C	A	T	T	C	G	G	A	A	C
454	Haplotype 8	T	T	C	T	T	C	T	C	A	T	C	C	A	A	G	A	C
454	Haplotype 9	G	A	T	C	T	C	T	C	G	C	C	T	G	A	A	A	C

		**167**	**170**	**185**	**200**	**218**	**221**	**227**	**235**	**239**	**242[a]**	260	261[a]	269	281	287	299	320	323[a]	326	338	341	365	368	383	389	397
454	Haplotype 6	G	G	G	G	T	G	C	T	T	C	C	T	T	T	A	T	T	G	G	T	A	T	T	A	A	G
454	Haplotype 7	G	G	G	G	C	A	C	G	A	C	C	T	T	T	A	T	A	T	A	C	T	A	A	G	C	A
454	Haplotype 8	G	A	A	A	T	G	T	G	C	C	T	G	A	T	A	T	A	T	A	C	T	A	A	G	C	A
454	Haplotype 9	A	G	G	G	C	G	T	G	C	C	T	G	A	G	T	T	A	T	A	T	T	A	A	G	C	A

		Read counts: TF0-17	TF0-20	TF0-36	TF0-38	TF5-5	TF5-12	TF5-20	TF5-28
454	Haplotype 6	0	536	0	0	279	1	1	225
454	Haplotype 7	0	0	0	0	0	0	290	0
454	Haplotype 8	236	0	1	240	256	5	0	0
454	Haplotype 9	0	2	4	0	0	0	221	8
	Total	236	538	5	240	535	6	512	233
	Other (<5%)	405	384	171	471	728	322	670	332

[a] SNP affecting *ApeKI* restriction site

TP80194

Haplotypes with perfect match with GBS TP

		Position	43	81[a]	88	174	188	225	282	348								
		Mt sequence	**T**	**G**	A	C	A	G	T	T	Read counts							
		Ms Sanger consensus	**T**	**G**	A	C	R	G	T	T	TF0-17	TF0-20	TF0-36	TF0-38	TF5-5	TF5-12	TF5-20	TF5-28
GBS		A1	**T**	-	-	-	-	-	-	-	16	33	16	6	7	6	20	2
		A2	**A**	-	-	-	-	-	-	-	0	21	0	0	0	0	0	0
454	Haplotype 1	A1-like	**T**	**G**	A	T	G	G	T	T	215	201	1	0	153	118	129	129
	Haplotype 2	A1-like	**T**	**G**	A	C	G	G	T	A	223	75	64	7	292	32	5	49
	Haplotype 3	A1-like	**T**	**G**	A	C	A	G	T	T	0	37	195	206	2	19	166	4
	Haplotype 4	A1-like	**T**	**G**	G	C	A	G	T	T	1	18	278	17	0	21	113	3
	Haplotype 5	A1-like	**T**	**G**	A	C	G	G	T	T	82	58	54	7	126	25	16	27
	Haplotype 6	A1-like	**T**	**G**	A	C	G	A	T	T	0	2	1	0	157	113	0	90
	Haplotype 7	A1-like	**T**	**G**	A	T	G	G	T	A	82	38	0	0	64	15	2	46
	Haplotype 8	A2-like	**A**	**G**	G	C	A	G	T	T	1	68	1	86	1	80	0	0
	TP80194	A1-like	**T**								603	429	593	237	794	343	431	348
		A2-like	**A**								1	68	1	86	1	80	0	0
		Other (<5%) A1	**T**								83	97	244	102	172	115	199	111
		Other (<5%) A2	**A**								1	80	1	68	1	113	0	0

Haplotypes with imperfect match with GBS TP

											TF0-17	TF0-20	TF0-36	TF0-38	TF5-5	TF5-12	TF5-20	TF5-28
454	Haplotype 2[b]		**T**	**G**	A	C	G	G	T	A	66	22	18	4	64	14	10	15
	Haplotype 3[b]		**T**	**G**	A	C	A	G	T	T	1	10	31	55	7	17	24	5
	Haplotype 4[b]		**T**	**G**	G	C	A	G	T	T	0	1	48	9	0	2	22	12
	Haplotype 9		**T**	**T**	A	C	G	G	T	A	0	0	0	19	5	43	2	60
		Total									67	33	97	87	76	76	58	92
		Other (<5%)									190	179	186	227	235	323	308	324

[a] SNP affecting *ApeKI* restriction site

[b] Haplotype containing GBS allele with sequencing errors in the 64 bp of the GBS TP

TP79240

Haplotypes with perfect match with GBS TP

Position			43	81[a]	88	174	188	225	282	348	Read counts							
Mt sequence			T	G	A	C	A	G	**T**	T								
Ms Sanger consensus			T	G	A	C	R	G	**T**	T	TF0-17	TF0-20	TF0-36	TF0-38	TF5-5	TF5-12	TF5-20	TF5-28
GBS		A1	-	-	-	-	-	-	**T**	-	18	29	6	31	2	49	20	17
		A2	-	-	-	-	-	-	**G**	-	0	0	0	0	0	0	1	0
454	Haplotype 1	A1-like	T	G	A	T	G	G	**T**	T	216	221	1	0	159	110	123	129
	Haplotype 2	A1-like	T	G	A	C	G	G	**T**	A	249	73	67	9	282	29	5	45
	Haplotype 3	A1-like	T	G	A	C	A	G	**T**	T	0	33	182	218	2	23	162	4
	Haplotype 4	A1-like	T	G	G	C	A	G	**T**	T	1	19	265	21	0	18	116	3
	Haplotype 5	A1-like	T	G	A	C	G	G	**T**	T	71	67	53	6	127	21	14	27
	Haplotype 6	A1-like	T	G	A	C	G	A	**T**	T	0	3	1	0	152	115	0	85
	Haplotype 7	A1-like	T	G	A	T	G	G	**T**	A	89	34	0	0	70	18	2	38
	Haplotype 8	A1-like	A	G	G	C	A	G	**T**	T	1	65	1	81	1	82	0	0
	Haplotype 9	A1-like	T	T	A	C	G	G	**T**	A	0	0	0	24	2	56	2	76
	Haplotype 10	A1-like	T	G	G	C	A	G	**T**	A	1	5	50	2	0	4	0	2
	TP79240	A1-like							**T**		628	520	620	361	795	476	424	409
		A2-like							**G**		0	0	0	0	0	0	0	0
		Other (<5%) A1							**T**		82	156	211	226	148	294	189	241
		Other (<5%) A2							**G**		0	1	0	0	0	0	136	1

Haplotypes with imperfect match with GBS TP

454	Haplotype 1[b]	T	G	A	T	G	G	**T**	T	50	29	18	20	30	31	31	24
	Haplotype 2[b]	T	G	A	C	G	G	**T**	A	47	23	15	2	84	20	12	19
	Haplotype 3[b]	T	G	A	C	A	G	**T**	T	1	17	46	44	7	16	25	5
	Haplotype 4[b]	T	G	G	C	A	G	**T**	T	1	1	61	8	0	10	20	28
	Total									99	70	140	74	121	77	88	76
	Other (<5%)									136	139	151	146	215	203	159	148

[a] SNP affecting *ApeKI* restriction site

[b] Haplotype containing GBS allele with sequencing errors in the 64 bp of the GBS TP

TP91313

Haplotypes with perfect match with GBS TP

	Position		**121**	**141**	156	174	190	199	206	208	230	247	253	415[a]	Read counts							
															TF0-17	TF0-20	TF0-36	TF0-38	TF5-5	TF5-12	TF5-20	TF5-28
	Mt sequence		**G**	**C**	T	C	T	G	C	G	G	G	G	C								
	Ms Sanger consensus		**G**	**C**	T	C	T	G	A	G	G	G	G	T								
GBS	A1		-	**C**	-	-	-	-	-	-	-	-	-	-	1	500	356	224	95	290	320	42
GBS	A2		-	**T**	-	-	-	-	-	-	-	-	-	-	47	0	0	0	0	0	0	0
454	Haplotype 1	A1-like	**G**	**C**	T	C	T	G	A	G	G	G	G	T	266	606	540	438	262	502	491	410
454	Haplotype 2	A1-like	**G**	**C**	T	C	T	G	A	G	G	G	G	C	30	0	3	3	117	103	110	94
454	Haplotype 3	A1-like	**G**	**C**	T	T	T	G	A	G	T	G	G	T	0	151	0	179	0	0	0	1
454	Haplotype 4	A1-like	**G**	**C**	T	C	T	G	A	G	G	G	A	T	2	2	0	234	0	0	0	0
454	Haplotype 5	A2-like	**G**	**T**	T	C	T	G	A	G	G	G	G	T	120	0	183	1	1	0	0	0
454	TP91313	A1-like		**C**											298	759	543	854	379	605	601	505
454	TP91313	A2-like		**T**											120	0	183	1	1	0	0	0
454	TP91313	Other (<5%) A1		**C**											44	56	5	81	74	67	81	71
454	TP91313	Other (<5%) A2		**T**											24	0	4	3	1	1	2	0

Haplotypes with imperfect match with GBS TP

			**121**	**141**	156	174	190	199	206	208	230	247	253	415	TF0-17	TF0-20	TF0-36	TF0-38	TF5-5	TF5-12	TF5-20	TF5-28
454	Haplotype 1[b]		**G**	**C**	T	C	T	G	A	G	G	G	G	T	47	105	116	67	57	111	84	93
454	Haplotype 5[b]		**G**	**T**	T	C	T	G	A	G	G	G	G	T	42	0	41	0	1	0	0	0
454	Haplotype 6		**A**	**C**	G	C	A	A	C	A	G	A	G	C	80	0	0	0	223	106	97	88
454	Haplotype 7		**A**	**C**	G	C	A	A	C	A	G	A	G	T	38	0	0	0	75	75	63	60
		Total													207	105	157	67	356	292	244	241
		Other (<5%)													136	56	17	84	204	145	130	135

[a] SNP affecting *ApeKI* restriction site

[b] Haplotype containing GBS allele with sequencing errors in the 64 bp of the GBS TP

TP32628

Haplotypes with perfect match with GBS TP

Position			47	**80**	**89**	**116**	**131**	158	176	194	198	218	269	335	353	383	389	395								
Mt sequence			C	**A**	**T**	**T**	**G**	T	T	T	C	A	C	T	T	C	A	G	Read counts							
Ms Sanger consensus			Y	**A**	**T**	**T**	**G**	T	C	T	C	A	C	T	T	T	A	G	TF0-17	TF0-20	TF0-36	TF0-38	TF5-5	TF5-12	TF5-20	TF5-28
GBS	A1		-	-	**T**	-	-	-	-	-	-	-	-	-	-	-	-	-	11	0	0	38	9	14	13	15
	A2		-	-	**C**	-	-	-	-	-	-	-	-	-	-	-	-	-	23	35	25	0	9	28	15	27
454	Haplotype 1	A1-like	T	**A**	**T**	**T**	**G**	T	C	C	C	A	C	T	T	T	A	G	39	0	0	1	6	17	6	36
	Haplotype 2	A2-like	T	**A**	**C**	**T**	**G**	C	T	T	C	A	C	T	C	C	A	G	19	55	22	0	4	18	1	9
	TP32628	A1-like			**T**														38	0	0	0	0	0	0	36
		A2-like			**C**														0	54	0	0	0	0	0	0
		Other (<5%) A1			**T**														21	1	12	34	12	34	27	30
		Other (<5%) A2			**C**														32	10	48	1	19	42	9	33

Haplotypes with imperfect match with GBS TP

		47	80	89	116	131	158	176	194	198	218	269	335	353	383	389	395	TF0-17	TF0-20	TF0-36	TF0-38	TF5-5	TF5-12	TF5-20	TF5-28
454	Haplotype 1[b]	T	**A**	**T**	**T**	**G**	T	C	C	C	A	C	T	T	T	A	G	76	1	0	17	11	87	50	136
	Haplotype 2[b]	T	**A**	**C**	**T**	**G**	C	T	T	C	A	C	T	C	C	A	G	32	130	51	0	9	52	16	13
	Haplotype 3	T	**A**	**C**	**T**	**A**	C	T	T	C	A	C	T	C	C	A	G	0	0	31	0	3	18	0	14
	Haplotype 4	G	**G**	**T**	**G**	**G**	T	C	T	T	A	T	C	T	T	G	A	0	1	14	0	424	0	444	1
	Haplotype 5	T	**A**	**T**	**T**	**G**	C	T	T	C	G	C	T	C	C	A	G	0	0	0	216	0	0	0	0
	Haplotype 6	T	**A**	**T**	**T**	**G**	T	C	T	C	A	C	C	T	T	G	A	0	0	5	161	0	0	0	0
	Haplotype 7	T	**A**	**T**	**T**	**G**	T	C	C	C	A	C	T	C	C	A	G	44	0	0	6	2	44	0	39
	Haplotype 8	T	**A**	**T**	**T**	**G**	T	C	T	C	A	C	T	C	C	A	G	7	16	43	62	1	13	0	1
	Haplotype 9	T	**A**	-	**T**	**G**	C	T	T	C	A	C	T	C	C	A	G	90	284	80	0	16	124	12	22
	Haplotype 10	T	**A**	-	**T**	**A**	C	T	T	C	A	C	T	C	C	A	G	0	0	78	0	3	43	0	28
	Haplotype 11	T	**A**	-	**T**	**G**	C	T	T	C	A	C	T	T	T	A	G	32	12	7	0	0	28	3	11
	Total																	281	444	309	462	469	409	525	265
	Other (<5%)																	225	120	236	384	347	295	563	228

[b] Haplotype containing GBS allele with sequencing errors in the 64 bp of the GBS TP

TP47889

Haplotypes with perfect match with GBS TP

Position			28	42	130	166	207	275	Read counts							
Mt sequence			G	T	**T**	C	T	C								
Ms Sanger consensus			G	T	**T**	T	T	A	TF0-17	TF0-20	TF0-36	TF0-38	TF5-5	TF5-12	TF5-20	TF5-28
GBS	A1		-	-	**T**	-	-	-	14	10	4	2	0	4	16	13
	A2		-	-	**C**	-	-	-	0	3	0	5	0	4	8	0
454	Haplotype 1	A1-like	G	T	**T**	T	T	A	49	101	86	6	187	112	114	190
	Haplotype 2	A1-like	G	C	**T**	A	T	A	0	0	51	79	0	0	0	0
	Haplotype 3	A1-like	G	T	**T**	T	A	A	0	0	0	0	1	0	2	91
	Haplotype 4	A1-like	G	T	**T**	T	T	C	112	0	0	0	0	0	69	0
	Haplotype 5	A1-like	T	T	**T**	T	T	A	25	49	0	0	0	0	0	0
	Haplotype 6	A2-like	G	T	**C**	T	T	A	0	173	51	294	153	262	162	3
	TP47889	A1-like			**T**				186	150	137	85	188	112	185	281
		A2-like			**C**				0	173	51	294	153	262	162	3
		Other (<5%) A1			**T**				111	56	66	45	63	46	93	115
		Other (<5%) A2			**C**				0	73	24	113	66	103	107	4

Haplotypes with imperfect match with GBS TP

			28	42	130	166	207	275	TF0-17	TF0-20	TF0-36	TF0-38	TF5-5	TF5-12	TF5-20	TF5-28
454	Haplotype 1[b]		G	T	**T**	T	T	A	6	11	7	0	28	10	15	41
	Haplotype 6[b]		G	T	**C**	T	T	A	0	18	1	34	20	33	16	1
		Total							6	29	8	34	48	43	31	42
		Other (<5%)							85	125	76	143	153	141	166	124

[b] Haplotype containing GBS allele with sequencing errors in the 64 bp of the GBS TP

TP61949

Haplotypes with perfect match with GBS TP

		Position	46	118	133	145	229	262								
									Read counts							
		Mt sequence	**C**	C	C	A	T	T								
		Ms Sanger consensus	**N**	T	C	A	C	T	TF0-17	TF0-20	TF0-36	TF0-38	TF5-5	TF5-12	TF5-20	TF5-28
GBS		A1	**C**	-	-	-	-	-	17	43	19	2	0	23	29	11
GBS		A2	**T**	-	-	-	-	-	1	20	0	9	21	3	5	0
454	Haplotype 1	A1-like	**C**	T	C	A	C	T	161	183	357	215	85	339	216	445
454	Haplotype 2	A1-like	**C**	T	C	A	T	T	5	18	0	16	6	35	10	2
454	Haplotype 3	A1-like	**C**	T	C	A	C	A	0	0	0	1	1	3	0	73
454	Haplotype 4	A2-like	**T**	C	C	G	T	T	51	46	0	3	130	1	87	0
454	Haplotype 5	A2-like	**T**	T	C	A	C	T	73	3	0	5	79	14	14	2
454	Haplotype 6	A2-like	**T**	C	T	G	T	T	0	0	0	0	0	74	0	0
454	Haplotype 7	A2-like	**T**	C	C	G	C	T	22	6	0	0	15	0	20	1
454	Haplotype 8	A2-like	**T**	C	T	G	C	T	0	0	0	0	1	32	0	0
454	Haplotype 9	A2-like	**T**	T	C	A	T	T	4	1	0	1	18	0	0	0
454	Haplotype 10	A2-like	**T**	-	C	G	T	T	0	0	0	41	2	0	0	2
454	TP61949	A1-like	**C**						166	201	357	232	92	377	226	520
454	TP61949	A2-like	**T**						150	56	0	50	245	121	121	5
454	TP61949	Other (<5%) A1	**C**						19	14	8	10	8	38	20	9
454	TP61949	Other (<5%) A2	**T**						11	2	0	12	7	11	12	1

Haplotypes with imperfect match with GBS TP

454	Haplotype 1[b]	**C**	T	C	A	C	T	33	36	50	34	16	71	47	100
454	Haplotype 4[b]	**T**	C	C	G	T	T	11	13	1	0	28	0	16	1
454	Haplotype 5[b]	**T**	T	C	A	C	T	26	0	0	1	18	6	6	1
454	Total							70	49	51	35	62	77	69	102
454	Other (<5%)							15	7	1	22	25	35	17	21

[b] Haplotype containing GBS allele with sequencing errors in the 64 bp of the GBS TP

TP14949

Haplotypes with perfect match with GBS TP

			46	**118**	133	145	229	262	Read counts							
Position			46	**118**	133	145	229	262								
Mt sequence			C	**C**	C	A	T	T								
Ms Sanger consensus			N	**T**	C	A	C	T	TF0-17	TF0-20	TF0-36	TF0-38	TF5-5	TF5-12	TF5-20	TF5-28
GBS		A1	-	**T**	-	-	-	-	0	124	39	11	10	24	113	61
		A2	-	**C**	-	-	-	-	0	30	0	0	0	153	91	1
454	Haplotype 1	A1-like	C	**T**	C	A	C	T	164	178	349	51	87	346	224	86
	Haplotype 2	A1-like	C	**T**	C	A	T	T	6	12	0	4	7	33	11	0
	Haplotype 4	A2-like	T	**C**	C	G	T	T	51	40	0	3	121	1	86	0
	Haplotype 5	A1-like	T	**T**	C	A	C	T	80	3	0	1	80	12	13	1
	Haplotype 6	A2-like	T	**C**	T	G	T	T	0	0	0	0	0	70	0	0
	Haplotype 8	A2-like	T	**C**	T	G	C	T	0	0	0	0	1	34	0	0
		A1-like		**T**					250	193	349	56	174	391	248	87
		A2-like		**C**					51	40	0	3	122	105	86	0
		Other (<5%) A1		**T**					14	9	7	1	25	8	11	17
	TP14949	Other (<5%) A2		**C**					39	14	0	0	29	46	41	0

Haplotypes with imperfect match with GBS TP

454	Haplotype 1[b]	C	**T**	C	A	C	T	30	41	58	198	14	64	39	459
	Haplotype 3	C	**T**	C	A	C	A	0	0	0	0	0	0	0	68
	Haplotype 4[b]	T	**C**	C	G	T	T	11	19	1	0	37	0	17	1
	Haplotype 10	T	-	C	G	T	T	1	0	0	49	4	0	0	2
	Total							42	60	59	247	55	64	56	530
	Other (<5%)							35	13	2	54	34	45	23	24

[b] Haplotype containing GBS allele with sequencing errors in the 64 bp of the GBS TP

TP31029

Haplotypes with perfect match with GBS TP

Position			**37**[a]	**51**	**58**	116	151	266	304	396[a]	Read counts							
Mt sequence			**A**	**G**	**T**	A	T	-	G	T								
Ms Sanger consensus			**A**	**G**	**T**	R	T	G	G	C	TF0-17	TF0-20	TF0-36	TF0-38	TF5-5	TF5-12	TF5-20	TF5-28
GBS	A1		-	-	**T**	-	-	-	-	-	31	39	10	14	9	6	11	0
	A2		-	-	**C**	-	-	-	-	-	0	0	0	0	0	0	5	8
454	Haplotype 1	A1-like	**A**	**G**	**T**	G	T	G	G	C	106	388	114	344	207	121	30	216
	Haplotype 2	A1-like	**A**	**G**	**T**	A	T	G	G	T	44	0	96	1	48	28	43	0
	Haplotype 3	A1-like	**A**	**G**	**T**	A	T	G	G	C	22	1	59	1	39	36	16	7
	Haplotype 4	A1-like	**A**	**G**	**T**	G	T	G	G	T	38	1	50	2	35	17	29	1
	Haplotype 5	A1-like	**A**	**G**	**T**	G	A	G	G	C	0	1	0	91	0	0	0	0
	Haplotype 6	A1-like	**A**	**G**	**T**	G	T	G	C	C	0	0	0	0	0	14	12	23
	Haplotype 7	A1-like	**A**	**G**	**T**	A	T	T	G	T	4	0	0	0	0	0	27	0
	Haplotype 8	A2-like	**A**	**G**	**C**	A	T	G	C	C	0	0	0	0	0	27	43	40
	Haplotype 9	A2-like	**A**	**G**	**C**	A	T	G	G	C	0	0	0	0	0	18	14	28
	TP31029	A1-like			**T**						214	391	319	439	329	216	157	247
		A2-like			**C**						0	0	0	0	0	45	57	68
		Other (<5%) A1			**T**						14	17	6	12	7	25	61	7
		Other (<5%) A2			**C**						0	2	2	2	0	12	22	14

Haplotypes with imperfect match with GBS TP

		37	51	58	116	151	266	304	396	TF0-17	TF0-20	TF0-36	TF0-38	TF5-5	TF5-12	TF5-20	TF5-28
454	Haplotype 10	**T**	**G**	**T**	G	T	G	G	C	0	0	0	0	107	0	0	0
	Haplotype 11	**A**	**A**	**T**	A	T	T	G	T	21	0	0	0	0	0	0	0
	Haplotype 1[b]	**A**	**G**	**T**	G	T	G	G	C	24	74	21	63	41	29	11	70
	Haplotype 2[b]	**A**	**G**	**T**	A	T	G	G	T	8	0	23	1	14	12	13	0
Total										53	74	44	64	162	41	24	70
Other (<5%)										59	14	35	36	76	40	69	45

[a] SNP affecting *ApeKI* restriction site

[b] Haplotype containing GBS allele with sequencing errors in the 64 bp of the GBS TP

TP46847

Haplotypes with perfect match with GBS TP

			50	143	155	170	187	311	332	343	360	Read counts							
Position			**50**	143	155	170	187	311	332	343	360	TF0-17	TF0-20	TF0-36	TF0-38	TF5-5	TF5-12	TF5-20	TF5-28
Mt sequence			**A**	T	C	G	C	A	T	A	T								
Ms Sanger consensus			**T**	T	Y	G	C	A	N	A	C								
GBS	A1		**T**	-	-	-	-	-	-	-	-	29	117	64	32	54	37	93	52
GBS	A2		**A**	-	-	-	-	-	-	-	-	45	32	50	31	6	12	20	8
454	Haplotype 1	A1-like	**T**	T	T	G	C	A	A	A	C	3	72	354	260	289	200	225	276
454	Haplotype 2	A1-like	**T**	T	C	G	T	A	A	A	C	157	0	0	0	0	0	0	0
454	Haplotype 3	A1-like	**T**	T	C	A	C	A	A	A	C	3	80	0	0	0	0	0	0
454	Haplotype 4	A2-like	**A**	A	C	G	C	G	C	G	T	0	62	1	0	0	0	0	0
454	Haplotype 5	A2-like	**A**	A	C	G	C	A	A	A	C	0	29	0	0	0	0	0	0
454	TP46847	A1-like	**T**									163	152	354	260	289	200	225	276
454	TP46847	A2-like	**A**									0	91	1	0	0	0	0	0
454	TP46847	Other (<5%) A1	**T**									6	91	22	11	12	21	14	9
454	TP46847	Other (<5%) A2	**A**									10	31	5	9	22	10	13	3

Haplotypes with imperfect match with GBS TP

											TF0-17	TF0-20	TF0-36	TF0-38	TF5-5	TF5-12	TF5-20	TF5-28
454	Haplotype 1[b]	**T**	T	T	G	C	A	A	A	C	0	30	133	78	107	58	79	157
454	Haplotype 2[b]	**T**	T	C	G	T	A	A	A	C	54	0	0	0	0	0	0	0
454	Haplotype 3[b]	**T**	T	C	A	C	A	A	A	C	0	35	0	0	0	0	0	0
454	Total										54	65	133	78	107	58	79	157
454	Other (<5%)										18	78	30	12	34	8	33	22

[b] Haplotype containing GBS allele with sequencing errors in the 64 bp of the GBS TP

TP17289

Haplotypes with perfect match with GBS TP

			50	110	185	263	388								
Position			**50**	110	185	263	388	Read counts							
Mt sequence			**T**	A	T	C	-								
Ms Sanger consensus			**T**	A	C	T	A	TF0-17	TF0-20	TF0-36	TF0-38	TF5-5	TF5-12	TF5-20	TF5-28
GBS		A1	**T**	-	-	-	-	8	50	34	10	14	37	45	0
GBS		A2	**C**	-	-	-	-	12	0	32	9	15	7	0	2
454	Haplotype 1	A1-like	**T**	A	A	T	A	127	159	86	83	161	145	35	57
454	Haplotype 2	A1-like	**T**	A	A	C	A	17	17	11	41	19	21	146	27
454	Haplotype 3	A1-like	**T**	G	A	C	A	0	0	2	0	0	0	59	3
454	Haplotype 4	A1-like	**T**	A	G	C	A	2	59	0	0	0	0	0	0
454	Haplotype 5	A1-like	**T**	A	A	C	G	0	0	0	0	0	0	0	31
454	Haplotype 6	A2-like	**C**	A	A	C	A	36	0	32	121	37	46	0	42
454	Haplotype 7	A2-like	**C**	A	A	T	A	10	0	12	33	18	13	0	11
454	TP17289	A1-like	**T**					146	235	99	124	180	166	240	118
454	TP17289	A2-like	**C**					46	0	44	154	55	59	0	53
454	TP17289	Other (<5%) A1	**T**					11	36	14	23	13	17	39	16
454	TP17289	Other (<5%) A2	**C**					8	0	16	41	9	10	0	14

Haplotypes with imperfect match with GBS TP

454	Haplotype 1[b]	**T**	A	A	T	A	22	37	25	12	39	22	6	18
454	Haplotype 2[b]	**T**	A	A	C	A	6	3	0	3	2	5	31	12
454	Haplotype 6[b]	**C**	A	A	C	A	5	0	6	21	10	7	0	17
454	Total						33	40	31	36	51	34	37	47
454	Other (<5%)						11	17	6	18	14	3	21	27

[b] Haplotype containing GBS allele with sequencing errors in the 64 bp of the GBS TP

<u>**TP1933**</u>

Haplotypes with perfect match with GBS TP

Position			**53**	226	248	249								
Mt sequence			**A**	T	T	A	Read counts							
Ms Sanger consensus			**M**	T	T	A	TF0-17	TF0-20	TF0-36	TF0-38	TF5-5	TF5-12	TF5-20	TF5-28
GBS		A1	**A**	-	-	-	34	19	13	20	16	29	7	9
		A2	**C**	-	-	-	0	50	5	17	9	20	19	24
454	Haplotype 1	A1-like	**A**	T	T	A	10	7	12	5	10	8	2	0
	Haplotype 2	A1-like	**A**	T	T	T	15	0	0	0	1	2	0	0
	Haplotype 3	A1-like	**A**	T	C	A	0	3	3	0	4	1	2	0
	Haplotype 4	A1-like	**A**	A	T	A	0	2	0	3	0	0	0	0
	Haplotype 5	A2-like	**C**	A	T	A	0	12	7	15	5	10	12	6
	Haplotype 6	A2-like	**C**	T	T	A	0	1	1	1	3	1	2	5
	Haplotype 7	A2-like	**C**	A	C	A	0	1	0	6	0	0	1	1
	TP1933	A1-like	**A**				25	12	15	8	15	11	4	0
		A2-like	**C**				0	14	8	22	8	11	15	12
		Other (<5%) A1	**A**				0	0	1	1	0	1	1	1
		Other (<5%) A2	**C**				0	0	1	5	1	1	3	7

Haplotypes with imperfect match with GBS TP

454	Haplotype 1[b]	**A**	T	T	A	4	6	4	5	7	4	1	2
	Haplotype 2[b]	**A**	T	T	T	4	1	0	1	1	0	0	0
	Haplotype 3[b]	**A**	T	C	A	0	2	0	0	1	3	0	0
	Haplotype 5[b]	**C**	A	T	A	0	7	5	6	2	12	14	6
	Haplotype 6[b]	**C**	T	T	A	0	1	2	2	1	1	2	8
	Haplotype 7[b]	**C**	A	C	A	0	0	0	3	0	1	1	2
	Haplotype 8	**C**	A	T	T	0	1	0	1	0	3	2	1
	Total					8	18	11	18	12	24	20	19
	Other (<5%)					4	3	1	2	4	4	1	10

TP26408

Haplotypes with perfect match with GBS TP

Position			53	226	248	249								
Mt sequence			**A**	T	T	A	Read counts							
Ms Sanger consensus			**M**	T	T	A	TF0-17	TF0-20	TF0-36	TF0-38	TF5-5	TF5-12	TF5-20	TF5-28
GBS		A1	**A**	-	-	-	19	13	11	4	22	10	24	0
		A2	**C**	-	-	-	0	12	19	43	0	8	64	6
454	Haplotype 1	A1-like	**A**	T	T	A	5	4	9	6	5	5	3	2
	Haplotype 2	A1-like	**A**	T	T	T	6	0	0	0	0	0	0	0
	Haplotype 3	A1-like	**A**	T	C	A	0	0	1	0	2	2	1	0
	Haplotype 4	A1-like	**A**	A	T	A	0	0	0	3	0	0	0	1
	Haplotype 5	A2-like	**C**	A	T	A	0	18	8	12	6	18	18	9
	Haplotype 6	A2-like	**C**	T	T	A	0	1	2	2	3	2	4	10
	Haplotype 7	A2-like	**C**	A	C	A	0	1	0	6	0	1	1	2
	Haplotype 8	A2-like	**C**	A	T	T	0	0	0	1	0	3	1	3
	TP26408	A1-like	**A**				11	4	11	10	7	7	5	5
		A2-like	**C**				0	20	10	21	9	24	24	24
		Other (<5%) A1	**A**				0	0	0	0	0	0	0	1
		Other (<5%) A2	**C**				0	1	1	4	0	2	2	4

Haplotypes with imperfect match with GBS TP

Haplotype 1[b]	**A**	T	T	A	9	9	7	4	12	7	0	0
Haplotype 2[b]	**A**	T	T	T	13	1	0	1	2	2	0	0
Haplotype 3[b]	**A**	T	C	A	0	5	2	0	3	2	1	0
Haplotype 4[b]	**A**	A	T	A	0	3	0	0	0	0	0	0
Haplotype 5[b]	**C**	A	T	A	0	1	4	9	1	4	8	3
Haplotype 6[b]	**C**	T	T	A	0	1	1	1	1	0	0	3
Haplotype 7[b]	**C**	A	C	A	0	0	0	3	0	0	1	1
Haplotype 8[a]	**C**	A	T	T	0	1	1	2	1	0	3	0
Total					22	21	15	20	20	15	13	7
Other (<5%)					4	1	0	1	4	4	0	8

[b] Haplotype containing GBS allele with sequencing errors in the 64 bp of the GBS TP

*Both TP are targetting the same SNP loci and haplotypes identified are identical
